# Circulating metabolites modulated by diet are associated with depression

**DOI:** 10.1038/s41380-023-02180-2

**Published:** 2023-07-26

**Authors:** Ashley van der Spek, Isobel D. Stewart, Brigitte Kühnel, Maik Pietzner, Tahani Alshehri, Friederike Gauß, Pirro G. Hysi, Siamak MahmoudianDehkordi, Almut Heinken, Annemarie I. Luik, Karl-Heinz Ladwig, Gabi Kastenmüller, Cristina Menni, Johannes Hertel, M. Arfan Ikram, Renée de Mutsert, Karsten Suhre, Christian Gieger, Konstantin Strauch, Henry Völzke, Thomas Meitinger, Massimo Mangino, Antonia Flaquer, Melanie Waldenberger, Annette Peters, Ines Thiele, Rima Kaddurah-Daouk, Boadie W. Dunlop, Frits R. Rosendaal, Nicholas J. Wareham, Tim D. Spector, Sonja Kunze, Hans Jörgen Grabe, Dennis O. Mook-Kanamori, Claudia Langenberg, Cornelia M. van Duijn, Najaf Amin

**Affiliations:** 1 Department of Epidemiology, Erasmus MC University Medical Center, Rotterdam, The Netherlands; 2 SkylineDx B.V., Rotterdam, The Netherlands; 3 MRC Epidemiology Unit, University of Cambridge, Cambridge, UK; 4 Research Unit of Molecular Epidemiology, Helmholtz Zentrum München, German Research Center for Environmental Health, D-85764 Neuherberg, Germany; 5 Institute of Epidemiology, Helmholtz Zentrum München, German Research Center for Environmental Health, D-85764 Neuherberg, Germany; 6 Precision Healthcare University Research Institute, Queen Mary University of London, London, UK; 7 Computational Medicine, Berlin Institute of Health at Charité, Universitätsmedizin Berlin, Berlin, Germany; 8 Department of Clinical Epidemiology, Leiden University Medical Center, Leiden, The Netherlands; 9 Institute of Clinical Chemistry and Laboratory Medicine, University Medicine Greifswald, Ferdinand-Sauerbruch-Str, 17475 Greifswald, Germany; 10 Department of Twins Research and Genetic Epidemiology, Kings College London, London, UK; 11 Department of Psychiatry and Behavioral Sciences, Duke University, Durham, NC USA; 12 School of Medicine, University of Galway, University Road, Galway, Ireland; 13 Inserm UMRS 1256 NGERE – Nutrition, Genetics, and Environmental Risk Exposure, University of Lorraine, Nancy, France; 14 Department of Psychosomatic Medicine and Psychotherapy, Klinikum rechts der Isar, Technische Universität München, Munich, Germany; 15 Institute of Computational Biology, Helmholtz Zentrum München, German Research Center for Environmental Health, D-85764 Neuherberg, Germany; 16 German Center for Diabetes Research (DZD e.V.), D-85764 Neuherberg, Germany; 17 Department of Psychiatry and Psychotherapy, University Medicine Greifswald, Ellernholzstrasse 1-2, 17489 Greifswald, Germany; 18 Department of Physiology and Biophysics, Weill Cornell Medicine-Qatar, Education City, PO 24144 Doha, Qatar; 19 Institute of Genetic Epidemiology, Helmholtz Zentrum München, German Research Center for Environmental Health, D-85764 Neuherberg, Germany; 20 Chair of Genetic Epidemiology, IBE, Faculty of Medicine, LMU Munich, Germany; 21 Institute of Community Medicine, University Medicine Greifswald, Walter-Rathenau Str. 48, 17475 Greifswald, Germany; 22 Institute of Human Genetics, Helmholtz Zentrum München, German Research Center for Environmental Health, D-85764 Neuherberg, Germany; 23 Institute of Human Genetics, Technische Universität München, Munich, Germany; 24 German Center for Cardiovascular Research (DZHK), Partner Site Munich Heart Alliance, Munich, Germany; 25 Ludwig-Maximilians-Universität München, IBE-Chair of Epidemiology, Munich, Germany; 26 Division of Microbiology, University of Galway, Galway, Ireland; 27 APC Microbiome, Ireland, Ireland; 28 Duke Institute of Brain Sciences, Duke University, Durham, NC USA; 29 Department of Medicine, Duke University, Durham, NC USA; 30 Department of Psychiatry and Behavioral Sciences, Emory University School of Medicine, Atlanta, GA US; 31 Department of Clinical Epidemiology, Department of Public Health and Primary Care, Leiden University Medical Center, Leiden, The Netherlands; 32 Nuffield Department of Population Health, University of Oxford, OX3 7LF Oxford, UK

**Keywords:** Depression, Molecular biology

## Abstract

Metabolome reflects the interplay of genome and exposome at molecular level and thus can provide deep insights into the pathogenesis of a complex disease like major depression. To identify metabolites associated with depression we performed a metabolome-wide association analysis in 13,596 participants from five European population-based cohorts characterized for depression, and circulating metabolites using ultra high-performance liquid chromatography/tandem accurate mass spectrometry (UHPLC/MS/MS) based Metabolon platform. We tested 806 metabolites covering a wide range of biochemical processes including those involved in lipid, amino-acid, energy, carbohydrate, xenobiotic and vitamin metabolism for their association with depression. In a conservative model adjusting for life style factors and cardiovascular and antidepressant medication use we identified 8 metabolites, including 6 novel, significantly associated with depression. In individuals with depression, increased levels of retinol (vitamin A), 1-palmitoyl-2-palmitoleoyl-GPC (16:0/16:1) (lecithin) and mannitol/sorbitol and lower levels of hippurate, 4-hydroxycoumarin, 2-aminooctanoate (alpha-aminocaprylic acid), 10-undecenoate (11:1n1) (undecylenic acid), 1-linoleoyl-GPA (18:2) (lysophosphatidic acid; LPA 18:2) are observed. These metabolites are either directly food derived or are products of host and gut microbial metabolism of food-derived products. Our Mendelian randomization analysis suggests that low hippurate levels may be in the causal pathway leading towards depression. Our findings highlight putative actionable targets for depression prevention that are easily modifiable through diet interventions.

## Introduction

Depression is one of the most common psychiatric disorders with an average lifetime prevalence of 11–15% [1]. A sharp increase in the prevalence of depression worldwide (33.7%; confidence interval 27.5–40.6) has been observed during the recent COVID-19 pandemic [2]. However, as the molecular mechanisms underlying depression remain elusive, the current treatment options for depression remain ineffective [3, 4]. The heritability of depression is estimated to be around 40% [5]. Several small effect (odds ratio <1.05) non-coding genetic variants have been identified to be associated with depression [6] but their contribution to the pathogenesis of depression remains unclear. There is also a range of environmental risk factors for morbidity including low education, diet and smoking [7]. There is increasing evidence that diet influences mood [8] by modulating the gut microbiome [9]. High consumption of fresh fruits, vegetables, whole grain, fish, and foods rich in antioxidants has been linked to improved gut health [9] and decreased risk of depression, while consumption of red and/or processed meat, refined grains, sweets and high-fat products is associated with increased risk of depression [10]. Large meta-analysis of clinical trials suggests that dietary interventions significantly reduce depressive symptoms, particularly in women [11] and the risk of several cardio-vascular diseases [12–14] that cluster strongly with depression [15].

Metabolome captures the downstream effects of genes, lifestyle factors such as diet, pathology and medication use and hence provides a useful tool to uncover biological mechanisms underlying complex diseases [16–18]. A novel hypothesis why circulating metabolites may be involved in depression is that these metabolites are involved in the gut-brain axis, i.e., the bi-directional signaling between the gut, its microbiome and the brain [19, 20]. Our study of 5283 patients with depression and 10,145 controls from nine Dutch cohorts [21] using a proton Nuclear Magnetic Resonance (NMR) metabolomics platform identified 21 cardiometabolic metabolites including apolipoproteins, very-low-density and high-density lipoprotein cholesterol (VLDLs and HDLs), di- and triglycerides, fatty acids, acetate, glycoprotein acetyls, tyrosine, and isoleucine [21]. Using the same metabolomics platform in the UK Biobank (*n* > 63,000), we confirmed most of these findings including the shift in the VLDL and HDL sub-fractions observed in depressed individuals in addition to finding disruption in the tricarboxylic acid (TCA) cycle - low citrate and high pyruvate levels in depressed individuals [22]. We further showed that the host gut microbiome partly explains the shift in VLDLs and HDLs observed in depressed individuals and that increase in VLDLs and changes in fatty acids are more likely to be a consequence of the disease [22]. These large-scale metabolomics studies are a major leap forward in understanding the pathogenesis in addition to identifying potential biomarkers and therapeutic targets for the disease. However, the NMR-based Nightingale platform is limited in the number of compounds measured and the metabolic pathways they cover, thus limiting the discovery of more diverse pathways. On the other hand, studies that have used larger mass spectrometry based metabolic platforms are small and have often resulted in false positive associations and inconsistent findings due to confounding bias resulting from the unaccounted for differences in lifestyle factors and medication use between cases and controls [23]. Some consensus is building that depression is associated with increased levels of glutamate, lactate, alanine, isobutyrate and sorbitol and with decreased levels of kynurenine, gamma aminobutyric acid (GABA), phenylalanine, tyrosine, creatinine, hypoxanthine, leucine, tryptophan, N-methylnicotinamide, β-aminoisobutyric acid, hippurate, amino-ethanol and malonate [23]. Again, whether these are causal associations or merely a consequence of the disease is not clear.

**Fig. 1:**
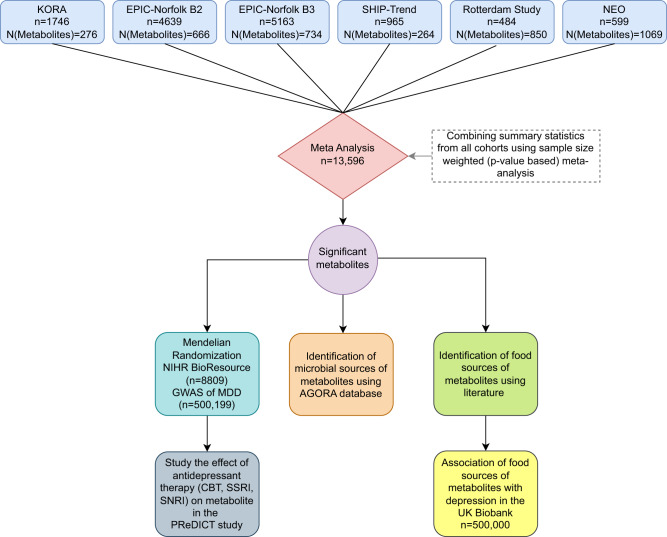
Flowchart of the study.

In the current study, using an untargeted mass spectrometry-based metabolomics (Metabolon) that measures over 800 compounds in a large sample (*n* = 13,596), we aim to 1) identify robust potential metabolic biomarkers of depression by minimizing confounding bias; 2) Elucidate causal relationships to identify possible therapeutic targets; 3) Study the association of major dietary sources of the metabolites with depression, 4) Study the impact of different types of antidepressant therapy on the (potentially causal) associated metabolites, and finally 5) identify the gut microbiota involved in the metabolism of the identified metabolites (Fig. 1).

## Methods

We first perform a metabolome-wide association analysis of depression in 13,596 individuals from five European cohorts, characterized for metabolomics using mass-spectrometry-based Metabolon platform analysing 806 metabolites that cover a wide range of metabolic pathways (Fig. 1). We build a conservative model adjusting for most known confounders including lifestyle factors and medication use as suggested by MacDonald et al. [23]. Next, we use Mendelian Randomization to infer causal associations using the NIHR BioResource (NBR) and publicly available summary statistics of the largest genome-wide association study (GWAS) of depression [6]. We then identify the major food sources of the metabolites using previous literature and study the association of these food sources with depression in the UK Biobank study (*N* < 500,000). For metabolites that show potentially causal relationship with depression, we study the impact of antidepressant therapy in the Predictors of Remission in Depression to Individual and Combined Treatments (PReDICT) study. Finally, we integrate our findings with those of the Virtual Metabolic Human (VMH) and Assembly of Gut Organisms through Reconstruction and Analysis (AGORA2) databases to identify gut microbiota involved in the metabolism of the identified metabolites (Fig. 1).

### Study populations

#### Metabolites association analysis

The association analysis of metabolite levels with depression was performed in 13,596 participants separately recruited in five different cohort studies (Table 1). The following cohort studies were included: the Rotterdam Study, the Study of Health in Pomerania (SHIP-TREND), the Cooperative Health Research in the Region of Augsburg (KORA) study, the European Prospective Investigation into Cancer (EPIC)-Norfolk Study, and the Netherlands Epidemiology of Obesity (NEO) study. Detailed information on these cohorts is provided in the Supplementary Materials. All participants provided written informed consent, studies were approved by their local ethics committees and conformed to the principles of the declaration of Helsinki. Patients or the public were not involved in the design, or conduct, or reporting, or dissemination plans of our research.

**Table 1:** Descriptive statistics of the study populations.

	Rotterdam Study	SHIP-trend	KORA	EPIC-Norfolk B2	EPIC-Norfolk B3	NEO
*N*	484	965	1746	4639	5163	599
Ncases/Ncontrols	-	-	-	638/4001	685/4478	-
Mean age (years) (SD)	73.1 (6.3)	50.1 (13.6)	61 (8.8)	59.9 (8.8)	59.6 (8.9)	55.8 (6.0)
Age range (years)	62–96	20–81	32–77	40–78	40–78	45–66
Females (%)	52.5	56.0	51.4	52.4	52.8	52.6
Mean BMI (kg/m^2^) (SD)	26.8 (3.7)	27.4 (4.6)	28.2 (4.8)	26.2 (3.7)	26.2 (3.8)	25.9 (4.0)
Smoking (%)	12.6	22.0	14.5	11.4	10.9	11.9
Medication						
Antidepressants (%)	3.7	4.0	5.6	4.5	3.8	5.3
Lipid-lowering medication (%)	10.5	7.8	16.7	1.4	1.5	7.7
Antihypertensives (%)	0.6	28.2	37.9	19.5	17.0	19.7
Antidiabetics (%)	5.4	0	7.5	1.9	2.0	2.7

#### Inference of causal relationships using Mendelian Randomization (MR)

To select instruments/proxies for metabolites for MR we used the results of the GWAS performed using the NBR. NBR – Rare Disease Study is a multi-center whole-exome and whole-genome sequencing study including up to 13,600 patients (http://bioresource.nihr.ac.uk/rare-diseases/rare-diseases/). The NBR–Rare Diseases study was approved by the East of England Cambridge South national research ethics committee (REC) under reference number: 13/EE/0325. The inclusion and exclusion criteria, as well as other steps of quality control, adjustment and transformations followed the same analytical steps as described before [24].

#### Identification and association of major dietary sources of the depression-associated metabolites

Major dietary sources of the depression-associated metabolites were identified through literature search. Association of depression with the dietary sources of the depression-associated metabolites was performed in the UK Biobank study. UK Biobank is a prospective cohort study including ~500,000 participants aged 40–69 years at baseline recruited between 2006 and 2010. The aim of the study is to investigate the effects of genetic and environmental factors on the risk of common multifactorial diseases. Participants have provided a detailed information on lifestyle, medical history and nutritional habits; basic variables such weight, height, blood pressure etc. were measured; and blood and urine samples were taken. Detailed information about the cohort is provided in the Supplementary Materials. The current study is a part of the UKB project 54520.

#### Impact of anti-depressant therapy on depression-associated (causal) metabolites

The effects of various depression treatments including cognitive behavioral therapy (CBT) and antidepressants SSRI (escitalopram) and SNRI (duloxetine) on the depression-associated metabolites were studied in the PReDICT study. The design of PReDICT study has been published previously [25]. Details on the study and the metabolomics assessments are provided in the Supplementary Materials.

### Depression assessment

In the Rotterdam Study, depressive symptoms were assessed with the 20-item version of the Centre for Epidemiologic Studies Depression (CES-D) scale (Supplementary Table 1), a self-report measure of depressive symptoms experienced during the prior week [26]. The total score ranges from 0 to 60, where a higher score indicates more depressive symptoms. In the SHIP-trend and KORA cohorts, depressive symptoms were assessed with the Patient Health Questionnaire 9 (PHQ-9) [27], where each of the nine DSM-IV criteria for depression are scored from 0 to 3. The total score ranges from 0 to 27 where higher score indicates a greater depression severity. In KORA a brief interview version of PHQ-9 called Patient Health Questionnaire Depression (PHQ-D) module was used to measure depression [27, 28]. In the EPIC-Norfolk study depression was assessed using the following question: “Has the doctor ever told you that you have any of the following: depression requiring treatment?” with answers “yes” or “no”. In the NEO cohort, depressive symptoms were assessed using the Inventory Depressive Symptomatology Self Report questionnaire (IDS-SR30) [29], which assesses specific depressive symptoms (via a 4-level response system) during the last week and their severity. The total score ranges from 0 to 84, with higher scores indicating higher severity. Thus, in all cohorts except EPIC-Norfolk, depression in participants was measured on a quantitative scale and used as such in the analysis.

In the UKB study, we used the derived lifetime probable major depressive disorder measure as described in Smith et al. [30]. We further defined current depressive symptoms by summing the responses to four questions related to mood in the past two weeks. These include, (1) Over the past two weeks, how often have you felt down, depressed or hopeless?, (2) Over the past two weeks, how often have you had little interest or pleasure in doing things?, (3) Over the past two weeks, how often have you felt tense, fidgety or restless? and (4) Over the past two weeks, how often have you felt tired or had little energy? Answers could be given on a four-point scale ranging from 0 to 3 (0 = not at all, 1 = several days, 2 = more than half of the days and 3 = nearly every day). The total score ranged from 0 to 12 where higher score indicating more severe depression.

In the PReDICT study, participants were treatment-naive adults defined as having never previously received a minimally adequate course of treatment with an antidepressant medication or evidence-based psychotherapy for a mood disorder, aged 18 to 65 years with moderate-to-severe, non-psychotic MDD depression as assessed by the Structured Clinical Interview for DSM-IV [31] and a psychiatrist’s evaluation, and if they scored ≥18 on the HRSD17. Eligible patients were randomized equally to one of three 12-week treatment arms: (1) cognitive behavior therapy (CBT, 16 sessions); (2) duloxetine (30–60 mg/d); or (3) escitalopram (10–20 mg/d).

### Metabolomics measurements

In all studies, the metabolome was quantified using the Metabolon platform (Metabolon Inc., Durham, USA) (Supplementary Table 1). Different versions of the platform have been used and details on the platforms are included in the Supplementary Materials. In each study, metabolites with ≥40% missing values were removed prior to the analysis and for the remaining metabolites, missing metabolite values were replaced with half of the detection limit for that particular metabolite [32]. Subsequently, a natural logarithm transformation was applied to all metabolites and metabolites were scaled to standard deviation units.

In the PReDICT study, metabolites were quantified using targeted metabolomics platforms including ultra-performance liquid chromatography triple quadrupole mass spectrometry (UPLC-TQMS) (Waters XEVO TQ-S, Milford, USA) and gas chromatography time-of-flight mass spectrometry (GC-TOFMS) (Leco Corporation, St Joseph, USA). Metabolites with >20% missing values were excluded. Then, metabolites were log-transformed, imputed and scaled to mean zero and variance 1. Details are provided in the Supplementary Materials.

Non-targeted metabolite detection and quantification was conducted by the metabolomics provider Metabolon, Inc. (Durham, USA) on fasting plasma samples of 10,654 participants from the UK Bioresource. The metabolomic dataset measured by Metabolon included 1069 compounds of known structural identity belonging to the following broad categories - amino-acids, peptides, carbohydrates, energy intermediates, lipids, nucleotides, cofactors and vitamins, and xenobiotics. Metabolites data were day-median normalized, and inverse normalized, as the metabolite concentrations were not normally distributed. Metabolites with more than 20% missing values were excluded leaving 722 metabolites of known chemical identity for analysis.

### Genotyping

For the GWAS of metabolites, genotyping in the UK bioresource was carried out with a high-density array data (Affymetrix UK Biobank Axiom® Array). Genotypes were subsequently imputed using information from the Human Reference Consortium imputation panel (version r1.1, 2016) [33]. Only individuals of full European ancestry (*N* = 8809) were included in the analyzes in the discovery cohort.

### Statistical analyzes

#### Metabolites association analysis

Linear regression analyzes were used to test the association between the levels of each metabolite (dependent variable) and depression. Three different models were tested, where the first model (model 1) was adjusted for age and sex only, the second model (model 2) was additionally adjusted for antidepressant medication usage, and the third model was an extension of the second model (model 3) with additional adjustment for lipid-lowering medication (yes/no), antihypertensive medication (yes/no), antidiabetic medication (yes/no), BMI (kg/m^2^), and current smoking (yes/no). The summary statistics from all cohorts were combined in a sample size-weighted meta-analysis using METAL software [34]. Sample size weighted meta-analysis was used since the depression measurement scales were different among cohorts. Only metabolites that were present in two or more studies were included. To investigate the robustness of our findings, a sensitivity analysis was performed by including only cohorts that assessed metabolites with the most recent version of the Metabolon platform (HD4).

#### Association analysis of major depressive disorder with dietary sources of the depression-associated metabolites in the UK Biobank

Food proxies for the depression-associated metabolites were identified through the literature. We used logistic regression analysis to test the association between major depressive disorder and dietary sources of metabolites. Age, sex, body mass index (BMI), socio-economic status and principal components (PCs) were used as covariates in the analysis. For the association of current depressive symptoms, we used linear regression analysis. Since vitamin A is fat-soluble and can cross the blood-brain barrier, we performed additional association with white matter hyperintensity (WMH) volume – a measure of brain damage and also associated with MDD -- assessed 4–8 years after the baseline assessment. Linear regression analysis was used with the WMH volume as the dependent variable, vitamin supplements (vitamin A, and vitamin D as a negative control) as the independent variable, and age, sex, BMI, head size and principal components as covariates. All analyzes were performed in R.

#### Metabolites, their food sources and inflammation

Metabolites that were found to be associated with depression were tested for association with C-Reactive protein (CRP) as a measure of inflammation in EPIC-Norfolk, NEO and SHIP-Trend cohorts. CRP was natural log transformed and used as an outcome in a linear regression model adjusted for age and sex, and metabolite level as the independent variable. Further, food sources of metabolites were associated with baseline CRP levels in the UK Biobank cohort in a linear regression analysis adjusted for age, sex, BMI, socio-economic status and PCs.

#### Metabolite GWAS for Mendelian Randomization (MR) analysis

To test for association between metabolite levels and genotypes, we built linear regression models where the outcome was defined as the transformed level of each metabolite, predicted by the allele dosage at each polymorphic (MAF > 0.01) genotyped or imputed genetic variant. In addition, analyzes were adjusted for age, sex and BMI. All analyzes were conducted using the PLINK software (https://www.cog-genomics.org/plink/2.0/).

#### Mendelian Randomization (MR) analysis

To understand the relationship between the identified metabolites and major depression we performed bidirectional two-sample MR analysis. For major depression we used the independent genome-wide significant single nucleotide polymorphisms (SNPs) reported by Howard et al. [6] as instrumental variables. Summary statistics for these IVs were extracted from Howard et al. The summary statistics for the metabolites were extracted from the GWAS performed in UK Bioresource. Of the identified metabolites in this study (model 3), GWAS results were available for six metabolites including 2-aminooctanoate, 10-undecenoate (11:1n1), 1-palmitoyl-2-palmitoleoyl-GPC (16:0/16:1), hippurate, mannitol/sorbitol and retinol (Supplementary Table 2). The instrumental variables for these six metabolites and their summary statistics were extracted from the same GWAS. Because of scarcity in GWAS-grade significance for SNPs associated with these metabolites, we used independent SNPs that showed the strongest association with a *p*-value < 10^−06^ as instruments (Supplementary Table 3). The summary statistics for depression for these instrumental variables were extracted from the publicly available dataset (2019 PGC UKB Depression Genome-wide; https://www.med.unc.edu/pgc/download-results/mdd/). For the analysis we used the ‘mr_allmethods’ option of the R (https://cran.r-project.org/) library “MendelianRandomization” [35] that reports the results from the median method (simple, weighted and penalized), Inverse variance weighted and Egger methods (penalized, robust and penalized & robust).

#### Effect of antidepressant therapy on metabolites in PReDICT study

To examine the strength and significance of metabolite concentration changes within each of the three treatment arms, i.e., (1) CBT (16 sessions); (2) duloxetine (30–60 mg/d); or (3) escitalopram (10–20 mg/d), linear mixed effect models (with random intercept) with metabolite levels (in log scale) as the dependent variable, were fitted while correcting for age, sex, BMI, and baseline HRSD17. Then, the R package “emmeans” was used to compute the least squared means of the contrasts of interest (week 12 vs. baseline) and their corresponding *p*-values.

To detect whether metabolites levels were associated with clinical outcomes, linear regression analyzes corrected for age, sex and treatment arm were performed. Dependent variables (Baseline HRSD17, Week 12 HRSD17, and 12 weeks change in HRSD17) were regressed on either of following independent variables: 1) baseline metabolite, 2) week 12 metabolite, 3) 2 weeks change in metabolites and 4) 12 weeks change in metabolites.

#### Linking metabolites to human and/or gut metabolism

To assess whether the identified metabolites are products of human metabolism, gut microbial metabolism, or both, we integrated our findings with those of the VMH and Assembly of Gut Organisms through Reconstruction and Analysis (AGORA2) databases. Additional information is provided in the Supplementary Materials.

## Results

This study includes 13,596 participants from five independent cohorts including the Rotterdam Study (RS), the Study of Health in Pomerania (SHIP-TREND), the Cooperative Health Research in the Region of Augsburg (KORA) study, the European Prospective Investigation into Cancer (EPIC)-Norfolk Study, and the Netherlands Epidemiology of Obesity (NEO) study. A detailed description of the study participants is provided in Table 1. Depression was measured on a quantitative scale in all cohorts except the EPIC-Norfolk study, where the participants reported depression on a yes/no scale. The mean age ranged from 50.1 years in SHIP-Trend to 73.1 years in the Rotterdam Study. The percentage of female participants (51–56%) and mean body mass index (BMI; between 26–28 kg/m^2^) were comparable between studies. There were differences in the percentage of smokers between the cohorts, ranging from 11% in EPIC-Norfolk and to 22% in SHIP-Trend.

**Fig. 2:**
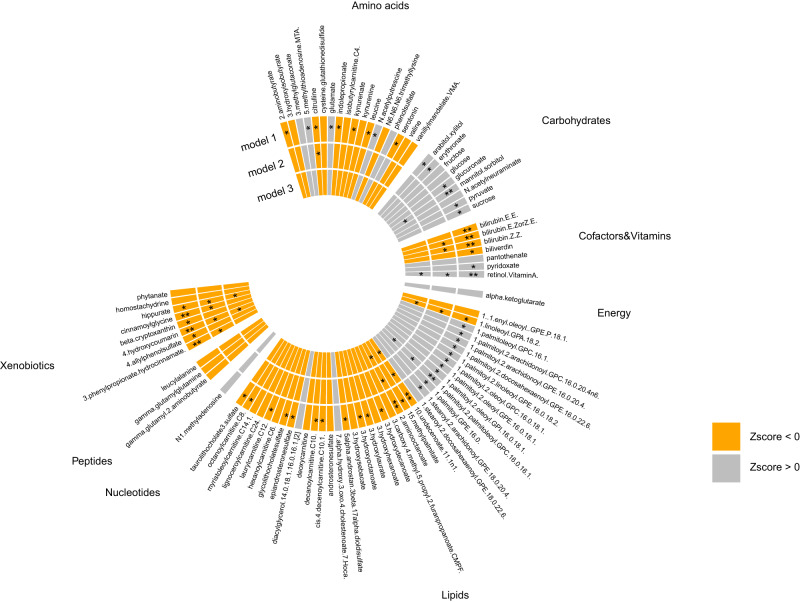
Results of Metabolome-wide association analysis. This plot shows the top findings of the association analysis of metabolites with depressive symptoms, for all three models tested. Only metabolites with FDR *p*-value < 0.1 are shown in this Figure. The associations with a negative Z-score are depicted in gray, while the positive associations are depicted in orange. The plot is divided per metabolite subgroup. Significance levels: **:FDR < 0.001, *:FDR < 0.05. Script for Figure modified from Nath et al. [100].

When testing for an association with depression adjusting for age and sex, 53 (41 novel) metabolites were significantly associated with depression after adjusting for multiple testing (false discovery rate (FDR) < 0.05; Table 2 & Fig. 2). These include nine metabolites in the amino acid metabolism pathway including five previously associated with depression (leucine, kynurenate, citrulline, glutamate and serotonin) [23, 36, 37]. In addition, significant association was found for six carbohydrates (one novel), six cofactors and vitamins, all of which were novel, 26 lipids (25 novel), and six xenobiotics (five novel) (Table 2).

**Table 2:** Top findings of the association analysis of metabolites with depression (FDR corrected *p-value* < 0.05 in model 1).

			Model 1				Model 2				Model 3			
Chemical ID	Name	Super pathway	*N*	Zscore	Direction^a^	FDR	*N*	Zscore	Direction^a^	FDR	*N*	Zscore	Direction^a^	FDR
100001197	10-undecenoate (11:1n1)	Lipid	13,596	−5.12	+-----	8.02E-05	13,556	−3.94	+-----	1.7E-02	13549	−3.79	+-----	2.71E-02
100001740	mannitol/sorbitol	Carbohydrate	12,631	5.14	+?-+++	8.02E-05	12,592	3.39	+?-+++	5.02E-02	12586	3.58	+?-+++	4.47E-02
1090	bilirubin (Z,Z)	Cofactors and Vitamins	13,596	−5.33	------	8.02E-05	13,556	−3.60	------	3.5E-02	13549	−2.84	------	1.83E-01
100001950	bilirubin (E,E)^a^	Cofactors and Vitamins	13,596	−5.25	------	8.02E-05	13,556	−3.33	------	5.13E-02	13549	−2.73	------	2.08E-01
100002049	4-hydroxycoumarin	Xenobiotics	10,885	−4.99	-??---	1.30E-04	10,847	−4.48	-??---	4.0E-03	10847	−4.17	-??---	1.12E-02
100008984	1-palmitoyl-2-palmitoleoyl-GPC (16:0/16:1)^a^	Lipid	10,885	4.84	+??+++	1.94E-04	10,847	3.58	+??+++	3.5E-02	10847	3.51	+??+++	4.47E-02
100001951	bilirubin (E,Z or Z,E)^a^	Cofactors and Vitamins	12,631	−4.74	-?----	2.83E-04	12,592	−3.50	-?----	3.5E-02	12586	−2.98	-?----	1.37E-01
498	retinol (Vitamin A)	Cofactors and Vitamins	10,885	4.65	+??+++	3.58E-04	10,847	3.89	+??+++	1.7E-02	10847	4.14	+??+++	1.12E-02
100004227	2-aminooctanoate	Lipid	11,850	−4.65	--?---	3.58E-04	11,811	−4.00	--?---	1.7E-02	11810	−3.92	--?---	1.87E-02
100002253	cinnamoylglycine	Xenobiotics	10,885	−4.40	-??---	9.38E-04	10,847	−3.50	-??---	3.5E-02	10847	−3.32	-??---	6.71E-02
100000010	3-phenylpropionate (hydrocinnamate)	Xenobiotics	13,596	−4.42	------	9.38E-04	13,556	−3.73	------	2.9E-02	13549	−3.24	--+---	7.89E-02
100001251	decanoylcarnitine (C10)	Lipid	13,596	−4.35	+-----	1.02E-03	13,556	−3.35	+-----	5.13E-02	13549	−3.15	+-----	8.53E-02
1526	1-palmitoyl-2-oleoyl-GPE (16:0/18:1)	Lipid	10,885	4.36	+??+++	1.02E-03	10,847	2.64	-??+++	2.12E-01	10847	2.44	-??+++	2.68E-01
250	biliverdin	Cofactors and Vitamins	13,596	−4.29	------	1.24E-03	13,556	−2.88	------	1.40E-01	13549	−2.41	------	2.75E-01
504	serotonin	Amino acid	12,631	−4.24	-?----	1.49E-03	12,592	0.18	-?-+++	9.55E-01	12586	−0.02	-?-+++	9.92E-01
192	N-acetylputrescine	Amino acid	10,885	4.06	-??+++	3.01E-03	10,847	2.09	-??-++	3.52E-01	10847	2.16	-??+++	3.44E-01
100002259	cis-4-decenoylcarnitine (C10:1)	Lipid	13,596	−4.02	------	3.27E-03	13,556	−2.80	------	1.58E-01	13549	−2.47	------	2.68E-01
212	5-methylthioadenosine (MTA)	Amino acid	10,885	4.03	+??+++	3.27E-03	10,847	2.09	+??+++	3.52E-01	10847	1.78	+??+++	4.91E-01
100000997	3-hydroxydecanoate	Lipid	11,850	−3.97	+-?---	3.89E-03	11,811	−2.60	+-?---	2.23E-01	11810	−2.39	+-?---	2.75E-01
1539	1-palmitoyl-2-oleoyl-GPC (16:0/18:1)	Lipid	10,885	3.85	+??++-	6.01E-03	10,847	2.93	+??++-	1.36E-01	10847	2.76	+??++-	2.03E-01
100000014	hippurate	Xenobiotics	13,596	−3.80	------	7.10E-03	13,556	−4.17	------	1.1E-02	13549	−3.72	------	2.99E-02
100001392	laurylcarnitine (C12)	Lipid	13,596	−3.73	+-----	8.97E-03	13,556	−2.68	+-----	1.96E-01	13549	−2.61	+-----	2.48E-01
1128	2-aminobutyrate	Amino acid	13,596	−3.68	------	9.61E-03	13,556	−2.08	---+--	3.54E-01	13549	−1.84	---+--	4.74E-01
561	glutamate	Amino acid	13,596	3.70	++++++	9.61E-03	13,556	1.92	++++-+	4.13E-01	13549	1.15	++++-+	7.26E-01
98	kynurenate	Amino acid	10,885	−3.61	-??---	1.22E-02	10,847	−2.79	-??---	1.58E-01	10847	−2.50	-??---	2.68E-01
100001658	taurolithocholate 3-sulfate	Lipid	13,596	−3.52	------	1.45E-02	13,556	−3.09	------	9.31E-02	13549	−3.09	---+--	1.01E-01
100001511	1-palmitoleoyl-GPC (16:1)^a^	Lipid	13,596	3.54	+++++-	1.45E-02	13,556	2.43	+++++-	2.74E-01	13549	2.57	++++++	2.48E-01
100001112	3-hydroxylaurate	Lipid	10,885	−3.53	-??---	1.45E-02	10,847	−2.54	-??---	2.42E-01	10847	−2.43	-??---	2.69E-01
100008990	1-palmitoyl-2-arachidonoyl-GPE (16:0/20:4)^a^	Lipid	10,885	3.52	-??+++	1.45E-02	10,847	2.22	-??+++	3.00E-01	10847	2.23	-??+++	3.27E-01
100000773	3-hydroxyoctanoate	Lipid	11,850	−3.54	+-?---	1.45E-02	11,811	−2.25	+-?---	3.00E-01	11810	−2.13	+-?---	3.63E-01
100001868	4-allylphenol sulfate	Xenobiotics	10,885	−3.48	-??---	1.58E-02	10,847	−2.86	-??-+-	1.46E-01	10847	−2.46	-??-+-	2.68E-01
100001247	octanoylcarnitine (C8)	Lipid	13,596	−3.47	+-----	1.58E-02	13,556	−2.62	+-----	2.14E-01	13549	−2.48	+-----	2.68E-01
100001083	indolepropionate	Amino acid	13,596	−3.48	------	1.58E-02	13,556	−2.95	------	1.35E-01	13549	−2.12	------	3.66E-01
100001977	beta-cryptoxanthin	Xenobiotics	6246	−3.46	-???--	1.60E-02	6208	−3.25	-???--	5.74E-02	6208	−2.59	-???--	2.48E-01
100006430	arabitol/xylitol	Carbohydrate	12,631	3.44	+?++++	1.65E-02	12,592	2.48	+?++++	2.51E-01	12586	2.69	+?++++	2.15E-01
100009082	1-linoleoyl-GPA (18:2)^a^	Lipid	10,885	−3.39	-??---	1.91E-02	10,847	−3.57	-??---	3.5E-02	10847	−3.53	-??---	4.47E-02
100001870	1-palmitoyl-2-linoleoyl-GPE (16:0/18:2)	Lipid	10,885	3.39	+??+++	1.91E-02	10,847	2.02	+??+++	3.78E-01	10847	2.18	+??+++	3.41E-01
100000257	glucuronate	Carbohydrate	10,885	3.35	+??+++	2.18E-02	10,847	2.09	+??+++	3.52E-01	10847	1.93	+??+++	4.51E-01
391	citrulline	Amino acid	13,596	−3.31	------	2.45E-02	13,556	−3.53	------	3.5E-02	13549	−3.29	------	7.22E-02
100001121	pyridoxate	Cofactors and Vitamins	13,596	3.29	++-++-	2.50E-02	13,556	2.71	++-++-	1.83E-01	13549	3.17	++-++-	8.53E-02
100002021	5alpha-androstan-3beta,17alpha-diol disulfate	Lipid	10,885	−3.25	-??---	2.79E-02	10,847	−2.05	-??---	3.67E-01	10847	−1.63	-??---	5.35E-01
100001287	epiandrosterone sulfate	Lipid	13,596	−3.16	-+----	3.59E-02	13,556	−1.71	-+----	4.70E-01	13549	−1.73	-+----	5.03E-01
100008914	1-palmitoyl-2-arachidonoyl-GPC (16:0/20:4n6)	Lipid	10,885	3.13	-??+++	3.89E-02	10,847	2.92	-??+++	1.36E-01	10847	2.94	-??++-	1.52E-01
100001320	erythronate^a^	Carbohydrate	12,631	3.12	+?++++	3.89E-02	12,592	1.76	+?++++	4.55E-01	12586	1.77	+?++++	4.91E-01
935	sucrose	Carbohydrate	10,885	3.13	+??+++	3.89E-02	10,847	1.61	+??+-+	4.99E-01	10847	1.16	+??+-+	7.21E-01
100001567	1-palmitoyl-GPE (16:0)	Lipid	13,596	3.10	++-++-	4.01E-02	13,556	2.15	++-++-	3.28E-01	13549	2.57	++++++	2.48E-01
100008991	1-palmitoyl-2-docosahexaenoyl-GPE (16.0/22.6)	Lipid	10,401	3.06	???+++	4.26E-02	10,401	2.43	???+++	2.74E-01	10401	2.71	???+++	2.15E-01
100001657	glycolithocholate sulfate^a^	Lipid	11,850	−3.06	--?--+	4.26E-02	11,811	−2.46	--?---	2.60E-01	11810	−2.29	--?---	3.09E-01
100008977	1-stearoyl-2-arachidonoyl-GPE (18:0/20:4)	Lipid	10,885	3.07	-??+++	4.26E-02	10,847	1.94	-??+++	4.07E-01	10847	1.93	-??+++	4.51E-01
823	pyruvate	Carbohydrate	13,596	3.06	++++-+	4.26E-02	13,556	2.28	++++-+	2.91E-01	13549	1.84	+-++-+	4.74E-01
397	leucine	Amino acid	13,596	−3.04	--+--+	4.44E-02	13,556	−2.73	--+--+	1.76E-01	13549	−3.33	------	6.71E-02
100002945	15-methylpalmitate	Lipid	13,596	−3.04	+-----	4.44E-02	13,556	−2.77	+-----	1.63E-01	13549	−2.40	+-----	2.75E-01
100009066	1-palmitoyl-2-oleoyl-GPI (16:0/18:1)^a^	Lipid	6246	3.03	+???++	4.50E-02	6208	1.96	+???+-	4.01E-01	6208	1.86	+???+-	4.66E-01

^a^The order of the direction column: Rotterdam Study, SHIP-trend, KORA, EPIC-Norfolk B2, EPIC-Norfolk B3, NEO.

When adjusting for antidepressant use (model 2), 12 metabolites remained significantly associated (FDR < 0.05) with depression (Table 2, Fig. 2), suggesting that most associations observed with depression were confounded by antidepressant medication use. Of the amino acids, only citrulline remained significantly associated with depression after adjustment for antidepressant medication (Table 2, Fig. 2). Other metabolites that remained significantly associated with depression in the extended model included four xenobiotics (4-hydroxycoumarin, hippurate, 3-phenylpropionate (hydrocinnamate) and cinnamoylglycine), four lipids (2-aminooctanoate, 10-undecenoate (11:1n1), 1-palmitoyl-2-palmitoleoyl-GPC (16:0/16:1) and 1-linoleoyl-GPA (18:2)), and three cofactors and vitamins (retinol (vitamin A), bilirubin (Z,Z), bilirubin (E,Z or Z,E)). Among these, higher levels of 1-palmitoyl-2-palmitoleoyl-GPC (16:0/16:1) and retinol (vitamin A) were associated with an increased risk of depression, while the others were associated with a decreased risk (Fig. 2).

We subsequently build a more conservative model, further adjusting for other medication use, including lipid-lowering medication, antihypertensive medication, antidiabetic medication, BMI and current smoking (model 3). Seven out of the 12 metabolites remained significantly associated with depression (Table 2). These included retinol (vitamin A), hippurate, 4-hydroxycoumarin (coumarol), 2-aminooctanoate (alpha-aminocaprylic acid), 10-undecenoate (11:1n1) (undecylenic acid), 1-palmitoyl-2-palmitoleoyl-GPC (16:0/16:1) (Lecithin), and 1-linoleoyl-GPA (18:2) (lysophosphatidic acid). Additionally, mannitol/sorbitol appeared statistically significant in model 3. Complete results of the meta-analysis are available in Supplementary Table 4.

There was no significant evidence for effect modification by sex (Supplementary Table 5) and the directionality of effects tended to be consistent in men and women. Effect sizes appeared to be stronger in women. Results were consistent across various versions of the Metabolon platform and depression assessing instruments and a sensitivity meta-analysis, which only included results from cohorts that had assessed metabolites on the most recent (HD4) platform, showed that they remained essentially unchanged (Supplementary Table 6).

### Association of depression with dietary sources of metabolites in the UK Biobank

For hippurate, fresh fruits and vegetables were identified as the primary source [38]. For 4-hydroxycoumarin vitamin K antagonists were identified as the primary source [39, 40]. For vitamin A/retinol UK Biobank had both vitamin supplement intake and retinol intake from food available. Egg yolk was identified as the primary source of lecithin [41], legumes a proxy for lysophosphatidic acid [42] and artificial sweeteners were identified as the primary source of mannitol/sorbitol [43]. We were unable to identify food proxies for undecylenic acid and alpha-aminocaprylic acid. In a cross-sectional analysis in the UK Biobank, we found a significant positive association of vitamin A intake from supplements with both measures of depression including current depressive symptoms (beta = 0.23, *p*-value = 1.25 × 10^−25^) and lifetime MDD (OR = 1.40, *p*-value = 9.72 × 10^−18^). However, vitamin D supplement intake (negative control) was also significantly positively associated with both measures of depression (Table 3), suggesting that depressed individuals take more vitamin supplements than non-depressed individuals do. Since both vitamin A and vitamin D are fat-soluble and can cross the blood-brain barrier, we performed additional association with the depression-associated brain pathology, i.e., white matter hyperintensity (WMH) volume. Only vitamin A supplement intake was found to be associated with higher volume of WMH (beta = 490.49, *p*-value = 0.04, Supplementary Table 7), suggesting a possible role of vitamin A in brain damage. To address the issue of reverse causality we observed in case of supplement use, we additionally tested the association of depression with retinol intake estimated from the food consumed in the previous 24 h. Significant positive association of estimated retinol intake was observed with both measures of depression (current depressive symptoms, *p*-value = 1.26 × 10^−08^; lifetime MDD, *p*-value = 1.4 × 10^−03^). However, the effect estimates were small (Table 3), which may in part be explained by the imprecision of food consumption questionnaires. The primary sources of hippurate, i.e., fresh fruits (*β* = −0.058, *P*-value = 3.29 × 10^−191^) and vegetables (*β* = −0.029, *P*-value = 2.11 × 10^−82^) intake was significantly reduced in individuals experiencing depressive symptoms at the time of assessment. Further fresh fruits intake was also significantly reduced in individuals who had MDD (*β* = −0.035, *P*-value = 1.42 × 10^−19^). Artificial sweetener – source of mannitol/sorbitol – intake was significantly increased in individuals experiencing symptoms of depression (*β* = 0.165, *P*-value = 2.8 × 10^−^^21^) and those with MDD (*β* = 0.193, *P*-value = 3.65 × 10^−16^). Intake of legumes (source of lysophosphatidic acid) was inversely associated with current depressive symptoms (*β* = −0.063, *P*-value = 0.03) and positively associated with lifetime MDD (*β* = 0.108, *P*-value = 0.008). Moreover, consumption of eggs – a source of lecithin -- was increased both in individuals experiencing symptoms of depression at the time of assessment (*β* = 0.05, *P*-value = 0.001) and lifetime MDD (*β* = 0.05, *P*-value = 0.018). Use of vitamin K antagonists, a proxy for 4-hydroxycoumarin, was positively associated with current depressive symptoms (beta = 0.43, *p*-value = 1.04 × 10^−46^) but not with lifetime MDD (Table 3).

**Table 3:** Results of association of depression outcomes with dietary sources of metabolites in the UK Biobank.

		Current depressive symptoms				Lifetime MDD			
Metabolite	Food Proxy	Beta	SE	*P*-value	*N*	Beta	SE	*P*-value	*N*
Retinol	Vitamin A supplements	0.243928	0.022056	2.00E-28	303,274	0.351808	0.039820	1.00E-18	189,118
Retinol	Retinol intake from food	0.000227	0.000040	1.12E-08	61,201	0.000170	0.000055	2.14E-03	38,648
Negative Control	Vitamin D supplements	0.236158	0.015930	1.05E-49	311,932	0.370265	0.028033	7.87E-40	193,957
Hippurate	Fresh Fruits	−0.058028	0.001966	3.29E-191	433,153	−0.035044	0.003872	1.42E-19	263,822
Hippurate	Raw Vegetables	−0.028271	0.001470	2.11E-82	430,354	0.004597	0.002687	8.71E-02	261,767
4-hydroxycoumarin	Vitamin K antagonists	0.315052	0.029455	1.07E-26	434,232	0.054569	0.057843	3.45E-01	264,658
mannitol/sorbitol	Artificial sweetener	0.164990	0.017416	2.80E-21	63,079	0.193453	0.023738	3.65E-16	39,829
1-linoleoyl-GPA (18:2)	Legumes	−0.063329	0.029184	3.00E-02	63,079	0.107547	0.040704	8.24E-03	39,829
1-palmitoyl-2-palmitoleoyl-GPC (16:0/16:1)	Eggs	0.049938	0.015835	1.61E-03	63,079	0.052034	0.021952	1.78E-02	39,829

#### Metabolites, their food sources and inflammation

Consistent with the association of depression, levels of hippurate (*β* = −0.09, *P*-value = 0), alpha-aminocaprylic acid (*β* = −0.08, *P*-value = 2.5 × 10^−^^12^), lysophosphatidic acid (*β*= −0.15, *P-*value = 0) and 4-hydroxycoumarin (*β* = −0.09, *P*-value = 3.3 × 10^−16^) were all significantly associated with reduced levels of CRP (Supplementary Table 8). Retinol levels were associated with reduced levels of CRP (*β* = −0.07, *P*-value = 3.3 × 10^−10^). Further food sources of hippurate, retinol, lysophosphatidic acid, mannitol/sorbitol and lecithin including fresh fruits (*β* = −0.04, *P*-value = 0), vegetables (*β* = −0.02, *P*-value = 1.2 × 10^−199^), retinol intake from food (*β* = 6.0 × 10^−05^, *P*-value = 6.7 × 10^−04^), eggs (*β* = 0.04, *P*-value = 7.9 × 10^−07^) and artificial sweetener use (*β* = 0.02, *P*-value = 0.012) were all significantly associated with CRP and consistent with the findings of depression (Supplementary Table 9).

### Mendelian randomization analysis

Testing the hypothesis that major depression results in changes of circulating metabolites in the Mendelian randomization analysis (MR), nominally significant results were obtained for 2-aminooctanoate and 10-undecenoate (11:1n1), under the MR-Egger method and weighted median method, respectively. However, these findings did not remain significant after adjustment for multiple testing (Supplementary Table 10). MR models in which we tested the hypothesis that levels of circulating metabolites increase the risk of depression provided significant evidence for a causal relation between hippurate and the risk of depression, both in the MR-Egger robust and penalized-robust methods (Supplementary Table 11). The effect estimate was consistent with the inverse relationship observed between hippurate and major depression in this study. However, a significant intercept was also observed suggesting pleiotropy. To exclude a pleiotropic effect, we studied the effect of intervention on the metabolite in the PReDICT trial.

### Effect of antidepressant therapy on hippurate

To further evaluate the impact of antidepressant therapy including cognitive behavioral therapy (CBT), duloxetine – a serotonin-norepinephrine reuptake inhibitor (SNRI) and escitalopram – a selective serotonin reuptake inhibitor (SSRI) on hippurate we consulted the PReDICT study. The PReDICT study allows us to test the effect of antidepressant therapy on the metabolite levels in circulation by measuring the metabolite levels before and after the antidepressant therapy. In PReDICT, we found that levels of hippurate in the circulation increased significantly from baseline to week 12 only after treatment with escitalopram (estimated week 12 vs. baseline difference = 0.45, 95% confidence interval (CI; 0.16,0.74), *p*-value = 0.002; Supplementary Fig. 1), but not in the cognitive behavior therapy (CBT) and duloxetine treatment arms (CBT: estimated difference = −0.02, 95% CI (−0.39,0.33) and *p*-value = 0.87; duloxetine: estimated difference = 0.13, 95% CI (−0.17,0.44) and *p*-value = 0.38). In this study, we could not show a relation between hippurate and depression as the study recruited patients only and lacked healthy controls. In patients receiving pharmacotherapy (escitalopram and duloxetine arms), the association of baseline depression as measured by the 17-item Hamilton Rating Scale for Depression (HRSD17) and baseline hippurate was not statistically significant (beta = 0.04, 95% CI (−0.03,0.11), *p*-value = 0.27). Further, no significant association was observed between depression in week 12 as measured by the HRSD17 and week 12 hippurate (beta = 0.09, *p*-value = 0.45) and 12 weeks change in HRSD17 and 12 weeks change in hippurate (beta = 0.02, 95% CI (−0.65, 1.57), *p*-value = 0.85).

### Linking the human circulating metabolome to gut microbiome metabolism

Of the 53 metabolites identified in this study in model 1, 28 metabolites could be matched to a unique VMH metabolite ID. For each metabolite, the presence or absence in the global human reconstruction, Recon3D [44], and a resource of 7206 reconstructions of human gut microbes, AGORA2 (https://www.biorxiv.org/content/10.1101/2020.11.09.375451v1) was retrieved. In total, 12 metabolites were present in both the human and gut microbial metabolic networks, three were only present in gut microbes, and 13 were only present in human (Supplementary Table 12). To further investigate potential links between the microbiome and metabolites associated with depression, the potential of the 7206 AGORA2 strains to consume or secrete the 15 microbial metabolites identified in this study was computed. Since hippurate is synthesized in the liver and renal cortex from the microbial metabolite benzoate [45], the uptake and secretion potential for benzoate was also predicted for the 7206 strains.

A wide range of genera and species were involved in the uptake of mannitol (Supplementary Table 13, Supplementary Fig. 2). Mannitol is largely secreted by several species of the genus *Bacteroides* followed by *Lactobacillus*, among others (Supplementary Table 13). Both genera have previously been found to be associated with depression [46]. In total, 3616 AGORA2 strains mainly of the Gammaproteobacteria and Bacilli classes (Supplementary Table 14, Supplementary Fig. 2) synthesized benzoate as a product of benzamide (VMH reaction ID: BZAMAH). Interestingly, benzamides are a class of antipsychotic medication.

## Discussion

In this study, we identified novel associations with depression for six metabolites, including retinol (vitamin A), 4-hydroxycoumarin, 2-aminooctanoate, 10-undecenoate (11:1n1), 1-palmitoyl-2-palmitoleoyl-GPC (16:0/16:1), 1-linoleoyl-GPA (18:2) and confirmed the association of hippurate and mannitol/sorbitol. We found that the relation of hippurate and depression may be causal and that hippurate levels can be modified by a specific antidepressant, escitalopram. Further, consistent with the association of the metabolites with depression, the consumption of dietary sources of hippurate and 1-linoleoyl-GPA (18:2) including fresh fruits, vegetables and legumes was significantly reduced in individuals with higher depressive symptoms, and associated with significantly decreased levels of CRP. In addition, the consumption of dietary sources of retinol, mannitol/sorbitol and 1-palmitoyl-2-palmitoleoyl-GPC (16:0/16:1) including retinol score estimated from diet, artificial sweeteners and eggs was significantly increased in individuals with higher depressive symptoms and associated with increased blood levels of CRP.

One of the most interesting findings of this study is the identification of the association of higher levels of retinol (active form of vitamin A) with depression. There have been several case reports of individuals with vitamin A intoxication with no previous history of depression, who developed symptoms of depression and even psychosis when overdosed with vitamin A [47, 48]. Depressive symptoms resolved upon discontinuation of vitamin A, implying that depression may be a side effect of vitamin A intake [47]. Animal models have suggested elevated monoamine oxidase enzyme activity and depression-related behavior upon vitamin A supplementation [49, 50]. Our study is the first to link higher levels of retinol in blood with depression in the general population. Retinol and its derivatives known as retinoids are lipid soluble and can cross the blood-brain barrier. Vitamin A is required for brain development and functioning [51, 52]. However, excess of vitamin A is neurotoxic and may result in brain shrinkage [52]. Brain areas high in retinoic acid signaling and receptors overlap with areas of relevance to stress and depression [53]. Further, vitamin A is known to increase the synthesis of triglyceride-rich very low-density lipoproteins (VLDLs) and apolipoproteins in the serum [54, 55], which we found associated with depression in our previous study [21]. Since food is the primary source of vitamin A, an important question to answer is whether vitamin A intake is associated to depression. In the UK Biobank, we found significant increase in dietary retinol intake in individuals with depression. Thus, our findings ask for intervention studies that evaluate prospectively the effect of vitamin A reduction in depressed patients.

Two of the most strongly associated metabolites with depression were xenobiotics, hippurate and 4-hydroxycoumarin. In line with the findings of our study, decreased urine and plasma levels of hippurate have consistently been associated with unipolar and bipolar depression in several studies and it has been suggested as a biomarker for depressive disorders [23]. Our MR analysis suggests that low hippurate levels in circulation are a part of the causal pathway leading to depression. However, as the MR could not exclude a pleiotropic effect, our findings yield a hypothesis that requires further evaluation in a clinical trial. While we could not show an association between hippurate and depression in the PReDICT study, as the study lacked healthy controls, hippurate levels were higher 12 weeks after initiation of SSRI therapy (escitalopram) but not for SNRI or CBT, raising the question whether blood levels of hippurate can be used in clinical trials for compliance and efficacy of SSRIs specifically. Hippurate is derived from benzoate and polyphenols and is reported to be a metabolomic marker of gut microbiome diversity [38], fruits and vegetables intake [56], diet quality [57] and metabolic health [58]. In line with the decreased levels of hippurate in depressed individuals found in our metabolome-wide association analysis, we found significantly decreased fresh fruit intake among individuals with depression in the UKB, which is consistent with the findings of the previous studies that high consumption of fruits, vegetables, nuts, and legumes is associated with a reduced risk of depression [8, 59]. Further, both hippurate and its food sources including fresh fruits and vegetables were associated with significantly reduced inflammation in our study, which is in line with the findings of the previous clinical trials [60]. We hypothesize that this reduction is mediated through hippurate, which requires evaluation in future studies.

The metabolite 4-hydroxycoumarin is a fungal derivative of coumarin. Coumarins are found naturally in plants and spices [61] and coumarin is converted into 4-hydroxycoumarin by fungi [39]. 4-hydroxycoumarin is then converted into dicoumarol in the presence of formaldehyde [39]. Dicoumarol is an anticoagulant (warfarin) that inhibits the synthesis of vitamin K, also called vitamin K antagonist, and is commonly used to treat thromboembolic diseases [40]. In the UKB, we found significant positive association of anticoagulant use (vitamin K antagonists) with major depression. A history of depression is a risk factor for thromboembolism [62–64]. Antidepressants are also known to interact with warfarin [65] and are also associated with increased risk of thromboembolism [66]. Taking all findings together, we hypothesize that depression/antidepressant use depletes 4-hydroxycoumarin in circulation leading to thromboembolism. Vitamin K has been shown to act in the nervous system as it is involved in sphingolipid synthesis [67]. Sphingolipids are present in high concentrations in cell membranes of neuronal and glial cells [68]. Sphingolipids are essential for important cellular events, including proliferation, differentiation, senescence, cell-cell interactions, and transformation [69] and they have been linked to aging, Alzheimer’s disease, and Parkinson’s disease [70–72]. Further, sphingolipids were found to play a crucial role in the development of depression- and anxiety-related behaviors in mice [73, 74] and depression is seen often in patients with sphingolipid storage diseases [75–79]. Treatment with escitalopram /citalopram is also associated with changes in sphingolipids [80]. In our study, we did not find an association of depression with circulating sphingolipids present on the Metabolon platform. However, we cannot exclude that 4-hydroxycoumarin in the blood affects sphingolipid metabolism in the brain specifically.

Other metabolites that were found to be significant in our study include mannitol/sorbitol, of which increased levels were associated with depression. Higher levels of sorbitol in plasma and urine have previously been consistently reported in patients with unipolar and bipolar depression and, like hippurate, it has been suggested as a diagnostic biomarker of depression [23]. Mannitol/sorbitol are sugar alcohols found in food such as fruits and berries and often used in diet/sugar free foods as sweeteners [81]. Fructose reduced diets have been shown to improve gastrointestinal disorders, depression and mood disorders [82]. Our AGORA2 analysis suggests that mannitol is mainly secreted by several species of *Bacteroides*, *Lactobacillus*, *Fructobacillus*, *Alistipes* and *Bifidobacterium*. Interestingly, all genera, except for *Fructobacillus* have previously been associated with depression [46], asking for further studies on the role of the microbiome, circulating levels of mannitol and depression.

Finally, there were four lipids identified in our study (2-aminooctanoate, 10-undecenoate (11:1n1), 1-palmitoyl-2-palmitoleoyl-GPC (16:0/16:1) and 1-linoleoyl-GPA (18:2)) significantly associated with depression. 1-Palmitoyl-2-palmitoleoyl-GPC (16:0/16:1) also known as phosphatidylcholine (16:0/16:1) or lecithin is commonly found in foods like egg yolk and soybean, and is a precursor of choline. Lecithin is believed to cause depression by increasing the production of acetylcholine in the brain [83]. When fed to animals and humans, lecithin significantly increases the levels of choline in blood and brain and of acetylcholine in brain [84–86]. Our study is the first to show higher circulating levels of lecithin in the depressed individuals from the general population. The other three lipids 2-aminooctanoate, 10-undecenoate (11:1n1) and 1-linoleoyl-GPA (18:2) were negatively associated with depression. 2-Aminooctanoate (alpha-aminocaprylic acid) and 10-undecenoate (11:1n1) (undecylenic acid) are neutral hydrophobic molecules for which there is not much known in the literature. Lower levels of 10-undecenoate (11:1n1) have been found in individuals with non-alcoholic fatty liver disease [87]. 1-linoleoyl-GPA (18:2) is a lysophosphatidic acid (LPA 18:2). LPA is a bioactive membrane lipid that acts on at least six distinct G protein‐coupled receptors (LPA_1–6_) and plays a role in pain sensitivity and emotional regulation [88]. LPA knock out mice exhibit anxiety-related behaviour [88, 89].

We found that decreased plasma levels of serotonin, kynurenate, leucine and citrulline and higher levels of glutamate were associated with depression. Lower plasma/serum levels of serotonin, kynurenate, citrulline and leucine and higher levels of glutamate have been reported in relationship to depression in earlier studies [23, 36, 37, 90], which also appears consistent with our findings of model 1. However, we and others have shown that antidepressants affect plasma/serum levels of serotonin, glutamate, leucine and kynurenine [91–94]. An important finding of our study is that only citrulline remained significantly associated with depression after adjusting for antidepressant medication use, suggesting the other metabolites change as a results from the medication. Lower levels of citrulline and its precursor arginine were previously associated to depression in unmedicated individuals [37, 95]. Interestingly, treatment with SSRIs significantly increase the levels of plasma citrulline [80]. Further, levels of plasma citrulline were found to be significantly increased two hours post treatment with ketamine, suggesting a possible mechanism of action of the rapid acting drug [96]. Citrulline is an intermediate in the urea cycle and linked to nitric-oxide synthesis [97]. It is absorbed by the gut and has useful therapeutic effects against cardiovascular diseases [98]. In our study the association of citrulline with depression lost its significance, albeit not completely, after adjusting for cardiovascular medication use and BMI.

Our study is the first large-scale effort combining metabolites measured on assorted, untargeted metabolomics platforms (Metabolon) studied in relationship to depression. In addition to confirming several previously identified metabolites in smaller studies, we successfully identified novel metabolites that are associated with depression. Our findings are robust across different versions of the Metabolon platform or the criteria assessing presence of clinical or subclinical depression. A possible limitation of our study is that we have meta-analyzed summary statistics from multiple cohorts that are different in their baseline characteristics including age. Depression in old age is considered to have a different pathogenesis compared to depression in younger individuals. Further, there are differences in metabolomics platform versions and instruments that were used by different cohorts to assess depression, all of which have a negative impact on the statistical power of the study. Older versions of the Metabolon platform reported significantly fewer known metabolites compared to the more recent implementations. Another limitation of our study is the presence of residual confounding. Despite adjusting for most known confounders including medication use and the lifestyle factors smoking and BMI, confounding may still be present and may influence the results [99]. Further, our MR analysis was most likely underpowered lacking strong instrumental variables for the associated metabolites.

Analysing circulating levels of 806 metabolites from untargeted metabolomics platforms in 13,596 individuals, we identified six new associations of metabolites with depression including retinol (vitamin A), 4-hydroxycoumarin and four lipids, 2-aminooctanoate, 10-undecenoate (11:1n1), 1-palmitoyl-2-palmitoleoyl-GPC (16:0/16:1) and 1-linoleoyl-GPA (18:2), while confirming known associations of hippurate and mannitol/sorbitol. We further show that previously identified associations of depression with metabolites belonging to the amino-acid pathways including serotonin, kynurenate, leucine and glutamate are likely explained by antidepressant medication. Our findings point to effective preventive targets, as most of these metabolites are food derived and thus can be altered in patients by modifying diet.

### Data Sharing

The datasets generated during and/or analyzed during the current study are not publicly available due to restrictions based on privacy regulations and informed consent of the participants. All summary statistics are provided in the Supplementary Material. For individual cohort level data, respective Principal Investigators can be contacted. For the Rotterdam Study data, requests should be directed towards the management team of the Rotterdam Study (secretariat.epi@erasmusmc.nl), which has a protocol for approving data requests.

The EPIC-Norfolk data can be requested by bona fide researchers for specified scientific purposes via the study website (https://www.mrc-epid.cam.ac.uk/research/studies/epic-norfolk/). Data will either be shared through an institutional data sharing agreement or arrangements will be made for analyzes to be conducted remotely without the need for data transfer.

### Supplementary information

Supplement
