# Supplementary material for: Circulating metabolites modulated by diet are associated with depression
Source: Mol Psychiatry. 2023 Jul 26;28(9):3874–87. doi: 10.1038/s41380-023-02180-2 (PMC10730409; doi:10.1038/s41380-023-02180-2)
Supplement: Supplementary file 1 — Supplement [file 41380_2023_2180_MOESM1_ESM.pdf]

Supplementary Materials

"Circulating metabolites modulated by diet are associated with depression"

Ashley van der Spek[1,2#] (PhD), Isobel D Stewart[3#] (PhD), Brigitte Kühnel[4,5#] (PhD), Maik Pietzner[3,6#] (PhD), Tahani Alshehri[7#] (MSc), Friederike Gauß[6] (PhD), Pirro G Hysi[8] (PhD), Siamak MahmoudianDehkordi[9] (PhD), Almut Heinken[10] (PhD), Annemarie I. Luik[1] (PhD), Karl-Heinz Ladwig[5,11] (PhD), Gabi Kastenmüller[12,13] (PhD), Cristina Menni[8] (PhD), Johannes Hertel[10,14] (PhD), M Arfan Ikram[1] (PhD), Renée de Mutsert[7] (PhD), Karsten Suhre[15] (PhD), Christian Gieger[4,5,13] (PhD), Konstantin Strauch[16,17] (PhD), Henry Völzke[18] (PhD), Thomas Meitinger[19,20] (PhD), Massimo Mangino[8] (PhD), Antonia Flaquer[16,17] (PhD), Melanie Waldenberger[4,5,21] (PhD), Annette Peters[4,5,21,22] (PhD), Ines Thiele[10,23,24] (PhD), Rima Kaddurah-Daouk[9,25,26] (PhD), Boadie W Dunlop[27] (PhD), Frits R. Rosendaal[7] (PhD), Nicholas J Wareham[3] (PhD), Tim D Spector[8] (PhD), Sonja Kunze[4,5‡] (PhD), Hans Jörgen Grabe[14‡] (PhD), Dennis O Mook-Kanamori[28‡] (PhD), Claudia Langenberg[3‡] (PhD), Cornelia M van Duijn[1,29‡] (PhD), Najaf Amin[1,29‡]*(PhD)

1 Department of Epidemiology, Erasmus MC University Medical Center, Rotterdam, The Netherlands.
2 SkylineDx B.V., Rotterdam, The Netherlands.
3 MRC Epidemiology Unit, University of Cambridge, Cambridge, United Kingdom.
4 Research Unit of Molecular Epidemiology, Helmholtz Zentrum München, German Research Center for Environmental Health, D-85764, Neuherberg , Germany.
5 Institute of Epidemiology, Helmholtz Zentrum München, German Research Center for Environmental Health, D-85764, Neuherberg, Germany.
6 Institute of Clinical Chemistry and Laboratory Medicine, University Medicine Greifswald, Ferdinand-Sauerbruch-Str, 17475 Greifswald, Germany.
7 Department of Clinical Epidemiology, Leiden University Medical Center, Leiden, The Netherlands.
8 Department of Twins Research and Genetic Epidemiology, Kings College London, London, UK.
9 Department of Psychiatry and Behavioral Sciences, Duke University, Durham, NC, USA.
10 School of Medicine, National University of Ireland, Galway, University Road, Galway, Ireland.
11 Department of Psychosomatic Medicine and Psychotherapy, Klinikum rechts der Isar, Technische Universität München, Munich, Germany.
12 Institute of Computational Biology, Helmholtz Zentrum München, German Research Center for Environmental Health, D-85764 Neuherberg, Germany.
13 German Center for Diabetes Research (DZD e.V.), D-85764, Neuherberg, Germany.

14 Department of Psychiatry and Psychotherapy, University Medicine Greifswald, Ellernholzstrasse 1-2, 17489 Greifswald, Germany.
15 Department of Physiology and Biophysics, Weill Cornell Medicine-Qatar, Education City, PO 24144 Doha, Qatar.
16 Institute of Genetic Epidemiology, Helmholtz Zentrum München, German Research Center for Environmental Health, D-85764, Neuherberg, Germany.
17 Chair of Genetic Epidemiology, IBE, Faculty of Medicine, LMU Munich, Germany.
18 Institute of Community Medicine, University Medicine Greifswald, Walter-Rathenau Str. 48, 17475 Greifswald, Germany.
19 Institute of Human Genetics, Helmholtz Zentrum München, German Research Center for Environmental Health, D-85764, Neuherberg, Germany.
20 Institute of Human Genetics, Technische Universität München, Munich, Germany.
21 German Center for Cardiovascular Research (DZHK), Partner Site Munich Heart Alliance, Munich, Germany.
22 Ludwig-Maximilians-Universität München, IBE-Chair of Epidemiology, Munich, Germany.
23 Division of Microbiology, National University of Galway, Galway, Ireland.
24 APC Microbiome Ireland, Ireland.
25 Duke Institute of Brain Sciences, Duke University, Durham, NC, USA.
26 Department of Medicine, Duke University, Durham, NC, USA.
27 Department of Psychiatry and Behavioral Sciences, Emory University School of Medicine, Atlanta, GA, United States.
28 Department of Clinical Epidemiology, Department of Public Health and Primary Care, Leiden University Medical Center, Leiden, The Netherlands.
29 Nuffield Department of Population Health, University of Oxford, OX3 7LF, Oxford, United Kingdom.

# These authors contributed equally
ǂ These authors contributed equally

* Corresponding author:
Dr. Najaf Amin (najaf.amin@ndph.ox.ac.uk)
Nuffield Department of Population Health, Oxford University,
Richard Doll Building, OX3 7LF
Oxford, UK

Study populations

The Rotterdam Study: The Rotterdam Study is a population-based cohort study from the well-defined Ommoord district within Rotterdam, The Netherlands. It is designed to investigate occurrence and determinants of diseases in the elderly[1]. Initially, the RS included 7983 participants in 1990 who underwent an at-home interview, extensive physical examination at baseline and during follow-op examinations that occur every 3-4 years (RS-I). The RS was extended with two more cohorts in 2000 (RS-II) and 2005 (RS-III) and contains a total of 14,926 participants. In this study, data of the third follow-up visit of the first cohort (RSI-3) was used as metabolomic measurements were available for this cohort. The RS has been approved by the Medical Ethics Committee of the Erasmus MC (registration number MEC 02.1015) and by the Dutch Ministry of Health, Welfare and Sport (Population Screening Act WBO, license number 1071272-159521-PG). The RS has been entered into the Netherlands National Trial Register (NTR; www.trialregister.nl) and into the WHO International Clinical Trials Registry Platform (ICTRP; www.who.int/ictrp/network/primary/en/) under shared catalogue number NTR6831. All participants provided written informed consent to participate in the study and to have their information obtained from treating physicians.

The Study of Health in Pomerania (SHIP-Trend) study: The Study of Health in Pomerania (SHIP-TREND) is a population-based study located in West Pomerania, a rural region in north-east Germany[2]. A stratified random sample of 8826 adults aged 20-79 years was drawn from population registries. Sample selection was facilitated by centralization of local population registries in the Federal State of Mecklenburg-West Pomerania. Stratification variables were age, sex and city/county of residence. Baseline examinations were conducted between 2008 and 2012. Out of all invitations 4420 choose to participate (50.1% response). All participants gave written informed consent before taking part in the study. The study was approved by the local ethics committee and conformed to the principles of the declaration of Helsinki.

The Cooperative Health Research in the Region of Augsburg (KORA) study: KORA (Cooperative Health Research in the Region of Augsburg) is a research platform of independent population-based health surveys and subsequent follow-up examinations of individuals of German nationality resident in the region of Augsburg in Southern Germany. Study design, sampling method and data collection have been described in detail elsewhere[3]. The survey S4 was conducted in 1999-2001, and comprised of 4261 subjects aged 25 to 74 years. The follow-up examination F4, which was used for this study, was carried out in 2006-2008 and included 3080 participants. Participants completed a lifestyle questionnaire, including details on depression, and underwent standardized examinations with blood samples taken, as described elsewhere[3,4]. The KORA cohort ethical approval was granted by the ethics committee of the Bavarian Medical Association (REC reference numbers: #06068) and all were carried out in accordance with the principles of the Declaration of Helsinki. This covers consent for the use of biological material, including genetics. All research participants have signed informed consent prior to taking part in any research activities. The KORA data protection procedures were approved by the responsible data protection officer of the Helmholtz Zentrum München.

The European Prospective Investigation of Cancer, Norfolk (EPIC-Norfolk) study: The European Prospective Investigation into Cancer (EPIC)-Norfolk Study is a prospective population based cohort study and one of the study sites within the wider EPIC programme [5]. 30,445 men or women between 40 and 79 years of age at baseline were recruited from NHS GP practices in Norfolk, UK, between 1993 and 1997. At baseline, participants completed a health and lifestyle questionnaire which asked about medical history, dietary habits, lifestyle and other known risk factors, and 25,639 participants attended a clinic examination at which blood samples and anthropometric measures were taken. Participants were not requested to fast prior to blood sampling and were largely unfasted. All participants gave written informed consent and the study was approved by the Norwich Local Ethics Committee (REC Ref. 98CN01).

The Netherlands Epidemiology of Obesity (NEO) study: The NEO study is a population-based, cross sectional study designed to investigate pathways that lead to obesity-related diseases. The NEO study started in 2008 and includes 6,671 individuals (1997 participants with current depressive mood) aged 45–65 years, with an oversampling of individuals with overweight or obesity. The study design and population is described in detail elsewhere [6]. Men and women living in the greater area of Leiden (in the West of the Netherlands) were invited by letters sent by GPs and municipalities and by local advertisements. They were invited to respond if they were aged between 45 and 65 years and had a self-reported BMI of 27 kg/m^2 or higher. In addition, all inhabitants aged between 45 and 65 years from one municipality (Leiderdorp) were invited to participate, irrespective of their BMI. In total, 6,671 participants agreed to join the study. The Medical Ethical Committee of the Leiden University Medical Center (LUMC) approved the design of the study. All participants gave their written informed consent [6].

PReDICT study: The design of the Emory Predictors of Remission in Depression to Individual and Combined Treatments (PReDICT) study has been published previously [7], and the clinical results of the trial are presented elsewhere [8]. The overarching goal of PReDICT was to identify clinical and biological moderators of outcomes to CBT and antidepressant medication. The study was conducted through the Mood and Anxiety Disorders Program at Emory University, including a purely Spanish-language location at Grady Hospital. The Emory Institutional Review Board and the Grady Hospital Research Oversight Committee approved the study. All patients provided written, informed consent prior to beginning study procedures.

Adults aged 18–65 years were eligible to participate if they met DSM-IV criteria for a primary current diagnosis of nonpsychotic major depression as assessed by the Structured Clinical Interview for DSM-IV (40) and a psychiatrist's evaluation, and if they scored $18 on the 17-item Hamilton Depression Rating Scale (HAM-D). Additionally, patients were required to be treatment naive, defined as having

never previously received a minimally adequate course of treatment with an antidepressant medication or evidence-based psychotherapy for a mood disorder. Exclusion criteria included a lifetime history of bipolar disorder, primary psychotic disorder, or dementia, or meeting DSM-IV criteria for any of the following in the past 12 months: obsessive compulsive disorder, eating disorder, substance dependence (except for nicotine and caffeine), or dissociative disorder. Meeting DSM-IV criteria for substance abuse within the past 3months or a positive urine test for drugs of abuse at the screening visit were also exclusionary. Pregnant or breast-feeding women and patients with a medical condition that could interfere with the study or the interpretation of the study results were excluded. Patients scoring >=15 on the HAM-D at the baseline visit were randomly assigned 1:1:1 to 12 weeks of treatment with one of three treatments: 1) a selective serotonin reuptake inhibitor (SSRI), escitalopram, 10–20 mg/day; 2) a serotonin norepinephrine reuptake inhibitor (SNRI), duloxetine, 30–60 mg/ day; or 3) CBT, 16 individual 50-minute sessions. The medications were dispensed in a double-blind manner in compounded purple capsules and were dosed flexibly based on patient tolerability and response. CBT was delivered in accordance with Beck and colleagues' manual. Symptom severity using the HAM-D, Hamilton Anxiety Rating Scale, and Beck Depression Inventory was assessed weekly by blinded raters for the first 6 weeks after randomization and then every other week until week 12. In addition, the Childhood Trauma Questionnaire was completed prior to randomization. Patients were not permitted to use benzodiazepines, antipsychotics, anxiolytics, chronic opiates, or any other psychoactive medication, with the exception of hypnotics up to three times per week, though not on the night before MRI scans or ratings assessments. The PReDICT study also included a second treatment phase for nonremitters.

Remission was defined as a $HRSD_{17}$ score <=7 at both weeks 10 and 12. Treatment failure was defined as <30% reduction from the baseline $HRSD_{17}$ score at week 12. Response without remission was defined as nonremitters with a week- 12 $HRSD_{17}$ score <50% reduction from baseline, and partial response was defined as a week-12 $HRSD_{17}$ score with 30%-49% reduction from baseline.

UK Biobank: The UK Biobank cohort is a population-based cohort consisting of 501,726 individuals recruited at 23 centres across the United Kingdom (https://www.ukbiobank.ac.uk). Since 2006 UK Biobank has collected biological, medical and lifestyle data on these half million participants aged between 40-69 years. Participants include individuals mainly of European origin, however, also include other ethnic groups including those of African, Asian and South-Asian ancestry. We used the derived lifetime probable major depressive disorder (field id: 20126) measure as described in Smith et al. 2013.[9] We further defined current depressive symptoms by summing the responses to four questions related to mood in the past two weeks. These include, (1) Over the past two weeks, how often have you felt down, depressed or hopeless? (field ID: 2050) (2) Over the past two weeks, how often have you had little interest or pleasure in doing things? (field ID: 2060), (3) Over the past two weeks, how often have you felt tense, fidgety or restless? (field ID: 2070) and (4) Over the past two weeks, how often have you felt tired or had little energy (field ID: 2080)? Answers could be given on a four-point scale ranging from 0-3 (0 = not at all, 1 = several days, 2 = more than half of the days and 3 = nearly every day). The total score ranged from 0-12 where higher score indicating more severe depression. Fresh fruits and raw vegetables intake was extracted from the diet – lifestyle and environment – touchscreen questionnaire. Individuals were asked “how many pieces of fresh fruit would you eat per day” (field ID: 1309) and "On average how many heaped tablespoons of SALAD or RAW vegetables would you eat per DAY?” (field ID: 1299). Vitamin supplement data were extracted from the medication – health and medical history – touchscreen questionnaire (filed ID: 6155). Data on vitamin K antagonists was extracted from the medication data (field ID: 20003). Data for white matter hyperintensities (WMH) was extracted from the first brain-imaging visit (field ID: 25781). Retinol from food intake was estimated from food using the online 24-hour recall questionnaire (field ID: 100018). Legume/pulses intake was assessed with the question "How many servings of other beans (kidney beans/chick peas/butter beans etc) or lentils did you have?" (field ID: 104010), artificial sweetener use was assessed with the question "How many teaspoons/tablets of sweetener (e.g. Canderel) did you add to your coffee or tea (per drink)?" (field IDs: 100380 & 100500), egg intake was assessed with the

question "How many whole eggs (e.g. fried, boiled, poached) did you have?" (field ID: 102940) using online 24-hour recall questionnaire. Principal components (PCs) to adjust for population stratification were extracted using the field ID: 22009. Briefly, the top 40 PCs were constructed using a set of 407,219 unrelated, high quality samples and 147,604 high quality markers pruned to minimise linkage disequilibrium (LD). Corresponding PC-loadings were then computed and projected all samples onto the PCs, thus forming a set of PC scores for all samples in the cohort[10].

Metabolomics measurements

Rotterdam Study: Blood samples were collected at the time of examination (1997-1999). To obtain serum and plasma, tubes were centrifuged according to a protocol standardising time and conditions from the drawing of blood to centrifugation. All samples including the full blood are snap-frozen at − 196 °C using liquid nitrogen and stored at − 80 °C. Plasma metabolites of 488 dementia-free participants of the RS-I-3 were measured using ultra-high-performance liquid chromatography and gas chromatography coupled with tandem mass spectrometry by Metabolon Inc. Metabolites were measured on the HD-4 Metabolon platform as described previously.[11]

SHIP-trend: Non-targeted metabolomics analysis for metabolic profiling was conducted at the Genome Analysis Center, Helmholtz Zentrum München. Two separate LC-MS/MS analytical methods mere used as previously published, i.e. in positive and in negative ionization modes, were used to detect a broad metabolite panel 1. In this study, samples were divided into two sets according to the biological matrices of the samples, i.e. plasma and urine. On the day of extraction, samples were thawed on ice. A 100µL of the sample were pipetted into a 2mL 96-well plate. In addition to study samples, a human pooled reference plasma sample (Seralab, West Sussex, United Kingdom) and another pooled reference matrix of each sample set (Seralab, West Sussex, United Kingdom) were extracted and placed in 1 and 6 wells, respectively, of the 96-well plate. These samples served as technical replicates throughout the data set to assess process variability. Beside those samples, 100µL

of water was extracted as samples and placed in 6 wells of the 96-well plate to serve as process blanks. Protein was precipitated and the metabolites were extracted with 475µL methanol, containing four recovery standards to monitor the extraction efficiency. After centrifugation, the supernatant was split into 4 aliquots of 100µL each onto two 96-well microplates. The first 2 aliquots were used for LC-MS/MS analysis in positive and negative electrospray ionization mode. Two further aliquots were kept as a reserve. The extracts were dried on a TurboVap 96 (Zymark, Sotax, Lörrach, Germany). Prior to LC-MS/MS in positive ion mode, the samples were reconstituted with 0.1% formic acid (50µl for plasma, 100µl for urine). Whereas samples analyzed in negative ion mode were reconstituted with 6.5mM ammonium bicarbonate (50µl for plasma, 100µl for urine), pH 8.0. Reconstitution solvents for both ionization modes contained internal standards that allowed monitoring of instrument performance and also served as retention reference markers. To minimize human error, liquid handling was performed on a Hamilton Microlab STAR robot (Hamilton Bonaduz AG, Bonaduz, Switzerland). LC-MS/MS analysis was performed on a linear ion trap LTQ XL mass spectrometer (Thermo Fisher Scientific GmbH, Dreieich, Germany) coupled with a Waters Acquity UPLC system (Waters GmbH, Eschborn, Germany). Two separate columns (2.1 x 100 mm Waters BEH C18, 1.7 µm particle-size) were used either for acidic (solvent A: 0.1% formic acid in water, solvent B: 0.1% formic acid in methanol) and or for basic (A: 6.5mM ammonium bicarbonate, pH 8.0, B: 6.5mM ammonium bicarbonate in 95% methanol) mobile phase conditions, optimized for positive and negative electrospray ionization, respectively. After injection of the sample extracts, the columns were developed in a gradient of 99.5% A to 98% B over an 11 min run time at 350µLl/min flow rate. The eluent flow was directly run through the ESI source of the LTQ XL mass spectrometer. The mass spectrometer analysis alternated between MS and data-dependent MS/MS scans using dynamic exclusion and the scan range was from 80-1000 m/z. Metabolites were identified by Metabolon, Inc. from the LC-MS/MS data by automated multiparametric comparison with a proprietary library, containing retention times, m/z ratios, and related adduct/ fragment spectra2.

*KORA***:** Blood sampling: Blood samples for metabolic analysis and DNA extraction from KORA were collected between 2006 and 2008 as part of the KORA F4 follow-up. To avoid variation due to circadian rhythm, blood was drawn in the morning between 08:00 and 10:30 after a period of at least 10 h overnight fasting. Material was drawn into serum gel tubes, gently inverted twice and then allowed to rest for 30 min at room temperature (18-25 °C) to obtain complete coagulation. The material was then centrifuged for 10 min (2,750g at 15 °C). Serum was divided into aliquots and kept for a maximum of 6 h at 4 °C, after which it was frozen at -80 °C until analysis.

Metabolomics measurements: Metabolic profiling was done using the analytical platform of Metabolon, a commercial supplier of metabolic analyses. It incorporates two separate ultrahigh-performance liquid chromatography/tandem mass spectrometry (UHPLC/MS/MS2) injections and one gas chromatography/mass spectrometry (GC/MS) injection per sample. The UHPLC injections were optimized for basic and acidic species. A total of 295 known metabolites, spanning several relevant classes (amino acids, acylcarnitines, sphingomyelins, glycerophospholipids, carbohydrates, vitamins, lipids, nucleotides, peptides, xenobiotics and steroids) [1], as well as 225 unknown compounds were measured. The detection of the entire panel was carried out with 24 min of instrument analysis time (two injections at 12 min each), while maintaining low median process variability (>12% across all compounds). The resulting MS/MS2 data were searched against a standard library generated by Metabolon that included retention time, molecular mass to charge ratio (m/z), preferred adducts and in-source fragments as well as their associated MS/MS spectra for all molecules in the library. [1]

EPIC-Norfolk: Plasma citrate samples taken at baseline (in 1993 – 1998) were stored in the gas phase of liquid nitrogen at -175°C for long-term storage. Metabolite measurements were made using the Metabolon DiscoveryHD4® platform (Metabolon, Inc., Durham, USA). Measurements were made for three sets of samples - a type 2 diabetes case-cohort (N=1,503) followed by two quasi-randomly selected sets of approximately 6,000 samples. The analyses contributing to this study were performed using the latter two quasi-randomly selected sets, which were shipped in October 2015 and

January/February 2017 respectively after short-term storage at -70°C prior to shipment. Metabolite measures were corrected for run-day, by setting medians to one and normalising data points.

NEO: Fasting state blood samples from a sub-population (N=599) from the NEO study were sent for untargeted metabolomics measurements at Metabolon Inc. (Durham, North Carolina, USA) using their Metabolon Discovery HD4 platform. In brief, this process involves four independent ultra-high-performance liquid chromatography mass spectrometry (UHPLC-MS/MS) platforms [12,13]. Two platforms used positive ionization reverse phase chromatography, one used negative ionization reverse phase chromatography, and one used hydrophilic interaction liquid chromatography (HILIC) negative ionization (Rhee et al., 2019). In total, 1,365 metabolites were measured which included 840 endogenous, 296 unannotated, and 229 xenobiotic metabolites.

PReDICT Study: Biospecimens of human serum samples were profiled using targeted metabolomics protocols [14] and profiling protocols [15] previously established in our lab (Wei Jia), Bile acids were quantified by ultra-performance liquid chromatography triple quadrupole mass spectrometry (UPLC-TQMS) (Waters XEVO TQ-S, Milford, USA) and other metabolites were quantified by gas chromatography time-of-flight mass spectrometry (GC-TOFMS) (Leco Corporation, St Joseph, USA).
The following preprocessing steps on metabolite profiles were performed. First, metabolites with >20% missing values were excluded. Then, metabolites were log-transformed, imputed using Gsimp method [16] and scaled to mean zero and variance 1.

Linking metabolites to human and/or gut metabolism

To assess whether the identified metabolites are products of human metabolism, gut microbial metabolism, or both, we matched the metabolites associated with depression to the namespace of the Virtual Metabolic Human (VMH) database (www.vmh.life).[17] The VMH database is a resource combining human and gut microbiome metabolism, nutrition, and disease. It enables browsing and

querying the content of genome-scale reconstructions of human and gut microbial metabolism. A genome-scale reconstruction is a metabolic network containing the reactions, metabolites, and genes present in a given organism that was manually curated based on genomic, biochemical, and physiological data. It serves as a knowledge base for the target organism and can be converted into a mathematical model that can predict biological properties.

To identify specific gut microbial taxa potentially implicated in depression, we queried a resource of 7,206 genome-scale reconstructions of human gut microbes, AGORA2.[18] AGORA2 is an expansion of the previously published resource of 818 genome-scale reconstructions, AGORA,[19] in simulations. The capacity of all 7,206 strains to consume and/or secrete 16 microbial metabolites implicated in depression (see above) was computed as follows. Each AGORA model was allowed to take up each compound it could potentially transport. The range of flux through all exchange reactions corresponding to the 16 microbial metabolites was then computed for each model with the flux variability analysis (FVA)[20] implementation in the COBRA Toolbox.[21] By convention, negative flux through the exchange reactions corresponds to the capability to consume the metabolite, while positive flux corresponds to the capability to secrete the metabolite. The uptake and secretion capabilities for each individual metabolite were consequently extracted from all 7,206 models. The simulations were performed in MATLAB (Mathworks, Inc.) version R2018b with IBM CPLEX (IBM) as the linear programming solver.

References

1 Ikram, M. A. *et al.* Objectives, design and main findings until 2020 from the Rotterdam Study. *Eur. J. Epidemiol.* **35**, 483-517, doi:10.1007/s10654-020-00640-5 (2020).

2 Volzke, H. *et al.* Cohort profile: the study of health in Pomerania. *Int. J. Epidemiol.* **40**, 294-307, doi:10.1093/ije/dyp394 (2011).

3 Holle, R., Happich, M., Lowel, H., Wichmann, H. E. & Group, M. K. S. KORA--a research platform for population based health research. *Gesundheitswesen* **67 Suppl 1**, S19-25, doi:10.1055/s-2005-858235 (2005).

4 Wichmann, H. E., Gieger, C., Illig, T. & Group, M. K. S. KORA-gen--resource for population genetics, controls and a broad spectrum of disease phenotypes. *Gesundheitswesen* **67 Suppl 1**, S26-30, doi:10.1055/s-2005-858226 (2005).

5 Day, N. *et al.* EPIC-Norfolk: study design and characteristics of the cohort. European Prospective Investigation of Cancer. *Br. J. Cancer* **80 Suppl 1**, 95-103 (1999).

6 de Mutsert, R. *et al.* The Netherlands Epidemiology of Obesity (NEO) study: study design and data collection. *Eur. J. Epidemiol.* **28**, 513-523, doi:10.1007/s10654-013-9801-3 (2013).

7 Dunlop, B. W. *et al.* Predictors of remission in depression to individual and combined treatments (PReDICT): study protocol for a randomized controlled trial. *Trials* **13**, 106, doi:10.1186/1745-6215-13-106 (2012).

8 Dunlop, B. W. *et al.* Effects of Patient Preferences on Outcomes in the Predictors of Remission in Depression to Individual and Combined Treatments (PReDICT) Study. *Am J Psychiatry* **174**, 546-556, doi:10.1176/appi.ajp.2016.16050517 (2017).

9 Smith, D. J. *et al.* Prevalence and characteristics of probable major depression and bipolar disorder within UK biobank: cross-sectional study of 172,751 participants. *PLoS One* **8**, e75362, doi:10.1371/journal.pone.0075362 (2013).

10 Clare, B. *et al.* Genome-wide genetic data on ~500,000 UK Biobank participants. *bioRxiv*, 166298, doi:10.1101/166298 (2017).

11 Shin, S. Y. *et al.* An atlas of genetic influences on human blood metabolites. *Nat Genet* **46**, 543-550, doi:10.1038/ng.2982 (2014).

12 Evans, A. M., *et al*. High Resolution Mass Spectrometry Improves Data Quantity and Quality as Compared to Unit Mass Resolution Mass Spectrometry in HighThroughput Profiling Metabolomics. *Metabolomics* **4**, 132, doi:10.4172/2153-0769.1000132 (2014).

13 Rhee, E. P. *et al.* Variability of Two Metabolomic Platforms in CKD. *Clin. J. Am. Soc. Nephrol.* **14**, 40-48, doi:10.2215/CJN.07070618 (2019).

14 Xie, G. *et al.* Profiling of serum bile acids in a healthy Chinese population using UPLC-MS/MS. *J Proteome Res* **14**, 850-859, doi:10.1021/pr500920q (2015).

15 Qiu, Y. *et al.* Serum metabolite profiling of human colorectal cancer using GC-TOFMS and UPLC-QTOFMS. *J Proteome Res* **8**, 4844-4850, doi:10.1021/pr9004162 (2009).

16 Wei, R. *et al.* GSimp: A Gibbs sampler based left-censored missing value imputation approach for metabolomics studies. *PLoS Comput Biol* **14**, e1005973, doi:10.1371/journal.pcbi.1005973 (2018).

17 Noronha, A. *et al.* The Virtual Metabolic Human database: integrating human and gut microbiome metabolism with nutrition and disease. *Nucleic Acids Res.* **47**, D614-D624, doi:10.1093/nar/gky992 (2019).

18 Heinken, A. *et al.* AGORA2: Large scale reconstruction of the microbiome highlights wide-spread drug-metabolising capacities. *bioRxiv*, 2020.2011.2009.375451, doi:10.1101/2020.11.09.375451 (2020).

19 Magnusdottir, S. *et al.* Generation of genome-scale metabolic reconstructions for 773 members of the human gut microbiota. *Nat. Biotechnol.* **35**, 81-89, doi:10.1038/nbt.3703 (2017).

20 Gudmundsson, S. & Thiele, I. Computationally efficient flux variability analysis. *BMC Bioinformatics* **11**, 489, doi:10.1186/1471-2105-11-489 (2010).

21 Heirendt, L. *et al.* Creation and analysis of biochemical constraint-based models using the COBRA Toolbox v.3.0. *Nat. Protoc.* **14**, 639-702, doi:10.1038/s41596-018-0098-2 10.1038/s41596-018-0098-2 [pii] (2019).

Supplementary Table description

Supplementary Table 1: Metabolites and depression assessment in cohort included in the metabolome-wide association analysis

Supplementary Table 2: Instruments used for major depression for Mendelian Randomization analysis. For each SNP association results with each metabolite and major depression are shown.

Supplementary Table 3: Instruments used for associated metabolites for Mendelian Randomization analysis. For each SNP association results with each metabolite and major depression are shown.

Supplementary Table 4: Results of metabolome-wide association analysis for each of the 3 models analysed.

Supplementary Table 5: Results of the sex-stratified association analysis of all metabolites with depression for model 2.

Supplementary Table 6: Results of the sensitivity analysis, excluding the cohorts which measured metabolites on older Metabolon platforms.

Supplementary Table 7: Results of the association of white matter hyperintensity volume with vitamin supplements.

Supplementary Table 8: Association of significant metabolites with C-Reactive protein (CRP)

Supplementary Table 9: Association of food sources of significant metabolites with C-Reactive protein (CRP)

Supplementary Table 10: Results of the MR analysis with major depression as exposure and metabolites as outcome.

Supplementary Table 11: Results of the MR analysis with metabolites as exposure and major depression as outcome.

Supplementary Table 12: Results of the human and gut microbiome metabolic network analysis.

Supplementary Table 13: Depression-associated metabolites included in AGORA2 that could be consumed by at least one AGORA2 strain.

Supplementary Table 14: Depression-associated metabolites included in AGORA2 that could be produced by at least one AGORA2 strain.

Supplementary Figure Legends:

Supplementary Figure 1: Change in ciculating hippurate levels over 12 weeks of treatment

Supplementary Figure 2: Gut microbial genera involved in the metabolism of depression-associated metabolites

Supplementary Table 1: Metabolites and depression assessment in cohort included in the metabolome-wide association analysis

Cohort	Metabolon Platform version (N metabolites)	Total sample (N)	Depression assessment
Rotterdam Study	HD4 (850)	484	CESD-D (quantitative)
KORA	HD2 (276)	1688	PHQ-9 (quantitative)
SHIP-trend	HD3 (264)	978	PHQ-9 (quantitative)
EPIC-Norfolk (batch 2)	HD4 (666)	4639	Depression diagnosis Yes/No
EPIC-Norfolk (batch 3)	HD4 (734)	5163	Depression diagnosis Yes/No
NEO	HD4 (1069)	599	IDS-SR30 (quantitative)
Total		13551	

Supplementary Table 2: List of SNPs used as instrumental variables for MDD in the MR analysis.

Metabolite	Rs.ID	ID	REF	ALT	OBS_CT	BETA	beta_flip	SE	Beta_MDD	SE_MDD	AF	A1_mdd
1-palmitoyl-2-palmitoleoyl-GPC (16:0/16:1)	rs301799	1:8489302	C	T	8809	-0.016679	-0.016679	0.0148724	-0.0254	0.0022	0.5694	T
1-palmitoyl-2-palmitoleoyl-GPC (16:0/16:1)	rs1002656	1:37192741	C	T	8809	0.0309417	0.0309417	0.0165583	-0.021	0.0024	0.7033	T
1-palmitoyl-2-palmitoleoyl-GPC (16:0/16:1)	rs11579246	1:50559162	A	G	8809	-0.0099682	0.00996824	0.0282309	0.032	0.0037	0.9067	A
1-palmitoyl-2-palmitoleoyl-GPC (16:0/16:1)	rs1890946	1:52342427	T	C	8809	0.00816282	-0.0081628	0.0148638	-0.0194	0.0022	0.4671	T
1-palmitoyl-2-palmitoleoyl-GPC (16:0/16:1)	rs10789214	1:67146817	C	T	8809	-0.0103862	-0.0103862	0.0150075	0.0137	0.0022	0.5661	T
1-palmitoyl-2-palmitoleoyl-GPC (16:0/16:1)	rs2568958	1:72765116	G	A	8809	-0.022035	-0.022035	0.01517	0.0336	0.0022	0.6156	A
1-palmitoyl-2-palmitoleoyl-GPC (16:0/16:1)	rs10890020	1:73668836	A	G	8809	0.0171533	-0.0171533	0.0147873	-0.0267	0.0022	0.5156	A
1-palmitoyl-2-palmitoleoyl-GPC (16:0/16:1)	rs113188507	1:80809636	G	A	8809	0.0303049	0.0303049	0.01648	0.0234	0.0025	0.2838	A
1-palmitoyl-2-palmitoleoyl-GPC (16:0/16:1)	rs10913112	1:175913828	C	T	8809	0.00689667	0.00689667	0.0152653	-0.0214	0.0023	0.3767	T
1-palmitoyl-2-palmitoleoyl-GPC (16:0/16:1)	rs169235	1:181740924	A	G	8809	0.00287852	-0.0028785	0.0172281	-0.017	0.0025	0.753	A
1-palmitoyl-2-palmitoleoyl-GPC (16:0/16:1)	rs17641524	1:197704717	C	T	8809	-0.0064299	-0.0064299	0.0183088	-0.0243	0.0027	0.2091	T
1-palmitoyl-2-palmitoleoyl-GPC (16:0/16:1)	rs12052908	2:22503044	A	T	8809	0.00563725	-0.0056373	0.0149254	-0.0211	0.0022	0.5325	A
1-palmitoyl-2-palmitoleoyl-GPC (16:0/16:1)	rs1568452	2:58012833	C	T	8809	-0.0095178	-0.0095178	0.0151309	0.0293	0.0022	0.3851	T
1-palmitoyl-2-palmitoleoyl-GPC (16:0/16:1)	rs7585722	2:86819128	T	C	8809	-0.0015156	0.00151559	0.021659	-0.0215	0.003	0.8458	T
1-palmitoyl-2-palmitoleoyl-GPC (16:0/16:1)	rs1226412	2:157111313	C	T	8809	0.0297457	0.0297457	0.0180967	0.0232	0.0027	0.7917	T
1-palmitoyl-2-palmitoleoyl-GPC (16:0/16:1)	rs62188629	2:208044470	G	A	8809	-0.0082045	-0.0082045	0.0159577	0.017	0.0024	0.3136	A
1-palmitoyl-2-palmitoleoyl-GPC (16:0/16:1)	rs13084037	3:49214066	G	A	8809	-0.0148961	-0.0148961	0.0175923	-0.0217	0.0025	0.774	A
1-palmitoyl-2-palmitoleoyl-GPC (16:0/16:1)	rs6783233	3:117509984	C	T	8809	0.0157931	0.0157931	0.0164392	0.0177	0.0024	0.2833	T
1-palmitoyl-2-palmitoleoyl-GPC (16:0/16:1)	rs1095626	3:157977962	T	C	8809	-0.0084741	0.00847412	0.0150818	-0.0285	0.0022	0.5799	T
1-palmitoyl-2-palmitoleoyl-GPC (16:0/16:1)	rs7685686	4:3207142	A	G	8809	-0.0212279	0.0212279	0.0150686	0.0173	0.0022	0.5753	A
1-palmitoyl-2-palmitoleoyl-GPC (16:0/16:1)	rs34937911	4:42110353	T	C	8809	-0.0246218	0.0246218	0.0231734	0.0244	0.0034	0.8838	T
1-palmitoyl-2-palmitoleoyl-GPC (16:0/16:1)	rs45510091	4:123186393	A	G	8809	-0.0093231	0.00932309	0.0321424	0.0463	0.0049	0.9472	A
1-palmitoyl-2-palmitoleoyl-GPC (16:0/16:1)	rs60157091	5:61509655	C	T	8809	-0.0029079	-0.0029079	0.0147478	0.0205	0.0022	0.515	T
1-palmitoyl-2-palmitoleoyl-GPC (16:0/16:1)	rs3099439	5:87545318	C	T	8809	-0.005723	-0.005723	0.0147631	-0.0208	0.0022	0.5288	T
1-palmitoyl-2-palmitoleoyl-GPC (16:0/16:1)	rs10061069	5:93071630	G	C	8809	-0.021844	-0.021844	0.0179958	-0.025	0.0026	0.2212	C
1-palmitoyl-2-palmitoleoyl-GPC (16:0/16:1)	rs30266	5:103972357	G	A	8809	0.0539079	0.0539079	0.0158119	0.0323	0.0023	0.3296	A
1-palmitoyl-2-palmitoleoyl-GPC (16:0/16:1)	rs11135349	5:164523472	A	C	8809	0.0158113	-0.0158113	0.0148737	-0.025	0.0022	0.4713	A
1-palmitoyl-2-palmitoleoyl-GPC (16:0/16:1)	rs200949	6:27835435	A	G	8809	0.0358659	-0.0358659	0.0209049	0.0433	0.0034	0.8744	A
1-palmitoyl-2-palmitoleoyl-GPC (16:0/16:1)	rs7758630	6:101387304	T	A	8809	0.0193492	0.0193492	0.0152012	-0.0165	0.0022	0.4051	A
1-palmitoyl-2-palmitoleoyl-GPC (16:0/16:1)	rs1933802	6:105365891	C	G	8809	-0.0297858	0.0297858	0.0147856	-0.0198	0.0022	0.4536	C
1-palmitoyl-2-palmitoleoyl-GPC (16:0/16:1)	rs2876520	6:142996618	C	G	8809	-0.0067687	0.00676869	0.0148878	-0.0187	0.0022	0.5271	C
1-palmitoyl-2-palmitoleoyl-GPC (16:0/16:1)	rs2029865	6:165121844	T	A	8809	-0.0155879	-0.0155879	0.0147123	-0.0184	0.0022	0.4534	A
1-palmitoyl-2-palmitoleoyl-GPC (16:0/16:1)	rs3823624	7:2110346	T	C	8809	-0.0388627	0.0388627	0.0189466	0.0294	0.0028	0.8067	T
1-palmitoyl-2-palmitoleoyl-GPC (16:0/16:1)	rs2043539	7:12253880	G	A	8809	0.0103317	0.0103317	0.0149918	0.022	0.0022	0.4177	A
1-palmitoyl-2-palmitoleoyl-GPC (16:0/16:1)	rs2247523	7:82454404	C	G	8809	-0.0229446	0.0229446	0.0150366	-0.0159	0.0021	0.5319	C
1-palmitoyl-2-palmitoleoyl-GPC (16:0/16:1)	rs16887442	7:82936909	T	C	8809	-0.0179589	0.0179589	0.0148975	0.0148	0.0022	0.4347	T
1-palmitoyl-2-palmitoleoyl-GPC (16:0/16:1)	rs58104186	7:109099919	G	A	8809	0.00251463	0.00251463	0.014788	0.019	0.0022	0.4689	A
1-palmitoyl-2-palmitoleoyl-GPC (16:0/16:1)	rs7807677	7:117502574	C	T	8809	0.0093877	0.0093877	0.0148374	0.0211	0.0022	0.5505	T
1-palmitoyl-2-palmitoleoyl-GPC (16:0/16:1)	rs7837935	8:65562019	T	G	8809	0.00889921	-0.0088992	0.0207913	-0.0226	0.0031	0.1522	T
1-palmitoyl-2-palmitoleoyl-GPC (16:0/16:1)	rs1354115	9:2983774	C	A	8809	-0.0181909	-0.0181909	0.0153349	0.0186	0.0022	0.6243	A
1-palmitoyl-2-palmitoleoyl-GPC (16:0/16:1)	rs263645	9:17016503	A	T	8809	0.0046549	-0.0046549	0.0148716	0.0176	0.0022	0.5438	A
1-palmitoyl-2-palmitoleoyl-GPC (16:0/16:1)	rs3793577	9:23737627	A	G	8809	0.00448335	-0.0044834	0.0148122	-0.0211	0.0022	0.4665	A
1-palmitoyl-2-palmitoleoyl-GPC (16:0/16:1)	rs34653192	9:31124452	G	C	8809	0.00549135	0.00549135	0.0159897	-0.0196	0.0024	0.3196	C
1-palmitoyl-2-palmitoleoyl-GPC (16:0/16:1)	rs7030813	9:36999369	C	T	8809	-0.0093423	-0.0093423	0.0154398	0.0251	0.0022	0.3736	T
1-palmitoyl-2-palmitoleoyl-GPC (16:0/16:1)	rs10817969	9:119731045	T	G	8809	0.00591582	-0.0059158	0.016671	0.0169	0.0024	0.7173	T
1-palmitoyl-2-palmitoleoyl-GPC (16:0/16:1)	rs913930	9:120484009	G	A	8809	0.00111163	0.00111163	0.0155459	-0.0219	0.0023	0.6433	A
1-palmitoyl-2-palmitoleoyl-GPC (16:0/16:1)	rs2670139	9:126634255	T	C	8809	-0.0089434	0.00894335	0.0174718	-0.0178	0.0025	0.7609	T
1-palmitoyl-2-palmitoleoyl-GPC (16:0/16:1)	rs997934	10:1795194	T	C	8809	-0.002811	0.002811	0.0153977	0.0163	0.0023	0.3795	T
1-palmitoyl-2-palmitoleoyl-GPC (16:0/16:1)	rs1021363	10:106610839	A	G	8809	0.00791531	-0.0079153	0.0157253	0.0226	0.0023	0.3547	A
1-palmitoyl-2-palmitoleoyl-GPC (16:0/16:1)	rs1448938	11:30892824	A	G	8809	0.0162699	-0.0162699	0.0150113	0.0172	0.0022	0.4171	A
1-palmitoyl-2-palmitoleoyl-GPC (16:0/16:1)	rs2509805	11:57650796	T	C	8809	0.0213869	-0.0213869	0.0158826	0.0191	0.0024	0.3209	T
1-palmitoyl-2-palmitoleoyl-GPC (16:0/16:1)	rs198457	11:61471678	C	T	8809	-0.0427193	-0.0427193	0.019066	-0.0213	0.0028	0.1925	T
1-palmitoyl-2-palmitoleoyl-GPC (16:0/16:1)	rs58621819	11:65314830	A	T	8809	0.00112493	-0.0011249	0.0182925	-0.0169	0.0027	0.7903	A
1-palmitoyl-2-palmitoleoyl-GPC (16:0/16:1)	rs7117514	11:70544937	A	G	8809	-0.0127702	0.0127702	0.014716	-0.0166	0.0022	0.5417	A
1-palmitoyl-2-palmitoleoyl-GPC (16:0/16:1)	rs7932640	11:88744425	T	C	8809	0.0207069	-0.0207069	0.0149869	0.0227	0.0022	0.4417	T
1-palmitoyl-2-palmitoleoyl-GPC (16:0/16:1)	rs61902811	11:113370758	G	A	8809	-0.0078997	-0.0078997	0.0154003	-0.0289	0.0022	0.3682	A
1-palmitoyl-2-palmitoleoyl-GPC (16:0/16:1)	rs78337797	12:23987925	T	G	8809	0.0114514	-0.0114514	0.022551	0.0229	0.0034	0.8781	T
1-palmitoyl-2-palmitoleoyl-GPC (16:0/16:1)	rs56314503	12:84465022	T	G	8809	-0.0048668	0.00486676	0.0177706	-0.0203	0.0025	0.7487	T
1-palmitoyl-2-palmitoleoyl-GPC (16:0/16:1)	rs3213572	12:121205078	G	A	8809	-0.0005075	-0.0005075	0.0148221	0.0196	0.0021	0.4745	A
1-palmitoyl-2-palmitoleoyl-GPC (16:0/16:1)	rs1343605	13:53647048	A	C	8809	0.00822311	-0.0082231	0.0151004	0.0231	0.0022	0.384	A
1-palmitoyl-2-palmitoleoyl-GPC (16:0/16:1)	rs9592461	13:66941792	A	G	8809	-0.0386442	0.0386442	0.014834	0.0243	0.0022	0.4874	A
1-palmitoyl-2-palmitoleoyl-GPC (16:0/16:1)	rs4772087	13:99115041	C	T	8809	0.0413298	0.0413298	0.0153868	0.0209	0.0023	0.3732	T
1-palmitoyl-2-palmitoleoyl-GPC (16:0/16:1)	rs61990288	14:42074726	A	G	8809	-0.0097015	0.00970149	0.0147996	-0.0249	0.0021	0.5083	A
1-palmitoyl-2-palmitoleoyl-GPC (16:0/16:1)	rs1956373	14:60141822	T	G	8809	-0.0195814	0.0195814	0.0169602	-0.0169	0.0025	0.7436	T
1-palmitoyl-2-palmitoleoyl-GPC (16:0/16:1)	rs1152578	14:64697037	C	T	8809	-0.0137058	-0.0137058	0.0149939	-0.0152	0.0022	0.4357	T
1-palmitoyl-2-palmitoleoyl-GPC (16:0/16:1)	rs1045430	14:75130235	G	T	8809	-0.0361594	-0.0361594	0.0147927	-0.0226	0.0022	0.4792	T
1-palmitoyl-2-palmitoleoyl-GPC (16:0/16:1)	rs10149470	14:104017953	G	A	8809	0.00726832	0.00726832	0.0148075	-0.0206	0.0021	0.4869	A
1-palmitoyl-2-palmitoleoyl-GPC (16:0/16:1)	rs8037355	15:37643831	C	T	8809	0.0103171	0.0103171	0.0149098	-0.0192	0.0022	0.5556	T
1-palmitoyl-2-palmitoleoyl-GPC (16:0/16:1)	rs34488670	15:47684936	T	C	8809	-0.0227264	0.0227264	0.0186341	-0.0193	0.0027	0.7887	T
1-palmitoyl-2-palmitoleoyl-GPC (16:0/16:1)	rs7193263	16:6315880	G	A	8809	0.00541914	0.00541914	0.0156772	-0.0206	0.0023	0.6679	A
1-palmitoyl-2-palmitoleoyl-GPC (16:0/16:1)	rs7198928	16:7666402	T	C	8809	0.011052	-0.011052	0.0153513	0.0219	0.0022	0.6159	T
1-palmitoyl-2-palmitoleoyl-GPC (16:0/16:1)	rs7200826	16:13066833	C	T	8809	0.0172078	0.0172078	0.0168803	0.027	0.0025	0.2551	T
1-palmitoyl-2-palmitoleoyl-GPC (16:0/16:1)	rs56887639	16:13755530	A	G	8809	0.00448274	-0.0044827	0.0168533	-0.0171	0.0024	0.7264	A
1-palmitoyl-2-palmitoleoyl-GPC (16:0/16:1)	rs12923444	16:21639710	A	C	8809	-0.0055456	0.00554561	0.0152856	-0.0237	0.0023	0.5625	A
1-palmitoyl-2-palmitoleoyl-GPC (16:0/16:1)	rs75581564	17:27363750	G	A	8809	0.0573637	0.0573637	0.0229071	0.0248	0.0034	0.1165	A
1-palmitoyl-2-palmitoleoyl-GPC (16:0/16:1)	rs12967855	18:35138245	A	G	8809	0.0031316	-0.0031316	0.015869	0.0207	0.0023	0.3295	A
1-palmitoyl-2-palmitoleoyl-GPC (16:0/16:1)	rs7227069	18:50731802	G	A	8809	0.0101077	0.0101077	0.014985	0.0242	0.0022	0.4326	A
1-palmitoyl-2-palmitoleoyl-GPC (16:0/16:1)	rs62091461	18:52488672	C	T	8809	0.0214435	0.0214435	0.0177353	-0.0199	0.0026	0.2274	T
1-palmitoyl-2-palmitoleoyl-GPC (16:0/16:1)	rs12967143	18:53099012	G	C	8809	0.0209805	0.0209805	0.0159405	-0.0256	0.0024	0.6984	C
1-palmitoyl-2-palmitoleoyl-GPC (16:0/16:1)	rs7241572	18:77580712	G	A	8809	0.0348433	0.0348433	0.0184047	0.02	0.0027	0.201	A
1-palmitoyl-2-palmitoleoyl-GPC (16:0/16:1)	rs12624433	20:44680853	G	A	8809	-0.0312402	-0.0312402	0.0168969	0.0191	0.0025	0.2584	A
1-palmitoyl-2-palmitoleoyl-GPC (16:0/16:1)	rs5995992	22:41487218	T	C	8809	-0.0039792	0.00397922	0.0161838	-0.0301	0.0024	0.7155	T
2-aminooctanoate	rs301799	1:8489302	C	T	8368	-0.00365574	-0.00365574	0.0152564	-0.0254	0.0022	0.5694	T
2-aminooctanoate	rs1002656	1:37192741	C	T	8368	0.0137975	0.0137975	0.0170164	-0.021	0.0024	0.7033	T
2-aminooctanoate	rs11579246	1:50559162	A	G	8368	-0.0174144	0.0174144	0.0290004	0.032	0.0037	0.9067	A
2-aminooctanoate	rs1890946	1:52342427	T	C	8368	-0.00230939	0.00230939	0.0152327	-0.0194	0.0022	0.4671	T

2-aminooctanoate	rs10789214	1:67146817	C	T	8368	-0.0136698	-0.0136698	0.0153959	0.0137	0.0022	0.5661	T
2-aminooctanoate	rs2568958	1:72765116	G	A	8368	0.033273	0.033273	0.0155542	0.0336	0.0022	0.6156	A
2-aminooctanoate	rs10890020	1:73668836	A	G	8368	-0.0130445	0.0130445	0.0151886	-0.0267	0.0022	0.5156	A
2-aminooctanoate	rs113188507	1:80809636	G	A	8368	-0.0031652	-0.0031652	0.0168986	0.0234	0.0025	0.2838	A
2-aminooctanoate	rs10913112	1:175913828	C	T	8368	-0.00283098	-0.00283098	0.0157057	-0.0214	0.0023	0.3767	T
2-aminooctanoate	rs169235	1:181740924	A	G	8368	0.00836582	-0.00836582	0.0176679	-0.017	0.0025	0.753	A
2-aminooctanoate	rs17641524	1:197704717	C	T	8368	0.00274731	0.00274731	0.018848	-0.0243	0.0027	0.2091	T
2-aminooctanoate	rs12052908	2:22503044	A	T	8368	0.0181002	-0.0181002	0.0153385	-0.0211	0.0022	0.5325	A
2-aminooctanoate	rs1568452	2:58012833	C	T	8368	0.0103659	0.0103659	0.0155446	0.0293	0.0022	0.3851	T
2-aminooctanoate	rs7585722	2:86819128	T	C	8368	-0.0328779	0.0328779	0.0222408	-0.0215	0.003	0.8458	T
2-aminooctanoate	rs1226412	2:157111313	C	T	8368	-0.00405949	-0.00405949	0.0186313	0.0232	0.0027	0.7917	T
2-aminooctanoate	rs62188629	2:208044470	G	A	8368	-0.00458658	-0.00458658	0.0163868	0.017	0.0024	0.3136	A
2-aminooctanoate	rs13084037	3:49214066	G	A	8368	0.015954	0.015954	0.0181034	-0.0217	0.0025	0.774	A
2-aminooctanoate	rs6783233	3:117509984	C	T	8368	0.0117456	0.0117456	0.0169286	0.0177	0.0024	0.2833	T
2-aminooctanoate	rs1095626	3:157977962	T	C	8368	0.00915149	-0.00915149	0.015502	-0.0285	0.0022	0.5799	T
2-aminooctanoate	rs7685686	4:3207142	A	G	8368	0.0184181	-0.0184181	0.0154796	0.0173	0.0022	0.5753	A
2-aminooctanoate	rs34937911	4:42110353	T	C	8368	-0.00529753	0.00529753	0.0237166	0.0244	0.0034	0.8838	T
2-aminooctanoate	rs45510091	4:123186393	A	G	8368	-0.0872008	0.0872008	0.0329486	0.0463	0.0049	0.9472	A
2-aminooctanoate	rs60157091	5:61509655	C	T	8368	-0.0110903	-0.0110903	0.0151498	0.0205	0.0022	0.515	T
2-aminooctanoate	rs3099439	5:87545318	C	T	8368	-0.000176724	-0.000176724	0.0151671	-0.0208	0.0022	0.5288	T
2-aminooctanoate	rs10061069	5:93071630	G	C	8368	0.0106413	0.0106413	0.0184126	-0.025	0.0026	0.2212	C
2-aminooctanoate	rs30266	5:103972357	G	A	8368	-0.00294046	-0.00294046	0.0162763	0.0323	0.0023	0.3296	A
2-aminooctanoate	rs11135349	5:164523472	A	C	8368	0.00100699	-0.00100699	0.0152594	-0.025	0.0022	0.4713	A
2-aminooctanoate	rs200949	6:27835435	A	G	8368	-0.0319067	0.0319067	0.0214291	0.0433	0.0034	0.8744	A
2-aminooctanoate	rs7758630	6:101387304	T	A	8368	-0.0149174	-0.0149174	0.0156348	-0.0165	0.0022	0.4051	A
2-aminooctanoate	rs1933802	6:105365891	C	G	8368	0.000857848	-0.000857848	0.0151835	-0.0198	0.0022	0.4536	C
2-aminooctanoate	rs2876520	6:142996618	C	G	8368	-0.00229018	0.00229018	0.0152999	-0.0187	0.0022	0.5271	C
2-aminooctanoate	rs2029865	6:165121844	T	A	8368	0.00173148	0.00173148	0.0150948	-0.0184	0.0022	0.4534	A
2-aminooctanoate	rs3823624	7:2110346	T	C	8368	0.00976366	-0.00976366	0.0195025	0.0294	0.0028	0.8067	T
2-aminooctanoate	rs2043539	7:12253880	G	A	8368	0.00322329	0.00322329	0.0153834	0.022	0.0022	0.4177	A
2-aminooctanoate	rs2247523	7:82454404	C	G	8368	0.0174629	-0.0174629	0.0154526	-0.0159	0.0021	0.5319	C
2-aminooctanoate	rs16887442	7:82936909	T	C	8368	0.0245764	-0.0245764	0.0153102	0.0148	0.0022	0.4347	T
2-aminooctanoate	rs58104186	7:109099919	G	A	8368	-0.00412312	-0.00412312	0.0151791	0.019	0.0022	0.4689	A
2-aminooctanoate	rs7807677	7:117502574	C	T	8368	-4.40E-05	-0.000044	0.0152341	0.0211	0.0022	0.5505	T
2-aminooctanoate	rs7837935	8:65562019	T	G	8368	0.0208991	-0.0208991	0.0213311	-0.0226	0.0031	0.1522	T
2-aminooctanoate	rs1354115	9:2983774	C	A	8368	0.0272541	0.0272541	0.0157571	0.0186	0.0022	0.6243	A
2-aminooctanoate	rs263645	9:17016503	A	T	8368	0.00359421	-0.00359421	0.0153035	0.0176	0.0022	0.5438	A
2-aminooctanoate	rs3793577	9:23737627	A	G	8368	0.0177669	-0.0177669	0.0151842	-0.0211	0.0022	0.4665	A
2-aminooctanoate	rs34653192	9:31124452	G	C	8368	0.0158734	0.0158734	0.0163916	-0.0196	0.0024	0.3196	C
2-aminooctanoate	rs7030813	9:36999369	C	T	8368	0.0236272	0.0236272	0.0158733	0.0251	0.0022	0.3736	T
2-aminooctanoate	rs10817969	9:119731045	T	G	8368	-0.0227633	0.0227633	0.0171227	0.0169	0.0024	0.7173	T
2-aminooctanoate	rs913930	9:120484009	G	A	8368	0.00993232	0.00993232	0.0159341	-0.0219	0.0023	0.6433	A
2-aminooctanoate	rs2670139	9:126634255	T	C	8368	0.0241322	-0.0241322	0.0178825	-0.0178	0.0025	0.7609	T
2-aminooctanoate	rs997934	10:1795194	T	C	8368	0.00511084	-0.00511084	0.0158085	0.0163	0.0023	0.3795	T
2-aminooctanoate	rs1021363	10:106610839	A	G	8368	0.0215179	-0.0215179	0.0161744	0.0226	0.0023	0.3547	A
2-aminooctanoate	rs1448938	11:30892824	A	G	8368	0.00965653	-0.00965653	0.0154093	0.0172	0.0022	0.4171	A
2-aminooctanoate	rs2509805	11:57650796	T	C	8368	0.00306595	-0.00306595	0.0163368	0.0191	0.0024	0.3209	T
2-aminooctanoate	rs198457	11:61471678	C	T	8368	0.0217612	0.0217612	0.0195855	-0.0213	0.0028	0.1925	T
2-aminooctanoate	rs58621819	11:65314830	A	T	8368	-0.0194025	0.0194025	0.0187445	-0.0169	0.0027	0.7903	A
2-aminooctanoate	rs7117514	11:70544937	A	G	8368	0.000545165	-0.000545165	0.015133	-0.0166	0.0022	0.5417	A
2-aminooctanoate	rs7932640	11:88744425	T	C	8368	-0.00615549	0.00615549	0.0153593	0.0227	0.0022	0.4417	T
2-aminooctanoate	rs61902811	11:113370758	G	A	8368	0.0110178	0.0110178	0.0158214	-0.0289	0.0022	0.3682	A
2-aminooctanoate	rs78337797	12:23987925	T	G	8368	0.00725981	-0.00725981	0.0232106	0.0229	0.0034	0.8781	T
2-aminooctanoate	rs56314503	12:84465022	T	G	8368	-0.0123929	0.0123929	0.018272	-0.0203	0.0025	0.7487	T
2-aminooctanoate	rs3213572	12:121205078	G	A	8368	0.00868272	0.00868272	0.0152043	0.0196	0.0021	0.4745	A
2-aminooctanoate	rs1343605	13:53647048	A	C	8368	-0.010995	0.010995	0.0155188	0.0231	0.0022	0.384	A
2-aminooctanoate	rs9592461	13:66941792	A	G	8368	0.0115912	-0.0115912	0.0152105	0.0243	0.0022	0.4874	A
2-aminooctanoate	rs4772087	13:99115041	C	T	8368	0.00240737	0.00240737	0.015836	0.0209	0.0023	0.3732	T
2-aminooctanoate	rs61990288	14:42074726	A	G	8368	0.00933691	-0.00933691	0.0152206	-0.0249	0.0021	0.5083	A
2-aminooctanoate	rs1956373	14:60141822	T	G	8368	-0.00834803	0.00834803	0.0173763	-0.0169	0.0025	0.7436	T
2-aminooctanoate	rs1152578	14:64697037	C	T	8368	0.00528097	0.00528097	0.015374	-0.0152	0.0022	0.4357	T
2-aminooctanoate	rs1045430	14:75130235	G	T	8368	0.00524004	0.00524004	0.0152034	-0.0226	0.0022	0.4792	T
2-aminooctanoate	rs10149470	14:104017953	G	A	8368	0.00816805	0.00816805	0.0152141	-0.0206	0.0021	0.4869	A
2-aminooctanoate	rs8037355	15:37643831	C	T	8368	0.00483722	0.00483722	0.0152932	-0.0192	0.0022	0.5556	T
2-aminooctanoate	rs34488670	15:47684936	T	C	8368	-0.00407093	0.00407093	0.0191417	-0.0193	0.0027	0.7887	T
2-aminooctanoate	rs7193263	16:6315880	G	A	8368	-0.0186022	-0.0186022	0.0160684	-0.0206	0.0023	0.6679	A
2-aminooctanoate	rs7198928	16:7666402	T	C	8368	-0.029451	0.029451	0.0157511	0.0219	0.0022	0.6159	T
2-aminooctanoate	rs7200826	16:13066833	C	T	8368	-0.00598558	-0.00598558	0.0173638	0.027	0.0025	0.2551	T
2-aminooctanoate	rs56887639	16:13755530	A	G	8368	-0.00136895	0.00136895	0.0173104	-0.0171	0.0024	0.7264	A
2-aminooctanoate	rs12923444	16:21639710	A	C	8368	-0.00501555	0.00501555	0.0156926	-0.0237	0.0023	0.5625	A
2-aminooctanoate	rs75581564	17:27363750	G	A	8368	-0.0426777	-0.0426777	0.023447	0.0248	0.0034	0.1165	A
2-aminooctanoate	rs12967855	18:35138245	A	G	8368	0.0124571	-0.0124571	0.0163138	0.0207	0.0023	0.3295	A
2-aminooctanoate	rs7227069	18:50731802	G	A	8368	-0.016036	-0.016036	0.015405	0.0242	0.0022	0.4326	A
2-aminooctanoate	rs62091461	18:52488672	C	T	8368	-0.0200604	-0.0200604	0.0182228	-0.0199	0.0026	0.2274	T
2-aminooctanoate	rs12967143	18:53099012	G	C	8368	-0.00251879	-0.00251879	0.0163739	-0.0256	0.0024	0.6984	C
2-aminooctanoate	rs7241572	18:77580712	G	A	8368	-0.0188537	-0.0188537	0.0189404	0.02	0.0027	0.201	A
2-aminooctanoate	rs12624433	20:44680853	G	A	8368	0.0260545	0.0260545	0.017382	0.0191	0.0025	0.2584	A
2-aminooctanoate	rs5995992	22:41487218	T	C	8368	0.0110774	-0.0110774	0.0166747	-0.0301	0.0024	0.7155	T
10-undecenoate (11:1n1)	rs301799	1:8489302	C	T	8802	-0.0123635	-0.0123635	0.015053	-0.0254	0.0022	0.5694	T
10-undecenoate (11:1n1)	rs1002656	1:37192741	C	T	8802	-0.00516961	-0.00516961	0.0167666	-0.021	0.0024	0.7033	T
10-undecenoate (11:1n1)	rs11579246	1:50559162	A	G	8802	0.00970482	-0.00970482	0.028584	0.032	0.0037	0.9067	A
10-undecenoate (11:1n1)	rs1890946	1:52342427	T	C	8802	0.0112274	-0.0112274	0.0150504	-0.0194	0.0022	0.4671	T
10-undecenoate (11:1n1)	rs10789214	1:67146817	C	T	8802	-0.00392558	-0.00392558	0.0151908	0.0137	0.0022	0.5661	T
10-undecenoate (11:1n1)	rs2568958	1:72765116	G	A	8802	0.00903916	0.00903916	0.0153577	0.0336	0.0022	0.6156	A
10-undecenoate (11:1n1)	rs10890020	1:73668836	A	G	8802	-0.0179884	0.0179884	0.014972	-0.0267	0.0022	0.5156	A
10-undecenoate (11:1n1)	rs113188507	1:80809636	G	A	8802	0.0104685	0.0104685	0.0166917	0.0234	0.0025	0.2838	A
10-undecenoate (11:1n1)	rs10913112	1:175913828	C	T	8802	0.00778146	0.00778146	0.0154507	-0.0214	0.0023	0.3767	T
10-undecenoate (11:1n1)	rs169235	1:181740924	A	G	8802	0.000473339	-0.000473339	0.0174368	-0.017	0.0025	0.753	A
10-undecenoate (11:1n1)	rs17641524	1:197704717	C	T	8802	-0.0232314	-0.0232314	0.0185367	-0.0243	0.0027	0.2091	T

10-undecenoate (11:1n1)	rs12052908	2:22503044	A	T	8802	-0.00317866	0.00317866	0.0151044	-0.0211	0.0022	0.5325	A
10-undecenoate (11:1n1)	rs1568452	2:58012833	C	T	8802	0.00767173	0.00767173	0.0153166	0.0293	0.0022	0.3851	T
10-undecenoate (11:1n1)	rs7585722	2:86819128	T	C	8802	-0.0386866	0.0386866	0.0219144	-0.0215	0.003	0.8458	T
10-undecenoate (11:1n1)	rs1226412	2:157111313	C	T	8802	0.0243548	0.0243548	0.0183161	0.0232	0.0027	0.7917	T
10-undecenoate (11:1n1)	rs62188629	2:208044470	G	A	8802	-0.00187108	-0.00187108	0.0161532	0.017	0.0024	0.3136	A
10-undecenoate (11:1n1)	rs13084037	3:49214066	G	A	8802	-0.0367715	-0.0367715	0.0178022	-0.0217	0.0025	0.774	A
10-undecenoate (11:1n1)	rs6783233	3:117509984	C	T	8802	0.00771316	0.00771316	0.0166386	0.0177	0.0024	0.2833	T
10-undecenoate (11:1n1)	rs1095626	3:157977962	T	C	8802	-0.0106736	0.0106736	0.0152679	-0.0285	0.0022	0.5799	T
10-undecenoate (11:1n1)	rs7685686	4:3207142	A	G	8802	-0.0120439	0.0120439	0.0152512	0.0173	0.0022	0.5753	A
10-undecenoate (11:1n1)	rs34937911	4:42110353	T	C	8802	0.00209852	-0.00209852	0.023452	0.0244	0.0034	0.8838	T
10-undecenoate (11:1n1)	rs45510091	4:123186393	A	G	8802	-0.0296289	0.0296289	0.0325368	0.0463	0.0049	0.9472	A
10-undecenoate (11:1n1)	rs60157091	5:61509655	C	T	8802	0.0169962	0.0169962	0.0149317	0.0205	0.0022	0.515	T
10-undecenoate (11:1n1)	rs3099439	5:87545318	C	T	8802	0.00175159	0.00175159	0.0149431	-0.0208	0.0022	0.5288	T
10-undecenoate (11:1n1)	rs10061069	5:93071630	G	C	8802	0.000263376	0.000263376	0.0182158	-0.025	0.0026	0.2212	C
10-undecenoate (11:1n1)	rs30266	5:103972357	G	A	8802	0.0183614	0.0183614	0.0160135	0.0323	0.0023	0.3296	A
10-undecenoate (11:1n1)	rs11135349	5:164523472	A	C	8802	-0.00740171	0.00740171	0.0150543	-0.025	0.0022	0.4713	A
10-undecenoate (11:1n1)	rs200949	6:27835435	A	G	8802	-0.01129	0.01129	0.0211625	0.0433	0.0034	0.8744	A
10-undecenoate (11:1n1)	rs7758630	6:101387304	T	A	8802	0.00163951	0.00163951	0.0153892	-0.0165	0.0022	0.4051	A
10-undecenoate (11:1n1)	rs1933802	6:105365891	C	G	8802	0.0123444	-0.0123444	0.0149673	-0.0198	0.0022	0.4536	C
10-undecenoate (11:1n1)	rs2876520	6:142996618	C	G	8802	-0.00613562	0.00613562	0.0150719	-0.0187	0.0022	0.5271	C
10-undecenoate (11:1n1)	rs2029865	6:165121844	T	A	8802	-0.00822642	-0.00822642	0.0148901	-0.0184	0.0022	0.4534	A
10-undecenoate (11:1n1)	rs3823624	7:2110346	T	C	8802	-0.00709957	0.00709957	0.0191809	0.0294	0.0028	0.8067	T
10-undecenoate (11:1n1)	rs2043539	7:12253880	G	A	8802	0.0178799	0.0178799	0.0151764	0.022	0.0022	0.4177	A
10-undecenoate (11:1n1)	rs2247523	7:82454404	C	G	8802	0.0034486	-0.0034486	0.0152254	-0.0159	0.0021	0.5319	C
10-undecenoate (11:1n1)	rs16887442	7:82936909	T	C	8802	-0.000319366	0.000319366	0.0150826	0.0148	0.0022	0.4347	T
10-undecenoate (11:1n1)	rs58104186	7:109099919	G	A	8802	-0.024009	-0.024009	0.0149679	0.019	0.0022	0.4689	A
10-undecenoate (11:1n1)	rs7807677	7:117502574	C	T	8802	-0.00434415	-0.00434415	0.0150212	0.0211	0.0022	0.5505	T
10-undecenoate (11:1n1)	rs7837935	8:65562019	T	G	8802	-0.00815366	0.00815366	0.0210445	-0.0226	0.0031	0.1522	T
10-undecenoate (11:1n1)	rs1354115	9:2983774	C	A	8802	-0.0403649	-0.0403649	0.0155184	0.0186	0.0022	0.6243	A
10-undecenoate (11:1n1)	rs263645	9:17016503	A	T	8802	-0.00114851	0.00114851	0.0150551	0.0176	0.0022	0.5438	A
10-undecenoate (11:1n1)	rs3793577	9:23737627	A	G	8802	0.0342616	-0.0342616	0.0149856	-0.0211	0.0022	0.4665	A
10-undecenoate (11:1n1)	rs34653192	9:31124452	G	C	8802	0.0111517	0.0111517	0.0161837	-0.0196	0.0024	0.3196	C
10-undecenoate (11:1n1)	rs7030813	9:36999369	C	T	8802	-0.0140342	-0.0140342	0.0156281	0.0251	0.0022	0.3736	T
10-undecenoate (11:1n1)	rs10817969	9:119731045	T	G	8802	-0.0186452	0.0186452	0.0168763	0.0169	0.0024	0.7173	T
10-undecenoate (11:1n1)	rs913930	9:120484009	G	A	8802	-0.0218643	-0.0218643	0.0157334	-0.0219	0.0023	0.6433	A
10-undecenoate (11:1n1)	rs2670139	9:126634255	T	C	8802	0.0255674	-0.0255674	0.0176876	-0.0178	0.0025	0.7609	T
10-undecenoate (11:1n1)	rs997934	10:1795194	T	C	8802	-0.0302921	0.0302921	0.0155826	0.0163	0.0023	0.3795	T
10-undecenoate (11:1n1)	rs1021363	10:106610839	A	G	8802	-0.03549	0.03549	0.0159186	0.0226	0.0023	0.3547	A
10-undecenoate (11:1n1)	rs1448938	11:30892824	A	G	8802	-0.00551915	0.00551915	0.0151987	0.0172	0.0022	0.4171	A
10-undecenoate (11:1n1)	rs2509805	11:57650796	T	C	8802	0.0167107	-0.0167107	0.0160823	0.0191	0.0024	0.3209	T
10-undecenoate (11:1n1)	rs198457	11:61471678	C	T	8802	-0.0105505	-0.0105505	0.0193044	-0.0213	0.0028	0.1925	T
10-undecenoate (11:1n1)	rs58621819	11:65314830	A	T	8802	-0.0315616	0.0315616	0.0185108	-0.0169	0.0027	0.7903	A
10-undecenoate (11:1n1)	rs7117514	11:70544937	A	G	8802	-0.0166428	0.0166428	0.0148934	-0.0166	0.0022	0.5417	A
10-undecenoate (11:1n1)	rs7932640	11:88744425	T	C	8802	0.00333243	-0.00333243	0.0151747	0.0227	0.0022	0.4417	T
10-undecenoate (11:1n1)	rs61902811	11:113370758	G	A	8802	-0.0371694	-0.0371694	0.0155833	-0.0289	0.0022	0.3682	A
10-undecenoate (11:1n1)	rs78337797	12:23987925	T	G	8802	0.0143049	-0.0143049	0.0228206	0.0229	0.0034	0.8781	T
10-undecenoate (11:1n1)	rs56314503	12:84465022	T	G	8802	-0.0206957	0.0206957	0.017985	-0.0203	0.0025	0.7487	T
10-undecenoate (11:1n1)	rs3213572	12:121205078	G	A	8802	0.0103033	0.0103033	0.0150042	0.0196	0.0021	0.4745	A
10-undecenoate (11:1n1)	rs1343605	13:53647048	A	C	8802	0.0109919	-0.0109919	0.0152838	0.0231	0.0022	0.384	A
10-undecenoate (11:1n1)	rs9592461	13:66941792	A	G	8802	0.00687139	-0.00687139	0.0150238	0.0243	0.0022	0.4874	A
10-undecenoate (11:1n1)	rs4772087	13:99115041	C	T	8802	-0.00916445	-0.00916445	0.0155797	0.0209	0.0023	0.3732	T
10-undecenoate (11:1n1)	rs61990288	14:42074726	A	G	8802	-0.00229076	0.00229076	0.0149836	-0.0249	0.0021	0.5083	A
10-undecenoate (11:1n1)	rs1956373	14:60141822	T	G	8802	0.00977464	-0.00977464	0.0171667	-0.0169	0.0025	0.7436	T
10-undecenoate (11:1n1)	rs1152578	14:64697037	C	T	8802	-0.0026073	-0.0026073	0.015176	-0.0152	0.0022	0.4357	T
10-undecenoate (11:1n1)	rs1045430	14:75130235	G	T	8802	-0.0336402	-0.0336402	0.0149812	-0.0226	0.0022	0.4792	T
10-undecenoate (11:1n1)	rs10149470	14:104017953	G	A	8802	-0.0224094	-0.0224094	0.0149847	-0.0206	0.0021	0.4869	A
10-undecenoate (11:1n1)	rs8037355	15:37643831	C	T	8802	-0.00886572	-0.00886572	0.0150924	-0.0192	0.0022	0.5556	T
10-undecenoate (11:1n1)	rs34488670	15:47684936	T	C	8802	0.0218996	-0.0218996	0.0188602	-0.0193	0.0027	0.7887	T
10-undecenoate (11:1n1)	rs7193263	16:6315880	G	A	8802	-0.0257422	-0.0257422	0.0158665	-0.0206	0.0023	0.6679	A
10-undecenoate (11:1n1)	rs7198928	16:7666402	T	C	8802	-0.0162228	0.0162228	0.015541	0.0219	0.0022	0.6159	T
10-undecenoate (11:1n1)	rs7200826	16:13066833	C	T	8802	-0.0228633	-0.0228633	0.0170889	0.027	0.0025	0.2551	T
10-undecenoate (11:1n1)	rs56887639	16:13755530	A	G	8802	0.025187	-0.025187	0.0170602	-0.0171	0.0024	0.7264	A
10-undecenoate (11:1n1)	rs12923444	16:21639710	A	C	8802	-0.00783554	0.00783554	0.0154693	-0.0237	0.0023	0.5625	A
10-undecenoate (11:1n1)	rs75581564	17:27363750	G	A	8802	-0.0305314	-0.0305314	0.0231906	0.0248	0.0034	0.1165	A
10-undecenoate (11:1n1)	rs12967855	18:35138245	A	G	8802	0.00106549	-0.00106549	0.0160678	0.0207	0.0023	0.3295	A
10-undecenoate (11:1n1)	rs7227069	18:50731802	G	A	8802	-0.00745628	-0.00745628	0.0151692	0.0242	0.0022	0.4326	A
10-undecenoate (11:1n1)	rs62091461	18:52488672	C	T	8802	-0.00795153	-0.00795153	0.0179518	-0.0199	0.0026	0.2274	T
10-undecenoate (11:1n1)	rs12967143	18:53099012	G	C	8802	-0.011071	-0.011071	0.016138	-0.0256	0.0024	0.6984	C
10-undecenoate (11:1n1)	rs7241572	18:77580712	G	A	8802	-0.00384062	-0.00384062	0.0186311	0.02	0.0027	0.201	A
10-undecenoate (11:1n1)	rs12624433	20:44680853	G	A	8802	-0.0182555	-0.0182555	0.0171088	0.0191	0.0025	0.2584	A
10-undecenoate (11:1n1)	rs5995992	22:41487218	T	C	8802	-0.0401233	0.0401233	0.0163778	-0.0301	0.0024	0.7155	T
Hippurate	rs301799	1:8489302	C	T	8809	0.0141826	0.0141826	0.0148528	-0.0254	0.0022	0.5694	T
Hippurate	rs1002656	1:37192741	C	T	8809	-0.00681887	-0.00681887	0.0165393	-0.021	0.0024	0.7033	T
Hippurate	rs11579246	1:50559162	A	G	8809	-0.00350767	0.00350767	0.0281934	0.032	0.0037	0.9067	A
Hippurate	rs1890946	1:52342427	T	C	8809	0.0106217	-0.0106217	0.0148438	-0.0194	0.0022	0.4671	T
Hippurate	rs10789214	1:67146817	C	T	8809	-0.0229166	-0.0229166	0.0149859	0.0137	0.0022	0.5661	T
Hippurate	rs2568958	1:72765116	G	A	8809	-0.010764	-0.010764	0.0151512	0.0336	0.0022	0.6156	A
Hippurate	rs10890020	1:73668836	A	G	8809	-0.00103688	0.00103688	0.0147687	-0.0267	0.0022	0.5156	A
Hippurate	rs113188507	1:80809636	G	A	8809	-0.00917418	-0.00917418	0.0164609	0.0234	0.0025	0.2838	A
Hippurate	rs10913112	1:175913828	C	T	8809	0.00926792	0.00926792	0.0152448	-0.0214	0.0023	0.3767	T
Hippurate	rs169235	1:181740924	A	G	8809	0.0205356	-0.0205356	0.0172037	-0.017	0.0025	0.753	A
Hippurate	rs17641524	1:197704717	C	T	8809	-0.0077805	-0.0077805	0.0182843	-0.0243	0.0027	0.2091	T
Hippurate	rs12052908	2:22503044	A	T	8809	-0.0178242	0.0178242	0.0149043	-0.0211	0.0022	0.5325	A
Hippurate	rs1568452	2:58012833	C	T	8809	-0.0134968	-0.0134968	0.0151104	0.0293	0.0022	0.3851	T
Hippurate	rs7585722	2:86819128	T	C	8809	0.0249323	-0.0249323	0.0216284	-0.0215	0.003	0.8458	T
Hippurate	rs1226412	2:157111313	C	T	8809	-0.0428743	-0.0428743	0.0180696	0.0232	0.0027	0.7917	T
Hippurate	rs62188629	2:208044470	G	A	8809	-0.00112523	-0.00112523	0.0159366	0.017	0.0024	0.3136	A
Hippurate	rs13084037	3:49214066	G	A	8809	-0.0167557	-0.0167557	0.0175687	-0.0217	0.0025	0.774	A
Hippurate	rs6783233	3:117509984	C	T	8809	0.00762416	0.00762416	0.0164179	0.0177	0.0024	0.2833	T

Hippurate	rs1095626	3:157977962	T	C	8809	-0.0172481	0.0172481	0.0150608	-0.0285	0.0022	0.5799	T
Hippurate	rs7685686	4:3207142	A	G	8809	0.0139713	-0.0139713	0.0150494	0.0173	0.0022	0.5753	A
Hippurate	rs34937911	4:42110353	T	C	8809	-0.0103301	0.0103301	0.0231437	0.0244	0.0034	0.8838	T
Hippurate	rs45510091	4:123186393	A	G	8809	0.000239956	-0.000239956	0.0320996	0.0463	0.0049	0.9472	A
Hippurate	rs60157091	5:61509655	C	T	8809	0.00569473	0.00569473	0.0147281	0.0205	0.0022	0.515	T
Hippurate	rs3099439	5:87545318	C	T	8809	-0.00747827	-0.00747827	0.0147433	-0.0208	0.0022	0.5288	T
Hippurate	rs10061069	5:93071630	G	C	8809	0.0199293	0.0199293	0.0179721	-0.025	0.0026	0.2212	C
Hippurate	rs30266	5:103972357	G	A	8809	-0.0135887	-0.0135887	0.0158006	0.0323	0.0023	0.3296	A
Hippurate	rs11135349	5:164523472	A	C	8809	-0.00563732	0.00563732	0.0148547	-0.025	0.0022	0.4713	A
Hippurate	rs200949	6:27835435	A	G	8809	-0.0245683	0.0245683	0.0208788	0.0433	0.0034	0.8744	A
Hippurate	rs7758630	6:101387304	T	A	8809	0.00102821	0.00102821	0.0151824	-0.0165	0.0022	0.4051	A
Hippurate	rs1933802	6:105365891	C	G	8809	0.0164437	-0.0164437	0.0147682	-0.0198	0.0022	0.4536	C
Hippurate	rs2876520	6:142996618	C	G	8809	-0.00995499	0.00995499	0.0148678	-0.0187	0.0022	0.5271	C
Hippurate	rs2029865	6:165121844	T	A	8809	0.0112855	0.0112855	0.0146931	-0.0184	0.0022	0.4534	A
Hippurate	rs3823624	7:2110346	T	C	8809	0.00550075	-0.00550075	0.0189258	0.0294	0.0028	0.8067	T
Hippurate	rs2043539	7:12253880	G	A	8809	-0.0150551	-0.0150551	0.0149713	0.022	0.0022	0.4177	A
Hippurate	rs2247523	7:82454404	C	G	8809	0.00341027	-0.00341027	0.0150184	-0.0159	0.0021	0.5319	C
Hippurate	rs16887442	7:82936909	T	C	8809	0.0133888	-0.0133888	0.0148781	0.0148	0.0022	0.4347	T
Hippurate	rs58104186	7:109099919	G	A	8809	0.00623074	0.00623074	0.0147682	0.019	0.0022	0.4689	A
Hippurate	rs7807677	7:117502574	C	T	8809	-0.03103	-0.03103	0.0148143	0.0211	0.0022	0.5505	T
Hippurate	rs7837935	8:65562019	T	G	8809	-0.0283004	0.0283004	0.0207616	-0.0226	0.0031	0.1522	T
Hippurate	rs1354115	9:2983774	C	A	8809	-0.0191054	-0.0191054	0.0153143	0.0186	0.0022	0.6243	A
Hippurate	rs263645	9:17016503	A	T	8809	-0.0287362	0.0287362	0.0148487	0.0176	0.0022	0.5438	A
Hippurate	rs3793577	9:23737627	A	G	8809	0.00102253	-0.00102253	0.0147925	-0.0211	0.0022	0.4665	A
Hippurate	rs34653192	9:31124452	G	C	8809	-0.0235014	-0.0235014	0.0159665	-0.0196	0.0024	0.3196	C
Hippurate	rs7030813	9:36999369	C	T	8809	-0.0400646	-0.0400646	0.0154136	0.0251	0.0022	0.3736	T
Hippurate	rs10817969	9:119731045	T	G	8809	0.00448921	-0.00448921	0.0166488	0.0169	0.0024	0.7173	T
Hippurate	rs913930	9:120484009	G	A	8809	0.0126216	0.0126216	0.0155246	-0.0219	0.0023	0.6433	A
Hippurate	rs2670139	9:126634255	T	C	8809	-0.0332044	0.0332044	0.0174452	-0.0178	0.0025	0.7609	T
Hippurate	rs997934	10:1795194	T	C	8809	-0.00331727	0.00331727	0.0153771	0.0163	0.0023	0.3795	T
Hippurate	rs1021363	10:106610839	A	G	8809	-0.0166568	0.0166568	0.0157036	0.0226	0.0023	0.3547	A
Hippurate	rs1448938	11:30892824	A	G	8809	0.0499084	-0.0499084	0.0149828	0.0172	0.0022	0.4171	A
Hippurate	rs2509805	11:57650796	T	C	8809	-0.00827274	0.00827274	0.0158628	0.0191	0.0024	0.3209	T
Hippurate	rs198457	11:61471678	C	T	8809	0.00221932	0.00221932	0.019046	-0.0213	0.0028	0.1925	T
Hippurate	rs58621819	11:65314830	A	T	8809	-0.0110743	0.0110743	0.0182677	-0.0169	0.0027	0.7903	A
Hippurate	rs7117514	11:70544937	A	G	8809	0.00158694	-0.00158694	0.014697	-0.0166	0.0022	0.5417	A
Hippurate	rs7932640	11:88744425	T	C	8809	-0.0331522	0.0331522	0.0149643	0.0227	0.0022	0.4417	T
Hippurate	rs61902811	11:113370758	G	A	8809	-0.0184083	-0.0184083	0.0153787	-0.0289	0.0022	0.3682	A
Hippurate	rs78337797	12:23987925	T	G	8809	0.0402659	-0.0402659	0.0225172	0.0229	0.0034	0.8781	T
Hippurate	rs56314503	12:84465022	T	G	8809	-0.00513718	0.00513718	0.0177469	-0.0203	0.0025	0.7487	T
Hippurate	rs3213572	12:121205078	G	A	8809	0.0127138	0.0127138	0.0148017	0.0196	0.0021	0.4745	A
Hippurate	rs1343605	13:53647048	A	C	8809	-0.020014	0.020014	0.015079	0.0231	0.0022	0.384	A
Hippurate	rs9592461	13:66941792	A	G	8809	-0.0039752	0.0039752	0.0148199	0.0243	0.0022	0.4874	A
Hippurate	rs4772087	13:99115041	C	T	8809	-0.0278672	-0.0278672	0.0153696	0.0209	0.0023	0.3732	T
Hippurate	rs61990288	14:42074726	A	G	8809	-0.0218143	0.0218143	0.0147784	-0.0249	0.0021	0.5083	A
Hippurate	rs1956373	14:60141822	T	G	8809	0.0132884	-0.0132884	0.0169382	-0.0169	0.0025	0.7436	T
Hippurate	rs1152578	14:64697037	C	T	8809	-0.0035377	-0.0035377	0.0149746	-0.0152	0.0022	0.4357	T
Hippurate	rs1045430	14:75130235	G	T	8809	-0.0123076	-0.0123076	0.0147774	-0.0226	0.0022	0.4792	T
Hippurate	rs10149470	14:104017953	G	A	8809	0.00715821	0.00715821	0.0147877	-0.0206	0.0021	0.4869	A
Hippurate	rs8037355	15:37643831	C	T	8809	0.00768815	0.00768815	0.0148901	-0.0192	0.0022	0.5556	T
Hippurate	rs34488670	15:47684936	T	C	8809	-0.0153624	0.0153624	0.0186101	-0.0193	0.0027	0.7887	T
Hippurate	rs7193263	16:6315880	G	A	8809	0.00178786	0.00178786	0.0156564	-0.0206	0.0023	0.6679	A
Hippurate	rs7198928	16:7666402	T	C	8809	-0.0376635	0.0376635	0.0153261	0.0219	0.0022	0.6159	T
Hippurate	rs7200826	16:13066833	C	T	8809	0.0054748	0.0054748	0.0168587	0.027	0.0025	0.2551	T
Hippurate	rs56887639	16:13755530	A	G	8809	-0.00050033	0.00050033	0.0168309	-0.0171	0.0024	0.7264	A
Hippurate	rs12923444	16:21639710	A	C	8809	0.0214787	-0.0214787	0.0152636	-0.0237	0.0023	0.5625	A
Hippurate	rs75581564	17:27363750	G	A	8809	0.0101536	0.0101536	0.0228844	0.0248	0.0034	0.1165	A
Hippurate	rs12967855	18:35138245	A	G	8809	-0.0140885	0.0140885	0.0158471	0.0207	0.0023	0.3295	A
Hippurate	rs7227069	18:50731802	G	A	8809	0.00263449	0.00263449	0.0149653	0.0242	0.0022	0.4326	A
Hippurate	rs62091461	18:52488672	C	T	8809	0.00894246	0.00894246	0.0177128	-0.0199	0.0026	0.2274	T
Hippurate	rs12967143	18:53099012	G	C	8809	0.0142635	0.0142635	0.01592	-0.0256	0.0024	0.6984	C
Hippurate	rs7241572	18:77580712	G	A	8809	-0.00969567	-0.00969567	0.0183836	0.02	0.0027	0.201	A
Hippurate	rs12624433	20:44680853	G	A	8809	0.0119615	0.0119615	0.0168772	0.0191	0.0025	0.2584	A
Hippurate	rs5995992	22:41487218	T	C	8809	-0.00871802	0.00871802	0.016162	-0.0301	0.0024	0.7155	T
Mannitol/Sorbitol	rs301799	1:8489302	C	T	8798	-0.0137667	-0.0137667	0.0150125	-0.0254	0.0022	0.5694	T
Mannitol/Sorbitol	rs1002656	1:37192741	C	T	8798	-0.00808711	-0.00808711	0.016718	-0.021	0.0024	0.7033	T
Mannitol/Sorbitol	rs11579246	1:50559162	A	G	8798	-0.00229079	0.00229079	0.0285059	0.032	0.0037	0.9067	A
Mannitol/Sorbitol	rs1890946	1:52342427	T	C	8798	-0.0337548	0.0337548	0.0150058	-0.0194	0.0022	0.4671	T
Mannitol/Sorbitol	rs10789214	1:67146817	C	T	8798	0.0181741	0.0181741	0.015155	0.0137	0.0022	0.5661	T
Mannitol/Sorbitol	rs2568958	1:72765116	G	A	8798	-0.00286696	-0.00286696	0.0153159	0.0336	0.0022	0.6156	A
Mannitol/Sorbitol	rs10890020	1:73668836	A	G	8798	0.0115925	-0.0115925	0.0149311	-0.0267	0.0022	0.5156	A
Mannitol/Sorbitol	rs113188507	1:80809636	G	A	8798	-0.0347659	-0.0347659	0.0166386	0.0234	0.0025	0.2838	A
Mannitol/Sorbitol	rs10913112	1:175913828	C	T	8798	0.033606	0.033606	0.0154074	-0.0214	0.0023	0.3767	T
Mannitol/Sorbitol	rs169235	1:181740924	A	G	8798	-0.0246836	0.0246836	0.0173949	-0.017	0.0025	0.753	A
Mannitol/Sorbitol	rs17641524	1:197704717	C	T	8798	-0.0173588	-0.0173588	0.018482	-0.0243	0.0027	0.2091	T
Mannitol/Sorbitol	rs12052908	2:22503044	A	T	8798	0.0228755	-0.0228755	0.0150655	-0.0211	0.0022	0.5325	A
Mannitol/Sorbitol	rs1568452	2:58012833	C	T	8798	0.00105298	0.00105298	0.0152769	0.0293	0.0022	0.3851	T
Mannitol/Sorbitol	rs7585722	2:86819128	T	C	8798	0.0177976	-0.0177976	0.0218585	-0.0215	0.003	0.8458	T
Mannitol/Sorbitol	rs1226412	2:157111313	C	T	8798	0.0199385	0.0199385	0.0182693	0.0232	0.0027	0.7917	T
Mannitol/Sorbitol	rs62188629	2:208044470	G	A	8798	-0.00807213	-0.00807213	0.0161093	0.017	0.0024	0.3136	A
Mannitol/Sorbitol	rs13084037	3:49214066	G	A	8798	0.0156129	0.0156129	0.0177596	-0.0217	0.0025	0.774	A
Mannitol/Sorbitol	rs6783233	3:117509984	C	T	8798	-0.0141453	-0.0141453	0.0165949	0.0177	0.0024	0.2833	T
Mannitol/Sorbitol	rs1095626	3:157977962	T	C	8798	0.0255857	-0.0255857	0.0152239	-0.0285	0.0022	0.5799	T
Mannitol/Sorbitol	rs7685686	4:3207142	A	G	8798	0.00705965	-0.00705965	0.0152156	0.0173	0.0022	0.5753	A
Mannitol/Sorbitol	rs34937911	4:42110353	T	C	8798	0.0156449	-0.0156449	0.0233949	0.0244	0.0034	0.8838	T
Mannitol/Sorbitol	rs45510091	4:123186393	A	G	8798	-0.00622341	0.00622341	0.032436	0.0463	0.0049	0.9472	A
Mannitol/Sorbitol	rs60157091	5:61509655	C	T	8798	-0.00669238	-0.00669238	0.0148928	0.0205	0.0022	0.515	T
Mannitol/Sorbitol	rs3099439	5:87545318	C	T	8798	-0.00818971	-0.00818971	0.0149088	-0.0208	0.0022	0.5288	T
Mannitol/Sorbitol	rs10061069	5:93071630	G	C	8798	0.0368584	0.0368584	0.0181635	-0.025	0.0026	0.2212	C

Mannitol/Sorbitol	rs30266	5:103972357	G	A	8798	0.0130275	0.0130275	0.0159709	0.0323	0.0023	0.3296	A
Mannitol/Sorbitol	rs11135349	5:164523472	A	C	8798	0.0137241	-0.0137241	0.0150206	-0.025	0.0022	0.4713	A
Mannitol/Sorbitol	rs200949	6:27835435	A	G	8798	0.000277472	-0.000277472	0.0211059	0.0433	0.0034	0.8744	A
Mannitol/Sorbitol	rs7758630	6:101387304	T	A	8798	-0.00283744	-0.00283744	0.0153492	-0.0165	0.0022	0.4051	A
Mannitol/Sorbitol	rs1933802	6:105365891	C	G	8798	-0.00219911	0.00219911	0.0149278	-0.0198	0.0022	0.4536	C
Mannitol/Sorbitol	rs2876520	6:142996618	C	G	8798	0.0216477	-0.0216477	0.0150289	-0.0187	0.0022	0.5271	C
Mannitol/Sorbitol	rs2029865	6:165121844	T	A	8798	0.00512563	0.00512563	0.0148572	-0.0184	0.0022	0.4534	A
Mannitol/Sorbitol	rs3823624	7:2110346	T	C	8798	0.0535715	-0.0535715	0.0191297	0.0294	0.0028	0.8067	T
Mannitol/Sorbitol	rs2043539	7:12253880	G	A	8798	-0.00103408	-0.00103408	0.0151381	0.022	0.0022	0.4177	A
Mannitol/Sorbitol	rs2247523	7:82454404	C	G	8798	-0.00357173	0.00357173	0.0151865	-0.0159	0.0021	0.5319	C
Mannitol/Sorbitol	rs16887442	7:82936909	T	C	8798	-0.0133208	0.0133208	0.0150414	0.0148	0.0022	0.4347	T
Mannitol/Sorbitol	rs58104186	7:109099919	G	A	8798	0.00198141	0.00198141	0.0149288	0.019	0.0022	0.4689	A
Mannitol/Sorbitol	rs7807677	7:117502574	C	T	8798	-0.00304723	-0.00304723	0.0149764	0.0211	0.0022	0.5505	T
Mannitol/Sorbitol	rs7837935	8:65562019	T	G	8798	-0.0458543	0.0458543	0.0209976	-0.0226	0.0031	0.1522	T
Mannitol/Sorbitol	rs1354115	9:2983774	C	A	8798	0.00844738	0.00844738	0.0154818	0.0186	0.0022	0.6243	A
Mannitol/Sorbitol	rs263645	9:17016503	A	T	8798	-0.000930523	0.000930523	0.0150147	0.0176	0.0022	0.5438	A
Mannitol/Sorbitol	rs3793577	9:23737627	A	G	8798	-0.00554376	0.00554376	0.0149556	-0.0211	0.0022	0.4665	A
Mannitol/Sorbitol	rs34653192	9:31124452	G	C	8798	0.00160302	0.00160302	0.0161451	-0.0196	0.0024	0.3196	C
Mannitol/Sorbitol	rs7030813	9:36999369	C	T	8798	-0.0119974	-0.0119974	0.0155934	0.0251	0.0022	0.3736	T
Mannitol/Sorbitol	rs10817969	9:119731045	T	G	8798	7.82E-05	-0.0000782	0.0168282	0.0169	0.0024	0.7173	T
Mannitol/Sorbitol	rs913930	9:120484009	G	A	8798	0.0161831	0.0161831	0.0156973	-0.0219	0.0023	0.6433	A
Mannitol/Sorbitol	rs2670139	9:126634255	T	C	8798	0.00838827	-0.00838827	0.0176489	-0.0178	0.0025	0.7609	T
Mannitol/Sorbitol	rs997934	10:1795194	T	C	8798	0.0294995	-0.0294995	0.0155411	0.0163	0.0023	0.3795	T
Mannitol/Sorbitol	rs1021363	10:106610839	A	G	8798	0.000877048	-0.000877048	0.0158732	0.0226	0.0023	0.3547	A
Mannitol/Sorbitol	rs1448938	11:30892824	A	G	8798	-0.00232577	0.00232577	0.0151535	0.0172	0.0022	0.4171	A
Mannitol/Sorbitol	rs2509805	11:57650796	T	C	8798	0.0265796	-0.0265796	0.016038	0.0191	0.0024	0.3209	T
Mannitol/Sorbitol	rs198457	11:61471678	C	T	8798	0.0106619	0.0106619	0.019252	-0.0213	0.0028	0.1925	T
Mannitol/Sorbitol	rs58621819	11:65314830	A	T	8798	0.0228458	-0.0228458	0.0184654	-0.0169	0.0027	0.7903	A
Mannitol/Sorbitol	rs7117514	11:70544937	A	G	8798	0.000684866	-0.000684866	0.0148604	-0.0166	0.0022	0.5417	A
Mannitol/Sorbitol	rs7932640	11:88744425	T	C	8798	0.00368136	-0.00368136	0.0151329	0.0227	0.0022	0.4417	T
Mannitol/Sorbitol	rs61902811	11:113370758	G	A	8798	-0.00988552	-0.00988552	0.0155475	-0.0289	0.0022	0.3682	A
Mannitol/Sorbitol	rs78337797	12:23987925	T	G	8798	-0.0319263	0.0319263	0.0227599	0.0229	0.0034	0.8781	T
Mannitol/Sorbitol	rs56314503	12:84465022	T	G	8798	0.0113427	-0.0113427	0.0179456	-0.0203	0.0025	0.7487	T
Mannitol/Sorbitol	rs3213572	12:121205078	G	A	8798	-0.0198657	-0.0198657	0.0149613	0.0196	0.0021	0.4745	A
Mannitol/Sorbitol	rs1343605	13:53647048	A	C	8798	-0.026999	0.026999	0.0152437	0.0231	0.0022	0.384	A
Mannitol/Sorbitol	rs9592461	13:66941792	A	G	8798	-0.00498302	0.00498302	0.0149822	0.0243	0.0022	0.4874	A
Mannitol/Sorbitol	rs4772087	13:99115041	C	T	8798	-0.00131071	-0.00131071	0.0155431	0.0209	0.0023	0.3732	T
Mannitol/Sorbitol	rs61990288	14:42074726	A	G	8798	0.00085253	-0.00085253	0.0149442	-0.0249	0.0021	0.5083	A
Mannitol/Sorbitol	rs1956373	14:60141822	T	G	8798	-0.0148888	0.0148888	0.0171215	-0.0169	0.0025	0.7436	T
Mannitol/Sorbitol	rs1152578	14:64697037	C	T	8798	-0.0129758	-0.0129758	0.0151412	-0.0152	0.0022	0.4357	T
Mannitol/Sorbitol	rs1045430	14:75130235	G	T	8798	0.0124827	0.0124827	0.0149477	-0.0226	0.0022	0.4792	T
Mannitol/Sorbitol	rs10149470	14:104017953	G	A	8798	-0.00285261	-0.00285261	0.0149518	-0.0206	0.0021	0.4869	A
Mannitol/Sorbitol	rs8037355	15:37643831	C	T	8798	0.0160367	0.0160367	0.0150541	-0.0192	0.0022	0.5556	T
Mannitol/Sorbitol	rs34488670	15:47684936	T	C	8798	-0.00913699	0.00913699	0.0188113	-0.0193	0.0027	0.7887	T
Mannitol/Sorbitol	rs7193263	16:6315880	G	A	8798	-0.0152856	-0.0152856	0.0158336	-0.0206	0.0023	0.6679	A
Mannitol/Sorbitol	rs7198928	16:7666402	T	C	8798	-0.0155215	0.0155215	0.0155005	0.0219	0.0022	0.6159	T
Mannitol/Sorbitol	rs7200826	16:13066833	C	T	8798	0.00083993	0.00083993	0.0170412	0.027	0.0025	0.2551	T
Mannitol/Sorbitol	rs56887639	16:13755530	A	G	8798	0.0170328	-0.0170328	0.0170172	-0.0171	0.0024	0.7264	A
Mannitol/Sorbitol	rs12923444	16:21639710	A	C	8798	0.0110647	-0.0110647	0.0154363	-0.0237	0.0023	0.5625	A
Mannitol/Sorbitol	rs75581564	17:27363750	G	A	8798	0.0307259	0.0307259	0.0231309	0.0248	0.0034	0.1165	A
Mannitol/Sorbitol	rs12967855	18:35138245	A	G	8798	-0.00451165	0.00451165	0.0160179	0.0207	0.0023	0.3295	A
Mannitol/Sorbitol	rs7227069	18:50731802	G	A	8798	0.00347246	0.00347246	0.0151286	0.0242	0.0022	0.4326	A
Mannitol/Sorbitol	rs62091461	18:52488672	C	T	8798	-0.00291459	-0.00291459	0.0179053	-0.0199	0.0026	0.2274	T
Mannitol/Sorbitol	rs12967143	18:53099012	G	C	8798	0.00589434	0.00589434	0.0160967	-0.0256	0.0024	0.6984	C
Mannitol/Sorbitol	rs7241572	18:77580712	G	A	8798	-0.0196915	-0.0196915	0.0185881	0.02	0.0027	0.201	A
Mannitol/Sorbitol	rs12624433	20:44680853	G	A	8798	-0.0108932	-0.0108932	0.01706	0.0191	0.0025	0.2584	A
Mannitol/Sorbitol	rs5995992	22:41487218	T	C	8798	0.0323255	-0.0323255	0.0163373	-0.0301	0.0024	0.7155	T
Retinol	rs301799	1:8489302	C	T	8809	0.015479	0.015479	0.0146947	-0.0254	0.0022	0.5694	T
Retinol	rs1002656	1:37192741	C	T	8809	0.0232938	0.0232938	0.0163617	-0.021	0.0024	0.7033	T
Retinol	rs11579246	1:50559162	A	G	8809	0.00817336	-0.00817336	0.0278935	0.032	0.0037	0.9067	A
Retinol	rs1890946	1:52342427	T	C	8809	0.0101938	-0.0101938	0.014686	-0.0194	0.0022	0.4671	T
Retinol	rs10789214	1:67146817	C	T	8809	0.0123962	0.0123962	0.0148279	0.0137	0.0022	0.5661	T
Retinol	rs2568958	1:72765116	G	A	8809	-0.0471398	-0.0471398	0.014982	0.0336	0.0022	0.6156	A
Retinol	rs10890020	1:73668836	A	G	8809	0.00318796	-0.00318796	0.0146116	-0.0267	0.0022	0.5156	A
Retinol	rs113188507	1:80809636	G	A	8809	-0.00257587	-0.00257587	0.0162861	0.0234	0.0025	0.2838	A
Retinol	rs10913112	1:175913828	C	T	8809	-0.00654661	-0.00654661	0.0150828	-0.0214	0.0023	0.3767	T
Retinol	rs169235	1:181740924	A	G	8809	0.0187405	-0.0187405	0.0170209	-0.017	0.0025	0.753	A
Retinol	rs17641524	1:197704717	C	T	8809	-0.0056388	-0.0056388	0.0180899	-0.0243	0.0027	0.2091	T
Retinol	rs12052908	2:22503044	A	T	8809	0.00657069	-0.00657069	0.0147469	-0.0211	0.0022	0.5325	A
Retinol	rs1568452	2:58012833	C	T	8809	-0.0180641	-0.0180641	0.0149491	0.0293	0.0022	0.3851	T
Retinol	rs7585722	2:86819128	T	C	8809	0.00118505	-0.00118505	0.0214	-0.0215	0.003	0.8458	T
Retinol	rs1226412	2:157111313	C	T	8809	-0.000385705	-0.000385705	0.0178831	0.0232	0.0027	0.7917	T
Retinol	rs62188629	2:208044470	G	A	8809	-0.0208272	-0.0208272	0.0157656	0.017	0.0024	0.3136	A
Retinol	rs13084037	3:49214066	G	A	8809	-0.00751946	-0.00751946	0.0173825	-0.0217	0.0025	0.774	A
Retinol	rs6783233	3:117509984	C	T	8809	-0.0247913	-0.0247913	0.0162413	0.0177	0.0024	0.2833	T
Retinol	rs1095626	3:157977962	T	C	8809	-0.0128493	0.0128493	0.0149011	-0.0285	0.0022	0.5799	T
Retinol	rs7685686	4:3207142	A	G	8809	-0.0139171	0.0139171	0.0148893	0.0173	0.0022	0.5753	A
Retinol	rs34937911	4:42110353	T	C	8809	0.00225519	-0.00225519	0.0228978	0.0244	0.0034	0.8838	T
Retinol	rs45510091	4:123186393	A	G	8809	-0.00286433	0.00286433	0.0317582	0.0463	0.0049	0.9472	A
Retinol	rs60157091	5:61509655	C	T	8809	-0.0286673	-0.0286673	0.0145683	0.0205	0.0022	0.515	T
Retinol	rs3099439	5:87545318	C	T	8809	0.00842414	0.00842414	0.0145865	-0.0208	0.0022	0.5288	T
Retinol	rs10061069	5:93071630	G	C	8809	-0.00322719	-0.00322719	0.0177821	-0.025	0.0026	0.2212	C
Retinol	rs30266	5:103972357	G	A	8809	0.0344049	0.0344049	0.0156289	0.0323	0.0023	0.3296	A
Retinol	rs11135349	5:164523472	A	C	8809	-0.0129814	0.0129814	0.0146961	-0.025	0.0022	0.4713	A
Retinol	rs200949	6:27835435	A	G	8809	-0.0236279	0.0236279	0.0206568	0.0433	0.0034	0.8744	A
Retinol	rs7758630	6:101387304	T	A	8809	0.0172065	0.0172065	0.0150198	-0.0165	0.0022	0.4051	A
Retinol	rs1933802	6:105365891	C	G	8809	-0.0193723	0.0193723	0.0146107	-0.0198	0.0022	0.4536	C
Retinol	rs2876520	6:142996618	C	G	8809	-0.0215901	0.0215901	0.0147082	-0.0187	0.0022	0.5271	C
Retinol	rs2029865	6:165121844	T	A	8809	0.00520216	0.00520216	0.0145372	-0.0184	0.0022	0.4534	A

Retinol	rs3823624	7:2110346	T	C	8809	-0.0102145	0.0102145	0.0187243	0.0294	0.0028	0.8067	T
Retinol	rs2043539	7:12253880	G	A	8809	0.0145395	0.0145395	0.0148121	0.022	0.0022	0.4177	A
Retinol	rs2247523	7:82454404	C	G	8809	-0.0134527	0.0134527	0.014858	-0.0159	0.0021	0.5319	C
Retinol	rs16887442	7:82936909	T	C	8809	0.0371143	-0.0371143	0.0147153	0.0148	0.0022	0.4347	T
Retinol	rs58104186	7:109099919	G	A	8809	-0.000405302	-0.000405302	0.0146113	0.019	0.0022	0.4689	A
Retinol	rs7807677	7:117502574	C	T	8809	0.00711789	0.00711789	0.0146602	0.0211	0.0022	0.5505	T
Retinol	rs7837935	8:65562019	T	G	8809	0.0247158	-0.0247158	0.0205412	-0.0226	0.0031	0.1522	T
Retinol	rs1354115	9:2983774	C	A	8809	0.0181107	0.0181107	0.0151515	0.0186	0.0022	0.6243	A
Retinol	rs263645	9:17016503	A	T	8809	0.0139422	-0.0139422	0.0146931	0.0176	0.0022	0.5438	A
Retinol	rs3793577	9:23737627	A	G	8809	-0.0153454	0.0153454	0.0146342	-0.0211	0.0022	0.4665	A
Retinol	rs34653192	9:31124452	G	C	8809	0.00128993	0.00128993	0.0157986	-0.0196	0.0024	0.3196	C
Retinol	rs7030813	9:36999369	C	T	8809	-0.0130063	-0.0130063	0.0152549	0.0251	0.0022	0.3736	T
Retinol	rs10817969	9:119731045	T	G	8809	-0.0140569	0.0140569	0.0164712	0.0169	0.0024	0.7173	T
Retinol	rs913930	9:120484009	G	A	8809	-0.0386196	-0.0386196	0.0153545	-0.0219	0.0023	0.6433	A
Retinol	rs2670139	9:126634255	T	C	8809	0.0187218	-0.0187218	0.017262	-0.0178	0.0025	0.7609	T
Retinol	rs997934	10:1795194	T	C	8809	0.00584156	-0.00584156	0.0152135	0.0163	0.0023	0.3795	T
Retinol	rs1021363	10:106610839	A	G	8809	-0.0158515	0.0158515	0.0155366	0.0226	0.0023	0.3547	A
Retinol	rs1448938	11:30892824	A	G	8809	-0.00363834	0.00363834	0.0148328	0.0172	0.0022	0.4171	A
Retinol	rs2509805	11:57650796	T	C	8809	0.020929	-0.020929	0.0156927	0.0191	0.0024	0.3209	T
Retinol	rs198457	11:61471678	C	T	8809	-0.0136632	-0.0136632	0.0188429	-0.0213	0.0028	0.1925	T
Retinol	rs58621819	11:65314830	A	T	8809	0.00648067	-0.00648067	0.0180737	-0.0169	0.0027	0.7903	A
Retinol	rs7117514	11:70544937	A	G	8809	-0.00240133	0.00240133	0.0145407	-0.0166	0.0022	0.5417	A
Retinol	rs7932640	11:88744425	T	C	8809	-0.0151563	0.0151563	0.0148084	0.0227	0.0022	0.4417	T
Retinol	rs61902811	11:113370758	G	A	8809	-0.0104896	-0.0104896	0.015216	-0.0289	0.0022	0.3682	A
Retinol	rs78337797	12:23987925	T	G	8809	0.00491961	-0.00491961	0.0222817	0.0229	0.0034	0.8781	T
Retinol	rs56314503	12:84465022	T	G	8809	-0.00411494	0.00411494	0.0175582	-0.0203	0.0025	0.7487	T
Retinol	rs3213572	12:121205078	G	A	8809	0.00563422	0.00563422	0.0146447	0.0196	0.0021	0.4745	A
Retinol	rs1343605	13:53647048	A	C	8809	-0.0140124	0.0140124	0.0149194	0.0231	0.0022	0.384	A
Retinol	rs9592461	13:66941792	A	G	8809	-0.02439	0.02439	0.01466	0.0243	0.0022	0.4874	A
Retinol	rs4772087	13:99115041	C	T	8809	0.0262063	0.0262063	0.0152065	0.0209	0.0023	0.3732	T
Retinol	rs61990288	14:42074726	A	G	8809	0.00209322	-0.00209322	0.014623	-0.0249	0.0021	0.5083	A
Retinol	rs1956373	14:60141822	T	G	8809	0.0174152	-0.0174152	0.0167576	-0.0169	0.0025	0.7436	T
Retinol	rs1152578	14:64697037	C	T	8809	-0.0172267	-0.0172267	0.0148142	-0.0152	0.0022	0.4357	T
Retinol	rs1045430	14:75130235	G	T	8809	-0.00319843	-0.00319843	0.0146207	-0.0226	0.0022	0.4792	T
Retinol	rs10149470	14:104017953	G	A	8809	0.0100284	0.0100284	0.0146302	-0.0206	0.0021	0.4869	A
Retinol	rs8037355	15:37643831	C	T	8809	0.0103902	0.0103902	0.0147315	-0.0192	0.0022	0.5556	T
Retinol	rs34488670	15:47684936	T	C	8809	-0.0109303	0.0109303	0.0184125	-0.0193	0.0027	0.7887	T
Retinol	rs7193263	16:6315880	G	A	8809	0.00740984	0.00740984	0.0154897	-0.0206	0.0023	0.6679	A
Retinol	rs7198928	16:7666402	T	C	8809	0.0406553	-0.0406553	0.0151621	0.0219	0.0022	0.6159	T
Retinol	rs7200826	16:13066833	C	T	8809	-0.00888801	-0.00888801	0.0166792	0.027	0.0025	0.2551	T
Retinol	rs56887639	16:13755530	A	G	8809	-0.00295155	0.00295155	0.0166518	-0.0171	0.0024	0.7264	A
Retinol	rs12923444	16:21639710	A	C	8809	-0.0168565	0.0168565	0.0151019	-0.0237	0.0023	0.5625	A
Retinol	rs75581564	17:27363750	G	A	8809	-0.0260138	-0.0260138	0.0226396	0.0248	0.0034	0.1165	A
Retinol	rs12967855	18:35138245	A	G	8809	0.0138829	-0.0138829	0.0156786	0.0207	0.0023	0.3295	A
Retinol	rs7227069	18:50731802	G	A	8809	0.0489618	0.0489618	0.014797	0.0242	0.0022	0.4326	A
Retinol	rs62091461	18:52488672	C	T	8809	-0.0142771	-0.0142771	0.017524	-0.0199	0.0026	0.2274	T
Retinol	rs12967143	18:53099012	G	C	8809	0.0323309	0.0323309	0.0157477	-0.0256	0.0024	0.6984	C
Retinol	rs7241572	18:77580712	G	A	8809	-0.00946219	-0.00946219	0.0181881	0.02	0.0027	0.201	A
Retinol	rs12624433	20:44680853	G	A	8809	0.0137145	0.0137145	0.0166975	0.0191	0.0025	0.2584	A
Retinol	rs5995992	22:41487218	T	C	8809	0.00950149	-0.00950149	0.01599	-0.0301	0.0024	0.7155	T

Supplementary Table 3: List of SNPs used as instruments for metabolites in the MR analysis (p-value < 10-6).

SNP	Chr	Pos	REF	ALT	N	Beta	SE	P	Metabolite	A1	FREQ	BETA_MDD	SE_MDD	PVAL	allele_match	betaMDD_fli	freq_alt
rs10155039	3	103759892	C	A	8809	-0.1134	0.0217	1.83E-07	hippurate	A	0.1345	0.0054	0.0063	0.3933	1	0.0054	0.1345
rs1934328	6	45465753	A	T	8809	-0.0765	0.0148	2.42E-07	hippurate	A	0.5094	0.0035	0.0043	0.4213	2	-0.0035	0.4906
rs799449	7	44784697	C	T	8809	-0.0790	0.0149	1.12E-07	hippurate	T	0.555	-0.0096	0.0044	0.02754	1	-0.0096	0.555
rs78713202	12	126902741	T	A	8809	-0.2729	0.0536	3.70E-07	hippurate	A	0.0182	0.0143	0.0165	0.3887	1	0.0143	0.0182
rs11211403	1	47392855	G	T	8802	0.4247	0.0219	2.85E-82	10-undecenoate	T	0.8693	-0.0038	0.0064	0.5566	1	-0.0038	0.8693
rs61906491	11	117102200	G	A	8802	0.1130	0.0226	5.58E-07	10-undecenoate	A	0.1338	0.0087	0.0066	0.1899	1	0.0087	0.1338
rs4605140	15	101521308	A	T	8802	0.0927	0.0185	5.82E-07	10-undecenoate	A	0.7927	-0.0009	0.0053	0.8676	2	0.0009	0.2073
rs76486095	16	54899439	C	T	8802	-0.2471	0.0468	1.31E-07	10-undecenoate	T	0.0256	-0.0014	0.0143	0.9225	1	-0.0014	0.0256
rs6705977	2	73849170	C	G	8368	-0.4673	0.0180	3.00E-143	2-aminooctanoate	C	0.7723	-0.0029	0.0051	0.5718	2	0.0029	0.2277
rs74637339	3	15628040	T	A	8368	0.2512	0.0341	1.80E-13	2-aminooctanoate	A	0.0543	0.0009	0.0095	0.924	1	0.0009	0.0543
rs13107325	4	103188709	C	T	8368	-0.1519	0.0286	1.08E-07	2-aminooctanoate	T	0.0726	0.0242	0.0084	0.003878	1	0.0242	0.0726
rs9374863	6	120424885	T	C	8368	0.0921	0.0188	9.80E-07	2-aminooctanoate	T	0.7797	-0.0105	0.0052	0.04371	2	0.0105	0.2203
rs146720224	11	20119222	G	A	8368	0.6825	0.1355	4.80E-07	2-aminooctanoate	A	0.0077	0.0222	0.0267	0.405	1	0.0222	0.0077
rs2320604	13	22524159	G	A	8368	0.1184	0.0238	6.53E-07	2-aminooctanoate	A	0.8814	0.003	0.0066	0.6499	1	0.003	0.8814
rs219372	14	60475838	C	T	8368	0.1153	0.0234	8.08E-07	2-aminooctanoate	T	0.1236	-0.0064	0.0065	0.3302	1	-0.0064	0.1236
rs8014023	14	73972289	A	G	8368	0.0752	0.0153	8.31E-07	2-aminooctanoate	A	0.5335	-0.0008	0.0043	0.8541	2	0.0008	0.4665
rs117816977	16	85759946	G	A	8368	-0.3319	0.0666	6.30E-07	2-aminooctanoate	A	0.0146	0.0058	0.0187	0.7547	1	0.0058	0.0146
rs780094	2	27741237	T	C	8809	0.0859	0.0151	1.39E-08	1-palmitoyl-2-palmitoleoyl-GPC	T	0.3851	0.0037	0.0044	0.3985	2	-0.0037	0.6149
rs10865864	3	3628981	A	G	8809	-0.1059	0.0215	8.61E-07	1-palmitoyl-2-palmitoleoyl-GPC	A	0.7947	0.004	0.0054	0.4503	2	-0.004	0.2053
rs60296378	4	56946072	G	A	8809	0.0879	0.0178	7.81E-07	1-palmitoyl-2-palmitoleoyl-GPC	A	0.2232	0.0067	0.0052	0.1933	1	0.0067	0.2232
rs603424	10	102075479	G	A	8809	-0.1200	0.0193	5.01E-10	1-palmitoyl-2-palmitoleoyl-GPC	A	0.1727	0.0131	0.0057	0.02048	1	0.0131	0.1727
rs17777943	10	103746504	G	A	8809	0.1271	0.0254	5.67E-07	1-palmitoyl-2-palmitoleoyl-GPC	A	0.094	-0.0078	0.0076	0.2995	1	-0.0078	0.094
rs174541	11	61565908	T	C	8809	0.0901	0.0156	7.98E-09	1-palmitoyl-2-palmitoleoyl-GPC	T	0.6404	-0.0139	0.0045	0.001971	2	0.0139	0.3596
rs117352320	15	58709264	T	C	8809	0.2445	0.0495	8.11E-07	1-palmitoyl-2-palmitoleoyl-GPC	T	0.9768	0.0173	0.0149	0.2455	2	-0.0173	0.0232
rs113090602	3	1782444	G	A	8809	0.1437	0.0291	8.35E-07	retinol	A	0.074	0.0023	0.0084	0.7862	1	0.0023	0.074
rs1291921	3	136036226	A	G	8809	0.0893	0.0165	6.32E-08	retinol	A	0.2718	0.0008	0.0049	0.8637	2	-0.0008	0.7282
rs10761741	10	65066186	G	T	8809	-0.0904	0.0149	1.30E-09	retinol	T	0.4205	-0.0064	0.0044	0.1445	1	-0.0064	0.4205
rs11187547	10	95359865	A	G	8809	-0.1324	0.0152	3.80E-18	retinol	A	0.6238	-0.0045	0.0046	0.3264	2	0.0045	0.3762
rs1667229	18	29142206	T	C	8809	-0.0879	0.0148	3.14E-09	retinol	T	0.4582	-0.0012	0.0043	0.7766	2	0.0012	0.5418

Supplementary Table 4: Results of the association analyses of all metabolites with depression. This Table includes results for model 1, 2, and model 3.
The order of the direction column: RS, SHIP-trend, KORA, EPIC-Norfolk B2, EPIC-Norfolk B3, NEO. Significant results are highlighted (FDR corrected p-value < 0.05).

					Model 1					Model 2					Model 3				
Alternative name	novelty	Metabolite	Name	Super_pathway	N	Zscore	P.value	Direction*	FDR	N	Zscore	P.value	Direction*	FDR	N	Zscore	P.value	Direction*	FDR
Undecylenic acid	novel	100001197	10-undecenoate (11:1n1)	Lipid	13596	-5.12	3.03E-07	+-----	8.02E-05	13556	-3.94	8.19E-05	+-----	1.7E-02	13549	-3.79	1.53E-04	+-----	2.71E-02
	not	100001740	mannitol/sorbitol	Carbohydrate	12631	5.14	2.80E-07	+?-+++	8.02E-05	12592	3.39	7.11E-04	+?-+++	5.02E-02	12586	3.58	3.45E-04	+?-+++	4.47E-02
	novel	1090	bilirubin (Z,Z)	Cofactors and Vitamins	13596	-5.33	9.91E-08	------	8.02E-05	13556	-3.60	3.20E-04	------	3.5E-02	13549	-2.84	4.49E-03	------	1.83E-01
	novel	100001950	bilirubin (E,E)*	Cofactors and Vitamins	13596	-5.25	1.54E-07	------	8.02E-05	13556	-3.33	8.71E-04	------	5.13E-02	13549	-2.73	6.26E-03	------	2.08E-01
	novel	100002049	4-hydroxycoumarin	Xenobiotics	10885	-4.99	6.14E-07	-??---	1.30E-04	10847	-4.48	7.51E-06	-??---	4.0E-03	10847	-4.17	3.03E-05	-??---	1.12E-02
lecithin/PC(16:0/16:1)	novel	100008984	1-palmitoyl-2-palmitoleoyl-GPC (16:0/16:1)*	Lipid	10885	4.84	1.28E-06	+??+++	1.94E-04	10847	3.58	3.49E-04	+??+++	3.5E-02	10847	3.51	4.47E-04	+??+++	4.47E-02
	novel	100001951	bilirubin (E,Z or Z,E)*	Cofactors and Vitamins	12631	-4.74	2.13E-06	-?----	2.83E-04	12592	-3.50	4.65E-04	-?----	3.5E-02	12586	-2.98	2.84E-03	-?----	1.37E-01
	novel	498	retinol (Vitamin A)	Cofactors and Vitamins	10885	4.65	3.37E-06	+??+++	3.58E-04	10847	3.89	9.88E-05	+??+++	1.7E-02	10847	4.14	3.52E-05	+??+++	1.12E-02
	novel	100004227	2-aminooctanoate	Lipid	11850	-4.65	3.33E-06	--?---	3.58E-04	11811	-4.00	6.30E-05	--?---	1.7E-02	11810	-3.92	8.82E-05	--?---	1.87E-02
	novel	100002253	cinnamoylglycine	Xenobiotics	10885	-4.40	1.06E-05	-??---	9.38E-04	10847	-3.50	4.58E-04	-??---	3.5E-02	10847	-3.32	8.86E-04	-??---	6.71E-02
	novel	100000010	3-phenylpropionate (hydrocinnamate)	Xenobiotics	13596	-4.42	9.81E-06	------	9.38E-04	13556	-3.73	1.91E-04	------	2.9E-02	13549	-3.24	1.19E-03	--+---	7.89E-02
	novel	100001251	decanoylcarnitine (C10)	Lipid	13596	-4.35	1.34E-05	+-----	1.02E-03	13556	-3.35	8.04E-04	+-----	5.13E-02	13549	-3.15	1.61E-03	+-----	8.53E-02
PE(16:0/18:1)	novel	1526	1-palmitoyl-2-oleoyl-GPE (16:0/18:1)	Lipid	10885	4.36	1.30E-05	+??+++	1.02E-03	10847	2.64	8.41E-03	-??+++	2.12E-01	10847	2.44	1.47E-02	-??+++	2.68E-01
	novel	250	biliverdin	Cofactors and Vitamins	13596	-4.29	1.75E-05	------	1.24E-03	13556	-2.88	3.95E-03	------	1.40E-01	13549	-2.41	1.61E-02	------	2.75E-01
	not	504	serotonin	Amino acid	12631	-4.24	2.25E-05	-?----	1.49E-03	12592	0.18	8.54E-01	-?-+++	9.55E-01	12586	-0.02	9.87E-01	-?-+++	9.92E-01
	novel	192	N-acetylputrescine	Amino acid	10885	4.06	4.83E-05	-??+++	3.01E-03	10847	2.09	3.68E-02	-??-++	3.52E-01	10847	2.16	3.05E-02	-??+++	3.44E-01
	novel	100002259	cis-4-decenoylcarnitine (C10:1)	Lipid	13596	-4.02	5.87E-05	------	3.27E-03	13556	-2.80	5.13E-03	------	1.58E-01	13549	-2.47	1.35E-02	------	2.68E-01
	novel	212	5-methylthioadenosine (MTA)	Amino acid	10885	4.03	5.67E-05	+??+++	3.27E-03	10847	2.09	3.64E-02	+??+++	3.52E-01	10847	1.78	7.52E-02	+??+++	4.91E-01
	novel	100000997	3-hydroxydecanoate	Lipid	11850	-3.97	7.34E-05	+-?---	3.89E-03	11811	-2.60	9.45E-03	+-?---	2.23E-01	11810	-2.39	1.68E-02	+-?---	2.75E-01
	novel	1539	1-palmitoyl-2-oleoyl-GPC (16:0/18:1)	Lipid	10885	3.85	1.19E-04	+??++-	6.01E-03	10847	2.93	3.39E-03	+??++-	1.36E-01	10847	2.76	5.75E-03	+??++-	2.03E-01
	not	100000014	hippurate	Xenobiotics	13596	-3.80	1.47E-04	------	7.10E-03	13556	-4.17	3.02E-05	------	1.1E-02	13549	-3.72	1.98E-04	------	2.99E-02
	novel	100001392	laurylcarnitine (C12)	Lipid	13596	-3.73	1.95E-04	+-----	8.97E-03	13556	-2.68	7.41E-03	+-----	1.96E-01	13549	-2.61	8.95E-03	+-----	2.48E-01
	novel	1128	2-aminobutyrate	Amino acid	13596	-3.68	2.36E-04	------	9.61E-03	13556	-2.08	3.76E-02	---+--	3.54E-01	13549	-1.84	6.62E-02	---+--	4.74E-01
	not	561	glutamate	Amino acid	13596	3.70	2.19E-04	++++++	9.61E-03	13556	1.92	5.46E-02	++++-+	4.13E-01	13549	1.15	2.49E-01	++++-+	7.26E-01
	not	98	kynurenate	Amino acid	10885	-3.61	3.12E-04	-??---	1.22E-02	10847	-2.79	5.35E-03	-??---	1.58E-01	10847	-2.50	1.24E-02	-??---	2.68E-01
	novel	100001658	taurolithocholate 3-sulfate	Lipid	13596	-3.52	4.37E-04	------	1.45E-02	13556	-3.09	2.02E-03	------	9.31E-02	13549	-3.09	2.00E-03	---+--	1.01E-01
	novel	100001511	1-palmitoleoyl-GPC (16:1)*	Lipid	13596	3.54	4.03E-04	+++++-	1.45E-02	13556	2.43	1.53E-02	+++++-	2.74E-01	13549	2.57	1.01E-02	++++++	2.48E-01
	novel	100001112	3-hydroxylaurate	Lipid	10885	-3.53	4.16E-04	-??---	1.45E-02	10847	-2.54	1.10E-02	-??---	2.42E-01	10847	-2.43	1.50E-02	-??---	2.69E-01
	novel	100008990	1-palmitoyl-2-arachidonoyl-GPE (16:0/20:4)*	Lipid	10885	3.52	4.36E-04	-??+++	1.45E-02	10847	2.22	2.65E-02	-??+++	3.00E-01	10847	2.23	2.60E-02	-??+++	3.27E-01
	novel	100000773	3-hydroxyoctanoate	Lipid	11850	-3.54	3.96E-04	+-?---	1.45E-02	11811	-2.25	2.47E-02	+-?---	3.00E-01	11810	-2.13	3.32E-02	+-?---	3.63E-01
	novel	100001868	4-allylphenol sulfate	Xenobiotics	10885	-3.48	5.09E-04	-??---	1.58E-02	10847	-2.86	4.27E-03	-??-+-	1.46E-01	10847	-2.46	1.39E-02	-??-+-	2.68E-01
	novel	100001247	octanoylcarnitine (C8)	Lipid	13596	-3.47	5.21E-04	+-----	1.58E-02	13556	-2.62	8.69E-03	+-----	2.14E-01	13549	-2.48	1.33E-02	+-----	2.68E-01
	novel	100001083	indolepropionate	Amino acid	13596	-3.48	4.99E-04	------	1.58E-02	13556	-2.95	3.18E-03	------	1.35E-01	13549	-2.12	3.42E-02	------	3.66E-01
	novel	100001977	beta-cryptoxanthin	Xenobiotics	6246	-3.46	5.44E-04	-???--	1.60E-02	6208	-3.25	1.14E-03	-???--	5.74E-02	6208	-2.59	9.68E-03	-???--	2.48E-01
	not	100006430	arabitol/xylitol	Carbohydrate	12631	3.44	5.77E-04	+?++++	1.65E-02	12592	2.48	1.30E-02	+?++++	2.51E-01	12586	2.69	7.08E-03	+?++++	2.15E-01
	novel	100009082	1-linoleoyl-GPA (18:2)*	Lipid	10885	-3.39	6.89E-04	-??---	1.91E-02	10847	-3.57	3.63E-04	-??---	3.5E-02	10847	-3.53	4.19E-04	-??---	4.47E-02
PE(16:0/18:2)	novel	100001870	1-palmitoyl-2-linoleoyl-GPE (16:0/18:2)	Lipid	10885	3.39	7.03E-04	+??+++	1.91E-02	10847	2.02	4.32E-02	+??+++	3.78E-01	10847	2.18	2.93E-02	+??+++	3.41E-01
	novel	100000257	glucuronate	Carbohydrate	10885	3.35	8.24E-04	+??+++	2.18E-02	10847	2.09	3.65E-02	+??+++	3.52E-01	10847	1.93	5.34E-02	+??+++	4.51E-01
	not	391	citrulline	Amino acid	13596	-3.31	9.47E-04	------	2.45E-02	13556	-3.53	4.20E-04	------	3.5E-02	13549	-3.29	1.02E-03	------	7.22E-02
	novel	100001121	pyridoxate	Cofactors and Vitamins	13596	3.29	9.89E-04	++-++-	2.50E-02	13556	2.71	6.73E-03	++-++-	1.83E-01	13549	3.17	1.53E-03	++-++-	8.53E-02
	novel	100002021	5alpha-androstan-3beta,17alpha-diol disulfate	Lipid	10885	-3.25	1.14E-03	-??---	2.79E-02	10847	-2.05	4.00E-02	-??---	3.67E-01	10847	-1.63	1.03E-01	-??---	5.35E-01
	not	100001287	epiandrosterone sulfate	Lipid	13596	-3.16	1.56E-03	-+----	3.59E-02	13556	-1.71	8.68E-02	-+----	4.70E-01	13549	-1.73	8.35E-02	-+----	5.03E-01
PC(16:0/20:4)	novel	100008914	1-palmitoyl-2-arachidonoyl-GPC (16:0/20:4n6)	Lipid	10885	3.13	1.75E-03	-??+++	3.89E-02	10847	2.92	3.47E-03	-??+++	1.36E-01	10847	2.94	3.29E-03	-??++-	1.52E-01
	not	100001320	erythronate*	Carbohydrate	12631	3.12	1.80E-03	+?++++	3.89E-02	12592	1.76	7.85E-02	+?++++	4.55E-01	12586	1.77	7.70E-02	+?++++	4.91E-01
	not	935	sucrose	Carbohydrate	10885	3.13	1.77E-03	+??+++	3.89E-02	10847	1.61	1.08E-01	+??+-+	4.99E-01	10847	1.16	2.45E-01	+??+-+	7.21E-01
LysoPE(16:0)	novel	100001567	1-palmitoyl-GPE (16:0)	Lipid	13596	3.10	1.93E-03	++-++-	4.01E-02	13556	2.15	3.12E-02	++-++-	3.28E-01	13549	2.57	1.03E-02	++++++	2.48E-01
PE(38:6)	novel	100008991	1-palmitoyl-2-docosahexaenoyl-GPE (16.0/22.6)	Lipid	10401	3.06	2.21E-03	???+++	4.26E-02	10401	2.43	1.51E-02	???+++	2.74E-01	10401	2.71	6.83E-03	???+++	2.15E-01
	novel	100001657	glycolithocholate sulfate*	Lipid	11850	-3.06	2.20E-03	--?--+	4.26E-02	11811	-2.46	1.37E-02	--?---	2.60E-01	11810	-2.29	2.22E-02	--?---	3.09E-01
PE(18:0/20:4)	novel	100008977	1-stearoyl-2-arachidonoyl-GPE (18:0/20:4)	Lipid	10885	3.07	2.12E-03	-??+++	4.26E-02	10847	1.94	5.19E-02	-??+++	4.07E-01	10847	1.93	5.40E-02	-??+++	4.51E-01
	not	823	pyruvate	Carbohydrate	13596	3.06	2.20E-03	++++-+	4.26E-02	13556	2.28	2.28E-02	++++-+	2.91E-01	13549	1.84	6.56E-02	+-++-+	4.74E-01
	not	397	leucine	Amino acid	13596	-3.04	2.39E-03	--+--+	4.44E-02	13556	-2.73	6.30E-03	--+--+	1.76E-01	13549	-3.33	8.74E-04	------	6.71E-02
	novel	100002945	15-methylpalmitate	Lipid	13596	-3.04	2.36E-03	+-----	4.44E-02	13556	-2.77	5.68E-03	+-----	1.63E-01	13549	-2.40	1.64E-02	+-----	2.75E-01
PC(16:0/18:1)	novel	100009066	1-palmitoyl-2-oleoyl-GPI (16:0/18:1)*	Lipid	6246	3.03	2.46E-03	+???++	4.50E-02	6208	1.96	5.02E-02	+???+-	4.01E-01	6208	1.86	6.23E-02	+???+-	4.66E-01
		100006367	3-hydroxyhexanoate	Lipid	10885	-2.971	2.97E-03	+??---	0.05	10847	-2.015	0.04	+??---	0.38	10847	-1.94	0.05	+??---	0.45

		1162	N-acetylneuraminate	Carbohydrate	10885	2.95	3.17E-03	+??+++	0.05	10847	1.731	0.08	+??+++	0.47	10847	1.227	0.22	+??+-+	0.69
		100003179	leucylalanine	Peptide	2829	-2.939	3.29E-03	-?-??-	0.05	2790	-2.883	3.94E-03	-?-??-	0.14	2784	-2.234	0.03	-?-??-	0.33
		100001073	androsterone sulfate	Lipid	13596	-2.937	3.32E-03	------	0.05	13556	-1.482	0.14	------	0.55	13549	-1.441	0.15	-+----	0.62
		100001510	phenol sulfate	Amino acid	13596	2.932	3.37E-03	-+++++	0.05	13556	3.261	1.11E-03	-+++++	0.06	13549	3.381	7.23E-04	-+++++	0.06
		100001437	cysteine-glutathione disulfide	Amino acid	12631	-2.925	3.45E-03	-?----	0.06	12592	-1.638	0.10	-?-+--	0.49	12586	-1.256	0.21	-?-+--	0.68
		100000781	hexanoylcarnitine (C6)	Lipid	13596	-2.914	3.57E-03	+-----	0.06	13556	-2.13	0.03	---+--	0.34	13549	-2.337	0.02	------	0.29
		1111	vanillylmandelate (VMA)	Amino acid	10885	-2.843	4.47E-03	+??---	0.07	10847	-0.552	0.58	+??--+	0.86	10847	-0.125	0.90	+??--+	0.97
		100008992	1-stearoyl-2-docosahexaenoyl-GPE (18.0/22.6)	Lipid	10401	2.835	4.58E-03	???+++	0.07	10401	2.213	0.03	???+++	0.30	10401	2.395	0.02	???+++	0.27
		1140	gamma-glutamylglutamine	Peptide	12631	-2.811	4.94E-03	-?-+--	0.07	12592	-1.043	0.30	-?-+--	0.71	12586	-0.635	0.53	-?-+-+	0.87
		100000265	kynurenine	Amino acid	13596	-2.775	5.52E-03	------	0.08	13556	-3.039	2.37E-03	------	0.10	13549	-3.218	1.29E-03	------	0.08
		1024	pantothenate	Cofactors and Vitamins	13596	2.768	5.64E-03	++-+++	0.08	13556	2.009	0.04	++-+++	0.38	13549	2.15	0.03	++-+++	0.35
		572	glucose	Carbohydrate	12631	2.763	5.73E-03	+?++++	0.08	12592	1.735	0.08	+?++++	0.47	12586	1.671	0.09	+?++++	0.52
		1242	N1-methyladenosine	Nucleotide	13596	2.767	5.66E-03	--++++	0.08	13556	0.923	0.36	--++++	0.77	13549	0.743	0.46	---++-	0.85
		566	valine	Amino acid	13596	-2.738	6.18E-03	--+--+	0.08	13556	-2.456	0.01	--+--+	0.26	13549	-2.901	3.72E-03	--+---	0.16
		100002458	3-methylglutaconate	Amino acid	10885	2.734	6.25E-03	+??+++	0.08	10847	2.794	5.21E-03	+??+++	0.16	10847	2.789	5.28E-03	+??+++	0.20
		100001550	homostachydrine*	Xenobiotics	13596	-2.729	6.35E-03	+-----	0.08	13556	-2.189	0.03	+-----	0.31	13549	-2.313	0.02	+-----	0.30
		93	alpha-ketoglutarate	Energy	12631	2.737	6.20E-03	+?++++	0.08	12592	2.237	0.03	+?++-+	0.30	12586	2.071	0.04	+?++-+	0.39
		111	3-hydroxyisobutyrate	Amino acid	11850	-2.748	5.99E-03	+-?---	0.08	11811	-2.129	0.03	+-?---	0.34	11810	-1.913	0.06	+-?---	0.46
		100001502	gamma-glutamyl-2-aminobutyrate	Peptide	10401	-2.732	6.30E-03	???---	0.08	10401	-0.61	0.54	???+--	0.85	10401	-0.643	0.52	???+--	0.87
		189	N6,N6,N6-trimethyllysine	Amino acid	10885	-2.718	6.57E-03	-??---	0.08	10847	-2.555	0.01	-??---	0.24	10847	-2.666	7.68E-03	-??---	0.23
		466	phytanate	Xenobiotics	10885	-2.705	6.83E-03	+??---	0.08	10847	-1.932	0.05	+??---	0.41	10847	-1.863	0.06	+??---	0.47
		100001609	7-alpha-hydroxy-3-oxo-4-cholestenoate (7-Hoca)	Lipid	13596	2.721	6.51E-03	+++-++	0.08	13556	2.102	0.04	+++--+	0.35	13549	1.668	0.10	+++--+	0.52
		878	fructose	Carbohydrate	12631	2.708	6.78E-03	+?-+++	0.08	12592	1.768	0.08	+?-+++	0.45	12586	1.642	0.10	+?-+++	0.53
		100001662	deoxycarnitine	Lipid	11850	-2.708	6.78E-03	--?---	0.08	11811	-1.425	0.15	-+?---	0.56	11810	-1.424	0.15	-+?---	0.63
		100001055	isobutyrylcarnitine (C4)	Amino acid	13596	-2.686	7.24E-03	------	0.09	13556	-2.824	4.74E-03	------	0.16	13549	-2.473	0.01	------	0.27
		100006051	myristoleoylcarnitine (C14:1)*	Lipid	12631	-2.689	7.17E-03	+?----	0.09	12592	-1.543	0.12	+?----	0.53	12586	-1.563	0.12	+?----	0.57
		100005372	1-(1-enyl-oleoyl)-GPE (P-18:1)*	Lipid	11850	-2.676	7.45E-03	-+?---	0.09	11811	-2.881	3.96E-03	-+?---	0.14	11810	-2.597	9.40E-03	-+?---	0.25
		100001145	3-hydroxysebacate	Lipid	5722	-2.643	8.22E-03	+??-?-	0.09	5684	-1.915	0.06	+??-?-	0.41	5684	-1.936	0.05	+??-?-	0.45
		100015834	lignoceroylcarnitine (C24)*	Lipid	6246	-2.632	8.48E-03	-???--	0.10	6208	-2.322	0.02	-???--	0.28	6208	-2.271	0.02	-???--	0.31
		100010935	diacylglycerol (14:0/18:1, 16:0/16:1) [2]*	Lipid	1083	2.625	8.67E-03	+????+	0.10	1045	1.821	0.07	+????+	0.44	1045	1.517	0.13	+????+	0.58
		100001178	3-carboxy-4-methyl-5-propyl-2-furanpropanoate (CMPF)	Lipid	13596	-2.616	8.90E-03	+-----	0.10	13556	-1.725	0.08	+-----	0.47	13549	-1.53	0.13	+-----	0.58
		100000743	2-hydroxyoctanoate	Lipid	10885	-2.606	9.16E-03	-??-+-	0.10	10847	-2.483	0.01	-??-+-	0.25	10847	-2.503	0.01	-??-+-	0.27
		100001597	tiglylcarnitine (C5:1-DC)	Amino acid	13596	-2.599	9.36E-03	--+---	0.10	13556	-1.652	0.10	--+---	0.48	13549	-1.316	0.19	--+-+-	0.68
		100002008	5alpha-androstan-3alpha,17alpha-diol monosulfate	Lipid	1083	-2.579	9.90E-03	-????-	0.10	1045	-2.175	0.03	-????-	0.32	1045	-2.211	0.03	-????-	0.33
		100005717	1-palmitoyl-GPG (16:0)*	Lipid	10885	2.582	9.82E-03	+??+++	0.10	10847	1.633	0.10	+??+++	0.49	10847	1.694	0.09	+??+++	0.52
		100002014	5alpha-pregnan-3beta,20alpha-diol monosulfate (2)	Lipid	10885	-2.559	0.01	+??---	0.11	10847	-1.293	0.20	+??---	0.62	10847	-1.338	0.18	+??---	0.67
		444	ornithine	Amino acid	12631	-2.551	0.01	+?----	0.11	12592	-1.897	0.06	+?-+--	0.42	12586	-2.086	0.04	+?----	0.38
		100008952	1-palmitoleoylglycerol (16:1)*	Lipid	10885	2.536	0.01	+??+++	0.11	10847	1.805	0.07	+??-++	0.44	10847	1.737	0.08	+??-++	0.50
		100002094	gamma-CEHC	Cofactors and Vitamins	10885	-2.532	0.01	-??--+	0.11	10847	-1.913	0.06	-??--+	0.41	10847	-1.622	0.10	-??--+	0.54
		100010959	diacylglycerol (12:0/18:1, 14:0/16:1, 16:0/14:1) [2]*	Lipid	1083	2.523	0.01	+????+	0.11	1045	1.705	0.09	+????+	0.47	1045	1.604	0.11	+????+	0.55
		100001393	isovalerylcarnitine (C5)	Amino acid	13596	-2.512	0.01	---+--	0.11	13556	-2.045	0.04	---+--	0.37	13549	-2.343	0.02	---+--	0.29
		100000706	alpha-hydroxyisocaproate	Amino acid	11850	-2.514	0.01	--?+--	0.11	11811	-1.854	0.06	+-?+--	0.43	11810	-1.699	0.09	+-?+--	0.52
		100002488	isoursodeoxycholate	Lipid	6246	2.498	0.01	+???++	0.12	6208	2.537	0.01	+???++	0.24	6208	2.58	9.89E-03	+???++	0.25
		100009220	1-oleoyl-2-docosahexaenoyl-GPE (18:1/22:6)*	Lipid	5762	2.483	0.01	????++	0.12	5762	2.146	0.03	????++	0.33	5762	2.282	0.02	????++	0.31
		100001313	gamma-glutamylmethionine	Peptide	13596	-2.446	0.01	+--+--	0.13	13556	-1.08	0.28	+--+-+	0.70	13549	-1.085	0.28	+--+--	0.75
		880	adenine	Nucleotide	10885	2.447	0.01	-??+++	0.13	10847	0.64	0.52	-??+++	0.84	10847	0.855	0.39	-??+++	0.83
		100009009	1-(1-enyl-palmitoyl)-2-linoleoyl-GPC (P-16:0/18:2)*	Lipid	10885	-2.445	0.01	-??---	0.13	10847	-1.173	0.24	-??+--	0.66	10847	-0.757	0.45	-??+--	0.85
		1022	picolinate	Amino acid	6246	-2.431	0.02	+???--	0.13	6208	-2.355	0.02	+???--	0.28	6208	-2.267	0.02	+???--	0.31
		533	urea	Amino acid	13596	-2.433	0.01	+-----	0.13	13556	-2.595	9.45E-03	+-----	0.22	13549	-2.172	0.03	+-----	0.34
		100010901	gamma-glutamyl-alpha-lysine	Peptide	6246	-2.425	0.02	+???--	0.13	6208	-1.959	0.05	+???--	0.40	6208	-1.981	0.05	+???--	0.43
		100001561	2-palmitoleoyl-GPC (16:1)*	Lipid	6687	2.422	0.02	++?+?+	0.13	6648	1.83	0.07	++?+?+	0.44	6647	1.758	0.08	++?+?+	0.50
		100000870	saccharin	Xenobiotics	10401	2.412	0.02	???+++	0.14	10401	1.532	0.13	???+++	0.53	10401	1.599	0.11	???+++	0.55
		100001211	sebacate (decanedioate)	Lipid	10885	-2.406	0.02	-??--+	0.14	10847	-2.043	0.04	-??---	0.37	10847	-2.085	0.04	-??---	0.38
		100003210	valylleucine	Peptide	1083	-2.399	0.02	-????-	0.14	1045	-2.156	0.03	-????-	0.33	1045	-1.619	0.11	-????-	0.54
		100001212	guanidinosuccinate	Amino acid	1083	-2.399	0.02	-????-	0.14	1045	-1.844	0.07	-????-	0.43	1045	-1.252	0.21	-????-	0.68
		100009264	glycochenodeoxycholate glucuronide (1)	Lipid	10885	2.386	0.02	+??+++	0.14	10847	1.976	0.05	+??+++	0.40	10847	1.817	0.07	+??+++	0.49
		100005384	O-sulfo-L-tyrosine	Xenobiotics	13596	-2.374	0.02	++----	0.15	13556	-1.231	0.22	++----	0.64	13549	-0.976	0.33	++----	0.79
		244	beta-alanine	Nucleotide	10401	-2.365	0.02	???---	0.15	10401	-1.796	0.07	???---	0.44	10401	-1.772	0.08	???---	0.49
		100001571	1-arachidonoyl-GPE (20:4n6)*	Lipid	13596	2.355	0.02	++++++	0.15	13556	1.836	0.07	++++++	0.44	13549	1.871	0.06	++++++	0.47

		100003151	linoleoylcarnitine (C18:2)*	Lipid	10885	-2.356	0.02	-??---	0.15	10847	-1.229	0.22	-??---	0.64	10847	-0.763	0.45	-??+--	0.85
		100009139	1-myristoyl-2-arachidonoyl-GPC (14:0/20:4)*	Lipid	10401	2.329	0.02	???+++	0.16	10401	1.571	0.12	???++-	0.52	10401	1.83	0.07	???+++	0.48
		100015759	stearoylcholine*	Lipid	6246	-2.312	0.02	-???--	0.16	6208	-2.505	0.01	-???--	0.25	6208	-2.453	0.01	-???--	0.27
		55	1-methylnicotinamide	Cofactors and Vitamins	10885	2.304	0.02	-??++-	0.16	10847	1.782	0.07	-??++-	0.45	10847	2.003	0.05	-??++-	0.41
		100003434	imidazole propionate	Amino acid	10885	2.302	0.02	+??+++	0.16	10847	1.644	0.10	+??+++	0.48	10847	1.558	0.12	+??+++	0.57
		100015967	carotene diol (2)	Xenobiotics	6246	-2.301	0.02	+???--	0.16	6208	-1.161	0.25	+???--	0.66	6208	-0.593	0.55	+???--	0.87
		100004318	indolin-2-one	Xenobiotics	5722	-2.297	0.02	+??-?-	0.16	5684	-2.554	0.01	+??-?-	0.24	5684	-2.356	0.02	+??-?-	0.29
		100000987	2-linoleoylglycerol (18:2)	Lipid	10885	-2.269	0.02	-??---	0.17	10847	-1.887	0.06	-??---	0.42	10847	-1.626	0.10	-??---	0.54
		100002871	1-adrenoyl-GPC (22:4)*	Lipid	9802	2.269	0.02	???++?	0.17	9802	1.368	0.17	???++?	0.59	9802	1.29	0.20	???++?	0.68
		1268	gamma-glutamylleucine	Peptide	13596	-2.255	0.02	+-----	0.18	13556	-2.119	0.03	+-----	0.34	13549	-2.544	0.01	+-----	0.26
		1113	4-acetamidobutanoate	Amino acid	13596	2.242	0.02	++++++	0.18	13556	1.863	0.06	+++-+-	0.43	13549	2.024	0.04	+++++-	0.41
		100006726	linoleoyl ethanolamide	Lipid	10885	-2.242	0.02	-??--+	0.18	10847	-1.353	0.18	-??--+	0.59	10847	-1.265	0.21	-??--+	0.68
		1124	citrate	Energy	13596	-2.23	0.03	+-----	0.18	13556	-1.824	0.07	+-----	0.44	13549	-1.537	0.12	++----	0.57
		100015851	docosapentaenoylcarnitine (C22:5n3)*	Lipid	5762	-2.224	0.03	????--	0.18	5762	-2.305	0.02	????--	0.29	5762	-2.174	0.03	????--	0.34
		100001395	1-linoleoyl-GPC (18:2)	Lipid	13596	-2.226	0.03	------	0.18	13556	-1.908	0.06	------	0.41	13549	-1.149	0.25	-+----	0.73
		892	nonadecanoate (19:0)	Lipid	13596	-2.215	0.03	+-----	0.19	13556	-1.353	0.18	+--+--	0.59	13549	-1.009	0.31	+-++--	0.78
		1528	1-palmitoyl-2-linoleoyl-GPI (16:0/18:2)	Lipid	6246	2.197	0.03	+???++	0.19	6208	1.559	0.12	+???+-	0.52	6208	1.803	0.07	+???+-	0.49
		800	cysteine	Amino acid	12631	-2.197	0.03	-?+---	0.19	12592	-1.455	0.15	-?+---	0.55	12586	-1.226	0.22	-?+---	0.69
		100008993	1-palmitoyl-2-arachidonoyl-GPI (16:0/20:4)*	Lipid	10885	2.19	0.03	+??+++	0.19	10847	1.453	0.15	+??++-	0.55	10847	1.662	0.10	+??++-	0.52
		100000442	quinate	Xenobiotics	12631	-2.181	0.03	+?+---	0.19	12592	-1.854	0.06	+?+---	0.43	12586	-2.054	0.04	+?+---	0.40
		100002990	oleoyl-linoleoyl-glycerol (18:1/18:2) [2]	Lipid	10885	2.183	0.03	-??+++	0.19	10847	1.519	0.13	+??+++	0.53	10847	1.655	0.10	+??++-	0.53
		100002183	S-methylmethionine	Amino acid	5762	-2.182	0.03	????--	0.19	5762	-1.791	0.07	????--	0.45	5762	-1.434	0.15	????--	0.63
		923	dihydroorotate	Nucleotide	6246	-2.162	0.03	+???-+	0.19	6208	-2.281	0.02	+???-+	0.29	6208	-2.199	0.03	+???--	0.34
		100009345	1-palmitoleoyl-2-linolenoyl-GPC (16:1/18:3)*	Lipid	10885	2.156	0.03	+??+++	0.19	10847	1.673	0.09	+??+++	0.47	10847	1.785	0.07	+??+++	0.49
		1110	N-acetylalanine	Amino acid	13596	2.168	0.03	++++++	0.19	13556	1.767	0.08	-+++++	0.45	13549	1.727	0.08	-++++-	0.50
		100010930	palmitoleoyl-linoleoyl-glycerol (16:1/18:2) [1]*	Lipid	6246	2.163	0.03	+???++	0.19	6208	1.41	0.16	+???++	0.57	6208	1.468	0.14	+???++	0.60
		100001501	oleoylcarnitine (C18:1)	Lipid	13596	-2.172	0.03	++----	0.19	13556	-1.459	0.14	++----	0.55	13549	-1.406	0.16	++----	0.64
		100001054	butyrylcarnitine (C4)	Lipid	13596	2.158	0.03	-+++++	0.19	13556	1.765	0.08	-+++++	0.45	13549	1.328	0.18	-+++++	0.68
		100009142	1-stearoyl-2-docosapentaenoyl-GPC (18:0/22:5n6)*	Lipid	9802	2.171	0.03	???++?	0.19	9802	1.302	0.19	???++?	0.62	9802	1.263	0.21	???++?	0.68
		100001277	10-nonadecenoate (19:1n9)	Lipid	13596	-2.173	0.03	+-----	0.19	13556	-1.217	0.22	+-----	0.65	13549	-1.257	0.21	+-----	0.68
		100006651	3,4-methyleneheptanoate	Xenobiotics	10401	-2.16	0.03	???--+	0.19	10401	-1.274	0.20	???--+	0.63	10401	-1.207	0.23	???--+	0.70
		503	serine	Amino acid	12631	-2.164	0.03	-?+---	0.19	12592	-0.576	0.56	-?++--	0.86	12586	-0.374	0.71	-?++--	0.91
		100015641	N-oleoylserine	Lipid	6246	-2.153	0.03	+???--	0.19	6208	-1.77	0.08	+???--	0.45	6208	-1.59	0.11	+???--	0.55
		100001613	tetradecanedioate	Lipid	11850	-2.148	0.03	+-?---	0.19	11811	-1.453	0.15	+-?---	0.55	11810	-1.444	0.15	++?---	0.62
		100010917	palmitoyl-oleoyl-glycerol (16:0/18:1) [2]*	Lipid	6246	2.147	0.03	+???++	0.19	6208	0.883	0.38	+???++	0.79	6208	0.68	0.50	+???+-	0.87
		100001554	2-arachidonoylglycerophosphocholine*	Lipid	10767	2.137	0.03	?+?++?	0.20	10766	2.315	0.02	?+?++?	0.28	10765	2.311	0.02	?+?++?	0.30
		112	3-hydroxy-3-methylglutarate	Lipid	10885	2.135	0.03	+??+-+	0.20	10847	1.361	0.17	+??+-+	0.59	10847	1.409	0.16	+??+-+	0.64
		100001167	pro-hydroxy-pro	Amino acid	13596	-2.135	0.03	++----	0.20	13556	-1.395	0.16	++---+	0.57	13549	-1.265	0.21	++---+	0.68
		100008976	1-stearoyl-2-linoleoyl-GPE (18:0/18:2)*	Lipid	10885	2.141	0.03	+??+++	0.20	10847	0.803	0.42	+??-++	0.81	10847	0.872	0.38	+??-++	0.82
		100000672	1-myristoyl-2-palmitoyl-GPC (14:0/16:0)	Lipid	10401	2.113	0.03	???+++	0.20	10401	1.591	0.11	???++-	0.51	10401	1.751	0.08	???+++	0.50
		100009338	5-bromotryptophan	Amino acid	10286	-2.114	0.03	-??--?	0.20	10248	-1.195	0.23	-??--?	0.65	10248	-1.256	0.21	-??--?	0.68
		100001335	eicosenoate (20:1)	Lipid	13596	-2.103	0.04	+-----	0.21	13556	-0.745	0.46	+--+--	0.83	13549	-0.89	0.37	+-----	0.82
		100001988	5alpha-pregnan-3beta,20alpha-diol disulfate	Lipid	11850	-2.092	0.04	+-?---	0.21	11811	-0.865	0.39	+-?---	0.79	11810	-1.035	0.30	+-?---	0.77
		100002027	androstenediol (3alpha, 17alpha) monosulfate (3)	Lipid	10885	-2.067	0.04	-??---	0.22	10847	-1.179	0.24	-??---	0.66	10847	-1.338	0.18	-??---	0.67
		100015838	eicosenoylcarnitine (C20:1)*	Lipid	6246	-2.059	0.04	+???--	0.23	6208	-1.617	0.11	+???--	0.49	6208	-1.55	0.12	+???--	0.57
		100006098	3-hydroxypyridine sulfate	Xenobiotics	10885	-2.056	0.04	-??---	0.23	10847	-1.679	0.09	+??---	0.47	10847	-2.007	0.04	+??---	0.41
		100005391	3-(3-hydroxyphenyl)propionate sulfate	Xenobiotics	10885	-2.044	0.04	-??---	0.23	10847	-2.29	0.02	-??---	0.29	10847	-2.107	0.04	-??---	0.37
		100002356	17-methylstearate	Lipid	11850	-2.025	0.04	+-?---	0.24	11811	-1.166	0.24	+-?+--	0.66	11810	-1.12	0.26	+-?+--	0.74
		100015966	carotene diol (1)	Xenobiotics	6246	-2.027	0.04	-???--	0.24	6208	-1.049	0.29	+???--	0.71	6208	-0.493	0.62	+???--	0.90
		2051	methylsuccinate	Amino acid	10885	-2.02	0.04	+??---	0.24	10847	-2.506	0.01	+??---	0.25	10847	-2.494	0.01	+??---	0.27
		100001992	androstenediol (3beta,17beta) disulfate (1)	Lipid	13596	2.014	0.04	-+++++	0.24	13556	2.264	0.02	++++++	0.30	13549	1.727	0.08	+++++-	0.50
		100001271	1-stearoyl-GPC (18:0)	Lipid	13596	-2.008	0.04	-+-+--	0.24	13556	-2.362	0.02	-+----	0.28	13549	-1.927	0.05	-+----	0.45
		100002514	hydantoin-5-propionic acid	Amino acid	10885	-2.008	0.04	+??--+	0.24	10847	-2.1	0.04	+??--+	0.35	10847	-1.88	0.06	+??---	0.47
		381	2-aminoadipate	Amino acid	10885	-2.005	0.04	-??---	0.24	10847	-2.078	0.04	-??---	0.35	10847	-2.216	0.03	-??---	0.33
		100005985	sphingomyelin (d18:2/14:0, d18:1/14:1)*	Lipid	10885	2.003	0.05	+??++-	0.24	10847	1.03	0.30	+??++-	0.72	10847	1.325	0.19	+??++-	0.68
		100001256	N-acetylphenylalanine	Amino acid	11850	1.998	0.05	++?-++	0.25	11811	1.535	0.12	++?-++	0.53	11810	1.339	0.18	++?-+-	0.67
		100001402	5-acetylamino-6-formylamino-3-methyluracil	Xenobiotics	10885	-1.994	0.05	-??+--	0.25	10847	-2.061	0.04	-??+--	0.37	10847	-2.327	0.02	-??---	0.29
		100001022	threonate	Cofactors and Vitamins	12631	-1.992	0.05	-?----	0.25	12592	-1.446	0.15	-?--+-	0.55	12586	-0.766	0.44	-?+-+-	0.85
		100000487	glycylvaline	Peptide	2829	1.986	0.05	+?+??-	0.25	2790	1.67	0.09	-?+??-	0.47	2784	1.542	0.12	-?+??-	0.57

		100008918	1-(1-enyl-stearoyl)-2-arachidonoyl-GPC (P-18:0/20:4)	Lipid	9802	-1.979	0.05	???--?	0.25	9802	-1.386	0.17	???--?	0.58	9802	-1.409	0.16	???--?	0.64
		100001994	androstenediol (3beta,17beta) disulfate (2)	Lipid	13596	-1.984	0.05	--+---	0.25	13556	-0.765	0.44	--+---	0.82	13549	-1.037	0.30	--+---	0.77
		100001856	1-stearoyl-2-oleoyl-GPE (18:0/18:1)	Lipid	10885	1.982	0.05	+??+++	0.25	10847	0.65	0.52	+??-++	0.84	10847	0.484	0.63	+??-+-	0.91
		891	margarate (17:0)	Lipid	13596	-1.975	0.05	+-----	0.25	13556	-1.26	0.21	+--+--	0.63	13549	-1.146	0.25	+--+--	0.73
		100006438	citraconate/glutaconate	Energy	10885	-1.97	0.05	+??---	0.25	10847	-1.499	0.13	+??---	0.54	10847	-1.776	0.08	+??---	0.49
		100000846	erythritol	Xenobiotics	12631	1.97	0.05	-?++++	0.25	12592	0.969	0.33	-?+-++	0.76	12586	1.271	0.20	-?++++	0.68
		803	mannose	Carbohydrate	12631	1.963	0.05	+?++++	0.25	12592	0.932	0.35	+?+--+	0.77	12586	0.408	0.68	+?++-+	0.91
		100005353	1-nonadecanoyl-GPC (19:0)	Lipid	9802	-1.959	0.05	???--?	0.25	9802	-2.263	0.02	???--?	0.30	9802	-2.399	0.02	???--?	0.27
		888	caprate (10:0)	Lipid	13596	-1.956	0.05	+-----	0.25	13556	-1.811	0.07	+-----	0.44	13549	-1.557	0.12	+-----	0.57
		100004054	margaroylcarnitine*	Lipid	6246	-1.95	0.05	+???--	0.26	6208	-2.399	0.02	+???--	0.28	6208	-2.217	0.03	+???--	0.33
		100000708	isovalerate	Amino acid	11366	-1.951	0.05	?-?---	0.26	11365	-1.287	0.20	?-?---	0.62	11364	-1.289	0.20	?-?---	0.68
		100001403	5-acetylamino-6-amino-3-methyluracil	Xenobiotics	10885	-1.948	0.05	-??+--	0.26	10847	-2.332	0.02	-??+--	0.28	10847	-2.733	6.27E-03	-??+--	0.21
		1869	2-hydroxyhippurate (salicylurate)	Xenobiotics	10885	-1.938	0.05	-??---	0.26	10847	-2.229	0.03	-??---	0.30	10847	-2.108	0.04	-??---	0.37
		482	lactate	Carbohydrate	13596	1.942	0.05	++++-+	0.26	13556	1.183	0.24	++++-+	0.66	13549	0.971	0.33	++++-+	0.79
		811	alanine	Amino acid	13596	1.933	0.05	-+++-+	0.26	13556	1.908	0.06	--++-+	0.41	13549	1.86	0.06	--++-+	0.47
		100002951	eicosanodioate	Lipid	10885	-1.928	0.05	-??---	0.26	10847	-0.714	0.48	-??---	0.83	10847	-0.785	0.43	-??---	0.85
		100015835	cerotoylcarnitine (C26)*	Lipid	6246	-1.919	0.06	-???--	0.26	6208	-1.935	0.05	-???--	0.41	6208	-1.641	0.10	+???--	0.53
		100001540	pyroglutamine*	Amino acid	13596	1.917	0.06	++++++	0.26	13556	1.028	0.30	++++-+	0.72	13549	1.136	0.26	++++-+	0.73
		100005673	1-docosapentaenoyl-GPC (22:5n6)*	Lipid	9802	1.923	0.05	???++?	0.26	9802	0.963	0.34	???++?	0.76	9802	0.88	0.38	???-+?	0.82
		254	3-hydroxybutyrate (BHBA)	Lipid	13596	-1.919	0.06	+-----	0.26	13556	-0.867	0.39	++----	0.79	13549	-0.772	0.44	++----	0.85
		100001416	orotidine	Nucleotide	10885	1.91	0.06	-??+++	0.26	10847	1.678	0.09	+??+++	0.47	10847	1.756	0.08	+??+++	0.50
		100015837	arachidonoylcarnitine (C20:4)	Lipid	6246	-1.909	0.06	-???--	0.26	6208	-1.623	0.10	-???--	0.49	6208	-1.554	0.12	-???--	0.57
		100001987	5alpha-androstan-3beta,17beta-diol disulfate	Lipid	13596	-1.912	0.06	-++---	0.26	13556	-0.509	0.61	-++-+-	0.88	13549	-0.819	0.41	-++-+-	0.84
		100000282	N-acetylglutamate	Amino acid	10885	1.883	0.06	+??+++	0.27	10847	1.856	0.06	+??+++	0.43	10847	1.884	0.06	+??++-	0.47
		100015760	linoleoylcholine*	Lipid	6246	-1.882	0.06	-???--	0.27	6208	-1.893	0.06	-???--	0.42	6208	-1.673	0.09	-???--	0.52
		407	lysine	Amino acid	13596	-1.885	0.06	--++--	0.27	13556	-1.245	0.21	--++--	0.63	13549	-1.183	0.24	--++--	0.72
		100002113	cysteine sulfinic acid	Amino acid	10885	1.873	0.06	+??+++	0.28	10847	1.039	0.30	+??-++	0.71	10847	0.398	0.69	+??--+	0.91
		100003915	palmitic amide	Lipid	10401	1.86	0.06	???-++	0.28	10401	2.35	0.02	???-++	0.28	10401	2.31	0.02	???-++	0.30
		100001064	glycolithocholate	Lipid	10885	-1.858	0.06	-??--+	0.28	10847	-1.529	0.13	-??--+	0.53	10847	-1.4	0.16	-??--+	0.64
		1084	N-acetylvaline	Amino acid	10885	1.857	0.06	+??+++	0.28	10847	1.299	0.19	+??+++	0.62	10847	1.305	0.19	+??++-	0.68
		535	uridine	Nucleotide	13596	-1.85	0.06	----+-	0.28	13556	-1.485	0.14	---+--	0.54	13549	-1.005	0.31	---+++	0.78
		100008915	1-palmitoyl-2-docosahexaenoyl-GPC (16:0/22:6)	Lipid	10401	1.837	0.07	???++-	0.29	10401	2.502	0.01	???++-	0.25	10401	2.769	5.62E-03	???++-	0.20
		273	cortisone	Lipid	13596	-1.836	0.07	+----+	0.29	13556	-1.184	0.24	+--+++	0.66	13549	-1.146	0.25	+--+++	0.73
		100006627	suberoylcarnitine (C8-DC)	Lipid	1083	1.831	0.07	+????+	0.29	1045	1.511	0.13	+????+	0.53	1045	1.207	0.23	+????+	0.70
		100006435	N-acetylglucosamine/N-acetylgalactosamine	Carbohydrate	10885	1.832	0.07	+??+++	0.29	10847	1.089	0.28	+??+++	0.70	10847	0.75	0.45	+??+++	0.85
		100000882	3-hydroxymyristate	Lipid	1083	-1.827	0.07	-????-	0.29	1045	-1.393	0.16	-????-	0.57	1045	-1.113	0.27	-????-	0.74
		100009336	eicosapentaenoylcholine	Lipid	10885	-1.804	0.07	-??---	0.30	10847	-1.68	0.09	-??---	0.47	10847	-1.544	0.12	-??---	0.57
		100010926	linoleoyl-linolenoyl-glycerol (18:2/18:3) [1]*	Lipid	1083	1.804	0.07	+????+	0.30	1045	1.679	0.09	+????+	0.47	1045	1.52	0.13	+????+	0.58
		100003696	succinimide	Xenobiotics	10401	-1.807	0.07	???---	0.30	10401	-1.402	0.16	???+--	0.57	10401	-1.362	0.17	???+-+	0.67
		100003470	pregnanediol-3-glucuronide	Lipid	11850	-1.811	0.07	+-?-+-	0.30	11811	-1.129	0.26	+-?-+-	0.67	11810	-1.229	0.22	+-?-+-	0.69
		100003674	prolylglycine	Peptide	10885	-1.806	0.07	+??-+-	0.30	10847	-1.269	0.20	+??-+-	0.63	10847	-0.992	0.32	+??-+-	0.79
		100001429	1-margaroylglycerol (17:0)	Lipid	1083	-1.817	0.07	-????-	0.30	1045	-1.958	0.05	-????-	0.40	1045	-0.985	0.32	-????-	0.79
		100001654	1-arachidonoyl-GPI (20:4)*	Lipid	13596	1.815	0.07	+++++-	0.30	13556	1.041	0.30	++++--	0.71	13549	0.979	0.33	++-+--	0.79
		100001614	hexadecanedioate	Lipid	11850	-1.803	0.07	+-?---	0.30	11811	-0.941	0.35	+-?--+	0.77	11810	-0.932	0.35	+-?--+	0.81
		1094	thyroxine	Amino acid	10885	1.799	0.07	+??+++	0.30	10847	2.327	0.02	+??+++	0.28	10847	2.118	0.03	+??+++	0.37
		100001254	N-acetyltryptophan	Amino acid	10885	1.788	0.07	+??++-	0.30	10847	1.765	0.08	+??++-	0.45	10847	1.693	0.09	+??++-	0.52
		821	pseudouridine	Nucleotide	13596	1.79	0.07	++++++	0.30	13556	0.763	0.45	++++-+	0.82	13549	0.721	0.47	++++-+	0.86
		100000802	acetylcarnitine (C2)	Lipid	13596	-1.779	0.08	+-----	0.31	13556	-0.981	0.33	+--+-+	0.75	13549	-1.176	0.24	+--+--	0.72
		100009051	1-stearoyl-2-dihomo-linolenoyl-GPC (18:0/20:3n3 or 6)*	Lipid	9802	1.776	0.08	???++?	0.31	9802	0.405	0.69	???++?	0.90	9802	0.453	0.65	???++?	0.91
		100008980	1-stearoyl-2-linoleoyl-GPC (18:0/18:2)*	Lipid	10885	-1.77	0.08	-??---	0.31	10847	-1.651	0.10	-??---	0.48	10847	-1.482	0.14	-??---	0.60
		100001106	1,3-dimethylurate	Xenobiotics	10885	-1.765	0.08	+??---	0.31	10847	-1.829	0.07	+??---	0.44	10847	-1.989	0.05	+??---	0.42
		100008934	2-hydroxylaurate	Lipid	5647	1.757	0.08	+???+?	0.31	5609	1.939	0.05	+???+?	0.41	5609	1.861	0.06	+???+?	0.47
		893	arachidate (20:0)	Lipid	11850	-1.757	0.08	+-?---	0.31	11811	-0.937	0.35	+-?---	0.77	11810	-0.889	0.37	+-?---	0.82
		100001446	5-methyluridine (ribothymidine)	Nucleotide	11850	-1.753	0.08	++?---	0.31	11811	-0.769	0.44	+-?---	0.82	11810	-0.733	0.46	+-?---	0.86
		1537	1-palmitoyl-2-linoleoyl-GPC (16:0/18:2)	Lipid	10885	1.743	0.08	+??++-	0.31	10847	1.705	0.09	+??++-	0.47	10847	2.032	0.04	+??++-	0.41
		100015785	nisinate (24:6n3)	Lipid	5762	1.743	0.08	????++	0.31	5762	1.823	0.07	????++	0.44	5762	1.862	0.06	????++	0.47
		815	tyrosine	Amino acid	13596	-1.738	0.08	--+---	0.31	13556	-1.562	0.12	--+---	0.52	13549	-1.838	0.07	--+---	0.47
		100015840	dihomo-linolenoylcarnitine (20:3n3 or 6)*	Lipid	6246	-1.736	0.08	-???--	0.31	6208	-1.751	0.08	-???--	0.46	6208	-1.572	0.12	-???--	0.56
		100000616	1-stearoyl-2-arachidonoyl-GPI (18:0/20:4)	Lipid	10885	1.737	0.08	-??+++	0.31	10847	1.414	0.16	+??++-	0.57	10847	1.557	0.12	+??++-	0.57

		100009138	1-myristoyl-2-linoleoyl-GPC (14:0/18:2)*	Lipid	9802	1.729	0.08	???++?	0.31	9802	1.251	0.21	???++?	0.63	9802	1.384	0.17	???++?	0.65
		1235	gamma-glutamylhistidine	Peptide	10885	1.745	0.08	+??++-	0.31	10847	1.224	0.22	+??++-	0.64	10847	1.323	0.19	+??++-	0.68
		480	proline	Amino acid	13596	1.73	0.08	-+++-+	0.31	13556	1.445	0.15	-+++-+	0.55	13549	1.181	0.24	-+++-+	0.72
		439	stearate (18:0)	Lipid	13596	-1.728	0.08	+-----	0.31	13556	-0.891	0.37	+-----	0.79	13549	-0.881	0.38	+-----	0.82
		267	choline phosphate	Lipid	10885	-1.729	0.08	-??---	0.31	10847	-0.453	0.65	-??+--	0.89	10847	-0.461	0.64	-??+--	0.91
		100002154	ergothioneine	Xenobiotics	12631	-1.742	0.08	-?----	0.31	12592	-0.093	0.93	-?-++-	0.97	12586	0.354	0.72	-?-++-	0.92
		100001270	myristoylcarnitine (C14)	Lipid	10885	-1.718	0.09	+??---	0.32	10847	-1.059	0.29	+??---	0.71	10847	-1.051	0.29	+??---	0.76
		100000774	phenyllactate (PLA)	Amino acid	13596	-1.711	0.09	+-+---	0.32	13556	-1.469	0.14	+-+---	0.55	13549	-1.415	0.16	+-+---	0.64
		100010934	diacylglycerol (14:0/18:1, 16:0/16:1) [1]*	Lipid	1083	1.711	0.09	+????+	0.32	1045	0.893	0.37	+????-	0.79	1045	0.694	0.49	+????-	0.87
		100001400	1-methylurate	Xenobiotics	12631	-1.704	0.09	+?----	0.32	12592	-2.217	0.03	-?----	0.30	12586	-2.696	7.01E-03	-?----	0.21
		100001383	1-myristoylglycerophosphocholine (14:0)	Lipid	10767	1.707	0.09	?-?++?	0.32	10766	1	0.32	?-?++?	0.73	10765	1.137	0.26	?+?++?	0.73
		100001527	hexanoylglycine	Lipid	5722	-1.704	0.09	-??-?-	0.32	5684	-0.631	0.53	-??+?-	0.84	5684	-0.522	0.60	-??-?-	0.89
		100001655	1-palmitoyl-GPI (16:0)	Lipid	13596	1.707	0.09	+-+++-	0.32	13556	0.399	0.69	+--++-	0.90	13549	0.498	0.62	+--+--	0.90
		100001989	glycocholenate sulfate*	Lipid	11850	-1.698	0.09	-+?--+	0.32	11811	-0.617	0.54	-+?--+	0.85	11810	-0.92	0.36	-+?--+	0.82
		100003001	1-(1-enyl-stearoyl)-GPE (P-18:0)*	Lipid	10885	-1.688	0.09	-??---	0.33	10847	-1.889	0.06	-??---	0.42	10847	-1.635	0.10	-??---	0.53
		100010927	linoleoyl-linolenoyl-glycerol (18:2/18:3) [2]*	Lipid	5647	-1.674	0.09	-???-?	0.33	5609	-2.192	0.03	-???-?	0.31	5609	-2.069	0.04	-???-?	0.39
		100001509	2-methylbutyrylcarnitine (C5)	Amino acid	8957	-1.672	0.09	---?--	0.33	8917	-1.55	0.12	---?--	0.52	8910	-2.002	0.05	---?--	0.41
		1002	allantoin	Nucleotide	10885	1.673	0.09	+??+++	0.33	10847	0.647	0.52	+??+++	0.84	10847	0.858	0.39	+??++-	0.83
		100009015	1-(1-enyl-stearoyl)-2-docosahexaenoyl-GPC (P-18:0/22:6)*	Lipid	9802	-1.675	0.09	???--?	0.33	9802	-0.711	0.48	???+-?	0.83	9802	-0.568	0.57	???+-?	0.88
		100002989	oleoyl-linoleoyl-glycerol (18:1/18:2) [1]	Lipid	10885	1.67	0.10	-??+++	0.33	10847	1.155	0.25	+??+++	0.66	10847	1.26	0.21	+??+++	0.68
		100002026	androstenediol (3alpha, 17alpha) monosulfate (2)	Lipid	10885	-1.668	0.10	-??---	0.33	10847	-0.762	0.45	+??---	0.82	10847	-0.864	0.39	+??---	0.83
		1053	3-ureidopropionate	Nucleotide	10885	-1.661	0.10	+??--+	0.34	10847	-2.175	0.03	-??--+	0.32	10847	-2.005	0.04	-??--+	0.41
		415	methionine	Amino acid	13596	-1.66	0.10	--++--	0.34	13556	-0.595	0.55	--++--	0.86	13549	-0.611	0.54	--++--	0.87
		100000611	1-palmityl-GPC (O-16:0)	Lipid	9802	-1.654	0.10	???--?	0.34	9802	-1.999	0.05	???--?	0.39	9802	-2.009	0.04	???--?	0.41
		932	caprylate (8:0)	Lipid	13596	-1.651	0.10	-+----	0.34	13556	-1.704	0.09	-+----	0.47	13549	-1.569	0.12	-+----	0.56
		100006129	vanillactate	Amino acid	6246	1.651	0.10	+???+-	0.34	6208	1.435	0.15	+???+-	0.55	6208	1.424	0.15	+???+-	0.63
		100001755	4-vinylphenol sulfate	Xenobiotics	13596	1.656	0.10	-++++-	0.34	13556	1.14	0.25	-++++-	0.67	13549	-0.472	0.64	-+--+-	0.91
		100000453	paraxanthine	Xenobiotics	13596	-1.645	0.10	-+-+--	0.34	13556	-1.733	0.08	-+++--	0.47	13549	-1.796	0.07	-+++--	0.49
		565	tryptophan	Amino acid	13596	-1.646	0.10	--+---	0.34	13556	-0.838	0.40	--++--	0.81	13549	-0.92	0.36	---+--	0.82
		100004284	dimethyl sulfone	Xenobiotics	11850	-1.639	0.10	--?---	0.34	11811	-1.957	0.05	--?---	0.40	11810	-1.695	0.09	--?---	0.52
		100001461	1-stearoyl-GPE (18:0)	Lipid	13596	1.641	0.10	+++++-	0.34	13556	0.546	0.59	+++++-	0.87	13549	0.849	0.40	++++++	0.83
		1004	xanthine	Nucleotide	13596	-1.634	0.10	-----+	0.34	13556	-1.33	0.18	---+-+	0.60	13549	-1.485	0.14	---+-+	0.60
		100002070	2-hydroxyglutarate	Lipid	7992	1.629	0.10	-?+?++	0.34	7953	2.407	0.02	-?+?++	0.28	7947	2.377	0.02	-?+?+-	0.28
		100000956	8-hydroxyoctanoate	Lipid	5722	-1.626	0.10	-??-?-	0.34	5684	-1.799	0.07	-??-?-	0.44	5684	-1.739	0.08	-??-?-	0.50
		100004541	acisoga	Amino acid	13596	-1.626	0.10	-----+	0.34	13556	-1.318	0.19	-----+	0.61	13549	-1.709	0.09	---+--	0.52
		100010919	oleoyl-oleoyl-glycerol (18:1/18:1) [2]*	Lipid	6246	1.63	0.10	+???++	0.34	6208	0.661	0.51	+???++	0.84	6208	0.467	0.64	+???+-	0.91
		100001337	linolenate [alpha or gamma; (18:3n3 or 6)]	Lipid	13596	-1.626	0.10	+----+	0.34	13556	-0.554	0.58	+----+	0.86	13549	-0.395	0.69	+----+	0.91
		100006282	umbelliferone sulfate	Xenobiotics	10885	-1.624	0.10	-??-+-	0.34	10847	-1.171	0.24	-??-+-	0.66	10847	-1.091	0.28	-??-+-	0.75
		100009055	palmitoyl-linoleoyl-glycerol (16:0/18:2) [2]*	Lipid	10885	1.614	0.11	-??+++	0.35	10847	0.929	0.35	-??+++	0.77	10847	1.097	0.27	-??+++	0.75
		100001034	indoleacetate	Amino acid	13596	-1.609	0.11	-+----	0.35	13556	-1.597	0.11	++----	0.50	13549	-1.304	0.19	++----	0.68
		1023	sarcosine	Amino acid	10885	-1.61	0.11	+??---	0.35	10847	-0.826	0.41	+??-+-	0.81	10847	-0.642	0.52	+??-+-	0.87
		180	linoleate (18:2n6)	Lipid	13596	-1.603	0.11	------	0.35	13556	-0.378	0.71	---+--	0.91	13549	-0.14	0.89	---+-+	0.96
		922	N-stearoyl-sphinganine (d18:0/18:0)*	Lipid	1083	1.6	0.11	+????+	0.35	1045	1.182	0.24	+????+	0.66	1045	0.568	0.57	+????-	0.88
		501	salicylate	Xenobiotics	10885	-1.597	0.11	-??---	0.35	10847	-1.71	0.09	-??---	0.47	10847	-1.601	0.11	-??---	0.55
		100004110	3-methyl catechol sulfate (2)	Xenobiotics	10885	-1.578	0.11	-??-+-	0.36	10847	-1.347	0.18	+??-+-	0.59	10847	-2.23	0.03	+??---	0.33
		100001162	propionylcarnitine (C3)	Lipid	13596	-1.574	0.12	--++--	0.36	13556	-2.052	0.04	--++--	0.37	13549	-2.334	0.02	---+--	0.29
		2054	ethylmalonate	Amino acid	10885	1.575	0.12	+??+++	0.36	10847	1.243	0.21	-??+++	0.63	10847	1.352	0.18	-??+++	0.67
		100008930	oleate/vaccenate (18:1)	Lipid	12631	-1.566	0.12	+?----	0.36	12592	-0.657	0.51	+?-+--	0.84	12586	-0.76	0.45	+?-+--	0.85
		313	sphinganine	Lipid	11850	1.565	0.12	-+?+++	0.36	11811	0.632	0.53	-+?+++	0.84	11810	0.564	0.57	-+?+++	0.88
		266	cholesterol	Lipid	12631	1.559	0.12	+?+++-	0.37	12592	0.8	0.42	+?++--	0.81	12586	1.019	0.31	+?++--	0.78
		100015832	behenoylcarnitine (C22)*	Lipid	6246	-1.556	0.12	-???--	0.37	6208	-1.486	0.14	-???--	0.54	6208	-1.394	0.16	-???--	0.64
		100003901	2-stearoyl-GPE (18:0)*	Lipid	10885	1.553	0.12	+??++-	0.37	10847	0.738	0.46	+??+--	0.83	10847	0.914	0.36	+??+-+	0.82
		100002102	N-acetyl-beta-alanine	Nucleotide	11850	1.55	0.12	+-?-++	0.37	11811	1.157	0.25	+-?-++	0.66	11810	1.171	0.24	+-?-+-	0.72
		100004111	4-methylcatechol sulfate	Xenobiotics	11850	-1.537	0.12	-+?---	0.38	11811	-1.666	0.10	++?---	0.48	11810	-1.648	0.10	-+?---	0.53
		100009027	sphingomyelin (d18:0/18:0, d19:0/17:0)*	Lipid	6246	1.537	0.12	+???++	0.38	6208	0.617	0.54	+???++	0.85	6208	0.666	0.51	+???++	0.87
		100010950	stearoyl-arachidonoyl-glycerol (18:0/20:4) [2]*	Lipid	1083	-1.532	0.13	-????-	0.38	1045	-1.563	0.12	-????-	0.52	1045	-1.456	0.15	-????-	0.61
		100001102	dodecanedioate	Lipid	11850	-1.527	0.13	++?---	0.38	11811	-1.009	0.31	++?---	0.73	11810	-0.836	0.40	++?--+	0.83
		100010924	palmitoyl-arachidonoyl-glycerol (16:0/20:4) [1]*	Lipid	6246	1.521	0.13	+???+-	0.39	6208	0.762	0.45	+???+-	0.82	6208	0.819	0.41	+???+-	0.84
		799	betaine	Amino acid	13596	-1.517	0.13	-+---+	0.39	13556	-0.942	0.35	-+---+	0.77	13549	-0.79	0.43	-+---+	0.85

		1221	creatine	Amino acid	13596	-1.509	0.13	++---+	0.39	13556	-1.175	0.24	++---+	0.66	13549	-1.567	0.12	++----	0.56
		100009141	1-stearoyl-2-docosapentaenoyl-GPC (18:0/22:5n3)*	Lipid	9802	1.509	0.13	???++?	0.39	9802	1.005	0.31	???+-?	0.73	9802	0.922	0.36	???+-?	0.82
		100010937	oleoyl-arachidonoyl-glycerol (18:1/20:4) [2]*	Lipid	6246	1.508	0.13	-???++	0.39	6208	0.774	0.44	-???++	0.81	6208	0.752	0.45	-???++	0.85
		100015968	carotene diol (3)	Xenobiotics	6246	-1.505	0.13	-???--	0.39	6208	-0.577	0.56	+???+-	0.86	6208	-0.209	0.83	+???+-	0.95
		100002106	sphingomyelin (d18:1/18:1, d18:2/18:0)	Lipid	10885	1.503	0.13	+??++-	0.39	10847	1.046	0.30	+??+--	0.71	10847	1.147	0.25	+??+-+	0.73
		1087	erucate (22:1n9)	Lipid	11850	-1.498	0.13	++?---	0.40	11811	-0.641	0.52	++?---	0.84	11810	-0.715	0.47	++?---	0.86
		881	cytosine	Nucleotide	6246	-1.49	0.14	-???--	0.40	6208	-1.843	0.07	-???--	0.43	6208	-1.664	0.10	-???--	0.52
		100002009	5alpha-pregnan-3beta,20beta-diol monosulfate (1)	Lipid	10885	-1.49	0.14	-??---	0.40	10847	-0.612	0.54	-??+-	0.85	10847	-0.556	0.58	-??+-	0.88
		100001618	1-myristoylglycerol (14:0)	Lipid	10885	1.483	0.14	+??+++	0.40	10847	0.435	0.66	+??-++	0.90	10847	0.429	0.67	+??-++	0.91
		100002749	S-methylcysteine	Amino acid	13596	-1.482	0.14	+---+-	0.40	13556	-0.323	0.75	+--++-	0.92	13549	-0.182	0.86	+--++-	0.95
		278	cysteinylglycine	Amino acid	10885	-1.48	0.14	-??--+	0.40	10847	-1.248	0.21	-??--+	0.63	10847	-1.128	0.26	-??-++	0.73
		100001445	1-palmitoyl-GPA (16:0)	Lipid	11251	-1.466	0.14	--?--?	0.41	11212	-2.233	0.03	--?--?	0.30	11211	-2.267	0.02	--?--?	0.31
		100001882	glycosyl-N-stearoyl-sphingosine (d18:1/18:0)	Lipid	10885	-1.467	0.14	+??---	0.41	10847	-1.875	0.06	+??---	0.42	10847	-1.592	0.11	+??---	0.55
		1547	N-stearoyl-sphingosine (d18:1/18:0)*	Lipid	6246	1.463	0.14	+???++	0.41	6208	0.166	0.87	+???-+	0.96	6208	-0.067	0.95	+???-+	0.98
		100015845	docosahexaenoylcarnitine (C22:6)*	Lipid	5762	-1.462	0.14	????--	0.41	5762	-1.112	0.27	????--	0.68	5762	-1.028	0.30	????--	0.77
		100001604	hydroquinone sulfate	Xenobiotics	10885	1.459	0.14	-??++-	0.41	10847	0.767	0.44	-??++-	0.82	10847	0.382	0.70	-??+-	0.91
		1538	stearoyl sphingomyelin (d18:1/18:0)	Lipid	10885	1.457	0.15	+??+-+	0.41	10847	0.949	0.34	+??+-+	0.77	10847	0.921	0.36	+??+-+	0.82
		100003000	1-(1-enyl-palmitoyl)-GPE (P-16:0)*	Lipid	10885	-1.449	0.15	-??---	0.41	10847	-1.551	0.12	-??---	0.52	10847	-1.113	0.27	-??---	0.74
		100004635	methionine sulfone	Amino acid	10885	1.449	0.15	-??++-	0.41	10847	0.301	0.76	-??++-	0.92	10847	0.378	0.71	-??++-	0.91
		100000665	docosahexaenoate (DHA; 22:6n3)	Lipid	13596	-1.45	0.15	+--+--	0.41	13556	-0.398	0.69	+--+--	0.90	13549	0.014	0.99	+--+--	0.99
		100001569	1-oleoyl-GPE (18:1)	Lipid	13596	1.442	0.15	+++-+-	0.41	13556	0.722	0.47	+++-++	0.83	13549	0.682	0.50	+++-++	0.87
		100001810	dimethylarginine (SDMA + ADMA)	Amino acid	12631	-1.435	0.15	-?----	0.42	12592	-1.35	0.18	-?---+	0.59	12586	-1.508	0.13	-?---+	0.58
		100001262	gamma-glutamyl-epsilon-lysine	Peptide	10885	-1.436	0.15	+??---	0.42	10847	-0.847	0.40	-??+-	0.80	10847	-0.924	0.36	-??+-	0.82
		100002185	indole-3-carboxylic acid	Amino acid	5722	-1.431	0.15	-??-?-	0.42	5684	-1.688	0.09	-??-?-	0.47	5684	-1.509	0.13	-??-?-	0.58
		100015792	sphingomyelin (d18:1/25:0, d19:0/24:1, d20:1/23:0, d19:1/24:0)*	Lipid	6246	-1.428	0.15	-???--	0.42	6208	-2.024	0.04	-???--	0.38	6208	-1.84	0.07	-???--	0.47
		100009271	3-hydroxybutyrylcarnitine (2)	Lipid	10885	-1.427	0.15	+??--+	0.42	10847	-1.373	0.17	+??--+	0.59	10847	-1.433	0.15	+??---	0.63
		100009054	palmitoleoyl-oleoyl-glycerol (16:1/18:1) [2]*	Lipid	1083	1.417	0.16	+????+	0.42	1045	1.083	0.28	+????+	0.70	1045	0.698	0.48	+????-	0.87
		100015625	glycosyl-N-behenoyl-sphingadienine (d18:2/22:0)*	Lipid	5762	-1.402	0.16	????--	0.43	5762	-1.812	0.07	????--	0.44	5762	-1.278	0.20	????--	0.68
		806	dimethylglycine	Amino acid	10885	-1.402	0.16	+??---	0.43	10847	-1.089	0.28	+??---	0.70	10847	-1.271	0.20	+??---	0.68
		100001851	N-acetylserine	Amino acid	10885	1.406	0.16	+??++-	0.43	10847	1.075	0.28	+??++-	0.70	10847	1.101	0.27	+??++-	0.75
		100009181	1-stearoyl-2-oleoyl-GPI (18:0/18:1)*	Lipid	6246	1.405	0.16	+???+-	0.43	6208	0.71	0.48	+???+-	0.83	6208	0.811	0.42	+???++	0.84
		100004634	3-methoxytyramine sulfate	Amino acid	1083	1.404	0.16	+????-	0.43	1045	1.053	0.29	+????-	0.71	1045	0.716	0.47	+????-	0.86
		100008953	2-palmitoleoylglycerol (16:1)*	Lipid	5123	1.4	0.16	+??+??	0.43	5085	0.577	0.56	+??+??	0.86	5085	0.468	0.64	+??+??	0.91
		100001040	1-linoleoylglycerol (18:2)	Lipid	12631	-1.387	0.17	-?----	0.43	12592	-1.519	0.13	-?----	0.53	12586	-1.294	0.20	-?----	0.68
		100001314	gamma-glutamylthreonine	Peptide	12631	-1.388	0.17	-?+---	0.43	12592	-1.182	0.24	-?+---	0.66	12586	-1.306	0.19	-?+---	0.68
		100015727	ceramide (d16:1/24:1, d18:1/22:1)*	Lipid	1083	1.395	0.16	+????+	0.43	1045	1.2	0.23	+????+	0.65	1045	1.066	0.29	+????+	0.76
		100009166	phosphatidylcholine (16:0/22:5n3, 18:1/20:4)*	Lipid	9802	1.388	0.17	???++?	0.43	9802	1.007	0.31	???-+?	0.73	9802	0.984	0.33	???-+?	0.79
		100010916	palmitoyl-oleoyl-glycerol (16:0/18:1) [1]*	Lipid	6246	1.39	0.16	+???++	0.43	6208	0.37	0.71	+???+-	0.91	6208	0.322	0.75	+???+-	0.92
		1231	dihomo-linoleate (20:2n6)	Lipid	13596	-1.384	0.17	+-----	0.43	13556	-0.067	0.95	+--+--	0.97	13549	-0.136	0.89	+--+-+	0.96
		100006314	sphingomyelin (d17:1/16:0, d18:1/15:0, d16:1/17:0)*	Lipid	10885	-1.382	0.17	-??+--	0.43	10847	-1.686	0.09	-??+--	0.47	10847	-1.518	0.13	-??+--	0.58
		100015836	ximenoylcarnitine (C26:1)*	Lipid	6246	-1.373	0.17	-???--	0.44	6208	-1.458	0.14	-???--	0.55	6208	-1.268	0.20	-???--	0.68
		100002875	1-(1-enyl-palmitoyl)-GPC (P-16:0)*	Lipid	10885	-1.369	0.17	-??+--	0.44	10847	-1.64	0.10	-??+--	0.49	10847	-1.248	0.21	-??+--	0.68
		100003432	dihydroferulic acid	Xenobiotics	10401	-1.363	0.17	???---	0.44	10401	-1.34	0.18	???---	0.60	10401	-1.242	0.21	???---	0.68
		100000656	1-stearoyl-GPI (18:0)	Lipid	12631	1.362	0.17	+?+++-	0.44	12592	0.497	0.62	+?++--	0.88	12586	0.585	0.56	+?++--	0.87
		100001208	1-methylimidazoleacetate	Amino acid	10885	1.36	0.17	-??-++	0.44	10847	1.495	0.13	-??-++	0.54	10847	1.266	0.21	-??-++	0.68
		100002028	androstenediol (3beta,17beta) monosulfate (1)	Lipid	10885	1.351	0.18	+??+--	0.45	10847	1.114	0.27	+??++-	0.68	10847	0.934	0.35	+??++-	0.81
		1025	pipecolate	Amino acid	13596	1.344	0.18	+-+++-	0.45	13556	1.536	0.12	+-+++-	0.53	13549	1.673	0.09	+-+++-	0.52
		100002063	1-docosapentaenoylglycerophosphocholine (22:5n3)*	Lipid	10767	1.338	0.18	?+?+-?	0.46	10766	1.26	0.21	?+?+-?	0.63	10765	1.195	0.23	?+?+-?	0.71
		818	malonate	Lipid	9802	-1.335	0.18	???-+?	0.46	9802	-1.299	0.19	???--?	0.62	9802	-1.283	0.20	???--?	0.68
		100004208	O-methylcatechol sulfate	Xenobiotics	10885	-1.331	0.18	-??---	0.46	10847	-1.756	0.08	+??---	0.46	10847	-2.441	0.01	+??---	0.27
		100001605	catechol sulfate	Xenobiotics	13596	-1.333	0.18	+++---	0.46	13556	-1.644	0.10	+++---	0.48	13549	-1.648	0.10	+++---	0.53
		100001423	4-hydroxyhippurate	Xenobiotics	11850	1.324	0.19	++?++-	0.46	11811	0.092	0.93	++?+--	0.97	11810	0.422	0.67	++?+--	0.91
		477	pristanate	Lipid	6246	-1.324	0.19	+???--	0.46	6208	-0.69	0.49	+???--	0.84	6208	-0.411	0.68	+???--	0.91
		231	arginine	Amino acid	13596	-1.318	0.19	---+-+	0.46	13556	-1.69	0.09	---+--	0.47	13549	-1.805	0.07	-----+	0.49
		437	pelargonate (9:0)	Lipid	13596	-1.32	0.19	-+----	0.46	13556	-1.046	0.30	-+----	0.71	13549	-0.773	0.44	-+----	0.85
		100004328	sphingomyelin (d18:1/14:0, d16:1/16:0)*	Lipid	10885	1.318	0.19	+??++-	0.46	10847	0.324	0.75	+??+--	0.92	10847	0.594	0.55	+??+--	0.87
		100005850	3-methylglutarylcarnitine (2)	Amino acid	10885	1.316	0.19	+??+++	0.47	10847	1.976	0.05	+??+++	0.40	10847	1.885	0.06	+??+++	0.47
		100001778	1-linoleoyl-GPI (18:2)*	Lipid	10885	1.314	0.19	+??++-	0.47	10847	0.627	0.53	+??++-	0.84	10847	0.875	0.38	+??++-	0.82
		100002060	1-docosahexaenoylglycerophosphoethanolamine*	Lipid	10767	1.306	0.19	?-?++?	0.47	10766	1.718	0.09	?-?++?	0.47	10765	1.856	0.06	?+?++?	0.47

		49	putrescine	Amino acid	1083	-1.306	0.19	-????-	0.47	1045	-1.367	0.17	-????-	0.59	1045	-1.297	0.19	-????-	0.68
		355	histidine	Amino acid	13596	-1.305	0.19	---+--	0.47	13556	-0.918	0.36	---+--	0.78	13549	-0.692	0.49	---+--	0.87
		100009406	palmitoleoylcarnitine (C16:1)*	Lipid	10885	-1.3	0.19	+??---	0.47	10847	-0.148	0.88	+??+--	0.96	10847	-0.174	0.86	+??+--	0.96
		100000936	3-methyl-2-oxobutyrate	Amino acid	13596	-1.291	0.20	--+--+	0.48	13556	-0.662	0.51	--+--+	0.84	13549	-0.917	0.36	--+---	0.82
		100001869	1-stearoyl-2-arachidonoyl-GPC (18:0/20:4)	Lipid	10885	1.291	0.20	-??+++	0.48	10847	1.083	0.28	-??+++	0.70	10847	0.908	0.36	-??+++	0.82
		100005371	1-eicosatrienoylglycerophosphoethanolamine*	Lipid	10767	1.292	0.20	?-?++?	0.48	10766	-0.014	0.99	?-?++?	0.99	10765	-0.073	0.94	?-?++?	0.98
		100001148	5-hydroxyhexanoate	Lipid	10885	1.287	0.20	+??++-	0.48	10847	0.363	0.72	+??-+-	0.91	10847	0.519	0.60	+??-+-	0.89
		100001806	o-cresol sulfate	Xenobiotics	10885	1.285	0.20	-??+++	0.48	10847	0.835	0.40	-??+-+	0.81	10847	-1.231	0.22	-??---	0.69
		100002018	5alpha-androstan-3alpha,17beta-diol monosulfate (1)	Lipid	10885	-1.281	0.20	-??---	0.48	10847	-0.531	0.60	-??-+-	0.87	10847	-0.548	0.58	-??-+-	0.89
		100002953	16-hydroxypalmitate	Lipid	6246	-1.275	0.20	+???--	0.48	6208	-0.931	0.35	+???--	0.77	6208	-0.888	0.37	+???-+	0.82
		100009232	thioproline	Xenobiotics	10885	-1.275	0.20	-??--+	0.48	10847	-0.398	0.69	-??--+	0.90	10847	-0.264	0.79	-??--+	0.93
		100009147	1-stearyl-GPC (O-18:0)*	Lipid	9802	-1.267	0.21	???--?	0.49	9802	-1.626	0.10	???--?	0.49	9802	-1.718	0.09	???--?	0.51
		100001278	10-heptadecenoate (17:1n7)	Lipid	13596	-1.26	0.21	+-----	0.49	13556	-0.452	0.65	+--+--	0.89	13549	-0.566	0.57	+--+--	0.88
		100001466	3-methylcytidine	Nucleotide	6246	1.253	0.21	+???++	0.50	6208	1.328	0.18	+???++	0.60	6208	1.119	0.26	+???++	0.74
		913	maltose	Carbohydrate	10885	1.245	0.21	-??+++	0.50	10847	-0.097	0.92	-??-++	0.97	10847	-0.213	0.83	-??-++	0.95
		100000039	methionine sulfoxide	Amino acid	10885	1.235	0.22	+??+++	0.50	10847	0.504	0.61	+??-+-	0.88	10847	0.557	0.58	+??-+-	0.88
		100002876	1-(1-enyl-oleoyl)-GPC (P-18:1)*	Lipid	9802	-1.233	0.22	???--?	0.51	9802	-1.139	0.25	???--?	0.67	9802	-1.239	0.22	???--?	0.68
		100000042	3-methylhistidine	Amino acid	11850	-1.228	0.22	-+?---	0.51	11811	-1.208	0.23	-+?---	0.65	11810	-1.078	0.28	-+?---	0.75
		1224	cys-gly, oxidized	Amino acid	10885	-1.211	0.23	-??-+-	0.52	10847	-1.213	0.22	-??-+-	0.65	10847	-1.26	0.21	-??-+-	0.68
		356	cortisol	Lipid	13596	-1.203	0.23	+--+++	0.53	13556	-1.299	0.19	+--+++	0.62	13549	-0.968	0.33	+--+++	0.79
		100008929	2-methylcitrate/homocitrate	Energy	1083	-1.201	0.23	+????-	0.53	1045	-1.214	0.22	+????-	0.65	1045	-0.983	0.33	+????-	0.79
		100010918	oleoyl-oleoyl-glycerol (18:1/18:1) [1]*	Lipid	6246	1.198	0.23	+???+-	0.53	6208	0.148	0.88	+???+-	0.96	6208	-0.03	0.98	+???+-	0.99
		100001652	2-palmitoylglycerophosphoethanolamine*	Lipid	10767	1.195	0.23	?-?++?	0.53	10766	0.343	0.73	?-?++?	0.92	10765	0.423	0.67	?-?++?	0.91
		100006298	lignoceroyl sphingomyelin (d18:1/24:0)	Lipid	6246	-1.188	0.23	-???+-	0.53	6208	-1.521	0.13	-???--	0.53	6208	-1.302	0.19	-???--	0.68
		100000295	tartarate	Xenobiotics	1083	-1.188	0.23	-????-	0.53	1045	-1.175	0.24	-????-	0.66	1045	-1.089	0.28	-????-	0.75
		100015831	linolenoylcarnitine (C18:3)*	Lipid	6246	-1.187	0.24	+???--	0.53	6208	-0.709	0.48	+???--	0.83	6208	-0.471	0.64	+???-+	0.91
		1125	5,6-dihydrouracil	Nucleotide	1083	1.185	0.24	+????+	0.53	1045	1.531	0.13	+????+	0.53	1045	1.927	0.05	+????+	0.45
		100010869	2,3-dihydroxy-2-methylbutyrate	Amino acid	6246	-1.183	0.24	-???-+	0.53	6208	-1.557	0.12	-???-+	0.52	6208	-1.79	0.07	-???-+	0.49
		100009153	1-stearoyl-2-meadoyl-GPC (18:0/20:3n9)*	Lipid	9802	1.18	0.24	???++?	0.54	9802	0.741	0.46	???++?	0.83	9802	0.519	0.60	???++?	0.89
		100008989	1-palmitoyl-2-eicosapentaenoyl-GPC (16:0/20:5)*	Lipid	9802	1.172	0.24	???++?	0.54	9802	1.464	0.14	???++?	0.55	9802	1.469	0.14	???++?	0.60
		100010941	linoleoyl-linoleoyl-glycerol (18:2/18:2) [1]*	Lipid	6246	1.173	0.24	-???++	0.54	6208	1.157	0.25	-???++	0.66	6208	1.363	0.17	-???++	0.67
		100004046	N-acetylcarnosine	Peptide	11850	-1.171	0.24	+-?---	0.54	11811	-0.881	0.38	+-?+--	0.79	11810	-0.81	0.42	+-?+--	0.84
		100005864	methyl glucopyranoside (alpha + beta)	Xenobiotics	10885	-1.168	0.24	-??-+-	0.54	10847	-0.895	0.37	-??-+-	0.79	10847	-0.676	0.50	-??-+-	0.87
		100001267	piperine	Xenobiotics	13596	-1.161	0.25	----++	0.55	13556	-0.475	0.63	----++	0.89	13549	-0.189	0.85	----++	0.95
		100003271	beta-citrylglutamate	Amino acid	6246	-1.158	0.25	+???--	0.55	6208	-1.896	0.06	+???--	0.42	6208	-1.946	0.05	+???--	0.45
		100001026	galactonate	Carbohydrate	10885	1.156	0.25	+??+--	0.55	10847	0.774	0.44	+??+--	0.81	10847	0.89	0.37	+??+--	0.82
		100001405	1-methylxanthine	Xenobiotics	12631	-1.154	0.25	+?++--	0.55	12592	-1	0.32	+?++--	0.73	12586	-1.348	0.18	+?++--	0.67
		100000776	palmitoylcarnitine (C16)	Lipid	13596	-1.15	0.25	+--+--	0.55	13556	-0.783	0.43	++-+--	0.81	13549	-0.633	0.53	++-+--	0.87
		1629	taurochenodeoxycholate	Lipid	11850	-1.132	0.26	-+?---	0.56	11811	-0.916	0.36	-+?---	0.78	11810	-1.101	0.27	-+?---	0.75
		100015620	lactosyl-N-nervonoyl-sphingosine (d18:1/24:1)*	Lipid	6246	-1.134	0.26	+???--	0.56	6208	-1.25	0.21	+???--	0.63	6208	-1.046	0.30	+???-+	0.77
		100000406	ribitol	Carbohydrate	10885	1.132	0.26	+??+-+	0.56	10847	0.923	0.36	+??+-+	0.77	10847	0.881	0.38	+??+-+	0.82
		100008903	1,2-dilinoleoyl-GPC (18:2/18:2)	Lipid	10286	-1.131	0.26	-??--?	0.56	10248	-0.651	0.52	-??-+?	0.84	10248	-0.592	0.55	-??-+?	0.87
		100010936	oleoyl-arachidonoyl-glycerol (18:1/20:4) [1]*	Lipid	6246	1.134	0.26	-???++	0.56	6208	0.422	0.67	-???++	0.90	6208	0.391	0.70	-???++	0.91
		100001181	docosapentaenoate (n3 DPA; 22:5n3)	Lipid	13596	-1.133	0.26	+--+--	0.56	13556	-0.093	0.93	+--+--	0.97	13549	0.074	0.94	+--+--	0.98
		100009036	1-margaroyl-2-oleoyl-GPC (17:0/18:1)*	Lipid	9802	1.129	0.26	???++?	0.56	9802	0.267	0.79	???+-?	0.93	9802	0.043	0.97	???+-?	0.99
		100001391	stearoylcarnitine (C18)	Lipid	13596	-1.126	0.26	++-+--	0.56	13556	-0.804	0.42	++-+--	0.81	13549	-0.618	0.54	++-+--	0.87
		100004555	benzoylcarnitine*	Xenobiotics	1083	-1.121	0.26	+????-	0.56	1045	-1.162	0.25	+????-	0.66	1045	-1.127	0.26	+????-	0.73
		229	arachidonate (20:4n6)	Lipid	13596	1.111	0.27	-++++-	0.57	13556	1.079	0.28	-++++-	0.70	13549	1.243	0.21	-++++-	0.68
		100009333	docosahexaenoylcholine	Lipid	10885	-1.108	0.27	-??---	0.57	10847	-0.869	0.39	-??+--	0.79	10847	-0.756	0.45	-??---	0.85
		100004089	2-hydroxydecanoate	Lipid	11850	-1.108	0.27	-+?-+-	0.57	11811	-0.428	0.67	-+?-++	0.90	11810	-0.398	0.69	-+?-++	0.91
		100001315	p-cresol sulfate	Xenobiotics	13596	-1.105	0.27	++----	0.57	13556	-1.66	0.10	++----	0.48	13549	-1.279	0.20	++----	0.68
		512	taurine	Amino acid	10885	-1.104	0.27	-??+--	0.57	10847	-0.748	0.45	-??+--	0.82	10847	-0.978	0.33	-??---	0.79
		234	aspartate	Amino acid	12631	1.102	0.27	+?++++	0.57	12592	0.35	0.73	+?+---	0.92	12586	0.079	0.94	+?+---	0.98
		376	isoleucine	Amino acid	13596	-1.093	0.27	--+--+	0.58	13556	-1.042	0.30	+-+--+	0.71	13549	-1.622	0.10	+-+---	0.54
		100001552	1-eicosatrienoylglycerophosphocholine (20:3)*	Lipid	10767	1.09	0.28	?-?++?	0.58	10766	-0.416	0.68	?-?++?	0.90	10765	-0.413	0.68	?-?+-?	0.91
		100004575	N2,N5-diacetylornithine	Amino acid	5722	-1.083	0.28	-??+?-	0.58	5684	-0.395	0.69	-??+?-	0.90	5684	-0.2	0.84	-??+?-	0.95
		100009403	1-eicosapentaenoylglycerol (20:5)*	Lipid	5722	1.079	0.28	+??+?-	0.59	5684	1.355	0.18	+??+?-	0.59	5684	1.259	0.21	+??+?-	0.68
		1504	oleamide	Lipid	10401	1.069	0.29	???-++	0.59	10401	1.849	0.06	???-++	0.43	10401	1.83	0.07	???-++	0.48
		100009069	1-(1-enyl-palmitoyl)-2-linoleoyl-GPE (P-16:0/18:2)*	Lipid	10885	-1.069	0.28	-??--+	0.59	10847	-1.312	0.19	-??--+	0.61	10847	-1.013	0.31	-??--+	0.78

		100009021	1-palmityl-2-arachidonoyl-GPC (O-16:0/20:4)*	Lipid	9802	-1.072	0.28	???--?	0.59	9802	-1.025	0.31	???--?	0.72	9802	-0.967	0.33	???--?	0.79
		100008921	1-palmitoyl-2-stearoyl-GPC (16:0/18:0)	Lipid	10885	-1.072	0.28	-??-+-	0.59	10847	-0.933	0.35	-??-+-	0.77	10847	-0.583	0.56	-??-+-	0.87
		100001207	4-imidazoleacetate	Amino acid	5123	1.066	0.29	-??+??	0.59	5085	1.631	0.10	-??+??	0.49	5085	1.647	0.10	-??+??	0.53
		100004299	N-acetyl-1-methylhistidine*	Amino acid	10885	-1.065	0.29	-??--+	0.59	10847	-0.967	0.33	-??---	0.76	10847	-1.029	0.30	-??---	0.77
		100008957	sphingomyelin (d18:2/24:1, d18:1/24:2)*	Lipid	10885	1.058	0.29	-??++-	0.59	10847	0.882	0.38	-??++-	0.79	10847	1.061	0.29	-??+++	0.76
		100001843	gamma-glutamylalanine	Peptide	5722	1.052	0.29	+??+?+	0.60	5684	1.782	0.07	+??+?+	0.45	5684	2.011	0.04	+??+?+	0.41
		100000285	N-alpha-acetylornithine	Amino acid	1083	-1.054	0.29	-????-	0.60	1045	-1.179	0.24	-????-	0.66	1045	-1.752	0.08	-????-	0.50
		100001468	N1-Methyl-2-pyridone-5-carboxamide	Cofactors and Vitamins	13596	1.054	0.29	+--++-	0.60	13556	0.469	0.64	+--++-	0.89	13549	0.658	0.51	+--++-	0.87
		100002873	1-lignoceroyl-GPC (24:0)	Lipid	5722	-1.049	0.29	+??-?-	0.60	5684	-0.618	0.54	+??-?-	0.85	5684	-0.272	0.79	-??-?+	0.93
		100002029	androstenediol (3beta,17beta) monosulfate (2)	Lipid	10885	-1.045	0.30	-??+--	0.60	10847	-0.614	0.54	-??+--	0.85	10847	-0.779	0.44	-??+--	0.85
		100010925	palmitoyl-arachidonoyl-glycerol (16:0/20:4) [2]*	Lipid	6246	1.046	0.30	-???++	0.60	6208	0.191	0.85	-???+-	0.95	6208	0.2	0.84	-???+-	0.95
		100000657	1,2-dipalmitoyl-GPC (16:0/16:0)	Lipid	10885	1.041	0.30	-??++-	0.60	10847	1.308	0.19	-??++-	0.61	10847	1.675	0.09	-??+++	0.52
		1442	beta-hydroxyisovalerate	Amino acid	13596	-1.036	0.30	--+---	0.60	13556	-1.377	0.17	--+---	0.59	13549	-1.259	0.21	--+---	0.68
		100004182	3b-hydroxy-5-cholenoic acid	Lipid	1083	1.032	0.30	+????+	0.60	1045	1.159	0.25	+????+	0.66	1045	1.279	0.20	+????+	0.68
		100000998	citramalate	Amino acid	9802	1.04	0.30	???++?	0.60	9802	1.164	0.24	???++?	0.66	9802	1.091	0.28	???++?	0.75
		279	cystine	Amino acid	12631	1.035	0.30	+?-+-+	0.60	12592	0.681	0.50	+?-+-+	0.84	12586	0.708	0.48	+?-+-+	0.87
		100001279	hyocholate	Lipid	5722	-1.034	0.30	+??-?-	0.60	5684	-0.59	0.56	+??-?-	0.86	5684	-0.608	0.54	+??-?-	0.87
		100001557	2-linoleoylglycerophosphocholine*	Lipid	10767	-1.033	0.30	?+?--?	0.60	10766	-0.563	0.57	?+?--?	0.86	10765	-0.423	0.67	?+?--?	0.91
		917	asparagine	Amino acid	12631	-1.035	0.30	-?----	0.60	12592	-0.175	0.86	-?-++-	0.96	12586	0.16	0.87	-?-+++	0.96
		100002122	3-hydroxyhippurate	Xenobiotics	11850	-1.021	0.31	-+?---	0.61	11811	-1.325	0.19	++?---	0.60	11810	-1.197	0.23	++?---	0.71
		100001257	N-acetylasparagine	Amino acid	10885	1.013	0.31	-??+++	0.61	10847	1.336	0.18	-??+++	0.60	10847	1.496	0.13	-??+++	0.59
		100008955	tricosanoyl sphingomyelin (d18:1/23:0)*	Lipid	6246	-0.998	0.32	-???--	0.61	6208	-1.599	0.11	-???--	0.50	6208	-1.141	0.25	-???--	0.73
		100003260	carboxyethyl-GABA	Amino acid	6246	0.993	0.32	+???+-	0.61	6208	0.947	0.34	+???+-	0.77	6208	0.982	0.33	-???+-	0.79
		100001956	N-methylproline	Amino acid	11850	-1.001	0.32	-+?---	0.61	11811	-1.217	0.22	-+?---	0.65	11810	-0.957	0.34	-+?---	0.80
		100006260	6-hydroxyindole sulfate	Xenobiotics	10885	-1.011	0.31	+??---	0.61	10847	-1.128	0.26	+??---	0.67	10847	-0.873	0.38	+??---	0.82
		100010895	2'-O-methylcytidine	Nucleotide	6246	-1.002	0.32	-???+-	0.61	6208	-0.826	0.41	-???+-	0.81	6208	-0.854	0.39	-???+-	0.83
		100015745	glycosyl ceramide (d18:2/24:1, d18:1/24:2)*	Lipid	6246	-0.992	0.32	+???--	0.61	6208	-1.181	0.24	+???--	0.66	6208	-0.748	0.45	+???-+	0.85
		100001452	isovalerylglycine	Amino acid	10885	-1.009	0.31	-??-+-	0.61	10847	-0.864	0.39	-??-+-	0.79	10847	-0.691	0.49	-??-+-	0.87
		100009014	1-(1-enyl-palmitoyl)-2-arachidonoyl-GPC (P-16:0/20:4)*	Lipid	10885	-1.001	0.32	-??---	0.61	10847	-0.761	0.45	-??---	0.82	10847	-0.654	0.51	-??---	0.87
		302	deoxycholate	Lipid	12631	1.002	0.32	+?++-+	0.61	12592	0.591	0.55	+?+--+	0.86	12586	0.611	0.54	+?++++	0.87
		100009161	1-(1-enyl-palmitoyl)-2-myristoyl-GPC (P-16:0/14:0)*	Lipid	9802	-1.013	0.31	???--?	0.61	9802	-0.432	0.67	???+-?	0.90	9802	-0.608	0.54	???+-?	0.87
		100006290	sphingomyelin (d18:1/20:0, d16:1/22:0)*	Lipid	10885	1.011	0.31	-??++-	0.61	10847	0.431	0.67	-??+--	0.90	10847	0.585	0.56	+??+--	0.87
		100001586	gulonate*	Cofactors and Vitamins	10885	0.998	0.32	+??++-	0.61	10847	0.542	0.59	+??++-	0.87	10847	0.544	0.59	+??++-	0.89
		100010940	diacylglycerol (16:1/18:2 [2], 16:0/18:3 [1])*	Lipid	1083	1.003	0.32	+????+	0.61	1045	0.266	0.79	+????-	0.93	1045	0.495	0.62	+????-	0.90
		2050	eicosapentaenoate (EPA; 20:5n3)	Lipid	13596	-0.992	0.32	+--+--	0.61	13556	-0.58	0.56	+--+--	0.86	13549	-0.345	0.73	+-++--	0.92
		100006379	C-glycosyltryptophan	Amino acid	12631	1.006	0.31	+?++++	0.61	12592	0.113	0.91	+?+--+	0.97	12586	-0.171	0.86	+?++--	0.96
		100001275	phenylacetylglycine	Peptide	9802	0.999	0.32	???+-?	0.61	9802	-0.114	0.91	???+-?	0.97	9802	0.053	0.96	???+-?	0.99
		100001635	ectoine	Xenobiotics	6246	0.988	0.32	+???+-	0.61	6208	0.734	0.46	+???+-	0.83	6208	0.802	0.42	+???+-	0.84
		519	myristate (14:0)	Lipid	13596	-0.985	0.32	+-----	0.61	13556	-0.329	0.74	+-++--	0.92	13549	-0.224	0.82	+-++--	0.94
		100006294	behenoyl sphingomyelin (d18:1/22:0)*	Lipid	6246	-0.984	0.33	-???--	0.61	6208	-1.475	0.14	-???--	0.55	6208	-1.171	0.24	-???--	0.72
		100001541	2-hydroxy-3-methylvalerate	Amino acid	11850	-0.978	0.33	--?---	0.62	11811	-1.207	0.23	--?---	0.65	11810	-1.155	0.25	--?---	0.72
		100001129	O-acetylhomoserine	Amino acid	5238	0.975	0.33	???+?-	0.62	5238	1.134	0.26	???+?-	0.67	5238	1.3	0.19	???+?+	0.68
		100004083	glycohyocholate	Lipid	10885	-0.974	0.33	-??-+-	0.62	10847	-0.889	0.37	-??-+-	0.79	10847	-0.974	0.33	-??-+-	0.79
		100001033	beta-sitosterol	Lipid	5762	-0.966	0.33	????--	0.62	5762	-1.335	0.18	????--	0.60	5762	-1.024	0.31	????--	0.77
		100008994	1-stearoyl-2-linoleoyl-GPI (18:0/18:2)	Lipid	10885	0.965	0.33	-??++-	0.62	10847	0.624	0.53	-??++-	0.84	10847	0.957	0.34	-??++-	0.80
		339	glutarate (pentanedioate)	Lipid	10885	-0.967	0.33	+??--+	0.62	10847	-1.073	0.28	+??--+	0.70	10847	-0.884	0.38	+??--+	0.82
		100015786	sphingomyelin (d18:0/20:0, d16:0/22:0)*	Lipid	6246	0.968	0.33	+???++	0.62	6208	0.304	0.76	+???+-	0.92	6208	0.593	0.55	+???+-	0.87
		424	palmitate (16:0)	Lipid	13596	-0.96	0.34	+-----	0.62	13556	-0.105	0.92	+-++--	0.97	13549	-0.17	0.87	+--+--	0.96
		100001294	gamma-glutamylglycine	Peptide	10885	-0.959	0.34	+??---	0.62	10847	0.103	0.92	+??+--	0.97	10847	-0.029	0.98	+??+-+	0.99
		1239	2-hydroxystearate	Lipid	13596	-0.951	0.34	++----	0.63	13556	-1.171	0.24	++----	0.66	13549	-0.998	0.32	++---+	0.79
		100001229	stearidonate (18:4n3)	Lipid	12631	-0.949	0.34	+?+---	0.63	12592	-0.165	0.87	+?++-+	0.96	12586	-0.058	0.95	+?++-+	0.98
		100015882	glycosyl ceramide (d18:1/20:0, d16:1/22:0)*	Lipid	6246	-0.943	0.35	+???--	0.63	6208	-1.384	0.17	+???--	0.58	6208	-1.134	0.26	+???-+	0.73
		100015833	arachidoylcarnitine (C20)*	Lipid	6246	-0.938	0.35	+???--	0.63	6208	-0.417	0.68	+???+-	0.90	6208	-0.201	0.84	+???--	0.95
		342	glycocholate	Lipid	13596	-0.936	0.35	-+--++	0.63	13556	-1.157	0.25	----++	0.66	13549	-1.208	0.23	----++	0.70
		1102	gamma-glutamyltyrosine	Peptide	13596	-0.93	0.35	-++---	0.64	13556	-1.125	0.26	-++---	0.67	13549	-1.37	0.17	------	0.66
		100001651	2-oleoylglycerophosphoethanolamine*	NA	5604	0.931	0.35	?-?+??	0.64	5603	0.129	0.90	?-?+??	0.97	5602	0.049	0.96	?-?+??	0.99
		100006092	tyramine O-sulfate	Amino acid	10885	-0.927	0.35	-??-+-	0.64	10847	-1.256	0.21	-??---	0.63	10847	-1.459	0.14	-??---	0.61
		100004499	6-oxopiperidine-2-carboxylate	Amino acid	10885	0.927	0.35	+??+-+	0.64	10847	0.216	0.83	-??+-+	0.95	10847	0.247	0.80	+??+--	0.94
		100001334	N-acetylproline	Amino acid	5238	0.923	0.36	???+?+	0.64	5238	0.569	0.57	???+?+	0.86	5238	0.527	0.60	???+?+	0.89

		100009030	lactosyl-N-palmitoyl-sphingosine (d18:1/16:0)	Lipid	10885	-0.924	0.36	+??+--	0.64	10847	-0.154	0.88	+??+--	0.96	10847	-0.087	0.93	+??+-+	0.98
		100004295	2-piperidinone	Xenobiotics	11366	0.914	0.36	?+?+-+	0.64	11365	0.409	0.68	?+?+-+	0.90	11364	0.448	0.65	?+?+-+	0.91
		100000792	dehydroisoandrosterone sulfate (DHEA-S)	Lipid	13596	-0.914	0.36	+--+--	0.64	13556	-0.169	0.87	+-++--	0.96	13549	-0.373	0.71	+-++--	0.91
		136	cholate	Lipid	13596	-0.915	0.36	+++-+-	0.64	13556	-0.392	0.70	+++-+-	0.91	13549	-0.286	0.78	+++-+-	0.93
		100008919	1-(1-enyl-stearoyl)-2-oleoyl-GPE (P-18:0/18:1)	Lipid	6246	0.91	0.36	+???++	0.65	6208	0.308	0.76	+???-+	0.92	6208	0.293	0.77	+???-+	0.93
		100000551	4-methyl-2-oxopentanoate	Amino acid	13596	-0.89	0.37	--+---	0.66	13556	0.153	0.88	--+-+-	0.96	13549	0.098	0.92	--+++-	0.98
		100009045	phenylacetylglutamate	Peptide	1083	-0.886	0.38	-????-	0.66	1045	-0.887	0.37	-????-	0.79	1045	-0.703	0.48	-????-	0.87
		1492	linoleamide (18:2n6)	Lipid	5238	-0.885	0.38	???-?+	0.66	5238	-0.443	0.66	???-?+	0.90	5238	-0.481	0.63	???-?+	0.91
		100006056	N-formylphenylalanine	Amino acid	5238	0.882	0.38	???+?-	0.66	5238	0.756	0.45	???+?-	0.82	5238	0.632	0.53	???+?-	0.87
		100015846	nervonoylcarnitine (C24:1)*	Lipid	6246	0.874	0.38	-???+-	0.67	6208	1.605	0.11	+???+-	0.50	6208	1.772	0.08	+???+-	0.49
		1137	oleoyl ethanolamide	Lipid	10885	-0.875	0.38	+??---	0.67	10847	-0.257	0.80	+??---	0.93	10847	-0.441	0.66	+??---	0.91
		100000011	phenylacetate	Amino acid	5762	0.87	0.38	????+-	0.67	5762	0.15	0.88	????+-	0.96	5762	0.402	0.69	????+-	0.91
		100006171	eugenol sulfate	Xenobiotics	10885	-0.865	0.39	-??--+	0.67	10847	-0.528	0.60	-??-++	0.87	10847	-0.499	0.62	-??-++	0.90
		100008917	1-(1-enyl-stearoyl)-2-oleoyl-GPC (P-18:0/18:1)	Lipid	9802	-0.861	0.39	???--?	0.67	9802	-0.82	0.41	???--?	0.81	9802	-1.175	0.24	???--?	0.72
		100006293	sphingomyelin (d18:1/20:2, d18:2/20:1, d16:1/22:2)*	Lipid	6246	0.861	0.39	-???++	0.67	6208	0.718	0.47	-???++	0.83	6208	1.064	0.29	-???++	0.76
		181	laurate (12:0)	Lipid	13596	-0.863	0.39	+-----	0.67	13556	-0.365	0.71	+-+---	0.91	13549	-0.017	0.99	+++--+	0.99
		100010949	stearoyl-arachidonoyl-glycerol (18:0/20:4) [1]*	Lipid	1083	-0.858	0.39	-????-	0.67	1045	-1.047	0.30	-????-	0.71	1045	-1.066	0.29	-????-	0.76
		1021	5-oxoproline	Amino acid	13596	-0.858	0.39	+++---	0.67	13556	-0.422	0.67	++++--	0.90	13549	-0.247	0.80	++++--	0.94
		358	hypotaurine	Amino acid	10885	0.853	0.39	-??-+-	0.67	10847	1.439	0.15	-??-+-	0.55	10847	1.238	0.22	-??-++	0.68
		100001264	1-margaroylglycerophosphocholine (17:0)	Lipid	10767	-0.853	0.39	?-?--?	0.67	10766	-1.123	0.26	?-?--?	0.67	10765	-1.216	0.22	?+?--?	0.70
		100008904	1-stearoyl-2-oleoyl-GPC (18:0/18:1)	Lipid	10885	0.855	0.39	+??+++	0.67	10847	-0.283	0.78	+??---	0.92	10847	-0.554	0.58	+??---	0.88
		197	S-adenosylhomocysteine (SAH)	Amino acid	5123	0.855	0.39	+??+??	0.67	5085	-0.425	0.67	+??-??	0.90	5085	-0.197	0.84	+??-??	0.95
		100008920	sphingomyelin (d18:1/17:0, d17:1/18:0, d19:1/16:0)	Lipid	10885	-0.849	0.40	+??+--	0.68	10847	-1.073	0.28	+??+--	0.70	10847	-0.975	0.33	+??+--	0.79
		100001296	stachydrine	Xenobiotics	13596	-0.846	0.40	--+---	0.68	13556	-0.786	0.43	--+---	0.81	13549	-0.296	0.77	--+---	0.93
		100001386	heme	Cofactors and Vitamins	7834	-0.841	0.40	----??	0.68	7794	-0.841	0.40	----??	0.80	7787	-1.173	0.24	----??	0.72
		100002024	5alpha-androstan-3beta,17beta-diol monosulfate (2)	Lipid	10885	-0.841	0.40	-??-+-	0.68	10847	-0.503	0.61	-??-+-	0.88	10847	-0.647	0.52	-??-+-	0.87
		100001999	21-hydroxypregnenolone disulfate	Lipid	11850	-0.841	0.40	+-?---	0.68	11811	-0.115	0.91	+-?+--	0.97	11810	-0.454	0.65	+-?---	0.91
		100003119	N-oleoyltaurine	Lipid	1083	0.827	0.41	+????-	0.69	1045	0.809	0.42	+????-	0.81	1045	0.611	0.54	+????-	0.87
		100015788	sphingomyelin (d18:2/18:1)*	Lipid	6246	-0.826	0.41	-???+-	0.69	6208	-1.067	0.29	-???--	0.70	6208	-0.621	0.53	-???--	0.87
		798	adenosine	Nucleotide	1083	0.824	0.41	+????+	0.69	1045	1.157	0.25	+????+	0.66	1045	0.985	0.32	+????+	0.79
		100000447	gentisate	Amino acid	10885	-0.822	0.41	-??-+-	0.69	10847	-0.858	0.39	-??-+-	0.79	10847	-0.516	0.61	-??-+-	0.89
		100000584	2-arachidonoylglycerol (20:4)	Lipid	1083	-0.822	0.41	-????+	0.69	1045	-0.566	0.57	-????+	0.86	1045	-0.191	0.85	-????+	0.95
		100001232	5-dodecenoate (12:1n7)	Lipid	13596	-0.819	0.41	+-----	0.69	13556	-0.292	0.77	+--++-	0.92	13549	-0.206	0.84	---+++	0.95
		100000007	carnitine	Lipid	13596	0.808	0.42	-+++-+	0.70	13556	1.007	0.31	-+++-+	0.73	13549	0.488	0.63	-+++-+	0.90
		100001556	2-oleoylglycerophosphocholine*	Lipid	10767	0.809	0.42	?+?+-?	0.70	10766	0.221	0.83	?+?+-?	0.95	10765	0.059	0.95	?+?+-?	0.98
		100001274	N-acetylthreonine	Amino acid	13596	0.806	0.42	+++++-	0.70	13556	0.364	0.72	++++--	0.91	13549	0.388	0.70	++++--	0.91
		100002462	5-(galactosylhydroxy)-L-lysine	Amino acid	1083	-0.793	0.43	+????-	0.71	1045	-0.878	0.38	+????-	0.79	1045	-1.574	0.12	+????-	0.56
		100001398	3,7-dimethylurate	Xenobiotics	10885	-0.793	0.43	-??+--	0.71	10847	-0.949	0.34	-??+--	0.77	10847	-1.054	0.29	-??+--	0.76
		100006126	4-vinylguaiacol sulfate	Xenobiotics	10286	0.796	0.43	-??-+?	0.71	10248	0.198	0.84	+??-+?	0.95	10248	-0.615	0.54	-??-+?	0.87
		2029	azelate (nonanedioate)	Lipid	11850	-0.794	0.43	-+?-+-	0.71	11811	-0.545	0.59	-+?-+-	0.87	11810	-0.308	0.76	-+?-+-	0.93
		100001526	malonylcarnitine	Lipid	6246	0.789	0.43	+???++	0.71	6208	0.996	0.32	+???++	0.73	6208	0.911	0.36	+???++	0.82
		100004542	2-aminoheptanoate	Lipid	11850	-0.783	0.43	--?-++	0.71	11811	-0.665	0.51	--?-++	0.84	11810	-0.736	0.46	--?-++	0.86
		100001570	1-linoleoyl-GPE (18:2)*	Lipid	13596	0.784	0.43	+++---	0.71	13556	0.284	0.78	+++-+-	0.92	13549	0.618	0.54	+++--+	0.87
		100015839	dihomo-linoleoylcarnitine (C20:2)*	Lipid	6246	-0.782	0.43	-???--	0.71	6208	-0.556	0.58	+???--	0.86	6208	-0.325	0.75	+???--	0.92
		100010922	linoleoyl-arachidonoyl-glycerol (18:2/20:4) [1]*	Lipid	6246	0.781	0.44	-???++	0.71	6208	0.81	0.42	-???+-	0.81	6208	1.034	0.30	-???+-	0.77
		100001293	N-acetylhistidine	Amino acid	10885	0.776	0.44	-??-+-	0.71	10847	0.689	0.49	-??++-	0.84	10847	0.591	0.55	-??++-	0.87
		100001253	N-acetylglutamine	Amino acid	10885	-0.774	0.44	+??--+	0.71	10847	-0.709	0.48	+??--+	0.83	10847	-0.805	0.42	+??--+	0.84
		100009135	1-(1-enyl-stearoyl)-2-linoleoyl-GPC (P-18:0/18:2)*	Lipid	9802	-0.775	0.44	???+-?	0.71	9802	0.07	0.94	???+-?	0.97	9802	0.041	0.97	???+-?	0.99
		100004112	3-methyl catechol sulfate (1)	Xenobiotics	11850	-0.771	0.44	-+?---	0.72	11811	-0.584	0.56	++?---	0.86	11810	-1.529	0.13	++?---	0.58
		536	2'-deoxyuridine	Nucleotide	6246	-0.767	0.44	+???-+	0.72	6208	-0.279	0.78	+???-+	0.92	6208	-0.145	0.89	+???-+	0.96
		100009052	palmitoyl-linoleoyl-glycerol (16:0/18:2) [1]*	Lipid	10885	0.764	0.45	-??+++	0.72	10847	0.644	0.52	+??+-+	0.84	10847	0.756	0.45	+??+++	0.85
		158	5,6-dihydrothymine	Nucleotide	10885	-0.761	0.45	+??---	0.72	10847	-0.177	0.86	+??+--	0.96	10847	0.022	0.98	+??+-+	0.99
		240	3-(4-hydroxyphenyl)lactate	Amino acid	13596	-0.758	0.45	+++---	0.72	13556	-0.858	0.39	++++--	0.79	13549	-0.958	0.34	++++--	0.80
		100001734	N6-acetyllysine	Amino acid	10885	0.755	0.45	+??+--	0.72	10847	0.078	0.94	+??+--	0.97	10847	0.274	0.78	+??+--	0.93
		100009130	1-oleoyl-2-docosahexaenoyl-GPC (18:1/22:6)*	Lipid	10401	0.753	0.45	???++-	0.72	10401	1.039	0.30	???++-	0.71	10401	1.319	0.19	???++-	0.68
		1136	valerate	Lipid	10401	-0.75	0.45	???+--	0.73	10401	-0.752	0.45	???+--	0.82	10401	-0.846	0.40	???+--	0.83
		100000963	homocitrulline	Amino acid	12631	0.748	0.45	+?+-+-	0.73	12592	-0.048	0.96	+?+-+-	0.98	12586	0.026	0.98	+?+-+-	0.99
		100001743	tryptophan betaine	Amino acid	13596	-0.743	0.46	-++---	0.73	13556	0.339	0.73	-++++-	0.92	13549	0.311	0.76	-++++-	0.93
		100001777	1-oleoyl-GPI (18:1)*	Lipid	11850	0.741	0.46	++?++-	0.73	11811	-0.329	0.74	++?+--	0.92	11810	-0.418	0.68	++?---	0.91

		100001324	ADpSGEGDFXAEGGGVR*	Peptide	7992	-0.739	0.46	-?-?++	0.73	7953	-1.202	0.23	-?-?-+	0.65	7947	-1.483	0.14	-?-?--	0.60
		100005352	1-eicosenoylglycerophosphocholine (20:1n9)*	Lipid	10767	-0.735	0.46	?-?--?	0.73	10766	-0.493	0.62	?+?--?	0.88	10765	-0.583	0.56	?+?--?	0.87
		100009004	1-(1-enyl-stearoyl)-2-docosahexaenoyl-GPE (P-18:0/22:6)*	Lipid	9802	-0.735	0.46	???+-?	0.73	9802	0.341	0.73	???+-?	0.92	9802	0.644	0.52	???+-?	0.87
		100001092	trigonelline (N'-methylnicotinate)	Cofactors and Vitamins	11850	-0.736	0.46	++?---	0.73	11811	-0.293	0.77	++?---	0.92	11810	-0.315	0.75	++?---	0.93
		100000436	glycodeoxycholate	Lipid	11850	0.729	0.47	-+?--+	0.73	11811	0.524	0.60	-+?--+	0.87	11810	0.587	0.56	-+?--+	0.87
		100006116	methyl-4-hydroxybenzoate sulfate	Xenobiotics	10885	0.729	0.47	-??++-	0.73	10847	0.336	0.74	-??+--	0.92	10847	0.623	0.53	-??+--	0.87
		100016038	(N(1) + N(8))-acetylspermidine	Amino acid	5762	-0.728	0.47	????--	0.73	5762	-1.092	0.27	????--	0.70	5762	-1.269	0.20	????--	0.68
		1123	chenodeoxycholate	Lipid	10885	-0.725	0.47	+??---	0.73	10847	-0.232	0.82	+??---	0.94	10847	-0.324	0.75	+??---	0.92
		100001397	1,3,7-trimethylurate	Xenobiotics	10885	-0.711	0.48	+??---	0.74	10847	-1.44	0.15	+??---	0.55	10847	-1.436	0.15	+??---	0.63
		1254	glycerol	Lipid	12631	0.711	0.48	+?+--+	0.74	12592	1.148	0.25	+?++++	0.67	12586	1.109	0.27	+?++++	0.74
		100002152	andro steroid monosulfate C19H28O6S (1)*	Lipid	10885	-0.71	0.48	+??--+	0.74	10847	-0.556	0.58	+??--+	0.86	10847	-0.861	0.39	+??--+	0.83
		100001563	2-myristoylglycerophosphocholine*	NA	5604	-0.714	0.48	?-?-??	0.74	5603	-0.798	0.43	?-?-??	0.81	5602	-0.826	0.41	?-?-??	0.83
		100002107	palmitoyl sphingomyelin (d18:1/16:0)	Lipid	12631	-0.712	0.48	-?++--	0.74	12592	-0.674	0.50	-?++--	0.84	12586	-0.454	0.65	-?++--	0.91
		100006191	p-cresol-glucuronide*	Amino acid	10885	0.713	0.48	+??-+-	0.74	10847	-0.079	0.94	+??-+-	0.97	10847	-0.104	0.92	+??-+-	0.98
		100009335	dihomo-linolenoyl-choline	Lipid	10885	-0.706	0.48	-??++-	0.74	10847	-1.287	0.20	-??---	0.62	10847	-1.248	0.21	-??---	0.68
		100005403	etiocholanolone glucuronide	Lipid	10885	-0.706	0.48	-??---	0.74	10847	-0.641	0.52	-??---	0.84	10847	-0.833	0.40	-??---	0.83
		100001276	N-acetylisoleucine	Amino acid	5238	-0.703	0.48	???-?+	0.74	5238	-0.627	0.53	???-?+	0.84	5238	-0.822	0.41	???-?-	0.83
		100010896	2'-O-methyluridine	Nucleotide	1083	-0.696	0.49	+????-	0.75	1045	-0.581	0.56	+????-	0.86	1045	-0.803	0.42	+????-	0.84
		1082	N-acetylleucine	Amino acid	10885	0.695	0.49	+??+++	0.75	10847	0.721	0.47	+??+-+	0.83	10847	0.574	0.57	+??++-	0.88
		100015735	ceramide (d18:1/14:0, d16:1/16:0)*	Lipid	6246	0.695	0.49	+???+-	0.75	6208	-0.381	0.70	+???--	0.91	6208	-0.263	0.79	+???--	0.93
		100001621	glycerophosphoinositol*	Lipid	1083	0.693	0.49	+????-	0.75	1045	0.571	0.57	+????-	0.86	1045	1.011	0.31	+????-	0.78
		100001674	2-arachidonoylglycerophosphoethanolamine*	Lipid	10767	0.683	0.49	?-?+-?	0.75	10766	0.107	0.91	?-?+-?	0.97	10765	0.062	0.95	?-?+-?	0.98
		100001126	gamma-glutamylvaline	Peptide	13596	-0.672	0.50	+--+-+	0.75	13556	-1.399	0.16	+----+	0.57	13549	-1.808	0.07	+-----	0.49
		100002227	4-cholesten-3-one	Lipid	10286	-0.672	0.50	+??--?	0.75	10248	-1.562	0.12	+??--?	0.52	10248	-1.529	0.13	+??--?	0.58
		100001580	docosapentaenoate (n6 DPA; 22:5n6)	Lipid	13112	0.673	0.50	?---++	0.75	13110	0.38	0.70	?---+-	0.91	13103	0.45	0.65	?---++	0.91
		100002784	2-oxoarginine*	Amino acid	6246	0.676	0.50	+???+-	0.75	6208	0.305	0.76	+???+-	0.92	6208	0.358	0.72	+???+-	0.92
		100009007	1-(1-enyl-palmitoyl)-2-oleoyl-GPC (P-16:0/18:1)*	Lipid	10885	-0.675	0.50	+??---	0.75	10847	-0.33	0.74	+??-+-	0.92	10847	-0.262	0.79	+??---	0.93
		100015962	N-trimethyl 5-aminovalerate	Amino acid	7992	0.678	0.50	+?+?--	0.75	7953	0.188	0.85	+?+?-+	0.95	7947	0.021	0.98	+?+?--	0.99
		100001767	pyrraline	Xenobiotics	10885	-0.668	0.50	-??-+-	0.76	10847	-0.724	0.47	-??-+-	0.83	10847	-0.465	0.64	-??-+-	0.91
		100001300	alpha-hydroxyisovalerate	Amino acid	13596	-0.664	0.51	--+---	0.76	13556	-0.72	0.47	--+---	0.83	13549	-0.909	0.36	--+---	0.82
		100006292	sphingomyelin (d18:1/20:1, d18:2/20:0)*	Lipid	10885	0.659	0.51	-??+--	0.76	10847	0.641	0.52	-??+--	0.84	10847	0.719	0.47	-??+-+	0.86
		100001295	gamma-glutamyltryptophan	Peptide	6246	-0.657	0.51	+???--	0.76	6208	-0.17	0.86	+???--	0.96	6208	-0.265	0.79	+???--	0.93
		100001006	N-acetylglycine	Amino acid	12631	0.651	0.51	-?-++-	0.77	12592	1.732	0.08	-?-+++	0.47	12586	1.905	0.06	-?++++	0.46
		100001990	taurocholenate sulfate	Lipid	10401	-0.648	0.52	???--+	0.77	10401	0.038	0.97	???+-+	0.98	10401	-0.467	0.64	???+--	0.91
		100009000	1-(1-enyl-palmitoyl)-2-docosahexaenoyl-GPE (P-16:0/22:6)*	Lipid	9802	-0.644	0.52	???+-?	0.77	9802	0.429	0.67	???+-?	0.90	9802	0.757	0.45	???+-?	0.85
		100004523	N-delta-acetylornithine	Amino acid	13596	-0.642	0.52	---++-	0.77	13556	-0.724	0.47	---++-	0.83	13549	-0.433	0.66	--+++-	0.91
		100008956	sphingomyelin (d18:2/23:0, d18:1/23:1, d17:1/24:1)*	Lipid	6246	-0.634	0.53	-???+-	0.77	6208	-1.311	0.19	-???--	0.61	6208	-0.83	0.41	-???--	0.83
		100009025	sphingomyelin (d18:1/21:0, d17:1/22:0, d16:1/23:0)*	Lipid	6246	-0.636	0.52	+???+-	0.77	6208	-1.267	0.21	+???--	0.63	6208	-0.799	0.42	+???--	0.84
		100002129	pregnenolone sulfate	Lipid	11850	-0.635	0.53	+-?+--	0.77	11811	-0.25	0.80	+-?+--	0.94	11810	-0.599	0.55	+-?+--	0.87
		100001433	1-arachidonylglycerol (20:4)	Lipid	10885	-0.631	0.53	-??+--	0.77	10847	-0.789	0.43	-??+--	0.81	10847	-0.747	0.45	-??+--	0.85
		100001413	N4-acetylcytidine	Nucleotide	6612	0.631	0.53	--??+?	0.77	6573	0.224	0.82	--??+?	0.95	6572	0.213	0.83	--??+?	0.95
		100006082	4-hydroxychlorothalonil	Xenobiotics	10885	-0.628	0.53	-??+--	0.77	10847	-0.69	0.49	-??+--	0.84	10847	-0.445	0.66	-??+--	0.91
		100009332	arachidonoylcholine	Lipid	10885	-0.624	0.53	-??+--	0.78	10847	-0.881	0.38	-??+--	0.79	10847	-0.897	0.37	-??+--	0.82
		100001615	octadecanedioate	Lipid	7468	-0.624	0.53	+?--?+	0.78	7429	-0.021	0.98	+?--?+	0.99	7423	0.015	0.99	+?-+?+	0.99
		100001551	1-arachidonoyl-GPC (20:4n6)*	Lipid	13596	0.619	0.54	-+-++-	0.78	13556	0.466	0.64	-+-++-	0.89	13549	0.693	0.49	-+-++-	0.87
		100001948	succinylcarnitine (C4-DC)	Energy	13596	0.62	0.54	+-++++	0.78	13556	-0.044	0.96	---+--	0.98	13549	-0.265	0.79	---++-	0.93
		100003240	N-stearoyltaurine	Lipid	1083	0.616	0.54	+????-	0.78	1045	0.731	0.46	+????-	0.83	1045	0.806	0.42	+????-	0.84
		100005834	9-hydroxystearate	Lipid	5238	0.613	0.54	???-?+	0.78	5238	1.118	0.26	???+?+	0.68	5238	1.101	0.27	???+?+	0.75
		100006360	dopamine 4-sulfate	Amino acid	1083	0.612	0.54	+????-	0.78	1045	0.667	0.50	+????-	0.84	1045	0.583	0.56	+????-	0.87
		100004561	N-formylanthranilic acid	Amino acid	6246	-0.61	0.54	-???--	0.78	6208	-1.236	0.22	-???--	0.64	6208	-1.013	0.31	-???--	0.78
		100000463	indolelactate	Amino acid	13596	0.605	0.55	-++++-	0.78	13556	0.829	0.41	-++++-	0.81	13549	0.906	0.36	-++++-	0.82
		100006190	2-acetamidophenol sulfate	Xenobiotics	10885	0.606	0.54	+??++-	0.78	10847	0.377	0.71	+??++-	0.91	10847	0.827	0.41	+??++-	0.83
		100009225	1-(1-enyl-stearoyl)-2-linoleoyl-GPE (P-18:0/18:2)*	Lipid	10885	-0.603	0.55	-??-+-	0.78	10847	-0.893	0.37	-??---	0.79	10847	-0.697	0.49	-??--+	0.87
		564	threonine	Amino acid	13596	-0.604	0.55	-+++--	0.78	13556	0.39	0.70	-+++--	0.91	13549	0.371	0.71	-+++-+	0.91
		275	creatinine	Amino acid	13596	0.61	0.54	+--++-	0.78	13556	-0.192	0.85	+--++-	0.95	13549	0.232	0.82	+--++-	0.94
		1628	glycochenodeoxycholate	Lipid	13596	0.598	0.55	-++-+-	0.78	13556	0.696	0.49	-++-++	0.83	13549	0.422	0.67	-++---	0.91
		100001198	myristoleate (14:1n5)	Lipid	13596	-0.598	0.55	+-+---	0.78	13556	-0.135	0.89	+-+---	0.97	13549	-0.177	0.86	+-+--+	0.96
		100001417	phenylacetylglutamine	Peptide	13596	0.598	0.55	+++-+-	0.78	13556	-0.559	0.58	+++---	0.86	13549	-0.14	0.89	+++---	0.96
		100009162	1-(1-enyl-palmitoyl)-2-palmitoyl-GPC (P-16:0/16:0)*	Lipid	10885	0.596	0.55	+??+--	0.78	10847	0.446	0.66	+??+--	0.90	10847	0.656	0.51	+??+--	0.87

		100015789	sphingomyelin (d18:2/24:2)*	Lipid	6246	0.593	0.55	-???++	0.78	6208	0.585	0.56	-???++	0.86	6208	1.084	0.28	-???++	0.75
		100000961	homoarginine	Amino acid	10885	0.587	0.56	-??+++	0.78	10847	0.705	0.48	-??++-	0.83	10847	0.89	0.37	-??++-	0.82
		340	glycine	Amino acid	12631	-0.588	0.56	-?+---	0.78	12592	0.261	0.79	-?++-+	0.93	12586	0.435	0.66	-?++-+	0.91
		100008999	1-(1-enyl-stearoyl)-2-arachidonoyl-GPE (P-18:0/20:4)*	Lipid	10885	-0.59	0.56	-??--+	0.78	10847	-0.525	0.60	-??+--	0.87	10847	-0.395	0.69	-??+-+	0.91
		252	succinate	Energy	10885	-0.585	0.56	-??---	0.79	10847	-0.533	0.59	-??---	0.87	10847	-0.236	0.81	-??+--	0.94
		100001409	N1-methylinosine	Nucleotide	10885	0.583	0.56	-??+++	0.79	10847	-0.229	0.82	+??--+	0.94	10847	-0.502	0.62	+??---	0.90
		1489	palmitoyl ethanolamide	Lipid	6246	0.577	0.56	+???+-	0.79	6208	0.927	0.35	+???+-	0.77	6208	0.888	0.37	+???+-	0.82
		100001733	hexanoylglutamine	Lipid	6246	-0.574	0.57	+???--	0.79	6208	-0.532	0.60	+???--	0.87	6208	-0.655	0.51	+???--	0.87
		100000016	suberate (octanedioate)	Lipid	10885	-0.575	0.57	+??--+	0.79	10847	-0.325	0.75	+??--+	0.92	10847	-0.362	0.72	+??--+	0.92
		100000784	theanine	Xenobiotics	10286	0.574	0.57	-??+-?	0.79	10248	-0.323	0.75	-??+-?	0.92	10248	-0.341	0.73	-??+-?	0.92
		1134	urate	Nucleotide	13596	-0.566	0.57	--+--+	0.79	13556	-0.425	0.67	--+--+	0.90	13549	-0.78	0.44	---+--	0.85
		50	spermidine	Amino acid	6246	-0.566	0.57	+???--	0.79	6208	-0.716	0.47	-???--	0.83	6208	-0.49	0.62	-???--	0.90
		100006121	1-dihomo-linolenylglycerol (20:3)	Lipid	10885	0.571	0.57	-??+--	0.79	10847	-0.273	0.78	-??+--	0.93	10847	-0.212	0.83	-??+--	0.95
		100001108	3-methylxanthine	Xenobiotics	13596	0.566	0.57	-+++--	0.79	13556	-0.287	0.77	-+++--	0.92	13549	-0.197	0.84	-+++--	0.95
		100001768	N6-carboxymethyllysine	Carbohydrate	6246	-0.559	0.58	+???--	0.79	6208	-0.692	0.49	+???--	0.84	6208	-0.597	0.55	+???--	0.87
		62	12,13-DiHOME	Lipid	10401	-0.546	0.59	???-++	0.80	10401	-0.568	0.57	???-++	0.86	10401	-0.331	0.74	???-++	0.92
		235	2-hydroxyphenylacetate	Amino acid	1083	-0.543	0.59	+????-	0.80	1045	-0.835	0.40	+????-	0.81	1045	-1.289	0.20	+????-	0.68
		926	caproate (6:0)	Lipid	11366	-0.544	0.59	?+?+--	0.80	11365	-0.86	0.39	?+?+--	0.79	11364	-0.947	0.34	?+?+--	0.80
		100009227	1-linoleoyl-GPG (18:2)*	Lipid	6246	-0.533	0.59	-???-+	0.81	6208	-0.531	0.60	-???-+	0.87	6208	-0.343	0.73	-???-+	0.92
		100015752	glycosyl-N-(2-hydroxynervonoyl)-sphingosine (d18:1/24:1(2OH))*	Lipid	5762	-0.526	0.60	????-+	0.81	5762	-1.043	0.30	????-+	0.71	5762	-0.992	0.32	????-+	0.79
		100001590	isobutyrylglycine	Amino acid	10885	0.529	0.60	-??++-	0.81	10847	0.751	0.45	-??++-	0.82	10847	0.893	0.37	-??++-	0.82
		100001182	docosadienoate (22:2n6)	Lipid	6687	-0.527	0.60	+-?-?+	0.81	6648	0.791	0.43	+-?+?+	0.81	6647	0.682	0.50	+-?+?+	0.87
		100008998	gamma-tocopherol/beta-tocopherol	Cofactors and Vitamins	10885	-0.522	0.60	-??-++	0.81	10847	-0.851	0.39	-??--+	0.80	10847	-0.937	0.35	-??---	0.81
		100001566	1-docosahexaenoylglycerophosphocholine (22:6n3)*	Lipid	10767	0.52	0.60	?-?+-?	0.82	10766	0.998	0.32	?+?+-?	0.73	10765	1.159	0.25	?+?+-?	0.72
		100005383	1-methyl-2-piperidinecarboxylic acid	Xenobiotics	11366	-0.518	0.60	?-?-+-	0.82	11365	-0.812	0.42	?-?---	0.81	11364	-0.712	0.48	?+?---	0.86
		100000711	4-acetylphenol sulfate	Xenobiotics	10401	-0.509	0.61	???-+-	0.82	10401	-1.722	0.09	???-+-	0.47	10401	-1.757	0.08	???-+-	0.50
		100000008	benzoate	Xenobiotics	12147	0.507	0.61	??-++-	0.82	12146	0.683	0.49	??-++-	0.84	12140	0.759	0.45	??+++-	0.85
		100001562	2-palmitoyl-GPC (16:0)*	Lipid	13596	0.507	0.61	+--+--	0.82	13556	0.196	0.85	+--+--	0.95	13549	0.702	0.48	+--+-+	0.87
		1256	choline	Lipid	13596	-0.514	0.61	+--++-	0.82	13556	-0.8	0.42	---++-	0.81	13549	-0.679	0.50	---++-	0.87
		100009337	caffeic acid sulfate	Xenobiotics	9802	-0.513	0.61	???-+?	0.82	9802	-0.462	0.64	???-+?	0.89	9802	-0.37	0.71	???-+?	0.91
		1488	arachidonoyl ethanolamide	Lipid	1083	-0.508	0.61	-????-	0.82	1045	0.118	0.91	-????+	0.97	1045	-0.239	0.81	-????-	0.94
		100002067	pregn steroid monosulfate C21H34O5S*	Lipid	11850	-0.509	0.61	+-?+--	0.82	11811	-0.055	0.96	+-?++-	0.98	11810	-0.138	0.89	+-?+--	0.96
		100015791	sphingomyelin (d18:2/23:1)*	Lipid	6246	0.508	0.61	+???+-	0.82	6208	-0.295	0.77	+???--	0.92	6208	0.096	0.92	+???-+	0.98
		100009037	1-margaroyl-2-linoleoyl-GPC (17:0/18:2)*	Lipid	9802	-0.502	0.62	???+-?	0.82	9802	-0.636	0.52	???+-?	0.84	9802	-0.61	0.54	???+-?	0.87
		100001384	1-arachidoyl-GPC (20:0)	Lipid	9802	-0.503	0.61	???+-?	0.82	9802	-0.458	0.65	???+-?	0.89	9802	-0.612	0.54	???+-?	0.87
		1026	phosphoethanolamine	Lipid	10401	-0.501	0.62	???-+-	0.82	10401	0.605	0.55	???++-	0.85	10401	0.532	0.59	???++-	0.89
		100001577	N-acetylcitrulline	Amino acid	10885	0.501	0.62	+??-++	0.82	10847	0.517	0.61	+??-++	0.87	10847	0.433	0.66	+??-++	0.91
		100000808	cysteine s-sulfate	Amino acid	10401	0.505	0.61	???+++	0.82	10401	0.133	0.89	???-++	0.97	10401	-0.124	0.90	???--+	0.97
		100001359	aconitate [cis or trans]	Energy	5722	0.5	0.62	+??+?-	0.82	5684	0.016	0.99	+??-?-	0.99	5684	-0.07	0.94	+??-?-	0.98
		100001125	threonylphenylalanine	Peptide	1083	-0.497	0.62	-????-	0.82	1045	0.117	0.91	-????+	0.97	1045	0.583	0.56	-????+	0.87
		100001048	2-palmitoylglycerol (16:0)	Lipid	5722	0.497	0.62	+??+?+	0.82	5684	0.153	0.88	+??-?+	0.96	5684	0.348	0.73	+??+?+	0.92
		100006373	1,2,3-benzenetriol sulfate (1)	Xenobiotics	9802	0.494	0.62	???-+?	0.82	9802	0.873	0.38	???-+?	0.79	9802	0.813	0.42	???-+?	0.84
		100001624	3-(3-hydroxyphenyl)propionate	Xenobiotics	10885	-0.492	0.62	+??-+-	0.82	10847	-0.59	0.56	+??-+-	0.86	10847	-0.462	0.64	+??-+-	0.91
		100001925	cyclo(leu-pro)	NA	5604	-0.49	0.62	?+?-??	0.82	5603	0.163	0.87	?+?-??	0.96	5602	0.332	0.74	?+?+??	0.92
		100006614	adipoylcarnitine (C6-DC)	Lipid	10885	0.49	0.62	-??+-+	0.82	10847	0.098	0.92	-??+-+	0.97	10847	-0.154	0.88	-??+--	0.96
		100001579	2-hydroxypalmitate	Lipid	13596	-0.479	0.63	+--+--	0.83	13556	-0.563	0.57	+--+--	0.86	13549	-0.197	0.84	++-+--	0.95
		100009008	1-(1-enyl-palmitoyl)-2-docosahexaenoyl-GPC (P-16:0/22:6)*	Lipid	9802	-0.475	0.64	???+-?	0.83	9802	0.281	0.78	???+-?	0.92	9802	0.478	0.63	???+-?	0.91
		1342	3-methoxytyrosine	Amino acid	13596	-0.474	0.64	+++---	0.83	13556	0.198	0.84	+++--+	0.95	13549	0.338	0.74	+++--+	0.92
		100008916	1-stearoyl-2-docosahexaenoyl-GPC (18:0/22:6)	Lipid	10401	0.465	0.64	???+-+	0.84	10401	0.88	0.38	???+-+	0.79	10401	0.905	0.37	???+--	0.82
		100001620	glycerophosphoethanolamine	Lipid	10885	-0.46	0.65	+??---	0.84	10847	-0.96	0.34	+??---	0.76	10847	-0.724	0.47	+??---	0.86
		925	heptanoate (7:0)	Lipid	12032	-0.453	0.65	+?-+-?	0.84	11993	-0.656	0.51	+?-+-?	0.84	11987	-0.565	0.57	+?-+-?	0.88
		1518	N-palmitoyl-sphingosine (d18:1/16:0)	Lipid	10885	0.453	0.65	+??+++	0.84	10847	-0.497	0.62	+??+--	0.88	10847	-0.491	0.62	+??---	0.90
		100000299	xanthosine	Nucleotide	1083	0.453	0.65	+????-	0.84	1045	0.587	0.56	+????-	0.86	1045	0.369	0.71	+????-	0.91
		310	cystathionine	Amino acid	10885	0.454	0.65	+??-++	0.84	10847	0.337	0.74	+??+-+	0.92	10847	0.203	0.84	+??+-+	0.95
		100000827	1-palmitoylglycerol (16:0)	Lipid	13596	0.451	0.65	-+++--	0.84	13556	-0.09	0.93	-+++--	0.97	13549	-0.049	0.96	-+++--	0.99
		100006375	3-methoxycatechol sulfate (1)	Xenobiotics	10885	0.449	0.65	+??++-	0.84	10847	0.414	0.68	+??++-	0.90	10847	0.354	0.72	+??++-	0.92
		100002126	16a-hydroxy DHEA 3-sulfate	Lipid	10885	-0.443	0.66	+??+-+	0.85	10847	-0.156	0.88	+??+-+	0.96	10847	-0.368	0.71	+??--+	0.91
		980	pentadecanoate (15:0)	Lipid	7468	-0.44	0.66	+?+-?-	0.85	7429	0.063	0.95	+?++?-	0.97	7423	0.331	0.74	+?++?-	0.92
		100001051	1-methylhistidine	Amino acid	10885	-0.436	0.66	-??+--	0.85	10847	-0.712	0.48	-??+--	0.83	10847	-0.791	0.43	-??+--	0.85

		100002167	12-HETE	Lipid	12631	-0.437	0.66	-?----	0.85	12592	-0.632	0.53	-?-+--	0.84	12586	-0.617	0.54	-?---+	0.87
		100009334	palmitoloelycholine	Lipid	6246	-0.435	0.66	+???+-	0.85	6208	-0.979	0.33	+???--	0.75	6208	-0.666	0.51	+???--	0.87
		100006296	sphingomyelin (d18:1/22:2, d18:2/22:1, d16:1/24:2)*	Lipid	1083	-0.431	0.67	-????-	0.85	1045	-0.463	0.64	-????-	0.89	1045	0.537	0.59	-????+	0.89
		361	inosine	Nucleotide	12631	-0.432	0.67	+?+--+	0.85	12592	-0.478	0.63	+?+--+	0.89	12586	-0.454	0.65	+?+--+	0.91
		100001086	N-(2-furoyl)glycine	Xenobiotics	10885	-0.43	0.67	+??-+-	0.85	10847	-1.081	0.28	+??-+-	0.70	10847	-1.253	0.21	+??-+-	0.68
		100002769	argininate*	Amino acid	6246	-0.425	0.67	-???+-	0.85	6208	-0.721	0.47	-???+-	0.83	6208	-0.571	0.57	-???+-	0.88
		1099	guanosine	Nucleotide	12631	0.424	0.67	+?+--+	0.85	12592	0.514	0.61	+?+--+	0.87	12586	0.446	0.66	+?+--+	0.91
		100001269	campesterol	Lipid	6246	-0.418	0.68	-???+-	0.85	6208	-0.742	0.46	-???--	0.83	6208	-0.62	0.54	-???--	0.87
		100006184	2-methoxyresorcinol sulfate	Xenobiotics	9802	0.418	0.68	???-+?	0.85	9802	0.204	0.84	???-+?	0.95	9802	0.029	0.98	???-+?	0.99
		100003892	lanthionine	Xenobiotics	10885	0.416	0.68	-??-++	0.85	10847	0.436	0.66	-??-++	0.90	10847	0.622	0.53	-??-++	0.87
		100005351	1-eicosapentaenoylglycerophosphocholine (20:5n3)*	Lipid	10767	-0.414	0.68	?-?+-?	0.85	10766	-0.093	0.93	?-?+-?	0.97	10765	-0.147	0.88	?-?+-?	0.96
		1648	taurocholate	Lipid	10885	-0.412	0.68	-??-+-	0.85	10847	-0.703	0.48	-??-+-	0.83	10847	-0.719	0.47	-??-+-	0.86
		179	9,10-DiHOME	Lipid	10885	-0.413	0.68	-??-+-	0.85	10847	-0.288	0.77	-??-++	0.92	10847	-0.09	0.93	-??-++	0.98
		2053	tricarballylate	Energy	9802	0.408	0.68	???++?	0.86	9802	0.878	0.38	???++?	0.79	9802	0.789	0.43	???++?	0.85
		100000096	4-guanidinobutanoate	Amino acid	10885	0.406	0.68	+??-+-	0.86	10847	0.118	0.91	+??-+-	0.97	10847	0.261	0.79	+??-+-	0.93
		171	hypoxanthine	Nucleotide	13596	-0.402	0.69	+----+	0.86	13556	-0.464	0.64	-----+	0.89	13549	-0.619	0.54	-----+	0.87
		460	phenylalanine	Amino acid	13596	-0.4	0.69	+-++--	0.86	13556	-0.21	0.83	--++--	0.95	13549	-0.461	0.65	+-++--	0.91
		100001263	1-palmitoyl-GPC (16:0)	Lipid	13596	0.395	0.69	+--++-	0.86	13556	-0.114	0.91	---++-	0.97	13549	0.439	0.66	---++-	0.91
		100000036	3-methyl-2-oxovalerate	Amino acid	13596	-0.394	0.69	--+--+	0.86	13556	0.161	0.87	+-+--+	0.96	13549	-0.103	0.92	+-+---	0.98
		100000263	imidazole lactate	Amino acid	10885	-0.391	0.70	-??+--	0.86	10847	-0.475	0.63	-??+--	0.89	10847	-0.367	0.71	-??+--	0.91
		445	orotate	Nucleotide	10401	0.391	0.70	???+--	0.86	10401	0.267	0.79	???+--	0.93	10401	0.334	0.74	???+--	0.92
		272	corticosterone	Lipid	1083	0.386	0.70	+????+	0.87	1045	0.431	0.67	+????+	0.90	1045	0.826	0.41	+????+	0.83
		100004329	sphingomyelin (d18:2/16:0, d18:1/16:1)*	Lipid	10885	0.38	0.70	-??+--	0.87	10847	0.028	0.98	-??+--	0.99	10847	0.314	0.75	-??+--	0.93
		100006374	1,2,3-benzenetriol sulfate (2)	Xenobiotics	10885	0.377	0.71	+??++-	0.87	10847	0.297	0.77	+??-+-	0.92	10847	0.207	0.84	+??-+-	0.95
		100001412	N2,N2-dimethylguanosine	Nucleotide	11850	-0.373	0.71	+-?-++	0.87	11811	-0.721	0.47	+-?--+	0.83	11810	-0.566	0.57	+-?---	0.88
		100005986	sphingomyelin (d18:1/24:1, d18:2/24:0)*	Lipid	6246	-0.369	0.71	-???--	0.88	6208	-0.89	0.37	-???--	0.79	6208	-0.639	0.52	-???-+	0.87
		100001193	adrenate (22:4n6)	Lipid	13596	-0.36	0.72	+-++-+	0.88	13556	-0.166	0.87	++++-+	0.96	13549	-0.367	0.71	++++--	0.91
		1001	trans-4-hydroxyproline	Amino acid	13596	-0.354	0.72	-+--++	0.88	13556	-0.438	0.66	-+--++	0.90	13549	-0.677	0.50	-+--+-	0.87
		828	arabinose	Carbohydrate	10401	-0.354	0.72	???-++	0.88	10401	-0.066	0.95	???-++	0.97	10401	-0.038	0.97	???-++	0.99
		100002528	sulfate*	Xenobiotics	10885	0.348	0.73	-??++-	0.88	10847	0.069	0.95	-??++-	0.97	10847	0.244	0.81	-??++-	0.94
		100001739	dihomo-linolenate (20:3n3 or n6)	Lipid	13596	0.348	0.73	--++--	0.88	13556	-0.121	0.90	--++--	0.97	13549	-0.137	0.89	--++--	0.96
		100009035	1-pentadecanoyl-2-linoleoyl-GPC (15:0/18:2)*	Lipid	9802	0.35	0.73	???-+?	0.88	9802	0.028	0.98	???-+?	0.99	9802	0.066	0.95	???-+?	0.98
		100015790	sphingomyelin (d18:2/21:0, d16:2/23:0)*	Lipid	6246	-0.344	0.73	+???+-	0.88	6208	-0.985	0.32	+???--	0.74	6208	-0.606	0.54	+???--	0.87
		100000015	xanthurenate	Amino acid	10885	-0.344	0.73	-??++-	0.88	10847	0.18	0.86	-??++-	0.96	10847	0.374	0.71	-??++-	0.91
		100009233	palmitoylcholine	Lipid	10885	-0.34	0.73	-??+--	0.89	10847	-0.796	0.43	-??+--	0.81	10847	-0.717	0.47	-??+--	0.86
		100001431	1-pentadecanoylglycerol (15:0)	Lipid	5722	-0.336	0.74	-??-?+	0.89	5684	-0.307	0.76	-??-?+	0.92	5684	-0.303	0.76	-??-?+	0.93
		100001272	1-oleoyl-GPC (18:1)	Lipid	13596	-0.33	0.74	+--+--	0.89	13556	-0.923	0.36	+-----	0.77	13549	-0.545	0.59	++----	0.89
		100000580	1,5-anhydroglucitol (1,5-AG)	Carbohydrate	13596	-0.327	0.74	--+++-	0.89	13556	0.291	0.77	--++++	0.92	13549	-0.271	0.79	--+--+	0.93
		100000943	2-oleoylglycerol (18:1)	Lipid	10286	-0.324	0.75	+??+-?	0.89	10248	-0.817	0.41	+??+-?	0.81	10248	-0.822	0.41	+??--?	0.83
		100004326	3-acetylphenol sulfate	Xenobiotics	6246	-0.313	0.75	-???+-	0.90	6208	-0.692	0.49	+???--	0.84	6208	-1.085	0.28	-???--	0.75
		100006361	dopamine 3-O-sulfate	Amino acid	10885	0.314	0.75	+??--+	0.90	10847	0.175	0.86	+??--+	0.96	10847	0.517	0.61	+??--+	0.89
		100005466	N-acetyltaurine	Amino acid	10885	0.317	0.75	+??-++	0.90	10847	0.056	0.96	+??--+	0.98	10847	-0.457	0.65	+??---	0.91
		1261	12-HHTrE	Lipid	1083	-0.312	0.76	-????+	0.90	1045	-0.165	0.87	-????+	0.96	1045	0.373	0.71	-????+	0.91
		1218	acetoacetate	Lipid	10401	0.312	0.75	???-+-	0.90	10401	0.344	0.73	???-+-	0.92	10401	0.311	0.76	???-+-	0.93
		100000269	glycerophosphorylcholine (GPC)	Lipid	13596	-0.317	0.75	-+-++-	0.90	13556	-0.32	0.75	-+--+-	0.92	13549	0.266	0.79	-++-+-	0.93
		100001104	N-acetyltyrosine	Amino acid	10885	0.313	0.75	-??+++	0.90	10847	0.073	0.94	-??-++	0.97	10847	0.19	0.85	-??-+-	0.95
		537	trans-urocanate	Amino acid	6246	-0.315	0.75	+???--	0.90	6208	0.045	0.96	+???--	0.98	6208	0.078	0.94	+???-+	0.98
		1052	glycerate	Carbohydrate	12631	-0.309	0.76	+?+---	0.90	12592	-0.045	0.96	+?++--	0.98	12586	0.346	0.73	+?++--	0.92
		100015640	N-palmitoylserine	Lipid	5762	-0.307	0.76	????--	0.90	5762	-0.65	0.52	????--	0.84	5762	-0.593	0.55	????-+	0.87
		100002911	glycoursodeoxycholate	Lipid	11850	0.306	0.76	-+?-+-	0.90	11811	0.809	0.42	-+?-++	0.81	11810	0.579	0.56	-+?-++	0.87
		100001435	1-linolenoylglycerol (18:3)	Lipid	10885	0.304	0.76	+??-++	0.90	10847	0.006	1.00	+??-++	1.00	10847	0.127	0.90	+??-++	0.97
		1135	ursodeoxycholate	Lipid	10885	0.297	0.77	+??-++	0.90	10847	0.818	0.41	+??+++	0.81	10847	0.774	0.44	+??+++	0.85
		100003594	phenylalanyltryptophan	NA	5604	0.297	0.77	?-?+??	0.90	5603	0.597	0.55	?-?+??	0.86	5602	0.738	0.46	?-?+??	0.86
		100015744	ceramide (d18:2/24:1, d18:1/24:2)*	Lipid	6246	0.3	0.76	+???++	0.90	6208	-0.369	0.71	-???-+	0.91	6208	-0.289	0.77	-???-+	0.93
		1141	4-hydroxyphenylpyruvate	Amino acid	10885	-0.298	0.77	+??+--	0.90	10847	-0.384	0.70	+??+--	0.91	10847	-0.215	0.83	+??+--	0.95
		100001612	N-acetyl-aspartyl-glutamate (NAAG)	Amino acid	10401	-0.292	0.77	???-++	0.90	10401	0.07	0.94	???-++	0.97	10401	0.076	0.94	???-++	0.98
		100000924	1-oleoylglycerol (18:1)	Lipid	13596	0.284	0.78	++-++-	0.91	13556	-0.785	0.43	++----	0.81	13549	-1.024	0.31	++----	0.77
		100000445	theobromine	Xenobiotics	13596	-0.284	0.78	-+-+--	0.91	13556	-0.809	0.42	-+-+--	0.81	13549	-0.606	0.54	-+-+--	0.87
		100001170	3-hydroxy-2-ethylpropionate	Amino acid	10885	0.285	0.78	+??+--	0.91	10847	-0.192	0.85	+??+--	0.95	10847	0.103	0.92	+??+--	0.98

		100006108	phenylacetylcarnitine	Peptide	10885	0.282	0.78	+??-++	0.91	10847	-0.532	0.59	+??---	0.87	10847	-0.302	0.76	+??---	0.93
		100001876	sphinganine-1-phosphate	Lipid	6246	-0.279	0.78	-???+-	0.91	6208	-0.696	0.49	-???--	0.83	6208	-0.721	0.47	-???--	0.86
		432	nicotinamide	Cofactors and Vitamins	11850	0.274	0.78	-+?+++	0.91	11811	0.278	0.78	-+?+++	0.92	11810	0.391	0.70	-+?+++	0.91
		100009331	oleoylcholine	Lipid	10885	-0.271	0.79	-??+--	0.91	10847	-0.883	0.38	-??+--	0.79	10847	-0.942	0.35	-??---	0.80
		100009002	1-(1-enyl-palmitoyl)-2-arachidonoyl-GPE (P-16:0/20:4)*	Lipid	10885	-0.272	0.79	-??+-+	0.91	10847	-0.422	0.67	-??--+	0.90	10847	-0.17	0.86	-??+-+	0.96
		1114	3-aminoisobutyrate	Nucleotide	10885	0.267	0.79	-??++-	0.91	10847	0.674	0.50	-??++-	0.84	10847	0.782	0.43	-??++-	0.85
		297	sphingosine	Lipid	11850	0.268	0.79	++?-++	0.91	11811	-0.054	0.96	--?+-+	0.98	11810	-0.193	0.85	--?+--	0.95
		452	palmitoleate (16:1n7)	Lipid	13596	0.264	0.79	++---+	0.92	13556	0.94	0.35	++++++	0.77	13549	0.744	0.46	++++++	0.85
		100010923	linoleoyl-arachidonoyl-glycerol (18:2/20:4) [2]*	Lipid	1083	-0.254	0.80	-????+	0.92	1045	-0.056	0.96	-????+	0.98	1045	-0.489	0.62	-????+	0.90
		100005350	1-linolenoyl-GPC (18:3)*	Lipid	11850	-0.249	0.80	+-?---	0.92	11811	-0.905	0.37	+-?---	0.79	11810	-0.762	0.45	++?--+	0.85
		100002952	docosadioate	Lipid	6246	-0.247	0.80	-???--	0.93	6208	0.114	0.91	-???+-	0.97	6208	-0.006	1.00	-???+-	1.00
		194	N-formylmethionine	Amino acid	11850	-0.246	0.81	+-?-+-	0.93	11811	-0.318	0.75	--?-+-	0.92	11810	-0.224	0.82	--?-+-	0.94
		100005389	ferulic acid 4-sulfate	Xenobiotics	9802	0.242	0.81	???-+?	0.93	9802	-0.372	0.71	???-+?	0.91	9802	-0.212	0.83	???-+?	0.95
		100015623	lactosyl-N-behenoyl-sphingosine (d18:1/22:0)*	Lipid	6246	0.24	0.81	+???+-	0.93	6208	-0.083	0.93	+???+-	0.97	6208	0.098	0.92	+???-+	0.98
		100015609	N-palmitoyl-sphingadienine (d18:2/16:0)*	Lipid	6246	0.244	0.81	-???+-	0.93	6208	0.029	0.98	-???+-	0.99	6208	0.096	0.92	-???+-	0.98
		100003926	3-hydroxybutyrylcarnitine (1)	Lipid	11850	0.23	0.82	++?-++	0.93	11811	0.686	0.49	++?-++	0.84	11810	0.537	0.59	++?-++	0.89
		100009343	1-linoleoyl-2-linolenoyl-GPC (18:2/18:3)*	Lipid	10885	-0.226	0.82	+??-+-	0.93	10847	0.012	0.99	+??-+-	0.99	10847	0.417	0.68	+??-++	0.91
		826	xylose	Carbohydrate	6246	0.223	0.82	+???-+	0.93	6208	0.091	0.93	+???-+	0.97	6208	0.1	0.92	+???--	0.98
		100015793	sphingomyelin (d17:2/16:0, d18:2/15:0)*	Lipid	6246	0.221	0.82	+???+-	0.94	6208	-0.34	0.73	-???+-	0.92	6208	0.057	0.95	+???+-	0.98
		100003686	N-palmitoylglycine	Lipid	10885	-0.21	0.83	+??--+	0.94	10847	0.849	0.40	+??+-+	0.80	10847	0.892	0.37	+??+-+	0.82
		100010955	perfluorooctanesulfonic acid (PFOS)	Xenobiotics	6246	-0.215	0.83	-???--	0.94	6208	0.129	0.90	-???+-	0.97	6208	0.339	0.73	-???++	0.92
		100015787	sphingomyelin (d18:1/19:0, d19:1/18:0)*	Lipid	6246	0.211	0.83	+???+-	0.94	6208	-0.486	0.63	+???--	0.88	6208	-0.249	0.80	+???--	0.94
		100002927	S-methylcysteine sulfoxide	Amino acid	6246	0.207	0.84	+???+-	0.94	6208	0.731	0.47	+???+-	0.83	6208	0.951	0.34	+???+-	0.80
		338	gluconate	Xenobiotics	10885	-0.208	0.84	+??+-+	0.94	10847	-1.195	0.23	+??---	0.65	10847	-0.905	0.37	+??+--	0.82
		100002417	2,3-dihydroxyisovalerate	Xenobiotics	10885	-0.206	0.84	+??+--	0.94	10847	0.562	0.57	+??++-	0.86	10847	0.622	0.53	+??++-	0.87
		563	glutamine	Amino acid	13596	-0.2	0.84	-+-+-+	0.94	13556	0.742	0.46	-+-+-+	0.83	13549	0.953	0.34	-+-+-+	0.80
		100002173	1-pentadecanoylglycerophosphocholine (15:0)*	Lipid	10767	0.198	0.84	?-?++?	0.94	10766	-0.453	0.65	?-?+-?	0.89	10765	-0.389	0.70	?-?+-?	0.91
		100006115	arabonate/xylonate	Carbohydrate	10885	0.202	0.84	+??-++	0.94	10847	-0.087	0.93	+??-++	0.97	10847	-0.052	0.96	+??-+-	0.99
		100000840	tartronate (hydroxymalonate)	Xenobiotics	10885	0.193	0.85	+??++-	0.94	10847	0.183	0.85	+??++-	0.96	10847	0.499	0.62	+??++-	0.90
		144	4-hydroxyphenylacetate	Amino acid	5238	-0.194	0.85	???-?+	0.94	5238	-0.319	0.75	???-?+	0.92	5238	-0.363	0.72	???-?-	0.92
		100006370	3beta-hydroxy-5-cholestenoate	Lipid	6246	0.194	0.85	-???++	0.94	6208	0.506	0.61	-???++	0.88	6208	0.117	0.91	-???-+	0.97
		100001396	7-methylxanthine	Xenobiotics	13596	-0.17	0.86	-+++--	0.95	13556	-0.666	0.51	-+++--	0.84	13549	-1.026	0.30	-++---	0.77
		100001322	ADSGEGDFXAEGGGVR*	Peptide	7992	0.174	0.86	+?-?-+	0.95	7953	-0.091	0.93	+?+?-+	0.97	7947	-0.858	0.39	+?-?-+	0.83
		100001415	N6-carbamoylthreonyladenosine	Nucleotide	11850	-0.169	0.87	++?-+-	0.95	11811	-0.777	0.44	++?---	0.81	11810	-0.839	0.40	++?---	0.83
		100001323	DSGEGDFXAEGGGVR*	Peptide	13596	-0.179	0.86	---+++	0.95	13556	-0.432	0.67	---+-+	0.90	13549	-0.803	0.42	---+--	0.84
		100009160	1-(1-enyl-palmitoyl)-2-palmitoleoyl-GPC (P-16:0/16:1)*	Lipid	10885	-0.176	0.86	+??-+-	0.95	10847	-0.284	0.78	+??-+-	0.92	10847	-0.286	0.77	+??-++	0.93
		344	guanidinoacetate	Amino acid	10885	-0.169	0.87	+??+--	0.95	10847	0.183	0.86	+??+--	0.96	10847	0.25	0.80	+??+-+	0.94
		100001456	7-methylguanine	Nucleotide	12631	0.17	0.86	+?+-++	0.95	12592	0.338	0.74	+?+-++	0.92	12586	0.147	0.88	+?+-++	0.96
		100006295	sphingomyelin (d18:1/22:1, d18:2/22:0, d16:1/24:1)*	Lipid	10885	-0.169	0.87	-??++-	0.95	10847	-0.361	0.72	-??+--	0.91	10847	-0.125	0.90	-??+--	0.97
		100000258	glycerol 3-phosphate	Lipid	12631	-0.174	0.86	+?++--	0.95	12592	0.017	0.99	+?-++-	0.99	12586	0.128	0.90	+?+++-	0.97
		100009038	myristoyl dihydrosphingomyelin (d18:0/14:0)*	Lipid	6246	0.172	0.86	+???+-	0.95	6208	-0.416	0.68	+???--	0.90	6208	0.025	0.98	+???--	0.99
		100009272	glycosyl-N-palmitoyl-sphingosine (d18:1/16:0)	Lipid	10885	-0.162	0.87	+??+--	0.95	10847	-0.874	0.38	+??+--	0.79	10847	-0.852	0.39	+??--+	0.83
		100003397	trimethylamine N-oxide	Lipid	10885	0.162	0.87	-??+-+	0.95	10847	-0.637	0.52	-??+--	0.84	10847	-0.507	0.61	-??+--	0.90
		100001765	3-methyladipate	Lipid	1083	-0.161	0.87	+????-	0.95	1045	-0.011	0.99	+????-	0.99	1045	0.157	0.88	+????-	0.96
		100001993	pregnen-diol disulfate C21H34O8S2*	Lipid	11850	0.151	0.88	++?-+-	0.95	11811	0.752	0.45	++?-+-	0.82	11810	0.502	0.62	++?-+-	0.90
		100009026	behenoyl dihydrosphingomyelin (d18:0/22:0)*	Lipid	6246	0.154	0.88	-???++	0.95	6208	-0.452	0.65	-???--	0.89	6208	-0.275	0.78	-???--	0.93
		100009407	pimeloylcarnitine/3-methyladipoylcarnitine (C7-DC)	Lipid	10885	-0.154	0.88	-??+--	0.95	10847	0.285	0.78	-??+--	0.92	10847	0.263	0.79	-??+-+	0.93
		100004552	1-eicosapentaenoylglycerophosphoethanolamine*	Lipid	10767	0.151	0.88	?-?+-?	0.95	10766	-0.128	0.90	?-?+-?	0.97	10765	-0.263	0.79	?-?+-?	0.93
		827	cytidine	Nucleotide	6246	-0.151	0.88	-???++	0.95	6208	-0.131	0.90	-???++	0.97	6208	-0.196	0.84	-???++	0.95
		100001399	1,7-dimethylurate	Xenobiotics	11850	-0.149	0.88	++?+--	0.95	11811	-0.594	0.55	++?+--	0.86	11810	-0.478	0.63	++?+--	0.91
		461	phosphate	Energy	13596	0.147	0.88	-++---	0.95	13556	-0.228	0.82	-++---	0.94	13549	-0.332	0.74	-++---	0.92
		100001731	indoleacetylglutamine	Amino acid	6246	0.142	0.89	+???+-	0.96	6208	-0.188	0.85	+???+-	0.95	6208	-0.155	0.88	+???+-	0.96
		100002500	formiminoglutamate	Amino acid	5762	-0.135	0.89	????-+	0.96	5762	-0.5	0.62	????-+	0.88	5762	-1.007	0.31	????--	0.78
		825	uracil	Nucleotide	10885	-0.134	0.89	+??--+	0.96	10847	-0.38	0.70	+??--+	0.91	10847	-0.173	0.86	+??--+	0.96
		100000626	sphingosine 1-phosphate	Lipid	10885	0.132	0.89	-??+--	0.96	10847	-0.099	0.92	-??+--	0.97	10847	-0.223	0.82	-??+--	0.94
		100001327	HWESASXX*	Peptide	3195	-0.131	0.90	+-+???	0.96	3155	-0.499	0.62	+-+???	0.88	3148	-0.599	0.55	+-+???	0.87
		100001757	thymol sulfate	Xenobiotics	10885	0.123	0.90	-??++-	0.96	10847	0.676	0.50	-??++-	0.84	10847	0.63	0.53	-??++-	0.87
		100000787	N-acetylaspartate (NAA)	Amino acid	10885	0.122	0.90	-??-+-	0.96	10847	0.359	0.72	-??-+-	0.91	10847	0.681	0.50	-??-++	0.87
		330	fumarate	Energy	10885	0.125	0.90	+??-+-	0.96	10847	0.309	0.76	+??-+-	0.92	10847	0.49	0.62	+??-++	0.90

		100001266	N-acetylarginine	Amino acid	10885	0.122	0.90	-??++-	0.96	10847	0.282	0.78	-??++-	0.92	10847	0.335	0.74	-??++-	0.92
		100006642	glycodeoxycholate sulfate	Lipid	10885	-0.119	0.91	-??-++	0.96	10847	-0.331	0.74	-??--+	0.92	10847	-0.318	0.75	-??-+-	0.92
		100001485	gamma-glutamylisoleucine*	Peptide	11850	-0.111	0.91	+-?--+	0.97	11811	-0.751	0.45	+-?--+	0.82	11810	-0.953	0.34	+-?---	0.80
		100000054	5-hydroxylysine	Amino acid	10885	-0.11	0.91	+??-++	0.97	10847	-0.166	0.87	+??-++	0.96	10847	-0.311	0.76	+??-+-	0.93
		363	myo-inositol	Lipid	12631	-0.113	0.91	-?++--	0.97	12592	-0.2	0.84	-?++--	0.95	12586	0.279	0.78	-?++--	0.93
		132	3-phosphoglycerate	Carbohydrate	9802	0.104	0.92	???+-?	0.97	9802	0.192	0.85	???+-?	0.95	9802	0.175	0.86	???+-?	0.96
		100001481	1-docosahexaenoylglycerol (22:6)	Lipid	10885	0.101	0.92	+??+-+	0.97	10847	0.562	0.57	+??+-+	0.86	10847	0.661	0.51	+??+-+	0.87
		100001408	2-myristoylglycerol (14:0)	Lipid	5123	0.102	0.92	+??-??	0.97	5085	0.054	0.96	+??-??	0.98	5085	0.087	0.93	+??-??	0.98
		100004322	2-aminophenol sulfate	Xenobiotics	10885	-0.099	0.92	+??++-	0.97	10847	-0.213	0.83	+??-+-	0.95	10847	0.292	0.77	+??-+-	0.93
		100000707	maleate	Lipid	10885	0.089	0.93	+??--+	0.97	10847	-0.202	0.84	+??--+	0.95	10847	-0.452	0.65	+??--+	0.91
		100009005	1-(1-enyl-palmitoyl)-2-oleoyl-GPE (P-16:0/18:1)*	Lipid	10885	-0.094	0.93	+??-++	0.97	10847	-0.504	0.61	-??--+	0.88	10847	-0.427	0.67	-??--+	0.91
		1668	taurodeoxycholate	Lipid	11850	0.092	0.93	--?+-+	0.97	11811	-0.328	0.74	--?+-+	0.92	11810	-0.351	0.73	--?+-+	0.92
		100002128	17alpha-hydroxypregnenolone 3-sulfate	Lipid	1083	-0.092	0.93	+????-	0.97	1045	0.229	0.82	+????-	0.94	1045	0.255	0.80	+????-	0.94
		100000437	theophylline	Xenobiotics	13596	-0.091	0.93	++++--	0.97	13556	-0.254	0.80	++++--	0.93	13549	-0.023	0.98	++++--	0.99
		100001150	propionylglycine	Lipid	10885	0.083	0.93	-??++-	0.97	10847	0.61	0.54	-??++-	0.85	10847	0.755	0.45	-??+++	0.85
		100006641	glycochenodeoxycholate sulfate	Lipid	10885	0.077	0.94	-??-+-	0.98	10847	-0.025	0.98	-??-+-	0.99	10847	-0.219	0.83	-??-+-	0.95
		100008928	2-hydroxybutyrate/2-hydroxyisobutyrate	Amino acid	12631	-0.074	0.94	+?++--	0.98	12592	0.538	0.59	+?++--	0.87	12586	0.595	0.55	+?-+--	0.87
		100009028	N-palmitoyl-sphinganine (d18:0/16:0)	Lipid	10885	0.065	0.95	-??-+-	0.98	10847	-0.504	0.61	-??-+-	0.88	10847	-0.401	0.69	-??-+-	0.91
		409	malate	Energy	13596	0.065	0.95	++++-+	0.98	13556	0.07	0.94	++++-+	0.97	13549	0.387	0.70	++++-+	0.91
		1083	N-acetylmethionine	Amino acid	10885	0.066	0.95	+??-+-	0.98	10847	0.38	0.70	+??-+-	0.91	10847	0.018	0.99	+??-+-	0.99
		100008954	palmitoyl dihydrosphingomyelin (d18:0/16:0)*	Lipid	10885	0.053	0.96	-??+--	0.98	10847	0.117	0.91	-??+--	0.97	10847	0.447	0.66	-??+--	0.91
		1104	methyl indole-3-acetate	Xenobiotics	10885	0.052	0.96	+??++-	0.98	10847	-0.519	0.60	+??---	0.87	10847	-0.39	0.70	+??---	0.91
		100001776	2-linoleoylglycerophosphoethanolamine*	Lipid	10767	0.056	0.96	?+?+-?	0.98	10766	-0.335	0.74	?-?--?	0.92	10765	-0.301	0.76	?+?--?	0.93
		35	S-1-pyrroline-5-carboxylate	Amino acid	10885	0.052	0.96	-??+-+	0.98	10847	0.106	0.92	-??+-+	0.97	10847	0.304	0.76	-??+-+	0.93
		1206	isocitrate	Energy	10401	-0.062	0.95	???-+-	0.98	10401	-0.209	0.83	???-+-	0.95	10401	0.12	0.90	???-+-	0.97
		241	phenylpyruvate	Amino acid	10885	0.058	0.95	-??++-	0.98	10847	-0.134	0.89	-??+--	0.97	10847	-0.093	0.93	-??+--	0.98
		100001611	3beta,7alpha-dihydroxy-5-cholestenoate	Lipid	1083	0.055	0.96	+????-	0.98	1045	0.19	0.85	-????+	0.95	1045	0.054	0.96	-????+	0.99
		100010928	linoleoyl-docosahexaenoyl-glycerol (18:2/22:6) [1]*	Lipid	1083	0.06	0.95	+????-	0.98	1045	0.113	0.91	+????-	0.97	1045	0.041	0.97	+????-	0.99
		100000841	oxalate (ethanedioate)	Cofactors and Vitamins	10885	0.049	0.96	-??++-	0.99	10847	0.805	0.42	-??++-	0.81	10847	1.365	0.17	-??++-	0.67
		331	gamma-glutamylglutamate	Peptide	10885	0.045	0.96	+??+--	0.99	10847	-0.13	0.90	+??+-+	0.97	10847	-0.269	0.79	+??+--	0.93
		500	riboflavin (Vitamin B2)	Cofactors and Vitamins	5762	0.04	0.97	????+-	0.99	5762	0.156	0.88	????+-	0.96	5762	0.29	0.77	????+-	0.93
		1105	alpha-tocopherol	Cofactors and Vitamins	12631	-0.043	0.97	-?++--	0.99	12592	-0.257	0.80	-?++--	0.93	12586	0.032	0.97	-?++--	0.99
		100001593	glutarylcarnitine (C5-DC)	Amino acid	13596	-0.037	0.97	--++--	0.99	13556	-0.476	0.63	--++--	0.89	13549	-0.416	0.68	--++--	0.91
		100002196	13-HODE + 9-HODE	Lipid	11850	0.025	0.98	++?-++	0.99	11811	0.39	0.70	-+?-++	0.91	11810	0.612	0.54	-+?-++	0.87
		100015731	N-palmitoyl-heptadecasphingosine (d17:1/16:0)*	Lipid	6246	-0.022	0.98	+???+-	0.99	6208	-0.908	0.36	+???--	0.78	6208	-0.682	0.50	+???--	0.87
		100001007	ribonate	Carbohydrate	10286	-0.018	0.99	+??-+?	1.00	10248	-1.008	0.31	+??--?	0.73	10248	-0.83	0.41	+??--?	0.83
		100000491	gamma-glutamylphenylalanine	Peptide	13596	-0.018	0.99	+-+---	1.00	13556	-0.079	0.94	+-+---	0.97	13549	-0.383	0.70	+-----	0.91
		100009131	1-linoleoyl-2-arachidonoyl-GPC (18:2/20:4n6)*	Lipid	10286	0.019	0.99	-??-+?	1.00	10248	-0.121	0.90	-??-+?	0.97	10248	-0.135	0.89	-??-+?	0.96
		100004442	1-arachidonoyl-GPA (20:4)	Lipid	10885	-0.011	0.99	-??++-	1.00	10847	-0.357	0.72	-??-+-	0.91	10847	-0.275	0.78	-??-+-	0.93
		100001756	4-ethylphenylsulfate	Xenobiotics	12631	0.004	1.00	-?+++-	1.00	12592	-0.673	0.50	-?-++-	0.84	12586	-1.396	0.16	-?----	0.64
		100001553	1-dihomo-linoleoylglycerophosphocholine (20:2n6)*	Lipid	10767	-0.004	1.00	?-?-+?	1.00	10766	-0.666	0.51	?-?-+?	0.84	10765	-0.691	0.49	?-?--?	0.87
		182	quinolinate	Cofactors and Vitamins	10885	-0.005	1.00	-??+--	1.00	10847	-0.588	0.56	-??+--	0.86	10847	-0.391	0.70	-??+--	0.91
		849	caffeine	Xenobiotics	13596	-0.004	1.00	++-+--	1.00	13556	-0.361	0.72	++++--	0.91	13549	-0.188	0.85	++-+--	0.95
		100000467	3-indoxyl sulfate	Amino acid	13596	-0.002	1.00	++--++	1.00	13556	-0.159	0.87	++--+-	0.96	13549	0.092	0.93	++--+-	0.98

Supplementary Table 5: Results of the sex-stratified association analysis of all metabolites with depression for model 2.
The order of the direction column: RS, SHIP-trend, KORA, EPIC-Norfolk B2, EPIC-Norfolk B3, NEO.

		ALL							MEN					WOMEN				
Metabolite	Name	N	Zscore	P.value	Direction	HetISq	HetPVal	FDR	N	Zscore	P.value	Direction	FDR	N	Zscore	P.value	Direction	FDR
100002049	4-hydroxycoumarin	10847	-4.48	7.51E-06	-??---	79.7	0.00	4.0E-03	5139	-1.39	0.16	+??+--	8.12E-01	5708	-4.469	7.85E-06	-??---	7.84E-03
100000014	hippurate	13556	-4.17	3.02E-05	------	49.4	0.08	1.1E-02	6404	-2.921	3.49E-03	+-----	4.54E-01	7151	-3.183	1.46E-03	-+----	1.24E-01
100004227	2-aminooctanoate	11811	-4.00	6.30E-05	--?---	45.5	0.12	1.7E-02	5563	-2.831	4.63E-03	--?---	4.54E-01	6248	-2.463	0.01	--?---	2.81E-01
100001197	10-undecenoate (11:1n1)	13556	-3.94	8.19E-05	+-----	22.7	0.26	1.7E-02	6404	-2.193	0.03	+-----	6.08E-01	7151	-3.212	1.32E-03	------	1.24E-01
498	retinol (Vitamin A)	10847	3.89	9.88E-05	+??+++	0	0.85	1.7E-02	5139	2.448	0.01	+??++-	4.92E-01	5708	3.766	1.66E-04	+??+++	4.39E-02
100000010	3-phenylpropionate (hydrocinnamate)	13556	-3.73	1.91E-04	------	48.2	0.09	2.9E-02	6404	-2.494	0.01	--+---	4.79E-01	7151	-3.028	2.46E-03	-+----	1.44E-01
1090	bilirubin (Z,Z)	13556	-3.60	3.20E-04	------	0	0.75	3.5E-02	6404	-2.121	0.03	---+--	6.08E-01	7151	-2.72	6.52E-03	-+----	2.19E-01
100008984	1-palmitoyl-2-palmitoleoyl-GPC (16:0/16:1)*	10847	3.58	3.49E-04	+??+++	0	0.43	3.5E-02	5139	1.782	0.07	+??++-	7.73E-01	5708	3.474	5.13E-04	+??+++	7.51E-02
100009082	1-linoleoyl-GPA (18:2)*	10847	-3.57	3.63E-04	-??---	60.6	0.05	3.5E-02	5139	-1.82	0.07	-??---	7.73E-01	5708	-2.719	6.56E-03	-??---	2.19E-01
391	citrulline	13556	-3.53	4.20E-04	------	0	0.92	3.5E-02	6404	-1.469	0.14	--++--	7.92E-01	7151	-2.911	3.60E-03	-+----	1.73E-01
100002253	cinnamoylglycine	10847	-3.50	4.58E-04	-??---	51.4	0.10	3.5E-02	5139	-1.301	0.19	+??---	8.30E-01	5708	-3.616	2.99E-04	-??---	5.29E-02
100001951	bilirubin (E,Z or Z,E)*	12592	-3.50	4.65E-04	-?----	19.6	0.29	3.5E-02	5980	-1.062	0.29	-?-+--	8.86E-01	6611	-3.359	7.83E-04	-?----	8.30E-02
100001740	mannitol/sorbitol	12592	3.39	7.11E-04	+?-+++	65.8	0.02	5.02E-02	5980	2.386	0.02	+?-+++	5.16E-01	6611	2.613	8.97E-03	-?-+++	2.43E-01
100001950	bilirubin (E,E)*	13556	-3.33	8.71E-04	------	0	0.71	5.13E-02	6404	-2.001	0.05	-+----	6.42E-01	7151	-2.499	0.01	------	2.79E-01
100001251	decanoylcarnitine (C10)	13556	-3.35	8.04E-04	+-----	62	0.02	5.13E-02	6404	-1.226	0.22	+--++-	8.59E-01	7151	-2.848	4.40E-03	+-----	2.03E-01
100001510	phenol sulfate	13556	3.261	1.11E-03	-+++++	34.2	0.18	0.06	6404	2.652	8.00E-03	++++++	4.54E-01	7151	2.274	0.02	-+++++	3.18E-01
100001977	beta-cryptoxanthin	6208	-3.25	1.14E-03	-???--	87.3	0.00	5.74E-02	2932	-0.74	0.46	-???+-	9.30E-01	3276	-3.659	2.53E-04	-???--	5.29E-02
100001658	taurolithocholate 3-sulfate	13556	-3.09	2.02E-03	------	40.7	0.13	9.31E-02	6404	-2.667	7.66E-03	------	4.54E-01	7151	-2.157	0.03	-+-+--	3.49E-01
100000265	kynurenine	13556	-3.039	2.37E-03	------	0	0.45	0.10	6404	-1.298	0.19	---+--	8.30E-01	7151	-2.798	5.14E-03	-----+	2.17E-01
100001083	indolepropionate	13556	-2.95	3.18E-03	------	33.7	0.18	1.35E-01	6404	-2.161	0.03	-----+	6.08E-01	7151	-2.114	0.03	------	3.51E-01
1539	1-palmitoyl-2-oleoyl-GPC (16:0/18:1)	10847	2.93	3.39E-03	+??++-	26.1	0.25	1.36E-01	5139	1.764	0.08	+??++-	7.73E-01	5708	2.666	7.68E-03	+??++-	2.32E-01
100008914	1-palmitoyl-2-arachidonoyl-GPC (16:0/20:4n6)	10847	2.92	3.47E-03	-??+++	19	0.30	1.36E-01	5139	1.312	0.19	-??+++	8.30E-01	5708	2.975	2.93E-03	+??++-	1.54E-01
250	biliverdin	13556	-2.88	3.95E-03	------	0	0.90	1.40E-01	6404	-2.544	0.01	------	4.74E-01	7151	-1.485	0.14	--+---	5.54E-01
100003179	leucylalanine	2790	-2.883	3.94E-03	-?-??-	0	0.53	0.14	1338	-2.471	0.01	-?-??-	4.92E-01	1451	-1.859	0.06	-?-??-	4.25E-01
100005372	1-(1-enyl-oleoyl)-GPE (P-18:1)*	11811	-2.881	3.96E-03	-+?---	33.7	0.20	0.14	5563	-1.482	0.14	++?---	7.92E-01	6248	-2.118	0.03	-+?---	3.51E-01
100001868	4-allylphenol sulfate	10847	-2.86	4.27E-03	-??-+-	77.9	0.00	1.46E-01	5139	-1.478	0.14	-??-+-	7.92E-01	5708	-2.142	0.03	-??-+-	3.51E-01
100001055	isobutyrylcarnitine (C4)	13556	-2.824	4.74E-03	------	31.6	0.20	0.16	6404	-0.375	0.71	+--+--	9.52E-01	7151	-3.029	2.46E-03	------	1.44E-01
100002458	3-methylglutaconate	10847	2.794	5.21E-03	+??+++	0	0.49	0.16	5139	1.065	0.29	+??-++	8.83E-01	5708	2.493	0.01	+??+++	2.79E-01
100002259	cis-4-decenoylcarnitine (C10:1)	13556	-2.80	5.13E-03	------	0	0.49	1.58E-01	6404	-0.919	0.36	---++-	9.04E-01	7151	-2.326	0.02	+-----	2.98E-01
98	kynurenate	10847	-2.79	5.35E-03	-??---	42.4	0.16	1.58E-01	5139	-0.711	0.48	+??+--	9.37E-01	5708	-2.756	5.86E-03	-??---	2.17E-01
100002945	15-methylpalmitate	13556	-2.77	5.68E-03	+-----	7.7	0.37	1.63E-01	6404	-1.081	0.28	+-----	8.77E-01	7151	-2.427	0.02	+-----	2.87E-01
397	leucine	13556	-2.73	6.30E-03	--+--+	77.3	0.00	1.76E-01	6404	-2.674	7.50E-03	+-++--	4.54E-01	7151	-1.19	0.23	--+--+	6.40E-01
100001121	pyridoxate	13556	2.71	6.73E-03	++-++-	71.5	0.00	1.83E-01	6404	1.841	0.07	++-++-	7.73E-01	7151	1.891	0.06	++-++-	4.20E-01
100001392	laurylcarnitine (C12)	13556	-2.68	7.41E-03	+-----	69	0.01	1.96E-01	6404	-0.342	0.73	++-++-	9.52E-01	7151	-2.763	5.73E-03	+-----	2.17E-01
1526	1-palmitoyl-2-oleoyl-GPE (16:0/18:1)	10847	2.64	8.41E-03	-??+++	0	0.40	2.12E-01	5139	-0.095	0.92	-??-++	9.90E-01	5708	3.447	5.67E-04	+??+++	7.51E-02
100001247	octanoylcarnitine (C8)	13556	-2.62	8.69E-03	+-----	51.2	0.07	2.14E-01	6404	-0.467	0.64	+--++-	9.47E-01	7151	-2.394	0.02	+-----	2.89E-01
100000997	3-hydroxydecanoate	11811	-2.60	9.45E-03	+-?---	24.8	0.26	2.23E-01	5563	-1.004	0.32	+-?---	9.04E-01	6248	-2.356	0.02	+-?---	2.98E-01
533	urea	13556	-2.595	9.45E-03	+-----	0	0.62	0.22	6404	-0.915	0.36	+---+-	9.04E-01	7151	-2.159	0.03	-----+	3.49E-01
189	N6,N6,N6-trimethyllysine	10847	-2.555	0.01	-??---	0	0.94	0.24	5139	-0.996	0.32	+??+--	9.04E-01	5708	-2.219	0.03	-??--+	3.43E-01
100004318	indolin-2-one	5684	-2.554	0.01	+??-?-	0	0.58	0.24	2704	0.22	0.83	+??+?-	9.54E-01	2980	-3.394	6.90E-04	-??-?-	8.13E-02
100002488	isoursodeoxycholate	6208	2.537	0.01	+???++	0	0.45	0.24	2932	0.887	0.38	+???-+	9.04E-01	3276	2.605	9.19E-03	-???++	2.43E-01
100001112	3-hydroxylaurate	10847	-2.54	1.10E-02	-??---	16.2	0.31	2.42E-01	5139	-0.962	0.34	+??---	9.04E-01	5708	-2.286	0.02	-??---	3.15E-01
100015759	stearoylcholine*	6208	-2.505	0.01	-???--	51.4	0.13	0.25	2932	-1.386	0.17	-???--	8.12E-01	3276	-1.949	0.05	-???--	4.02E-01
2051	methylsuccinate	10847	-2.506	0.01	+??---	0	0.41	0.25	5139	-1.228	0.22	+??---	8.59E-01	5708	-2.078	0.04	-??+--	3.51E-01
100008915	1-palmitoyl-2-docosahexaenoyl-GPC (16:0/22:6)	10401	2.502	0.01	???++-	67.4	0.05	0.25	4926	0.835	0.40	???++-	9.04E-01	5475	2.476	0.01	???++-	2.81E-01

100000743	2-hydroxyoctanoate	10847	-2.483	0.01	-??-+-	55.7	0.08	0.25	5139	-2.708	6.76E-03	-??---	4.54E-01	5708	-1.087	0.28	-??-+-	6.66E-01
100006430	arabitol/xylitol	12592	2.48	1.30E-02	+?++++	0	0.76	2.51E-01	5980	2.25	0.02	+?++++	6.08E-01	6611	1.693	0.09	+?++++	4.89E-01
100001657	glycolithocholate sulfate*	11811	-2.46	1.37E-02	--?---	0	0.92	2.60E-01	5563	-1.492	0.14	--?---	7.92E-01	6248	-2.092	0.04	-+?---	3.51E-01
566	valine	13556	-2.456	0.01	--+--+	66.6	0.01	0.26	6404	-2.408	0.02	+-+---	5.16E-01	7151	-1.128	0.26	--+--+	6.61E-01
100001511	1-palmitoleoyl-GPC (16:1)*	13556	2.43	1.53E-02	+++++-	0	0.88	2.74E-01	6404	2.523	0.01	+++++-	4.74E-01	7151	1.687	0.09	+-++++	4.89E-01
100008991	1-palmitoyl-2-docosahexaenoyl-GPE (16.0/22.6)	10401	2.43	1.51E-02	???+++	0	0.81	2.74E-01	4926	1.164	0.24	???+++	8.59E-01	5475	2.091	0.04	???++-	3.51E-01
100004054	margaroylcarnitine*	6208	-2.399	0.02	+???--	78.6	0.01	0.28	2932	-1.612	0.11	+???--	7.92E-01	3276	-1.246	0.21	+???-+	6.29E-01
100002070	2-hydroxyglutarate	7953	2.407	0.02	-?+?++	30.9	0.23	0.28	3773	1.124	0.26	-?+?++	8.69E-01	4179	2.138	0.03	-?+?+-	3.51E-01
100001403	5-acetylamino-6-amino-3-methyluracil	10847	-2.332	0.02	-??+--	53.7	0.09	0.28	5139	-2.754	5.89E-03	+??+--	4.54E-01	5708	-0.848	0.40	-??---	7.79E-01
100003915	palmitic amide	10401	2.35	0.02	???-++	64.4	0.06	0.28	4926	1.79	0.07	???-++	7.73E-01	5475	1.496	0.13	???-+-	5.51E-01
100001554	2-arachidonoylglycerophosphocholine*	10766	2.315	0.02	?+?++?	24.9	0.26	0.28	5066	1.476	0.14	?-?++?	7.92E-01	5700	2.414	0.02	?+?+-?	2.87E-01
100015834	lignoceroylcarnitine (C24)*	6208	-2.322	0.02	-???--	54.7	0.11	0.28	2932	-1.348	0.18	-???--	8.30E-01	3276	-1.345	0.18	+???--	5.89E-01
1094	thyroxine	10847	2.327	0.02	+??+++	32.9	0.21	0.28	5139	1.131	0.26	+??+++	8.69E-01	5708	1.834	0.07	+??+++	4.25E-01
1022	picolinate	6208	-2.355	0.02	+???--	87.2	0.00	0.28	2932	-0.397	0.69	+???+-	9.48E-01	3276	-2.676	7.46E-03	-???--	2.32E-01
100001271	1-stearoyl-GPC (18:0)	13556	-2.362	0.02	-+----	3.6	0.39	0.28	6404	0.029	0.98	++-+--	9.92E-01	7151	-1.968	0.05	-+----	3.97E-01
100015851	docosapentaenoylcarnitine (C22:5n3)*	5762	-2.305	0.02	????--	0	0.98	0.29	2719	0.688	0.49	????++	9.40E-01	3043	-3.129	1.76E-03	????--	1.24E-01
923	dihydroorotate	6208	-2.281	0.02	+???-+	0	0.39	0.29	2932	-2.582	9.83E-03	-???-+	4.74E-01	3276	-0.785	0.43	+???--	7.93E-01
100005391	3-(3-hydroxyphenyl)propionate sulfate	10847	-2.29	0.02	-??---	13.2	0.33	0.29	5139	-1.716	0.09	+??---	7.84E-01	5708	-1.671	0.09	-??---	4.92E-01
823	pyruvate	13556	2.28	2.28E-02	++++-+	62.5	0.02	2.91E-01	6404	1.654	0.10	++++++	7.92E-01	7151	1.352	0.18	+-++-+	5.88E-01
100001992	androstenediol (3beta,17beta) disulfate (1)	13556	2.264	0.02	++++++	0	0.79	0.30	6404	1.605	0.11	+++-++	7.92E-01	7151	1.121	0.26	--+++-	6.61E-01
100005353	1-nonadecanoyl-GPC (19:0)	9802	-2.263	0.02	???--?	0	0.61	0.30	4642	-0.657	0.51	???--?	9.44E-01	5160	-1.952	0.05	???--?	4.02E-01
93	alpha-ketoglutarate	12592	2.237	0.03	+?++-+	42.5	0.14	0.30	5980	1.762	0.08	+?++-+	7.73E-01	6611	1.617	0.11	+?-+++	5.20E-01
100001400	1-methylurate	12592	-2.217	0.03	-?----	0	0.79	0.30	5980	-1.491	0.14	+?++--	7.92E-01	6611	-1.584	0.11	-?--+-	5.30E-01
100000773	3-hydroxyoctanoate	11811	-2.25	2.47E-02	+-?---	0	0.46	3.00E-01	5563	-1.681	0.09	+-?---	7.92E-01	6248	-1.481	0.14	+-?---	5.54E-01
100008990	1-palmitoyl-2-arachidonoyl-GPE (16:0/20:4)*	10847	2.22	2.65E-02	-??+++	0	0.63	3.00E-01	5139	0.804	0.42	-??-++	9.11E-01	5708	2.301	0.02	+??++-	3.08E-01
100008992	1-stearoyl-2-docosahexaenoyl-GPE (18.0/22.6)	10401	2.213	0.03	???+++	0	0.72	0.30	4926	0.596	0.55	???+++	9.44E-01	5475	2.468	0.01	???++-	2.81E-01
1869	2-hydroxyhippurate (salicylurate)	10847	-2.229	0.03	-??---	71.1	0.02	0.30	5139	-0.594	0.55	+??+--	9.44E-01	5708	-2.404	0.02	-??---	2.87E-01
100001445	1-palmitoyl-GPA (16:0)	11212	-2.233	0.03	--?--?	0	0.76	0.30	5279	0.409	0.68	+-?++?	9.48E-01	5933	-2.802	5.07E-03	--?--?	2.17E-01
100001550	homostachydrine*	13556	-2.189	0.03	+-----	0	0.79	0.31	6404	-1.248	0.21	---+--	8.48E-01	7151	-1.683	0.09	+----+	4.89E-01
100010927	linoleoyl-linolenoyl-glycerol (18:2/18:3) [2]*	5609	-2.192	0.03	-???-?	0	0.34	0.31	2648	-0.821	0.41	-???-?	9.08E-01	2961	-1.694	0.09	-???-?	4.89E-01
1053	3-ureidopropionate	10847	-2.175	0.03	-??--+	0	0.47	0.32	5139	-1.421	0.16	-??--+	8.12E-01	5708	-2.13	0.03	+??---	3.51E-01
100002008	5alpha-androstan-3alpha,17alpha-diol monosulfate	1045	-2.175	0.03	-????-	0	0.80	0.32	497	-0.211	0.83	+????-	9.54E-01	548	-2.344	0.02	-????-	2.98E-01
100003210	valylleucine	1045	-2.156	0.03	-????-	0	0.64	0.33	497	-1.797	0.07	-????-	7.73E-01	548	-1.275	0.20	-????-	6.20E-01
100001567	1-palmitoyl-GPE (16:0)	13556	2.15	3.12E-02	++-++-	0	0.69	3.28E-01	6404	0.739	0.46	++-++-	9.30E-01	7151	2.514	0.01	+-+++-	2.79E-01
100009220	1-oleoyl-2-docosahexaenoyl-GPE (18:1/22:6)*	5762	2.146	0.03	????++	0	0.56	0.33	2719	1.444	0.15	????++	8.00E-01	3043	1.59	0.11	????++	5.30E-01
111	3-hydroxyisobutyrate	11811	-2.129	0.03	+-?---	36.2	0.18	0.34	5563	-0.749	0.45	+-?-+-	9.27E-01	6248	-2.077	0.04	+-?---	3.51E-01
100000781	hexanoylcarnitine (C6)	13556	-2.13	0.03	---+--	0	0.55	0.34	6404	0.296	0.77	+--+--	9.54E-01	7151	-2.272	0.02	------	3.18E-01
1268	gamma-glutamylleucine	13556	-2.119	0.03	+-----	0	0.64	0.34	6404	-1.304	0.19	+--+--	8.30E-01	7151	-1.257	0.21	-----+	6.25E-01
100001609	7-alpha-hydroxy-3-oxo-4-cholestenoate (7-Hoca)	13556	2.102	0.04	+++--+	72.6	0.00	0.35	6404	2.062	0.04	+++-++	6.24E-01	7151	1.304	0.19	+++-++	6.12E-01
100002514	hydantoin-5-propionic acid	10847	-2.1	0.04	+??--+	37.6	0.19	0.35	5139	-0.451	0.65	+??+--	9.48E-01	5708	-2.191	0.03	+??--+	3.46E-01
100000257	glucuronate	10847	2.09	3.65E-02	+??+++	0	0.57	3.52E-01	5139	2.063	0.04	+??++-	6.24E-01	5708	1.25	0.21	+??+++	6.29E-01
212	5-methylthioadenosine (MTA)	10847	2.09	3.64E-02	+??+++	38.9	0.18	3.52E-01	5139	2.035	0.04	+??+++	6.25E-01	5708	1.176	0.24	+??-++	6.40E-01
192	N-acetylputrescine	10847	2.09	3.68E-02	-??-++	54.8	0.08	3.52E-01	5139	0.055	0.96	-??-++	9.92E-01	5708	2.422	0.02	+??+++	2.87E-01
1128	2-aminobutyrate	13556	-2.08	3.76E-02	---+--	3.1	0.40	3.54E-01	6404	-0.761	0.45	---+-+	9.21E-01	7151	-1.892	0.06	---+--	4.20E-01
381	2-aminoadipate	10847	-2.078	0.04	-??---	0	0.59	0.35	5139	-0.016	0.99	-??++-	9.94E-01	5708	-2.358	0.02	-??---	2.98E-01
100001402	5-acetylamino-6-formylamino-3-methyluracil	10847	-2.061	0.04	-??+--	29.3	0.24	0.37	5139	-2.747	6.01E-03	-??--+	4.54E-01	5708	-0.722	0.47	-??+--	8.04E-01
100002021	5alpha-androstan-3beta,17alpha-diol disulfate	10847	-2.05	4.00E-02	-??---	25.5	0.26	3.67E-01	5139	-0.859	0.39	-??-+-	9.04E-01	5708	-2.191	0.03	-??---	3.46E-01
100001162	propionylcarnitine (C3)	13556	-2.052	0.04	--++--	25.8	0.24	0.37	6404	-0.506	0.61	+-++--	9.47E-01	7151	-1.832	0.07	-----+	4.25E-01
100001393	isovalerylcarnitine (C5)	13556	-2.045	0.04	---+--	56.5	0.04	0.37	6404	-0.402	0.69	+--+--	9.48E-01	7151	-2.004	0.05	---+--	3.79E-01
100001211	sebacate (decanedioate)	10847	-2.043	0.04	-??---	0	0.46	0.37	5139	0.281	0.78	-??-++	9.54E-01	5708	-2.7	6.92E-03	+??---	2.22E-01

100015792	sphingomyelin (d18:1/25:0, d19:0/24:1, d20:1/23:0, d19:1/24:0)*	6208	-2.024	0.04	-???--	66.2	0.05	0.38	2932	-2.506	0.01	-???--	4.79E-01	3276	0.004	1.00	-???+-	9.99E-01
100001870	1-palmitoyl-2-linoleoyl-GPE (16:0/18:2)	10847	2.02	4.32E-02	+??+++	0	0.85	3.78E-01	5139	0.483	0.63	-??+++	9.47E-01	5708	2.085	0.04	+??++-	3.51E-01
100006367	3-hydroxyhexanoate	10847	-2.015	0.04	+??---	34.4	0.21	0.38	5139	-1.274	0.20	+??---	8.30E-01	5708	-1.687	0.09	+??---	4.89E-01
1024	pantothenate	13556	2.009	0.04	++-+++	32.2	0.19	0.38	6404	0.469	0.64	+--+++	9.47E-01	7151	2.43	0.02	++-+++	2.87E-01
100000611	1-palmityl-GPC (O-16:0)	9802	-1.999	0.05	???--?	0	0.90	0.39	4642	0.093	0.93	???+-?	9.91E-01	5160	-2.256	0.02	???--?	3.23E-01
100010901	gamma-glutamyl-alpha-lysine	6208	-1.959	0.05	+???--	5.1	0.35	0.40	2932	-1.688	0.09	+???--	7.92E-01	3276	-0.987	0.32	+???-+	7.12E-01
100004284	dimethyl sulfone	11811	-1.957	0.05	--?---	0	0.89	0.40	5563	-1.301	0.19	--?+--	8.30E-01	6248	-1.56	0.12	-+?---	5.36E-01
100009264	glycochenodeoxycholate glucuronide (1)	10847	1.976	0.05	+??+++	0	0.89	0.40	5139	1.234	0.22	+??+++	8.56E-01	5708	1.189	0.23	-??+++	6.40E-01
100005850	3-methylglutarylcarnitine (2)	10847	1.976	0.05	+??+++	0	0.94	0.40	5139	0.845	0.40	+??++-	9.04E-01	5708	1.939	0.05	-??+++	4.06E-01
100009066	1-palmitoyl-2-oleoyl-GPI (16:0/18:1)*	6208	1.96	5.02E-02	+???+-	0	0.69	4.01E-01	2932	0.442	0.66	+???+-	9.48E-01	3276	2.606	9.16E-03	-???++	2.43E-01
100001429	1-margaroylglycerol (17:0)	1045	-1.958	0.05	-????-	28.6	0.24	0.40	497	-0.418	0.68	+????-	9.48E-01	548	-2.325	0.02	-????-	2.98E-01
100008977	1-stearoyl-2-arachidonoyl-GPE (18:0/20:4)	10847	1.94	5.19E-02	-??+++	0	0.39	4.07E-01	5139	0.863	0.39	-??+++	9.04E-01	5708	2.214	0.03	+??++-	3.43E-01
100008934	2-hydroxylaurate	5609	1.939	0.05	+???+?	78.3	0.03	0.41	2648	0.623	0.53	+???+?	9.44E-01	2961	2.019	0.04	+???+?	3.79E-01
100015835	cerotoylcarnitine (C26)*	6208	-1.935	0.05	-???--	61.5	0.07	0.41	2932	-0.943	0.35	-???+-	9.04E-01	3276	-1.106	0.27	+???--	6.61E-01
466	phytanate	10847	-1.932	0.05	+??---	54.5	0.09	0.41	5139	-0.575	0.57	+??+--	9.44E-01	5708	-1.907	0.06	+??--+	4.19E-01
561	glutamate	13556	1.92	5.46E-02	++++-+	71.7	0.00	4.13E-01	6404	2.241	0.03	++++-+	6.08E-01	7151	1.316	0.19	++++-+	6.06E-01
811	alanine	13556	1.908	0.06	--++-+	61.3	0.02	0.41	6404	1.301	0.19	++++--	8.30E-01	7151	1.627	0.10	--++-+	5.14E-01
100001145	3-hydroxysebacate	5684	-1.915	0.06	+??-?-	0	0.45	0.41	2704	-1.279	0.20	-??-?+	8.30E-01	2980	-1.58	0.11	+??-?-	5.30E-01
100001395	1-linoleoyl-GPC (18:2)	13556	-1.908	0.06	------	32	0.20	0.41	6404	-0.588	0.56	---++-	9.44E-01	7151	-1.498	0.13	------	5.51E-01
100002094	gamma-CEHC	10847	-1.913	0.06	-??--+	6.2	0.36	0.41	5139	-0.402	0.69	-??+-+	9.48E-01	5708	-1.971	0.05	-??--+	3.97E-01
100003271	beta-citrylglutamate	6208	-1.896	0.06	+???--	37.2	0.20	0.42	2932	-1.463	0.14	+???--	7.92E-01	3276	-1.208	0.23	+???--	6.37E-01
100000987	2-linoleoylglycerol (18:2)	10847	-1.887	0.06	-??---	0	0.48	0.42	5139	-1.108	0.27	-??-+-	8.74E-01	5708	-1.399	0.16	-??---	5.64E-01
100015760	linoleoylcholine*	6208	-1.893	0.06	-???--	75.2	0.02	0.42	2932	-0.837	0.40	-???--	9.04E-01	3276	-1.786	0.07	-???--	4.51E-01
444	ornithine	12592	-1.897	0.06	+?-+--	3.1	0.39	0.42	5980	-0.495	0.62	+?++--	9.47E-01	6611	-1.653	0.10	-?----	4.99E-01
100003001	1-(1-enyl-stearoyl)-GPE (P-18:0)*	10847	-1.889	0.06	-??---	2.2	0.38	0.42	5139	-0.479	0.63	+??---	9.47E-01	5708	-1.532	0.13	-??---	5.43E-01
100001882	glycosyl-N-stearoyl-sphingosine (d18:1/18:0)	10847	-1.875	0.06	+??---	76.5	0.01	0.42	5139	-1.561	0.12	+??---	7.92E-01	5708	-0.962	0.34	+??-++	7.24E-01
1113	4-acetamidobutanoate	13556	1.863	0.06	+++-+-	0	0.64	0.43	6404	1.173	0.24	-++++-	8.59E-01	7151	1.424	0.15	++--++	5.64E-01
100000282	N-acetylglutamate	10847	1.856	0.06	+??+++	0	0.50	0.43	5139	1.389	0.16	+??+++	8.12E-01	5708	1.237	0.22	-??-++	6.29E-01
100000442	quinate	12592	-1.854	0.06	+?+---	50.1	0.09	0.43	5980	-1.371	0.17	+?+---	8.21E-01	6611	-1.313	0.19	-?+---	6.08E-01
100000706	alpha-hydroxyisocaproate	11811	-1.854	0.06	+-?+--	25	0.26	0.43	5563	-1.171	0.24	++?+--	8.59E-01	6248	-1.041	0.30	--?+-+	6.89E-01
1504	oleamide	10401	1.849	0.06	???-++	54.5	0.11	0.43	4926	1.321	0.19	???-++	8.30E-01	5475	1.17	0.24	???-+-	6.40E-01
100001212	guanidinosuccinate	1045	-1.844	0.07	-????-	0	0.40	0.43	497	-2.211	0.03	-????-	6.08E-01	548	-0.336	0.74	-????-	9.24E-01
881	cytosine	6208	-1.843	0.07	-???--	47.4	0.15	0.43	2932	-0.399	0.69	-???+-	9.48E-01	3276	-2	0.05	-???--	3.79E-01
100001571	1-arachidonoyl-GPE (20:4n6)*	13556	1.836	0.07	++++++	0	0.78	0.44	6404	1.74	0.08	-+++++	7.84E-01	7151	1.573	0.12	++++++	5.30E-01
100001106	1,3-dimethylurate	10847	-1.829	0.07	+??---	0	0.46	0.44	5139	-2.646	8.15E-03	+??+--	4.54E-01	5708	-0.624	0.53	+??-+-	8.42E-01
100001561	2-palmitoleoyl-GPC (16:1)*	6648	1.83	0.07	++?+?+	0	0.88	0.44	3128	-0.258	0.80	++?-?+	9.54E-01	3520	2.581	9.86E-03	-+?+?+	2.55E-01
100015785	nisinate (24:6n3)	5762	1.823	0.07	????++	0	0.97	0.44	2719	1.035	0.30	????+-	9.04E-01	3043	1.552	0.12	????++	5.36E-01
1124	citrate	13556	-1.824	0.07	+-----	0	0.98	0.44	6404	-0.303	0.76	+---+-	9.54E-01	7151	-1.745	0.08	------	4.67E-01
100010935	diacylglycerol (14:0/18:1, 16:0/16:1) [2]*	1045	1.821	0.07	+????+	0	0.36	0.44	497	0.024	0.98	+????-	9.93E-01	548	2.559	0.01	+????+	2.59E-01
100015625	glycosyl-N-behenoyl-sphingadienine (d18:2/22:0)*	5762	-1.812	0.07	????--	70	0.07	0.44	2719	-1.503	0.13	????--	7.92E-01	3043	-0.934	0.35	????--	7.42E-01
888	caprate (10:0)	13556	-1.811	0.07	+-----	0	0.71	0.44	6404	-0.892	0.37	++-+--	9.04E-01	7151	-1.513	0.13	+----+	5.48E-01
100008952	1-palmitoleoylglycerol (16:1)*	10847	1.805	0.07	+??-++	0	0.40	0.44	5139	0.485	0.63	+??-++	9.47E-01	5708	2.204	0.03	+??+++	3.43E-01
244	beta-alanine	10401	-1.796	0.07	???---	0	0.89	0.44	4926	-1.059	0.29	???---	8.86E-01	5475	-1.341	0.18	???--+	5.89E-01
100000956	8-hydroxyoctanoate	5684	-1.799	0.07	-??-?-	0	0.75	0.44	2704	-0.827	0.41	-??-?-	9.04E-01	2980	-1.534	0.13	+??-?-	5.43E-01
100002183	S-methylmethionine	5762	-1.791	0.07	????--	72.4	0.06	0.45	2719	-0.954	0.34	????--	9.04E-01	3043	-1.384	0.17	????--	5.66E-01
100001843	gamma-glutamylalanine	5684	1.782	0.07	+??+?+	0	0.74	0.45	2704	2.32	0.02	+??+?-	5.68E-01	2980	0.68	0.50	+??-?+	8.23E-01
55	1-methylnicotinamide	10847	1.782	0.07	-??++-	64.4	0.04	0.45	5139	0.366	0.71	-??+--	9.52E-01	5708	1.653	0.10	-??++-	4.99E-01
100001254	N-acetyltryptophan	10847	1.765	0.08	+??++-	0	0.63	0.45	5139	1.924	0.05	+??+++	7.03E-01	5708	0.804	0.42	+??-+-	7.89E-01
878	fructose	12592	1.768	0.08	+?-+++	39.8	0.16	0.45	5980	1.495	0.13	+?-+++	7.92E-01	6611	1.167	0.24	+?-+-+	6.41E-01
100001054	butyrylcarnitine (C4)	13556	1.765	0.08	-+++++	0	0.60	0.45	6404	1.092	0.27	-++-++	8.74E-01	7151	1.604	0.11	-+++++	5.23E-01

1110	N-acetylalanine	13556	1.767	0.08	-+++++	0	0.55	0.45	6404	0.904	0.37	++++--	9.04E-01	7151	1.461	0.14	-+++++	5.54E-01
100015641	N-oleoylserine	6208	-1.77	0.08	+???--	39.9	0.19	0.45	2932	-0.491	0.62	+???-+	9.47E-01	3276	-2.032	0.04	+???--	3.79E-01
100001320	erythronate*	12592	1.76	7.85E-02	+?++++	17.2	0.30	4.55E-01	5980	3.022	2.51E-03	+?++++	4.54E-01	6611	0.255	0.80	+?-+-+	9.47E-01
100004208	O-methylcatechol sulfate	10847	-1.756	0.08	+??---	0	0.78	0.46	5139	-0.392	0.70	+??--+	9.49E-01	5708	-1.776	0.08	+??---	4.51E-01
100015840	dihomo-linolenoylcarnitine (20:3n3 or 6)*	6208	-1.751	0.08	-???--	17.1	0.30	0.46	2932	0.639	0.52	+???+-	9.44E-01	3276	-2.106	0.04	-???--	3.51E-01
100000453	paraxanthine	13556	-1.733	0.08	-+++--	35.4	0.17	0.47	6404	-1.557	0.12	-+++-+	7.92E-01	7151	-1.12	0.26	-+----	6.61E-01
100001006	N-acetylglycine	12592	1.732	0.08	-?-+++	0	0.49	0.47	5980	1.566	0.12	-?-+++	7.92E-01	6611	1.068	0.29	-?+++-	6.77E-01
1162	N-acetylneuraminate	10847	1.731	0.08	+??+++	0	0.53	0.47	5139	1.093	0.27	+??+--	8.74E-01	5708	1.511	0.13	+??+++	5.48E-01
572	glucose	12592	1.735	0.08	+?++++	69.8	0.01	0.47	5980	0.98	0.33	+?+-++	9.04E-01	6611	1.553	0.12	+?++-+	5.36E-01
100001178	3-carboxy-4-methyl-5-propyl-2-furanpropanoate (CMPF)	13556	-1.725	0.08	+-----	0	0.86	0.47	6404	-1.51	0.13	+-----	7.92E-01	7151	-0.915	0.36	--+---	7.47E-01
100002060	1-docosahexaenoylglycerophosphoethanolamine*	10766	1.718	0.09	?-?++?	0	0.52	0.47	5066	1.47	0.14	?+?++?	7.92E-01	5700	1.233	0.22	?-?++?	6.29E-01
100000711	4-acetylphenol sulfate	10401	-1.722	0.09	???-+-	75.6	0.02	0.47	4926	-0.865	0.39	???---	9.04E-01	5475	-1.543	0.12	???-+-	5.39E-01
100001287	epiandrosterone sulfate	13556	-1.71	8.68E-02	-+----	46.4	0.10	4.70E-01	6404	-1.835	0.07	------	7.73E-01	7151	-1.278	0.20	-+----	6.20E-01
1537	1-palmitoyl-2-linoleoyl-GPC (16:0/18:2)	10847	1.705	0.09	+??++-	59.6	0.06	0.47	5139	1.401	0.16	-??++-	8.12E-01	5708	1.071	0.28	+??++-	6.76E-01
932	caprylate (8:0)	13556	-1.704	0.09	-+----	13.8	0.33	0.47	6404	-0.955	0.34	-+-+--	9.04E-01	7151	-1.334	0.18	-+---+	5.94E-01
100010959	diacylglycerol (12:0/18:1, 14:0/16:1, 16:0/14:1) [2]*	1045	1.705	0.09	+????+	51.5	0.15	0.47	497	0.686	0.49	+????-	9.40E-01	548	1.947	0.05	+????+	4.02E-01
501	salicylate	10847	-1.71	0.09	-??---	55.1	0.08	0.47	5139	-0.165	0.87	+??+--	9.73E-01	5708	-2.178	0.03	-??---	3.46E-01
231	arginine	13556	-1.69	0.09	---+--	0	0.44	0.47	6404	-2.17	0.03	------	6.08E-01	7151	-0.492	0.62	---+-+	8.80E-01
100006098	3-hydroxypyridine sulfate	10847	-1.679	0.09	+??---	9.7	0.34	0.47	5139	-1.074	0.28	+??---	8.82E-01	5708	-1.431	0.15	-??-+-	5.64E-01
100001416	orotidine	10847	1.678	0.09	+??+++	0	0.79	0.47	5139	0.928	0.35	+??+-+	9.04E-01	5708	1.469	0.14	+??+++	5.54E-01
100006314	sphingomyelin (d17:1/16:0, d18:1/15:0, d16:1/17:0)*	10847	-1.686	0.09	-??+--	74.2	0.01	0.47	5139	-0.83	0.41	-??+--	9.04E-01	5708	-1.017	0.31	+??---	6.96E-01
100002185	indole-3-carboxylic acid	5684	-1.688	0.09	-??-?-	52.2	0.12	0.47	2704	-0.537	0.59	-??-?-	9.44E-01	2980	-1.856	0.06	-??-?-	4.25E-01
100010926	linoleoyl-linolenoyl-glycerol (18:2/18:3) [1]*	1045	1.679	0.09	+????+	0	0.61	0.47	497	0.617	0.54	-????+	9.44E-01	548	1.438	0.15	+????+	5.64E-01
100009336	eicosapentaenoylcholine	10847	-1.68	0.09	-??---	59.4	0.06	0.47	5139	0.214	0.83	-??-+-	9.54E-01	5708	-2.104	0.04	-??---	3.51E-01
100009345	1-palmitoleoyl-2-linolenoyl-GPC (16:1/18:3)*	10847	1.673	0.09	+??+++	24.4	0.26	0.47	5139	2.387	0.02	+??+++	5.16E-01	5708	0.324	0.75	+??-+-	9.24E-01
100000487	glycylvaline	2790	1.67	0.09	-?+??-	38.6	0.20	0.47	1338	1.774	0.08	+?+??-	7.73E-01	1451	0.514	0.61	-?+??-	8.71E-01
100004111	4-methylcatechol sulfate	11811	-1.666	0.10	++?---	0	0.60	0.48	5563	-2.229	0.03	+-?+--	6.08E-01	6248	-0.427	0.67	-+?-+-	9.02E-01
100001315	p-cresol sulfate	13556	-1.66	0.10	++----	71.8	0.00	0.48	6404	-1.488	0.14	+--+--	7.92E-01	7151	-1.184	0.24	++----	6.40E-01
100001597	tiglylcarnitine (C5:1-DC)	13556	-1.652	0.10	--+---	61.2	0.02	0.48	6404	1.295	0.20	+--++-	8.30E-01	7151	-2.537	0.01	--+---	2.70E-01
100008980	1-stearoyl-2-linoleoyl-GPC (18:0/18:2)*	10847	-1.651	0.10	-??---	0	0.56	0.48	5139	-0.129	0.90	-??+--	9.77E-01	5708	-1.469	0.14	-??---	5.54E-01
100001605	catechol sulfate	13556	-1.644	0.10	+++---	43.9	0.11	0.48	6404	-0.868	0.39	+-+---	9.04E-01	7151	-1.417	0.16	-+----	5.64E-01
100003434	imidazole propionate	10847	1.644	0.10	+??+++	4.2	0.37	0.48	5139	-0.234	0.82	+??+-+	9.54E-01	5708	2.218	0.03	+??+++	3.43E-01
100002875	1-(1-enyl-palmitoyl)-GPC (P-16:0)*	10847	-1.64	0.10	-??+--	57.7	0.07	0.49	5139	0.031	0.98	+??++-	9.92E-01	5708	-1.621	0.10	-??---	5.17E-01
100001437	cysteine-glutathione disulfide	12592	-1.638	0.10	-?-+--	62.1	0.03	0.49	5980	0.182	0.86	-?-++-	9.68E-01	6611	-2.108	0.04	-?----	3.51E-01
100001207	4-imidazoleacetate	5085	1.631	0.10	-??+??	0	0.53	0.49	2420	1.589	0.11	-??+??	7.92E-01	2665	0.864	0.39	+??+??	7.70E-01
100005717	1-palmitoyl-GPG (16:0)*	10847	1.633	0.10	+??+++	0	0.65	0.49	5139	-0.876	0.38	+??-+-	9.04E-01	5708	2.654	7.95E-03	+??+++	2.32E-01
100009147	1-stearyl-GPC (O-18:0)*	9802	-1.626	0.10	???--?	0	0.42	0.49	4642	-0.479	0.63	???--?	9.47E-01	5160	-1.471	0.14	???--?	5.54E-01
100015837	arachidonoylcarnitine (C20:4)	6208	-1.623	0.10	-???--	16.8	0.30	0.49	2932	0.399	0.69	-???+-	9.48E-01	3276	-1.853	0.06	-???--	4.25E-01
100015838	eicosenoylcarnitine (C20:1)*	6208	-1.617	0.11	+???--	60.2	0.08	0.49	2932	0.58	0.56	+???+-	9.44E-01	3276	-2.107	0.04	+???--	3.51E-01
935	sucrose	10847	1.61	1.08E-01	+??+-+	37.8	0.19	4.99E-01	5139	0.542	0.59	+??+--	9.44E-01	5708	1.593	0.11	+??+-+	5.29E-01
100015846	nervonoylcarnitine (C24:1)*	6208	1.605	0.11	+???+-	75	0.02	0.50	2932	1.467	0.14	+???+-	7.92E-01	3276	1.074	0.28	-???+-	6.73E-01
100008955	tricosanoyl sphingomyelin (d18:1/23:0)*	6208	-1.599	0.11	-???--	64.9	0.06	0.50	2932	-1.67	0.09	+???--	7.92E-01	3276	-0.177	0.86	-???+-	9.68E-01
100001034	indoleacetate	13556	-1.597	0.11	++----	27.9	0.23	0.50	6404	-0.555	0.58	+--+--	9.44E-01	7151	-1.627	0.10	-+----	5.14E-01
100000672	1-myristoyl-2-palmitoyl-GPC (14:0/16:0)	10401	1.591	0.11	???++-	0	0.52	0.51	4926	0.432	0.67	???++-	9.48E-01	5475	1.859	0.06	???+++	4.25E-01
100009139	1-myristoyl-2-arachidonoyl-GPC (14:0/20:4)*	10401	1.571	0.12	???++-	0	0.52	0.52	4926	0.951	0.34	???++-	9.04E-01	5475	1.821	0.07	???+++	4.33E-01
100010950	stearoyl-arachidonoyl-glycerol (18:0/20:4) [2]*	1045	-1.563	0.12	-????-	38.9	0.20	0.52	497	-1.795	0.07	-????-	7.73E-01	548	-0.745	0.46	+????-	7.97E-01
100002227	4-cholesten-3-one	10248	-1.562	0.12	+??--?	0	0.69	0.52	4855	-1.123	0.26	+??--?	8.69E-01	5393	-0.684	0.49	-??+-?	8.22E-01
815	tyrosine	13556	-1.562	0.12	--+---	57.4	0.04	0.52	6404	-0.088	0.93	+-++--	9.92E-01	7151	-1.575	0.12	-++---	5.30E-01
1528	1-palmitoyl-2-linoleoyl-GPI (16:0/18:2)	6208	1.559	0.12	+???+-	0	0.64	0.52	2932	0.888	0.37	+???+-	9.04E-01	3276	1.681	0.09	+???++	4.89E-01
100010869	2,3-dihydroxy-2-methylbutyrate	6208	-1.557	0.12	-???-+	57.3	0.10	0.52	2932	0.013	0.99	+???-+	9.94E-01	3276	-1.794	0.07	-???-+	4.47E-01

100003000	1-(1-enyl-palmitoyl)-GPE (P-16:0)*	10847	-1.551	0.12	-??---	29.8	0.23	0.52	5139	-0.586	0.56	+??---	9.44E-01	5708	-1.105	0.27	-??+--	6.61E-01
100001509	2-methylbutyrylcarnitine (C5)	8917	-1.55	0.12	---?--	0	0.74	0.52	4197	-0.483	0.63	+--?+-	9.47E-01	4719	-1.294	0.20	---?-+	6.13E-01
100006051	myristoleoylcarnitine (C14:1)*	12592	-1.543	0.12	+?----	6.5	0.37	0.53	5980	0.749	0.45	+?+++-	9.27E-01	6611	-2.143	0.03	+?----	3.51E-01
100000870	saccharin	10401	1.532	0.13	???+++	0	0.77	0.53	4926	1.464	0.14	???+++	7.92E-01	5475	0.76	0.45	???+++	7.97E-01
1025	pipecolate	13556	1.536	0.12	+-+++-	48.3	0.08	0.53	6404	1.638	0.10	-++++-	7.92E-01	7151	0.508	0.61	+--++-	8.74E-01
1125	5,6-dihydrouracil	1045	1.531	0.13	+????+	0	0.74	0.53	497	0.423	0.67	+????+	9.48E-01	548	1.962	0.05	+????+	4.00E-01
100001256	N-acetylphenylalanine	11811	1.535	0.12	++?-++	35.7	0.18	0.53	5563	0.05	0.96	++?+-+	9.92E-01	6248	1.722	0.08	++?-++	4.77E-01
100001064	glycolithocholate	10847	-1.529	0.13	-??--+	0	0.91	0.53	5139	-0.978	0.33	-??---	9.04E-01	5708	-1.118	0.26	-??--+	6.61E-01
100006298	lignoceroyl sphingomyelin (d18:1/24:0)	6208	-1.521	0.13	-???--	62.9	0.07	0.53	2932	-1.334	0.18	-???--	8.30E-01	3276	-0.373	0.71	-???+-	9.15E-01
100001040	1-linoleoylglycerol (18:2)	12592	-1.519	0.13	-?----	0	0.95	0.53	5980	-1.081	0.28	-?----	8.77E-01	6611	-0.846	0.40	-?+---	7.81E-01
100002990	oleoyl-linoleoyl-glycerol (18:1/18:2) [2]	10847	1.519	0.13	+??+++	0	0.99	0.53	5139	0.83	0.41	-??+++	9.04E-01	5708	1.882	0.06	+??+++	4.23E-01
100006627	suberoylcarnitine (C8-DC)	1045	1.511	0.13	+????+	0	0.53	0.53	497	0.051	0.96	+????-	9.92E-01	548	1.909	0.06	+????+	4.19E-01
100001208	1-methylimidazoleacetate	10847	1.495	0.13	-??-++	23.3	0.27	0.54	5139	1.454	0.15	-??+++	8.00E-01	5708	0.745	0.46	-??-++	7.97E-01
100006438	citraconate/glutaconate	10847	-1.499	0.13	+??---	0	0.40	0.54	5139	-0.846	0.40	+??---	9.04E-01	5708	-1.405	0.16	-??---	5.64E-01
535	uridine	13556	-1.485	0.14	---+--	16.7	0.31	0.54	6404	-0.644	0.52	---++-	9.44E-01	7151	-1.076	0.28	+-----	6.73E-01
100015832	behenoylcarnitine (C22)*	6208	-1.486	0.14	-???--	73.5	0.02	0.54	2932	0.308	0.76	-???+-	9.54E-01	3276	-1.746	0.08	-???--	4.67E-01
100001073	androsterone sulfate	13556	-1.482	0.14	------	19.7	0.29	0.55	6404	-2.017	0.04	------	6.34E-01	7151	-0.778	0.44	-++---	7.95E-01
100006294	behenoyl sphingomyelin (d18:1/22:0)*	6208	-1.475	0.14	-???--	51.2	0.13	0.55	2932	-1.246	0.21	-???--	8.48E-01	3276	-0.278	0.78	+???+-	9.40E-01
100001501	oleoylcarnitine (C18:1)	13556	-1.459	0.14	++----	53.9	0.05	0.55	6404	1.387	0.17	++-++-	8.12E-01	7151	-2.372	0.02	++----	2.98E-01
100008989	1-palmitoyl-2-eicosapentaenoyl-GPC (16:0/20:5)*	9802	1.464	0.14	???++?	0	0.48	0.55	4642	0.836	0.40	???++?	9.04E-01	5160	1.513	0.13	???++?	5.48E-01
100008993	1-palmitoyl-2-arachidonoyl-GPI (16:0/20:4)*	10847	1.453	0.15	+??++-	0	0.58	0.55	5139	0.457	0.65	+??-+-	9.48E-01	5708	1.951	0.05	+??++-	4.02E-01
100001613	tetradecanedioate	11811	-1.453	0.15	+-?---	0	0.53	0.55	5563	0.35	0.73	+-?++-	9.52E-01	6248	-2.175	0.03	++?---	3.46E-01
100000774	phenyllactate (PLA)	13556	-1.469	0.14	+-+---	23.9	0.25	0.55	6404	-0.215	0.83	+-++--	9.54E-01	7151	-1.584	0.11	--+---	5.30E-01
100015836	ximenoylcarnitine (C26:1)*	6208	-1.458	0.14	-???--	63.6	0.06	0.55	2932	-0.298	0.77	+???+-	9.54E-01	3276	-1.022	0.31	-???--	6.96E-01
800	cysteine	12592	-1.455	0.15	-?+---	0	0.79	0.55	5980	-0.153	0.88	-?++--	9.76E-01	6611	-1.468	0.14	-?+--+	5.54E-01
100001022	threonate	12592	-1.446	0.15	-?--+-	58.6	0.05	0.55	5980	-1.334	0.18	-?----	8.30E-01	6611	-0.632	0.53	-?+-+-	8.38E-01
480	proline	13556	1.445	0.15	-+++-+	41.7	0.13	0.55	6404	0.6	0.55	-+++--	9.44E-01	7151	1.683	0.09	-+++++	4.89E-01
358	hypotaurine	10847	1.439	0.15	-??-+-	17.8	0.30	0.55	5139	1.577	0.11	-??+++	7.92E-01	5708	0.251	0.80	-??-+-	9.49E-01
100001397	1,3,7-trimethylurate	10847	-1.44	0.15	+??---	0	0.70	0.55	5139	-1.342	0.18	+??+-+	8.30E-01	5708	-0.986	0.32	+??-+-	7.12E-01
100006129	vanillactate	6208	1.435	0.15	+???+-	73.7	0.02	0.55	2932	2.427	0.02	+???+-	5.04E-01	3276	0.027	0.98	+???--	9.94E-01
100001662	deoxycarnitine	11811	-1.425	0.15	-+?---	0	0.91	0.56	5563	-0.556	0.58	+-?+--	9.44E-01	6248	-1.203	0.23	-+?--+	6.40E-01
100000616	1-stearoyl-2-arachidonoyl-GPI (18:0/20:4)	10847	1.414	0.16	+??++-	0	0.89	0.57	5139	0.808	0.42	-??+++	9.11E-01	5708	1.793	0.07	+??++-	4.47E-01
100010930	palmitoleoyl-linoleoyl-glycerol (16:1/18:2) [1]*	6208	1.41	0.16	+???++	0	0.82	0.57	2932	0.624	0.53	+???+-	9.44E-01	3276	1.844	0.07	+???++	4.25E-01
100003696	succinimide	10401	-1.402	0.16	???+--	27.2	0.25	0.57	4926	-0.32	0.75	???+--	9.54E-01	5475	-1.397	0.16	???--+	5.64E-01
100001126	gamma-glutamylvaline	13556	-1.399	0.16	+----+	0.3	0.41	0.57	6404	-0.036	0.97	+-++--	9.92E-01	7151	-1.163	0.24	-----+	6.41E-01
100001167	pro-hydroxy-pro	13556	-1.395	0.16	++---+	0	0.76	0.57	6404	-1.438	0.15	++----	8.02E-01	7151	-0.103	0.92	++--++	9.78E-01
100000882	3-hydroxymyristate	1045	-1.393	0.16	-????-	0	0.55	0.57	497	-0.157	0.88	+????-	9.76E-01	548	-1.666	0.10	-????-	4.94E-01
100008918	1-(1-enyl-stearoyl)-2-arachidonoyl-GPC (P-18:0/20:4)	9802	-1.386	0.17	???--?	0	0.35	0.58	4642	0.493	0.62	???++?	9.47E-01	5160	-1.707	0.09	???--?	4.85E-01
100015882	glycosyl ceramide (d18:1/20:0, d16:1/22:0)*	6208	-1.384	0.17	+???--	28.2	0.25	0.58	2932	-1.748	0.08	+???--	7.84E-01	3276	-0.189	0.85	+???--	9.68E-01
1442	beta-hydroxyisovalerate	13556	-1.377	0.17	--+---	0	0.47	0.59	6404	-1.267	0.21	++----	8.36E-01	7151	-0.8	0.42	--+-++	7.89E-01
100009271	3-hydroxybutyrylcarnitine (2)	10847	-1.373	0.17	+??--+	0	0.48	0.59	5139	0.202	0.84	-??+-+	9.57E-01	5708	-1.879	0.06	+??---	4.23E-01
49	putrescine	1045	-1.367	0.17	-????-	0	0.76	0.59	497	-1.798	0.07	-????-	7.73E-01	548	-0.09	0.93	+????-	9.78E-01
100002871	1-adrenoyl-GPC (22:4)*	9802	1.368	0.17	???++?	0	0.44	0.59	4642	1.172	0.24	???-+?	8.59E-01	5160	1.488	0.14	???++?	5.54E-01
112	3-hydroxy-3-methylglutarate	10847	1.361	0.17	+??+-+	75.1	0.01	0.59	5139	1.989	0.05	+??+-+	6.51E-01	5708	0.12	0.90	+??+-+	9.77E-01
100004110	3-methyl catechol sulfate (2)	10847	-1.347	0.18	+??-+-	0	0.40	0.59	5139	-0.893	0.37	+??---	9.04E-01	5708	-1.155	0.25	-??-+-	6.43E-01
100009403	1-eicosapentaenoylglycerol (20:5)*	5684	1.355	0.18	+??+?-	0	0.58	0.59	2704	1.005	0.31	+??+?+	9.04E-01	2980	0.863	0.39	-??+?-	7.70E-01
892	nonadecanoate (19:0)	13556	-1.353	0.18	+--+--	50.7	0.07	0.59	6404	0.559	0.58	--+++-	9.44E-01	7151	-1.87	0.06	+-----	4.25E-01
100001810	dimethylarginine (SDMA + ADMA)	12592	-1.35	0.18	-?---+	0	0.88	0.59	5980	0.129	0.90	+?++--	9.77E-01	6611	-1.55	0.12	-?---+	5.36E-01
100006726	linoleoyl ethanolamide	10847	-1.353	0.18	-??--+	0	0.74	0.59	5139	0.036	0.97	+??--+	9.92E-01	5708	-1.707	0.09	-??---	4.85E-01
100003432	dihydroferulic acid	10401	-1.34	0.18	???---	43.6	0.17	0.60	4926	-0.671	0.50	???+--	9.44E-01	5475	-1.225	0.22	???-+-	6.31E-01

100001033	beta-sitosterol	5762	-1.335	0.18	????--	76.4	0.04	0.60	2719	1.278	0.20	????+-	8.30E-01	3043	-2.471	0.01	????--	2.81E-01
100001257	N-acetylasparagine	10847	1.336	0.18	-??+++	0	0.85	0.60	5139	0.984	0.33	-??++-	9.04E-01	5708	0.924	0.36	-??-++	7.42E-01
1004	xanthine	13556	-1.33	0.18	---+-+	35.2	0.17	0.60	6404	-0.103	0.92	+--+-+	9.88E-01	7151	-1.461	0.14	---+-+	5.54E-01
100001466	3-methylcytidine	6208	1.328	0.18	+???++	0	0.89	0.60	2932	1.552	0.12	+???++	7.92E-01	3276	0.479	0.63	-???++	8.80E-01
100002122	3-hydroxyhippurate	11811	-1.325	0.19	++?---	32.7	0.20	0.60	5563	-2.136	0.03	++?---	6.08E-01	6248	-0.194	0.85	-+?-+-	9.68E-01
100004541	acisoga	13556	-1.318	0.19	-----+	0	0.60	0.61	6404	0.886	0.38	-+++-+	9.04E-01	7151	-2.094	0.04	------	3.51E-01
100008956	sphingomyelin (d18:2/23:0, d18:1/23:1, d17:1/24:1)*	6208	-1.311	0.19	-???--	65.2	0.06	0.61	2932	-0.812	0.42	+???+-	9.11E-01	3276	-0.594	0.55	-???--	8.50E-01
100009069	1-(1-enyl-palmitoyl)-2-linoleoyl-GPE (P-16:0/18:2)*	10847	-1.312	0.19	-??--+	0	0.73	0.61	5139	-0.181	0.86	-??-+-	9.68E-01	5708	-1.127	0.26	-??--+	6.61E-01
100000657	1,2-dipalmitoyl-GPC (16:0/16:0)	10847	1.308	0.19	-??++-	8	0.35	0.61	5139	1.214	0.22	-??++-	8.59E-01	5708	0.755	0.45	-??++-	7.97E-01
1084	N-acetylvaline	10847	1.299	0.19	+??+++	0	0.99	0.62	5139	1.699	0.09	+??++-	7.89E-01	5708	0.482	0.63	-??-++	8.80E-01
356	cortisol	13556	-1.299	0.19	+--+++	80.8	0.00	0.62	6404	0.917	0.36	+--+++	9.04E-01	7151	-2.213	0.03	+-----	3.43E-01
100009142	1-stearoyl-2-docosapentaenoyl-GPC (18:0/22:5n6)*	9802	1.302	0.19	???++?	0	0.97	0.62	4642	0.673	0.50	???++?	9.44E-01	5160	1.496	0.13	???++?	5.51E-01
818	malonate	9802	-1.299	0.19	???--?	40.6	0.19	0.62	4642	-0.276	0.78	???-+?	9.54E-01	5160	-1.306	0.19	???--?	6.12E-01
100002014	5alpha-pregnan-3beta,20alpha-diol monosulfate (2)	10847	-1.293	0.20	+??---	0	0.40	0.62	5139	-1.926	0.05	+??---	7.03E-01	5708	-1.289	0.20	-??-+-	6.16E-01
100000708	isovalerate	11365	-1.287	0.20	?-?---	0	0.94	0.62	5350	-1.164	0.24	?-?+-+	8.59E-01	6015	-0.733	0.46	?+?-+-	7.98E-01
100009335	dihomo-linolenoyl-choline	10847	-1.287	0.20	-??---	50	0.11	0.62	5139	-1.058	0.29	-??---	8.86E-01	5708	-0.543	0.59	-??++-	8.62E-01
100006651	3,4-methyleneheptanoate	10401	-1.274	0.20	???--+	52.6	0.12	0.63	4926	-0.714	0.48	???--+	9.37E-01	5475	-1.095	0.27	???--+	6.62E-01
100003674	prolylglycine	10847	-1.269	0.20	+??-+-	48.8	0.12	0.63	5139	-0.47	0.64	+??-+-	9.47E-01	5708	-1.038	0.30	-??-+-	6.91E-01
100009025	sphingomyelin (d18:1/21:0, d17:1/22:0, d16:1/23:0)*	6208	-1.267	0.21	+???--	72.5	0.03	0.63	2932	-1.486	0.14	-???--	7.92E-01	3276	0.104	0.92	+???--	9.78E-01
407	lysine	13556	-1.245	0.21	--++--	32.7	0.19	0.63	6404	-1.519	0.13	--+---	7.92E-01	7151	-0.411	0.68	--++-+	9.06E-01
100002063	1-docosapentaenoylglycerophosphocholine (22:5n3)*	10766	1.26	0.21	?+?+-?	0	0.41	0.63	5066	1.192	0.23	?+?++?	8.59E-01	5700	1.547	0.12	?+?+-?	5.36E-01
891	margarate (17:0)	13556	-1.26	0.21	+--+--	3.3	0.40	0.63	6404	1.102	0.27	+-+++-	8.74E-01	7151	-2.08	0.04	+-----	3.51E-01
100009138	1-myristoyl-2-linoleoyl-GPC (14:0/18:2)*	9802	1.251	0.21	???++?	6.7	0.30	0.63	4642	0.904	0.37	???++?	9.04E-01	5160	1.188	0.23	???-+?	6.40E-01
278	cysteinylglycine	10847	-1.248	0.21	-??--+	36	0.20	0.63	5139	-0.854	0.39	-??---	9.04E-01	5708	-0.756	0.45	-??-++	7.97E-01
100015620	lactosyl-N-nervonoyl-sphingosine (d18:1/24:1)*	6208	-1.25	0.21	+???--	0	0.45	0.63	2932	-0.524	0.60	+???--	9.44E-01	3276	-1.065	0.29	-???--	6.79E-01
100006092	tyramine O-sulfate	10847	-1.256	0.21	-??---	0	0.71	0.63	5139	0.255	0.80	+??+-+	9.54E-01	5708	-1.753	0.08	-??-+-	4.64E-01
2054	ethylmalonate	10847	1.243	0.21	-??+++	0	0.74	0.63	5139	0.243	0.81	-??-++	9.54E-01	5708	1.457	0.15	+??+++	5.54E-01
100004561	N-formylanthranilic acid	6208	-1.236	0.22	-???--	0	0.77	0.64	2932	-0.638	0.52	-???--	9.44E-01	3276	-1.219	0.22	-???--	6.31E-01
100005384	O-sulfo-L-tyrosine	13556	-1.231	0.22	++----	0	0.63	0.64	6404	-0.292	0.77	+-++-+	9.54E-01	7151	-1.162	0.25	++----	6.41E-01
100003151	linoleoylcarnitine (C18:2)*	10847	-1.229	0.22	-??---	0	0.58	0.64	5139	1.678	0.09	-??++-	7.92E-01	5708	-2.45	0.01	-??---	2.86E-01
1235	gamma-glutamylhistidine	10847	1.224	0.22	+??++-	0	0.42	0.64	5139	0.9	0.37	+??++-	9.04E-01	5708	0.991	0.32	-??+++	7.12E-01
100001277	10-nonadecenoate (19:1n9)	13556	-1.217	0.22	+-----	0	0.46	0.65	6404	1.088	0.28	+-+++-	8.76E-01	7151	-1.984	0.05	+-----	3.88E-01
100001956	N-methylproline	11811	-1.217	0.22	-+?---	0	0.41	0.65	5563	-0.616	0.54	--?-+-	9.44E-01	6248	-0.978	0.33	-+?--+	7.16E-01
100008929	2-methylcitrate/homocitrate	1045	-1.214	0.22	+????-	76.2	0.04	0.65	497	-0.411	0.68	+????-	9.48E-01	548	-1.235	0.22	+????-	6.29E-01
1224	cys-gly, oxidized	10847	-1.213	0.22	-??-+-	51.9	0.10	0.65	5139	-0.292	0.77	-??-+-	9.54E-01	5708	-0.822	0.41	-??-++	7.84E-01
100001541	2-hydroxy-3-methylvalerate	11811	-1.207	0.23	--?---	0	0.94	0.65	5563	-0.446	0.66	++?+--	9.48E-01	6248	-1.03	0.30	--?---	6.93E-01
100000042	3-methylhistidine	11811	-1.208	0.23	-+?---	0	0.90	0.65	5563	-0.382	0.70	--?-+-	9.52E-01	6248	-0.884	0.38	-+?---	7.62E-01
100001324	ADpSGEGDFXAEGGGVR*	7953	-1.202	0.23	-?-?-+	0	0.41	0.65	3773	-0.285	0.78	-?-?++	9.54E-01	4179	-1.523	0.13	-?-?--	5.46E-01
100015727	ceramide (d16:1/24:1, d18:1/22:1)*	1045	1.2	0.23	+????+	0	0.44	0.65	497	0.936	0.35	+????+	9.04E-01	548	0.762	0.45	+????+	7.97E-01
100009338	5-bromotryptophan	10248	-1.195	0.23	-??--?	0	0.97	0.65	4855	-1.541	0.12	-??--?	7.92E-01	5393	-0.518	0.60	-??--?	8.70E-01
338	gluconate	10847	-1.195	0.23	+??---	0	0.77	0.65	5139	-0.338	0.74	+??+--	9.52E-01	5708	-1.085	0.28	-??--+	6.66E-01
100001314	gamma-glutamylthreonine	12592	-1.182	0.24	-?+---	23.3	0.27	0.66	5980	-1.377	0.17	-?+---	8.20E-01	6611	-0.386	0.70	-?+--+	9.11E-01
100002027	androstenediol (3alpha, 17alpha) monosulfate (3)	10847	-1.179	0.24	-??---	0	0.40	0.66	5139	-0.862	0.39	-??--+	9.04E-01	5708	-1.199	0.23	-??---	6.40E-01
482	lactate	13556	1.183	0.24	++++-+	49.8	0.08	0.66	6404	0.893	0.37	-+++-+	9.04E-01	7151	0.915	0.36	++++-+	7.47E-01
100015745	glycosyl ceramide (d18:2/24:1, d18:1/24:2)*	6208	-1.181	0.24	+???--	0	0.48	0.66	2932	-0.61	0.54	+???--	9.44E-01	3276	-1.055	0.29	-???--	6.84E-01
922	N-stearoyl-sphinganine (d18:0/18:0)*	1045	1.182	0.24	+????+	0	0.53	0.66	497	-0.412	0.68	+????-	9.48E-01	548	2.186	0.03	+????+	3.46E-01
100000285	N-alpha-acetylornithine	1045	-1.179	0.24	-????-	0	0.51	0.66	497	-0.054	0.96	+????-	9.92E-01	548	-1.602	0.11	-????-	5.23E-01
273	cortisone	13556	-1.184	0.24	+--+++	68.8	0.01	0.66	6404	-0.019	0.99	+--+++	9.94E-01	7151	-1.405	0.16	+---+-	5.64E-01
100009009	1-(1-enyl-palmitoyl)-2-linoleoyl-GPC (P-16:0/18:2)*	10847	-1.173	0.24	-??+--	44.3	0.15	0.66	5139	1.361	0.17	-??++-	8.21E-01	5708	-2.648	8.11E-03	-??---	2.32E-01
798	adenosine	1045	1.157	0.25	+????+	0	0.85	0.66	497	1.191	0.23	+????+	8.59E-01	548	0.796	0.43	+????+	7.91E-01

100006282	umbelliferone sulfate	10847	-1.171	0.24	-??-+-	41.1	0.17	0.66	5139	0.853	0.39	+??++-	9.04E-01	5708	-2.264	0.02	-??---	3.20E-01
100002356	17-methylstearate	11811	-1.166	0.24	+-?+--	63.4	0.03	0.66	5563	0.979	0.33	+-?++-	9.04E-01	6248	-1.998	0.05	+-?---	3.79E-01
1221	creatine	13556	-1.175	0.24	++---+	0	0.44	0.66	6404	-1.028	0.30	++----	9.04E-01	7151	-0.691	0.49	++---+	8.21E-01
100004555	benzoylcarnitine*	1045	-1.162	0.25	+????-	74.8	0.05	0.66	497	-0.88	0.38	+????-	9.04E-01	548	-0.628	0.53	+????-	8.40E-01
100000295	tartarate	1045	-1.175	0.24	-????-	0	0.48	0.66	497	-0.806	0.42	-????+	9.11E-01	548	-1.286	0.20	+????-	6.16E-01
100002989	oleoyl-linoleoyl-glycerol (18:1/18:2) [1]	10847	1.155	0.25	+??+++	0	0.98	0.66	5139	0.723	0.47	-??+++	9.37E-01	5708	1.434	0.15	+??+++	5.64E-01
100015967	carotene diol (2)	6208	-1.161	0.25	+???--	69.9	0.04	0.66	2932	0.705	0.48	+???+-	9.39E-01	3276	-2.004	0.05	-???--	3.79E-01
100002102	N-acetyl-beta-alanine	11811	1.157	0.25	+-?-++	14.9	0.32	0.66	5563	0.664	0.51	++?-++	9.44E-01	6248	0.926	0.35	+-?-++	7.42E-01
100004182	3b-hydroxy-5-cholenoic acid	1045	1.159	0.25	+????+	0	0.81	0.66	497	0.654	0.51	+????+	9.44E-01	548	0.749	0.45	+????+	7.97E-01
342	glycocholate	13556	-1.157	0.25	----++	0	0.62	0.66	6404	0.342	0.73	-++-++	9.52E-01	7151	-1.604	0.11	-----+	5.23E-01
100010941	linoleoyl-linoleoyl-glycerol (18:2/18:2) [1]*	6208	1.157	0.25	-???++	0	0.85	0.66	2932	0.358	0.72	-???++	9.52E-01	3276	1.428	0.15	+???++	5.64E-01
100000998	citramalate	9802	1.164	0.24	???++?	0	0.42	0.66	4642	0.153	0.88	???+-?	9.76E-01	5160	1.344	0.18	???++?	5.89E-01
1239	2-hydroxystearate	13556	-1.171	0.24	++----	0	0.81	0.66	6404	0.105	0.92	++---+	9.88E-01	7151	-1.237	0.22	-++---	6.29E-01
1254	glycerol	12592	1.148	0.25	+?++++	0	0.64	0.67	5980	1.274	0.20	+?++++	8.30E-01	6611	0.724	0.47	+?+++-	8.03E-01
100001755	4-vinylphenol sulfate	13556	1.14	0.25	-++++-	41.9	0.13	0.67	6404	1.4	0.16	+++++-	8.12E-01	7151	0.406	0.68	-+--++	9.06E-01
100002876	1-(1-enyl-oleoyl)-GPC (P-18:1)*	9802	-1.139	0.25	???--?	0	0.58	0.67	4642	-0.274	0.78	???-+?	9.54E-01	5160	-0.889	0.37	???+-?	7.60E-01
100001264	1-margaroylglycerophosphocholine (17:0)	10766	-1.123	0.26	?-?--?	0	0.78	0.67	5066	1.207	0.23	?+?+-?	8.59E-01	5700	-1.732	0.08	?-?--?	4.72E-01
100006260	6-hydroxyindole sulfate	10847	-1.128	0.26	+??---	6.6	0.36	0.67	5139	-1.132	0.26	+??+--	8.69E-01	5708	-0.742	0.46	+??-+-	7.98E-01
1102	gamma-glutamyltyrosine	13556	-1.125	0.26	-++---	0	0.78	0.67	6404	0.89	0.37	++++--	9.04E-01	7151	-1.442	0.15	-+----	5.64E-01
100001129	O-acetylhomoserine	5238	1.134	0.26	???+?-	0	0.59	0.67	2491	0.494	0.62	???+?-	9.47E-01	2747	0.97	0.33	???+?+	7.20E-01
100003470	pregnanediol-3-glucuronide	11811	-1.129	0.26	+-?-+-	45.6	0.12	0.67	5563	-0.065	0.95	+-?+++	9.92E-01	6248	-2.029	0.04	++?---	3.79E-01
100005834	9-hydroxystearate	5238	1.118	0.26	???+?+	76.7	0.04	0.68	2491	1.001	0.32	???+?+	9.04E-01	2747	0.496	0.62	???+?+	8.80E-01
100002028	androstenediol (3beta,17beta) monosulfate (1)	10847	1.114	0.27	+??++-	0	0.86	0.68	5139	0.085	0.93	-??+-+	9.92E-01	5708	1.093	0.27	+??++-	6.63E-01
100015845	docosahexaenoylcarnitine (C22:6)*	5762	-1.112	0.27	????--	77	0.04	0.68	2719	0.13	0.90	????+-	9.77E-01	3043	-1.246	0.21	????--	6.29E-01
100016038	(N(1) + N(8))-acetylspermidine	5762	-1.092	0.27	????--	0	0.71	0.70	2719	-1.065	0.29	????--	8.83E-01	3043	-0.573	0.57	????-+	8.55E-01
100006435	N-acetylglucosamine/N-acetylgalactosamine	10847	1.089	0.28	+??+++	52.8	0.10	0.70	5139	1.217	0.22	+??+++	8.59E-01	5708	0.543	0.59	-??+-+	8.62E-01
806	dimethylglycine	10847	-1.089	0.28	+??---	37.8	0.19	0.70	5139	-0.076	0.94	+??---	9.92E-01	5708	-0.983	0.33	+??--+	7.14E-01
229	arachidonate (20:4n6)	13556	1.079	0.28	-++++-	14.5	0.32	0.70	6404	0.862	0.39	-+--++	9.04E-01	7151	0.933	0.35	-++++-	7.42E-01
100009054	palmitoleoyl-oleoyl-glycerol (16:1/18:1) [2]*	1045	1.083	0.28	+????+	0	0.47	0.70	497	0.865	0.39	+????-	9.04E-01	548	0.717	0.47	+????+	8.05E-01
100001869	1-stearoyl-2-arachidonoyl-GPC (18:0/20:4)	10847	1.083	0.28	-??+++	47.6	0.13	0.70	5139	0.351	0.73	-??+++	9.52E-01	5708	1.764	0.08	-??+++	4.57E-01
100001313	gamma-glutamylmethionine	13556	-1.08	0.28	+--+-+	61.6	0.02	0.70	6404	0.325	0.75	++-+--	9.54E-01	7151	-1.391	0.16	+---+-+	5.64E-01
100001086	N-(2-furoyl)glycine	10847	-1.081	0.28	+??-+-	49.6	0.11	0.70	5139	-0.111	0.91	+??---	9.85E-01	5708	-1.316	0.19	-??-+-	6.06E-01
100001851	N-acetylserine	10847	1.075	0.28	+??++-	0	0.93	0.70	5139	1.332	0.18	+??++-	8.30E-01	5708	0.382	0.70	-??-++	9.13E-01
339	glutarate (pentanedioate)	10847	-1.073	0.28	+??--+	0	0.69	0.70	5139	-0.524	0.60	+??--+	9.44E-01	5708	-0.812	0.42	+??--+	7.85E-01
100008920	sphingomyelin (d18:1/17:0, d17:1/18:0, d19:1/16:0)	10847	-1.073	0.28	+??+--	60.7	0.05	0.70	5139	-0.625	0.53	+??+--	9.44E-01	5708	-0.45	0.65	+??+-+	8.98E-01
100015788	sphingomyelin (d18:2/18:1)*	6208	-1.067	0.29	-???--	36.7	0.21	0.70	2932	-0.735	0.46	-???+-	9.31E-01	3276	-0.556	0.58	-???--	8.62E-01
100015752	glycosyl-N-(2-hydroxynervonoyl)-sphingosine (d18:1/24:1(2OH))	5762	-1.043	0.30	????-+	35.5	0.21	0.71	2719	-2.623	8.72E-03	????-+	4.62E-01	3043	0.449	0.65	????++	8.98E-01
100004634	3-methoxytyramine sulfate	1045	1.053	0.29	+????-	71.8	0.06	0.71	497	1.794	0.07	+????-	7.73E-01	548	-0.138	0.89	-????-	9.77E-01
100010949	stearoyl-arachidonoyl-glycerol (18:0/20:4) [1]*	1045	-1.047	0.30	-????-	0	0.51	0.71	497	-1.724	0.08	-????-	7.84E-01	548	-0.231	0.82	+????-	9.55E-01
100001654	1-arachidonoyl-GPI (20:4)*	13556	1.041	0.30	++++--	5.3	0.38	0.71	6404	1.549	0.12	++-+++	7.92E-01	7151	0.476	0.63	+-++--	8.81E-01
376	isoleucine	13556	-1.042	0.30	+-+--+	65.2	0.01	0.71	6404	-1.628	0.10	+-+---	7.92E-01	7151	0.106	0.92	--+--+	9.78E-01
437	pelargonate (9:0)	13556	-1.046	0.30	-+----	0	0.92	0.71	6404	-1.566	0.12	----+-	7.92E-01	7151	0.081	0.94	++----	9.78E-01
100015966	carotene diol (1)	6208	-1.049	0.29	+???--	60.6	0.08	0.71	2932	1.086	0.28	+???+-	8.76E-01	3276	-2.031	0.04	-???--	3.79E-01
100001270	myristoylcarnitine (C14)	10847	-1.059	0.29	+??---	69.4	0.02	0.71	5139	0.721	0.47	+??++-	9.37E-01	5708	-1.378	0.17	+??---	5.67E-01
100002106	sphingomyelin (d18:1/18:1, d18:2/18:0)	10847	1.046	0.30	+??+--	0	0.41	0.71	5139	0.477	0.63	+??+--	9.47E-01	5708	1.381	0.17	-??+-+	5.66E-01
100009130	1-oleoyl-2-docosahexaenoyl-GPC (18:1/22:6)*	10401	1.039	0.30	???++-	13.2	0.32	0.71	4926	0.505	0.61	???++-	9.47E-01	5475	0.973	0.33	???++-	7.20E-01
100002113	cysteine sulfinic acid	10847	1.039	0.30	+??-++	75.2	0.01	0.71	5139	0.464	0.64	+??-++	9.48E-01	5708	1.09	0.28	+??+-+	6.64E-01
1140	gamma-glutamylglutamine	12592	-1.043	0.30	-?-+--	63.6	0.03	0.71	5980	0.08	0.94	-?-+--	9.92E-01	6611	-1.216	0.22	-?-+--	6.31E-01
100005985	sphingomyelin (d18:2/14:0, d18:1/14:1)*	10847	1.03	0.30	+??++-	54.5	0.09	0.72	5139	0.938	0.35	+??++-	9.04E-01	5708	1.266	0.21	-??+++	6.21E-01
100001540	pyroglutamine*	13556	1.028	0.30	++++-+	0	0.74	0.72	6404	2.733	6.28E-03	+++++-	4.54E-01	7151	-0.177	0.86	+-+--+	9.68E-01

100009021	1-palmityl-2-arachidonoyl-GPC (O-16:0/20:4)*	9802	-1.025	0.31	???--?	0	0.78	0.72	4642	0.031	0.98	???-+?	9.92E-01	5160	-1.008	0.31	???--?	6.99E-01
100009166	phosphatidylcholine (16:0/22:5n3, 18:1/20:4)*	9802	1.007	0.31	???-+?	8.3	0.30	0.73	4642	2.776	5.51E-03	???++?	4.54E-01	5160	-0.287	0.77	???-+?	9.39E-01
100000007	carnitine	13556	1.007	0.31	-+++-+	0	0.76	0.73	6404	1.239	0.22	+++++-	8.51E-01	7151	0.824	0.41	-+++-+	7.84E-01
100009141	1-stearoyl-2-docosapentaenoyl-GPC (18:0/22:5n3)*	9802	1.005	0.31	???+-?	27.2	0.24	0.73	4642	0.888	0.37	???++?	9.04E-01	5160	1.401	0.16	???++?	5.64E-01
100001007	ribonate	10248	-1.008	0.31	+??--?	53.6	0.12	0.73	4855	0.633	0.53	+??-+?	9.44E-01	5393	-1.633	0.10	+??--?	5.13E-01
100001102	dodecanedioate	11811	-1.009	0.31	++?---	50.8	0.09	0.73	5563	-0.099	0.92	+-?+--	9.89E-01	6248	-1.052	0.29	++?--+	6.84E-01
100001405	1-methylxanthine	12592	-1	0.32	+?++--	36.7	0.18	0.73	5980	-1.588	0.11	+?++--	7.92E-01	6611	-0.296	0.77	+?-++-	9.37E-01
100001383	1-myristoylglycerophosphocholine (14:0)	10766	1	0.32	?-?++?	0	0.82	0.73	5066	1.176	0.24	?+?++?	8.59E-01	5700	0.885	0.38	?-?++?	7.62E-01
100001566	1-docosahexaenoylglycerophosphocholine (22:6n3)*	10766	0.998	0.32	?+?+-?	0	0.55	0.73	5066	0.143	0.89	?+?--?	9.77E-01	5700	1.402	0.16	?-?++?	5.64E-01
100001526	malonylcarnitine	6208	0.996	0.32	+???++	0	0.56	0.73	2932	0.926	0.35	+???++	9.04E-01	3276	0.593	0.55	-???++	8.50E-01
100015790	sphingomyelin (d18:2/21:0, d16:2/23:0)*	6208	-0.985	0.32	+???--	39.8	0.19	0.74	2932	-0.64	0.52	+???--	9.44E-01	3276	-0.419	0.68	-???-+	9.04E-01
100000802	acetylcarnitine (C2)	13556	-0.981	0.33	+--+-+	0	0.70	0.75	6404	1.517	0.13	+-++++	7.92E-01	7151	-2.001	0.05	+----+	3.79E-01
100009334	palmitoloelycholine	6208	-0.979	0.33	+???--	58	0.09	0.75	2932	0.484	0.63	+???+-	9.47E-01	3276	-1.593	0.11	+???--	5.29E-01
100000846	erythritol	12592	0.969	0.33	-?+-++	35.6	0.18	0.76	5980	3.195	1.40E-03	+?++++	3.71E-01	6611	-0.782	0.43	-?+--+	7.93E-01
100004299	N-acetyl-1-methylhistidine*	10847	-0.967	0.33	-??---	0	0.68	0.76	5139	0.984	0.33	+??+--	9.04E-01	5708	-1.343	0.18	-??--+	5.89E-01
100005673	1-docosapentaenoyl-GPC (22:5n6)*	9802	0.963	0.34	???++?	0	0.36	0.76	4642	-0.39	0.70	???-+?	9.49E-01	5160	1.803	0.07	???++?	4.42E-01
100001620	glycerophosphoethanolamine	10847	-0.96	0.34	+??---	14.3	0.32	0.76	5139	-0.795	0.43	+??-+-	9.11E-01	5708	-0.079	0.94	+??++-	9.78E-01
100001398	3,7-dimethylurate	10847	-0.949	0.34	-??+--	0	0.68	0.77	5139	-1.164	0.24	-??+--	8.59E-01	5708	-0.407	0.68	-??---	9.06E-01
1538	stearoyl sphingomyelin (d18:1/18:0)	10847	0.949	0.34	+??+-+	59	0.06	0.77	5139	-0.072	0.94	+??+--	9.92E-01	5708	1.578	0.11	+??+-+	5.30E-01
452	palmitoleate (16:1n7)	13556	0.94	0.35	++++++	0	0.99	0.77	6404	2.295	0.02	++++++	5.76E-01	7151	-0.314	0.75	---+--	9.26E-01
100003260	carboxyethyl-GABA	6208	0.947	0.34	+???+-	0	0.72	0.77	2932	1.445	0.15	+???+-	8.00E-01	3276	0.292	0.77	-???++	9.39E-01
799	betaine	13556	-0.942	0.35	-+---+	14.1	0.32	0.77	6404	1.327	0.18	-+++++	8.30E-01	7151	-1.477	0.14	-+---+	5.54E-01
100001614	hexadecanedioate	11811	-0.941	0.35	+-?--+	58	0.05	0.77	5563	0.646	0.52	+-?+++	9.44E-01	6248	-1.784	0.07	+-?---	4.51E-01
893	arachidate (20:0)	11811	-0.937	0.35	+-?---	0	0.67	0.77	5563	0.237	0.81	--?+++	9.54E-01	6248	-1.182	0.24	+-?---	6.40E-01
100008921	1-palmitoyl-2-stearoyl-GPC (16:0/18:0)	10847	-0.933	0.35	-??-+-	54.3	0.09	0.77	5139	-0.564	0.57	-??-+-	9.44E-01	5708	-0.32	0.75	-??-+-	9.24E-01
100002953	16-hydroxypalmitate	6208	-0.931	0.35	+???--	20.3	0.29	0.77	2932	0.304	0.76	+???+-	9.54E-01	3276	-1.178	0.24	+???--	6.40E-01
803	mannose	12592	0.932	0.35	+?+--+	60.3	0.04	0.77	5980	0.051	0.96	+?+--+	9.92E-01	6611	1.117	0.26	+?++-+	6.61E-01
1489	palmitoyl ethanolamide	6208	0.927	0.35	+???+-	0	0.72	0.77	2932	1.679	0.09	+???++	7.92E-01	3276	0.029	0.98	+???+-	9.94E-01
100009055	palmitoyl-linoleoyl-glycerol (16:0/18:2) [2]*	10847	0.929	0.35	-??+++	65.9	0.03	0.77	5139	-0.134	0.89	-??+-+	9.77E-01	5708	1.833	0.07	-??+++	4.25E-01
100000406	ribitol	10847	0.923	0.36	+??+-+	0	0.39	0.77	5139	0.884	0.38	+??+++	9.04E-01	5708	0.825	0.41	+??+-+	7.84E-01
100001272	1-oleoyl-GPC (18:1)	13556	-0.923	0.36	+-----	0.9	0.41	0.77	6404	0.586	0.56	+++++-	9.44E-01	7151	-0.913	0.36	+---+--	7.47E-01
1242	N1-methyladenosine	13556	0.923	0.36	--++++	0	0.88	0.77	6404	0.644	0.52	-+++--	9.44E-01	7151	0.731	0.47	---+++	7.98E-01
355	histidine	13556	-0.918	0.36	---+--	10.8	0.35	0.78	6404	-1.895	0.06	---+--	7.41E-01	7151	0.063	0.95	--+++-	9.85E-01
1629	taurochenodeoxycholate	11811	-0.916	0.36	-+?---	0	0.92	0.78	5563	-0.505	0.61	-+?---	9.47E-01	6248	-0.821	0.41	+-?---	7.84E-01
100015731	N-palmitoyl-heptadecasphingosine (d17:1/16:0)*	6208	-0.908	0.36	+???--	0	0.44	0.78	2932	-2.756	5.85E-03	-???--	4.54E-01	3276	1.341	0.18	+???++	5.89E-01
100005350	1-linolenoyl-GPC (18:3)*	11811	-0.905	0.37	+-?---	0	0.43	0.79	5563	0.265	0.79	++?-+-	9.54E-01	6248	-0.712	0.48	+-?---	8.09E-01
100002462	5-(galactosylhydroxy)-L-lysine	1045	-0.878	0.38	+????-	0	0.41	0.79	497	-1.52	0.13	+????-	7.92E-01	548	0.331	0.74	-????+	9.24E-01
100004046	N-acetylcarnosine	11811	-0.881	0.38	+-?+--	0	0.43	0.79	5563	-1.536	0.12	+-?+--	7.92E-01	6248	0.082	0.93	--?++-	9.78E-01
439	stearate (18:0)	13556	-0.891	0.37	+-----	0	0.67	0.79	6404	1.424	0.15	++++++	8.12E-01	7151	-1.915	0.06	+-----	4.19E-01
100008957	sphingomyelin (d18:2/24:1, d18:1/24:2)*	10847	0.882	0.38	-??++-	53.2	0.09	0.79	5139	1.096	0.27	-??++-	8.74E-01	5708	0.424	0.67	-??++-	9.02E-01
100010917	palmitoyl-oleoyl-glycerol (16:0/18:1) [2]*	6208	0.883	0.38	+???++	0	0.62	0.79	2932	-0.831	0.41	-???--	9.04E-01	3276	2.346	0.02	+???++	2.98E-01
100009045	phenylacetylglutamate	1045	-0.887	0.37	-????-	0	0.85	0.79	497	-0.834	0.40	-????-	9.04E-01	548	-0.428	0.67	-????+	9.02E-01
100005864	methyl glucopyranoside (alpha + beta)	10847	-0.895	0.37	-??-+-	21.8	0.28	0.79	5139	0.82	0.41	+??-+-	9.08E-01	5708	-1.672	0.09	-??---	4.92E-01
100009331	oleoylcholine	10847	-0.883	0.38	-??+--	56.5	0.08	0.79	5139	-0.753	0.45	-??---	9.27E-01	5708	-0.375	0.71	-??++-	9.15E-01
100009332	arachidonoylcholine	10847	-0.881	0.38	-??+--	61.3	0.05	0.79	5139	-0.732	0.46	-??-+-	9.33E-01	5708	-0.35	0.73	-??+--	9.21E-01
100009225	1-(1-enyl-stearoyl)-2-linoleoyl-GPE (P-18:0/18:2)*	10847	-0.893	0.37	-??---	0	1.00	0.79	5139	0.648	0.52	-??++-	9.44E-01	5708	-1.128	0.26	-??--+	6.61E-01
100004083	glycohyocholate	10847	-0.889	0.37	-??-+-	40.2	0.17	0.79	5139	-0.571	0.57	-??-+-	9.44E-01	5708	-0.685	0.49	-??-+-	8.22E-01
2053	tricarballylate	9802	0.878	0.38	???++?	0	0.77	0.79	4642	0.569	0.57	???++?	9.44E-01	5160	0.593	0.55	???++?	8.50E-01
100008916	1-stearoyl-2-docosahexaenoyl-GPC (18:0/22:6)	10401	0.88	0.38	???+-+	0	0.41	0.79	4926	-0.345	0.73	???+-+	9.52E-01	5475	1.61	0.11	???+++	5.22E-01
100005986	sphingomyelin (d18:1/24:1, d18:2/24:0)*	6208	-0.89	0.37	-???--	0	0.81	0.79	2932	0.285	0.78	+???--	9.54E-01	3276	-1.011	0.31	-???--	6.98E-01

100006373	1,2,3-benzenetriol sulfate (1)	9802	0.873	0.38	???-+?	24.3	0.25	0.79	4642	0.261	0.79	???+-?	9.54E-01	5160	0.866	0.39	???-+?	7.70E-01
100009272	glycosyl-N-palmitoyl-sphingosine (d18:1/16:0)	10847	-0.874	0.38	+??+--	34.9	0.20	0.79	5139	-0.242	0.81	+??+--	9.54E-01	5708	-0.733	0.46	+??--+	7.98E-01
100010934	diacylglycerol (14:0/18:1, 16:0/16:1) [1]*	1045	0.893	0.37	+????-	54.3	0.14	0.79	497	-0.149	0.88	+????-	9.77E-01	548	1.553	0.12	+????+	5.36E-01
100009333	docosahexaenoylcholine	10847	-0.869	0.39	-??+--	57.7	0.07	0.79	5139	-1.293	0.20	+??---	8.30E-01	5708	0.022	0.98	-??++-	9.95E-01
100001988	5alpha-pregnan-3beta,20alpha-diol disulfate	11811	-0.865	0.39	+-?---	0	0.49	0.79	5563	-0.587	0.56	+-?-++	9.44E-01	6248	-1.715	0.09	--?---	4.82E-01
254	3-hydroxybutyrate (BHBA)	13556	-0.867	0.39	++----	0	0.48	0.79	6404	0.572	0.57	++-+++	9.44E-01	7151	-1.528	0.13	++----	5.44E-01
100001452	isovalerylglycine	10847	-0.864	0.39	-??-+-	58.4	0.07	0.79	5139	-1.434	0.15	-??---	8.04E-01	5708	0.084	0.93	-??-+-	9.78E-01
100000447	gentisate	10847	-0.858	0.39	-??-+-	31.5	0.22	0.79	5139	0.779	0.44	+??++-	9.11E-01	5708	-1.654	0.10	-??-+-	4.99E-01
240	3-(4-hydroxyphenyl)lactate	13556	-0.858	0.39	++++--	55.8	0.05	0.79	6404	0.142	0.89	+-++--	9.77E-01	7151	-0.75	0.45	-+++--	7.97E-01
926	caproate (6:0)	11365	-0.86	0.39	?+?+--	0	0.66	0.79	5350	0.076	0.94	?-?+-+	9.92E-01	6015	-0.833	0.40	?+?---	7.84E-01
100008998	gamma-tocopherol/beta-tocopherol	10847	-0.851	0.39	-??--+	0	0.67	0.80	5139	0.28	0.78	-??+-+	9.54E-01	5708	-0.815	0.42	-??-++	7.85E-01
100003686	N-palmitoylglycine	10847	0.849	0.40	+??+-+	42.1	0.16	0.80	5139	0.803	0.42	+??+-+	9.11E-01	5708	0.371	0.71	+??-+-	9.15E-01
100001262	gamma-glutamyl-epsilon-lysine	10847	-0.847	0.40	-??-+-	0	0.62	0.80	5139	0.227	0.82	-??++-	9.54E-01	5708	-1.201	0.23	+??---	6.40E-01
100001386	heme	7794	-0.841	0.40	----??	0	0.86	0.80	3685	-1.768	0.08	--+-??	7.73E-01	4108	0.118	0.91	-+-+??	9.77E-01
235	2-hydroxyphenylacetate	1045	-0.835	0.40	+????-	49.2	0.16	0.81	497	-1.865	0.06	+????-	7.73E-01	548	0.611	0.54	+????+	8.44E-01
100001806	o-cresol sulfate	10847	0.835	0.40	-??+-+	0	0.75	0.81	5139	0.597	0.55	+??+-+	9.44E-01	5708	0.646	0.52	-??++-	8.35E-01
565	tryptophan	13556	-0.838	0.40	--++--	0	0.43	0.81	6404	-0.259	0.80	+-++--	9.54E-01	7151	-0.815	0.42	---+--	7.85E-01
266	cholesterol	12592	0.8	0.42	+?++--	50.8	0.09	0.81	5980	2.084	0.04	+?+++-	6.18E-01	6611	0.114	0.91	+?-+--	9.77E-01
100001391	stearoylcarnitine (C18)	13556	-0.804	0.42	++-+--	71.7	0.00	0.81	6404	2.054	0.04	+++++-	6.24E-01	7151	-1.898	0.06	++----	4.20E-01
1256	choline	13556	-0.8	0.42	---++-	13.5	0.33	0.81	6404	-1.634	0.10	+-----	7.92E-01	7151	0.229	0.82	-+-++-	9.55E-01
100000445	theobromine	13556	-0.809	0.42	-+-+--	0	0.75	0.81	6404	-1.561	0.12	--++--	7.92E-01	7151	0.035	0.97	++-++-	9.92E-01
100010895	2'-O-methylcytidine	6208	-0.826	0.41	-???+-	78.6	0.01	0.81	2932	-1.477	0.14	-???--	7.92E-01	3276	-0.028	0.98	-???+-	9.94E-01
100000463	indolelactate	13556	0.829	0.41	-++++-	0	0.70	0.81	6404	1.303	0.19	++-+-+	8.30E-01	7151	0.104	0.92	-++++-	9.78E-01
100000841	oxalate (ethanedioate)	10847	0.805	0.42	-??++-	79.4	0.00	0.81	5139	1.164	0.24	-??++-	8.59E-01	5708	0.235	0.81	-??++-	9.54E-01
100008917	1-(1-enyl-stearoyl)-2-oleoyl-GPC (P-18:0/18:1)	9802	-0.82	0.41	???--?	0	0.77	0.81	4642	0.87	0.38	???++?	9.04E-01	5160	-1.582	0.11	???--?	5.30E-01
100003119	N-oleoyltaurine	1045	0.809	0.42	+????-	8.2	0.30	0.81	497	0.882	0.38	+????+	9.04E-01	548	0.321	0.75	+????-	9.24E-01
100009233	palmitoylcholine	10847	-0.796	0.43	-??+--	67.6	0.03	0.81	5139	-0.807	0.42	-??---	9.11E-01	5708	-0.332	0.74	-??++-	9.24E-01
100005383	1-methyl-2-piperidinecarboxylic acid	11365	-0.812	0.42	?-?---	0	0.92	0.81	5350	0.675	0.50	?+?++-	9.44E-01	6015	-1.226	0.22	?-?---	6.31E-01
1023	sarcosine	10847	-0.826	0.41	+??-+-	0	0.54	0.81	5139	0.558	0.58	+??-++	9.44E-01	5708	-1.03	0.30	-??---	6.93E-01
100000943	2-oleoylglycerol (18:1)	10248	-0.817	0.41	+??+-?	0	0.60	0.81	4855	-0.469	0.64	+??--?	9.47E-01	5393	-0.492	0.62	+??+-?	8.80E-01
100001563	2-myristoylglycerophosphocholine*	5603	-0.798	0.43	?-?-??	0	0.82	0.81	2631	-0.441	0.66	?+?-??	9.48E-01	2972	-0.569	0.57	?-?-??	8.59E-01
1135	ursodeoxycholate	10847	0.818	0.41	+??+++	0	0.60	0.81	5139	-0.311	0.76	+??+-+	9.54E-01	5708	1.285	0.20	-??-++	6.16E-01
100010922	linoleoyl-arachidonoyl-glycerol (18:2/20:4) [1]*	6208	0.81	0.42	-???+-	0	0.80	0.81	2932	-0.134	0.89	-???+-	9.77E-01	3276	1.909	0.06	+???++	4.19E-01
100008976	1-stearoyl-2-linoleoyl-GPE (18:0/18:2)*	10847	0.803	0.42	+??-++	0	0.72	0.81	5139	0.062	0.95	-??+-+	9.92E-01	5708	1.274	0.20	+??-++	6.20E-01
100002911	glycoursodeoxycholate	11811	0.809	0.42	-+?-++	36.6	0.18	0.81	5563	-0.082	0.93	++?---	9.92E-01	6248	1.273	0.20	-+?-++	6.20E-01
100001182	docosadienoate (22:2n6)	6648	0.791	0.43	+-?+?+	0	0.62	0.81	3128	2.125	0.03	++?+?+	6.08E-01	3520	-0.739	0.46	+-?-?-	7.98E-01
100001433	1-arachidonylglycerol (20:4)	10847	-0.789	0.43	-??+--	23	0.27	0.81	5139	0.101	0.92	-??+++	9.88E-01	5708	-0.737	0.46	-??+--	7.98E-01
100001296	stachydrine	13556	-0.786	0.43	--+---	6	0.38	0.81	6404	-0.343	0.73	+-+++-	9.52E-01	7151	-0.676	0.50	-++---	8.23E-01
100000924	1-oleoylglycerol (18:1)	13556	-0.785	0.43	++----	0	0.50	0.81	6404	-0.202	0.84	++----	9.57E-01	7151	-0.437	0.66	+---+-	9.01E-01
100000776	palmitoylcarnitine (C16)	13556	-0.783	0.43	++-+--	66.8	0.01	0.81	6404	1.117	0.26	++-++-	8.72E-01	7151	-1.196	0.23	+--+--	6.40E-01
100001415	N6-carbamoylthreonyladenosine	11811	-0.777	0.44	++?---	0	0.78	0.81	5563	-0.787	0.43	+-?-+-	9.11E-01	6248	0.088	0.93	++?--+	9.78E-01
100001026	galactonate	10847	0.774	0.44	+??+--	0	0.46	0.81	5139	0.354	0.72	-??++-	9.52E-01	5708	0.56	0.58	+??+--	8.61E-01
100010937	oleoyl-arachidonoyl-glycerol (18:1/20:4) [2]*	6208	0.774	0.44	-???++	0	0.66	0.81	2932	0.231	0.82	-???++	9.54E-01	3276	1.527	0.13	+???++	5.44E-01
100001446	5-methyluridine (ribothymidine)	11811	-0.769	0.44	+-?---	0	0.83	0.82	5563	-0.541	0.59	++?-+-	9.44E-01	6248	-0.543	0.59	+-?+--	8.62E-01
821	pseudouridine	13556	0.763	0.45	++++-+	0	0.64	0.82	6404	1.136	0.26	++++--	8.69E-01	7151	0.298	0.77	++---+	9.36E-01
100001994	androstenediol (3beta,17beta) disulfate (2)	13556	-0.765	0.44	--+---	0	0.68	0.82	6404	-1.019	0.31	+-+--+	9.04E-01	7151	-0.843	0.40	--+++-	7.81E-01
100002026	androstenediol (3alpha, 17alpha) monosulfate (2)	10847	-0.762	0.45	+??---	0	0.90	0.82	5139	-0.472	0.64	+??+-+	9.47E-01	5708	-0.745	0.46	+??---	7.97E-01
100010924	palmitoyl-arachidonoyl-glycerol (16:0/20:4) [1]*	6208	0.762	0.45	+???+-	0	0.49	0.82	2932	-0.363	0.72	+???-+	9.52E-01	3276	1.448	0.15	+???+-	5.61E-01
100001604	hydroquinone sulfate	10847	0.767	0.44	-??++-	13.7	0.32	0.82	5139	0.264	0.79	+??+-+	9.54E-01	5708	0.614	0.54	-??-+-	8.44E-01
100009014	1-(1-enyl-palmitoyl)-2-arachidonoyl-GPC (P-16:0/20:4)*	10847	-0.761	0.45	-??---	0	0.60	0.82	5139	-0.297	0.77	-??-+-	9.54E-01	5708	-0.317	0.75	-??+--	9.24E-01

100006056	N-formylphenylalanine	5238	0.756	0.45	???+?-	47.6	0.17	0.82	2491	0.542	0.59	???+?-	9.44E-01	2747	0.487	0.63	???+?-	8.80E-01
100001590	isobutyrylglycine	10847	0.751	0.45	-??++-	70.5	0.02	0.82	5139	0.403	0.69	-??+--	9.48E-01	5708	0.932	0.35	-??++-	7.42E-01
100001993	pregnen-diol disulfate C21H34O8S2*	11811	0.752	0.45	++?-+-	0	0.88	0.82	5563	0.304	0.76	++?-++	9.54E-01	6248	0.134	0.89	+-?++-	9.77E-01
1136	valerate	10401	-0.752	0.45	???+--	0	0.37	0.82	4926	-0.064	0.95	???+--	9.92E-01	5475	-0.751	0.45	???---	7.97E-01
100001485	gamma-glutamylisoleucine*	11811	-0.751	0.45	+-?--+	0	0.69	0.82	5563	-0.006	1.00	++?+--	9.96E-01	6248	-0.4	0.69	+-?-++	9.07E-01
512	taurine	10847	-0.748	0.45	-??+--	25.6	0.26	0.82	5139	0.435	0.66	-??+--	9.48E-01	5708	-1.046	0.30	-??---	6.87E-01
100001335	eicosenoate (20:1)	13556	-0.745	0.46	+--+--	0	0.72	0.83	6404	1.471	0.14	++++-+	7.92E-01	7151	-2.002	0.05	+-----	3.79E-01
563	glutamine	13556	0.742	0.46	-+-+-+	27.8	0.23	0.83	6404	-0.476	0.63	---+-+	9.47E-01	7151	1.644	0.10	++++-+	5.03E-01
100009153	1-stearoyl-2-meadoyl-GPC (18:0/20:3n9)*	9802	0.741	0.46	???++?	0	0.78	0.83	4642	0.488	0.63	???++?	9.47E-01	5160	0.914	0.36	???++?	7.47E-01
100001269	campesterol	6208	-0.742	0.46	-???--	32.5	0.23	0.83	2932	0.284	0.78	-???+-	9.54E-01	3276	-0.964	0.34	+???--	7.23E-01
100003901	2-stearoyl-GPE (18:0)*	10847	0.738	0.46	+??+--	49.8	0.11	0.83	5139	1.55	0.12	+??++-	7.92E-01	5708	0.443	0.66	+??+-+	9.00E-01
100002951	eicosanodioate	10847	-0.714	0.48	-??---	0	0.74	0.83	5139	-1.204	0.23	-??---	8.59E-01	5708	0.087	0.93	-??++-	9.78E-01
100015831	linolenoylcarnitine (C18:3)*	6208	-0.709	0.48	+???--	0	0.71	0.83	2932	1.041	0.30	+???++	9.00E-01	3276	-1.423	0.15	+???--	5.64E-01
100001569	1-oleoyl-GPE (18:1)	13556	0.722	0.47	+++-++	24.6	0.25	0.83	6404	0.832	0.41	-++-+-	9.04E-01	7151	0.616	0.54	+++-++	8.43E-01
100001635	ectoine	6208	0.734	0.46	+???+-	59.3	0.09	0.83	2932	-0.545	0.59	+???--	9.44E-01	3276	1.171	0.24	+???+-	6.40E-01
100001253	N-acetylglutamine	10847	-0.709	0.48	+??--+	0	0.74	0.83	5139	0.591	0.55	+??++-	9.44E-01	5708	-1.151	0.25	+??--+	6.46E-01
100004523	N-delta-acetylornithine	13556	-0.724	0.47	---++-	44.5	0.11	0.83	6404	0.604	0.55	--+-+-	9.44E-01	7151	-1.102	0.27	-+-+--	6.61E-01
1082	N-acetylleucine	10847	0.721	0.47	+??+-+	0	0.74	0.83	5139	0.542	0.59	+??+-+	9.44E-01	5708	0.482	0.63	-??-++	8.80E-01
100001051	1-methylhistidine	10847	-0.712	0.48	-??+--	0	0.61	0.83	5139	-0.64	0.52	+??+--	9.44E-01	5708	-0.154	0.88	-??+-+	9.72E-01
50	spermidine	6208	-0.716	0.47	-???--	0	0.86	0.83	2932	-0.488	0.63	-???+-	9.47E-01	3276	-0.439	0.66	+???-+	9.01E-01
100001412	N2,N2-dimethylguanosine	11811	-0.721	0.47	+-?--+	0	0.70	0.83	5563	0.344	0.73	+-?+--	9.52E-01	6248	-1.125	0.26	+-?-++	6.61E-01
100009181	1-stearoyl-2-oleoyl-GPI (18:0/18:1)*	6208	0.71	0.48	+???+-	0	0.81	0.83	2932	-0.218	0.83	+???--	9.54E-01	3276	1.268	0.20	-???++	6.21E-01
100001300	alpha-hydroxyisovalerate	13556	-0.72	0.47	--+---	38.4	0.15	0.83	6404	0.247	0.81	-++---	9.54E-01	7151	-0.906	0.36	--+---	7.51E-01
100009015	1-(1-enyl-stearoyl)-2-docosahexaenoyl-GPC (P-18:0/22:6)*	9802	-0.711	0.48	???+-?	60.1	0.11	0.83	4642	0.239	0.81	???+-?	9.54E-01	5160	-0.858	0.39	???+-?	7.73E-01
100001767	pyrraline	10847	-0.724	0.47	-??-+-	0	0.53	0.83	5139	-0.243	0.81	+??-+-	9.54E-01	5708	-0.827	0.41	-??-+-	7.84E-01
100006293	sphingomyelin (d18:1/20:2, d18:2/20:1, d16:1/22:2)*	6208	0.718	0.47	-???++	0	0.85	0.83	2932	0.102	0.92	-???++	9.88E-01	3276	0.759	0.45	-???++	7.97E-01
100003240	N-stearoyltaurine	1045	0.731	0.46	+????-	81.5	0.02	0.83	497	0.087	0.93	+????-	9.92E-01	548	0.774	0.44	+????-	7.97E-01
100002927	S-methylcysteine sulfoxide	6208	0.731	0.47	+???+-	40.5	0.19	0.83	2932	-0.03	0.98	-???+-	9.92E-01	3276	0.662	0.51	+???+-	8.32E-01
100002769	argininate*	6208	-0.721	0.47	-???+-	54.8	0.11	0.83	2932	-0.039	0.97	+???+-	9.92E-01	3276	-0.484	0.63	-???-+	8.80E-01
1648	taurocholate	10847	-0.703	0.48	-??-+-	0	0.58	0.83	5139	-0.67	0.50	-??-++	9.44E-01	5708	-0.506	0.61	-??---	8.74E-01
100000961	homoarginine	10847	0.705	0.48	-??++-	0	0.80	0.83	5139	0.539	0.59	-??++-	9.44E-01	5708	0.241	0.81	-??-++	9.52E-01
100001876	sphinganine-1-phosphate	6208	-0.696	0.49	-???--	0	0.41	0.83	2932	1.657	0.10	-???++	7.92E-01	3276	-1.923	0.05	-???--	4.16E-01
1628	glycochenodeoxycholate	13556	0.696	0.49	-++-++	49.8	0.08	0.83	6404	0.842	0.40	-+++--	9.04E-01	7151	0.349	0.73	-++-++	9.21E-01
100004326	3-acetylphenol sulfate	6208	-0.692	0.49	+???--	12.3	0.32	0.84	2932	-1.309	0.19	+???--	8.30E-01	3276	0.049	0.96	-???+-	9.88E-01
100006082	4-hydroxychlorothalonil	10847	-0.69	0.49	-??+--	57.2	0.07	0.84	5139	-0.431	0.67	-??-+-	9.48E-01	5708	-0.371	0.71	+??+--	9.15E-01
477	pristanate	6208	-0.69	0.49	+???--	69.2	0.04	0.84	2932	-0.336	0.74	+???+-	9.53E-01	3276	-0.422	0.67	+???--	9.02E-01
100001768	N6-carboxymethyllysine	6208	-0.692	0.49	+???--	36.4	0.21	0.84	2932	0.079	0.94	+???+-	9.92E-01	3276	-0.862	0.39	-???--	7.70E-01
100001293	N-acetylhistidine	10847	0.689	0.49	-??++-	0	0.86	0.84	5139	0.707	0.48	-??++-	9.38E-01	5708	0.563	0.57	+??-++	8.61E-01
100003926	3-hydroxybutyrylcarnitine (1)	11811	0.686	0.49	++?-++	8.4	0.36	0.84	5563	2.188	0.03	++?+++	6.08E-01	6248	-0.709	0.48	++?---	8.10E-01
100000008	benzoate	12146	0.683	0.49	??-++-	49.7	0.11	0.84	5767	-0.288	0.77	??-+--	9.54E-01	6378	1.013	0.31	??-++-	6.98E-01
279	cystine	12592	0.681	0.50	+?-+-+	61	0.04	0.84	5980	0.914	0.36	+?-+++	9.04E-01	6611	0.708	0.48	+?-+-+	8.10E-01
100001756	4-ethylphenylsulfate	12592	-0.673	0.50	-?-++-	55.6	0.06	0.84	5980	-1.031	0.30	+?----	9.04E-01	6611	-0.246	0.81	-?+++-	9.50E-01
100001757	thymol sulfate	10847	0.676	0.50	-??++-	17	0.31	0.84	5139	0.7	0.48	-??++-	9.40E-01	5708	0.377	0.71	-??++-	9.15E-01
100002107	palmitoyl sphingomyelin (d18:1/16:0)	12592	-0.674	0.50	-?++--	40.8	0.15	0.84	5980	0.442	0.66	+?++--	9.48E-01	6611	-0.816	0.41	-?-+--	7.85E-01
1114	3-aminoisobutyrate	10847	0.674	0.50	-??++-	0	0.68	0.84	5139	0.37	0.71	-??++-	9.52E-01	5708	0.324	0.75	-??-+-	9.24E-01
100001396	7-methylxanthine	13556	-0.666	0.51	-+++--	0	0.69	0.84	6404	-1.194	0.23	-+++--	8.59E-01	7151	0.153	0.88	-+--++	9.72E-01
100004542	2-aminoheptanoate	11811	-0.665	0.51	--?-++	38.6	0.16	0.84	5563	-0.847	0.40	--?+-+	9.04E-01	6248	0.009	0.99	-+?-++	9.98E-01
100006360	dopamine 4-sulfate	1045	0.667	0.50	+????-	76.2	0.04	0.84	497	0.348	0.73	+????-	9.52E-01	548	0.639	0.52	+????-	8.35E-01
100001553	1-dihomo-linoleoylglycerophosphocholine (20:2n6)*	10766	-0.666	0.51	?-?-+?	0	0.65	0.84	5066	-0.113	0.91	?+?-+?	9.85E-01	5700	-0.295	0.77	?-?-+?	9.37E-01
100000936	3-methyl-2-oxobutyrate	13556	-0.662	0.51	--+--+	55.5	0.05	0.84	6404	-0.396	0.69	--+-++	9.48E-01	7151	-0.395	0.69	+-+--+	9.07E-01

100010919	oleoyl-oleoyl-glycerol (18:1/18:1) [2]*	6208	0.661	0.51	+???++	0	0.96	0.84	2932	-0.416	0.68	-???--	9.48E-01	3276	1.703	0.09	+???++	4.87E-01
100005403	etiocholanolone glucuronide	10847	-0.641	0.52	-??---	0	0.83	0.84	5139	-1.482	0.14	+??--+	7.92E-01	5708	-0.483	0.63	-??+--	8.80E-01
100008930	oleate/vaccenate (18:1)	12592	-0.657	0.51	+?-+--	0	0.92	0.84	5980	1.282	0.20	+?-+++	8.30E-01	6611	-1.486	0.14	-?----	5.54E-01
880	adenine	10847	0.64	0.52	-??+++	0	0.44	0.84	5139	-1.133	0.26	-??+-+	8.69E-01	5708	1.778	0.08	-??+++	4.51E-01
1002	allantoin	10847	0.647	0.52	+??+++	0	0.96	0.84	5139	1.139	0.25	+??-+-	8.69E-01	5708	0.179	0.86	-??+-+	9.68E-01
100008903	1,2-dilinoleoyl-GPC (18:2/18:2)	10248	-0.651	0.52	-??-+?	0	0.72	0.84	4855	1.009	0.31	-??++?	9.04E-01	5393	-1.264	0.21	+??--?	6.21E-01
100015640	N-palmitoylserine	5762	-0.65	0.52	????--	0	0.76	0.84	2719	-1.004	0.32	????--	9.04E-01	3043	-0.177	0.86	????-+	9.68E-01
100002167	12-HETE	12592	-0.632	0.53	-?-+--	0	0.98	0.84	5980	0.696	0.49	-?-+++	9.40E-01	6611	-1.404	0.16	-?----	5.64E-01
100009052	palmitoyl-linoleoyl-glycerol (16:0/18:2) [1]*	10847	0.644	0.52	+??+-+	0	0.80	0.84	5139	-0.555	0.58	-??--+	9.44E-01	5708	1.477	0.14	+??+++	5.54E-01
100001527	hexanoylglycine	5684	-0.631	0.53	-??+?-	0	0.38	0.84	2704	0.524	0.60	-??+?-	9.44E-01	2980	-1.237	0.22	-??-?-	6.29E-01
100009037	1-margaroyl-2-linoleoyl-GPC (17:0/18:2)*	9802	-0.636	0.52	???+-?	0	0.49	0.84	4642	0.543	0.59	???+-?	9.44E-01	5160	-1.123	0.26	???--?	6.61E-01
100003397	trimethylamine N-oxide	10847	-0.637	0.52	-??+--	0	0.94	0.84	5139	-0.547	0.58	+??+--	9.44E-01	5708	-0.527	0.60	-??-+-	8.67E-01
100001778	1-linoleoyl-GPI (18:2)*	10847	0.627	0.53	+??++-	0	0.40	0.84	5139	0.56	0.58	+??++-	9.44E-01	5708	0.517	0.61	+??+--	8.70E-01
313	sphinganine	11811	0.632	0.53	-+?+++	0	0.88	0.84	5563	0.595	0.55	-+?+-+	9.44E-01	6248	0.454	0.65	-+?++-	8.97E-01
925	heptanoate (7:0)	11993	-0.656	0.51	+?-+-?	0	0.75	0.84	5696	0.422	0.67	-?-+-?	9.48E-01	6296	-0.99	0.32	+?---?	7.12E-01
100006292	sphingomyelin (d18:1/20:1, d18:2/20:0)*	10847	0.641	0.52	-??+--	0	0.42	0.84	5139	0.431	0.67	+??++-	9.48E-01	5708	0.643	0.52	-??+-+	8.35E-01
100001276	N-acetylisoleucine	5238	-0.627	0.53	???-?+	0	0.54	0.84	2491	0.362	0.72	???+?+	9.52E-01	2747	-1.173	0.24	???-?+	6.40E-01
1087	erucate (22:1n9)	11811	-0.641	0.52	++?---	14.3	0.32	0.84	5563	0.234	0.81	++?+-+	9.54E-01	6248	-0.943	0.35	++?---	7.37E-01
100001856	1-stearoyl-2-oleoyl-GPE (18:0/18:1)	10847	0.65	0.52	+??-++	0	0.60	0.84	5139	0.005	1.00	-??+-+	9.96E-01	5708	1.226	0.22	+??-++	6.31E-01
100008994	1-stearoyl-2-linoleoyl-GPI (18:0/18:2)	10847	0.624	0.53	-??++-	0	0.50	0.84	5139	0.245	0.81	+??-+-	9.54E-01	5708	0.784	0.43	-??++-	7.93E-01
100002873	1-lignoceroyl-GPC (24:0)	5684	-0.618	0.54	+??-?-	0	0.93	0.85	2704	-0.709	0.48	-??-?+	9.37E-01	2980	-0.285	0.78	+??-?-	9.39E-01
100009027	sphingomyelin (d18:0/18:0, d19:0/17:0)*	6208	0.617	0.54	+???++	0	0.49	0.85	2932	-0.586	0.56	+???--	9.44E-01	3276	1.683	0.09	+???++	4.89E-01
100001989	glycocholenate sulfate*	11811	-0.617	0.54	-+?--+	0	0.44	0.85	5563	-0.358	0.72	++?--+	9.52E-01	6248	-0.574	0.57	-+?--+	8.55E-01
100001150	propionylglycine	10847	0.61	0.54	-??++-	44	0.15	0.85	5139	0.871	0.38	+??++-	9.04E-01	5708	0.238	0.81	-??+++	9.54E-01
100002009	5alpha-pregnan-3beta,20beta-diol monosulfate (1)	10847	-0.612	0.54	-??-+-	5.3	0.37	0.85	5139	-0.452	0.65	+??---	9.48E-01	5708	-1.262	0.21	-??---	6.22E-01
100001502	gamma-glutamyl-2-aminobutyrate	10401	-0.61	0.54	???+--	50.9	0.13	0.85	4926	0.441	0.66	???+--	9.48E-01	5475	-0.94	0.35	???---	7.39E-01
100002029	androstenediol (3beta,17beta) monosulfate (2)	10847	-0.614	0.54	-??+--	0	0.83	0.85	5139	-0.246	0.81	-??+-+	9.54E-01	5708	-0.871	0.38	-??---	7.70E-01
1026	phosphoethanolamine	10401	0.605	0.55	???++-	73.1	0.02	0.85	4926	-0.835	0.40	???---	9.04E-01	5475	1.106	0.27	???++-	6.61E-01
100001399	1,7-dimethylurate	11811	-0.594	0.55	++?+--	57.2	0.05	0.86	5563	-1.637	0.10	+-?+--	7.92E-01	6248	0.399	0.69	-+?+--	9.07E-01
415	methionine	13556	-0.595	0.55	--++--	19.3	0.29	0.86	6404	-0.836	0.40	-+++--	9.04E-01	7151	-0.083	0.93	--++-+	9.78E-01
100003594	phenylalanyltryptophan	5603	0.597	0.55	?-?+??	68	0.08	0.86	2631	-0.795	0.43	?-?-??	9.11E-01	2972	1.269	0.20	?-?+??	6.21E-01
100001624	3-(3-hydroxyphenyl)propionate	10847	-0.59	0.56	+??-+-	24.4	0.27	0.86	5139	-1.711	0.09	+??---	7.84E-01	5708	0.453	0.65	-??-+-	8.97E-01
302	deoxycholate	12592	0.591	0.55	+?+--+	0	0.71	0.86	5980	0.67	0.50	+?+-++	9.44E-01	6611	0.052	0.96	-?++-+	9.88E-01
100001279	hyocholate	5684	-0.59	0.56	+??-?-	0	0.50	0.86	2704	0.227	0.82	+??+?-	9.54E-01	2980	-0.828	0.41	-??-?+	7.84E-01
182	quinolinate	10847	-0.588	0.56	-??+--	0	0.41	0.86	5139	0.785	0.43	-??+--	9.11E-01	5708	-1.197	0.23	-??--+	6.40E-01
100000299	xanthosine	1045	0.587	0.56	+????-	32.6	0.22	0.86	497	0.311	0.76	+????-	9.54E-01	548	0.661	0.51	+????+	8.32E-01
100004112	3-methyl catechol sulfate (1)	11811	-0.584	0.56	++?---	46.6	0.11	0.86	5563	-0.773	0.44	++?---	9.13E-01	6248	-0.171	0.86	-+?-+-	9.68E-01
100015789	sphingomyelin (d18:2/24:2)*	6208	0.585	0.56	-???++	0	0.52	0.86	2932	0.587	0.56	+???++	9.44E-01	3276	0.105	0.92	-???++	9.78E-01
100010896	2'-O-methyluridine	1045	-0.581	0.56	+????-	76.8	0.04	0.86	497	-0.349	0.73	+????-	9.52E-01	548	-0.262	0.79	+????-	9.43E-01
2050	eicosapentaenoate (EPA; 20:5n3)	13556	-0.58	0.56	+--+--	0	0.42	0.86	6404	0.178	0.86	++-+--	9.70E-01	7151	-0.679	0.50	---+--	8.23E-01
100015968	carotene diol (3)	6208	-0.577	0.56	+???+-	55.8	0.10	0.86	2932	0.908	0.36	-???+-	9.04E-01	3276	-1.498	0.13	+???--	5.51E-01
503	serine	12592	-0.576	0.56	-?++--	11.3	0.34	0.86	5980	-0.058	0.95	+?-+--	9.92E-01	6611	-0.737	0.46	-?++--	7.98E-01
100008953	2-palmitoleoylglycerol (16:1)*	5085	0.577	0.56	+??+??	0	0.77	0.86	2420	0.051	0.96	+??-??	9.92E-01	2665	0.658	0.51	+??+??	8.32E-01
100001334	N-acetylproline	5238	0.569	0.57	???+?+	0	0.54	0.86	2491	-0.889	0.37	???-?+	9.04E-01	2747	1.343	0.18	???+?+	5.89E-01
100002417	2,3-dihydroxyisovalerate	10847	0.562	0.57	+??++-	0	0.83	0.86	5139	0.696	0.49	+??++-	9.40E-01	5708	0.175	0.86	-??++-	9.68E-01
100001579	2-hydroxypalmitate	13556	-0.563	0.57	+--+--	0	0.54	0.86	6404	0.644	0.52	++-+-+	9.44E-01	7151	-1.062	0.29	+-++--	6.80E-01
100001557	2-linoleoylglycerophosphocholine*	10766	-0.563	0.57	?+?--?	0	0.82	0.86	5066	0.63	0.53	?-?++?	9.44E-01	5700	-0.675	0.50	?+?--?	8.23E-01
62	12,13-DiHOME	10401	-0.568	0.57	???-++	54	0.11	0.86	4926	-0.374	0.71	???-++	9.52E-01	5475	-0.516	0.61	???--+	8.70E-01
100001481	1-docosahexaenoylglycerol (22:6)	10847	0.562	0.57	+??+-+	0	0.48	0.86	5139	-0.297	0.77	+??+-+	9.54E-01	5708	1.037	0.30	-??++-	6.91E-01
100001621	glycerophosphoinositol*	1045	0.571	0.57	+????-	80.8	0.02	0.86	497	-0.268	0.79	+????-	9.54E-01	548	1.023	0.31	+????+	6.96E-01

100000584	2-arachidonoylglycerol (20:4)	1045	-0.566	0.57	-????+	0	0.48	0.86	497	-0.167	0.87	-????-	9.73E-01	548	-0.731	0.46	-????+	7.98E-01
100001337	linolenate [alpha or gamma; (18:3n3 or 6)]	13556	-0.554	0.58	+----+	0	0.88	0.86	6404	1.249	0.21	++-+++	8.48E-01	7151	-1.383	0.17	------	5.66E-01
100002152	andro steroid monosulfate C19H28O6S (1)*	10847	-0.556	0.58	+??--+	0.5	0.39	0.86	5139	-1.047	0.30	+??+-+	8.93E-01	5708	-0.102	0.92	+??-+-	9.78E-01
100001417	phenylacetylglutamine	13556	-0.559	0.58	+++---	40	0.14	0.86	6404	-0.687	0.49	+-++--	9.40E-01	7151	-0.349	0.73	+++---	9.21E-01
100015839	dihomo-linoleoylcarnitine (C20:2)*	6208	-0.556	0.58	+???--	0	0.81	0.86	2932	0.27	0.79	-???+-	9.54E-01	3276	-0.563	0.57	+???--	8.61E-01
1111	vanillylmandelate (VMA)	10847	-0.552	0.58	+??--+	0	0.96	0.86	5139	1.098	0.27	+??+++	8.74E-01	5708	-1.648	0.10	+??--+	5.02E-01
100001461	1-stearoyl-GPE (18:0)	13556	0.546	0.59	+++++-	0	0.97	0.87	6404	0.844	0.40	-+-++-	9.04E-01	7151	0.763	0.45	+-+++-	7.97E-01
2029	azelate (nonanedioate)	11811	-0.545	0.59	-+?-+-	0	0.63	0.87	5563	-0.079	0.94	-+?-+-	9.92E-01	6248	-0.538	0.59	++?---	8.63E-01
100001586	gulonate*	10847	0.542	0.59	+??++-	20.8	0.29	0.87	5139	-0.195	0.85	+??---	9.60E-01	5708	0.822	0.41	+??++-	7.84E-01
100008928	2-hydroxybutyrate/2-hydroxyisobutyrate	12592	0.538	0.59	+?++--	5.2	0.38	0.87	5980	1.388	0.17	+?--++	8.12E-01	6611	-0.052	0.96	+?++--	9.88E-01
252	succinate	10847	-0.533	0.59	-??---	0	0.71	0.87	5139	1.366	0.17	-??++-	8.21E-01	5708	-1.515	0.13	-??---	5.48E-01
100006108	phenylacetylcarnitine	10847	-0.532	0.59	+??---	0	0.94	0.87	5139	0.534	0.59	+??+--	9.44E-01	5708	-1.109	0.27	-??---	6.61E-01
100002018	5alpha-androstan-3alpha,17beta-diol monosulfate (1)	10847	-0.531	0.60	-??-+-	48.9	0.12	0.87	5139	-0.533	0.59	-??-+-	9.44E-01	5708	-0.532	0.60	-??-+-	8.65E-01
100001733	hexanoylglutamine	6208	-0.532	0.60	+???--	0.1	0.37	0.87	2932	-0.639	0.52	-???-+	9.44E-01	3276	-0.131	0.90	+???--	9.77E-01
100009227	1-linoleoyl-GPG (18:2)*	6208	-0.531	0.60	-???-+	9.9	0.33	0.87	2932	0.298	0.77	+???-+	9.54E-01	3276	-0.674	0.50	-???-+	8.23E-01
100006171	eugenol sulfate	10847	-0.528	0.60	-??-++	0	0.75	0.87	5139	-0.131	0.90	+??-++	9.77E-01	5708	-0.639	0.52	-??---	8.35E-01
100000436	glycodeoxycholate	11811	0.524	0.60	-+?--+	43.2	0.13	0.87	5563	-0.104	0.92	-+?-++	9.88E-01	6248	0.736	0.46	++?+-+	7.98E-01
100008999	1-(1-enyl-stearoyl)-2-arachidonoyl-GPE (P-18:0/20:4)*	10847	-0.525	0.60	-??+--	7.7	0.35	0.87	5139	-0.029	0.98	-??-+-	9.92E-01	5708	-0.094	0.92	-??+-+	9.78E-01
1104	methyl indole-3-acetate	10847	-0.519	0.60	+??---	0	0.52	0.87	5139	0.421	0.67	+??+--	9.48E-01	5708	-0.685	0.49	-??-+-	8.22E-01
100001577	N-acetylcitrulline	10847	0.517	0.61	+??-++	0	0.74	0.87	5139	-0.506	0.61	-??+--	9.47E-01	5708	1.226	0.22	+??-++	6.31E-01
1099	guanosine	12592	0.514	0.61	+?+--+	0	0.51	0.87	5980	0.068	0.95	+?+--+	9.92E-01	6611	0.262	0.79	+?-+-+	9.43E-01
100009028	N-palmitoyl-sphinganine (d18:0/16:0)	10847	-0.504	0.61	-??-+-	0	0.87	0.88	5139	-1.448	0.15	-??---	8.00E-01	5708	0.768	0.44	+??+++	7.97E-01
100001987	5alpha-androstan-3beta,17beta-diol disulfate	13556	-0.509	0.61	-++-+-	38.3	0.15	0.88	6404	-0.935	0.35	-++--+	9.04E-01	7151	-0.84	0.40	-+--+-	7.82E-01
100000039	methionine sulfoxide	10847	0.504	0.61	+??-+-	8.9	0.35	0.88	5139	0.853	0.39	+??+-+	9.04E-01	5708	-0.121	0.90	+??-+-	9.77E-01
100006370	3beta-hydroxy-5-cholestenoate	6208	0.506	0.61	-???++	50.3	0.13	0.88	2932	0.607	0.54	-???++	9.44E-01	3276	0.469	0.64	-???++	8.85E-01
100002024	5alpha-androstan-3beta,17beta-diol monosulfate (2)	10847	-0.503	0.61	-??-+-	61.4	0.05	0.88	5139	-0.299	0.77	-??-+-	9.54E-01	5708	-0.667	0.50	-??-+-	8.29E-01
100009005	1-(1-enyl-palmitoyl)-2-oleoyl-GPE (P-16:0/18:1)*	10847	-0.504	0.61	-??--+	0	0.89	0.88	5139	0.27	0.79	+??+-+	9.54E-01	5708	-0.345	0.73	-??--+	9.22E-01
100002500	formiminoglutamate	5762	-0.5	0.62	????-+	32.3	0.22	0.88	2719	-2.135	0.03	????--	6.08E-01	3043	0.908	0.36	????++	7.51E-01
100001327	HWESASXX*	3155	-0.499	0.62	+-+???	62.8	0.07	0.88	1478	0.785	0.43	--+???	9.11E-01	1676	-1.14	0.25	+-+???	6.56E-01
1518	N-palmitoyl-sphingosine (d18:1/16:0)	10847	-0.497	0.62	+??+--	0	0.41	0.88	5139	-0.397	0.69	+??+--	9.48E-01	5708	0.275	0.78	+??--+	9.41E-01
100000656	1-stearoyl-GPI (18:0)	12592	0.497	0.62	+?++--	44.2	0.13	0.88	5980	0.165	0.87	+?-++-	9.73E-01	6611	0.87	0.38	+?+++-	7.70E-01
100005352	1-eicosenoylglycerophosphocholine (20:1n9)*	10766	-0.493	0.62	?+?--?	0	0.96	0.88	5066	-0.035	0.97	?-?-+?	9.92E-01	5700	-0.311	0.76	?+?--?	9.27E-01
100015787	sphingomyelin (d18:1/19:0, d19:1/18:0)*	6208	-0.486	0.63	+???--	47.1	0.15	0.88	2932	-1.618	0.11	+???--	7.92E-01	3276	1.046	0.30	+???++	6.87E-01
100001267	piperine	13556	-0.475	0.63	----++	50.8	0.07	0.89	6404	-1.408	0.16	+----+	8.12E-01	7151	0.221	0.83	----+-	9.59E-01
361	inosine	12592	-0.478	0.63	+?+--+	41.3	0.15	0.89	5980	-0.852	0.39	+?+--+	9.04E-01	6611	-0.204	0.84	+?+--+	9.66E-01
100001593	glutarylcarnitine (C5-DC)	13556	-0.476	0.63	--++--	57.7	0.04	0.89	6404	0.528	0.60	+-+++-	9.44E-01	7151	-0.611	0.54	--++-+	8.44E-01
100000263	imidazole lactate	10847	-0.475	0.63	-??+--	0	0.49	0.89	5139	0.353	0.72	+??+--	9.52E-01	5708	-0.783	0.43	-??---	7.93E-01
100001468	N1-Methyl-2-pyridone-5-carboxamide	13556	0.469	0.64	+--++-	56.5	0.04	0.89	6404	0.631	0.53	++-++-	9.44E-01	7151	0.178	0.86	+--++-	9.68E-01
100001551	1-arachidonoyl-GPC (20:4n6)*	13556	0.466	0.64	-+-++-	17.3	0.30	0.89	6404	0.159	0.87	-+--+-	9.75E-01	7151	0.994	0.32	-+-++-	7.12E-01
100006296	sphingomyelin (d18:1/22:2, d18:2/22:1, d16:1/24:2)*	1045	-0.463	0.64	-????-	0	0.91	0.89	497	-0.533	0.59	+????-	9.44E-01	548	-0.163	0.87	-????+	9.72E-01
171	hypoxanthine	13556	-0.464	0.64	-----+	0	0.77	0.89	6404	0.224	0.82	+-+--+	9.54E-01	7151	-0.659	0.51	-----+	8.32E-01
100009337	caffeic acid sulfate	9802	-0.462	0.64	???-+?	79.4	0.03	0.89	4642	-0.742	0.46	???--?	9.30E-01	5160	-0.17	0.87	???-+?	9.68E-01
100001384	1-arachidoyl-GPC (20:0)	9802	-0.458	0.65	???+-?	0	0.35	0.89	4642	0.933	0.35	???++?	9.04E-01	5160	-0.621	0.53	???+-?	8.43E-01
100009026	behenoyl dihydrosphingomyelin (d18:0/22:0)*	6208	-0.452	0.65	-???--	0	0.96	0.89	2932	-1.339	0.18	+???--	8.30E-01	3276	0.969	0.33	-???++	7.20E-01
100001278	10-heptadecenoate (17:1n7)	13556	-0.452	0.65	+--+--	0	0.86	0.89	6404	1.202	0.23	++-+++	8.59E-01	7151	-1.104	0.27	------	6.61E-01
267	choline phosphate	10847	-0.453	0.65	-??+--	29.5	0.24	0.89	5139	-0.578	0.56	-??+--	9.44E-01	5708	-0.001	1.00	-??++-	9.99E-01
100002173	1-pentadecanoylglycerophosphocholine (15:0)*	10766	-0.453	0.65	?-?+-?	51.6	0.13	0.89	5066	0.276	0.78	?-?+-?	9.54E-01	5700	-0.534	0.59	?-?-+?	8.65E-01
100009162	1-(1-enyl-palmitoyl)-2-palmitoyl-GPC (P-16:0/16:0)*	10847	0.446	0.66	+??+--	55.1	0.08	0.90	5139	1.135	0.26	-??++-	8.69E-01	5708	-0.383	0.70	+??+--	9.13E-01
1492	linoleamide (18:2n6)	5238	-0.443	0.66	???-?+	7.8	0.30	0.90	2491	-0.59	0.56	???-?+	9.44E-01	2747	-0.254	0.80	???-?-	9.47E-01
1001	trans-4-hydroxyproline	13556	-0.438	0.66	-+--++	0	0.63	0.90	6404	-1.59	0.11	+-----	7.92E-01	7151	0.692	0.49	-+--++	8.21E-01

100004089	2-hydroxydecanoate	11811	-0.428	0.67	-+?-++	19.7	0.29	0.90	5563	-1.385	0.17	-+?---	8.12E-01	6248	0.519	0.60	-+?-++	8.70E-01
272	corticosterone	1045	0.431	0.67	+????+	0	0.77	0.90	497	1.138	0.26	-????+	8.69E-01	548	-0.645	0.52	+????-	8.35E-01
100006290	sphingomyelin (d18:1/20:0, d16:1/22:0)*	10847	0.431	0.67	-??+--	20.7	0.29	0.90	5139	-0.839	0.40	-??+--	9.04E-01	5708	1.61	0.11	+??++-	5.22E-01
100009002	1-(1-enyl-palmitoyl)-2-arachidonoyl-GPE (P-16:0/20:4)*	10847	-0.422	0.67	-??--+	0	0.50	0.90	5139	-0.924	0.36	-??-+-	9.04E-01	5708	0.606	0.54	-??+-+	8.46E-01
100009000	1-(1-enyl-palmitoyl)-2-docosahexaenoyl-GPE (P-16:0/22:6)*	9802	0.429	0.67	???+-?	48.7	0.16	0.90	4642	-0.751	0.45	???--?	9.27E-01	5160	1.192	0.23	???+-?	6.40E-01
100009161	1-(1-enyl-palmitoyl)-2-myristoyl-GPC (P-16:0/14:0)*	9802	-0.432	0.67	???+-?	0	0.55	0.90	4642	0.747	0.46	???+-?	9.28E-01	5160	-1.391	0.16	???--?	5.64E-01
100003892	lanthionine	10847	0.436	0.66	-??-++	0	0.53	0.90	5139	0.592	0.55	+??-++	9.44E-01	5708	0.007	0.99	-??-+-	9.98E-01
1134	urate	13556	-0.425	0.67	--+--+	0	0.95	0.90	6404	0.415	0.68	++++-+	9.48E-01	7151	-0.187	0.85	--++-+	9.68E-01
100010936	oleoyl-arachidonoyl-glycerol (18:1/20:4) [1]*	6208	0.422	0.67	-???++	0	0.51	0.90	2932	0.329	0.74	-???++	9.54E-01	3276	0.979	0.33	+???++	7.16E-01
100001323	DSGEGDFXAEGGGVR*	13556	-0.432	0.67	---+-+	6.3	0.38	0.90	6404	0.327	0.74	---+++	9.54E-01	7151	-0.764	0.45	-----+	7.97E-01
197	S-adenosylhomocysteine (SAH)	5085	-0.425	0.67	+??-??	0	0.32	0.90	2420	-0.113	0.91	+??-??	9.85E-01	2665	-0.329	0.74	+??-??	9.24E-01
100001618	1-myristoylglycerol (14:0)	10847	0.435	0.66	+??-++	0	0.49	0.90	5139	-0.039	0.97	+??+--	9.92E-01	5708	0.95	0.34	+??-++	7.33E-01
1021	5-oxoproline	13556	-0.422	0.67	++++--	12.3	0.34	0.90	6404	-0.026	0.98	-+++--	9.92E-01	7151	-0.284	0.78	+++---	9.39E-01
100009038	myristoyl dihydrosphingomyelin (d18:0/14:0)*	6208	-0.416	0.68	+???--	52.9	0.12	0.90	2932	-1.25	0.21	+???--	8.48E-01	3276	0.865	0.39	+???++	7.70E-01
100001552	1-eicosatrienoylglycerophosphocholine (20:3)*	10766	-0.416	0.68	?-?++?	54.1	0.11	0.90	5066	-0.563	0.57	?-?--?	9.44E-01	5700	0.604	0.55	?-?++?	8.46E-01
100006375	3-methoxycatechol sulfate (1)	10847	0.414	0.68	+??++-	0	0.65	0.90	5139	0.68	0.50	+??++-	9.44E-01	5708	0.268	0.79	-??-+-	9.43E-01
100015833	arachidoylcarnitine (C20)*	6208	-0.417	0.68	+???+-	65.6	0.05	0.90	2932	0.341	0.73	+???+-	9.52E-01	3276	-0.338	0.74	+???+-	9.23E-01
100004295	2-piperidinone	11365	0.409	0.68	?+?+-+	12.2	0.33	0.90	5350	0.391	0.70	?+?--+	9.49E-01	6015	0.354	0.72	?-?+-+	9.21E-01
100000665	docosahexaenoate (DHA; 22:6n3)	13556	-0.398	0.69	+--+--	50.6	0.07	0.90	6404	0.467	0.64	++-+-+	9.47E-01	7151	-0.905	0.37	+--+--	7.51E-01
100009051	1-stearoyl-2-dihomo-linolenoyl-GPC (18:0/20:3n3 or 6)*	9802	0.405	0.69	???++?	0	0.82	0.90	4642	-0.237	0.81	???+-?	9.54E-01	5160	1.457	0.15	???++?	5.54E-01
100009232	thioproline	10847	-0.398	0.69	-??--+	60.5	0.06	0.90	5139	0.253	0.80	-??+-+	9.54E-01	5708	-0.339	0.73	-??--+	9.23E-01
100001655	1-palmitoyl-GPI (16:0)	13556	0.399	0.69	+--++-	34.8	0.18	0.90	6404	-0.136	0.89	++--+-	9.77E-01	7151	0.867	0.39	+-+++-	7.70E-01
100004575	N2,N5-diacetylornithine	5684	-0.395	0.69	-??+?-	74.7	0.02	0.90	2704	0.616	0.54	-??+?-	9.44E-01	2980	-0.826	0.41	-??+?-	7.84E-01
100002196	13-HODE + 9-HODE	11811	0.39	0.70	-+?-++	0	0.64	0.91	5563	0.634	0.53	++?-++	9.44E-01	6248	0.021	0.98	--?-++	9.95E-01
136	cholate	13556	-0.392	0.70	+++-+-	66.5	0.01	0.91	6404	-0.423	0.67	++-+--	9.48E-01	7151	-0.149	0.88	+++-+-	9.74E-01
564	threonine	13556	0.39	0.70	-+++--	57	0.04	0.91	6404	-0.119	0.91	-+++--	9.83E-01	7151	0.12	0.90	-+-+-+	9.77E-01
180	linoleate (18:2n6)	13556	-0.378	0.71	---+--	0	0.99	0.91	6404	1.584	0.11	++-+++	7.92E-01	7151	-1.36	0.17	------	5.83E-01
100015735	ceramide (d18:1/14:0, d16:1/16:0)*	6208	-0.381	0.70	+???--	62.5	0.07	0.91	2932	-1.34	0.18	+???--	8.30E-01	3276	0.842	0.40	+???-+	7.81E-01
1141	4-hydroxyphenylpyruvate	10847	-0.384	0.70	+??+--	0	0.72	0.91	5139	0.818	0.41	+??+++	9.10E-01	5708	-1.237	0.22	-??+--	6.29E-01
1083	N-acetylmethionine	10847	0.38	0.70	+??-+-	0	0.49	0.91	5139	0.731	0.46	+??++-	9.33E-01	5708	-0.579	0.56	-??-+-	8.55E-01
825	uracil	10847	-0.38	0.70	+??--+	0	0.71	0.91	5139	0.453	0.65	+??-++	9.48E-01	5708	-0.829	0.41	+??+--	7.84E-01
100001580	docosapentaenoate (n6 DPA; 22:5n6)	13110	0.38	0.70	?---+-	0	0.72	0.91	6191	-0.409	0.68	?+--++	9.48E-01	6918	0.693	0.49	?--++-	8.21E-01
100006190	2-acetamidophenol sulfate	10847	0.377	0.71	+??++-	0	0.68	0.91	5139	0.215	0.83	+??++-	9.54E-01	5708	0.348	0.73	+??++-	9.21E-01
100010916	palmitoyl-oleoyl-glycerol (16:0/18:1) [1]*	6208	0.37	0.71	+???+-	0	0.49	0.91	2932	-2.297	0.02	+???--	5.76E-01	3276	2.779	5.45E-03	+???++	2.17E-01
100000787	N-acetylaspartate (NAA)	10847	0.359	0.72	-??-+-	0	0.59	0.91	5139	1.261	0.21	-??++-	8.42E-01	5708	-0.486	0.63	-??-+-	8.80E-01
100015744	ceramide (d18:2/24:1, d18:1/24:2)*	6208	-0.369	0.71	-???-+	0	0.91	0.91	2932	1.178	0.24	+???++	8.59E-01	3276	-1.095	0.27	-???-+	6.62E-01
100001148	5-hydroxyhexanoate	10847	0.363	0.72	+??-+-	0	0.54	0.91	5139	-0.858	0.39	-??--+	9.04E-01	5708	0.79	0.43	+??++-	7.93E-01
849	caffeine	13556	-0.361	0.72	++++--	31.2	0.20	0.91	6404	-0.714	0.48	-+++-+	9.37E-01	7151	-0.188	0.85	+++-+-	9.68E-01
181	laurate (12:0)	13556	-0.365	0.71	+-+---	0	0.56	0.91	6404	0.537	0.59	++++--	9.44E-01	7151	-0.548	0.58	+-+---	8.62E-01
100005389	ferulic acid 4-sulfate	9802	-0.372	0.71	???-+?	70.3	0.07	0.91	4642	-0.537	0.59	???--?	9.44E-01	5160	-0.179	0.86	???-+?	9.68E-01
100001274	N-acetylthreonine	13556	0.364	0.72	++++--	0	0.95	0.91	6404	0.655	0.51	++++--	9.44E-01	7151	0.143	0.89	--+-++	9.77E-01
100006295	sphingomyelin (d18:1/22:1, d18:2/22:0, d16:1/24:1)*	10847	-0.361	0.72	-??+--	72.9	0.01	0.91	5139	-0.015	0.99	-??++-	9.94E-01	5708	-0.077	0.94	-??+--	9.79E-01
100004442	1-arachidonoyl-GPA (20:4)	10847	-0.357	0.72	-??-+-	59.3	0.06	0.91	5139	0.483	0.63	-??-++	9.47E-01	5708	-0.59	0.56	-??+--	8.52E-01
234	aspartate	12592	0.35	0.73	+?+---	0	0.79	0.92	5980	-0.116	0.91	+?++-+	9.83E-01	6611	0.49	0.62	-?+-+-	8.80E-01
100009007	1-(1-enyl-palmitoyl)-2-oleoyl-GPC (P-16:0/18:1)*	10847	-0.33	0.74	+??-+-	43.6	0.15	0.92	5139	1.394	0.16	+??++-	8.12E-01	5708	-1.868	0.06	+??---	4.25E-01
519	myristate (14:0)	13556	-0.329	0.74	+-++--	0	0.71	0.92	6404	1.362	0.17	++++--	8.21E-01	7151	-1.102	0.27	+-----	6.61E-01
1218	acetoacetate	10401	0.344	0.73	???-+-	0	0.48	0.92	4926	1.303	0.19	???+++	8.30E-01	5475	-0.576	0.56	???-+-	8.55E-01
1668	taurodeoxycholate	11811	-0.328	0.74	--?+-+	0	0.42	0.92	5563	-1.178	0.24	--?--+	8.59E-01	6248	0.417	0.68	--?+-+	9.04E-01
100006116	methyl-4-hydroxybenzoate sulfate	10847	0.336	0.74	-??+--	51	0.11	0.92	5139	1.074	0.28	+??++-	8.82E-01	5708	-0.654	0.51	-??+--	8.34E-01
100006642	glycodeoxycholate sulfate	10847	-0.331	0.74	-??--+	0	0.75	0.92	5139	-0.895	0.37	-??-++	9.04E-01	5708	0.025	0.98	-??+--	9.94E-01

100009004	1-(1-enyl-stearoyl)-2-docosahexaenoyl-GPE (P-18:0/22:6)*	9802	0.341	0.73	???+-?	76	0.04	0.92	4642	0.422	0.67	???+-?	9.48E-01	5160	0.341	0.73	???+-?	9.23E-01
100001652	2-palmitoylglycerophosphoethanolamine*	10766	0.343	0.73	?-?++?	0	0.68	0.92	5066	0.383	0.70	?-?-+?	9.52E-01	5700	0.401	0.69	?-?+-?	9.07E-01
100001776	2-linoleoylglycerophosphoethanolamine*	10766	-0.335	0.74	?-?--?	0	0.98	0.92	5066	-0.212	0.83	?-?-+?	9.54E-01	5700	0.037	0.97	?+?--?	9.92E-01
310	cystathionine	10847	0.337	0.74	+??+-+	0	0.47	0.92	5139	-0.167	0.87	+??+--	9.73E-01	5708	0.612	0.54	+??-++	8.44E-01
100015793	sphingomyelin (d17:2/16:0, d18:2/15:0)*	6208	-0.34	0.73	-???+-	60.8	0.08	0.92	2932	0.161	0.87	+???+-	9.74E-01	3276	-0.173	0.86	-???--	9.68E-01
100001777	1-oleoyl-GPI (18:1)*	11811	-0.329	0.74	++?+--	30.5	0.22	0.92	5563	0.137	0.89	++?-+-	9.77E-01	6248	-0.269	0.79	+-?+--	9.43E-01
100001456	7-methylguanine	12592	0.338	0.74	+?+-++	0	0.85	0.92	5980	0.135	0.89	+?++--	9.77E-01	6611	0.062	0.95	+?--++	9.85E-01
100001743	tryptophan betaine	13556	0.339	0.73	-++++-	6.1	0.38	0.92	6404	-0.039	0.97	-+++--	9.92E-01	7151	0.327	0.74	-+--+-	9.24E-01
100000016	suberate (octanedioate)	10847	-0.325	0.75	+??--+	0	0.55	0.92	5139	-0.687	0.49	-??+-+	9.40E-01	5708	0.104	0.92	+??-++	9.78E-01
100002749	S-methylcysteine	13556	-0.323	0.75	+--++-	4.1	0.39	0.92	6404	-0.468	0.64	----++	9.47E-01	7151	-0.115	0.91	+--++-	9.77E-01
100000784	theanine	10248	-0.323	0.75	-??+-?	8	0.34	0.92	4855	0.391	0.70	-??+-?	9.49E-01	5393	-0.567	0.57	-??+-?	8.59E-01
100004328	sphingomyelin (d18:1/14:0, d16:1/16:0)*	10847	0.324	0.75	+??+--	74	0.01	0.92	5139	0.196	0.84	-??+--	9.60E-01	5708	0.853	0.39	+??++-	7.77E-01
144	4-hydroxyphenylacetate	5238	-0.319	0.75	???-?+	0	0.62	0.92	2491	1.146	0.25	???+?+	8.69E-01	2747	-1.216	0.22	???-?-	6.31E-01
194	N-formylmethionine	11811	-0.318	0.75	--?-+-	0	0.73	0.92	5563	0.944	0.35	+-?++-	9.04E-01	6248	-1.159	0.25	--?-++	6.41E-01
100000269	glycerophosphorylcholine (GPC)	13556	-0.32	0.75	-+--+-	54.2	0.05	0.92	6404	-0.52	0.60	-+--+-	9.44E-01	7151	0.49	0.62	++-++-	8.80E-01
536	2'-deoxyuridine	6208	-0.279	0.78	+???-+	0	0.86	0.92	2932	1.487	0.14	+???+-	7.92E-01	3276	-1.445	0.15	-???-+	5.63E-01
100001108	3-methylxanthine	13556	-0.287	0.77	-+++--	0	0.81	0.92	6404	-1.394	0.16	--++--	8.12E-01	7151	0.617	0.54	++-+++	8.43E-01
100009407	pimeloylcarnitine/3-methyladipoylcarnitine (C7-DC)	10847	0.285	0.78	-??+--	0	0.42	0.92	5139	1.205	0.23	-??+-+	8.59E-01	5708	-0.479	0.63	-??+--	8.80E-01
100004635	methionine sulfone	10847	0.301	0.76	-??++-	0	0.57	0.92	5139	1.19	0.23	-??+++	8.59E-01	5708	0.09	0.93	-??++-	9.78E-01
100009160	1-(1-enyl-palmitoyl)-2-palmitoleoyl-GPC (P-16:0/16:1)*	10847	-0.284	0.78	+??-+-	0	0.69	0.92	5139	0.983	0.33	-??++-	9.04E-01	5708	-1.366	0.17	+??---	5.78E-01
100015786	sphingomyelin (d18:0/20:0, d16:0/22:0)*	6208	0.304	0.76	+???+-	0	0.83	0.92	2932	-0.714	0.48	+???--	9.37E-01	3276	1.5	0.13	+???++	5.51E-01
100008904	1-stearoyl-2-oleoyl-GPC (18:0/18:1)	10847	-0.283	0.78	+??---	0	0.88	0.92	5139	-0.531	0.60	+??---	9.44E-01	5708	0.636	0.52	+??-++	8.37E-01
100001431	1-pentadecanoylglycerol (15:0)	5684	-0.307	0.76	-??-?+	0	0.79	0.92	2704	-0.577	0.56	-??-?+	9.44E-01	2980	-0.114	0.91	-??+?-	9.77E-01
100006374	1,2,3-benzenetriol sulfate (2)	10847	0.297	0.77	+??-+-	62.6	0.05	0.92	5139	0.501	0.62	+??+--	9.47E-01	5708	0.088	0.93	-??-+-	9.78E-01
432	nicotinamide	11811	0.278	0.78	-+?+++	43.5	0.13	0.92	5563	-0.397	0.69	--?+--	9.48E-01	6248	0.831	0.41	-+?+++	7.84E-01
100001570	1-linoleoyl-GPE (18:2)*	13556	0.284	0.78	+++-+-	26.3	0.24	0.92	6404	0.429	0.67	+--++-	9.48E-01	7151	0.557	0.58	+++-++	8.62E-01
100008919	1-(1-enyl-stearoyl)-2-oleoyl-GPE (P-18:0/18:1)	6208	0.308	0.76	+???-+	0	0.59	0.92	2932	0.427	0.67	+???++	9.48E-01	3276	0.494	0.62	+???++	8.80E-01
100001266	N-acetylarginine	10847	0.282	0.78	-??++-	0	0.97	0.92	5139	0.411	0.68	-??++-	9.48E-01	5708	0.174	0.86	+??-++	9.68E-01
330	fumarate	10847	0.309	0.76	+??-+-	0	0.90	0.92	5139	0.41	0.68	+??-++	9.48E-01	5708	-0.101	0.92	+??+--	9.78E-01
100000580	1,5-anhydroglucitol (1,5-AG)	13556	0.291	0.77	--++++	0	0.73	0.92	6404	-0.268	0.79	+-++--	9.54E-01	7151	0.949	0.34	---+++	7.33E-01
100001092	trigonelline (N'-methylnicotinate)	11811	-0.293	0.77	++?---	65	0.02	0.92	5563	0.289	0.77	++?+--	9.54E-01	6248	-0.675	0.50	++?-+-	8.23E-01
100002784	2-oxoarginine*	6208	0.305	0.76	+???+-	0	0.41	0.92	2932	-0.33	0.74	+???--	9.54E-01	3276	0.629	0.53	-???++	8.40E-01
100009008	1-(1-enyl-palmitoyl)-2-docosahexaenoyl-GPC (P-16:0/22:6)*	9802	0.281	0.78	???+-?	56.9	0.13	0.92	4642	0.155	0.88	???+-?	9.76E-01	5160	0.473	0.64	???+-?	8.83E-01
179	9,10-DiHOME	10847	-0.288	0.77	-??-++	1.3	0.39	0.92	5139	0.073	0.94	+??-++	9.92E-01	5708	-0.451	0.65	-??-+-	8.98E-01
100015791	sphingomyelin (d18:2/23:1)*	6208	-0.295	0.77	+???--	0	0.60	0.92	2932	-0.067	0.95	+???+-	9.92E-01	3276	0.021	0.98	-???-+	9.95E-01
100001232	5-dodecenoate (12:1n7)	13556	-0.292	0.77	+--++-	0	0.94	0.92	6404	-0.017	0.99	++-+--	9.94E-01	7151	-0.175	0.86	---++-	9.68E-01
100006121	1-dihomo-linolenylglycerol (20:3)	10847	-0.273	0.78	-??+--	5.5	0.37	0.93	5139	-0.785	0.43	-??+--	9.11E-01	5708	0.604	0.55	-??++-	8.46E-01
100010940	diacylglycerol (16:1/18:2 [2], 16:0/18:3 [1])*	1045	0.266	0.79	+????-	13.1	0.28	0.93	497	-1.051	0.29	-????-	8.91E-01	548	1.492	0.14	+????+	5.54E-01
100009036	1-margaroyl-2-oleoyl-GPC (17:0/18:1)*	9802	0.267	0.79	???+-?	0	0.50	0.93	4642	0.787	0.43	???+-?	9.11E-01	5160	0.135	0.89	???-+?	9.77E-01
445	orotate	10401	0.267	0.79	???+--	41.3	0.18	0.93	4926	-0.648	0.52	???+--	9.44E-01	5475	0.55	0.58	???+-+	8.62E-01
340	glycine	12592	0.261	0.79	-?++-+	0	0.90	0.93	5980	0.561	0.57	+?++--	9.44E-01	6611	0.11	0.91	-?++++	9.78E-01
100000437	theophylline	13556	-0.254	0.80	++++--	31.1	0.20	0.93	6404	-1.625	0.10	-+++-+	7.92E-01	7151	0.638	0.52	+++++-	8.35E-01
1105	alpha-tocopherol	12592	-0.257	0.80	-?++--	44	0.13	0.93	5980	0.217	0.83	-?+++-	9.54E-01	6611	-0.284	0.78	-?-+--	9.39E-01
1137	oleoyl ethanolamide	10847	-0.257	0.80	+??---	0	0.89	0.93	5139	0.147	0.88	+??-++	9.77E-01	5708	-0.414	0.68	-??+--	9.06E-01
100002129	pregnenolone sulfate	11811	-0.25	0.80	+-?+--	18.8	0.29	0.94	5563	-0.03	0.98	+-?+-+	9.92E-01	6248	-0.787	0.43	+-?+--	7.93E-01
100002128	17alpha-hydroxypregnenolone 3-sulfate	1045	0.229	0.82	+????-	72.5	0.06	0.94	497	0.855	0.39	+????+	9.04E-01	548	-0.537	0.59	+????-	8.63E-01
1123	chenodeoxycholate	10847	-0.232	0.82	+??---	0	0.59	0.94	5139	-0.938	0.35	+??---	9.04E-01	5708	0.463	0.64	+??-+-	8.91E-01
461	phosphate	13556	-0.228	0.82	-++---	0.5	0.41	0.94	6404	-0.386	0.70	-++--+	9.51E-01	7151	0.093	0.93	-+++--	9.78E-01
100001409	N1-methylinosine	10847	-0.229	0.82	+??--+	0	0.66	0.94	5139	-0.144	0.89	+??+-+	9.77E-01	5708	-0.086	0.93	+??-++	9.78E-01
100001413	N4-acetylcytidine	6573	0.224	0.82	--??+?	0	0.60	0.95	3072	-0.043	0.97	+-??+?	9.92E-01	3501	0.376	0.71	-+??+?	9.15E-01

100001556	2-oleoylglycerophosphocholine*	10766	0.221	0.83	?+?+-?	0	0.81	0.95	5066	1.304	0.19	?+?++?	8.30E-01	5700	-0.075	0.94	?-?+-?	9.79E-01
100004499	6-oxopiperidine-2-carboxylate	10847	0.216	0.83	-??+-+	0	0.44	0.95	5139	0.882	0.38	-??++-	9.04E-01	5708	-0.395	0.69	+??+-+	9.07E-01
100004322	2-aminophenol sulfate	10847	-0.213	0.83	+??-+-	0	0.77	0.95	5139	0.231	0.82	+??-+-	9.54E-01	5708	-0.309	0.76	-??-+-	9.27E-01
100001562	2-palmitoyl-GPC (16:0)*	13556	0.196	0.85	+--+--	51	0.07	0.95	6404	1.328	0.18	++-++-	8.30E-01	7151	-0.399	0.69	+--+--	9.07E-01
1342	3-methoxytyrosine	13556	0.198	0.84	+++--+	18.7	0.29	0.95	6404	1.244	0.21	+++++-	8.48E-01	7151	-0.786	0.43	-++--+	7.93E-01
363	myo-inositol	12592	-0.2	0.84	-?++--	38.9	0.16	0.95	5980	1.214	0.22	+?++--	8.59E-01	6611	-1.124	0.26	-?-+--	6.61E-01
132	3-phosphoglycerate	9802	0.192	0.85	???+-?	0	0.55	0.95	4642	1.098	0.27	???++?	8.74E-01	5160	-0.494	0.62	???+-?	8.80E-01
100010925	palmitoyl-arachidonoyl-glycerol (16:0/20:4) [2]*	6208	0.191	0.85	-???+-	0	0.72	0.95	2932	-0.937	0.35	-???--	9.04E-01	3276	1.298	0.19	+???++	6.12E-01
100006126	4-vinylguaiacol sulfate	10248	0.198	0.84	+??-+?	64.5	0.06	0.95	4855	0.973	0.33	+??-+?	9.04E-01	5393	-0.544	0.59	-??-+?	8.62E-01
1206	isocitrate	10401	-0.209	0.83	???-+-	0	0.38	0.95	4926	-0.787	0.43	???+--	9.11E-01	5475	0.633	0.53	???-+-	8.38E-01
100000707	maleate	10847	-0.202	0.84	+??--+	80	0.00	0.95	5139	0.761	0.45	+??+-+	9.21E-01	5708	-1.034	0.30	+??--+	6.92E-01
100006184	2-methoxyresorcinol sulfate	9802	0.204	0.84	???-+?	0	0.38	0.95	4642	0.711	0.48	???++?	9.37E-01	5160	-0.198	0.84	???-+?	9.67E-01
460	phenylalanine	13556	-0.21	0.83	--++--	0	0.46	0.95	6404	-0.339	0.73	+-++--	9.52E-01	7151	-0.188	0.85	--++-+	9.68E-01
100001170	3-hydroxy-2-ethylpropionate	10847	-0.192	0.85	+??+--	50.3	0.11	0.95	5139	-0.011	0.99	+??++-	9.95E-01	5708	-0.205	0.84	+??---	9.66E-01
275	creatinine	13556	-0.192	0.85	+--++-	0	0.76	0.95	6404	-0.006	1.00	+--+--	9.96E-01	7151	-0.196	0.84	----++	9.68E-01
100001611	3beta,7alpha-dihydroxy-5-cholestenoate	1045	0.19	0.85	-????+	0	0.73	0.95	497	0.844	0.40	-????+	9.04E-01	548	-0.543	0.59	+????-	8.62E-01
100001731	indoleacetylglutamine	6208	-0.188	0.85	+???+-	0	0.41	0.95	2932	0.258	0.80	+???+-	9.54E-01	3276	-0.267	0.79	-???+-	9.43E-01
100015962	N-trimethyl 5-aminovalerate	7953	0.188	0.85	+?+?-+	0	0.71	0.95	3773	-0.061	0.95	-?-?+-	9.92E-01	4179	0.719	0.47	+?+?-+	8.05E-01
344	guanidinoacetate	10847	0.183	0.86	+??+--	3.7	0.37	0.96	5139	2.258	0.02	+??++-	6.08E-01	5708	-1.17	0.24	+??--+	6.40E-01
100000840	tartronate (hydroxymalonate)	10847	0.183	0.85	+??++-	67.7	0.03	0.96	5139	1.129	0.26	+??++-	8.69E-01	5708	-0.761	0.45	+??-+-	7.97E-01
504	serotonin	12592	0.18	8.54E-01	-?-+++	65.5	0.02	9.55E-01	5980	0.457	0.65	-?++-+	9.48E-01	6611	-0.438	0.66	-?-+++	9.01E-01
100001193	adrenate (22:4n6)	13556	-0.166	0.87	++++-+	0	0.71	0.96	6404	2.018	0.04	-+++++	6.34E-01	7151	-1.57	0.12	+-+---	5.31E-01
100001295	gamma-glutamyltryptophan	6208	-0.17	0.86	+???--	0	0.96	0.96	2932	1.557	0.12	+???++	7.92E-01	3276	-1.302	0.19	-???--	6.12E-01
100000054	5-hydroxylysine	10847	-0.166	0.87	+??-++	35.2	0.20	0.96	5139	-1.128	0.26	+??---	8.69E-01	5708	0.696	0.49	+??-++	8.20E-01
100000792	dehydroisoandrosterone sulfate (DHEA-S)	13556	-0.169	0.87	+-++--	0	0.54	0.96	6404	-0.872	0.38	--+--+	9.04E-01	7151	0.128	0.90	+-+++-	9.77E-01
1547	N-stearoyl-sphingosine (d18:1/18:0)*	6208	0.166	0.87	+???-+	82.9	0.00	0.96	2932	-0.782	0.43	+???-+	9.11E-01	3276	1.408	0.16	+???++	5.64E-01
100001925	cyclo(leu-pro)	5603	0.163	0.87	?+?-??	0	0.58	0.96	2631	0.806	0.42	?+?+??	9.11E-01	2972	-0.585	0.56	?-?-??	8.55E-01
100001229	stearidonate (18:4n3)	12592	-0.165	0.87	+?++-+	0	0.74	0.96	5980	0.76	0.45	+?++++	9.21E-01	6611	-0.486	0.63	-?++--	8.80E-01
1261	12-HHTrE	1045	-0.165	0.87	-????+	0	0.36	0.96	497	0.531	0.60	-????+	9.44E-01	548	-1.124	0.26	-????-	6.61E-01
158	5,6-dihydrothymine	10847	-0.177	0.86	+??+--	0	0.90	0.96	5139	-0.621	0.53	+??---	9.44E-01	5708	0.22	0.83	-??+++	9.59E-01
100006361	dopamine 3-O-sulfate	10847	0.175	0.86	+??--+	65.4	0.03	0.96	5139	0.348	0.73	+??---	9.52E-01	5708	-0.155	0.88	+??--+	9.72E-01
100000036	3-methyl-2-oxovalerate	13556	0.161	0.87	+-+--+	67.4	0.01	0.96	6404	-0.228	0.82	+-+--+	9.54E-01	7151	0.517	0.61	--++++	8.70E-01
100000015	xanthurenate	10847	0.18	0.86	-??++-	67	0.03	0.96	5139	-0.122	0.90	+??+--	9.81E-01	5708	0.146	0.88	-??++-	9.75E-01
917	asparagine	12592	-0.175	0.86	-?-++-	0	0.41	0.96	5980	-0.027	0.98	-?-+--	9.92E-01	6611	-0.175	0.86	-?--+-	9.68E-01
100000467	3-indoxyl sulfate	13556	-0.159	0.87	++--+-	49.8	0.08	0.96	6404	-0.655	0.51	++++-+	9.44E-01	7151	0.245	0.81	++--+-	9.50E-01
500	riboflavin (Vitamin B2)	5762	0.156	0.88	????+-	0	0.33	0.96	2719	1.171	0.24	????+-	8.59E-01	3043	-0.641	0.52	????--	8.35E-01
100002126	16a-hydroxy DHEA 3-sulfate	10847	-0.156	0.88	+??+-+	4.4	0.37	0.96	5139	-1.101	0.27	+??--+	8.74E-01	5708	0.167	0.87	+??+--	9.70E-01
100001048	2-palmitoylglycerol (16:0)	5684	0.153	0.88	+??-?+	0	0.90	0.96	2704	0.34	0.73	+??+?-	9.52E-01	2980	-0.213	0.83	+??-?+	9.63E-01
100009030	lactosyl-N-palmitoyl-sphingosine (d18:1/16:0)	10847	-0.154	0.88	+??+--	54.5	0.09	0.96	5139	0.219	0.83	+??+--	9.54E-01	5708	-0.702	0.48	+??+-+	8.14E-01
100000551	4-methyl-2-oxopentanoate	13556	0.153	0.88	--+-+-	73.5	0.00	0.96	6404	0.019	0.98	--+-+-	9.94E-01	7151	0.309	0.76	--+-+-	9.27E-01
100009406	palmitoleoylcarnitine (C16:1)*	10847	-0.148	0.88	+??+--	13.2	0.33	0.96	5139	1.888	0.06	+??++-	7.45E-01	5708	-1.176	0.24	+??---	6.40E-01
100010918	oleoyl-oleoyl-glycerol (18:1/18:1) [1]*	6208	0.148	0.88	+???+-	0	0.41	0.96	2932	-1.288	0.20	-???--	8.30E-01	3276	1.857	0.06	+???++	4.25E-01
100000011	phenylacetate	5762	0.15	0.88	????+-	56.7	0.13	0.96	2719	-0.04	0.97	????+-	9.92E-01	3043	-0.202	0.84	????+-	9.66E-01
100001198	myristoleate (14:1n5)	13556	-0.135	0.89	+-+---	0	0.95	0.97	6404	1.498	0.13	+++++-	7.92E-01	7151	-0.917	0.36	--+---	7.47E-01
331	gamma-glutamylglutamate	10847	-0.13	0.90	+??+-+	38.5	0.18	0.97	5139	1.65	0.10	+??++-	7.92E-01	5708	-0.688	0.49	+??--+	8.22E-01
100000096	4-guanidinobutanoate	10847	0.118	0.91	+??-+-	16.9	0.31	0.97	5139	0.991	0.32	+??-+-	9.04E-01	5708	-0.339	0.73	-??-+-	9.23E-01
100010928	linoleoyl-docosahexaenoyl-glycerol (18:2/22:6) [1]*	1045	0.113	0.91	+????-	71.9	0.06	0.97	497	-0.815	0.42	+????-	9.11E-01	548	0.969	0.33	+????-	7.20E-01
100001263	1-palmitoyl-GPC (16:0)	13556	-0.114	0.91	---++-	71.7	0.00	0.97	6404	0.8	0.42	-+-++-	9.11E-01	7151	-0.212	0.83	---++-	9.63E-01
100009131	1-linoleoyl-2-arachidonoyl-GPC (18:2/20:4n6)*	10248	-0.121	0.90	-??-+?	42.6	0.18	0.97	4855	-0.772	0.44	-??-+?	9.13E-01	5393	0.605	0.54	-??++?	8.46E-01
100000808	cysteine s-sulfate	10401	0.133	0.89	???-++	0	0.69	0.97	4926	0.689	0.49	???-++	9.40E-01	5475	-0.533	0.59	???---	8.65E-01

100001651	2-oleoylglycerophosphoethanolamine*	5603	0.129	0.90	?-?+??	0	0.89	0.97	2631	-0.543	0.59	?+?-??	9.44E-01	2972	0.546	0.59	?-?+??	8.62E-01
827	cytidine	6208	-0.131	0.90	-???++	0	0.55	0.97	2932	-0.645	0.52	-???--	9.44E-01	3276	0.377	0.71	-???++	9.15E-01
100006379	C-glycosyltryptophan	12592	0.113	0.91	+?+--+	0	0.44	0.97	5980	0.562	0.57	+?++--	9.44E-01	6611	-0.321	0.75	-?+--+	9.24E-01
100004552	1-eicosapentaenoylglycerophosphoethanolamine*	10766	-0.128	0.90	?-?+-?	5.9	0.35	0.97	5066	0.664	0.51	?-?++?	9.44E-01	5700	-0.264	0.79	?-?+-?	9.43E-01
100001739	dihomo-linolenate (20:3n3 or n6)	13556	-0.121	0.90	--++--	0	0.69	0.97	6404	0.211	0.83	+++---	9.54E-01	7151	0.004	1.00	--++--	9.99E-01
100001999	21-hydroxypregnenolone disulfate	11811	-0.115	0.91	+-?+--	0	0.81	0.97	5563	0.202	0.84	+-?+-+	9.57E-01	6248	-0.577	0.56	++?---	8.55E-01
100010955	perfluorooctanesulfonic acid (PFOS)	6208	0.129	0.90	-???+-	0	0.93	0.97	2932	0.205	0.84	+???+-	9.57E-01	3276	0.512	0.61	-???+-	8.71E-01
100001275	phenylacetylglycine	9802	-0.114	0.91	???+-?	79.9	0.03	0.97	4642	0.176	0.86	???+-?	9.70E-01	5160	-0.446	0.66	???+-?	8.99E-01
241	phenylpyruvate	10847	-0.134	0.89	-??+--	30.9	0.23	0.97	5139	-0.032	0.97	+??-+-	9.92E-01	5708	-0.481	0.63	-??+--	8.80E-01
100008954	palmitoyl dihydrosphingomyelin (d18:0/16:0)*	10847	0.117	0.91	-??+--	28.4	0.24	0.97	5139	-0.029	0.98	+??+--	9.92E-01	5708	0.334	0.74	-??+--	9.24E-01
100001125	threonylphenylalanine	1045	0.117	0.91	-????+	0	0.82	0.97	497	0.061	0.95	-????+	9.92E-01	548	-0.188	0.85	-????-	9.68E-01
100002952	docosadioate	6208	0.114	0.91	-???+-	0	0.85	0.97	2932	0.057	0.95	-???++	9.92E-01	3276	0.154	0.88	+???+-	9.72E-01
1488	arachidonoyl ethanolamide	1045	0.118	0.91	-????+	0	0.52	0.97	497	-0.086	0.93	-????+	9.92E-01	548	-0.03	0.98	-????+	9.94E-01
424	palmitate (16:0)	13556	-0.105	0.92	+-++--	0	0.90	0.97	6404	1.8	0.07	++++++	7.73E-01	7151	-1.232	0.22	+-----	6.29E-01
100000626	sphingosine 1-phosphate	10847	-0.099	0.92	-??+--	43.3	0.15	0.97	5139	1.543	0.12	-??+++	7.92E-01	5708	-0.925	0.35	-??+--	7.42E-01
100001181	docosapentaenoate (n3 DPA; 22:5n3)	13556	-0.093	0.93	+--+--	0	0.84	0.97	6404	1.274	0.20	++-+++	8.30E-01	7151	-0.658	0.51	---+--	8.32E-01
100001322	ADSGEGDFXAEGGGVR*	7953	-0.091	0.93	+?+?-+	63.3	0.04	0.97	3773	-1.137	0.26	-?-?-+	8.69E-01	4179	0.547	0.58	+?+?-+	8.62E-01
826	xylose	6208	0.091	0.93	+???-+	0	0.50	0.97	2932	0.881	0.38	-???++	9.04E-01	3276	-0.406	0.68	+???-+	9.06E-01
100006614	adipoylcarnitine (C6-DC)	10847	0.098	0.92	-??+-+	0	0.70	0.97	5139	0.848	0.40	-??++-	9.04E-01	5708	-0.187	0.85	+??--+	9.68E-01
100001294	gamma-glutamylglycine	10847	0.103	0.92	+??+--	0	0.40	0.97	5139	0.848	0.40	+??+--	9.04E-01	5708	-0.014	0.99	+??+-+	9.96E-01
35	S-1-pyrroline-5-carboxylate	10847	0.106	0.92	-??+-+	35.2	0.20	0.97	5139	0.794	0.43	-??+++	9.11E-01	5708	-0.41	0.68	-??--+	9.06E-01
100000827	1-palmitoylglycerol (16:0)	13556	-0.09	0.93	-+++--	0	0.89	0.97	6404	0.699	0.48	++++-+	9.40E-01	7151	-0.578	0.56	--++--	8.55E-01
913	maltose	10847	-0.097	0.92	-??-++	61.5	0.05	0.97	5139	-0.624	0.53	-??--+	9.44E-01	5708	0.349	0.73	+??-++	9.21E-01
100002154	ergothioneine	12592	-0.093	0.93	-?-++-	43.9	0.13	0.97	5980	-0.478	0.63	-?--+-	9.47E-01	6611	0.309	0.76	-?-++-	9.27E-01
100001423	4-hydroxyhippurate	11811	0.092	0.93	++?+--	0	0.79	0.97	5563	-0.414	0.68	+-?+--	9.48E-01	6248	0.348	0.73	++?++-	9.21E-01
100005351	1-eicosapentaenoylglycerophosphocholine (20:5n3)*	10766	-0.093	0.93	?-?+-?	0	0.54	0.97	5066	0.23	0.82	?+?+-?	9.54E-01	5700	0.153	0.88	?-?+-?	9.72E-01
100001674	2-arachidonoylglycerophosphoethanolamine*	10766	0.107	0.91	?-?+-?	0	0.63	0.97	5066	-0.168	0.87	?+?-+?	9.73E-01	5700	0.579	0.56	?-?+-?	8.55E-01
100006115	arabonate/xylonate	10847	-0.087	0.93	+??-++	26.6	0.25	0.97	5139	1.195	0.23	+??-++	8.59E-01	5708	-0.719	0.47	+??---	8.05E-01
100015623	lactosyl-N-behenoyl-sphingosine (d18:1/22:0)*	6208	-0.083	0.93	+???+-	0	0.91	0.97	2932	-0.913	0.36	+???--	9.04E-01	3276	0.431	0.67	-???+-	9.01E-01
100001734	N6-acetyllysine	10847	0.078	0.94	+??+--	0	0.41	0.97	5139	-1.398	0.16	+??+--	8.12E-01	5708	0.983	0.33	+??++-	7.14E-01
100006191	p-cresol-glucuronide*	10847	-0.079	0.94	+??-+-	54.7	0.08	0.97	5139	-0.44	0.66	+??---	9.48E-01	5708	-0.025	0.98	+??--+	9.94E-01
100000491	gamma-glutamylphenylalanine	13556	-0.079	0.94	+-+---	0	0.77	0.97	6404	0.287	0.77	++-+--	9.54E-01	7151	-0.117	0.91	+-+-++	9.77E-01
100009135	1-(1-enyl-stearoyl)-2-linoleoyl-GPC (P-18:0/18:2)*	9802	0.07	0.94	???+-?	39.9	0.20	0.97	4642	1.937	0.05	???++?	6.99E-01	5160	-1.475	0.14	???--?	5.54E-01
409	malate	13556	0.07	0.94	++++-+	0	0.50	0.97	6404	1.781	0.07	-+++++	7.73E-01	7151	-1.185	0.24	+-++--	6.40E-01
1231	dihomo-linoleate (20:2n6)	13556	-0.067	0.95	+--+--	0	0.95	0.97	6404	1.568	0.12	++++++	7.92E-01	7151	-1.222	0.22	+--+--	6.31E-01
100001612	N-acetyl-aspartyl-glutamate (NAAG)	10401	0.07	0.94	???-++	49.8	0.14	0.97	4926	0.973	0.33	???+++	9.04E-01	5475	-0.899	0.37	???-+-	7.54E-01
100002528	sulfate*	10847	0.069	0.95	-??++-	70.2	0.02	0.97	5139	0.66	0.51	+??++-	9.44E-01	5708	-0.616	0.54	-??-+-	8.43E-01
100001104	N-acetyltyrosine	10847	0.073	0.94	-??-++	0	0.93	0.97	5139	-0.184	0.85	-??---	9.68E-01	5708	0.224	0.82	-??-++	9.58E-01
828	arabinose	10401	-0.066	0.95	???-++	0	0.83	0.97	4926	0.472	0.64	???++-	9.47E-01	5475	-0.345	0.73	???-++	9.22E-01
980	pentadecanoate (15:0)	7429	0.063	0.95	+?++?-	0.3	0.39	0.97	3545	2.089	0.04	+?++?-	6.18E-01	3883	-1.327	0.18	+?--?-	5.98E-01
100010923	linoleoyl-arachidonoyl-glycerol (18:2/20:4) [2]*	1045	-0.056	0.96	-????+	36.2	0.21	0.98	497	-1.249	0.21	-????-	8.48E-01	548	1.053	0.29	+????+	6.84E-01
297	sphingosine	11811	-0.054	0.96	--?+-+	0	1.00	0.98	5563	0.623	0.53	++?+-+	9.44E-01	6248	-0.679	0.50	--?-+-	8.23E-01
100005466	N-acetyltaurine	10847	0.056	0.96	+??--+	0	0.52	0.98	5139	0.622	0.53	+??+-+	9.44E-01	5708	-0.121	0.90	+??-+-	9.77E-01
100001408	2-myristoylglycerol (14:0)	5085	0.054	0.96	+??-??	0	0.45	0.98	2420	0.306	0.76	+??-??	9.54E-01	2665	-0.119	0.91	+??-??	9.77E-01
100002067	pregn steroid monosulfate C21H34O5S*	11811	-0.055	0.96	+-?++-	0	0.44	0.98	5563	0.016	0.99	+-?+-+	9.94E-01	6248	-0.32	0.75	+-?++-	9.24E-01
100000963	homocitrulline	12592	-0.048	0.96	+?+-+-	0	0.90	0.98	5980	0.529	0.60	+?+-+-	9.44E-01	6611	-0.431	0.67	-?+---	9.01E-01
537	trans-urocanate	6208	0.045	0.96	+???--	0	0.96	0.98	2932	-0.458	0.65	+???-+	9.48E-01	3276	0.319	0.75	-???+-	9.24E-01
100001948	succinylcarnitine (C4-DC)	13556	-0.044	0.96	---+--	0	1.00	0.98	6404	-0.138	0.89	+--++-	9.77E-01	7151	0.476	0.63	-+--++	8.81E-01
1052	glycerate	12592	-0.045	0.96	+?++--	56	0.06	0.98	5980	0.078	0.94	+?+---	9.92E-01	6611	-0.133	0.89	+?++--	9.77E-01
100001990	taurocholenate sulfate	10401	0.038	0.97	???+-+	0	0.79	0.98	4926	-0.577	0.56	???--+	9.44E-01	5475	0.096	0.92	???+--	9.78E-01

100015609	N-palmitoyl-sphingadienine (d18:2/16:0)*	6208	0.029	0.98	-???+-	0	0.72	0.99	2932	-0.599	0.55	-???--	9.44E-01	3276	0.71	0.48	-???++	8.10E-01
100009035	1-pentadecanoyl-2-linoleoyl-GPC (15:0/18:2)*	9802	0.028	0.98	???-+?	0	0.69	0.99	4642	0.216	0.83	???+-?	9.54E-01	5160	-0.202	0.84	???-+?	9.66E-01
100004329	sphingomyelin (d18:2/16:0, d18:1/16:1)*	10847	0.028	0.98	-??+--	64.7	0.04	0.99	5139	0.141	0.89	-??+--	9.77E-01	5708	0.366	0.71	-??+--	9.18E-01
100006641	glycochenodeoxycholate sulfate	10847	-0.025	0.98	-??-+-	0	0.93	0.99	5139	-0.44	0.66	-??+-+	9.48E-01	5708	0.08	0.94	+??-+-	9.78E-01
100001615	octadecanedioate	7429	-0.021	0.98	+?--?+	26.1	0.26	0.99	3545	-0.223	0.82	+?--?+	9.54E-01	3883	0.057	0.95	+?--?-	9.87E-01
100001359	aconitate [cis or trans]	5684	0.016	0.99	+??-?-	0	0.65	0.99	2704	0.895	0.37	+??+?+	9.04E-01	2980	-0.439	0.66	+??-?-	9.01E-01
100000258	glycerol 3-phosphate	12592	0.017	0.99	+?-++-	0	0.59	0.99	5980	0.502	0.62	-?++--	9.47E-01	6611	0.015	0.99	+?--+-	9.96E-01
100005371	1-eicosatrienoylglycerophosphoethanolamine*	10766	-0.014	0.99	?-?++?	0	0.44	0.99	5066	0.628	0.53	?+?-+?	9.44E-01	5700	-0.018	0.99	?-?++?	9.96E-01
100009343	1-linoleoyl-2-linolenoyl-GPC (18:2/18:3)*	10847	0.012	0.99	+??-+-	44.1	0.15	0.99	5139	1.109	0.27	+??++-	8.74E-01	5708	-0.602	0.55	+??-+-	8.47E-01
100001765	3-methyladipate	1045	-0.011	0.99	+????-	62	0.10	0.99	497	0.452	0.65	+????-	9.48E-01	548	-0.432	0.67	+????-	9.01E-01
100001435	1-linolenoylglycerol (18:3)	10847	0.006	1.00	+??-++	0	0.51	1.00	5139	-0.769	0.44	+??--+	9.15E-01	5708	0.932	0.35	+??-++	7.42E-01

Supplementary Table 6: Results of the sensitivity analysis, excluding the cohorts which measured metabolites on older Metabolon platforms
The order of the direction column: RS, EPIC-Norfolk B2, EPIC-Norfolk B3, NEO. Significant results are highlighted (FDR corrected p-value < 0.05).

Metabolite	Name	N	Zscore	P.value	Direction	HetISq	HetPVal	FDR
100002049	4.hydroxycoumarin	10847	-4.479	7.51E-06	----	79.7	1.99E-03	3.93E-03
100001740	mannitol.sorbitol	10847	4.303	1.68E-05	++++	0	5.78E-01	5.87E-03
498	retinol.VitaminA.	10847	3.894	9.88E-05	++++	0	8.46E-01	2.59E-02
100000010	3.phenylpropionate.hydrocinnamate.	10847	-3.8	1.45E-04	----	63.9	3.98E-02	3.03E-02
100000014	hippurate	10847	-3.733	1.89E-04	----	69.4	2.03E-02	3.30E-02
100008984	1.palmitoyl.2.palmitoleoyl.GPC.16.0.16.1.	10847	3.576	3.49E-04	++++	0	4.30E-01	4.75E-02
100009082	1.linoleoyl.GPA.18.2.	10847	-3.566	3.63E-04	----	60.6	5.46E-02	4.75E-02
100002253	cinnamoylglycine	10847	-3.504	4.58E-04	----	51.4	1.03E-01	4.80E-02
100001121	pyridoxate	10847	3.433	5.97E-04	+++-	54.6	8.53E-02	5.68E-02
100004227	2.aminooctanoate	10847	-3.386	7.09E-04	----	38.6	1.81E-01	6.19E-02
100001977	beta.cryptoxanthin	6208	-3.253	1.14E-03	-?--	87.3	3.75E-04	7.80E-02
100005372	1..1.enyl.oleoyl..GPE.P.18.1.	10847	-3.161	1.57E-03	----	26.4	2.54E-01	9.69E-02
100001951	bilirubin.E.ZorZ.E.	10847	-3.13	1.75E-03	----	38.4	1.81E-01	1.02E-01
1090	bilirubin.Z.Z.	10847	-3.105	1.90E-03	----	0	4.61E-01	1.05E-01
100001197	10.undecenoate.11.1n1.	10847	-2.937	3.32E-03	+---	10.8	3.39E-01	1.58E-01
100008914	1.palmitoyl.2.arachidonoyl.GPC.16.0.20.4n6.	10847	2.923	3.47E-03	-+++	19	2.95E-01	1.58E-01
391	citrulline	10847	-2.956	3.12E-03	----	0	7.40E-01	1.58E-01
1539	1.palmitoyl.2.oleoyl.GPC.16.0.18.1.	10847	2.93	3.39E-03	+++-	26.1	2.55E-01	1.58E-01
100001868	4.allylphenolsulfate	10847	-2.858	4.27E-03	--+-	77.9	3.52E-03	1.86E-01
1024	pantothenate	10847	2.844	4.45E-03	++++	0	8.24E-01	1.86E-01
100002458	3.methylglutaconate	10847	2.794	5.21E-03	++++	0	4.86E-01	2.00E-01
98	kynurenate	10847	-2.785	5.35E-03	----	42.4	1.57E-01	2.00E-01
250	biliverdin	10847	-2.699	6.96E-03	----	0	7.39E-01	2.51E-01
100001950	bilirubin.E.E.	10847	-2.671	7.57E-03	----	0	6.16E-01	2.56E-01
1526	1.palmitoyl.2.oleoyl.GPE.16.0.18.1.	10847	2.635	8.41E-03	-+++	0	3.95E-01	2.75E-01
100003179	leucylalanine	1045	-2.592	9.54E-03	-??-	0	6.82E-01	3.03E-01
100008915	1.palmitoyl.2.docosahexaenoyl.GPC.16.0.22.6.	10401	2.502	1.24E-02	?++-	67.4	4.66E-02	3.16E-01
189	N6.N6.N6.trimethyllysine	10847	-2.555	1.06E-02	----	0	9.40E-01	3.16E-01
100002488	isoursodeoxycholate	6208	2.537	1.12E-02	+?++	0	4.50E-01	3.16E-01
100001112	3.hydroxylaurate	10847	-2.542	1.10E-02	----	16.2	3.10E-01	3.16E-01
100015759	stearoylcholine	6208	-2.505	1.23E-02	-?--	51.4	1.28E-01	3.16E-01
2051	methylsuccinate	10847	-2.506	1.22E-02	+---	0	4.13E-01	3.16E-01
100004318	indolin.2.one	5684	-2.554	1.06E-02	+-?-	0	5.83E-01	3.16E-01
100000743	2.hydroxyoctanoate	10847	-2.483	1.30E-02	--+-	55.7	7.98E-02	3.25E-01
100001400	1.methylurate	10847	-2.336	1.95E-02	----	0	7.68E-01	3.46E-01
100001567	1.palmitoyl.GPE.16.0.	10847	2.435	1.49E-02	+++-	0	6.50E-01	3.46E-01
100001403	5.acetylamino.6.amino.3.methyluracil	10847	-2.332	1.97E-02	-+--	53.7	9.04E-02	3.46E-01
100008991	1.palmitoyl.2.docosahexaenoyl.GPE.16.0.22.6.	10401	2.43	1.51E-02	?+++	0	8.06E-01	3.46E-01
100001511	1.palmitoleoyl.GPC.16.1.	10847	2.345	1.90E-02	+++-	0	6.76E-01	3.46E-01
100001510	phenolsulfate	10847	2.318	2.05E-02	-+++	46.4	1.33E-01	3.46E-01
100003915	palmiticamide	10401	2.35	1.88E-02	?-++	64.4	6.05E-02	3.46E-01
100015834	lignoceroylcarnitine.C24.	6208	-2.322	2.03E-02	-?--	54.7	1.10E-01	3.46E-01
1022	picolinate	6208	-2.355	1.85E-02	+?--	87.2	4.17E-04	3.46E-01
100004054	margaroylcarnitine	6208	-2.399	1.64E-02	+?--	78.6	9.32E-03	3.46E-01
1094	thyroxine	10847	2.327	2.00E-02	++++	32.9	2.15E-01	3.46E-01
100015851	docosapentaenoylcarnitine.C22.5n3.	5762	-2.305	2.12E-02	??--	0	9.78E-01	3.49E-01
815	tyrosine	10847	-2.288	2.21E-02	----	49	1.17E-01	3.50E-01
923	dihydroorotate	6208	-2.281	2.25E-02	+?-+	0	3.92E-01	3.50E-01
100005391	3..3.hydroxyphenyl.propionatesulfate	10847	-2.29	2.20E-02	----	13.2	3.27E-01	3.50E-01
100006430	arabitol.xylitol	10847	2.258	2.39E-02	++++	0	6.08E-01	3.53E-01
100005353	1.nonadecanoyl.GPC.19.0.	9802	-2.263	2.37E-02	?--?	0	6.12E-01	3.53E-01
100001657	glycolithocholatesulfate	10847	-2.258	2.40E-02	----	0	8.53E-01	3.53E-01
100008992	1.stearoyl.2.docosahexaenoyl.GPE.18.0.22.6.	10401	2.213	2.69E-02	?+++	0	7.19E-01	3.60E-01
100001605	catecholsulfate	10847	-2.222	2.63E-02	+---	50.6	1.08E-01	3.60E-01
100000453	paraxanthine	10847	-2.209	2.72E-02	-+--	43.6	1.50E-01	3.60E-01
100008990	1.palmitoyl.2.arachidonoyl.GPE.16.0.20.4.	10847	2.218	2.65E-02	-+++	0	6.27E-01	3.60E-01
1869	2.hydroxyhippurate.salicylurate.	10847	-2.229	2.58E-02	----	71.1	1.56E-02	3.60E-01
100010927	linoleoyl.linolenoyl.glycerol.18.2.18.3.[2]	5609	-2.192	2.84E-02	-?-?	0	3.39E-01	3.72E-01
100002008	5alpha.androstan.3alpha.17alpha.diolmonosulfate	1045	-2.175	2.96E-02	-??-	0	8.02E-01	3.78E-01
1053	3.ureidopropionate	10847	-2.175	2.96E-02	---+	0	4.70E-01	3.78E-01
100001162	propionylcarnitine.C3.	10847	-2.152	3.14E-02	-+--	44.5	1.44E-01	3.87E-01
100000997	3.hydroxydecanoate	10847	-2.158	3.09E-02	+---	24.8	2.63E-01	3.87E-01
100003210	valylleucine	1045	-2.156	3.11E-02	-??-	0	6.43E-01	3.87E-01
100000442	quinate	10847	-2.142	3.22E-02	+---	55.4	8.10E-02	3.88E-01
100009220	1.oleoyl.2.docosahexaenoyl.GPE.18.1.22.6.	5762	2.146	3.19E-02	??++	0	5.60E-01	3.88E-01
100002945	15.methylpalmitate	10847	-2.134	3.28E-02	+---	15.9	3.12E-01	3.91E-01
192	N.acetylputrescine	10847	2.088	3.68E-02	--++	54.8	8.44E-02	4.10E-01
100000257	glucuronate	10847	2.091	3.65E-02	++++	0	5.66E-01	4.10E-01
100002514	hydantoin.5.propionicacid	10847	-2.1	3.57E-02	+--+	37.6	1.86E-01	4.10E-01
100001055	isobutyrylcarnitine.C4.	10847	-2.091	3.65E-02	----	49.5	1.15E-01	4.10E-01

212	5.methylthioadenosine.MTA.	10847	2.092	3.64E-02	++++	38.9	1.78E-01	4.10E-01
381	2.aminoadipate	10847	-2.078	3.77E-02	----	0	5.90E-01	4.11E-01
566	valine	10847	-2.08	3.75E-02	---+	0	5.03E-01	4.11E-01
100000265	kynurenine	10847	-2.066	3.88E-02	----	0	6.39E-01	4.19E-01
100001402	5.acetylamino.6.formylamino.3.methyluracil	10847	-2.061	3.93E-02	-+--	29.3	2.36E-01	4.20E-01
100002021	5alpha.androstan.3beta.17alpha.dioldisulfate	10847	-2.054	4.00E-02	----	25.5	2.59E-01	4.23E-01
100001211	sebacate.decanedioate.	10847	-2.043	4.11E-02	----	0	4.64E-01	4.30E-01
100001870	1.palmitoyl.2.linoleoyl.GPE.16.0.18.2.	10847	2.022	4.32E-02	++++	0	8.45E-01	4.39E-01
100015792	sphingomyelin.d18.1.25.0.d19.0.24.1.d20.1.23.0.d	6208	-2.024	4.29E-02	-?--	66.2	5.17E-02	4.39E-01
100006367	3.hydroxyhexanoate	10847	-2.015	4.39E-02	+---	34.4	2.06E-01	4.42E-01
1025	pipecolate	10847	1.99	4.66E-02	+++-	52.2	9.87E-02	4.43E-01
100001314	gamma.glutamylthreonine	10847	-1.933	5.32E-02	----	0	9.83E-01	4.43E-01
100004111	4.methylcatecholsulfate	10847	-1.981	4.76E-02	+---	0	8.17E-01	4.43E-01
100000611	1.palmityl.GPC.O.16.0.	9802	-1.999	4.57E-02	?--?	0	9.04E-01	4.43E-01
100010901	gamma.glutamyl.alpha.lysine	6208	-1.959	5.01E-02	+?--	5.1	3.49E-01	4.43E-01
100008977	1.stearoyl.2.arachidonoyl.GPE.18.0.20.4.	10847	1.944	5.19E-02	-+++	0	3.93E-01	4.43E-01
100001445	1.palmitoyl.GPA.16.0.	10248	-1.961	4.99E-02	---?	0	6.65E-01	4.43E-01
878	fructose	10847	1.957	5.04E-02	++++	49.4	1.15E-01	4.43E-01
100005850	3.methylglutarylcarnitine.2.	10847	1.976	4.82E-02	++++	0	9.44E-01	4.43E-01
100001006	N.acetylglycine	10847	1.996	4.60E-02	-+++	0	5.09E-01	4.43E-01
100009066	1.palmitoyl.2.oleoyl.GPI.16.0.18.1.	6208	1.959	5.02E-02	+?+-	0	6.86E-01	4.43E-01
466	phytanate	10847	-1.932	5.34E-02	+---	54.5	8.61E-02	4.43E-01
100008934	2.hydroxylaurate	5609	1.939	5.26E-02	+?+?	78.3	3.20E-02	4.43E-01
100009264	glycochenodeoxycholateglucuronide.1.	10847	1.976	4.81E-02	++++	0	8.87E-01	4.43E-01
100015835	cerotoylcarnitine.C26.	6208	-1.935	5.30E-02	-?--	61.5	7.43E-02	4.43E-01
533	urea	10847	-1.968	4.90E-02	+---	0	8.48E-01	4.43E-01
100001429	1.margaroylglycerol.17.0.	1045	-1.958	5.02E-02	-??-	28.6	2.37E-01	4.43E-01
100001145	3.hydroxysebacate	5684	-1.915	5.55E-02	+-?-	0	4.48E-01	4.56E-01
100002094	gamma.CEHC	10847	-1.913	5.58E-02	---+	6.2	3.62E-01	4.56E-01
100001271	1.stearoyl.GPC.18.0.	10847	-1.907	5.65E-02	----	10	3.43E-01	4.59E-01
100001658	taurolithocholate3.sulfate	10847	-1.89	5.88E-02	----	0	3.93E-01	4.59E-01
100003271	beta.citrylglutamate	6208	-1.896	5.80E-02	+?--	37.2	2.04E-01	4.59E-01
100000773	3.hydroxyoctanoate	10847	-1.887	5.92E-02	+---	0	4.32E-01	4.59E-01
100015760	linoleoylcholine	6208	-1.893	5.84E-02	-?--	75.2	1.77E-02	4.59E-01
100000987	2.linoleoylglycerol.18.2.	10847	-1.887	5.92E-02	----	0	4.79E-01	4.59E-01
100003001	1..1.enyl.stearoyl..GPE.P.18.0.	10847	-1.889	5.89E-02	----	2.2	3.81E-01	4.59E-01
100001882	glycosyl.N.stearoyl.sphingosine.d18.1.18.0.	10847	-1.875	6.08E-02	+---	76.5	5.20E-03	4.65E-01
397	leucine	10847	-1.857	6.34E-02	---+	42.4	1.57E-01	4.68E-01
100000282	N.acetylglutamate	10847	1.856	6.35E-02	++++	0	4.96E-01	4.68E-01
100001554	2.arachidonoylglycerophosphocholine	9802	1.861	6.27E-02	?++?	23.9	2.52E-01	4.68E-01
100004284	dimethylsulfone	10847	-1.858	6.31E-02	----	0	7.78E-01	4.68E-01
1504	oleamide	10401	1.849	6.44E-02	?-++	54.5	1.11E-01	4.68E-01
100001083	indolepropionate	10847	-1.851	6.42E-02	----	31.1	2.26E-01	4.68E-01
881	cytosine	6208	-1.843	6.54E-02	-?--	47.4	1.49E-01	4.69E-01
100001212	guanidinosuccinate	1045	-1.844	6.52E-02	-??-	0	4.04E-01	4.69E-01
100001550	homostachydrine	10847	-1.822	6.84E-02	+---	0	5.21E-01	4.78E-01
100001106	1.3.dimethylurate	10847	-1.829	6.74E-02	+---	0	4.64E-01	4.78E-01
100015785	nisinate.24.6n3.	5762	1.823	6.83E-02	??++	0	9.74E-01	4.78E-01
100010935	diacylglycerol.14.0.18.1.16.0.16.1.[2]	1045	1.821	6.87E-02	+??+	0	3.64E-01	4.78E-01
100001034	indoleacetate	10847	-1.819	6.89E-02	+---	29.3	2.36E-01	4.78E-01
100015625	glycosyl.N.behenoyl.sphingadienine.d18.2.22.0.	5762	-1.812	6.99E-02	??--	70	6.78E-02	4.79E-01
55	1.methylnicotinamide	10847	1.782	7.48E-02	-++-	64.4	3.80E-02	4.79E-01
100001843	gamma.glutamylalanine	5684	1.782	7.47E-02	++?+	0	7.42E-01	4.79E-01
100002060	1.docosahexaenoylglycerophosphoethanolamine	9802	1.809	7.05E-02	?++?	0	3.25E-01	4.79E-01
244	beta.alanine	10401	-1.796	7.25E-02	?---	0	8.91E-01	4.79E-01
100001393	isovalerylcarnitine.C5.	10847	-1.787	7.39E-02	-+--	73.4	1.04E-02	4.79E-01
100008952	1.palmitoleoylglycerol.16.1.	10847	1.805	7.11E-02	+-++	0	3.96E-01	4.79E-01
100000956	8.hydroxyoctanoate	5684	-1.799	7.20E-02	--?-	0	7.54E-01	4.79E-01
100002122	3.hydroxyhippurate	10847	-1.788	7.37E-02	+---	0	4.50E-01	4.79E-01
100000706	alpha.hydroxyisocaproate	10847	-1.781	7.50E-02	++--	43.7	1.49E-01	4.79E-01
100002183	S.methylmethionine	5762	-1.791	7.33E-02	??--	72.4	5.71E-02	4.79E-01
444	ornithine	10847	-1.776	7.57E-02	++--	27.3	2.48E-01	4.80E-01
100001287	epiandrosteronesulfate	10847	-1.765	7.76E-02	----	66.4	3.01E-02	4.84E-01
100001254	N.acetyltryptophan	10847	1.765	7.76E-02	+++-	0	6.27E-01	4.84E-01
100015641	N.oleoylserine	6208	-1.77	7.68E-02	+?--	39.9	1.89E-01	4.84E-01
100004208	O.methylcatecholsulfate	10847	-1.756	7.90E-02	+---	0	7.76E-01	4.87E-01
100015840	dihomo.linolenoylcarnitine.20.3n3or6.	6208	-1.751	7.99E-02	-?--	17.1	2.99E-01	4.89E-01
93	alpha.ketoglutarate	10847	1.738	8.22E-02	++-+	51.1	1.05E-01	5.00E-01
1162	N.acetylneuraminate	10847	1.731	8.34E-02	++++	0	5.27E-01	5.05E-01
1537	1.palmitoyl.2.linoleoyl.GPC.16.0.18.2.	10847	1.705	8.82E-02	+++-	59.6	5.96E-02	5.10E-01
100000711	4.acetylphenolsulfate	10401	-1.722	8.51E-02	?-+-	75.6	1.67E-02	5.10E-01
100001251	decanoylcarnitine.C10.	10847	-1.715	8.64E-02	+---	38.8	1.79E-01	5.10E-01
100010959	diacylglycerol.12.0.18.1.14.0.16.1.16.0.14.1.[2]	1045	1.705	8.82E-02	+??+	51.5	1.51E-01	5.10E-01
501	salicylate	10847	-1.71	8.73E-02	----	55.1	8.30E-02	5.10E-01

800	cysteine	10847	-1.707	8.78E-02	----	0	8.56E-01	5.10E-01
100001597	tiglylcarnitine.C5.1.DC.	10847	-1.71	8.72E-02	----	75.1	7.29E-03	5.10E-01
100006098	3.hydroxypyridinesulfate	10847	-1.679	9.32E-02	+---	9.7	3.44E-01	5.14E-01
100001416	orotidine	10847	1.678	9.33E-02	++++	0	7.92E-01	5.14E-01
100009336	eicosapentaenoylcholine	10847	-1.68	9.30E-02	----	59.4	6.03E-02	5.14E-01
100010926	linoleoyl.linolenoyl.glycerol.18.2.18.3.[1]	1045	1.679	9.32E-02	+??+	0	6.09E-01	5.14E-01
100002185	indole.3.carboxylicacid	5684	-1.688	9.13E-02	--?-	52.2	1.23E-01	5.14E-01
100006314	sphingomyelin.d17.1.16.0.d18.1.15.0.d16.1.17.0.	10847	-1.686	9.18E-02	-+--	74.2	8.68E-03	5.14E-01
100009345	1.palmitoleoyl.2.linolenoyl.GPC.16.1.18.3.	10847	1.673	9.44E-02	++++	24.4	2.65E-01	5.15E-01
932	caprylate.8.0.	10847	-1.65	9.89E-02	----	10.5	3.41E-01	5.30E-01
100008980	1.stearoyl.2.linoleoyl.GPC.18.0.18.2.	10847	-1.651	9.87E-02	----	0	5.59E-01	5.30E-01
100002259	cis.4.decenoylcarnitine.C10.1.	10847	-1.649	9.92E-02	----	0	9.06E-01	5.30E-01
100003434	imidazolepropionate	10847	1.644	1.00E-01	++++	4.2	3.72E-01	5.33E-01
100002875	1..1.enyl.palmitoyl..GPC.P.16.0.	10847	-1.64	1.01E-01	-+--	57.7	6.88E-02	5.34E-01
100005717	1.palmitoyl.GPG.16.0.	10847	1.633	1.03E-01	++++	0	6.54E-01	5.35E-01
100001207	4.imidazoleacetate	5085	1.631	1.03E-01	-+??	0	5.34E-01	5.35E-01
100009147	1.stearyl.GPC.O.18.0.	9802	-1.626	1.04E-01	?--?	0	4.22E-01	5.39E-01
100015837	arachidonoylcarnitine.C20.4.	6208	-1.623	1.05E-01	-?--	16.8	3.01E-01	5.39E-01
100015838	eicosenoylcarnitine.C20.1.	6208	-1.617	1.06E-01	+?--	60.2	8.13E-02	5.43E-01
935	sucrose	10847	1.608	1.08E-01	++-+	37.8	1.85E-01	5.47E-01
100015846	nervonoylcarnitine.C24.1.	6208	1.605	1.09E-01	+?+-	75	1.84E-02	5.49E-01
100008955	tricosanoylsphingomyelin.d18.1.23.0.	6208	-1.599	1.10E-01	-?--	64.9	5.81E-02	5.53E-01
100000672	1.myristoyl.2.palmitoyl.GPC.14.0.16.0.	10401	1.591	1.12E-01	?++-	0	5.21E-01	5.57E-01
100002070	2.hydroxyglutarate	6208	1.587	1.13E-01	-?++	33.6	2.22E-01	5.57E-01
100001662	deoxycarnitine	10847	-1.581	1.14E-01	----	0	9.31E-01	5.59E-01
100009139	1.myristoyl.2.arachidonoyl.GPC.14.0.20.4.	10401	1.571	1.16E-01	?++-	0	5.18E-01	5.64E-01
1528	1.palmitoyl.2.linoleoyl.GPI.16.0.18.2.	6208	1.559	1.19E-01	+?+-	0	6.43E-01	5.64E-01
100010869	2.3.dihydroxy.2.methylbutyrate	6208	-1.557	1.20E-01	-?-+	57.3	9.63E-02	5.64E-01
100002227	4.cholesten.3.one	10248	-1.562	1.18E-01	+--?	0	6.86E-01	5.64E-01
100010950	stearoyl.arachidonoyl.glycerol.18.0.20.4.[2]	1045	-1.563	1.18E-01	-??-	38.9	2.01E-01	5.64E-01
100001040	1.linoleoylglycerol.18.2.	10847	-1.566	1.17E-01	----	0	9.12E-01	5.64E-01
100001315	p.cresolsulfate	10847	-1.554	1.20E-01	+---	6	3.63E-01	5.64E-01
100003000	1..1.enyl.palmitoyl..GPE.P.16.0.	10847	-1.551	1.21E-01	----	29.8	2.34E-01	5.65E-01
1125	5.6.dihydrouracil	1045	1.531	1.26E-01	+??+	0	7.42E-01	5.66E-01
100000870	saccharin	10401	1.532	1.26E-01	?+++	0	7.73E-01	5.66E-01
100001561	2.palmitoleoyl.GPC.16.1.	5684	1.533	1.25E-01	++?+	0	7.74E-01	5.66E-01
1124	citrate	10847	-1.532	1.26E-01	+---	0	8.78E-01	5.66E-01
100001064	glycolithocholate	10847	-1.529	1.26E-01	---+	0	9.11E-01	5.66E-01
111	3.hydroxyisobutyrate	10847	-1.529	1.26E-01	+---	2.2	3.81E-01	5.66E-01
240	3..4.hydroxyphenyl.lactate	10847	-1.539	1.24E-01	++--	62.9	4.44E-02	5.66E-01
100001992	androstenediol.3beta.17beta.disulfate.1.	10847	1.539	1.24E-01	++++	0	7.77E-01	5.66E-01
100002990	oleoyl.linoleoyl.glycerol.18.1.18.2.[2]	10847	1.519	1.29E-01	++++	0	9.94E-01	5.67E-01
100001073	androsteronesulfate	10847	-1.518	1.29E-01	----	50.3	1.10E-01	5.67E-01
100006298	lignoceroylsphingomyelin.d18.1.24.0.	6208	-1.521	1.28E-01	-?--	62.9	6.74E-02	5.67E-01
100006627	suberoylcarnitine.C8.DC.	1045	1.511	1.31E-01	+??+	0	5.29E-01	5.71E-01
100006438	citraconate.glutaconate	10847	-1.499	1.34E-01	+---	0	3.96E-01	5.79E-01
1268	gamma.glutamylleucine	10847	-1.497	1.34E-01	+---	0	6.57E-01	5.79E-01
100001208	1.methylimidazoleacetate	10847	1.495	1.35E-01	--++	23.3	2.71E-01	5.79E-01
1102	gamma.glutamyltyrosine	10847	-1.484	1.38E-01	----	0	7.18E-01	5.84E-01
100015832	behenoylcarnitine.C22.	6208	-1.486	1.37E-01	-?--	73.5	2.30E-02	5.84E-01
100006294	behenoylsphingomyelin.d18.1.22.0.	6208	-1.475	1.40E-01	-?--	51.2	1.29E-01	5.90E-01
100001613	tetradecanedioate	10847	-1.462	1.44E-01	+---	3.7	3.74E-01	5.92E-01
888	caprate.10.0.	10847	-1.463	1.43E-01	+---	0	4.27E-01	5.92E-01
100008989	1.palmitoyl.2.eicosapentaenoyl.GPC.16.0.20.5.	9802	1.464	1.43E-01	?++?	0	4.81E-01	5.92E-01
100008993	1.palmitoyl.2.arachidonoyl.GPI.16.0.20.4.	10847	1.453	1.46E-01	+++-	0	5.83E-01	5.94E-01
100015836	ximenoylcarnitine.C26.1.	6208	-1.458	1.45E-01	-?--	63.6	6.40E-02	5.94E-01
100000774	phenyllactate.PLA.	10847	-1.45	1.47E-01	+---	0	6.44E-01	5.94E-01
100001397	1.3.7.trimethylurate	10847	-1.44	1.50E-01	+---	0	6.99E-01	5.96E-01
100006129	vanillactate	6208	1.435	1.51E-01	+?+-	73.7	2.22E-02	5.96E-01
100000042	3.methylhistidine	10847	-1.434	1.52E-01	----	0	9.86E-01	5.96E-01
358	hypotaurine	10847	1.439	1.50E-01	--+-	17.8	3.02E-01	5.96E-01
100001320	erythronate	10847	1.425	1.54E-01	++++	33.6	2.10E-01	5.98E-01
100001417	phenylacetylglutamine	10847	-1.421	1.55E-01	+---	0	6.33E-01	5.98E-01
100001022	threonate	10847	-1.424	1.55E-01	--+-	68.8	2.21E-02	5.98E-01
100000616	1.stearoyl.2.arachidonoyl.GPI.18.0.20.4.	10847	1.414	1.57E-01	+++-	0	8.86E-01	6.03E-01
100010930	palmitoleoyl.linoleoyl.glycerol.16.1.18.2.[1]	6208	1.41	1.59E-01	+?++	0	8.21E-01	6.04E-01
100003696	succinimide	10401	-1.402	1.61E-01	?+--	27.2	2.53E-01	6.08E-01
376	isoleucine	10847	-1.387	1.65E-01	+--+	18.3	2.99E-01	6.11E-01
100000781	hexanoylcarnitine.C6.	10847	-1.394	1.63E-01	-+--	0	5.65E-01	6.11E-01
100008918	1..1.enyl.stearoyl..2.arachidonoyl.GPC.P.18.0.20.4	9802	-1.386	1.66E-01	?--?	0	3.51E-01	6.11E-01
100001501	oleoylcarnitine.C18.1.	10847	-1.393	1.64E-01	+---	39.4	1.76E-01	6.11E-01
407	lysine	10847	-1.391	1.64E-01	-+--	38.9	1.79E-01	6.11E-01
100001956	N.methylproline	10847	-1.384	1.66E-01	----	12.2	3.32E-01	6.11E-01
100015882	glycosylceramide.d18.1.20.0.d16.1.22.0.	6208	-1.384	1.66E-01	+?--	28.2	2.49E-01	6.11E-01

100000882	3.hydroxymyristate	1045	-1.393	1.64E-01	-??-	0	5.49E-01	6.11E-01
100001167	pro.hydroxy.pro	10847	-1.378	1.68E-01	+--+	0	6.42E-01	6.16E-01
1113	4.acetamidobutanoate	10847	1.366	1.72E-01	+-+-	0	4.11E-01	6.18E-01
100009271	3.hydroxybutyrylcarnitine.2.	10847	-1.373	1.70E-01	+--+	0	4.78E-01	6.18E-01
100002871	1.adrenoyl.GPC.22.4.	9802	1.368	1.71E-01	?++?	0	4.40E-01	6.18E-01
49	putrescine	1045	-1.367	1.72E-01	-??-	0	7.62E-01	6.18E-01
112	3.hydroxy.3.methylglutarate	10847	1.361	1.73E-01	++-+	75.1	7.16E-03	6.20E-01
100009403	1.eicosapentaenoylglycerol.20.5.	5684	1.355	1.75E-01	++?-	0	5.83E-01	6.25E-01
100006726	linoleoylethanolamide	10847	-1.353	1.76E-01	---+	0	7.44E-01	6.25E-01
100004110	3.methylcatecholsulfate.2.	10847	-1.347	1.78E-01	+-+-	0	4.02E-01	6.25E-01
1221	creatine	10847	-1.345	1.79E-01	+--+	0	5.48E-01	6.25E-01
100001257	N.acetylasparagine	10847	1.336	1.82E-01	-+++	0	8.51E-01	6.28E-01
100003432	dihydroferulicacid	10401	-1.34	1.80E-01	?---	43.6	1.70E-01	6.28E-01
100001033	beta.sitosterol	5762	-1.335	1.82E-01	??--	76.4	3.97E-02	6.28E-01
100001466	3.methylcytidine	6208	1.328	1.84E-01	+?++	0	8.85E-01	6.35E-01
100004112	3.methylcatecholsulfate.1.	10847	-1.301	1.93E-01	+---	0	8.59E-01	6.45E-01
100000657	1.2.dipalmitoyl.GPC.16.0.16.0.	10847	1.308	1.91E-01	-++-	8	3.53E-01	6.45E-01
100006051	myristoleoylcarnitine.C14.1.	10847	-1.289	1.98E-01	+---	27.3	2.48E-01	6.45E-01
100002014	5alpha.pregnan.3beta.20alpha.diolmonosulfate.2.	10847	-1.293	1.96E-01	+---	0	4.01E-01	6.45E-01
100009142	1.stearoyl.2.docosapentaenoyl.GPC.18.0.22.5n6.	9802	1.302	1.93E-01	?++?	0	9.74E-01	6.45E-01
1084	N.acetylvaline	10847	1.299	1.94E-01	++++	0	9.90E-01	6.45E-01
818	malonate	9802	-1.299	1.94E-01	?--?	40.6	1.94E-01	6.45E-01
100009335	dihomo.linolenoyl.choline	10847	-1.287	1.98E-01	----	50	1.12E-01	6.45E-01
100001247	octanoylcarnitine.C8.	10847	-1.289	1.97E-01	+---	5.7	3.64E-01	6.45E-01
1239	2.hydroxystearate	10847	-1.296	1.95E-01	+---	0	7.39E-01	6.45E-01
100005384	O.sulfo.L.tyrosine	10847	-1.308	1.91E-01	+---	0	4.29E-01	6.45E-01
100001178	3.carboxy.4.methyl.5.propyl.2.furanpropanoate.CI	10847	-1.315	1.89E-01	+---	0	8.07E-01	6.45E-01
100009069	1..1.enyl.palmitoyl..2.linoleoyl.GPE.P.16.0.18.2.	10847	-1.312	1.89E-01	---+	0	7.32E-01	6.45E-01
100008956	sphingomyelin.d18.2.23.0.d18.1.23.1.d17.1.24.1.	6208	-1.311	1.90E-01	-?--	65.2	5.66E-02	6.45E-01
100001405	1.methylxanthine	10847	-1.247	2.12E-01	++--	46.3	1.33E-01	6.47E-01
572	glucose	10847	1.282	2.00E-01	++++	75.9	5.93E-03	6.47E-01
100006092	tyramineO.sulfate	10847	-1.256	2.09E-01	----	0	7.10E-01	6.47E-01
100009138	1.myristoyl.2.linoleoyl.GPC.14.0.18.2.	9802	1.251	2.11E-01	?++?	6.7	3.01E-01	6.47E-01
100002102	N.acetyl.beta.alanine	10847	1.269	2.05E-01	+-++	31.6	2.22E-01	6.47E-01
100001810	dimethylarginine.SDMA+ADMA.	10847	-1.247	2.13E-01	---+	0	7.51E-01	6.47E-01
100001300	alpha.hydroxyisovalerate	10847	-1.248	2.12E-01	----	0	6.57E-01	6.47E-01
100006651	3.4.methyleneheptanoate	10401	-1.274	2.03E-01	?--+	52.6	1.21E-01	6.47E-01
1128	2.aminobutyrate	10847	-1.266	2.06E-01	-+--	0	4.04E-01	6.47E-01
278	cysteinylglycine	10847	-1.248	2.12E-01	---+	36	1.96E-01	6.47E-01
100015620	lactosyl.N.nervonoyl.sphingosine.d18.1.24.1.	6208	-1.25	2.11E-01	+?--	0	4.45E-01	6.47E-01
1442	beta.hydroxyisovalerate	10847	-1.249	2.12E-01	----	0	8.41E-01	6.47E-01
100003674	prolylglycine	10847	-1.269	2.05E-01	+-+-	48.8	1.19E-01	6.47E-01
100001395	1.linoleoyl.GPC.18.2.	10847	-1.284	1.99E-01	----	49.9	1.12E-01	6.47E-01
100009025	sphingomyelin.d18.1.21.0.d17.1.22.0.d16.1.23.0.	6208	-1.267	2.05E-01	+?--	72.5	2.65E-02	6.47E-01
2054	ethylmalonate	10847	1.243	2.14E-01	-+++	0	7.38E-01	6.47E-01
100004561	N.formylanthranilicacid	6208	-1.236	2.16E-01	-?--	0	7.69E-01	6.51E-01
100003151	linoleoylcarnitine.C18.2.	10847	-1.229	2.19E-01	----	0	5.76E-01	6.58E-01
1235	gamma.glutamylhistidine	10847	1.224	2.21E-01	+++-	0	4.23E-01	6.61E-01
1224	cys.gly.oxidized	10847	-1.213	2.25E-01	--+-	51.9	1.01E-01	6.61E-01
100001571	1.arachidonoyl.GPE.20.4n6.	10847	1.218	2.23E-01	++++	0	9.15E-01	6.61E-01
823	pyruvate	10847	1.218	2.23E-01	++-+	62.9	4.44E-02	6.61E-01
100008929	2.methylcitrate.homocitrate	1045	-1.214	2.25E-01	+??-	76.2	4.05E-02	6.61E-01
100015727	ceramide.d16.1.24.1.d18.1.22.1.	1045	1.2	2.30E-01	+??+	0	4.43E-01	6.75E-01
100009338	5.bromotryptophan	10248	-1.195	2.32E-01	---?	0	9.65E-01	6.75E-01
338	gluconate	10847	-1.195	2.32E-01	+---	0	7.73E-01	6.75E-01
100001392	laurylcarnitine.C12.	10847	-1.193	2.33E-01	+---	58.7	6.39E-02	6.75E-01
100001509	2.methylbutyrylcarnitine.C5.	6208	-1.189	2.35E-01	-?--	0	6.86E-01	6.79E-01
100000285	N.alpha.acetylornithine	1045	-1.179	2.38E-01	-??-	0	5.10E-01	6.80E-01
100002027	androstenediol.3alpha.17alpha.monosulfate.3.	10847	-1.179	2.39E-01	----	0	3.96E-01	6.80E-01
100001102	dodecanedioate	10847	-1.182	2.37E-01	+---	60.4	5.58E-02	6.80E-01
100015745	glycosylceramide.d18.2.24.1.d18.1.24.2.	6208	-1.181	2.38E-01	+?--	0	4.83E-01	6.80E-01
922	N.stearoyl.sphinganine.d18.0.18.0.	1045	1.182	2.37E-01	+??+	0	5.28E-01	6.80E-01
100006282	umbelliferonesulfate	10847	-1.171	2.42E-01	--+-	41.1	1.65E-01	6.81E-01
100000295	tartarate	1045	-1.175	2.40E-01	-??-	0	4.84E-01	6.81E-01
100001437	cysteine.glutathionedisulfide	10847	-1.173	2.41E-01	-+--	69	2.15E-02	6.81E-01
100009009	1..1.enyl.palmitoyl..2.linoleoyl.GPC.P.16.0.18.2.	10847	-1.173	2.41E-01	-+--	44.3	1.46E-01	6.81E-01
100001296	stachydrine	10847	-1.172	2.41E-01	----	0	5.44E-01	6.81E-01
100001987	5alpha.androstan.3beta.17beta.dioldisulfate	10847	-1.159	2.46E-01	--+-	45.2	1.40E-01	6.83E-01
100002989	oleoyl.linoleoyl.glycerol.18.1.18.2.[1]	10847	1.155	2.48E-01	++++	0	9.79E-01	6.83E-01
100010941	linoleoyl.linoleoyl.glycerol.18.2.18.2.[1]	6208	1.157	2.47E-01	-?++	0	8.49E-01	6.83E-01
100004182	3b.hydroxy.5.cholenoicacid	1045	1.159	2.46E-01	+??+	0	8.09E-01	6.83E-01
100004555	benzoylcarnitine	1045	-1.162	2.45E-01	+??-	74.8	4.63E-02	6.83E-01
100000998	citramalate	9802	1.164	2.44E-01	?++?	0	4.24E-01	6.83E-01
798	adenosine	1045	1.157	2.47E-01	+??+	0	8.48E-01	6.83E-01

100015967	carotenediol.2.	6208	-1.161	2.46E-01	+?--	69.9	3.61E-02	6.83E-01
100001541	2.hydroxy.3.methylvalerate	10847	-1.152	2.49E-01	----	0	8.47E-01	6.85E-01
100002876	1..1.enyl.oleoyl..GPC.P.18.1.	9802	-1.139	2.55E-01	?--?	0	5.77E-01	6.98E-01
100001129	O.acetylhomoserine	5238	1.134	2.57E-01	?+?-	0	5.88E-01	6.99E-01
100001264	1.margaroylglycerophosphocholine.17.0.	9802	-1.133	2.57E-01	?--?	0	5.03E-01	6.99E-01
100006260	6.hydroxyindolesulfate	10847	-1.128	2.59E-01	+---	6.6	3.60E-01	7.00E-01
1242	N1.methyladenosine	10847	1.123	2.61E-01	-+++	0	7.46E-01	7.01E-01
1629	taurochenodeoxycholate	10847	-1.114	2.65E-01	----	0	9.73E-01	7.06E-01
461	phosphate	10847	-1.113	2.66E-01	----	0	8.35E-01	7.06E-01
100005834	9.hydroxystearate	5238	1.118	2.64E-01	?+?+	76.7	3.83E-02	7.06E-01
1021	5.oxoproline	10847	-1.11	2.67E-01	++--	0	4.17E-01	7.06E-01
100015845	docosahexaenoylcarnitine.C22.6.	5762	-1.112	2.66E-01	??--	77	3.70E-02	7.06E-01
100002028	androstenediol.3beta.17beta.monosulfate.1.	10847	1.114	2.65E-01	+++-	0	8.58E-01	7.06E-01
100001383	1.myristoylglycerophosphocholine.14.0.	9802	1.104	2.70E-01	?++?	0	6.93E-01	7.07E-01
100000708	isovalerate	10401	-1.102	2.71E-01	?---	0	9.13E-01	7.07E-01
136	cholate	10847	-1.103	2.70E-01	+-+-	72.9	1.14E-02	7.07E-01
811	alanine	10847	1.097	2.73E-01	-+-+	61.4	5.11E-02	7.11E-01
100016038	N.1.+N.8...acetylspermidine	5762	-1.092	2.75E-01	??--	0	7.14E-01	7.14E-01
806	dimethylglycine	10847	-1.089	2.76E-01	+---	37.8	1.85E-01	7.14E-01
100006435	N.acetylglucosamine.N.acetylgalactosamine	10847	1.089	2.76E-01	++++	52.8	9.54E-02	7.14E-01
100001086	N..2.furoyl.glycine	10847	-1.081	2.80E-01	+-+-	49.6	1.14E-01	7.18E-01
100001869	1.stearoyl.2.arachidonoyl.GPC.18.0.20.4.	10847	1.083	2.79E-01	-+++	47.6	1.26E-01	7.18E-01
100009054	palmitoleoyl.oleoyl.glycerol.16.1.18.1.[2]	1045	1.083	2.79E-01	+??+	0	4.67E-01	7.18E-01
100001851	N.acetylserine	10847	1.075	2.83E-01	+++-	0	9.35E-01	7.20E-01
100008920	sphingomyelin.d18.1.17.0.d17.1.18.0.d19.1.16.0.	10847	-1.073	2.84E-01	++--	60.7	5.43E-02	7.20E-01
339	glutarate.pentanedioate.	10847	-1.073	2.83E-01	+--+	0	6.92E-01	7.20E-01
100015788	sphingomyelin.d18.2.18.1.	6208	-1.067	2.86E-01	-?--	36.7	2.06E-01	7.23E-01
100001263	1.palmitoyl.GPC.16.0.	10847	1.04	2.98E-01	-++-	73	1.11E-02	7.24E-01
100001468	N1.Methyl.2.pyridone.5.carboxamide	10847	1.044	2.97E-01	+++-	66.8	2.89E-02	7.24E-01
100009130	1.oleoyl.2.docosahexaenoyl.GPC.18.1.22.6.	10401	1.039	2.99E-01	?++-	13.2	3.16E-01	7.24E-01
100004541	acisoga	10847	-1.039	2.99E-01	---+	9.2	3.47E-01	7.24E-01
100002106	sphingomyelin.d18.1.18.1.d18.2.18.0.	10847	1.046	2.96E-01	++--	0	4.15E-01	7.24E-01
100010949	stearoyl.arachidonoyl.glycerol.18.0.20.4.[1]	1045	-1.047	2.95E-01	-??-	0	5.14E-01	7.24E-01
100001270	myristoylcarnitine.C14.	10847	-1.059	2.90E-01	+---	69.4	2.02E-02	7.24E-01
342	glycocholate	10847	-1.043	2.97E-01	--++	15.4	3.15E-01	7.24E-01
100015752	glycosyl.N..2.hydroxynervonoyl..sphingosine.d18.1	5762	-1.043	2.97E-01	??-+	35.5	2.13E-01	7.24E-01
100001399	1.7.dimethylurate	10847	-1.051	2.93E-01	++--	53.9	8.93E-02	7.24E-01
100001054	butyrylcarnitine.C4.	10847	1.061	2.89E-01	-+++	0	5.15E-01	7.24E-01
100002063	1.docosapentaenoylglycerophosphocholine.22.5n3	9802	1.051	2.94E-01	?+-?	35.5	2.13E-01	7.24E-01
100004634	3.methoxytyraminesulfate	1045	1.053	2.93E-01	+??-	71.8	5.97E-02	7.24E-01
100015966	carotenediol.1.	6208	-1.049	2.94E-01	+?--	60.6	7.90E-02	7.24E-01
100002113	cysteinesulfinicacid	10847	1.039	2.99E-01	+-++	75.2	7.14E-03	7.24E-01
100005985	sphingomyelin.d18.2.14.0.d18.1.14.1.	10847	1.03	3.03E-01	+++-	54.5	8.59E-02	7.30E-01
1087	erucate.22.1n9.	10847	-1.028	3.04E-01	+---	0	4.62E-01	7.30E-01
849	caffeine	10847	-1.029	3.04E-01	++--	0	5.11E-01	7.30E-01
100009021	1.palmityl.2.arachidonoyl.GPC.O.16.0.20.4.	9802	-1.025	3.05E-01	?--?	0	7.78E-01	7.32E-01
100000936	3.methyl.2.oxobutyrate	10847	-1.014	3.11E-01	---+	0	9.37E-01	7.42E-01
100001562	2.palmitoyl.GPC.16.0.	10847	1.009	3.13E-01	++--	51.8	1.01E-01	7.42E-01
100009166	phosphatidylcholine.16.0.22.5n3.18.1.20.4.	9802	1.007	3.14E-01	?-+?	8.3	2.96E-01	7.42E-01
100009141	1.stearoyl.2.docosapentaenoyl.GPC.18.0.22.5n3.	9802	1.005	3.15E-01	?+-?	27.2	2.41E-01	7.42E-01
100001007	ribonate	10248	-1.008	3.14E-01	+--?	53.6	1.16E-01	7.42E-01
100001526	malonylcarnitine	6208	0.996	3.19E-01	+?++	0	5.56E-01	7.49E-01
100001566	1.docosahexaenoylglycerophosphocholine.22.6n3	9802	0.985	3.25E-01	?+-?	15.4	2.77E-01	7.57E-01
100001654	1.arachidonoyl.GPI.20.4.	10847	0.987	3.23E-01	++--	40.6	1.68E-01	7.57E-01
100015790	sphingomyelin.d18.2.21.0.d16.2.23.0.	6208	-0.985	3.25E-01	+?--	39.8	1.90E-01	7.57E-01
100009334	palmitoloelycholine	6208	-0.979	3.28E-01	+?--	58	9.22E-02	7.63E-01
926	caproate.6.0.	10401	-0.972	3.31E-01	?+--	0	5.10E-01	7.67E-01
100001126	gamma.glutamylvaline	10847	-0.972	3.31E-01	+--+	8.8	3.49E-01	7.67E-01
100004299	N.acetyl.1.methylhistidine	10847	-0.967	3.34E-01	----	0	6.84E-01	7.69E-01
100002107	palmitoylsphingomyelin.d18.1.16.0.	10847	-0.966	3.34E-01	-+--	49.3	1.16E-01	7.69E-01
100005673	1.docosapentaenoyl.GPC.22.5n6.	9802	0.963	3.36E-01	?++?	0	3.62E-01	7.69E-01
100001620	glycerophosphoethanolamine	10847	-0.96	3.37E-01	+---	14.3	3.21E-01	7.70E-01
100001398	3.7.dimethylurate	10847	-0.949	3.43E-01	-+--	0	6.78E-01	7.78E-01
279	cystine	10847	0.951	3.42E-01	++-+	68.5	2.32E-02	7.78E-01
100003260	carboxyethyl.GABA	6208	0.947	3.44E-01	+?+-	0	7.15E-01	7.78E-01
415	methionine	10847	-0.946	3.44E-01	-+--	41	1.66E-01	7.78E-01
1538	stearoylsphingomyelin.d18.1.18.0.	10847	0.949	3.43E-01	++-+	59	6.24E-02	7.78E-01
100002953	16.hydroxypalmitate	6208	-0.931	3.52E-01	+?--	20.3	2.85E-01	7.84E-01
100005350	1.linolenoyl.GPC.18.3.	10847	-0.932	3.51E-01	+---	20.7	2.86E-01	7.84E-01
100001256	N.acetylphenylalanine	10847	0.934	3.51E-01	+-++	0	4.42E-01	7.84E-01
100008921	1.palmitoyl.2.stearoyl.GPC.16.0.18.0.	10847	-0.933	3.51E-01	--+-	54.3	8.69E-02	7.84E-01
100009055	palmitoyl.linoleoyl.glycerol.16.0.18.2.[2]	10847	0.929	3.53E-01	-+++	65.9	3.20E-02	7.85E-01
1489	palmitoylethanolamide	6208	0.927	3.54E-01	+?+-	0	7.24E-01	7.85E-01
100000406	ribitol	10847	0.923	3.56E-01	++-+	0	3.93E-01	7.88E-01

100001989	glycocholenatesulfate	10847	-0.915	3.60E-01	---+	0	4.82E-01	7.94E-01
100001182	docosadienoate.22.2n6.	5684	0.912	3.62E-01	++?+	0	4.66E-01	7.96E-01
100015731	N.palmitoyl.heptadecasphingosine.d17.1.16.0.	6208	-0.908	3.64E-01	+?--	0	4.40E-01	7.99E-01
100002462	5..galactosylhydroxy..L.lysine	1045	-0.878	3.80E-01	+??-	0	4.06E-01	8.00E-01
100001396	7.methylxanthine	10847	-0.892	3.72E-01	-+--	0	4.69E-01	8.00E-01
100008957	sphingomyelin.d18.2.24.1.d18.1.24.2.	10847	0.882	3.78E-01	-++-	53.2	9.33E-02	8.00E-01
100004083	glycohyocholate	10847	-0.889	3.74E-01	--+-	40.2	1.71E-01	8.00E-01
100001277	10.nonadecenoate.19.1n9.	10847	-0.884	3.77E-01	+---	4.8	3.69E-01	8.00E-01
100009331	oleoylcholine	10847	-0.883	3.77E-01	-+--	56.5	7.54E-02	8.00E-01
893	arachidate.20.0.	10847	-0.901	3.68E-01	+---	0	4.96E-01	8.00E-01
100009332	arachidonoylcholine	10847	-0.881	3.78E-01	-+--	61.3	5.15E-02	8.00E-01
100008916	1.stearoyl.2.docosahexaenoyl.GPC.18.0.22.6.	10401	0.88	3.79E-01	?+-+	0	4.11E-01	8.00E-01
563	glutamine	10847	0.891	3.73E-01	-+-+	46.7	1.31E-01	8.00E-01
100009272	glycosyl.N.palmitoyl.sphingosine.d18.1.16.0.	10847	-0.874	3.82E-01	++--	34.9	2.03E-01	8.00E-01
100006373	1.2.3.benzenetriolsulfate.1.	9802	0.873	3.83E-01	?-+?	24.3	2.50E-01	8.00E-01
100009333	docosahexaenoylcholine	10847	-0.869	3.85E-01	-+--	57.7	6.89E-02	8.00E-01
2053	tricarballylate	9802	0.878	3.80E-01	?++?	0	7.72E-01	8.00E-01
2029	azelate.nonanedioate.	10847	-0.868	3.85E-01	--+-	0	7.75E-01	8.00E-01
100009045	phenylacetylglutamate	1045	-0.887	3.75E-01	-??-	0	8.46E-01	8.00E-01
100009225	1..1.enyl.stearoyl..2.linoleoyl.GPE.P.18.0.18.2.	10847	-0.893	3.72E-01	----	0	9.96E-01	8.00E-01
100010934	diacylglycerol.14.0.18.1.16.0.16.1.[1]	1045	0.893	3.72E-01	+??-	54.3	1.39E-01	8.00E-01
100010917	palmitoyl.oleoyl.glycerol.16.0.18.1.[2]	6208	0.883	3.77E-01	+?++	0	6.18E-01	8.00E-01
100005864	methylglucopyranoside.alpha+beta.	10847	-0.895	3.71E-01	--+-	21.8	2.80E-01	8.00E-01
100005986	sphingomyelin.d18.1.24.1.d18.2.24.0.	6208	-0.89	3.74E-01	-?--	0	8.06E-01	8.00E-01
100001452	isovalerylglycine	10847	-0.864	3.88E-01	--+-	58.4	6.56E-02	8.02E-01
100000437	theophylline	10847	-0.864	3.88E-01	++--	41.2	1.64E-01	8.02E-01
100000447	gentisate	10847	-0.858	3.91E-01	--+-	31.5	2.24E-01	8.08E-01
100008998	gamma.tocopherol.beta.tocopherol	10847	-0.851	3.95E-01	---+	0	6.65E-01	8.10E-01
100001262	gamma.glutamyl.epsilon.lysine	10847	-0.847	3.97E-01	--+-	0	6.17E-01	8.10E-01
100003686	N.palmitoylglycine	10847	0.849	3.96E-01	++-+	42.1	1.59E-01	8.10E-01
1004	xanthine	10847	-0.851	3.95E-01	-+-+	57.6	6.94E-02	8.10E-01
892	nonadecanoate.19.0.	10847	-0.848	3.97E-01	++--	63.4	4.21E-02	8.10E-01
100005383	1.methyl.2.piperidinecarboxylicacid	10401	-0.843	3.99E-01	?---	0	8.09E-01	8.13E-01
235	2.hydroxyphenylacetate	1045	-0.835	4.04E-01	+??-	49.2	1.61E-01	8.15E-01
100001806	o.cresolsulfate	10847	0.835	4.04E-01	-+-+	0	7.49E-01	8.15E-01
100001415	N6.carbamoylthreonyladenosine	10847	-0.836	4.03E-01	+---	0	6.52E-01	8.15E-01
100001755	4.vinylphenolsulfate	10847	0.837	4.03E-01	-++-	60.8	5.37E-02	8.15E-01
100010895	2..O.methylcytidine	6208	-0.826	4.09E-01	-?+-	78.6	9.31E-03	8.18E-01
100001391	stearoylcarnitine.C18.	10847	-0.826	4.09E-01	++--	69.3	2.06E-02	8.18E-01
1023	sarcosine	10847	-0.826	4.09E-01	+-+-	0	5.36E-01	8.18E-01
100008917	1..1.enyl.stearoyl..2.oleoyl.GPC.P.18.0.18.1.	9802	-0.82	4.13E-01	?--?	0	7.66E-01	8.18E-01
100000943	2.oleoylglycerol.18.1.	10248	-0.817	4.14E-01	++-?	0	6.02E-01	8.18E-01
1135	ursodeoxycholate	10847	0.818	4.13E-01	++++	0	5.97E-01	8.18E-01
100000841	oxalate.ethanedioate.	10847	0.805	4.21E-01	-++-	79.4	2.27E-03	8.21E-01
100010922	linoleoyl.arachidonoyl.glycerol.18.2.20.4.[1]	6208	0.81	4.18E-01	-?+-	0	7.97E-01	8.21E-01
100008976	1.stearoyl.2.linoleoyl.GPE.18.0.18.2.	10847	0.803	4.22E-01	+-++	0	7.22E-01	8.21E-01
100003470	pregnanediol.3.glucuronide	10847	-0.803	4.22E-01	+-+-	53.2	9.34E-02	8.21E-01
100004089	2.hydroxydecanoate	10847	-0.804	4.21E-01	--++	2.5	3.80E-01	8.21E-01
100000008	benzoate	10401	0.813	4.16E-01	?++-	65.2	5.67E-02	8.21E-01
100003119	N.oleoyltaurine	1045	0.809	4.18E-01	+??-	8.2	2.97E-01	8.21E-01
480	proline	10847	0.8	4.24E-01	-+-+	56	7.77E-02	8.21E-01
100009233	palmitoylcholine	10847	-0.796	4.26E-01	-+--	67.6	2.61E-02	8.21E-01
100001994	androstenediol.3beta.17beta.disulfate.2.	10847	-0.789	4.30E-01	----	0	9.21E-01	8.25E-01
356	cortisol	10847	0.789	4.30E-01	++++	0	5.91E-01	8.25E-01
100001433	1.arachidonylglycerol.20.4.	10847	-0.789	4.30E-01	-+--	23	2.73E-01	8.25E-01
100001335	eicosenoate.20.1.	10847	-0.779	4.36E-01	++--	0	4.24E-01	8.28E-01
100001026	galactonate	10847	0.774	4.39E-01	++--	0	4.58E-01	8.28E-01
100001446	5.methyluridine.ribothymidine.	10847	-0.783	4.34E-01	+---	0	6.93E-01	8.28E-01
100010937	oleoyl.arachidonoyl.glycerol.18.1.20.4.[2]	6208	0.774	4.39E-01	-?++	0	6.64E-01	8.28E-01
891	margarate.17.0.	10847	-0.785	4.33E-01	++--	24.6	2.64E-01	8.28E-01
100000445	theobromine	10847	-0.766	4.44E-01	-+--	0	4.86E-01	8.32E-01
100001604	hydroquinonesulfate	10847	0.767	4.43E-01	-++-	13.7	3.24E-01	8.32E-01
100002026	androstenediol.3alpha.17alpha.monosulfate.2.	10847	-0.762	4.46E-01	+---	0	9.00E-01	8.32E-01
1110	N.acetylalanine	10847	0.762	4.46E-01	-+++	0	8.99E-01	8.32E-01
100010924	palmitoyl.arachidonoyl.glycerol.16.0.20.4.[1]	6208	0.762	4.46E-01	+?+-	0	4.91E-01	8.32E-01
100009014	1..1.enyl.palmitoyl..2.arachidonoyl.GPC.P.16.0.20.	10847	-0.761	4.47E-01	----	0	5.99E-01	8.32E-01
452	palmitoleate.16.1n7.	10847	0.758	4.48E-01	++++	0	9.61E-01	8.33E-01
100001092	trigonelline.N..methylnicotinate.	10847	-0.757	4.49E-01	+---	65.3	3.44E-02	8.33E-01
100006056	N.formylphenylalanine	5238	0.756	4.49E-01	?+?-	47.6	1.67E-01	8.33E-01
100001988	5alpha.pregnan.3beta.20alpha.dioldisulfate	10847	-0.752	4.52E-01	+---	9.9	3.44E-01	8.34E-01
100001590	isobutyrylglycine	10847	0.751	4.53E-01	-++-	70.5	1.73E-02	8.34E-01
1136	valerate	10401	-0.752	4.52E-01	?+--	0	3.74E-01	8.34E-01
512	taurine	10847	-0.748	4.55E-01	-+--	25.6	2.58E-01	8.37E-01
439	stearate.18.0.	10847	-0.745	4.56E-01	+---	0	4.34E-01	8.38E-01

100001269	campesterol	6208	-0.742	4.58E-01	-?--	32.5	2.28E-01	8.38E-01
100009153	1.stearoyl.2.meadoyl.GPC.18.0.20.3n9.	9802	0.741	4.59E-01	?++?	0	7.76E-01	8.38E-01
100004523	N.delta.acetylornithine	10847	-0.742	4.58E-01	-++-	66.5	2.99E-02	8.38E-01
100006293	sphingomyelin.d18.1.20.2.d18.2.20.1.d16.1.22.2.	6208	0.718	4.73E-01	-?++	0	8.46E-01	8.38E-01
100000665	docosahexaenoate.DHA.22.6n3.	10847	0.703	4.82E-01	++--	32.1	2.20E-01	8.38E-01
266	cholesterol	10847	0.729	4.66E-01	++--	63.1	4.34E-02	8.38E-01
100002927	S.methylcysteinesulfoxide	6208	0.731	4.65E-01	+?+-	40.5	1.86E-01	8.38E-01
100003901	2.stearoyl.GPE.18.0.	10847	0.738	4.60E-01	++--	49.8	1.13E-01	8.38E-01
100000961	homoarginine	10847	0.705	4.81E-01	-++-	0	7.99E-01	8.38E-01
1254	glycerol	10847	0.722	4.70E-01	++++	0	6.44E-01	8.38E-01
100009181	1.stearoyl.2.oleoyl.GPI.18.0.18.1.	6208	0.71	4.78E-01	+?+-	0	8.09E-01	8.38E-01
100002951	eicosanodioate	10847	-0.714	4.76E-01	----	0	7.44E-01	8.38E-01
100001557	2.linoleoylglycerophosphocholine	9802	-0.709	4.78E-01	?--?	0	7.88E-01	8.38E-01
100001635	ectoine	6208	0.734	4.63E-01	+?+-	59.3	8.59E-02	8.38E-01
100001253	N.acetylglutamine	10847	-0.709	4.78E-01	+--+	0	7.43E-01	8.38E-01
361	inosine	10847	-0.73	4.66E-01	+--+	51.8	1.01E-01	8.38E-01
100003240	N.stearoyltaurine	1045	0.731	4.65E-01	+??-	81.5	2.02E-02	8.38E-01
100001051	1.methylhistidine	10847	-0.712	4.76E-01	-+--	0	6.13E-01	8.38E-01
503	serine	10847	-0.704	4.81E-01	-+--	30.3	2.31E-01	8.38E-01
1648	taurocholate	10847	-0.703	4.82E-01	--+-	0	5.77E-01	8.38E-01
437	pelargonate.9.0.	10847	-0.738	4.60E-01	----	0	9.15E-01	8.38E-01
100009015	1..1.enyl.stearoyl..2.docosahexaenoyl.GPC.P.18.0.	9802	-0.711	4.77E-01	?+-?	60.1	1.14E-01	8.38E-01
1082	N.acetylleucine	10847	0.721	4.71E-01	++-+	0	7.45E-01	8.38E-01
100002769	argininate	6208	-0.721	4.71E-01	-?+-	54.8	1.10E-01	8.38E-01
100001993	pregnen.dioldisulfateC21H34O8S2	10847	0.714	4.75E-01	+-+-	0	7.60E-01	8.38E-01
50	spermidine	6208	-0.716	4.74E-01	-?--	0	8.59E-01	8.38E-01
100015831	linolenoylcarnitine.C18.3.	6208	-0.709	4.79E-01	+?--	0	7.07E-01	8.38E-01
100001767	pyrraline	10847	-0.724	4.69E-01	--+-	0	5.28E-01	8.38E-01
799	betaine	10847	-0.7	4.84E-01	---+	10.8	3.39E-01	8.40E-01
100001485	gamma.glutamylisoleucine	10847	-0.696	4.87E-01	+--+	0	5.25E-01	8.41E-01
100001876	sphinganine.1.phosphate	6208	-0.696	4.86E-01	-?--	0	4.07E-01	8.41E-01
100004326	3.acetylphenolsulfate	6208	-0.692	4.89E-01	+?--	12.3	3.20E-01	8.41E-01
100001768	N6.carboxymethyllysine	6208	-0.692	4.89E-01	+?--	36.4	2.07E-01	8.41E-01
100001293	N.acetylhistidine	10847	0.689	4.91E-01	-++-	0	8.57E-01	8.41E-01
100006082	4.hydroxychlorothalonil	10847	-0.69	4.90E-01	-+--	57.2	7.15E-02	8.41E-01
477	pristanate	6208	-0.69	4.90E-01	+?--	69.2	3.90E-02	8.41E-01
100001614	hexadecanedioate	10847	-0.674	5.00E-01	+--+	66.3	3.06E-02	8.49E-01
1114	3.aminoisobutyrate	10847	0.674	5.00E-01	-++-	0	6.78E-01	8.49E-01
100001757	thymolsulfate	10847	0.676	4.99E-01	-++-	17	3.06E-01	8.49E-01
100004542	2.aminoheptanoate	10847	-0.67	5.03E-01	--++	53.8	8.98E-02	8.52E-01
100001272	1.oleoyl.GPC.18.1.	10847	-0.666	5.05E-01	+---	37.2	1.89E-01	8.52E-01
100006360	dopamine4.sulfate	1045	0.667	5.05E-01	+??-	76.2	4.02E-02	8.52E-01
100001756	4.ethylphenylsulfate	10847	-0.641	5.21E-01	-++-	66.7	2.91E-02	8.53E-01
100008994	1.stearoyl.2.linoleoyl.GPI.18.0.18.2.	10847	0.624	5.33E-01	-++-	0	4.96E-01	8.53E-01
880	adenine	10847	0.64	5.22E-01	-+++	0	4.42E-01	8.53E-01
1002	allantoin	10847	0.647	5.18E-01	++++	0	9.62E-01	8.53E-01
100001778	1.linoleoyl.GPI.18.2.	10847	0.627	5.31E-01	+++-	0	4.01E-01	8.53E-01
231	arginine	10847	-0.625	5.32E-01	-+--	0	9.27E-01	8.53E-01
100005403	etiocholanoloneglucuronide	10847	-0.641	5.22E-01	----	0	8.25E-01	8.53E-01
100001276	N.acetylisoleucine	5238	-0.627	5.30E-01	?-?+	0	5.38E-01	8.53E-01
1001	trans.4.hydroxyproline	10847	-0.638	5.24E-01	--++	0	5.71E-01	8.53E-01
100001655	1.palmitoyl.GPI.16.0.	10847	0.656	5.12E-01	+++-	56.9	7.31E-02	8.53E-01
100009052	palmitoyl.linoleoyl.glycerol.16.0.18.2.[1]	10847	0.644	5.20E-01	++-+	0	7.97E-01	8.53E-01
100001580	docosapentaenoate.n6DPA.22.5n6.	10401	0.653	5.14E-01	?-+-	0	4.63E-01	8.53E-01
100006292	sphingomyelin.d18.1.20.1.d18.2.20.0.	10847	0.641	5.22E-01	-+--	0	4.21E-01	8.53E-01
100009037	1.margaroyl.2.linoleoyl.GPC.17.0.18.2.	9802	-0.636	5.25E-01	?+-?	0	4.93E-01	8.53E-01
100008903	1.2.dilinoleoyl.GPC.18.2.18.2.	10248	-0.651	5.15E-01	--+?	0	7.18E-01	8.53E-01
100015640	N.palmitoylserine	5762	-0.65	5.16E-01	??--	0	7.63E-01	8.53E-01
565	tryptophan	10847	-0.634	5.26E-01	-+--	12.4	3.31E-01	8.53E-01
100001527	hexanoylglycine	5684	-0.631	5.28E-01	-+?-	0	3.84E-01	8.53E-01
100003397	trimethylamineN.oxide	10847	-0.637	5.24E-01	-+--	0	9.38E-01	8.53E-01
100001856	1.stearoyl.2.oleoyl.GPE.18.0.18.1.	10847	0.65	5.16E-01	+-++	0	6.03E-01	8.53E-01
100010919	oleoyl.oleoyl.glycerol.18.1.18.1.[2]	6208	0.661	5.09E-01	+?++	0	9.64E-01	8.53E-01
100000007	carnitine	10847	0.625	5.32E-01	-+-+	0	5.55E-01	8.53E-01
100000269	glycerophosphorylcholine.GPC.	10847	-0.663	5.08E-01	--+-	63.8	4.04E-02	8.53E-01
100000580	1.5.anhydroglucitol.1.5.AG.	10847	0.626	5.32E-01	-+++	0	8.79E-01	8.53E-01
1628	glycochenodeoxycholate	10847	-0.621	5.35E-01	--++	0	8.23E-01	8.54E-01
100000802	acetylcarnitine.C2.	10847	-0.618	5.37E-01	++-+	0	4.98E-01	8.55E-01
100009027	sphingomyelin.d18.0.18.0.d19.0.17.0.	6208	0.617	5.38E-01	+?++	0	4.89E-01	8.55E-01
100002873	1.lignoceroyl.GPC.24.0.	5684	-0.618	5.37E-01	+-?-	0	9.32E-01	8.55E-01
100002029	androstenediol.3beta.17beta.monosulfate.2.	10847	-0.614	5.39E-01	-+--	0	8.34E-01	8.56E-01
100001150	propionylglycine	10847	0.61	5.42E-01	-++-	44	1.48E-01	8.56E-01
100001502	gamma.glutamyl.2.aminobutyrate	10401	-0.61	5.42E-01	?+--	50.9	1.31E-01	8.56E-01
100002009	5alpha.pregnan.3beta.20beta.diolmonosulfate.1.	10847	-0.612	5.40E-01	--+-	5.3	3.66E-01	8.56E-01

1026	phosphoethanolamine	10401	0.605	5.45E-01	?++-	73.1	2.41E-02	8.59E-01
100004046	N.acetylcarnosine	10847	-0.605	5.45E-01	++--	3.2	3.77E-01	8.59E-01
504	serotonin	10847	0.598	5.50E-01	-+++	70.8	1.63E-02	8.62E-01
100001652	2.palmitoylglycerophosphoethanolamine	9802	0.588	5.57E-01	?++?	0	9.13E-01	8.65E-01
100001279	hyocholate	5684	-0.59	5.55E-01	+-?-	0	4.98E-01	8.65E-01
1134	urate	10847	-0.59	5.55E-01	---+	0	8.94E-01	8.65E-01
100001624	3..3.hydroxyphenyl.propionate	10847	-0.59	5.55E-01	+-+-	24.4	2.65E-01	8.65E-01
100000467	3.indoxylsulfate	10847	-0.591	5.55E-01	+-+-	1.5	3.85E-01	8.65E-01
182	quinolinate	10847	-0.588	5.57E-01	-+--	0	4.08E-01	8.65E-01
100000299	xanthosine	1045	0.587	5.57E-01	+??-	32.6	2.23E-01	8.65E-01
100015789	sphingomyelin.d18.2.24.2.	6208	0.585	5.59E-01	-?++	0	5.16E-01	8.65E-01
100010896	2..O.methyluridine	1045	-0.581	5.62E-01	+??-	76.8	3.78E-02	8.68E-01
100001621	glycerophosphoinositol	1045	0.571	5.68E-01	+??-	80.8	2.25E-02	8.69E-01
100001481	1.docosahexaenoylglycerol.22.6.	10847	0.562	5.74E-01	++-+	0	4.83E-01	8.69E-01
100002749	S.methylcysteine	10847	0.568	5.70E-01	+++-	0	6.80E-01	8.69E-01
100001193	adrenate.22.4n6.	10847	-0.563	5.73E-01	++-+	0	5.66E-01	8.69E-01
100002417	2.3.dihydroxyisovalerate	10847	0.562	5.74E-01	+++-	0	8.33E-01	8.69E-01
100001413	N4.acetylcytidine	5609	0.565	5.72E-01	-?+?	0	7.04E-01	8.69E-01
273	cortisone	10847	0.564	5.73E-01	++++	0	9.34E-01	8.69E-01
100001334	N.acetylproline	5238	0.569	5.69E-01	?+?+	0	5.35E-01	8.69E-01
100008953	2.palmitoleoylglycerol.16.1.	5085	0.577	5.64E-01	++??	0	7.66E-01	8.69E-01
62	12.13.DiHOME	10401	-0.568	5.70E-01	?-++	54	1.14E-01	8.69E-01
100015968	carotenediol.3.	6208	-0.577	5.64E-01	+?+-	55.8	1.04E-01	8.69E-01
100000584	2.arachidonoylglycerol.20.4.	1045	-0.566	5.71E-01	-??+	0	4.76E-01	8.69E-01
100000846	erythritol	10847	0.532	5.95E-01	--++	42.7	1.55E-01	8.71E-01
100002152	androsteroidmonosulfateC19H28O6S.1.	10847	-0.556	5.78E-01	+--+	0.5	3.89E-01	8.71E-01
100002154	ergothioneine	10847	0.534	5.94E-01	-++-	31.1	2.26E-01	8.71E-01
100001461	1.stearoyl.GPE.18.0.	10847	0.536	5.92E-01	+++-	0	8.43E-01	8.71E-01
100005352	1.eicosenoylglycerophosphocholine.20.1n9.	9802	-0.531	5.95E-01	?--?	0	8.33E-01	8.71E-01
229	arachidonate.20.4n6.	10847	0.553	5.80E-01	-++-	0	4.34E-01	8.71E-01
100001733	hexanoylglutamine	6208	-0.532	5.95E-01	+?--	0.1	3.68E-01	8.71E-01
100006379	C.glycosyltryptophan	10847	-0.531	5.96E-01	+--+	0	8.40E-01	8.71E-01
100002167	12.HETE	10847	-0.554	5.79E-01	-+--	0	9.45E-01	8.71E-01
254	3.hydroxybutyrate.BHBA.	10847	-0.541	5.88E-01	+---	0	5.94E-01	8.71E-01
100002356	17.methylstearate	10847	-0.531	5.96E-01	++--	55.3	8.15E-02	8.71E-01
100002018	5alpha.androstan.3alpha.17beta.diolmonosulfate.	10847	-0.531	5.96E-01	--+-	48.9	1.18E-01	8.71E-01
100001586	gulonate	10847	0.542	5.88E-01	+++-	20.8	2.85E-01	8.71E-01
100006171	eugenolsulfate	10847	-0.528	5.97E-01	--++	0	7.50E-01	8.71E-01
100009227	1.linoleoyl.GPG.18.2.	6208	-0.531	5.96E-01	-?-+	9.9	3.30E-01	8.71E-01
100015839	dihomo.linoleoylcarnitine.C20.2.	6208	-0.556	5.78E-01	+?--	0	8.12E-01	8.71E-01
100006108	phenylacetylcarnitine	10847	-0.532	5.95E-01	+---	0	9.36E-01	8.71E-01
252	succinate	10847	-0.533	5.94E-01	----	0	7.13E-01	8.71E-01
561	glutamate	10847	0.535	5.92E-01	++-+	71.4	1.49E-02	8.71E-01
1111	vanillylmandelate.VMA.	10847	-0.552	5.81E-01	+--+	0	9.59E-01	8.71E-01
100008999	1..1.enyl.stearoyl..2.arachidonoyl.GPE.P.18.0.20.4	10847	-0.525	6.00E-01	-+--	7.7	3.54E-01	8.73E-01
1104	methylindole.3.acetate	10847	-0.519	6.04E-01	+---	0	5.18E-01	8.75E-01
100001577	N.acetylcitrulline	10847	0.517	6.05E-01	+-++	0	7.44E-01	8.76E-01
100001386	heme	5085	-0.507	6.12E-01	--??	0	4.39E-01	8.79E-01
100001324	ADpSGEGDFXAEGGGVR	6208	-0.506	6.13E-01	-?-+	0	4.75E-01	8.79E-01
100002024	5alpha.androstan.3beta.17beta.diolmonosulfate.2	10847	-0.503	6.15E-01	--+-	61.4	5.08E-02	8.79E-01
100000039	methioninesulfoxide	10847	0.504	6.14E-01	+-+-	8.9	3.48E-01	8.79E-01
100009005	1..1.enyl.palmitoyl..2.oleoyl.GPE.P.16.0.18.1.	10847	-0.504	6.15E-01	---+	0	8.93E-01	8.79E-01
100009028	N.palmitoyl.sphinganine.d18.0.16.0.	10847	-0.504	6.14E-01	--+-	0	8.74E-01	8.79E-01
100006370	3beta.hydroxy.5.cholestenoate	6208	0.506	6.13E-01	-?++	50.3	1.34E-01	8.79E-01
100002500	formiminoglutamate	5762	-0.5	6.17E-01	??-+	32.3	2.24E-01	8.80E-01
1518	N.palmitoyl.sphingosine.d18.1.16.0.	10847	-0.497	6.19E-01	++--	0	4.10E-01	8.82E-01
100015787	sphingomyelin.d18.1.19.0.d19.1.18.0.	6208	-0.486	6.27E-01	+?--	47.1	1.51E-01	8.89E-01
100001615	octadecanedioate	5684	0.482	6.30E-01	+-?+	33.2	2.24E-01	8.90E-01
100001229	stearidonate.18.4n3.	10847	-0.477	6.33E-01	++-+	0	7.50E-01	8.92E-01
100000263	imidazolelactate	10847	-0.475	6.35E-01	-+--	0	4.87E-01	8.93E-01
100001777	1.oleoyl.GPI.18.1.	10847	-0.469	6.39E-01	++--	45.2	1.40E-01	8.96E-01
313	sphinganine	10847	0.466	6.42E-01	-+++	0	8.09E-01	8.96E-01
100000036	3.methyl.2.oxovalerate	10847	-0.466	6.42E-01	+--+	0	8.13E-01	8.96E-01
100001739	dihomo.linolenate.20.3n3orn6.	10847	-0.466	6.42E-01	-+--	0	6.80E-01	8.96E-01
100001553	1.dihomo.linoleoylglycerophosphocholine.20.2n6.	9802	-0.463	6.43E-01	?-+?	0	4.62E-01	8.96E-01
100006296	sphingomyelin.d18.1.22.2.d18.2.22.1.d16.1.24.2.	1045	-0.463	6.44E-01	-??-	0	9.06E-01	8.96E-01
100009337	caffeicacidsulfate	9802	-0.462	6.44E-01	?-+?	79.4	2.77E-02	8.96E-01
100001384	1.arachidoyl.GPC.20.0.	9802	-0.458	6.47E-01	?+-?	0	3.51E-01	8.97E-01
100008930	oleate.vaccenate.18.1.	10847	-0.45	6.53E-01	++--	0	8.60E-01	9.00E-01
267	cholinephosphate	10847	-0.453	6.51E-01	-+--	29.5	2.35E-01	9.00E-01
100009026	behenoyldihydrosphingomyelin.d18.0.22.0.	6208	-0.452	6.52E-01	-?--	0	9.61E-01	9.00E-01
100009162	1..1.enyl.palmitoyl..2.palmitoyl.GPC.P.16.0.16.0.	10847	0.446	6.55E-01	++--	55.1	8.28E-02	9.03E-01
1052	glycerate	10847	-0.445	6.56E-01	++--	62.1	4.79E-02	9.03E-01
272	corticosterone	1045	0.431	6.66E-01	+??+	0	7.74E-01	9.03E-01

100009000	1..1.enyl.palmitoyl..2.docosahexaenoyl.GPE.P.16.0	9802	0.429	6.68E-01	?+-?	48.7	1.63E-01	9.03E-01
100001267	piperine	10847	0.422	6.73E-01	--++	54.1	8.82E-02	9.03E-01
100008928	2.hydroxybutyrate.2.hydroxyisobutyrate	10847	0.424	6.72E-01	++--	28.2	2.43E-01	9.03E-01
100009161	1..1.enyl.palmitoyl..2.myristoyl.GPC.P.16.0.14.0.	9802	-0.432	6.66E-01	?+-?	0	5.53E-01	9.03E-01
100003892	lanthionine	10847	0.436	6.63E-01	--++	0	5.27E-01	9.03E-01
100006290	sphingomyelin.d18.1.20.0.d16.1.22.0.	10847	0.431	6.67E-01	-+--	20.7	2.86E-01	9.03E-01
100000924	1.oleoylglycerol.18.1.	10847	-0.441	6.59E-01	+---	0	8.89E-01	9.03E-01
100000463	indolelactate	10847	0.433	6.65E-01	-++-	0	4.74E-01	9.03E-01
1492	linoleamide.18.2n6.	5238	-0.443	6.58E-01	?-?+	7.8	2.98E-01	9.03E-01
100001618	1.myristoylglycerol.14.0.	10847	0.435	6.63E-01	+-++	0	4.92E-01	9.03E-01
100010936	oleoyl.arachidonoyl.glycerol.18.1.20.4.[1]	6208	0.422	6.73E-01	-?++	0	5.12E-01	9.03E-01
100001609	7.alpha.hydroxy.3.oxo.4.cholestenoate.7.Hoca.	10847	0.435	6.64E-01	+--+	60.3	5.63E-02	9.03E-01
100001412	N2.N2.dimethylguanosine	10847	-0.429	6.68E-01	+--+	0	7.10E-01	9.03E-01
197	S.adenosylhomocysteine.SAH.	5085	-0.425	6.71E-01	+-??	0	3.20E-01	9.03E-01
100009002	1..1.enyl.palmitoyl..2.arachidonoyl.GPE.P.16.0.20.	10847	-0.422	6.73E-01	---+	0	5.04E-01	9.03E-01
100006375	3.methoxycatecholsulfate.1.	10847	0.414	6.79E-01	+++-	0	6.53E-01	9.04E-01
803	mannose	10847	0.415	6.78E-01	+--+	65.2	3.48E-02	9.04E-01
100015833	arachidoylcarnitine.C20.	6208	-0.417	6.76E-01	+?+-	65.6	5.45E-02	9.04E-01
100009038	myristoyldihydrosphingomyelin.d18.0.14.0.	6208	-0.416	6.77E-01	+?--	52.9	1.20E-01	9.04E-01
100009051	1.stearoyl.2.dihomo.linolenoyl.GPC.18.0.20.3n3or	9802	0.405	6.86E-01	?++?	0	8.18E-01	9.09E-01
100000487	glycylvaline	1045	-0.398	6.90E-01	-??-	0	8.69E-01	9.09E-01
100009232	thioproline	10847	-0.398	6.91E-01	---+	60.5	5.53E-02	9.09E-01
100001579	2.hydroxypalmitate	10847	-0.401	6.88E-01	++--	24.2	2.66E-01	9.09E-01
355	histidine	10847	-0.395	6.93E-01	-+--	36.1	1.96E-01	9.09E-01
100004575	N2.N5.diacetylornithine	5684	-0.395	6.93E-01	-+?-	74.7	1.93E-02	9.09E-01
100000827	1.palmitoylglycerol.16.0.	10847	-0.393	6.95E-01	-+--	0	7.64E-01	9.10E-01
100006190	2.acetamidophenolsulfate	10847	0.377	7.06E-01	+++-	0	6.82E-01	9.11E-01
100001313	gamma.glutamylmethionine	10847	-0.38	7.04E-01	++-+	71.7	1.40E-02	9.11E-01
100001540	pyroglutamine	10847	0.388	6.98E-01	++-+	0	7.97E-01	9.11E-01
100015735	ceramide.d18.1.14.0.d16.1.16.0.	6208	-0.381	7.03E-01	+?--	62.5	6.97E-02	9.11E-01
100003926	3.hydroxybutyrylcarnitine.1.	10847	0.378	7.05E-01	+-++	12.1	3.32E-01	9.11E-01
1141	4.hydroxyphenylpyruvate	10847	-0.384	7.01E-01	++--	0	7.22E-01	9.11E-01
825	uracil	10847	-0.38	7.04E-01	+--+	0	7.07E-01	9.11E-01
363	myo.inositol	10847	-0.385	7.01E-01	-+--	52.1	9.95E-02	9.11E-01
1083	N.acetylmethionine	10847	0.38	7.04E-01	+-+-	0	4.87E-01	9.11E-01
925	heptanoate.7.0.	10248	-0.369	7.12E-01	++-?	0	6.66E-01	9.15E-01
100010916	palmitoyl.oleoyl.glycerol.16.0.18.1.[1]	6208	0.37	7.11E-01	+?+-	0	4.89E-01	9.15E-01
100015744	ceramide.d18.2.24.1.d18.1.24.2.	6208	-0.369	7.12E-01	-?-+	0	9.06E-01	9.15E-01
100005389	ferulicacid4.sulfate	9802	-0.372	7.10E-01	?-+?	70.3	6.67E-02	9.15E-01
100000787	N.acetylaspartate.NAA.	10847	0.359	7.19E-01	--+-	0	5.86E-01	9.15E-01
100001148	5.hydroxyhexanoate	10847	0.363	7.17E-01	+-+-	0	5.45E-01	9.15E-01
564	threonine	10847	-0.361	7.18E-01	-+--	63	4.39E-02	9.15E-01
100005371	1.eicosatrienoylglycerophosphoethanolamine	9802	0.365	7.15E-01	?++?	0	8.61E-01	9.15E-01
100006295	sphingomyelin.d18.1.22.1.d18.2.22.0.d16.1.24.1.	10847	-0.361	7.18E-01	-+--	72.9	1.13E-02	9.15E-01
100004442	1.arachidonoyl.GPA.20.4.	10847	-0.357	7.21E-01	--+-	59.3	6.10E-02	9.16E-01
100002067	pregnsteroidmonosulfateC21H34O5S	10847	0.355	7.23E-01	+++-	0	6.26E-01	9.17E-01
1099	guanosine	10847	0.349	7.27E-01	+--+	6	3.63E-01	9.20E-01
100001323	DSGEGDFXAEGGGVR	10847	0.35	7.26E-01	-+-+	0	4.67E-01	9.20E-01
100000656	1.stearoyl.GPI.18.0.	10847	0.347	7.29E-01	++--	57.6	6.95E-02	9.20E-01
100001551	1.arachidonoyl.GPC.20.4n6.	10847	0.346	7.30E-01	-++-	36.2	1.95E-01	9.20E-01
100015962	N.trimethyl5.aminovalerate	6208	-0.346	7.30E-01	+?-+	0	9.20E-01	9.20E-01
100009004	1..1.enyl.stearoyl..2.docosahexaenoyl.GPE.P.18.0.	9802	0.341	7.33E-01	?+-?	76	4.13E-02	9.21E-01
100006116	methyl.4.hydroxybenzoatesulfate	10847	0.336	7.37E-01	-+--	51	1.06E-01	9.21E-01
171	hypoxanthine	10847	-0.334	7.39E-01	---+	0	4.84E-01	9.21E-01
100006642	glycodeoxycholatesulfate	10847	-0.331	7.41E-01	---+	0	7.46E-01	9.21E-01
1218	acetoacetate	10401	0.344	7.31E-01	?-+-	0	4.79E-01	9.21E-01
100001108	3.methylxanthine	10847	-0.332	7.40E-01	-+--	0	5.25E-01	9.21E-01
1342	3.methoxytyrosine	10847	-0.33	7.41E-01	+--+	30.6	2.29E-01	9.21E-01
100009007	1..1.enyl.palmitoyl..2.oleoyl.GPC.P.16.0.18.1.	10847	-0.33	7.41E-01	+-+-	43.6	1.50E-01	9.21E-01
100001674	2.arachidonoylglycerophosphoethanolamine	9802	0.34	7.34E-01	?+-?	0	5.96E-01	9.21E-01
310	cystathionine	10847	0.337	7.36E-01	++-+	0	4.71E-01	9.21E-01
181	laurate.12.0.	10847	-0.332	7.40E-01	+---	22.6	2.75E-01	9.21E-01
100015793	sphingomyelin.d17.2.16.0.d18.2.15.0.	6208	-0.34	7.34E-01	-?+-	60.8	7.82E-02	9.21E-01
100004328	sphingomyelin.d18.1.14.0.d16.1.16.0.	10847	0.324	7.46E-01	++--	74	9.10E-03	9.23E-01
100000016	suberate.octanedioate.	10847	-0.325	7.45E-01	+--+	0	5.47E-01	9.23E-01
100000784	theanine	10248	-0.323	7.46E-01	-+-?	8	3.37E-01	9.23E-01
100001776	2.linoleoylglycerophosphoethanolamine	9802	-0.322	7.47E-01	?--?	0	8.59E-01	9.23E-01
144	4.hydroxyphenylacetate	5238	-0.319	7.50E-01	?-?+	0	6.16E-01	9.25E-01
100002196	13.HODE+9.HODE	10847	0.315	7.53E-01	--++	0	4.74E-01	9.25E-01
100015786	sphingomyelin.d18.0.20.0.d16.0.22.0.	6208	0.304	7.61E-01	+?+-	0	8.31E-01	9.26E-01
330	fumarate	10847	0.309	7.57E-01	+-+-	0	9.03E-01	9.26E-01
302	deoxycholate	10847	0.304	7.61E-01	+--+	0	6.29E-01	9.26E-01
100002784	2.oxoarginine	6208	0.305	7.60E-01	+?+-	0	4.11E-01	9.26E-01
100001431	1.pentadecanoylglycerol.15.0.	5684	-0.307	7.59E-01	--?+	0	7.86E-01	9.26E-01

100008919	1..1.enyl.stearoyl..2.oleoyl.GPE.P.18.0.18.1.	6208	0.308	7.58E-01	+?-+	0	5.87E-01	9.26E-01
100008904	1.stearoyl.2.oleoyl.GPC.18.0.18.1.	10847	-0.283	7.77E-01	+---	0	8.78E-01	9.26E-01
100009008	1..1.enyl.palmitoyl..2.docosahexaenoyl.GPC.P.16.0	9802	0.281	7.78E-01	?+-?	56.9	1.28E-01	9.26E-01
100004635	methioninesulfone	10847	0.301	7.64E-01	-++-	0	5.67E-01	9.26E-01
100001569	1.oleoyl.GPE.18.1.	10847	-0.295	7.68E-01	+-++	0	5.34E-01	9.26E-01
100001266	N.acetylarginine	10847	0.282	7.78E-01	-++-	0	9.71E-01	9.26E-01
100009160	1..1.enyl.palmitoyl..2.palmitoleoyl.GPC.P.16.0.16.1	10847	-0.284	7.77E-01	+-+-	0	6.86E-01	9.26E-01
100009407	pimeloylcarnitine.3.methyladipoylcarnitine.C7.DC.	10847	0.285	7.75E-01	-+--	0	4.19E-01	9.26E-01
100006374	1.2.3.benzenetriolsulfate.2.	10847	0.297	7.67E-01	+-+-	62.6	4.57E-02	9.26E-01
100000963	homocitrulline	10847	-0.287	7.74E-01	+-+-	0	8.87E-01	9.26E-01
100015791	sphingomyelin.d18.2.23.1.	6208	-0.295	7.68E-01	+?--	0	6.00E-01	9.26E-01
1105	alpha.tocopherol	10847	-0.295	7.68E-01	-+--	57.9	6.81E-02	9.26E-01
535	uridine	10847	-0.285	7.75E-01	-+--	0	9.28E-01	9.26E-01
179	9.10.DiHOME	10847	-0.288	7.73E-01	--++	1.3	3.85E-01	9.26E-01
460	phenylalanine	10847	-0.277	7.82E-01	-+--	0	6.54E-01	9.27E-01
536	2..deoxyuridine	6208	-0.279	7.81E-01	+?-+	0	8.56E-01	9.27E-01
100006121	1.dihomo.linolenylglycerol.20.3.	10847	-0.273	7.85E-01	-+--	5.5	3.66E-01	9.29E-01
100010940	diacylglycerol.16.1.18.2[2].16.0.18.3[1].	1045	0.266	7.90E-01	+??-	13.1	2.83E-01	9.31E-01
445	orotate	10401	0.267	7.90E-01	?+--	41.3	1.82E-01	9.31E-01
100001337	linolenate[alphaorgamma..18.3n3or6.]	10847	-0.269	7.88E-01	+--+	0	7.38E-01	9.31E-01
100009036	1.margaroyl.2.oleoyl.GPC.17.0.18.1.	9802	0.267	7.90E-01	?+-?	0	5.04E-01	9.31E-01
1137	oleoylethanolamide	10847	-0.257	7.98E-01	+---	0	8.91E-01	9.37E-01
100001198	myristoleate.14.1n5.	10847	-0.254	7.99E-01	+---	0	9.54E-01	9.37E-01
432	nicotinamide	10847	0.245	8.07E-01	-+++	57.6	6.96E-02	9.42E-01
100001409	N1.methylinosine	10847	-0.229	8.19E-01	+--+	0	6.60E-01	9.45E-01
1123	chenodeoxycholate	10847	-0.232	8.17E-01	+---	0	5.86E-01	9.45E-01
100002128	17alpha.hydroxypregnenolone3.sulfate	1045	0.229	8.19E-01	+??-	72.5	5.65E-02	9.45E-01
1256	choline	10847	-0.233	8.16E-01	-++-	34.4	2.06E-01	9.45E-01
100001552	1.eicosatrienoylglycerophosphocholine.20.3.	9802	0.226	8.21E-01	?++?	0	8.88E-01	9.47E-01
100004499	6.oxopiperidine.2.carboxylate	10847	0.216	8.29E-01	-+-+	0	4.41E-01	9.55E-01
100004322	2.aminophenolsulfate	10847	-0.213	8.31E-01	+-+-	0	7.70E-01	9.56E-01
100006126	4.vinylguaiacolsulfate	10248	0.198	8.43E-01	+-+?	64.5	5.97E-02	9.57E-01
275	creatinine	10847	0.195	8.45E-01	+++-	0	5.82E-01	9.57E-01
100000707	maleate	10847	-0.202	8.40E-01	+--+	80	1.83E-03	9.57E-01
100001181	docosapentaenoate.n3DPA.22.5n3.	10847	0.208	8.35E-01	++--	0	6.57E-01	9.57E-01
821	pseudouridine	10847	0.2	8.42E-01	++-+	0	6.25E-01	9.57E-01
519	myristate.14.0.	10847	-0.199	8.42E-01	++--	0	4.99E-01	9.57E-01
1206	isocitrate	10401	-0.209	8.34E-01	?-+-	0	3.77E-01	9.57E-01
100004552	1.eicosapentaenoylglycerophosphoethanolamine	9802	0.195	8.45E-01	?+-?	0	3.17E-01	9.57E-01
100006184	2.methoxyresorcinolsulfate	9802	0.204	8.38E-01	?-+?	0	3.76E-01	9.57E-01
100010925	palmitoyl.arachidonoyl.glycerol.16.0.20.4.[2]	6208	0.191	8.48E-01	-?+-	0	7.16E-01	9.57E-01
132	3.phosphoglycerate	9802	0.192	8.48E-01	?+-?	0	5.54E-01	9.57E-01
100001170	3.hydroxy.2.ethylpropionate	10847	-0.192	8.48E-01	++--	50.3	1.10E-01	9.57E-01
100001611	3beta.7alpha.dihydroxy.5.cholestenoate	1045	0.19	8.50E-01	-??+	0	7.30E-01	9.58E-01
100001731	indoleacetylglutamine	6208	-0.188	8.51E-01	+?+-	0	4.09E-01	9.58E-01
100006361	dopamine3.O.sulfate	10847	0.175	8.61E-01	+--+	65.4	3.40E-02	9.58E-01
100000840	tartronate.hydroxymalonate.	10847	0.183	8.55E-01	+++-	67.7	2.56E-02	9.58E-01
100000776	palmitoylcarnitine.C16.	10847	0.18	8.57E-01	++--	59.8	5.85E-02	9.58E-01
100000015	xanthurenate	10847	0.18	8.57E-01	-++-	67	2.82E-02	9.58E-01
344	guanidinoacetate	10847	0.183	8.55E-01	++--	3.7	3.74E-01	9.58E-01
1668	taurodeoxycholate	10847	-0.175	8.61E-01	-+-+	17.2	3.05E-01	9.58E-01
100001556	2.oleoylglycerophosphocholine	9802	0.176	8.60E-01	?+-?	0	5.29E-01	9.58E-01
158	5.6.dihydrothymine	10847	-0.177	8.60E-01	++--	0	8.96E-01	9.58E-01
100001295	gamma.glutamyltryptophan	6208	-0.17	8.65E-01	+?--	0	9.65E-01	9.60E-01
100004295	2.piperidinone	10401	0.171	8.64E-01	?+-+	29.8	2.41E-01	9.60E-01
1261	12.HHTrE	1045	-0.165	8.69E-01	-??+	0	3.62E-01	9.60E-01
100000054	5.hydroxylysine	10847	-0.166	8.68E-01	+-++	35.2	2.01E-01	9.60E-01
1547	N.stearoyl.sphingosine.d18.1.18.0.	6208	0.166	8.68E-01	+?-+	82.9	2.91E-03	9.60E-01
100000011	phenylacetate	5762	0.15	8.81E-01	??+-	56.7	1.29E-01	9.60E-01
234	aspartate	10847	-0.156	8.76E-01	+---	0	9.99E-01	9.60E-01
100002126	16a.hydroxyDHEA3.sulfate	10847	-0.156	8.76E-01	++-+	4.4	3.71E-01	9.60E-01
100001048	2.palmitoylglycerol.16.0.	5684	0.153	8.79E-01	+-?+	0	9.04E-01	9.60E-01
500	riboflavin.VitaminB2.	5762	0.156	8.76E-01	??+-	0	3.28E-01	9.60E-01
100001232	5.dodecenoate.12.1n7.	10847	0.154	8.78E-01	+++-	0	9.55E-01	9.60E-01
409	malate	10847	-0.164	8.70E-01	++-+	26.2	2.55E-01	9.60E-01
100009406	palmitoleoylcarnitine.C16.1.	10847	-0.148	8.83E-01	++--	13.2	3.26E-01	9.60E-01
100009030	lactosyl.N.palmitoyl.sphingosine.d18.1.16.0.	10847	-0.154	8.78E-01	++--	54.5	8.63E-02	9.60E-01
100002173	1.pentadecanoylglycerophosphocholine.15.0.	9802	0.152	8.79E-01	?+-?	0	5.78E-01	9.60E-01
100010918	oleoyl.oleoyl.glycerol.18.1.18.1.[1]	6208	0.148	8.82E-01	+?+-	0	4.09E-01	9.60E-01
100001593	glutarylcarnitine.C5.DC.	10847	-0.157	8.75E-01	-+--	0	5.10E-01	9.60E-01
2050	eicosapentaenoate.EPA.20.5n3.	10847	-0.145	8.85E-01	++--	22.9	2.73E-01	9.62E-01
100002129	pregnenolonesulfate	10847	0.14	8.89E-01	++--	5.2	3.67E-01	9.65E-01
100000808	cysteines.sulfate	10401	0.133	8.94E-01	?-++	0	6.93E-01	9.69E-01
241	phenylpyruvate	10847	-0.134	8.94E-01	-+--	30.9	2.27E-01	9.69E-01

100010955	perfluorooctanesulfonicacid.PFOS.	6208	0.129	8.97E-01	-?+-	0	9.32E-01	9.69E-01
331	gamma.glutamylglutamate	10847	-0.13	8.97E-01	++-+	38.5	1.81E-01	9.69E-01
827	cytidine	6208	-0.131	8.96E-01	-?++	0	5.53E-01	9.69E-01
100001125	threonylphenylalanine	1045	0.117	9.07E-01	-??+	0	8.20E-01	9.70E-01
100008954	palmitoyldihydrosphingomyelin.d18.0.16.0.	10847	0.117	9.07E-01	-+--	28.4	2.42E-01	9.70E-01
100000096	4.guanidinobutanoate	10847	0.118	9.06E-01	+-+-	16.9	3.07E-01	9.70E-01
1488	arachidonoylethanolamide	1045	0.118	9.06E-01	-??+	0	5.15E-01	9.70E-01
100009131	1.linoleoyl.2.arachidonoyl.GPC.18.2.20.4n6.	10248	-0.121	9.04E-01	--+?	42.6	1.75E-01	9.70E-01
1140	gamma.glutamylglutamine	10847	-0.123	9.02E-01	-+--	48.5	1.21E-01	9.70E-01
100001275	phenylacetylglycine	9802	-0.114	9.09E-01	?+-?	79.9	2.58E-02	9.70E-01
100002911	glycoursodeoxycholate	10847	0.116	9.08E-01	--++	0	8.07E-01	9.70E-01
100010928	linoleoyl.docosahexaenoyl.glycerol.18.2.22.6.[1]	1045	0.113	9.10E-01	+??-	71.9	5.91E-02	9.70E-01
340	glycine	10847	0.122	9.03E-01	-+-+	0	8.06E-01	9.70E-01
100002952	docosadioate	6208	0.114	9.09E-01	-?+-	0	8.51E-01	9.70E-01
100001570	1.linoleoyl.GPE.18.2.	10847	-0.111	9.12E-01	+-+-	48.3	1.21E-01	9.71E-01
100001322	ADSGEGDFXAEGGGVR	6208	-0.104	9.17E-01	+?-+	75.5	1.68E-02	9.71E-01
35	S.1.pyrroline.5.carboxylate	10847	0.106	9.16E-01	-+-+	35.2	2.01E-01	9.71E-01
100001948	succinylcarnitine.C4.DC.	10847	0.103	9.18E-01	-+--	0	9.92E-01	9.71E-01
980	pentadecanoate.15.0.	5684	-0.105	9.16E-01	++?-	31	2.35E-01	9.71E-01
100001294	gamma.glutamylglycine	10847	0.103	9.18E-01	++--	0	3.97E-01	9.71E-01
100000626	sphingosine1.phosphate	10847	-0.099	9.21E-01	-+--	43.3	1.52E-01	9.73E-01
913	maltose	10847	-0.097	9.23E-01	--++	61.5	5.07E-02	9.73E-01
100006614	adipoylcarnitine.C6.DC.	10847	0.098	9.22E-01	-+-+	0	6.96E-01	9.73E-01
100000792	dehydroisoandrosteronesulfate.DHEA.S.	10847	0.091	9.28E-01	++--	0	5.31E-01	9.74E-01
826	xylose	6208	0.091	9.27E-01	+?-+	0	5.01E-01	9.74E-01
100015623	lactosyl.N.behenoyl.sphingosine.d18.1.22.0.	6208	-0.083	9.34E-01	+?+-	0	9.10E-01	9.75E-01
100006115	arabonate.xylonate	10847	-0.087	9.30E-01	+-++	26.6	2.52E-01	9.75E-01
194	N.formylmethionine	10847	-0.083	9.34E-01	--+-	0	6.94E-01	9.75E-01
100005351	1.eicosapentaenoylglycerophosphocholine.20.5n3	9802	0.084	9.33E-01	?+-?	0	3.41E-01	9.75E-01
100001734	N6.acetyllysine	10847	0.078	9.38E-01	++--	0	4.09E-01	9.76E-01
1231	dihomo.linoleate.20.2n6.	10847	0.077	9.39E-01	++--	0	7.85E-01	9.76E-01
100006191	p.cresol.glucuronide	10847	-0.079	9.37E-01	+-+-	54.7	8.48E-02	9.76E-01
100002528	sulfate	10847	0.069	9.45E-01	-++-	70.2	1.80E-02	9.78E-01
100001104	N.acetyltyrosine	10847	0.073	9.42E-01	--++	0	9.29E-01	9.78E-01
180	linoleate.18.2n6.	10847	-0.067	9.47E-01	-+--	0	9.90E-01	9.78E-01
100001612	N.acetyl.aspartyl.glutamate.NAAG.	10401	0.07	9.44E-01	?-++	49.8	1.36E-01	9.78E-01
100009135	1..1.enyl.stearoyl..2.linoleoyl.GPC.P.18.0.18.2.	9802	0.07	9.44E-01	?+-?	39.9	1.97E-01	9.78E-01
828	arabinose	10401	-0.066	9.47E-01	?-++	0	8.30E-01	9.78E-01
100010923	linoleoyl.arachidonoyl.glycerol.18.2.20.4.[2]	1045	-0.056	9.55E-01	-??+	36.2	2.11E-01	9.80E-01
100005466	N.acetyltaurine	10847	0.056	9.55E-01	+--+	0	5.21E-01	9.80E-01
100000551	4.methyl.2.oxopentanoate	10847	0.06	9.52E-01	--+-	0	6.99E-01	9.80E-01
917	asparagine	10847	0.05	9.60E-01	-++-	17.4	3.04E-01	9.80E-01
100001274	N.acetylthreonine	10847	0.052	9.58E-01	++--	0	8.72E-01	9.80E-01
100001743	tryptophanbetaine	10847	-0.053	9.57E-01	-++-	33.9	2.09E-01	9.80E-01
100000491	gamma.glutamylphenylalanine	10847	-0.056	9.56E-01	+---	0	5.04E-01	9.80E-01
100001278	10.heptadecenoate.17.1n7.	10847	-0.058	9.54E-01	++--	0	7.84E-01	9.80E-01
100001408	2.myristoylglycerol.14.0.	5085	0.054	9.57E-01	+-??	0	4.46E-01	9.80E-01
537	trans.urocanate	6208	0.045	9.64E-01	+?--	0	9.64E-01	9.83E-01
100001456	7.methylguanine	10847	0.042	9.66E-01	+-++	0	8.38E-01	9.84E-01
100001990	taurocholenatesulfate	10401	0.038	9.70E-01	?+-+	0	7.95E-01	9.86E-01
100001423	4.hydroxyhippurate	10847	-0.027	9.79E-01	++--	0	6.73E-01	9.88E-01
100004329	sphingomyelin.d18.2.16.0.d18.1.16.1.	10847	0.028	9.78E-01	-+--	64.7	3.68E-02	9.88E-01
100006641	glycochenodeoxycholatesulfate	10847	-0.025	9.80E-01	--+-	0	9.34E-01	9.88E-01
297	sphingosine	10847	-0.033	9.74E-01	-+-+	0	9.80E-01	9.88E-01
100015609	N.palmitoyl.sphingadienine.d18.2.16.0.	6208	0.029	9.77E-01	-?+-	0	7.17E-01	9.88E-01
100009035	1.pentadecanoyl.2.linoleoyl.GPC.15.0.18.2.	9802	0.028	9.78E-01	?-+?	0	6.93E-01	9.88E-01
424	palmitate.16.0.	10847	-0.026	9.79E-01	++--	0	6.84E-01	9.88E-01
482	lactate	10847	-0.032	9.75E-01	++-+	24.2	2.66E-01	9.88E-01
100000258	glycerol3.phosphate	10847	0.021	9.83E-01	+++-	0	4.19E-01	9.91E-01
100001999	21.hydroxypregnenolonedisulfate	10847	0.015	9.88E-01	++--	0	7.09E-01	9.92E-01
100001359	aconitate[cisortrans]	5684	0.016	9.87E-01	+-?-	0	6.45E-01	9.92E-01
100009343	1.linoleoyl.2.linolenoyl.GPC.18.2.18.3.	10847	0.012	9.90E-01	+-+-	44.1	1.47E-01	9.93E-01
100001765	3.methyladipate	1045	-0.011	9.91E-01	+??-	62	1.05E-01	9.93E-01
100000436	glycodeoxycholate	10847	0.01	9.92E-01	---+	26.4	2.54E-01	9.93E-01
100001435	1.linolenoylglycerol.18.3.	10847	0.006	9.95E-01	+-++	0	5.12E-01	9.95E-01

Supplementary Table 7: Results of the association of white matter hyperintensity volume with vitamin supplements.

variable	beta	se	p_value	N
vitaminA	490.486558	235.941265	0.03764081	26231
vitaminD	234.969952	172.580142	0.17336333	26861

Supplementary Table 8: Association of significant metabolites with C-Reactive protein (CRP)

**Metabolite**	**effect**	**SE**	**p-value**
Retinol	-0.067522	0.010918	6.22E-10
Hippurate	-0.097785	0.010954	0
10-undecenoate (11:1n1)	0.020261	0.010815	0.06101
4-hydroxycoumarin	-0.087644	0.010738	3.33E-16
2-aminooctanoate	-0.076589	0.010937	2.51E-12
mannitol/sorbitol	0.003993	0.010876	0.71355
1-linoleoyl-GPA (18:2)*	-0.154226	0.010708	0

Supplementary Table 9: Association of food sources of significant metabolites with C-Reactive protein (CRP)

Metabolite	Food source	beta	se	p_value	N
Retinol	Vitamin A supplements	-0.02307	0.00994	2.03E-02	321733
Retinol	Retinol from food	0.00006	0.00002	6.69E-04	63264
Hippurate	Fresh fruits	-0.04316	0.00088	0.00E+00	458920
Hippurate	Raw vegetables	-0.01994	0.00066	1.12E-199	454703
4-Hydroxycoumarin	Vitamin K antagonist	0.16592	0.01321	3.72E-36	461450
Mannitol/sorbitol	Artificial sweetener	0.01901	0.00809	1.88E-02	65205
1-linoleoyl-GPA (18:2)*	Legumes	-0.07386	0.01364	6.16E-08	65205
1-palmitoyl-2-palmitoleoyl-GPC (16:0/16:1)	Egg	0.03632	0.00736	7.98E-07	65205

Supplementary Table 10: Results of the MR analysis with major depression as exposure and metabolites as outcome.

Metabolite	Method	Estimate	Std Error	Lower 95% CI	Upper 95% CI	P-value
1-palmitoyl-2-palmitoleoyl-GPC (16:0/16:1)						
	Simple median	-0.06	0.12	-0.30	0.18	0.62
	Weighted median	-0.07	0.12	-0.32	0.17	0.56
	Penalized weighted median	-0.15	0.12	-0.39	0.09	0.23
	IVW	0.09	0.09	-0.09	0.28	0.33
	Penalized IVW	0.03	0.09	-0.14	0.21	0.73
	Robust IVW	0.04	0.11	-0.18	0.25	0.74
	Penalized robust IVW	0.01	0.10	-0.18	0.20	0.94
	MR-Egger	0.60	0.44	-0.26	1.47	0.17
	(intercept)	-0.01	0.01	-0.03	0.01	0.24
	Penalized MR-Egger	0.42	0.44	-0.44	1.27	0.34
	(intercept)	-0.01	0.01	-0.03	0.01	0.40
	Robust MR-Egger	0.54	0.69	-0.82	1.90	0.43
	(intercept)	-0.01	0.01	-0.04	0.02	0.43
	Penalized robust MR-Egger	0.46	0.61	-0.73	1.65	0.45
	(intercept)	-0.01	0.01	-0.03	0.02	0.45
2-aminooctanoate						
	Simple median	-0.12	0.12	-0.35	0.12	0.33
	Weighted median	-0.05	0.12	-0.29	0.18	0.65
	Penalized weighted median	-0.09	0.12	-0.32	0.14	0.46
	IVW	0.06	0.08	-0.10	0.22	0.48
	Penalized IVW	0.05	0.08	-0.12	0.21	0.58
	Robust IVW	0.02	0.10	-0.18	0.21	0.87
	Penalized robust IVW	0.02	0.10	-0.17	0.20	0.87
	MR-Egger	0.85	0.39	0.09	1.61	0.03
	(intercept)	-0.02	0.01	-0.03	0.00	0.04
	Penalized MR-Egger	0.85	0.39	0.09	1.61	0.03
	(intercept)	-0.02	0.01	-0.03	0.00	0.04
	Robust MR-Egger	0.81	0.52	-0.21	1.83	0.12
	(intercept)	-0.02	0.01	-0.04	0.00	0.11
	Penalized robust MR-Egger	0.81	0.52	-0.21	1.83	0.12
	(intercept)	-0.02	0.01	-0.04	0.00	0.11
10-undecenoate (11:1n1)						
	Simple median	0.12	0.12	-0.12	0.36	0.33
	Weighted median	0.24	0.12	0.00	0.48	0.05
	Penalized weighted median	0.25	0.12	0.01	0.49	0.04
	IVW	0.14	0.09	-0.03	0.31	0.11
	Penalized IVW	0.15	0.08	-0.01	0.32	0.07
	Robust IVW	0.15	0.09	-0.03	0.32	0.10
	Penalized robust IVW	0.15	0.09	-0.02	0.32	0.09
	MR-Egger	0.07	0.41	-0.73	0.88	0.86
	(intercept)	0.00	0.01	-0.02	0.02	0.87
	Penalized MR-Egger	0.09	0.40	-0.69	0.87	0.83
	(intercept)	0.00	0.01	-0.02	0.02	0.86
	Robust MR-Egger	0.10	0.34	-0.57	0.77	0.77
	(intercept)	0.00	0.01	-0.01	0.02	0.89
	Penalized robust MR-Egger	0.10	0.33	-0.54	0.75	0.76
	(intercept)	0.00	0.01	-0.01	0.02	0.88
Hippurate						
	Simple median	-0.06	0.12	-0.30	0.17	0.59
	Weighted median	-0.10	0.12	-0.34	0.13	0.40
	Penalized weighted median	-0.10	0.12	-0.33	0.14	0.42
	IVW	-0.10	0.09	-0.28	0.07	0.23
	Penalized IVW	-0.08	0.08	-0.25	0.08	0.32
	Robust IVW	-0.09	0.09	-0.26	0.08	0.28
	Penalized robust IVW	-0.09	0.09	-0.26	0.08	0.32
	MR-Egger	0.00	0.41	-0.80	0.81	0.99
	(intercept)	0.00	0.01	-0.02	0.02	0.79
	Penalized MR-Egger	-0.11	0.39	-0.88	0.65	0.77
	(intercept)	0.00	0.01	-0.02	0.02	0.94
	Robust MR-Egger	-0.09	0.37	-0.81	0.64	0.82
	(intercept)	0.00	0.01	-0.02	0.02	0.98
	Penalized robust MR-Egger	-0.12	0.36	-0.82	0.58	0.74
	(intercept)	0.00	0.01	-0.01	0.02	0.93

Mannitol/Sorbitol						
	Simple median	0.04	0.12	-0.19	0.26	0.76
	Weighted median	0.04	0.12	-0.19	0.27	0.76
	Penalized weighted median	0.04	0.12	-0.19	0.27	0.72
	IVW	0.00	0.08	-0.17	0.16	0.97
	Penalized IVW	0.01	0.08	-0.15	0.17	0.87
	Robust IVW	0.04	0.09	-0.13	0.21	0.62
	Penalized robust IVW	0.04	0.08	-0.12	0.21	0.60
	MR-Egger	0.34	0.39	-0.42	1.10	0.38
	(intercept)	-0.01	0.01	-0.02	0.01	0.37
	Penalized MR-Egger	0.45	0.38	-0.30	1.20	0.24
	(intercept)	-0.01	0.01	-0.03	0.01	0.24
	Robust MR-Egger	0.41	0.34	-0.25	1.07	0.22
	(intercept)	-0.01	0.01	-0.02	0.01	0.27
	Penalized robust MR-Egger	0.43	0.33	-0.22	1.07	0.20
	(intercept)	-0.01	0.01	-0.02	0.01	0.25
Retinol						
	Simple median	-0.02	0.12	-0.26	0.22	0.88
	Weighted median	0.06	0.12	-0.18	0.29	0.65
	Penalized weighted median	0.07	0.12	-0.17	0.30	0.59
	IVW	-0.04	0.09	-0.22	0.14	0.67
	Penalized IVW	-0.03	0.08	-0.20	0.13	0.72
	Robust IVW	-0.03	0.10	-0.23	0.16	0.73
	Penalized robust IVW	-0.03	0.09	-0.21	0.15	0.75
	MR-Egger	0.15	0.43	-0.69	0.99	0.73
	(intercept)	0.00	0.01	-0.02	0.01	0.65
	Penalized MR-Egger	0.40	0.40	-0.39	1.19	0.32
	(intercept)	-0.01	0.01	-0.03	0.01	0.27
	Robust MR-Egger	0.24	0.53	-0.80	1.28	0.65
	(intercept)	-0.01	0.01	-0.03	0.02	0.59
	Penalized robust MR-Egger	0.34	0.44	-0.52	1.20	0.44
	(intercept)	-0.01	0.01	-0.03	0.01	0.39

Supplementary Table 11: Results of the MR analysis with metabolites as exposure and major depression as outcome.

Metabolites	Method	Estimate	Std Error	Lower 95% CI	Upper 95% CI	P-value
1-palmitoyl-2-palmitoleoyl-GPC (16:0/16:1)						
	Simple median	-0.04	0.03	-0.11	0.02	2.14E-01
	Weighted median	-0.03	0.03	-0.10	0.03	2.92E-01
	Penalized weighted median	-0.05	0.03	-0.11	0.01	8.92E-02
	IVW	0.00	0.04	-0.07	0.07	9.75E-01
	Penalized IVW	-0.03	0.03	-0.08	0.02	2.37E-01
	Robust IVW	0.00	0.04	-0.09	0.08	9.05E-01
	Penalized robust IVW	-0.03	0.04	-0.10	0.03	3.31E-01
	MR-Egger	-0.21	0.14	-0.48	0.06	1.31E-01
	(intercept)	0.02	0.01	-0.01	0.05	1.22E-01
	Penalized MR-Egger	-0.21	0.14	-0.48	0.06	1.31E-01
	(intercept)	0.02	0.01	-0.01	0.05	1.22E-01
	Robust MR-Egger	-0.21	0.10	-0.40	-0.02	3.28E-02
	(intercept)	0.02	0.01	0.00	0.05	6.04E-02
	Penalized robust MR-Egger	-0.21	0.10	-0.40	-0.02	3.28E-02
	(intercept)	0.02	0.01	0.00	0.05	6.04E-02
2-aminooctanoate						
	Simple median	0.00	0.02	-0.03	0.04	8.48E-01
	Weighted median	0.00	0.01	-0.02	0.02	6.43E-01
	Penalized weighted median	0.00	0.01	-0.02	0.02	6.58E-01
	IVW	0.00	0.01	-0.03	0.02	6.98E-01
	Penalized IVW	0.00	0.01	-0.02	0.02	8.54E-01
	Robust IVW	0.00	0.00	-0.01	0.01	4.55E-01
	Penalized robust IVW	0.00	0.00	-0.01	0.01	5.89E-01
	MR-Egger	-0.01	0.02	-0.05	0.03	6.74E-01
	(intercept)	0.00	0.00	-0.01	0.01	8.06E-01
	Penalized MR-Egger	-0.01	0.02	-0.04	0.02	5.45E-01
	(intercept)	0.00	0.00	-0.01	0.01	5.39E-01
	Robust MR-Egger	-0.01	0.01	-0.03	0.01	3.21E-01
	(intercept)	0.00	0.00	-0.01	0.01	6.64E-01
	Penalized robust MR-Egger	-0.01	0.01	-0.03	0.01	2.68E-01
	(intercept)	0.00	0.00	0.00	0.01	5.25E-01
10-undecenoate (11:1n1)						
	Simple median	0.01	0.03	-0.05	0.06	7.81E-01
	Weighted median	-0.01	0.01	-0.03	0.02	6.72E-01
	Penalized weighted median	-0.01	0.01	-0.03	0.02	6.72E-01
	IVW	0.00	0.01	-0.03	0.02	8.67E-01
	Penalized IVW	0.00	0.01	-0.03	0.02	8.67E-01
	Robust IVW	0.00	0.01	-0.03	0.02	7.41E-01
	Penalized robust IVW	0.00	0.01	-0.03	0.02	7.41E-01
	MR-Egger	-0.02	0.02	-0.07	0.02	3.36E-01
	(intercept)	0.01	0.01	-0.01	0.02	2.85E-01
	Penalized MR-Egger	-0.02	0.02	-0.07	0.02	3.36E-01
	(intercept)	0.01	0.01	-0.01	0.02	2.85E-01
	Robust MR-Egger	-0.02	0.01	-0.04	0.00	2.97E-02
	(intercept)	0.01	0.00	0.00	0.01	1.56E-01
	Penalized robust MR-Egger	-0.02	0.01	-0.04	0.00	2.97E-02
	(intercept)	0.01	0.00	0.00	0.01	1.56E-01
Hippurate						
	Simple median	0.00	0.04	-0.07	0.07	9.79E-01
	Weighted median	0.01	0.04	-0.07	0.08	8.76E-01
	Penalized weighted median	-0.01	0.04	-0.08	0.06	8.65E-01
	IVW	0.02	0.04	-0.06	0.10	6.43E-01
	Penalized IVW	0.02	0.04	-0.06	0.10	6.43E-01
	Robust IVW	0.02	0.04	-0.06	0.10	6.44E-01
	Penalized robust IVW	0.02	0.04	-0.06	0.10	6.44E-01
	MR-Egger	-0.14	0.09	-0.30	0.03	1.17E-01
	(intercept)	0.02	0.01	0.00	0.03	5.59E-02
	Penalized MR-Egger	-0.14	0.09	-0.30	0.03	1.17E-01
	(intercept)	0.02	0.01	0.00	0.03	5.59E-02
	Robust MR-Egger	-0.13	0.04	-0.22	-0.05	1.59E-03
	(intercept)	0.02	0.00	0.01	0.02	6.01E-04
	Penalized robust MR-Egger	-0.13	0.04	-0.22	-0.05	1.59E-03
	(intercept)	0.02	0.00	0.01	0.02	6.01E-04

Retinol						
	Simple median	-0.01	0.03	-0.07	0.05	7.62E-01
	Weighted median	-0.01	0.03	-0.06	0.04	6.36E-01
	Penalized weighted median	-0.01	0.03	-0.06	0.04	6.36E-01
	IVW	0.00	0.02	-0.04	0.04	9.62E-01
	Penalized IVW	0.00	0.02	-0.04	0.04	9.62E-01
	Robust IVW	0.00	0.03	-0.06	0.05	8.75E-01
	Penalized robust IVW	0.00	0.03	-0.06	0.05	8.75E-01
	MR-Egger	-0.08	0.10	-0.29	0.12	4.27E-01
	(intercept)	0.01	0.01	-0.01	0.03	4.23E-01
	Penalized MR-Egger	-0.08	0.10	-0.29	0.12	4.27E-01
	(intercept)	0.01	0.01	-0.01	0.03	4.23E-01
	Robust MR-Egger	-0.08	0.07	-0.22	0.06	2.64E-01
	(intercept)	0.01	0.01	-0.01	0.02	3.19E-01
	Penalized robust MR-Egger	-0.08	0.07	-0.22	0.06	2.64E-01
	(intercept)	0.01	0.01	-0.01	0.02	3.19E-01

Supplementary Table 12: Results of the human and gut microbiome metabolic network analysis.

Chemical ID	Name	VMH ID	Present in AGORA2 (microbes)	Present in Recon3D (human)	Altered in IBD [1]	Altered in PD [2]	Altered in PD [3]
100001121	pyridoxate	4pyrdx	x	x			
212	5-methylthioadenosine (MTA)	5mta	x	x	x		
100006430	arabitol	abt	x	x			
192	N-acetylputrescine	aprut		x			
100000014	hippurate	bgly		x			
1090	bilirubin (Z,Z)	bilirub		x			
100001950	bilirubin (E,E)*	bilirub		x			
100001951	bilirubin (E,Z or Z,E)*	bilirub		x			
250	biliverdin	biliverd		x			
1128	2-aminobutyrate	C02356		x			
100001251	decanoylcarnitine (C10)	c10crn		x			
100001247	octanoylcarnitine (C8)	c8crn		x			
391	citrulline	citr_L	x	x			
100001392	laurylcarnitine (C12)	ddeccrn		x			
100000257	glucuronate	glcur	x	x	x		
561	glutamate	glu_L	x	x	x		x
100001657	glycolithocholate sulfate*	HC02197		x			
100001658	taurolithocholate 3-sulfate	HC02198		x			
100001083	indolepropionate	ind3ppa	x				
98	kynurenate	kynate		x			
397	leucine	leu_L	x	x	x		
100001740	mannitol	mnl	x				x
823	pyruvate	pyr	x	x	x		x
498	retinol (Vitamin A)	retinol	x	x			
100001740	sorbitol	sbt_L	x				
504	serotonin	srtn	x	x	x		
935	sucrose	sucr	x	x			
100006430	xylitol	xylt	x	x			

[1] https://www.biorxiv.org/content/10.1101/640649v1

[2] Integrated Analyses of Microbiome and Longitudinal Metabolome Data Reveal Microbial-Host Interactions on Sulfur Metabolism in Parkinson's Disease. Hertel J, Harms AC, Heinken A, Baldini Fet al, Cell Rep. 2019 No

[3] Parkinson's disease-associated alterations of the gut microbiome predict disease-relevant changes in metabolic functions. Baldini F, Hertel J, Sandt E, Thinnes CC et al, BMC Biol. 2020 Jun 9;18(1):62.

ov 12;29(7):1767-1777.e8

Supplementary Table 13: Depression-associated metabolites included in AGORA2 that could be consumed by at least one AGORA2 strain.
Shown is the uptake flux potential in mmol * g dry weight^{-1} * hr^{-1}.

VMH ID	5mta	abt	glcur	glu_L	leu_L	mnl	pyr	sucr	xylt
**Description**	**5-Methylthioadenosin**	**L-arabinitol**	**D-glucuronate**	**L-glutamate**	**L-leucine**	**D-Mannitol**	**Pyruvate**	**Sucrose**	**xylitol**
Abiotrophia_defectiva_ATCC_49176	0	0	0	-1000	-175.34384	0	0	-517.11027	0
Acaricomes_phytoseiuli_DSM_14247	0	0	0	-1000	-14.29848	0	0	-541.66667	0
Acaryochloris_marina_MBIC11017	0	0	0	-1000	-17.387458	0	0	0	0
Acetanaerobacterium_elongatum_CGMCC_1_501	0	0	0	0	-15.008236	0	0	-824.28831	0
Acetatifactor_muris_GP69	0	0	0	-39.18103493	-20.231364	0	0	-857.14286	0
Acetivibrio_cellulolyticus_CD2	0	0	0	0	-24.433562	0	0	0	0
Acetivibrio_ethanolgignens_ACET_33324	0	0	0	-47.6209129	-33.966418	-881.7196	-1000	0	0
Acetobacterium_wieringae_DSM_1911	0	0	0	-40.16255762	-18.94287	0	0	0	0
Acetonema_longum_DSM_6540	0	0	0	-1000	-26.498965	-1000	-1000	0	0
Achromobacter_insuavis_AXX_A	0	0	0	-1000	-27.943354	0	0	0	0
Achromobacter_xylosoxidans_A8	-1000	0	0	-1000	-39.453495	0	0	0	0
Achromobacter_xylosoxidans_ERR2221244	0	0	0	-1000	-32.589901	0	0	0	0
Achromobacter_xylosoxidans_ERR2221245	0	0	0	-1000	-32.589901	0	0	0	0
Achromobacter_xylosoxidans_ERR2221246	0	0	0	-1000	-32.589901	0	0	0	0
Achromobacter_xylosoxidans_ERR2221247	0	0	0	-1000	-32.589901	0	0	0	0
Achromobacter_xylosoxidans_NBRC_15126	0	0	0	-1000	-28.971716	0	0	0	0
Achromobacter_xylosoxidans_NCTC10807	0	0	0	-1000	-32.639546	0	0	0	0
Acidaminobacter_hydrogenoformans_DSM_2784	0	0	0	-1000	-1000	0	-1000	0	0
Acidaminococcus_fermentans_DSM_20731	0	0	0	-1000	-11.064285	0	0	0	0
Acidaminococcus_intestini_RyC_MR95	0	0	0	-1000	-166.69141	0	0	0	0
Acidaminococcus_sp_BV3L6	0	0	0	-1000	-5.7061404	0	0	0	0
Acidaminococcus_sp_D21	0	0	0	-1000	-18.582285	0	0	0	0
Acidaminococcus_sp_HPA0509	0	0	0	-1000	-9.2403373	0	0	0	0
Acidobacterium_ailaaui_PMMR2	0	0	-75.8657827	-1000	0	0	0	-1000	0
Acidobacterium_capsulatum_ATCC_51196	0	0	-1000	0	0	0	0	0	0
Acidovorax_caeni_R_24608	0	0	0	-1000	-1000	0	-1000	0	0
Acinetobacter_baumannii_1656_2	0	0	0	-1000	-1000	0	0	0	0
Acinetobacter_baumannii_3990	0	0	0	-1000	-1000	0	0	0	0
Acinetobacter_baumannii_48055	0	0	0	-1000	-1000	0	0	0	0
Acinetobacter_baumannii_53264	0	0	0	-1000	-1000	0	0	0	0
Acinetobacter_baumannii_6013113	0	0	0	-1000	-1000	0	0	0	0
Acinetobacter_baumannii_6013150	0	0	0	-1000	-1000	0	0	0	0
Acinetobacter_baumannii_6014059	0	0	0	-1000	-1000	0	0	0	0
Acinetobacter_baumannii_AA_014	0	0	0	-1000	-1000	0	0	0	0
Acinetobacter_baumannii_AB_1536_8	0	0	0	-1000	-1000	0	0	0	0
Acinetobacter_baumannii_AB_1582_8	0	0	0	-1000	-1000	0	0	0	0
Acinetobacter_baumannii_AB_1594_8	0	0	0	-1000	-1000	0	0	0	0
Acinetobacter_baumannii_AB_1595_8	0	0	0	-1000	-1000	0	0	0	0
Acinetobacter_baumannii_AB_1649_8	0	0	0	-1000	-1000	0	0	0	0
Acinetobacter_baumannii_AB_1650_8	0	0	0	-1000	-1000	0	0	0	0
Acinetobacter_baumannii_AB_1766_8	0	0	0	-1000	-1000	0	0	0	0
Acinetobacter_baumannii_AB_2007_09_110_01_	0	0	0	-1000	-1000	0	0	0	0
Acinetobacter_baumannii_AB_2007_16_25_01_7	0	0	0	-1000	-1000	0	0	0	0
Acinetobacter_baumannii_AB_2007_16_27_01	0	0	0	-1000	-1000	0	0	0	0
Acinetobacter_baumannii_AB_2008_15_34_7	0	0	0	-1000	-1000	0	0	0	0
Acinetobacter_baumannii_AB_2008_15_45	0	0	0	-1000	-1000	0	0	0	0
Acinetobacter_baumannii_AB_2008_15_52	0	0	0	-1000	-1000	0	0	0	0
Acinetobacter_baumannii_AB_2008_15_69	0	0	0	-1000	-1000	0	0	0	0
Acinetobacter_baumannii_AB_2008_15_70	0	0	0	-1000	-1000	0	0	0	0
Acinetobacter_baumannii_AB_2008_15_71	0	0	0	-1000	-1000	0	0	0	0
Acinetobacter_baumannii_AB_2008_23_01_01_7	0	0	0	-1000	-1000	0	0	0	0
Acinetobacter_baumannii_AB_2008_23_07_01_7	0	0	0	-1000	-1000	0	0	0	0
Acinetobacter_baumannii_AB_2009_04_01_7	0	0	0	-1000	-1000	0	0	0	0
Acinetobacter_baumannii_AB_2009_04_02_7	0	0	0	-1000	-1000	0	0	0	0
Acinetobacter_baumannii_AB_515_8	0	0	0	-1000	-1000	0	0	0	0
Acinetobacter_baumannii_AB_908_12	0	0	0	-1000	-1000	0	0	0	0
Acinetobacter_baumannii_AB_908_13	0	0	0	-1000	-1000	0	0	0	0
Acinetobacter_baumannii_AB_909_01_7	0	0	0	-1000	-1000	0	0	0	0
Acinetobacter_baumannii_AB_909_02_7	0	0	0	-1000	-1000	0	0	0	0
Acinetobacter_baumannii_AB_909_05	0	0	0	-1000	-1000	0	0	0	0
Acinetobacter_baumannii_AB_TG19617	0	0	0	-1000	-1000	0	0	0	0
Acinetobacter_baumannii_AB_TG2018	0	0	0	-1000	-1000	0	0	0	0
Acinetobacter_baumannii_AB_TG2022	0	0	0	-1000	-1000	0	0	0	0
Acinetobacter_baumannii_AB_TG2023	0	0	0	-1000	-1000	0	0	0	0
Acinetobacter_baumannii_AB_TG2026	0	0	0	-1000	-1000	0	0	0	0
Acinetobacter_baumannii_AB_TG2028	0	0	0	-1000	-1000	0	0	0	0
Acinetobacter_baumannii_AB_TG2030	0	0	0	-1000	-1000	0	0	0	0
Acinetobacter_baumannii_AB_TG2031	0	0	0	-1000	-1000	0	0	0	0
Acinetobacter_baumannii_AB_TG27335	0	0	0	-1000	-1000	0	0	0	0
Acinetobacter_baumannii_AB_TG27339	0	0	0	-1000	-1000	0	0	0	0
Acinetobacter_baumannii_AB_TG27343	0	0	0	-1000	-1000	0	0	0	0
Acinetobacter_baumannii_AB0057	0	0	0	-1000	-1000	-1000	0	0	0
Acinetobacter_baumannii_Ab11111	0	0	0	-1000	-1000	0	0	0	0
Acinetobacter_baumannii_AB1H8	0	0	0	-1000	-1000	0	0	0	0
Acinetobacter_baumannii_AB210	0	0	0	-1000	-1000	0	0	0	0
Acinetobacter_baumannii_AB307_0294	0	0	0	-1000	-1000	0	0	0	0
Acinetobacter_baumannii_AB31	0	0	0	-1000	-1000	0	0	0	0
Acinetobacter_baumannii_Ab33333	0	0	0	-1000	-1000	0	0	0	0
Acinetobacter_baumannii_AB405E4	0	0	0	-1000	-1000	0	0	0	0
Acinetobacter_baumannii_Ab44444	0	0	0	-1000	-1000	0	0	0	0
Acinetobacter_baumannii_AB4A3	0	0	0	-1000	-1000	0	0	0	0
Acinetobacter_baumannii_AB5256	0	0	0	-1000	-1000	0	0	0	0
Acinetobacter_baumannii_AB5711	0	0	0	-1000	-1000	0	0	0	0
Acinetobacter_baumannii_AB900	0	0	0	-1000	-1000	0	0	0	0
Acinetobacter_baumannii_ABIsac_ColiR	0	0	0	-1000	-1000	0	0	0	0

Acinetobacter_baumannii_ABNIH1	0	0	0	-1000	-1000	0	0	0	0
Acinetobacter_baumannii_ABNIH11	0	0	0	-1000	-1000	0	0	0	0
Acinetobacter_baumannii_ABNIH14	0	0	0	-1000	-1000	0	0	0	0
Acinetobacter_baumannii_ABNIH15	0	0	0	-1000	-1000	0	0	0	0
Acinetobacter_baumannii_ABNIH16	0	0	0	-1000	-1000	0	0	0	0
Acinetobacter_baumannii_ABNIH18	0	0	0	-1000	-1000	0	0	0	0
Acinetobacter_baumannii_ABNIH19	0	0	0	-1000	-1000	0	0	0	0
Acinetobacter_baumannii_ABNIH22	0	0	0	-1000	-1000	0	0	0	0
Acinetobacter_baumannii_ABNIH23	0	0	0	-1000	-1000	0	0	0	0
Acinetobacter_baumannii_ABNIH25	0	0	0	-1000	-1000	0	0	0	0
Acinetobacter_baumannii_ABNIH26	0	0	0	-1000	-1000	0	0	0	0
Acinetobacter_baumannii_ABNIH3	0	0	0	-1000	-1000	0	0	0	0
Acinetobacter_baumannii_ABNIH4	0	0	0	-1000	-1000	0	0	0	0
Acinetobacter_baumannii_ABNIH5	0	0	0	-1000	-1000	0	0	0	0
Acinetobacter_baumannii_ABNIH6	0	0	0	-1000	-1000	0	0	0	0
Acinetobacter_baumannii_AC12	0	0	0	-1000	-1000	0	0	0	0
Acinetobacter_baumannii_AC30	0	0	0	-1000	-1000	0	0	0	0
Acinetobacter_baumannii_ACICU	0	0	0	-1000	-1000	0	0	0	0
Acinetobacter_baumannii_ANC_4097	0	0	0	-1000	-1000	0	0	0	0
Acinetobacter_baumannii_ATCC_17978	0	0	0	-1000	-1000	0	0	0	0
Acinetobacter_baumannii_ATCC_19606	0	0	0	-1000	-1000	0	0	0	0
Acinetobacter_baumannii_AYE	0	0	0	-1000	-1000	0	0	0	0
Acinetobacter_baumannii_BZICU_2	0	0	0	-1000	-1000	0	0	0	0
Acinetobacter_baumannii_Canada_BC_5	0	0	0	-1000	-1000	0	0	0	0
Acinetobacter_baumannii_Canada_BC1	0	0	0	-1000	-1000	0	0	0	0
Acinetobacter_baumannii_IS_116	0	0	0	-1000	-1000	0	0	0	0
Acinetobacter_baumannii_IS_123	0	0	0	-1000	-1000	0	0	0	0
Acinetobacter_baumannii_IS_143	0	0	0	-1000	-1000	0	0	0	0
Acinetobacter_baumannii_IS_235	0	0	0	-1000	-1000	0	0	0	0
Acinetobacter_baumannii_IS_251	0	0	0	-1000	-1000	0	0	0	0
Acinetobacter_baumannii_IS_58	0	0	0	-1000	-1000	0	0	0	0
Acinetobacter_baumannii_LAC_4	0	0	0	-1000	-1000	0	0	0	0
Acinetobacter_baumannii_MDR_TJ	0	0	0	-1000	-1000	0	0	0	0
Acinetobacter_baumannii_MDR_ZJ06	0	0	0	-1000	-1000	0	0	0	0
Acinetobacter_baumannii_MSP4_16	0	0	0	-1000	-1000	0	0	0	0
Acinetobacter_baumannii_Naval_113	0	0	0	-1000	-1000	0	0	0	0
Acinetobacter_baumannii_Naval_13	0	0	0	-1000	-1000	0	0	0	0
Acinetobacter_baumannii_Naval_17	0	0	0	-1000	-1000	0	0	0	0
Acinetobacter_baumannii_Naval_18	0	0	0	-1000	-1000	0	0	0	0
Acinetobacter_baumannii_Naval_2	0	0	0	-1000	-1000	0	0	0	0
Acinetobacter_baumannii_Naval_21	0	0	0	-1000	-1000	0	0	0	0
Acinetobacter_baumannii_Naval_57	0	0	0	-1000	-1000	0	0	0	0
Acinetobacter_baumannii_Naval_72	0	0	0	-1000	-1000	0	0	0	0
Acinetobacter_baumannii_Naval_78	0	0	0	-1000	-1000	0	0	0	0
Acinetobacter_baumannii_Naval_81	0	0	0	-1000	-1000	0	0	0	0
Acinetobacter_baumannii_Naval_82	0	0	0	-1000	-1000	0	0	0	0
Acinetobacter_baumannii_Naval_83	0	0	0	-1000	-1000	0	0	0	0
Acinetobacter_baumannii_NIPH_1362	0	0	0	-1000	-1000	0	0	0	0
Acinetobacter_baumannii_NIPH_146	0	0	0	-1000	-1000	0	0	0	0
Acinetobacter_baumannii_NIPH_1669	0	0	0	-1000	-1000	0	0	0	0
Acinetobacter_baumannii_NIPH_1734	0	0	0	-1000	-1000	0	0	0	0
Acinetobacter_baumannii_NIPH_190	0	0	0	-1000	-1000	0	0	0	0
Acinetobacter_baumannii_NIPH_201	0	0	0	-1000	-1000	0	0	0	0
Acinetobacter_baumannii_NIPH_2061	0	0	0	-1000	-1000	0	0	0	0
Acinetobacter_baumannii_NIPH_24	0	0	0	-1000	-1000	0	0	0	0
Acinetobacter_baumannii_NIPH_290	0	0	0	-1000	-1000	0	0	0	0
Acinetobacter_baumannii_NIPH_329	0	0	0	-1000	-1000	0	0	0	0
Acinetobacter_baumannii_NIPH_335	0	0	0	-1000	-1000	0	0	0	0
Acinetobacter_baumannii_NIPH_410	0	0	0	-1000	-1000	0	0	0	0
Acinetobacter_baumannii_NIPH_527	0	0	0	-1000	-1000	0	0	0	0
Acinetobacter_baumannii_NIPH_528	0	0	0	-1000	-1000	0	0	0	0
Acinetobacter_baumannii_NIPH_60	0	0	0	-1000	-1000	0	0	0	0
Acinetobacter_baumannii_NIPH_601	0	0	0	-1000	-1000	0	0	0	0
Acinetobacter_baumannii_NIPH_615	0	0	0	-1000	-1000	0	0	0	0
Acinetobacter_baumannii_NIPH_67	0	0	0	-1000	-1000	0	0	0	0
Acinetobacter_baumannii_NIPH_70	0	0	0	-1000	-1000	0	0	0	0
Acinetobacter_baumannii_NIPH_80	0	0	0	-1000	-1000	0	0	0	0
Acinetobacter_baumannii_OIFC0162	0	0	0	-1000	-1000	0	0	0	0
Acinetobacter_baumannii_OIFC032	0	0	0	-1000	-1000	0	0	0	0
Acinetobacter_baumannii_OIFC035	0	0	0	-1000	-1000	0	0	0	0
Acinetobacter_baumannii_OIFC047	0	0	0	-1000	-1000	0	0	0	0
Acinetobacter_baumannii_OIFC065	0	0	0	-1000	-1000	0	0	0	0
Acinetobacter_baumannii_OIFC074	0	0	0	-1000	-1000	0	0	0	0
Acinetobacter_baumannii_OIFC087	0	0	0	-1000	-1000	0	0	0	0
Acinetobacter_baumannii_OIFC098	0	0	0	-1000	-1000	0	0	0	0
Acinetobacter_baumannii_OIFC099	0	0	0	-1000	-1000	0	0	0	0
Acinetobacter_baumannii_OIFC109	0	0	0	-1000	-1000	0	0	0	0
Acinetobacter_baumannii_OIFC110	0	0	0	-1000	-1000	0	0	0	0
Acinetobacter_baumannii_OIFC111	0	0	0	-1000	-1000	0	0	0	0
Acinetobacter_baumannii_OIFC137	0	0	0	-1000	-1000	0	0	0	0
Acinetobacter_baumannii_OIFC143	0	0	0	-1000	-1000	0	0	0	0
Acinetobacter_baumannii_OIFC180	0	0	0	-1000	-1000	0	0	0	0
Acinetobacter_baumannii_OIFC189	0	0	0	-1000	-1000	0	0	0	0
Acinetobacter_baumannii_OIFC338	0	0	0	-1000	-1000	0	0	0	0
Acinetobacter_baumannii_SDF	0	0	0	-1000	-1000	0	0	0	0
Acinetobacter_baumannii_TCDC_AB0715	0	0	0	-1000	-1000	0	0	0	0
Acinetobacter_baumannii_TYTH_1	0	0	0	-1000	-1000	0	0	0	0
Acinetobacter_baumannii_UMB001	0	0	0	-1000	-1000	0	0	0	0
Acinetobacter_baumannii_UMB003	0	0	0	-1000	-1000	0	0	0	0
Acinetobacter_baumannii_W7282	0	0	0	-1000	-1000	0	0	0	0
Acinetobacter_baumannii_WC_348	0	0	0	-1000	-1000	0	0	0	0

Acinetobacter_baumannii_WC_692	0	0	0	-1000	-1000	0	0	0	0
Acinetobacter_baumannii_WC_A_694	0	0	0	-1000	-1000	0	0	0	0
Acinetobacter_baumannii_WC_A_92	0	0	0	-1000	-1000	0	0	0	0
Acinetobacter_baumannii_WM99c	0	0	0	-1000	-1000	0	0	0	0
Acinetobacter_baumannii_ZWS1122	0	0	0	-1000	-1000	0	0	0	0
Acinetobacter_baumannii_ZWS1219	0	0	0	-1000	-1000	0	0	0	0
Acinetobacter_calcoaceticus_ANC_3680	0	0	0	-1000	-1000	0	0	0	0
Acinetobacter_calcoaceticus_ANC_3811	0	0	0	-1000	-1000	0	0	0	0
Acinetobacter_calcoaceticus_DSM_30006_CIP_81	0	0	0	-1000	-1000	0	0	0	0
Acinetobacter_calcoaceticus_NIPH_13	0	0	0	-1000	-1000	0	0	0	0
Acinetobacter_calcoaceticus_PHEA_2	0	0	0	-1000	-1000	0	0	0	0
Acinetobacter_calcoaceticus_RUH2202	0	0	0	-1000	-1000	0	0	0	0
Acinetobacter_calcoaceticus_subsp_anitratus_XM	0	0	0	-1000	-1000	0	0	0	0
Acinetobacter_calcoaceticus_TG19585	0	0	0	-1000	-1000	0	0	0	0
Acinetobacter_calcoaceticus_TG19588	0	0	0	-1000	-1000	0	0	0	0
Acinetobacter_calcoaceticus_TG19593	0	0	0	-1000	-1000	0	0	0	0
Acinetobacter_guillouiae_CIP_63_46	0	0	0	-1000	-23.623997	0	0	0	0
Acinetobacter_guillouiae_KCTC_23200	0	0	0	-1000	-20.044498	0	0	0	0
Acinetobacter_guillouiae_MSP4_18	0	0	0	-1000	-20.044498	0	0	0	0
Acinetobacter_guillouiae_NIPH_991	0	0	0	-1000	-22.227783	0	0	0	0
Acinetobacter_haemolyticus_ATCC_19194	0	0	0	-1000	-1000	0	0	0	0
Acinetobacter_haemolyticus_CIP_64_3	0	0	0	-1000	-1000	0	0	0	0
Acinetobacter_haemolyticus_NIPH_261	0	0	0	-1000	-1000	0	0	0	0
Acinetobacter_haemolyticus_TG19599	0	0	0	-1000	-1000	0	0	0	0
Acinetobacter_haemolyticus_TG21157	0	0	0	-1000	-1000	0	0	0	0
Acinetobacter_johnsonii_ANC_3681	0	0	0	-1000	-1000	0	0	0	0
Acinetobacter_johnsonii_CIP_64_6	0	0	0	-1000	-1000	0	0	0	0
Acinetobacter_johnsonii_SH046	0	0	0	-1000	-1000	-547.33728	0	0	0
Acinetobacter_junii_CIP_107470	0	0	0	-1000	-1000	0	0	0	0
Acinetobacter_junii_CIP_64_5	0	0	0	-1000	-1000	0	0	0	0
Acinetobacter_junii_NIPH_182	0	0	0	-1000	-1000	0	0	0	0
Acinetobacter_junii_SH205	0	0	0	-1000	-1000	0	0	0	0
Acinetobacter_lwoffii_CIP_70_31	0	0	0	-1000	-1000	0	0	0	0
Acinetobacter_lwoffii_ERR2221242	0	0	0	-1000	-1000	0	0	0	0
Acinetobacter_lwoffii_NCTC_5866	0	0	0	-1000	-1000	0	0	0	0
Acinetobacter_lwoffii_NIPH_478	0	0	0	-1000	-1000	0	0	0	0
Acinetobacter_lwoffii_NIPH_715	0	0	0	-1000	-1000	0	0	0	0
Acinetobacter_lwoffii_SH145	0	0	0	-1000	-1000	0	0	0	0
Acinetobacter_lwoffii_TG19636	0	0	0	-1000	-1000	0	0	0	0
Acinetobacter_lwoffii_WJ10621	0	0	0	-1000	-1000	0	0	0	0
Acinetobacter_nosocomialis_NIPH_2119	0	0	0	-1000	-1000	0	0	0	0
Acinetobacter_nosocomialis_WC_487	0	0	0	-1000	-1000	0	0	0	0
Acinetobacter_pittii_ANC_3678	0	0	0	-1000	-1000	0	0	0	0
Acinetobacter_pittii_ANC_4050	0	0	0	-1000	-1000	0	0	0	0
Acinetobacter_pittii_ANC_4052	0	0	0	-1000	-1000	0	0	0	0
Acinetobacter_pittii_AP_882	0	0	0	-1000	-1000	0	0	0	0
Acinetobacter_pittii_CIP_70_29	0	0	0	-1000	-1000	0	0	0	0
Acinetobacter_pittii_D499	0	0	0	-1000	-1000	0	0	0	0
Acinetobacter_pittii_IEC338SC	0	0	0	-1000	-1000	0	0	0	0
Acinetobacter_pittii_TG6411	0	0	0	-1000	-1000	0	0	0	0
Acinetobacter_pittii_WC_136	0	0	0	-1000	-1000	0	0	0	0
Acinetobacter_radioresistens_DSM_6976	0	0	0	-1000	-1000	0	0	0	0
Acinetobacter_radioresistens_NIPH_2130	0	0	0	-1000	-1000	0	0	0	0
Acinetobacter_radioresistens_SH164	0	0	0	-1000	-1000	0	0	0	0
Acinetobacter_radioresistens_SK82	0	0	0	-1000	-1000	0	0	0	0
Acinetobacter_radioresistens_TG02010	0	0	0	-1000	-1000	0	0	0	0
Acinetobacter_radioresistens_WC_A_157	0	0	0	-1000	-1000	0	0	0	0
Acinetobacter_sp_ATCC_27244	0	0	0	-1000	-1000	0	0	0	0
Acinetobacter_sp_OIFC021	0	0	0	-1000	-1000	0	0	0	0
Acinetobacter_sp_RUH2624	0	0	0	-1000	-1000	0	0	0	0
Acinetobacter_sp_SH024	0	0	0	-1000	-1000	0	0	0	0
Acinetobacter_sp_WC_141	0	0	0	-1000	-1000	0	0	0	0
Acinetobacter_sp_WC_323	0	0	0	-1000	-1000	0	0	0	0
Actinobacillus_pleuropneumoniae_L20	0	0	0	-1000	-49.425511	-1000	0	-1000	0
Actinobacillus_pleuropneumoniae_S8	0	0	0	-1000	-46.522405	-1000	0	-1000	0
Actinobacillus_pleuropneumoniae_serovar_1_str	0	0	0	-1000	-44.954357	-1000	0	-1000	0
Actinobacillus_pleuropneumoniae_serovar_10_st	0	0	0	-1000	-46.605181	-1000	0	-1000	0
Actinobacillus_pleuropneumoniae_serovar_11_st	0	0	0	-1000	-46.674126	-1000	0	-1000	0
Actinobacillus_pleuropneumoniae_serovar_12_st	0	0	0	-1000	-42.588848	-1000	0	-1000	0
Actinobacillus_pleuropneumoniae_serovar_13_st	0	0	0	-1000	-46.522405	-1000	0	-1000	0
Actinobacillus_pleuropneumoniae_serovar_2_str	0	0	0	-1000	-46.522405	-1000	0	-1000	0
Actinobacillus_pleuropneumoniae_serovar_2_str	0	0	0	-1000	-44.407303	-1000	0	-1000	0
Actinobacillus_pleuropneumoniae_serovar_3_str	0	0	0	-1000	-46.63047	-1000	0	-1000	0
Actinobacillus_pleuropneumoniae_serovar_4_str	0	0	0	-1000	-44.302888	-1000	0	-1000	0
Actinobacillus_pleuropneumoniae_serovar_6_str	0	0	0	-1000	-46.547604	-1000	0	-1000	0
Actinobacillus_pleuropneumoniae_serovar_7_str	0	0	0	-1000	-45.808943	-1000	0	-1000	0
Actinobacillus_pleuropneumoniae_serovar_9_str	0	0	0	-1000	-46.605181	-1000	0	-1000	0
Actinomyces_bowdenii_OH5050	0	0	0	-1000	-35.016171	0	0	-916.66667	0
Actinomyces_cardiffensis_F0333	0	0	0	-555.5555556	-15.120941	0	0	-736.11205	0
Actinomyces_europaeus_ACS_120_V_Col10b	0	0	0	0	-29.108985	0	0	0	0
Actinomyces_europaeus_UMB0652_ERR1203664	0	0	0	0	-29.273618	0	0	-509.88416	0
Actinomyces_georgiae_DSM_6843	0	0	0	0	-14.91928	-1000	0	-829.91916	0
Actinomyces_gerencseriae_DSM_6844	0	0	0	-1000	-17.51153	0	0	-724.83221	0
Actinomyces_graevenitzii_C83	0	0	0	-32.67787547	-23.633468	0	0	-666.66667	0
Actinomyces_hongkongensis_HKU8	0	0	0	0	-16.540014	0	0	0	0
Actinomyces_israelii_DSM_43320	0	0	0	-1000	-18.583892	0	0	-922.81879	0
Actinomyces_johnsonii_F0510	0	0	0	-1000	-45.527396	0	0	-1000	0
Actinomyces_johnsonii_F0542	0	0	0	-1000	-37.614769	0	0	-1000	0
Actinomyces_massiliensis_4401292	0	0	0	-1000	-30.457288	0	0	-549.75556	0
Actinomyces_massiliensis_F0489	0	0	0	-1000	-29.427759	0	0	-547.77126	0
Actinomyces_meyeri_DSM_20733	0	0	0	0	-12.92372	0	0	-349.6035	0

Actinomyces_naeslundii_ERR2221356	0	0	0	-1000	-28.609703	0	0	-550.47521	0
Actinomyces_naeslundii_str_Howell_279	0	0	0	-1000	-39.151365	0	0	-1000	0
Actinomyces_odontolyticus_ATCC_17982	0	0	0	-36.73532068	-26.65744	0	0	-1000	0
Actinomyces_odontolyticus_F0309	0	0	0	-61.39348147	-22.997157	0	0	0	0
Actinomyces_oris_K20	0	0	0	0	-32.254	0	0	-1000	0
Actinomyces_oris_T14V	0	0	0	-1000	-21.60378	0	0	-875	0
Actinomyces_sp_HPA0247	0	0	0	-42.8764547	-23.95294	0	0	0	0
Actinomyces_sp_ICM39	0	0	0	-41.62399493	-23.252895	0	0	0	0
Actinomyces_sp_ICM47	0	0	0	-41.31872776	-23.953135	0	0	0	0
Actinomyces_timonensis_DSM_23838	0	0	0	-1000	-30.048295	-1000	0	-982.57373	0
Actinomyces_turicensis_ACS_279_V_Col4	0	0	0	-30.76150823	0	0	0	-991.82518	0
Actinomyces_urogenitalis_DSM_15434	0	0	0	0	-19.438544	0	0	-829.50368	0
Actinomyces_urogenitalis_S6_C4	0	0	0	-1000	-17.005328	0	0	-464.59913	0
Actinomyces_viscosus_C505	0	0	0	-1000	-34.116294	0	0	-1000	0
Acutalibacter_muris_KB18	0	0	0	0	-9.807382	0	0	-257.68558	0
Adlercreutzia_equolifaciens_DSM_19450	0	0	0	-508.1967213	-92.725819	0	0	0	0
Adlercreutzia_equolifaciens_ERR2221192	0	0	0	-34.49998415	-21.420002	0	0	0	0
Adlercreutzia_muris_DSM_29508	0	0	0	-10.88314198	-4.0684239	0	0	0	0
Advenella_mimigardefordensis_DPN7	0	0	0	-1000	-18.026325	0	0	0	0
Aerococcus_christensenii_CCUG28831	0	0	0	-1000	-10.993706	0	0	0	0
Aerococcus_viridans_ATCC_11563	0	0	0	-1000	-18.984315	-1000	0	-906.33953	0
Aerococcus_viridans_LL1	0	0	0	-42.0978668	-21.533153	-1000	0	-729.06404	0
Aeromicrobium_massiliense_JC14	0	0	0	-1000	-12.097982	0	0	0	0
Aeromonas_caviae_Ae398	0	0	0	-1000	-45.107939	-1000	0	-1000	0
Aeromonas_caviae_FDAARGOS_72	0	0	0	-1000	-42.364964	-1000	0	-1000	0
Aeromonas_caviae_FDAARGOS_75	0	0	0	-1000	-42.364963	-1000	0	-1000	0
Aeromonas_dhakensis_173	0	0	0	-1000	-55.870794	-1000	-1000	-1000	0
Aeromonas_dhakensis_277	0	0	0	-1000	-59.29928	-1000	-1000	-1000	0
Aeromonas_dhakensis_SSU	0	0	0	-1000	-55.870794	-1000	-1000	-1000	0
Aeromonas_hydrophila_116	0	0	0	-1000	-42.584612	-1000	0	-1000	0
Aeromonas_hydrophila_14	0	0	0	-1000	-55.422606	-1000	0	-1000	0
Aeromonas_hydrophila_187	0	0	0	-1000	-55.422606	-1000	0	-1000	0
Aeromonas_hydrophila_259	0	0	0	-1000	-55.422606	-1000	0	-1000	0
Aeromonas_hydrophila_AH10	0	0	0	-1000	-57.739988	-1000	0	-1000	0
Aeromonas_hydrophila_ML09_119	0	0	0	-1000	-55.503502	-1000	0	-1000	0
Aeromonas_hydrophila_RB_AH	0	0	0	-1000	-57.739988	-1000	0	-1000	0
Aeromonas_hydrophila_SNUFPC_A8	0	0	0	-1000	-55.468093	-1000	0	-1000	0
Aeromonas_hydrophila_subsp_hydrophila_ATCC_	0	0	0	-1000	-50.992483	-1000	0	-1000	0
Aeromonas_jandaei_Riv2	0	0	0	-1000	-63.509675	-1000	0	0	0
Aeromonas_media_ARB13	0	0	0	-1000	-42.791115	-1000	0	0	0
Aeromonas_media_WS	0	0	0	-1000	-57.760594	-1000	0	-1000	0
Aeromonas_veronii_AER39	0	0	0	-1000	-53.912176	-1000	0	-1000	0
Aeromonas_veronii_AER397	0	0	0	-1000	-40.452899	-1000	0	-1000	0
Aeromonas_veronii_AMC34	0	0	-1000	-1000	-53.964429	-1000	0	-1000	0
Aeromonas_veronii_AMC35	0	0	0	-1000	-40.452895	-1000	0	-1000	0
Aeromonas_veronii_AVNIH1	0	0	0	-1000	-43.333601	-1000	0	-1000	0
Aeromonas_veronii_B565	0	0	0	-1000	-62.255121	-1000	0	-1000	0
Aeromonas_veronii_TH0426	0	0	0	-1000	-43.330652	-1000	0	-1000	0
Afipia_birgiae_34632	0	0	0	0	-28.928337	-1000	0	-1000	0
Aggregatibacter_aphrophilus_ATCC_33389	0	0	0	-1000	-49.222693	0	0	-1000	0
Aggregatibacter_aphrophilus_F0387	0	0	0	-1000	-40.411301	0	0	-1000	0
Aggregatibacter_aphrophilus_NJ8700	0	0	0	-61.62464986	-39.057362	0	0	-1000	0
Aggregatibacter_aphrophilus_W10433	0	0	0	-1000	-37.288861	0	0	-822.78481	0
Aggregatibacter_segnis_933_AAPH_107_59761_2	0	0	0	-1000	-36.002027	0	0	-750	0
Aggregatibacter_segnis_ATCC_33393	0	0	0	-1000	-36.010766	0	0	-877.55102	0
Agrobacterium_fabrum_str_C58	0	0	0	-1000	-6.7850633	0	0	0	0
Agrobacterium_tumefaciens_CCNWGS0286	0	0	0	-1000	-20.851597	0	-1000	0	0
Agrobacterium_tumefaciens_F2	0	0	0	-1000	-36.089813	0	-1000	0	0
Agrobacterium_tumefaciens_str_Cherry_2E_2_2	0	0	0	-1000	-19.885335	0	-1000	0	0
Akkermansia_muciniphila_ATCC_BAA_835	0	0	0	-1000	0	0	0	0	0
Akkermansia_muciniphila_YL44	0	0	0	-1000	-21.824	0	0	0	0
Akkermansia_sp_KLE1797	0	0	0	-1000	-24.998036	0	0	0	0
Akkermansia_sp_KLE1798	0	0	0	-1000	-24.998036	0	0	0	0
Alcaligenes_faecalis_NBIB_017	0	0	0	-1000	-1000	0	0	0	0
Alcaligenes_faecalis_subsp_faecalis_NBRC_13111	0	0	0	-1000	-1000	0	0	0	0
Alcaligenes_faecalis_subsp_faecalis_NCIB_8687	0	0	0	-1000	-1000	0	0	0	0
Alcaligenes_faecalis_ZD02	0	0	0	-1000	-1000	0	0	0	0
Aliivibrio_fischeri_ES114	0	0	0	-1000	-45.011244	-1000	0	0	0
Aliivibrio_fischeri_MJ11	0	0	0	-1000	-46.643572	-1000	0	0	0
Aliivibrio_salmonicida_LFI1238	0	0	0	-1000	-36.450915	-1000	0	0	0
Alistipes_finegoldii_DSM_17242	0	0	0	-1000	0	-1000	0	0	0
Alistipes_ihumii_AP11	0	0	0	0	-11.766913	0	0	0	0
Alistipes_indistinctus_ERR2221377	0	0	-1000	-1000	-29.99574	-1000	0	-1000	0
Alistipes_indistinctus_YIT_12060	0	0	-1000	-1000	0	-1000	0	-1000	0
Alistipes_nov_ERR2221210	0	0	0	-166.6666667	-2.1587123	0	0	0	0
Alistipes_nov_ERR2221392	0	0	0	-204.8192771	-8.0724894	-609.7561	0	0	0
Alistipes_obesi_isolate_ph8	0	0	0	0	-10.706539	-1000	0	0	0
Alistipes_onderdonkii_DSM_19147	0	0	0	-1000	0	-1000	0	0	0
Alistipes_onderdonkii_ERR1022348	0	0	0	-1000	-32.368302	-1000	0	0	0
Alistipes_onderdonkii_ERR1022461	0	0	0	-1000	-32.631644	-1000	0	0	0
Alistipes_onderdonkii_ERR1203947	0	0	0	-1000	-34.191175	-1000	0	0	0
Alistipes_onderdonkii_ERR1204041	0	0	0	-1000	-34.191122	-1000	0	0	0
Alistipes_onderdonkii_ERR2221102	0	0	0	-1000	-32.632407	-1000	0	0	0
Alistipes_onderdonkii_ERR2230089	0	0	0	-1000	-32.368729	-1000	0	0	0
Alistipes_onderdonkii_ERR2230093	0	0	0	-1000	-32.63278	-1000	0	0	0
Alistipes_putredinis_DSM_17216	0	0	0	-1000	-1000	0	0	0	0
Alistipes_senegalensis_JC50	0	0	-1000	0	-31.461512	0	0	0	0
Alistipes_shahii_ERR2221103	0	0	-1000	-1000	-32.109	0	0	0	0
Alistipes_shahii_ERR2221307	0	0	-1000	-1000	-32.108993	0	0	0	0
Alistipes_shahii_ERR2221376	0	0	-1000	-1000	-33.676355	0	0	0	0
Alistipes_shahii_WAL_8301	0	0	0	-1000	0	0	0	0	0

Alistipes_sp_AL_1	0	0	0	-1000	-32.631275	-1000	0	0	0
Alistipes_sp_cv1_ERR1022444	0	0	-1000	-1000	-25.701547	0	0	-492.69481	0
Alistipes_sp_HGB5	0	0	0	-1000	-35.18427	-1000	0	0	0
Alistipes_timonensis_ERR2221390	0	0	-1000	-1000	-32.312538	0	0	0	0
Alistipes_timonensis_JC136	0	0	-1000	-1000	-14.949948	0	0	0	0
Alkalibaculum_bacchi_DSM_22112_Ga0244545_1	0	0	0	0	-14.24499	0	-1000	0	0
Alkaliphilus_transvaalensis_ATCC_700919	0	0	0	-29.88775106	-15.130035	0	0	0	0
Allobaculum_stercoricanis_DSM_13633	0	0	0	0	-13.763347	0	0	-277.51259	0
Alloiococcus_otitis_ATCC_51267	0	0	0	-13.37986614	-15.303432	0	0	0	0
Alloprevotella_rava_F0323	0	0	0	0	-25.935851	0	0	0	0
Alloprevotella_tannerae_ATCC_51259	0	0	0	-35.37208285	-1000	0	0	0	0
Alloscardovia_omnicolens_1036_GVAG_103_587	0	0	0	0	-26.828557	0	0	-555.44475	0
Alloscardovia_omnicolens_DSM_21503	0	0	0	0	-26.828544	0	0	-682.66979	0
Alloscardovia_omnicolens_F0580	0	0	0	0	-26.828551	0	0	-555.44475	0
Amazonia_massiliensis_MS4	0	0	0	0	-16.838542	0	0	0	0
Aminivibrio_pyruvatiphilus_DSM_25964	0	0	0	-1000	-1000	0	-1000	0	0
Anaerobacillus_macyae_DSM_16346	0	0	0	-49.91719014	-20.035958	-1000	0	0	0
Anaerobaculum_hydrogeniformans_OS1_ATCC_B	0	0	0	-1000	-921.05263	0	0	0	0
Anaerobiospirillum_succiniciproducens_DSM_64(	0	0	0	-1000	-26.615306	0	0	-570.79646	0
Anaerococcus_hydrogenalis_ACS_025_V_Sch4	0	0	0	-1000	-52.493997	-1000	0	-670.06235	0
Anaerococcus_hydrogenalis_DSM_7454	0	0	0	-1000	-26.641362	-599.57067	0	0	0
Anaerococcus_lactolyticus_ATCC_51172	0	0	0	-1000	-29.309425	0	0	0	0
Anaerococcus_lactolyticus_S7_1_13	0	0	0	-1000	-29.286331	0	0	0	0
Anaerococcus_obesiensis_ph10	0	0	0	-1000	-28.632203	0	0	0	0
Anaerococcus_prevotii_ACS_065_V_Col13	0	0	0	-24.92783784	-24.516617	0	0	-670.50024	0
Anaerococcus_prevotii_DSM_20548	0	0	0	-1000	-19.228986	0	0	-516.10472	0
Anaerococcus_senegalensis_JC48	0	0	0	-26.73790001	-17.15381	0	0	0	0
Anaerococcus_tetradius_ATCC_35098	0	0	-1000	-1000	-44.414199	0	0	0	0
Anaerococcus_tetradius_MJR8151	0	0	0	-1000	-43.181708	0	0	0	0
Anaerococcus_vaginalis_ATCC_51170	0	0	0	-1000	-23.041695	0	0	0	0
Anaerofustis_stercorihominis_DSM_17244	0	0	0	0	-205.80418	-666.66667	0	-333.33333	0
Anaeroglobus_geminatus_F0357	0	0	0	-23.37899813	-1000	0	0	0	0
Anaeroplasma_bactoclasticum_ATCC_27112	0	0	0	0	-24.78393	0	0	-254.1225	0
Anaerosalibacter_bizertensis_Med78_601_WT_4'	0	0	0	-1000	-15.323376	0	-1000	0	0
Anaerosalibacter_massiliensis_ND1	0	0	0	-24.22921417	-15.263632	-896.0396	0	0	0
Anaerosporobacter_mobilis_DSM_15930	0	0	0	-1000	-20.638315	0	0	-916.66667	0
Anaerostipes_caccae_DSM_14662	0	0	0	-54.0782955	0	-1000	0	-1000	0
Anaerostipes_caccae_ERR1203925	0	0	0	-39.80419575	-20.4687	-1000	0	-1000	0
Anaerostipes_caccae_ERR171259	0	0	0	-52.08554545	-26.893244	-1000	0	-1000	0
Anaerostipes_caccae_ERR2221104	0	0	0	-36.88161535	-19.055115	-1000	0	-1000	0
Anaerostipes_caccae_ERR2221232	0	0	0	-40.08951342	-20.685964	-1000	0	-1000	0
Anaerostipes_hadrus_DSM_3319	0	0	0	-1000	-203.61184	-1000	0	-629.03226	0
Anaerostipes_hadrus_ERR1022288	0	0	0	-41.58404333	-21.458378	-1000	0	-580.12821	0
Anaerostipes_hadrus_ERR1022326	0	0	0	-48.85353354	-25.223624	-1000	0	-580.12821	0
Anaerostipes_hadrus_ERR1022376	0	0	0	-53.36497619	-27.490962	-1000	0	-936.50794	0
Anaerostipes_hadrus_ERR1022422	0	0	0	-47.1934264	-24.364459	-1000	0	-580.12821	0
Anaerostipes_hadrus_ERR1022423	0	0	0	-1000	-25.709459	-1000	0	-604.16667	0
Anaerostipes_hadrus_ERR1022473	0	0	0	-47.19008649	-24.362734	-1000	0	-580.12821	0
Anaerostipes_hadrus_ERR2221197	0	0	0	-48.80663458	-25.199334	-1000	0	-580.12821	0
Anaerostipes_hadrus_ERR2221199	0	0	0	-50.91768432	-26.30025	-1000	0	-594.72817	0
Anaerostipes_nov_ERR2221209	0	0	0	-25.69994348	-13.198564	-1000	0	-612.27472	0
Anaerostipes_sp_3_2_56FAA	0	0	0	-1000	-24.879844	-1000	0	-780.95238	0
Anaerotignum_lactatifermentans_DSM_14214	0	0	0	-1000	-14.990025	-1000	-1000	0	0
Anaerotignum_propionicum_DSM_1682	0	0	0	0	-1000	0	0	0	0
Anaerotruncus_colihominis_DSM_17241	0	0	0	0	-21.676036	0	-1000	0	0
Anaerotruncus_colihominis_ERR1022453	0	0	0	0	-28.521973	0	-1000	0	0
Anaerotruncus_colihominis_ERR171260	0	0	0	0	-28.521991	0	-1000	0	0
Anaerotruncus_colihominis_ERR2221105	0	0	0	0	-28.521985	0	-1000	0	0
Anaerotruncus_nov_ERR2221396	0	0	0	-519.4489361	-16.687064	0	0	0	0
Anaerotruncus_sp_G3_2012	0	0	-503.2575098	-965.5172414	-15.034592	0	0	0	0
Anaerovorax_odorimutans_DSM_5092	0	0	0	0	-30.654038	0	0	0	0
Aneurinibacillus_aneurinilyticus_ATCC_12856	0	0	0	-1000	-31.658095	0	0	0	0
Arcanobacterium_haemolyticum_DSM_20595	0	0	0	-40.59323419	-32.68004	0	0	0	0
Arcobacter_butzleri_7h1h	0	0	0	-1000	-16.203368	0	0	0	0
Arcobacter_butzleri_ED_1	0	0	0	-35.37763267	-13.220237	0	0	0	0
Arcobacter_butzleri_JV22	0	0	0	-10.39671982	-4.5942646	0	0	0	0
Arcobacter_butzleri_RM4018	0	0	0	-1000	0	0	0	0	0
Ardenticatena_maritima_110S	0	0	0	-1000	-10.514126	0	-1000	-390.86294	0
Arthrobacter_castelli_DSM_16402	0	0	0	-1000	-29.333755	0	0	0	0
Atopobium_minutum_10063974	0	0	0	0	-18.509119	0	0	0	0
Atopobium_parvulum_DSM_20469	0	0	0	-21.42226878	-18.831072	0	0	-842.91436	0
Atopobium_rimae_ATCC_49626	0	0	0	-33.36878836	-32.777687	0	0	-1000	0
Atopobium_vaginae_CMW7778A	0	0	0	0	-25.192234	0	0	0	0
Atopobium_vaginae_DSM_15829	0	0	0	0	-25.774383	0	0	0	0
Atopobium_vaginae_PB189_T1_4	0	0	0	0	-25.199241	0	0	0	0
Auritidibacter_ignavus_IMMIB_L_1656	0	0	0	-1000	-1000	0	0	0	0
Bacillus_altitudinis_41KF2b	0	0	0	-1000	-23.716635	-1000	0	-1000	0
Bacillus_altitudinis_S_1	0	0	0	-1000	-35.139253	-1000	0	0	0
Bacillus_amyloliquefaciens_DSM7	0	0	0	-1000	-29.530524	-1000	0	-1000	0
Bacillus_amyloliquefaciens_FZB42	0	0	-1000	-1000	-28.643585	-1000	0	-1000	0
Bacillus_amyloliquefaciens_LL3	0	0	0	-1000	-26.73575	-1000	0	-923.07692	0
Bacillus_amyloliquefaciens_subsp_amyloliquefaci	0	0	-1000	-1000	-27.063487	-1000	0	-1000	0
Bacillus_amyloliquefaciens_TA208	0	0	0	-1000	-27.126677	-1000	0	-1000	0
Bacillus_amyloliquefaciens_XH7	0	0	0	-1000	-27.126677	-1000	0	-1000	0
Bacillus_amyloliquefaciens_Y2	0	0	-1000	-1000	-27.713691	-1000	0	-923.07692	0
Bacillus_andreraoultii_KW_12	0	0	0	-1000	-17.752222	0	0	-1000	0
Bacillus_atrophaeus_1013_1	0	0	0	-1000	-44.327964	-1000	0	-1000	0
Bacillus_atrophaeus_1013_2	0	0	0	-1000	-44.327964	-1000	0	-1000	0
Bacillus_atrophaeus_1942	0	0	0	-1000	-44.327995	-1000	0	-1000	0
Bacillus_atrophaeus_ATCC_49822_1	0	0	0	-1000	-35.532642	-1000	0	-1000	0
Bacillus_atrophaeus_ATCC_49822_2	0	0	0	-1000	-44.327964	-1000	0	-1000	0

Bacillus_atrophaeus_ATCC_9372_1	0	0	0	-1000	-37.574727	-1000	0	-1000	0
Bacillus_atrophaeus_ATCC_9372_2	0	0	0	-1000	-37.577315	-1000	0	-1000	0
Bacillus_atrophaeus_BACI051_E	0	0	0	-1000	-37.577315	-1000	0	-1000	0
Bacillus_atrophaeus_BACI051_N	0	0	0	-1000	-37.591557	-1000	0	-1000	0
Bacillus_atrophaeus_C89	0	0	0	-1000	-37.577315	-1000	0	-1000	0
Bacillus_atrophaeus_Detrick_1	0	0	0	-1000	-37.577315	-1000	0	-1000	0
Bacillus_atrophaeus_Detrick_2	0	0	0	-1000	-37.577315	-1000	0	-1000	0
Bacillus_atrophaeus_Detrick_3	0	0	0	-1000	-37.577315	-1000	0	-1000	0
Bacillus_atrophaeus_DJHJ8	0	0	0	-1000	-36.290092	-1000	0	-923.07692	0
Bacillus_atrophaeus_str_Dugway	0	0	0	-1000	-37.574727	-1000	0	-1000	0
Bacillus_atrophaeus_UCMB_5137	0	0	0	-1000	-46.115001	-1000	0	-1000	0
Bacillus_cereus_03BB102	-1000	0	0	-1000	-44.829257	0	0	0	0
Bacillus_cereus_03BB108	0	0	0	-1000	-55.256999	0	0	0	0
Bacillus_cereus_172560W	0	0	0	-1000	-54.159929	0	0	0	0
Bacillus_cereus_95	0	0	0	-1000	-52.483593	0	0	0	0
Bacillus_cereus_AH1134	0	0	0	-1000	-54.159967	0	0	0	0
Bacillus_cereus_AH1271	0	0	0	-1000	-44.267612	0	0	0	0
Bacillus_cereus_AH1272	0	0	0	-1000	-54.445338	0	0	0	0
Bacillus_cereus_AH1273	0	0	0	-1000	-32.681614	0	0	0	0
Bacillus_cereus_AH187_F4810_72	-1000	0	0	-1000	-41.015298	0	0	0	0
Bacillus_cereus_AH676	0	0	0	-1000	-54.754806	0	0	0	0
Bacillus_cereus_AH820	-1000	0	0	-1000	-52.420747	0	0	0	0
Bacillus_cereus_AND1407	0	0	0	-1000	-44.718014	0	0	0	0
Bacillus_cereus_ATCC_10876	0	0	0	-1000	-54.159967	0	0	0	0
Bacillus_cereus_ATCC_10987	0	0	0	-1000	-38.655822	0	0	0	0
Bacillus_cereus_ATCC_14579	-1000	0	0	-1000	-40.882076	0	0	0	0
Bacillus_cereus_ATCC_4342	0	0	0	-1000	-35.846935	0	0	0	0
Bacillus_cereus_B4264	-1000	0	0	-1000	-32.402936	0	0	0	0
Bacillus_cereus_B5_2	0	0	0	-1000	-35.471783	0	0	0	0
Bacillus_cereus_BAG1O_1	0	0	0	-1000	-37.143991	0	0	0	0
Bacillus_cereus_BAG1O_3	0	0	0	-1000	-35.852326	0	0	0	0
Bacillus_cereus_BAG1X1_1	0	0	0	-1000	-36.448111	0	0	0	0
Bacillus_cereus_BAG1X1_2	0	0	0	-1000	-34.458074	0	0	0	0
Bacillus_cereus_BAG1X1_3	0	0	0	-1000	-37.143991	0	0	0	0
Bacillus_cereus_BAG1X2_1	0	0	0	-1000	-36.448113	0	0	0	0
Bacillus_cereus_BAG1X2_2	0	0	0	-1000	-36.44809	0	0	0	0
Bacillus_cereus_BAG1X2_3	0	0	0	-1000	-36.448099	0	0	0	0
Bacillus_cereus_BAG2O_1	0	0	0	-1000	-36.448102	0	0	0	0
Bacillus_cereus_BAG2O_2	0	0	0	-1000	-35.270136	0	0	0	0
Bacillus_cereus_BAG2O_3	0	0	0	-1000	-35.471786	0	0	0	0
Bacillus_cereus_BAG2X1_1	0	0	0	-1000	-35.142888	0	0	0	0
Bacillus_cereus_BAG2X1_3	0	0	0	-1000	-36.257038	0	0	0	0
Bacillus_cereus_BAG3O_1	0	0	0	-1000	-35.436489	0	0	0	0
Bacillus_cereus_BAG3O_2	0	0	0	-1000	-33.781611	0	0	0	0
Bacillus_cereus_BAG3X2_1	0	0	0	-1000	-35.247269	0	0	0	0
Bacillus_cereus_BAG3X2_2	0	0	0	-1000	-37.619804	0	0	0	0
Bacillus_cereus_BAG4O_1	0	0	0	-1000	-33.773926	0	0	0	0
Bacillus_cereus_BAG4X12_1	0	0	0	-1000	-34.906605	0	0	0	0
Bacillus_cereus_BAG4X2_1	0	0	0	-1000	-35.293167	0	0	0	0
Bacillus_cereus_BAG5O_1	0	0	0	-1000	-36.24744	0	0	0	0
Bacillus_cereus_BAG5X1_1	0	0	0	-1000	-34.506282	0	0	0	0
Bacillus_cereus_BAG5X12_1	0	0	0	-1000	-34.906603	0	0	0	0
Bacillus_cereus_BAG6O_1	0	0	0	-1000	-36.265654	0	0	0	0
Bacillus_cereus_BAG6O_2	0	0	0	-1000	-35.529406	0	0	0	0
Bacillus_cereus_BAG6X1_2	0	0	0	-1000	-34.44102	0	0	0	0
Bacillus_cereus_BDRD_Cer4	0	0	0	-1000	-30.747784	0	0	0	0
Bacillus_cereus_BDRD_ST196	0	0	0	-1000	-36.132485	0	0	0	0
Bacillus_cereus_BDRD_ST24	0	0	0	-1000	-31.379552	0	0	0	0
Bacillus_cereus_BDRD_ST26	0	0	0	-1000	-1000	0	0	0	0
Bacillus_cereus_BGSC_6E1	0	0	0	-1000	-33.822604	0	0	0	0
Bacillus_cereus_biovar_anthracis_str_CI	0	0	0	-1000	-31.851833	0	0	0	0
Bacillus_cereus_BMG1_7	0	0	0	-1000	-35.713997	0	0	0	0
Bacillus_cereus_E33L	-1000	0	0	-1000	-37.785938	0	0	0	0
Bacillus_cereus_F	0	0	0	-1000	-41.990278	0	0	0	0
Bacillus_cereus_F65185	0	0	0	-1000	-34.909288	0	0	0	0
Bacillus_cereus_F837	0	0	0	-1000	-41.816272	0	0	0	0
Bacillus_cereus_FORC_005	0	0	0	-1000	-52.297607	0	0	0	0
Bacillus_cereus_FRI_35	0	0	0	-1000	-36.615704	0	0	0	0
Bacillus_cereus_G9241	0	0	0	-1000	-40.518119	0	0	0	0
Bacillus_cereus_G9842	-1000	0	0	-1000	-43.762359	0	0	0	0
Bacillus_cereus_H3081_97	0	0	0	-1000	-37.570912	0	0	0	0
Bacillus_cereus_HD73	0	0	0	-1000	-34.906598	0	0	0	0
Bacillus_cereus_HuA2_1	0	0	0	-1000	-37.2033	0	0	0	0
Bacillus_cereus_HuA2_3	0	0	0	-1000	-34.3323	0	0	0	0
Bacillus_cereus_HuA2_4	0	0	0	-1000	-34.665949	0	0	0	0
Bacillus_cereus_HuA2_9	0	0	0	-1000	-37.743962	0	0	0	0
Bacillus_cereus_HuA3_9	0	0	0	-1000	-37.764122	0	0	0	0
Bacillus_cereus_HuA4_10	0	0	0	-1000	-37.481983	0	0	0	0
Bacillus_cereus_HuB1_1	0	0	0	-1000	-34.45809	0	0	0	0
Bacillus_cereus_HuB13_1	0	0	0	-1000	-35.714006	0	0	0	0
Bacillus_cereus_HuB2_9	0	0	0	-1000	-35.193791	0	0	0	0
Bacillus_cereus_HuB4_4	0	0	0	-1000	-33.813738	0	0	0	0
Bacillus_cereus_HuB5_5	0	0	0	-1000	-34.272009	0	0	0	0
Bacillus_cereus_IS075	0	0	0	-1000	-34.996602	0	0	0	0
Bacillus_cereus_IS195	0	0	0	-1000	-34.442489	0	0	0	0
Bacillus_cereus_IS845_00	0	0	0	-1000	-34.442489	0	0	0	0
Bacillus_cereus_ISP2954	0	0	0	-1000	-35.713992	0	0	0	0
Bacillus_cereus_ISP3191	0	0	0	-1000	-32.907595	0	0	0	0
Bacillus_cereus_K_5975c	0	0	0	-1000	-34.396645	0	0	0	0
Bacillus_cereus_LCT_BC244	0	0	0	-1000	-34.445624	0	0	0	0
Bacillus_cereus_m1293	0	0	0	-1000	-34.470484	0	0	0	0

Bacillus_cereus_m1550	0	0	0	-1000	-35.657647	0	0	0	0
Bacillus_cereus_MC118	0	0	0	-1000	-34.373793	0	0	0	0
Bacillus_cereus_MC67	0	0	0	-1000	-34.373651	0	0	0	0
Bacillus_cereus_MSX_A1	0	0	0	-1000	-34.188756	0	0	0	0
Bacillus_cereus_MSX_A12	0	0	0	-1000	-34.4425	0	0	0	0
Bacillus_cereus_MSX_D12	0	0	0	-1000	-35.031339	0	0	0	0
Bacillus_cereus_NC7401	-1000	0	0	-1000	-34.442446	0	0	0	0
Bacillus_cereus_NVH0597_99	0	0	0	-1000	-40.224423	0	0	0	0
Bacillus_cereus_Q1	-1000	0	0	-1000	-34.442446	0	0	0	0
Bacillus_cereus_R309803	0	0	0	-1000	-35.549033	0	0	0	0
Bacillus_cereus_Rock1_15	0	0	0	-1000	-31.595582	0	0	0	0
Bacillus_cereus_Rock1_3	0	0	0	-1000	-35.193953	0	0	0	0
Bacillus_cereus_Rock3_28	0	0	0	-1000	-51.698594	0	0	0	0
Bacillus_cereus_Rock3_29	0	0	0	-1000	-35.118325	0	0	0	0
Bacillus_cereus_Rock3_42	0	0	0	-1000	-35.164639	0	0	0	0
Bacillus_cereus_Rock3_44	0	0	0	-1000	-41.285872	0	0	0	0
Bacillus_cereus_Rock4_18	0	0	0	-1000	-35.137408	0	0	0	0
Bacillus_cereus_Rock4_2	0	0	0	-1000	-47.131155	0	0	0	0
Bacillus_cereus_SJ1	0	0	0	-1000	-32.670791	0	0	0	0
Bacillus_cereus_str_Schrouff	0	0	0	-1000	-34.396633	0	0	0	0
Bacillus_cereus_subsp_cytotoxis_NVH_391_98	0	0	0	-1000	-35.00395	0	0	0	0
Bacillus_cereus_TIAC219	0	0	0	-1000	-35.059766	0	0	0	0
Bacillus_cereus_VD014	0	0	0	-1000	-34.188745	0	0	0	0
Bacillus_cereus_VD021	0	0	0	-1000	-35.099015	0	0	0	0
Bacillus_cereus_VD022	0	0	0	-1000	-35.059702	0	0	0	0
Bacillus_cereus_VD045	0	0	0	-1000	-33.206188	0	0	0	0
Bacillus_cereus_VD048	0	0	0	-1000	-35.881642	0	0	0	0
Bacillus_cereus_VD102	0	0	0	-1000	-34.372615	0	0	0	0
Bacillus_cereus_VD107	0	0	0	-1000	-34.242113	0	0	0	0
Bacillus_cereus_VD115	0	0	0	-1000	-37.251112	0	0	0	0
Bacillus_cereus_VD118	0	0	0	-1000	-35.38511	0	0	0	0
Bacillus_cereus_VD131	0	0	0	-1000	-36.736519	0	0	0	0
Bacillus_cereus_VD133	0	0	0	-1000	-35.483915	0	0	0	0
Bacillus_cereus_VD136	0	0	0	-1000	-33.640222	0	0	0	0
Bacillus_cereus_VD140	0	0	0	-1000	-35.297132	0	0	0	0
Bacillus_cereus_VD142	0	0	0	-1000	-36.399774	0	0	0	0
Bacillus_cereus_VD146	0	0	0	-1000	-37.036252	0	0	0	0
Bacillus_cereus_VD148	0	0	0	-1000	-34.332304	0	0	0	0
Bacillus_cereus_VD154	0	0	0	-1000	-32.750618	0	0	0	0
Bacillus_cereus_VD156	0	0	0	-1000	-33.909537	0	0	0	0
Bacillus_cereus_VD166	0	0	0	-1000	-31.381059	0	0	0	0
Bacillus_cereus_VD169	0	0	0	-1000	-31.379535	0	0	0	0
Bacillus_cereus_VD184	0	0	0	-1000	-34.321479	0	0	0	0
Bacillus_cereus_VD196	0	0	0	-1000	-34.697666	0	0	0	0
Bacillus_cereus_VD200	0	0	0	-1000	-32.446298	0	0	0	0
Bacillus_cereus_VD214	0	0	0	-1000	-35.193781	0	0	0	0
Bacillus_cereus_VDM006	0	0	0	-1000	-36.479538	0	0	0	0
Bacillus_cereus_VDM021	0	0	0	-1000	-36.479554	0	0	0	0
Bacillus_cereus_VDM034	0	0	0	-1000	-35.035889	0	0	0	0
Bacillus_cereus_VDM053	0	0	0	-1000	-36.337896	0	0	0	0
Bacillus_cereus_VDM062	0	0	0	-1000	-35.035888	0	0	0	0
Bacillus_cereus_W	0	0	0	-1000	-42.128214	0	0	0	0
Bacillus_clausii_KSM_K16	0	0	0	-1000	-22.917106	-1000	0	-1000	0
Bacillus_endophyticus_2102	0	0	-1000	-1000	-29.976741	-1000	0	-1000	0
Bacillus_firmus_DS1	0	0	0	-1000	-32.634188	-1000	0	0	0
Bacillus_fordii_DSM_16014	0	0	-1000	-1000	-25.556311	0	0	0	0
Bacillus_halodurans_C_125	0	0	0	-1000	0	-1000	0	-1000	0
Bacillus_halotolerans_RRC_101	0	0	-1000	-1000	-34.340913	-1000	0	-1000	0
Bacillus_infantis_NRRL_B_14911	0	0	0	-1000	-32.660218	-1000	0	-1000	0
Bacillus_kwashiorkori_SIT6	0	0	0	-9.887751939	-17.212738	-1000	0	-568.85764	0
Bacillus_licheniformis_5NAP23	0	0	-1000	-1000	-43.374601	-1000	0	-1000	0
Bacillus_licheniformis_ATCC_14580	0	0	-1000	-1000	-33.948926	-1000	0	-1000	0
Bacillus_licheniformis_ERR2221106	0	0	-1000	-1000	-44.211642	-1000	0	-1000	0
Bacillus_licheniformis_ERR2230107	0	0	-1000	-1000	-44.15018	-1000	0	-1000	0
Bacillus_licheniformis_ERR2230161	0	0	-1000	-1000	-44.150177	-1000	0	-1000	0
Bacillus_licheniformis_HRBL_15TDI7	0	0	-1000	-1000	-44.202865	-1000	0	-1000	0
Bacillus_massilioanorexius_AP8	0	0	0	-1000	-24.665616	-1000	0	-1000	0
Bacillus_massiliosenegalensis_JC6	0	0	0	-1000	-23.675673	-1000	0	0	0
Bacillus_megaterium_27Col1_1E	0	0	0	-1000	-24.650802	-1000	0	-1000	0
Bacillus_megaterium_DSM319	-1000	0	0	-1000	-31.604662	-1000	0	-1000	0
Bacillus_megaterium_NBRC_15308_ATCC_14581	0	0	0	-1000	-26.457435	-1000	0	-1000	0
Bacillus_megaterium_NCT_2	0	0	0	-1000	-28.068873	-1000	0	-1000	0
Bacillus_megaterium_QM_B1551	-13.52386055	0	0	-1000	-30.653396	-1000	0	-1000	0
Bacillus_megaterium_SF185	0	0	0	-1000	-46.56748	-1000	0	-1000	0
Bacillus_megaterium_WSH_002	-1000	0	0	-1000	-30.19598	-1000	0	-1000	0
Bacillus_mojavensis_RO_H_1	0	0	-1000	-1000	-35.480357	-1000	0	-1000	0
Bacillus_mycoides_AH603	0	0	0	-1000	-44.600041	0	0	0	0
Bacillus_mycoides_AH621	0	0	0	-1000	-52.738927	0	0	0	0
Bacillus_mycoides_BHP	0	0	0	-1000	-51.996025	0	0	0	0
Bacillus_mycoides_BtB2_4	0	0	0	-1000	-43.314407	0	0	0	0
Bacillus_mycoides_CER057	0	0	0	-1000	-35.88474	0	0	0	0
Bacillus_mycoides_CER074	0	0	0	-1000	-35.884764	0	0	0	0
Bacillus_mycoides_DSM_2048	0	0	0	-1000	-41.990357	0	0	-1000	0
Bacillus_mycoides_VD078	0	0	0	-1000	-35.78629	0	0	0	0
Bacillus_mycoides_VDM019	0	0	0	-1000	-35.773484	0	0	0	0
Bacillus_mycoides_VDM022	0	0	0	-1000	-35.909925	0	0	0	0
Bacillus_nealsonii_AAU1	0	-1000	0	-1000	-17.997099	-1000	0	-1000	0
Bacillus_nov_ERR2221302	0	0	0	-1000	-36.259277	0	0	0	0
Bacillus_paralicheniformis_ATCC_9945a	0	0	-1000	-1000	-25.807665	-1000	0	0	0
Bacillus_pseudofirmus_OF4	0	0	0	-1000	-23.761645	-1000	0	-1000	0
Bacillus_pseudomycoides_219298	0	0	0	-1000	-36.229671	0	0	0	0

Bacillus_pseudomycoides_Rock1_4	0	0	0	-1000	-42.238265	0	0	-1000	0
Bacillus_pseudomycoides_Rock3_17	0	0	0	-1000	-43.473356	0	0	-1000	0
Bacillus_pumilus_ATCC_7061	0	0	0	-1000	-29.404864	-1000	0	-1000	0
Bacillus_pumilus_SAFR_032	0	0	0	-1000	-28.097306	-1000	0	-1000	0
Bacillus_pumilus_TUAT1	0	0	0	-1000	-34.738952	-1000	0	-1000	0
Bacillus_rubiinfantis_mt2	0	0	0	-1000	-21.43254	0	0	0	0
Bacillus_simplex_ERR2221243	0	0	-1000	-1000	-29.429711	-1000	0	-833.33333	0
Bacillus_smithii_7_3_47FAA	0	0	0	-73.35761958	-15.540577	0	0	0	0
Bacillus_sonorensis_L12	0	0	-1000	-1000	-32.358295	-1000	0	-1000	0
Bacillus_sp_7_6_55CFAA_CT2	0	0	0	-1000	-35.655357	0	0	0	0
Bacillus_subtilis_B_1	0	0	-1000	-1000	-34.979157	-1000	0	-1000	0
Bacillus_subtilis_BSn5	0	0	-1000	-1000	-31.700872	-1000	0	-1000	0
Bacillus_subtilis_ERR2221132	0	0	-1000	-1000	-36.401083	-1000	0	-1000	0
Bacillus_subtilis_QB928	0	0	-1000	-1000	-33.480083	-1000	0	-1000	0
Bacillus_subtilis_str_168	0	0	-1000	-1000	-41.353756	-1000	0	-1000	0
Bacillus_subtilis_subsp_natto_BEST195	0	0	-1000	-1000	-29.808699	-1000	0	-1000	0
Bacillus_subtilis_subsp_spizizenii_ATCC_6633	0	0	-1000	-1000	-29.782264	-1000	0	-1000	0
Bacillus_subtilis_subsp_spizizenii_DV1_B_1	0	0	-1000	-1000	-27.708363	-1000	0	-1000	0
Bacillus_subtilis_subsp_spizizenii_str_W23	0	0	-1000	-1000	-29.782263	-1000	0	-1000	0
Bacillus_subtilis_subsp_spizizenii_TU_B_10	0	0	-1000	-1000	-31.732781	-1000	0	-1000	0
Bacillus_subtilis_subsp_subtilis_516_BAMY_102_	0	0	-1000	-1000	-26.805537	-1000	0	-1000	0
Bacillus_subtilis_subsp_subtilis_6051_HGW	0	0	-1000	-1000	-33.208112	-1000	0	-1000	0
Bacillus_subtilis_subsp_subtilis_str_BAB_1	0	0	-1000	-1000	-31.700869	-1000	0	-1000	0
Bacillus_subtilis_subsp_subtilis_str_BSP1	0	0	-1000	-1000	-31.824743	-1000	0	-1000	0
Bacillus_subtilis_subsp_subtilis_str_JH642	0	0	-1000	-1000	-32.361895	0	0	-1000	0
Bacillus_subtilis_subsp_subtilis_str_NCIB_3610	0	0	-1000	-1000	-32.36473	0	0	-1000	0
Bacillus_subtilis_subsp_subtilis_str_RO_NN_1	0	0	-1000	-1000	-26.80431	-1000	0	-1000	0
Bacillus_subtilis_subsp_subtilis_str_SMY	0	0	-1000	-1000	-32.362202	0	0	-1000	0
Bacillus_thermoamylovorans_1A1	0	0	0	-1000	-26.660354	0	0	-1000	0
Bacillus_thuringiensis_BMB171	0	0	0	-1000	-28.945896	0	0	0	0
Bacillus_thuringiensis_Bt407	-8.939038979	0	0	-1000	-29.153718	0	0	0	0
Bacillus_thuringiensis_ERR2230164	0	0	0	-1000	-36.259279	0	0	0	0
Bacillus_thuringiensis_HD_771	-16.92749732	0	0	-1000	-29.392095	0	0	0	0
Bacillus_thuringiensis_HD_789	-1000	0	0	-1000	-29.633347	0	0	0	0
Bacillus_thuringiensis_HD571	0	0	0	-1000	-36.339536	0	0	0	0
Bacillus_thuringiensis_IBL_200	0	0	0	-1000	-31.100426	0	0	0	0
Bacillus_thuringiensis_IBL_4222	0	0	0	-1000	-29.52177	0	0	0	0
Bacillus_thuringiensis_MC28	-17.37633251	0	0	-1000	-30.459111	0	0	0	0
Bacillus_thuringiensis_serovar_andalousiensis_BG	0	0	0	-1000	-28.249435	0	0	0	0
Bacillus_thuringiensis_serovar_berliner_ATCC_10	0	0	0	-1000	-26.326979	0	0	0	0
Bacillus_thuringiensis_serovar_chinensis_CT_43	-16.28908836	0	0	-1000	-28.963103	0	0	0	0
Bacillus_thuringiensis_serovar_finitimus_YBT_020	-17.06578552	0	0	-1000	-29.852171	0	0	0	0
Bacillus_thuringiensis_serovar_huazhongensis_BG	0	0	0	-1000	-29.206986	0	0	0	0
Bacillus_thuringiensis_serovar_konkukian_str_97	0	0	0	-1000	-27.850442	0	0	0	0
Bacillus_thuringiensis_serovar_kurstaki_str_HD73	0	0	0	-1000	-31.092022	0	0	0	0
Bacillus_thuringiensis_serovar_kurstaki_str_T03a	0	0	0	-1000	-31.112714	0	0	0	0
Bacillus_thuringiensis_serovar_monterrey_BGSC_	0	0	0	-1000	-30.864485	0	0	0	0
Bacillus_thuringiensis_serovar_pakistani_str_T13	0	0	0	-1000	-28.142916	0	0	0	0
Bacillus_thuringiensis_serovar_pondicheriensis_B	0	0	0	-1000	-28.377241	0	0	0	0
Bacillus_thuringiensis_serovar_pulsiensis_BGSC_4	0	0	0	-1000	-27.809695	0	0	0	0
Bacillus_thuringiensis_serovar_thuringiensis_str_	-16.92718804	0	0	-1000	-29.395068	0	0	0	0
Bacillus_thuringiensis_serovar_thuringiensis_str_	0	0	0	-1000	-36.764179	0	0	-1000	0
Bacillus_thuringiensis_serovar_tochigiensis_BGSC	0	0	0	-1000	-31.631666	0	0	0	0
Bacillus_thuringiensis_str_Al_Hakam	-1000	0	0	-1000	-30.475095	0	0	0	0
Bacillus_timonensis_10403023	0	0	0	-1000	-33.495902	0	0	-1000	0
Bacillus_toyonensis_BAG1O_2	0	0	0	-1000	-43.717499	0	0	-1000	0
Bacillus_toyonensis_HuB4_10	0	0	0	-1000	-43.525348	0	0	-1000	0
Bacillus_vallismortis_DV1_F_3	0	0	-1000	-1000	-29.807672	-1000	0	-1000	0
Bacillus_velezensis_AS433	0	0	-1000	-1000	-34.860765	-1000	0	-1000	0
Bacillus_velezensis_L_H15	0	0	-1000	-1000	-34.860784	-1000	0	-875	0
Bacillus_velezensis_UCMB5036	0	0	-1000	-1000	-34.860769	-1000	0	-1000	0
Bacillus_wiedmannii_BAG2X1_2	0	0	0	-1000	-43.183509	0	0	0	0
Bacillus_wiedmannii_BAG5X2_1	0	0	0	-1000	-43.37275	0	0	0	0
Bacillus_wiedmannii_BAG6X1_1	0	0	0	-1000	-43.183368	0	0	0	0
Bacillus_wiedmannii_MM3	0	0	0	-1000	-34.102821	0	0	0	0
Bacteroidales_nov_ERR2221200	0	0	-1000	0	-27.687463	0	0	0	0
Bacteroides_acidifaciens_1e8A	0	0	-1000	0	-28.764148	0	0	0	0
Bacteroides_acidifaciens_ERR2221207	0	0	-1000	0	-25.494536	0	0	0	0
Bacteroides_acidifaciens_ERR2221208	0	0	-1000	0	-26.395809	0	0	0	0
Bacteroides_acidifaciens_ERR2221211	0	0	-1000	0	-25.124567	0	0	0	0
Bacteroides_acidifaciens_JCM_10556	0	0	-1000	0	-25.857435	0	0	0	0
Bacteroides_barnesiae_DSM_18169_JCM_13652	0	0	0	0	-1000	0	0	-284.31328	0
Bacteroides_caccae_ATCC_43185	0	0	-1000	0	-1000	0	0	-1000	0
Bacteroides_caccae_CL03T12C61	0	0	-1000	0	-1000	0	0	-1000	0
Bacteroides_caccae_ERR1022396	0	0	-1000	0	-1000	0	0	-1000	0
Bacteroides_caccae_ERR1022460	0	0	-1000	0	-1000	0	0	-1000	0
Bacteroides_caccae_ERR2221107	0	0	-1000	0	-1000	0	0	-1000	0
Bacteroides_caccae_ERR2221357	0	0	-1000	0	-1000	0	0	-1000	0
Bacteroides_caecimuris_I48	0	0	-1000	0	-1000	0	0	0	0
Bacteroides_cellulosilyticus_CL02T12C19	0	0	-1000	0	-1000	0	0	-1000	0
Bacteroides_cellulosilyticus_DSM_14838	0	0	-1000	0	-1000	0	0	-1000	0
Bacteroides_cellulosilyticus_ERR1022414	0	0	-1000	0	-1000	0	0	-1000	0
Bacteroides_cellulosilyticus_ERR2221108	0	0	-1000	0	-1000	0	0	-1000	0
Bacteroides_cellulosilyticus_ERR2221262	0	0	-1000	0	-1000	0	0	-1000	0
Bacteroides_cellulosilyticus_ERR2221326	0	0	-1000	0	-1000	0	0	-1000	0
Bacteroides_cellulosilyticus_ERR2230100	0	0	-1000	0	-1000	0	0	-1000	0
Bacteroides_cellulosilyticus_WH2	0	0	-1000	0	-1000	0	0	-1000	0
Bacteroides_clarus_ERR2221196	0	0	-1000	0	-1000	0	0	-761.25609	0
Bacteroides_clarus_ERR2221286	0	0	-1000	0	-1000	0	0	-761.25609	0
Bacteroides_clarus_ERR2230074	0	0	-1000	0	-1000	0	0	-761.25609	0
Bacteroides_clarus_YIT_12056	0	0	-1000	0	-1000	0	0	-1000	0

Bacteroides_coprocola_M16_DSM_17136	0	0	0	0	-1000	0	0	-547.2827	0
Bacteroides_coprophilus_DSM_18228	0	0	0	0	-1000	0	0	-389.77778	0
Bacteroides_coprosuis_DSM_18011	0	0	0	0	-1000	0	0	0	0
Bacteroides_dorei_CL02T00C15	0	0	-1000	-1000	-1000	0	0	-1000	0
Bacteroides_dorei_CL02T12C06	0	0	-1000	-1000	-1000	0	0	-1000	0
Bacteroides_dorei_CL03T12C01	0	0	-1000	-1000	-1000	0	0	-1000	0
Bacteroides_dorei_DSM_17855	0	0	-1000	-1000	-1000	0	0	-802.12702	0
Bacteroides_dorei_ERR2221252	0	0	-1000	-1000	-1000	0	0	-813.75358	0
Bacteroides_dorei_ERR2230082	0	0	-1000	-1000	-1000	0	0	-813.75358	0
Bacteroides_eggerthii_1_2_48FAA	0	0	-1000	0	-1000	0	0	0	0
Bacteroides_eggerthii_DSM_20697	0	0	-1000	0	-1000	0	0	0	0
Bacteroides_faecis_ERR1022362	0	0	-1000	0	-1000	0	0	-750	0
Bacteroides_faecis_ERR1203949	0	0	-1000	0	-1000	0	0	-750	0
Bacteroides_faecis_ERR1204043	0	0	-1000	0	-1000	0	0	-750	0
Bacteroides_faecis_ERR2221133	0	0	-1000	0	-1000	0	0	-750	0
Bacteroides_faecis_MAJ27	0	0	-1000	0	-1000	0	0	-1000	0
Bacteroides_finegoldii_DSM_17565	0	0	-1000	0	-1000	0	0	-988.81432	0
Bacteroides_finegoldii_ERR1022298	0	0	-1000	0	-1000	0	0	-680.55556	0
Bacteroides_finegoldii_ERR1022319	0	0	-1000	0	-1000	0	0	-666.66667	0
Bacteroides_fluxus_YIT_12057	0	0	-1000	0	-1000	-1000	0	-1000	0
Bacteroides_fragilis_3_1_12	0	0	-1000	0	-1000	0	0	-1000	0
Bacteroides_fragilis_638R	0	0	-1000	-1000	-1000	0	0	-1000	0
Bacteroides_fragilis_BOB25	0	0	-1000	0	-1000	0	0	-1000	0
Bacteroides_fragilis_CL03T00C08	0	0	-1000	0	-1000	0	0	-1000	0
Bacteroides_fragilis_CL03T12C07	0	0	-1000	0	-1000	0	0	-1000	0
Bacteroides_fragilis_CL05T00C42	0	0	-1000	0	-1000	0	0	-1000	0
Bacteroides_fragilis_CL05T12C13	0	0	-1000	0	-1000	0	0	-1000	0
Bacteroides_fragilis_CL07T00C01	0	0	-1000	0	-1000	0	0	-1000	0
Bacteroides_fragilis_CL07T12C05	0	0	-1000	0	-1000	0	0	-1000	0
Bacteroides_fragilis_ERR1022457	0	0	-1000	0	-1000	0	0	-1000	0
Bacteroides_fragilis_ERR1203962	0	0	-1000	0	-1000	0	0	-1000	0
Bacteroides_fragilis_ERR1204056	0	0	-1000	0	-1000	0	0	-1000	0
Bacteroides_fragilis_ERR2221134	0	0	-1000	0	-1000	0	0	-1000	0
Bacteroides_fragilis_ERR2221268	0	0	-1000	0	-1000	0	0	-1000	0
Bacteroides_fragilis_ERR2221298	0	0	-1000	0	-1000	0	0	-1000	0
Bacteroides_fragilis_ERR2230126	0	0	-1000	0	-1000	0	0	-1000	0
Bacteroides_fragilis_ERR2230137	0	0	-1000	0	-1000	0	0	-1000	0
Bacteroides_fragilis_HMW_610	0	0	-1000	0	-1000	0	0	-1000	0
Bacteroides_fragilis_HMW_615	0	0	-1000	0	-1000	0	0	-1000	0
Bacteroides_fragilis_HMW_616	0	0	-1000	0	-1000	0	0	-1000	0
Bacteroides_fragilis_NCTC_9343	0	0	-1000	-1000	-1000	0	0	-1000	0
Bacteroides_fragilis_str_3397_T10	0	0	-1000	0	-18.213321	0	0	0	0
Bacteroides_fragilis_str_3986_T_B_9	0	0	-1000	0	-1000	0	0	-1000	0
Bacteroides_fragilis_str_DS_208	0	0	-1000	0	-1000	0	0	-1000	0
Bacteroides_fragilis_YCH46	0	0	-1000	-1000	-1000	0	0	-1000	0
Bacteroides_gallinarum_DSM_18171_JCM_13658	0	0	0	0	-1000	0	0	-740.25974	0
Bacteroides_graminisolvens_DSM_19988	0	0	-1000	-1000	-1000	0	0	-1000	0
Bacteroides_helcogenes_P_36_108	0	0	-1000	0	-1000	0	0	-629.16667	0
Bacteroides_intestinalis_341_DSM_17393	0	0	-1000	0	-1000	0	0	-1000	0
Bacteroides_intestinalis_KLE1704	0	0	-1000	0	-1000	0	0	-614.15897	0
Bacteroides_luti_DSM_26991	0	0	-1000	0	-1000	0	0	-1000	0
Bacteroides_massiliensis_B84634	0	0	-1000	0	-1000	0	0	-1000	0
Bacteroides_neonati_MS4	0	0	0	-1000	-1000	0	0	0	0
Bacteroides_nordii_CL02T12C05	0	0	-1000	-1000	-1000	0	0	-1000	0
Bacteroides_nordii_ERR2221135	0	0	-1000	0	-1000	0	0	-708.33333	0
Bacteroides_nov_ERR1022359	0	0	-1000	0	-1000	0	0	0	0
Bacteroides_nov_ERR1022361	0	0	-1000	0	-1000	0	0	0	0
Bacteroides_nov_ERR1022456	0	0	-1000	0	-1000	0	0	0	0
Bacteroides_nov_ERR2221137	0	0	-1000	0	-1000	0	0	0	0
Bacteroides_nov_ERR2221300	0	0	-1000	0	-1000	0	0	0	0
Bacteroides_nov_ERR2221375	0	0	-1000	0	-1000	0	0	0	0
Bacteroides_nov_ERR2221405	0	0	-1000	0	-1000	0	0	0	0
Bacteroides_oleiciplenus_YIT_12058	0	0	-1000	-1000	-1000	-1000	0	-1000	0
Bacteroides_ovatus_3_8_47FAA	0	0	-1000	0	-1000	0	0	-1000	0
Bacteroides_ovatus_ATCC_8483	0	0	-1000	0	-1000	0	0	-1000	0
Bacteroides_ovatus_CL02T12C04	0	0	-1000	0	-1000	0	0	-1000	0
Bacteroides_ovatus_CL03T12C18	0	0	-1000	0	-1000	0	0	-1000	0
Bacteroides_ovatus_ERR1203959	0	0	-1000	0	-1000	0	0	-1000	0
Bacteroides_ovatus_ERR1204053	0	0	-1000	0	-1000	0	0	-1000	0
Bacteroides_ovatus_ERR2221136	0	0	-1000	0	-1000	0	0	-1000	0
Bacteroides_ovatus_ERR2230063	0	0	-1000	0	-1000	0	0	-1000	0
Bacteroides_ovatus_SD_CC_2a	0	0	-1000	0	-1000	-1000	0	-1000	0
Bacteroides_ovatus_SD_CMC_3f	0	0	-1000	0	-1000	0	0	-1000	0
Bacteroides_pectinophilus_ATCC_43243	0	0	-1000	-37.33562607	-18.772398	0	0	0	0
Bacteroides_plebeius_M12_DSM_17135	0	0	-1000	0	-1000	0	0	-953.7037	0
Bacteroides_propionicifaciens_DSM_19291_JCM_	0	0	0	0	-1000	0	-1000	0	0
Bacteroides_pyogenes_DSM20611	0	0	0	0	-1000	0	0	0	0
Bacteroides_pyogenes_F0041	0	0	0	0	-1000	0	0	0	0
Bacteroides_pyogenes_JCM_10003	0	0	0	0	-1000	0	0	-2.5541966	0
Bacteroides_pyogenes_JCM_6292	0	0	0	0	-1000	0	0	0	0
Bacteroides_rodentium_JCM_16496	0	0	-1000	0	-24.585902	0	0	-423.07692	0
Bacteroides_salanitronis_DSM_18170	0	0	-956.6377585	0	-1000	0	0	-274.41623	0
Bacteroides_salyersiae_CL02T12C01	0	0	-1000	-1000	-1000	0	0	-1000	0
Bacteroides_salyersiae_ERR1022329	0	0	-1000	-1000	-1000	0	0	-750	0
Bacteroides_salyersiae_WAL_10018	0	0	-1000	-1000	-1000	0	0	-1000	0
Bacteroides_sartorii_dnLKV3	0	0	-1000	-1000	-1000	0	0	0	0
Bacteroides_sartorii_JCM_17136	0	0	-1000	-1000	-1000	0	0	0	0
Bacteroides_sp_1_1_14	0	0	-1000	-1000	-1000	0	0	0	0
Bacteroides_sp_1_1_30	0	0	-1000	0	-1000	0	0	0	0
Bacteroides_sp_1_1_6	0	0	-1000	0	-1000	0	0	-1000	0
Bacteroides_sp_14_A	0	0	-1000	0	-1000	0	0	0	0

Bacteroides_sp_2_1_22	0	0	-1000	0	-1000	0	0	0	0
Bacteroides_sp_2_1_33B	0	0	0	0	-1000	0	0	0	0
Bacteroides_sp_2_1_7	0	0	0	0	-1000	0	0	0	0
Bacteroides_sp_2_2_4	0	0	-1000	0	-1000	0	0	0	0
Bacteroides_sp_20_3	0	0	0	0	-1000	0	0	0	0
Bacteroides_sp_3_1_19	0	0	0	0	-1000	0	0	0	0
Bacteroides_sp_3_1_23	0	0	-1000	0	-1000	0	0	0	0
Bacteroides_sp_3_1_33FAA	0	0	-1000	-1000	-1000	0	0	0	0
Bacteroides_sp_3_1_40A	0	0	-322.853688	-1000	-1000	0	0	0	0
Bacteroides_sp_3_2_5	0	0	-1000	0	-1000	0	0	0	0
Bacteroides_sp_4_1_36	0	0	-1000	0	-1000	0	0	0	0
Bacteroides_sp_4_3_47FAA	0	0	-378.0687398	-1000	-1000	0	0	0	0
Bacteroides_sp_9_1_42FAA	0	0	-1000	-1000	-1000	0	0	0	0
Bacteroides_sp_D1	0	0	-1000	0	-1000	0	0	0	0
Bacteroides_sp_D2	0	0	-1000	0	-1000	0	0	0	0
Bacteroides_sp_D20	0	0	-1000	0	-1000	0	0	0	0
Bacteroides_sp_D22	0	0	-1000	0	-1000	0	0	0	0
Bacteroides_sp_HPS0048	0	0	-1000	0	-1000	0	0	0	0
Bacteroides_stercoris_ATCC_43183	0	0	-1000	0	-1000	0	0	-1000	0
Bacteroides_stercoris_CC31F	0	0	-1000	0	-1000	0	0	-1000	0
Bacteroides_stercoris_ERR1203945	0	0	-1000	0	-1000	0	0	-1000	0
Bacteroides_stercoris_ERR1204039	0	0	-1000	0	-1000	0	0	-1000	0
Bacteroides_stercoris_ERR2230062	0	0	-1000	0	-1000	0	0	-1000	0
Bacteroides_stercoris_ERR2230094	0	0	-1000	0	-1000	0	0	-1000	0
Bacteroides_stercoris_ERR2230103	0	0	-1000	0	-1000	0	0	-1000	0
Bacteroides_stercoris_ERR2230124	0	0	-1000	0	-1000	0	0	-1000	0
Bacteroides_stercoris_ERR2230142	0	0	-1000	0	-1000	0	0	-1000	0
Bacteroides_stercoris_ERR2230162	0	0	-1000	0	-1000	0	0	-1000	0
Bacteroides_thetaiotaomicron_3731	0	0	-1000	0	-1000	0	0	-1000	0
Bacteroides_thetaiotaomicron_7330	0	0	-1000	0	-1000	0	0	-1000	0
Bacteroides_thetaiotaomicron_CL09T03C10	0	0	-1000	0	-1000	0	0	-720.58824	0
Bacteroides_thetaiotaomicron_dnLKV9	0	0	-1000	0	-1000	0	0	-1000	0
Bacteroides_thetaiotaomicron_ERR1022331	0	0	-1000	0	-1000	0	0	-1000	0
Bacteroides_thetaiotaomicron_ERR1022413	0	0	-1000	0	-1000	0	0	-1000	0
Bacteroides_thetaiotaomicron_ERR1022459	0	0	-1000	0	-1000	0	0	-1000	0
Bacteroides_thetaiotaomicron_ERR2221264	0	0	-1000	0	-1000	0	0	-1000	0
Bacteroides_thetaiotaomicron_ERR2221274	0	0	-1000	0	-1000	0	0	-1000	0
Bacteroides_thetaiotaomicron_ERR2221312	0	0	-1000	0	-1000	0	0	-1000	0
Bacteroides_thetaiotaomicron_ERR2230081	0	0	-1000	0	-1000	0	0	-1000	0
Bacteroides_thetaiotaomicron_VPI_5482	0	0	-1000	-1000	-1000	0	0	-1000	0
Bacteroides_timonensis_AP1	0	0	-1000	-1000	-1000	0	0	0	0
Bacteroides_uniformis_ATCC_8492	0	0	-1000	0	-1000	0	0	-1000	0
Bacteroides_uniformis_CL03T00C23	0	0	-1000	0	-1000	0	0	-1000	0
Bacteroides_uniformis_CL03T12C37	0	0	-1000	0	-1000	0	0	-1000	0
Bacteroides_uniformis_dnLKV2	0	0	-1000	0	-1000	0	0	-1000	0
Bacteroides_uniformis_ERR1022271	0	0	-1000	0	-1000	0	0	-1000	0
Bacteroides_uniformis_ERR1022322	0	0	-1000	0	-1000	0	0	-1000	0
Bacteroides_uniformis_ERR1022360	0	0	-1000	0	-1000	0	0	-1000	0
Bacteroides_uniformis_ERR1022363	0	0	-1000	0	-1000	0	0	-1000	0
Bacteroides_uniformis_ERR1022412	0	0	-1000	0	-1000	0	0	-1000	0
Bacteroides_uniformis_ERR1203944	0	0	-1000	0	-1000	0	0	-1000	0
Bacteroides_uniformis_ERR1204038	0	0	-1000	0	-1000	0	0	-1000	0
Bacteroides_uniformis_ERR2221138	0	0	-1000	0	-1000	0	0	-1000	0
Bacteroides_uniformis_ERR2221253	0	0	-1000	0	-1000	0	0	-1000	0
Bacteroides_uniformis_ERR2221261	0	0	-1000	0	-1000	0	0	-1000	0
Bacteroides_uniformis_ERR2221281	0	0	-1000	0	-1000	0	0	-1000	0
Bacteroides_uniformis_ERR2230106	0	0	-1000	0	-1000	0	0	-1000	0
Bacteroides_uniformis_ERR2230119	0	0	-1000	0	-1000	0	0	-1000	0
Bacteroides_uniformis_ERR2230138	0	0	-1000	0	-1000	0	0	-1000	0
Bacteroides_uniformis_ERR2230139	0	0	-1000	0	-1000	0	0	-1000	0
Bacteroides_uniformis_ERR2230141	0	0	-1000	0	-1000	0	0	-1000	0
Bacteroides_uniformis_ERR2230143	0	0	-1000	0	-1000	0	0	-1000	0
Bacteroides_uniformis_ERR2230159	0	0	-1000	0	-1000	0	0	-1000	0
Bacteroides_uniformis_ERR2230160	0	0	-1000	0	-1000	0	0	-1000	0
Bacteroides_uniformis_str_3978_T3_i	0	0	-1000	0	-1000	0	0	-1000	0
Bacteroides_ureolyticus_DSM_20703	0	0	0	-1000	-8.0864179	0	0	0	0
Bacteroides_vulgatus_ATCC_8482	0	0	-1000	-1000	-1000	0	0	-1000	0
Bacteroides_vulgatus_CL09T03C04	0	0	-1000	-1000	-1000	0	0	-1000	0
Bacteroides_vulgatus_dnLKV7	0	0	-1000	-1000	-1000	0	0	-1000	0
Bacteroides_vulgatus_ERR1022358	0	0	-1000	-1000	-1000	0	0	-1000	0
Bacteroides_vulgatus_ERR1022411	0	0	-1000	-1000	-1000	0	0	-1000	0
Bacteroides_vulgatus_ERR1022458	0	0	-1000	-1000	-1000	0	0	-1000	0
Bacteroides_vulgatus_ERR1203951	0	0	-1000	-1000	-1000	0	0	-1000	0
Bacteroides_vulgatus_ERR1204045	0	0	-1000	-1000	-1000	0	0	-1000	0
Bacteroides_vulgatus_ERR2221206	0	0	-1000	-1000	-1000	0	0	-1000	0
Bacteroides_vulgatus_ERR2221254	0	0	-1000	-1000	-1000	0	0	-1000	0
Bacteroides_vulgatus_ERR2221256	0	0	-1000	-1000	-1000	0	0	-1000	0
Bacteroides_vulgatus_ERR2221259	0	0	-1000	-1000	-1000	0	0	-1000	0
Bacteroides_vulgatus_ERR2221267	0	0	-1000	-1000	-1000	0	0	-1000	0
Bacteroides_vulgatus_ERR2221272	0	0	-1000	-1000	-1000	0	0	-1000	0
Bacteroides_vulgatus_ERR2221273	0	0	-1000	-1000	-1000	0	0	-1000	0
Bacteroides_vulgatus_ERR2221276	0	0	-1000	-1000	-1000	0	0	-1000	0
Bacteroides_vulgatus_ERR2230079	0	0	-1000	-1000	-1000	0	0	-1000	0
Bacteroides_vulgatus_ERR2230086	0	0	-1000	-1000	-1000	0	0	-1000	0
Bacteroides_vulgatus_ERR2230091	0	0	-1000	-1000	-1000	0	0	-1000	0
Bacteroides_vulgatus_ERR2230095	0	0	-1000	-1000	-1000	0	0	-1000	0
Bacteroides_vulgatus_ERR2230128	0	0	-1000	-1000	-1000	0	0	-1000	0
Bacteroides_vulgatus_ERR2230134	0	0	-1000	-1000	-1000	0	0	-1000	0
Bacteroides_vulgatus_ERR2230150	0	0	-1000	-1000	-1000	0	0	-1000	0
Bacteroides_vulgatus_mpk	0	0	-1000	-1000	-1000	0	0	-1000	0
Bacteroides_vulgatus_PC510	0	0	-1000	-1000	-1000	0	0	-1000	0

Bacteroides_xylanisolvens_CL03T12C04	0	0	-1000	0	-1000	-1000	0	-1000	0
Bacteroides_xylanisolvens_ERR1022297	0	0	-1000	-1000	-1000	-1000	0	-1000	0
Bacteroides_xylanisolvens_ERR2221139	0	0	-1000	0	-1000	-1000	0	-1000	0
Bacteroides_xylanisolvens_ERR2221366	0	0	-1000	0	-1000	-1000	0	-1000	0
Bacteroides_xylanisolvens_ERR2230096	0	0	-1000	0	-1000	-1000	0	-1000	0
Bacteroides_xylanisolvens_ERR2230097	0	0	-1000	0	-1000	-1000	0	-1000	0
Bacteroides_xylanisolvens_SD_CC_1b	0	0	-1000	0	-1000	-1000	0	-1000	0
Bacteroides_xylanisolvens_XB1A	0	0	-1000	0	-1000	-1000	0	-1000	0
Bacteroides_xylanolyticus_DSM_3808	0	0	0	-1000	-1000	0	0	-1000	0
Bacteroidetes_bacterium_oral_taxon_272_str_F0	0	0	-1000	0	-22.798949	0	0	0	0
Bacteroidetes_oral_taxon_274_str_F0058	0	0	0	0	-18.38083	0	0	0	0
Barnesiella_intestinihominis_ERR1203957	0	0	0	0	-22.802079	0	0	0	0
Barnesiella_intestinihominis_ERR1204051	0	0	0	-1000	-22.802078	0	0	0	0
Barnesiella_intestinihominis_ERR2221140	0	0	0	0	-22.638979	0	0	0	0
Barnesiella_intestinihominis_ERR2230085	0	0	0	0	-32.103508	0	0	0	0
Barnesiella_intestinihominis_YIT_11860	0	0	0	-1000	0	0	0	0	0
Barnesiella_viscericola_DSM_18177	0	0	0	0	-22.623731	0	0	-666.66667	0
Bartonella_quintana_RM_11	0	0	0	-1000	-8.5299346	0	0	0	0
Bartonella_quintana_Toulouse	0	0	0	-1000	-100	0	0	0	0
Bdellovibrio_bacteriovorus_HD100	-2.971944557	0	0	0	-10.775718	0	-500	-388.88889	0
Bdellovibrio_bacteriovorus_SSB218315	0	0	0	0	-10.55957	0	-316.83168	-259.62345	0
Bdellovibrio_bacteriovorus_str_Tiberius	0	0	0	0	-10.704322	0	-500	-259.84712	0
Bdellovibrio_bacteriovorus_W	0	0	0	0	-9.5504101	0	-306.93069	-259.45651	0
Bifidobacterium_adolescentis_ATCC_15703	0	0	0	0	-157.12997	-1000	0	-1000	0
Bifidobacterium_adolescentis_BBMN23	0	0	0	-1000	-20.75764	-1000	0	-1000	0
Bifidobacterium_adolescentis_DSM_20087	0	0	0	-1000	-22.477536	-1000	0	-1000	0
Bifidobacterium_adolescentis_ERR1022283	0	0	0	-1000	-20.882521	-1000	0	-1000	0
Bifidobacterium_adolescentis_ERR1022320	0	0	0	-1000	-21.731196	-1000	0	-1000	0
Bifidobacterium_adolescentis_ERR1022366	0	0	0	-1000	-20.735481	-1000	0	-1000	0
Bifidobacterium_adolescentis_ERR1203960	0	0	0	-1000	-21.73113	-1000	0	-1000	0
Bifidobacterium_adolescentis_ERR2221193	0	0	0	-1000	-21.920598	-1000	0	-1000	0
Bifidobacterium_adolescentis_ERR2221322	0	0	0	-1000	-20.883421	-1000	0	-1000	0
Bifidobacterium_adolescentis_ERR2230053	0	0	0	-1000	-23.605689	-1000	0	-1000	0
Bifidobacterium_adolescentis_ERR2230077	0	0	0	-1000	-20.882302	-1000	0	-1000	0
Bifidobacterium_adolescentis_ERR2230153	0	0	0	-1000	-20.882306	-1000	0	-1000	0
Bifidobacterium_adolescentis_L2_32	0	0	0	-1000	-23.986596	-1000	0	-1000	0
Bifidobacterium_angulatum_DSM_20098	0	0	0	0	-27.395874	0	0	-1000	0
Bifidobacterium_angulatum_GT102	0	0	0	-1000	-20.188485	0	0	-1000	0
Bifidobacterium_animalis_ERR2221337	0	0	0	0	-22.144381	0	0	-750	0
Bifidobacterium_animalis_ERR2221385	0	0	0	0	-22.144373	0	0	-750	0
Bifidobacterium_animalis_lactis_AD011	0	0	0	0	-19.034434	0	0	-754.71698	0
Bifidobacterium_animalis_lactis_BB_12	0	0	0	0	-177.95878	0	0	-1000	0
Bifidobacterium_animalis_lactis_Bi_07	0	0	0	0	-173.99804	0	0	-1000	0
Bifidobacterium_animalis_lactis_Bl_04_ATCC_SD	0	0	0	0	-173.9937	0	0	-1000	0
Bifidobacterium_animalis_lactis_CNCM_I_2494	0	0	0	0	-173.60596	0	0	-916.99605	0
Bifidobacterium_animalis_lactis_DSM_10140	0	0	0	0	-173.9937	0	0	-1000	0
Bifidobacterium_animalis_lactis_V9	0	0	0	0	-174.34818	0	0	-1000	0
Bifidobacterium_animalis_RH	0	0	0	0	-22.175064	0	0	-775.5102	0
Bifidobacterium_animalis_subsp_animalis_ATCC_	0	0	0	0	-21.002222	0	0	-881.89903	0
Bifidobacterium_animalis_subsp_animalis_ATCC_	0	0	0	0	-20.453112	0	0	-750	0
Bifidobacterium_animalis_subsp_animalis_YL2	0	0	0	0	-20.417992	0	0	-775.5102	0
Bifidobacterium_animalis_subsp_lactis_B420	0	0	0	0	-21.669597	0	0	-887.88989	0
Bifidobacterium_animalis_subsp_lactis_BLC1	0	0	0	0	-21.669597	0	0	-887.88989	0
Bifidobacterium_animalis_subsp_lactis_BS_01	0	0	0	0	-21.669597	0	0	-887.88989	0
Bifidobacterium_animalis_subsp_lactis_HN019	0	0	0	0	-21.669597	0	0	-887.88989	0
Bifidobacterium_asteroides_DSM_20089	0	0	0	-1000	-17.107634	0	0	-1000	0
Bifidobacterium_asteroides_Hma3	0	0	0	-1000	-17.193808	0	0	-1000	0
Bifidobacterium_asteroides_PRL2011	0	0	0	-1000	-33.137883	0	0	-1000	0
Bifidobacterium_bifidum_156B	0	0	0	-46.35800734	-22.216782	0	0	-1000	0
Bifidobacterium_bifidum_ATCC_29521	0	0	0	-43.02607743	-17.776741	0	0	-1000	0
Bifidobacterium_bifidum_BGN4	0	0	0	-250.6738071	-156.94466	0	0	-1000	0
Bifidobacterium_bifidum_ERR1022335	0	0	0	-46.50240423	-22.286079	0	0	-1000	0
Bifidobacterium_bifidum_LMG_13195	0	0	0	-50.01128397	-23.654698	0	0	-1000	0
Bifidobacterium_bifidum_NCIMB_41171	0	0	0	0	-24.945624	0	0	-1000	0
Bifidobacterium_bifidum_PRL2010	0	0	0	-500	-156.94312	0	0	-1000	0
Bifidobacterium_bifidum_S17	0	0	0	-333.3333333	-156.94102	0	0	-1000	0
Bifidobacterium_boum_DSM_20432	0	0	0	0	-19.031967	0	0	-674.01961	0
Bifidobacterium_breve_12L	0	0	0	0	-23.287052	0	0	-1000	0
Bifidobacterium_breve_2L	0	0	0	0	-23.287052	0	0	-1000	0
Bifidobacterium_breve_689b	0	0	0	0	-23.289089	0	0	-1000	0
Bifidobacterium_breve_ACS_071_V_Sch8b	0	0	0	0	-24.112964	0	0	-1000	0
Bifidobacterium_breve_CECT_7263	0	0	0	0	-24.067054	0	0	-1000	0
Bifidobacterium_breve_DPC_6330	0	0	0	0	-23.566982	0	0	-1000	0
Bifidobacterium_breve_DSM_20213	0	0	0	0	-23.911324	0	0	-1000	0
Bifidobacterium_breve_ERR2230051	0	0	0	0	-26.343896	0	0	-1000	0
Bifidobacterium_breve_HPH0326	0	0	0	0	-156.91386	0	0	-1000	0
Bifidobacterium_breve_JCM_7017	0	0	0	0	-23.289089	0	0	-1000	0
Bifidobacterium_breve_JCM_7019	0	0	0	0	-22.893866	0	0	-1000	0
Bifidobacterium_breve_MCC_0121	0	0	0	0	-23.28911	0	0	-1000	0
Bifidobacterium_breve_MCC_0305	0	0	0	0	-22.930411	0	0	-1000	0
Bifidobacterium_breve_MCC_0476	0	0	0	0	-21.459767	0	0	-1000	0
Bifidobacterium_breve_MCC_1094	0	0	0	0	-23.287062	0	0	-1000	0
Bifidobacterium_breve_MCC_1114	0	0	0	0	-22.357935	0	0	-1000	0
Bifidobacterium_breve_MCC_1128	0	0	0	0	-23.253685	0	0	-1000	0
Bifidobacterium_breve_MCC_1340	0	0	0	0	-22.93039	0	0	-1000	0
Bifidobacterium_breve_MCC_1454	0	0	0	0	-23.289068	0	0	-1000	0
Bifidobacterium_breve_MCC_1604	0	0	0	0	-22.930401	0	0	-1000	0
Bifidobacterium_breve_MCC_1605	0	0	0	0	-22.893908	0	0	-1000	0
Bifidobacterium_breve_NCFB_2258	0	0	0	0	-21.48964	0	0	-1000	0
Bifidobacterium_breve_UCC2003_NCIMB8807	0	0	0	0	-173.34453	0	0	-1000	0
Bifidobacterium_catenulatum_DSM_16992	0	0	0	0	-26.944647	0	0	-1000	0

Bifidobacterium_choerinum_DSM_20434	0	0	0	0	-19.246815	0	0	-741.37931	0
Bifidobacterium_coryneforme_Bma6	0	0	0	-1000	-33.451989	0	0	-1000	0
Bifidobacterium_coryneforme_DSM_20216	0	0	0	-1000	0	0	0	-1000	0
Bifidobacterium_dentium_ATCC_27678	0	0	0	-1000	-174.59103	-1000	0	-839.71236	0
Bifidobacterium_dentium_ATCC_27679	0	0	0	-1000	-24.559811	-1000	0	-987.06107	0
Bifidobacterium_dentium_Bd1	0	0	0	-1000	-23.194376	-1000	0	-1000	0
Bifidobacterium_dentium_JCM_1195_DSM_2043	0	0	0	-1000	-19.216084	-1000	0	-729.87039	0
Bifidobacterium_dentium_JCVIHMP022	0	0	0	-1000	-26.673398	-1000	0	-977.73655	0
Bifidobacterium_gallicum_DSM_20093	0	0	0	0	-21.717982	0	0	-1000	0
Bifidobacterium_indicum_LMG_11587_DSM_202	0	0	0	-1000	-26.773822	0	0	-1000	0
Bifidobacterium_kashiwanohense_DSM_21854	0	0	0	0	-25.833492	0	0	-1000	0
Bifidobacterium_longum_BG7	0	0	0	0	-23.905237	0	0	-1000	0
Bifidobacterium_longum_BXY01	0	0	0	0	-24.906511	0	0	-1000	0
Bifidobacterium_longum_DJO10A	0	0	0	0	-175.33541	0	0	-1000	0
Bifidobacterium_longum_E18	0	0	0	0	-156.90538	0	0	-1000	0
Bifidobacterium_longum_ERR2221141	0	0	0	0	-22.240631	0	0	-1000	0
Bifidobacterium_longum_ERR2221351	0	0	0	0	-22.240617	0	0	-1000	0
Bifidobacterium_longum_ERR2221409	0	0	0	0	-22.240618	0	0	-1000	0
Bifidobacterium_longum_ERR2230052	0	0	0	0	-22.800769	0	0	-1000	0
Bifidobacterium_longum_ERR2230120	0	0	0	0	-22.240631	0	0	-1000	0
Bifidobacterium_longum_ERR2230133	0	0	0	0	-22.24063	0	0	-1000	0
Bifidobacterium_longum_ERR2230158	0	0	0	0	-22.24063	0	0	-1000	0
Bifidobacterium_longum_infantis_157F_NC	0	0	-1000	0	-21.320312	0	0	-1000	0
Bifidobacterium_longum_infantis_ATCC_15697	0	0	-1000	0	-27.240395	-1000	0	-1000	0
Bifidobacterium_longum_longum_ATCC_55813	0	0	0	0	-27.763614	0	0	-1000	0
Bifidobacterium_longum_longum_BBMN68	0	0	0	0	-175.90979	0	0	-1000	0
Bifidobacterium_longum_longum_CCUG_52486	0	0	0	0	-31.464864	0	0	-1000	0
Bifidobacterium_longum_longum_JCM_1217	0	0	0	0	-159.17999	0	0	-1000	0
Bifidobacterium_longum_longum_JDM301	0	0	0	0	-158.85206	0	0	-1000	0
Bifidobacterium_longum_NCC2705	0	0	0	0	-27.763614	0	0	-1000	0
Bifidobacterium_longum_subsp_infantis_BT1	0	0	0	-1000	-25.279265	0	0	-1000	0
Bifidobacterium_longum_subsp_longum_1_6B	0	0	0	0	-29.569323	0	0	-1000	0
Bifidobacterium_longum_subsp_longum_2_2B	0	0	0	0	-26.478138	0	0	-1000	0
Bifidobacterium_longum_subsp_longum_35B	0	0	0	0	-26.600731	0	0	-1000	0
Bifidobacterium_longum_subsp_longum_44B	0	0	0	0	-27.186371	0	0	-1000	0
Bifidobacterium_longum_subsp_longum_CMCC_	0	0	0	0	-27.180559	0	0	-1000	0
Bifidobacterium_longum_subsp_longum_F8	0	0	0	0	-27.157416	0	0	-1000	0
Bifidobacterium_longum_subsp_longum_KACC_9	0	0	0	0	-27.525375	0	0	-1000	0
Bifidobacterium_longum_subsp_longum_NCIMB8	0	0	0	0	-23.905244	0	0	-1000	0
Bifidobacterium_mongoliense_DSM_21395	0	0	0	0	-23.741035	0	0	-940	0
Bifidobacterium_pseudocatenulatum_DSM_2043	0	0	0	0	-24.437923	0	0	-1000	0
Bifidobacterium_pseudocatenulatum_ERR102235	0	0	0	-284.8788638	-19.850048	0	0	-1000	0
Bifidobacterium_pseudocatenulatum_ERR120397	0	0	0	-284.8788638	-19.849943	0	0	-1000	0
Bifidobacterium_pseudocatenulatum_ERR120407	0	0	0	-284.8788638	-19.849945	0	0	-1000	0
Bifidobacterium_pseudocatenulatum_ERR222114	0	0	0	-284.8788638	-19.859452	0	0	-1000	0
Bifidobacterium_pseudocatenulatum_IPLA36007	0	0	0	0	-17.201889	0	0	-1000	0
Bifidobacterium_pseudolongum_subsp_Pseudolo	0	0	0	0	-24.591406	0	0	-886.36364	0
Bifidobacterium_pullorum_DSM_20433	0	0	0	-36.87183908	-18.563814	0	0	-1000	0
Bifidobacterium_pullorum_LMG_21816	0	0	0	-29.20894026	-19.078053	0	0	-1000	0
Bifidobacterium_ruminantium_DSM_6489	0	0	0	-1000	-23.857175	-1000	0	-819.49934	0
Bifidobacterium_scardovii_JCM_12489	0	0	0	0	-22.744226	0	0	-730.15385	0
Bifidobacterium_sp_MSTE12	0	0	0	-1000	-25.449099	0	0	-645.34884	0
Bifidobacterium_stercoris_DSM_24849	0	0	0	-1000	-19.682238	-1000	0	-710.59324	0
Bifidobacterium_stercoris_ERR1204054	0	0	0	-1000	-19.328756	-1000	0	-703.9801	0
Bifidobacterium_thermacidophilum_subsp_therm	0	0	0	0	-25.100846	0	0	-850	0
Bifidobacterium_thermophilum_RBL67	0	0	0	0	-28.356953	0	0	-805.94406	0
Bilophila_sp_4_1_30	0	0	0	-1000	-31.600888	0	0	0	0
Bilophila_wadsworthia_3_1_6	0	0	0	-29.27302795	-14.383283	0	-1000	0	0
Bilophila_wadsworthia_ATCC_49260	0	0	0	-1000	-38.29544	0	-1000	0	0
Bittarella_massiliensis_GD6	0	0	0	0	-9.9885555	0	0	0	0
Blastococcus_massiliensis_AP3	0	0	0	0	-33.299327	0	0	0	0
Blautia_coccoides_YL58	0	0	0	-50.92345484	-26.350794	-1000	0	-729.16667	0
Blautia_faecis_ERR2221099	0	0	0	-42.95274868	-21.971434	0	0	-601.69492	0
Blautia_gnavus_ATCC_29149	0	0	0	-72.88244969	-30.599512	0	0	-753.66082	0
Blautia_gnavus_ERR1022310	0	0	0	-48.69430492	-23.561042	0	0	-620.96774	0
Blautia_gnavus_ERR1203926	0	0	0	-48.30469154	-23.37563	0	0	-816.66667	0
Blautia_gnavus_ERR171257	0	0	0	-48.88876122	-23.655249	0	0	-620.96774	0
Blautia_gnavus_ERR2221233	0	0	0	-48.30468486	-23.375627	0	0	-816.66667	0
Blautia_hansenii_VPI_C7_24_DSM_20583	0	0	0	-206.8965517	-24.633441	0	0	0	0
Blautia_hydrogenotrophica_DSM_10507	0	0	0	-1000	-1000	0	0	0	0
Blautia_hydrogenotrophica_ERR1022315	0	0	0	-1000	-1000	0	0	-916.66667	0
Blautia_luti_DSM_14534	0	0	0	-39.48917894	-19.785293	0	0	-1000	0
Blautia_luti_ERR1022337	0	0	0	-42.74250344	-21.853563	0	0	-783.85417	0
Blautia_luti_ERR1022379	0	0	0	-43.9346761	-22.394046	0	0	-519.50803	0
Blautia_luti_ERR1022471	0	0	0	-47.71522653	-24.384415	0	0	-820.76162	0
Blautia_luti_ERR1203942	0	0	0	-48.57298166	-24.820848	0	0	-820.76541	0
Blautia_luti_ERR2221098	0	0	0	-48.57196195	-24.82032	0	0	-820.76078	0
Blautia_massiliensis_GD9	0	0	0	-32.93342633	-17.391674	0	0	-714.28571	0
Blautia_nov_ERR1022275	0	0	0	-48.6882292	-22.87672	0	0	-748.1203	0
Blautia_nov_ERR1022294	0	0	0	-45.80424714	-23.536201	0	0	-583.979	0
Blautia_nov_ERR1022295	0	0	0	-32.62130496	-16.734134	0	0	-508.90589	0
Blautia_nov_ERR1022302	0	0	0	-34.00743711	-17.394995	0	0	-682.29167	0
Blautia_nov_ERR1022306	0	0	0	-48.68815064	-22.876683	0	0	-748.1203	0
Blautia_nov_ERR1022338	0	0	0	-49.64976814	-23.329715	0	0	-750	0
Blautia_nov_ERR1022339	0	0	0	-41.21766018	-21.145963	0	0	-512.79119	0
Blautia_nov_ERR1022340	0	0	0	-42.24624435	-21.673379	0	0	-511.95752	0
Blautia_nov_ERR1022378	0	0	0	-50.73853434	-22.146104	0	0	-616.43836	0
Blautia_nov_ERR1022424	0	0	0	-42.95272195	-21.971421	0	0	-786.45833	0
Blautia_nov_ERR1022426	0	0	0	-42.69719708	-21.925471	0	0	-783.85417	0
Blautia_nov_ERR1022435	0	0	0	-41.21767306	-21.145966	0	0	-513.99495	0
Blautia_nov_ERR1203955	0	0	0	-41.21768101	-21.145969	0	0	-513.99495	0

Blautia_nov_ERR1204049	0	0	0	-41.21768101	-21.145969	0	0	-513.99495	0
Blautia_nov_ERR1204065	0	0	0	-48.6881395	-22.876678	0	0	-748.1203	0
Blautia_nov_ERR2221143	0	0	0	-52.47204561	-26.932185	0	0	-916.66667	0
Blautia_nov_ERR2221170	0	0	0	-102.5257664	-46.323882	0	0	-936.27208	0
Blautia_nov_ERR2221260	0	0	0	-41.13423049	-21.10317	0	0	-513.99496	0
Blautia_nov_ERR2221265	0	0	0	-41.13423049	-21.10317	0	0	-513.99496	0
Blautia_nov_ERR2221269	0	0	0	-34.10718291	-17.443794	0	0	-564.81481	0
Blautia_nov_ERR2221270	0	0	0	-41.13422974	-21.103169	0	0	-513.99496	0
Blautia_nov_ERR2221279	0	0	0	-1000	-49.091464	0	0	-937.47271	0
Blautia_nov_ERR2221370	0	0	0	0	-26.895203	0	0	-588.46397	0
Blautia_nov_ERR2221404	0	0	0	-52.47204459	-26.932185	0	0	-916.66667	0
Blautia_nov_ERR2221410	0	0	0	-52.47203129	-26.932178	0	0	-916.66667	0
Blautia_nov_ERR2230061	0	0	0	-45.7255218	-23.495704	0	0	-583.94026	0
Blautia_nov_ERR2230064	0	0	0	-41.13420787	-21.103164	0	0	-513.99495	0
Blautia_nov_ERR2230066	0	0	0	-50.92304126	-26.000328	0	0	-502.37983	0
Blautia_nov_ERR2230067	0	0	0	-41.21946331	-21.146876	0	0	-513.99555	0
Blautia_nov_ERR2230071	0	0	0	-45.72208338	-23.493935	0	0	-583.94021	0
Blautia_nov_ERR2230072	0	0	0	-41.13422974	-21.103169	0	0	-513.99496	0
Blautia_nov_ERR2230073	0	0	0	-45.7255299	-23.495708	0	0	-583.94026	0
Blautia_nov_ERR2230075	0	0	0	-45.72551981	-23.495702	0	0	-583.94026	0
Blautia_nov_ERR2230099	0	0	0	-44.75070436	-22.894757	0	0	-583.67519	0
Blautia_nov_ERR2230110	0	0	0	-41.13422974	-21.103169	0	0	-513.99496	0
Blautia_nov_ERR2230116	0	0	0	-44.99290645	-23.104382	0	0	-726.19048	0
Blautia_nov_ERR2230130	0	0	0	-43.37198054	-22.272147	0	0	-714.28571	0
Blautia_nov_ERR2230136	0	0	0	-42.25363772	-21.675275	0	0	-511.9588	0
Blautia_nov_ERR2230152	0	0	0	-34.10717535	-17.44379	0	0	-564.81481	0
Blautia_obeum_A2_162	0	0	0	-1000	-22.671466	0	0	0	0
Blautia_obeum_ATCC_29174	0	0	0	-1000	-24.802031	0	0	-900.53763	0
Blautia_producta_DSM_2950	0	0	0	-1000	-173.09061	0	0	-854.48393	0
Blautia_producta_ERR171261	0	0	0	-62.11141817	-28.073586	0	0	-972.22222	0
Blautia_producta_ERR2221380	0	0	0	-57.71410118	-26.163327	0	0	-729.16667	0
Blautia_sp_YL58	0	0	0	-50.92084911	-26.348627	0	0	-729.16667	0
Blautia_torques_ATCC_27756	0	0	0	-1000	-21.37712	0	0	0	0
Blautia_torques_ERR1022291	0	0	0	-47.71605966	-23.490914	0	0	-816.66667	0
Blautia_torques_ERR1022304	0	0	0	-45.86508216	-22.829336	0	0	0	0
Blautia_torques_ERR1022325	0	0	0	-46.60785345	-22.949463	0	0	-557.69231	0
Blautia_torques_ERR1022357	0	0	0	-45.86498987	-22.82929	0	0	0	0
Blautia_torques_ERR1022404	0	0	0	-47.11395668	-23.289955	0	0	-791.66667	0
Blautia_torques_ERR1022479	0	0	0	-52.41460289	-25.852493	0	0	-791.66667	0
Blautia_torques_ERR1203943	0	0	0	-47.48166584	-23.37641	0	0	-557.69231	0
Blautia_torques_ERR1203966	0	0	0	-45.8650455	-22.829318	0	0	0	0
Blautia_torques_ERR1204037	0	0	0	-47.48167246	-23.376413	0	0	-557.69231	0
Blautia_torques_ERR1204060	0	0	0	-45.86503932	-22.829315	0	0	0	0
Blautia_torques_ERR2221100	0	0	0	-46.14204536	-22.721819	0	0	-791.66667	0
Blautia_torques_ERR2221189	0	0	0	-33.06863574	-16.454704	0	0	0	0
Blautia_torques_ERR2230088	0	0	0	-33.06854327	-16.454658	0	0	0	0
Blautia_torques_ERR2230104	0	0	0	-45.86501295	-22.829302	0	0	0	0
Blautia_torques_L2_14	0	0	0	-1000	-22.620392	0	0	-704.85223	0
Blautia_wexlerae_AGR2146	0	0	0	-38.33184139	-18.736702	0	0	-518.28549	0
Blautia_wexlerae_DSM_19850	0	0	0	-1000	-19.558484	0	0	0	0
Blautia_wexlerae_ERR1022296	0	0	0	-41.58355998	-21.198831	0	0	-818.83744	0
Blautia_wexlerae_ERR1022377	0	0	0	-44.15456664	-22.505354	0	0	-825.52083	0
Blautia_wexlerae_ERR1022425	0	0	0	-49.62717468	-25.35712	0	0	-895.83333	0
Blautia_wexlerae_ERR1204036	0	0	0	-48.37394274	-24.719587	0	0	-818.83745	0
Blautia_wexlerae_ERR2230049	0	0	0	-48.74031439	-24.905941	0	0	-817.41243	0
Bordetella_hinzii_F582	0	0	0	-1000	-28.56418	0	0	0	0
Bordetella_hinzii_OH87_BAL007II	0	0	0	-1000	-30.866	0	0	0	0
Borkfalkia_ceftriaxoniphila_HDS1380	0	0	0	0	-19.534894	0	0	-272.0767	0
Bosea_thiooxidans_DSM_9653	0	0	0	-1000	-13.48154	0	0	0	0
Brachybacterium_paraconglomeratum_LC44	0	0	0	-1000	0	-1000	0	-1000	0
Brachyspira_aalborgi_513A	0	0	0	-1000	-29.432888	0	0	0	0
Brachyspira_aalborgi_PC2022III	0	0	0	-1000	-27.557534	0	0	0	0
Brachyspira_aalborgi_PC2777IV	0	0	0	-1000	-18.604731	0	0	0	0
Brachyspira_aalborgi_PC3053II	0	0	0	-1000	-24.029972	0	0	0	0
Brachyspira_aalborgi_PC3517II	0	0	0	-1000	-19.119599	-1000	0	0	0
Brachyspira_aalborgi_PC3714II	0	0	0	-1000	-18.619118	0	0	0	0
Brachyspira_aalborgi_PC390II	0	0	0	-1000	-18.64779	0	0	0	0
Brachyspira_aalborgi_PC3939II	0	0	0	-1000	-18.647788	0	0	0	0
Brachyspira_aalborgi_PC3997IV	0	0	0	-34.90444142	-19.032566	-1000	0	0	0
Brachyspira_aalborgi_PC4226IV	0	0	0	-1000	-18.63335	0	0	0	0
Brachyspira_aalborgi_PC4580III	0	0	0	-1000	-18.184915	-890.41096	0	0	0
Brachyspira_aalborgi_PC4597II	0	0	0	-1000	-23.465176	0	0	0	0
Brachyspira_aalborgi_PC5099IV	0	0	0	-1000	-18.63335	0	0	0	0
Brachyspira_aalborgi_PC5538III_lc	0	0	0	-1000	-21.680766	-1000	0	0	0
Brachyspira_aalborgi_PC5587_p	0	0	0	-1000	-23.4424	0	0	0	0
Brachyspira_aalborgi_PC5587_u	0	0	0	-1000	-23.465179	0	0	0	0
Brachyspira_aalborgi_W1	0	0	0	-1000	-23.730046	-1000	0	0	0
Brachyspira_pilosicoli_95_1000	0	0	0	-37.967766	-25.158232	0	-1000	-750	0
Brachyspira_pilosicoli_B2904	0	0	-1000	-42.68778966	-30.074749	-1000	-1000	-750	0
Brachyspira_pilosicoli_P43_6_78	0	0	0	-1000	-36.083092	0	-1000	-438.01886	0
Brachyspira_pilosicoli_WesB	0	0	-1000	-39.13108614	-27.30275	0	-1000	-750	0
Bradyrhizobium_elkanii_USDA_76	0	0	0	-1000	-46.668477	0	0	0	0
Bradyrhizobium_japonicum_E109	0	0	0	-1000	-34.474721	0	0	0	0
Bradyrhizobium_japonicum_USDA_110	0	0	0	-1000	-21.651616	-1000	0	0	0
Bradyrhizobium_japonicum_USDA_124	0	0	0	-1000	-20.333127	0	0	0	0
Bradyrhizobium_japonicum_USDA_6	0	0	0	-1000	-28.476459	0	0	0	0
Bradyrhizobium_japonicum_WSM2793	0	0	0	-1000	-18.03337	0	0	0	0
Bradyrhizobium_yuanmingense_BR3267	0	0	0	-1000	-17.914419	-1000	-1000	0	0
Bradyrhizobium_yuanmingense_CCBAU_05623	0	0	0	-1000	-21.129793	-1000	-1000	0	0
Bradyrhizobium_yuanmingense_CCBAU_25021	0	0	0	-1000	-21.272593	-1000	-1000	0	0
Bradyrhizobium_yuanmingense_CCBAU_35157	0	0	0	-1000	-1000	-1000	-1000	0	0

Brevibacillus_agri_5_2	0	0	0	-1000	-22.617763	-1000	0	-1000	0
Brevibacillus_agri_BAB_2500	0	0	0	-1000	-25.195293	-1000	0	-1000	0
Brevibacillus_borstelensis_AK1	0	0	0	-1000	-24.516349	0	0	0	0
Brevibacillus_brevis_FJAT_0809_GLX	0	0	0	-1000	-28.697221	-1000	0	-1000	0
Brevibacillus_brevis_NBRC_100599	0	0	0	-1000	-32.286063	-1000	0	-1000	0
Brevibacterium_casei_S18	0	0	0	-1000	0	0	0	-1000	0
Brevibacterium_linens_BL2	0	0	0	-1000	-15.212955	0	0	0	0
Brevibacterium_massiliense_5401308	0	0	0	-1000	-18.720904	0	-1000	0	0
Brevibacterium_senegalense_JC43	0	0	0	-1000	-6.3429713	0	0	0	0
Brevundimonas_bacteroides_DSM_4726	0	0	0	-1000	0	0	-1000	-666.66667	0
Brevundimonas_diminuta_470_4	0	0	0	-1000	-1000	0	-1000	0	0
Brevundimonas_subvibrioides_ATCC_15264	0	0	0	-1000	0	0	-1000	-523.4514	0
Brochothrix_thermosphacta_DSM_20171	0	0	0	-1000	-1000	-1000	0	-1000	0
Bulleidia_extructa_W1219	0	0	0	0	-17.689364	0	0	0	0
Burkholderia_cenocepacia_AU_1054	0	0	-1000	-1000	-40.346555	-1000	-1000	-1000	0
Burkholderia_cenocepacia_H111	0	0	-1000	-1000	-32.959742	-1000	-1000	-1000	0
Burkholderia_cenocepacia_HI2424	0	0	-1000	-1000	-40.346555	-1000	-1000	-1000	0
Burkholderia_cenocepacia_J2315	0	0	-1000	-1000	-44.557937	-1000	-1000	-1000	0
Burkholderia_cenocepacia_MC0_3	0	0	-1000	-1000	-40.351927	-1000	-1000	-1000	0
Burkholderia_cenocepacia_PC184	0	0	-1000	-1000	-41.210824	-1000	-1000	-1000	0
Burkholderia_cepacia_GG4	0	0	-1000	-1000	-1000	-1000	-1000	-1000	0
Burkholderia_cepacia_LO6	0	0	-1000	-1000	-1000	-1000	-1000	-1000	0
Burkholderiales_bacterium_1_1_47	0	0	0	-1000	0	0	0	0	0
Burkholderiales_bacterium_YL45	0	0	0	-1000	-16.82028	0	0	0	0
Butyricicoccus_nov_ERR2221182	0	0	0	-1000	-26.476497	0	0	-358.97175	0
Butyricicoccus_pullicaecorum_1_2	0	0	0	-1000	-22.52604	0	0	-645.83333	0
Butyricimonas_synergistica_DSM_23225	0	0	0	-1000	-48.170121	0	0	0	0
Butyricimonas_virosa_DSM_23226	0	0	0	-43.52085135	-42.147902	0	0	0	0
Butyricimonas_virosa_ERR2221144	0	0	0	-1000	-42.507414	0	0	0	0
Butyrivibrio_crossotus_DSM_2876	0	0	0	-56.45999363	-26.891482	0	0	0	0
Butyrivibrio_fibrisolvens_16_4	0	0	0	0	-23.385572	0	0	-782.40741	0
Butyrivibrio_proteoclasticus_B316	0	0	-1000	0	-18.889958	0	0	-478.09207	0
Campylobacter_coli_1098	0	0	0	-1000	-23.354428	0	-1000	0	0
Campylobacter_coli_111_3	0	0	0	-1000	-23.352825	0	-1000	0	0
Campylobacter_coli_1148	0	0	0	-1000	-23.340224	0	-1000	0	0
Campylobacter_coli_132_6	0	0	0	-1000	-20.889208	0	-1000	0	0
Campylobacter_coli_1417	0	0	0	-1000	-24.74529	0	-1000	0	0
Campylobacter_coli_14983A	0	0	0	-1000	-14.489601	0	-1000	0	0
Campylobacter_coli_151_9	0	0	0	-1000	-23.58195	0	-1000	0	0
Campylobacter_coli_1891	0	0	0	-1000	-24.194474	0	-1000	0	0
Campylobacter_coli_1909	0	0	0	-1000	-23.354428	0	-1000	0	0
Campylobacter_coli_1948	0	0	0	-1000	-23.354386	0	-1000	0	0
Campylobacter_coli_1957	0	0	0	-1000	-23.352798	0	-1000	0	0
Campylobacter_coli_1961	0	0	0	-1000	-23.354455	0	-1000	0	0
Campylobacter_coli_202_04	0	0	0	-1000	-23.340197	0	-1000	0	0
Campylobacter_coli_2548	0	0	0	-1000	-24.247132	0	-1000	0	0
Campylobacter_coli_2553	0	0	0	-1000	-23.352876	0	-1000	0	0
Campylobacter_coli_2680	0	0	0	-1000	-23.711914	0	-1000	0	0
Campylobacter_coli_2685	0	0	0	-1000	-23.372369	0	-1000	0	0
Campylobacter_coli_2698	0	0	0	-1000	-22.472309	0	-1000	0	0
Campylobacter_coli_317_04	0	0	0	-1000	-15.622702	0	-1000	0	0
Campylobacter_coli_37_05	0	0	0	-1000	-23.352798	0	-1000	0	0
Campylobacter_coli_59_2	0	0	0	-1000	-23.127849	0	-1000	0	0
Campylobacter_coli_67_8	0	0	0	-1000	-26.378806	0	-1000	0	0
Campylobacter_coli_7_1	0	0	0	-1000	-20.966795	0	-1000	0	0
Campylobacter_coli_80352	0	0	0	-1000	-23.642295	0	-1000	0	0
Campylobacter_coli_84_2	0	0	0	-1000	-23.340172	0	-1000	0	0
Campylobacter_coli_86119	0	0	0	-1000	-23.580314	0	-1000	0	0
Campylobacter_coli_90_3	0	0	0	-1000	-23.325248	0	-1000	0	0
Campylobacter_coli_BFR_CA_9557	0	0	0	-1000	-14.755747	0	-1000	0	0
Campylobacter_coli_BIGS0003	0	0	0	-1000	-35.095541	0	-1000	0	0
Campylobacter_coli_H56	0	0	0	-1000	-23.340197	0	-1000	0	0
Campylobacter_coli_H6	0	0	0	-1000	-24.358354	0	-1000	0	0
Campylobacter_coli_H8	0	0	0	-1000	-16.410048	0	-1000	0	0
Campylobacter_coli_H9	0	0	0	-1000	-16.515202	0	-1000	0	0
Campylobacter_coli_JV20	0	0	0	-1000	-19.818897	0	-1000	0	0
Campylobacter_coli_LMG_23336	0	0	0	-1000	-23.340172	0	-1000	0	0
Campylobacter_coli_LMG_23341	0	0	0	-1000	-23.352773	0	-1000	0	0
Campylobacter_coli_LMG_23342	0	0	0	-1000	-15.621539	0	-1000	0	0
Campylobacter_coli_LMG_9853	0	0	0	-1000	-23.340197	0	-1000	0	0
Campylobacter_coli_LMG_9854	0	0	0	-1000	-23.340197	0	-1000	0	0
Campylobacter_coli_LMG_9860	0	0	0	-1000	-23.340275	0	-1000	0	0
Campylobacter_coli_RM2228	0	0	0	-1000	-23.352851	0	-1000	0	0
Campylobacter_coli_YH501	0	0	0	-1000	-14.755791	0	-1000	0	0
Campylobacter_coli_Z156	0	0	0	-1000	-21.482814	0	-1000	0	0
Campylobacter_coli_Z163	0	0	0	-1000	-21.472171	0	-1000	0	0
Campylobacter_concisus_13826	0	0	0	0	0	0	-1000	0	0
Campylobacter_concisus_UNSWCD	0	0	0	0	0	0	-1000	0	0
Campylobacter_curvus_525_92	0	0	0	-1000	0	0	-1000	0	0
Campylobacter_curvus_DSM_6644	0	0	0	-1000	0	0	-1000	0	0
Campylobacter_fetus_subsp_fetus_006A_0073	0	0	0	-1000	-5.2034107	0	0	0	0
Campylobacter_fetus_subsp_fetus_82_40	0	0	0	-1000	-7.9649666	0	0	0	0
Campylobacter_fetus_subsp_venerealis_NCTC_1	0	0	0	-15.81995028	-9.3913373	0	0	0	0
Campylobacter_gracilis_RM3268	0	0	0	-500	-8.1010493	0	0	0	0
Campylobacter_hominis_ATCC_BAA_381	0	0	0	-500	-51.502146	0	-833.33333	0	0
Campylobacter_hyointestinalis_subsp_hyointestin	0	0	0	-1000	-8.2470329	0	0	0	0
Campylobacter_jejuni_CJM1cam	0	0	0	-1000	-12.073077	0	-1000	0	0
Campylobacter_jejuni_jejuni_81_176	0	0	0	0	-119.04762	0	-1000	0	0
Campylobacter_jejuni_jejuni_ICDCCJ07004	0	0	0	0	-133.92857	0	-1000	0	0
Campylobacter_jejuni_jejuni_M1	0	0	0	0	-119.04762	0	-1000	0	0
Campylobacter_jejuni_jejuni_NCTC_11168	0	0	0	0	-119.04762	0	-1000	0	0

Campylobacter_jejuni_NCTC11351	0	0	0	-1000	-7.6520333	0	-1000	0	0
Campylobacter_jejuni_RM1221	0	0	0	-1000	-15.525113	0	-1000	0	0
Campylobacter_jejuni_str_NCCP_No_15742	0	0	0	-1000	-28.013685	0	-1000	0	0
Campylobacter_jejuni_subsp_doylei_269_97	0	0	0	-1000	-20.725917	0	-1000	0	0
Campylobacter_jejuni_subsp_jejuni_04197	0	0	0	-1000	-26.821523	0	-1000	0	0
Campylobacter_jejuni_subsp_jejuni_04199	0	0	0	-1000	-26.916981	0	-1000	0	0
Campylobacter_jejuni_subsp_jejuni_110_21	0	0	0	-1000	-23.260194	0	-1000	0	0
Campylobacter_jejuni_subsp_jejuni_1213	0	0	0	-1000	-20.624511	0	-1000	0	0
Campylobacter_jejuni_subsp_jejuni_129_258	0	0	0	-1000	-12.938901	0	-1000	0	0
Campylobacter_jejuni_subsp_jejuni_1336	0	0	0	-1000	-24.742989	0	-1000	0	0
Campylobacter_jejuni_subsp_jejuni_140_16	0	0	0	-1000	-22.878141	0	-1000	0	0
Campylobacter_jejuni_subsp_jejuni_1798	0	0	0	-1000	-23.147794	0	-1000	0	0
Campylobacter_jejuni_subsp_jejuni_1854	0	0	0	-1000	-17.239776	0	-1000	0	0
Campylobacter_jejuni_subsp_jejuni_1893	0	0	0	-1000	-15.757511	0	-1000	0	0
Campylobacter_jejuni_subsp_jejuni_1997_1	0	0	0	-1000	-22.88281	0	-1000	0	0
Campylobacter_jejuni_subsp_jejuni_1997_11	0	0	0	-1000	-17.700034	0	-1000	0	0
Campylobacter_jejuni_subsp_jejuni_1997_7	0	0	0	-1000	-23.113261	0	-1000	0	0
Campylobacter_jejuni_subsp_jejuni_2008_1025	0	0	0	-1000	-31.479161	0	-1000	0	0
Campylobacter_jejuni_subsp_jejuni_2008_831	0	0	0	-1000	-23.318448	0	-1000	0	0
Campylobacter_jejuni_subsp_jejuni_2008_894	0	0	0	-1000	-12.093909	0	-1000	0	0
Campylobacter_jejuni_subsp_jejuni_2008_988	0	0	0	-1000	-23.637521	0	-1000	0	0
Campylobacter_jejuni_subsp_jejuni_260_94	0	0	0	-1000	-23.637442	0	-1000	0	0
Campylobacter_jejuni_subsp_jejuni_414	0	0	0	-1000	-7.1166681	0	-1000	0	0
Campylobacter_jejuni_subsp_jejuni_51037	0	0	0	-1000	-20.154358	0	-1000	0	0
Campylobacter_jejuni_subsp_jejuni_53161	0	0	0	-1000	-9.415517	0	-1000	0	0
Campylobacter_jejuni_subsp_jejuni_55037	0	0	0	-1000	-20.158685	0	-1000	0	0
Campylobacter_jejuni_subsp_jejuni_60004	0	0	0	-1000	-27.084953	0	-1000	0	0
Campylobacter_jejuni_subsp_jejuni_6399	0	0	0	-1000	-35.071951	0	-1000	0	0
Campylobacter_jejuni_subsp_jejuni_81116	0	0	0	-1000	-25.670815	0	-1000	0	0
Campylobacter_jejuni_subsp_jejuni_84_25	0	0	0	-1000	-23.26022	0	-1000	0	0
Campylobacter_jejuni_subsp_jejuni_86605	0	0	0	-1000	-17.001763	0	-1000	0	0
Campylobacter_jejuni_subsp_jejuni_87330	0	0	0	-1000	-27.796166	0	-1000	0	0
Campylobacter_jejuni_subsp_jejuni_CF93_6	0	0	0	-1000	-38.796301	0	-1000	0	0
Campylobacter_jejuni_subsp_jejuni_CG8421	0	0	0	-1000	-34.223816	0	-1000	0	0
Campylobacter_jejuni_subsp_jejuni_CG8486	0	0	0	-1000	-7.1382929	0	-1000	0	0
Campylobacter_jejuni_subsp_jejuni_D2600	0	0	0	-1000	-25.667862	0	-1000	0	0
Campylobacter_jejuni_subsp_jejuni_DFVF1099	0	0	0	-1000	-6.9969173	0	-1000	0	0
Campylobacter_jejuni_subsp_jejuni_H22082	0	0	0	-1000	-26.897926	0	-1000	0	0
Campylobacter_jejuni_subsp_jejuni_HB93_13	0	0	0	-1000	-32.939989	0	-1000	0	0
Campylobacter_jejuni_subsp_jejuni_IA3902	0	0	0	-1000	-35.126282	0	-1000	0	0
Campylobacter_jejuni_subsp_jejuni_ICDCCJ07001	0	0	0	-1000	-7.1205297	0	-1000	0	0
Campylobacter_jejuni_subsp_jejuni_ICDCCJ07002	0	0	0	-1000	-20.623145	0	-1000	0	0
Campylobacter_jejuni_subsp_jejuni_LMG_23210	0	0	0	-1000	-20.165633	0	-1000	0	0
Campylobacter_jejuni_subsp_jejuni_LMG_23211	0	0	0	-1000	-20.173263	0	-1000	0	0
Campylobacter_jejuni_subsp_jejuni_LMG_23216	0	0	0	-1000	-29.933437	0	-1000	0	0
Campylobacter_jejuni_subsp_jejuni_LMG_23218	0	0	0	-1000	-23.290141	0	-1000	0	0
Campylobacter_jejuni_subsp_jejuni_LMG_23223	0	0	0	-1000	-11.218535	0	-1000	0	0
Campylobacter_jejuni_subsp_jejuni_LMG_23264	0	0	0	-1000	-35.071968	0	-1000	0	0
Campylobacter_jejuni_subsp_jejuni_LMG_23269	0	0	0	-1000	-21.996374	0	-1000	0	0
Campylobacter_jejuni_subsp_jejuni_LMG_23357	0	0	0	-1000	-23.541204	0	-1000	0	0
Campylobacter_jejuni_subsp_jejuni_LMG_9081	0	0	0	-1000	-15.470672	0	-1000	0	0
Campylobacter_jejuni_subsp_jejuni_LMG_9217	0	0	0	-1000	-35.148745	0	-1000	0	0
Campylobacter_jejuni_subsp_jejuni_LMG_9872	0	0	0	-1000	-21.988837	0	-1000	0	0
Campylobacter_jejuni_subsp_jejuni_LMG_9879	0	0	0	-1000	-35.148728	0	-1000	0	0
Campylobacter_jejuni_subsp_jejuni_NCTC_11168	0	0	0	-1000	-35.071951	0	-1000	0	0
Campylobacter_jejuni_subsp_jejuni_NW	0	0	0	-1000	-15.672638	0	-1000	0	0
Campylobacter_jejuni_subsp_jejuni_P110B	0	0	0	-1000	-24.677448	0	-1000	0	0
Campylobacter_jejuni_subsp_jejuni_P854	0	0	0	-1000	-23.609585	0	-1000	0	0
Campylobacter_jejuni_subsp_jejuni_PT14	0	0	0	-1000	-37.23596	0	-1000	0	0
Campylobacter_jejuni_subsp_jejuni_RB922	0	0	0	-1000	-35.699517	0	-1000	0	0
Campylobacter_jejuni_subsp_jejuni_S3	0	0	0	-1000	-23.300163	0	-1000	0	0
Campylobacter_jejuni_subsp_jejuni_xy259	0	0	0	-1000	-35.598112	0	-1000	0	0
Campylobacter_lari_RM2100	0	0	0	-7.675455457	-7.3784718	0	0	0	0
Campylobacter_rectus_RM3267	0	0	0	0	-7.5077017	0	0	0	0
Campylobacter_showae_CSUNSWCD	0	0	0	-10.61504635	-7.5501232	0	0	0	0
Campylobacter_showae_RM3277	0	0	0	-1000	-12.937532	0	0	0	0
Campylobacter_sp_10_1_50	0	0	0	0	-8.5598507	0	0	0	0
Campylobacter_sputorum_bv_faecalis_CCUG_207	0	0	0	-1000	-10.3304	0	0	0	0
Campylobacter_troglodytis_MIT_05_9149A	0	0	0	-1000	-13.6248	0	0	0	0
Campylobacter_upsaliensis_JV21	0	0	0	0	-12.918389	0	-1000	0	0
Campylobacter_upsaliensis_RM3195	0	0	0	-1000	-15.104386	0	-1000	0	0
Campylobacter_ureolyticus_ACS_301_V_Sch3b	0	0	0	-1000	-8.5191718	0	0	0	0
Campylobacter_ureolyticus_CIT007	0	0	0	-1000	-7.462023	0	0	0	0
Campylobacter_ureolyticus_RIGS_9880	0	0	0	-1000	-8.5189904	0	0	0	0
Campylobacter_ureolyticus_UMB0112	0	0	0	-1000	-8.5260351	0	0	0	0
Candidatus_Dorea_massiliensis_AP6	0	0	0	-1000	-23.454834	0	0	0	0
Candidatus_Nitrososphaera_gargensis_Ga9_2	0	0	0	0	0	0	-1000	0	0
Candidatus_Pelagibacter_sp_IMCC9063	0	0	0	-12.08782483	-7.8698285	0	0	0	0
Candidatus_Saccharimonas_aalborgensis	0	0	0	-0.295012465	-1.9630673	0	0	0	0
Candidatus_Soleaferrea_massiliensis_AP7	0	0	0	0	-2.0639109	0	0	0	0
Capnocytophaga_granulosa_ATCC_51502	0	0	0	-25.89651179	-1000	0	0	-666.66667	0
Capnocytophaga_leadbetteri_DSM_22902	0	0	0	-1000	-1000	0	0	0	0
Capnocytophaga_ochracea_DSM_7271	0	0	0	-26.80905859	-1000	0	0	-415.72581	0
Capnocytophaga_ochracea_F0287	0	0	0	-36.81616857	-1000	0	0	-906.25	0
Capnocytophaga_ochracea_str_Holt_25	0	0	0	-36.81616857	-1000	0	0	-906.25	0
Capnocytophaga_sp_CM59	0	0	0	-1000	-1000	0	0	0	0
Capnocytophaga_sp_oral_taxon_326_str_F0382	0	0	0	-1000	-1000	0	0	0	0
Capnocytophaga_sp_oral_taxon_329_str_F0087	0	0	0	-1000	-1000	0	0	0	0
Capnocytophaga_sp_oral_taxon_332_str_F0381	0	0	0	-1000	-1000	0	0	0	0
Capnocytophaga_sp_oral_taxon_335_str_F0486	0	0	0	-1000	-1000	0	0	0	0
Capnocytophaga_sp_oral_taxon_336_str_F0502	0	0	0	0	-1000	0	0	0	0

Capnocytophaga_sp_oral_taxon_338_str_F0234	0	0	0	0	-1000	0	0	0	0
Capnocytophaga_sp_oral_taxon_412_str_F0487	0	0	0	0	-1000	0	0	0	0
Capnocytophaga_sputigena_ATCC_33612	0	0	0	-34.69846555	-1000	0	0	-1000	0
Cardiobacterium_hominis_612_ETAR_1001_5485	0	0	0	-1000	-22.379609	-1000	0	-738.86713	0
Cardiobacterium_valvarum_F0432	0	0	0	-1000	-36.36453	-1000	0	-666.66667	0
Carnobacterium_maltaromaticum_ATCC_35586	0	0	0	-1000	-37.784527	-1000	0	-1000	0
Carnobacterium_maltaromaticum_DSM_20342	0	0	0	-1000	-37.387849	-1000	0	-881.19835	0
Carnobacterium_maltaromaticum_DSM_20722	0	0	0	-1000	-37.991132	-1000	0	-1000	0
Carnobacterium_maltaromaticum_LMA28	0	0	0	-1000	-37.388415	-1000	0	-1000	0
Catabacter_hongkongensis_HKU16	0	0	0	-1000	-11.542264	0	0	0	0
Catenibacterium_mitsuokai_DSM_15897	0	0	0	0	-20.18884	0	0	-368.25725	0
Catenibacterium_mitsuokai_ERR1022284	0	0	-1000	0	-31.631744	0	0	-887.5	0
Catenibacterium_mitsuokai_ERR2221198	0	0	0	0	-22.746752	0	0	-496.139	0
Catonella_morbi_ATCC_51271	0	0	0	-1000	-29.902728	0	0	-818.18182	0
Cedecea_davisae_DSM_4568	0	0	0	-1000	-60.015666	-1000	0	-1000	0
Cellulomonas_massiliensis_JC225	0	0	0	0	-16.878677	0	0	-548.18976	0
Cellulomonas_massiliensis_MGYG_HGUT_01416	0	0	0	0	-29.208176	0	0	-739.39226	0
Cellulosilyticum_lentocellum_DSM_5427	0	0	0	-1000	-17.942274	0	-1000	-942.35101	0
Cellulosimicrobium_cellulans_J36	0	0	0	-1000	-38.073412	-700	-1000	-350	0
Centipeda_periodontii_DSM_2778	0	0	0	0	-29.811664	-1000	0	-583.33333	0
Cetobacterium_somerae_ATCC_BAA_47401	0	0	0	-1000	-42.018646	0	-1000	-1000	0
Chlorobium_phaeobacteroides_BS1	0	0	0	-1000	-12.240006	0	0	0	0
Chlorobium_phaeobacteroides_DSM_266	0	0	0	0	-17.394524	0	0	0	0
Christensenella_massiliensis_Marseille_P2438	0	0	0	-1000	-15.093325	0	0	0	0
Christensenella_minuta_DSM_22607	0	0	0	-1000	-19.443712	0	0	0	0
Christensenellaceae_nov_ERR1022455	0	0	0	0	-28.352757	0	0	0	0
Citrobacter_amalonaticus_FDAARGOS_122	0	0	-1000	-1000	-71.68655	-1000	0	0	0
Citrobacter_amalonaticus_FDAARGOS_166	0	0	-1000	-1000	-59.764922	-1000	0	0	0
Citrobacter_amalonaticus_Y19	0	0	-1000	-1000	-79.575022	-1000	0	-1000	0
Citrobacter_amalonaticus_YG6	0	0	-1000	-1000	-59.76092	-1000	0	0	0
Citrobacter_amalonaticus_YG8	0	0	-1000	-1000	-59.76092	-1000	0	0	0
Citrobacter_freundii_4_7_47CFAA	0	0	-1000	-1000	-57.866297	-1000	0	-1000	0
Citrobacter_freundii_ATCC_8090	0	0	-1000	-1000	-79.292886	-1000	0	-1000	0
Citrobacter_freundii_ERR2221339	0	0	-1000	-1000	-59.439823	-1000	0	-1000	0
Citrobacter_freundii_FDAARGOS_61	0	0	-1000	-1000	-59.76092	-1000	0	-1000	0
Citrobacter_freundii_FDAARGOS_73	0	0	-1000	-1000	-59.443786	-1000	0	-1000	0
Citrobacter_freundii_GTC_09479	0	0	-1000	-1000	-74.457316	-1000	0	-1000	0
Citrobacter_freundii_GTC_09629	0	0	-1000	-1000	-73.728636	-1000	0	-1000	0
Citrobacter_freundii_str_ballerup_7851_39	0	0	-1000	-1000	-74.650961	-1000	0	-1000	0
Citrobacter_freundii_UCI_31	0	0	-1000	-1000	-79.037192	-1000	0	-1000	0
Citrobacter_koseri_ATCC_BAA_895	-1000	0	-1000	-1000	-200	-1000	0	-1000	0
Citrobacter_koseri_FDAARGOS_164	0	0	-1000	-1000	-70.422135	-1000	0	-1000	0
Citrobacter_nov_ERR2221352	0	0	-1000	-1000	-59.439827	-1000	0	-1000	0
Citrobacter_portucalensis_P10159	0	0	-1000	-1000	-50.517346	-1000	0	-1000	0
Citrobacter_rodentium_ICC168	-1000	0	-1000	-1000	-48.318549	-1000	0	0	0
Citrobacter_sp_30_2	0	0	-1000	-1000	-52.529295	-1000	0	0	0
Citrobacter_sp_A1	0	0	-1000	-1000	-59.024166	-1000	0	-1000	0
Citrobacter_sp_KTE151	0	0	-1000	-1000	-59.609345	-1000	0	0	0
Citrobacter_sp_KTE30	0	0	-1000	-1000	-59.285932	-1000	0	-1000	0
Citrobacter_sp_KTE32	0	0	-1000	-1000	-56.944599	-1000	0	0	0
Citrobacter_sp_L17	0	0	-1000	-1000	-59.024166	-1000	0	-1000	0
Citrobacter_youngae_ATCC_29220	0	0	-1000	-1000	-52.472046	-1000	0	-1000	0
Cloacibacillus_evryensis_DSM_19522	0	0	0	-1000	-38.127805	0	0	0	0
Cloacibacterium_normanense_DSM_15886	0	0	0	-12.9006591	0	0	0	0	0
Clostridiaceae_nov_ERR1022373	0	0	0	-46.02123946	-23.621198	0	0	-418.79637	0
Clostridiaceae_nov_ERR2221146	0	0	0	-52.64214765	-27.104469	0	0	-1000	0
Clostridiaceae_nov_ERR2221328	0	0	0	-1000	-47.252538	0	0	-1000	0
Clostridiaceae_nov_ERR2221330	0	0	0	-1000	-37.065901	0	0	0	0
Clostridiaceae_nov_ERR2221331	0	0	0	-1000	-37.065903	0	0	0	0
Clostridiaceae_nov_ERR2221345	0	0	0	-1000	-27.998162	0	0	0	0
Clostridiaceae_nov_ERR2221361	0	0	0	-1000	-27.998146	0	0	0	0
Clostridiaceae_nov_ERR2221383	0	0	0	-51.87864748	-24.230415	-1000	0	0	0
Clostridiaceae_nov_ERR2230154	0	0	0	-45.94201871	-23.338925	0	0	-418.83117	0
Clostridiales_incertae_sedis_nov_ERR1022334	0	0	0	0	-25.788296	0	0	0	0
Clostridiales_incertae_sedis_nov_ERR1022419	0	0	0	-1000	-38.049534	0	0	0	0
Clostridiales_incertae_sedis_nov_ERR2221176	0	0	0	0	-32.778266	0	0	0	0
Clostridiales_incertae_sedis_nov_ERR2221277	0	0	0	-1000	-37.573148	0	0	0	0
Clostridiales_incertae_sedis_nov_ERR2221283	0	0	0	0	-32.778272	0	0	0	0
Clostridiales_incertae_sedis_nov_ERR2221367	0	0	0	-1000	-40.087625	0	0	0	0
Clostridiales_incertae_sedis_nov_ERR2221388	0	0	0	0	-28.129365	0	0	0	0
Clostridiales_sp_1_7_47FAA	0	0	0	-1000	-17.232789	0	0	-571.42857	0
Clostridioides_difficile_002_P50_2011	0	0	0	-1000	-1000	-1000	-1000	-1000	0
Clostridioides_difficile_050_P50_2011	0	0	0	-1000	-1000	-1000	-1000	-1000	0
Clostridioides_difficile_2007855	0	0	0	-80.16045946	-1000	-1000	-1000	-1000	0
Clostridioides_difficile_630	-1000	0	0	-1000	-1000	-1000	-1000	-1000	0
Clostridioides_difficile_6503	0	0	0	-1000	-1000	-1000	-1000	-1000	0
Clostridioides_difficile_70_100_2010	0	0	0	-1000	-1000	-1000	-1000	-1000	0
Clostridioides_difficile_ATCC_43255	0	0	0	-1000	-1000	-1000	-1000	-1000	0
Clostridioides_difficile_ATCC_9689	0	0	0	-1000	-1000	-1000	-1000	-1000	0
Clostridioides_difficile_BI1	-511.6684409	0	0	-1000	-1000	-1000	-1000	-1000	0
Clostridioides_difficile_CD196	-1000	0	0	-1000	-1000	-1000	-1000	0	0
Clostridioides_difficile_CD37	0	0	0	-1000	-1000	-1000	-1000	-1000	0
Clostridioides_difficile_CIP_107932	0	0	0	-1000	-1000	-1000	-1000	-1000	0
Clostridioides_difficile_ERR1204032	0	0	0	-1000	-1000	-1000	-1000	-878.78788	0
Clostridioides_difficile_ERR2221119	0	0	0	-1000	-1000	0	-1000	0	0
Clostridioides_difficile_ERR2221219	0	0	0	-1000	-1000	0	-1000	0	0
Clostridioides_difficile_ERR2221225	0	0	0	-1000	-1000	-1000	-1000	-878.78788	0
Clostridioides_difficile_M120	0	0	0	-1000	-1000	-1000	-1000	-1000	0
Clostridioides_difficile_M68	0	0	0	-1000	-1000	-1000	-1000	-1000	0
Clostridioides_difficile_NAP07	0	0	0	-1000	-1000	-1000	-1000	0	0
Clostridioides_difficile_NAP08	0	0	0	-1000	-1000	-1000	-1000	0	0

Clostridioides_difficile_QCD_23m63	0	0	0	-1000	-1000	-1000	-1000	-1000	0
Clostridioides_difficile_QCD_37x79	0	0	0	-1000	-1000	-1000	-1000	-1000	0
Clostridioides_difficile_QCD_63q42	0	0	0	-1000	-1000	-1000	-1000	-1000	0
Clostridioides_difficile_QCD_66c26	0	0	0	-1000	-1000	-1000	-1000	-1000	0
Clostridioides_difficile_QCD_76w55	0	0	0	-1000	-1000	-1000	-1000	-1000	0
Clostridioides_difficile_QCD_97b34	0	0	0	-1000	-1000	-1000	-1000	-1000	0
Clostridioides_difficile_R20291	-1000	0	0	-1000	-1000	-1000	-1000	0	0
Clostridioides_mangenotii_LM2	0	0	0	-1000	-1000	0	-1000	0	0
Clostridioides_mangenotii_TR	0	0	0	-1000	-1000	0	-1000	0	0
Clostridium_acetobutylicum_ATCC_824	0	0	0	-1000	-26.322539	-1000	0	-1000	0
Clostridium_acetobutylicum_DSM_1731	0	0	0	-1000	-26.362349	-1000	0	-900	0
Clostridium_acetobutylicum_EA_2018	0	0	0	-1000	-26.342713	-1000	0	-900	0
Clostridium_aerotolerans_DSM_5434	0	0	0	-1000	-18.296025	0	0	0	0
Clostridium_amazonitimonense_MGYG_HGUT_0	0	0	0	-1000	-22.151308	0	0	0	0
Clostridium_asparagiforme_DSM_15981	0	0	-1000	-1000	-45.210301	0	0	-1000	0
Clostridium_baratii_796_15	0	0	0	-1000	-22.188532	0	0	-514.48619	0
Clostridium_baratii_ERR1022470	0	0	0	-1000	-44.341624	0	0	-515.05811	0
Clostridium_baratii_str_Sullivan	0	0	0	-1000	-22.188554	0	0	-514.48618	0
Clostridium_bartlettii_DSM_16795	0	0	0	-75.87804038	-1000	-1000	-1000	-534.26178	0
Clostridium_beijerinckii_G117	0	0	0	-1000	-49.947345	-1000	0	-1000	0
Clostridium_beijerinckii_NCIMB_8052	0	0	0	-1000	-38.057576	-1000	0	-1000	0
Clostridium_boliviensis_ERR171272	0	0	0	-1000	-22.911341	0	0	-895.83333	0
Clostridium_bolteae_90A5	0	0	0	-1000	-41.769925	0	0	-955.55556	0
Clostridium_bolteae_90A9	0	0	0	-1000	-39.522402	0	0	-955.55556	0
Clostridium_bolteae_90B3	0	0	0	-1000	-39.522348	0	0	-955.55556	0
Clostridium_bolteae_90B7	0	0	0	-1000	-41.769917	0	0	-955.55556	0
Clostridium_bolteae_90B8	0	0	0	-1000	-41.007378	0	0	-886.93134	0
Clostridium_bolteae_ATCC_BAA_613	0	0	0	-1000	-32.114715	0	0	-1000	0
Clostridium_botulinum_A_str_ATCC_19397	0	0	0	-1000	-1000	0	0	0	0
Clostridium_botulinum_A_str_ATCC_3502	0	0	0	-1000	-1000	0	0	0	0
Clostridium_botulinum_A_str_Hall	0	0	0	-1000	-1000	0	0	0	0
Clostridium_botulinum_A2_str_Kyoto	0	0	0	-1000	-1000	0	0	0	0
Clostridium_botulinum_A3_str_Loch_Maree	0	0	0	-1000	-1000	0	0	0	0
Clostridium_botulinum_B_str_Eklund_17B	-1000	0	0	-1000	-67.977486	0	0	-1000	0
Clostridium_botulinum_B1_str_Okra	0	0	0	-1000	-63.9352	0	0	-1000	0
Clostridium_botulinum_Ba4_str_657	0	0	0	-1000	-63.929953	0	0	-1000	0
Clostridium_botulinum_Bf	0	0	0	-1000	-1000	0	0	-1000	0
Clostridium_botulinum_BKT015925	0	0	0	-1000	-59.925378	0	0	-1000	0
Clostridium_botulinum_C_str_Eklund	0	0	0	-1000	-44.600946	0	0	-889.63211	0
Clostridium_botulinum_CB11_1_1	0	0	0	-1000	-54.677757	0	0	-1000	0
Clostridium_botulinum_CDC66177	0	0	0	-1000	-50.161657	0	0	-1000	0
Clostridium_botulinum_D_str_1873	0	0	0	-1000	-25.29845	0	0	-1000	0
Clostridium_botulinum_E1_str_BoNT_E_Beluga	0	0	0	-1000	-37.135572	0	0	-1000	0
Clostridium_botulinum_E3_str_Alaska_E43	-1000	0	0	-1000	-67.873824	0	0	-1000	0
Clostridium_botulinum_F_str_230613	0	0	0	-1000	-1000	0	0	-916.10054	0
Clostridium_botulinum_F_str_Langeland	0	0	0	-1000	-63.200255	0	0	-1000	0
Clostridium_botulinum_H04402_065	0	0	0	-1000	-63.921462	0	0	-1000	0
Clostridium_botulinum_NCTC_2916	0	0	0	-1000	-68.15963	0	0	-1000	0
Clostridium_butyricum_5521	0	0	0	-1000	-62.966207	0	0	-1000	0
Clostridium_butyricum_60E_3	0	0	0	-1000	-46.896799	0	0	-1000	0
Clostridium_butyricum_DKU_01	0	0	0	-1000	-56.945867	0	0	-1000	0
Clostridium_butyricum_DSM_10702	0	0	0	-1000	-214.32353	0	0	-1000	0
Clostridium_butyricum_E4_str_BoNT_E_BL5262	0	0	0	-1000	-216.50344	0	0	-1000	0
Clostridium_cadaveris_AGR2141	0	0	0	-34.26895808	-1000	0	0	0	0
Clostridium_celatum_DSM_1785	0	0	-1000	-400	-30.123985	0	0	-1000	0
Clostridium_cellobioparum_DSM_1351	0	0	0	0	-22.360521	0	0	0	0
Clostridium_chauvoei_JF4335	0	0	0	-35.33182146	-24.702995	0	0	-753.58005	0
Clostridium_citroniae_WAL_17108	0	0	-1000	-1000	-36.407508	0	0	-887.92802	0
Clostridium_clariflavum_DSM_19732	0	0	0	0	-26.78388	0	0	0	0
Clostridium_clostridioforme_2_1_49FAA	0	0	-1000	-1000	-39.077439	0	0	-944.44444	0
Clostridium_clostridioforme_90A1	0	0	-1000	-1000	-30.559807	0	0	-1000	0
Clostridium_clostridioforme_90A3	0	0	-1000	-1000	-30.559974	0	0	-1000	0
Clostridium_clostridioforme_90A4	0	0	-1000	-1000	-30.559785	0	0	-1000	0
Clostridium_clostridioforme_90A6	0	0	-1000	-1000	-30.559845	0	0	-1000	0
Clostridium_clostridioforme_90A7	0	0	0	-1000	-39.407018	0	0	-955.55556	0
Clostridium_clostridioforme_90A8	0	0	-1000	-1000	-32.579085	0	0	-1000	0
Clostridium_clostridioforme_90B1	0	0	-1000	-1000	-30.559931	0	0	-1000	0
Clostridium_clostridioforme_CM201	0	0	-1000	-1000	-40.643993	0	0	-1000	0
Clostridium_clostridioforme_YL32	0	0	0	-1000	-27.553157	0	0	-757.0179	0
Clostridium_cochlearium_NCTC13027	0	0	0	-1000	-33.612235	0	-1000	0	0
Clostridium_cocleatum_ATCC_29902	0	0	0	-979.8269613	-17.921389	0	0	-508.21632	0
Clostridium_colicanis_209318	0	0	-1000	-67.36038806	-31.233918	0	0	-540.53974	0
Clostridium_colicanis_DSM_13634	0	0	-1000	-892.8571429	-18.925901	-857.33553	0	-428.03339	0
Clostridium_culturomicsense_CL_6	0	0	0	-1000	-17.89476	0	0	0	0
Clostridium_dakarense_FF1	0	0	0	-1000	-17.508302	0	0	-673.87692	0
Clostridium_disporicum_2789STDY5608827	0	0	0	-1000	-19.04374	-1000	0	-1000	0
Clostridium_fimetarium_DSM_9179	0	0	0	0	-17.850489	0	0	0	0
Clostridium_glycolicum_ATCC_14880	0	0	0	-1000	-1000	0	0	0	0
Clostridium_hathewayi_12489931	0	0	-1000	-66.65143281	-32.076955	0	0	-1000	0
Clostridium_hathewayi_DSM_13479	0	0	0	-32.51049152	-20.69043	0	0	-916.66667	0
Clostridium_hathewayi_WAL_18680	0	0	0	-1000	-24.591188	0	0	-916.66667	0
Clostridium_hiranonis_TO_931_DSM_13275	0	0	0	-32.026599	-1000	0	0	-515.38038	0
Clostridium_hylemonae_DSM_15053	0	0	0	-1000	-1000	0	0	-750.75062	0
Clostridium_ihumii_AP5	0	0	0	-1000	-17.296033	0	0	0	0
Clostridium_indolis_DSM_755	0	0	0	-1000	-23.886385	-1000	0	0	0
Clostridium_innocuum_2959	0	0	0	0	-28.282684	-1000	0	-1000	0
Clostridium_innocuum_I46	0	0	0	0	-26.906921	-1000	0	-750	0
Clostridium_isatidis_DSM_15098	0	0	0	-41.66770812	-21.203146	-865.38462	0	-416.66667	0
Clostridium_jeddahense_JCD	0	0	0	-31.56689074	-15.668546	0	0	0	0
Clostridium_jeddahtimonense_CL_2	0	0	0	-30.69967136	-19.660691	0	0	-831.27572	0
Clostridium_lavalense_NLAE_zl_G277	0	0	0	-1000	-18.878904	0	0	-884.65743	0

Clostridium_leptum_DSM_753	0	0	0	-1000	-19.228575	-708.33333	0	0	0
Clostridium_massilioamazoniensis_ND2	0	0	0	-1000	-13.641239	0	0	0	0
Clostridium_methoxybenzovorans_SR3	0	0	0	-1000	-20.630117	0	0	-1000	0
Clostridium_methylpentosum_R2_DSM_5476	0	0	0	0	0	0	0	0	0
Clostridium_nexile_DSM_1787	0	0	0	-1000	-20.831805	0	-1000	0	0
Clostridium_papyrosolvens_C7	0	0	0	0	-16.994033	0	0	0	0
Clostridium_papyrosolvens_DSM_2782	0	0	0	-1000	-22.883115	0	0	0	0
Clostridium_paraputrificum_AGR2156	0	0	0	-49.28941389	-23.01637	0	0	-1000	0
Clostridium_paraputrificum_ERR1022469	0	0	0	-1000	-44.059608	0	0	-1000	0
Clostridium_pasteurianum_BC1	0	0	0	-55.36026048	0	-1000	0	-644.44444	0
Clostridium_pasteurianum_DSM_525_ATCC_601	0	0	0	-52.82981511	-22.177225	-1000	0	-666.66667	0
Clostridium_perfringens_ATCC_13124	-1000	0	0	-1000	-33.762186	0	0	-1000	0
Clostridium_perfringens_B_str_ATCC_3626	0	0	0	-1000	-72.812778	0	0	-1000	0
Clostridium_perfringens_C_str_JGS1495	0	0	-1000	-1000	-65.843444	0	0	-1000	0
Clostridium_perfringens_CPE_str_F4969	0	0	-1000	-1000	-71.650467	0	0	-1000	0
Clostridium_perfringens_D_str_JGS1721	0	0	-1000	-1000	-74.351209	0	0	-1000	0
Clostridium_perfringens_E_str_JGS1987	0	0	-1000	-1000	-93.655131	0	0	-1000	0
Clostridium_perfringens_F262	0	0	-1000	-1000	-73.636501	0	0	-1000	0
Clostridium_perfringens_NCTC_8239	0	0	-1000	-1000	-75.616812	0	0	-1000	0
Clostridium_perfringens_SM101	0	0	0	-1000	-34.778449	0	0	-1000	0
Clostridium_perfringens_str_13	-1000	0	-1000	-1000	-60.39211	0	0	-1000	0
Clostridium_perfringens_WAL_14572	0	0	-1000	-1000	-73.605274	0	0	-1000	0
Clostridium_polynesiense_MS1	0	-99.3485342	0	-17.44098982	-11.416787	-619.26606	0	-310.97561	-95.611285
Clostridium_polysaccharolyticum_DSM_1801	0	0	0	0	-15.201685	0	0	-383.02107	0
Clostridium_ramosum_VPI_0427_DSM_1402	0	0	0	-1000	-20.972597	0	-1000	-1000	0
Clostridium_saccharogumia_DSM_17460	0	0	0	-1000	-18.982374	0	0	-988.09524	0
Clostridium_saccharolyticum_WM1	0	0	0	-1000	-24.09951	-1000	-1000	-751.00402	0
Clostridium_saccharoperbutylacetonicum_N1_4_	-1000	0	0	-1000	-35.963741	-1000	0	-1000	0
Clostridium_sartagoforme_AAU1	0	0	0	-1000	-27.241581	-1000	0	-1000	0
Clostridium_saudiense_JCC	0	0	0	-40.58505398	-19.06341	-1000	0	-1000	0
Clostridium_scindens_ATCC_35704	0	0	0	-1000	-26.721873	0	-1000	0	0
Clostridium_senegalense_JC122	0	0	0	-1000	-17.562599	0	0	0	0
Clostridium_sp_7_2_43FAA	0	0	-1000	-1000	-36.126417	-1000	0	-1000	0
Clostridium_sp_7_3_54FAA	0	0	0	-52.74543915	-24.932434	0	0	-792.89941	0
Clostridium_sp_ASF356	0	0	0	-1000	-21.5428	-1000	0	-918.59792	0
Clostridium_sp_ATCC_BAA_442	0	0	0	-1000	-29.873816	0	0	0	0
Clostridium_sp_BNL1100	0	0	0	0	-13.08582	0	0	0	0
Clostridium_sp_D5	0	0	0	-1000	-27.791615	0	0	-791.66667	0
Clostridium_sp_DL_VIII	0	0	0	-1000	-27.07938	-1000	0	0	0
Clostridium_sp_HGF2	0	0	0	0	-25.475519	-1000	0	-739.58333	0
Clostridium_sp_KLE_1755	0	0	0	-36.90756157	-15.840867	0	0	-750	0
Clostridium_sp_L2_50	0	0	0	-1000	0	0	0	0	0
Clostridium_sp_M62_1	0	0	0	-1000	-18.025257	0	0	0	0
Clostridium_sp_MSTE9	0	0	0	-56.91923021	-27.831259	0	0	0	0
Clostridium_sp_SS2_1	0	0	0	-1000	-11.475578	-573.17073	0	-296.05263	0
Clostridium_sp_SY8519	0	0	0	-1000	-205.09734	0	0	0	0
Clostridium_sphenoides_JCM_1415	0	0	0	-1000	-18.068117	0	-1000	0	0
Clostridium_spiroforme_DSM_1552	0	0	0	0	-20.122415	0	0	-293.04405	0
Clostridium_sporogenes_ATCC_15579	0	0	0	-1000	-1000	0	-1000	0	0
Clostridium_sporogenes_PA_3679	0	0	0	-1000	-1000	0	-1000	0	0
Clostridium_sporosphaeroides_DSM_1294	0	0	0	-50.29479134	-24.252727	0	0	0	0
Clostridium_stercorarium_subsp_leptospartum_	0	0	0	0	-14.596035	0	0	0	0
Clostridium_stercorarium_subsp_stercorarium_D	0	0	-1000	0	-24.275342	0	0	-762.16911	0
Clostridium_stercorarium_subsp_thermolacticum	0	0	0	0	-16.263405	0	0	-761.8054	0
Clostridium_sticklandii_DSM_519	0	0	0	-1000	-1000	0	0	0	0
Clostridium_sulfidigenes_113A_c1	0	0	0	-1000	-1000	0	-1000	0	0
Clostridium_symbiosum_ATCC_14940	0	0	0	-1000	-25.656697	0	-1000	0	0
Clostridium_symbiosum_WAL_14163	0	0	0	-1000	-23.841051	0	-1000	0	0
Clostridium_symbiosum_WAL_14673	0	0	0	-1000	-23.826888	0	-1000	0	0
Clostridium_tertium_Gcol_A43_Gcol_A43_1	0	0	0	-1000	-18.628044	-855.06268	0	-427.68025	0
Clostridium_tyrobutyricum_DSM_2637	0	0	0	-1000	0	-1000	0	0	0
Clostridium_tyrobutyricum_UC7086	0	0	0	-1000	0	-1000	0	0	0
Clostridium_viride_DSM_6836	0	0	0	0	-16.158891	0	0	0	0
Cohnella_laeviribosi_DSM_21336	0	-1000	0	0	-17.988285	-1000	0	-750	-1000
Collinsella_aerofaciens_ATCC_25986	0	0	0	-89.82314223	-35.046274	0	0	-495.19231	0
Collinsella_aerofaciens_ERR1022282	0	0	0	0	-25.149784	0	0	-466.14583	0
Collinsella_aerofaciens_ERR1022300	0	0	0	0	-25.131968	0	0	-466.14583	0
Collinsella_aerofaciens_ERR1022416	0	0	0	0	-25.59717	0	0	-712.99537	0
Collinsella_aerofaciens_ERR1203940	0	0	0	0	-25.131988	0	0	-466.14583	0
Collinsella_aerofaciens_ERR1204034	0	0	0	0	-25.131988	0	0	-466.14583	0
Collinsella_aerofaciens_ERR2221153	0	0	0	0	-25.912783	0	0	-466.14583	0
Collinsella_aerofaciens_ERR2230078	0	0	0	0	-25.551307	0	0	-466.14583	0
Collinsella_aerofaciens_ERR2230087	0	0	0	0	-25.551311	0	0	-466.14583	0
Collinsella_aerofaciens_ERR2230090	0	0	0	0	-25.551314	0	0	-466.14583	0
Collinsella_aerofaciens_ERR2230102	0	0	0	0	-25.551313	0	0	-466.14583	0
Collinsella_aerofaciens_ERR2230112	0	0	0	0	-25.551312	0	0	-466.14583	0
Collinsella_aerofaciens_ERR2230122	0	0	0	0	-25.551317	0	0	-466.14583	0
Collinsella_aerofaciens_ERR2230127	0	0	0	0	-25.551315	0	0	-466.14583	0
Collinsella_aerofaciens_ERR2230140	0	0	0	0	-25.551311	0	0	-466.14583	0
Collinsella_aerofaciens_ERR2230145	0	0	0	0	-25.551309	0	0	-466.14583	0
Collinsella_aerofaciens_ERR2230147	0	0	0	0	-25.551311	0	0	-466.14583	0
Collinsella_aerofaciens_ERR2230156	0	0	0	0	-25.551312	0	0	-466.14583	0
Collinsella_intestinalis_DSM_13280	0	0	0	0	-21.302733	0	0	0	0
Collinsella_massiliensis_An5	0	0	0	0	-14.289171	-1000	0	-534.59205	-1000
Collinsella_sp_4_8_47FAA	0	0	0	0	-25.101611	0	0	-276.10192	0
Collinsella_sp_MS5	0	0	0	0	-25.107054	-1000	0	0	0
Collinsella_stercoris_DSM_13279	0	0	0	0	-26.347872	0	0	0	0
Collinsella_tanakaei_YIT_12063	0	0	-1000	0	-33.163125	-1000	0	-1000	0
Comamonas_aquatica_CJG	0	0	0	-1000	-1000	0	0	0	0
Comamonas_aquatica_DA1877	0	0	0	-1000	-1000	0	0	0	0
Comamonas_aquatica_NBRC_14918	0	0	0	-1000	-1000	0	0	0	0

Comamonas_terrigena_FDAARGOS_394_pRIID_9	0	0	0	-1000	-1000	0	0	0	0
Comamonas_terrigena_NBRC_13299	0	0	0	-1000	-1000	0	0	0	0
Comamonas_testosteroni_ATCC_11996	0	0	0	-1000	-1000	0	0	0	0
Comamonas_testosteroni_CNB_2	-128.2051282	0	0	-1000	-1000	0	0	0	0
Comamonas_testosteroni_KF_1	0	0	0	-1000	-1000	0	0	0	0
Comamonas_testosteroni_NBRC_100989	0	0	0	-1000	-1000	0	0	0	0
Comamonas_testosteroni_S44	0	0	0	-1000	-1000	0	0	0	0
Coprobacillus_cateniformis_29_1	0	0	0	0	-19.202275	0	0	-641.60401	0
Coprobacillus_sp_3_3_56FAA	0	0	0	-1000	-26.496331	0	0	-562.5	0
Coprobacillus_sp_8_2_54BFAA	0	0	0	-1000	-26.796083	0	0	-562.5	0
Coprobacillus_sp_D7	0	0	0	-1000	-22.429604	0	0	-459.88672	0
Coprobacter_fastidiosus_NSB1	0	0	-1000	0	-19.420023	0	0	0	0
Coprococcus_catus_ERR2221258	0	0	0	-1000	-35.798212	-839.54991	0	0	0
Coprococcus_catus_GD_7	0	0	0	-1000	-25.053286	-1000	0	0	0
Coprococcus_comes_ATCC_27758	0	0	0	-75.96593832	0	0	0	-1000	0
Coprococcus_comes_ERR1022290	0	0	0	-57.60214558	-26.85571	0	0	-1000	0
Coprococcus_comes_ERR1022354	0	0	0	-57.60207663	-26.855694	0	0	-1000	0
Coprococcus_comes_ERR1022382	0	0	0	-57.64480765	-26.875226	0	0	-1000	0
Coprococcus_comes_ERR1022427	0	0	0	-57.60218695	-26.855719	0	0	-1000	0
Coprococcus_comes_ERR1022476	0	0	0	-58.08173431	-27.029114	0	0	-1000	0
Coprococcus_comes_ERR1204042	0	0	0	-57.07927032	-26.467955	0	0	-1000	0
Coprococcus_eutactus_ATCC_27759	0	0	0	-1000	-32.616986	0	0	-1000	0
Coprococcus_eutactus_ERR1022287	0	0	0	-50.79968824	-25.911623	0	0	-997.63033	0
Coprococcus_eutactus_ERR1022301	0	0	0	-51.09507491	-26.175411	0	0	-926.13763	0
Coprococcus_eutactus_ERR1022346	0	0	0	-50.79965873	-25.911608	0	0	-997.63033	0
Coprococcus_eutactus_ERR1022477	0	0	0	-53.9778233	-27.651546	0	0	-997.63033	0
Coprococcus_eutactus_ERR1203946	0	0	0	-51.09508739	-26.175417	0	0	-926.13763	0
Coprococcus_eutactus_ERR1204040	0	0	0	-51.09508486	-26.175416	0	0	-926.13763	0
Coprococcus_eutactus_ERR2221154	0	0	0	-45.65756796	-23.291022	0	0	-954.54545	0
Coprococcus_nov_ERR1022278	0	0	0	-1000	-12.95475	0	0	-423.35766	0
Coprococcus_nov_ERR1203948	0	0	0	-42.30149555	-19.430356	0	0	-513.6636	0
Coprococcus_nov_ERR2221394	0	0	0	-42.82462038	-22.04405	0	0	-692.30769	0
Coprococcus_nov_ERR2230098	0	0	0	-1000	-27.020339	0	0	0	0
Coprococcus_sp_HPP0048	0	0	0	0	-21.882674	0	0	0	0
Coprococcus_sp_HPP0074	0	0	0	0	-16.383472	0	0	0	0
Corynebacterium_accolens_AH4003	0	0	0	-1000	-29.280309	0	0	0	0
Corynebacterium_accolens_ATCC_49725	0	0	0	-1000	-22.3164	0	0	0	0
Corynebacterium_ammoniagenes_DSM_20306	0	0	0	-1000	-18.141395	0	0	0	0
Corynebacterium_amycolatum_SK46	0	0	0	-1000	-34.627499	0	0	0	0
Corynebacterium_argentoratense_CNM_46305_c	0	0	0	0	-10.397374	0	0	0	0
Corynebacterium_argentoratense_DSM_44202	0	0	0	0	-10.308465	0	0	0	0
Corynebacterium_aurimucosum_ATCC_700975	0	0	0	-1000	-23.604035	0	0	-1000	0
Corynebacterium_casei_LMG_S_19264	0	0	0	-1000	-12.142787	0	0	0	0
Corynebacterium_casei_UCMA_3821	0	0	0	-1000	-12.137733	0	0	0	0
Corynebacterium_coyleae_UMB0147_16933_8_1	0	0	0	-1000	-13.701798	0	0	0	0
Corynebacterium_durum_F0235	0	0	0	-52.91611661	-21.410235	-1000	0	-596.10215	0
Corynebacterium_efficiens_YS_314	0	0	0	-1000	-29.873594	0	0	0	0
Corynebacterium_glucuronolyticum_ATCC_51866	0	0	0	-1000	-22.031692	0	0	-876.54321	0
Corynebacterium_glucuronolyticum_ATCC_51867	0	0	0	-1000	0	0	0	-861.11111	0
Corynebacterium_ihumii_GD7	0	0	0	-1000	-14.957697	-1000	0	0	0
Corynebacterium_jeddahense_JCB	0	0	0	-1000	-9.8204407	0	0	0	0
Corynebacterium_jeikeium_Cj30184_10	0	0	0	-29.92693835	-10.689034	0	0	0	0
Corynebacterium_kroppenstedtii_DNF00591	0	0	0	-1000	-27.556807	0	0	-665.84767	0
Corynebacterium_kroppenstedtii_DSM_44385	0	0	0	-1000	0	0	0	-958.33333	0
Corynebacterium_mastitidis_DSM_44356	0	0	0	-1000	-15.022081	0	0	-362.46408	0
Corynebacterium_matruchotii_ATCC_14266	0	0	0	-46.75698392	-19.554436	0	0	-532.48811	0
Corynebacterium_matruchotii_ATCC_33806	0	0	0	-46.17308933	-19.350096	0	0	-505.26316	0
Corynebacterium_matruchotii_NCTC10254	0	0	0	-0.888178761	-19.28441	0	0	-507.80764	0
Corynebacterium_propinquum_DSM_44285	0	0	0	-1000	-24.92908	0	0	0	0
Corynebacterium_pseudodiphtheriticum_DSM_4	0	0	0	-1000	-10.660299	0	0	0	0
Corynebacterium_pseudodiphtheriticum_ERR222	0	0	0	-1000	-7.3259383	0	0	0	0
Corynebacterium_pseudogenitalium_ATCC_3303	0	0	0	-1000	-22.428714	0	0	0	0
Corynebacterium_sp_HFH0082	0	0	0	-1000	-29.125029	0	0	-773.80952	0
Corynebacterium_striatum_ATCC_6940	0	0	0	-1000	-37.210595	0	0	-828.94737	0
Corynebacterium_tuberculostearicum_SK141	0	0	0	-1000	-18.022131	0	0	-742.30056	0
Corynebacterium_ulcerans_809	0	0	0	-1000	0	0	0	0	0
Corynebacterium_ulcerans_BR_AD22	0	0	0	-1000	-37.309787	0	0	0	0
Corynebacterium_ureicelerivorans_DSM_45051	0	0	0	-1000	-28.629093	0	0	0	0
Corynebacterium_variabile_DSM_44702	0	0	0	-1000	0	0	0	0	0
Corynebacterium_variabile_Mu292	0	0	0	-1000	0	0	0	0	0
Cronobacter_sakazakii_2151	0	0	-1000	-1000	-53.279751	-1000	-1000	-1000	0
Cronobacter_sakazakii_680	0	0	-1000	-1000	-19.968519	-1000	-1000	-1000	0
Cronobacter_sakazakii_696	0	0	-1000	-1000	-50.516875	-1000	-1000	-1000	0
Cronobacter_sakazakii_701	0	0	-1000	-1000	-36.957587	-1000	-1000	-1000	0
Cronobacter_sakazakii_ATCC_BAA_894	-1000	0	-1000	-1000	-54.539679	-1000	-1000	-1000	0
Cronobacter_sakazakii_E764	0	0	-1000	-1000	-58.414727	-1000	-1000	-1000	0
Cronobacter_sakazakii_ES15	-26.09998227	0	-1000	-1000	-53.70981	-1000	-1000	-1000	0
Cronobacter_sakazakii_ES35	0	0	-1000	-1000	-51.158305	-1000	-1000	-1000	0
Cronobacter_sakazakii_ES713	0	0	-1000	-1000	-53.000776	-1000	-1000	-1000	0
Cronobacter_sakazakii_SP291	-26.10003318	0	-1000	-1000	-53.70981	-1000	-1000	-1000	0
Cryocola_sp_340MFSha3_1	0	0	0	-1000	-12.820874	-1000	0	-639.24845	0
Cryptobacterium_curtum_DSM_15641	0	0	0	-1000	0	0	0	0	0
Cuneatibacter_caecimuris_DSM_29486	0	0	0	0	-15.977038	0	0	0	0
Cupriavidus_metallidurans_CH34	-7.050216106	0	0	-1000	-19.120745	0	0	0	0
Curtobacterium_flaccumfaciens_MEB126	0	0	0	0	-28.8501	-1000	-1000	-1000	0
Curtobacterium_flaccumfaciens_UCD_AKU	0	0	0	0	-23.812308	-1000	-1000	-1000	0
Curvibacter_gracilis_ATCC_BAA_807	0	0	0	-1000	-1000	0	0	0	0
Cutibacterium_acnes_266	0	0	-1000	0	-34.324413	0	0	0	0
Cutibacterium_acnes_6609	0	0	-1000	0	-28.494157	0	0	0	0
Cutibacterium_acnes_ATCC_11828	0	0	-1000	0	-33.193945	0	0	0	0
Cutibacterium_acnes_C1	0	0	-1000	0	-34.390503	0	0	0	0

Cutibacterium_acnes_ERR2221336	0	0	0	0	-36.094266	0	0	0	0
Cutibacterium_acnes_ERR2221340	0	0	0	0	-36.094264	0	0	0	0
Cutibacterium_acnes_ERR2221379	0	0	0	0	-36.094264	0	0	0	0
Cutibacterium_acnes_FZ1_2_0	0	0	-1000	0	-34.324413	0	0	0	0
Cutibacterium_acnes_HL001PA1	0	0	-1000	0	-31.30811	0	0	0	0
Cutibacterium_acnes_HL002PA1	0	0	-1000	0	-30.367005	0	0	0	0
Cutibacterium_acnes_HL002PA2	0	0	-1000	0	-34.324421	0	0	0	0
Cutibacterium_acnes_HL002PA3	0	0	-1000	0	-34.324421	0	0	0	0
Cutibacterium_acnes_HL005PA1	0	0	-1000	0	-31.898813	0	0	0	0
Cutibacterium_acnes_HL005PA2	0	0	-1000	0	-31.924168	0	0	0	0
Cutibacterium_acnes_HL005PA4	0	0	-1000	0	-32.417449	0	0	0	0
Cutibacterium_acnes_HL007PA1	0	0	-1000	0	-34.324429	0	0	0	0
Cutibacterium_acnes_HL013PA1	0	0	-1000	0	-34.278532	0	0	0	0
Cutibacterium_acnes_HL013PA2	0	0	0	0	-29.991617	0	0	0	0
Cutibacterium_acnes_HL020PA1	0	0	-1000	0	-34.324421	0	0	0	0
Cutibacterium_acnes_HL025PA1	0	0	-1000	0	-32.348104	0	0	0	0
Cutibacterium_acnes_HL027PA1	0	0	-1000	0	-34.390503	0	0	0	0
Cutibacterium_acnes_HL027PA2	0	0	-1000	0	-34.324421	0	0	0	0
Cutibacterium_acnes_HL030PA1	0	0	-1000	0	-29.068376	0	0	0	0
Cutibacterium_acnes_HL036PA1	0	0	-1000	0	-34.324421	0	0	0	0
Cutibacterium_acnes_HL036PA2	0	0	-1000	0	-34.324421	0	0	0	0
Cutibacterium_acnes_HL037PA1	0	0	-1000	0	-29.988711	0	0	0	0
Cutibacterium_acnes_HL037PA2	0	0	0	0	-16.280613	0	0	0	0
Cutibacterium_acnes_HL038PA1	0	0	-1000	0	-34.324429	0	0	0	0
Cutibacterium_acnes_HL043PA1	0	0	-1000	0	-34.324429	0	0	0	0
Cutibacterium_acnes_HL043PA2	0	0	-1000	0	-34.324429	0	0	0	0
Cutibacterium_acnes_HL044PA1	0	0	0	0	-15.119276	0	0	0	0
Cutibacterium_acnes_HL045PA1	0	0	-1000	0	-34.324429	0	0	0	0
Cutibacterium_acnes_HL046PA1	0	0	-1000	0	-32.417449	0	0	0	0
Cutibacterium_acnes_HL046PA2	0	0	-1000	0	-34.322514	0	0	0	0
Cutibacterium_acnes_HL050PA1	0	0	-1000	0	-31.993278	0	0	0	0
Cutibacterium_acnes_HL050PA2	0	0	-1000	0	-31.089891	0	0	0	0
Cutibacterium_acnes_HL050PA3	0	0	-1000	0	-34.390503	0	0	0	0
Cutibacterium_acnes_HL053PA1	0	0	-1000	0	-34.324429	0	0	0	0
Cutibacterium_acnes_HL053PA2	0	0	-1000	0	-34.324421	0	0	0	0
Cutibacterium_acnes_HL056PA1	0	0	-1000	0	-34.324429	0	0	0	0
Cutibacterium_acnes_HL059PA1	0	0	-1000	0	-30.001169	0	0	0	0
Cutibacterium_acnes_HL059PA2	0	0	-1000	0	-34.390503	0	0	0	0
Cutibacterium_acnes_HL060PA1	0	0	-1000	0	-32.721162	0	0	0	0
Cutibacterium_acnes_HL063PA1	0	0	-1000	0	-32.348104	0	0	0	0
Cutibacterium_acnes_HL063PA2	0	0	-1000	0	-34.387303	0	0	0	0
Cutibacterium_acnes_HL067PA1	0	0	-1000	0	-34.390503	0	0	0	0
Cutibacterium_acnes_HL072PA1	0	0	-1000	0	-34.324421	0	0	0	0
Cutibacterium_acnes_HL072PA2	0	0	-1000	0	-32.373304	0	0	0	0
Cutibacterium_acnes_HL074PA1	0	0	-1000	0	-34.358543	0	0	0	0
Cutibacterium_acnes_HL078PA1	0	0	-1000	0	-32.348104	0	0	0	0
Cutibacterium_acnes_HL082PA1	0	0	-1000	0	-34.065158	0	0	0	0
Cutibacterium_acnes_HL082PA2	0	0	-1000	0	-33.195282	0	0	0	0
Cutibacterium_acnes_HL083PA1	0	0	-1000	0	-34.324421	0	0	0	0
Cutibacterium_acnes_HL083PA2	0	0	-1000	0	-34.390503	0	0	0	0
Cutibacterium_acnes_HL086PA1	0	0	0	0	-31.373986	0	0	0	0
Cutibacterium_acnes_HL087PA2	0	0	0	0	-31.373986	0	0	0	0
Cutibacterium_acnes_HL087PA3	0	0	-1000	0	-34.390503	0	0	0	0
Cutibacterium_acnes_HL092PA1	0	0	-1000	0	-34.324421	0	0	0	0
Cutibacterium_acnes_HL096PA1	0	0	-1000	0	-32.852035	0	0	0	0
Cutibacterium_acnes_HL096PA2	0	0	-1000	0	-32.85202	0	0	0	0
Cutibacterium_acnes_HL096PA3	0	0	-1000	0	-34.324421	0	0	0	0
Cutibacterium_acnes_HL097PA1	0	0	-1000	0	-32.100718	0	0	0	0
Cutibacterium_acnes_HL099PA1	0	0	-1000	0	-34.324429	0	0	0	0
Cutibacterium_acnes_HL103PA1	0	0	-1000	0	-33.197945	0	0	0	0
Cutibacterium_acnes_HL110PA1	0	0	-1000	0	-29.936361	0	0	0	0
Cutibacterium_acnes_HL110PA2	0	0	-1000	0	-32.348104	0	0	0	0
Cutibacterium_acnes_HL110PA3	0	0	-1000	0	-33.19395	0	0	0	0
Cutibacterium_acnes_HL110PA4	0	0	-1000	0	-29.876071	0	0	0	0
Cutibacterium_acnes_J139	0	0	-1000	0	-33.192613	0	0	0	0
Cutibacterium_acnes_J165	0	0	-1000	0	-34.324413	0	0	0	0
Cutibacterium_acnes_KPA171202	0	0	-1000	0	-42.917618	0	0	0	0
Cutibacterium_acnes_PA_12_1_L1	0	0	0	0	-36.094267	0	0	0	0
Cutibacterium_acnes_PA_12_1_R1	0	0	0	0	-36.094265	0	0	0	0
Cutibacterium_acnes_PA_15_2_L1	0	0	0	0	-36.094257	0	0	0	0
Cutibacterium_acnes_PA_21_1_L1	0	0	0	0	-36.09427	0	0	0	0
Cutibacterium_acnes_PA_30_2_L1	0	0	0	0	-36.094269	0	0	0	0
Cutibacterium_acnes_PRP_38	0	0	-1000	0	-32.452653	0	0	0	0
Cutibacterium_acnes_SK137	0	0	-1000	0	-34.324421	0	0	0	0
Cutibacterium_acnes_SK182	0	0	-1000	0	-34.324421	0	0	0	0
Cutibacterium_acnes_SK187	0	0	-1000	0	-34.318683	0	0	0	0
Cutibacterium_acnes_TypeIA2_P_acn17	0	0	-1000	0	-34.390503	0	0	0	0
Cutibacterium_acnes_TypeIA2_P_acn31	0	0	-1000	0	-33.944451	0	0	0	0
Cutibacterium_acnes_TypeIA2_P_acn33	0	0	-1000	0	-34.390493	0	0	0	0
Cutibacterium_granulosum_TM11	0	0	0	0	-12.967056	0	0	0	0
Dakarella_massiliensis_ND3	0	0	0	-1000	-8.9687013	0	0	0	0
Dechlorosoma_suillum_PS	0	0	0	-44.49030368	-1000	0	0	0	0
Dehalobacterium_formicoaceticum_DMC	0	0	0	0	-7.185801	0	0	0	0
Delftia_acidovorans_CCUG_15835	0	0	0	-1000	-1000	-1000	-1000	0	0
Delftia_acidovorans_CCUG_274B	0	0	0	-1000	-1000	-1000	-1000	0	0
Delftia_acidovorans_SPH_1	-1000	0	0	-1000	-1000	-1000	-1000	0	0
Dermabacter_sp_HFH0086	0	0	0	-57.04245227	0	0	0	0	0
Dermacoccus_nishinomiyaensis_DSM_20448	0	0	0	0	-30.602227	0	0	0	0
Dermacoccus_sp_Ellin185	0	0	0	0	0	0	0	0	0
Desmospora_sp_8437	0	0	0	-1000	0	-833.33333	0	0	0
Desulfitobacterium_hafniense_DCB_2	0	0	0	0	-43.142482	0	-1000	0	0

Desulfitobacterium_hafniense_DP7	0	0	0	0	-42.803131	0	-1000	0	0
Desulfitobacterium_hafniense_PCP_1	0	0	0	-85.87533201	-43.795319	0	-1000	0	0
Desulfitobacterium_hafniense_Y51	0	0	0	0	-42.808521	0	-1000	0	0
Desulfomicrobium_orale_DSM_12838	0	0	0	-31.66732623	-16.67032	0	-1000	0	0
Desulfovibrio_desulfuricans_subsp_aestuarii_DSM	0	0	0	-1000	-28.46622	0	-1000	0	0
Desulfovibrio_desulfuricans_subsp_desulfuricans	0	0	0	-1000	-32.631397	0	-1000	0	0
Desulfovibrio_desulfuricans_subsp_desulfuricans	0	0	0	-1000	-35.30712	0	-1000	0	0
Desulfovibrio_desulfuricans_subsp_desulfuricans	0	0	0	0	-23.923236	0	-1000	0	0
Desulfovibrio_legallii_KHC7	0	0	0	-1000	-18.758343	0	0	0	0
Desulfovibrio_piger_ATCC_29098	0	0	0	-1000	-15.323405	0	-1000	0	0
Desulfovibrio_sp_3_1_syn3	0	0	0	-1000	-22.687396	0	-1000	0	0
Desulfovibrio_sp_6_1_46AFAA	0	0	0	-1000	-32.605505	0	0	0	0
Dialister_invisus_DSM_15470	0	0	0	-1000	-3.320738	0	0	0	0
Dialister_micraerophilus_DSM_19965	0	0	0	-1000	-1.8481058	0	0	0	0
Dialister_microaerophilus_UPII_345_E	0	0	0	-1000	-4.2609393	0	0	0	0
Dialister_pneumosintes_F0677	0	0	0	-1000	-7.7536958	0	0	0	0
Dialister_succinatiphilus_YIT_11850	0	0	0	-1000	-24.859816	0	0	0	0
Dielma_fastidiosa_JC13	0	0	0	0	-18.088327	0	0	0	0
Dietzia_cinnamea_NBRC_102147	0	0	0	0	-1000	0	0	0	0
Dietzia_cinnamea_P4	0	0	0	0	0	0	0	0	0
Dolosigranulum_pigrum_ATCC_51524	0	0	0	-32.41580294	-32.018607	-1000	0	-583.33333	0
Dolosigranulum_pigrum_KPL1931_CDC4294_98	0	0	0	-29.80014691	-29.435001	0	0	-671.42857	0
Dorea_formicigenerans_4_6_53AFAA	0	0	0	-1000	-23.074935	0	0	-964.28571	0
Dorea_formicigenerans_ATCC_27755	0	0	0	-1000	-21.7679	0	0	0	0
Dorea_formicigenerans_ERR1022384	0	0	0	-51.46339332	-22.601098	0	0	0	0
Dorea_formicigenerans_ERR1203975	0	0	0	-45.09930871	-20.041082	0	0	-515.95745	0
Dorea_formicigenerans_ERR1204069	0	0	0	-45.09931627	-20.041086	0	0	-515.95745	0
Dorea_formicigenerans_ERR2221255	0	0	0	-56.94477319	-26.79856	0	0	0	0
Dorea_formicigenerans_ERR2221257	0	0	0	-56.94477319	-26.79856	0	0	0	0
Dorea_formicigenerans_ERR2221275	0	0	0	-56.94478555	-26.91858	0	0	-1000	0
Dorea_formicigenerans_ERR2230157	0	0	0	-56.94478555	-26.91858	0	0	-1000	0
Dorea_longicatena_DSM_13814	0	0	0	-1000	0	0	0	-1000	0
Dorea_longicatena_ERR1022309	0	0	0	-53.35120482	-26.536786	0	0	-1000	0
Dorea_longicatena_ERR1022324	0	0	0	-1000	-25.596429	0	0	-1000	0
Dorea_longicatena_ERR1022383	0	0	-1000	-1000	-27.843981	0	0	-1000	0
Dorea_longicatena_ERR1022428	0	0	0	-51.67096114	-24.484909	0	0	-1000	0
Dorea_longicatena_ERR1022475	0	0	0	-53.21236159	-26.542077	0	0	-1000	0
Dorea_longicatena_ERR1203969	0	0	0	-53.35119315	-26.536778	0	0	-1000	0
Dorea_longicatena_ERR1204063	0	0	0	-53.35120482	-26.536786	0	0	-1000	0
Dorea_longicatena_ERR2221186	0	0	0	-1000	-25.459273	0	0	-1000	0
Dorea_longicatena_ERR2221271	0	0	0	-43.27492332	-20.110008	0	0	-1000	0
Dorea_longicatena_ERR2230069	0	0	0	-43.27491151	-20.110002	0	0	-1000	0
Dorea_nov_ERR2230118	0	0	0	-38.94179943	-17.728964	0	0	-599.61853	0
Dyadobacter_beijingensis_DSM_21582	0	0	-1000	-1000	0	-1000	0	-1000	0
Dyadobacter_fermentans_DSM_18053	0	0	0	-1000	0	-1000	0	-1000	0
Dysgonomonas_gadei_ATCC_BAA_286	0	0	-1000	-1000	0	0	0	-1000	0
Dysgonomonas_mossii_DSM_22836	0	0	-1000	-1000	-35.602625	-1000	0	-1000	0
Edwardsiella_tarda_080813	0	0	-1000	-1000	-26.429476	-1000	0	0	0
Edwardsiella_tarda_ATCC_23685	0	0	-1000	-1000	-48.949998	0	0	0	0
Edwardsiella_tarda_C07_087	0	0	-1000	-1000	-52.007419	0	0	0	0
Edwardsiella_tarda_DT	0	0	-1000	-1000	-43.249753	0	0	0	0
Edwardsiella_tarda_EIB202	0	0	-1000	-1000	-43.065219	0	0	0	0
Edwardsiella_tarda_FL6_60	0	0	-1000	-1000	-200	0	0	0	0
Edwardsiella_tarda_FL95_01	0	0	-1000	-1000	-38.557937	0	0	0	0
Edwardsiella_tarda_NBRC_105688	0	0	-1000	-1000	-43.268859	0	0	0	0
Eggerthella_lenta_1160AFAA	0	0	0	-1000	-41.116175	0	0	0	0
Eggerthella_lenta_11C	0	0	0	-1000	-41.115681	0	0	0	0
Eggerthella_lenta_1356FAA	0	0	0	-1000	0	0	0	0	0
Eggerthella_lenta_14A	0	0	0	-1000	-41.115644	0	0	0	0
Eggerthella_lenta_16A	0	0	0	-1000	-41.115644	0	0	0	0
Eggerthella_lenta_19C	0	0	0	-1000	-41.055324	0	0	0	0
Eggerthella_lenta_22C	0	0	0	-1000	-41.055324	0	0	0	0
Eggerthella_lenta_28B	0	0	0	-1000	-43.060138	0	0	0	0
Eggerthella_lenta_326I6NA	0	0	0	-1000	-41.11578	0	0	0	0
Eggerthella_lenta_A2	0	0	0	-1000	-40.726477	0	0	0	0
Eggerthella_lenta_AB12n2	0	0	0	-1000	-41.115761	0	0	0	0
Eggerthella_lenta_AB8n2	0	0	0	-1000	-41.493952	0	0	0	0
Eggerthella_lenta_AN51LG	0	0	0	-1000	-43.060025	0	0	0	0
Eggerthella_lenta_ATCC_25559	0	0	0	-1000	-41.299716	0	0	0	0
Eggerthella_lenta_C592	0	0	0	-1000	-41.115632	0	0	0	0
Eggerthella_lenta_CC75D52	0	0	0	-1000	-43.511001	0	0	0	0
Eggerthella_lenta_CC82BHI2	0	0	0	-1000	-41.055117	0	0	0	0
Eggerthella_lenta_CC86D54	0	0	0	-1000	-41.055236	0	0	0	0
Eggerthella_lenta_DSM_11767	0	0	0	-1000	-41.115741	0	0	0	0
Eggerthella_lenta_DSM_11863	0	0	0	-1000	-43.683943	0	0	0	0
Eggerthella_lenta_DSM_15644	0	0	0	-1000	-41.115689	0	0	0	0
Eggerthella_lenta_DSM_2243	0	0	0	0	0	0	0	0	0
Eggerthella_lenta_HGA1	0	0	0	-1000	-41.055268	0	0	0	0
Eggerthella_lenta_MR1n12	0	0	0	-1000	-41.055199	0	0	0	0
Eggerthella_lenta_RC46F	0	0	0	-1000	-41.115685	0	0	0	0
Eggerthella_lenta_Valencia	0	0	0	-1000	-41.115732	0	0	0	0
Eggerthella_lenta_W1BHI6	0	0	0	-1000	-41.055302	0	0	0	0
Eggerthella_sinensis_DSM_16107	0	0	0	-1000	-15.721595	0	0	0	0
Eggerthella_sp_1_3_56FAA	0	0	0	-1000	-24.445609	0	0	0	0
Eggerthella_sp_YY7918	0	0	0	-1000	0	0	0	0	0
Eggerthia_catenaformis_OT_569	0	0	0	-35.61454353	-20.836271	0	0	-667.43057	0
Eikenella_corrodens_ATCC_23834	0	0	0	-1000	-12.273342	0	0	0	0
Eisenbergiella_nov_ERR2221109	0	0	0	-65.88901323	-33.251152	0	0	-875	0
Eisenbergiella_nov_ERR2221178	0	0	0	-65.82893926	-33.228339	0	0	-875	0
Eisenbergiella_tayi_ERR1022439	0	0	0	-52.71292337	-26.900779	0	0	-750	0
Eisenbergiella_tayi_NML110678	0	0	0	0	-15.939532	0	0	-887.44142	0

Elusimicrobium_minutum_Pei191	0	0	0	0	-17.001	0	0	0	0
Enhydrobacter_aerosaccus_ATCC_27094	0	0	0	-1000	-1000	0	-1000	-1000	0
Enorma_massiliensis_ERR1022365	0	0	0	0	-26.792496	0	0	0	0
Enorma_timonensis_GD5	0	0	0	0	-15.58574	-1000	0	0	0
Enterobacter_aerogenes_EA1509E	-1000	0	-1000	-1000	-57.948435	-1000	0	-1000	0
Enterobacter_aerogenes_ERR2221162	0	0	-1000	-1000	-38.478653	-1000	0	-1000	0
Enterobacter_aerogenes_FGI35	0	0	-1000	-1000	-57.992927	-1000	0	-1000	0
Enterobacter_aerogenes_KCTC_2190	-1000	0	-1000	-1000	-60.338147	-1000	0	-1000	0
Enterobacter_asburiae_L1	0	0	-1000	-1000	-41.012247	-1000	0	-1000	0
Enterobacter_asburiae_LF7a	-1000	0	-1000	-1000	-85.200256	-1000	0	-1000	0
Enterobacter_cancerogenus_ATCC_35316	0	0	-1000	-1000	-43.712583	-1000	0	0	0
Enterobacter_cloacae_EcWSU1	-1000	0	-1000	-1000	-79.955716	-1000	0	-1000	0
Enterobacter_cloacae_ERR2221114	0	0	-1000	-1000	-61.437568	-1000	0	-1000	0
Enterobacter_cloacae_ERR2221117	0	0	-1000	-1000	-47.567275	-1000	0	-1000	0
Enterobacter_cloacae_ERR2221156	0	0	-1000	-1000	-61.532884	-1000	0	-1000	0
Enterobacter_cloacae_ERR2221157	0	0	-1000	-1000	-61.437572	-1000	0	-1000	0
Enterobacter_cloacae_ERR2221194	0	0	-1000	-1000	-47.567262	-1000	0	-1000	0
Enterobacter_cloacae_GGT036	0	0	-1000	-1000	-61.532884	-1000	0	-1000	0
Enterobacter_cloacae_subsp_cloacae_08XA1	0	0	-1000	-1000	-81.844056	-1000	0	-1000	0
Enterobacter_cloacae_subsp_cloacae_ATCC_1304	-1000	0	-1000	-1000	-84.29916	-1000	0	-1000	0
Enterobacter_cloacae_subsp_cloacae_ENHKU01	0	0	-1000	-1000	-83.520218	-1000	0	-1000	0
Enterobacter_cloacae_subsp_cloacae_GS1	0	0	-1000	-1000	-39.462001	-1000	0	-1000	0
Enterobacter_cloacae_subsp_dissolvens_SDM	-1000	0	-1000	-1000	-84.173099	-1000	0	-1000	0
Enterobacter_hormaechei_ATCC_49162	0	0	-1000	-1000	-73.669108	-1000	0	-1000	0
Enterobacter_hormaechei_FDAARGOS_68	0	0	-1000	-1000	-47.370853	-1000	0	-1000	0
Enterobacter_hormaechei_YT2	0	0	-1000	-1000	-78.642893	-1000	0	-1000	0
Enterobacter_hormaechei_YT3	0	0	-1000	-1000	-78.642893	-1000	0	-1000	0
Enterobacter_lignolyticus_G5	0	0	-1000	-1000	-39.061659	-1000	0	0	0
Enterobacter_lignolyticus_SCF1	0	0	-1000	-1000	-53.019297	-1000	0	0	0
Enterobacter_ludwigii_UW5	0	0	-1000	-1000	-61.063026	-1000	0	-1000	0
Enterobacter_mori_800721_17	0	0	-1000	-1000	-61.063026	-1000	0	0	0
Enterobacter_mori_LMG_25706	0	0	-1000	-1000	-59.602487	-1000	0	0	0
Enterobacter_nov_ERR2221249	0	0	-1000	-1000	-60.459737	-1000	0	0	0
Enterobacter_nov_ERR2221289	0	0	-1000	-1000	-41.248152	-1000	0	0	0
Enterobacter_nov_ERR2221350	0	0	-1000	-1000	-41.248152	-1000	0	0	0
Enterobacter_nov_ERR2221354	0	0	-1000	-1000	-38.48552	-1000	0	-1000	0
Enterobacter_roggenkampii_35734	0	0	-1000	-1000	-39.064462	-1000	0	-1000	0
Enterobacter_sp_MGH_8	0	0	-1000	-1000	-47.439398	-1000	0	0	0
Enterobacteriaceae_bacterium_9_2_54FAA	0	0	-1000	-1000	-43.93534	-1000	0	-1000	0
Enterococcus_asini_ATCC_700915	0	0	0	0	-25.7038	0	0	0	0
Enterococcus_avium_ATCC_14025	0	-1000	0	-1000	-26.497059	-1000	-1000	-1000	0
Enterococcus_caccae_ATCC_BAA_1240	0	0	0	0	-25.696691	0	-1000	-1000	0
Enterococcus_casseliflavus_ATCC_12755	0	0	0	-1000	-25.326765	-1000	0	-1000	0
Enterococcus_casseliflavus_EC10	0	0	0	-1000	-41.813788	-1000	0	-1000	0
Enterococcus_casseliflavus_EC20	0	0	0	-1000	-52.214335	-1000	0	-1000	0
Enterococcus_casseliflavus_EC30	0	0	0	-1000	-22.297092	-1000	0	-764.94164	0
Enterococcus_cecorum_DSM_20682	0	0	-1000	0	-26.583421	0	-1000	-1000	0
Enterococcus_dispar_ATCC_51266	0	0	0	-1000	-24.384307	0	-1000	0	0
Enterococcus_durans_ATCC_6056	0	0	0	0	-30.734351	0	0	0	0
Enterococcus_durans_ERR2230121	0	0	0	0	-32.217122	0	0	0	0
Enterococcus_durans_FB129_CNAB_4	0	0	0	0	-25.614729	-1000	0	0	0
Enterococcus_durans_IPLA_655	0	0	0	-54.37583417	-36.239336	0	0	0	0
Enterococcus_faecalis_12030	0	0	0	-600	-24.11716	-1000	-1000	-1000	0
Enterococcus_faecalis_12107	0	0	0	-600	-23.244921	-1000	-1000	-1000	0
Enterococcus_faecalis_1448E03	0	0	0	-600	-21.88944	-1000	-1000	-1000	0
Enterococcus_faecalis_182970	0	0	0	-600	-23.170054	-1000	-1000	-1000	0
Enterococcus_faecalis_19116	0	0	0	-600	-21.889468	-1000	-1000	-1000	0
Enterococcus_faecalis_2630V05	0	0	0	-600	-23.170054	-1000	-1000	-1000	0
Enterococcus_faecalis_2924	0	0	0	-600	-23.170054	-1000	-1000	-1000	0
Enterococcus_faecalis_5952	0	0	0	-600	-23.23808	-1000	-1000	-1000	0
Enterococcus_faecalis_599	0	0	0	-604.3956044	-37.542987	-1000	-1000	-1000	0
Enterococcus_faecalis_599951	0	0	0	-600	-23.988656	-1000	-1000	-1000	0
Enterococcus_faecalis_62	0	0	0	-600	-26.739001	-1000	-1000	-1000	0
Enterococcus_faecalis_7330082_2	0	0	0	-600	-23.276242	-1000	-1000	-1000	0
Enterococcus_faecalis_7330112_3	0	0	0	-600	-23.483482	-1000	-1000	-1000	0
Enterococcus_faecalis_7330245_2	0	0	0	-600	-21.211725	-1000	-1000	-1000	0
Enterococcus_faecalis_7330257_1	0	0	0	-1000	-23.039866	-1000	-1000	-1000	0
Enterococcus_faecalis_7330259_5	0	0	0	-600	-21.889468	-1000	-1000	-1000	0
Enterococcus_faecalis_7330948_5	0	0	0	-600	-21.892589	-1000	-1000	-1000	0
Enterococcus_faecalis_7430275_3	0	0	0	-600	-21.892619	-1000	-1000	-1000	0
Enterococcus_faecalis_7430315_3	0	0	0	-600	-21.211687	-1000	-1000	-1000	0
Enterococcus_faecalis_7430416_3	0	0	0	-600	-21.892601	-1000	-1000	-1000	0
Enterococcus_faecalis_7430821_4	0	0	0	-600	-21.892609	-1000	-1000	-1000	0
Enterococcus_faecalis_79_3	0	0	0	-600	-24.11716	-1000	-1000	-1000	0
Enterococcus_faecalis_A_2_1	0	0	0	-600	-23.49218	-1000	-1000	-1000	0
Enterococcus_faecalis_A_3_1	0	0	0	-600	-23.244935	-1000	-1000	-1000	0
Enterococcus_faecalis_AR01_DG	0	0	0	-600	-24.356875	-1000	-1000	-1000	0
Enterococcus_faecalis_ATCC_10100	0	0	0	-604.3956044	-37.594714	-1000	-1000	-1000	0
Enterococcus_faecalis_ATCC_19433	0	0	0	-604.3956044	-37.701026	-1000	-1000	-1000	0
Enterococcus_faecalis_ATCC_27275	0	0	0	-604.3956044	-37.594714	-1000	-1000	-1000	0
Enterococcus_faecalis_ATCC_27959	0	0	0	-604.3956044	-37.594438	-1000	-1000	-1000	0
Enterococcus_faecalis_ATCC_29200	0	0	0	-604.3956044	-37.588565	-1000	-1000	-1000	0
Enterococcus_faecalis_ATCC_29212	0	0	0	-604.3956044	-37.589628	-1000	-1000	-1000	0
Enterococcus_faecalis_ATCC_35038	0	0	0	-604.3956044	-37.612837	-1000	-1000	-1000	0
Enterococcus_faecalis_ATCC_4200	0	0	0	-604.3956044	-37.583113	-1000	-1000	-1000	0
Enterococcus_faecalis_ATCC_6055	0	0	0	-604.3956044	-37.594714	-1000	-1000	-1000	0
Enterococcus_faecalis_B_4_111	0	0	0	-600	-21.892629	-1000	-1000	-1000	0
Enterococcus_faecalis_B1005	0	0	0	-533.3333333	-23.23808	-1000	-1000	-1000	0
Enterococcus_faecalis_B1138	0	0	0	-533.3333333	-23.238092	-1000	-1000	-1000	0
Enterococcus_faecalis_B1249	0	0	0	-533.3333333	-23.238068	-1000	-1000	-1000	0
Enterococcus_faecalis_B1290	0	0	0	-533.3333333	-23.23808	-1000	-1000	-1000	0

Enterococcus_faecalis_B1327		0	0	0	-533.3333333	-23.23808	-1000	-1000	-1000	0
Enterococcus_faecalis_B1376		0	0	0	-533.3333333	-23.238092	-1000	-1000	-1000	0
Enterococcus_faecalis_B1385		0	0	0	-533.3333333	-23.23808	-1000	-1000	-1000	0
Enterococcus_faecalis_B1441		0	0	0	-533.3333333	-23.238068	-1000	-1000	-1000	0
Enterococcus_faecalis_B1505		0	0	0	-533.3333333	-23.238057	-1000	-1000	-1000	0
Enterococcus_faecalis_B1532		0	0	0	-533.3333333	-23.238092	-1000	-1000	-1000	0
Enterococcus_faecalis_B15725		0	0	0	-533.3333333	-23.23808	-1000	-1000	-1000	0
Enterococcus_faecalis_B1586		0	0	0	-533.3333333	-23.238045	-1000	-1000	-1000	0
Enterococcus_faecalis_B1618		0	0	0	-533.3333333	-23.238092	-1000	-1000	-1000	0
Enterococcus_faecalis_B1623		0	0	0	-533.3333333	-23.23808	-1000	-1000	-1000	0
Enterococcus_faecalis_B16457		0	0	0	-600	-21.892619	-1000	-1000	-1000	0
Enterococcus_faecalis_B1678		0	0	0	-533.3333333	-31.78497	-1000	-1000	-1000	0
Enterococcus_faecalis_B1696		0	0	0	-533.3333333	-23.238036	-1000	-1000	-1000	0
Enterococcus_faecalis_B1719		0	0	0	-533.3333333	-23.23808	-1000	-1000	-1000	0
Enterococcus_faecalis_B1734		0	0	0	-533.3333333	-23.23808	-1000	-1000	-1000	0
Enterococcus_faecalis_B1843		0	0	0	-533.3333333	-23.23808	-1000	-1000	-1000	0
Enterococcus_faecalis_B1851		0	0	0	-533.3333333	-23.23808	-1000	-1000	-1000	0
Enterococcus_faecalis_B1874		0	0	0	-533.3333333	-23.238068	-1000	-1000	-1000	0
Enterococcus_faecalis_B1921		0	0	0	-600	-23.244877	-1000	-1000	-1000	0
Enterococcus_faecalis_B1933		0	0	0	-533.3333333	-23.238092	-1000	-1000	-1000	0
Enterococcus_faecalis_B2202		0	0	0	-533.3333333	-23.23808	-1000	-1000	-1000	0
Enterococcus_faecalis_B2207		0	0	0	-600	-23.244889	-1000	-1000	-1000	0
Enterococcus_faecalis_B2211		0	0	0	-533.3333333	-23.23808	-1000	-1000	-1000	0
Enterococcus_faecalis_B2255		0	0	0	-533.3333333	-23.238068	-1000	-1000	-1000	0
Enterococcus_faecalis_B2277		0	0	0	-533.3333333	-23.23808	-1000	-1000	-1000	0
Enterococcus_faecalis_B2391		0	0	0	-533.3333333	-23.23808	-1000	-1000	-1000	0
Enterococcus_faecalis_B2488		0	0	0	-533.3333333	-23.238092	-1000	-1000	-1000	0
Enterococcus_faecalis_B2535		0	0	0	-533.3333333	-23.23808	-1000	-1000	-1000	0
Enterococcus_faecalis_B2557		0	0	0	-533.3333333	-23.23808	-1000	-1000	-1000	0
Enterococcus_faecalis_B2593		0	0	0	-533.3333333	-23.238011	-1000	-1000	-1000	0
Enterococcus_faecalis_B2670		0	0	0	-533.3333333	-23.238068	-1000	-1000	-1000	0
Enterococcus_faecalis_B2685		0	0	0	-533.3333333	-23.238092	-1000	-1000	-1000	0
Enterococcus_faecalis_B2687		0	0	0	-533.3333333	-23.238068	-1000	-1000	-1000	0
Enterococcus_faecalis_B2802		0	0	0	-533.3333333	-23.238092	-1000	-1000	-1000	0
Enterococcus_faecalis_B2864		0	0	0	-533.3333333	-23.23808	-1000	-1000	-1000	0
Enterococcus_faecalis_B2867		0	0	0	-533.3333333	-23.238092	-1000	-1000	-1000	0
Enterococcus_faecalis_B2949		0	0	0	-533.3333333	-23.23808	-1000	-1000	-1000	0
Enterococcus_faecalis_B3031		0	0	0	-533.3333333	-23.238103	-1000	-1000	-1000	0
Enterococcus_faecalis_B3042		0	0	0	-533.3333333	-23.238068	-1000	-1000	-1000	0
Enterococcus_faecalis_B3053		0	0	0	-533.3333333	-23.238092	-1000	-1000	-1000	0
Enterococcus_faecalis_B3119		0	0	0	-533.3333333	-23.238092	-1000	-1000	-1000	0
Enterococcus_faecalis_B3126		0	0	0	-533.3333333	-23.238092	-1000	-1000	-1000	0
Enterococcus_faecalis_B3196		0	0	0	-533.3333333	-23.238092	-1000	-1000	-1000	0
Enterococcus_faecalis_B3286		0	0	0	-533.3333333	-23.238103	-1000	-1000	-1000	0
Enterococcus_faecalis_B3336		0	0	0	-533.3333333	-23.238045	-1000	-1000	-1000	0
Enterococcus_faecalis_B4008		0	0	0	-533.3333333	-23.238068	-1000	-1000	-1000	0
Enterococcus_faecalis_B4018		0	0	0	-533.3333333	-23.237967	-1000	-1000	-1000	0
Enterococcus_faecalis_B4148		0	0	0	-533.3333333	-23.23808	-1000	-1000	-1000	0
Enterococcus_faecalis_B4163		0	0	0	-533.3333333	-23.23808	-1000	-1000	-1000	0
Enterococcus_faecalis_B4259		0	0	0	-533.3333333	-23.23808	-1000	-1000	-1000	0
Enterococcus_faecalis_B4267		0	0	0	-533.3333333	-23.238092	-1000	-1000	-1000	0
Enterococcus_faecalis_B4270		0	0	0	-533.3333333	-23.23808	-1000	-1000	-1000	0
Enterococcus_faecalis_B4411		0	0	0	-533.3333333	-23.238092	-1000	-1000	-1000	0
Enterococcus_faecalis_B4568		0	0	0	-533.3333333	-23.238092	-1000	-1000	-1000	0
Enterococcus_faecalis_B4638		0	0	0	-533.3333333	-23.23808	-1000	-1000	-1000	0
Enterococcus_faecalis_B4672		0	0	0	-533.3333333	-23.238092	-1000	-1000	-1000	0
Enterococcus_faecalis_B4674		0	0	0	-533.3333333	-23.238023	-1000	-1000	-1000	0
Enterococcus_faecalis_B4969		0	0	0	-533.3333333	-23.238103	-1000	-1000	-1000	0
Enterococcus_faecalis_B5035		0	0	0	-533.3333333	-23.23808	-1000	-1000	-1000	0
Enterococcus_faecalis_B5076		0	0	0	-533.3333333	-23.238068	-1000	-1000	-1000	0
Enterococcus_faecalis_B56765		0	0	0	-600	-23.49832	-1000	-1000	-1000	0
Enterococcus_faecalis_B594		0	0	0	-533.3333333	-23.238092	-1000	-1000	-1000	0
Enterococcus_faecalis_B653		0	0	0	-600	-23.483482	-1000	-1000	-1000	0
Enterococcus_faecalis_B69486		0	0	0	-533.3333333	-23.244852	-1000	-1000	-1000	0
Enterococcus_faecalis_B84847		0	0	0	-600	-22.734817	-1000	-1000	-1000	0
Enterococcus_faecalis_B878		0	0	0	-533.3333333	-23.238092	-1000	-1000	-1000	0
Enterococcus_faecalis_B939		0	0	0	-533.3333333	-23.238092	-1000	-1000	-1000	0
Enterococcus_faecalis_C19315WT		0	0	0	-600	-27.792737	-1000	-1000	-1000	0
Enterococcus_faecalis_CH116		0	0	0	-600	-23.492189	-1000	-1000	-1000	0
Enterococcus_faecalis_CH136		0	0	0	-600	-23.492189	-1000	-1000	-1000	0
Enterococcus_faecalis_CH188		0	0	0	-604.3956044	-37.588892	-1000	-1000	-1000	0
Enterococcus_faecalis_CH19		0	0	0	-600	-23.492198	-1000	-1000	-1000	0
Enterococcus_faecalis_CH570		0	0	0	-533.3333333	-23.238036	-1000	-1000	-1000	0
Enterococcus_faecalis_Com_2		0	0	0	-600	-21.892629	-1000	-1000	-1000	0
Enterococcus_faecalis_Com_6		0	0	0	-600	-22.493952	-1000	-1000	-1000	0
Enterococcus_faecalis_Com1		0	0	0	-600	-21.889488	-1000	-1000	-1000	0
Enterococcus_faecalis_Com7		0	0	0	-600	-24.127875	-1000	-1000	-1000	0
Enterococcus_faecalis_D1		0	0	0	-600	-23.276242	-1000	-1000	-1000	0
Enterococcus_faecalis_D173		0	0	0	-600	-22.361253	-1000	-1000	-1000	0
Enterococcus_faecalis_D3		0	0	0	-600	-21.211715	-1000	-1000	-1000	0
Enterococcus_faecalis_D32		0	0	0	-604.3956044	-37.588749	-1000	-1000	-1000	0
Enterococcus_faecalis_D6		0	0	0	-604.3956044	-37.41049	-1000	-1000	-1000	0
Enterococcus_faecalis_DAPTO_512		0	0	0	-604.3956044	-37.589806	-1000	-1000	-1000	0
Enterococcus_faecalis_DAPTO_516		0	0	0	-604.3956044	-25.113981	-1000	-1000	-1000	0
Enterococcus_faecalis_DS16		0	0	0	-600	-23.244944	-1000	-1000	-1000	0
Enterococcus_faecalis_DS5		0	0	0	-600	-25.365286	-1000	-1000	-1000	0
Enterococcus_faecalis_E1		0	0	0	-600	-23.279714	-1000	-1000	-1000	0
Enterococcus_faecalis_E1Sol		0	0	0	-604.3956044	-24.998861	-1000	-1000	-1000	0
Enterococcus_faecalis_E99		0	0	0	-600	-22.547043	-1000	-1000	-1000	0
Enterococcus_faecalis_EnGen0253		0	0	0	-604.3956044	-26.077666	-1000	-1000	-1000	0
Enterococcus_faecalis_ERR1203921		0	0	0	-533.3333333	-31.738087	-1000	-1000	-1000	0

Enterococcus_faecalis_ERR1203927	0	0	0	-727.2727273	-31.904661	-1000	-1000	-1000	0
Enterococcus_faecalis_ERR1203928	0	0	0	-533.3333333	-31.738056	-1000	-1000	-1000	0
Enterococcus_faecalis_ERR1203931	0	0	0	-533.3333333	-31.73832	-1000	-1000	-1000	0
Enterococcus_faecalis_ERR1204022	0	0	0	-533.3333333	-31.738061	-1000	-1000	-1000	0
Enterococcus_faecalis_ERR1204025	0	0	0	-533.3333333	-31.738318	-1000	-1000	-1000	0
Enterococcus_faecalis_ERR2221203	0	0	0	-533.3333333	-31.738212	-1000	-1000	-1000	0
Enterococcus_faecalis_ERR2221223	0	0	0	-533.3333333	-31.738198	-1000	-1000	-1000	0
Enterococcus_faecalis_ERR2221228	0	0	0	-533.3333333	-31.738088	-1000	-1000	-1000	0
Enterococcus_faecalis_ERR2221234	0	0	0	-533.3333333	-31.738014	-1000	-1000	-1000	0
Enterococcus_faecalis_ERR2221236	0	0	0	-727.2727273	-31.904667	-1000	-1000	-1000	0
Enterococcus_faecalis_ERR2221251	0	0	0	-533.3333333	-31.738109	-1000	-1000	-1000	0
Enterococcus_faecalis_ERR2221313	0	0	0	-533.3333333	-31.738034	-1000	-1000	-1000	0
Enterococcus_faecalis_ERR2221314	0	0	0	-533.3333333	-31.73804	-1000	-1000	-1000	0
Enterococcus_faecalis_ERR2221315	0	0	0	-533.3333333	-31.738031	-1000	-1000	-1000	0
Enterococcus_faecalis_ERR2221341	0	0	0	-727.2727273	-31.904673	-1000	-1000	-1000	0
Enterococcus_faecalis_ERR2221346	0	0	0	-727.2727273	-31.904669	-1000	-1000	-1000	0
Enterococcus_faecalis_ERR2230084	0	0	0	-727.2727273	-31.904687	-1000	-1000	-1000	0
Enterococcus_faecalis_ERR2230105	0	0	0	-727.2727273	-31.904682	-1000	-1000	-1000	0
Enterococcus_faecalis_ERR2230117	0	0	0	-727.2727273	-31.904687	-1000	-1000	-1000	0
Enterococcus_faecalis_ERR2230123	0	0	0	-727.2727273	-31.904687	-1000	-1000	-1000	0
Enterococcus_faecalis_ERR2230129	0	0	0	-727.2727273	-31.904682	-1000	-1000	-1000	0
Enterococcus_faecalis_ERV103	0	0	0	-538.4615385	-25.75519	-1000	-1000	-1000	0
Enterococcus_faecalis_ERV116	0	0	0	-538.4615385	-25.75519	-1000	-1000	-1000	0
Enterococcus_faecalis_ERV129	0	0	0	-1000	-26.426816	-1000	-1000	-1000	0
Enterococcus_faecalis_ERV25	0	0	0	-1000	-26.426816	-1000	-1000	-1000	0
Enterococcus_faecalis_ERV31	0	0	0	-538.4615385	-25.791248	-1000	-1000	-1000	0
Enterococcus_faecalis_ERV37	0	0	0	-538.4615385	-25.268016	-1000	-1000	-1000	0
Enterococcus_faecalis_ERV41	0	0	0	-538.4615385	-25.755205	-1000	-1000	-1000	0
Enterococcus_faecalis_ERV62	0	0	0	-1000	-26.470973	-1000	-1000	-1000	0
Enterococcus_faecalis_ERV63	0	0	0	-538.4615385	-25.755205	-1000	-1000	-1000	0
Enterococcus_faecalis_ERV65	0	0	0	-538.4615385	-25.791263	-1000	-1000	-1000	0
Enterococcus_faecalis_ERV68	0	0	0	-538.4615385	-25.791263	-1000	-1000	-1000	0
Enterococcus_faecalis_ERV72	0	0	0	-1000	-26.470973	-1000	-1000	-1000	0
Enterococcus_faecalis_ERV81	0	0	0	-538.4615385	-25.755205	-1000	-1000	-1000	0
Enterococcus_faecalis_ERV85	0	0	0	-1000	-26.470973	-1000	-1000	-1000	0
Enterococcus_faecalis_ERV93	0	0	0	-538.4615385	-25.755205	-1000	-1000	-1000	0
Enterococcus_faecalis_F1	0	0	0	-600	-24.222202	-1000	-1000	-1000	0
Enterococcus_faecalis_FA2_2	0	0	0	-600	-24.222215	-1000	-1000	-1000	0
Enterococcus_faecalis_Fly_2	0	0	0	-1000	-23.039854	-1000	-1000	-1000	0
Enterococcus_faecalis_Fly1	0	0	0	-604.3956044	-26.168382	-1000	-1000	-1000	0
Enterococcus_faecalis_HEF39	0	0	0	-600	-21.889419	-1000	-1000	-1000	0
Enterococcus_faecalis_HH22	0	0	0	-500	-23.62638	-1000	-1000	-1000	0
Enterococcus_faecalis_HIP11704	0	0	0	-604.3956044	-25.941249	-1000	-1000	-1000	0
Enterococcus_faecalis_JH1	0	0	0	-604.3956044	-25.974179	-1000	-1000	-1000	0
Enterococcus_faecalis_KB1	0	0	0	-727.2727273	-31.904745	-1000	-1000	-1000	0
Enterococcus_faecalis_M7	0	0	0	-604.3956044	-25.853052	-1000	-1000	-1000	0
Enterococcus_faecalis_Merz151	0	0	0	-533.3333333	-24.059978	-1000	-1000	-1000	0
Enterococcus_faecalis_Merz192	0	0	0	-600	-23.244967	-1000	-1000	-1000	0
Enterococcus_faecalis_Merz204	0	0	0	-1000	-24.356949	-1000	-1000	-1000	0
Enterococcus_faecalis_Merz89	0	0	0	-600	-23.244967	-1000	-1000	-1000	0
Enterococcus_faecalis_Merz96	0	0	0	-604.3956044	-25.113772	-1000	-1000	-1000	0
Enterococcus_faecalis_MMH594	0	0	0	-538.4615385	-25.57515	-1000	-1000	-1000	0
Enterococcus_faecalis_Ned10	0	0	0	-600	-23.492208	-1000	-1000	-1000	0
Enterococcus_faecalis_OG1RF_ATCC_47077	0	0	0	-522.7272727	-27.246754	-1000	-1000	-1000	0
Enterococcus_faecalis_OG1X	0	0	0	-604.3956044	-26.077631	-1000	-1000	-1000	0
Enterococcus_faecalis_Pan7	0	0	0	-600	-21.892609	-1000	-1000	-1000	0
Enterococcus_faecalis_PC1_1	0	0	0	-743.2432432	-27.358988	-1000	-1000	-1000	0
Enterococcus_faecalis_R508	0	0	0	-604.3956044	-25.506198	-1000	-1000	-1000	0
Enterococcus_faecalis_R712	0	0	0	-604.3956044	-25.323252	-1000	-1000	-1000	0
Enterococcus_faecalis_RC73	0	0	0	-1000	-24.356949	-1000	-1000	-1000	0
Enterococcus_faecalis_RM3817	0	0	0	-1000	-25.551698	-1000	-1000	-1000	0
Enterococcus_faecalis_RM4679	0	0	0	-600	-23.49218	-1000	-1000	-1000	0
Enterococcus_faecalis_RMC1	0	0	0	-600	-23.498302	-1000	-1000	-1000	0
Enterococcus_faecalis_RMC5	0	0	0	-600	-24.188992	-1000	-1000	-1000	0
Enterococcus_faecalis_RMC65	0	0	0	-600	-23.244956	-1000	-1000	-1000	0
Enterococcus_faecalis_S613	0	0	0	-604.3956044	-25.323252	-1000	-1000	-1000	0
Enterococcus_faecalis_SF100	0	0	0	-533.3333333	-23.238068	-1000	-1000	-1000	0
Enterococcus_faecalis_SF105	0	0	0	-600	-23.492208	-1000	-1000	-1000	0
Enterococcus_faecalis_SF1592	0	0	0	-533.3333333	-23.238045	-1000	-1000	-1000	0
Enterococcus_faecalis_SF19	0	0	0	-533.3333333	-23.238068	-1000	-1000	-1000	0
Enterococcus_faecalis_SF21520	0	0	0	-533.3333333	-23.961175	-1000	-1000	-1000	0
Enterococcus_faecalis_SF21521	0	0	0	-600	-24.222228	-1000	-1000	-1000	0
Enterococcus_faecalis_SF24396	0	0	0	-600	-21.892601	-1000	-1000	-1000	0
Enterococcus_faecalis_SF24397	0	0	0	-533.3333333	-22.490667	-1000	-1000	-1000	0
Enterococcus_faecalis_SF24413	0	0	0	-533.3333333	-23.961175	-1000	-1000	-1000	0
Enterococcus_faecalis_SF26630	0	0	0	-533.3333333	-23.961149	-1000	-1000	-1000	0
Enterococcus_faecalis_SF28073	0	0	0	-533.3333333	-24.059994	-1000	-1000	-1000	0
Enterococcus_faecalis_SF339	0	0	0	-600	-23.492198	-1000	-1000	-1000	0
Enterococcus_faecalis_SF350	0	0	0	-600	-24.216477	-1000	-1000	-1000	0
Enterococcus_faecalis_SF370	0	0	0	-533.3333333	-23.238057	-1000	-1000	-1000	0
Enterococcus_faecalis_SF5039	0	0	0	-600	-24.216471	-1000	-1000	-1000	0
Enterococcus_faecalis_SF6375	0	0	0	-600	-24.216466	-1000	-1000	-1000	0
Enterococcus_faecalis_SS_6	0	0	0	-600	-23.244935	-1000	-1000	-1000	0
Enterococcus_faecalis_SS_7	0	0	0	-600	-24.218487	-1000	-1000	-1000	0
Enterococcus_faecalis_str_C_19315_led_1b_pp_S	0	0	0	-600	-27.792757	-1000	-1000	-1000	0
Enterococcus_faecalis_str_Symbioflor_1	0	0	0	-604.3956044	-25.506154	-1000	-1000	-1000	0
Enterococcus_faecalis_T1	0	0	0	-604.3956044	-25.971904	-1000	-1000	-1000	0
Enterococcus_faecalis_T10	0	0	0	-600	-22.497256	-1000	-1000	-1000	0
Enterococcus_faecalis_T11	0	0	0	-538.4615385	-25.75954	-1000	-1000	-1000	0
Enterococcus_faecalis_T12	0	0	0	-600	-22.361296	-1000	-1000	-1000	0
Enterococcus_faecalis_T13	0	0	0	-600	-23.244935	-1000	-1000	-1000	0

Enterococcus_faecalis_T14	0	0	0	-600	-23.492208	-1000	-1000	-1000	0
Enterococcus_faecalis_T16	0	0	0	-600	-23.279714	-1000	-1000	-1000	0
Enterococcus_faecalis_T17	0	0	0	-600	-23.492173	-1000	-1000	-1000	0
Enterococcus_faecalis_T18	0	0	0	-600	-22.361279	-1000	-1000	-1000	0
Enterococcus_faecalis_T19	0	0	0	-600	-21.892629	-1000	-1000	-1000	0
Enterococcus_faecalis_T2	0	0	0	-604.3956044	-26.061221	-1000	-1000	-1000	0
Enterococcus_faecalis_T20	0	0	0	-600	-23.498266	-1000	-1000	-1000	0
Enterococcus_faecalis_T21	0	0	0	-600	-23.234609	-1000	-1000	-1000	0
Enterococcus_faecalis_T3	0	0	0	-600	-26.845469	-1000	-1000	-1000	0
Enterococcus_faecalis_T4	0	0	0	-600	-23.49218	-1000	-1000	-1000	0
Enterococcus_faecalis_T5	0	0	0	-600	-21.892619	-1000	-1000	-1000	0
Enterococcus_faecalis_T6	0	0	0	-600	-21.889458	-1000	-1000	-1000	0
Enterococcus_faecalis_T7	0	0	0	-600	-22.305615	-1000	-1000	-1000	0
Enterococcus_faecalis_T8	0	0	0	-604.3956044	-26.16856	-1000	-1000	-1000	0
Enterococcus_faecalis_T9	0	0	0	-600	-23.528655	-1000	-1000	-1000	0
Enterococcus_faecalis_TR161	0	0	0	-533.3333333	-23.238068	-1000	-1000	-1000	0
Enterococcus_faecalis_TR197	0	0	0	-1000	-22.822222	-1000	-1000	-1000	0
Enterococcus_faecalis_TUSoD_Ef11	0	0	0	-604.3956044	-25.699621	-1000	-1000	-1000	0
Enterococcus_faecalis_TX0012	0	0	0	-1000	-25.179016	-1000	-1000	-1000	0
Enterococcus_faecalis_TX0017	0	0	0	-604.3956044	-25.968813	-1000	-1000	-1000	0
Enterococcus_faecalis_TX0027	0	0	0	-604.3956044	-25.978459	-1000	-1000	-1000	0
Enterococcus_faecalis_TX0031	0	0	0	-1000	-26.433574	-1000	-1000	-1000	0
Enterococcus_faecalis_TX0043	0	0	0	-604.3956044	-25.972061	-1000	-1000	-1000	0
Enterococcus_faecalis_TX0102	0	0	0	-604.3956044	-27.964255	-1000	-1000	-1000	0
Enterococcus_faecalis_TX0104	0	0	0	-522.7272727	-33.331538	-1000	-1000	-1000	0
Enterococcus_faecalis_TX0109	0	0	0	-604.3956044	-26.168736	-1000	-1000	-1000	0
Enterococcus_faecalis_TX0309A	0	0	0	-538.4615385	-25.759118	-1000	-1000	-1000	0
Enterococcus_faecalis_TX0309B	0	0	0	-538.4615385	-25.759118	-1000	-1000	-1000	0
Enterococcus_faecalis_TX0312	0	0	0	-604.3956044	-24.967271	-1000	-1000	-1000	0
Enterococcus_faecalis_TX0411	0	0	0	-604.3956044	-25.972107	-1000	-1000	-1000	0
Enterococcus_faecalis_TX0470	0	0	0	-604.3956044	-25.041542	-1000	-1000	-1000	0
Enterococcus_faecalis_TX0630	0	0	0	-604.3956044	-25.941485	-1000	-1000	-1000	0
Enterococcus_faecalis_TX0635_WH245	0	0	0	-600	-23.238068	-1000	-1000	-1000	0
Enterococcus_faecalis_TX0645	0	0	0	-604.3956044	-26.055934	-1000	-1000	-1000	0
Enterococcus_faecalis_TX0855	0	0	0	-604.3956044	-25.941452	-1000	-1000	-1000	0
Enterococcus_faecalis_TX0860	0	0	0	-604.3956044	-26.061362	-1000	-1000	-1000	0
Enterococcus_faecalis_TX1302	0	0	0	-529.4117647	-21.815292	-1000	-1000	-1000	0
Enterococcus_faecalis_TX1322	0	0	0	-522.7272727	-33.527044	-1000	-1000	-1000	0
Enterococcus_faecalis_TX1337RF	0	0	0	-1000	-30.455922	-1000	-1000	-1000	0
Enterococcus_faecalis_TX1341	0	0	0	-604.3956044	-25.978459	-1000	-1000	-1000	0
Enterococcus_faecalis_TX1342	0	0	0	-529.4117647	-21.815292	-1000	-1000	-1000	0
Enterococcus_faecalis_TX1346	0	0	0	-604.3956044	-25.004679	-1000	-1000	-1000	0
Enterococcus_faecalis_TX2134	0	0	0	-522.7272727	-33.512366	-1000	-1000	-1000	0
Enterococcus_faecalis_TX2137	0	0	0	-604.3956044	-24.943629	-1000	-1000	-1000	0
Enterococcus_faecalis_TX2141	0	0	0	-604.3956044	-26.053614	-1000	-1000	-1000	0
Enterococcus_faecalis_TX4000	0	0	0	-604.3956044	-25.974304	-1000	-1000	-1000	0
Enterococcus_faecalis_TX4244	0	0	0	-604.3956044	-26.01207	-1000	-1000	-1000	0
Enterococcus_faecalis_TX4248	0	0	0	-1000	-26.437604	-1000	-1000	-1000	0
Enterococcus_faecalis_UAA1014	0	0	0	-600	-23.177678	-1000	-1000	-1000	0
Enterococcus_faecalis_UAA1180	0	0	0	-600	-23.177689	-1000	-1000	-1000	0
Enterococcus_faecalis_UAA1489	0	0	0	-533.3333333	-23.961201	-1000	-1000	-1000	0
Enterococcus_faecalis_UAA409	0	0	0	-600	-24.218468	-1000	-1000	-1000	0
Enterococcus_faecalis_UAA702	0	0	0	-600	-26.16318	-1000	-1000	-1000	0
Enterococcus_faecalis_UAA769	0	0	0	-600	-24.222215	-1000	-1000	-1000	0
Enterococcus_faecalis_UAA823	0	0	0	-533.3333333	-23.96765	-1000	-1000	-1000	0
Enterococcus_faecalis_UAA902	0	0	0	-600	-23.170061	-1000	-1000	-1000	0
Enterococcus_faecalis_UAA903	0	0	0	-600	-23.170054	-1000	-1000	-1000	0
Enterococcus_faecalis_UAA904	0	0	0	-600	-23.170054	-1000	-1000	-1000	0
Enterococcus_faecalis_UAA905	0	0	0	-600	-23.170049	-1000	-1000	-1000	0
Enterococcus_faecalis_UAA906	0	0	0	-600	-23.170054	-1000	-1000	-1000	0
Enterococcus_faecalis_UAA907	0	0	0	-600	-23.170068	-1000	-1000	-1000	0
Enterococcus_faecalis_UAA943	0	0	0	-600	-23.170061	-1000	-1000	-1000	0
Enterococcus_faecalis_UAA948	0	0	0	-1000	-24.796724	-1000	-1000	-1000	0
Enterococcus_faecalis_V583	0	0	0	-522.7272727	-27.182788	-1000	-1000	-1000	0
Enterococcus_faecalis_V587	0	0	0	-533.3333333	-23.961124	-1000	-1000	-1000	0
Enterococcus_faecalis_WH257	0	0	0	-600	-23.238057	-1000	-1000	-1000	0
Enterococcus_faecalis_WH571	0	0	0	-600	-23.238068	-1000	-1000	-1000	0
Enterococcus_faecalis_X98	0	0	0	-604.3956044	-25.971967	-1000	-1000	-1000	0
Enterococcus_faecalis_YI6_1	0	0	0	-600	-23.494741	-1000	-1000	-1000	0
Enterococcus_faecium_109_A1	0	0	0	0	-25.614719	-1000	0	0	0
Enterococcus_faecium_1141733	0	0	0	-1000	-25.822827	-1000	0	0	0
Enterococcus_faecium_1230933	0	0	0	0	-25.861122	-1000	0	-1000	0
Enterococcus_faecium_1231408	0	0	0	-1000	-25.855251	-1000	0	0	0
Enterococcus_faecium_1231410	0	0	0	0	-25.855342	-1000	0	0	0
Enterococcus_faecium_1231501	0	0	0	0	-25.79867	-1000	0	0	0
Enterococcus_faecium_1231502	0	0	0	0	-25.881561	-1000	0	0	0
Enterococcus_faecium_2006_70_121	0	0	0	0	-24.021727	0	0	0	0
Enterococcus_faecium_504	0	0	0	0	-25.857055	-1000	0	0	0
Enterococcus_faecium_505	0	0	0	0	-25.821884	-1000	0	0	0
Enterococcus_faecium_7330381_1	0	0	0	0	-25.614719	-1000	0	0	0
Enterococcus_faecium_7330446_2	0	0	0	-1000	-25.614729	-1000	0	-1000	0
Enterococcus_faecium_7330519_3	0	0	0	-1000	-25.614719	-1000	0	-1000	0
Enterococcus_faecium_7330614_1	0	0	0	-1000	-25.614702	-1000	0	-1000	0
Enterococcus_faecium_7330884_2	0	0	0	0	-25.614712	-1000	0	-1000	0
Enterococcus_faecium_7430166_3	0	0	0	-1000	-25.614702	-1000	0	-1000	0
Enterococcus_faecium_841V03	0	0	0	-1000	-25.614719	-1000	0	-1000	0
Enterococcus_faecium_9730219_1	0	0	0	-1000	-25.614739	-1000	0	-1000	0
Enterococcus_faecium_9730357_1	0	0	0	-1000	-25.614712	-1000	0	-1000	0
Enterococcus_faecium_9731349_1	0	0	0	0	-25.491877	-1000	0	-1000	0
Enterococcus_faecium_9731352_4	0	0	0	-1000	-25.614712	-1000	0	-1000	0
Enterococcus_faecium_9830091_5	0	0	0	-1000	-25.614712	-1000	0	-1000	0

Enterococcus_faecium_9830512_2	0	0	0	-1000	-25.614702	-1000	0	-1000	0
Enterococcus_faecium_9830565_4	0	0	0	-1000	-25.614719	-1000	0	0	0
Enterococcus_faecium_9930238_2	0	0	0	0	-25.614719	-1000	0	0	0
Enterococcus_faecium_9931110_4	0	0	0	-1000	-25.614692	-1000	0	-1000	0
Enterococcus_faecium_A17_Sv1	0	0	0	-1000	-25.614702	-1000	0	-1000	0
Enterococcus_faecium_ATCC_8459	0	0	0	0	-25.833977	-1000	0	0	0
Enterococcus_faecium_Aus0004	0	0	0	0	-25.858549	-1000	0	0	0
Enterococcus_faecium_C309	0	0	0	0	-25.884884	-1000	0	-1000	0
Enterococcus_faecium_C68	0	0	0	0	-25.85858	-1000	0	0	0
Enterococcus_faecium_Com12	0	0	0	0	-25.825733	-1000	0	0	0
Enterococcus_faecium_Com15	0	0	0	0	-25.852573	-1000	0	0	0
Enterococcus_faecium_CRL1879	0	0	0	0	-25.587912	-1000	0	0	0
Enterococcus_faecium_D344SRF	0	0	0	0	-25.857348	-1000	0	0	0
Enterococcus_faecium_DO	0	0	0	-1000	-25.05311	-1000	0	0	0
Enterococcus_faecium_E0045	0	0	0	0	-25.491812	-1000	0	-1000	0
Enterococcus_faecium_E0120	0	0	0	0	-25.64832	-1000	0	0	0
Enterococcus_faecium_E0164	0	0	0	0	-25.614538	-1000	0	0	0
Enterococcus_faecium_E0269	0	0	0	0	-25.614538	-1000	0	-1000	0
Enterococcus_faecium_E0333	0	0	0	0	-25.654418	-1000	0	0	0
Enterococcus_faecium_E0679	0	0	0	0	-25.614692	-1000	0	0	0
Enterococcus_faecium_E0680	0	0	0	-1000	-25.614692	-1000	0	0	0
Enterococcus_faecium_E0688	0	0	0	-1000	-25.614712	-1000	0	-1000	0
Enterococcus_faecium_E1007	0	0	0	0	-25.614712	-1000	0	0	0
Enterococcus_faecium_E1039	0	0	0	0	-25.870931	-1000	0	0	0
Enterococcus_faecium_E1050	0	0	0	0	-25.614739	-1000	0	0	0
Enterococcus_faecium_E1071	0	0	0	-1000	-25.854267	-1000	0	0	0
Enterococcus_faecium_E1133	0	0	0	0	-25.611807	-1000	0	0	0
Enterococcus_faecium_E1162	0	0	0	0	-25.884822	-1000	0	0	0
Enterococcus_faecium_E1185	0	0	0	0	-25.610394	-1000	0	0	0
Enterococcus_faecium_E1258	0	0	0	0	-25.61475	-1000	0	0	0
Enterococcus_faecium_E1293	0	0	0	-1000	-25.614712	-1000	0	0	0
Enterococcus_faecium_E1321	0	0	0	0	-25.614681	-1000	0	0	0
Enterococcus_faecium_E1392	0	0	0	0	-25.611879	-1000	0	-1000	0
Enterococcus_faecium_E1552	0	0	0	-1000	-25.614681	-1000	0	-1000	0
Enterococcus_faecium_E1573	0	0	0	0	-25.614651	-1000	0	0	0
Enterococcus_faecium_E1574	0	0	0	0	-25.648283	-1000	0	0	0
Enterococcus_faecium_E1575	0	0	0	0	-25.614548	-1000	0	0	0
Enterococcus_faecium_E1576	0	0	0	0	-25.614702	-1000	0	0	0
Enterococcus_faecium_E1578	0	0	0	0	-25.614692	-1000	0	0	0
Enterococcus_faecium_E1590	0	0	0	-1000	-25.614739	-1000	0	-1000	0
Enterococcus_faecium_E1604	0	0	0	0	-25.61463	-1000	0	0	0
Enterococcus_faecium_E1613	0	0	0	0	-25.614651	-1000	0	0	0
Enterococcus_faecium_E1620	0	0	0	0	-25.614487	-1000	0	-1000	0
Enterococcus_faecium_E1622	0	0	0	0	-25.61475	-1000	0	0	0
Enterococcus_faecium_E1623	0	0	0	0	-25.614739	-1000	0	0	0
Enterococcus_faecium_E1626	0	0	0	0	-25.614719	-1000	0	0	0
Enterococcus_faecium_E1627	0	0	0	-1000	-25.611988	-1000	0	0	0
Enterococcus_faecium_E1630	0	0	0	0	-25.613139	-1000	0	0	0
Enterococcus_faecium_E1634	0	0	0	0	-25.14891	-1000	0	0	0
Enterococcus_faecium_E1636	0	0	0	0	-26.070634	0	0	0	0
Enterococcus_faecium_E1644	0	0	0	0	-25.614641	-1000	0	0	0
Enterococcus_faecium_E1731	0	0	0	0	-25.611967	-1000	0	0	0
Enterococcus_faecium_E1861	0	0	0	0	-25.614712	-1000	0	0	0
Enterococcus_faecium_E1904	0	0	0	0	-25.611674	-1000	0	0	0
Enterococcus_faecium_E1972	0	0	0	0	-25.614692	-1000	0	0	0
Enterococcus_faecium_E2039	0	0	0	0	-25.614712	-1000	0	0	0
Enterococcus_faecium_E2071	0	0	0	0	-25.648314	-1000	0	0	0
Enterococcus_faecium_E2134	0	0	0	0	-25.614528	-1000	0	0	0
Enterococcus_faecium_E2297	0	0	0	0	-25.643712	-1000	0	0	0
Enterococcus_faecium_E2369	0	0	0	0	-25.614641	-1000	0	0	0
Enterococcus_faecium_E2560	0	0	0	0	-25.611688	-1000	0	0	0
Enterococcus_faecium_E2620	0	0	0	0	-15.677814	-893.58813	0	0	0
Enterococcus_faecium_E2883	0	0	0	0	-25.61194	-1000	0	0	0
Enterococcus_faecium_E2966	0	0	0	0	-25.61195	-1000	0	0	0
Enterococcus_faecium_E3083	0	0	0	-1000	-25.584106	-1000	0	0	0
Enterococcus_faecium_E3346	0	0	0	0	-25.614668	-1000	0	0	0
Enterococcus_faecium_E3548	0	0	0	-1000	-25.584048	-1000	0	0	0
Enterococcus_faecium_E4215	0	0	0	0	-25.614641	-1000	0	-1000	0
Enterococcus_faecium_E4389	0	0	0	-1000	-25.614681	-1000	0	-1000	0
Enterococcus_faecium_E6012	0	0	-1000	0	-33.566478	-1000	0	0	0
Enterococcus_faecium_E6045	0	0	-1000	0	-33.566574	-1000	0	0	0
Enterococcus_faecium_E8sv3	0	0	0	-1000	-25.614712	-1000	0	-1000	0
Enterococcus_faecium_E980	0	0	0	0	-25.854235	-1000	0	0	0
Enterococcus_faecium_EnGen0305	0	0	0	0	-25.827665	-1000	0	0	0
Enterococcus_faecium_EnGen0308	0	0	0	0	-25.852335	-1000	0	0	0
Enterococcus_faecium_EnGen0312	0	0	0	0	-25.855701	-1000	0	0	0
Enterococcus_faecium_EnGen0314	0	0	0	0	-25.855592	-1000	0	0	0
Enterococcus_faecium_EnGen0316	0	0	0	0	-25.855576	-1000	0	0	0
Enterococcus_faecium_EnGen0318	0	0	0	0	-25.855619	-1000	0	0	0
Enterococcus_faecium_EnGen0319	0	0	0	0	-25.855529	-1000	0	0	0
Enterococcus_faecium_EnGen0321	0	0	0	0	-25.855576	-1000	0	0	0
Enterococcus_faecium_EnGen0323	0	0	0	0	-25.855635	-1000	0	0	0
Enterococcus_faecium_EnGen0375	0	0	0	0	-25.855838	-1000	0	0	0
Enterococcus_faecium_EnGen0376	0	0	0	0	-25.855838	-1000	0	0	0
Enterococcus_faecium_EnGen0377	0	0	0	0	-25.855827	-1000	0	0	0
Enterococcus_faecium_ERR2221216	0	0	0	0	-27.388737	-1000	0	0	0
Enterococcus_faecium_ERR2221309	0	0	0	0	-28.910364	-1000	0	0	0
Enterococcus_faecium_ERR2221310	0	0	0	0	-27.389031	-1000	0	-1000	0
Enterococcus_faecium_ERR2221317	0	0	0	0	-27.388747	-1000	0	0	0
Enterococcus_faecium_ERR2221332	0	0	0	0	-27.388682	-1000	0	0	0
Enterococcus_faecium_ERV1	0	0	0	0	-25.855827	-1000	0	0	0

Enterococcus_faecium_F9730129_1	0	0	0	0	-25.503493	-1000	0	-1000	0
Enterococcus_faecium_H17243	0	0	0	0	-25.61476	-1000	0	0	0
Enterococcus_faecium_H17575	0	0	0	-1000	-25.614712	-1000	0	-1000	0
Enterococcus_faecium_HF50104	0	0	0	-1000	-25.614729	-1000	0	-1000	0
Enterococcus_faecium_HF50105	0	0	0	-1000	-25.614712	-1000	0	-1000	0
Enterococcus_faecium_HF50106	0	0	0	-1000	-25.614702	-1000	0	-1000	0
Enterococcus_faecium_HF50203	0	0	0	0	-25.614729	-1000	0	-1000	0
Enterococcus_faecium_HF50204	0	0	0	-1000	-25.614702	-1000	0	-1000	0
Enterococcus_faecium_HF50215	0	0	0	-1000	-25.614702	-1000	0	-1000	0
Enterococcus_faecium_HM1071	0	0	0	-1000	-25.61463	-1000	0	0	0
Enterococcus_faecium_HM1072	0	0	0	0	-25.610418	-1000	0	0	0
Enterococcus_faecium_HM1073	0	0	0	0	-25.611998	-1000	0	0	0
Enterococcus_faecium_HM1074	0	0	0	0	-25.614692	-1000	0	0	0
Enterococcus_faecium_LCT_EF128	0	0	0	0	-25.853954	-1000	0	0	0
Enterococcus_faecium_LCT_EF20	0	0	0	-1000	-25.856714	-1000	0	0	0
Enterococcus_faecium_LCT_EF258	0	0	0	-1000	-25.856714	-1000	0	0	0
Enterococcus_faecium_LCT_EF90	0	0	0	-1000	-25.856808	-1000	0	0	0
Enterococcus_faecium_PC4_1	0	0	0	0	-25.858596	-1000	0	-1000	0
Enterococcus_faecium_S658_3	0	0	0	-1000	-25.614702	-1000	0	-1000	0
Enterococcus_faecium_TC_6	0	0	0	0	-25.634065	-1000	0	-1000	0
Enterococcus_faecium_TX0082	0	0	0	0	-25.858612	-1000	0	0	0
Enterococcus_faecium_TX0133A	0	0	0	0	-25.882059	-1000	0	0	0
Enterococcus_faecium_TX0133a04	0	0	0	0	-25.882059	-1000	0	0	0
Enterococcus_faecium_TX0133B	0	0	0	0	-25.882059	-1000	0	0	0
Enterococcus_faecium_TX0133C	0	0	0	0	-25.882059	-1000	0	0	0
Enterococcus_faecium_TX1330	0	0	0	0	-25.618855	-1000	0	-1000	0
Enterococcus_faecium_U0317	0	0	0	0	-25.884869	-1000	0	0	0
Enterococcus_faecium_UAA1007	0	0	0	0	-25.611961	-1000	0	0	0
Enterococcus_faecium_UAA1019	0	0	0	0	-25.614702	-1000	0	0	0
Enterococcus_faecium_UAA1022	0	0	0	0	-25.614729	-1000	0	0	0
Enterococcus_faecium_UAA1023	0	0	0	-1000	-25.614719	-1000	0	0	0
Enterococcus_faecium_UAA1024	0	0	0	-1000	-25.614702	-1000	0	0	0
Enterococcus_faecium_UAA1025	0	0	0	0	-25.614651	-1000	0	-1000	0
Enterococcus_faecium_UAA1280	0	0	0	0	-25.61476	-1000	0	0	0
Enterococcus_faecium_UAA1433	0	0	0	0	-25.61194	-1000	0	0	0
Enterococcus_faecium_UAA1484	0	0	0	0	-25.614641	-1000	0	0	0
Enterococcus_faecium_UAA210	0	0	0	0	-25.611978	-1000	0	0	0
Enterococcus_faecium_UAA407	0	0	0	0	-25.614719	-1000	0	0	0
Enterococcus_faecium_UAA430	0	0	0	0	-25.614712	-1000	0	0	0
Enterococcus_faecium_UAA431	0	0	0	-1000	-25.614729	-1000	0	0	0
Enterococcus_faecium_UAA714	0	0	0	-1000	-25.614719	-1000	0	0	0
Enterococcus_faecium_UAA715	0	0	0	-1000	-25.614719	-1000	0	0	0
Enterococcus_faecium_UAA716	0	0	0	-1000	-25.614702	-1000	0	0	0
Enterococcus_faecium_UAA718	0	0	0	-1000	-25.614702	-1000	0	0	0
Enterococcus_faecium_UAA719	0	0	0	0	-25.614719	-1000	0	0	0
Enterococcus_faecium_UAA720	0	0	0	0	-25.611916	-1000	0	0	0
Enterococcus_faecium_UAA721	0	0	0	0	-25.614719	-1000	0	0	0
Enterococcus_faecium_UAA722	0	0	0	0	-25.491858	-1000	0	0	0
Enterococcus_faecium_UAA723	0	0	0	-1000	-25.614729	-1000	0	0	0
Enterococcus_faecium_UAA724	0	0	0	-1000	-25.61477	-1000	0	0	0
Enterococcus_faecium_UAA725	0	0	0	0	-25.614712	-1000	0	0	0
Enterococcus_faecium_UAA825	0	0	0	0	-25.614712	-1000	0	0	0
Enterococcus_faecium_UAA909	0	0	0	0	-25.614702	-1000	0	0	0
Enterococcus_faecium_UAA910	0	0	0	-1000	-25.61195	-1000	0	0	0
Enterococcus_faecium_UAA911	0	0	0	0	-25.616163	-1000	0	0	0
Enterococcus_faecium_UAA944	0	0	0	0	-25.614739	-1000	0	0	0
Enterococcus_faecium_UAA945	0	0	0	0	-25.61476	-1000	0	0	0
Enterococcus_faecium_UAA947	0	0	0	0	-25.61193	-1000	0	-1000	0
Enterococcus_faecium_UAA949	0	0	0	0	-25.614729	-1000	0	0	0
Enterococcus_faecium_UAA950	0	0	0	0	-25.614739	-1000	0	0	0
Enterococcus_faecium_UAA951	0	0	0	0	-25.614702	-1000	0	0	0
Enterococcus_faecium_UAA952	0	0	0	0	-25.614692	-1000	0	0	0
Enterococcus_faecium_UC7251	0	0	0	-1000	-25.800994	-1000	0	0	0
Enterococcus_faecium_VAN_219	0	0	0	0	-25.61475	-1000	0	0	0
Enterococcus_faecium_VAN_222	0	0	0	0	-25.61475	-1000	0	0	0
Enterococcus_faecium_VAN_327	0	0	0	0	-25.648351	-1000	0	0	0
Enterococcus_faecium_VAN_332	0	0	0	0	-25.614781	-1000	0	0	0
Enterococcus_faecium_VAN_335	0	0	0	0	-25.648331	-1000	0	0	0
Enterococcus_faecium_VAN_342	0	0	0	0	-25.61476	-1000	0	-1000	0
Enterococcus_faecium_VAN_345	0	0	0	0	-25.61476	-1000	0	0	0
Enterococcus_faecium_VAN_476	0	0	0	0	-25.61475	-1000	0	0	0
Enterococcus_faecium_VRE_108	0	0	-1000	0	-33.540159	-1000	0	-1000	0
Enterococcus_faecium_VRE_110	0	0	0	0	-25.611879	-1000	0	0	0
Enterococcus_faecium_VRE_13	0	0	0	0	-25.611705	-1000	0	0	0
Enterococcus_faecium_VRE_84	0	0	-1000	0	-33.420117	-1000	0	-1000	0
Enterococcus_flavescens_ERR2221217	0	0	0	-1000	-53.764891	-1000	0	-938.47758	0
Enterococcus_flavescens_ERR2221403	0	0	0	-1000	-53.764889	-1000	0	-938.47758	0
Enterococcus_gallinarum_EG2	0	0	0	-1000	-28.457555	-1000	0	-1000	0
Enterococcus_gallinarum_ERR2221218	0	0	0	-1000	-44.021918	-1000	0	-906.25	0
Enterococcus_gallinarum_ERR2221324	0	0	0	-1000	-44.015955	-1000	0	-906.25	0
Enterococcus_gallinarum_ERR2221329	0	0	0	-1000	-48.331002	-1000	0	-1000	0
Enterococcus_gallinarum_ERR2221342	0	0	0	-1000	-43.530984	-1000	0	-906.25	0
Enterococcus_gallinarum_ERR2221347	0	0	0	-1000	-43.244551	-1000	0	-605.35714	0
Enterococcus_gallinarum_ERR2221362	0	0	0	-1000	-43.536437	-1000	0	-906.25	0
Enterococcus_gilvus_ATCC_BAA_350	0	0	0	-1000	-27.921497	-1000	0	-1000	0
Enterococcus_gilvus_CR1	0	0	0	-1000	-37.228374	-1000	0	-1000	0
Enterococcus_hirae_ATCC_9790	0	0	0	0	-25.721018	0	0	-1000	0
Enterococcus_mundtii_ATCC_882	0	0	0	-30.05808191	-18.807541	-1000	0	0	0
Enterococcus_mundtii_crl1656	0	0	0	0	-11.500312	-1000	0	0	0
Enterococcus_mundtii_CRL35	0	0	0	0	-18.572596	-1000	0	0	0
Enterococcus_mundtii_QU_25	0	0	0	-30.01594522	-18.780826	-1000	0	0	0

Enterococcus_pallens_ATCC_BAA_351	0	0	0	-1000	-23.09644	-1000	0	-1000	0
Enterococcus_pallens_DSM_15690	0	0	0	-1000	-17.701619	-1000	0	-1000	0
Enterococcus_phoeniculicola_ATCC_BAA_412	0	0	0	-45.04013572	-22.792104	-1000	0	-1000	0
Enterococcus_raffinosus_ATCC_49464	0	0	0	-1000	-38.387837	-1000	0	0	0
Enterococcus_raffinosus_DSM_5633	0	0	0	-1000	-38.377932	-1000	0	-1000	0
Enterococcus_raffinosus_NBRC_100492	0	0	0	-1000	-38.377932	-1000	0	-1000	0
Enterococcus_saccharolyticus_30_1	0	0	0	-1000	-28.250939	-1000	0	-1000	0
Enterococcus_saccharolyticus_ATCC_43076	0	0	0	-48.81876524	-24.155458	-1000	0	-1000	0
Enterococcus_saccharolyticus_DSM8903	0	0	0	0	-15.259358	0	0	-1000	0
Enterococcus_sp_7L76	0	0	0	-53.51205821	-28.628924	-1000	0	-750	0
Enterococcus_sp_C1	0	0	0	-1000	-44.032011	-1000	0	-927.75665	0
Enterococcus_sp_GMD2E	0	0	0	0	-28.399994	-1000	0	0	0
Enterococcus_sp_GMD3E	0	0	0	0	-19.495698	-1000	0	0	0
Enterococcus_sp_GMD4E	0	0	0	0	-17.465667	-1000	0	0	0
Enterococcus_sp_GMD5E	0	0	0	0	-28.680569	-1000	0	0	0
Enterorhabdus_caecimuris_B7	0	0	0	-1000	-24.262749	0	0	0	0
Enterorhabdus_mucosicola_DSM_19490	0	0	0	-9.617445114	-4.066181	0	0	0	0
Eremococcus_coleocola_ACS_139_V_Col8	0	0	0	-53.97622731	-31.141974	0	0	0	0
Erwinia_psidii_IBSBF_435	0	0	-1000	-1000	-54.827623	-1000	0	-1000	0
Erwinia_rhapontici_BIGb0435	0	0	-1000	-1000	-39.243532	-1000	0	-1000	-1000
Erysipelatoclostridium_nov_ERR2221185	0	0	0	-345.2615813	-16.314861	0	0	-411.04294	0
Erysipelatoclostridium_nov_ERR2221240	0	0	0	0	-10.990385	0	0	0	0
Erysipelatoclostridium_ramosum_ERR1203924	0	0	0	-1000	-24.726245	0	-1000	-1000	0
Erysipelatoclostridium_ramosum_ERR171271	0	0	0	-1000	-24.726332	0	-1000	-1000	0
Erysipelatoclostridium_ramosum_ERR2221150	0	0	0	-1000	-24.726284	0	-1000	-1000	0
Erysipelatoclostridium_ramosum_ERR2221231	0	0	0	-1000	-24.726247	0	-1000	-1000	0
Erysipelatoclostridium_ramosum_ERR2221239	0	0	0	-1000	-24.558053	0	-1000	-1000	0
Erysipelothrix_rhusiopathiae_ATCC_19414	0	0	0	0	-15.326158	0	0	-764.16608	0
Erysipelothrix_rhusiopathiae_str_Fujisawa	0	0	0	0	-15.326163	0	0	-764.16598	0
Erysipelothrix_rhusiopathiae_SY1027	0	0	0	0	-15.282184	0	0	-521.2766	0
Erysipelotrichaceae_bacterium_2_2_44A	0	0	0	0	-22.026528	-1000	0	-710.48513	0
Erysipelotrichaceae_bacterium_21_3	0	0	0	0	-21.801543	-1000	0	-697.53086	0
Erysipelotrichaceae_bacterium_5_2_54FAA	0	0	0	0	-18.471509	0	0	0	0
Erysipelotrichaceae_bacterium_6_1_45	0	0	0	0	-22.025473	-1000	0	-710.48513	0
Erysipelotrichaceae_bacterium_I46	0	0	0	0	-21.801724	-1000	0	-697.53086	0
Erysipelotrichaceae_bacterium_sp_3_1_53	0	0	0	0	-18.057673	-1000	0	0	0
Erysipelotrichaceae_nov_ERR1022332	0	0	0	-340.133206	-11.326834	0	0	-369.49517	0
Erysipelotrichaceae_nov_ERR1022333	0	0	0	-342.5329241	-11.415327	0	0	-369.49517	0
Erysipelotrichaceae_nov_ERR1022368	0	0	0	-342.4430028	-10.361487	0	0	-365.74074	0
Erysipelotrichaceae_nov_ERR1022369	0	0	0	0	-21.801472	-1000	0	-697.53086	0
Erysipelotrichaceae_nov_ERR1022417	0	0	0	0	-18.235122	-816.43836	0	0	0
Erysipelotrichaceae_nov_ERR1022464	0	0	0	-350.0982931	-15.662049	0	0	-413.04348	0
Erysipelotrichaceae_nov_ERR1203923	0	0	0	0	-21.801533	-1000	0	-697.53086	0
Erysipelotrichaceae_nov_ERR1203963	0	0	0	-352.9212421	-15.524331	0	0	-413.04348	0
Erysipelotrichaceae_nov_ERR1204057	0	0	0	-352.9212432	-15.524326	0	0	-413.04348	0
Erysipelotrichaceae_nov_ERR171269	0	0	0	0	-19.822021	-1000	0	-676.59574	0
Erysipelotrichaceae_nov_ERR2221179	0	0	0	0	-15.338321	0	0	-533.33333	0
Erysipelotrichaceae_nov_ERR2221230	0	0	0	0	-21.801537	-1000	0	-697.53086	0
Erysipelotrichaceae_nov_ERR2221343	0	0	0	0	-11.805303	0	0	-305.55556	0
Erysipelotrichaceae_nov_ERR2230080	0	0	0	0	-15.338317	0	0	-533.33333	0
Erysipelotrichaceae_nov_ERR2230108	0	0	0	-352.909288	-15.514377	0	0	-413.04348	0
Escherichia_albertii_EC06_170	0	0	-1000	-1000	-50.673054	-1000	0	0	0
Escherichia_albertii_KF1	0	0	-1000	-1000	-63.418444	-1000	0	0	0
Escherichia_albertii_TW07627	0	0	-1000	-1000	-63.72734	-1000	0	0	0
Escherichia_albertii_TW11588	0	0	0	-1000	-26.019331	-1000	0	0	0
Escherichia_coli_0_1288	0	0	-1000	-1000	-71.433465	-1000	0	0	0
Escherichia_coli_0_1304	0	0	-1000	-1000	-70.924192	-1000	0	0	0
Escherichia_coli_042	-1000	0	-1000	-1000	-71.128194	-1000	0	0	0
Escherichia_coli_07798	0	0	-1000	-1000	-82.202067	-1000	0	0	0
Escherichia_coli_09BKT078844	0	0	-1000	-1000	-70.950081	-1000	0	0	0
Escherichia_coli_1_2264	0	0	-1000	-1000	-70.949993	-1000	0	0	0
Escherichia_coli_1_2741	0	0	-1000	-1000	-71.454765	-1000	0	0	0
Escherichia_coli_10_0821	0	0	-1000	-1000	-70.95011	-1000	0	0	0
Escherichia_coli_10_0833	0	0	-1000	-1000	-70.950081	-1000	0	0	0
Escherichia_coli_10_0869	0	0	-1000	-1000	-70.950081	-1000	0	0	0
Escherichia_coli_101_1	0	0	-1000	-1000	-71.285714	-1000	0	0	0
Escherichia_coli_1357	0	0	-1000	-1000	-55.062626	-1000	0	0	0
Escherichia_coli_174750	0	0	-1000	-1000	-71.454676	-1000	0	0	0
Escherichia_coli_174900	0	0	-1000	-1000	-70.936382	-1000	0	0	0
Escherichia_coli_178900	0	0	-1000	-1000	-61.789923	-1000	0	0	0
Escherichia_coli_179100	0	0	-1000	-1000	-71.479437	-1000	0	0	0
Escherichia_coli_179550	0	0	-1000	-1000	-61.406041	-1000	0	0	0
Escherichia_coli_180050	0	0	-1000	-1000	-71.433436	-1000	0	0	0
Escherichia_coli_180200	0	0	-1000	-1000	-70.949963	-1000	0	0	0
Escherichia_coli_180600	0	0	-1000	-1000	-71.468456	-1000	0	0	0
Escherichia_coli_1827_70	0	0	-1000	-1000	-70.665407	-1000	0	0	0
Escherichia_coli_199900_1	0	0	-1000	-1000	-61.83226	-1000	0	0	0
Escherichia_coli_2_3916	0	0	-1000	-1000	-71.428417	-1000	0	0	0
Escherichia_coli_2_4168	0	0	-1000	-1000	-71.442099	-1000	0	0	0
Escherichia_coli_201600_1	0	0	-1000	-1000	-71.441544	-1000	0	0	0
Escherichia_coli_2362_75	0	0	-1000	-1000	-71.42912	-1000	0	0	0
Escherichia_coli_2534_86	0	0	-1000	-1000	-70.950022	-1000	0	0	0
Escherichia_coli_2719100	0	0	-1000	-1000	-71.419606	-1000	0	0	0
Escherichia_coli_2720900	0	0	-1000	-1000	-70.949963	-1000	0	0	0
Escherichia_coli_2722950	0	0	-1000	-1000	-71.353801	-1000	0	0	0
Escherichia_coli_2726800	0	0	-1000	-1000	-70.92846	-1000	0	0	0
Escherichia_coli_2726950	0	0	-1000	-1000	-71.468486	-1000	0	0	0
Escherichia_coli_2729250	0	0	-1000	-1000	-71.373195	-1000	0	0	0
Escherichia_coli_2730350	0	0	-1000	-1000	-61.83226	-1000	0	0	0
Escherichia_coli_2730450	0	0	-1000	-1000	-71.433495	-1000	0	0	0
Escherichia_coli_2731150	0	0	-1000	-1000	-71.419669	-1000	0	-1000	0

Escherichia_coli_2733950	0	0	-1000	-1000	-71.454706	-1000	0	0	0
Escherichia_coli_2735000	0	0	-1000	-1000	-70.949993	-1000	0	0	0
Escherichia_coli_2741950	0	0	-1000	-1000	-71.433495	-1000	0	0	0
Escherichia_coli_2747800	0	0	-1000	-1000	-61.394358	-1000	0	0	0
Escherichia_coli_2749250	0	0	-1000	-1000	-70.96231	-1000	0	0	0
Escherichia_coli_2756500	0	0	-1000	-1000	-61.406142	-1000	0	0	0
Escherichia_coli_2762100	0	0	-1000	-1000	-61.790076	-1000	0	-1000	0
Escherichia_coli_2770900	0	0	-1000	-1000	-70.96228	-1000	0	0	0
Escherichia_coli_2780750	0	0	-1000	-1000	-61.832286	-1000	0	0	0
Escherichia_coli_2785200	0	0	-1000	-1000	-71.454676	-1000	0	0	0
Escherichia_coli_2788150	0	0	-1000	-1000	-70.950022	-1000	0	0	0
Escherichia_coli_2845350	0	0	-1000	-1000	-61.52599	-1000	0	0	0
Escherichia_coli_2845650	0	0	-1000	-1000	-70.623219	-1000	0	0	0
Escherichia_coli_2846750	0	0	-1000	-1000	-71.412757	-1000	0	-1000	0
Escherichia_coli_2848050	0	0	-1000	-1000	-70.910562	-1000	0	0	0
Escherichia_coli_2850400	0	0	-1000	-1000	-70.949993	-1000	0	0	0
Escherichia_coli_2850750	0	0	-1000	-1000	-70.949993	-1000	0	0	0
Escherichia_coli_2851500	0	0	-1000	-1000	-70.9759	-1000	0	0	0
Escherichia_coli_2853500	0	0	-1000	-1000	-61.406116	-1000	0	0	0
Escherichia_coli_2854350	0	0	-1000	-1000	-66.058332	-1000	0	0	0
Escherichia_coli_2860050	0	0	-1000	-1000	-75.143668	-1000	0	0	0
Escherichia_coli_2860650	0	0	-1000	-1000	-70.96231	-1000	0	0	0
Escherichia_coli_2861200	0	0	-1000	-1000	-71.433495	-1000	0	-1000	0
Escherichia_coli_2862600	0	0	-1000	-1000	-71.454646	-1000	0	0	0
Escherichia_coli_2864350	0	0	-1000	-1000	-71.433525	-1000	0	0	0
Escherichia_coli_2865200	0	0	-1000	-1000	-70.96231	-1000	0	0	0
Escherichia_coli_2866350	0	0	-1000	-1000	-70.962339	-1000	0	0	0
Escherichia_coli_2866450	0	0	-1000	-1000	-70.949993	-1000	0	0	0
Escherichia_coli_2866550	0	0	-1000	-1000	-61.305379	-1000	0	0	0
Escherichia_coli_2866750	0	0	-1000	-1000	-70.975989	-1000	0	0	0
Escherichia_coli_2867750	0	0	-1000	-1000	-70.949993	-1000	0	0	0
Escherichia_coli_2871950	0	0	-1000	-1000	-71.366841	-1000	0	-1000	0
Escherichia_coli_2872000	0	0	-1000	-1000	-71.379975	-1000	0	-1000	0
Escherichia_coli_2872800	0	0	-1000	-1000	-70.949993	-1000	0	0	0
Escherichia_coli_2875000	0	0	-1000	-1000	-70.949993	-1000	0	0	0
Escherichia_coli_2875150	0	0	-1000	-1000	-71.441544	-1000	0	-1000	0
Escherichia_coli_3_2303	0	0	-1000	-1000	-70.950022	-1000	0	0	0
Escherichia_coli_3_2608	0	0	-1000	-1000	-70.949993	-1000	0	0	0
Escherichia_coli_3_3884	0	0	-1000	-1000	-70.94993	-1000	0	0	0
Escherichia_coli_3_4870	0	0	-1000	-1000	-70.950081	-1000	0	0	0
Escherichia_coli_3003	0	0	-1000	-1000	-82.193228	-1000	0	0	0
Escherichia_coli_3006	0	0	-1000	-1000	-71.433465	-1000	0	0	0
Escherichia_coli_3431	0	0	-1000	-1000	-71.468398	-1000	0	0	0
Escherichia_coli_4_0522	0	0	-1000	-1000	-70.97593	-1000	0	0	0
Escherichia_coli_4_0967	0	0	-1000	-1000	-70.950051	-1000	0	0	0
Escherichia_coli_4_1_47FAA	0	0	-1000	-1000	-53.645273	-1000	0	0	0
Escherichia_coli_5_0588	0	0	-1000	-1000	-71.468423	-1000	0	0	0
Escherichia_coli_5_0959	0	0	-1000	-1000	-71.433465	-1000	0	0	0
Escherichia_coli_5_2239	0	0	-1000	-1000	-70.950081	-1000	0	0	0
Escherichia_coli_536	-1000	0	-1000	-1000	-83.268023	-1000	0	0	0
Escherichia_coli_53638	0	0	-1000	-1000	-71.468368	-1000	0	0	0
Escherichia_coli_536A	0	0	-1000	-1000	-59.746424	-1000	0	0	0
Escherichia_coli_541_1	0	0	-1000	-1000	-70.949993	-1000	0	0	0
Escherichia_coli_541_15	0	0	-1000	-1000	-79.657767	-1000	0	0	0
Escherichia_coli_5412	0	0	-1000	-1000	-79.091433	-1000	0	0	0
Escherichia_coli_55989	-1000	0	-1000	-1000	-79.488516	-1000	0	0	0
Escherichia_coli_5905	0	0	-1000	-1000	-79.080093	-1000	0	0	0
Escherichia_coli_6_0172	0	0	-1000	-1000	-79.080128	-1000	0	0	0
Escherichia_coli_7_1982	0	0	-1000	-1000	-79.080128	-1000	0	0	0
Escherichia_coli_75	0	0	-1000	-1000	-79.196476	-1000	0	0	0
Escherichia_coli_8_0416	0	0	-1000	-1000	-79.080128	-1000	0	0	0
Escherichia_coli_8_0566	0	0	-1000	-1000	-79.661602	-1000	0	0	0
Escherichia_coli_8_0569	0	0	-1000	-1000	-79.661602	-1000	0	0	0
Escherichia_coli_8_0586	0	0	-1000	-1000	-79.080128	-1000	0	0	0
Escherichia_coli_8_2524	0	0	-1000	-1000	-79.080093	-1000	0	0	0
Escherichia_coli_83972	0	0	-1000	-1000	-95.790668	-1000	0	0	0
Escherichia_coli_88_0221	0	0	-1000	-1000	-79.068718	-1000	0	0	0
Escherichia_coli_88_1042	0	0	-1000	-1000	-68.417639	-1000	0	0	0
Escherichia_coli_88_1467	0	0	-1000	-1000	-79.080093	-1000	0	0	0
Escherichia_coli_89_0511	0	0	-1000	-1000	-68.417639	-1000	0	0	0
Escherichia_coli_9_0111	0	0	-1000	-1000	-79.091354	-1000	0	0	0
Escherichia_coli_9_1649	0	0	-1000	-1000	-95.790668	-1000	0	0	0
Escherichia_coli_90_0039	0	0	-1000	-1000	-79.080093	-1000	0	0	0
Escherichia_coli_90_0091	0	0	-1000	-1000	-79.080128	-1000	0	0	0
Escherichia_coli_90_2281	0	0	-1000	-1000	-68.417639	-1000	0	0	0
Escherichia_coli_900105_10e	0	0	-1000	-1000	-79.080015	-1000	0	0	0
Escherichia_coli_93_001	0	0	-1000	-1000	-79.080093	-1000	0	0	0
Escherichia_coli_93_0055	0	0	-1000	-1000	-79.080093	-1000	0	0	0
Escherichia_coli_93_0056	0	0	-1000	-1000	-79.080093	-1000	0	0	0
Escherichia_coli_93_0624	0	0	-1000	-1000	-79.079976	-1000	0	0	0
Escherichia_coli_94_0618	0	0	-1000	-1000	-79.080128	-1000	0	0	0
Escherichia_coli_95_0083	0	0	-1000	-1000	-79.06887	-1000	0	0	0
Escherichia_coli_95_0183	0	0	-1000	-1000	-79.080093	-1000	0	0	0
Escherichia_coli_95_0941	0	0	-1000	-1000	-79.079898	-1000	0	0	0
Escherichia_coli_95_0943	0	0	-1000	-1000	-79.080128	-1000	0	0	0
Escherichia_coli_95_1288	0	0	-1000	-1000	-79.080093	-1000	0	0	0
Escherichia_coli_96_0107	0	0	-1000	-1000	-79.080128	-1000	0	0	0
Escherichia_coli_96_0109	0	0	-1000	-1000	-79.080128	-1000	0	0	0
Escherichia_coli_96_0427	0	0	-1000	-1000	-68.417639	-1000	0	0	0
Escherichia_coli_96_0428	0	0	-1000	-1000	-79.080093	-1000	0	0	0
Escherichia_coli_96_0497	0	0	-1000	-1000	-79.657767	-1000	0	0	0

Escherichia_coli_96_0932	0	0	-1000	-1000	-79.080128	-1000	0	0	0
Escherichia_coli_96_0939	0	0	-1000	-1000	-79.080128	-1000	0	0	0
Escherichia_coli_96_154	0	0	-1000	-1000	-79.079976	-1000	0	0	0
Escherichia_coli_97_0003	0	0	-1000	-1000	-79.080167	-1000	0	0	0
Escherichia_coli_97_0007	0	0	-1000	-1000	-79.131099	-1000	0	0	0
Escherichia_coli_97_0010	0	0	-1000	-1000	-79.080093	-1000	0	0	0
Escherichia_coli_97_0246	0	0	-1000	-1000	-78.2703	-1000	0	0	0
Escherichia_coli_97_0259	0	0	-1000	-1000	-79.196554	-1000	0	0	0
Escherichia_coli_97_0264	0	0	-1000	-1000	-79.079976	-1000	0	0	0
Escherichia_coli_99_0670	0	0	-1000	-1000	-79.080167	-1000	0	0	0
Escherichia_coli_99_0672	0	0	-1000	-1000	-79.080128	-1000	0	0	0
Escherichia_coli_99_0678	0	0	-1000	-1000	-79.080167	-1000	0	0	0
Escherichia_coli_99_0713	0	0	-1000	-1000	-79.080128	-1000	0	0	0
Escherichia_coli_99_0741	0	0	-1000	-1000	-79.673148	-1000	0	0	0
Escherichia_coli_99_0814	0	0	-1000	-1000	-79.080093	-1000	0	0	0
Escherichia_coli_99_0815	0	0	-1000	-1000	-79.080054	-1000	0	0	0
Escherichia_coli_99_0816	0	0	-1000	-1000	-79.080128	-1000	0	0	0
Escherichia_coli_99_0839	0	0	-1000	-1000	-79.080128	-1000	0	0	0
Escherichia_coli_99_0848	0	0	-1000	-1000	-79.080128	-1000	0	0	0
Escherichia_coli_99_1753	0	0	-1000	-1000	-79.091393	-1000	0	0	0
Escherichia_coli_99_1762	0	0	-1000	-1000	-79.080128	-1000	0	0	0
Escherichia_coli_99_1775	0	0	-1000	-1000	-75.407525	-1000	0	0	0
Escherichia_coli_99_1781	0	0	-1000	-1000	-79.080167	-1000	0	0	0
Escherichia_coli_99_1805	0	0	-1000	-1000	-79.080015	-1000	0	0	0
Escherichia_coli_AA86	0	0	-1000	-1000	-95.790668	-1000	0	0	0
Escherichia_coli_ABU_83972	-1000	0	-1000	-1000	-96.324974	-1000	0	0	0
Escherichia_coli_AD30	0	0	-1000	-1000	-79.661641	-1000	0	-1000	0
Escherichia_coli_AI27	0	0	-1000	-1000	-79.091315	-1000	0	0	0
Escherichia_coli_APEC_O1	-1000	0	-1000	-1000	-96.311482	-1000	0	0	0
Escherichia_coli_APEC_O78	-1000	0	-1000	-1000	-79.674107	-1000	0	0	0
Escherichia_coli_ARS4_2123	0	0	-1000	-1000	-95.794003	-1000	0	0	0
Escherichia_coli_ATCC_25922	0	0	-1000	-1000	-95.790668	-1000	0	0	0
Escherichia_coli_ATCC_700728	0	0	-1000	-1000	-79.080128	-1000	0	0	0
Escherichia_coli_ATCC_8739	-1000	0	-1000	-1000	-79.662521	-1000	0	0	0
Escherichia_coli_B_str_REL606	0	0	-1000	-1000	-79.678863	-1000	0	0	0
Escherichia_coli_B088	0	0	-1000	-1000	-79.067578	-1000	0	0	0
Escherichia_coli_B093	0	0	-1000	-1000	-79.678903	-1000	0	0	0
Escherichia_coli_B171	0	0	-1000	-1000	-79.125329	-1000	0	0	0
Escherichia_coli_B185	0	0	-1000	-1000	-78.636388	-1000	0	0	0
Escherichia_coli_B354	0	0	-1000	-1000	-79.207809	-1000	0	0	0
Escherichia_coli_B41	0	0	-1000	-1000	-79.661641	-1000	0	0	0
Escherichia_coli_B799	0	0	-1000	-1000	-79.079703	-1000	0	0	0
Escherichia_coli_B7A	0	0	-1000	-1000	-67.728817	-1000	0	0	0
Escherichia_coli_BCE001_MS16	0	0	-1000	-1000	-79.091315	-1000	0	0	0
Escherichia_coli_BCE002_MS12	0	0	-1000	-1000	-79.091315	-1000	0	0	0
Escherichia_coli_BCE006_MS_23	0	0	-1000	-1000	-78.785672	-1000	0	0	0
Escherichia_coli_BCE007_MS_11	0	0	-1000	-1000	-79.657846	-1000	0	0	0
Escherichia_coli_BCE008_MS_13	0	0	-1000	-1000	-79.657806	-1000	0	-1000	0
Escherichia_coli_BCE011_MS_01	0	0	-1000	-1000	-79.594755	-1000	0	0	0
Escherichia_coli_BCE019_MS_13	0	0	-1000	-1000	-79.657886	-1000	0	0	0
Escherichia_coli_BCE030_MS_09	0	0	-1000	-1000	-68.917531	-1000	0	0	0
Escherichia_coli_BCE032_MS_12	0	0	-1000	-1000	-79.657925	-1000	0	0	0
Escherichia_coli_BCE034_MS_14	0	0	-1000	-1000	-79.080093	-1000	0	0	0
Escherichia_coli_BL21	0	0	-1000	-1000	-53.784923	-1000	0	0	0
Escherichia_coli_BL21DE3	0	0	-1000	-1000	-73.926159	-1000	0	0	0
Escherichia_coli_BW25113	0	0	-1000	-1000	-63.20775	-1000	0	0	0
Escherichia_coli_BW2952	-1000	0	-1000	-1000	-79.66256	-1000	0	0	0
Escherichia_coli_C_34666	0	0	-1000	-1000	-79.673267	-1000	0	0	0
Escherichia_coli_C639_08	0	0	-1000	-1000	-95.794003	-1000	0	-1000	0
Escherichia_coli_C844_97	0	0	-1000	-1000	-95.794003	-1000	0	0	0
Escherichia_coli_CB7326	0	0	-1000	-1000	-79.080167	-1000	0	0	0
Escherichia_coli_CFT073	-1000	0	-1000	-1000	-96.324974	-1000	0	0	0
Escherichia_coli_chi7122	0	0	-1000	-1000	-79.673188	-1000	0	0	0
Escherichia_coli_cloneA_i1	0	0	-1000	-1000	-79.678982	-1000	0	0	0
Escherichia_coli_CUMT8	0	0	-1000	-1000	-79.079976	-1000	0	0	0
Escherichia_coli_D9	0	0	-1000	-1000	-48.363953	-1000	0	0	0
Escherichia_coli_DEC10A	0	0	-1000	-1000	-69.456172	-1000	0	0	0
Escherichia_coli_DEC10B	0	0	-1000	-1000	-79.080054	-1000	0	0	0
Escherichia_coli_DEC10C	0	0	-1000	-1000	-80.107836	-1000	0	0	0
Escherichia_coli_DEC10D	0	0	-1000	-1000	-68.417541	-1000	0	0	0
Escherichia_coli_DEC10E	0	0	-1000	-1000	-79.657806	-1000	0	0	0
Escherichia_coli_DEC10F	0	0	-1000	-1000	-79.712149	-1000	0	0	0
Escherichia_coli_DEC11A	0	0	-1000	-1000	-79.080015	-1000	0	0	0
Escherichia_coli_DEC11B	0	0	-1000	-1000	-68.417507	-1000	0	0	0
Escherichia_coli_DEC11C	0	0	-1000	-1000	-85.289315	-1000	0	0	0
Escherichia_coli_DEC11D	0	0	-1000	-1000	-79.091354	-1000	0	0	0
Escherichia_coli_DEC11E	0	0	-1000	-1000	-79.103135	-1000	0	0	0
Escherichia_coli_DEC12A	0	0	-1000	-1000	-79.080054	-1000	0	0	0
Escherichia_coli_DEC12B	0	0	-1000	-1000	-79.080015	-1000	0	0	0
Escherichia_coli_DEC12C	0	0	-1000	-1000	-79.080054	-1000	0	0	0
Escherichia_coli_DEC12D	0	0	-1000	-1000	-79.080054	-1000	0	0	0
Escherichia_coli_DEC12E	0	0	-1000	-1000	-79.091354	-1000	0	0	0
Escherichia_coli_DEC13A	0	0	-1000	-1000	-79.673108	-1000	0	0	0
Escherichia_coli_DEC13B	0	0	-1000	-1000	-79.673148	-1000	0	0	0
Escherichia_coli_DEC13C	0	0	-1000	-1000	-81.495161	-1000	0	0	0
Escherichia_coli_DEC13D	0	0	-1000	-1000	-79.673108	-1000	0	0	0
Escherichia_coli_DEC13E	0	0	-1000	-1000	-79.673188	-1000	0	0	0
Escherichia_coli_DEC14A	0	0	-1000	-1000	-79.657846	-1000	0	0	0
Escherichia_coli_DEC14B	0	0	-1000	-1000	-79.632968	-1000	0	0	0
Escherichia_coli_DEC14C	0	0	-1000	-1000	-79.657767	-1000	0	0	0
Escherichia_coli_DEC14D	0	0	-1000	-1000	-79.196476	-1000	0	0	0

Escherichia_coli_DEC15A	0	0	-1000	-1000	-79.080015	-1000	0	0	0
Escherichia_coli_DEC15B	0	0	-1000	-1000	-79.080015	-1000	0	0	0
Escherichia_coli_DEC15C	0	0	-1000	-1000	-68.412637	-1000	0	0	0
Escherichia_coli_DEC15D	0	0	-1000	-1000	-79.080015	-1000	0	0	0
Escherichia_coli_DEC15E	0	0	-1000	-1000	-80.897145	-1000	0	0	0
Escherichia_coli_DEC1A	0	0	-1000	-1000	-73.894252	-1000	0	0	0
Escherichia_coli_DEC1B	0	0	-1000	-1000	-81.477738	-1000	0	0	0
Escherichia_coli_DEC1C	0	0	-1000	-1000	-79.656008	-1000	0	0	0
Escherichia_coli_DEC1D	0	0	-1000	-1000	-81.477708	-1000	0	0	0
Escherichia_coli_DEC1E	0	0	-1000	-1000	-79.655968	-1000	0	0	0
Escherichia_coli_DEC2A	0	0	-1000	-1000	-79.655968	-1000	0	0	0
Escherichia_coli_DEC2B	0	0	-1000	-1000	-79.656008	-1000	0	0	0
Escherichia_coli_DEC2C	0	0	-1000	-1000	-79.656008	-1000	0	0	0
Escherichia_coli_DEC2D	0	0	-1000	-1000	-79.656008	-1000	0	0	0
Escherichia_coli_DEC2E	0	0	-1000	-1000	-73.910248	-1000	0	0	0
Escherichia_coli_DEC3A	0	0	-1000	-1000	-79.080093	-1000	0	0	0
Escherichia_coli_DEC3B	0	0	-1000	-1000	-79.080093	-1000	0	0	0
Escherichia_coli_DEC3C	0	0	-1000	-1000	-73.368611	-1000	0	0	0
Escherichia_coli_DEC3D	0	0	-1000	-1000	-73.368611	-1000	0	0	0
Escherichia_coli_DEC3E	0	0	-1000	-1000	-79.080167	-1000	0	0	0
Escherichia_coli_DEC3F	0	0	-1000	-1000	-79.091467	-1000	0	0	0
Escherichia_coli_DEC4A	0	0	-1000	-1000	-81.089338	-1000	0	0	0
Escherichia_coli_DEC4B	0	0	-1000	-1000	-79.080128	-1000	0	0	0
Escherichia_coli_DEC4C	0	0	-1000	-1000	-80.94874	-1000	0	0	0
Escherichia_coli_DEC4D	0	0	-1000	-1000	-79.131054	-1000	0	0	0
Escherichia_coli_DEC4E	0	0	-1000	-1000	-79.125908	-1000	0	0	0
Escherichia_coli_DEC4F	0	0	-1000	-1000	-70.336799	-1000	0	0	0
Escherichia_coli_DEC5A	0	0	-1000	-1000	-79.068757	-1000	0	0	0
Escherichia_coli_DEC5B	0	0	-1000	-1000	-79.080054	-1000	0	0	0
Escherichia_coli_DEC5C	0	0	-1000	-1000	-79.080093	-1000	0	0	0
Escherichia_coli_DEC5D	0	0	-1000	-1000	-79.080093	-1000	0	0	0
Escherichia_coli_DEC5E	0	0	-1000	-1000	-79.080054	-1000	0	0	0
Escherichia_coli_DEC6A	0	0	-1000	-1000	-79.594755	-1000	0	0	0
Escherichia_coli_DEC6B	0	0	-1000	-1000	-73.205847	-1000	0	0	0
Escherichia_coli_DEC6C	0	0	-1000	-1000	-79.708173	-1000	0	0	0
Escherichia_coli_DEC6D	0	0	-1000	-1000	-78.797393	-1000	0	0	0
Escherichia_coli_DEC6E	0	0	-1000	-1000	-79.646344	-1000	0	0	0
Escherichia_coli_DEC7A	0	0	-1000	-1000	-85.905449	-1000	0	-1000	0
Escherichia_coli_DEC7B	0	0	-1000	-1000	-79.673227	-1000	0	0	0
Escherichia_coli_DEC7C	0	0	-1000	-1000	-85.905449	-1000	0	0	0
Escherichia_coli_DEC7D	0	0	-1000	-1000	-85.905449	-1000	0	-1000	0
Escherichia_coli_DEC7E	0	0	-1000	-1000	-85.905449	-1000	0	0	0
Escherichia_coli_DEC8A	0	0	-1000	-1000	-80.8972	-1000	0	0	0
Escherichia_coli_DEC8B	0	0	-1000	-1000	-79.080015	-1000	0	0	0
Escherichia_coli_DEC8C	0	0	-1000	-1000	-80.908654	-1000	0	0	0
Escherichia_coli_DEC8D	0	0	-1000	-1000	-68.417541	-1000	0	0	0
Escherichia_coli_DEC8E	0	0	-1000	-1000	-79.080093	-1000	0	0	0
Escherichia_coli_DEC9A	0	0	-1000	-1000	-79.080015	-1000	0	0	0
Escherichia_coli_DEC9B	0	0	-1000	-1000	-68.451751	-1000	0	0	0
Escherichia_coli_DEC9C	0	0	-1000	-1000	-79.500616	-1000	0	0	0
Escherichia_coli_DEC9D	0	0	-1000	-1000	-79.079976	-1000	0	0	0
Escherichia_coli_DEC9E	0	0	-1000	-1000	-79.079976	-1000	0	0	0
Escherichia_coli_DH1	-1000	0	-1000	-1000	-79.66256	-1000	0	0	0
Escherichia_coli_E101	0	0	-1000	-1000	-78.63662	-1000	0	0	0
Escherichia_coli_E110019	0	0	-1000	-1000	-79.130907	-1000	0	0	0
Escherichia_coli_E1167	0	0	-1000	-1000	-79.079859	-1000	0	0	0
Escherichia_coli_E128010	0	0	-1000	-1000	-80.897145	-1000	0	0	0
Escherichia_coli_E1520	0	0	-1000	-1000	-79.661522	-1000	0	0	0
Escherichia_coli_E22	0	0	-1000	-1000	-79.119605	-1000	0	0	0
Escherichia_coli_E2348_69	0	0	-1000	-1000	-79.678982	-1000	0	0	0
Escherichia_coli_E24377A	-1000	0	-1000	-1000	-68.931565	-1000	0	-1000	0
Escherichia_coli_E482	0	0	-1000	-1000	-79.673069	-1000	0	0	0
Escherichia_coli_EC1734	0	0	-1000	-1000	-79.080128	-1000	0	0	0
Escherichia_coli_EC1735	0	0	-1000	-1000	-79.080128	-1000	0	0	0
Escherichia_coli_EC1736	0	0	-1000	-1000	-79.080128	-1000	0	0	0
Escherichia_coli_EC1737	0	0	-1000	-1000	-79.080128	-1000	0	0	0
Escherichia_coli_EC1738	0	0	-1000	-1000	-79.080093	-1000	0	0	0
Escherichia_coli_EC1845	0	0	-1000	-1000	-79.080128	-1000	0	0	0
Escherichia_coli_EC1846	0	0	-1000	-1000	-79.080093	-1000	0	0	0
Escherichia_coli_EC1847	0	0	-1000	-1000	-79.080128	-1000	0	0	0
Escherichia_coli_EC1848	0	0	-1000	-1000	-79.080128	-1000	0	0	0
Escherichia_coli_EC1849	0	0	-1000	-1000	-79.080128	-1000	0	0	0
Escherichia_coli_EC1850	0	0	-1000	-1000	-79.080128	-1000	0	0	0
Escherichia_coli_EC1856	0	0	-1000	-1000	-79.080128	-1000	0	0	0
Escherichia_coli_EC1862	0	0	-1000	-1000	-79.080093	-1000	0	0	0
Escherichia_coli_EC1863	0	0	-1000	-1000	-79.080128	-1000	0	0	0
Escherichia_coli_EC1864	0	0	-1000	-1000	-79.080128	-1000	0	0	0
Escherichia_coli_EC1865	0	0	-1000	-1000	-95.100797	-1000	0	0	0
Escherichia_coli_EC1866	0	0	-1000	-1000	-79.080128	-1000	0	0	0
Escherichia_coli_EC1869	0	0	-1000	-1000	-79.080128	-1000	0	0	0
Escherichia_coli_EC1870	0	0	-1000	-1000	-79.080167	-1000	0	0	0
Escherichia_coli_EC302_04	0	0	-1000	-1000	-79.196437	-1000	0	0	0
Escherichia_coli_EC4013	0	0	-1000	-1000	-79.080128	-1000	0	0	0
Escherichia_coli_EC4100B	0	0	-1000	-1000	-79.673227	-1000	0	0	0
Escherichia_coli_EC4196	0	0	-1000	-1000	-79.080128	-1000	0	0	0
Escherichia_coli_EC4203	0	0	-1000	-1000	-79.080128	-1000	0	0	0
Escherichia_coli_EC4402	0	0	-1000	-1000	-79.080093	-1000	0	0	0
Escherichia_coli_EC4421	0	0	-1000	-1000	-79.080128	-1000	0	0	0
Escherichia_coli_EC4422	0	0	-1000	-1000	-79.080128	-1000	0	0	0
Escherichia_coli_EC4436	0	0	-1000	-1000	-79.080128	-1000	0	0	0
Escherichia_coli_EC4437	0	0	-1000	-1000	-79.080128	-1000	0	0	0

Escherichia_coli_EC4439	0	0	-1000	-1000	-79.080128	-1000	0	0	0
Escherichia_coli_EC4448	0	0	-1000	-1000	-79.080167	-1000	0	0	0
Escherichia_coli_ED1a	-1000	0	-1000	-1000	-96.324974	-1000	0	0	0
Escherichia_coli_Envira_10_1	0	0	-1000	-1000	-79.091354	-1000	0	0	0
Escherichia_coli_Envira_8_11	0	0	-1000	-1000	-79.091354	-1000	0	0	0
Escherichia_coli_EPEC_C342_62	0	0	-1000	-1000	-80.897145	-1000	0	0	0
Escherichia_coli_EPECa12	0	0	-1000	-1000	-81.495161	-1000	0	0	0
Escherichia_coli_EPECa14	0	0	-1000	-1000	-70.670514	-1000	0	0	0
Escherichia_coli_ERR1203920	0	0	-1000	-1000	-83.171468	-1000	0	0	0
Escherichia_coli_ERR2221112	0	0	-1000	-1000	-62.910293	-1000	0	0	0
Escherichia_coli_ERR2221113	0	0	-1000	-1000	-62.910293	-1000	0	0	0
Escherichia_coli_ERR2221128	0	0	-1000	-1000	-83.171468	-1000	0	0	0
Escherichia_coli_ERR2221214	0	0	-1000	-1000	-83.171468	-1000	0	0	0
Escherichia_coli_ERR2221221	0	0	-1000	-1000	-63.212227	-1000	0	0	0
Escherichia_coli_ERR2221222	0	0	-1000	-1000	-83.171468	-1000	0	0	0
Escherichia_coli_ERR2221227	0	0	-1000	-1000	-83.171468	-1000	0	0	0
Escherichia_coli_ERR2221250	0	0	-1000	-1000	-63.212227	-1000	0	0	0
Escherichia_coli_ERR2221318	0	0	-1000	-1000	-83.171468	-1000	0	0	0
Escherichia_coli_ERR2221398	0	0	-1000	-1000	-63.20775	-1000	0	0	0
Escherichia_coli_ERR2221401	0	0	-1000	-1000	-63.20775	-1000	0	0	0
Escherichia_coli_ETEC_H10407	-1000	0	-1000	-1000	-79.657806	-1000	0	0	0
Escherichia_coli_F11	0	0	-1000	-1000	-99.812667	-1000	0	0	0
Escherichia_coli_FDA504	0	0	-1000	-1000	-79.080093	-1000	0	0	0
Escherichia_coli_FDA505	0	0	-1000	-1000	-68.417639	-1000	0	0	0
Escherichia_coli_FDA506	0	0	-1000	-1000	-79.080167	-1000	0	0	0
Escherichia_coli_FDA507	0	0	-1000	-1000	-79.080128	-1000	0	0	0
Escherichia_coli_FDA517	0	0	-1000	-1000	-79.080128	-1000	0	0	0
Escherichia_coli_FDAARGOS_170	0	0	-1000	-1000	-83.171468	-1000	0	0	0
Escherichia_coli_FRIK1985	0	0	-1000	-1000	-79.080128	-1000	0	0	0
Escherichia_coli_FRIK1990	0	0	-1000	-1000	-79.080093	-1000	0	0	0
Escherichia_coli_FRIK1996	0	0	-1000	-1000	-79.080093	-1000	0	0	0
Escherichia_coli_FRIK1997	0	0	-1000	-1000	-79.080128	-1000	0	0	0
Escherichia_coli_FRIK1999	0	0	-1000	-1000	-79.080128	-1000	0	0	0
Escherichia_coli_FRIK2001	0	0	-1000	-1000	-79.080093	-1000	0	0	0
Escherichia_coli_FRIK523	0	0	-1000	-1000	-79.080128	-1000	0	0	0
Escherichia_coli_FRIK920	0	0	-1000	-1000	-79.080167	-1000	0	0	0
Escherichia_coli_FVEC1302	0	0	-1000	-1000	-80.990548	-1000	0	0	0
Escherichia_coli_FVEC1412	0	0	-1000	-1000	-80.990548	-1000	0	0	0
Escherichia_coli_G58_1	0	0	-1000	-1000	-79.661641	-1000	0	0	0
Escherichia_coli_H120	0	0	-1000	-1000	-79.118654	-1000	0	0	0
Escherichia_coli_H252	0	0	-1000	-1000	-95.777332	-1000	0	0	0
Escherichia_coli_H263	0	0	-1000	-1000	-95.777332	-1000	0	0	0
Escherichia_coli_H299	0	0	-1000	-1000	-79.678625	-1000	0	0	0
Escherichia_coli_H30	0	0	-1000	-1000	-79.080015	-1000	0	0	0
Escherichia_coli_H397	0	0	-1000	-1000	-95.777332	-1000	0	0	0
Escherichia_coli_H489	0	0	-1000	-1000	-79.678625	-1000	0	0	0
Escherichia_coli_H494	0	0	-1000	-1000	-79.657806	-1000	0	0	0
Escherichia_coli_H591	0	0	-1000	-1000	-79.079742	-1000	0	0	0
Escherichia_coli_H730	0	0	-1000	-1000	-68.920369	-1000	0	0	0
Escherichia_coli_H736	0	0	-1000	-1000	-78.826342	-1000	0	0	0
Escherichia_coli_HM26	0	0	-1000	-1000	-79.657846	-1000	0	0	0
Escherichia_coli_HM27	0	0	-1000	-1000	-79.720144	-1000	0	0	0
Escherichia_coli_HM46	0	0	-1000	-1000	-79.207809	-1000	0	0	0
Escherichia_coli_HM605	0	0	-1000	-1000	-79.667156	-1000	0	0	0
Escherichia_coli_HM65	0	0	-1000	-1000	-99.812667	-1000	0	0	0
Escherichia_coli_HS	-1000	0	-1000	-1000	-79.207809	-1000	0	0	0
Escherichia_coli_IAI1	-1000	0	-1000	-1000	-79.674067	-1000	0	0	0
Escherichia_coli_IAI39	-1000	0	-1000	-1000	-85.911464	-1000	0	0	0
Escherichia_coli_IHE3034	-1000	0	-1000	-1000	-96.311489	-1000	0	0	0
Escherichia_coli_IMT2125	0	0	-1000	-1000	-55.303229	-1000	0	0	0
Escherichia_coli_J53	0	0	-1000	-1000	-79.661602	-1000	0	0	0
Escherichia_coli_JB1_95	0	0	-1000	-1000	-79.068757	-1000	0	0	0
Escherichia_coli_Jurua_18_11	0	0	-1000	-1000	-79.673148	-1000	0	0	0
Escherichia_coli_Jurua_20_10	0	0	-1000	-1000	-79.080015	-1000	0	0	0
Escherichia_coli_KD2	0	0	-1000	-1000	-79.213615	-1000	0	0	0
Escherichia_coli_KO11	-1000	0	-1000	-1000	-53.634601	-1000	0	0	0
Escherichia_coli_KTE1	0	0	-1000	-1000	-80.990507	-1000	0	0	0
Escherichia_coli_KTE10	0	0	-1000	-1000	-79.660286	-1000	0	0	0
Escherichia_coli_KTE100	0	0	-1000	-1000	-79.672712	-1000	0	0	0
Escherichia_coli_KTE101	0	0	-1000	-1000	-79.67291	-1000	0	0	0
Escherichia_coli_KTE102	0	0	-1000	-1000	-95.784	-1000	0	0	0
Escherichia_coli_KTE103	0	0	-1000	-1000	-79.657529	-1000	0	0	0
Escherichia_coli_KTE104	0	0	-1000	-1000	-95.790668	-1000	0	0	0
Escherichia_coli_KTE105	0	0	-1000	-1000	-80.990788	-1000	0	0	0
Escherichia_coli_KTE106	0	0	-1000	-1000	-95.790668	-1000	0	0	0
Escherichia_coli_KTE107	0	0	-1000	-1000	-79.090964	-1000	0	0	0
Escherichia_coli_KTE108	0	0	-1000	-1000	-79.657331	-1000	0	0	0
Escherichia_coli_KTE109	0	0	-1000	-1000	-95.800673	-1000	0	0	0
Escherichia_coli_KTE11	0	0	-1000	-1000	-46.201808	-1000	0	0	0
Escherichia_coli_KTE111	0	0	-1000	-1000	-79.079664	-1000	0	0	0
Escherichia_coli_KTE112	0	0	-1000	-1000	-78.63662	-1000	0	0	0
Escherichia_coli_KTE113	0	0	-1000	-1000	-95.800673	-1000	0	0	0
Escherichia_coli_KTE114	0	0	-1000	-1000	-45.698311	-1000	0	0	0
Escherichia_coli_KTE115	0	0	-1000	-1000	-79.657688	-1000	0	0	0
Escherichia_coli_KTE116	0	0	-1000	-1000	-80.990507	-1000	0	0	0
Escherichia_coli_KTE117	0	0	-1000	-1000	-78.636465	-1000	0	0	0
Escherichia_coli_KTE118	0	0	-1000	-1000	-95.777332	-1000	0	0	0
Escherichia_coli_KTE119	0	0	-1000	-1000	-79.672871	-1000	0	0	0
Escherichia_coli_KTE12	0	0	-1000	-1000	-79.079781	-1000	0	0	0
Escherichia_coli_KTE120	0	0	-1000	-1000	-79.661681	-1000	0	0	0
Escherichia_coli_KTE121	0	0	-1000	-1000	-79.657331	-1000	0	0	0

Escherichia_coli_KTE122	0	0	-1000	-1000	-80.990708	-1000	0	0	0
Escherichia_coli_KTE123	0	0	-1000	-1000	-95.777332	-1000	0	0	0
Escherichia_coli_KTE124	0	0	-1000	-1000	-95.790668	-1000	0	0	0
Escherichia_coli_KTE125	0	0	-1000	-1000	-79.207927	-1000	0	0	0
Escherichia_coli_KTE126	0	0	-1000	-1000	-95.800673	-1000	0	0	0
Escherichia_coli_KTE127	0	0	-1000	-1000	-80.987319	-1000	0	0	0
Escherichia_coli_KTE128	0	0	-1000	-1000	-80.9878	-1000	0	0	0
Escherichia_coli_KTE129	0	0	-1000	-1000	-79.678903	-1000	0	0	0
Escherichia_coli_KTE13	0	0	-1000	-1000	-79.079703	-1000	0	0	0
Escherichia_coli_KTE130	0	0	-1000	-1000	-79.661205	-1000	0	0	0
Escherichia_coli_KTE131	0	0	-1000	-1000	-95.790668	-1000	0	0	0
Escherichia_coli_KTE132	0	0	-1000	-1000	-79.661166	-1000	0	0	0
Escherichia_coli_KTE133	0	0	-1000	-1000	-95.800673	-1000	0	0	0
Escherichia_coli_KTE134	0	0	-1000	-1000	-80.98768	-1000	0	0	0
Escherichia_coli_KTE135	0	0	-1000	-1000	-79.079624	-1000	0	0	0
Escherichia_coli_KTE136	0	0	-1000	-1000	-79.079781	-1000	0	0	0
Escherichia_coli_KTE137	0	0	-1000	-1000	-95.790668	-1000	0	0	0
Escherichia_coli_KTE138	0	0	-1000	-1000	-79.079976	-1000	0	0	0
Escherichia_coli_KTE139	0	0	-1000	-1000	-95.790668	-1000	0	0	0
Escherichia_coli_KTE14	0	0	-1000	-1000	-79.661166	-1000	0	0	0
Escherichia_coli_KTE140	0	0	-1000	-1000	-80.98772	-1000	0	0	0
Escherichia_coli_KTE141	0	0	-1000	-1000	-95.790668	-1000	0	0	0
Escherichia_coli_KTE142	0	0	-1000	-1000	-79.057073	-1000	0	0	0
Escherichia_coli_KTE143	0	0	-1000	-1000	-68.935543	-1000	0	0	0
Escherichia_coli_KTE144	0	0	-1000	-1000	-79.196358	-1000	0	0	0
Escherichia_coli_KTE145	0	0	-1000	-1000	-95.790668	-1000	0	0	0
Escherichia_coli_KTE146	0	0	-1000	-1000	-79.196397	-1000	0	0	0
Escherichia_coli_KTE147	0	0	-1000	-1000	-79.207692	-1000	0	0	0
Escherichia_coli_KTE148	0	0	-1000	-1000	-95.790668	-1000	0	0	0
Escherichia_coli_KTE15	0	0	-1000	-1000	-95.790668	-1000	0	0	0
Escherichia_coli_KTE150	0	0	-1000	-1000	-95.800673	-1000	0	0	0
Escherichia_coli_KTE153	0	0	-1000	-1000	-95.790668	-1000	0	0	0
Escherichia_coli_KTE154	0	0	-1000	-1000	-79.678625	-1000	0	0	0
Escherichia_coli_KTE155	0	0	-1000	-1000	-79.661483	-1000	0	-1000	0
Escherichia_coli_KTE156	0	0	-1000	-1000	-79.661324	-1000	0	-1000	0
Escherichia_coli_KTE157	0	0	-1000	-1000	-79.678942	-1000	0	0	0
Escherichia_coli_KTE158	0	0	-1000	-1000	-80.98752	-1000	0	0	0
Escherichia_coli_KTE159	0	0	-1000	-1000	-46.201808	-1000	0	0	0
Escherichia_coli_KTE16	0	0	-1000	-1000	-95.790668	-1000	0	0	0
Escherichia_coli_KTE160	0	0	-1000	-1000	-95.790668	-1000	0	0	0
Escherichia_coli_KTE161	0	0	-1000	-1000	-79.661285	-1000	0	0	0
Escherichia_coli_KTE162	0	0	-1000	-1000	-95.790668	-1000	0	0	0
Escherichia_coli_KTE163	0	0	-1000	-1000	-79.673227	-1000	0	0	0
Escherichia_coli_KTE165	0	0	-1000	-1000	-95.800673	-1000	0	0	0
Escherichia_coli_KTE166	0	0	-1000	-1000	-79.673148	-1000	0	0	0
Escherichia_coli_KTE167	0	0	-1000	-1000	-95.790668	-1000	0	0	0
Escherichia_coli_KTE168	0	0	-1000	-1000	-79.678982	-1000	0	0	0
Escherichia_coli_KTE169	0	0	-1000	-1000	-79.678784	-1000	0	0	0
Escherichia_coli_KTE17	0	0	-1000	-1000	-95.800673	-1000	0	0	0
Escherichia_coli_KTE170	0	0	-1000	-1000	-80.987279	-1000	0	0	0
Escherichia_coli_KTE171	0	0	-1000	-1000	-79.67299	-1000	0	0	0
Escherichia_coli_KTE172	0	0	-1000	-1000	-44.062833	-1000	0	0	0
Escherichia_coli_KTE173	0	0	-1000	-1000	-95.790668	-1000	0	0	0
Escherichia_coli_KTE174	0	0	-1000	-1000	-95.790668	-1000	0	0	0
Escherichia_coli_KTE175	0	0	-1000	-1000	-95.790668	-1000	0	0	0
Escherichia_coli_KTE176	0	0	-1000	-1000	-79.667474	-1000	0	0	0
Escherichia_coli_KTE177	0	0	-1000	-1000	-80.98784	-1000	0	0	0
Escherichia_coli_KTE178	0	0	-1000	-1000	-72.459402	-1000	0	0	0
Escherichia_coli_KTE179	0	0	-1000	-1000	-95.777332	-1000	0	0	0
Escherichia_coli_KTE18	0	0	-1000	-1000	-95.800673	-1000	0	0	0
Escherichia_coli_KTE180	0	0	-1000	-1000	-95.777332	-1000	0	0	0
Escherichia_coli_KTE181	0	0	-1000	-1000	-80.990507	-1000	0	0	0
Escherichia_coli_KTE182	0	0	-1000	-1000	-79.678586	-1000	0	0	0
Escherichia_coli_KTE183	0	0	-1000	-1000	-95.790668	-1000	0	0	0
Escherichia_coli_KTE184	0	0	-1000	-1000	-79.079937	-1000	0	0	0
Escherichia_coli_KTE185	0	0	-1000	-1000	-95.800673	-1000	0	0	0
Escherichia_coli_KTE186	0	0	-1000	-1000	-95.790668	-1000	0	0	0
Escherichia_coli_KTE187	0	0	-1000	-1000	-95.790668	-1000	0	0	0
Escherichia_coli_KTE188	0	0	-1000	-1000	-96.324974	-1000	0	0	0
Escherichia_coli_KTE189	0	0	-1000	-1000	-95.790668	-1000	0	0	0
Escherichia_coli_KTE19	0	0	-1000	-1000	-91.652011	-1000	0	0	0
Escherichia_coli_KTE190	0	0	-1000	-1000	-80.990507	-1000	0	0	0
Escherichia_coli_KTE191	0	0	-1000	-1000	-95.790668	-1000	0	0	0
Escherichia_coli_KTE192	0	0	-1000	-1000	-95.800673	-1000	0	0	0
Escherichia_coli_KTE193	0	0	-1000	-1000	-78.636234	-1000	0	0	0
Escherichia_coli_KTE194	0	0	-1000	-1000	-95.800673	-1000	0	0	0
Escherichia_coli_KTE195	0	0	-1000	-1000	-95.790668	-1000	0	0	0
Escherichia_coli_KTE196	0	0	-1000	-1000	-78.636311	-1000	0	0	0
Escherichia_coli_KTE197	0	0	-1000	-1000	-79.661483	-1000	0	-1000	0
Escherichia_coli_KTE198	0	0	-1000	-1000	-79.661126	-1000	0	0	0
Escherichia_coli_KTE199	0	0	-1000	-1000	-79.657569	-1000	0	0	0
Escherichia_coli_KTE2	0	0	-1000	-1000	-80.990227	-1000	0	0	0
Escherichia_coli_KTE20	0	0	-1000	-1000	-79.657648	-1000	0	0	0
Escherichia_coli_KTE200	0	0	-1000	-1000	-80.990507	-1000	0	0	0
Escherichia_coli_KTE201	0	0	-1000	-1000	-95.790668	-1000	0	0	0
Escherichia_coli_KTE202	0	0	-1000	-1000	-79.20777	-1000	0	0	0
Escherichia_coli_KTE203	0	0	-1000	-1000	-79.079937	-1000	0	0	0
Escherichia_coli_KTE204	0	0	-1000	-1000	-80.9876	-1000	0	0	0
Escherichia_coli_KTE205	0	0	-1000	-1000	-79.678665	-1000	0	0	0
Escherichia_coli_KTE206	0	0	-1000	-1000	-95.790668	-1000	0	0	0
Escherichia_coli_KTE207	0	0	-1000	-1000	-95.800673	-1000	0	0	0

Escherichia_coli_KTE208	0	0	-1000	-1000	-80.98768	-1000	0	0	0
Escherichia_coli_KTE209	0	0	-1000	-1000	-95.790668	-1000	0	0	0
Escherichia_coli_KTE21	0	0	-1000	-1000	-79.657767	-1000	0	0	0
Escherichia_coli_KTE210	0	0	-1000	-1000	-79.673029	-1000	0	0	0
Escherichia_coli_KTE211	0	0	-1000	-1000	-95.790668	-1000	0	0	0
Escherichia_coli_KTE212	0	0	-1000	-1000	-79.672831	-1000	0	0	0
Escherichia_coli_KTE213	0	0	-1000	-1000	-80.98764	-1000	0	0	0
Escherichia_coli_KTE214	0	0	-1000	-1000	-95.790668	-1000	0	0	0
Escherichia_coli_KTE215	0	0	-1000	-1000	-95.790668	-1000	0	0	0
Escherichia_coli_KTE216	0	0	-1000	-1000	-95.790668	-1000	0	0	0
Escherichia_coli_KTE217	0	0	-1000	-1000	-95.800673	-1000	0	0	0
Escherichia_coli_KTE218	0	0	-1000	-1000	-95.790668	-1000	0	0	0
Escherichia_coli_KTE219	0	0	-1000	-1000	-79.678586	-1000	0	0	0
Escherichia_coli_KTE22	0	0	-1000	-1000	-95.777332	-1000	0	0	0
Escherichia_coli_KTE220	0	0	-1000	-1000	-95.790668	-1000	0	0	0
Escherichia_coli_KTE221	0	0	-1000	-1000	-79.673029	-1000	0	0	0
Escherichia_coli_KTE222	0	0	-1000	-1000	-79.67299	-1000	0	0	0
Escherichia_coli_KTE223	0	0	-1000	-1000	-95.790668	-1000	0	0	0
Escherichia_coli_KTE224	0	0	-1000	-1000	-79.678506	-1000	0	0	0
Escherichia_coli_KTE225	0	0	-1000	-1000	-80.98756	-1000	0	0	0
Escherichia_coli_KTE226	0	0	-1000	-1000	-95.790668	-1000	0	0	0
Escherichia_coli_KTE227	0	0	-1000	-1000	-79.667315	-1000	0	0	0
Escherichia_coli_KTE228	0	0	-1000	-1000	-80.98756	-1000	0	0	0
Escherichia_coli_KTE229	0	0	-1000	-1000	-95.777332	-1000	0	0	0
Escherichia_coli_KTE23	0	0	-1000	-1000	-95.800673	-1000	0	0	0
Escherichia_coli_KTE230	0	0	-1000	-1000	-96.324974	-1000	0	0	0
Escherichia_coli_KTE231	0	0	-1000	-1000	-80.98776	-1000	0	0	0
Escherichia_coli_KTE232	0	0	-1000	-1000	-79.673069	-1000	0	0	0
Escherichia_coli_KTE233	0	0	-1000	-1000	-79.672831	-1000	0	0	0
Escherichia_coli_KTE234	0	0	-1000	-1000	-79.672593	-1000	0	0	0
Escherichia_coli_KTE235	0	0	-1000	-1000	-80.990427	-1000	0	0	0
Escherichia_coli_KTE236	0	0	-1000	-1000	-79.657688	-1000	0	0	0
Escherichia_coli_KTE237	0	0	-1000	-1000	-79.657252	-1000	0	0	0
Escherichia_coli_KTE24	0	0	-1000	-1000	-68.935543	-1000	0	0	0
Escherichia_coli_KTE240	0	0	-1000	-1000	-95.777332	-1000	0	0	0
Escherichia_coli_KTE25	0	0	-1000	-1000	-95.800673	-1000	0	0	0
Escherichia_coli_KTE26	0	0	-1000	-1000	-80.990548	-1000	0	0	0
Escherichia_coli_KTE27	0	0	-1000	-1000	-79.667196	-1000	0	0	0
Escherichia_coli_KTE28	0	0	-1000	-1000	-95.800673	-1000	0	0	0
Escherichia_coli_KTE29	0	0	-1000	-1000	-79.091081	-1000	0	0	0
Escherichia_coli_KTE3	0	0	-1000	-1000	-95.777332	-1000	0	0	0
Escherichia_coli_KTE31	0	0	-1000	-1000	-45.698311	-1000	0	0	0
Escherichia_coli_KTE33	0	0	-1000	-1000	-79.672752	-1000	0	0	0
Escherichia_coli_KTE34	0	0	-1000	-1000	-79.661364	-1000	0	0	0
Escherichia_coli_KTE35	0	0	-1000	-1000	-79.661364	-1000	0	0	0
Escherichia_coli_KTE36	0	0	-1000	-1000	-79.678744	-1000	0	0	0
Escherichia_coli_KTE37	0	0	-1000	-1000	-68.935646	-1000	0	0	0
Escherichia_coli_KTE38	0	0	-1000	-1000	-68.935303	-1000	0	0	0
Escherichia_coli_KTE39	0	0	-1000	-1000	-95.790668	-1000	0	0	0
Escherichia_coli_KTE4	0	0	-1000	-1000	-95.777332	-1000	0	0	0
Escherichia_coli_KTE40	0	0	-1000	-1000	-79.196241	-1000	0	0	0
Escherichia_coli_KTE41	0	0	-1000	-1000	-54.977754	-1000	0	0	0
Escherichia_coli_KTE42	0	0	-1000	-1000	-79.661443	-1000	0	0	0
Escherichia_coli_KTE43	0	0	-1000	-1000	-79.678823	-1000	0	0	0
Escherichia_coli_KTE44	0	0	-1000	-1000	-79.67295	-1000	0	0	0
Escherichia_coli_KTE45	0	0	-1000	-1000	-95.800673	-1000	0	0	0
Escherichia_coli_KTE46	0	0	-1000	-1000	-95.800673	-1000	0	0	0
Escherichia_coli_KTE47	0	0	-1000	-1000	-95.800673	-1000	0	0	0
Escherichia_coli_KTE48	0	0	-1000	-1000	-78.63635	-1000	0	0	0
Escherichia_coli_KTE49	0	0	-1000	-1000	-95.790668	-1000	0	-1000	0
Escherichia_coli_KTE5	0	0	-1000	-1000	-79.667156	-1000	0	0	0
Escherichia_coli_KTE50	0	0	-1000	-1000	-79.207613	-1000	0	0	0
Escherichia_coli_KTE51	0	0	-1000	-1000	-79.661205	-1000	0	0	0
Escherichia_coli_KTE53	0	0	-1000	-1000	-95.790668	-1000	0	0	0
Escherichia_coli_KTE54	0	0	-1000	-1000	-79.207809	-1000	0	0	0
Escherichia_coli_KTE55	0	0	-1000	-1000	-95.790668	-1000	0	0	0
Escherichia_coli_KTE56	0	0	-1000	-1000	-79.67299	-1000	0	0	0
Escherichia_coli_KTE57	0	0	-1000	-1000	-79.678784	-1000	0	0	0
Escherichia_coli_KTE58	0	0	-1000	-1000	-95.777332	-1000	0	0	0
Escherichia_coli_KTE59	0	0	-1000	-1000	-95.777332	-1000	0	0	0
Escherichia_coli_KTE6	0	0	-1000	-1000	-95.790668	-1000	0	0	0
Escherichia_coli_KTE60	0	0	-1000	-1000	-95.790668	-1000	0	0	0
Escherichia_coli_KTE61	0	0	-1000	-1000	-79.661324	-1000	0	0	0
Escherichia_coli_KTE62	0	0	-1000	-1000	-79.667315	-1000	0	0	0
Escherichia_coli_KTE63	0	0	-1000	-1000	-95.790668	-1000	0	0	0
Escherichia_coli_KTE64	0	0	-1000	-1000	-79.681463	-1000	0	0	0
Escherichia_coli_KTE65	0	0	-1000	-1000	-95.777332	-1000	0	0	0
Escherichia_coli_KTE66	0	0	-1000	-1000	-79.196358	-1000	0	0	0
Escherichia_coli_KTE67	0	0	-1000	-1000	-85.43492	-1000	0	0	0
Escherichia_coli_KTE68	0	0	-1000	-1000	-80.990227	-1000	0	-1000	0
Escherichia_coli_KTE69	0	0	-1000	-1000	-68.935405	-1000	0	0	0
Escherichia_coli_KTE7	0	0	-1000	-1000	-79.667275	-1000	0	0	0
Escherichia_coli_KTE70	0	0	-1000	-1000	-68.935405	-1000	0	0	0
Escherichia_coli_KTE71	0	0	-1000	-1000	-79.655259	-1000	0	0	0
Escherichia_coli_KTE72	0	0	-1000	-1000	-95.790668	-1000	0	0	0
Escherichia_coli_KTE73	0	0	-1000	-1000	-79.672791	-1000	0	0	0
Escherichia_coli_KTE74	0	0	-1000	-1000	-68.935405	-1000	0	0	0
Escherichia_coli_KTE75	0	0	-1000	-1000	-79.67295	-1000	0	0	0
Escherichia_coli_KTE76	0	0	-1000	-1000	-95.784	-1000	0	-1000	0
Escherichia_coli_KTE77	0	0	-1000	-1000	-79.661483	-1000	0	0	0
Escherichia_coli_KTE78	0	0	-1000	-1000	-79.672831	-1000	0	0	0

Escherichia_coli_KTE79	0	0	-1000	-1000	-79.67295	-1000	0	0	0
Escherichia_coli_KTE8	0	0	-1000	-1000	-95.790668	-1000	0	0	0
Escherichia_coli_KTE80	0	0	-1000	-1000	-80.98764	-1000	0	0	0
Escherichia_coli_KTE81	0	0	-1000	-1000	-79.661443	-1000	0	0	0
Escherichia_coli_KTE82	0	0	-1000	-1000	-80.990748	-1000	0	0	0
Escherichia_coli_KTE83	0	0	-1000	-1000	-80.990628	-1000	0	0	0
Escherichia_coli_KTE84	0	0	-1000	-1000	-95.800673	-1000	0	0	0
Escherichia_coli_KTE85	0	0	-1000	-1000	-79.678942	-1000	0	0	0
Escherichia_coli_KTE86	0	0	-1000	-1000	-95.790668	-1000	0	0	0
Escherichia_coli_KTE87	0	0	-1000	-1000	-95.790668	-1000	0	0	0
Escherichia_coli_KTE88	0	0	-1000	-1000	-95.790668	-1000	0	0	0
Escherichia_coli_KTE89	0	0	-1000	-1000	-95.790668	-1000	0	0	0
Escherichia_coli_KTE9	0	0	-1000	-1000	-79.673029	-1000	0	0	0
Escherichia_coli_KTE90	0	0	-1000	-1000	-79.080054	-1000	0	0	0
Escherichia_coli_KTE91	0	0	-1000	-1000	-79.079976	-1000	0	0	0
Escherichia_coli_KTE93	0	0	-1000	-1000	-95.790668	-1000	0	0	0
Escherichia_coli_KTE94	0	0	-1000	-1000	-95.800673	-1000	0	0	0
Escherichia_coli_KTE95	0	0	-1000	-1000	-81.028451	-1000	0	0	0
Escherichia_coli_KTE96	0	0	-1000	-1000	-46.201808	-1000	0	0	0
Escherichia_coli_KTE97	0	0	-1000	-1000	-95.790668	-1000	0	0	0
Escherichia_coli_KTE98	0	0	-1000	-1000	-79.678823	-1000	0	0	0
Escherichia_coli_KTE99	0	0	-1000	-1000	-95.790668	-1000	0	0	0
Escherichia_coli_LCT_EC106	0	0	-1000	-1000	-95.790668	-1000	0	0	0
Escherichia_coli_LCT_EC52	0	0	-1000	-1000	-95.790668	-1000	0	0	0
Escherichia_coli_LCT_EC59	0	0	-1000	-1000	-95.790668	-1000	0	0	0
Escherichia_coli_LF82	-1000	0	-1000	-1000	-96.335091	-1000	0	0	0
Escherichia_coli_LT_68	0	0	-1000	-1000	-53.878332	-1000	0	0	0
Escherichia_coli_M605	0	0	-1000	-1000	-95.790668	-1000	0	0	0
Escherichia_coli_M718	0	0	-1000	-1000	-78.837667	-1000	0	-1000	0
Escherichia_coli_M863	0	0	-1000	-1000	-79.634845	-1000	0	0	0
Escherichia_coli_MA6	0	0	-1000	-1000	-79.080128	-1000	0	0	0
Escherichia_coli_MP020940_1	0	0	-1000	-1000	-79.080054	-1000	0	0	0
Escherichia_coli_MP020980_1	0	0	-1000	-1000	-79.657846	-1000	0	0	0
Escherichia_coli_MP020980_2	0	0	-1000	-1000	-79.657846	-1000	0	0	0
Escherichia_coli_MP021017_1	0	0	-1000	-1000	-79.657846	-1000	0	-1000	0
Escherichia_coli_MP021017_10	0	0	-1000	-1000	-79.673227	-1000	0	-1000	0
Escherichia_coli_MP021017_11	0	0	-1000	-1000	-79.673227	-1000	0	-1000	0
Escherichia_coli_MP021017_12	0	0	-1000	-1000	-68.917428	-1000	0	-1000	0
Escherichia_coli_MP021017_2	0	0	-1000	-1000	-79.657886	-1000	0	-1000	0
Escherichia_coli_MP021017_3	0	0	-1000	-1000	-79.657886	-1000	0	-1000	0
Escherichia_coli_MP021017_4	0	0	-1000	-1000	-79.657886	-1000	0	-1000	0
Escherichia_coli_MP021017_5	0	0	-1000	-1000	-79.617642	-1000	0	-1000	0
Escherichia_coli_MP021017_6	0	0	-1000	-1000	-79.657886	-1000	0	-1000	0
Escherichia_coli_MP021017_9	0	0	-1000	-1000	-68.925792	-1000	0	-1000	0
Escherichia_coli_MP021552_11	0	0	-1000	-1000	-79.673267	-1000	0	0	0
Escherichia_coli_MP021552_12	0	0	-1000	-1000	-79.673302	-1000	0	0	0
Escherichia_coli_MP021552_7	0	0	-1000	-1000	-79.667513	-1000	0	0	0
Escherichia_coli_MP021552_8	0	0	-1000	-1000	-79.673267	-1000	0	0	0
Escherichia_coli_MP021561_2	0	0	-1000	-1000	-79.657925	-1000	0	0	0
Escherichia_coli_MP021561_3	0	0	-1000	-1000	-79.068757	-1000	0	0	0
Escherichia_coli_MP021566_1	0	0	-1000	-1000	-79.657846	-1000	0	0	0
Escherichia_coli_MS_107_1	0	0	-1000	-1000	-79.080015	-1000	0	-1000	0
Escherichia_coli_MS_115_1	0	0	-1000	-1000	-79.094033	-1000	0	0	0
Escherichia_coli_MS_116_1	0	0	-1000	-1000	-79.65585	-1000	0	0	0
Escherichia_coli_MS_119_7	0	0	-1000	-1000	-79.079976	-1000	0	-1000	0
Escherichia_coli_MS_124_1	0	0	-1000	-1000	-79.766252	-1000	0	0	0
Escherichia_coli_MS_145_7	0	0	-1000	-1000	-79.079937	-1000	0	0	0
Escherichia_coli_MS_146_1	0	0	-1000	-1000	-79.661602	-1000	0	0	0
Escherichia_coli_MS_182_1	0	0	-1000	-1000	-79.657806	-1000	0	0	0
Escherichia_coli_MS_185_1	0	0	-1000	-1000	-95.790668	-1000	0	0	0
Escherichia_coli_MS_187_1	0	0	-1000	-1000	-79.678784	-1000	0	0	0
Escherichia_coli_MS_196_1	0	0	-1000	-1000	-79.661522	-1000	0	0	0
Escherichia_coli_MS_198_1	0	0	-1000	-1000	-80.98768	-1000	0	0	0
Escherichia_coli_MS_200_1	0	0	-1000	-1000	-95.790668	-1000	0	0	0
Escherichia_coli_MS_21_1	0	0	-1000	-1000	-79.678942	-1000	0	0	0
Escherichia_coli_MS_45_1	0	0	-1000	-1000	-95.790668	-1000	0	0	0
Escherichia_coli_MS_69_1	0	0	-1000	-1000	-78.642187	-1000	0	0	0
Escherichia_coli_MS_78_1	0	0	-1000	-1000	-79.657727	-1000	0	0	0
Escherichia_coli_MS_79_10	0	0	-1000	-1000	-52.519641	-1000	0	-1000	0
Escherichia_coli_MS_84_1	0	0	-1000	-1000	-79.673188	-1000	0	0	0
Escherichia_coli_MS_85_1	0	0	-1000	-1000	-79.673188	-1000	0	0	0
Escherichia_coli_Mt1B1	0	0	-1000	-1000	-83.171468	-1000	0	0	0
Escherichia_coli_N1	0	0	-1000	-1000	-78.636581	-1000	0	0	0
Escherichia_coli_NA114	-1000	0	-1000	-1000	-79.679822	-1000	0	0	0
Escherichia_coli_NC101	0	0	-1000	-1000	-95.800673	-1000	0	0	0
Escherichia_coli_NCCP15647	0	0	-1000	-1000	-79.657846	-1000	0	0	0
Escherichia_coli_NCCP15657	0	0	-1000	-1000	-79.657846	-1000	0	0	0
Escherichia_coli_NCCP15658	0	0	-1000	-1000	-79.080093	-1000	0	0	0
Escherichia_coli_NE037	0	0	-1000	-1000	-79.080128	-1000	0	0	0
Escherichia_coli_NE098	0	0	-1000	-1000	-79.080128	-1000	0	0	0
Escherichia_coli_NE1487	0	0	-1000	-1000	-79.080128	-1000	0	0	0
Escherichia_coli_Nissle_1917	0	0	-1000	-1000	-95.790668	-1000	0	0	0
Escherichia_coli_O08	0	0	-1000	-1000	-79.657886	-1000	0	0	0
Escherichia_coli_O103H2_str_12009	0	0	-1000	-1000	-85.289315	-1000	0	0	0
Escherichia_coli_O103H2_str_CVM9450	0	0	-1000	-1000	-79.080015	-1000	0	0	0
Escherichia_coli_O103H25_str_CVM9340	0	0	-1000	-1000	-79.080093	-1000	0	0	0
Escherichia_coli_O103H25_str_NIPH_11060424	0	0	-1000	-1000	-79.080093	-1000	0	0	0
Escherichia_coli_O104_H4_str_01_09591	0	0	-1000	-1000	-68.681365	-1000	0	0	0
Escherichia_coli_O104_H4_str_04_8351	0	0	-1000	-1000	-79.091276	-1000	0	0	0
Escherichia_coli_O104_H4_str_09_7901	0	0	-1000	-1000	-79.091315	-1000	0	0	0
Escherichia_coli_O104_H4_str_11_02030	0	0	-1000	-1000	-79.091354	-1000	0	0	0

Escherichia_coli_O104_H4_str_11_02033_1	0	0	-1000	-1000	-79.091315	-1000	0	0	0
Escherichia_coli_O104_H4_str_11_02092	0	0	-1000	-1000	-79.091354	-1000	0	0	0
Escherichia_coli_O104_H4_str_11_02093	0	0	-1000	-1000	-79.091354	-1000	0	0	0
Escherichia_coli_O104_H4_str_11_02281	0	0	-1000	-1000	-79.091354	-1000	0	0	0
Escherichia_coli_O104_H4_str_11_02318	0	0	-1000	-1000	-79.091354	-1000	0	0	0
Escherichia_coli_O104_H4_str_11_02913	0	0	-1000	-1000	-79.091354	-1000	0	0	0
Escherichia_coli_O104_H4_str_11_03439	0	0	-1000	-1000	-79.091354	-1000	0	0	0
Escherichia_coli_O104_H4_str_11_03943	0	0	-1000	-1000	-79.091354	-1000	0	0	0
Escherichia_coli_O104_H4_str_11_04080	0	0	-1000	-1000	-79.091354	-1000	0	0	0
Escherichia_coli_O104_H4_str_11_3677	0	0	-1000	-1000	-79.091276	-1000	0	0	0
Escherichia_coli_O104_H4_str_11_4404	0	0	-1000	-1000	-79.091237	-1000	0	0	0
Escherichia_coli_O104_H4_str_11_4522	0	0	-1000	-1000	-79.091237	-1000	0	0	0
Escherichia_coli_O104_H4_str_11_4623	0	0	-1000	-1000	-79.091276	-1000	0	0	0
Escherichia_coli_O104_H4_str_11_4632_C1	0	0	-1000	-1000	-79.091237	-1000	0	0	0
Escherichia_coli_O104_H4_str_11_4632_C2	0	0	-1000	-1000	-79.091198	-1000	0	0	0
Escherichia_coli_O104_H4_str_11_4632_C3	0	0	-1000	-1000	-79.091198	-1000	0	0	0
Escherichia_coli_O104_H4_str_11_4632_C4	0	0	-1000	-1000	-79.091237	-1000	0	0	0
Escherichia_coli_O104_H4_str_11_4632_C5	0	0	-1000	-1000	-79.091276	-1000	0	0	0
Escherichia_coli_O104_H4_str_2009EL_2050	-1000	0	-1000	-1000	-79.091354	-1000	0	0	0
Escherichia_coli_O104_H4_str_2009EL_2071	-1000	0	-1000	-1000	-79.091315	-1000	0	0	0
Escherichia_coli_O104_H4_str_2011C_3493	-1000	0	-1000	-1000	-68.770998	-1000	0	0	0
Escherichia_coli_O104_H4_str_C227_11	0	0	-1000	-1000	-79.079976	-1000	0	0	0
Escherichia_coli_O104_H4_str_C236_11	0	0	-1000	-1000	-79.091315	-1000	0	0	0
Escherichia_coli_O104_H4_str_Ec11_4984	0	0	-1000	-1000	-79.091198	-1000	0	0	0
Escherichia_coli_O104_H4_str_Ec11_4986	0	0	-1000	-1000	-79.091237	-1000	0	0	0
Escherichia_coli_O104_H4_str_Ec11_4987	0	0	-1000	-1000	-79.091159	-1000	0	0	0
Escherichia_coli_O104_H4_str_Ec11_4988	0	0	-1000	-1000	-79.091198	-1000	0	0	0
Escherichia_coli_O104_H4_str_Ec11_5603	0	0	-1000	-1000	-79.091198	-1000	0	0	0
Escherichia_coli_O104_H4_str_Ec11_5604	0	0	-1000	-1000	-79.091237	-1000	0	0	0
Escherichia_coli_O104_H4_str_Ec11_6006	0	0	-1000	-1000	-79.091159	-1000	0	0	0
Escherichia_coli_O104_H4_str_Ec11_9450	0	0	-1000	-1000	-79.09112	-1000	0	0	0
Escherichia_coli_O104_H4_str_Ec11_9941	0	0	-1000	-1000	-79.091237	-1000	0	0	0
Escherichia_coli_O104_H4_str_Ec11_9990	0	0	-1000	-1000	-79.091198	-1000	0	0	0
Escherichia_coli_O104_H4_str_Ec12_0465	0	0	-1000	-1000	-79.091237	-1000	0	0	0
Escherichia_coli_O104_H4_str_Ec12_0466	0	0	-1000	-1000	-79.091159	-1000	0	0	0
Escherichia_coli_O104_H4_str_GOS1	0	0	-1000	-1000	-79.091354	-1000	0	0	0
Escherichia_coli_O104_H4_str_GOS2	0	0	-1000	-1000	-79.091354	-1000	0	0	0
Escherichia_coli_O104_H4_str_H112180280	0	0	-1000	-1000	-79.091315	-1000	0	0	0
Escherichia_coli_O104_H4_str_H112180282	0	0	-1000	-1000	-79.091354	-1000	0	0	0
Escherichia_coli_O104_H4_str_H112180283	0	0	-1000	-1000	-62.887443	-1000	0	0	0
Escherichia_coli_O104_H4_str_LB226692	0	0	-1000	-1000	-62.887435	-1000	0	0	0
Escherichia_coli_O104_H4_str_ON2010	0	0	-1000	-1000	-79.091315	-1000	0	0	0
Escherichia_coli_O104_H4_str_ON2011	0	0	-1000	-1000	-79.090734	-1000	0	0	0
Escherichia_coli_O104_H4_str_TY_2482	0	0	-1000	-1000	-62.887443	-1000	0	0	0
Escherichia_coli_O10K5LH4_str_ATCC_23506	0	0	-1000	-1000	-79.655929	-1000	0	0	0
Escherichia_coli_O111_H_str_11128	0	0	-1000	-1000	-62.835165	-1000	0	0	0
Escherichia_coli_O111_H11_str_CFSAN001630	0	0	-1000	-1000	-59.809967	-1000	0	0	0
Escherichia_coli_O111_H11_str_CVM9534	0	0	-1000	-1000	-62.887435	-1000	0	0	0
Escherichia_coli_O111_H11_str_CVM9553	0	0	-1000	-1000	-61.146294	-1000	0	0	0
Escherichia_coli_O111_H8_str_CFSAN001632	0	0	-1000	-1000	-62.887443	-1000	0	0	0
Escherichia_coli_O111_H8_str_CVM9570	0	0	-1000	-1000	-62.887435	-1000	0	0	0
Escherichia_coli_O111_H8_str_CVM9574	0	0	-1000	-1000	-62.887443	-1000	0	0	0
Escherichia_coli_O111_H8_str_CVM9602	0	0	-1000	-1000	-55.062626	-1000	0	0	0
Escherichia_coli_O111_H8_str_CVM9634	0	0	-1000	-1000	-55.062626	-1000	0	0	0
Escherichia_coli_O113_H21_str_CL_3	0	0	-1000	-1000	-55.303236	-1000	0	0	0
Escherichia_coli_O121_H19_str_MT_2	0	0	-1000	-1000	-53.86275	-1000	0	0	0
Escherichia_coli_O127_H27_str_C43_90	0	0	-1000	-1000	-55.283793	-1000	0	0	0
Escherichia_coli_O127_H6_str_E2348_69	-1000	0	-1000	-1000	-55.432242	-1000	0	0	0
Escherichia_coli_O145_H28_str_4865_96	0	0	-1000	-1000	-53.629856	-1000	0	0	0
Escherichia_coli_O157_H_str_493_89	0	0	-1000	-1000	-53.634454	-1000	0	0	0
Escherichia_coli_O157_H_str_H_2687	0	0	-1000	-1000	-59.810059	-1000	0	0	0
Escherichia_coli_O157_H7_EDL933	0	0	-1000	-1000	-68.427385	-1000	0	0	0
Escherichia_coli_O157_H7_str_1044	0	0	-1000	-1000	-79.131054	-1000	0	0	0
Escherichia_coli_O157_H7_str_1125	0	0	-1000	-1000	-53.52452	-1000	0	0	0
Escherichia_coli_O157_H7_str_2149	0	0	-1000	-1000	-47.426316	-1000	0	0	0
Escherichia_coli_O157_H7_str_EC1212	0	0	-1000	-1000	-53.616197	-1000	0	0	0
Escherichia_coli_O157_H7_str_EC4009	0	0	-1000	-1000	-53.470279	-1000	0	0	0
Escherichia_coli_O157_H7_str_EC4042	0	0	-1000	-1000	-53.607089	-1000	0	0	0
Escherichia_coli_O157_H7_str_EC4045	0	0	-1000	-1000	-53.616228	-1000	0	0	0
Escherichia_coli_O157_H7_str_EC4084	0	0	-1000	-1000	-53.452139	-1000	0	0	0
Escherichia_coli_O157_H7_str_EC4115	-1000	0	-1000	-1000	-53.840798	-1000	0	0	0
Escherichia_coli_O157_H7_str_EC4191	0	0	-1000	-1000	-53.616135	-1000	0	0	0
Escherichia_coli_O157_H7_str_EC536	0	0	-1000	-1000	-53.616135	-1000	0	0	0
Escherichia_coli_O157_H7_str_FRIK2000	0	0	-1000	-1000	-53.616135	-1000	0	0	0
Escherichia_coli_O157_H7_str_FRIK966	0	0	-1000	-1000	-53.616135	-1000	0	0	0
Escherichia_coli_O157_H7_str_G5101	0	0	-1000	-1000	-53.616259	-1000	0	0	0
Escherichia_coli_O157_H7_str_H093800014	0	0	-1000	-1000	-53.616166	-1000	0	0	0
Escherichia_coli_O157_H7_str_LSU_61	0	0	-1000	-1000	-53.616259	-1000	0	0	0
Escherichia_coli_O157_H7_str_Sakai	-1000	0	-1000	-1000	-66.1176	-1000	0	-1000	0
Escherichia_coli_O157_H7_str_TW14359	-1000	0	-1000	-1000	-53.616228	-1000	0	0	0
Escherichia_coli_O157_H7_str_TW14588	0	0	-1000	-1000	-53.616228	-1000	0	0	0
Escherichia_coli_O157_str_NCCP15738	0	0	-1000	-1000	-55.303236	-1000	0	-1000	0
Escherichia_coli_O157_str_NCCP15739	0	0	-1000	-1000	-53.616166	-1000	0	0	0
Escherichia_coli_O25b_H4_ST131_str_EC958	0	0	-1000	-1000	-55.25166	-1000	0	0	0
Escherichia_coli_O26_H11_str_11368	-1000	0	-1000	-1000	-55.001884	-1000	0	0	0
Escherichia_coli_O26_H11_str_CFSAN001629	0	0	-1000	-1000	-55.001884	-1000	0	0	0
Escherichia_coli_O26_H11_str_CVM10021	0	0	-1000	-1000	-55.001884	-1000	0	0	0
Escherichia_coli_O26_H11_str_CVM10026	0	0	-1000	-1000	-55.001884	-1000	0	0	0
Escherichia_coli_O26_H11_str_CVM10030	0	0	-1000	-1000	-55.001884	-1000	0	0	0
Escherichia_coli_O26_H11_str_CVM10224	0	0	-1000	-1000	-55.001884	-1000	0	0	0
Escherichia_coli_O26_H11_str_CVM9942	0	0	-1000	-1000	-55.001884	-1000	0	0	0

Escherichia_coli_O26_H11_str_CVM9952	0	0	-1000	-1000	-55.001884	-1000	0	0	0
Escherichia_coli_O32_H37_str_P4	0	0	-1000	-1000	-55.283793	-1000	0	0	0
Escherichia_coli_O45_H2_str_03_EN_705	0	0	-1000	-1000	-55.062626	-1000	0	0	0
Escherichia_coli_O5_K4_L_H4_str_ATCC_23502	0	0	-1000	-1000	-53.872039	-1000	0	0	0
Escherichia_coli_O55_H7_str_3256_97	0	0	-1000	-1000	-53.616259	-1000	0	0	0
Escherichia_coli_O55_H7_str_CB9615	-1000	0	-1000	-1000	-53.840829	-1000	0	0	0
Escherichia_coli_O55_H7_str_USDA_5905	0	0	-1000	-1000	-53.616166	-1000	0	0	0
Escherichia_coli_O7_K1_str_CE10	0	0	-1000	-1000	-72.459402	-1000	0	0	0
Escherichia_coli_O83_H1_str_NRG_857C	0	0	-1000	-1000	-72.537501	-1000	0	0	0
Escherichia_coli_O91	0	0	-1000	-1000	-55.062626	-1000	0	0	0
Escherichia_coli_O91_H21_str_B2F1	0	0	-1000	-1000	-55.303236	-1000	0	0	0
Escherichia_coli_OK1180	0	0	-1000	-1000	-55.062626	-1000	0	0	0
Escherichia_coli_ONT_H33_str_C48_93	0	0	-1000	-1000	-55.269219	-1000	0	0	0
Escherichia_coli_P0298942_1	0	0	-1000	-1000	-53.775672	-1000	0	0	0
Escherichia_coli_P0298942_10	0	0	-1000	-1000	-53.77564	-1000	0	0	0
Escherichia_coli_P0298942_11	0	0	-1000	-1000	-53.77564	-1000	0	0	0
Escherichia_coli_P0298942_12	0	0	-1000	-1000	-53.77564	-1000	0	0	0
Escherichia_coli_P0298942_14	0	0	-1000	-1000	-53.77564	-1000	0	0	0
Escherichia_coli_P0298942_15	0	0	-1000	-1000	-53.77564	-1000	0	0	0
Escherichia_coli_P0298942_3	0	0	-1000	-1000	-53.559069	-1000	0	0	0
Escherichia_coli_P0298942_4	0	0	-1000	-1000	-53.77564	-1000	0	0	0
Escherichia_coli_P0298942_6	0	0	-1000	-1000	-53.77564	-1000	0	0	0
Escherichia_coli_P0298942_7	0	0	-1000	-1000	-53.77564	-1000	0	0	0
Escherichia_coli_P0299438_10	0	0	-1000	-1000	-53.766447	-1000	0	0	0
Escherichia_coli_P0299438_11	0	0	-1000	-1000	-53.548143	-1000	0	0	0
Escherichia_coli_P0299438_2	0	0	-1000	-1000	-53.844462	-1000	0	-1000	0
Escherichia_coli_P0299438_3	0	0	-1000	-1000	-53.548143	-1000	0	0	0
Escherichia_coli_P0299438_4	0	0	-1000	-1000	-53.061929	-1000	0	0	0
Escherichia_coli_P0299438_5	0	0	-1000	-1000	-52.965973	-1000	0	0	0
Escherichia_coli_P0299438_6	0	0	-1000	-1000	-53.548143	-1000	0	0	0
Escherichia_coli_P0299438_7	0	0	-1000	-1000	-53.548143	-1000	0	0	0
Escherichia_coli_P0299438_8	0	0	-1000	-1000	-53.548143	-1000	0	0	0
Escherichia_coli_P0299438_9	0	0	-1000	-1000	-53.844423	-1000	0	-1000	0
Escherichia_coli_P0299483_1	0	0	-1000	-1000	-54.913499	-1000	0	0	0
Escherichia_coli_P0299483_2	0	0	-1000	-1000	-55.303236	-1000	0	0	0
Escherichia_coli_P0299483_3	0	0	-1000	-1000	-55.303236	-1000	0	0	0
Escherichia_coli_P02997067_6	0	0	-1000	-1000	-55.001884	-1000	0	0	0
Escherichia_coli_P0299917_1	0	0	-1000	-1000	-55.001884	-1000	0	0	0
Escherichia_coli_P0299917_10	0	0	-1000	-1000	-55.062626	-1000	0	0	0
Escherichia_coli_P0299917_2	0	0	-1000	-1000	-55.062626	-1000	0	0	0
Escherichia_coli_P0299917_4	0	0	-1000	-1000	-55.062626	-1000	0	0	0
Escherichia_coli_P0299917_5	0	0	-1000	-1000	-54.754211	-1000	0	0	0
Escherichia_coli_P0299917_6	0	0	-1000	-1000	-55.062626	-1000	0	0	0
Escherichia_coli_P0299917_7	0	0	-1000	-1000	-55.062626	-1000	0	0	0
Escherichia_coli_P0299917_8	0	0	-1000	-1000	-55.062626	-1000	0	0	0
Escherichia_coli_P0299917_9	0	0	-1000	-1000	-55.062626	-1000	0	0	0
Escherichia_coli_P0301867_1	0	0	-1000	-1000	-53.775726	-1000	0	0	0
Escherichia_coli_P0301867_11	0	0	-1000	-1000	-53.775703	-1000	0	0	0
Escherichia_coli_P0301867_13	0	0	-1000	-1000	-53.775703	-1000	0	0	0
Escherichia_coli_P0301867_2	0	0	-1000	-1000	-53.775703	-1000	0	0	0
Escherichia_coli_P0301867_3	0	0	-1000	-1000	-53.775703	-1000	0	0	0
Escherichia_coli_P0301867_4	0	0	-1000	-1000	-53.775703	-1000	0	0	0
Escherichia_coli_P0301867_5	0	0	-1000	-1000	-53.775703	-1000	0	0	0
Escherichia_coli_P0301867_7	0	0	-1000	-1000	-53.775703	-1000	0	0	0
Escherichia_coli_P0301904_3	0	0	-1000	-1000	-54.913499	-1000	0	0	0
Escherichia_coli_P0302293_10	0	0	-1000	-1000	-55.001884	-1000	0	0	0
Escherichia_coli_P0302293_2	0	0	-1000	-1000	-55.001884	-1000	0	0	0
Escherichia_coli_P0302293_3	0	0	-1000	-1000	-55.001884	-1000	0	0	0
Escherichia_coli_P0302293_4	0	0	-1000	-1000	-55.001884	-1000	0	0	0
Escherichia_coli_P0302293_6	0	0	-1000	-1000	-55.001884	-1000	0	0	0
Escherichia_coli_P0302293_7	0	0	-1000	-1000	-55.001884	-1000	0	0	0
Escherichia_coli_P0302293_8	0	0	-1000	-1000	-55.001884	-1000	0	0	0
Escherichia_coli_P0302293_9	0	0	-1000	-1000	-55.001884	-1000	0	0	0
Escherichia_coli_P0302308_1	0	0	-1000	-1000	-53.548174	-1000	0	0	0
Escherichia_coli_P0302308_10	0	0	-1000	-1000	-53.548174	-1000	0	0	0
Escherichia_coli_P0302308_11	0	0	-1000	-1000	-53.548174	-1000	0	0	0
Escherichia_coli_P0302308_12	0	0	-1000	-1000	-53.548174	-1000	0	0	0
Escherichia_coli_P0302308_13	0	0	-1000	-1000	-53.548234	-1000	0	0	0
Escherichia_coli_P0302308_14	0	0	-1000	-1000	-53.548174	-1000	0	0	0
Escherichia_coli_P0302308_2	0	0	-1000	-1000	-52.91599	-1000	0	0	0
Escherichia_coli_P0302308_3	0	0	-1000	-1000	-53.548174	-1000	0	0	0
Escherichia_coli_P0302308_4	0	0	-1000	-1000	-53.548174	-1000	0	0	0
Escherichia_coli_P0302308_5	0	0	-1000	-1000	-53.548174	-1000	0	0	0
Escherichia_coli_P0304777_1	0	0	-1000	-1000	-55.303229	-1000	0	0	0
Escherichia_coli_P0304777_10	0	0	-1000	-1000	-55.225544	-1000	0	0	0
Escherichia_coli_P0304777_11	0	0	-1000	-1000	-55.303236	-1000	0	0	0
Escherichia_coli_P0304777_12	0	0	-1000	-1000	-55.303236	-1000	0	0	0
Escherichia_coli_P0304777_13	0	0	-1000	-1000	-55.303236	-1000	0	0	0
Escherichia_coli_P0304777_14	0	0	-1000	-1000	-55.303236	-1000	0	0	0
Escherichia_coli_P0304777_15	0	0	-1000	-1000	-55.303236	-1000	0	0	0
Escherichia_coli_P0304777_2	0	0	-1000	-1000	-55.303236	-1000	0	0	0
Escherichia_coli_P0304777_3	0	0	-1000	-1000	-55.303236	-1000	0	0	0
Escherichia_coli_P0304777_4	0	0	-1000	-1000	-55.303236	-1000	0	0	0
Escherichia_coli_P0304777_7	0	0	-1000	-1000	-55.293513	-1000	0	0	0
Escherichia_coli_P0304777_8	0	0	-1000	-1000	-55.303236	-1000	0	0	0
Escherichia_coli_P0304777_9	0	0	-1000	-1000	-55.303236	-1000	0	0	0
Escherichia_coli_P0304799_3	0	0	-1000	-1000	-55.243318	-1000	0	0	0
Escherichia_coli_P0304816_1	0	0	-1000	-1000	-54.982652	-1000	0	0	0
Escherichia_coli_P0304816_11	0	0	-1000	-1000	-54.982652	-1000	0	0	0
Escherichia_coli_P0304816_12	0	0	-1000	-1000	-54.982652	-1000	0	0	0
Escherichia_coli_P0304816_13	0	0	-1000	-1000	-54.982652	-1000	0	0	0

Escherichia_coli_P0304816_14	0	0	-1000	-1000	-54.982652	-1000	0	0	0
Escherichia_coli_P0304816_15	0	0	-1000	-1000	-54.982652	-1000	0	0	0
Escherichia_coli_P0304816_2	0	0	-1000	-1000	-54.982652	-1000	0	0	0
Escherichia_coli_P0304816_3	0	0	-1000	-1000	-55.001884	-1000	0	0	0
Escherichia_coli_P0304816_4	0	0	-1000	-1000	-54.905857	-1000	0	0	0
Escherichia_coli_P0304816_5	0	0	-1000	-1000	-54.982652	-1000	0	0	0
Escherichia_coli_P0304816_6	0	0	-1000	-1000	-45.834903	-1000	0	0	0
Escherichia_coli_P0304816_7	0	0	-1000	-1000	-55.001884	-1000	0	0	0
Escherichia_coli_P0304816_8	0	0	-1000	-1000	-55.001884	-1000	0	0	0
Escherichia_coli_P0304816_9	0	0	-1000	-1000	-54.982652	-1000	0	0	0
Escherichia_coli_P0305260_1	0	0	-1000	-1000	-53.844462	-1000	0	-1000	0
Escherichia_coli_P0305260_10	0	0	-1000	-1000	-53.88594	-1000	0	-1000	0
Escherichia_coli_P0305260_11	0	0	-1000	-1000	-53.844423	-1000	0	-1000	0
Escherichia_coli_P0305260_12	0	0	-1000	-1000	-53.844423	-1000	0	-1000	0
Escherichia_coli_P0305260_13	0	0	-1000	-1000	-53.862906	-1000	0	-1000	0
Escherichia_coli_P0305260_15	0	0	-1000	-1000	-53.862867	-1000	0	-1000	0
Escherichia_coli_P0305260_2	0	0	-1000	-1000	-55.283785	-1000	0	-1000	0
Escherichia_coli_P0305260_3	0	0	-1000	-1000	-53.844423	-1000	0	-1000	0
Escherichia_coli_P0305260_4	0	0	-1000	-1000	-53.844423	-1000	0	-1000	0
Escherichia_coli_P0305260_5	0	0	-1000	-1000	-53.844423	-1000	0	-1000	0
Escherichia_coli_P0305260_6	0	0	-1000	-1000	-53.862906	-1000	0	-1000	0
Escherichia_coli_P0305260_7	0	0	-1000	-1000	-53.862867	-1000	0	-1000	0
Escherichia_coli_P0305260_8	0	0	-1000	-1000	-53.465825	-1000	0	-1000	0
Escherichia_coli_P0305260_9	0	0	-1000	-1000	-53.844423	-1000	0	-1000	0
Escherichia_coli_p0305293_1	0	0	-1000	-1000	-53.548143	-1000	0	0	0
Escherichia_coli_p0305293_10	0	0	-1000	-1000	-53.548081	-1000	0	0	0
Escherichia_coli_p0305293_11	0	0	-1000	-1000	-53.548143	-1000	0	0	0
Escherichia_coli_p0305293_12	0	0	-1000	-1000	-53.548081	-1000	0	0	0
Escherichia_coli_p0305293_14	0	0	-1000	-1000	-53.548081	-1000	0	0	0
Escherichia_coli_p0305293_15	0	0	-1000	-1000	-53.548081	-1000	0	0	0
Escherichia_coli_p0305293_2	0	0	-1000	-1000	-53.548081	-1000	0	0	0
Escherichia_coli_p0305293_3	0	0	-1000	-1000	-53.548081	-1000	0	0	0
Escherichia_coli_p0305293_4	0	0	-1000	-1000	-53.548112	-1000	0	0	0
Escherichia_coli_p0305293_5	0	0	-1000	-1000	-53.402625	-1000	0	0	0
Escherichia_coli_p0305293_6	0	0	-1000	-1000	-53.548197	-1000	0	0	0
Escherichia_coli_p0305293_7	0	0	-1000	-1000	-52.966126	-1000	0	0	0
Escherichia_coli_p0305293_8	0	0	-1000	-1000	-53.548081	-1000	0	0	0
Escherichia_coli_p0305293_9	0	0	-1000	-1000	-53.548174	-1000	0	0	0
Escherichia_coli_P12b	-1000	0	-1000	-1000	-63.362269	-1000	0	0	0
Escherichia_coli_PA10	0	0	-1000	-1000	-53.616197	-1000	0	0	0
Escherichia_coli_PA11	0	0	-1000	-1000	-53.616166	-1000	0	0	0
Escherichia_coli_PA13	0	0	-1000	-1000	-53.616135	-1000	0	0	0
Escherichia_coli_PA14	0	0	-1000	-1000	-53.616166	-1000	0	0	0
Escherichia_coli_PA15	0	0	-1000	-1000	-53.616166	-1000	0	0	0
Escherichia_coli_PA19	0	0	-1000	-1000	-53.616135	-1000	0	0	0
Escherichia_coli_PA2	0	0	-1000	-1000	-53.616166	-1000	0	0	0
Escherichia_coli_PA22	0	0	-1000	-1000	-53.616166	-1000	0	0	0
Escherichia_coli_PA23	0	0	-1000	-1000	-53.616197	-1000	0	0	0
Escherichia_coli_PA24	0	0	-1000	-1000	-53.616197	-1000	0	0	0
Escherichia_coli_PA25	0	0	-1000	-1000	-53.616166	-1000	0	0	0
Escherichia_coli_PA28	0	0	-1000	-1000	-53.616166	-1000	0	0	0
Escherichia_coli_PA3	0	0	-1000	-1000	-53.616197	-1000	0	0	0
Escherichia_coli_PA31	0	0	-1000	-1000	-53.616197	-1000	0	0	0
Escherichia_coli_PA32	0	0	-1000	-1000	-53.616166	-1000	0	0	0
Escherichia_coli_PA33	0	0	-1000	-1000	-53.616197	-1000	0	0	0
Escherichia_coli_PA34	0	0	-1000	-1000	-53.616166	-1000	0	0	0
Escherichia_coli_PA35	0	0	-1000	-1000	-53.616097	-1000	0	0	0
Escherichia_coli_PA38	0	0	-1000	-1000	-53.616228	-1000	0	0	0
Escherichia_coli_PA4	0	0	-1000	-1000	-53.616166	-1000	0	0	0
Escherichia_coli_PA40	0	0	-1000	-1000	-53.616135	-1000	0	0	0
Escherichia_coli_PA41	0	0	-1000	-1000	-53.616166	-1000	0	0	0
Escherichia_coli_PA42	0	0	-1000	-1000	-53.616166	-1000	0	0	0
Escherichia_coli_PA45	0	0	-1000	-1000	-53.616166	-1000	0	0	0
Escherichia_coli_PA47	0	0	-1000	-1000	-53.616166	-1000	0	0	0
Escherichia_coli_PA48	0	0	-1000	-1000	-53.616135	-1000	0	0	0
Escherichia_coli_PA5	0	0	-1000	-1000	-53.616197	-1000	0	0	0
Escherichia_coli_PA7	0	0	-1000	-1000	-53.616197	-1000	0	0	0
Escherichia_coli_PA8	0	0	-1000	-1000	-53.616166	-1000	0	0	0
Escherichia_coli_PA9	0	0	-1000	-1000	-53.616166	-1000	0	0	0
Escherichia_coli_PCN033	0	0	-1000	-1000	-53.403618	-1000	0	0	0
Escherichia_coli_RN587_1	0	0	-1000	-1000	-72.424743	-1000	0	0	0
Escherichia_coli_S17	0	0	-1000	-1000	-55.283785	-1000	0	0	0
Escherichia_coli_S88	-1000	0	-1000	-1000	-55.412708	-1000	0	0	0
Escherichia_coli_SCI_07	0	0	-1000	-1000	-72.518684	-1000	0	0	0
Escherichia_coli_SE11	-1000	0	-1000	-1000	-200	-1000	0	0	0
Escherichia_coli_SE15	-1000	0	-1000	-1000	-72.459402	-1000	0	0	0
Escherichia_coli_SEPT362	0	0	-1000	-1000	-55.495312	-1000	0	-1000	0
Escherichia_coli_SMS_3_5	-1000	0	-1000	-1000	-55.61538	-1000	0	-1000	0
Escherichia_coli_STEC_7v	0	0	-1000	-1000	-55.241962	-1000	0	0	0
Escherichia_coli_STEC_94C	0	0	-1000	-1000	-55.001884	-1000	0	0	0
Escherichia_coli_STEC_B2F1	0	0	-1000	-1000	-55.303236	-1000	0	0	0
Escherichia_coli_STEC_C165_02	0	0	-1000	-1000	-53.611602	-1000	0	0	0
Escherichia_coli_STEC_DG131_3	0	0	-1000	-1000	-53.862813	-1000	0	0	0
Escherichia_coli_STEC_EH250	0	0	-1000	-1000	-55.038436	-1000	0	0	0
Escherichia_coli_STEC_H_1_8	0	0	-1000	-1000	-55.001884	-1000	0	0	0
Escherichia_coli_STEC_MHI813	0	0	-1000	-1000	-53.136851	-1000	0	0	0
Escherichia_coli_STEC_O31	0	0	-1000	-1000	-55.303229	-1000	0	0	0
Escherichia_coli_STEC_S1191	0	0	-1000	-1000	-55.222562	-1000	0	0	0
Escherichia_coli_str_clone_D_i14	0	0	-1000	-1000	-69.807913	-1000	0	0	0
Escherichia_coli_str_clone_D_i2	0	0	-1000	-1000	-69.807913	-1000	0	0	0
Escherichia_coli_str_K_12_substr_DH10B	0	0	-1000	-1000	-55.222555	-1000	0	0	0

Escherichia_coli_str_K_12_substr_MDS42	0	0	-1000	-1000	-55.283793	-1000	0	0	0
Escherichia_coli_str_K_12_substr_MG1655	0	0	-1000	-1000	-64.350256	-1000	0	0	0
Escherichia_coli_str_K_12_substr_MG1655star	0	0	-1000	-1000	-55.222562	-1000	0	0	0
Escherichia_coli_str_K_12_substr_W3110	0	0	-1000	-1000	-55.222555	-1000	0	0	0
Escherichia_coli_SWW33	0	0	-1000	-1000	-72.478188	-1000	0	0	0
Escherichia_coli_TA007	0	0	-1000	-1000	-53.872132	-1000	0	-1000	0
Escherichia_coli_TA124	0	0	-1000	-1000	-55.241962	-1000	0	0	0
Escherichia_coli_TA143	0	0	-1000	-1000	-53.096616	-1000	0	0	0
Escherichia_coli_TA206	0	0	-1000	-1000	-72.353269	-1000	0	0	0
Escherichia_coli_TA271	0	0	-1000	-1000	-54.60402	-1000	0	0	0
Escherichia_coli_TA280	0	0	-1000	-1000	-53.629979	-1000	0	0	0
Escherichia_coli_ThroopD	0	0	-1000	-1000	-55.303236	-1000	0	0	0
Escherichia_coli_TT12B	0	0	-1000	-1000	-53.616197	-1000	0	0	0
Escherichia_coli_TW00353	0	0	-1000	-1000	-55.043351	-1000	0	0	0
Escherichia_coli_TW06591	0	0	-1000	-1000	-53.634454	-1000	0	0	0
Escherichia_coli_TW07793	0	0	-1000	-1000	-72.537501	-1000	0	0	0
Escherichia_coli_TW07945	0	0	-1000	-1000	-53.616166	-1000	0	0	0
Escherichia_coli_TW09098	0	0	-1000	-1000	-53.616197	-1000	0	0	0
Escherichia_coli_TW09109	0	0	-1000	-1000	-53.616166	-1000	0	0	0
Escherichia_coli_TW10119	0	0	-1000	-1000	-53.616166	-1000	0	0	0
Escherichia_coli_TW10246	0	0	-1000	-1000	-53.616166	-1000	0	0	0
Escherichia_coli_TW10509	0	0	-1000	-1000	-55.303236	-1000	0	0	0
Escherichia_coli_TW10598	0	0	-1000	-1000	-55.303236	-1000	0	0	0
Escherichia_coli_TW10722	0	0	-1000	-1000	-53.548112	-1000	0	0	0
Escherichia_coli_TW10828	0	0	-1000	-1000	-53.519407	-1000	0	0	0
Escherichia_coli_TW11039	0	0	-1000	-1000	-53.616197	-1000	0	0	0
Escherichia_coli_TW11681	0	0	-1000	-1000	-53.862844	-1000	0	0	0
Escherichia_coli_TW14301	0	0	-1000	-1000	-53.616166	-1000	0	0	0
Escherichia_coli_TW14425	0	0	-1000	-1000	-55.303236	-1000	0	0	0
Escherichia_coli_TW15901	0	0	-1000	-1000	-55.043351	-1000	0	0	0
Escherichia_coli_TX1999	0	0	-1000	-1000	-53.750185	-1000	0	-1000	0
Escherichia_coli_UM146	-1000	0	-1000	-1000	-72.434368	-1000	0	0	0
Escherichia_coli_UMN026	-1000	0	-1000	-1000	-53.742042	-1000	0	0	0
Escherichia_coli_UMNF18	0	0	-1000	-1000	-55.222562	-1000	0	0	0
Escherichia_coli_UMNK88	-1000	0	-1000	-1000	-55.351184	-1000	0	-1000	0
Escherichia_coli_UTI89_UPEC	-1000	0	-1000	-1000	-53.971935	-1000	0	0	0
Escherichia_coli_W	-1000	0	-1000	-1000	-53.634601	-1000	0	0	0
Escherichia_coli_W26	0	0	-1000	-1000	-53.746692	-1000	0	0	0
Escherichia_coli_XH001	0	0	-1000	-1000	-55.222562	-1000	0	0	0
Escherichia_coli_XH140A	0	0	-1000	-1000	-55.222555	-1000	0	0	0
Escherichia_coli_Xuzhou21	-1000	0	-1000	-1000	-53.840798	-1000	0	0	0
Escherichia_fergusonii_ATCC_35469	-1000	0	-1000	-1000	-79.08452	-1000	0	0	0
Escherichia_fergusonii_B253	0	0	-1000	-1000	-48.106004	-1000	0	0	0
Escherichia_fergusonii_ECD227	0	0	-1000	-1000	-49.097241	-1000	0	0	0
Escherichia_hermannii_NBRC_105704	0	0	-1000	-1000	-58.726659	-1000	0	0	0
Escherichia_sp_1_1_43	0	0	-1000	-1000	-29.316896	-1000	0	0	0
Escherichia_sp_3_2_53FAA	0	0	-1000	-1000	-44.053428	-1000	0	0	0
Escherichia_sp_4_1_40B	0	0	-1000	-1000	-56.403883	-1000	0	0	0
Escherichia_sp_TW09308	0	0	-1000	-1000	-50.406287	-1000	0	0	0
Ethanoligenens_harbinense_YUAN_3	-5.124562443	0	0	-320	-11.767516	-925.92593	0	-510.86957	0
Eubacterium_biforme_DSM_3989	0	0	0	-1000	-27.209171	0	-1000	0	0
Eubacterium_brachy_ATCC_33089	0	0	0	0	-1000	0	0	0	0
Eubacterium_callanderi_KIST612	0	0	0	-1000	-1000	0	0	0	0
Eubacterium_cellulosolvens_6	0	0	0	0	-19.299272	0	0	-949.68553	0
Eubacterium_cellulosolvens_LD2006	0	0	0	0	-16.206961	0	0	-271.85815	0
Eubacterium_cylindroides_T2_87	0	0	0	0	-168.1591	0	-1000	0	0
Eubacterium_desmolans_ATCC_43058	0	0	0	0	-17.155001	0	0	0	0
Eubacterium_dolichum_DSM_3991	0	0	0	0	-22.188255	0	0	-866.9867	0
Eubacterium_eligens_ATCC_27750	0	0	0	-882.9787234	0	0	-1000	0	0
Eubacterium_hallii_DSM_3353	0	0	0	-1000	-31.95811	-1000	-1000	0	0
Eubacterium_hallii_L2_7	0	0	0	-1000	-22.872419	-1000	0	-1000	0
Eubacterium_infirmum_F0142	0	0	0	-47.46145283	-33.597066	0	0	0	0
Eubacterium_limosum_DSM_20543	0	0	0	-1000	-1000	-1000	0	0	0
Eubacterium_limosum_ERR2221373	0	0	0	-1000	-1000	-1000	0	0	0
Eubacterium_oxidoreducens_DSM_3217	0	0	0	0	-10.584703	0	0	0	0
Eubacterium_plexicaudatum_ASF492	0	0	0	-32.16406166	-16.460581	0	0	-342.99517	0
Eubacterium_pyruvativorans_KHPC4	0	0	0	0	-8.8988051	0	-1000	0	0
Eubacterium_ramulus_ATCC_29099	0	0	0	-1000	-202.48974	0	0	0	0
Eubacterium_ramulus_ERR1022349	0	0	0	-45.73842259	-23.301897	0	0	-276.14243	0
Eubacterium_rectale_ATCC_33656	0	0	0	-1000	0	0	0	-405.17241	0
Eubacterium_rectale_DSM_17629	0	0	0	-1000	0	0	0	-446.42857	0
Eubacterium_rectale_ERR1022318	0	0	0	-1000	-25.724043	0	0	-450.21645	0
Eubacterium_rectale_ERR1022399	0	0	0	-1000	-25.259495	0	0	-450.21645	0
Eubacterium_rectale_ERR1022482	0	0	0	-1000	-25.597	0	0	-428.57143	0
Eubacterium_rectale_ERR1203958	0	0	0	-1000	-25.305354	0	0	-450.21645	0
Eubacterium_rectale_ERR1204052	0	0	0	-1000	-25.305354	0	0	-450.21645	0
Eubacterium_rectale_ERR2221160	0	0	0	-1000	-21.184504	0	0	-417.74892	0
Eubacterium_rectale_ERR2230060	0	0	0	-1000	-25.72405	0	0	-471.23894	0
Eubacterium_rectale_M104_1	0	0	0	-1000	0	0	0	-427.98354	0
Eubacterium_saphenum_ATCC_49989	0	0	0	0	-8.9263909	0	0	0	0
Eubacterium_siraeum_70_3	0	0	0	-1000	-20.785546	0	0	0	0
Eubacterium_siraeum_DSM_15702	0	0	0	-1000	-20.239067	0	0	0	0
Eubacterium_siraeum_V10Sc8a	0	0	0	-40.41340791	-24.335457	0	0	0	0
Eubacterium_sp_14_2	0	0	0	0	-2.0174848	0	0	-12.455516	0
Eubacterium_sp_3_1_31	0	0	0	0	-18.242551	-816.43836	0	0	0
Eubacterium_sulci_ATCC_35585	0	0	0	0	-21.090264	0	-1000	0	0
Eubacterium_ventriosum_ATCC_27560	0	0	0	-1000	0	0	0	0	0
Eubacterium_yurii_ATCC_43714	0	0	0	-1000	-17.31392	0	0	-274.77484	0
Eubacterium_yurii_subsp_margaretiae_ATCC_437	0	0	0	-1000	-15.687059	0	0	-5.6688981	0
Ewingella_americana_ATCC_33852	0	0	-1000	-1000	-30.678546	-1000	0	0	0
Ewingella_americana_BRK18a	0	0	-1000	-1000	-30.770674	-1000	0	0	0

Exiguobacterium_aurantiacum_DSM_6208	0	0	0	-1000	-20.987983	-1000	0	-1000	0
Exiguobacterium_marinum_ERR2221224	0	0	0	-39.62146832	-29.755442	-1000	0	-604.16667	0
Exiguobacterium_nov_ERR2221364	0	0	0	-1000	-22.76023	0	0	0	0
Exiguobacterium_undae_ERR2221363	0	0	0	-1000	-22.758862	-1000	0	0	0
Extibacter_muris_DSM_28560	0	0	0	-53.76561503	-27.79438	0	0	-500	0
Facklamia_languida_CCUG_37842	0	0	0	-20.06373402	-15.529721	0	0	0	0
Faecalibacterium_cf_ERR1022328	0	0	0	-500	-24.820512	0	0	-352.27273	0
Faecalibacterium_cf_prausnitzii_KLE1255	0	0	-1000	-1000	-23.516771	0	0	0	0
Faecalibacterium_prausnitzii_A2_165	0	0	-1000	0	-27.117246	0	0	-1000	0
Faecalibacterium_prausnitzii_ERR1022279	0	0	0	0	-22.972399	0	0	-784.09091	0
Faecalibacterium_prausnitzii_ERR1022327	0	0	0	-1000	-23.070503	0	0	-784.09091	0
Faecalibacterium_prausnitzii_ERR1022484	0	0	0	-47.79306641	-23.069603	0	0	-828.125	0
Faecalibacterium_prausnitzii_ERR2221188	0	0	0	0	-23.049874	0	0	-556.26314	0
Faecalibacterium_prausnitzii_L2_6	0	0	-1000	-1000	-25.074216	0	0	0	0
Faecalibacterium_prausnitzii_M21_2	0	0	-1000	-1000	-27.117246	0	0	0	0
Faecalibacterium_prausnitzii_SL3_3	0	0	-1000	-1000	-29.060065	0	0	0	0
Filifactor_alocis_ATCC_35896	0	0	0	-580	-7.8643236	0	0	0	0
Finegoldia_magna_ACS_171_V_Col3	0	0	0	-133.3333333	-20.918286	0	0	0	0
Finegoldia_magna_ATCC_29328	0	0	0	-26.42171016	-20.19118	0	0	0	0
Finegoldia_magna_ATCC_53516	0	0	0	-133.3333333	-20.977509	0	0	0	0
Finegoldia_magna_BVS033A4	0	0	0	-133.3333333	-20.780752	0	0	0	0
Finegoldia_magna_SY403409CC001050417	0	0	0	-133.3333333	-20.910934	0	0	0	0
Flaviflexus_massiliensis_SIT4	0	0	0	0	-13.227062	0	0	0	0
Flavobacterium_cheniae_CGMCC_16844	0	0	0	-15.43853715	-14.827847	0	0	0	0
Flavobacterium_cheniae_DSM_22462	0	0	0	-15.43853051	-14.827841	0	0	0	0
Flavobacterium_cucumis_DSM_18830	0	0	0	-9.979623874	0	0	0	0	0
Flavonifractor_plautii_ATCC_29863	0	0	0	-50.99218497	-19.843801	0	0	-1000	0
Flavonifractor_plautii_ERR1022312	0	0	0	-101.7626158	-50.828253	0	0	-571.18967	0
Flavonifractor_plautii_ERR1022406	0	0	0	-92.51594732	-46.138434	0	0	-564.72102	0
Flavonifractor_plautii_ERR1022420	0	0	0	-1000	-51.525663	0	0	-740.74074	0
Flavonifractor_plautii_ERR1022446	0	0	0	-103.8469189	-51.787508	0	0	-1000	0
Flavonifractor_plautii_ERR2221213	0	0	0	-103.1708249	-51.450755	0	0	-505.65162	0
Flavonifractor_plautii_ERR2221305	0	0	0	-103.8466441	-51.7875	0	0	-1000	0
Flavonifractor_plautii_YL31	0	0	0	-103.8473026	-51.787508	0	0	-1000	0
Frisingicoccus_caecimuris_DSM_28559	0	0	0	-83.86263252	-43.043947	0	0	0	0
Fructobacillus_durionis_DSM_19113	0	0	0	0	-10.357737	-60.480366	0	-791.76565	0
Fructobacillus_pseudoficulneus_DSM_15468	0	0	0	0	-10.448656	-30.794364	0	0	0
Fructobacillus_tropaeoli_F214_1	0	0	0	0	-10.469915	-37.639441	0	0	0
Fulvimarina_pelagi_DSM_15513	0	0	0	-1000	-18.207579	-1000	-1000	0	0
Fulvimarina_pelagi_HTCC2506	0	0	0	-1000	-18.28562	-1000	-1000	0	0
Fusicatenibacter_saccharivorans_ERR1022307	0	0	0	-932.0834278	-21.309764	0	0	-338.64539	0
Fusicatenibacter_saccharivorans_ERR1022400	0	0	0	-980.3193465	-21.537363	0	0	-458.39802	0
Fusicatenibacter_saccharivorans_ERR1022437	0	0	0	-931.9463072	-21.309865	0	0	-278.66401	0
Fusobacterium_gonidiaformans_3_1_5R	0	0	0	-1000	-26.120053	0	-1000	0	0
Fusobacterium_gonidiaformans_ATCC_25563	0	0	0	-1000	-26.120053	0	-1000	0	0
Fusobacterium_mortiferum_ATCC_9817	0	0	0	-1000	-33.31034	0	-1000	-1000	0
Fusobacterium_necrogenes_NCTC10723	0	0	0	-14.74348856	-9.1414056	0	0	0	0
Fusobacterium_necrophorum_D12	0	0	0	-1000	-25.931855	0	-1000	0	0
Fusobacterium_necrophorum_subsp_funduliform	0	0	0	-1000	-25.690561	0	-1000	0	0
Fusobacterium_necrophorum_subsp_funduliform	0	0	0	-1000	-28.862955	0	-1000	0	0
Fusobacterium_necrophorum_subsp_funduliform	0	0	0	-1000	-38.382914	0	-1000	0	0
Fusobacterium_nucleatum_subsp_animalis_11_3	0	0	0	-1000	-41.504537	0	-1000	0	0
Fusobacterium_nucleatum_subsp_animalis_21_1	0	0	0	-1000	-34.424153	0	-1000	0	0
Fusobacterium_nucleatum_subsp_animalis_3_1_	0	0	0	-1000	-24.499951	0	-1000	0	0
Fusobacterium_nucleatum_subsp_animalis_4_8	0	0	0	-1000	-200	0	-1000	0	0
Fusobacterium_nucleatum_subsp_animalis_7_1	0	0	0	-1000	-27.791979	0	-1000	0	0
Fusobacterium_nucleatum_subsp_animalis_ATCC	0	0	0	-1000	-35.197027	0	-1000	0	0
Fusobacterium_nucleatum_subsp_animalis_D11	0	0	0	-1000	-22.383167	0	-1000	0	0
Fusobacterium_nucleatum_subsp_animalis_OT_4	0	0	0	-1000	-32.785863	0	-1000	0	0
Fusobacterium_nucleatum_subsp_nucleatum_AT	0	0	0	-1000	-33.721836	0	-1000	0	0
Fusobacterium_nucleatum_subsp_nucleatum_AT	0	0	0	-1000	-29.546483	0	-1000	0	0
Fusobacterium_nucleatum_subsp_polymorphum	0	0	0	-1000	-40.203116	0	-1000	-834.27283	0
Fusobacterium_nucleatum_subsp_polymorphum	0	0	0	-1000	-39.606888	0	-1000	0	0
Fusobacterium_nucleatum_subsp_vincentii_3_1_	0	0	0	-1000	-29.683005	0	-1000	0	0
Fusobacterium_nucleatum_subsp_vincentii_3_1_	0	0	0	-1000	-200	0	-1000	0	0
Fusobacterium_nucleatum_subsp_vincentii_4_1_	0	0	0	-1000	-29.683005	0	-1000	0	0
Fusobacterium_nucleatum_subsp_vincentii_ATCC	0	0	0	-1000	-24.455518	0	-1000	0	0
Fusobacterium_perfoetens_ATCC_29250	0	0	0	-1000	-22.88142	0	0	-270.89957	0
Fusobacterium_periodonticum_1_1_41FAA	0	0	0	-1000	-24.478729	0	-1000	0	0
Fusobacterium_periodonticum_2_1_31	0	0	0	-1000	-23.747223	0	-1000	0	0
Fusobacterium_periodonticum_ATCC_33693	0	0	0	-1000	-35.829104	0	-1000	-777.80813	0
Fusobacterium_russii_ATCC_25533	0	0	0	-1000	-35.104945	0	0	0	0
Fusobacterium_ulcerans_12_1B	0	0	0	-1000	-50.165474	0	-1000	0	0
Fusobacterium_ulcerans_ATCC_49185	0	0	0	-60.663493	-32.454938	0	-1000	0	0
Fusobacterium_varium_ATCC_27725	0	0	0	-1000	-31.780832	0	-1000	0	0
Gardnerella_vaginalis_00703Bmash	0	0	0	-17.69835809	-12.588243	0	0	0	0
Gardnerella_vaginalis_14019_MetR	0	0	0	-1000	-14.626345	0	0	0	0
Gardnerella_vaginalis_JCP7672	0	0	0	-18.56813271	-13.215718	0	0	0	0
Gemella_haemolysans_ATCC_10379	0	0	0	-18.90962512	-12.355699	0	0	-595.37898	0
Gemella_haemolysans_M341	0	0	0	-37.77140241	-37.308583	-1000	0	-500	0
Gemella_morbillorum_M424	0	0	0	-18.86706513	-15.277898	-1000	0	-669.99578	0
Gemella_sanguinis_ATCC700632	0	0	0	-20.81365359	-16.995181	-1000	0	-775.74632	0
Gemella_sanguinis_M325	0	0	0	-18.90296438	-15.306968	-1000	0	-670.40771	0
Gemmata_obscuriglobus_DSM_5831	0	0	0	-1000	-13.741752	0	0	-558.82353	0
Gemmata_obscuriglobus_UQM_2246	0	0	0	-1000	-17.345706	0	0	-583.33333	0
Gemmatimonas_aurantiaca_T_27	0	0	0	-1000	-14.282074	0	0	-504.52681	0
Gemmiger_formicilis_ATCC_27749	0	0	0	-33.64514254	-15.059159	0	0	-267.62424	0
Gemmiger_formicilis_ERR1203953	0	0	0	-37.83881119	-16.768705	0	0	-270.48416	0
Gemmiger_formicilis_ERR1204047	0	0	0	-37.83879807	-16.768698	0	0	-270.48417	0
Gemmiger_formicilis_ERR2221191	0	0	0	0	-24.278676	0	0	-446.71463	0
Geobacillus_stearothermophilus_ATCC_7953	0	0	0	-1000	-22.319772	0	0	-625	0

Geobacillus_vulcani_PSS1	0	0	0	-1000	-25.278409	-1000	0	-1000	0
Gordonia_rubripertincta_NBRC_101908	0	0	0	0	-23.401411	-1000	0	-1000	0
Gordonia_terrae_C_6	0	0	0	0	-31.765803	-1000	-1000	-1000	0
Gordonia_terrae_NBRC_100016	0	0	0	0	-38.707445	-1000	-1000	-1000	0
Gordonibacter_pamelaeae_7_10_1_bT_DSM_193	0	0	0	0	0	0	-1000	0	0
Gordonibacter_pamelaeae_ERR2221155	0	0	0	-1000	-29.289807	0	-1000	0	0
Gordonibacter_pamelaeae_ERR2221387	0	0	0	-1000	-29.289797	0	-1000	0	0
Gottschalkia_acidurici_9a	0	0	0	-26.70657653	-10.913432	0	0	0	0
Gottschalkia_purinilyticum_DSM_1384	0	0	0	-26.31101593	-12.221412	0	0	0	0
Gracilibacter_sp_BRH_c7a	0	0	0	-1000	-12.244868	0	0	0	0
Granulicatella_adiacens_ATCC_49175	0	0	0	0	-19.763902	0	0	-1000	0
Granulicatella_elegans_ATCC_700633	0	0	0	0	-16.535607	0	0	-1000	0
Grimontia_hollisae_CIP_101886	0	0	0	-1000	-54.568194	0	0	0	0
Guyana_massiliensis_LF_3	0	0	0	-340.3939882	-11.527947	0	0	-413.04348	0
Haemophilus_aegyptius_ATCC_11116	0	0	0	-52.36944926	-37.387973	0	0	0	0
Haemophilus_haemolyticus_HK386	0	0	0	-1000	-37.362675	0	0	0	0
Haemophilus_haemolyticus_M19501	0	0	0	-39.60489449	-38.606852	0	0	0	0
Haemophilus_haemolyticus_M21127	0	0	0	-1000	-38.91923	0	0	0	0
Haemophilus_haemolyticus_M21621	0	0	0	-46.96398443	-19.575667	0	0	0	0
Haemophilus_haemolyticus_M21639	0	0	0	-1000	-37.011227	0	0	0	0
Haemophilus_influenzae_10810	0	0	0	-61.29549113	-21.590862	0	0	0	0
Haemophilus_influenzae_477	0	0	0	-90.63402369	-36.95977	0	0	0	0
Haemophilus_influenzae_6P18H1	0	0	0	-71.64180319	-21.229517	0	0	0	0
Haemophilus_influenzae_7P49H1	0	0	0	-87.20199286	-22.243217	0	0	0	0
Haemophilus_influenzae_86_028NP	0	0	0	-61.28989177	-21.497154	0	0	0	0
Haemophilus_influenzae_F3031	0	0	0	-83.04418866	-21.366799	0	0	0	0
Haemophilus_influenzae_F3047	0	0	0	-84.41100491	-23.414394	0	0	0	0
Haemophilus_influenzae_Hi375	0	0	0	-93.5683683	-37.228564	0	0	0	0
Haemophilus_influenzae_NT127	0	0	0	-82.14520407	-22.250101	0	0	0	0
Haemophilus_influenzae_PittAA	0	0	0	-75.73324561	-23.568593	0	0	0	0
Haemophilus_influenzae_PittEE	0	0	0	-90.63264982	-36.929335	0	0	0	0
Haemophilus_influenzae_PittGG	0	0	0	-93.57155932	-37.419839	0	0	0	0
Haemophilus_influenzae_PittHH	0	0	0	-1000	-25.026468	0	0	0	0
Haemophilus_influenzae_PittII	0	0	0	-46.01995961	-24.613291	0	0	0	0
Haemophilus_influenzae_R2846	0	0	0	-1000	-37.864928	0	0	0	0
Haemophilus_influenzae_R2866	0	0	0	-46.1067189	-24.657694	0	0	0	0
Haemophilus_influenzae_Rd_KW20	0	0	0	-1000	-20.538274	0	0	0	0
Haemophilus_influenzae_RdAW	0	0	0	-1000	-20.538268	0	0	0	0
Haemophilus_parahaemolyticus_CCUG_3716_371	0	0	0	-1000	-32.653722	0	0	-717.3913	0
Haemophilus_parahaemolyticus_G321	0	0	0	-1000	-32.554104	0	0	-873.13433	0
Haemophilus_parahaemolyticus_HK385	0	0	0	-1000	-32.701108	0	0	-750	0
Haemophilus_parainfluenzae_215035_2_ISO5	0	0	0	-1000	-38.241377	0	0	-906.25	0
Haemophilus_parainfluenzae_ATCC_33392	0	0	0	-1000	-41.157658	0	0	-1000	0
Haemophilus_parainfluenzae_HK2019	0	0	0	-1000	-40.954847	0	0	-1000	0
Haemophilus_parainfluenzae_HK262	0	0	0	-1000	-40.04085	0	0	-1000	0
Haemophilus_parainfluenzae_T3T1	0	0	0	-123.5702657	-47.095688	0	0	-959.85401	0
Haemophilus_paraphrohaemolyticus_CCUG_3718	0	0	0	-1000	-32.886079	0	0	-717.3913	0
Haemophilus_paraphrohaemolyticus_HK411	0	0	0	-1000	-33.028575	0	0	-750	0
Haemophilus_pittmaniae_HK_85	0	0	0	-1000	-38.126262	0	0	-363.80894	0
Haemophilus_pittmaniae_NCTC13334	0	0	0	-1000	-37.126234	0	0	-801.86283	0
Haemophilus_sputorum_CCUG_13788	0	0	0	-73.17073171	-28.403875	0	0	-861.11111	0
Haemophilus_sputorum_HK_2154	0	0	0	-1000	-27.498862	0	0	-1000	0
Hafnia_alvei_ATCC_51873	0	0	-1000	-1000	-64.230398	-1000	0	0	0
Hafnia_alvei_BIDMC_31	0	-1000	-1000	-1000	-63.520636	-1000	0	-1000	0
Hafnia_alvei_ERR2221402	0	0	-1000	-1000	-38.423426	-1000	0	-1000	0
Hafnia_alvei_FB1	0	0	-1000	-1000	-34.277442	-1000	0	-1000	0
Hafnia_alvei_HUMV_5920	0	0	-1000	-1000	-38.420484	-1000	0	-1000	0
Hafnia_paralvei_FDAARGOS_158	0	0	-1000	-1000	-38.363752	-1000	0	-1000	0
Halococcus_morrhuae_DSM1307	0	0	0	-1000	-12.763754	0	0	0	0
Halomonas_campaniensis_LS21	0	0	0	-1000	-24.044764	0	0	-1000	0
Helicobacter_apodemus_SCJK1	0	0	0	0	-8.6046791	0	0	0	0
Helicobacter_bilis_ATCC_43879	0	0	0	-1000	0	0	0	0	0
Helicobacter_bilis_WiWa	0	0	0	-1000	0	0	0	0	0
Helicobacter_canadensis_MIT_98_5491	0	0	0	-11.19978499	-56.074766	0	0	0	0
Helicobacter_cinaedi_CCUG_18818	0	0	0	0	-12.457873	0	0	0	0
Helicobacter_fennelliae_MRY12_0050	0	0	0	-1000	-7.9475119	0	0	0	0
Helicobacter_fennelliae_NCTC13102	0	0	0	-1000	-7.8636861	0	0	0	0
Helicobacter_hepaticus_ATCC_51449	0	0	0	0	-4.6588632	0	0	0	0
Helicobacter_pullorum_MIT_98_5489	0	0	0	-1000	-12.690132	0	0	0	0
Helicobacter_pylori_2017	0	0	0	-1000	-16.29573	0	0	0	0
Helicobacter_pylori_2018	0	0	0	-1000	-17.798402	0	0	0	0
Helicobacter_pylori_26695	0	0	0	-1000	-14.122596	0	-1000	0	0
Helicobacter_pylori_26695_1MET	0	0	0	-1000	-17.745458	0	0	0	0
Helicobacter_pylori_35A	0	0	0	-1000	-17.867878	0	0	0	0
Helicobacter_pylori_51	0	0	0	-1000	-8.3287098	0	0	0	0
Helicobacter_pylori_52	0	0	0	-1000	-21.334369	0	0	0	0
Helicobacter_pylori_83	0	0	0	-1000	-15.483698	0	0	0	0
Helicobacter_pylori_8A3	0	0	0	-1000	-8.6926089	0	0	0	0
Helicobacter_pylori_908	0	0	0	-1000	-16.090782	0	0	0	0
Helicobacter_pylori_98_10	0	0	0	-1000	-16.24174	0	0	0	0
Helicobacter_pylori_A45	0	0	0	-1000	-8.9166548	0	0	0	0
Helicobacter_pylori_Aklavik117	0	0	0	-1000	-17.866864	0	0	0	0
Helicobacter_pylori_Aklavik86	0	0	0	-1000	-16.619367	0	0	0	0
Helicobacter_pylori_B128	0	0	0	0	-8.3949478	0	0	0	0
Helicobacter_pylori_B38	0	0	0	-1000	-8.923869	0	0	0	0
Helicobacter_pylori_B45	0	0	0	-1000	-8.3952503	0	0	0	0
Helicobacter_pylori_B8	0	0	0	-1000	-8.2942945	0	0	0	0
Helicobacter_pylori_BCS100H1	0	0	0	0	-9.1442908	0	0	0	0
Helicobacter_pylori_BM013A	0	0	0	-1000	-17.745299	0	0	0	0
Helicobacter_pylori_CCHI_33	0	0	0	-1000	-16.620295	0	0	0	0
Helicobacter_pylori_CPY1124	0	0	0	-1000	-18.554371	0	0	0	0

Helicobacter_pylori_CPY1313	0	0	0	-1000	-16.620404	0	0	0	0
Helicobacter_pylori_CPY1662	0	0	0	-1000	-8.2952197	0	0	0	0
Helicobacter_pylori_CPY1962	0	0	0	-1000	-16.620389	0	0	0	0
Helicobacter_pylori_CPY3281	0	0	0	-1000	-20.645049	0	0	0	0
Helicobacter_pylori_CPY6081	0	0	0	-1000	-17.867937	0	0	0	0
Helicobacter_pylori_CPY6261	0	0	0	-1000	-18.343025	0	0	0	0
Helicobacter_pylori_CPY6271	0	0	0	-1000	-8.9160996	0	0	0	0
Helicobacter_pylori_CPY6311	0	0	0	-1000	-16.62044	0	0	0	0
Helicobacter_pylori_Cuz20	0	0	0	-1000	-9.2704271	0	0	0	0
Helicobacter_pylori_ELS37	0	0	0	-1000	-8.9166253	0	0	0	0
Helicobacter_pylori_F16	0	0	0	-1000	-17.882497	0	0	0	0
Helicobacter_pylori_F30	0	0	0	-1000	-17.867895	0	0	0	0
Helicobacter_pylori_F32	0	0	0	-1000	-8.9160908	0	0	0	0
Helicobacter_pylori_F57	0	0	0	-1000	-17.867937	0	0	0	0
Helicobacter_pylori_G27	0	0	0	-1000	-8.5983371	0	0	0	0
Helicobacter_pylori_GAM100Ai	0	0	0	-1000	-16.635137	0	0	0	0
Helicobacter_pylori_GAM101Biv	0	0	0	-1000	-18.282241	0	0	0	0
Helicobacter_pylori_GAM103Bi	0	0	0	-1000	-17.867736	0	0	0	0
Helicobacter_pylori_GAM105Ai	0	0	0	-1000	-18.281211	0	0	0	0
Helicobacter_pylori_GAM114Ai	0	0	0	-1000	-18.282148	0	0	0	0
Helicobacter_pylori_GAM115Ai	0	0	0	-1000	-18.403139	0	0	0	0
Helicobacter_pylori_GAM118Bi	0	0	0	-1000	-18.540576	0	0	0	0
Helicobacter_pylori_GAM119Bi	0	0	0	-1000	-18.282334	0	0	0	0
Helicobacter_pylori_GAM120Ai	0	0	0	-1000	-16.620324	0	0	0	0
Helicobacter_pylori_GAM121Aii	0	0	0	-1000	-18.282241	0	0	0	0
Helicobacter_pylori_GAM201Ai	0	0	0	-1000	-18.281025	0	0	0	0
Helicobacter_pylori_GAM210Bi	0	0	0	-1000	-18.281118	0	0	0	0
Helicobacter_pylori_GAM231Ai	0	0	0	-1000	-9.122436	0	0	0	0
Helicobacter_pylori_GAM239Bi	0	0	0	-1000	-18.282334	0	0	0	0
Helicobacter_pylori_GAM244Ai	0	0	0	-1000	-9.1223897	0	0	0	0
Helicobacter_pylori_GAM245Ai	0	0	0	-1000	-18.282334	0	0	0	0
Helicobacter_pylori_GAM246Ai	0	0	0	-1000	-18.281211	0	0	0	0
Helicobacter_pylori_GAM249T	0	0	0	-1000	-18.282241	0	0	0	0
Helicobacter_pylori_GAM250AFi	0	0	0	-1000	-18.281025	0	0	0	0
Helicobacter_pylori_GAM250T	0	0	0	-1000	-18.282148	0	0	0	0
Helicobacter_pylori_GAM252Bi	0	0	0	-1000	-18.281025	0	0	0	0
Helicobacter_pylori_GAM252T	0	0	0	-1000	-18.282148	0	0	0	0
Helicobacter_pylori_GAM254Ai	0	0	0	-1000	-18.281118	0	0	0	0
Helicobacter_pylori_GAM260ASi	0	0	0	-1000	-18.281118	0	0	0	0
Helicobacter_pylori_GAM260Bi	0	0	0	-1000	-17.884957	0	0	0	0
Helicobacter_pylori_GAM260BSi	0	0	0	-1000	-18.280932	0	0	0	0
Helicobacter_pylori_GAM263BFi	0	0	0	-1000	-18.282334	0	0	0	0
Helicobacter_pylori_GAM264Ai	0	0	0	-1000	-18.282148	0	0	0	0
Helicobacter_pylori_GAM265BSii	0	0	0	-1000	-18.281118	0	0	0	0
Helicobacter_pylori_GAM270ASi	0	0	0	-1000	-18.29911	0	0	0	0
Helicobacter_pylori_GAM42Ai	0	0	0	-1000	-18.282241	0	0	0	0
Helicobacter_pylori_GAM80Ai	0	0	0	-1000	-18.403056	0	0	0	0
Helicobacter_pylori_GAM83Bi	0	0	0	-1000	-18.281025	0	0	0	0
Helicobacter_pylori_GAM83T	0	0	0	-1000	-18.281025	0	0	0	0
Helicobacter_pylori_GAM96Ai	0	0	0	-1000	-18.281025	0	0	0	0
Helicobacter_pylori_Gambia94_24	0	0	0	-1000	-17.867803	0	0	0	0
Helicobacter_pylori_GAMchJs106B	0	0	0	-1000	-18.282055	0	0	0	0
Helicobacter_pylori_GAMchJs114i	0	0	0	-1000	-18.281118	0	0	0	0
Helicobacter_pylori_GAMchJs117Ai	0	0	0	-1000	-18.282241	0	0	0	0
Helicobacter_pylori_GAMchJs124i	0	0	0	-1000	-18.282241	0	0	0	0
Helicobacter_pylori_GAMchJs136i	0	0	0	-1000	-16.620295	0	0	0	0
Helicobacter_pylori_HLJHP193	0	0	0	-1000	-9.0940135	0	0	0	0
Helicobacter_pylori_HLJHP253	0	0	0	-1000	-16.620389	0	0	0	0
Helicobacter_pylori_HLJHP256	0	0	0	-1000	-8.9165217	0	0	0	0
Helicobacter_pylori_HLJHP271	0	0	0	-1000	-8.9165383	0	0	0	0
Helicobacter_pylori_Hp_A_11	0	0	0	-1000	-8.9251948	0	0	0	0
Helicobacter_pylori_Hp_A_14	0	0	0	-1000	-8.9166087	0	0	0	0
Helicobacter_pylori_Hp_A_16	0	0	0	-1000	-16.620295	0	0	0	0
Helicobacter_pylori_Hp_A_17	0	0	0	-1000	-16.620295	0	0	0	0
Helicobacter_pylori_Hp_A_20	0	0	0	-1000	-17.86777	0	0	0	0
Helicobacter_pylori_Hp_A_26	0	0	0	-1000	-8.9166087	0	0	0	0
Helicobacter_pylori_Hp_A_27	0	0	0	-1000	-8.9558808	0	0	0	0
Helicobacter_pylori_Hp_A_4	0	0	0	-1000	-17.86777	0	0	0	0
Helicobacter_pylori_Hp_A_5	0	0	0	-1000	-17.86777	0	0	0	0
Helicobacter_pylori_Hp_A_6	0	0	0	-1000	-16.620295	0	0	0	0
Helicobacter_pylori_Hp_A_8	0	0	0	-1000	-17.86777	0	0	0	0
Helicobacter_pylori_Hp_A_9	0	0	0	-1000	-8.9166548	0	0	0	0
Helicobacter_pylori_Hp_H_1	0	0	0	-1000	-17.867844	0	0	0	0
Helicobacter_pylori_Hp_H_10	0	0	0	-1000	-16.620295	0	0	0	0
Helicobacter_pylori_Hp_H_11	0	0	0	-1000	-8.9166253	0	0	0	0
Helicobacter_pylori_Hp_H_16	0	0	0	-1000	-17.867844	0	0	0	0
Helicobacter_pylori_Hp_H_18	0	0	0	-1000	-18.6952	0	0	0	0
Helicobacter_pylori_Hp_H_19	0	0	0	-1000	-17.883474	0	0	0	0
Helicobacter_pylori_Hp_H_21	0	0	0	-1000	-17.86777	0	0	0	0
Helicobacter_pylori_Hp_H_23	0	0	0	-1000	-16.620324	0	0	0	0
Helicobacter_pylori_Hp_H_24	0	0	0	-1000	-17.86777	0	0	0	0
Helicobacter_pylori_Hp_H_24b	0	0	0	-1000	-17.86777	0	0	0	0
Helicobacter_pylori_Hp_H_24c	0	0	0	-1000	-17.86777	0	0	0	0
Helicobacter_pylori_Hp_H_27	0	0	0	-1000	-10.07599	0	0	0	0
Helicobacter_pylori_Hp_H_28	0	0	0	-1000	-8.2947351	0	0	0	0
Helicobacter_pylori_Hp_H_29	0	0	0	-1000	-17.867803	0	0	0	0
Helicobacter_pylori_Hp_H_3	0	0	0	-1000	-17.867878	0	0	0	0
Helicobacter_pylori_Hp_H_30	0	0	0	-1000	-17.867703	0	0	0	0
Helicobacter_pylori_Hp_H_34	0	0	0	-1000	-17.86777	0	0	0	0
Helicobacter_pylori_Hp_H_36	0	0	0	-1000	-17.867803	0	0	0	0
Helicobacter_pylori_Hp_H_4	0	0	0	-1000	-17.867803	0	0	0	0

Helicobacter_pylori_Hp_H_41	0	0	0	-1000	-17.86777	0	0	0	0
Helicobacter_pylori_Hp_H_42	0	0	0	-1000	-16.620324	0	0	0	0
Helicobacter_pylori_Hp_H_43	0	0	0	-1000	-8.2951798	0	0	0	0
Helicobacter_pylori_Hp_H_44	0	0	0	-1000	-17.867803	0	0	0	0
Helicobacter_pylori_Hp_H_45	0	0	0	-1000	-8.295212	0	0	0	0
Helicobacter_pylori_Hp_H_5b	0	0	0	-1000	-16.633948	0	0	0	0
Helicobacter_pylori_Hp_H_6	0	0	0	-1000	-8.2947351	0	0	0	0
Helicobacter_pylori_Hp_H_9	0	0	0	-1000	-8.9160908	0	0	0	0
Helicobacter_pylori_Hp_M1	0	0	0	-1000	-17.867803	0	0	0	0
Helicobacter_pylori_Hp_M2	0	0	0	-1000	-16.620324	0	0	0	0
Helicobacter_pylori_Hp_M3	0	0	0	-1000	-17.86777	0	0	0	0
Helicobacter_pylori_Hp_M4	0	0	0	-1000	-16.620295	0	0	0	0
Helicobacter_pylori_Hp_M5	0	0	0	-1000	-17.86777	0	0	0	0
Helicobacter_pylori_Hp_M6	0	0	0	-1000	-17.867803	0	0	0	0
Helicobacter_pylori_Hp_M9	0	0	0	-1000	-17.86777	0	0	0	0
Helicobacter_pylori_Hp_P_1	0	0	0	-1000	-17.867803	0	0	0	0
Helicobacter_pylori_Hp_P_11	0	0	0	-1000	-16.620324	0	0	0	0
Helicobacter_pylori_Hp_P_11b	0	0	0	-1000	-17.867803	0	0	0	0
Helicobacter_pylori_Hp_P_13	0	0	0	-1000	-20.691663	0	0	0	0
Helicobacter_pylori_Hp_P_13b	0	0	0	-1000	-16.62036	0	0	0	0
Helicobacter_pylori_Hp_P_15	0	0	0	-1000	-8.9166253	0	0	0	0
Helicobacter_pylori_Hp_P_15b	0	0	0	-1000	-8.3252311	0	0	0	0
Helicobacter_pylori_Hp_P_16	0	0	0	-1000	-8.9552106	0	0	0	0
Helicobacter_pylori_Hp_P_1b	0	0	0	-1000	-16.618467	0	0	0	0
Helicobacter_pylori_Hp_P_2	0	0	0	-1000	-17.88355	0	0	0	0
Helicobacter_pylori_Hp_P_23	0	0	0	-1000	-8.6507807	0	0	0	0
Helicobacter_pylori_Hp_P_25	0	0	0	-1000	-15.328684	0	0	0	0
Helicobacter_pylori_Hp_P_25c	0	0	0	-1000	-17.86777	0	0	0	0
Helicobacter_pylori_Hp_P_25d	0	0	0	-1000	-17.86777	0	0	0	0
Helicobacter_pylori_Hp_P_26	0	0	0	-1000	-18.222959	0	0	0	0
Helicobacter_pylori_Hp_P_28b	0	0	0	-1000	-16.620295	0	0	0	0
Helicobacter_pylori_Hp_P_2b	0	0	0	-1000	-17.88355	0	0	0	0
Helicobacter_pylori_Hp_P_3	0	0	0	-1000	-17.86777	0	0	0	0
Helicobacter_pylori_Hp_P_30	0	0	0	-1000	-8.9166341	0	0	0	0
Helicobacter_pylori_Hp_P_3b	0	0	0	-1000	-16.619367	0	0	0	0
Helicobacter_pylori_Hp_P_4	0	0	0	-1000	-16.620324	0	0	0	0
Helicobacter_pylori_Hp_P_41	0	0	0	-1000	-17.867803	0	0	0	0
Helicobacter_pylori_Hp_P_4c	0	0	0	-1000	-16.620324	0	0	0	0
Helicobacter_pylori_Hp_P_4d	0	0	0	-1000	-17.867803	0	0	0	0
Helicobacter_pylori_Hp_P_62	0	0	0	-1000	-16.621253	0	0	0	0
Helicobacter_pylori_Hp_P_74	0	0	0	-1000	-8.2951977	0	0	0	0
Helicobacter_pylori_Hp_P_8	0	0	0	-1000	-17.86777	0	0	0	0
Helicobacter_pylori_Hp_P_8b	0	0	0	-1000	-16.620295	0	0	0	0
Helicobacter_pylori_HP116Bi	0	0	0	-1000	-18.282334	0	0	0	0
Helicobacter_pylori_Hp238	0	0	0	-1000	-17.745753	0	0	0	0
Helicobacter_pylori_HP250AFii	0	0	0	-1000	-18.281025	0	0	0	0
Helicobacter_pylori_HP250AFiii	0	0	0	-1000	-18.282148	0	0	0	0
Helicobacter_pylori_HP250AFiV	0	0	0	-1000	-18.280932	0	0	0	0
Helicobacter_pylori_HP250ASi	0	0	0	-1000	-18.281025	0	0	0	0
Helicobacter_pylori_HP250ASii	0	0	0	-1000	-18.281025	0	0	0	0
Helicobacter_pylori_HP250BFii	0	0	0	-1000	-17.02807	0	0	0	0
Helicobacter_pylori_HP250BFiii	0	0	0	-1000	-18.281025	0	0	0	0
Helicobacter_pylori_HP250BFiV	0	0	0	-1000	-18.280932	0	0	0	0
Helicobacter_pylori_HP250BSi	0	0	0	-1000	-18.281025	0	0	0	0
Helicobacter_pylori_HP260AFi	0	0	0	-1000	-18.281118	0	0	0	0
Helicobacter_pylori_HP260AFii	0	0	0	-1000	-18.281118	0	0	0	0
Helicobacter_pylori_HP260ASii	0	0	0	-1000	-18.281118	0	0	0	0
Helicobacter_pylori_HP260BFii	0	0	0	-1000	-18.281025	0	0	0	0
Helicobacter_pylori_HP260Bi	0	0	0	-1000	-17.86777	0	0	0	0
Helicobacter_pylori_HPAG1	0	0	0	-1000	-8.9740257	0	0	0	0
Helicobacter_pylori_HUP_B14	0	0	0	-1000	-8.9166087	0	0	0	0
Helicobacter_pylori_India7	0	0	0	-1000	-17.883572	0	0	0	0
Helicobacter_pylori_J99	0	0	0	-1000	-15.786795	0	0	0	0
Helicobacter_pylori_Lithuania75	0	0	0	-1000	-8.9166253	0	0	0	0
Helicobacter_pylori_MALT	0	0	0	-1000	-8.1426459	0	0	0	0
Helicobacter_pylori_N6	0	0	0	-1000	-8.1941255	0	0	0	0
Helicobacter_pylori_NAK7	0	0	0	-1000	-8.9166087	0	0	0	0
Helicobacter_pylori_NQ1671	0	0	0	-1000	-8.916588	0	0	0	0
Helicobacter_pylori_NQ1707	0	0	0	0	-18.695345	0	0	0	0
Helicobacter_pylori_NQ1712	0	0	0	-1000	-8.2834437	0	0	0	0
Helicobacter_pylori_NQ315	0	0	0	-1000	-8.2765477	0	0	0	0
Helicobacter_pylori_NQ352	0	0	0	-1000	-9.3310712	0	0	0	0
Helicobacter_pylori_NQ367	0	0	0	-1000	-8.4828128	0	0	0	0
Helicobacter_pylori_NQ392	0	0	0	-1000	-17.334039	0	0	0	0
Helicobacter_pylori_NQ4044	0	0	0	-1000	-8.2943124	0	0	0	0
Helicobacter_pylori_NQ4053	0	0	0	-1000	-8.9166253	0	0	0	0
Helicobacter_pylori_NQ4060	0	0	0	-1000	-8.9990035	0	0	0	0
Helicobacter_pylori_NQ4076	0	0	0	-1000	-8.4452964	0	0	0	0
Helicobacter_pylori_NQ4099	0	0	0	-1000	-8.5944823	0	0	0	0
Helicobacter_pylori_NQ4110	0	0	0	-1000	-8.916588	0	0	0	0
Helicobacter_pylori_NQ4161	0	0	0	-1000	-8.9166087	0	0	0	0
Helicobacter_pylori_NQ4191	0	0	0	-1000	-8.916588	0	0	0	0
Helicobacter_pylori_NQ4200	0	0	0	-1000	-8.9166087	0	0	0	0
Helicobacter_pylori_NQ4216	0	0	0	-1000	-8.9166253	0	0	0	0
Helicobacter_pylori_NQ4228	0	0	0	-1000	-8.2951977	0	0	0	0
Helicobacter_pylori_OK113	0	0	0	-1000	-8.2952376	0	0	0	0
Helicobacter_pylori_OK310	0	0	0	-1000	-16.620404	0	0	0	0
Helicobacter_pylori_P12	0	0	0	-1000	-8.3449086	0	0	0	0
Helicobacter_pylori_PeCan18	0	0	0	-1000	-17.867844	0	0	0	0
Helicobacter_pylori_PeCan4	0	0	0	-1000	-8.9166253	0	0	0	0
Helicobacter_pylori_Puno120	0	0	0	-1000	-17.867878	0	0	0	0

Helicobacter_pylori_Puno135	0	0	0	-1000	-17.866822	0	0	0	0
Helicobacter_pylori_R036d	0	0	0	-1000	-8.9553039	0	0	0	0
Helicobacter_pylori_R037c	0	0	0	-1000	-8.9166253	0	0	0	0
Helicobacter_pylori_R038b	0	0	0	-1000	-8.2951977	0	0	0	0
Helicobacter_pylori_R046Wa	0	0	0	-1000	-8.2951798	0	0	0	0
Helicobacter_pylori_R055a	0	0	0	-1000	-8.3418547	0	0	0	0
Helicobacter_pylori_R056a	0	0	0	-1000	-8.9160908	0	0	0	0
Helicobacter_pylori_R32b	0	0	0	-1000	-8.3015134	0	0	0	0
Helicobacter_pylori_Rif1	0	0	0	-1000	-8.9729807	0	0	0	0
Helicobacter_pylori_Rif2	0	0	0	-1000	-8.9729807	0	0	0	0
Helicobacter_pylori_Sat464	0	0	0	-1000	-17.867803	0	0	0	0
Helicobacter_pylori_Shi112	0	0	0	-1000	-8.9166341	0	0	0	0
Helicobacter_pylori_Shi169	0	0	0	-1000	-8.9166253	0	0	0	0
Helicobacter_pylori_Shi417	0	0	0	-1000	-17.867895	0	0	0	0
Helicobacter_pylori_Shi470	0	0	0	-1000	-14.806488	0	0	0	0
Helicobacter_pylori_SJM180	0	0	0	-1000	-8.9166253	0	0	0	0
Helicobacter_pylori_SNT49	0	0	0	-1000	-8.9166087	0	0	0	0
Helicobacter_pylori_SouthAfrica7	0	0	0	-1000	-17.883675	0	0	0	0
Helicobacter_pylori_UM007	0	0	0	-1000	-17.867895	0	0	0	0
Helicobacter_pylori_UM018	0	0	0	-1000	-8.916588	0	0	0	0
Helicobacter_pylori_UM034	0	0	0	-1000	-16.62044	0	0	0	0
Helicobacter_pylori_UM037	0	0	0	-1000	-8.9166253	0	0	0	0
Helicobacter_pylori_UM045	0	0	0	-1000	-8.9171433	0	0	0	0
Helicobacter_pylori_UM054	0	0	0	-1000	-17.867803	0	0	0	0
Helicobacter_pylori_UM066	0	0	0	-1000	-18.695778	0	0	0	0
Helicobacter_pylori_UM299	0	0	0	-1000	-8.9166341	0	0	0	0
Helicobacter_pylori_v225d	0	0	0	-1000	-17.945536	0	0	0	0
Helicobacter_pylori_XZ274	0	0	0	-1000	-16.534006	0	0	0	0
Helicobacter_rodentium_ATCC_700285	0	0	0	-16.96820882	-7.941897	0	0	0	0
Helicobacter_trogontum_50960_7	0	0	0	-1000	-7.8994704	0	0	0	0
Helicobacter_typhlonius_ATCC_BAA_367	0	0	0	-195.1219512	-4.5662017	0	0	0	0
Helicobacter_winghamensis_ATCC_BAA_430	0	0	0	-10.12420629	-4.5076076	0	0	0	0
Herbaspirillum_huttiense_1147	0	-1000	-1000	-1000	-1000	-1000	0	0	-1000
Herbaspirillum_huttiense_AU6965_10_3	0	-1000	-1000	-1000	-1000	-1000	0	0	-1000
Herbaspirillum_huttiense_NFYY_53159	0	-1000	-1000	-1000	-1000	-1000	0	0	-1000
Herbaspirillum_huttiense_subsp_putei_IAM_150	0	-1000	-1000	-1000	-1000	-1000	0	0	-1000
Holdemania_filiformis_VPI_J1_31B_1_DSM_1204	0	0	0	0	-19.102204	0	0	-387.5	0
Holdemania_massiliensis_AP2	0	0	0	0	-16.31467	0	0	-751.31638	0
Hydrotalea_flava_isolate_2	0	0	0	0	-14.218046	0	0	0	0
Intestinibacter_bartlettii_ERR1022395	0	0	0	-77.96912371	-1000	-1000	-1000	-1000	0
Intestinibacter_bartlettii_ERR1022418	0	0	0	-77.96178733	-1000	-1000	-1000	-1000	0
Intestinibacter_nov_ERR2221096	0	0	0	-26.38454514	-18.766434	0	0	0	0
Intestinimonas_butyriciproducens_AF211	0	0	0	0	-12.603151	0	0	0	0
Intestinimonas_butyriciproducens_ERR1022450	0	0	0	0	-29.99121	0	0	0	0
Intestinimonas_butyriciproducens_SRB_521_5_I	0	0	0	0	-30.874936	0	0	0	0
Intestinimonas_nov_ERR1022454	0	0	0	-1000	-41.539063	0	0	0	0
Intestinimonas_nov_ERR171285	0	0	0	-1000	-41.53875	0	0	0	0
Jeddahella_massiliensis_OL_1	0	0	0	-32.88914304	-16.549987	-1000	0	-927.68497	0
Johnsonella_ignava_ATCC_51276	0	0	0	-15.8539595	-13.033074	0	0	-679.54193	0
Jonquetella_anthropi_DSM_22815	0	0	0	-1000	-1000	0	0	0	0
Jonquetella_anthropi_E3_33_E1	0	0	0	-1000	-1000	0	0	0	0
Kallipyga_massiliensis_ph2	0	0	0	-18.74572349	-14.588529	0	0	0	0
Kandleria_vitulina_DSM_20405	0	0	0	-39.2557633	-20.839753	0	0	-803.23617	0
Kingella_denitrificans_ATCC_33394	0	0	0	-23.69423766	-16.333248	0	0	0	0
Kingella_denitrificans_isolate_GC77	0	0	0	-18.1861828	-16.306427	0	0	0	0
Kingella_oralis_ATCC_51147	0	0	0	-1000	-40.335121	0	0	0	0
Klebsiella_aerogenes_CAV1320	0	0	-1000	-1000	-39.774814	-1000	0	-1000	0
Klebsiella_aerogenes_G7	0	0	-1000	-1000	-39.774775	-1000	0	-1000	0
Klebsiella_michiganensis_10_5242	0	0	-1000	-1000	-41.248158	-1000	0	-1000	0
Klebsiella_michiganensis_10_5250	0	0	-1000	-1000	-60.455641	-1000	0	-1000	0
Klebsiella_michiganensis_FDAARGOS_66	0	0	-1000	-1000	-41.248152	-1000	0	-1000	0
Klebsiella_michiganensis_M1	0	0	-1000	-1000	-41.248158	-1000	0	-1000	0
Klebsiella_michiganensis_MGH87	0	0	-1000	-1000	-60.455645	-1000	0	-1000	0
Klebsiella_oxytoca_10_5243	0	0	-1000	-1000	-36.902214	-1000	0	-1000	0
Klebsiella_oxytoca_10_5245	0	0	-1000	-1000	-36.49958	-1000	0	-1000	0
Klebsiella_oxytoca_10_5248	0	0	-1000	-1000	-36.533337	-1000	0	-1000	0
Klebsiella_oxytoca_11492_1	0	0	-1000	-1000	-36.533337	-1000	0	-1000	0
Klebsiella_oxytoca_CAV1099	0	0	-1000	-1000	-41.248152	-1000	0	-1000	0
Klebsiella_oxytoca_CAV1335	0	0	-1000	-1000	-41.248158	-1000	0	-1000	0
Klebsiella_oxytoca_CAV1374	0	0	-1000	-1000	-60.455645	-1000	0	-1000	0
Klebsiella_oxytoca_CHS143	0	0	-1000	-1000	-41.248152	-1000	0	-1000	0
Klebsiella_oxytoca_E718	-1000	0	-1000	-1000	-36.531379	-1000	0	-1000	0
Klebsiella_oxytoca_ERR2221287	0	0	-1000	-1000	-41.248152	-1000	0	-1000	0
Klebsiella_oxytoca_ERR2221335	0	0	-1000	-1000	-41.248152	-1000	0	-1000	0
Klebsiella_oxytoca_ERR2221348	0	0	-1000	-1000	-41.248152	-1000	0	-1000	0
Klebsiella_oxytoca_ERR2221399	0	0	-1000	-1000	-41.248152	-1000	0	-1000	0
Klebsiella_oxytoca_ERR2221400	0	0	-1000	-1000	-41.248158	-1000	0	-1000	0
Klebsiella_oxytoca_ICU1_2b	0	0	-1000	-1000	-41.248152	-1000	0	-1000	0
Klebsiella_oxytoca_JKo3	0	0	-1000	-1000	-60.455641	-1000	0	-1000	0
Klebsiella_oxytoca_KCTC_1686	-1000	0	-1000	-1000	-60.293499	-1000	0	-1000	0
Klebsiella_oxytoca_MGH88	0	0	-1000	-1000	-41.248152	-1000	0	-1000	0
Klebsiella_pneumoniae_1162281	0	-1000	-1000	-1000	-68.13572	-1000	0	-1000	0
Klebsiella_pneumoniae_1191100241	0	-1000	-1000	-1000	-62.889934	-1000	0	-1000	0
Klebsiella_pneumoniae_342	0	0	-1000	-1000	-58.634312	-1000	0	0	0
Klebsiella_pneumoniae_361_1301	0	-1000	-1000	-1000	-68.287784	-1000	0	-1000	0
Klebsiella_pneumoniae_500_1420	0	-1000	-1000	-1000	-68.287784	-1000	0	-1000	0
Klebsiella_pneumoniae_540_1460	0	-1000	-1000	-1000	-68.278742	-1000	0	-1000	0
Klebsiella_pneumoniae_646_1568	0	-1000	-1000	-1000	-68.278641	-1000	0	-1000	0
Klebsiella_pneumoniae_ATCC_25955	0	-1000	-1000	-1000	-68.287742	-1000	0	-1000	0
Klebsiella_pneumoniae_ATCC_BAA_1705	0	-1000	-1000	-1000	-68.278742	-1000	0	-1000	0
Klebsiella_pneumoniae_ATCC_BAA_2146	0	-1000	-1000	-1000	-68.278775	-1000	0	-1000	0

Klebsiella_pneumoniae_CHS153	0	-1000	-1000	-1000	-49.771413	-1000	0	-1000	0
Klebsiella_pneumoniae_CHS222	0	-1000	-1000	-1000	-49.77141	-1000	0	-1000	0
Klebsiella_pneumoniae_DMC0526	0	-1000	-1000	-1000	-68.278742	-1000	0	-1000	0
Klebsiella_pneumoniae_ERR2221115	0	-1000	-1000	-1000	-49.771474	-1000	0	-1000	0
Klebsiella_pneumoniae_ERR2221116	0	-1000	-1000	-1000	-49.771635	-1000	0	-1000	0
Klebsiella_pneumoniae_ERR2221118	0	-1000	-1000	-1000	-49.771426	-1000	0	-1000	0
Klebsiella_pneumoniae_ERR2221121	0	-1000	-1000	-1000	-49.771439	-1000	0	-1000	0
Klebsiella_pneumoniae_ERR2221297	0	-1000	-1000	-1000	-49.549948	-1000	0	-1000	0
Klebsiella_pneumoniae_ERR2221368	0	-1000	-1000	-1000	-49.7716	-1000	0	-1000	0
Klebsiella_pneumoniae_ERR2221372	0	-1000	-1000	-1000	-49.7716	-1000	0	-1000	0
Klebsiella_pneumoniae_G5_2	0	-1000	-1000	-1000	-68.287742	-1000	0	-1000	0
Klebsiella_pneumoniae_hvKP1	0	-1000	-1000	-1000	-68.278674	-1000	0	-1000	0
Klebsiella_pneumoniae_JH1	0	-1000	-1000	-1000	-68.288479	-1000	0	-1000	0
Klebsiella_pneumoniae_KCTC_2242	-1000	-1000	-1000	-1000	-68.287784	-1000	0	-1000	0
Klebsiella_pneumoniae_KP_11	0	-1000	-1000	-1000	-68.283627	-1000	0	-1000	0
Klebsiella_pneumoniae_NTUH_K2044	-1000	-1000	-1000	-1000	-68.288411	-1000	0	-1000	0
Klebsiella_pneumoniae_pneumoniae_MGH78578	-1000	-1000	-1000	-1000	-44.573767	-1000	0	-1000	0
Klebsiella_pneumoniae_PR04	0	-1000	-1000	-1000	-68.446015	-1000	0	-1000	0
Klebsiella_pneumoniae_subsp_pneumoniae_1084	0	-1000	-1000	-1000	-68.287742	-1000	0	-1000	0
Klebsiella_pneumoniae_subsp_pneumoniae_12_	0	-1000	-1000	-1000	-68.019466	-1000	0	-1000	0
Klebsiella_pneumoniae_subsp_pneumoniae_DSM	0	-1000	-1000	-1000	-67.963278	-1000	0	-1000	0
Klebsiella_pneumoniae_subsp_pneumoniae_Ecl8	0	-1000	-1000	-1000	-68.288378	-1000	0	-1000	0
Klebsiella_pneumoniae_subsp_pneumoniae_HS1	0	-1000	-1000	-1000	-68.377288	-1000	0	-1000	0
Klebsiella_pneumoniae_subsp_pneumoniae_KPN	0	-1000	-1000	-1000	-68.278708	-1000	0	-1000	0
Klebsiella_pneumoniae_subsp_pneumoniae_KPN	0	-1000	-1000	-1000	-68.278708	-1000	0	-1000	0
Klebsiella_pneumoniae_subsp_pneumoniae_KPN	0	-1000	-1000	-1000	-68.278708	-1000	0	-1000	0
Klebsiella_pneumoniae_subsp_pneumoniae_KPN	0	-1000	-1000	-1000	-68.318185	-1000	0	-1000	0
Klebsiella_pneumoniae_subsp_pneumoniae_KPN	0	-1000	-1000	-1000	-68.278056	-1000	0	-1000	0
Klebsiella_pneumoniae_subsp_pneumoniae_KPN	0	-1000	-1000	-1000	-68.278708	-1000	0	-1000	0
Klebsiella_pneumoniae_subsp_pneumoniae_KPN	0	-1000	-1000	-1000	-68.278708	-1000	0	-1000	0
Klebsiella_pneumoniae_subsp_pneumoniae_KPN	0	-1000	-1000	-1000	-68.318185	-1000	0	-1000	0
Klebsiella_pneumoniae_subsp_pneumoniae_KPN	0	-1000	-1000	-1000	-68.278708	-1000	0	-1000	0
Klebsiella_pneumoniae_subsp_pneumoniae_KPN	0	-1000	-1000	-1000	-68.278708	-1000	0	-1000	0
Klebsiella_pneumoniae_subsp_pneumoniae_KPN	0	-1000	-1000	-1000	-68.278708	-1000	0	-1000	0
Klebsiella_pneumoniae_subsp_pneumoniae_KPN	0	-1000	-1000	-1000	-68.287826	-1000	0	-1000	0
Klebsiella_pneumoniae_subsp_pneumoniae_KPN	0	-1000	-1000	-1000	-68.278708	-1000	0	-1000	0
Klebsiella_pneumoniae_subsp_pneumoniae_KPN	0	-1000	-1000	-1000	-68.278708	-1000	0	-1000	0
Klebsiella_pneumoniae_subsp_pneumoniae_KPN	0	-1000	-1000	-1000	-68.278708	-1000	0	-1000	0
Klebsiella_pneumoniae_subsp_pneumoniae_KPN	0	-1000	-1000	-1000	-68.278708	-1000	0	-1000	0
Klebsiella_pneumoniae_subsp_pneumoniae_KPN	0	-1000	-1000	-1000	-68.278708	-1000	0	-1000	0
Klebsiella_pneumoniae_subsp_pneumoniae_KPN	0	-1000	-1000	-1000	-68.278708	-1000	0	-1000	0
Klebsiella_pneumoniae_subsp_pneumoniae_KPN	0	-1000	-1000	-1000	-68.287826	-1000	0	-1000	0
Klebsiella_pneumoniae_subsp_pneumoniae_KPN	0	-1000	-1000	-1000	-68.278708	-1000	0	-1000	0
Klebsiella_pneumoniae_subsp_pneumoniae_KpQ	0	-1000	-1000	-1000	-68.278476	-1000	0	-1000	0
Klebsiella_pneumoniae_subsp_pneumoniae_LZ	0	-1000	-1000	-1000	-67.963236	-1000	0	-1000	0
Klebsiella_pneumoniae_subsp_pneumoniae_ST2	0	-1000	-1000	-1000	-68.278809	-1000	0	-1000	0
Klebsiella_pneumoniae_subsp_pneumoniae_ST2	0	-1000	-1000	-1000	-64.274945	-1000	0	-1000	0
Klebsiella_pneumoniae_subsp_pneumoniae_ST2	0	-1000	-1000	-1000	-68.282983	-1000	0	-1000	0
Klebsiella_pneumoniae_subsp_pneumoniae_ST5	0	-1000	-1000	-1000	-59.063463	-1000	0	-1000	0
Klebsiella_pneumoniae_subsp_pneumoniae_WG	0	-1000	-1000	-1000	-68.2877	-1000	0	-1000	0
Klebsiella_pneumoniae_subsp_pneumoniae_WG	0	-1000	-1000	-1000	-68.28791	-1000	0	-1000	0
Klebsiella_pneumoniae_subsp_pneumoniae_WG	0	-1000	-1000	-1000	-68.28791	-1000	0	-1000	0
Klebsiella_pneumoniae_subsp_rhinoscleromatis_	0	-1000	-1000	-1000	-67.635454	-1000	0	-1000	0
Klebsiella_pneumoniae_UHKPC_52	0	-1000	-1000	-1000	-68.287784	-1000	0	-1000	0
Klebsiella_pneumoniae_UHKPC01	0	-1000	-1000	-1000	-68.278708	-1000	0	-1000	0
Klebsiella_pneumoniae_UHKPC04	0	-1000	-1000	-1000	-68.278742	-1000	0	-1000	0
Klebsiella_pneumoniae_UHKPC05	0	-1000	-1000	-1000	-68.278708	-1000	0	-1000	0
Klebsiella_pneumoniae_UHKPC09	0	-1000	-1000	-1000	-68.278708	-1000	0	-1000	0
Klebsiella_pneumoniae_UHKPC22	0	-1000	-1000	-1000	-68.278742	-1000	0	-1000	0
Klebsiella_pneumoniae_UHKPC23	0	-1000	-1000	-1000	-68.278708	-1000	0	-1000	0
Klebsiella_pneumoniae_UHKPC24	0	-1000	-1000	-1000	-68.287784	-1000	0	-1000	0
Klebsiella_pneumoniae_UHKPC26	0	-1000	-1000	-1000	-68.278708	-1000	0	-1000	0
Klebsiella_pneumoniae_UHKPC27	0	-1000	-1000	-1000	-68.278708	-1000	0	-1000	0
Klebsiella_pneumoniae_UHKPC29	0	-1000	-1000	-1000	-68.287826	-1000	0	-1000	0
Klebsiella_pneumoniae_UHKPC32	0	-1000	-1000	-1000	-68.278742	-1000	0	-1000	0
Klebsiella_pneumoniae_UHKPC40	0	-1000	-1000	-1000	-68.278708	-1000	0	-1000	0
Klebsiella_pneumoniae_UHKPC45	0	-1000	-1000	-1000	-68.00496	-1000	0	-1000	0
Klebsiella_pneumoniae_UHKPC48	0	-1000	-1000	-1000	-68.278742	-1000	0	-1000	0
Klebsiella_pneumoniae_UHKPC57	0	-1000	-1000	-1000	-68.28858	-1000	0	-1000	0
Klebsiella_pneumoniae_UHKPC81	0	-1000	-1000	-1000	-68.288512	-1000	0	-1000	0
Klebsiella_pneumoniae_VAKPC252	0	-1000	-1000	-1000	-68.287826	-1000	0	-1000	0
Klebsiella_pneumoniae_VAKPC254	0	-1000	-1000	-1000	-68.453463	-1000	0	-1000	0
Klebsiella_pneumoniae_VAKPC269	0	-1000	-1000	-1000	-68.278674	-1000	0	-1000	0
Klebsiella_pneumoniae_VAKPC270	0	-1000	-1000	-1000	-68.278708	-1000	0	-1000	0
Klebsiella_pneumoniae_VAKPC276	0	-1000	-1000	-1000	-68.287826	-1000	0	-1000	0
Klebsiella_pneumoniae_VAKPC278	0	-1000	-1000	-1000	-68.273824	-1000	0	-1000	0
Klebsiella_pneumoniae_VAKPC280	0	-1000	-1000	-1000	-68.287784	-1000	0	-1000	0
Klebsiella_pneumoniae_VAKPC297	0	-1000	-1000	-1000	-68.278742	-1000	0	-1000	0
Klebsiella_pneumoniae_VAKPC309	0	-1000	-1000	-1000	-68.278708	-1000	0	-1000	0
Klebsiella_quasipneumoniae_700603	0	-1000	-1000	-1000	-58.580899	-1000	0	-1000	0
Klebsiella_sp_4_1_44FAA	0	0	-1000	-1000	-39.586313	-1000	0	-1000	0
Klebsiella_sp_M5al	0	0	-1000	-1000	-41.248158	-1000	0	-1000	0
Klebsiella_sp_MS_92_3	0	0	-1000	-1000	-39.421785	-1000	0	-1000	0
Klebsiella_sp_OBRC7	0	0	-1000	-1000	-41.251012	-1000	0	-1000	0
Klebsiella_variicola_1_1_55	0	0	-1000	-1000	-44.021756	-1000	0	-1000	0
Klebsiella_variicola_KV321_ZB100000	0	0	-1000	-1000	-49.46576	-1000	0	-1000	0
Kluyvera_ascorbata_ATCC_33433	0	0	-1000	-1000	-58.592612	-1000	0	-1000	0
Kluyvera_cryocrescens_L2	0	0	-1000	-1000	-34.282719	-1000	0	-1000	0
Kluyvera_cryocrescens_NBRC_102467	0	0	-1000	-1000	-34.339417	-1000	0	-1000	0
Kluyvera_georgiana_ATCC_51603	0	0	-1000	-1000	-29.512142	-1000	0	-1000	0
Kluyvera_intermedia_FOSA7093	0	0	-1000	-1000	-33.48388	-1000	0	-1000	0

Kluyvera_intermedia_NBRC_102594_ATCC_3311(	0	0	-1000	-1000	-34.282663	-1000	0	-1000	0
Kocuria_palustris_CD07_3	0	0	0	-1000	-21.810381	-1000	0	-666.66667	0
Kocuria_palustris_PEL	0	0	0	-1000	-33.128799	-1000	0	-1000	0
Kocuria_rhizophila_DC2201	0	0	0	-1000	0	0	0	-1000	0
Kocuria_rhizophila_P7_4	0	0	0	0	0	0	0	-1000	0
Kosakonia_sacchari_SP1	0	0	-1000	-1000	-42.008719	-1000	0	-1000	0
Kurthia_massiliensis_JC30	0	0	0	-1000	-1000	0	0	0	0
Kurthia_senegalensis_JC8E	0	0	0	-1000	-15.14613	0	0	0	0
Kytococcus_sedentarius_1083_KSED	0	0	0	-41.3826873	-34.877996	0	0	0	0
Kytococcus_sedentarius_262_KSED	0	0	0	-41.38276135	-23.990257	0	0	0	0
Kytococcus_sedentarius_DSM_20547	0	0	0	-21.19446024	-16.01721	0	0	0	0
Lachnoanaerobaculum_orale_DSM_24553	0	0	0	-53.14830373	-27.279287	0	0	-477.35192	0
Lachnoanaerobaculum_saburreum_DNF00896	0	0	0	-56.2630908	-27.81985	0	0	-483.14607	0
Lachnoanaerobaculum_saburreum_DSM_3986	0	0	0	-53.05331921	-27.324826	0	0	-500	0
Lachnoanaerobaculum_saburreum_F0468	0	0	0	-52.33656	-26.955663	0	0	-500	0
Lachnobacterium_bovis_AE2004	0	0	0	-46.43356993	-23.793183	0	0	-390.59933	0
Lachnobacterium_bovis_C6A12	0	0	0	-43.32873705	-22.198891	0	0	-449.5114	0
Lachnobacterium_bovis_DSM_14045	0	0	0	-47.94302726	-24.649856	0	0	-458.47111	0
Lachnobacterium_bovis_NK4B19	0	0	0	-31.8871779	-16.159594	0	0	-394.44362	0
Lachnobacterium_bovis_S1b	0	0	0	-43.32889191	-22.198971	0	0	-449.5114	0
Lachnoclostridium_nov_ERR1022273	0	0	0	-43.64184094	-22.531621	0	0	0	0
Lachnoclostridium_nov_ERR1022276	0	0	0	-1000	-17.21028	-541.42993	0	0	0
Lachnoclostridium_nov_ERR1022305	0	0	0	0	-16.256487	0	0	-666.66667	0
Lachnoclostridium_nov_ERR1022387	0	0	0	-46.0728777	-23.686102	0	0	-756.2724	0
Lachnoclostridium_nov_ERR1022388	0	0	0	-1000	-21.243476	-833.33333	0	0	0
Lachnoclostridium_nov_ERR1022392	0	0	0	-53.96565729	-27.221482	0	0	-791.66667	0
Lachnoclostridium_nov_ERR1022405	0	0	0	-42.68778977	-22.112181	0	0	0	0
Lachnoclostridium_nov_ERR1022430	0	0	0	-75.50474084	-33.74987	0	0	-1000	0
Lachnoclostridium_nov_ERR1022434	0	0	0	-41.8176602	-19.584243	0	0	0	0
Lachnoclostridium_nov_ERR1203939	0	0	0	-1000	-26.644389	0	0	-750	0
Lachnoclostridium_nov_ERR1203968	0	0	0	-31.34918782	-21.286361	0	0	0	0
Lachnoclostridium_nov_ERR1204033	0	0	0	-1000	-26.644389	0	0	-750	0
Lachnoclostridium_nov_ERR1204062	0	0	0	-31.34918586	-21.28636	0	0	0	0
Lachnoclostridium_nov_ERR171262	0	0	0	-60.38851577	-27.061085	0	0	-764.25856	0
Lachnoclostridium_nov_ERR171273	0	0	0	-52.94860636	-27.370481	0	0	0	0
Lachnoclostridium_nov_ERR171281	0	0	0	-1000	-37.009238	-1000	0	0	0
Lachnoclostridium_nov_ERR2221145	0	0	0	-60.38931023	-27.217994	0	0	-770.83333	0
Lachnoclostridium_nov_ERR2221158	0	0	0	-1000	-27.687832	0	0	-791.66667	0
Lachnoclostridium_nov_ERR2221159	0	0	0	-1000	-27.669087	0	0	-791.66667	0
Lachnoclostridium_nov_ERR2230065	0	0	0	-45.30080534	-23.342961	0	0	-933.71212	0
Lachnoclostridium_nov_ERR2230076	0	0	0	-50.45673979	-25.793324	0	0	-501.55672	0
Lachnoclostridium_nov_ERR2230092	0	0	0	-51.88204421	-26.674878	0	0	-791.66667	0
Lachnoclostridium_nov_ERR2230109	0	0	0	-1000	-21.546914	-552.58104	0	0	0
Lachnoclostridium_nov_ERR2230115	0	0	0	-43.18544556	-22.294939	0	0	0	0
Lachnoclostridium_nov_ERR2230132	0	0	0	-1000	-24.146222	0	0	0	0
Lachnoclostridium_nov_ERR2230151	0	0	0	-1000	-24.146217	0	0	0	0
Lachnoclostridium_sp_YL32	0	0	0	-1000	-27.556567	0	0	-752.96328	0
Lachnospira_multipara_ATCC_19207	0	0	0	-1000	0	0	0	-879.31034	0
Lachnospira_multipara_D15d	0	0	0	-31.72347942	-15.997912	0	0	-390.83115	0
Lachnospira_multipara_LB2003	0	0	0	-32.17641928	-16.781332	0	0	-416.30148	0
Lachnospira_multipara_MC2003	0	0	0	-32.19820254	-16.793033	0	0	-498.30146	0
Lachnospira_nov_ERR1022391	0	0	0	-42.84111489	-22.031901	0	0	0	0
Lachnospira_nov_ERR1022394	0	0	0	-36.80227409	-18.888437	0	0	0	0
Lachnospira_nov_ERR1203977	0	0	0	-36.69805002	-18.835132	0	0	0	0
Lachnospira_nov_ERR1204071	0	0	0	-36.69804174	-18.835128	0	0	0	0
Lachnospira_pectinoschiza_ERR1022352	0	0	0	-35.37385698	-18.095335	0	0	0	0
Lachnospira_pectinoschiza_ERR1022401	0	0	0	-1000	-18.728206	0	0	0	0
Lachnospira_pectinoschiza_M83	0	0	0	-32.08753797	-16.771252	0	0	-528.75593	0
Lachnospiraceae_bacterium_1_1_57FAA	0	0	0	-1000	-22.882361	0	0	0	0
Lachnospiraceae_bacterium_1_4_56FAA	0	0	0	-36.4838707	-18.835757	0	0	0	0
Lachnospiraceae_bacterium_2_1_46FAA	0	0	0	0	-12.688737	0	0	0	0
Lachnospiraceae_bacterium_2_1_58FAA	0	0	-1000	-48.7203733	-25.093165	0	0	-614.94253	0
Lachnospiraceae_bacterium_3_1_46FAA	0	0	0	-34.69770289	-17.881127	0	0	0	0
Lachnospiraceae_bacterium_3_1_57FAA_CT1	0	0	0	0	-37.331352	0	0	-1000	0
Lachnospiraceae_bacterium_5_1_57FAA	0	0	0	-53.12591453	-27.462472	0	0	0	0
Lachnospiraceae_bacterium_6_1_63FAA	0	0	0	-24.37905244	-15.463835	0	0	-690.97222	0
Lachnospiraceae_bacterium_7_1_58FAA	0	0	0	-1000	-17.639576	0	0	0	0
Lachnospiraceae_bacterium_9_1_43BFAA	0	0	0	0	-16.383376	0	0	0	0
Lachnospiraceae_bacterium_oral_taxon_082_str_	0	0	0	-26.13908032	-18.503361	0	0	-133.33333	0
Lachnospiraceae_bacterium_sp_5_1_63FAA	0	0	0	-1000	-10.440916	-187.5	0	-100	0
Lachnospiraceae_bacterium_sp_8_1_57FAA	0	0	0	-1000	-9.1254944	0	0	0	0
Lachnospiraceae_nov_ERR1022272	0	0	0	-1000	-13.195947	0	0	0	0
Lachnospiraceae_nov_ERR1022274	0	0	0	0	-23.873611	0	0	0	0
Lachnospiraceae_nov_ERR1022277	0	0	0	-31.24831027	-16.04154	0	0	0	0
Lachnospiraceae_nov_ERR1022289	0	0	0	-26.59646517	-13.554885	0	0	-597.22222	0
Lachnospiraceae_nov_ERR1022303	0	0	0	-22.12124729	-11.391764	-542.31608	0	0	0
Lachnospiraceae_nov_ERR1022308	0	0	0	-67.08735817	-34.251471	0	0	-1000	0
Lachnospiraceae_nov_ERR1022341	0	0	0	-36.88820218	-18.906519	0	0	0	0
Lachnospiraceae_nov_ERR1022342	0	0	0	0	-17.687599	0	0	-497.2067	0
Lachnospiraceae_nov_ERR1022343	0	0	0	0	-15.779472	0	0	-614.58333	0
Lachnospiraceae_nov_ERR1022351	0	0	0	-22.38530435	-11.527732	-540.75907	0	0	0
Lachnospiraceae_nov_ERR1022353	0	0	0	-1000	-39.297918	0	0	-833.92088	0
Lachnospiraceae_nov_ERR1022380	0	0	0	-51.94234414	-24.64491	0	0	-786.88525	0
Lachnospiraceae_nov_ERR1022381	0	0	0	-1000	-27.551957	0	0	-752.96627	0
Lachnospiraceae_nov_ERR1022386	0	0	0	-34.25667032	-22.103648	0	0	0	0
Lachnospiraceae_nov_ERR1022389	0	0	0	-1000	-21.45057	0	0	-517.85714	0
Lachnospiraceae_nov_ERR1022390	0	0	0	0	-13.372302	0	0	-505.19797	0
Lachnospiraceae_nov_ERR1022398	0	0	0	-38.05694403	-19.587278	-568.7467	0	0	0
Lachnospiraceae_nov_ERR1022402	0	0	0	0	-27.33776	0	0	-417.06698	0
Lachnospiraceae_nov_ERR1022429	0	0	0	-52.42165616	-24.864343	0	0	-786.88525	0
Lachnospiraceae_nov_ERR1022431	0	0	0	-45.81259578	-21.505876	0	0	-500	0

Lachnospiraceae_nov_ERR1022433	0	0	0	-46.61621706	-21.710564	0	0	-723.52941	0
Lachnospiraceae_nov_ERR1022436	0	0	0	-46.34638186	-23.806495	0	0	-500.71278	0
Lachnospiraceae_nov_ERR1022478	0	0	0	-20.51499122	-10.51219	-536.34181	0	0	0
Lachnospiraceae_nov_ERR1022480	0	0	0	-38.05684344	-19.587183	-568.74642	0	0	0
Lachnospiraceae_nov_ERR1022481	0	0	0	0	-25.294544	0	0	-431.77104	0
Lachnospiraceae_nov_ERR1203950	0	0	0	-45.75384956	-21.478227	0	0	-500	0
Lachnospiraceae_nov_ERR1203952	0	0	0	-43.14830584	-22.110897	0	0	-500	0
Lachnospiraceae_nov_ERR1203964	0	0	0	-36.64067019	-18.86666	-667.80837	0	0	0
Lachnospiraceae_nov_ERR1203967	0	0	0	-29.40645302	-12.993622	0	0	0	0
Lachnospiraceae_nov_ERR1203970	0	0	0	-36.64067047	-18.86666	-667.80837	0	0	0
Lachnospiraceae_nov_ERR1203972	0	0	0	-25.72718358	-13.246239	0	0	0	0
Lachnospiraceae_nov_ERR1204044	0	0	0	-45.75384934	-21.478227	0	0	-500	0
Lachnospiraceae_nov_ERR1204046	0	0	0	-43.14830455	-22.110897	0	0	-500	0
Lachnospiraceae_nov_ERR1204058	0	0	0	-36.64066141	-18.866655	-667.80838	0	0	0
Lachnospiraceae_nov_ERR1204061	0	0	0	-29.40645302	-12.993622	0	0	0	0
Lachnospiraceae_nov_ERR1204064	0	0	0	-36.64067047	-18.86666	-667.80837	0	0	0
Lachnospiraceae_nov_ERR1204066	0	0	0	-25.72718364	-13.246239	0	0	0	0
Lachnospiraceae_nov_ERR171267	0	0	0	-53.93198031	-24.107065	0	0	-1000	0
Lachnospiraceae_nov_ERR171274	0	0	0	-42.77047521	-20.344206	0	0	-704.91803	0
Lachnospiraceae_nov_ERR171283	0	0	0	0	-26.547514	0	0	-602.17289	0
Lachnospiraceae_nov_ERR2221097	0	0	0	-21.4381346	-10.970296	-538.90936	0	0	0
Lachnospiraceae_nov_ERR2221111	0	0	0	-1000	-27.045233	0	0	-805.55556	0
Lachnospiraceae_nov_ERR2221148	0	0	0	-1000	-19.065348	-1000	0	0	0
Lachnospiraceae_nov_ERR2221151	0	0	0	-52.42345642	-24.865204	0	0	-786.88525	0
Lachnospiraceae_nov_ERR2221161	0	0	0	-1000	-14.18648	0	0	0	0
Lachnospiraceae_nov_ERR2221181	0	0	0	-21.43813563	-10.970297	-538.90936	0	0	0
Lachnospiraceae_nov_ERR2221183	0	0	0	-1000	-27.55211	0	0	-752.96628	0
Lachnospiraceae_nov_ERR2221187	0	0	0	-37.61489009	-19.36006	-571.36055	0	0	0
Lachnospiraceae_nov_ERR2221212	0	0	0	-1000	-27.55648	0	0	-752.96328	0
Lachnospiraceae_nov_ERR2221263	0	0	0	-25.57093315	-13.162856	0	0	0	0
Lachnospiraceae_nov_ERR2221266	0	0	0	-25.57093364	-13.162856	0	0	0	0
Lachnospiraceae_nov_ERR2221278	0	0	0	0	-23.250468	-1000	0	-747.25275	0
Lachnospiraceae_nov_ERR2221285	0	0	0	-67.08722679	-34.251368	0	0	-1000	0
Lachnospiraceae_nov_ERR2221344	0	0	0	0	-24.346731	0	0	-833.33333	0
Lachnospiraceae_nov_ERR2221369	0	0	0	-1000	-31.890471	0	0	-658.17202	0
Lachnospiraceae_nov_ERR2221389	0	0	0	-1000	-27.823912	0	0	-722.22222	0
Lachnospiraceae_nov_ERR2230050	0	0	0	-1000	-27.559052	0	0	-752.96141	0
Lachnospiraceae_nov_ERR2230056	0	0	0	-22.50840526	-10.932722	0	0	0	0
Lachnospiraceae_nov_ERR2230057	0	0	0	0	-26.392074	0	0	-281.78659	0
Lachnospiraceae_nov_ERR2230058	0	0	0	-25.57093315	-13.162856	0	0	0	0
Lachnospiraceae_nov_ERR2230083	0	0	0	-34.97696603	-17.324829	0	0	-260.44094	0
Lachnospiraceae_nov_ERR2230113	0	0	0	-1000	-39.349274	0	0	-833.92088	0
Lachnospiraceae_nov_ERR2230114	0	0	0	-1000	-14.186469	0	0	0	0
Lachnospiraceae_nov_ERR2230144	0	0	0	-30.50313162	-19.48385	0	0	0	0
Lachnospiraceae_nov_ERR2230148	0	0	0	-44.47246087	-21.017074	0	0	-672.57513	0
Lachnospiraceae_oral_taxon_107_str_F0167	0	0	0	-37.76438682	-26.86053	0	0	-426.57343	0
Lactobacillus_acidipiscis_ACA_DC_1533	0	0	0	-1000	-17.183611	-1000	0	0	0
Lactobacillus_acidipiscis_DSM_15836	0	0	0	-1000	-17.18178	-1000	0	0	0
Lactobacillus_acidipiscis_KCTC_13900	0	0	0	-1000	-15.445323	0	0	0	0
Lactobacillus_acidophilus_ATCC_4796	0	0	0	0	-21.479918	0	0	-1000	0
Lactobacillus_acidophilus_La_14	0	0	0	-300	-29.215597	0	0	-1000	0
Lactobacillus_acidophilus_NCFM	0	0	0	-480	-173.30765	0	0	-1000	0
Lactobacillus_agilis_DSM_20509	0	0	0	-1000	-18.52988	-1000	0	-524.74932	0
Lactobacillus_agilis_UMNLA4	0	0	0	-1000	-18.550662	-1000	0	-805.06683	0
Lactobacillus_algidus_CMTALT10	0	0	0	-18.81335975	-15.46591	0	0	-713.2212	0
Lactobacillus_algidus_DSM_15638	0	0	0	-18.81335975	-15.46591	0	0	0	0
Lactobacillus_amylolyticus_DSM_11664	0	0	0	0	-19.032519	0	0	-505.8842	0
Lactobacillus_amylovorus_30SC	0	0	0	0	-25.016016	-1000	0	-808.04954	0
Lactobacillus_amylovorus_GRL_1112	0	0	0	-480	-26.36948	-1000	0	-875	0
Lactobacillus_amylovorus_GRL1118	0	0	0	0	-27.903965	0	0	-808.04954	0
Lactobacillus_animalis_ERR2221125	0	0	0	-39.24356132	-27.779776	-1000	0	-750	0
Lactobacillus_animalis_ERR2221280	0	0	0	-40.42202695	-27.11796	0	0	-750	0
Lactobacillus_animalis_ERR2221327	0	0	0	-40.46879856	-27.146015	0	0	-750	0
Lactobacillus_animalis_KCTC_3501	0	0	0	-42.09938118	-25.752695	0	0	-1000	0
Lactobacillus_animalis_NP51	0	0	0	-26.09361011	-16.128915	0	0	-755.62911	0
Lactobacillus_antri_DSM_16041	0	0	0	-1000	-32.388258	0	0	-1000	0
Lactobacillus_apis_LMG_26964	0	0	0	0	-14.944953	0	0	-428.54426	0
Lactobacillus_apodemi_DSM_16634_JCM_16172	0	0	0	-1000	-20.007428	0	0	-750	0
Lactobacillus_brevis_ATCC_367	0	0	-1000	-1000	-26.352999	0	0	0	0
Lactobacillus_brevis_KB290	0	0	-1000	-1000	-42.689052	0	0	0	0
Lactobacillus_brevis_subsp_gravesensis_ATCC_27	0	0	-1000	-1000	-25.039438	0	0	0	0
Lactobacillus_buchneri_ATCC_11577	0	0	0	-1000	-24.890005	0	0	0	0
Lactobacillus_buchneri_CD034	0	0	-1000	-1000	-44.853064	0	0	0	0
Lactobacillus_buchneri_NRRL_B_30929	0	0	-1000	-1000	-44.839543	0	0	0	0
Lactobacillus_camelliae_DSM_22697_JCM_13995	0	0	0	0	-7.1422285	-416.66667	0	0	0
Lactobacillus_casei_12A	0	0	0	-1000	-24.025263	-1000	0	-1000	0
Lactobacillus_casei_21_1	0	0	0	-1000	-23.929044	-1000	0	-1000	0
Lactobacillus_casei_A2_362	0	0	0	0	-23.18738	-1000	0	-1000	0
Lactobacillus_casei_ATCC_334	0	0	0	-1000	-25.289281	-1000	0	-1000	0
Lactobacillus_casei_casei_BL23	0	0	0	-1000	-25.606765	-1000	0	-1000	0
Lactobacillus_casei_ERR2221237	0	0	0	0	-22.849346	-1000	0	-1000	0
Lactobacillus_casei_ERR2221291	0	0	0	0	-22.649833	-1000	0	-1000	0
Lactobacillus_casei_ERR2221303	0	0	0	0	-22.840641	-1000	0	-1000	0
Lactobacillus_casei_ERR2221304	0	0	0	0	-22.988092	-1000	0	-1000	0
Lactobacillus_casei_ERR2221325	0	0	0	0	-22.649827	-1000	0	-1000	0
Lactobacillus_casei_UW4	0	0	0	0	-23.806405	-1000	0	-1000	0
Lactobacillus_casei_W56	0	0	0	-1000	-24.030081	-1000	0	-1000	0
Lactobacillus_coleohominis_101_4_CHN	0	0	0	-1000	-23.353024	0	0	-756.67336	0
Lactobacillus_coryniformis_subsp_coryniformis_(	0	0	0	-49.30378161	-21.723139	-1000	0	-1000	0
Lactobacillus_coryniformis_subsp_coryniformis_K	0	0	0	-46.92721744	-22.018249	-1000	0	-1000	0
Lactobacillus_crispatus_125_2_CHN	0	0	0	0	-22.290861	-1000	0	-600	0

Lactobacillus_crispatus_214_1	0	0	0	0	-16.946181	-1000	0	-625.88595	0
Lactobacillus_crispatus_CTV_05	0	0	0	0	-21.355806	-1000	0	-823.52941	0
Lactobacillus_crispatus_FB049_03	0	0	0	0	-25.193993	-1000	0	-765.7231	0
Lactobacillus_crispatus_FB077_07	0	0	0	0	-25.254348	-1000	0	-765.72281	0
Lactobacillus_crispatus_JV_V01	0	0	0	0	-25.361809	-1000	0	-650.73263	0
Lactobacillus_crispatus_MV_1A_US	0	0	0	0	-25.363978	-1000	0	-650.74045	0
Lactobacillus_crispatus_MV_3A_US	0	0	0	0	-25.053057	-1000	0	-558.85444	0
Lactobacillus_crispatus_ST1	0	0	0	0	-25.364137	-1000	0	-762.70973	0
Lactobacillus_curvatus_CRL_705	0	0	0	-19.43304917	-15.95189	0	0	-850	0
Lactobacillus_curvatus_ERR2221163	0	0	0	-19.47180405	-16.805582	0	0	-584.41558	0
Lactobacillus_curvatus_JCM_1096_DSM_20019	0	0	0	-18.70817509	-15.149805	0	0	-522.34892	0
Lactobacillus_delbrueckii_subsp_bulgaricus_2038	0	0	0	-1000	-27.574991	-1000	0	0	0
Lactobacillus_delbrueckii_subsp_bulgaricus_ATC(	0	0	0	-1000	-168.12921	-1000	0	0	0
Lactobacillus_delbrueckii_subsp_bulgaricus_ATC(	0	0	0	-1000	-23.899002	0	0	0	0
Lactobacillus_delbrueckii_subsp_bulgaricus_CNCI	0	0	0	-1000	-22.589905	-1000	0	0	0
Lactobacillus_delbrueckii_subsp_bulgaricus_CNCI	0	0	0	-1000	-26.793131	-1000	0	0	0
Lactobacillus_delbrueckii_subsp_bulgaricus_ND0	0	0	0	-1000	-34.736244	-1000	0	0	0
Lactobacillus_delbrueckii_subsp_delbrueckii_DSM	0	0	0	-26.85017219	-17.139511	0	0	-523.71637	0
Lactobacillus_delbrueckii_ZN7a_9	0	0	0	-1000	-22.167665	0	0	0	0
Lactobacillus_dextrinicus_DSM_20335	0	0	0	-19.72965562	-16.252793	0	0	-510.33496	0
Lactobacillus_equicursoris_66c	0	0	0	0	-29.510589	-1000	0	0	0
Lactobacillus_equicursoris_DSM_19284	0	0	0	0	-30.658057	-1000	0	0	0
Lactobacillus_farciminis_KCTC_3681_DSM_20184	0	0	0	-1000	-39.362738	0	0	-666.66667	0
Lactobacillus_fermentum_28_3_CHN	0	0	0	-1000	-40.429755	0	0	-1000	0
Lactobacillus_fermentum_ATCC_14931	0	0	0	-333.3333333	-25.96734	0	0	-980.76923	0
Lactobacillus_fermentum_CECT_5716	0	0	0	-1000	-32.977286	0	0	-945.05495	0
Lactobacillus_fermentum_F_6	0	0	0	-1000	-40.14183	0	0	-1000	0
Lactobacillus_fermentum_IFO_3956	0	0	0	-333.3333333	-176.06042	0	0	-500	0
Lactobacillus_florum_2F	0	0	0	0	-16.227561	0	0	0	0
Lactobacillus_fructivorans_KCTC_3543_DSM_202	0	0	0	-31.04197228	-28.984299	0	0	0	0
Lactobacillus_frumenti_DSM_13145	0	0	0	-15.63351014	-13.295757	0	0	-508.69214	0
Lactobacillus_gasseri_202_4	0	0	0	-1000	-28.273694	0	0	-1000	0
Lactobacillus_gasseri_224_1	0	0	0	-1000	-19.528813	0	0	-1000	0
Lactobacillus_gasseri_ATCC_33323	0	0	0	0	-181.13083	0	0	-1000	0
Lactobacillus_gasseri_CECT_5714	0	0	0	-433.3333333	-28.711443	0	0	-1000	0
Lactobacillus_gasseri_ERR1203930	0	0	0	-411.7647059	-27.22282	0	0	-1000	0
Lactobacillus_gasseri_ERR1204024	0	0	0	-411.7647059	-27.22282	0	0	-1000	0
Lactobacillus_gasseri_ERR2221164	0	0	0	-416.6666667	-27.911136	0	0	-1000	0
Lactobacillus_gasseri_ERR2221406	0	0	0	-416.6666667	-27.91113	0	0	-1000	0
Lactobacillus_gasseri_JV_V03	0	0	0	-1000	-28.294436	0	0	-838.70968	0
Lactobacillus_gasseri_K7	0	0	0	-1000	-28.492502	0	0	-770.13438	0
Lactobacillus_gasseri_MV_22	0	0	0	-411.7647059	-27.222795	0	0	-1000	0
Lactobacillus_gasseri_SJ_9E_US	0	0	0	-1000	-28.273639	0	0	-1000	0
Lactobacillus_gasseri_SV_16A_US	0	0	0	-1000	-25.945896	0	0	-1000	0
Lactobacillus_gastricus_PS3	0	0	0	-37.30691535	-14.66843	0	0	-779.40057	0
Lactobacillus_helveticus_DPC_4571	0	0	0	-320	-167.54182	0	0	0	0
Lactobacillus_helveticus_DSM_20075	0	0	0	0	-14.930043	0	0	0	0
Lactobacillus_helveticus_H10	0	0	0	0	-22.680082	0	0	-823.60248	0
Lactobacillus_helveticus_R0052	0	0	0	0	-28.58939	0	0	0	0
Lactobacillus_hilgardii_ATCC_8290	0	0	0	-1000	-25.039093	0	0	0	0
Lactobacillus_iners_AB_1	0	0	0	0	-23.965809	0	0	0	0
Lactobacillus_iners_ATCC_55195	0	0	0	0	-24.830401	0	0	0	0
Lactobacillus_iners_DSM_13335	0	0	0	0	-15.595294	0	0	0	0
Lactobacillus_iners_LactinV_03V1_b	0	0	0	0	-23.714932	0	0	0	0
Lactobacillus_iners_LactinV_09V1_c	0	0	0	0	-28.374465	0	0	0	0
Lactobacillus_iners_LactinV_11V1_d	0	0	0	0	-25.001495	0	0	0	0
Lactobacillus_iners_LEAF_2052A_d	0	0	0	0	-27.668381	0	0	0	0
Lactobacillus_iners_LEAF_2053A_b	0	0	0	0	-26.469717	0	0	0	0
Lactobacillus_iners_LEAF_2062A_h1	0	0	0	0	-26.24021	0	0	0	0
Lactobacillus_iners_LEAF_3008A_a	0	0	0	0	-28.293131	0	0	0	0
Lactobacillus_iners_SPIN_1401G	0	0	0	0	-28.293131	0	0	0	0
Lactobacillus_iners_SPIN_2503V10_D	0	0	0	0	-28.049562	0	0	0	0
Lactobacillus_iners_UPII_143_D	0	0	0	0	-28.101248	0	0	0	0
Lactobacillus_iners_UPII_60_B	0	0	0	0	-28.291213	0	0	0	0
Lactobacillus_ingluviei_str_Autruche_4	0	0	0	-1000	-28.148357	0	0	-1000	0
Lactobacillus_intestinalis_ERR2221123	0	0	0	0	-27.598529	-1000	0	-694.44444	0
Lactobacillus_jensenii_115_3_CHN	0	0	0	-1000	-29.587591	0	0	-769.94815	0
Lactobacillus_jensenii_1153	0	0	0	-24.80383675	-20.137881	0	0	-540.23643	0
Lactobacillus_jensenii_269_3	0	0	0	-73.69839292	-33.523263	0	0	-888.88889	0
Lactobacillus_jensenii_27_2_CHN	0	0	0	-1000	-30.169049	0	0	-774.4034	0
Lactobacillus_jensenii_JV_V16	0	0	0	-1000	-29.587774	0	0	-769.94831	0
Lactobacillus_jensenii_SJ_7A_US	0	0	0	-73.71530436	-33.547968	0	0	-771.51084	0
Lactobacillus_johnsonii_ATCC_33200	0	0	0	-1000	-28.640936	0	0	-763.17195	0
Lactobacillus_johnsonii_DPC_6026	0	0	0	0	-43.17541	0	0	-583.33333	0
Lactobacillus_johnsonii_ERR2221124	0	0	0	-30.07514187	-26.235648	0	0	-579.54545	0
Lactobacillus_johnsonii_ERR2221205	0	0	0	-30.07514771	-26.235652	0	0	-579.54545	0
Lactobacillus_johnsonii_FI9785	0	0	0	-1000	-28.402042	0	0	-763.04378	0
Lactobacillus_johnsonii_NCC_533	0	0	0	0	-800	0	0	-666.66667	0
Lactobacillus_johnsonii_pf01	0	0	0	-25.90052616	-21.292373	0	0	-756.07426	0
Lactobacillus_kalixensis_DSM_16043	0	0	0	0	-15.683701	-1000	0	-626.1625	0
Lactobacillus_kefiranofaciens_ZW3	0	0	0	0	-18.352155	0	0	0	0
Lactobacillus_kunkeei_DSM_12361_ATCC_70030	0	0	0	-30.21081176	-17.248502	-1000	0	-1000	0
Lactobacillus_kunkeei_EFB6	0	0	0	-21.89275039	-17.29844	-1000	0	-1000	0
Lactobacillus_kunkeei_MP2	0	0	0	-29.60228876	-17.13869	-1000	0	-1000	0
Lactobacillus_lindneri_DSM_20690_JCM_11027	0	0	0	-18.10699287	-11.9058	0	0	0	0
Lactobacillus_lindneri_TMW_1_2007	0	0	0	-18.0747358	-11.889712	0	0	0	0
Lactobacillus_mali_KCTC_3596_DSM_20444	0	0	0	-1000	-19.44867	-1000	0	-875	0
Lactobacillus_manihotivorans_DSM_13343_JCM_	0	0	0	0	-16.781356	0	0	-625.34576	0
Lactobacillus_mindensis_DSM_14500	0	0	0	-33.79624689	-17.341459	0	0	0	0
Lactobacillus_mucosae_LM1	0	0	0	-41.13190575	-24.694624	0	0	-1000	0
Lactobacillus_murinus_ASF361	0	0	0	-33.51050472	-20.142013	-1000	0	-760.18526	0

Lactobacillus_murinus_ERR2221126	0	0	0	-39.31817575	-27.832594	-1000	0	-750	0
Lactobacillus_nov_ERR2221311	0	0	0	-1000	-38.075227	0	0	0	0
Lactobacillus_nov_ERR2221384	0	0	0	0	-27.750262	-1000	0	0	0
Lactobacillus_oligofermentans_DSM_15707_LMG	0	0	0	-778.4179473	-18.445596	0	0	0	0
Lactobacillus_oris_F0423	0	0	0	-1000	-32.015391	0	0	-1000	0
Lactobacillus_oris_PB013_T2_3	0	0	0	-1000	-28.205776	0	0	-1000	0
Lactobacillus_panis_DSM_6035	0	0	0	-41.25302227	-14.254169	0	0	-624.11548	0
Lactobacillus_parabrevis_ATCC_53295	0	0	0	-33.18007835	-32.992613	0	0	0	0
Lactobacillus_parabuchneri_DSM_5707_NBRC_1(	0	0	0	-1000	-18.276994	0	0	-511.76095	0
Lactobacillus_parabuchneri_FAM21829	0	0	0	-1000	-18.276991	0	0	-511.76094	0
Lactobacillus_paracasei_32G	0	0	0	0	-23.58664	-1000	0	-1000	0
Lactobacillus_paracasei_BD_II	0	0	0	0	-23.859164	-1000	0	-1000	0
Lactobacillus_paracasei_CRF28	0	0	0	0	-23.768987	-1000	0	-1000	0
Lactobacillus_paracasei_Lc_10	0	0	0	0	-23.835965	-1000	0	-1000	0
Lactobacillus_paracasei_LC2W	0	0	0	0	-23.859164	-1000	0	-1000	0
Lactobacillus_paracasei_LPC_37	0	0	0	0	-23.686866	-1000	0	-1000	0
Lactobacillus_paracasei_M36	0	0	0	0	-23.731984	-1000	0	-1000	0
Lactobacillus_paracasei_subsp_paracasei_8700_2	0	0	0	-1000	-25.332204	-1000	0	-875	0
Lactobacillus_paracasei_subsp_paracasei_ATCC_2	0	0	0	-1000	-25.332204	-1000	0	-875	0
Lactobacillus_paracasei_subsp_paracasei_Lpp120	0	0	0	0	-23.454672	-1000	0	-1000	0
Lactobacillus_paracasei_subsp_paracasei_Lpp122	0	0	0	-1000	-23.900399	-1000	0	-1000	0
Lactobacillus_paracasei_subsp_paracasei_Lpp17	0	0	0	-1000	-24.025279	-1000	0	-1000	0
Lactobacillus_paracasei_subsp_paracasei_Lpp225	0	0	0	-1000	-24.077675	-1000	0	-1000	0
Lactobacillus_paracasei_subsp_paracasei_Lpp226	0	0	0	-1000	-23.851864	-1000	0	-1000	0
Lactobacillus_paracasei_subsp_paracasei_Lpp230	0	0	0	-1000	-23.92747	-1000	0	-1000	0
Lactobacillus_paracasei_subsp_paracasei_Lpp46	0	0	0	-1000	-24.025263	-1000	0	-1000	0
Lactobacillus_paracasei_subsp_paracasei_Lpp49	0	0	0	-1000	-24.025247	-1000	0	-1000	0
Lactobacillus_paracasei_subsp_paracasei_Lpp74	0	0	0	0	-23.686866	-1000	0	-1000	0
Lactobacillus_paracasei_subsp_tolerans_Lpl14	0	0	0	-1000	-24.077675	-1000	0	-1000	0
Lactobacillus_paracasei_subsp_tolerans_Lpl7	0	0	0	-1000	-24.077642	-1000	0	-1000	0
Lactobacillus_paracasei_T71499	0	0	0	0	-23.859132	-1000	0	-1000	0
Lactobacillus_paracasei_UCD174	0	0	0	0	-23.496786	-1000	0	-1000	0
Lactobacillus_paracasei_UW1	0	0	0	0	-23.128238	-1000	0	-1000	0
Lactobacillus_paracasei_Zhang	0	0	0	0	-23.807714	-1000	0	-1000	0
Lactobacillus_paraplantarum_D2_1	0	0	0	-1000	-21.871344	-1000	0	-605.98275	0
Lactobacillus_paraplantarum_DSM_10667	0	0	0	-1000	-21.332855	-1000	0	-625.73038	0
Lactobacillus_pasteurii_DSM_23907_CRBIP_24_7	0	0	0	0	-26.854088	-1000	0	-750	0
Lactobacillus_pentosus_KCA1	0	0	0	-500	-21.62411	-1000	0	-1000	0
Lactobacillus_plantarum_16	0	0	0	-1000	-35.164074	-1000	0	-1000	0
Lactobacillus_plantarum_ERR2221349	0	0	0	-1000	-30.870638	-1000	0	-1000	0
Lactobacillus_plantarum_IPLA88	0	0	0	-1000	-35.07858	-1000	0	-1000	0
Lactobacillus_plantarum_JDM1	0	0	0	-1000	-178.57093	-1000	0	-1000	0
Lactobacillus_plantarum_subsp_plantarum_ATCC	0	0	0	-1000	-29.052767	-1000	0	-1000	0
Lactobacillus_plantarum_subsp_plantarum_NC8	0	0	0	-1000	-34.232561	-1000	0	-1000	0
Lactobacillus_plantarum_subsp_plantarum_P_8	0	0	0	-1000	-35.113403	-1000	0	-1000	0
Lactobacillus_plantarum_subsp_plantarum_ST_III	0	0	0	-1000	-35.17009	-1000	0	-1000	0
Lactobacillus_plantarum_UCMA_3037	0	0	0	-1000	-34.725192	-1000	0	-1000	0
Lactobacillus_plantarum_WCFS1	0	0	0	-1000	-29.648368	-1000	0	-1000	0
Lactobacillus_plantarum_ZJ316	0	0	0	-1000	-34.78109	-1000	0	-1000	0
Lactobacillus_pobuzihii_E100301_KCTC_13174	0	0	0	0	-27.187751	0	0	0	0
Lactobacillus_pontis_DSM_8475	0	0	0	-37.18523443	-15.446288	0	0	-524.17849	0
Lactobacillus_pontis_UMB0683_21837_8_53_1	0	0	0	-1000	-16.137569	0	0	-1000	0
Lactobacillus_rapi_DSM_19907_JCM_15042	0	0	0	-43.94688938	-19.011723	0	0	-513.09251	0
Lactobacillus_reuteri_100_23	0	0	0	-1000	-40.533746	0	0	-1000	0
Lactobacillus_reuteri_ATCC_53608	0	0	0	-1000	-40.394847	0	0	-1000	0
Lactobacillus_reuteri_CF48_3A	0	0	0	-388.8888889	-27.419006	0	0	-1000	0
Lactobacillus_reuteri_DSM_20016	0	0	0	-1000	-41.903973	0	0	-1000	0
Lactobacillus_reuteri_ERR2221127	0	0	0	-1000	-39.38233	0	0	-812.5	0
Lactobacillus_reuteri_ERR2221204	0	0	0	-1000	-39.399878	0	0	-812.5	0
Lactobacillus_reuteri_F275_JCM_1112	0	0	0	-388.8888889	-28.975134	0	0	-755.22741	0
Lactobacillus_reuteri_I49	0	0	0	-1000	-39.429964	0	0	-812.5	0
Lactobacillus_reuteri_I5007	0	0	0	-1000	-38.497463	0	0	-1000	0
Lactobacillus_reuteri_lpuph	0	0	0	-1000	-41.428778	0	0	-1000	0
Lactobacillus_reuteri_mlc3	0	0	0	-1000	-43.842599	0	0	-1000	0
Lactobacillus_reuteri_MM2_3	0	0	0	-768.5185185	-29.345553	0	0	-758.01293	0
Lactobacillus_reuteri_MM4_1A	0	0	0	-727.2727273	-34.890417	0	0	-1000	0
Lactobacillus_reuteri_SD2112_ATCC_55730	0	0	0	-388.8888889	-28.573236	0	0	-1000	0
Lactobacillus_rhamnosus_ATCC_21052	0	0	-1000	-1000	-29.360707	-1000	0	-1000	0
Lactobacillus_rhamnosus_ATCC_8530	0	0	-1000	-1000	-29.364571	-1000	0	-1000	0
Lactobacillus_rhamnosus_ERR1203919	0	0	0	0	-27.916271	-1000	0	-1000	0
Lactobacillus_rhamnosus_ERR2221226	0	0	0	0	-27.916271	-1000	0	-1000	0
Lactobacillus_rhamnosus_GG_ATCC_53103	0	0	-1000	-1000	-175.81314	-1000	0	-1000	0
Lactobacillus_rhamnosus_HN001	0	0	-1000	-1000	-29.626061	-1000	0	-1000	0
Lactobacillus_rhamnosus_Lc_705	0	0	-1000	-1000	-29.364531	-1000	0	-1000	0
Lactobacillus_rhamnosus_LMS2_1	0	0	-1000	-1000	-25.401455	-1000	0	-1000	0
Lactobacillus_rhamnosus_LRHMDP2	0	0	-1000	-1000	-29.194185	-1000	0	-1000	0
Lactobacillus_rhamnosus_LRHMDP3	0	0	-1000	-1000	-29.371323	-1000	0	-1000	0
Lactobacillus_rhamnosus_R0011	0	0	-1000	-1000	-29.364531	-1000	0	-1000	0
Lactobacillus_rodentium_DSM_24759	0	0	0	0	-23.344698	0	0	-694.44444	0
Lactobacillus_ruminis_ATCC_25644	0	0	0	0	-18.200254	0	0	-522.21096	0
Lactobacillus_ruminis_ATCC_27782	0	0	0	-36.23272942	-22.515076	0	0	-1000	0
Lactobacillus_ruminis_DSM_20403_NBRC_10216	0	0	0	-51.1774308	-22.703672	0	0	-516.3341	0
Lactobacillus_ruminis_ERR2221165	0	0	0	0	-21.741198	0	0	-592.10526	0
Lactobacillus_ruminis_ERR2221355	0	0	0	0	-21.742468	0	0	-592.10526	0
Lactobacillus_ruminis_SPM0211	0	0	0	0	-24.497962	0	0	-820.54315	0
Lactobacillus_saerimneri_30a	0	0	0	0	-17.808153	0	0	-349.1766	0
Lactobacillus_sakei_subsp_sakei_23K	0	0	0	-27.54567462	-169.10509	0	0	-717.94872	0
Lactobacillus_sakei_subsp_sakei_DSM_20017_JCI	0	0	0	-26.96275933	-24.111986	0	0	-638.29787	0
Lactobacillus_salivarius_CECT_5713	0	0	0	-1000	-30.174596	-1000	0	-1000	0
Lactobacillus_salivarius_ERR2221215	0	0	0	-1000	-28.720745	-1000	0	-1000	0
Lactobacillus_salivarius_ERR2221301	0	0	0	-1000	-28.720745	-1000	0	-1000	0

Lactobacillus_salivarius_ERR2221323	0	0	0	-1000	-28.72075	-1000	0	-1000	0
Lactobacillus_salivarius_GJ_24	0	0	0	-1000	-29.63663	-1000	0	-1000	0
Lactobacillus_salivarius_HO66_ATCC_11741	0	0	0	-1000	-25.940863	-1000	0	-1000	0
Lactobacillus_salivarius_NIAS840	0	0	0	-1000	-29.435297	-1000	0	-1000	0
Lactobacillus_salivarius_salivarius_UCC118	0	0	0	-1000	-26.068293	-1000	0	-1000	0
Lactobacillus_sanfranciscensis_DSM_20451	0	0	0	-22.3975121	-18.755972	0	0	0	0
Lactobacillus_sanfranciscensis_TMW_1_1304	0	0	0	-28.15024281	-27.09458	0	0	0	0
Lactobacillus_sanfranciscensis_TMW_1_2137	0	0	0	-22.4059022	-15.291322	0	0	0	0
Lactobacillus_siliginis_DSM_22696	0	0	0	0	-22.291326	0	0	0	0
Lactobacillus_siliginis_NBRC_101315	0	0	0	0	-22.291234	0	0	0	0
Lactobacillus_suebicus_DSM_5007_KCTC_3549	0	0	-1000	-1000	-31.477524	0	0	0	0
Lactobacillus_taiwanensis_DSM_21401	0	0	0	0	-27.114626	0	0	-600	0
Lactobacillus_ultunensis_DSM_16047	0	0	0	0	-24.125318	-1000	0	-1000	0
Lactobacillus_vaccinostercus_DSM_20634	0	0	0	-1000	-18.636554	0	0	0	0
Lactobacillus_vaginalis_ATCC_49540	0	0	0	-42.40801051	-26.694681	0	0	-1000	0
Lactobacillus_vaginalis_ERR2221166	0	0	0	-1000	-38.20136	0	0	-506.86574	0
Lactobacillus_versmoldensis_DSM_14857_KCTC_	0	0	0	-58.06779877	-29.804303	0	0	0	0
Lactobacillus_zeae_DSM_20178_KCTC_3804	0	0	-1000	0	-27.934263	-521.51675	0	-260.75837	0
Lactococcus_garvieae_21881	0	0	0	-1000	-38.614025	-1000	0	0	0
Lactococcus_garvieae_8831	0	0	0	-1000	-38.711814	-1000	0	0	0
Lactococcus_garvieae_ATCC_49156	0	0	0	0	-18.869993	-1000	0	0	0
Lactococcus_garvieae_DCC43	0	0	0	0	-38.274737	0	0	0	0
Lactococcus_garvieae_I113	0	0	0	0	-38.233211	-1000	0	0	0
Lactococcus_garvieae_IPLA_31405	0	0	0	-1000	-38.711163	-1000	0	0	0
Lactococcus_garvieae_Lg2	0	0	0	0	-38.223461	-1000	0	0	0
Lactococcus_garvieae_LG9	0	0	0	-1000	-38.593017	-1000	0	0	0
Lactococcus_garvieae_Tac2	0	0	0	-1000	-38.480751	-1000	0	0	0
Lactococcus_garvieae_TB25	0	0	0	-1000	-38.711683	-1000	0	0	0
Lactococcus_garvieae_UNIUD074	0	0	0	-1000	-38.592758	-1000	0	0	0
Lactococcus_lactis_subsp_cremoris_A76	0	0	0	0	-16.468039	0	0	0	0
Lactococcus_lactis_subsp_cremoris_CNCM_I_163	0	0	0	-1000	-19.351858	-1000	0	0	0
Lactococcus_lactis_subsp_cremoris_MG1363	0	0	0	-1000	-18.301278	-1000	0	0	0
Lactococcus_lactis_subsp_cremoris_NZ9000	0	0	0	-1000	-18.301278	-1000	0	0	0
Lactococcus_lactis_subsp_cremoris_SK11	0	0	0	0	-24.898458	-1000	0	0	0
Lactococcus_lactis_subsp_cremoris_UC509_9	0	0	0	0	-18.326469	-1000	0	0	0
Lactococcus_lactis_subsp_lactis_CV56	0	0	0	-1000	-19.351854	-1000	0	0	0
Lactococcus_lactis_subsp_lactis_Il1403	0	0	0	-1000	-20.692521	-1000	0	0	0
Lactococcus_lactis_subsp_lactis_IO_1	0	0	0	0	-19.345386	-1000	0	0	0
Lactococcus_lactis_subsp_lactis_KF147	0	0	0	-1000	-19.32712	-1000	0	0	0
Lactococcus_lactis_subsp_lactis_YF11	0	0	0	-1000	-19.329967	-1000	0	0	0
Lactococcus_raffinolactis_4877	0	0	0	0	-23.020049	-1000	0	-1000	0
Lactonifactor_longoviformis_DSM_17459	0	0	0	-41.435488	-14.361347	0	0	-1000	0
Laribacter_hongkongensis_HLHK9	0	0	0	-1000	-14.310451	0	0	0	0
Lautropia_mirabilis_ATCC_51599	0	0	0	-1000	-30.774217	-1000	0	-819.04762	0
Leclercia_adecarboxylata_ATCC_23216_NBRC_10	0	0	-1000	-1000	-30.659307	-1000	0	0	0
Leclercia_adecarboxylata_KY2	0	0	-1000	-1000	-32.079097	-1000	0	0	0
Leminorella_grimontii_ATCC_33999	0	0	0	-1000	-37.05977	0	0	0	0
Leptotrichia_buccalis_C_1013_b	-507.6586433	0	0	-1000	-26.789984	0	0	-552.14153	0
Leptotrichia_goodfellowii_DSM_19756	0	0	0	-1000	-16.003001	0	0	-422.5077	0
Leptotrichia_goodfellowii_F0264	0	0	0	-1000	-32.866105	0	0	-538.88889	0
Leptotrichia_hofstadii_DSM_21651	0	0	0	-1000	-25.22271	0	0	-420.14577	0
Leptotrichia_hofstadii_F0254	0	0	0	-1000	-27.33223	0	0	-358.58586	0
Leptotrichia_hongkongensis_JMUB5056	0	0	0	-25.81132612	-17.509588	0	0	-418.17325	0
Leptotrichia_shahii_DSM_19757	0	0	0	-1000	-27.456612	0	0	-472.54335	0
Leptotrichia_wadei_DSM_19758	0	0	0	-1000	-27.511438	0	0	-477.27273	0
Leptotrichia_wadei_F0279	0	0	0	-1000	-25.256364	0	0	-465.42553	0
Leptotrichia_wadei_KA00185	0	0	0	-1000	-25.281048	0	0	-465.42553	0
Leuconostoc_argentinum_KCTC_3773	0	0	0	0	-16.676373	0	0	-983.32124	0
Leuconostoc_carnosum_JB16	0	0	0	0	-26.406621	0	0	-524.56272	0
Leuconostoc_citreum_1300_LCIT_132_3537_308	0	0	0	0	-27.831856	-1000	0	-514.41738	0
Leuconostoc_citreum_KM20	0	0	0	0	-31.66906	-1000	0	-506.8829	0
Leuconostoc_citreum_LBAE_C10	0	0	0	0	-31.205513	-1000	0	-506.80825	0
Leuconostoc_citreum_LBAE_C11	0	0	0	0	-45.5088	-1000	0	-511.09303	0
Leuconostoc_citreum_LBAE_E16	0	0	0	0	-31.66906	-1000	0	-506.8829	0
Leuconostoc_gelidum_JB7	0	0	0	0	0	0	0	-1000	0
Leuconostoc_gelidum_KCTC_3527	0	0	0	0	0	0	0	-1000	0
Leuconostoc_gelidum_subsp_gasicomitatum_C12	0	0	0	0	-31.020589	0	0	-600	0
Leuconostoc_lactis_KACC_91922	0	0	0	0	-17.780901	0	0	-989.00465	0
Leuconostoc_mesenteroides_ERR2221167	0	0	0	0	-24.114214	0	0	0	0
Leuconostoc_mesenteroides_subsp_cremoris_AT	0	0	0	0	-26.419748	0	0	0	0
Leuconostoc_mesenteroides_subsp_mesenteroid	0	0	0	0	-26.035492	0	0	0	0
Leuconostoc_mesenteroides_subsp_mesenteroid	0	0	0	0	-28.574268	0	0	-508.70044	0
Leuconostoc_pseudomesenteroides_4882	0	0	0	0	-16.689806	-1000	0	-758.77636	0
Leuconostoc_pseudomesenteroides_ERR2221296	0	0	0	0	-31.949176	-1000	0	-700	0
Listeria_grayi_DSM_20601	0	0	0	0	-1000	-1000	0	0	0
Listeria_innocua_ATCC_33091	0	0	0	-1000	-1000	0	0	-541.66667	0
Listeria_innocua_Clip11262	0	0	0	-1000	-1000	0	0	-541.66667	0
Listeria_monocytogenes_07PF0776	0	0	0	-1000	-1000	0	0	-1000	0
Listeria_monocytogenes_08_5578	0	0	0	-1000	-1000	0	0	-1000	0
Listeria_monocytogenes_08_5923	0	0	0	-1000	-1000	0	0	-1000	0
Listeria_monocytogenes_10403S	0	0	0	-1000	-1000	0	0	-1000	0
Listeria_monocytogenes_4a_L99	0	0	0	-1000	-1000	0	0	-859.64912	0
Listeria_monocytogenes_4b_F2365	0	0	0	-1000	-1000	0	0	-857.14286	0
Listeria_monocytogenes_ATCC_19117	0	0	0	-1000	-1000	0	0	-1000	0
Listeria_monocytogenes_Clip80459	0	0	0	-1000	-1000	0	0	-729.16667	0
Listeria_monocytogenes_EGD_e	0	0	0	-1000	-1000	0	0	-780.13368	0
Listeria_monocytogenes_F6900	0	0	0	-1000	-1000	0	0	-1000	0
Listeria_monocytogenes_Finland_1988	0	0	0	-1000	-1000	0	0	-857.14286	0
Listeria_monocytogenes_FSL_J1_194	0	0	0	-1000	-1000	0	0	-1000	0
Listeria_monocytogenes_FSL_J2_071	0	0	0	-1000	-1000	0	0	-1000	0
Listeria_monocytogenes_FSL_N1_017	0	0	0	-1000	-1000	0	0	-1000	0

Listeria_monocytogenes_FSL_N3_165	0	0	0	-1000	-1000	0	0	-1000	0
Listeria_monocytogenes_FSL_R2_503	0	0	0	-1000	-1000	0	0	-1000	0
Listeria_monocytogenes_FSL_R2_561	0	0	0	-1000	-1000	0	0	-857.14286	0
Listeria_monocytogenes_HCC23	0	0	0	-1000	-1000	0	0	-1000	0
Listeria_monocytogenes_HPB2262	0	0	0	-1000	-1000	0	0	-1000	0
Listeria_monocytogenes_J0161_FSL_R2_499	0	0	0	-1000	-1000	0	0	-857.14286	0
Listeria_monocytogenes_J2818	0	0	0	-1000	-1000	0	0	-1000	0
Listeria_monocytogenes_JF5171	0	0	0	-1000	-1000	0	0	-729.16667	0
Listeria_monocytogenes_L312	0	0	0	-1000	-1000	0	0	-1000	0
Listeria_monocytogenes_L99	0	0	0	-1000	-1000	0	0	-1000	0
Listeria_monocytogenes_M7	0	0	0	-1000	-1000	0	0	-1000	0
Listeria_monocytogenes_N53_1	0	0	0	-1000	-1000	0	0	-1000	0
Listeria_monocytogenes_serotype_1_2a_str_NCC	0	0	0	0	-1000	0	0	-1000	0
Listeria_monocytogenes_serotype_1_2b_str_SLC	0	0	0	-1000	-1000	0	0	-950.69405	0
Listeria_monocytogenes_serotype_1_2c_str_SLC	0	0	0	-1000	-1000	0	0	-759.03793	0
Listeria_monocytogenes_serotype_4b_str_LL195	0	0	0	-1000	-1000	0	0	-1000	0
Listeria_monocytogenes_serotype_7_SLCC_2482	0	0	0	-1000	-1000	0	0	-857.14286	0
Listeria_monocytogenes_SLCC_2378	0	0	0	-1000	-1000	0	0	-857.14286	0
Listeria_monocytogenes_SLCC_2540	0	0	0	-1000	-1000	0	0	-857.14286	0
Listeria_monocytogenes_SLCC_7179	0	0	0	-1000	-1000	0	0	-857.14286	0
Listeria_monocytogenes_SLCC2376	0	0	0	-1000	-1000	0	0	-1000	0
Listeria_monocytogenes_SLCC2479	0	0	0	-1000	-1000	0	0	-780.13368	0
Listeria_monocytogenes_SLCC5850	0	0	0	-1000	-1000	0	0	-1000	0
Listeria_monocytogenes_str_1_2a_F6854	0	0	0	-1000	-1000	0	0	-1000	0
Listeria_monocytogenes_str_4b_H7858	0	0	0	-1000	-1000	0	0	-1000	0
Listeria_monocytogenes_str_Scott_A	0	0	0	-1000	-1000	0	0	-1000	0
Loktanella_vestfoldensis_SMR4r	0	0	0	-31.46980277	-1000	0	0	0	0
Longibaculum_muris_DSM_29487	0	0	0	0	-10.745366	0	0	-305.55556	0
Longicatena_caecimuris_DSM_29481	0	0	0	0	-23.189858	-853.59116	-1000	0	0
Lysinibacillus_fusiformis_ZB2	0	0	0	-1000	-25.047959	0	-1000	0	0
Lysinibacillus_fusiformis_ZC1	0	0	0	-1000	-25.289584	0	-1000	0	0
Lysinibacillus_sphaericus_2362	0	0	0	-1000	-23.278514	0	-1000	0	0
Lysinibacillus_sphaericus_C3_41	0	0	0	-1000	-20.555812	0	-1000	0	0
Lysinibacillus_sphaericus_OT4b_31	0	0	0	-1000	-21.63126	0	-1000	0	0
Lysinibacillus_sphaericus_OT4b25	0	0	0	-1000	-23.278514	0	-1000	0	0
Lysinibacillus_sphaericus_OT4b49	0	0	0	-1000	-23.278514	0	-1000	0	0
Marinospirillum_alkaliphilum_DSM_21637	0	0	0	0	-17.631531	0	0	0	0
Marvinbryantia_formatexigens_I_52_DSM_14469	0	0	-1000	0	-24.865015	0	0	-597.8943	0
Megamonas_funiformis_YIT_11815	0	0	0	-1000	-42.603133	-1000	0	-1000	0
Megamonas_hypermegale_ART12_1	0	0	0	-1000	-30.890824	-1000	0	-1000	0
Megamonas_rupellensis_DSM_19944	0	0	0	-1000	-29.686585	-948.80668	0	-518.10585	0
Megasphaera_elsdenii_DSM_20460	0	0	0	-1000	-1000	-1000	-1000	-821.42857	0
Megasphaera_massiliensis_NP3	0	0	0	-17.97869745	-9.892861	0	0	0	0
Megasphaera_micronuciformis_F0359	0	0	0	-1000	-14.403417	0	0	0	0
Megasphaera_nov_ERR1022370	0	0	0	-1000	-12.916413	0	0	0	0
Megasphaera_paucivorans_DSM_16981	0	0	0	0	-19.131991	0	0	0	0
Meiothermus_silvanus_DSM_9946	-12.53984268	0	0	-1000	-15.112064	-1000	-1000	0	0
Melainabacterium_MEL_A1	0	0	0	-21.23552124	-100.50251	0	0	0	0
Merdibacter_massiliensis_Marseille_P3254	0	0	0	0	-13.51988	0	0	-377.45947	0
Mesorhizobium_loti_MAFF303099	0	0	0	-1000	-38.187765	-1000	0	-1000	0
Mesorhizobium_loti_NZP2037	0	0	0	0	-16.705187	-1000	0	-964.28571	0
Metakosakonia_massiliensis_JC163	0	0	-1000	-1000	-31.804013	-1000	0	0	0
Methanobrevibacter_ruminantium_M1	0	0	0	-1000	0	0	0	0	0
Methanobrevibacter_smithii_ATCC_35061	0	0	0	0	0	0	-1000	0	0
Methanomassiliicoccus_luminyensis_B10	0	0	0	0	0	0	0	0	0
Methanosphaera_stadtmanae_DSM_3091	0	0	0	0	-6.593416	0	0	0	0
Methylobacterium_mesophilicum_SR1_6_6	0	0	0	-1000	-36.990646	0	-1000	0	0
Methylobacterium_populi_BJ001	0	0	0	-1000	-37.66519	0	-1000	0	0
Methylobacterium_radiotolerans_JCM_2831	0	0	0	-1000	-36.733312	0	-1000	0	0
Methyloversatilis_universalis_EHg5	0	0	0	-1000	-16.755778	0	0	0	0
Methyloversatilis_universalis_FAM5	0	0	0	-1000	-34.030273	0	0	0	0
Methyloversatilis_universalis_Fam50001	0	0	0	-1000	-37.028966	0	0	0	0
Microbacterium_gubbeenense_DSM_15944	0	0	0	0	0	-1000	0	0	0
Microbacterium_oleivorans_NBRC_103075	0	0	0	0	-1000	-1000	-1000	-1000	0
Microbacterium_paraoxydans_77MFTsu3_2	0	0	0	0	-1000	-1000	0	-1000	0
Micrococcus_luteus_1058_MLUT	0	0	0	-1000	-1000	0	-1000	-1000	0
Micrococcus_luteus_ERR2221282	0	0	0	-1000	-1000	0	-1000	-553.62128	0
Micrococcus_luteus_NCTC_2665	0	0	0	-1000	-26.041843	0	-1000	-1000	0
Micrococcus_luteus_NDB3Y10	0	0	0	-1000	0	0	-1000	-553.39862	0
Micrococcus_luteus_RIT304	0	0	0	-1000	-1000	0	-1000	-750	0
Micrococcus_luteus_RIT305	0	0	0	-1000	-1000	0	-1000	-1000	0
Micrococcus_luteus_RIT324w	0	0	0	-1000	-1000	0	-1000	-553.6245	0
Micrococcus_luteus_SK58	0	0	0	-1000	0	0	-1000	-1000	0
Micrococcus_luteus_trpE16	0	0	0	-1000	-1000	0	-1000	-975.15528	0
Micromonospora_aurantiaca_ATCC_27029	0	0	0	-1000	-31.268846	0	0	0	0
Microvirga_massiliensis_JC119	0	0	0	-1000	-15.894531	0	0	0	0
Mitsuokella_jalaludinii_DSM_13811	0	0	0	-1000	-55.853843	0	0	-832	0
Mitsuokella_jalaludinii_ERR1022286	0	0	0	-1000	-32.972075	-1000	0	-540.47218	0
Mitsuokella_multacida_DSM_20544	0	0	0	-1000	-31.361563	-750	0	-375	0
Mobiluncus_curtisii_ATCC_43063	0	0	0	-68.33197797	-36.609087	0	0	0	0
Mobiluncus_curtisii_ATCC_51333	0	0	0	-40.71521545	-19.840348	0	0	0	0
Mobiluncus_curtisii_subsp_holmesii_ATCC_3524	0	0	0	-36.54124921	-18.83924	0	0	0	0
Mobiluncus_mulieris_28_1	0	0	0	-1000	-29.545076	0	0	0	0
Mobiluncus_mulieris_ATCC_35239	0	0	0	-1000	-29.544189	0	0	0	0
Mobiluncus_mulieris_ATCC_35243	0	0	0	-1000	-29.544561	0	0	0	0
Mobiluncus_mulieris_FB024_16	0	0	0	-1000	-22.158807	0	0	0	0
Mogibacterium_sp_CM50	0	0	0	-1000	-18.582672	0	0	0	0
Mogibacterium_timidum_ATCC_33093	0	0	0	-1000	-8.5123964	0	0	0	0
Moraxella_bovoculi_22581	0	0	0	-1000	-1000	0	0	0	0
Moraxella_bovoculi_237	0	0	0	-1000	-1000	0	0	0	0
Moraxella_canis_CCUG_8415A_8415T1	0	0	0	-1000	-1000	0	0	0	0

Moraxella_catarrhalis_101P30B1	0	0	0	-1000	0	0	0	0	0
Moraxella_catarrhalis_103P14B1	0	0	0	-1000	0	0	0	0	0
Moraxella_catarrhalis_12P80B1	0	0	0	-1000	0	0	0	0	0
Moraxella_catarrhalis_25240	0	0	0	-1000	-21.643393	0	0	0	0
Moraxella_catarrhalis_46P47B1	0	0	0	-1000	0	0	0	0	0
Moraxella_catarrhalis_7169	0	0	0	-1000	0	0	0	0	0
Moraxella_catarrhalis_BC1	0	0	0	-1000	0	0	0	0	0
Moraxella_catarrhalis_BC7	0	0	0	-1000	0	0	0	0	0
Moraxella_catarrhalis_BC8	0	0	0	-1000	0	0	0	0	0
Moraxella_catarrhalis_CO72	0	0	0	-1000	0	0	0	0	0
Moraxella_catarrhalis_O35E	0	0	0	-1000	0	0	0	0	0
Moraxella_catarrhalis_RH4	0	0	0	-1000	0	0	0	0	0
Morganella_morganii_FDAARGOS_172	0	0	0	-1000	-49.520516	0	0	0	0
Morganella_morganii_FDAARGOS_63	0	0	0	-1000	-51.345833	0	0	0	0
Morganella_morganii_SC01	0	0	0	-1000	-36.447579	0	0	-1000	0
Morganella_morganii_subsp_morganii_KT	0	0	0	-1000	-58.480694	0	0	0	0
Mucispirillum_schaedleri_ASF457	0	0	0	-47.43055996	-13.006038	0	0	0	0
Muribaculum_intestinale_YL27	0	0	-1000	0	-27.687489	0	0	0	0
Muricomes_intestini_DSM_29489	0	0	0	-833.3333333	-18.449161	-1000	0	-526.04167	0
Murimonas_intestini_DSM_26524	0	0	0	0	-23.377214	-1000	0	-791.66667	0
Mycobacterium_abscessus_3A_0119_R	0	0	0	0	-16.594393	0	0	0	0
Mycobacterium_abscessus_3A_0122_R	0	0	0	0	-16.594393	0	0	0	0
Mycobacterium_abscessus_3A_0122_S	0	0	0	0	-16.594393	0	0	0	0
Mycobacterium_abscessus_3A_0731	0	0	0	0	-16.594393	0	0	0	0
Mycobacterium_abscessus_3A_0810_R	0	0	0	0	-16.594393	0	0	0	0
Mycobacterium_abscessus_3A_0930_R	0	0	0	0	-16.594393	0	0	0	0
Mycobacterium_abscessus_3A_0930_S	0	0	0	0	-16.594393	0	0	0	0
Mycobacterium_abscessus_47J26	0	0	0	0	-19.762122	0	0	0	0
Mycobacterium_abscessus_4S_0116_R	0	0	0	0	-12.375075	0	0	0	0
Mycobacterium_abscessus_4S_0116_S	0	0	0	0	-12.375075	0	0	0	0
Mycobacterium_abscessus_4S_0206	0	0	0	0	-12.372641	0	0	0	0
Mycobacterium_abscessus_4S_0303	0	0	0	0	-12.375075	0	0	0	0
Mycobacterium_abscessus_4S_0726_RA	0	0	0	0	-12.375075	0	0	0	0
Mycobacterium_abscessus_4S_0726_RB	0	0	0	0	-12.375075	0	0	0	0
Mycobacterium_abscessus_5S_0304	0	0	0	0	-16.679438	0	0	0	0
Mycobacterium_abscessus_5S_0421	0	0	0	0	-16.679438	0	0	0	0
Mycobacterium_abscessus_5S_0422	0	0	0	0	-16.679438	0	0	0	0
Mycobacterium_abscessus_5S_0708	0	0	0	0	-16.679438	0	0	0	0
Mycobacterium_abscessus_5S_0817	0	0	0	0	-16.679438	0	0	0	0
Mycobacterium_abscessus_5S_0921	0	0	0	0	-16.679438	0	0	0	0
Mycobacterium_abscessus_5S_1212	0	0	0	0	-16.679438	0	0	0	0
Mycobacterium_abscessus_5S_1215	0	0	0	0	0	0	0	0	0
Mycobacterium_abscessus_6G_0125_R	0	0	0	0	-16.594393	0	0	0	0
Mycobacterium_abscessus_6G_0212	0	0	0	0	-16.594393	0	0	0	0
Mycobacterium_abscessus_9808	0	0	0	0	-22.210295	0	0	0	0
Mycobacterium_abscessus_M115	0	0	0	0	-16.679443	0	0	0	0
Mycobacterium_abscessus_M139	0	0	0	0	-16.679449	0	0	0	0
Mycobacterium_abscessus_M152	0	0	0	0	-16.679449	0	0	0	0
Mycobacterium_abscessus_M154	0	0	0	0	-16.672485	0	0	0	0
Mycobacterium_abscessus_M156	0	0	0	0	-1000	0	0	0	0
Mycobacterium_abscessus_M159	0	0	0	0	-16.563379	0	0	0	0
Mycobacterium_abscessus_M172	0	0	0	0	-16.672469	0	0	0	0
Mycobacterium_abscessus_M24	0	0	0	0	-20.948999	0	0	0	0
Mycobacterium_abscessus_M93	0	0	0	0	-16.638013	0	0	0	0
Mycobacterium_abscessus_M94	0	0	0	0	-19.766577	0	0	0	0
Mycobacterium_abscessus_subsp_bolletii_50594	0	0	0	0	-16.679449	0	0	0	0
Mycobacterium_abscessus_subsp_bolletii_BD	0	0	0	0	-30.357138	0	0	0	0
Mycobacterium_avium_104	0	0	0	0	0	0	0	0	0
Mycobacterium_avium_subsp_avium_ATCC_2529	0	0	0	0	0	0	0	0	0
Mycobacterium_avium_subsp_paratuberculosis_	0	0	0	0	0	0	0	0	0
Mycobacterium_avium_subsp_paratuberculosis_	0	0	0	0	0	0	0	0	0
Mycobacterium_avium_subsp_paratuberculosis_	0	0	0	0	0	0	0	0	0
Mycobacterium_avium_subsp_paratuberculosis_	0	0	0	0	0	0	0	0	0
Mycobacterium_avium_subsp_paratuberculosis_	0	0	0	0	0	0	0	0	0
Mycobacterium_avium_subsp_paratuberculosis_	0	0	0	0	0	0	0	0	0
Mycobacterium_fortuitum_subsp_fortuitum_DSM	0	0	0	0	-50.153238	0	0	0	0
Mycobacterium_tuberculosis_H37Rv	0	0	0	0	0	0	0	0	0
Mycobacterium_tuberculosis_variant_pinnipedii_	0	0	0	0	0	0	0	0	0
Mycoplasma_hominis_ATCC_23114	0	0	0	-4.345440474	-90.909091	0	0	0	0
Mycoplasma_hominis_ATCC_27545	0	0	0	-2.51259962	-4.1319615	0	0	0	0
Mycoplasma_hyopneumoniae_168	0	0	0	0	-10.487157	-281.21166	0	0	0
Mycoplasma_hyopneumoniae_168_L	0	0	0	0	-10.487157	-281.21166	0	0	0
Mycoplasma_hyopneumoniae_232	0	0	0	0	-10.480496	-281.01518	0	0	0
Mycoplasma_hyopneumoniae_7448	0	0	0	0	-10.487157	-281.21166	0	0	0
Mycoplasma_hyopneumoniae_J	0	0	0	0	-10.495019	-281.36623	0	0	0
Mycoplasma_pneumoniae_19294	0	0	0	-5.879794951	-9.6017857	-502.32977	0	0	0
Mycoplasma_pneumoniae_309	0	0	0	0	-84.996505	-604.43038	0	0	0
Mycoplasma_pneumoniae_FH	0	0	0	-5.879794951	-9.6017857	-502.32977	0	0	0
Mycoplasma_pneumoniae_M129	0	0	0	-5.879794951	-9.6017857	-502.32977	0	0	0
Mycoplasma_pneumoniae_M129_B7	0	0	0	-5.879794951	-9.6017857	-502.32977	0	0	0
Mycoplasma_pneumoniae_PI_1428	0	0	0	-5.879794951	-9.6017857	-502.32977	0	0	0
Mycoplasma_pneumoniae_PO1	0	0	0	-5.880156536	-9.602372	-502.32991	0	0	0
Myroides_odoratimimus_CCUG_10230	0	0	0	-1000	-7.1166751	0	0	0	0
Myroides_odoratimimus_CCUG_12700	0	0	0	-1000	-7.1166536	0	0	0	0
Myroides_odoratimimus_CCUG_12901	0	0	0	-1000	-7.1166708	0	0	0	0
Myroides_odoratimimus_CCUG_3837	0	0	0	-1000	-7.1182991	0	0	0	0
Myroides_odoratimimus_CIP_101113	0	0	0	-1000	-7.1111005	0	0	0	0
Myroides_odoratimimus_PR63039	0	0	0	-1000	-7.11878	0	0	0	0
Natranaerovirga_hydrolytica_DSM_24176	0	0	-1000	-1000	-22.982449	0	0	0	0
Natranaerovirga_pectinivora_DSM_24629	0	0	0	-1000	-25.080076	0	0	0	0
Neisseria_bacilliformis_914_NLAC_0_29744_6743	0	0	0	-1000	-17.070272	0	0	0	0

Neisseria_bacilliformis_ATCC_BAA_1200	0	0	0	-1000	-19.505157	0	0	0	0
Neisseria_cinerea_ATCC_14685	0	0	0	-26.99316812	-26.141616	0	0	0	0
Neisseria_elongata_subsp_glycolytica_ATCC_293	0	0	0	-1000	0	0	0	0	0
Neisseria_flavescens_NRL30031_H210	0	0	0	-1000	-26.342979	0	0	0	0
Neisseria_flavescens_SK114	0	0	0	-1000	-28.247872	0	0	0	0
Neisseria_lactamica_ATCC_23970	0	0	0	-1000	-22.52579	0	0	0	0
Neisseria_macacae_ATCC_33926	0	0	0	-1000	-27.569887	0	0	-760.41667	0
Neisseria_meningitidis_2842STDY5881431	0	0	0	-142.1052632	-23.104785	0	0	0	0
Neisseria_meningitidis_ATCC_13091	0	0	0	-191.9191919	-23.018883	0	0	0	0
Neisseria_mucosa_ATCC_25996	0	0	0	-1000	-27.588592	0	0	-812.5	0
Neisseria_mucosa_C102	0	0	0	-1000	-26.828308	0	0	-741.88106	0
Neisseria_shayeganii_871	0	0	0	0	-1000	0	0	0	0
Neisseria_sicca_4320	0	0	0	-1000	-25.213611	0	0	-708.33333	0
Neisseria_sicca_ATCC_29256	0	0	0	-1000	-31.884607	0	0	-773.14815	0
Neisseria_sicca_C2010005502	0	0	0	-1000	-32.559188	0	0	-773.14815	0
Neisseria_sicca_VK64	0	0	0	-1000	-32.61124	0	0	-742.71845	0
Neisseria_subflava_NJ9703	0	0	0	-1000	-28.247775	0	0	0	0
Nesterenkonia_massiliensis_NP1	0	0	0	-29.24937886	-10.646224	-1000	0	0	0
Nevskia_ramosa_DSM_11499	0	0	0	-1000	0	0	-1000	-875.5746	0
Nigerium_massiliense_SIT5	0	0	0	-1000	-16.061777	0	0	-543.99373	0
Nocardioides_massiliensis_GD13	0	0	0	-1000	-11.18261	0	0	0	0
Nosocomiicoccus_massiliensis_NP2	0	0	0	-3.221777362	-9.95276	0	0	0	0
Noviherbaspirillum_massiliense_JC206	0	0	0	-1000	-666.66667	0	0	0	0
Novosphingobium_aromaticivorans_DSM_12444	0	0	0	0	0	0	0	-617.15089	0
Numidum_massiliense_mt3	0	0	0	-1000	-20.315066	-1000	0	0	0
Obesumbacterium_proteus_ATCC_12841	0	0	-1000	-1000	-26.552602	-1000	0	0	0
Obesumbacterium_proteus_DSM_2777	0	0	-1000	-1000	-26.579898	-1000	0	0	0
Oceanobacillus_caeni_HM6	0	0	0	-1000	-32.124497	-1000	0	0	0
Oceanobacillus_jeddahense_S5	0	0	0	-1000	-21.829171	-1000	0	-1000	0
Oceanobacillus_massiliensis_str_N_diop	0	0	0	-38.38680624	-15.265318	-1000	0	-850.53777	0
Oceanobacillus_picturae_strain_S1	0	0	0	-1000	-18.931389	-1000	0	0	0
Ochrobactrum_anthropi_ATCC_49188	0	0	0	-1000	-1000	-1000	0	0	0
Ochrobactrum_anthropi_CTS_325	0	0	0	-1000	-1000	-1000	0	0	0
Ochrobactrum_anthropi_FRAF13	0	0	0	-1000	-1000	-1000	0	0	0
Ochrobactrum_intermedium_LMG_3301	0	0	0	-1000	-1000	-1000	0	0	0
Odoribacter_laneus_YIT_12061	0	0	0	-1000	-1000	0	0	0	0
Odoribacter_splanchnicus_1651_6_DSM_20712	0	0	0	-1000	-1000	0	0	0	0
Odoribacter_splanchnicus_ERR1203973	0	0	0	-1000	-1000	0	0	0	0
Odoribacter_splanchnicus_ERR1204067	0	0	0	-1000	-1000	0	0	0	0
Odoribacter_splanchnicus_ERR2230125	0	0	0	-1000	-1000	0	0	0	0
Oligella_urethralis_DNF00040	0	0	0	-1000	-1000	0	-1000	0	0
Oligella_urethralis_DSM_7531	0	0	0	-1000	-1000	0	-1000	0	0
Oligella_urethralis_UMB0345_21837_8_92_1	0	0	0	-1000	-1000	0	-1000	0	0
Olsenella_profusa_F019501	0	0	0	0	-35.544568	-1000	0	-1000	0
Olsenella_uli_DSM_7084	0	0	0	0	-43.989697	0	0	-781.6092	0
Oribacterium_sinus_F0268	0	0	0	-26.5937703	-21.534718	0	0	-900	0
Oribacterium_sp_oral_taxon_078_str_F0262	0	0	0	-27.60744259	-27.203947	0	0	-750	0
Oscillibacter_nov_ERR1022355	0	0	0	-59.65362927	-29.946936	0	0	0	0
Oscillibacter_nov_ERR1022407	0	0	0	-777.7188329	-25.52839	0	0	0	0
Oscillibacter_ruminantium_GH1	0	0	0	-37.52359608	-17.700215	0	0	0	0
Oscillibacter_sp_KLE_1728	0	0	0	-46.82190634	-24.032154	0	0	0	0
Oscillibacter_sp_KLE_1745	0	0	0	-65.86886533	-33.808335	0	0	0	0
Oxalobacter_formigenes_HOxBLS	0	0	0	-1000	-12.237386	0	0	0	0
Oxalobacter_formigenes_OXCC13	0	0	0	0	0	0	0	0	0
Paenibacillus_alvei_DSM_29	0	0	0	-1000	-26.29777	0	0	0	0
Paenibacillus_antibioticophila_GD11	0	0	0	-1000	-17.193515	-1000	0	-768.37052	0
Paenibacillus_barengoltzii_G22	0	0	0	0	-22.680222	-1000	0	-1000	0
Paenibacillus_daejeonensis_DSM_15491	0	0	0	0	0	-1000	0	0	0
Paenibacillus_graminis_RSA19	0	0	0	0	-17.480235	-1000	0	-1000	0
Paenibacillus_ihuae_GD6	0	0	0	0	-16.667426	-1000	0	-1000	0
Paenibacillus_lactis_154	0	0	0	0	-16.068629	-1000	0	-1000	0
Paenibacillus_senegalensis_JC66	0	0	0	0	-13.269878	0	0	0	0
Paenibacillus_sp_HGF5	0	0	0	0	-28.399186	-1000	0	-762.70185	0
Paenibacillus_sp_HGF7	0	0	0	0	0	0	0	0	0
Paenibacillus_sp_HGH0039	0	0	0	0	0	0	0	0	0
Paenibacillus_sp_HW567	0	0	0	0	-29.552574	-1000	0	-1000	0
Paenibacillus_sp_ICGEB2008	0	0	0	-1000	-22.140041	-1000	0	-916.66667	0
Paenibacillus_sp_JDR_2	0	0	0	0	-35.225835	-1000	0	-760.95391	0
Paeniclostridium_sordellii_8483	0	0	0	-1000	-1000	0	0	0	0
Paeniclostridium_sordellii_AF05_25	0	0	0	-1000	-1000	0	0	0	0
Paeniclostridium_sordellii_AM370	0	0	0	-1000	-1000	0	0	0	0
Paeniclostridium_sordellii_ATCC_9714	0	0	0	-1000	-1000	0	0	0	0
Paeniclostridium_sordellii_CBA7122	0	0	0	-1000	-1000	0	0	0	0
Paeniclostridium_sordellii_DA108	0	0	0	-1000	-1000	0	0	0	0
Paeniclostridium_sordellii_E204	0	0	0	-1000	-1000	0	0	0	0
Paeniclostridium_sordellii_JGS444	0	0	0	-1000	-1000	0	0	0	0
Paeniclostridium_sordellii_JGS445	0	0	0	-1000	-1000	0	0	0	0
Paeniclostridium_sordellii_JGS6956	0	0	0	-1000	-1000	0	0	0	0
Paeniclostridium_sordellii_JGS6961	0	0	0	-1000	-1000	0	0	0	0
Paeniclostridium_sordellii_MGYG_HGUT_00157	0	0	0	-1000	-1000	0	0	0	0
Paeniclostridium_sordellii_R15892	0	0	0	-1000	-1000	0	0	0	0
Paeniclostridium_sordellii_R26833	0	0	0	-1000	-1000	0	0	0	0
Paeniclostridium_sordellii_R27882	0	0	0	-1000	-1000	0	0	0	0
Paeniclostridium_sordellii_R28058	0	0	0	-1000	-1000	0	0	0	0
Paeniclostridium_sordellii_R29426	0	0	0	-1000	-1000	0	0	0	0
Paeniclostridium_sordellii_R30684	0	0	0	-1000	-1000	0	0	0	0
Paeniclostridium_sordellii_R31809	0	0	0	-1000	-1000	0	0	0	0
Paeniclostridium_sordellii_R32462	0	0	0	-1000	-1000	0	0	0	0
Paeniclostridium_sordellii_R32668	0	0	0	-1000	-1000	0	0	0	0
Paeniclostridium_sordellii_R32921	0	0	0	-1000	-1000	0	0	0	0
Paeniclostridium_sordellii_R32977	0	0	0	-1000	-1000	0	0	0	0

Paeniclostridium_sordellii_SSCC18392	0	0	0	-1000	-1000	0	0	0	0
Paeniclostridium_sordellii_SSCC18838	0	0	0	-1000	-1000	0	0	0	0
Paeniclostridium_sordellii_SSCC26591	0	0	0	-1000	-1000	0	0	0	0
Paeniclostridium_sordellii_SSCC32135	0	0	0	-1000	-1000	0	0	0	0
Paeniclostridium_sordellii_SSCC33587	0	0	0	-1000	-1000	0	0	0	0
Paeniclostridium_sordellii_SSCC33589	0	0	0	-1000	-1000	0	0	0	0
Paeniclostridium_sordellii_SSCC35109	0	0	0	-1000	-1000	0	0	0	0
Paeniclostridium_sordellii_SSCC37615	0	0	0	-1000	-1000	0	0	0	0
Paeniclostridium_sordellii_SSCC42239	0	0	0	-1000	-1000	0	0	0	0
Paeniclostridium_sordellii_UMC1	0	0	0	-1000	-1000	0	0	0	0
Paeniclostridium_sordellii_UMC164	0	0	0	-1000	-1000	0	0	0	0
Paeniclostridium_sordellii_UMC178	0	0	0	-1000	-1000	0	0	0	0
Paeniclostridium_sordellii_UMC4401	0	0	0	-1000	-1000	0	0	0	0
Paeniclostridium_sordellii_UMC4404	0	0	0	-1000	-1000	0	0	0	0
Paeniclostridium_sordellii_VPI_9048	0	0	0	-1000	-1000	0	0	0	0
Paeniclostridium_sordellii_W10	0	0	0	-1000	-1000	0	0	0	0
Paeniclostridium_sordellii_W2922	0	0	0	-1000	-1000	0	0	0	0
Paeniclostridium_sordellii_W2945	0	0	0	-1000	-1000	0	0	0	0
Paeniclostridium_sordellii_W2946	0	0	0	-1000	-1000	0	0	0	0
Paeniclostridium_sordellii_W2948	0	0	0	-1000	-1000	0	0	0	0
Paeniclostridium_sordellii_W2967	0	0	0	-1000	-1000	0	0	0	0
Paeniclostridium_sordellii_W2975	0	0	0	-1000	-1000	0	0	0	0
Paeniclostridium_sordellii_W3025	0	0	0	-1000	-1000	0	0	0	0
Paeniclostridium_sordellii_W3026	0	0	0	-1000	-1000	0	0	0	0
Paenisporosarcina_sp_HGH0030	0	0	0	0	-21.953375	0	0	0	0
Paenisporosarcina_sp_TG_14	0	0	0	-54.83746029	-22.203549	0	0	0	0
Paludibacter_jiangxiensis_NM7	0	0	-1000	0	0	0	0	-722.35294	0
Paludibacter_propionicigenes_WB4	0	-1000	-969.2307692	0	-18.515681	0	0	0	0
Pantoea_agglomerans_190	0	0	-1000	-1000	-39.24379	-1000	0	-1000	0
Pantoea_agglomerans_299R	0	0	-1000	-1000	-41.931951	-1000	0	-1000	0
Pantoea_agglomerans_3	0	0	-1000	-1000	-39.243842	-1000	0	-1000	0
Pantoea_agglomerans_4	0	0	-1000	-1000	-39.243816	-1000	0	-1000	0
Pantoea_agglomerans_IG1	0	0	-1000	-1000	-60.728963	-1000	0	-1000	0
Pantoea_agglomerans_MP2	0	0	-1000	-1000	-39.243839	-1000	0	-1000	0
Pantoea_agglomerans_P10c	0	0	-1000	-1000	-39.243791	-1000	0	-1000	0
Pantoea_sp_YR343	0	0	-1000	-1000	-39.243642	-1000	0	0	0
Papillibacter_cinnamivorans_DSM_12816	0	0	0	0	-8.0863094	0	0	0	0
Parabacteroides_chinchillae_DSM_29073	0	0	0	0	-16.341244	0	0	-516.64379	0
Parabacteroides_distasonis_ATCC_8503	0	0	-1000	0	-1000	0	0	-1000	0
Parabacteroides_distasonis_CL03T12C09	0	0	-1000	-1000	-1000	0	0	-1000	0
Parabacteroides_distasonis_CL09T03C24	0	0	-1000	-1000	-1000	0	0	-1000	0
Parabacteroides_distasonis_ERR1022281	0	0	-1000	-1000	-1000	0	0	-1000	0
Parabacteroides_distasonis_ERR1022330	0	0	-1000	-1000	-1000	0	0	-1000	0
Parabacteroides_distasonis_ERR1022415	0	0	-1000	-1000	-1000	0	0	-1000	0
Parabacteroides_distasonis_ERR1022462	0	0	-1000	-1000	-1000	0	0	-1000	0
Parabacteroides_distasonis_ERR1204048	0	0	-1000	-1000	-1000	0	0	-1000	0
Parabacteroides_distasonis_ERR2221129	0	0	-1000	-1000	-1000	0	0	-1000	0
Parabacteroides_distasonis_ERR2221168	0	0	-1000	-1000	-1000	0	0	-1000	0
Parabacteroides_distasonis_ERR2230146	0	0	-1000	-1000	-1000	0	0	-1000	0
Parabacteroides_distasonis_ERR2230165	0	0	-1000	-1000	-1000	0	0	-1000	0
Parabacteroides_goldsteinii_CL02T12C30	0	0	-1000	0	-1000	0	0	-1000	0
Parabacteroides_goldsteinii_dnLKV18	0	0	-1000	0	-1000	0	0	-1000	0
Parabacteroides_goldsteinii_DSM_19448_WAL_1	0	0	-1000	0	-1000	0	0	-1000	0
Parabacteroides_goldsteinii_ERR2230163	0	0	-1000	0	-1000	0	0	-637.56614	0
Parabacteroides_gordonii_DSM_23371	0	0	-1000	0	0	0	0	-1000	0
Parabacteroides_johnsonii_CL02T12C29	0	0	-1000	0	0	0	0	-1000	0
Parabacteroides_johnsonii_DSM_18315	0	0	-1000	0	0	0	0	-503.06748	0
Parabacteroides_merdae_ATCC_43184	0	0	-1000	0	-1000	0	0	-762.64151	0
Parabacteroides_merdae_CL03T12C32	0	0	-1000	0	-1000	0	0	-1000	0
Parabacteroides_merdae_CL09T00C40	0	0	-1000	0	-1000	0	0	-613.13869	0
Parabacteroides_merdae_ERR1022364	0	0	-1000	0	-1000	0	0	-613.13869	0
Parabacteroides_merdae_ERR2230111	0	0	-1000	0	-1000	0	0	-670.73171	0
Parabacteroides_nov_ERR1203954	0	0	0	-1000	-31.723489	0	0	0	0
Parabacteroides_sp_ASF519	0	0	-1000	0	-32.168386	0	0	0	0
Parabacteroides_sp_D13	0	0	0	0	0	0	0	0	0
Parabacteroides_sp_D25	0	0	0	-1000	-30.570726	0	0	0	0
Parabacteroides_sp_D26	0	0	0	-1000	-30.43622	0	0	0	0
Paraburkholderia_sediminicola_LMG_24238	0	0	0	-1000	-17.895907	0	0	0	0
Paraclostridium_bifermentans_ATCC_19299	0	0	0	-1000	-1000	-1000	0	0	0
Paraclostridium_bifermentans_ATCC_638	0	0	0	-1000	-1000	-1000	0	0	0
Paraclostridium_bifermentans_Cbm	0	0	0	-1000	-1000	-1000	0	0	0
Paraclostridium_bifermentans_MGYG_HGUT_000	0	0	0	-1000	-1000	-1000	0	0	0
Paraclostridium_bifermentans_MHMC_14	0	0	0	-1000	-1000	-1000	0	0	0
Paraclostridium_bifermentans_SU1074NT	0	0	0	-1000	-1000	-1000	0	0	0
Paraclostridium_bifermentans_WYM	0	0	0	-1000	-1000	-1000	0	0	0
Paracoccus_yeei_ATCC_BAA_599	0	0	0	-1000	-39.361987	0	0	0	0
Paraeggerthella_hongkongensis_RC2_2	0	0	0	0	-18.857209	0	0	0	0
Parapedobacter_koreensis_Jip14	0	0	-1000	-23.36576161	0	-1000	0	-1000	0
Paraprevotella_clara_YIT_11840	0	0	-1000	0	0	0	0	0	0
Paraprevotella_xylaniphila_YIT_11841	0	0	-709.5652174	0	0	0	0	0	0
Parascardovia_denticolens_DSM_10105_JCM_12	0	0	0	0	-19.994696	0	0	-522.93843	0
Parascardovia_denticolens_F0305	0	0	0	0	-19.994657	0	0	-522.93834	0
Parascardovia_denticolens_IPLA_20019	0	0	0	0	-18.473186	0	0	-522.91155	0
Parasporobacterium_paucivorans_DSM_15970	0	0	0	0	-8.0999944	0	0	0	0
Parasutterella_excrementihominis_YIT_11859	0	0	0	-1000	0	0	0	0	0
Parvibacter_caecicola_DSM_22242	0	0	0	-1000	-9.7468864	0	0	0	0
Parvibacter_caecicola_NM48_B13	0	0	0	-1000	-9.742673	0	0	0	0
Parvimonas_micra_ATCC_33270	0	0	0	-1000	-7.2381883	0	0	0	0
Parvimonas_sp_oral_taxon_110_str_F0139	0	0	0	-18.21877815	-17.99554	0	0	0	0
Pasteurella_caecimuris_NM44_TS2_9	0	0	0	-56.08671591	-37.870619	0	-1000	-833.33333	0
Pediococcus_acidilactici_7_4	0	0	0	-181.8181818	-16.016151	0	0	0	0

Pediococcus_acidilactici_D3	0	0	0	-370.0787402	-15.903849	0	0	0	0
Pediococcus_acidilactici_DSM_20284	0	0	0	-181.8181818	-15.979701	0	0	0	0
Pediococcus_acidilactici_ERR2221169	0	0	0	-293.1034483	-25.870485	0	0	0	0
Pediococcus_acidilactici_ERR2221292	0	0	0	-311.9266055	-21.321499	0	0	0	0
Pediococcus_acidilactici_ERR2221293	0	0	0	-266.6666667	-23.980514	0	0	0	0
Pediococcus_acidilactici_ERR2221294	0	0	0	-311.9266055	-21.320481	0	0	0	0
Pediococcus_acidilactici_ERR2221295	0	0	0	-311.9266055	-21.324552	0	0	0	0
Pediococcus_acidilactici_ERR2221299	0	0	0	-293.1034483	-26.016057	0	0	0	0
Pediococcus_acidilactici_ERR2221316	0	0	0	-266.6666667	-19.425543	0	0	0	0
Pediococcus_acidilactici_ERR2221333	0	0	0	-238.0952381	-22.219672	0	0	0	0
Pediococcus_acidilactici_ERR2221334	0	0	0	-238.0952381	-22.219672	0	0	0	0
Pediococcus_acidilactici_ERR2221382	0	0	0	-238.0952381	-22.219672	0	0	0	0
Pediococcus_acidilactici_MA18_5M	0	0	0	-272.7272727	-16.228133	0	0	0	0
Pediococcus_pentosaceus_ATCC_25745	0	0	0	-1000	-18.832393	0	0	-918.51852	0
Pediococcus_pentosaceus_IE_3	0	0	0	-1000	-15.586141	0	0	0	0
Pelomonas_saccharophila_DSM_654	0	0	0	-1000	-1000	0	-1000	-1000	0
Peptoniphilus_coxii_DNF00729	0	0	0	-1000	-5.0768949	0	0	-3.6412995	0
Peptoniphilus_duerdenii_ATCC_BAA_1640	0	0	0	-1000	-14.248775	0	0	0	0
Peptoniphilus_grossensis_35_6_1	0	0	0	-1000	-17.916044	0	0	0	0
Peptoniphilus_harei_ACS_146_V_Sch2b	0	0	0	-1000	-11.684788	0	0	0	0
Peptoniphilus_harei_ERR2221371	0	0	0	-1000	-14.537584	0	0	0	0
Peptoniphilus_indolicus_ATCC_29427	0	0	0	-1000	-12.057029	0	0	0	0
Peptoniphilus_lacrimalis_315_B	0	0	0	-1000	-10.122875	0	0	-7.2604262	0
Peptoniphilus_lacrimalis_DSM_7455	0	0	0	-1000	-8.6542553	0	0	0	0
Peptoniphilus_nov_ERR2221395	0	0	0	-1000	-15.885758	0	0	0	0
Peptoniphilus_obesi_ph1	0	0	0	-1000	-10.800215	0	0	0	0
Peptoniphilus_senegalensis_JC140	0	0	0	-1000	-15.002556	0	0	0	0
Peptoniphilus_timonensis_JC401	0	0	0	-1000	-6.0374988	0	0	0	0
Peptostreptococcus_anaerobius_653_L	0	0	0	-522.3880597	-22.923729	0	0	0	0
Peptostreptococcus_anaerobius_DSM_2949	0	0	0	-29.61669679	-1000	0	0	0	0
Peptostreptococcus_anaerobius_VPI_4330	0	0	0	-1000	-23.741811	0	0	0	0
Peptostreptococcus_russellii_Calf135	0	0	0	-1000	-17.082097	0	0	0	0
Peptostreptococcus_stomatis_DSM_17678	0	0	0	-1000	-1000	0	0	0	0
Phascolarctobacterium_faecium_DSM_14760	0	0	0	-1000	-10.079924	0	0	0	0
Phascolarctobacterium_succinatutens_YIT_12067	0	0	0	-1000	-13.552218	0	0	0	0
Phocaeicola_abscessus_CCUG_55929	0	0	-1000	-1000	-15.169321	0	-1000	0	0
Photobacterium_angustum_S14	0	0	0	-1000	-38.343521	0	0	0	0
Photobacterium_damselae_Phdp_Wu_1	0	0	0	-1000	-59.969071	0	0	-1000	0
Photobacterium_damselae_subsp_damselae_CIP_	0	0	0	-1000	-59.956984	0	0	0	0
Photobacterium_damselae_subsp_damselae_KC_	0	0	0	-1000	-60.051794	0	0	0	0
Photobacterium_leiognathi_subsp_mandapamen	0	0	0	-1000	-31.233351	0	0	0	0
Photobacterium_sp_SKA34	0	0	0	-1000	-38.343552	0	0	-1000	0
Photorhabdus_luminescens_BA1	0	0	0	-1000	-21.815107	-1000	0	0	0
Photorhabdus_luminescens_H1_H1_C1	0	0	0	-1000	-22.83047	-1000	0	0	0
Photorhabdus_luminescens_NBAII_H75HRPL105	0	0	0	-1000	-22.830411	-1000	0	0	0
Pirellula_staleyi_DSM_6068	0	0	-1000	-440	-15.226822	0	-1000	-458.33333	0
Plesiomonas_shigelloides_302_73	0	0	0	-1000	-54.408079	0	0	0	0
Plesiomonas_shigelloides_GN7	0	0	0	-1000	-41.006865	0	0	0	0
Polynesia_massiliensis_MS3	0	0	0	-33.78134057	-16.042142	0	0	-958.33333	0
Porphyromonadaceae_nov_ERR2221201	0	0	-736.3013699	0	-27.137981	0	0	0	0
Porphyromonadaceae_nov_ERR2221202	0	0	-700.7575758	-46.19736134	-27.011012	0	0	0	0
Porphyromonadaceae_nov_ERR2221365	0	0	0	0	-30.083693	0	0	0	0
Porphyromonadaceae_nov_ERR2221374	0	0	0	-1000	-30.768171	0	0	0	0
Porphyromonadaceae_nov_ERR2221378	0	0	0	-1000	-30.869393	0	0	0	0
Porphyromonas_asaccharolytica_DSM_20707	0	0	0	0	-1000	0	0	0	0
Porphyromonas_asaccharolytica_PR426713P_I	0	0	0	0	-1000	0	0	0	0
Porphyromonas_bennonis_DSM_23058_JCM_163	0	0	0	0	-9.7658781	0	0	0	0
Porphyromonas_endodontalis_ATCC_35406	0	0	0	0	-1000	0	0	0	0
Porphyromonas_gingivalis_A7436	0	0	0	0	-1000	0	0	0	0
Porphyromonas_gingivalis_ATCC_33277	0	0	0	0	-1000	0	0	0	0
Porphyromonas_gingivalis_TDC60	0	0	0	0	-1000	0	0	0	0
Porphyromonas_gingivalis_W83	0	0	0	0	-1000	0	0	0	0
Porphyromonas_gingivicanis_JCM_15907	0	0	0	0	-1000	0	0	0	0
Porphyromonas_somerae_DSM_23386	0	0	0	0	-1000	0	0	0	0
Porphyromonas_uenonis_60_3	0	0	0	0	-1000	0	0	0	0
Porphyromonas_uenonis_DSM_23387	0	0	0	0	-1000	0	0	0	0
Prevotella_albensis_DSM_11370	0	0	-1000	0	-1000	0	0	0	0
Prevotella_amnii_CRIS_21A_A	0	0	0	0	-3.0661948	0	0	0	0
Prevotella_amnii_DNF00058	0	0	0	0	-19.691129	0	0	0	0
Prevotella_amnii_DNF00307	0	0	0	0	-3.0814272	0	0	0	0
Prevotella_amnii_DSM_23384_JCM_14753	0	0	0	0	-3.0786271	0	0	0	0
Prevotella_aurantiaca_JCM_15754	0	0	0	0	-15.644414	0	0	-254.54138	0
Prevotella_baroniae_DSM_16972_JCM_13447	0	0	0	0	-2.3916155	0	0	-570.17544	0
Prevotella_baroniae_F0067	0	0	0	0	-2.4198109	0	0	-570.17544	0
Prevotella_bergensis_DSM_17361	0	0	-524.9439462	0	-10.402395	0	0	0	0
Prevotella_bivia_DSM_20514	0	0	0	0	-38.143868	0	0	0	0
Prevotella_bivia_JCVIHMP010	0	0	0	0	0	0	0	0	0
Prevotella_brevis_ATCC_19188	0	0	-1000	-1000	-1000	0	0	-1000	0
Prevotella_bryantii_B14	0	0	-1000	-85.05598622	-1000	0	0	-1000	0
Prevotella_bryantii_C21a	0	0	-1000	-48.26928799	-1000	0	0	-1000	0
Prevotella_buccae_ATCC_33574	0	0	-1000	0	-1000	0	0	-1000	0
Prevotella_buccae_D17	0	0	-1000	0	-1000	0	0	-1000	0
Prevotella_buccalis_ATCC_35310	0	0	0	0	-15.185149	0	0	-255.61906	0
Prevotella_buccalis_DNF00853	0	0	-260.8695652	0	-10.193393	0	0	-430.85106	0
Prevotella_buccalis_DNF00985	0	0	-261.3636364	0	-10.424315	0	0	-449.76148	0
Prevotella_buccalis_UMB0536_16933_8_22_1	0	0	0	0	-18.509621	0	0	-257.91063	0
Prevotella_conceptionensis_9403948	0	0	0	0	-36.807737	0	0	0	0
Prevotella_copri_CB7_DSM_18205	0	0	-1000	-1000	0	0	0	-852.0841	0
Prevotella_copri_ERR1022397	0	0	0	-1000	0	0	0	-587.57764	0
Prevotella_corporis_DSM_18810	0	0	0	0	-1000	0	0	0	0
Prevotella_corporis_MJR7716	0	0	0	0	-1000	0	0	0	0

Prevotella_dentalis_DSM_3688	0	0	-679.7979798	0	-22.662479	0	0	-270.89122	0
Prevotella_dentasini_JCM_15908	0	0	0	0	-12.519597	0	0	-393.76054	0
Prevotella_denticola_CRIS_18C_A	0	0	0	0	-1000	0	0	-1000	0
Prevotella_denticola_DSM20614	0	0	0	0	-1000	0	0	-1000	0
Prevotella_denticola_F0289	0	0	0	0	-1000	0	0	-1000	0
Prevotella_disiens_FB035_09AN	0	0	0	0	-1000	0	0	0	0
Prevotella_disiens_JCM_6334	0	0	0	0	-1000	0	0	0	0
Prevotella_enoeca_JCM_12259	0	0	0	0	-32.50374	0	0	0	0
Prevotella_histicola_F0411	0	0	0	0	-1000	0	0	-1000	0
Prevotella_histicola_JCM_15637_DNF00424	0	0	0	0	-1000	0	0	-1000	0
Prevotella_intermedia_17	0	0	0	0	-1000	0	0	-965.98639	0
Prevotella_intermedia_ATCC_25611	0	0	0	0	-1000	0	0	-901.97287	0
Prevotella_loescheii_DSM_19665	0	0	0	0	-34.332302	0	0	-934.82049	0
Prevotella_maculosa_DSM_19339_JCM_15638	0	0	-1000	0	-15.183848	0	0	-1000	0
Prevotella_maculosa_OT_289	0	0	-1000	0	-15.183766	0	0	-1000	0
Prevotella_melaninogenica_ATCC_25845	0	0	0	0	-1000	0	0	-1000	0
Prevotella_melaninogenica_D18	0	0	0	0	-1000	0	0	-1000	0
Prevotella_micans_DSM_21469_JCM_16134	0	0	0	0	-1000	0	0	-7.9020286	0
Prevotella_micans_F0438	0	0	0	0	-1000	0	0	-515.09207	0
Prevotella_multiformis_DSM_16608	0	0	0	0	-29.130506	0	0	-572.16495	0
Prevotella_multisaccharivorax_DSM_17128	0	0	0	0	-20.753559	0	0	-258.15034	0
Prevotella_nanceiensis_DSM_19126	0	0	0	-36.36007619	-35.443803	0	0	-276.34014	0
Prevotella_nigrescens_ATCC_33563	0	0	0	0	-1000	0	0	-980.43958	0
Prevotella_nigrescens_F0103	0	0	0	0	-1000	0	0	-512.94176	0
Prevotella_oralis_ATCC_33269	0	0	0	0	-1000	0	0	-1000	0
Prevotella_oralis_HGA0225	0	0	0	0	-1000	0	0	-1000	0
Prevotella_oris_C735	0	0	-1000	0	-25.766584	0	0	-508.72017	0
Prevotella_oris_DSM_18711_JCM_12252	0	0	-683.3333333	0	-29.102571	0	0	-502.6097	0
Prevotella_oris_F0302	0	0	-1000	-1000	-30.273189	0	0	-497.08804	0
Prevotella_oulorum_ATCC_43324	0	0	0	0	-21.71477	0	0	0	0
Prevotella_oulorum_F0390	0	0	0	0	-17.863922	0	0	0	0
Prevotella_oulorum_JCM_14966	0	0	0	0	-10.898971	0	0	0	0
Prevotella_pallens_ATCC_700821	0	0	0	0	-1000	0	0	0	0
Prevotella_pleuritidis_F0068	0	0	0	0	-33.983277	0	0	0	0
Prevotella_pleuritidis_JCM_14110	0	0	0	0	-20.02209	0	0	0	0
Prevotella_ruminicola_23	0	0	-1000	0	-1000	0	0	-1000	0
Prevotella_salivae_DSM_15606	0	0	-1000	0	-1000	0	0	-901.36054	0
Prevotella_shahii_JCM_12083	0	0	0	0	-33.016297	0	0	-571.10778	0
Prevotella_sp_C561	0	0	0	0	-24.236263	0	0	0	0
Prevotella_sp_oral_taxon_473_str_F0040	0	0	0	0	-20.626369	0	0	0	0
Prevotella_stercorea_DSM_18206	0	0	0	-1000	-1000	0	0	-784.76821	0
Prevotella_timonensis_4401737	0	0	0	0	-22.618307	0	0	0	0
Prevotella_timonensis_CRIS_5C_B1	0	0	0	0	-24.223104	0	0	0	0
Prevotella_veroralis_DSM_19559	0	0	0	0	-1000	0	0	-793.91574	0
Prevotella_veroralis_F0319	0	0	0	0	-1000	0	0	-1000	0
Propionibacterium_acidifaciens_DSM_21887	0	0	0	-1000	-21.405229	0	0	-1000	0
Propionibacterium_acidifaciens_F0233	0	0	0	-1000	-20.225429	0	0	-791.66667	0
Propionibacterium_acidipropionici_ATCC_4875	0	0	0	0	-1000	-1000	0	-534.55186	0
Propionibacterium_avidum_44067	0	0	-1000	0	-1000	0	0	-1000	0
Propionibacterium_avidum_ATCC_25577	0	0	0	0	-1000	0	0	-1000	0
Propionibacterium_freudenreichii_ERR2221306	0	0	0	-1000	-37.666622	0	0	0	0
Propionibacterium_freudenreichii_subsp_sherma	0	0	0	-1000	-1000	0	0	0	0
Propionibacterium_granulosum_DSM_20700	0	0	0	0	-1000	0	0	-759.25926	0
Propionibacterium_jensenii_DSM_20535	0	0	0	0	-1000	-1000	0	-1000	0
Propionibacterium_namnetense_NTS_31307302	0	0	0	0	-15.246024	0	-1000	-630.95238	0
Propionibacterium_namnetense_SK182B_JCVI	0	0	0	0	-17.614056	0	-1000	-627.90698	0
Propionibacterium_propionicum_F0230a	0	0	0	0	-1000	-1000	0	-1000	0
Propionibacterium_sp_409_HC1	0	0	-1000	0	-34.043983	0	0	0	0
Propionibacterium_sp_5_U_42AFAA	0	0	-1000	0	-28.529251	0	0	0	0
Propionibacterium_sp_HGH0353	0	0	0	0	-38.162702	0	0	0	0
Proteiniphilum_acetatigenes_DSM_18083	0	0	-1000	-1000	-22.010034	0	-1000	0	0
Proteus_mirabilis_AOUC_001	0	0	0	-1000	-40.201719	0	0	-1000	0
Proteus_mirabilis_ATCC_29906	0	0	0	-1000	-54.273522	0	0	-1000	0
Proteus_mirabilis_C05028	0	0	0	-1000	-38.879715	0	0	-1000	0
Proteus_mirabilis_CYPM1	0	0	0	-1000	-40.201719	0	0	-1000	0
Proteus_mirabilis_HI4320	0	0	0	-1000	-40.227644	0	0	-1000	0
Proteus_mirabilis_PR03	0	0	0	-1000	-38.879715	0	0	-1000	0
Proteus_mirabilis_WGLW4	0	0	0	-1000	-38.876697	0	0	-1000	0
Proteus_mirabilis_WGLW6	0	0	0	-1000	-38.882733	0	0	-1000	0
Proteus_penneri_ATCC_35198	0	0	0	-1000	-55.50123	0	0	-1000	0
Proteus_vulgaris_08MAS1600	0	0	0	-1000	-39.599393	0	0	-1000	0
Proteus_vulgaris_ATCC_49132	0	0	0	-1000	-39.604665	0	0	-1000	0
Proteus_vulgaris_CICC	0	0	0	-1000	-39.604662	0	0	-1000	0
Proteus_vulgaris_CSUR_P1867	0	0	0	-1000	-39.602026	0	0	-1000	0
Proteus_vulgaris_CSUR_P1868	0	0	0	-1000	-39.602029	0	0	-1000	0
Proteus_vulgaris_FDAARGOS_366	0	0	0	-1000	-39.604662	0	0	-1000	0
Proteus_vulgaris_FDAARGOS_556	0	0	0	-1000	-39.604665	0	0	-1000	0
Proteus_vulgaris_KCTC_2579	0	0	0	-1000	-39.678673	0	0	-1000	0
Proteus_vulgaris_MGYG_HGUT_02516	0	0	0	-1000	-39.678673	0	0	-1000	0
Proteus_vulgaris_NCTC10376	0	0	0	-1000	-39.604665	0	0	-1000	0
Proteus_vulgaris_NCTC13145	0	0	0	-1000	-39.604665	0	0	-1000	0
Proteus_vulgaris_NCTC401	0	0	0	-1000	-39.602026	0	0	-1000	0
Providencia_alcalifaciens_Dmel2	0	0	0	-1000	-39.670117	0	0	-1000	0
Providencia_alcalifaciens_DSM_30120	0	0	0	-1000	-52.670091	0	0	0	0
Providencia_burhodogranariea_DSM_19968	0	0	-1000	-1000	-45.538959	-1000	0	-1000	0
Providencia_rettgeri_Dmel1	0	0	0	-1000	-43.536803	-1000	0	-1000	0
Providencia_rettgeri_DSM_1131	0	0	0	-1000	-50.838619	-1000	0	0	0
Providencia_rustigianii_DSM_4541	0	0	0	-1000	-51.44198	0	0	-1000	0
Providencia_sneebia_DSM_19967	0	0	0	-1000	-35.697749	-1000	0	-1000	0
Providencia_stuartii_ATCC_25827	0	0	0	-1000	-47.919941	0	0	0	0
Providencia_stuartii_FDAARGOS_87	0	0	0	-1000	-45.656919	0	0	-1000	0

Providencia_stuartii_MRSN_2154	0	0	0	-1000	-34.517391	0	0	-1000	0
Pseudoalteromonas_arctica_A_37_1_2	0	0	0	-1000	0	-1000	0	-1000	0
Pseudoalteromonas_arctica_MelAa3	0	0	0	-1000	0	-1000	0	-1000	0
Pseudobutyrivibrio_ruminis_AD2017	0	0	0	-1000	-18.405247	0	0	-824.23307	0
Pseudoflavonifractor_capillosus_ERR1022447	0	0	0	0	-23.853744	0	0	-599.02218	0
Pseudoflavonifractor_capillosus_strain_ATCC_297	0	0	0	-43.14262175	-17.149165	0	0	-500	0
Pseudomonas_aeruginosa_152504	0	0	0	-1000	-1000	-1000	-1000	0	0
Pseudomonas_aeruginosa_18A	0	0	0	-1000	-1000	-1000	-1000	0	0
Pseudomonas_aeruginosa_19BR	0	0	0	-1000	-1000	-1000	-1000	0	0
Pseudomonas_aeruginosa_213BR	0	0	0	-1000	-1000	-1000	-1000	0	0
Pseudomonas_aeruginosa_2192	0	0	0	-1000	-1000	-1000	-1000	0	0
Pseudomonas_aeruginosa_39016	0	0	0	-1000	-1000	-1000	-1000	0	0
Pseudomonas_aeruginosa_9BR	0	0	0	-1000	-1000	-1000	-1000	0	0
Pseudomonas_aeruginosa_AH16	0	0	0	-1000	-1000	-1000	-1000	0	0
Pseudomonas_aeruginosa_ATCC_14886	0	0	0	-1000	-1000	-1000	-1000	0	0
Pseudomonas_aeruginosa_B136_33	0	0	0	-1000	-1000	-1000	-1000	0	0
Pseudomonas_aeruginosa_C3719	0	0	0	-1000	-1000	-1000	-1000	0	0
Pseudomonas_aeruginosa_CI27	0	0	0	-1000	-1000	-1000	-1000	0	0
Pseudomonas_aeruginosa_DK2	0	0	0	-1000	-1000	-1000	-1000	0	0
Pseudomonas_aeruginosa_DQ8	0	0	0	-1000	-1000	-1000	-1000	0	0
Pseudomonas_aeruginosa_E2	0	0	0	-1000	-1000	-1000	-1000	0	0
Pseudomonas_aeruginosa_F22031	0	0	0	-1000	-1000	-1000	-1000	0	0
Pseudomonas_aeruginosa_LCT_PA102	0	0	0	-1000	-1000	-1000	-1000	0	0
Pseudomonas_aeruginosa_LESB58	0	0	0	-1000	-1000	-1000	-1000	0	0
Pseudomonas_aeruginosa_M18	0	0	0	-1000	-1000	-1000	-1000	0	0
Pseudomonas_aeruginosa_MPAO1_P1	0	0	0	-1000	-1000	-1000	-1000	0	0
Pseudomonas_aeruginosa_MPAO1_P2	0	0	0	-1000	-1000	-1000	-1000	0	0
Pseudomonas_aeruginosa_MRW44_1	0	0	0	-1000	-1000	-1000	-1000	0	0
Pseudomonas_aeruginosa_MSH_10	0	0	0	-1000	-1000	-1000	-1000	0	0
Pseudomonas_aeruginosa_NCGM2_S1	-23.03449545	0	0	-1000	-1000	-1000	-1000	0	0
Pseudomonas_aeruginosa_NCMG1179	0	0	0	-1000	-1000	-1000	-1000	0	0
Pseudomonas_aeruginosa_PA14	0	0	0	-1000	-1000	-1000	-1000	0	0
Pseudomonas_aeruginosa_PA21_ST175	0	0	0	-1000	-1000	-1000	-1000	0	0
Pseudomonas_aeruginosa_PA45	0	0	0	-1000	-1000	-1000	-1000	0	0
Pseudomonas_aeruginosa_PA7	-24.21601239	0	0	-1000	-1000	-1000	-1000	0	0
Pseudomonas_aeruginosa_PABL056	0	0	0	-1000	-1000	-1000	-1000	0	0
Pseudomonas_aeruginosa_PACS2	0	0	0	-1000	-1000	-1000	-1000	0	0
Pseudomonas_aeruginosa_PADK2_CF510	0	0	0	-1000	-1000	-1000	-1000	0	0
Pseudomonas_aeruginosa_PAK	0	0	0	-1000	-1000	-1000	-1000	0	0
Pseudomonas_aeruginosa_PAO1	0	0	0	-1000	-1000	-1000	-1000	0	0
Pseudomonas_aeruginosa_PAO579	0	0	0	-1000	-1000	-1000	-1000	0	0
Pseudomonas_aeruginosa_PGPR2	0	0	0	-1000	-1000	-1000	-1000	0	0
Pseudomonas_aeruginosa_SJTD_1	0	0	0	-1000	-1000	-1000	-1000	0	0
Pseudomonas_aeruginosa_UCBPP_PA14	0	0	0	-1000	-1000	-1000	-1000	0	0
Pseudomonas_aeruginosa_XMG	0	0	0	-1000	-1000	-1000	-1000	0	0
Pseudomonas_alcaliphila_34	0	0	0	-1000	-1000	-1000	0	0	0
Pseudomonas_fluorescens_A506	0	0	0	-1000	-1000	-1000	-1000	-1000	0
Pseudomonas_fluorescens_F113	0	0	0	-1000	-1000	-1000	-1000	-1000	0
Pseudomonas_fluorescens_LMG_5329	0	0	0	-1000	-1000	-1000	-1000	-1000	0
Pseudomonas_fluorescens_NCIMB_11764	0	0	0	-1000	-1000	-1000	-1000	-1000	0
Pseudomonas_fluorescens_Pf_5	0	0	0	-1000	-22.987541	0	0	0	0
Pseudomonas_fluorescens_PfO_1	0	0	0	-1000	-1000	-1000	-1000	-1000	0
Pseudomonas_fluorescens_Q2_87	0	0	0	-1000	-1000	-1000	-1000	-1000	0
Pseudomonas_fluorescens_Q8r1_96	0	0	0	-1000	-1000	-1000	-1000	-1000	0
Pseudomonas_fluorescens_R124	0	0	0	-1000	-1000	-1000	-1000	-1000	0
Pseudomonas_fluorescens_SBW25	0	0	0	-1000	-1000	-1000	-1000	-1000	0
Pseudomonas_fluorescens_SS101	0	0	0	-1000	-1000	-1000	-1000	-1000	0
Pseudomonas_fluorescens_UK4	0	0	0	-1000	-1000	-1000	-1000	-1000	0
Pseudomonas_fluorescens_WH6	0	0	0	-1000	-1000	-1000	-1000	-1000	0
Pseudomonas_fragi_A22	0	0	0	-1000	-38.254245	0	0	0	0
Pseudomonas_fragi_B25	0	0	0	-1000	-39.036894	0	0	0	0
Pseudomonas_fragi_F1794	0	0	0	-1000	-38.350749	0	0	-1000	0
Pseudomonas_fragi_NBRC_3458	0	0	0	-1000	-38.307725	0	0	0	0
Pseudomonas_mendocina_DLHK	0	0	0	-1000	-28.490972	0	0	0	0
Pseudomonas_mendocina_EGD_AQ5	0	0	0	-1000	-1000	0	0	0	0
Pseudomonas_mendocina_NBRC_14162	0	0	0	-1000	-1000	0	0	0	0
Pseudomonas_mendocina_NEB698	0	0	0	-1000	-1000	0	0	0	0
Pseudomonas_monteilii_NBRC_103158	0	0	0	-1000	-1000	-1000	-1000	0	0
Pseudomonas_monteilii_QM	0	0	0	-1000	-1000	0	-1000	0	0
Pseudomonas_monteilii_USDA_ARS_USMARC_56	0	0	0	-1000	-1000	0	-1000	0	0
Pseudomonas_nitroreducens_HBP1	0	0	0	-1000	-1000	0	-1000	0	0
Pseudomonas_oleovorans_MOIL14HWK12	0	0	0	-1000	-27.096776	0	-1000	0	0
Pseudomonas_otitidis_LNU_E_001	0	0	0	-1000	-29.882158	0	0	0	0
Pseudomonas_psychrophila_HA_4	0	0	0	-1000	-25.57104	0	0	0	0
Pseudomonas_psychrophila_RGCB_166	0	0	0	-1000	-23.924135	0	0	0	0
Pseudomonas_putida_B001	0	0	0	-1000	-1000	0	-1000	0	0
Pseudomonas_putida_B6_2	0	0	0	-1000	-1000	0	-1000	0	0
Pseudomonas_putida_BIRD_1	0	0	0	-1000	-1000	0	-1000	0	0
Pseudomonas_putida_DLL_E4	0	0	0	-1000	-1000	0	-1000	0	0
Pseudomonas_putida_DOT_T1E	0	0	0	-1000	-1000	0	-1000	0	0
Pseudomonas_putida_F1	0	0	0	-1000	-1000	-1000	-1000	0	0
Pseudomonas_putida_GB_1	0	0	0	-1000	-1000	0	-1000	0	0
Pseudomonas_putida_H8234	0	0	0	-1000	-1000	0	-1000	0	0
Pseudomonas_putida_HB3267	0	0	0	-1000	-1000	0	-1000	0	0
Pseudomonas_putida_KT2440	0	0	0	-1000	-1000	0	-1000	0	0
Pseudomonas_putida_LS46	0	0	0	-1000	-1000	0	-1000	0	0
Pseudomonas_putida_NB2011	0	0	0	-1000	-1000	0	0	0	0
Pseudomonas_putida_NBRC_14164	0	0	0	-1000	-1000	0	-1000	0	0
Pseudomonas_putida_ND6	0	0	0	-1000	-1000	0	-1000	0	0
Pseudomonas_putida_PC2	0	0	0	-1000	-1000	0	-1000	0	0
Pseudomonas_putida_S12	0	0	0	-1000	-1000	0	-1000	0	0

Pseudomonas_putida_S16	0	0	0	-1000	-1000	0	-1000	0	0
Pseudomonas_putida_SJTE_1	0	0	0	-1000	-1000	0	-1000	0	0
Pseudomonas_putida_TRO1	0	0	0	-1000	-1000	0	-1000	0	0
Pseudomonas_putida_W619	0	0	0	-1000	-1000	0	-1000	0	0
Pseudomonas_stutzeri_28a24	0	0	0	-1000	-1000	0	-1000	0	0
Pseudomonas_stutzeri_A1501	0	0	0	-1000	-1000	0	-1000	0	0
Pseudomonas_stutzeri_ATCC_14405_CCUG_1615	0	0	0	-1000	-1000	0	-1000	0	0
Pseudomonas_stutzeri_ATCC_17588_LMG_11199	0	0	0	-1000	-36.200635	0	-1000	0	0
Pseudomonas_stutzeri_CCUG_29243	0	0	0	-1000	-1000	0	-1000	0	0
Pseudomonas_stutzeri_DSM_10701	0	0	0	-1000	-1000	0	-1000	0	0
Pseudomonas_stutzeri_DSM_4166	0	0	0	-1000	-1000	0	-1000	0	0
Pseudomonas_stutzeri_KOS6	0	0	0	-1000	-1000	0	-1000	0	0
Pseudomonas_stutzeri_NF13	0	0	0	-1000	-1000	0	-1000	0	0
Pseudomonas_stutzeri_RCH2	0	0	0	-1000	-1000	0	-1000	0	0
Pseudomonas_stutzeri_SDM_LAC	0	0	0	-1000	-30.529178	0	-1000	0	0
Pseudomonas_stutzeri_SLG510A3_8	0	0	0	-1000	-1000	0	-1000	0	0
Pseudomonas_stutzeri_T13	0	0	0	-1000	-1000	0	-1000	0	0
Pseudomonas_stutzeri_TS44	0	0	0	-1000	-1000	0	-1000	0	0
Pseudomonas_stutzeri_XLDN_R	0	0	0	-1000	-1000	0	-1000	0	0
Pseudoramibacter_alactolyticus_ATCC_23263	0	0	0	-1000	-1000	0	0	-420.06803	0
Pseudoxanthomonas_mexicana_CCH9_G4	0	0	0	-1000	0	0	0	0	0
Pyramidobacter_piscolens_W5455	0	0	0	-1000	-1000	0	0	0	0
Rahnella_aquatilis_CIP_78_65_ATCC_33071	0	0	-1000	-1000	-55.000646	-1000	0	-1000	0
Rahnella_aquatilis_DLL7529	0	0	0	-1000	-60.703406	-1000	0	-1000	0
Rahnella_aquatilis_HX2	-1000	0	-1000	-1000	-54.797725	-1000	0	-1000	0
Ralstonia_insidiosa_FC1138	0	0	0	-1000	-1000	0	0	0	0
Ralstonia_pickettii_12D	0	0	-1000	-1000	-1000	0	-1000	0	0
Ralstonia_pickettii_12J	0	0	-1000	-1000	-1000	0	-1000	0	0
Ralstonia_pickettii_5_7_47FAA	0	0	-1000	-1000	-32.58329	0	-1000	0	0
Ralstonia_pickettii_52	0	0	-1000	-1000	-1000	0	-1000	0	0
Ralstonia_pickettii_NBRC_102503	0	0	-1000	-1000	-1000	0	-1000	0	0
Ralstonia_pickettii_OR214	0	0	-1000	-1000	-1000	0	-1000	0	0
Ralstonia_sp_5_2_56FAA	0	0	0	-1000	-1000	0	0	0	0
Raoultella_ornithinolytica_10_5246	0	0	-1000	-1000	-35.754752	-1000	0	-1000	0
Raoultella_ornithinolytica_2_156_04_S1_C1	0	0	-1000	-1000	-39.596328	-1000	0	-1000	0
Raoultella_ornithinolytica_B6	-1000	0	-1000	-1000	-39.596361	-1000	0	-1000	0
Raoultella_ornithinolytica_ornithinolytica_strain_	0	0	-1000	-1000	-39.24445	-1000	0	-1000	0
Raoultella_planticola_ATCC_33531	0	0	-1000	-1000	-60.562951	-1000	0	-1000	0
Reyranella_massiliensis_521	0	0	0	-1000	-20.42074	0	0	0	0
Reyranella_soli_NBRC_108950	0	0	0	-1000	-28.824893	0	0	0	0
Rhizobium_giardinii_bv_giardinii_H152	0	0	-1000	-62.09910721	-20.138083	-1000	0	0	0
Rhizobium_leguminosarum_bv_phaseoli_4292	0	0	0	-1000	-1000	-1000	0	-1000	0
Rhizobium_leguminosarum_bv_phaseoli_CCGM1	0	0	0	-1000	-1000	-1000	0	0	0
Rhizobium_leguminosarum_bv_viciae_WSM1481	0	0	0	-1000	-1000	-1000	0	-1000	0
Rhizobium_leguminosarum_Vaf_108	0	0	0	-1000	-1000	-1000	0	-1000	0
Rhodococcus_equi_103S	0	0	0	-1000	-1000	0	-1000	0	0
Rhodococcus_equi_ATCC_33707	0	0	0	-1000	-30.702062	0	-1000	0	0
Rhodococcus_equi_NBRC_101255	0	0	0	-1000	-53.614847	0	-1000	0	0
Rhodococcus_erythropolis_BG43	0	0	0	-1000	-37.879798	-1000	-1000	-1000	0
Rhodococcus_erythropolis_PR4	0	0	0	-1000	-32.190265	-1000	-1000	-1000	0
Rhodococcus_erythropolis_SK121	0	0	0	-1000	-23.828946	-1000	-1000	-1000	0
Rhodococcus_erythropolis_XP	0	0	0	-1000	-25.337182	-1000	-1000	-1000	0
Rhodococcus_rhodochrous_ATCC_17895	0	0	0	-1000	-23.136814	-1000	-1000	-1000	0
Rhodococcus_rhodochrous_ATCC_21198	0	0	0	0	-38.132445	-1000	-1000	-1000	0
Rickettsiella_grylli	0	0	0	0	-6.3971879	0	0	0	0
Rikenella_microfusus_DSM_15922	0	0	-1000	0	-19.361023	0	0	0	0
Rikenellaceae_nov_ERR2221101	0	0	0	-1000	-7.3260209	0	0	0	0
Rikenellaceae_nov_ERR2221110	0	0	0	-1000	-7.3256927	0	0	0	0
Risungbinella_massiliensis_GD1	0	0	0	-1000	-14.23015	-1000	0	0	0
Robinsoniella_nov_ERR2221391	0	0	0	-1000	-18.362317	0	0	-601.19048	0
Robinsoniella_peoriensis_WT	0	0	0	-1000	-19.334505	0	0	-856.54995	0
Rodentibacter_pneumotropicus_DSM_21403	0	0	0	-1000	-22.900016	0	0	0	0
Romboutsia_nov_ERR1022375	0	0	0	-25.77510183	-18.332958	0	0	-861.44578	0
Romboutsia_nov_ERR1022465	0	0	0	-1000	-21.834732	0	0	0	0
Romboutsia_nov_ERR1022466	0	0	0	-1000	-21.834726	0	0	0	0
Roseburia_faecis_ERR1022321	0	0	0	-42.34783818	-21.762531	0	0	0	0
Roseburia_faecis_ERR2221408	0	0	0	-48.0432269	-24.541631	0	0	-543.80952	0
Roseburia_faecis_ERR2230155	0	0	0	-37.49801942	-19.262645	0	0	0	0
Roseburia_faecis_M72	0	0	0	-32.29635936	-15.794468	0	0	0	0
Roseburia_hominis_A2_183	0	0	-1000	-1000	-202.5228	0	0	0	0
Roseburia_hominis_ERR1022292	0	0	0	0	-25.174916	0	0	-1000	0
Roseburia_intestinalis_ERR1022474	0	0	-1000	-52.98686849	-25.699921	0	0	-1000	0
Roseburia_intestinalis_ERR2221171	0	0	-1000	-44.22094392	-21.448234	0	0	-1000	0
Roseburia_intestinalis_ERR2230070	0	0	-1000	-44.59481342	-21.630241	0	0	-1000	0
Roseburia_intestinalis_L1_82	0	0	-1000	-1000	-28.885512	0	0	-1000	0
Roseburia_intestinalis_XB6B4	0	0	-1000	-1000	-32.539843	0	0	-1000	0
Roseburia_inulinivorans_DSM_16841	0	0	0	-74.02806089	-29.234446	0	0	-1000	0
Roseburia_inulinivorans_ERR1022293	0	0	0	-52.39643276	-26.719215	0	0	-532.21891	0
Roseburia_inulinivorans_ERR1022345	0	0	0	-45.50463895	-23.220149	0	0	-527.16808	0
Roseburia_inulinivorans_ERR1203941	0	0	0	-46.96260766	-23.959656	0	0	-525.65994	0
Roseburia_nov_ERR1022344	0	0	0	-1000	-14.041897	0	0	-391.89189	0
Roseburia_nov_ERR1204035	0	0	0	-33.25284918	-16.988932	0	0	0	0
Roseburia_nov_ERR171282	0	0	0	-45.36237701	-23.425615	0	0	-474.28884	0
Roseburia_nov_ERR2221407	0	0	0	-48.04323017	-24.541633	0	0	-543.80952	0
Roseomonas_cervicalis_ATCC_49957	0	0	0	-1000	-1000	0	0	0	0
Roseomonas_mucosa_ATCC_BAA_692	0	0	0	-1000	-1000	0	0	0	0
Rothia_aeria_F0474	0	0	0	-1000	0	0	0	-666.66667	0
Rothia_dentocariosa_ATCC_17931	0	0	0	-1000	0	0	0	-666.66667	0
Rothia_dentocariosa_M567	0	0	0	-1000	0	0	0	-944.44444	0
Rothia_mucilaginosa_ATCC_25296	0	0	0	-29.55032038	0	0	0	-750	0
Rothia_mucilaginosa_DY_18	0	0	0	-41.97495604	0	0	0	-1000	0

Rothia_mucilaginosa_M508	0	0	0	-1000	0	0	0	-944.44444	0
Rothia_mucilaginosa_NUM_Rm6536	0	0	0	-33.31957886	-25.654454	0	0	-750	0
Rubrobacter_aplysinae_RV113	0	0	0	0	-13.508923	-1000	-1000	0	0
Rubrobacter_radiotolerans_DSM_5868	0	0	0	-1000	-13.084916	-1000	0	0	0
Rubrobacter_xylanophilus_DSM_9941	0	0	0	-1000	-1000	0	0	0	0
Rudanella_lutea_DSM_19387	0	0	0	0	0	0	0	0	0
Ruminiclostridium_nov_ERR1022440	0	0	0	-534.8244947	-18.065662	0	0	0	0
Ruminiclostridium_nov_ERR1022441	0	0	0	-1000	-21.082936	0	0	-514.91423	0
Ruminiclostridium_nov_ERR2221177	0	0	0	-527.2432447	-20.332765	0	0	0	0
Ruminiclostridium_sp_KB18	0	0	0	0	-11.02462	0	0	-255.93206	0
Ruminiclostridium_thermocellum_AD2	0	0	0	0	-18.914483	0	0	0	0
Ruminiclostridium_thermocellum_ATCC_27405	0	0	0	0	-18.915606	0	0	0	0
Ruminiclostridium_thermocellum_DSM_1313	0	0	0	0	-18.914473	0	0	0	0
Ruminiclostridium_thermocellum_DSM_2360	0	0	0	0	-18.914483	0	0	0	0
Ruminococcaceae_bacterium_D16	0	0	0	-1000	-32.431147	0	0	0	0
Ruminococcaceae_nov_ERR1022299	0	0	0	0	-10.548041	0	0	0	0
Ruminococcaceae_nov_ERR1022311	0	0	0	-1000	-15.632084	0	0	0	0
Ruminococcaceae_nov_ERR1022313	0	0	0	-783.7837838	-10.258322	-1000	0	-666.66667	0
Ruminococcaceae_nov_ERR1022317	0	0	0	0	-10.739926	0	0	0	0
Ruminococcaceae_nov_ERR1022336	0	0	0	-1000	-35.599328	0	0	0	0
Ruminococcaceae_nov_ERR1022409	0	0	0	-1000	-35.582366	0	0	0	0
Ruminococcaceae_nov_ERR1022442	0	0	0	-125	-2.0826727	0	0	0	0
Ruminococcaceae_nov_ERR1022443	0	0	0	0	-14.48352	0	0	0	0
Ruminococcaceae_nov_ERR1022445	0	0	0	-896.8253968	-33.72362	0	0	0	0
Ruminococcaceae_nov_ERR1022448	0	0	0	0	-8.2712115	0	0	0	0
Ruminococcaceae_nov_ERR1022449	0	0	0	-1000	-15.533664	0	0	0	0
Ruminococcaceae_nov_ERR1022451	0	0	0	-1000	-26.134027	0	0	0	0
Ruminococcaceae_nov_ERR1022452	0	0	0	-4.791058338	-9.4534932	0	0	0	0
Ruminococcaceae_nov_ERR1022483	0	0	0	0	-10.739945	0	0	0	0
Ruminococcaceae_nov_ERR1203965	0	0	0	-518.4969614	-26.296995	0	0	0	0
Ruminococcaceae_nov_ERR1204059	0	0	0	-896.8253968	-34.980213	0	0	0	0
Ruminococcaceae_nov_ERR2221149	0	0	0	-501.1702925	-10.285692	0	0	0	0
Ruminococcaceae_nov_ERR2221180	0	0	0	-1000	-14.646555	0	0	0	0
Ruminococcaceae_nov_ERR2221184	0	0	0	0	-14.623868	0	0	0	0
Ruminococcaceae_nov_ERR2221393	0	0	0	-500	-4.1876913	0	0	0	0
Ruminococcaceae_nov_ERR2221397	0	0	0	-1000	-14.646562	0	0	0	0
Ruminococcaceae_nov_ERR2230059	0	0	0	-5.119311337	-9.4768225	0	0	0	0
Ruminococcaceae_nov_ERR2230068	0	0	0	-818.1818182	-18.793945	0	0	0	0
Ruminococcus_albus_7	0	0	-1000	0	-24.230231	-1000	0	-682.83071	0
Ruminococcus_albus_8	0	0	-1000	0	0	-1000	0	-1000	0
Ruminococcus_bicirculans_80_3	0	0	0	-1000	-146.34146	0	0	0	0
Ruminococcus_bicirculans_ERR1022350	0	0	0	-1000	-18.33167	-537.62639	0	-268.41713	0
Ruminococcus_bicirculans_ERR1022408	0	0	0	-1000	-18.13788	-540.40286	0	-269.77399	0
Ruminococcus_bicirculans_ERR1022485	0	0	0	-1000	-18.327204	-537.61731	0	-268.41269	0
Ruminococcus_bicirculans_ERR2221172	0	0	0	-1000	-18.327217	-537.61733	0	-268.41271	0
Ruminococcus_bicirculans_ERR2230101	0	0	0	-1000	-18.331506	-537.62605	0	-268.41697	0
Ruminococcus_bromii_ATCC_27255	0	0	0	-1000	-16.795736	0	0	0	0
Ruminococcus_bromii_ERR1022486	0	0	0	-1000	0	0	0	0	0
Ruminococcus_bromii_ERR2221220	0	0	0	-1000	0	0	0	0	0
Ruminococcus_bromii_L2_63	0	0	0	-1000	0	0	0	0	0
Ruminococcus_callidus_ATCC_2776001	0	0	0	-1000	0	0	0	-463.31133	0
Ruminococcus_champanellensis_18P13	0	0	0	-1000	-18.548099	0	0	0	0
Ruminococcus_faecis_ERR1022432	0	0	0	-47.6184238	-24.248704	0	0	-760.23392	0
Ruminococcus_flavefaciens_ATCC_19208	0	0	0	0	-20.58775	0	0	0	0
Ruminococcus_flavefaciens_FD_1	0	0	0	0	0	0	0	0	0
Ruminococcus_gauvreauii_DSM_19829	0	0	0	-1000	-16.027237	-1000	0	-784.31373	0
Ruminococcus_lactaris_ATCC_29176	0	0	0	0	-17.549331	-862.06897	0	0	0
Ruminococcus_nov_ERR2230055	0	0	0	-5.119333021	-9.4768632	0	0	0	0
Ruminococcus_sp_5_1_39BFAA	0	0	0	-1000	-21.947013	0	0	-958.33333	0
Ruminococcus_sp_SR1_5	0	0	0	-1000	-28.414052	0	0	-1000	0
Ruthenibacterium_lactatiformans_585_1	0	0	0	0	-10.044517	0	0	0	0
Ruthenibacterium_lactatiformans_668	0	0	0	0	-10.053386	0	0	0	0
Saccharolobus_solfataricus_P2	0	0	0	-19.41042671	-16.377066	0	0	-11.64426	0
Salinibacterium_amurskyense_DSM_16400	0	0	0	0	-12.132199	-1000	0	0	0
Salmonella_bongori_BCW_1556	0	0	-1000	-1000	-36.052991	-1000	0	0	0
Salmonella_bongori_N268_08	0	0	-1000	-1000	-37.576007	-1000	0	0	0
Salmonella_bongori_NCTC_12419	-1000	0	-1000	-1000	-46.727475	-1000	0	0	0
Salmonella_bongori_serovar_44_z39_	0	0	-1000	-1000	-33.057368	-1000	0	0	0
Salmonella_bongori_serovar_66_z41_str_SA1998	0	0	-1000	-1000	-37.60089	-1000	0	0	0
Salmonella_enterica_enterica_sv_Typhimurium_L	-1000	0	-1000	-1000	-203.62475	-1000	-1000	0	0
Salmonella_enterica_subsp_arizonae_serovar_62	-1000	0	-1000	-1000	-46.934085	-1000	-1000	0	0
Salmonella_enterica_subsp_enterica_serovar_45	0	0	-1000	-1000	-54.967109	-1000	-1000	0	0
Salmonella_enterica_subsp_enterica_serovar_Ag	0	0	-1000	-1000	-55.536762	-1000	-1000	0	0
Salmonella_enterica_subsp_enterica_serovar_Ag	0	0	-1000	-1000	-55.430105	-1000	-1000	0	0
Salmonella_enterica_subsp_enterica_serovar_Ag	0	0	-1000	-1000	-55.547867	-1000	-1000	0	0
Salmonella_enterica_subsp_enterica_serovar_Ag	0	0	-1000	-1000	-55.547867	-1000	-1000	0	0
Salmonella_enterica_subsp_enterica_serovar_Ag	0	0	-1000	-1000	-55.547867	-1000	-1000	0	0
Salmonella_enterica_subsp_enterica_serovar_Ag	0	0	-1000	-1000	-55.547867	-1000	-1000	0	0
Salmonella_enterica_subsp_enterica_serovar_Ag	0	0	-1000	-1000	-55.547867	-1000	-1000	0	0
Salmonella_enterica_subsp_enterica_serovar_Ag	0	0	-1000	-1000	-55.547867	-1000	-1000	0	0
Salmonella_enterica_subsp_enterica_serovar_Ag	0	0	-1000	-1000	-55.536762	-1000	-1000	0	0
Salmonella_enterica_subsp_enterica_serovar_Ag	0	0	-1000	-1000	-55.536762	-1000	-1000	0	0
Salmonella_enterica_subsp_enterica_serovar_Ag	0	0	-1000	-1000	-55.547867	-1000	-1000	0	0
Salmonella_enterica_subsp_enterica_serovar_Ag	0	0	-1000	-1000	-55.547867	-1000	-1000	0	0
Salmonella_enterica_subsp_enterica_serovar_Ag	0	0	-1000	-1000	-55.547867	-1000	-1000	0	0
Salmonella_enterica_subsp_enterica_serovar_Ag	0	0	-1000	-1000	-55.547867	-1000	-1000	0	0
Salmonella_enterica_subsp_enterica_serovar_Ag	0	0	-1000	-1000	-55.547867	-1000	-1000	0	0
Salmonella_enterica_subsp_enterica_serovar_Ag	0	0	-1000	-1000	-55.536762	-1000	-1000	0	0
Salmonella_enterica_subsp_enterica_serovar_Ag	0	0	-1000	-1000	-55.547867	-1000	-1000	0	0
Salmonella_enterica_subsp_enterica_serovar_Ag	0	0	-1000	-1000	-55.547867	-1000	-1000	0	0
Salmonella_enterica_subsp_enterica_serovar_Ag	0	0	-1000	-1000	-55.547867	-1000	-1000	0	0

Salmonella_enterica_subsp_enterica_serovar_Ag	0	0	-1000	-1000	-55.547867	-1000	-1000	0	0
Salmonella_enterica_subsp_enterica_serovar_Ag	0	0	-1000	-1000	-55.542314	-1000	-1000	0	0
Salmonella_enterica_subsp_enterica_serovar_Ag	0	0	-1000	-1000	-55.547867	-1000	-1000	0	0
Salmonella_enterica_subsp_enterica_serovar_Ag	0	0	-1000	-1000	-55.54787	-1000	-1000	0	0
Salmonella_enterica_subsp_enterica_serovar_Ag	0	0	-1000	-1000	-55.547867	-1000	-1000	0	0
Salmonella_enterica_subsp_enterica_serovar_Ag	0	0	-1000	-1000	-55.547867	-1000	-1000	0	0
Salmonella_enterica_subsp_enterica_serovar_Ag	0	0	-1000	-1000	-57.30527	-1000	-1000	0	0
Salmonella_enterica_subsp_enterica_serovar_Ag	0	0	-1000	-1000	-55.547867	-1000	-1000	0	0
Salmonella_enterica_subsp_enterica_serovar_Ag	0	0	-1000	-1000	-55.547867	-1000	-1000	0	0
Salmonella_enterica_subsp_enterica_serovar_Ag	0	0	-1000	-1000	-58.042367	-1000	-1000	0	0
Salmonella_enterica_subsp_enterica_serovar_Ag	0	0	-1000	-1000	-56.289809	-1000	-1000	0	0
Salmonella_enterica_subsp_enterica_serovar_Ag	0	0	-1000	-1000	-55.547867	-1000	-1000	0	0
Salmonella_enterica_subsp_enterica_serovar_Ag	0	0	-1000	-1000	-55.547867	-1000	-1000	0	0
Salmonella_enterica_subsp_enterica_serovar_Ag	0	0	-1000	-1000	-55.547867	-1000	-1000	0	0
Salmonella_enterica_subsp_enterica_serovar_Ag	0	0	-1000	-1000	-55.547867	-1000	-1000	0	0
Salmonella_enterica_subsp_enterica_serovar_Ag	0	0	-1000	-1000	-55.516315	-1000	-1000	0	0
Salmonella_enterica_subsp_enterica_serovar_Ag	0	0	-1000	-1000	-60.505434	-1000	-1000	0	0
Salmonella_enterica_subsp_enterica_serovar_Ag	0	0	-1000	-1000	-55.547867	-1000	-1000	0	0
Salmonella_enterica_subsp_enterica_serovar_Ag	0	0	-1000	-1000	-57.30527	-1000	-1000	0	0
Salmonella_enterica_subsp_enterica_serovar_Ag	0	0	-1000	-1000	-57.30527	-1000	-1000	0	0
Salmonella_enterica_subsp_enterica_serovar_Ag	0	0	-1000	-1000	-55.547867	-1000	-1000	0	0
Salmonella_enterica_subsp_enterica_serovar_Ag	0	0	-1000	-1000	-58.880536	-1000	-1000	0	0
Salmonella_enterica_subsp_enterica_serovar_Ag	0	0	-1000	-1000	-55.547867	-1000	-1000	0	0
Salmonella_enterica_subsp_enterica_serovar_Ag	0	0	-1000	-1000	-55.547867	-1000	-1000	0	0
Salmonella_enterica_subsp_enterica_serovar_Ag	0	0	-1000	-1000	-55.547867	-1000	-1000	0	0
Salmonella_enterica_subsp_enterica_serovar_Ag	0	0	-1000	-1000	-55.536765	-1000	-1000	0	0
Salmonella_enterica_subsp_enterica_serovar_Ag	0	0	-1000	-1000	-58.893272	-1000	-1000	0	0
Salmonella_enterica_subsp_enterica_serovar_Ag	0	0	-1000	-1000	-55.547867	-1000	-1000	0	0
Salmonella_enterica_subsp_enterica_serovar_Ag	0	0	-1000	-1000	-55.547867	-1000	-1000	0	0
Salmonella_enterica_subsp_enterica_serovar_Ag	0	0	-1000	-1000	-55.547867	-1000	-1000	0	0
Salmonella_enterica_subsp_enterica_serovar_Ag	0	0	-1000	-1000	-55.547867	-1000	-1000	0	0
Salmonella_enterica_subsp_enterica_serovar_Ag	0	0	-1000	-1000	-55.547867	-1000	-1000	0	0
Salmonella_enterica_subsp_enterica_serovar_Ag	0	0	-1000	-1000	-58.018646	-1000	-1000	0	0
Salmonella_enterica_subsp_enterica_serovar_Ag	0	0	-1000	-1000	-55.547867	-1000	-1000	0	0
Salmonella_enterica_subsp_enterica_serovar_Ag	0	0	-1000	-1000	-55.547867	-1000	-1000	0	0
Salmonella_enterica_subsp_enterica_serovar_Ag	0	0	-1000	-1000	-55.547867	-1000	-1000	0	0
Salmonella_enterica_subsp_enterica_serovar_Ag	0	0	-1000	-1000	-55.555921	-1000	-1000	0	0
Salmonella_enterica_subsp_enterica_serovar_Ag	0	0	-1000	-1000	-55.547867	-1000	-1000	0	0
Salmonella_enterica_subsp_enterica_serovar_Ag	0	0	-1000	-1000	-55.547867	-1000	-1000	0	0
Salmonella_enterica_subsp_enterica_serovar_Ag	0	0	-1000	-1000	-55.547867	-1000	-1000	0	0
Salmonella_enterica_subsp_enterica_serovar_Ag	0	0	-1000	-1000	-55.547867	-1000	-1000	-1000	0
Salmonella_enterica_subsp_enterica_serovar_Ag	0	0	-1000	-1000	-55.547867	-1000	-1000	0	0
Salmonella_enterica_subsp_enterica_serovar_Ag	0	0	-1000	-1000	-55.536762	-1000	-1000	0	0
Salmonella_enterica_subsp_enterica_serovar_Ag	0	0	-1000	-1000	-55.547867	-1000	-1000	0	0
Salmonella_enterica_subsp_enterica_serovar_Ag	0	0	-1000	-1000	-55.536762	-1000	-1000	0	0
Salmonella_enterica_subsp_enterica_serovar_Ag	0	0	-1000	-1000	-55.547867	-1000	-1000	0	0
Salmonella_enterica_subsp_enterica_serovar_Ag	0	0	-1000	-1000	-55.547867	-1000	-1000	0	0
Salmonella_enterica_subsp_enterica_serovar_Ag	0	0	-1000	-1000	-54.252276	-1000	-1000	0	0
Salmonella_enterica_subsp_enterica_serovar_Ag	0	0	-1000	-1000	-55.536762	-1000	-1000	0	0
Salmonella_enterica_subsp_enterica_serovar_Ag	0	0	-1000	-1000	-55.547867	-1000	-1000	0	0
Salmonella_enterica_subsp_enterica_serovar_Ag	0	0	-1000	-1000	-55.547867	-1000	-1000	0	0
Salmonella_enterica_subsp_enterica_serovar_Ag	0	0	-1000	-1000	-54.813449	-1000	-1000	0	0
Salmonella_enterica_subsp_enterica_serovar_Ag	-1000	0	-1000	-1000	-55.536762	-1000	-1000	0	0
Salmonella_enterica_subsp_enterica_serovar_An	0	0	-1000	-1000	-60.352963	-1000	-1000	0	0
Salmonella_enterica_subsp_enterica_serovar_Ba	0	0	-1000	-1000	-60.39528	-1000	-1000	0	0
Salmonella_enterica_subsp_enterica_serovar_Ba	0	0	-1000	-1000	-57.69582	-1000	-1000	0	0
Salmonella_enterica_subsp_enterica_serovar_Ch	0	0	-1000	-1000	-58.527563	-1000	-1000	0	0
Salmonella_enterica_subsp_enterica_serovar_Ch	0	0	-1000	-1000	-49.726295	-1000	-1000	0	0
Salmonella_enterica_subsp_enterica_serovar_Du	0	0	-1000	-1000	-49.333698	-1000	-1000	0	0
Salmonella_enterica_subsp_enterica_serovar_Du	-1000	0	-1000	-1000	-58.708875	-1000	-1000	0	0
Salmonella_enterica_subsp_enterica_serovar_Du	0	0	-1000	-1000	-50.233172	-1000	-1000	0	0
Salmonella_enterica_subsp_enterica_serovar_Du	0	0	-1000	-1000	-59.564725	-1000	-1000	0	0
Salmonella_enterica_subsp_enterica_serovar_Du	0	0	-1000	-1000	-59.564725	-1000	-1000	0	0
Salmonella_enterica_subsp_enterica_serovar_En	0	0	-1000	-1000	-55.601952	-1000	-1000	0	0
Salmonella_enterica_subsp_enterica_serovar_En	0	0	-1000	-1000	-55.590617	-1000	-1000	0	0
Salmonella_enterica_subsp_enterica_serovar_En	0	0	-1000	-1000	-57.647832	-1000	-1000	0	0
Salmonella_enterica_subsp_enterica_serovar_En	0	0	-1000	-1000	-56.900234	-1000	-1000	0	0
Salmonella_enterica_subsp_enterica_serovar_En	0	0	-1000	-1000	-57.457978	-1000	-1000	0	0
Salmonella_enterica_subsp_enterica_serovar_En	0	0	-1000	-1000	-56.660802	-1000	-1000	0	0
Salmonella_enterica_subsp_enterica_serovar_En	0	0	-1000	-1000	-55.601952	-1000	-1000	0	0
Salmonella_enterica_subsp_enterica_serovar_En	0	0	-1000	-1000	-55.590617	-1000	-1000	0	0
Salmonella_enterica_subsp_enterica_serovar_En	0	0	-1000	-1000	-55.590617	-1000	-1000	0	0
Salmonella_enterica_subsp_enterica_serovar_En	0	0	-1000	-1000	-55.601952	-1000	-1000	0	0
Salmonella_enterica_subsp_enterica_serovar_En	0	0	-1000	-1000	-55.076509	-1000	-1000	0	0
Salmonella_enterica_subsp_enterica_serovar_En	0	0	-1000	-1000	-55.601952	-1000	-1000	0	0
Salmonella_enterica_subsp_enterica_serovar_En	0	0	-1000	-1000	-55.583063	-1000	-1000	0	0
Salmonella_enterica_subsp_enterica_serovar_En	0	0	-1000	-1000	-56.143626	-1000	-1000	0	0
Salmonella_enterica_subsp_enterica_serovar_En	0	0	-1000	-1000	-55.601972	-1000	-1000	0	0
Salmonella_enterica_subsp_enterica_serovar_En	0	0	-1000	-1000	-52.515015	-1000	-1000	0	0
Salmonella_enterica_subsp_enterica_serovar_En	0	0	-1000	-1000	-55.601972	-1000	-1000	0	0
Salmonella_enterica_subsp_enterica_serovar_En	0	0	-1000	-1000	-55.601972	-1000	-1000	0	0
Salmonella_enterica_subsp_enterica_serovar_En	0	0	-1000	-1000	-55.601952	-1000	-1000	0	0
Salmonella_enterica_subsp_enterica_serovar_En	0	0	-1000	-1000	-57.568382	-1000	-1000	0	0
Salmonella_enterica_subsp_enterica_serovar_En	0	0	-1000	-1000	-58.337812	-1000	-1000	0	0
Salmonella_enterica_subsp_enterica_serovar_En	0	0	-1000	-1000	-55.601952	-1000	-1000	0	0
Salmonella_enterica_subsp_enterica_serovar_En	0	0	-1000	-1000	-55.601952	-1000	-1000	0	0
Salmonella_enterica_subsp_enterica_serovar_En	0	0	-1000	-1000	-55.601972	-1000	-1000	0	0
Salmonella_enterica_subsp_enterica_serovar_En	0	0	-1000	-1000	-55.601972	-1000	-1000	0	0
Salmonella_enterica_subsp_enterica_serovar_En	0	0	-1000	-1000	-55.601972	-1000	-1000	0	0
Salmonella_enterica_subsp_enterica_serovar_En	0	0	-1000	-1000	-55.601972	-1000	-1000	0	0
Salmonella_enterica_subsp_enterica_serovar_En	0	0	-1000	-1000	-55.601972	-1000	-1000	0	0

Salmonella_enterica_subsp_enterica_serovar_Ent	0	0	-1000	-1000	-55.601952	-1000	-1000	0	0
Salmonella_enterica_subsp_enterica_serovar_Ent	0	0	-1000	-1000	-55.601972	-1000	-1000	0	0
Salmonella_enterica_subsp_enterica_serovar_Ent	0	0	-1000	-1000	-56.712134	-1000	-1000	0	0
Salmonella_enterica_subsp_enterica_serovar_Ent	0	0	-1000	-1000	-55.601952	-1000	-1000	0	0
Salmonella_enterica_subsp_enterica_serovar_Ent	0	0	-1000	-1000	-55.594395	-1000	-1000	0	0
Salmonella_enterica_subsp_enterica_serovar_Ent	0	0	-1000	-1000	-55.601952	-1000	-1000	0	0
Salmonella_enterica_subsp_enterica_serovar_Ent	0	0	-1000	-1000	-55.594414	-1000	-1000	0	0
Salmonella_enterica_subsp_enterica_serovar_Ent	0	0	-1000	-1000	-55.601972	-1000	-1000	0	0
Salmonella_enterica_subsp_enterica_serovar_Ent	0	0	-1000	-1000	-55.601952	-1000	-1000	0	0
Salmonella_enterica_subsp_enterica_serovar_Ent	0	0	-1000	-1000	-55.601952	-1000	-1000	0	0
Salmonella_enterica_subsp_enterica_serovar_Ent	0	0	-1000	-1000	-56.636891	-1000	-1000	0	0
Salmonella_enterica_subsp_enterica_serovar_Ent	0	0	-1000	-1000	-61.497721	-1000	-1000	0	0
Salmonella_enterica_subsp_enterica_serovar_Ent	0	0	-1000	-1000	-61.497721	-1000	-1000	0	0
Salmonella_enterica_subsp_enterica_serovar_Ent	0	0	-1000	-1000	-62.774652	-1000	-1000	0	0
Salmonella_enterica_subsp_enterica_serovar_Ent	0	0	-1000	-1000	-57.915267	-1000	-1000	0	0
Salmonella_enterica_subsp_enterica_serovar_Ent	0	0	-1000	-1000	-56.645038	-1000	-1000	0	0
Salmonella_enterica_subsp_enterica_serovar_Ent	0	0	-1000	-1000	-56.645664	-1000	-1000	0	0
Salmonella_enterica_subsp_enterica_serovar_Ent	0	0	-1000	-1000	-61.242821	-1000	-1000	0	0
Salmonella_enterica_subsp_enterica_serovar_Ent	0	0	-1000	-1000	-61.242821	-1000	-1000	0	0
Salmonella_enterica_subsp_enterica_serovar_Ent	0	0	-1000	-1000	-67.191309	-1000	-1000	0	0
Salmonella_enterica_subsp_enterica_serovar_Ent	0	0	-1000	-1000	-61.242821	-1000	-1000	0	0
Salmonella_enterica_subsp_enterica_serovar_Ent	0	0	-1000	-1000	-57.562139	-1000	-1000	0	0
Salmonella_enterica_subsp_enterica_serovar_Ent	0	0	-1000	-1000	-58.096872	-1000	-1000	0	0
Salmonella_enterica_subsp_enterica_serovar_Ent	0	0	-1000	-1000	-57.780968	-1000	-1000	0	0
Salmonella_enterica_subsp_enterica_serovar_Ent	0	0	-1000	-1000	-57.568392	-1000	-1000	0	0
Salmonella_enterica_subsp_enterica_serovar_Ent	0	0	-1000	-1000	-56.757056	-1000	-1000	0	0
Salmonella_enterica_subsp_enterica_serovar_Ent	0	0	-1000	-1000	-55.601952	-1000	-1000	0	0
Salmonella_enterica_subsp_enterica_serovar_Ent	0	0	-1000	-1000	-55.601952	-1000	-1000	0	0
Salmonella_enterica_subsp_enterica_serovar_Ent	0	0	-1000	-1000	-55.601952	-1000	-1000	0	0
Salmonella_enterica_subsp_enterica_serovar_Ent	0	0	-1000	-1000	-55.601952	-1000	-1000	0	0
Salmonella_enterica_subsp_enterica_serovar_Ent	0	0	-1000	-1000	-55.590617	-1000	-1000	0	0
Salmonella_enterica_subsp_enterica_serovar_Ent	0	0	-1000	-1000	-55.455483	-1000	-1000	0	0
Salmonella_enterica_subsp_enterica_serovar_Ent	0	0	-1000	-1000	-55.601972	-1000	-1000	0	0
Salmonella_enterica_subsp_enterica_serovar_Ent	0	0	-1000	-1000	-55.601972	-1000	-1000	0	0
Salmonella_enterica_subsp_enterica_serovar_Ent	0	0	-1000	-1000	-55.601972	-1000	-1000	0	0
Salmonella_enterica_subsp_enterica_serovar_Ent	0	0	-1000	-1000	-55.601941	-1000	-1000	0	0
Salmonella_enterica_subsp_enterica_serovar_Ent	0	0	-1000	-1000	-55.601952	-1000	-1000	0	0
Salmonella_enterica_subsp_enterica_serovar_Ent	0	0	-1000	-1000	-55.601952	-1000	-1000	0	0
Salmonella_enterica_subsp_enterica_serovar_Ent	0	0	-1000	-1000	-55.601952	-1000	-1000	0	0
Salmonella_enterica_subsp_enterica_serovar_Ent	0	0	-1000	-1000	-55.601972	-1000	-1000	0	0
Salmonella_enterica_subsp_enterica_serovar_Ent	0	0	-1000	-1000	-55.601952	-1000	-1000	0	0
Salmonella_enterica_subsp_enterica_serovar_Ent	0	0	-1000	-1000	-55.594395	-1000	-1000	0	0
Salmonella_enterica_subsp_enterica_serovar_Ent	0	0	-1000	-1000	-55.601952	-1000	-1000	0	0
Salmonella_enterica_subsp_enterica_serovar_Ent	0	0	-1000	-1000	-55.601952	-1000	-1000	0	0
Salmonella_enterica_subsp_enterica_serovar_Ent	0	0	-1000	-1000	-55.601972	-1000	-1000	0	0
Salmonella_enterica_subsp_enterica_serovar_Ent	0	0	-1000	-1000	-55.601952	-1000	-1000	0	0
Salmonella_enterica_subsp_enterica_serovar_Ent	0	0	-1000	-1000	-55.594384	-1000	-1000	0	0
Salmonella_enterica_subsp_enterica_serovar_Ent	0	0	-1000	-1000	-55.601952	-1000	-1000	0	0
Salmonella_enterica_subsp_enterica_serovar_Ent	0	0	-1000	-1000	-55.594384	-1000	-1000	0	0
Salmonella_enterica_subsp_enterica_serovar_Ent	0	0	-1000	-1000	-55.601952	-1000	-1000	0	0
Salmonella_enterica_subsp_enterica_serovar_Ent	0	0	-1000	-1000	-55.601972	-1000	-1000	0	0
Salmonella_enterica_subsp_enterica_serovar_Ent	0	0	-1000	-1000	-55.601972	-1000	-1000	0	0
Salmonella_enterica_subsp_enterica_serovar_Ent	0	0	-1000	-1000	-55.601972	-1000	-1000	0	0
Salmonella_enterica_subsp_enterica_serovar_Ent	0	0	-1000	-1000	-55.601972	-1000	-1000	0	0
Salmonella_enterica_subsp_enterica_serovar_Ent	0	0	-1000	-1000	-55.601972	-1000	-1000	0	0
Salmonella_enterica_subsp_enterica_serovar_Ent	0	0	-1000	-1000	-55.601952	-1000	-1000	0	0
Salmonella_enterica_subsp_enterica_serovar_Ent	0	0	-1000	-1000	-55.601952	-1000	-1000	0	0
Salmonella_enterica_subsp_enterica_serovar_Ent	0	0	-1000	-1000	-55.601972	-1000	-1000	0	0
Salmonella_enterica_subsp_enterica_serovar_Ent	0	0	-1000	-1000	-55.601972	-1000	-1000	0	0
Salmonella_enterica_subsp_enterica_serovar_Ent	0	0	-1000	-1000	-55.601972	-1000	-1000	0	0
Salmonella_enterica_subsp_enterica_serovar_Ent	0	0	-1000	-1000	-55.601972	-1000	-1000	0	0
Salmonella_enterica_subsp_enterica_serovar_Ent	0	0	-1000	-1000	-55.601972	-1000	-1000	0	0
Salmonella_enterica_subsp_enterica_serovar_Ent	0	0	-1000	-1000	-57.568382	-1000	-1000	0	0
Salmonella_enterica_subsp_enterica_serovar_Ent	0	0	-1000	-1000	-57.56503	-1000	-1000	0	0
Salmonella_enterica_subsp_enterica_serovar_Ent	0	0	-1000	-1000	-55.594395	-1000	-1000	0	0
Salmonella_enterica_subsp_enterica_serovar_Ent	0	0	-1000	-1000	-55.601952	-1000	-1000	0	0
Salmonella_enterica_subsp_enterica_serovar_Ent	0	0	-1000	-1000	-55.601952	-1000	-1000	0	0
Salmonella_enterica_subsp_enterica_serovar_Ent	0	0	-1000	-1000	-55.601952	-1000	-1000	0	0
Salmonella_enterica_subsp_enterica_serovar_Ent	0	0	-1000	-1000	-55.601952	-1000	-1000	0	0
Salmonella_enterica_subsp_enterica_serovar_Ent	0	0	-1000	-1000	-57.568392	-1000	-1000	0	0
Salmonella_enterica_subsp_enterica_serovar_Ent	0	0	-1000	-1000	-55.601941	-1000	-1000	0	0
Salmonella_enterica_subsp_enterica_serovar_Ent	0	0	-1000	-1000	-56.867831	-1000	-1000	0	0
Salmonella_enterica_subsp_enterica_serovar_Ent	0	0	-1000	-1000	-57.689749	-1000	-1000	0	0
Salmonella_enterica_subsp_enterica_serovar_Ent	0	0	-1000	-1000	-57.568392	-1000	-1000	0	0
Salmonella_enterica_subsp_enterica_serovar_Ent	0	0	-1000	-1000	-57.568392	-1000	-1000	0	0
Salmonella_enterica_subsp_enterica_serovar_Ent	0	0	-1000	-1000	-57.568392	-1000	-1000	0	0
Salmonella_enterica_subsp_enterica_serovar_Ent	0	0	-1000	-1000	-57.568392	-1000	-1000	0	0
Salmonella_enterica_subsp_enterica_serovar_Ent	0	0	-1000	-1000	-57.48814	-1000	-1000	0	0
Salmonella_enterica_subsp_enterica_serovar_Ga	-1000	0	-1000	-1000	-60.161528	-1000	-1000	0	0
Salmonella_enterica_subsp_enterica_serovar_Ga	-1000	0	-1000	-1000	-60.377861	-1000	-1000	0	0
Salmonella_enterica_subsp_enterica_serovar_Ga	0	0	-1000	-1000	-50.905856	-1000	-1000	0	0
Salmonella_enterica_subsp_enterica_serovar_Ga	0	0	-1000	-1000	-61.069863	-1000	-1000	0	0
Salmonella_enterica_subsp_enterica_serovar_Ha	0	0	-1000	-1000	-59.558152	-1000	-1000	0	0
Salmonella_enterica_subsp_enterica_serovar_Ha	0	0	-1000	-1000	-59.227303	-1000	-1000	0	0
Salmonella_enterica_subsp_enterica_serovar_Ha	0	0	-1000	-1000	-65.27115	-1000	-1000	0	0
Salmonella_enterica_subsp_enterica_serovar_Ha	0	0	-1000	-1000	-60.407963	-1000	-1000	0	0
Salmonella_enterica_subsp_enterica_serovar_Ha	0	0	-1000	-1000	-60.288802	-1000	-1000	0	0
Salmonella_enterica_subsp_enterica_serovar_He	0	0	-1000	-1000	-58.161459	-1000	-1000	0	0
Salmonella_enterica_subsp_enterica_serovar_He	0	0	-1000	-1000	-58.161459	-1000	-1000	0	0
Salmonella_enterica_subsp_enterica_serovar_He	0	0	-1000	-1000	-58.161459	-1000	-1000	0	0
Salmonella_enterica_subsp_enterica_serovar_He	0	0	-1000	-1000	-58.169003	-1000	-1000	0	0

Salmonella_enterica_subsp_enterica_serovar_He	0	0	-1000	-1000	-58.161459	-1000	-1000	0	0
Salmonella_enterica_subsp_enterica_serovar_He	-1000	0	-1000	-1000	-58.181581	-1000	-1000	0	0
Salmonella_enterica_subsp_enterica_serovar_He	0	0	-1000	-1000	-56.020563	-1000	-1000	0	0
Salmonella_enterica_subsp_enterica_serovar_He	0	0	-1000	-1000	-56.012891	-1000	-1000	0	0
Salmonella_enterica_subsp_enterica_serovar_He	0	0	-1000	-1000	-56.012891	-1000	-1000	0	0
Salmonella_enterica_subsp_enterica_serovar_He	0	0	-1000	-1000	-56.012891	-1000	-1000	0	0
Salmonella_enterica_subsp_enterica_serovar_He	-1000	0	-1000	-1000	-58.181581	-1000	-1000	0	0
Salmonella_enterica_subsp_enterica_serovar_He	0	0	-1000	-1000	-59.316193	-1000	-1000	0	0
Salmonella_enterica_subsp_enterica_serovar_Inf	0	0	-1000	-1000	-60.742159	-1000	-1000	0	0
Salmonella_enterica_subsp_enterica_serovar_Jav	0	0	-1000	-1000	-69.545109	-1000	-1000	0	0
Salmonella_enterica_subsp_enterica_serovar_Jav	0	0	-1000	-1000	-69.545109	-1000	-1000	0	0
Salmonella_enterica_subsp_enterica_serovar_Ker	0	0	-1000	-1000	-56.443568	-1000	-1000	0	0
Salmonella_enterica_subsp_enterica_serovar_Ker	0	0	-1000	-1000	-55.536762	-1000	-1000	0	0
Salmonella_enterica_subsp_enterica_serovar_Mb	0	0	-1000	-1000	-59.148398	-1000	-1000	0	0
Salmonella_enterica_subsp_enterica_serovar_Mi	0	0	-1000	-1000	-56.996538	-1000	-1000	0	0
Salmonella_enterica_subsp_enterica_serovar_Mo	0	0	-1000	-1000	-60.873998	-1000	-1000	0	0
Salmonella_enterica_subsp_enterica_serovar_Mo	0	0	-1000	-1000	-60.742162	-1000	-1000	0	0
Salmonella_enterica_subsp_enterica_serovar_Mo	0	0	-1000	-1000	-60.763023	-1000	-1000	0	0
Salmonella_enterica_subsp_enterica_serovar_Mo	0	0	-1000	-1000	-60.894953	-1000	-1000	0	0
Salmonella_enterica_subsp_enterica_serovar_Mo	0	0	-1000	-1000	-60.894953	-1000	-1000	0	0
Salmonella_enterica_subsp_enterica_serovar_Mo	0	0	-1000	-1000	-60.763027	-1000	-1000	0	0
Salmonella_enterica_subsp_enterica_serovar_Mo	0	0	-1000	-1000	-60.012862	-1000	-1000	0	0
Salmonella_enterica_subsp_enterica_serovar_Mo	0	0	-1000	-1000	-58.297367	-1000	-1000	0	0
Salmonella_enterica_subsp_enterica_serovar_Mo	0	0	-1000	-1000	-60.894953	-1000	-1000	0	0
Salmonella_enterica_subsp_enterica_serovar_Mo	0	0	-1000	-1000	-60.763023	-1000	-1000	0	0
Salmonella_enterica_subsp_enterica_serovar_Mo	0	0	-1000	-1000	-56.051277	-1000	-1000	0	0
Salmonella_enterica_subsp_enterica_serovar_Mo	0	0	-1000	-1000	-55.255247	-1000	-1000	0	0
Salmonella_enterica_subsp_enterica_serovar_Mo	0	0	-1000	-1000	-55.365263	-1000	-1000	0	0
Salmonella_enterica_subsp_enterica_serovar_Mo	0	0	-1000	-1000	-56.051277	-1000	-1000	0	0
Salmonella_enterica_subsp_enterica_serovar_Mo	0	0	-1000	-1000	-56.051277	-1000	-1000	0	0
Salmonella_enterica_subsp_enterica_serovar_Mo	0	0	-1000	-1000	-56.051277	-1000	-1000	0	0
Salmonella_enterica_subsp_enterica_serovar_Mo	0	0	-1000	-1000	-56.051277	-1000	-1000	0	0
Salmonella_enterica_subsp_enterica_serovar_Mo	0	0	-1000	-1000	-56.051277	-1000	-1000	0	0
Salmonella_enterica_subsp_enterica_serovar_Mo	0	0	-1000	-1000	-60.763027	-1000	-1000	0	0
Salmonella_enterica_subsp_enterica_serovar_Mo	0	0	-1000	-1000	-60.763023	-1000	-1000	0	0
Salmonella_enterica_subsp_enterica_serovar_Mo	0	0	-1000	-1000	-60.763027	-1000	-1000	0	0
Salmonella_enterica_subsp_enterica_serovar_Mu	0	0	-1000	-1000	-56.368738	-1000	-1000	0	0
Salmonella_enterica_subsp_enterica_serovar_Mu	0	0	-1000	-1000	-63.153236	-1000	-1000	0	0
Salmonella_enterica_subsp_enterica_serovar_Ne	0	0	-1000	-1000	-60.350729	-1000	-1000	0	0
Salmonella_enterica_subsp_enterica_serovar_Ne	0	0	-1000	-1000	-58.169003	-1000	-1000	0	0
Salmonella_enterica_subsp_enterica_serovar_Ne	0	0	-1000	-1000	-58.169003	-1000	-1000	0	0
Salmonella_enterica_subsp_enterica_serovar_Ne	0	0	-1000	-1000	-60.363394	-1000	-1000	0	0
Salmonella_enterica_subsp_enterica_serovar_Ne	0	0	-1000	-1000	-59.918046	-1000	-1000	0	0
Salmonella_enterica_subsp_enterica_serovar_Ne	0	0	-1000	-1000	-58.169003	-1000	-1000	0	0
Salmonella_enterica_subsp_enterica_serovar_Ne	0	0	-1000	-1000	-60.342909	-1000	-1000	0	0
Salmonella_enterica_subsp_enterica_serovar_Ne	0	0	-1000	-1000	-57.380847	-1000	-1000	0	0
Salmonella_enterica_subsp_enterica_serovar_Ne	0	0	-1000	-1000	-59.091701	-1000	-1000	0	0
Salmonella_enterica_subsp_enterica_serovar_Ne	0	0	-1000	-1000	-57.380847	-1000	-1000	0	0
Salmonella_enterica_subsp_enterica_serovar_Ne	0	0	-1000	-1000	-37.093603	-1000	-1000	0	0
Salmonella_enterica_subsp_enterica_serovar_Ne	0	0	-1000	-1000	-59.078479	-1000	-1000	0	0
Salmonella_enterica_subsp_enterica_serovar_Ne	0	0	-1000	-1000	-57.368893	-1000	-1000	0	0
Salmonella_enterica_subsp_enterica_serovar_Ne	0	0	-1000	-1000	-57.368893	-1000	-1000	0	0
Salmonella_enterica_subsp_enterica_serovar_Ne	0	0	-1000	-1000	-59.091701	-1000	-1000	0	0
Salmonella_enterica_subsp_enterica_serovar_Ne	0	0	-1000	-1000	-59.091701	-1000	-1000	0	0
Salmonella_enterica_subsp_enterica_serovar_Ne	0	0	-1000	-1000	-59.091701	-1000	-1000	0	0
Salmonella_enterica_subsp_enterica_serovar_Ne	0	0	-1000	-1000	-59.091701	-1000	-1000	0	0
Salmonella_enterica_subsp_enterica_serovar_Ne	0	0	-1000	-1000	-59.078479	-1000	-1000	0	0
Salmonella_enterica_subsp_enterica_serovar_Ne	0	0	-1000	-1000	-60.342911	-1000	-1000	0	0
Salmonella_enterica_subsp_enterica_serovar_Ne	0	0	-1000	-1000	-60.342909	-1000	-1000	0	0
Salmonella_enterica_subsp_enterica_serovar_Ne	0	0	-1000	-1000	-59.179484	-1000	-1000	0	0
Salmonella_enterica_subsp_enterica_serovar_Ne	0	0	-1000	-1000	-60.342909	-1000	-1000	0	0
Salmonella_enterica_subsp_enterica_serovar_Ne	0	0	-1000	-1000	-58.169003	-1000	-1000	0	0
Salmonella_enterica_subsp_enterica_serovar_Ne	0	0	-1000	-1000	-58.499613	-1000	-1000	0	0
Salmonella_enterica_subsp_enterica_serovar_Ne	0	0	-1000	-1000	-58.288742	-1000	-1000	0	0
Salmonella_enterica_subsp_enterica_serovar_Ne	0	0	-1000	-1000	-58.888946	-1000	-1000	0	0
Salmonella_enterica_subsp_enterica_serovar_Ne	0	0	-1000	-1000	-60.350729	-1000	-1000	0	0
Salmonella_enterica_subsp_enterica_serovar_Ne	0	0	-1000	-1000	-59.995421	-1000	-1000	0	0
Salmonella_enterica_subsp_enterica_serovar_Ne	-1000	0	-1000	-1000	-60.342911	-1000	-1000	0	0
Salmonella_enterica_subsp_enterica_serovar_Ne	0	0	-1000	-1000	-59.091701	-1000	-1000	0	0
Salmonella_enterica_subsp_enterica_serovar_Par	-1000	0	-1000	-1000	-54.224144	-1000	-1000	0	0
Salmonella_enterica_subsp_enterica_serovar_Par	0	0	-1000	-1000	-49.40759	-1000	-1000	0	0
Salmonella_enterica_subsp_enterica_serovar_Par	0	0	-1000	-1000	-54.216957	-1000	-1000	0	0
Salmonella_enterica_subsp_enterica_serovar_Par	0	0	-1000	-1000	-54.224144	-1000	-1000	0	0
Salmonella_enterica_subsp_enterica_serovar_Par	0	0	-1000	-1000	-54.224144	-1000	-1000	0	0
Salmonella_enterica_subsp_enterica_serovar_Par	0	0	-1000	-1000	-54.224144	-1000	-1000	0	0
Salmonella_enterica_subsp_enterica_serovar_Par	-1000	0	-1000	-1000	-60.35557	-1000	-1000	0	0
Salmonella_enterica_subsp_enterica_serovar_Par	-1000	0	-1000	-1000	-59.745274	-1000	-1000	0	0
Salmonella_enterica_subsp_enterica_serovar_Por	0	0	-1000	-1000	-58.150053	-1000	-1000	0	0
Salmonella_enterica_subsp_enterica_serovar_Po	0	0	-1000	-1000	-60.742159	-1000	-1000	0	0
Salmonella_enterica_subsp_enterica_serovar_Sai	0	0	-1000	-1000	-58.181585	-1000	-1000	0	0
Salmonella_enterica_subsp_enterica_serovar_Sch	-1000	0	-1000	-1000	-66.099993	-1000	-1000	0	0
Salmonella_enterica_subsp_enterica_serovar_Sch	0	0	-1000	-1000	-69.505623	-1000	-1000	0	0
Salmonella_enterica_subsp_enterica_serovar_Ser	0	0	-1000	-1000	-58.052386	-1000	-1000	0	0
Salmonella_enterica_subsp_enterica_serovar_Sta	0	0	-1000	-1000	-59.545871	-1000	-1000	0	0
Salmonella_enterica_subsp_enterica_serovar_Ter	0	0	-1000	-1000	-59.855303	-1000	-1000	0	0
Salmonella_enterica_subsp_enterica_serovar_Th	0	0	-1000	-1000	-56.012899	-1000	-1000	0	0
Salmonella_enterica_subsp_enterica_serovar_Typ	0	0	-1000	-1000	-55.73277	-1000	-1000	0	0
Salmonella_enterica_subsp_enterica_serovar_Typ	0	0	-1000	-1000	-56.386996	-1000	-1000	0	0
Salmonella_enterica_subsp_enterica_serovar_Typ	-1000	0	-1000	-1000	-55.807297	-1000	-1000	0	0
Salmonella_enterica_subsp_enterica_serovar_Typ	0	0	-1000	-1000	-59.403414	-1000	-1000	0	0
Salmonella_enterica_subsp_enterica_serovar_Typ	-1000	0	-1000	-1000	-55.501138	-1000	-1000	0	0

Salmonella_enterica_subsp_enterica_serovar_Typ	0	0	-1000	-1000	-57.869993	-1000	-1000	0	0
Salmonella_enterica_subsp_enterica_serovar_Typ	-1000	0	-1000	-1000	-56.267359	-1000	-1000	0	0
Salmonella_enterica_subsp_enterica_serovar_Typ	-1000	0	-1000	-1000	-56.26736	-1000	-1000	0	0
Salmonella_enterica_subsp_enterica_serovar_Typ	0	0	-1000	-1000	-60.768752	-1000	-1000	0	0
Salmonella_enterica_subsp_enterica_serovar_Typ	-1000	0	-1000	-1000	-55.115062	-1000	-1000	0	0
Salmonella_enterica_subsp_enterica_serovar_Typ	0	0	-1000	-1000	-66.114875	-1000	-1000	0	0
Salmonella_enterica_subsp_enterica_serovar_Typ	-1000	0	-1000	-1000	-58.120678	-1000	-1000	0	0
Salmonella_enterica_subsp_enterica_serovar_Typ	-1000	0	-1000	-1000	-57.949416	-1000	-1000	0	0
Salmonella_enterica_subsp_enterica_serovar_Typ	0	0	-1000	-1000	-66.114875	-1000	-1000	0	0
Salmonella_enterica_subsp_enterica_serovar_Typ	0	0	-1000	-1000	-58.13303	-1000	-1000	0	0
Salmonella_enterica_subsp_enterica_serovar_Typ	0	0	-1000	-1000	-58.13303	-1000	-1000	0	0
Salmonella_enterica_subsp_enterica_serovar_Typ	0	0	-1000	-1000	-58.13303	-1000	-1000	0	0
Salmonella_enterica_subsp_enterica_serovar_Typ	0	0	-1000	-1000	-58.13303	-1000	-1000	0	0
Salmonella_enterica_subsp_enterica_serovar_Typ	0	0	-1000	-1000	-54.700408	-1000	-1000	0	0
Salmonella_enterica_subsp_enterica_serovar_Typ	0	0	-1000	-1000	-58.13303	-1000	-1000	0	0
Salmonella_enterica_subsp_enterica_serovar_Typ	0	0	-1000	-1000	-58.13303	-1000	-1000	0	0
Salmonella_enterica_subsp_enterica_serovar_Typ	0	0	-1000	-1000	-58.582274	-1000	-1000	0	0
Salmonella_enterica_subsp_enterica_serovar_Typ	0	0	-1000	-1000	-58.13303	-1000	-1000	0	0
Salmonella_enterica_subsp_enterica_serovar_Typ	0	0	-1000	-1000	-58.13303	-1000	-1000	0	0
Salmonella_enterica_subsp_enterica_serovar_Typ	-1000	0	-1000	-1000	-66.114875	-1000	-1000	0	0
Salmonella_enterica_subsp_enterica_serovar_Typ	0	0	-1000	-1000	-53.078832	-1000	-1000	0	0
Salmonella_enterica_subsp_enterica_serovar_Typ	-1000	0	-1000	-1000	-58.120678	-1000	-1000	0	0
Salmonella_enterica_subsp_enterica_serovar_Typ	-1000	0	-1000	-1000	-55.115062	-1000	-1000	0	0
Salmonella_enterica_subsp_enterica_serovar_Vir	0	0	-1000	-1000	-60.407963	-1000	-1000	0	0
Salmonella_enterica_subsp_enterica_serovar_We	0	0	-1000	-1000	-59.977295	-1000	-1000	0	0
Salmonella_enterica_subsp_houtenae_serovar_1	0	0	-1000	-1000	-56.810815	-1000	-1000	0	0
Salmonella_enterica_subsp_houtenae_str_ATCC_	0	0	-1000	-1000	-54.824519	-1000	-1000	0	0
Salmonella_enterica_subsp_salamae_str_3588_0	0	0	-1000	-1000	-57.598406	-1000	-1000	0	0
Sarcina_nov_ERR1022285	0	0	0	-54.71697655	-27.702062	-1000	0	-1000	0
Sarcina_nov_ERR1022316	0	0	0	-54.71701209	-27.70208	-1000	0	-1000	0
Sarcina_nov_ERR1022347	0	0	0	-1000	-47.352979	0	0	0	0
Sarcina_nov_ERR1022371	0	0	0	-54.71700024	-27.702074	-1000	0	-1000	0
Sarcina_nov_ERR1022372	0	0	0	-37.67546009	-26.706655	0	0	-926.22951	0
Sarcina_nov_ERR1022421	0	0	0	-1000	-36.765255	0	0	0	0
Sarcina_nov_ERR1022468	0	0	0	-54.71699673	-27.702072	-1000	0	-1000	0
Sarcina_nov_ERR1203922	0	0	0	-1000	-31.985222	0	0	-1000	0
Sarcina_nov_ERR1203929	0	0	0	-1000	-47.993605	0	0	-1000	0
Sarcina_nov_ERR1203932	0	0	0	-1000	-31.664332	0	0	0	0
Sarcina_nov_ERR1204023	0	0	0	-1000	-47.993607	0	0	-1000	0
Sarcina_nov_ERR1204026	0	0	0	-1000	-31.664326	0	0	0	0
Sarcina_nov_ERR171255	0	0	0	-1000	-47.35298	0	0	-1000	0
Sarcina_nov_ERR171264	0	0	0	-47.78659921	-24.570918	0	0	0	0
Sarcina_nov_ERR171266	0	0	0	-54.71697786	-27.702063	-1000	0	-1000	0
Sarcina_nov_ERR171270	0	0	0	-55.22551024	-28.345449	0	0	-443.33807	0
Sarcina_nov_ERR2221152	0	0	0	-47.31051464	-24.300676	-849.53773	0	-422.5861	0
Sarcina_nov_ERR2221229	0	0	0	-1000	-31.985222	0	0	-1000	0
Sarcina_nov_ERR2221238	0	0	0	-55.09425907	-27.893072	0	0	-625	0
Sarcina_nov_ERR2221241	0	0	0	-1000	-38.594945	0	0	0	0
Sarcina_nov_ERR2221248	0	0	0	-1000	-47.331873	0	0	-1000	0
Sarcina_nov_ERR2221284	0	0	0	-54.97828992	-27.834359	-1000	0	-759.24738	0
Sarcina_nov_ERR2221290	0	0	0	-1000	-48.015329	0	0	-1000	0
Sarcina_nov_ERR2221381	0	0	0	-47.31052688	-24.300686	-849.53773	0	-422.58611	0
Sarcina_nov_ERR2230135	0	0	0	-55.13041888	-28.006708	0	0	-445.09804	0
Sarcina_nov_ERR2230149	0	0	0	-1000	-31.664339	0	0	0	0
Sarcina_ventriculi_ERR1022374	0	0	0	-38.87435417	-27.518421	-562.9978	0	-281.4989	0
Scardovia_inopinata_F0304	0	0	-1000	-23.82091124	-19.389301	0	0	-1000	0
Scardovia_wiggsiae_F0424	0	0	0	0	-18.290759	0	0	-524.72263	0
Schlesneria_paludicola_DSM_18645	0	0	0	-1000	0	0	0	-1000	0
Schwartzia_succinivorans_DSM_10502	0	0	0	-1000	-10.397448	0	0	0	0
Sediminibacterium_salmoneum_NBRC_103935	0	0	0	-1000	0	0	0	-258.89216	0
Selenomonas_bovis_8_14_1	0	0	0	-1000	-28.575171	0	0	0	0
Selenomonas_bovis_DSM_23594	0	0	0	-1000	-28.314114	0	0	-476.05178	0
Selenomonas_flueggei_ATCC_43531	0	0	0	-1000	-41.275286	-1000	0	-750	0
Selenomonas_infelix_ATCC_43532	0	0	0	0	-35.462542	-1000	0	-649.65986	0
Selenomonas_noxia_ATCC_43541	0	0	0	0	-29.961504	-1000	0	-546.51163	0
Selenomonas_noxia_F0398	0	0	0	0	-29.77309	-1000	0	-546.51163	0
Selenomonas_ruminantium_AC2024	0	0	0	-1000	-46.636154	-1000	0	-826.08696	0
Selenomonas_ruminantium_L14	0	0	0	-1000	-35.000491	-990.04975	0	-529.4319	0
Selenomonas_ruminantium_subsp_ruminantium_	0	0	0	-1000	-21.638224	-992.03187	0	-529.60526	0
Selenomonas_sp_F0473	0	0	0	0	-31.77123	-984.1629	0	-494.94098	0
Selenomonas_sputigena_ATCC_35185	0	0	0	-1000	-24.825606	0	0	-500	0
Sellimonas_intestinalis_BR72	0	0	0	0	-16.736115	0	0	-595.65625	0
Senegalimassilia_anaerobia_JC110	0	0	0	-1000	-10.409378	0	0	0	0
Serratia_fonticola_GS2	0	-1000	-1000	-1000	-61.398728	-1000	0	0	0
Serratia_fonticola_RB_25	0	-1000	-1000	-1000	-70.553109	-1000	0	0	0
Serratia_liquefaciens_ATCC_27592	-1000	0	-1000	-1000	-59.484827	-1000	0	-1000	0
Serratia_liquefaciens_FDAARGOS_125	0	0	-1000	-1000	-39.610285	-1000	0	-1000	0
Serratia_marcescens_LCT_SM213	0	-1000	-1000	-1000	-44.622389	-1000	0	-1000	0
Serratia_marcescens_subsp_marcescens_Db11	0	-1000	-1000	-1000	-64.780992	-1000	0	-1000	0
Serratia_marcescens_UCI88	0	-1000	-1000	-1000	-58.162005	-1000	0	-1000	0
Serratia_marcescens_WW4	-1000	-1000	-1000	-1000	-43.607926	-1000	0	-1000	0
Serratia_odorifera_DSM_4582	0	-1000	-1000	-1000	-51.711843	-1000	0	0	0
Serratia_rubidaea_NBRC_103169	0	0	-1000	-1000	-35.18084	-1000	0	-1000	0
Serratia_rubidaea_NCTC10848	0	0	-1000	-1000	-33.400469	-1000	0	-1000	0
Serratia_sp_FGI94	0	0	-1000	-1000	-39.24482	-1000	0	0	0
Shewanella_halifaxensis_HAW_EB4	0	0	0	-1000	-1000	0	-1000	0	0
Shewanella_woodyi_ATCC_51908	0	0	-1000	-1000	-1000	0	0	0	0
Shigella_dysenteriae_155_74	0	0	0	-1000	-37.901326	0	0	0	0
Shigella_dysenteriae_1617	-1000	0	0	-1000	-48.048918	0	0	-1000	0
Shigella_dysenteriae_225_75	0	0	-1000	-1000	-48.179902	0	0	0	0
Shigella_dysenteriae_CDC_74_1112	0	0	0	-1000	-48.24939	0	0	0	0

Shigella_dysenteriae_Sd197	-1000	0	-1000	-1000	-43.980817	0	0	-1000	0
Shigella_flexneri_1485_80	0	0	-1000	-1000	-37.91763	-1000	0	0	0
Shigella_flexneri_2002017	-1000	0	-1000	-1000	-63.836218	-1000	0	-1000	0
Shigella_flexneri_2747_71	0	0	-1000	-1000	-38.202723	-1000	0	0	0
Shigella_flexneri_2850_71	0	0	-1000	-1000	-38.164942	-1000	0	0	0
Shigella_flexneri_2930_71	0	0	-1000	-1000	-37.993451	-1000	0	0	0
Shigella_flexneri_2a_str_2457T	-1000	0	-1000	-1000	-38.16569	-1000	0	0	0
Shigella_flexneri_2a_str_301	-1000	0	-1000	-1000	-38.12749	-1000	0	0	0
Shigella_flexneri_2a_strain_BS1025	0	0	-1000	-1000	-33.589194	-1000	0	0	0
Shigella_flexneri_4343_70	0	0	-1000	-1000	-37.979386	-1000	0	0	0
Shigella_flexneri_5_str_8401	-1000	0	-1000	-1000	-38.359751	-1000	0	0	0
Shigella_flexneri_5a_str_M90T	0	0	-1000	-1000	-38.190129	-1000	0	0	0
Shigella_flexneri_6603_63	0	0	-1000	-1000	-38.190189	-1000	0	0	0
Shigella_flexneri_CCH060	0	0	-1000	-1000	-38.118491	-1000	0	0	0
Shigella_flexneri_CDC_796_83	0	0	0	-1000	-38.118415	-1000	0	0	0
Shigella_flexneri_K_218	0	0	-1000	-1000	-38.164866	-1000	0	0	0
Shigella_flexneri_K_227	0	0	-1000	-1000	-38.201623	-1000	0	0	0
Shigella_flexneri_K_272	0	0	0	-1000	-47.532359	-1000	0	0	0
Shigella_flexneri_K_304	0	0	-1000	-1000	-38.190113	-1000	0	0	0
Shigella_flexneri_K_315	0	0	0	-1000	-27.950939	-1000	0	0	0
Shigella_flexneri_SFJ17B	0	0	-1000	-1000	-38.164942	-1000	0	0	0
Shigella_flexneri_VA_6	0	0	-1000	-1000	-38.157573	-1000	0	0	0
Shigella_sonnei_3226_85	0	0	-1000	-1000	-38.642525	-1000	0	-1000	0
Shigella_sonnei_3233_85	0	0	-1000	-1000	-38.282206	-1000	0	-1000	0
Shigella_sonnei_4822_66	0	0	-1000	-1000	-38.190464	-1000	0	-1000	0
Shigella_sonnei_53G	-1000	0	-1000	-1000	-38.642437	-1000	0	-1000	0
Shigella_sonnei_FDAARGOS_128	0	0	-1000	-1000	-38.642517	-1000	0	-1000	0
Shigella_sonnei_FDAARGOS_71	0	0	-1000	-1000	-38.64251	-1000	0	-1000	0
Shigella_sonnei_Ss046	-1000	0	-1000	-1000	-63.836173	-1000	0	-1000	0
Shigella_sonnei_str_Moseley	0	0	-1000	-1000	-38.282104	-1000	0	-1000	0
Shuttleworthia_satelles_DSM_14600	0	0	-1000	-54.73001575	-26.819746	0	0	-397.72945	0
Silanimonas_lenta_DSM_16282	0	0	0	-1000	0	0	0	0	0
Sinobacterium_caligoides_DSM_100316	0	0	0	-1000	-1000	0	0	0	0
Slackia_equolifaciens_DSM_24851	0	0	0	-920.2329779	-22.197239	0	0	0	0
Slackia_exigua_ATCC_700122	0	0	0	-875	0	0	0	0	0
Slackia_faecicanis_DSM_17537	0	0	0	-16.38809195	-11.523388	0	0	0	0
Slackia_piriformis_YIT_12062	0	0	0	-19.80218429	0	0	0	0	0
Sneathia_sanguinegens_CCUG41628	0	0	0	-10.96859477	-16.042733	0	0	0	0
Solobacterium_moorei_DSM_22971	0	0	0	0	-18.989922	0	0	0	0
Solobacterium_moorei_F0204	0	0	0	0	-19.397148	0	0	-271.42589	0
Sphingobium_amiense_NBRC_102518	0	0	0	-1000	0	0	0	0	0
Sphingomonas_paucimobilis_HER1398	0	0	0	-1000	-27.081115	0	0	0	0
Sphingosinicella_microcystinivorans_B9	0	0	0	-1000	-1000	0	0	0	0
Sphingosinicella_microcystinivorans_DSM_19791	0	0	0	-1000	-1000	0	0	0	0
Spiroplasma_culicicola_AES_1	0	0	0	0	-10.769205	0	0	0	0
Spirosoma_linguale_DSM_74	0	0	0	-40.81710018	0	0	0	-1000	0
Sporobacter_termitidis_DSM_10068	0	0	0	-1000	-15.232551	0	0	0	0
Staphylococcus_arlettae_CVD059	0	0	0	-1000	-32.903923	-1000	0	-1000	0
Staphylococcus_aureus_04_02981	0	0	0	-1000	-38.161157	-1000	0	-1000	0
Staphylococcus_aureus_08BA02176	0	0	0	-1000	-38.097961	-1000	0	-1000	0
Staphylococcus_aureus_16K	0	0	0	-1000	-45.610685	-1000	0	-1000	0
Staphylococcus_aureus_3957	0	0	0	-1000	-38.095396	-1000	0	-1000	0
Staphylococcus_aureus_930918_3	0	0	0	-1000	-45.521708	-1000	0	-1000	0
Staphylococcus_aureus_A10102	0	0	0	-1000	-38.161157	-1000	0	-1000	0
Staphylococcus_aureus_A5937	0	0	0	-1000	-38.161157	-1000	0	-1000	0
Staphylococcus_aureus_A5948	0	0	0	-1000	-45.610685	-1000	0	-1000	0
Staphylococcus_aureus_A6224	0	0	0	-1000	-38.161157	-1000	0	-1000	0
Staphylococcus_aureus_A6300	0	0	0	-1000	-45.610685	-1000	0	-1000	0
Staphylococcus_aureus_A8115	0	0	0	-1000	-38.161157	-1000	0	-1000	0
Staphylococcus_aureus_A8117	0	0	0	-1000	-38.161157	-1000	0	-1000	0
Staphylococcus_aureus_A8796	0	0	0	-1000	-38.161157	-1000	0	-1000	0
Staphylococcus_aureus_A8819	0	0	0	-1000	-38.161157	-1000	0	-1000	0
Staphylococcus_aureus_A9299	0	0	0	-1000	-38.161157	-1000	0	-1000	0
Staphylococcus_aureus_A9635	0	0	0	-1000	-45.533319	-1000	0	-1000	0
Staphylococcus_aureus_A9719	0	0	0	-1000	-38.161157	-1000	0	-1000	0
Staphylococcus_aureus_A9754	0	0	0	-1000	-45.610685	-1000	0	-1000	0
Staphylococcus_aureus_A9765	0	0	0	-1000	-45.90837	-1000	0	-1000	0
Staphylococcus_aureus_A9781	0	0	0	-1000	-38.161157	-1000	0	-1000	0
Staphylococcus_aureus_B147830	0	0	0	-1000	-38.161157	-1000	0	-1000	0
Staphylococcus_aureus_B40723	0	0	0	-1000	-38.161157	-1000	0	-1000	0
Staphylococcus_aureus_B40950	0	0	0	-1000	-38.161157	-1000	0	-1000	0
Staphylococcus_aureus_B53639	0	0	0	-1000	-38.161157	-1000	0	-1000	0
Staphylococcus_aureus_CA_347	0	0	0	-1000	-45.533319	-1000	0	-1000	0
Staphylococcus_aureus_CN79	0	0	0	-1000	-45.533319	-1000	0	-1000	0
Staphylococcus_aureus_D30	0	0	0	-1000	-45.610685	-1000	0	-1000	0
Staphylococcus_aureus_HI010	0	0	0	-1000	-45.533319	-1000	0	-1000	0
Staphylococcus_aureus_HI010B	0	0	0	-1000	-45.533319	-1000	0	-1000	0
Staphylococcus_aureus_HI013	0	0	0	-1000	-45.829992	-1000	0	-1000	0
Staphylococcus_aureus_HI022	0	0	0	-1000	-45.533319	-1000	0	-1000	0
Staphylococcus_aureus_HI049	0	0	0	-1000	-45.533319	-1000	0	-1000	0
Staphylococcus_aureus_HI049B	0	0	0	-1000	-45.533319	-1000	0	-1000	0
Staphylococcus_aureus_HI049C	0	0	0	-1000	-45.533319	-1000	0	-1000	0
Staphylococcus_aureus_HI111	0	0	0	-1000	-45.533319	-1000	0	-1000	0
Staphylococcus_aureus_HI168	0	0	0	-1000	-45.533319	-1000	0	-1000	0
Staphylococcus_aureus_HIF003_B2N_C	0	0	0	-1000	-45.533319	-1000	0	-1000	0
Staphylococcus_aureus_KLT6	0	0	0	-1000	-38.095396	-1000	0	-1000	0
Staphylococcus_aureus_KT_314250	0	0	0	-1000	-45.536337	-1000	0	-1000	0
Staphylococcus_aureus_KT_Y21	0	0	0	-1000	-38.095396	-1000	0	-1000	0
Staphylococcus_aureus_LVP2	0	0	0	-1000	-38.095396	-1000	0	-1000	0
Staphylococcus_aureus_LVP5	0	0	0	-1000	-45.533319	-1000	0	-1000	0
Staphylococcus_aureus_M0001	0	0	0	-1000	-38.161157	-1000	0	-1000	0

Staphylococcus_aureus_M0006	0	0	0	-1000	-38.161157	-1000	0	-1000	0
Staphylococcus_aureus_M0029	0	0	0	-1000	-38.161157	-1000	0	-1000	0
Staphylococcus_aureus_M0035	0	0	0	-1000	-38.903053	-1000	0	-1000	0
Staphylococcus_aureus_M0045	0	0	0	-1000	-38.161157	-1000	0	-1000	0
Staphylococcus_aureus_M0055	0	0	0	-1000	-45.533319	-1000	0	-1000	0
Staphylococcus_aureus_M0060	0	0	0	-1000	-26.164031	-1000	0	-1000	0
Staphylococcus_aureus_M0066	0	0	0	-1000	-26.164016	-1000	0	-1000	0
Staphylococcus_aureus_M0075	0	0	0	-1000	-26.164012	-1000	0	-1000	0
Staphylococcus_aureus_M0077	0	0	0	-1000	-26.164047	-1000	0	-1000	0
Staphylococcus_aureus_M0102	0	0	0	-1000	-26.164012	-1000	0	-1000	0
Staphylococcus_aureus_M0103	0	0	0	-1000	-26.164035	-1000	0	-1000	0
Staphylococcus_aureus_M0104	0	0	0	-1000	-26.164051	-1000	0	-1000	0
Staphylococcus_aureus_M0108	0	0	0	-1000	-26.16402	-1000	0	-1000	0
Staphylococcus_aureus_M0144	0	0	0	-1000	-26.164043	-1000	0	-1000	0
Staphylococcus_aureus_M0150	0	0	0	-1000	-26.164051	-1000	0	-1000	0
Staphylococcus_aureus_M0154	0	0	0	-1000	-26.103123	-1000	0	-1000	0
Staphylococcus_aureus_M0171	0	0	0	-1000	-26.164039	-1000	0	-1000	0
Staphylococcus_aureus_M0173	0	0	0	-1000	-26.16402	-1000	0	-1000	0
Staphylococcus_aureus_M0177	0	0	0	-1000	-26.103126	-1000	0	-1000	0
Staphylococcus_aureus_M0192	0	0	0	-1000	-26.164012	-1000	0	-1000	0
Staphylococcus_aureus_M0197	0	0	0	-1000	-26.16406	-1000	0	-1000	0
Staphylococcus_aureus_M0200	0	0	0	-1000	-26.164039	-1000	0	-1000	0
Staphylococcus_aureus_M0210	0	0	0	-1000	-26.103131	-1000	0	-1000	0
Staphylococcus_aureus_M0212	0	0	0	-1000	-26.164012	-1000	0	-1000	0
Staphylococcus_aureus_M0213	0	0	0	-1000	-26.103131	-1000	0	-1000	0
Staphylococcus_aureus_M0216	0	0	0	-1000	-26.103128	-1000	0	-1000	0
Staphylococcus_aureus_M0221	0	0	0	-1000	-28.92112	-1000	0	-1000	0
Staphylococcus_aureus_M0235	0	0	0	-1000	-26.103128	-1000	0	-1000	0
Staphylococcus_aureus_M0237	0	0	0	-1000	-26.135822	-1000	0	-1000	0
Staphylococcus_aureus_M0239	0	0	0	-1000	-28.708139	-1000	0	-1000	0
Staphylococcus_aureus_M0240	0	0	0	-1000	-26.164012	-1000	0	-1000	0
Staphylococcus_aureus_M0250	0	0	0	-1000	-26.164012	-1000	0	-1000	0
Staphylococcus_aureus_M0252	0	0	0	-1000	-26.164012	-1000	0	-1000	0
Staphylococcus_aureus_M0270	0	0	0	-1000	-26.16402	-1000	0	-1000	0
Staphylococcus_aureus_M0273	0	0	0	-1000	-26.164039	-1000	0	-1000	0
Staphylococcus_aureus_M0279	0	0	0	-1000	-26.39949	-1000	0	-1000	0
Staphylococcus_aureus_M0280	0	0	0	-1000	-26.16402	-1000	0	-1000	0
Staphylococcus_aureus_M0288	0	0	0	-1000	-26.164043	-1000	0	-1000	0
Staphylococcus_aureus_M0294	0	0	0	-1000	-26.164035	-1000	0	-1000	0
Staphylococcus_aureus_M0306	0	0	0	-1000	-26.164016	-1000	0	-1000	0
Staphylococcus_aureus_M0312	0	0	0	-1000	-28.921128	-1000	0	-1000	0
Staphylococcus_aureus_M0326	0	0	0	-1000	-26.164039	-1000	0	-1000	0
Staphylococcus_aureus_M0328	0	0	0	-1000	-26.164039	-1000	0	-1000	0
Staphylococcus_aureus_M0329	0	0	0	-1000	-26.135822	-1000	0	-1000	0
Staphylococcus_aureus_M0330	0	0	0	-1000	-26.164012	-1000	0	-1000	0
Staphylococcus_aureus_M0334	0	0	0	-1000	-26.16407	-1000	0	-1000	0
Staphylococcus_aureus_M0340	0	0	0	-1000	-28.92112	-1000	0	-1000	0
Staphylococcus_aureus_M0347	0	0	0	-1000	-26.164012	-1000	0	-1000	0
Staphylococcus_aureus_M0350	0	0	0	-1000	-26.164066	-1000	0	-1000	0
Staphylococcus_aureus_M0351	0	0	0	-1000	-26.164023	-1000	0	-1000	0
Staphylococcus_aureus_M0363	0	0	0	-1000	-26.103123	-1000	0	-1000	0
Staphylococcus_aureus_M0364	0	0	0	-1000	-26.164012	-1000	0	-1000	0
Staphylococcus_aureus_M0367	0	0	0	-1000	-26.103126	-1000	0	-1000	0
Staphylococcus_aureus_M0374	0	0	0	-1000	-26.103133	-1000	0	-1000	0
Staphylococcus_aureus_M0375	0	0	0	-1000	-26.164035	-1000	0	-1000	0
Staphylococcus_aureus_M0391	0	0	0	-1000	-26.438344	-1000	0	-1000	0
Staphylococcus_aureus_M0396	0	0	0	-1000	-28.708146	-1000	0	-1000	0
Staphylococcus_aureus_M0404	0	0	0	-1000	-26.103126	-1000	0	-1000	0
Staphylococcus_aureus_M0408	0	0	0	-1000	-26.135837	-1000	0	-1000	0
Staphylococcus_aureus_M0415	0	0	0	-1000	-26.164016	-1000	0	-1000	0
Staphylococcus_aureus_M0424	0	0	0	-1000	-28.921128	-1000	0	-1000	0
Staphylococcus_aureus_M0427	0	0	0	-1000	-26.164012	-1000	0	-1000	0
Staphylococcus_aureus_M0438	0	0	0	-1000	-26.164016	-1000	0	-1000	0
Staphylococcus_aureus_M0450	0	0	0	-1000	-26.103143	-1000	0	-1000	0
Staphylococcus_aureus_M0455	0	0	0	-1000	-26.164023	-1000	0	-1000	0
Staphylococcus_aureus_M0460	0	0	0	-1000	-26.103128	-1000	0	-1000	0
Staphylococcus_aureus_M0467	0	0	0	-1000	-26.164012	-1000	0	-1000	0
Staphylococcus_aureus_M0468	0	0	0	-1000	-28.921135	-1000	0	-1000	0
Staphylococcus_aureus_M0478	0	0	0	-1000	-26.438349	-1000	0	-1000	0
Staphylococcus_aureus_M0489	0	0	0	-1000	-26.164035	-1000	0	-1000	0
Staphylococcus_aureus_M0493	0	0	0	-1000	-26.103121	-1000	0	-1000	0
Staphylococcus_aureus_M0494	0	0	0	-1000	-26.164023	-1000	0	-1000	0
Staphylococcus_aureus_M0510	0	0	0	-1000	-26.16402	-1000	0	-1000	0
Staphylococcus_aureus_M0513	0	0	0	-1000	-26.135837	-1000	0	-1000	0
Staphylococcus_aureus_M0528	0	0	0	-1000	-28.955997	-1000	0	-1000	0
Staphylococcus_aureus_M0529	0	0	0	-1000	-26.164031	-1000	0	-1000	0
Staphylococcus_aureus_M0531	0	0	0	-1000	-28.941763	-1000	0	-1000	0
Staphylococcus_aureus_M0536	0	0	0	-1000	-26.438349	-1000	0	-1000	0
Staphylococcus_aureus_M0539	0	0	0	-1000	-26.16402	-1000	0	-1000	0
Staphylococcus_aureus_M0547	0	0	0	-1000	-26.164023	-1000	0	-1000	0
Staphylococcus_aureus_M0562	0	0	0	-1000	-26.164027	-1000	0	-1000	0
Staphylococcus_aureus_M0565	0	0	0	-1000	-26.164023	-1000	0	-1000	0
Staphylococcus_aureus_M0571	0	0	0	-1000	-26.164023	-1000	0	-1000	0
Staphylococcus_aureus_M0580	0	0	0	-1000	-26.103128	-1000	0	-1000	0
Staphylococcus_aureus_M0584	0	0	0	-1000	-26.438344	-1000	0	-1000	0
Staphylococcus_aureus_M0586	0	0	0	-1000	-26.164055	-1000	0	-1000	0
Staphylococcus_aureus_M0602	0	0	0	-1000	-26.164027	-1000	0	-1000	0
Staphylococcus_aureus_M0622	0	0	0	-1000	-26.16402	-1000	0	-1000	0
Staphylococcus_aureus_M0628	0	0	0	-1000	-26.103128	-1000	0	-1000	0
Staphylococcus_aureus_M0633	0	0	0	-1000	-28.921144	-1000	0	-1000	0
Staphylococcus_aureus_M0646	0	0	0	-1000	-26.16402	-1000	0	-1000	0

Staphylococcus_aureus_M0648	0	0	0	-1000	-26.164023	-1000	0	-1000	0
Staphylococcus_aureus_M0660	0	0	0	-1000	-26.164023	-1000	0	-1000	0
Staphylococcus_aureus_M0663	0	0	0	-1000	-26.155159	-1000	0	-1000	0
Staphylococcus_aureus_M0673	0	0	0	-1000	-26.16402	-1000	0	-1000	0
Staphylococcus_aureus_M0676	0	0	0	-1000	-26.103131	-1000	0	-1000	0
Staphylococcus_aureus_M0687	0	0	0	-1000	-28.955997	-1000	0	-1000	0
Staphylococcus_aureus_M0692	0	0	0	-1000	-26.164035	-1000	0	-1000	0
Staphylococcus_aureus_M0695	0	0	0	-1000	-26.164016	-1000	0	-1000	0
Staphylococcus_aureus_M0719	0	0	0	-1000	-26.164051	-1000	0	-1000	0
Staphylococcus_aureus_M0769	0	0	0	-1000	-26.103121	-1000	0	-1000	0
Staphylococcus_aureus_M0770	0	0	0	-1000	-26.164016	-1000	0	-1000	0
Staphylococcus_aureus_M0780	0	0	0	-1000	-26.164016	-1000	0	-1000	0
Staphylococcus_aureus_M0792	0	0	0	-1000	-26.103136	-1000	0	-1000	0
Staphylococcus_aureus_M0799	0	0	0	-1000	-26.164035	-1000	0	-1000	0
Staphylococcus_aureus_M0822	0	0	0	-1000	-26.103121	-1000	0	-1000	0
Staphylococcus_aureus_M0823	0	0	0	-1000	-26.164035	-1000	0	-1000	0
Staphylococcus_aureus_M0831	0	0	0	-1000	-28.921128	-1000	0	-1000	0
Staphylococcus_aureus_M0844	0	0	0	-1000	-26.164016	-1000	0	-1000	0
Staphylococcus_aureus_M0871	0	0	0	-1000	-26.103136	-1000	0	-1000	0
Staphylococcus_aureus_M0877	0	0	0	-1000	-28.089316	-1000	0	-1000	0
Staphylococcus_aureus_M0892	0	0	0	-1000	-26.164016	-1000	0	-1000	0
Staphylococcus_aureus_M0900	0	0	0	-1000	-26.430245	-1000	0	-1000	0
Staphylococcus_aureus_M0927	0	0	0	-1000	-26.103118	-1000	0	-1000	0
Staphylococcus_aureus_M0934	0	0	0	-1000	-26.164039	-1000	0	-1000	0
Staphylococcus_aureus_M0943	0	0	0	-1000	-26.164016	-1000	0	-1000	0
Staphylococcus_aureus_M0944	0	0	0	-1000	-26.103121	-1000	0	-1000	0
Staphylococcus_aureus_M0946	0	0	0	-1000	-26.135826	-1000	0	-1000	0
Staphylococcus_aureus_M0953	0	0	0	-1000	-26.164008	-1000	0	-1000	0
Staphylococcus_aureus_M0964	0	0	0	-1000	-26.103118	-1000	0	-1000	0
Staphylococcus_aureus_M0978	0	0	0	-1000	-26.164016	-1000	0	-1000	0
Staphylococcus_aureus_M0994	0	0	0	-1000	-26.103123	-1000	0	-1000	0
Staphylococcus_aureus_M0998	0	0	0	-1000	-26.164016	-1000	0	-1000	0
Staphylococcus_aureus_M0999	0	0	0	-1000	-26.164016	-1000	0	-1000	0
Staphylococcus_aureus_M1	0	0	0	-1000	-28.921127	-1000	0	-1000	0
Staphylococcus_aureus_M1007	0	0	0	-1000	-28.921123	-1000	0	-1000	0
Staphylococcus_aureus_M1010	0	0	0	-1000	-26.103128	-1000	0	-1000	0
Staphylococcus_aureus_M1015	0	0	0	-1000	-26.164023	-1000	0	-1000	0
Staphylococcus_aureus_M1016	0	0	0	-1000	-26.103126	-1000	0	-1000	0
Staphylococcus_aureus_M1034	0	0	0	-1000	-26.397924	-1000	0	-1000	0
Staphylococcus_aureus_M1036	0	0	0	-1000	-26.103121	-1000	0	-1000	0
Staphylococcus_aureus_M1037	0	0	0	-1000	-26.103121	-1000	0	-1000	0
Staphylococcus_aureus_M1044	0	0	0	-1000	-26.164016	-1000	0	-1000	0
Staphylococcus_aureus_M1060	0	0	0	-1000	-26.103133	-1000	0	-1000	0
Staphylococcus_aureus_M1061	0	0	0	-1000	-26.164031	-1000	0	-1000	0
Staphylococcus_aureus_M1062	0	0	0	-1000	-26.164016	-1000	0	-1000	0
Staphylococcus_aureus_M1063	0	0	0	-1000	-26.164035	-1000	0	-1000	0
Staphylococcus_aureus_M1064	0	0	0	-1000	-28.921138	-1000	0	-1000	0
Staphylococcus_aureus_M1068	0	0	0	-1000	-26.164016	-1000	0	-1000	0
Staphylococcus_aureus_M1076	0	0	0	-1000	-26.16402	-1000	0	-1000	0
Staphylococcus_aureus_M1078	0	0	0	-1000	-26.103138	-1000	0	-1000	0
Staphylococcus_aureus_M1083	0	0	0	-1000	-26.103136	-1000	0	-1000	0
Staphylococcus_aureus_M1092	0	0	0	-1000	-26.103128	-1000	0	-1000	0
Staphylococcus_aureus_M1093	0	0	0	-1000	-26.103126	-1000	0	-1000	0
Staphylococcus_aureus_M1095	0	0	0	-1000	-26.164016	-1000	0	-1000	0
Staphylococcus_aureus_M1103	0	0	0	-1000	-26.164035	-1000	0	-1000	0
Staphylococcus_aureus_M1109	0	0	0	-1000	-26.164047	-1000	0	-1000	0
Staphylococcus_aureus_M1119	0	0	0	-1000	-26.164055	-1000	0	-1000	0
Staphylococcus_aureus_M1126	0	0	0	-1000	-26.100204	-1000	0	-1000	0
Staphylococcus_aureus_M1142	0	0	0	-1000	-26.103123	-1000	0	-1000	0
Staphylococcus_aureus_M1167	0	0	0	-1000	-26.164016	-1000	0	-1000	0
Staphylococcus_aureus_M1170	0	0	0	-1000	-26.103128	-1000	0	-1000	0
Staphylococcus_aureus_M1188	0	0	0	-1000	-26.438339	-1000	0	-1000	0
Staphylococcus_aureus_M1193	0	0	0	-1000	-26.164023	-1000	0	-1000	0
Staphylococcus_aureus_M1198	0	0	0	-1000	-26.103121	-1000	0	-1000	0
Staphylococcus_aureus_M1199	0	0	0	-1000	-26.164047	-1000	0	-1000	0
Staphylococcus_aureus_M1215	0	0	0	-1000	-26.164023	-1000	0	-1000	0
Staphylococcus_aureus_M1216	0	0	0	-1000	-26.425285	-1000	0	-1000	0
Staphylococcus_aureus_M1223	0	0	0	-1000	-28.921141	-1000	0	-1000	0
Staphylococcus_aureus_M1224	0	0	0	-1000	-25.885428	-1000	0	-1000	0
Staphylococcus_aureus_M1228	0	0	0	-1000	-28.744949	-1000	0	-1000	0
Staphylococcus_aureus_M1229	0	0	0	-1000	-26.103123	-1000	0	-1000	0
Staphylococcus_aureus_M1244	0	0	0	-1000	-26.164035	-1000	0	-1000	0
Staphylococcus_aureus_M1248	0	0	0	-1000	-26.16402	-1000	0	-1000	0
Staphylococcus_aureus_M1253	0	0	0	-1000	-26.164027	-1000	0	-1000	0
Staphylococcus_aureus_M1255	0	0	0	-1000	-26.103126	-1000	0	-1000	0
Staphylococcus_aureus_M1256	0	0	0	-1000	-26.190256	-1000	0	-1000	0
Staphylococcus_aureus_M1257	0	0	0	-1000	-26.164008	-1000	0	-1000	0
Staphylococcus_aureus_M1275	0	0	0	-1000	-26.164012	-1000	0	-1000	0
Staphylococcus_aureus_M1277	0	0	0	-1000	-26.164027	-1000	0	-1000	0
Staphylococcus_aureus_M1286	0	0	0	-1000	-26.164012	-1000	0	-1000	0
Staphylococcus_aureus_M1291	0	0	0	-1000	-26.103126	-1000	0	-1000	0
Staphylococcus_aureus_M1309	0	0	0	-1000	-26.103118	-1000	0	-1000	0
Staphylococcus_aureus_M1311	0	0	0	-1000	-28.89572	-1000	0	-1000	0
Staphylococcus_aureus_M1320	0	0	0	-1000	-26.103126	-1000	0	-1000	0
Staphylococcus_aureus_M1321	0	0	0	-1000	-26.103126	-1000	0	-1000	0
Staphylococcus_aureus_M1322	0	0	0	-1000	-26.164012	-1000	0	-1000	0
Staphylococcus_aureus_M1359	0	0	0	-1000	-26.164062	-1000	0	-1000	0
Staphylococcus_aureus_M1367	0	0	0	-1000	-26.16402	-1000	0	-1000	0
Staphylococcus_aureus_M1373	0	0	0	-1000	-26.164035	-1000	0	-1000	0
Staphylococcus_aureus_M1374	0	0	0	-1000	-26.103118	-1000	0	-1000	0
Staphylococcus_aureus_M1394	0	0	0	-1000	-26.103126	-1000	0	-1000	0

Staphylococcus_aureus_M1405	0	0	0	-1000	-26.103126	-1000	0	-1000	0
Staphylococcus_aureus_M1407	0	0	0	-1000	-26.438339	-1000	0	-1000	0
Staphylococcus_aureus_M1450	0	0	0	-1000	-26.103121	-1000	0	-1000	0
Staphylococcus_aureus_M1451	0	0	0	-1000	-26.164035	-1000	0	-1000	0
Staphylococcus_aureus_M1462	0	0	0	-1000	-28.921123	-1000	0	-1000	0
Staphylococcus_aureus_M1463	0	0	0	-1000	-26.103121	-1000	0	-1000	0
Staphylococcus_aureus_M1466	0	0	0	-1000	-26.16402	-1000	0	-1000	0
Staphylococcus_aureus_M1481	0	0	0	-1000	-26.164012	-1000	0	-1000	0
Staphylococcus_aureus_M1510	0	0	0	-1000	-26.164016	-1000	0	-1000	0
Staphylococcus_aureus_M1520	0	0	0	-1000	-26.103126	-1000	0	-1000	0
Staphylococcus_aureus_M1521	0	0	0	-1000	-26.164027	-1000	0	-1000	0
Staphylococcus_aureus_M1531	0	0	0	-1000	-26.164016	-1000	0	-1000	0
Staphylococcus_aureus_M1533	0	0	0	-1000	-26.103121	-1000	0	-1000	0
Staphylococcus_aureus_M1544	0	0	0	-1000	-26.16402	-1000	0	-1000	0
Staphylococcus_aureus_M1556	0	0	0	-1000	-26.103126	-1000	0	-1000	0
Staphylococcus_aureus_M1563	0	0	0	-1000	-26.164023	-1000	0	-1000	0
Staphylococcus_aureus_M1565	0	0	0	-1000	-26.164023	-1000	0	-1000	0
Staphylococcus_aureus_M1578	0	0	0	-1000	-26.103126	-1000	0	-1000	0
Staphylococcus_aureus_M2	0	0	0	-1000	-26.133497	-1000	0	-1000	0
Staphylococcus_aureus_NN54	0	0	0	-1000	-26.164003	-1000	0	-1000	0
Staphylococcus_aureus_O11	0	0	0	-1000	-26.592747	-1000	0	-1000	0
Staphylococcus_aureus_O46	0	0	0	-1000	-26.41874	-1000	0	-1000	0
Staphylococcus_aureus_PM1	0	0	0	-1000	-28.955979	-1000	0	-1000	0
Staphylococcus_aureus_PPUKM_261_2009	0	0	0	-1000	-28.836778	-1000	0	-1000	0
Staphylococcus_aureus_PPUKM_332_2009	0	0	0	-1000	-26.275807	-1000	0	-1000	0
Staphylococcus_aureus_PPUKM_377_2009	0	0	0	-1000	-25.696224	-1000	0	-1000	0
Staphylococcus_aureus_PPUKM_775_2009	0	0	0	-1000	-29.898857	-1000	0	-1000	0
Staphylococcus_aureus_RF122	0	0	0	-1000	-28.089307	-1000	0	-1000	0
Staphylococcus_aureus_ST228_10388	0	0	0	-1000	-38.161157	-1000	0	-1000	0
Staphylococcus_aureus_ST228_10497	0	0	0	-1000	-38.161157	-1000	0	-1000	0
Staphylococcus_aureus_ST228_15532	0	0	0	-1000	-38.161157	-1000	0	-1000	0
Staphylococcus_aureus_ST228_16035	0	0	0	-1000	-38.161157	-1000	0	-1000	0
Staphylococcus_aureus_ST228_18412	0	0	0	-1000	-38.161154	-1000	0	-1000	0
Staphylococcus_aureus_subsp_aureus_06BA1836	0	0	0	-1000	-26.164003	-1000	0	-1000	0
Staphylococcus_aureus_subsp_aureus_091751	0	0	0	-1000	-26.163999	-1000	0	-1000	0
Staphylococcus_aureus_subsp_aureus_103564	0	0	0	-1000	-28.724618	-1000	0	-1000	0
Staphylococcus_aureus_subsp_aureus_112808A	0	0	0	-1000	-25.403187	-1000	0	-1000	0
Staphylococcus_aureus_subsp_aureus_118	0	0	0	-1000	-26.163999	-1000	0	-1000	0
Staphylococcus_aureus_subsp_aureus_11819_97	0	0	0	-1000	-31.588341	-1000	0	-1000	0
Staphylococcus_aureus_subsp_aureus_120	0	0	0	-1000	-26.145076	-1000	0	-1000	0
Staphylococcus_aureus_subsp_aureus_122051	0	0	0	-1000	-26.135805	-1000	0	-1000	0
Staphylococcus_aureus_subsp_aureus_132	0	0	0	-1000	-31.588335	-1000	0	-1000	0
Staphylococcus_aureus_subsp_aureus_21172	0	0	0	-1000	-26.438322	-1000	0	-1000	0
Staphylococcus_aureus_subsp_aureus_21178	0	0	0	-1000	-28.955973	-1000	0	-1000	0
Staphylococcus_aureus_subsp_aureus_21189	0	0	0	-1000	-28.955973	-1000	0	-1000	0
Staphylococcus_aureus_subsp_aureus_21193	0	0	0	-1000	-31.588335	-1000	0	-1000	0
Staphylococcus_aureus_subsp_aureus_21195	0	0	0	-1000	-28.0718	-1000	0	-1000	0
Staphylococcus_aureus_subsp_aureus_21196	0	0	0	-1000	-26.164003	-1000	0	-1000	0
Staphylococcus_aureus_subsp_aureus_21200	0	0	0	-1000	-31.542119	-1000	0	-1000	0
Staphylococcus_aureus_subsp_aureus_21201	0	0	0	-1000	-26.431812	-1000	0	-1000	0
Staphylococcus_aureus_subsp_aureus_21202	0	0	0	-1000	-28.913609	-1000	0	-1000	0
Staphylococcus_aureus_subsp_aureus_21209	0	0	0	-1000	-28.955967	-1000	0	-1000	0
Staphylococcus_aureus_subsp_aureus_21232	0	0	0	-1000	-28.955967	-1000	0	-1000	0
Staphylococcus_aureus_subsp_aureus_21236	0	0	0	-1000	-26.164003	-1000	0	-1000	0
Staphylococcus_aureus_subsp_aureus_21252	0	0	0	-1000	-28.935262	-1000	0	-1000	0
Staphylococcus_aureus_subsp_aureus_21259	0	0	0	-1000	-31.506362	-1000	0	-1000	0
Staphylococcus_aureus_subsp_aureus_21262	0	0	0	-1000	-28.901198	-1000	0	-1000	0
Staphylococcus_aureus_subsp_aureus_21264	0	0	0	-1000	-26.135809	-1000	0	-1000	0
Staphylococcus_aureus_subsp_aureus_21266	0	0	0	-1000	-28.876073	-1000	0	-1000	0
Staphylococcus_aureus_subsp_aureus_21269	0	0	0	-1000	-31.090127	-1000	0	-1000	0
Staphylococcus_aureus_subsp_aureus_21272	0	0	0	-1000	-26.20069	-1000	0	-1000	0
Staphylococcus_aureus_subsp_aureus_21282	0	0	0	-1000	-28.921114	-1000	0	-1000	0
Staphylococcus_aureus_subsp_aureus_21283	0	0	0	-1000	-28.955973	-1000	0	-1000	0
Staphylococcus_aureus_subsp_aureus_21305	0	0	0	-1000	-31.053638	-1000	0	-1000	0
Staphylococcus_aureus_subsp_aureus_21310	0	0	0	-1000	-28.955973	-1000	0	-1000	0
Staphylococcus_aureus_subsp_aureus_21318	0	0	0	-1000	-26.550109	-1000	0	-1000	0
Staphylococcus_aureus_subsp_aureus_21331	0	0	0	-1000	-28.935262	-1000	0	-1000	0
Staphylococcus_aureus_subsp_aureus_21333	0	0	0	-1000	-28.724618	-1000	0	-1000	0
Staphylococcus_aureus_subsp_aureus_21334	0	0	0	-1000	-28.699815	-1000	0	-1000	0
Staphylococcus_aureus_subsp_aureus_21340	0	0	0	-1000	-28.955973	-1000	0	-1000	0
Staphylococcus_aureus_subsp_aureus_21342	0	0	0	-1000	-26.135809	-1000	0	-1000	0
Staphylococcus_aureus_subsp_aureus_21343	0	0	0	-1000	-26.164003	-1000	0	-1000	0
Staphylococcus_aureus_subsp_aureus_21345	0	0	0	-1000	-26.135809	-1000	0	-1000	0
Staphylococcus_aureus_subsp_aureus_333	0	0	0	-1000	-26.145076	-1000	0	-1000	0
Staphylococcus_aureus_subsp_aureus_3989	0	0	0	-1000	-26.145076	-1000	0	-1000	0
Staphylococcus_aureus_subsp_aureus_55_2053	0	0	0	-1000	-26.113839	-1000	0	-1000	0
Staphylococcus_aureus_subsp_aureus_58_424	0	0	0	-1000	-26.133238	-1000	0	-1000	0
Staphylococcus_aureus_subsp_aureus_65_1322	0	0	0	-1000	-26.135841	-1000	0	-1000	0
Staphylococcus_aureus_subsp_aureus_68_397	0	0	0	-1000	-26.135849	-1000	0	-1000	0
Staphylococcus_aureus_subsp_aureus_71193	0	0	0	-1000	-28.935272	-1000	0	-1000	0
Staphylococcus_aureus_subsp_aureus_A017934_	0	0	0	-1000	-28.697795	-1000	0	-1000	0
Staphylococcus_aureus_subsp_aureus_ATCC_518	0	0	0	-1000	-28.724629	-1000	0	-1000	0
Staphylococcus_aureus_subsp_aureus_ATCC_BA	0	0	0	-1000	-28.956068	-1000	0	-1000	0
Staphylococcus_aureus_subsp_aureus_Btn1260	0	0	0	-1000	-26.095227	-1000	0	-1000	0
Staphylococcus_aureus_subsp_aureus_C101	0	0	0	-1000	-26.135833	-1000	0	-1000	0
Staphylococcus_aureus_subsp_aureus_C160	0	0	0	-1000	-26.135828	-1000	0	-1000	0
Staphylococcus_aureus_subsp_aureus_C427	0	0	0	-1000	-26.135837	-1000	0	-1000	0
Staphylococcus_aureus_subsp_aureus_CBD_635	0	0	0	-1000	-26.164003	-1000	0	-1000	0
Staphylococcus_aureus_subsp_aureus_CGS00	0	0	0	-1000	-26.129625	-1000	0	-1000	0
Staphylococcus_aureus_subsp_aureus_CGS01	0	0	0	-1000	-26.411323	-1000	0	-1000	0
Staphylococcus_aureus_subsp_aureus_CGS03	0	0	0	-500	-25.871389	-1000	0	-1000	0

Staphylococcus_aureus_subsp_aureus_CIG1057	0	0	0	-1000	-26.103121	-1000	0	-1000	0
Staphylococcus_aureus_subsp_aureus_CIG1096	0	0	0	-1000	-26.103121	-1000	0	-1000	0
Staphylococcus_aureus_subsp_aureus_CIG1114	0	0	0	-1000	-28.955979	-1000	0	-1000	0
Staphylococcus_aureus_subsp_aureus_CIG1150	0	0	0	-1000	-26.164003	-1000	0	-1000	0
Staphylococcus_aureus_subsp_aureus_CIG1165	0	0	0	-1000	-26.103116	-1000	0	-1000	0
Staphylococcus_aureus_subsp_aureus_CIG1176	0	0	0	-1000	-26.135818	-1000	0	-1000	0
Staphylococcus_aureus_subsp_aureus_CIG1213	0	0	0	-1000	-26.103118	-1000	0	-1000	0
Staphylococcus_aureus_subsp_aureus_CIG1214	0	0	0	-1000	-26.135818	-1000	0	-1000	0
Staphylococcus_aureus_subsp_aureus_CIG1233	0	0	0	-1000	-26.135818	-1000	0	-1000	0
Staphylococcus_aureus_subsp_aureus_CIG1242	0	0	0	-1000	-26.135814	-1000	0	-1000	0
Staphylococcus_aureus_subsp_aureus_CIG1267	0	0	0	-1000	-26.135818	-1000	0	-1000	0
Staphylococcus_aureus_subsp_aureus_CIG149	0	0	0	-1000	-26.135814	-1000	0	-1000	0
Staphylococcus_aureus_subsp_aureus_CIG1500	0	0	0	-1000	-26.135818	-1000	0	-1000	0
Staphylococcus_aureus_subsp_aureus_CIG1524	0	0	0	-1000	-28.935267	-1000	0	-1000	0
Staphylococcus_aureus_subsp_aureus_CIG1605	0	0	0	-1000	-26.135818	-1000	0	-1000	0
Staphylococcus_aureus_subsp_aureus_CIG1612	0	0	0	-1000	-28.955979	-1000	0	-1000	0
Staphylococcus_aureus_subsp_aureus_CIG1750	0	0	0	-1000	-25.90672	-1000	0	-1000	0
Staphylococcus_aureus_subsp_aureus_CIG1769	0	0	0	-1000	-26.103118	-1000	0	-1000	0
Staphylococcus_aureus_subsp_aureus_CIG1770	0	0	0	-1000	-28.955979	-1000	0	-1000	0
Staphylococcus_aureus_subsp_aureus_CIG1835	0	0	0	-1000	-28.935262	-1000	0	-1000	0
Staphylococcus_aureus_subsp_aureus_CIG2018	0	0	0	-1000	-28.955985	-1000	0	-1000	0
Staphylococcus_aureus_subsp_aureus_CIG290	0	0	0	-1000	-28.901208	-1000	0	-1000	0
Staphylococcus_aureus_subsp_aureus_CIG547	0	0	0	-1000	-28.803265	-1000	0	-1000	0
Staphylococcus_aureus_subsp_aureus_CIGC128	0	0	0	-1000	-28.955979	-1000	0	-1000	0
Staphylococcus_aureus_subsp_aureus_CIGC340D	0	0	0	-1000	-26.164008	-1000	0	-1000	0
Staphylococcus_aureus_subsp_aureus_CIGC341D	0	0	0	-1000	-26.135814	-1000	0	-1000	0
Staphylococcus_aureus_subsp_aureus_CIGC345D	0	0	0	-1000	-26.323612	-1000	0	-1000	0
Staphylococcus_aureus_subsp_aureus_CIGC348	0	0	0	-1000	-26.103118	-1000	0	-1000	0
Staphylococcus_aureus_subsp_aureus_CIGC93	0	0	0	-1000	-28.955979	-1000	0	-1000	0
Staphylococcus_aureus_subsp_aureus_CM05	0	0	0	-1000	-26.164003	-1000	0	-1000	0
Staphylococcus_aureus_subsp_aureus_COL	0	0	0	-1000	-28.955979	-1000	0	-1000	0
Staphylococcus_aureus_subsp_aureus_D139	0	0	0	-1000	-26.355808	-1000	0	-1000	0
Staphylococcus_aureus_subsp_aureus_DR10	0	0	0	-1000	-28.935262	-1000	0	-1000	0
Staphylococcus_aureus_subsp_aureus_DSM_202	0	0	0	-1000	-28.921117	-1000	0	-1000	0
Staphylococcus_aureus_subsp_aureus_E1410	0	0	0	-1000	-26.106942	-1000	0	-1000	0
Staphylococcus_aureus_subsp_aureus_ECT_R_2	0	0	0	-1000	-26.135814	-1000	0	-1000	0
Staphylococcus_aureus_subsp_aureus_ED133	0	0	0	-1000	-28.70813	-1000	0	-1000	0
Staphylococcus_aureus_subsp_aureus_ED98	0	0	0	-1000	-26.103118	-1000	0	-1000	0
Staphylococcus_aureus_subsp_aureus_EMRSA16	0	0	0	-1000	-26.135833	-1000	0	-1000	0
Staphylococcus_aureus_subsp_aureus_GR1	0	0	0	-1000	-28.146068	-1000	0	-1000	0
Staphylococcus_aureus_subsp_aureus_H19	0	0	0	-1000	-25.092285	-1000	0	-1000	0
Staphylococcus_aureus_subsp_aureus_H29	0	0	0	-1000	-26.103118	-1000	0	-1000	0
Staphylococcus_aureus_subsp_aureus_HO_5096_	0	0	0	-1000	-28.913615	-1000	0	-1000	0
Staphylococcus_aureus_subsp_aureus_IS_105	0	0	0	-1000	-26.411328	-1000	0	-1000	0
Staphylococcus_aureus_subsp_aureus_IS_111	0	0	0	-1000	-28.955973	-1000	0	-1000	0
Staphylococcus_aureus_subsp_aureus_IS_122	0	0	0	-1000	-26.103116	-1000	0	-1000	0
Staphylococcus_aureus_subsp_aureus_IS_189	0	0	0	-1000	-28.959954	-1000	0	-1000	0
Staphylococcus_aureus_subsp_aureus_IS_250	0	0	0	-1000	-25.306305	0	0	-1000	0
Staphylococcus_aureus_subsp_aureus_IS_3	0	0	0	-1000	-26.103118	-1000	0	-1000	0
Staphylococcus_aureus_subsp_aureus_IS_55	0	0	0	-1000	-26.155826	-1000	0	-1000	0
Staphylococcus_aureus_subsp_aureus_IS_88	0	0	0	-1000	-28.592199	-1000	0	-1000	0
Staphylococcus_aureus_subsp_aureus_IS_99	0	0	0	-1000	-26.103116	-1000	0	-1000	0
Staphylococcus_aureus_subsp_aureus_IS_K	0	0	0	-1000	-26.620446	0	0	-1000	0
Staphylococcus_aureus_subsp_aureus_JH1	0	0	0	-1000	-26.164012	-1000	0	-1000	0
Staphylococcus_aureus_subsp_aureus_JH9	0	0	0	-1000	-26.164012	-1000	0	-1000	0
Staphylococcus_aureus_subsp_aureus_JKD6159	0	0	0	-1000	-28.955979	-1000	0	-1000	0
Staphylococcus_aureus_subsp_aureus_LCT_SA11	0	0	0	-1000	-26.135849	-1000	0	-1000	0
Staphylococcus_aureus_subsp_aureus_LGA251	0	0	0	-1000	-31.542139	-1000	0	-1000	0
Staphylococcus_aureus_subsp_aureus_M013	0	0	0	-1000	-28.724626	-1000	0	-1000	0
Staphylococcus_aureus_subsp_aureus_M1015	0	0	0	-1000	-26.135833	-1000	0	-1000	0
Staphylococcus_aureus_subsp_aureus_M809	0	0	0	-1000	-26.132368	-1000	0	-1000	0
Staphylococcus_aureus_subsp_aureus_M876	0	0	0	-1000	-26.135837	-1000	0	-1000	0
Staphylococcus_aureus_subsp_aureus_M899	0	0	0	-1000	-28.699832	-1000	0	-1000	0
Staphylococcus_aureus_subsp_aureus_MN8	0	0	0	-1000	-25.623079	-1000	0	-1000	0
Staphylococcus_aureus_subsp_aureus_MRGR3	0	0	0	-1000	-28.92111	-1000	0	-1000	0
Staphylococcus_aureus_subsp_aureus_MRSA131	0	0	0	-1000	-28.955967	-1000	0	-1000	0
Staphylococcus_aureus_subsp_aureus_MRSA177	0	0	0	-1000	-28.955967	-1000	0	-1000	0
Staphylococcus_aureus_subsp_aureus_MRSA252	0	0	0	-1000	-26.135814	-1000	0	-1000	0
Staphylococcus_aureus_subsp_aureus_MSHR113	0	0	0	-1000	-29.036551	-1000	0	-1000	0
Staphylococcus_aureus_subsp_aureus_MSSA476	0	0	0	-1000	-28.935262	-1000	0	-1000	0
Staphylococcus_aureus_subsp_aureus_Mu3	0	0	0	-1000	-23.733119	-1000	0	-1000	0
Staphylococcus_aureus_subsp_aureus_Mu50	0	0	0	-1000	-23.733114	-1000	0	-1000	0
Staphylococcus_aureus_subsp_aureus_Mu50_om	0	0	0	-1000	-23.733119	-1000	0	-1000	0
Staphylococcus_aureus_subsp_aureus_MW2	0	0	0	-1000	-28.935262	-1000	0	-1000	0
Staphylococcus_aureus_subsp_aureus_N315	0	0	0	-1000	-26.164008	-1000	0	-1000	0
Staphylococcus_aureus_subsp_aureus_NBRC_100	0	0	0	-1000	-40.505553	-1000	0	-1000	0
Staphylococcus_aureus_subsp_aureus_NCTC_832	0	0	0	-1000	-45.610685	-1000	0	-1000	0
Staphylococcus_aureus_subsp_aureus_NN50	0	0	0	-1000	-28.955973	-1000	0	-1000	0
Staphylococcus_aureus_subsp_aureus_PB32	0	0	0	-1000	-26.20069	-1000	0	-1000	0
Staphylococcus_aureus_subsp_aureus_RN4220	0	0	0	-1000	-28.954232	-1000	0	-1000	0
Staphylococcus_aureus_subsp_aureus_str_JKD60	0	0	0	-1000	-28.955985	-1000	0	-1000	0
Staphylococcus_aureus_subsp_aureus_str_JKD60	0	0	0	-1000	-28.955973	-1000	0	-1000	0
Staphylococcus_aureus_subsp_aureus_str_Newm	0	0	0	-1000	-28.955985	-1000	0	-1000	0
Staphylococcus_aureus_subsp_aureus_T0131	0	0	0	-1000	-28.955979	-1000	0	-1000	0
Staphylococcus_aureus_subsp_aureus_TCH130	0	0	0	-1000	-28.91365	-1000	0	-1000	0
Staphylococcus_aureus_subsp_aureus_TCH60	0	0	0	-1000	-26.135814	-1000	0	-1000	0
Staphylococcus_aureus_subsp_aureus_TCH70	0	0	0	-1000	-28.935262	-1000	0	-1000	0
Staphylococcus_aureus_subsp_aureus_TW20	0	0	0	-1000	-28.955979	-1000	0	-1000	0
Staphylococcus_aureus_subsp_aureus_USA300	0	0	0	-1000	-28.955979	-1000	0	-1000	0
Staphylococcus_aureus_subsp_aureus_USA300_F	0	0	0	-1000	-29.733206	-1000	0	-1000	0
Staphylococcus_aureus_subsp_aureus_USA300_T	0	0	0	-1000	-28.955973	-1000	0	-1000	0

Staphylococcus_aureus_subsp_aureus_USA300_T	0	0	0	-1000	-44.002919	-1000	0	-1000	0
Staphylococcus_aureus_subsp_aureus_VC40	0	0	0	-1000	-31.586441	-1000	0	-1000	0
Staphylococcus_aureus_subsp_aureus_VCU006	0	0	0	-1000	-28.955973	-1000	0	-1000	0
Staphylococcus_aureus_subsp_aureus_VH60	0	0	0	-1000	-26.145076	-1000	0	-1000	0
Staphylococcus_aureus_subsp_aureus_VRS1	0	0	0	-1000	-26.103116	-1000	0	-1000	0
Staphylococcus_aureus_subsp_aureus_VRS10	0	0	0	-1000	-26.103118	-1000	0	-1000	0
Staphylococcus_aureus_subsp_aureus_VRS11a	0	0	0	-1000	-26.16169	-1000	0	-1000	0
Staphylococcus_aureus_subsp_aureus_VRS11b	0	0	0	-1000	-26.16169	-1000	0	-1000	0
Staphylococcus_aureus_subsp_aureus_VRS2	0	0	0	-1000	-26.164008	-1000	0	-1000	0
Staphylococcus_aureus_subsp_aureus_VRS3a	0	0	0	-1000	-26.164003	-1000	0	-1000	0
Staphylococcus_aureus_subsp_aureus_VRS4	0	0	0	-1000	-26.164008	-1000	0	-1000	0
Staphylococcus_aureus_subsp_aureus_VRS5	0	0	0	-1000	-26.103118	-1000	0	-1000	0
Staphylococcus_aureus_subsp_aureus_VRS6	0	0	0	-1000	-26.103118	-1000	0	-1000	0
Staphylococcus_aureus_subsp_aureus_VRS7	0	0	0	-1000	-26.103118	-1000	0	-1000	0
Staphylococcus_aureus_subsp_aureus_VRS8	0	0	0	-1000	-26.103118	-1000	0	-1000	0
Staphylococcus_aureus_subsp_aureus_VRS9	0	0	0	-1000	-26.164003	-1000	0	-1000	0
Staphylococcus_aureus_subsp_aureus_WBG1004	0	0	0	-1000	-26.135833	-1000	0	-1000	0
Staphylococcus_aureus_subsp_aureus_WW2703_	0	0	0	-1000	-28.63654	-1000	0	-1000	0
Staphylococcus_aureus_VH221	0	0	0	-1000	-28.146068	-1000	0	-1000	0
Staphylococcus_capitis_CR0101	0	0	0	-1000	-20.460014	-1000	0	-1000	0
Staphylococcus_capitis_QN1	0	0	0	-1000	-25.31047	-1000	0	-1000	0
Staphylococcus_capitis_SK14	0	0	0	-1000	-26.747016	-1000	0	-1000	0
Staphylococcus_capitis_VCU116	0	0	0	-1000	-25.855271	-1000	0	-1000	0
Staphylococcus_caprae_C87	0	0	0	-1000	-27.709934	-1000	0	-1000	0
Staphylococcus_cohnii_hu_01	0	0	0	-1000	-31.968229	0	0	0	0
Staphylococcus_cohnii_subsp_cohnii_532	0	0	0	-1000	-36.825629	-1000	0	0	0
Staphylococcus_epidermidis_12142587	0	0	0	-1000	-26.537751	0	0	-1000	0
Staphylococcus_epidermidis_14_1_R1_SE	0	0	0	-1000	-25.326783	0	0	-1000	0
Staphylococcus_epidermidis_41tr	0	0	0	-1000	-26.239043	0	0	-1000	0
Staphylococcus_epidermidis_528m	0	0	0	-1000	-25.59934	0	0	-1000	0
Staphylococcus_epidermidis_A487	0	0	-1000	-1000	-31.187896	-1000	0	-1000	0
Staphylococcus_epidermidis_ATCC_12228	0	0	0	-1000	-28.858917	0	0	-1000	0
Staphylococcus_epidermidis_AU12_03	0	0	0	-1000	-28.510242	0	0	-1000	0
Staphylococcus_epidermidis_BCM_HMP0060	0	0	0	-1000	-27.769197	0	0	-1000	0
Staphylococcus_epidermidis_BVS058A4	0	0	0	-1000	-27.746788	0	0	-1000	0
Staphylococcus_epidermidis_ERR2221338	0	0	0	-1000	-47.582346	0	0	-1000	0
Staphylococcus_epidermidis_FRI909	0	0	0	-1000	-25.631559	0	0	-1000	0
Staphylococcus_epidermidis_M0881	0	0	0	-1000	-26.535543	0	0	-1000	0
Staphylococcus_epidermidis_M23864W2grey	0	0	0	-1000	-31.21829	0	0	-1000	0
Staphylococcus_epidermidis_NIH04003	0	0	0	-1000	-25.18411	0	0	-1000	0
Staphylococcus_epidermidis_NIH04008	0	0	0	-1000	-30.816415	0	0	-1000	0
Staphylococcus_epidermidis_NIH05001	0	0	0	-1000	-26.88066	0	0	-1000	0
Staphylococcus_epidermidis_NIH05003	0	0	0	-1000	-28.381924	0	0	-1000	0
Staphylococcus_epidermidis_NIH05005	0	0	0	-1000	-28.38192	0	0	-1000	0
Staphylococcus_epidermidis_NIH051475	0	0	0	-1000	-24.477287	0	0	-887.32394	0
Staphylococcus_epidermidis_NIH051668	0	0	0	-1000	-28.381911	0	0	-1000	0
Staphylococcus_epidermidis_NIH06004	0	0	0	-1000	-28.384049	0	0	-1000	0
Staphylococcus_epidermidis_NIH08001	0	0	0	-1000	-27.578664	0	0	-1000	0
Staphylococcus_epidermidis_NIHLM001	0	0	0	-1000	-27.390073	0	0	-1000	0
Staphylococcus_epidermidis_NIHLM003	0	0	0	-1000	-27.099888	0	0	-1000	0
Staphylococcus_epidermidis_NIHLM008	0	0	0	-1000	-29.517772	0	0	-1000	0
Staphylococcus_epidermidis_NIHLM015	0	0	0	-1000	-28.077835	0	0	-1000	0
Staphylococcus_epidermidis_NIHLM018	0	0	0	-1000	-26.561352	0	0	-1000	0
Staphylococcus_epidermidis_NIHLM020	0	0	0	-1000	-27.431286	0	0	-1000	0
Staphylococcus_epidermidis_NIHLM021	0	0	0	-1000	-25.039367	0	0	-1000	0
Staphylococcus_epidermidis_NIHLM023	0	0	0	-1000	-26.618127	0	0	-1000	0
Staphylococcus_epidermidis_NIHLM031	0	0	0	-1000	-28.836872	0	0	-1000	0
Staphylococcus_epidermidis_NIHLM037	0	0	0	-1000	-28.806334	0	0	-1000	0
Staphylococcus_epidermidis_NIHLM039	0	0	0	-1000	-27.63371	0	0	-1000	0
Staphylococcus_epidermidis_NIHLM040	0	0	0	-1000	-28.848115	0	0	-1000	0
Staphylococcus_epidermidis_NIHLM049	0	0	0	-1000	-27.455151	0	0	-1000	0
Staphylococcus_epidermidis_NIHLM053	0	0	0	-1000	-28.07783	0	0	-1000	0
Staphylococcus_epidermidis_NIHLM057	0	0	0	-1000	-28.078866	0	0	-1000	0
Staphylococcus_epidermidis_NIHLM061	0	0	0	-1000	-45.516241	0	0	-1000	0
Staphylococcus_epidermidis_NIHLM067	0	0	0	-1000	-25.680409	0	0	-1000	0
Staphylococcus_epidermidis_NIHLM070	0	0	0	-1000	-47.360302	0	0	-1000	0
Staphylococcus_epidermidis_NIHLM087	0	0	0	-1000	-26.922168	0	0	-1000	0
Staphylococcus_epidermidis_NIHLM088	0	0	0	-1000	-27.584269	0	0	-1000	0
Staphylococcus_epidermidis_NIHLM095	0	0	0	-1000	-28.27201	0	0	-1000	0
Staphylococcus_epidermidis_RP62A	0	0	0	-1000	-24.465626	0	0	-1000	0
Staphylococcus_epidermidis_SK135	0	0	0	-1000	-27.769144	0	0	-1000	0
Staphylococcus_epidermidis_UC7032	0	0	0	-1000	-26.683121	0	0	-1000	0
Staphylococcus_epidermidis_VCU028	0	0	0	-1000	-28.236545	0	0	-1000	0
Staphylococcus_epidermidis_VCU037	0	0	0	-1000	-26.535511	0	0	-1000	0
Staphylococcus_epidermidis_VCU041	0	0	0	-1000	-28.598369	0	0	-1000	0
Staphylococcus_epidermidis_VCU045	0	0	0	-1000	-31.244441	0	0	-1000	0
Staphylococcus_epidermidis_VCU065	0	0	0	-1000	-25.710025	0	0	-1000	0
Staphylococcus_epidermidis_VCU071	0	0	0	-1000	-26.346218	0	0	-1000	0
Staphylococcus_epidermidis_VCU081	0	0	0	-1000	-25.71086	0	0	-1000	0
Staphylococcus_epidermidis_VCU105	0	0	0	-1000	-26.163848	0	0	-1000	0
Staphylococcus_epidermidis_VCU109	0	0	0	-1000	-28.443232	0	0	-1000	0
Staphylococcus_epidermidis_VCU117	0	0	0	-1000	-26.424677	0	0	-1000	0
Staphylococcus_epidermidis_VCU118	0	0	0	-1000	-27.720927	0	0	-1000	0
Staphylococcus_epidermidis_VCU120	0	0	0	-1000	-26.534557	0	0	-1000	0
Staphylococcus_epidermidis_VCU123	0	0	0	-1000	-26.343766	0	0	-1000	0
Staphylococcus_epidermidis_VCU125	0	0	0	-1000	-28.445173	0	0	-1000	0
Staphylococcus_epidermidis_VCU126	0	0	0	-1000	-26.423729	0	0	-1000	0
Staphylococcus_epidermidis_VCU127	0	0	0	-1000	-28.38192	0	0	-1000	0
Staphylococcus_epidermidis_VCU128	0	0	0	-1000	-25.631564	0	0	-1000	0
Staphylococcus_epidermidis_VCU129	0	0	0	-1000	-28.599305	0	0	-1000	0
Staphylococcus_epidermidis_VCU144	0	0	0	-1000	-28.423999	0	0	-1000	0

Staphylococcus_epidermidis_W23144	0	0	0	-1000	-29.975386	0	0	-1000	0
Staphylococcus_equorum_subsp_equorum_Mu2	0	0	-1000	-1000	-36.047772	-1000	0	-1000	0
Staphylococcus_haemolyticus_JCSC1435	0	0	0	-1000	-27.383795	-1000	0	-1000	0
Staphylococcus_haemolyticus_R1P1	0	0	-1000	-1000	-31.693164	-1000	0	-1000	0
Staphylococcus_hominis_SK119	0	0	0	-1000	-24.061306	0	0	-1000	0
Staphylococcus_hominis_subsp_hominis_C80	0	0	0	-1000	-24.228809	0	0	-1000	0
Staphylococcus_hominis_subsp_hominis_ZBW5	0	0	0	-1000	-24.061306	0	0	-1000	0
Staphylococcus_hominis_VCU122	0	0	0	-1000	-23.118595	0	0	-1000	0
Staphylococcus_intermedius_NCTC_11048	0	0	0	-1000	-26.188925	-1000	0	-1000	0
Staphylococcus_lentus_ERR2221130	0	0	0	-1000	-32.078189	-1000	0	-1000	0
Staphylococcus_lugdunensis_ACS_027_V_Sch2	0	0	0	-1000	-25.925124	0	0	-1000	0
Staphylococcus_lugdunensis_HKU09_01	0	0	0	-1000	-26.594392	0	0	-1000	0
Staphylococcus_lugdunensis_M23590	0	0	0	-1000	-25.920661	0	0	-1000	0
Staphylococcus_lugdunensis_N920143	0	0	0	-1000	-25.925125	0	0	-1000	0
Staphylococcus_lugdunensis_VCU139	0	0	0	-1000	-25.038628	0	0	-1000	0
Staphylococcus_nepalensis_ERR2221195	0	0	0	-1000	-37.520761	-1000	0	-1000	0
Staphylococcus_pasteuri_BAB3	0	0	0	-1000	-38.304453	-1000	0	-1000	0
Staphylococcus_pasteuri_SP1	0	0	0	-1000	-21.629833	-1000	0	-1000	0
Staphylococcus_pettenkoferi_VCU012	0	0	0	-1000	-19.140057	-1000	0	-800	0
Staphylococcus_pseudintermedius_ED99	0	0	0	-1000	-25.899629	-1000	0	-1000	0
Staphylococcus_pseudintermedius_HKU10_03	0	0	0	-1000	-25.859672	-1000	0	-1000	0
Staphylococcus_saprophyticus_subsp_saprophyti	0	0	0	-1000	-28.36016	-1000	0	-1000	0
Staphylococcus_saprophyticus_subsp_saprophyti	0	0	0	-1000	-26.198253	-1000	0	-1000	0
Staphylococcus_sciuri_subsp_sciuri_DSM_20345	0	0	-1000	-1000	-32.592969	-1000	0	-1000	0
Staphylococcus_sciuri_subsp_sciuri_Z8	0	0	0	-1000	-16.657212	-1000	0	-1000	0
Staphylococcus_simulans_ACS_120_V_Sch1	0	0	-1000	-1000	-33.42823	-1000	0	-1000	0
Staphylococcus_succinus_SNUC_1280	0	0	0	-1000	-15.006798	-1000	0	-1000	0
Staphylococcus_succinus_subsp_casei_DSM_150	0	0	0	-1000	-17.483591	-1000	0	-1000	0
Staphylococcus_succinus_subsp_succinus_DSM_	0	0	0	-1000	-17.483591	-1000	0	-1000	0
Staphylococcus_vitulinus_F1028	0	0	0	-1000	-23.19231	-1000	0	-1000	0
Staphylococcus_warneri_L37603	0	0	-1000	-1000	-31.445403	-1000	0	-1000	0
Staphylococcus_warneri_SG1	0	0	-1000	-1000	-34.709099	-1000	0	-1000	0
Staphylococcus_xylosus_DMB3_Bh1	0	0	0	-1000	-22.021012	-1000	0	-1000	0
Staphylococcus_xylosus_ERR2221122	0	0	0	-1000	-53.679824	-1000	0	-1000	0
Staphylococcus_xylosus_ERR2221131	0	0	0	-1000	-53.679824	-1000	0	-1000	0
Staphylococcus_xylosus_NJ	0	0	-1000	-1000	-34.581819	-1000	0	-1000	0
Stenotrophomonas_maltophilia_Ab55555	0	0	0	-1000	0	0	-1000	-1000	0
Stenotrophomonas_maltophilia_ATCC_19867	0	0	0	-1000	0	0	-1000	-1000	0
Stenotrophomonas_maltophilia_AU12_09	0	0	0	-1000	-1000	0	-1000	-1000	0
Stenotrophomonas_maltophilia_D457	0	0	0	-1000	0	0	-1000	-1000	0
Stenotrophomonas_maltophilia_EPM1	0	0	0	-1000	0	0	-1000	-1000	0
Stenotrophomonas_maltophilia_ISMMS3	0	0	0	-1000	-1000	0	-1000	-877.30627	0
Stenotrophomonas_maltophilia_K279a	-501	0	0	-1000	0	0	-1000	-1000	0
Stenotrophomonas_maltophilia_PML168	0	0	0	-1000	0	0	-1000	-1000	0
Stenotrophomonas_maltophilia_R551_3	-500.5	0	0	-1000	0	0	-1000	-1000	0
Stenotrophomonas_maltophilia_RR_10	0	0	0	-1000	0	0	-1000	-901.26624	0
Stenotrophomonas_rhizophila_PCA13	0	0	0	-1000	0	0	0	0	0
Stomatobaculum_longum_ACC2	0	0	0	-21.24221006	-1000	0	0	-285.21767	0
Stoquefichus_massiliensis_AP9	0	0	0	-1000	-14.867064	0	0	0	0
Streptococcus_agalactiae_09mas018883	0	0	-1000	0	-30.231188	0	0	-1000	0
Streptococcus_agalactiae_2603V_R	0	0	-1000	0	-25.19894	0	0	-1000	0
Streptococcus_agalactiae_A909	0	0	-1000	0	-27.804906	0	0	-1000	0
Streptococcus_agalactiae_BSU108	0	0	-1000	0	-30.208373	0	0	-1000	0
Streptococcus_agalactiae_BSU133	0	0	-1000	0	-28.918629	0	0	-1000	0
Streptococcus_agalactiae_BSU165	0	0	-1000	0	-30.173113	0	0	-1000	0
Streptococcus_agalactiae_BSU167	0	0	-1000	0	-30.22659	0	0	-1000	0
Streptococcus_agalactiae_BSU174	0	0	-1000	0	-24.801109	0	0	-1000	0
Streptococcus_agalactiae_BSU178	0	0	-1000	0	-24.367271	0	0	-1000	0
Streptococcus_agalactiae_BSU247	0	0	-1000	0	-30.227477	0	0	-1000	0
Streptococcus_agalactiae_BSU248	0	0	-1000	0	-24.345509	0	0	-1000	0
Streptococcus_agalactiae_BSU252	0	0	-1000	0	-30.231219	0	0	-1000	0
Streptococcus_agalactiae_BSU253	0	0	-1000	0	-30.208389	0	0	-1000	0
Streptococcus_agalactiae_BSU260	0	0	-1000	0	-30.208381	0	0	-1000	0
Streptococcus_agalactiae_BSU442	0	0	-1000	0	-24.797349	0	0	-1000	0
Streptococcus_agalactiae_BSU447	0	0	-1000	0	-30.17801	0	0	-1000	0
Streptococcus_agalactiae_BSU450	0	0	-1000	0	-24.742945	0	0	-1000	0
Streptococcus_agalactiae_BSU451	0	0	-1000	0	-30.227453	0	0	-1000	0
Streptococcus_agalactiae_BSU454	0	0	-1000	0	-24.03777	0	0	-1000	0
Streptococcus_agalactiae_BSU92	0	0	-1000	0	-30.193549	0	0	-1000	0
Streptococcus_agalactiae_BSU96	0	0	-1000	0	-24.788423	0	0	-1000	0
Streptococcus_agalactiae_CCUG_17336	0	0	-1000	0	-24.802945	0	0	-1000	0
Streptococcus_agalactiae_CCUG_19094	0	0	-1000	0	-30.186612	0	0	-1000	0
Streptococcus_agalactiae_CCUG_24810	0	0	-1000	0	-30.186604	0	0	-1000	0
Streptococcus_agalactiae_CCUG_25532	0	0	-1000	0	-30.227469	0	0	-1000	0
Streptococcus_agalactiae_CCUG_28551	0	0	-1000	0	-30.193549	0	0	-1000	0
Streptococcus_agalactiae_CCUG_29376	0	0	-1000	0	-24.03777	0	0	-1000	0
Streptococcus_agalactiae_CCUG_29782	0	0	-1000	0	-30.174504	0	0	-1000	0
Streptococcus_agalactiae_CCUG_30636	0	0	-1000	0	-30.231204	0	0	-1000	0
Streptococcus_agalactiae_CCUG_37430	0	0	-1000	0	-24.100912	0	0	-1000	0
Streptococcus_agalactiae_CCUG_37736	0	0	-1000	0	-30.170183	0	0	-1000	0
Streptococcus_agalactiae_CCUG_37737	0	0	-1000	0	-30.186612	0	0	-1000	0
Streptococcus_agalactiae_CCUG_37738	0	0	-1000	0	-30.176314	0	0	-1000	0
Streptococcus_agalactiae_CCUG_37739	0	0	-1000	0	-30.208389	0	0	-1000	0
Streptococcus_agalactiae_CCUG_37740	0	0	-1000	0	-30.212127	0	0	-1000	0
Streptococcus_agalactiae_CCUG_37741	0	0	-1000	0	-30.168036	0	0	-1000	0
Streptococcus_agalactiae_CCUG_37742	0	0	0	0	-25.143334	0	0	-1000	0
Streptococcus_agalactiae_CCUG_38383	0	0	-1000	0	-28.772198	0	0	-1000	0
Streptococcus_agalactiae_CCUG_39096_A	0	0	-1000	0	-24.037761	0	0	-1000	0
Streptococcus_agalactiae_CCUG_44050	0	0	-1000	0	-30.333557	0	0	-1000	0
Streptococcus_agalactiae_CCUG_44074	0	0	-1000	0	-30.208389	0	0	-1000	0
Streptococcus_agalactiae_CCUG_44077	0	0	-1000	0	-30.087242	0	0	-1000	0

Streptococcus_agalactiae_CCUG_44110	0	0	-1000	0	-30.227777	0	0	-1000	0
Streptococcus_agalactiae_CCUG_44140	0	0	-1000	0	-30.231211	0	0	-1000	0
Streptococcus_agalactiae_CCUG_44186	0	0	-1000	0	-24.788425	0	0	-1000	0
Streptococcus_agalactiae_CCUG_45061	0	0	-1000	0	-30.186612	0	0	-1000	0
Streptococcus_agalactiae_CCUG_47293	0	0	-1000	0	-24.037761	0	0	-1000	0
Streptococcus_agalactiae_CCUG_49072	0	0	-1000	0	-30.231204	0	0	-1000	0
Streptococcus_agalactiae_CCUG_49086	0	0	-1000	0	-24.788424	0	0	-1000	0
Streptococcus_agalactiae_CCUG_49087	0	0	-1000	0	-24.788423	0	0	-1000	0
Streptococcus_agalactiae_CCUG_49100	0	0	-1000	0	-30.212111	0	0	-1000	0
Streptococcus_agalactiae_CCUG_91	0	0	-1000	0	-24.402746	0	0	-1000	0
Streptococcus_agalactiae_CJB111	0	0	-1000	0	-29.00855	0	0	-1000	0
Streptococcus_agalactiae_ERR2221173	0	0	0	0	-33.431487	0	0	-958.33333	0
Streptococcus_agalactiae_ERR2221321	0	0	0	0	-33.431487	0	0	-958.33333	0
Streptococcus_agalactiae_FSL_C1_487	0	0	-1000	0	-30.227453	0	0	-1000	0
Streptococcus_agalactiae_FSL_C1_494	0	0	-1000	0	-30.227453	0	0	-1000	0
Streptococcus_agalactiae_FSL_F2_338	0	0	-1000	0	-30.22743	0	0	-1000	0
Streptococcus_agalactiae_FSL_F2_343	0	0	-1000	0	-30.174496	0	0	-1000	0
Streptococcus_agalactiae_FSL_S3_001	0	0	-1000	0	-30.231204	0	0	-1000	0
Streptococcus_agalactiae_FSL_S3_003	0	0	-1000	0	-30.168036	0	0	-1000	0
Streptococcus_agalactiae_FSL_S3_005	0	0	-1000	0	-24.782837	0	0	-1000	0
Streptococcus_agalactiae_FSL_S3_014	0	0	-1000	0	-24.057687	0	0	-1000	0
Streptococcus_agalactiae_FSL_S3_023	0	0	0	0	-25.29508	0	0	-1000	0
Streptococcus_agalactiae_FSL_S3_026	0	0	-1000	0	-30.227446	0	0	-1000	0
Streptococcus_agalactiae_FSL_S3_034	0	0	-1000	0	-30.227438	0	0	-1000	0
Streptococcus_agalactiae_FSL_S3_062	0	0	-1000	0	-30.246786	0	0	-1000	0
Streptococcus_agalactiae_FSL_S3_090	0	0	-1000	0	-24.797355	0	0	-1000	0
Streptococcus_agalactiae_FSL_S3_102	0	0	-1000	0	-24.788423	0	0	-1000	0
Streptococcus_agalactiae_FSL_S3_105	0	0	-1000	0	-30.227438	0	0	-1000	0
Streptococcus_agalactiae_FSL_S3_128	0	0	0	0	-25.294879	0	0	-1000	0
Streptococcus_agalactiae_FSL_S3_137	0	0	-1000	0	-24.037761	0	0	-1000	0
Streptococcus_agalactiae_FSL_S3_170	0	0	-1000	0	-30.301783	0	0	-1000	0
Streptococcus_agalactiae_FSL_S3_222	0	0	0	0	-25.294879	0	0	-1000	0
Streptococcus_agalactiae_FSL_S3_268	0	0	-1000	0	-24.782837	0	0	-1000	0
Streptococcus_agalactiae_FSL_S3_277	0	0	-1000	0	-30.172523	0	0	-1000	0
Streptococcus_agalactiae_FSL_S3_337	0	0	-1000	0	-30.186619	0	0	-1000	0
Streptococcus_agalactiae_FSL_S3_586	0	0	-1000	0	-30.195332	0	0	-1000	0
Streptococcus_agalactiae_FSL_S3_603	0	0	-1000	0	-29.046229	0	0	-1000	0
Streptococcus_agalactiae_FSL_S3_608	0	0	-1000	0	-28.694498	0	0	-1000	0
Streptococcus_agalactiae_FSL_S3_654	0	0	-1000	0	-30.068782	0	0	-1000	0
Streptococcus_agalactiae_GB00002	0	0	-1000	0	-30.231219	0	0	-1000	0
Streptococcus_agalactiae_GB00003	0	0	-1000	0	-24.057677	0	0	-1000	0
Streptococcus_agalactiae_GB00012	0	0	-1000	0	-30.231204	0	0	-1000	0
Streptococcus_agalactiae_GB00013	0	0	-1000	0	-30.231204	0	0	-1000	0
Streptococcus_agalactiae_GB00018	0	0	-1000	0	-30.227477	0	0	-1000	0
Streptococcus_agalactiae_GB00020	0	0	-1000	0	-30.231204	0	0	-1000	0
Streptococcus_agalactiae_GB00082	0	0	-1000	0	-30.193564	0	0	-1000	0
Streptococcus_agalactiae_GB00083	0	0	-1000	0	-30.12341	0	0	-1000	0
Streptococcus_agalactiae_GB00084	0	0	-1000	0	-30.231204	0	0	-1000	0
Streptococcus_agalactiae_GB00092	0	0	-1000	0	-30.191687	0	0	-1000	0
Streptococcus_agalactiae_GB00097	0	0	-1000	0	-30.189223	0	0	-1000	0
Streptococcus_agalactiae_GB00111	0	0	-1000	0	-24.802945	0	0	-1000	0
Streptococcus_agalactiae_GB00112	0	0	-1000	0	-24.788422	0	0	-1000	0
Streptococcus_agalactiae_GB00115	0	0	-1000	0	-24.802947	0	0	-1000	0
Streptococcus_agalactiae_GB00174	0	0	-1000	0	-24.797349	0	0	-1000	0
Streptococcus_agalactiae_GB00190	0	0	-1000	0	-30.227477	0	0	-1000	0
Streptococcus_agalactiae_GB00202	0	0	-1000	0	-24.057677	0	0	-1000	0
Streptococcus_agalactiae_GB00206	0	0	-1000	0	-24.110627	0	0	-1000	0
Streptococcus_agalactiae_GB00219	0	0	-1000	0	-24.057677	0	0	-1000	0
Streptococcus_agalactiae_GB00226	0	0	-1000	0	-30.191695	0	0	-1000	0
Streptococcus_agalactiae_GB00241	0	0	-1000	0	-30.231204	0	0	-1000	0
Streptococcus_agalactiae_GB00245	0	0	-1000	0	-30.227477	0	0	-1000	0
Streptococcus_agalactiae_GB00247	0	0	-1000	0	-30.22561	0	0	-1000	0
Streptococcus_agalactiae_GB00264	0	0	-1000	0	-24.791793	0	0	-1000	0
Streptococcus_agalactiae_GB00279	0	0	-1000	0	-29.456039	0	0	-1000	0
Streptococcus_agalactiae_GB00300	0	0	-1000	0	-24.79179	0	0	-1000	0
Streptococcus_agalactiae_GB00535	0	0	-1000	0	-24.057682	0	0	-1000	0
Streptococcus_agalactiae_GB00543	0	0	-1000	0	-30.186612	0	0	-1000	0
Streptococcus_agalactiae_GB00548	0	0	-1000	0	-30.193549	0	0	-1000	0
Streptococcus_agalactiae_GB00555	0	0	-1000	0	-24.057682	0	0	-1000	0
Streptococcus_agalactiae_GB00557	0	0	-1000	0	-24.802947	0	0	-1000	0
Streptococcus_agalactiae_GB00561	0	0	-1000	0	-24.778768	0	0	-1000	0
Streptococcus_agalactiae_GB00588	0	0	-1000	0	-24.416356	0	0	-1000	0
Streptococcus_agalactiae_GB00601	0	0	-1000	0	-30.214427	0	0	-1000	0
Streptococcus_agalactiae_GB00614	0	0	-1000	0	-30.231211	0	0	-1000	0
Streptococcus_agalactiae_GB00640	0	0	-1000	0	-30.225602	0	0	-1000	0
Streptococcus_agalactiae_GB00651	0	0	-1000	0	-24.057677	0	0	-1000	0
Streptococcus_agalactiae_GB00653	0	0	-1000	0	-24.791791	0	0	-1000	0
Streptococcus_agalactiae_GB00654	0	0	-1000	0	-24.802947	0	0	-1000	0
Streptococcus_agalactiae_GB00663	0	0	-1000	0	-30.186612	0	0	-1000	0
Streptococcus_agalactiae_GB00679	0	0	-1000	0	-29.456061	0	0	-1000	0
Streptococcus_agalactiae_GB00864	0	0	-1000	0	-30.180712	0	0	-1000	0
Streptococcus_agalactiae_GB00865	0	0	-1000	0	-30.163614	0	0	-1000	0
Streptococcus_agalactiae_GB00867	0	0	-1000	0	-24.797356	0	0	-1000	0
Streptococcus_agalactiae_GB00874	0	0	-1000	0	-30.231196	0	0	-1000	0
Streptococcus_agalactiae_GB00884	0	0	-1000	0	-30.186619	0	0	-1000	0
Streptococcus_agalactiae_GB00887	0	0	-1000	0	-30.227477	0	0	-1000	0
Streptococcus_agalactiae_GB00888	0	0	-1000	0	-24.801108	0	0	-1000	0
Streptococcus_agalactiae_GB00891	0	0	-1000	0	-30.189215	0	0	-1000	0
Streptococcus_agalactiae_GB00893	0	0	-1000	0	-24.742952	0	0	-1000	0
Streptococcus_agalactiae_GB00900	0	0	-1000	0	-30.178002	0	0	-1000	0
Streptococcus_agalactiae_GB00901	0	0	-1000	0	-29.418701	0	0	-1000	0

Streptococcus_agalactiae_GB00904	0	0	-1000	0	-30.178002	0	0	-1000	0
Streptococcus_agalactiae_GB00909	0	0	-1000	0	-24.367319	0	0	-1000	0
Streptococcus_agalactiae_GB00911	0	0	0	0	-25.308976	0	0	-1000	0
Streptococcus_agalactiae_GB00914	0	0	-1000	0	-28.918644	0	0	-1000	0
Streptococcus_agalactiae_GB00919	0	0	-1000	0	-24.057677	0	0	-1000	0
Streptococcus_agalactiae_GB00922	0	0	-1000	0	-30.193549	0	0	-1000	0
Streptococcus_agalactiae_GB00923	0	0	-1000	0	-30.17801	0	0	-1000	0
Streptococcus_agalactiae_GB00924	0	0	-1000	0	-30.231204	0	0	-1000	0
Streptococcus_agalactiae_GB00929	0	0	-1000	0	-30.178002	0	0	-1000	0
Streptococcus_agalactiae_GB00932	0	0	-1000	0	-30.227477	0	0	-1000	0
Streptococcus_agalactiae_GB00933	0	0	0	0	-25.308976	0	0	-1000	0
Streptococcus_agalactiae_GB00954	0	0	-1000	0	-24.797353	0	0	-1000	0
Streptococcus_agalactiae_GB00955	0	0	-1000	0	-30.231211	0	0	-1000	0
Streptococcus_agalactiae_GB00963	0	0	-1000	0	-24.802949	0	0	-1000	0
Streptococcus_agalactiae_GB00965	0	0	-1000	0	-30.181881	0	0	-1000	0
Streptococcus_agalactiae_GB00975	0	0	-1000	0	-24.797353	0	0	-1000	0
Streptococcus_agalactiae_GB00984	0	0	-1000	0	-30.178018	0	0	-1000	0
Streptococcus_agalactiae_GB00986	0	0	0	0	-19.399374	0	0	-1000	0
Streptococcus_agalactiae_GB00999	0	0	-1000	0	-30.231196	0	0	-1000	0
Streptococcus_agalactiae_GD201008_001	0	0	-1000	0	-24.057662	0	0	-1000	0
Streptococcus_agalactiae_ILRI005	0	0	-1000	0	-30.231196	0	0	-1000	0
Streptococcus_agalactiae_ILRI112	0	0	-1000	0	-28.756739	0	0	-1000	0
Streptococcus_agalactiae_LADL_05_108a	0	0	-1000	0	-25.107097	0	0	-1000	0
Streptococcus_agalactiae_LADL_90_503	0	0	-1000	0	-25.234139	0	0	-1000	0
Streptococcus_agalactiae_LMG_14609	0	0	-1000	0	-25.091693	0	0	-1000	0
Streptococcus_agalactiae_LMG_14838	0	0	-1000	0	-30.100846	0	0	-1000	0
Streptococcus_agalactiae_LMG_15081	0	0	-1000	0	-29.431776	0	0	-1000	0
Streptococcus_agalactiae_LMG_15083	0	0	-1000	0	-24.037766	0	0	-1000	0
Streptococcus_agalactiae_LMG_15084	0	0	-1000	0	-24.100916	0	0	-1000	0
Streptococcus_agalactiae_LMG_15085	0	0	-1000	0	-24.788422	0	0	-1000	0
Streptococcus_agalactiae_LMG_15089	0	0	-1000	0	-30.027071	0	0	-1000	0
Streptococcus_agalactiae_LMG_15090	0	0	-1000	0	-24.037761	0	0	-1000	0
Streptococcus_agalactiae_LMG_15091	0	0	0	0	-33.565893	0	0	-1000	0
Streptococcus_agalactiae_LMG_15092	0	0	-1000	0	-29.332576	0	0	-1000	0
Streptococcus_agalactiae_LMG_15094	0	0	-1000	0	-24.788422	0	0	-1000	0
Streptococcus_agalactiae_LMG_15095	0	0	-1000	0	-24.788423	0	0	-1000	0
Streptococcus_agalactiae_MRI_Z1_012	0	0	-1000	0	-28.916574	0	0	-1000	0
Streptococcus_agalactiae_MRI_Z1_022	0	0	-1000	0	-28.87564	0	0	-1000	0
Streptococcus_agalactiae_MRI_Z1_023	0	0	0	0	-25.281133	0	0	-1000	0
Streptococcus_agalactiae_MRI_Z1_025	0	0	-1000	0	-30.231196	0	0	-1000	0
Streptococcus_agalactiae_MRI_Z1_035	0	0	-1000	0	-29.911293	0	0	-1000	0
Streptococcus_agalactiae_MRI_Z1_038	0	0	-1000	0	-24.907996	0	0	-1000	0
Streptococcus_agalactiae_MRI_Z1_198	0	0	-1000	0	-24.345509	0	0	-1000	0
Streptococcus_agalactiae_MRI_Z1_199	0	0	-1000	0	-30.208373	0	0	-1000	0
Streptococcus_agalactiae_MRI_Z1_200	0	0	-1000	0	-30.227461	0	0	-1000	0
Streptococcus_agalactiae_MRI_Z1_201	0	0	-1000	0	-30.227461	0	0	-1000	0
Streptococcus_agalactiae_MRI_Z1_202	0	0	-1000	0	-30.227453	0	0	-1000	0
Streptococcus_agalactiae_MRI_Z1_203	0	0	-1000	0	-30.227461	0	0	-1000	0
Streptococcus_agalactiae_MRI_Z1_204	0	0	-1000	0	-30.214427	0	0	-1000	0
Streptococcus_agalactiae_MRI_Z1_205	0	0	-1000	0	-30.231204	0	0	-1000	0
Streptococcus_agalactiae_MRI_Z1_206	0	0	-1000	0	-24.057677	0	0	-1000	0
Streptococcus_agalactiae_MRI_Z1_211	0	0	-1000	0	-30.231196	0	0	-1000	0
Streptococcus_agalactiae_MRI_Z1_212	0	0	-1000	0	-30.231196	0	0	-1000	0
Streptococcus_agalactiae_MRI_Z1_213	0	0	-1000	0	-30.18546	0	0	-1000	0
Streptococcus_agalactiae_MRI_Z1_214	0	0	-1000	0	-30.423894	0	0	-1000	0
Streptococcus_agalactiae_MRI_Z1_215	0	0	-1000	0	-30.212111	0	0	-1000	0
Streptococcus_agalactiae_MRI_Z1_216	0	0	-1000	0	-30.166425	0	0	-1000	0
Streptococcus_agalactiae_MRI_Z1_217	0	0	-1000	0	-30.166448	0	0	-1000	0
Streptococcus_agalactiae_MRI_Z1_218	0	0	-1000	0	-28.918644	0	0	-1000	0
Streptococcus_agalactiae_MRI_Z1_219	0	0	-1000	0	-28.900337	0	0	-1000	0
Streptococcus_agalactiae_NEM316	0	0	-1000	0	-30.193525	0	0	-1000	0
Streptococcus_agalactiae_PR06	0	0	-1000	0	-30.221421	0	0	-1000	0
Streptococcus_agalactiae_SA20_06	0	0	0	0	-37.866319	0	0	-1000	0
Streptococcus_agalactiae_SS1014	0	0	-1000	0	-29.019524	0	0	-1000	0
Streptococcus_agalactiae_SS1218	0	0	0	0	-24.453846	0	0	-774.8611	0
Streptococcus_agalactiae_SS1219	0	0	-1000	0	-25.228428	0	0	-1000	0
Streptococcus_agalactiae_STIR_CD_01	0	0	-1000	0	-24.057667	0	0	-1000	0
Streptococcus_agalactiae_STIR_CD_09	0	0	-1000	0	-25.234138	0	0	-1000	0
Streptococcus_agalactiae_STIR_CD_14	0	0	-1000	0	-30.193556	0	0	-1000	0
Streptococcus_agalactiae_STIR_CD_17	0	0	-1000	0	-24.731194	0	0	-1000	0
Streptococcus_agalactiae_STIR_CD_21	0	0	-1000	0	-24.057662	0	0	-1000	0
Streptococcus_agalactiae_STIR_CD_22	0	0	-1000	0	-24.057662	0	0	-1000	0
Streptococcus_agalactiae_STIR_CD_23	0	0	-1000	0	-24.037766	0	0	-1000	0
Streptococcus_agalactiae_STIR_CD_24	0	0	-1000	0	-24.057677	0	0	-1000	0
Streptococcus_agalactiae_STIR_CD_26	0	0	-1000	0	-24.037766	0	0	-1000	0
Streptococcus_agalactiae_STIR_CD_27	0	0	-1000	0	-24.037766	0	0	-1000	0
Streptococcus_agalactiae_STIR_CD_28	0	0	-1000	0	-24.037766	0	0	-1000	0
Streptococcus_agalactiae_STIR_CD_29	0	0	0	0	-24.344791	0	0	-714.74805	0
Streptococcus_agalactiae_str_Gottschalk_1002A	0	0	-1000	0	-30.208381	0	0	-1000	0
Streptococcus_agalactiae_str_Gottschalk_1003A	0	0	-1000	0	-30.186612	0	0	-1000	0
Streptococcus_agalactiae_str_Gottschalk_13227	0	0	-1000	0	-30.212111	0	0	-1000	0
Streptococcus_agalactiae_str_Gottschalk_2864	0	0	-1000	0	-30.231204	0	0	-1000	0
Streptococcus_agalactiae_str_Gottschalk_31825	0	0	-1000	0	-30.227469	0	0	-1000	0
Streptococcus_agalactiae_str_Gottschalk_992B	0	0	-1000	0	-28.786007	0	0	-1000	0
Streptococcus_agalactiae_str_Gottschalk_998A	0	0	-1000	0	-24.042905	0	0	-1000	0
Streptococcus_agalactiae_str_Gottschalk_999B	0	0	-1000	0	-24.037766	0	0	-1000	0
Streptococcus_agalactiae_ZQ0910	0	0	-1000	0	-24.057631	0	0	-1000	0
Streptococcus_anginosus_1_2_62CV	0	0	0	0	-19.045462	0	0	-592.10526	0
Streptococcus_anginosus_CCUG_39159	0	0	0	0	-16.115556	0	0	-944.44444	0
Streptococcus_anginosus_ERR2221320	0	0	0	0	-29.048322	0	0	-513.51351	0
Streptococcus_anginosus_F0211	0	0	0	0	-15.514699	0	0	-944.44444	0

Streptococcus_anginosus_SK1138	0	0	0	0	-16.131628	0	0	-944.44444	0
Streptococcus_anginosus_SK52	0	0	0	0	-15.421197	0	0	-710.80139	0
Streptococcus_australis_ATCC_700641	0	0	0	0	-20.895767	0	0	-1000	0
Streptococcus_australis_ERR2221360	0	0	0	0	-23.599908	0	0	-564.66877	0
Streptococcus_bovis_ATCC_700338	0	0	-1000	0	-29.025635	-1000	0	-674.24469	0
Streptococcus_constellatus_subsp_constellatus_S	0	0	0	0	-15.896884	0	0	-972.22222	0
Streptococcus_constellatus_subsp_pharyngis_SK	0	0	0	0	-17.905159	0	0	-871.79487	0
Streptococcus_cristatus_ATCC_51100	0	0	0	0	-21.026343	0	0	-533.20766	0
Streptococcus_cristatus_LRIIBV4	0	0	0	0	-27.738166	0	0	0	0
Streptococcus_danieliae_NM51_B2_22	0	0	0	0	-15.703475	-1000	0	0	0
Streptococcus_downei_F0415	0	0	0	0	-22.567964	-1000	0	-791.66667	0
Streptococcus_downei_MFe28	0	0	0	0	-22.568424	-1000	0	-791.66667	0
Streptococcus_dysgalactiae_subsp_dysgalactiae_	0	0	-1000	0	-30.637566	0	0	-1000	0
Streptococcus_dysgalactiae_subsp_equisimilis_A	0	0	-1000	0	-28.947136	-1000	0	-1000	0
Streptococcus_dysgalactiae_subsp_equisimilis_A	0	0	-1000	0	-28.782459	-1000	0	-1000	0
Streptococcus_dysgalactiae_subsp_equisimilis_G	0	0	-1000	0	-29.291018	-1000	0	-1000	0
Streptococcus_dysgalactiae_subsp_equisimilis_R	0	0	-1000	0	-28.624868	-1000	0	-882.35294	0
Streptococcus_dysgalactiae_subsp_equisimilis_S	0	0	-1000	0	-29.217268	-1000	0	-1000	0
Streptococcus_equi_subsp_equi_4047	0	0	-1000	0	-31.196259	0	0	-717.42809	0
Streptococcus_equi_subsp_zooepidemicus_ATCC	0	0	-1000	0	-28.010753	0	0	-480.28937	0
Streptococcus_equi_subsp_zooepidemicus_BHS5	0	0	-1000	0	-27.665449	0	0	-278.58054	0
Streptococcus_equi_subsp_zooepidemicus_MGC	0	0	-1000	0	-23.2397	0	0	-490.74074	0
Streptococcus_equinus_ATCC_9812	0	0	0	0	-22.002109	0	0	-507.31234	0
Streptococcus_gallolyticus_subsp_gallolyticus_AT	0	0	0	-434.7826087	-22.778036	-1000	0	-1000	0
Streptococcus_gallolyticus_subsp_gallolyticus_AT	0	0	0	-182.0573054	-21.342525	-1000	0	-873.32556	0
Streptococcus_gallolyticus_subsp_gallolyticus_TX	0	0	0	-181.9461009	-21.1681	-1000	0	-542.80987	0
Streptococcus_gallolyticus_UCN34	0	0	0	-182.0831409	-21.342525	-1000	0	-873.44655	0
Streptococcus_gordonii_str_Challis_substr_CH1	0	0	0	0	-19.487369	0	0	-1000	0
Streptococcus_infantarius_subsp_infantarius_AT	0	0	0	-181.8181818	-18.973833	0	0	-573.52941	0
Streptococcus_infantarius_subsp_infantarius_CJ1	0	0	0	-217.2362187	-21.364711	0	0	-561.93856	0
Streptococcus_infantis_ATCC_700779	0	0	0	-25.51213943	-20.913489	0	0	-983.14628	0
Streptococcus_infantis_SK1076	0	0	0	0	-15.378049	0	0	-764.49556	0
Streptococcus_infantis_SK970	0	0	0	0	-16.019327	0	0	-763.25494	0
Streptococcus_infantis_X	0	0	0	0	-16.047869	0	0	-1000	0
Streptococcus_intermedius_ATCC_27335	0	0	0	0	-15.428375	0	0	-1000	0
Streptococcus_intermedius_BA1	0	0	0	0	-15.428375	0	0	-1000	0
Streptococcus_intermedius_F0395	0	0	0	0	-16.103114	0	0	-1000	0
Streptococcus_intermedius_F0413	0	0	0	0	-16.011346	0	0	-1000	0
Streptococcus_intermedius_JTH08	0	0	0	0	-18.264519	0	0	-1000	0
Streptococcus_lutetiensis_033	0	0	0	0	-19.276607	0	0	-883.21805	0
Streptococcus_lutetiensis_ERR2230054	0	0	0	0	-21.04921	0	0	-909.09091	0
Streptococcus_macedonicus_679	0	0	0	-1000	-21.973636	0	0	-1000	0
Streptococcus_macedonicus_ACA_DC_198	0	0	0	-1000	-23.700656	-1000	0	-1000	0
Streptococcus_massiliensis_4401825	0	0	0	0	-15.354913	0	0	0	0
Streptococcus_massiliensis_DSM_18628	0	0	0	0	-15.354913	0	0	0	0
Streptococcus_mitis_11_5	0	0	0	0	-15.457718	0	0	-1000	0
Streptococcus_mitis_13_39	0	0	0	0	-16.019218	0	0	-1000	0
Streptococcus_mitis_ATCC_6249	0	0	0	0	-16.132943	0	0	-1000	0
Streptococcus_mitis_B6	0	0	0	0	-16.098389	0	0	-1000	0
Streptococcus_mitis_bv_2_str_F0392	0	0	0	0	-18.1057	0	0	-1000	0
Streptococcus_mitis_bv_2_str_SK95	0	0	0	0	-15.31851	0	0	-1000	0
Streptococcus_mitis_NCTC_12261	0	0	0	0	-18.696819	0	0	-1000	0
Streptococcus_mitis_SK1073	0	0	0	0	-15.925972	0	0	-1000	0
Streptococcus_mitis_SK1080	0	0	0	0	-15.940455	0	0	-1000	0
Streptococcus_mitis_SK321	0	0	0	0	-15.795663	0	0	-1000	0
Streptococcus_mitis_SK564	0	0	0	0	-15.799827	0	0	-1000	0
Streptococcus_mitis_SK569	0	0	0	0	-15.349919	0	0	-1000	0
Streptococcus_mitis_SK575	0	0	0	0	-15.796002	0	0	-1000	0
Streptococcus_mitis_SK579	0	0	0	0	-15.343751	0	0	-1000	0
Streptococcus_mitis_SK616	0	0	0	0	-15.348412	0	0	-1000	0
Streptococcus_mitis_SPAR10	0	0	0	0	-15.401634	0	0	-764.42963	0
Streptococcus_mutans_11A1	0	0	0	0	-18.594965	-1000	0	-1000	-1000
Streptococcus_mutans_11SSST2	0	0	0	0	-19.198083	-1000	0	-1000	-1000
Streptococcus_mutans_14D	0	0	0	0	-18.563929	-1000	0	-1000	-1000
Streptococcus_mutans_15JP3	0	0	0	0	-19.415571	-1000	0	-1000	-1000
Streptococcus_mutans_15VF2	0	0	0	0	-17.53707	-1000	0	-1000	-1000
Streptococcus_mutans_1SM1	0	0	0	0	-19.378934	-1000	0	-1000	-1000
Streptococcus_mutans_21	0	0	0	0	-18.595021	-1000	0	-1000	-1000
Streptococcus_mutans_24	0	0	0	0	-19.378964	-1000	0	-1000	-1000
Streptococcus_mutans_2ST1	0	0	0	0	-17.477672	-1000	0	-1000	-1000
Streptococcus_mutans_2VS1	0	0	0	0	-18.56417	-1000	0	-1000	-1000
Streptococcus_mutans_3SN1	0	0	0	0	-18.60551	-1000	0	-1000	-1000
Streptococcus_mutans_4SM1	0	0	0	0	-19.378964	-1000	0	-1000	-1000
Streptococcus_mutans_4VF1	0	0	0	0	-19.378934	-1000	0	-1000	-1000
Streptococcus_mutans_5DC8	0	0	0	0	-18.911292	-1000	0	-1000	-1000
Streptococcus_mutans_5SM3	0	0	0	0	-19.378994	-1000	0	-1000	-1000
Streptococcus_mutans_66_2A	0	0	0	0	-19.379025	-1000	0	-1000	-1000
Streptococcus_mutans_8ID3	0	0	0	0	-19.018636	-1000	0	-1000	-1000
Streptococcus_mutans_A19	0	0	0	0	-19.048362	-1000	0	-1000	-1000
Streptococcus_mutans_A9	0	0	0	0	-17.333086	-1000	0	-1000	-1000
Streptococcus_mutans_AC4446	0	0	0	0	-17.452266	-1000	0	-1000	-1000
Streptococcus_mutans_ATCC_25175	0	0	0	0	-28.381346	-1000	0	-1000	-1000
Streptococcus_mutans_B	0	0	0	0	-19.379408	-1000	0	-1000	-1000
Streptococcus_mutans_DSM_20523	0	0	0	0	-19.415519	-1000	0	-1000	-1000
Streptococcus_mutans_ERR2221174	0	0	0	0	-23.595531	-1000	0	-1000	-938.45535
Streptococcus_mutans_ERR2221288	0	0	0	0	-23.595535	-1000	0	-1000	-938.45535
Streptococcus_mutans_G123	0	0	0	0	-18.56419	-1000	0	-1000	-1000
Streptococcus_mutans_GS_5	0	0	0	0	-19.817281	-1000	0	-1000	-1000
Streptococcus_mutans_KK21	0	0	0	0	-19.093985	-1000	0	-1000	-1000
Streptococcus_mutans_KK23	0	0	0	0	-18.712572	-1000	0	-1000	-1000
Streptococcus_mutans_M21	0	0	0	0	-19.378994	-1000	0	-1000	-1000

Streptococcus_mutans_M230	0	0	0	0	-17.517411	-1000	0	-1000	-1000
Streptococcus_mutans_M2A	0	0	0	0	-19.020247	-1000	0	-1000	-1000
Streptococcus_mutans_N29	0	0	0	0	-18.564121	-1000	0	-1000	-1000
Streptococcus_mutans_N3209	0	0	0	0	-18.595021	-1000	0	-1000	-1000
Streptococcus_mutans_N34	0	0	0	0	-19.379438	-1000	0	-1000	-1000
Streptococcus_mutans_N66	0	0	0	0	-18.987761	-1000	0	-1000	-1000
Streptococcus_mutans_NCTC_11060	0	0	0	0	-18.765454	-1000	0	-1000	-1000
Streptococcus_mutans_NFSM1	0	0	0	0	-18.961945	-1000	0	-1000	-1000
Streptococcus_mutans_NFSM2	0	0	0	0	-19.563592	-1000	0	-1000	-1000
Streptococcus_mutans_NLML1	0	0	0	0	-19.378934	-1000	0	-1000	-1000
Streptococcus_mutans_NLML4	0	0	0	0	-18.56419	-1000	0	-1000	-1000
Streptococcus_mutans_NLML5	0	0	0	0	-19.41599	-1000	0	-1000	-1000
Streptococcus_mutans_NLML8	0	0	0	0	-22.084908	-1000	0	-1000	-1000
Streptococcus_mutans_NLML9	0	0	0	0	-17.440784	-1000	0	-1000	-1000
Streptococcus_mutans_NMT4863	0	0	0	0	-18.56386	-1000	0	-1000	-1000
Streptococcus_mutans_NN2025	0	0	0	0	-19.554902	-1000	0	-1000	-1000
Streptococcus_mutans_NV1996	0	0	0	0	-21.532092	-1000	0	-1000	-1000
Streptococcus_mutans_NVAB	0	0	0	0	-19.415545	-1000	0	-1000	-1000
Streptococcus_mutans_OMZ175	0	0	0	0	-19.677305	-1000	0	-1000	-1000
Streptococcus_mutans_R221	0	0	0	0	-17.477685	-1000	0	-1000	-1000
Streptococcus_mutans_S1B	0	0	0	0	-19.563611	-1000	0	-1000	-1000
Streptococcus_mutans_SA38	0	0	0	0	-18.695585	-1000	0	-1000	-1000
Streptococcus_mutans_SA41	0	0	0	0	-19.415545	-1000	0	-1000	-1000
Streptococcus_mutans_SF1	0	0	0	0	-19.378994	-1000	0	-1000	-1000
Streptococcus_mutans_SF12	0	0	0	0	-19.379025	-1000	0	-1000	-1000
Streptococcus_mutans_SF14	0	0	0	0	-17.64314	-1000	0	-1000	-1000
Streptococcus_mutans_SM1	0	0	0	0	-19.378964	-1000	0	-1000	-1000
Streptococcus_mutans_SM4	0	0	0	0	-19.671448	-1000	0	-1000	-1000
Streptococcus_mutans_SM6	0	0	0	0	-19.378994	-1000	0	-1000	-1000
Streptococcus_mutans_ST1	0	0	0	0	-19.415519	-1000	0	-1000	-1000
Streptococcus_mutans_ST6	0	0	0	0	-19.379025	-1000	0	-1000	-1000
Streptococcus_mutans_T4	0	0	0	0	-19.415519	-1000	0	-1000	-1000
Streptococcus_mutans_TCI_116	0	0	0	0	-18.846	-1000	0	-1000	-1000
Streptococcus_mutans_TCI_123	0	0	0	0	-19.093985	-1000	0	-1000	-1000
Streptococcus_mutans_TCI_125	0	0	0	0	-19.123312	-1000	0	-1000	-1000
Streptococcus_mutans_TCI_138	0	0	0	0	-17.452267	-1000	0	-1000	-1000
Streptococcus_mutans_TCI_145	0	0	0	0	-19.512554	-1000	0	-1000	-1000
Streptococcus_mutans_TCI_152	0	0	0	0	-17.625689	-1000	0	-1000	-1000
Streptococcus_mutans_TCI_153	0	0	0	0	-18.911287	-1000	0	-1000	-1000
Streptococcus_mutans_TCI_163	0	0	0	0	-19.52389	-1000	0	-1000	-1000
Streptococcus_mutans_TCI_169	0	0	0	0	-18.911287	-1000	0	-1000	-1000
Streptococcus_mutans_TCI_173	0	0	0	0	-17.863802	-1000	0	-1000	-1000
Streptococcus_mutans_TCI_177	0	0	0	0	-19.093985	-1000	0	-1000	-1000
Streptococcus_mutans_TCI_191	0	0	0	0	-19.168534	-1000	0	-1000	-1000
Streptococcus_mutans_TCI_196	0	0	0	0	-19.047062	-1000	0	-1000	-1000
Streptococcus_mutans_TCI_219	0	0	0	0	-19.048363	-1000	0	-1000	-1000
Streptococcus_mutans_TCI_222	0	0	0	0	-19.123312	-1000	0	-1000	-1000
Streptococcus_mutans_TCI_228	0	0	0	0	-17.625689	-1000	0	-1000	-1000
Streptococcus_mutans_TCI_234	0	0	0	0	-19.422356	-1000	0	-1000	-1000
Streptococcus_mutans_TCI_239	0	0	0	0	-19.048362	-1000	0	-1000	-1000
Streptococcus_mutans_TCI_242	0	0	0	0	-18.910015	-1000	0	-1000	-1000
Streptococcus_mutans_TCI_243	0	0	0	0	-18.855853	-1000	0	-1000	-1000
Streptococcus_mutans_TCI_249	0	0	0	0	-19.001285	-1000	0	-1000	-1000
Streptococcus_mutans_TCI_256	0	0	0	0	-19.048362	-1000	0	-1000	-1000
Streptococcus_mutans_TCI_268	0	0	0	0	-17.765124	-1000	0	-1000	-1000
Streptococcus_mutans_TCI_278	0	0	0	0	-17.679469	-1000	0	-1000	-1000
Streptococcus_mutans_TCI_294	0	0	0	0	-19.512554	-1000	0	-1000	-1000
Streptococcus_mutans_TCI_30	0	0	0	0	-19.093985	-1000	0	-1000	-1000
Streptococcus_mutans_TCI_399	0	0	0	0	-19.093982	-1000	0	-1000	-1000
Streptococcus_mutans_TCI_400	0	0	0	0	-17.625566	-1000	0	-1000	-1000
Streptococcus_mutans_TCI_51	0	0	0	0	-19.063682	-1000	0	-1000	-1000
Streptococcus_mutans_TCI_70	0	0	0	0	-19.093985	-1000	0	-1000	-1000
Streptococcus_mutans_TCI_75	0	0	0	0	-19.422356	-1000	0	-1000	-1000
Streptococcus_mutans_TCI_78	0	0	0	0	-19.422356	-1000	0	-1000	-1000
Streptococcus_mutans_TCI_82	0	0	0	0	-19.048362	-1000	0	-1000	-1000
Streptococcus_mutans_TCI_85	0	0	0	0	-18.911289	-1000	0	-1000	-1000
Streptococcus_mutans_TCI_86	0	0	0	0	-19.123317	-1000	0	-1000	-1000
Streptococcus_mutans_TCI_92	0	0	0	0	-19.093982	-1000	0	-1000	-1000
Streptococcus_mutans_TCI_96	0	0	0	0	-19.447505	-1000	0	-1000	-1000
Streptococcus_mutans_U138	0	0	0	0	-19.093982	-1000	0	-1000	-1000
Streptococcus_mutans_U2A	0	0	0	0	-19.677305	-1000	0	-1000	-1000
Streptococcus_mutans_U2B	0	0	0	0	-18.987397	-1000	0	-1000	-1000
Streptococcus_mutans_UA159	0	0	0	0	-17.745649	-1000	0	-1000	-1000
Streptococcus_mutans_W6	0	0	0	0	-18.563819	-1000	0	-1000	-1000
Streptococcus_nov_ERR2221358	0	0	0	0	-23.598684	0	0	0	0
Streptococcus_oralis_ATCC_35037	0	0	0	0	-16.0338	0	0	-1000	0
Streptococcus_oralis_ERR2221353	0	0	0	0	-23.598683	0	0	-564.66877	0
Streptococcus_oralis_SK10	0	0	0	0	-15.375798	0	0	-1000	0
Streptococcus_oralis_SK100	0	0	0	0	-16.045063	0	0	-1000	0
Streptococcus_oralis_SK1074	0	0	0	0	-15.569111	0	0	-764.45334	0
Streptococcus_oralis_SK255	0	0	0	0	-15.627386	0	0	-764.67796	0
Streptococcus_oralis_SK304	0	0	0	0	-15.569084	0	0	-764.4544	0
Streptococcus_oralis_SK313	0	0	0	0	-19.25582	0	0	-442.01031	0
Streptococcus_oralis_SK610	0	0	0	0	-15.40326	0	0	-1000	0
Streptococcus_oralis_subsp_tigurinus_1366	0	0	0	0	-20.320325	0	0	-441.04803	0
Streptococcus_oralis_subsp_tigurinus_2425	0	0	0	0	-20.163863	0	0	-519.23077	0
Streptococcus_oralis_subsp_tigurinus_UC5873_T	0	0	0	0	-19.868209	0	0	-442.01031	0
Streptococcus_oralis_Uo5	0	0	0	0	-20.876106	0	0	-1000	0
Streptococcus_parasanguinis_ATCC_15912	0	0	0	0	-16.608253	0	0	-833.33333	0
Streptococcus_parasanguinis_ATCC_903	0	0	0	0	-20.709417	0	0	-1000	0
Streptococcus_parasanguinis_ERR2221175	0	0	0	0	-26.027252	0	0	-626.50602	0

Streptococcus_parasanguinis_ERR2221190	0	0	0	0	-25.270638	0	0	-616.7979	0
Streptococcus_parasanguinis_F0405	0	0	0	0	-16.578053	0	0	-833.33333	0
Streptococcus_parasanguinis_FW213	0	0	0	0	-17.286462	0	0	-833.33333	0
Streptococcus_parasanguinis_SK236	0	0	0	0	-16.612772	0	0	-833.33333	0
Streptococcus_parauberis_KCTC_11537	0	0	0	0	-22.564634	-1000	0	-1000	0
Streptococcus_parauberis_KCTC_11980BP	0	0	0	0	-21.176159	-1000	0	-1000	0
Streptococcus_parauberis_KRS_02083	0	0	0	0	-22.004736	-1000	0	-1000	0
Streptococcus_parauberis_KRS_02109	0	0	0	0	-21.227924	-1000	0	-1000	0
Streptococcus_parauberis_NCFD_2020	0	0	0	0	-21.226821	-1000	0	-1000	0
Streptococcus_pasteurianus_651_SPAS_0_477_1	0	0	0	0	-20.899539	0	0	-734.93976	0
Streptococcus_pasteurianus_ATCC_43144	0	0	-1000	0	-26.370779	0	0	-833.33333	0
Streptococcus_peroris_ATCC_700780	0	0	0	-25.66570794	-20.517942	0	0	-1000	0
Streptococcus_pleomorphus_DSM_20574	0	0	0	-1000	-17.045746	-1000	0	0	0
Streptococcus_pneumoniae_1974M1_LZD	0	0	0	0	-19.350628	0	0	-1000	0
Streptococcus_pneumoniae_2009	0	0	0	0	-19.123651	-1000	0	-1000	0
Streptococcus_pneumoniae_2061376	0	0	0	0	-19.42968	-1000	0	-1000	0
Streptococcus_pneumoniae_2061617	0	0	0	0	-19.304926	0	0	-1000	0
Streptococcus_pneumoniae_2070005	0	0	0	0	-19.343384	-1000	0	-1000	0
Streptococcus_pneumoniae_2070035	0	0	0	0	-19.304926	-1000	0	-1000	0
Streptococcus_pneumoniae_2070109	0	0	0	0	-17.715474	0	0	-1000	0
Streptococcus_pneumoniae_2070425	0	0	0	0	-19.299103	0	0	-1000	0
Streptococcus_pneumoniae_2070531	0	0	0	0	-19.299103	0	0	-1000	0
Streptococcus_pneumoniae_2071004	0	0	0	0	-19.332663	-1000	0	-1000	0
Streptococcus_pneumoniae_2071247	0	0	0	0	-19.372603	0	0	-1000	0
Streptococcus_pneumoniae_2072047	0	0	0	0	-19.328988	0	0	-1000	0
Streptococcus_pneumoniae_2080076	0	0	0	0	-19.303445	0	0	-1000	0
Streptococcus_pneumoniae_2080913	0	0	0	0	-19.378365	-1000	0	-1000	0
Streptococcus_pneumoniae_2081074	0	0	0	0	-19.305161	-1000	0	-1000	0
Streptococcus_pneumoniae_2081685	0	0	0	0	-19.308346	0	0	-1000	0
Streptococcus_pneumoniae_2082170	0	0	0	0	-19.431249	-1000	0	-1000	0
Streptococcus_pneumoniae_2090008	0	0	0	0	-19.314568	-1000	0	-1000	0
Streptococcus_pneumoniae_23F	0	0	0	0	-30.588509	0	0	-1000	0
Streptococcus_pneumoniae_3063_00	0	0	0	0	-19.345114	0	0	-1000	0
Streptococcus_pneumoniae_357	0	0	0	0	-19.278753	-1000	0	-1000	0
Streptococcus_pneumoniae_4027_06	0	0	0	0	-19.346605	0	0	-1000	0
Streptococcus_pneumoniae_4075_00	0	0	0	0	-19.380754	0	0	-1000	0
Streptococcus_pneumoniae_459_5	0	0	0	0	-19.344222	-1000	0	-1000	0
Streptococcus_pneumoniae_5185_06	0	0	0	0	-19.346611	0	0	-1000	0
Streptococcus_pneumoniae_5652_06	0	0	0	0	-19.353009	0	0	-1000	0
Streptococcus_pneumoniae_5787_06	0	0	0	0	-19.353003	0	0	-1000	0
Streptococcus_pneumoniae_670_6B	0	0	0	0	-19.350614	-1000	0	-1000	0
Streptococcus_pneumoniae_6901_05	0	0	0	0	-19.346605	0	0	-1000	0
Streptococcus_pneumoniae_6963_05	0	0	0	0	-19.353003	0	0	-1000	0
Streptococcus_pneumoniae_70585	0	0	-1000	0	-30.457303	-1000	0	-1000	0
Streptococcus_pneumoniae_7286_06	0	0	0	0	-19.353009	0	0	-1000	0
Streptococcus_pneumoniae_7533_05	0	0	0	0	-19.353009	0	0	-1000	0
Streptococcus_pneumoniae_7879_04	0	0	0	0	-19.317978	0	0	-1000	0
Streptococcus_pneumoniae_8190_05	0	0	0	0	-19.353009	0	0	-1000	0
Streptococcus_pneumoniae_AP200	0	0	0	0	-19.374076	0	0	-1000	0
Streptococcus_pneumoniae_ATCC_700669	0	0	0	0	-19.378348	-1000	0	-1000	0
Streptococcus_pneumoniae_BS397	0	0	0	0	-19.236212	-1000	0	-1000	0
Streptococcus_pneumoniae_BS455	0	0	0	0	-19.311206	-1000	0	-1000	0
Streptococcus_pneumoniae_BS457	0	0	0	0	-19.304926	-1000	0	-1000	0
Streptococcus_pneumoniae_BS458	0	0	0	0	-19.304922	-1000	0	-1000	0
Streptococcus_pneumoniae_CDC0288_04	0	0	0	0	-19.259483	0	0	-1000	0
Streptococcus_pneumoniae_CDC1873_00	0	0	0	0	-19.348263	-1000	0	-1000	0
Streptococcus_pneumoniae_CDC3059_06	0	0	0	0	-19.346611	0	0	-1000	0
Streptococcus_pneumoniae_CGSP14	0	0	0	0	-19.304908	-1000	0	-1000	0
Streptococcus_pneumoniae_D39	0	0	0	0	-19.118379	-1000	0	-1000	0
Streptococcus_pneumoniae_England14_9	0	0	0	0	-19.304926	-1000	0	-1000	0
Streptococcus_pneumoniae_EU_NP01	0	0	0	0	-19.346605	-1000	0	-1000	0
Streptococcus_pneumoniae_EU_NP02	0	0	0	0	-19.376479	0	0	-1000	0
Streptococcus_pneumoniae_EU_NP03	0	0	0	0	-19.317091	-1000	0	-1000	0
Streptococcus_pneumoniae_EU_NP04	0	0	0	0	-19.344378	0	0	-1000	0
Streptococcus_pneumoniae_EU_NP05	0	0	0	0	-19.344231	0	0	-1000	0
Streptococcus_pneumoniae_G54	0	0	0	-510.4166667	-26.637668	-1000	0	-1000	0
Streptococcus_pneumoniae_GA02254	0	0	0	0	-19.350636	0	0	-1000	0
Streptococcus_pneumoniae_GA02270	0	0	0	0	-19.348268	-1000	0	-1000	0
Streptococcus_pneumoniae_GA02506	0	0	0	0	-19.350775	-1000	0	-1000	0
Streptococcus_pneumoniae_GA02714	0	0	0	0	-19.319463	-1000	0	-1000	0
Streptococcus_pneumoniae_GA04175	0	0	0	0	-19.352999	0	0	-1000	0
Streptococcus_pneumoniae_GA04216	0	0	0	0	-19.374233	0	0	-1000	0
Streptococcus_pneumoniae_GA04375	0	0	0	0	-19.162751	0	0	-1000	0
Streptococcus_pneumoniae_GA04672	0	0	0	0	-19.353003	0	0	-1000	0
Streptococcus_pneumoniae_GA05245	0	0	0	0	-19.344231	0	0	-1000	0
Streptococcus_pneumoniae_GA05248	0	0	0	0	-19.350636	0	0	-1000	0
Streptococcus_pneumoniae_GA05578	0	0	0	0	-19.350775	0	0	-1000	0
Streptococcus_pneumoniae_GA06083	0	0	0	0	-19.350628	0	0	-1000	0
Streptococcus_pneumoniae_GA07228	0	0	0	0	-19.350644	0	0	-1000	0
Streptococcus_pneumoniae_GA07643	0	0	0	0	-19.346609	-1000	0	-1000	0
Streptococcus_pneumoniae_GA07914	0	0	0	0	-19.350636	0	0	-1000	0
Streptococcus_pneumoniae_GA08780	0	0	0	0	-19.299107	0	0	-1000	0
Streptococcus_pneumoniae_GA08825	0	0	0	0	-19.181396	0	0	-1000	0
Streptococcus_pneumoniae_GA11304	0	0	0	0	-19.34388	-1000	0	-1000	0
Streptococcus_pneumoniae_GA11426	0	0	0	0	-19.346609	0	0	-1000	0
Streptococcus_pneumoniae_GA13224	0	0	0	0	-19.344239	0	0	-1000	0
Streptococcus_pneumoniae_GA13338	0	0	0	0	-19.29969	-1000	0	-1000	0
Streptococcus_pneumoniae_GA13430	0	0	0	0	-19.352995	-1000	0	-1000	0
Streptococcus_pneumoniae_GA13455	0	0	0	0	-19.179199	0	0	-1000	0
Streptococcus_pneumoniae_GA13494	0	0	0	0	-19.344235	0	0	-1000	0
Streptococcus_pneumoniae_GA13856	0	0	0	0	-19.348175	0	0	-1000	0

Streptococcus_pneumoniae_GA14373	0	0	0	0	-19.341864	-1000	0	-1000	0
Streptococcus_pneumoniae_GA14688	0	0	0	0	-19.378374	-1000	0	-1000	0
Streptococcus_pneumoniae_GA14798	0	0	0	0	-19.344241	0	0	-1000	0
Streptococcus_pneumoniae_GA16121	0	0	0	0	-19.161532	0	0	-1000	0
Streptococcus_pneumoniae_GA16242	0	0	0	0	-19.350636	-1000	0	-1000	0
Streptococcus_pneumoniae_GA16531	0	0	0	0	-19.457876	-1000	0	-1000	0
Streptococcus_pneumoniae_GA17301	0	0	0	0	-19.305474	0	0	-1000	0
Streptococcus_pneumoniae_GA17328	0	0	0	0	-19.338369	-1000	0	-1000	0
Streptococcus_pneumoniae_GA17371	0	0	0	0	-19.179199	0	0	-1000	0
Streptococcus_pneumoniae_GA17457	0	0	0	0	-19.322793	0	0	-1000	0
Streptococcus_pneumoniae_GA17484	0	0	0	0	-19.128183	0	0	-1000	0
Streptococcus_pneumoniae_GA17545	0	0	0	0	-19.342744	-1000	0	-1000	0
Streptococcus_pneumoniae_GA17570	0	0	0	0	-19.297623	0	0	-1000	0
Streptococcus_pneumoniae_GA17719	0	0	0	0	-19.181391	0	0	-1000	0
Streptococcus_pneumoniae_GA17971	0	0	0	0	-19.269098	0	0	-1000	0
Streptococcus_pneumoniae_GA18068	0	0	0	0	-19.302689	-1000	0	-1000	0
Streptococcus_pneumoniae_GA18523	0	0	0	0	-19.346611	0	0	-1000	0
Streptococcus_pneumoniae_GA19077	0	0	0	0	-18.120592	-1000	0	-1000	0
Streptococcus_pneumoniae_GA19101	0	0	0	0	-19.341864	0	0	-1000	0
Streptococcus_pneumoniae_GA19690	0	0	0	0	-19.346881	0	0	-1000	0
Streptococcus_pneumoniae_GA19923	0	0	0	0	-19.353015	0	0	-1000	0
Streptococcus_pneumoniae_GA40028	0	0	0	0	-19.128192	0	0	-1000	0
Streptococcus_pneumoniae_GA40183	0	0	0	0	-19.353003	0	0	-1000	0
Streptococcus_pneumoniae_GA40410	0	0	0	0	-19.346605	0	0	-1000	0
Streptococcus_pneumoniae_GA40563	0	0	0	0	-19.35186	-1000	0	-1000	0
Streptococcus_pneumoniae_GA41277	0	0	0	0	-19.347741	0	0	-1000	0
Streptococcus_pneumoniae_GA41301	0	0	0	0	-19.350628	0	0	-1000	0
Streptococcus_pneumoniae_GA41317	0	0	0	0	-19.288046	-1000	0	-1000	0
Streptococcus_pneumoniae_GA41410	0	0	0	0	-18.10829	0	0	-1000	0
Streptococcus_pneumoniae_GA41538	0	0	0	0	-19.116106	-1000	0	-1000	0
Streptococcus_pneumoniae_GA41565	0	0	0	0	-19.346596	-1000	0	-1000	0
Streptococcus_pneumoniae_GA41688	0	0	0	0	-19.298559	-1000	0	-1000	0
Streptococcus_pneumoniae_GA43257	0	0	0	0	-19.305474	0	0	-1000	0
Streptococcus_pneumoniae_GA43264	0	0	0	0	-18.082181	-1000	0	-1000	0
Streptococcus_pneumoniae_GA43265	0	0	0	0	-19.346605	0	0	-1000	0
Streptococcus_pneumoniae_GA43380	0	0	0	0	-19.346601	-1000	0	-1000	0
Streptococcus_pneumoniae_GA44128	0	0	0	0	-19.350632	-1000	0	-1000	0
Streptococcus_pneumoniae_GA44194	0	0	0	0	-19.345259	-1000	0	-1000	0
Streptococcus_pneumoniae_GA44288	0	0	0	0	-19.346611	0	0	-1000	0
Streptococcus_pneumoniae_GA44378	0	0	0	0	-19.350628	-1000	0	-1000	0
Streptococcus_pneumoniae_GA44386	0	0	0	0	-19.350942	-1000	0	-1000	0
Streptococcus_pneumoniae_GA44452	0	0	0	0	-18.122067	-1000	0	-1000	0
Streptococcus_pneumoniae_GA44500	0	0	0	0	-19.290064	0	0	-1000	0
Streptococcus_pneumoniae_GA44511	0	0	0	0	-19.299107	0	0	-1000	0
Streptococcus_pneumoniae_GA47033	0	0	0	0	-19.344378	0	0	-1000	0
Streptococcus_pneumoniae_GA47179	0	0	0	0	-19.350767	-1000	0	-1000	0
Streptococcus_pneumoniae_GA47210	0	0	0	0	-19.132802	0	0	-1000	0
Streptococcus_pneumoniae_GA47281	0	0	0	0	-19.353007	0	0	-1000	0
Streptococcus_pneumoniae_GA47283	0	0	0	0	-19.111197	0	0	-1000	0
Streptococcus_pneumoniae_GA47360	0	0	0	0	-19.353007	-1000	0	-1000	0
Streptococcus_pneumoniae_GA47388	0	0	0	0	-19.299107	0	0	-1000	0
Streptococcus_pneumoniae_GA47439	0	0	0	0	-19.350636	0	0	-1000	0
Streptococcus_pneumoniae_GA47461	0	0	0	0	-19.128183	0	0	-1000	0
Streptococcus_pneumoniae_GA47502	0	0	0	0	-19.353143	0	0	-1000	0
Streptococcus_pneumoniae_GA47522	0	0	0	0	-19.350628	0	0	-1000	0
Streptococcus_pneumoniae_GA47562	0	0	0	0	-19.350636	0	0	-1000	0
Streptococcus_pneumoniae_GA47597	0	0	0	0	-19.348268	0	0	-1000	0
Streptococcus_pneumoniae_GA47628	0	0	0	0	-19.181396	0	0	-1000	0
Streptococcus_pneumoniae_GA47688	0	0	0	0	-19.353009	0	0	-1000	0
Streptococcus_pneumoniae_GA47751	0	0	0	0	-18.087195	-1000	0	-1000	0
Streptococcus_pneumoniae_GA47760	0	0	0	0	-19.350636	0	0	-1000	0
Streptococcus_pneumoniae_GA47794	0	0	0	0	-19.177692	-1000	0	-1000	0
Streptococcus_pneumoniae_GA47901	0	0	0	0	-19.350632	-1000	0	-1000	0
Streptococcus_pneumoniae_GA47976	0	0	0	0	-19.298563	-1000	0	-1000	0
Streptococcus_pneumoniae_GA49138	0	0	0	0	-19.14297	0	0	-1000	0
Streptococcus_pneumoniae_GA49194	0	0	0	0	-19.289528	-1000	0	-1000	0
Streptococcus_pneumoniae_GA49447	0	0	0	0	-18.064368	-1000	0	-1000	0
Streptococcus_pneumoniae_GA49542	0	0	0	0	-19.305474	0	0	-1000	0
Streptococcus_pneumoniae_GA52306	0	0	0	0	-19.34289	0	0	-1000	0
Streptococcus_pneumoniae_GA52612	0	0	0	0	-19.350628	-1000	0	-1000	0
Streptococcus_pneumoniae_GA54354	0	0	0	0	-19.350771	-1000	0	-1000	0
Streptococcus_pneumoniae_GA54644	0	0	0	0	-19.297623	0	0	-1000	0
Streptococcus_pneumoniae_GA56113	0	0	0	0	-19.276964	0	0	-1000	0
Streptococcus_pneumoniae_GA58581	0	0	0	0	-19.298559	-1000	0	-1000	0
Streptococcus_pneumoniae_GA58771	0	0	0	0	-19.380754	0	0	-1000	0
Streptococcus_pneumoniae_GA58981	0	0	0	0	-19.344227	-1000	0	-1000	0
Streptococcus_pneumoniae_GA60080	0	0	0	0	-18.755193	-1000	0	-1000	0
Streptococcus_pneumoniae_GA60132	0	0	0	0	-19.350767	-1000	0	-1000	0
Streptococcus_pneumoniae_GA60190	0	0	0	0	-19.350775	0	0	-1000	0
Streptococcus_pneumoniae_GA62331	0	0	0	0	-19.350771	-1000	0	-1000	0
Streptococcus_pneumoniae_GA62681	0	0	0	0	-19.350632	0	0	-1000	0
Streptococcus_pneumoniae_gamPNI0373	0	0	0	0	-31.109779	0	0	-1000	0
Streptococcus_pneumoniae_Hungary19A_6	0	0	0	0	-19.144442	-1000	0	-1000	0
Streptococcus_pneumoniae_INV104	0	0	0	0	-19.350614	-1000	0	-1000	0
Streptococcus_pneumoniae_INV200	0	0	0	0	-19.304912	-1000	0	-1000	0
Streptococcus_pneumoniae_JJA	0	0	0	0	-19.209408	-1000	0	-1000	0
Streptococcus_pneumoniae_MLV_016	0	0	0	0	-19.374104	0	0	-1000	0
Streptococcus_pneumoniae_MNZ11b	0	0	0	0	-19.350628	0	0	-1000	0
Streptococcus_pneumoniae_MNZ14	0	0	0	0	-19.236208	-1000	0	-1000	0
Streptococcus_pneumoniae_MNZ37	0	0	0	0	-19.350636	0	0	-1000	0
Streptococcus_pneumoniae_MNZ41	0	0	0	0	-19.038456	-1000	0	-1000	0

Streptococcus_pneumoniae_NorthCarolina6A_23	0	0	0	0	-19.348268	-1000	0	-1000	0
Streptococcus_pneumoniae_NP070	0	0	0	0	-19.343238	-1000	0	-1000	0
Streptococcus_pneumoniae_NP112	0	0	0	0	-19.374102	-1000	0	-1000	0
Streptococcus_pneumoniae_NP127	0	0	0	0	-19.313079	-1000	0	-1000	0
Streptococcus_pneumoniae_NP141	0	0	0	0	-19.128186	0	0	-1000	0
Streptococcus_pneumoniae_OXC141	0	0	0	0	-19.344225	0	0	-1000	0
Streptococcus_pneumoniae_P1031	0	0	0	0	-19.350618	0	0	-1000	0
Streptococcus_pneumoniae_PCS125219	0	0	0	0	-19.350632	0	0	-1000	0
Streptococcus_pneumoniae_PCS70012	0	0	0	0	-19.350652	0	0	-1000	0
Streptococcus_pneumoniae_PCS8106	0	0	0	0	-19.350624	-1000	0	-1000	0
Streptococcus_pneumoniae_PCS8203	0	0	0	0	-28.624931	-1000	0	-1000	0
Streptococcus_pneumoniae_PNI0002	0	0	0	0	-19.350632	0	0	-1000	0
Streptococcus_pneumoniae_PNI0006	0	0	0	0	-19.321992	0	0	-1000	0
Streptococcus_pneumoniae_PNI0010	0	0	0	0	-19.350656	0	0	-1000	0
Streptococcus_pneumoniae_PNI0153	0	0	0	0	-19.378376	0	0	-1000	0
Streptococcus_pneumoniae_R6	0	0	0	0	-18.166995	-1000	0	-1000	0
Streptococcus_pneumoniae_R6M2_PG	0	0	0	0	-19.14682	-1000	0	-1000	0
Streptococcus_pneumoniae_SP11_BS70	0	0	0	0	-19.340147	0	0	-1000	0
Streptococcus_pneumoniae_SP14_BS292	0	0	0	0	-19.328779	-1000	0	-1000	0
Streptococcus_pneumoniae_SP18_BS74	0	0	0	0	-19.344235	-1000	0	-1000	0
Streptococcus_pneumoniae_SP19_BS75	0	0	0	0	-19.34674	-1000	0	-1000	0
Streptococcus_pneumoniae_SP195	0	0	0	0	-19.299107	0	0	-1000	0
Streptococcus_pneumoniae_SP23_BS72	0	0	0	0	-19.475021	-1000	0	-1000	0
Streptococcus_pneumoniae_SP3_BS71	0	0	0	0	-19.360583	0	0	-1000	0
Streptococcus_pneumoniae_SP6_BS73	0	0	0	0	-21.346325	0	0	-1000	0
Streptococcus_pneumoniae_SP9_BS68	0	0	0	0	-19.270611	0	0	-1000	0
Streptococcus_pneumoniae_SPAR27	0	0	0	0	-18.964028	-1000	0	-1000	0
Streptococcus_pneumoniae_SPAR55	0	0	0	0	-18.102696	-1000	0	-1000	0
Streptococcus_pneumoniae_SPAR95	0	0	0	0	-19.299698	-1000	0	-1000	0
Streptococcus_pneumoniae_SPN021198	0	0	0	0	-19.350638	0	0	-1000	0
Streptococcus_pneumoniae_SPN032672	0	0	0	0	-19.350628	0	0	-1000	0
Streptococcus_pneumoniae_SPN033038	0	0	0	0	-19.350628	0	0	-1000	0
Streptococcus_pneumoniae_SPN034156	0	0	0	0	-19.37174	0	0	-1000	0
Streptococcus_pneumoniae_SPN034183	0	0	0	0	-19.374089	0	0	-1000	0
Streptococcus_pneumoniae_SPN061370	0	0	0	0	-19.344231	0	0	-1000	0
Streptococcus_pneumoniae_SPN072838	0	0	0	0	-19.350636	0	0	-1000	0
Streptococcus_pneumoniae_SPN1041	0	0	0	0	-19.350632	0	0	-1000	0
Streptococcus_pneumoniae_SPN7465	0	0	0	0	-19.350632	0	0	-1000	0
Streptococcus_pneumoniae_SPNA45	0	0	0	0	-19.339308	-1000	0	-1000	0
Streptococcus_pneumoniae_ST556	0	0	0	0	-19.208618	0	0	-1000	0
Streptococcus_pneumoniae_SV35	0	0	0	0	-19.209409	-1000	0	-1000	0
Streptococcus_pneumoniae_SV36	0	0	0	0	-19.348535	0	0	-1000	0
Streptococcus_pneumoniae_Taiwan19F_14	0	0	0	0	-30.601949	0	0	-1000	0
Streptococcus_pneumoniae_TCH8431_19A	0	0	0	0	-19.352994	0	0	-1000	0
Streptococcus_pneumoniae_TIGR4	0	0	0	0	-19.444733	-1000	0	-1000	0
Streptococcus_pseudopneumoniae_IS7493	0	0	0	-274.916245	-20.927698	-1000	0	-983.21798	0
Streptococcus_pyogenes_A20	0	0	0	0	-15.96228	0	0	-611.76656	0
Streptococcus_pyogenes_Alab49	0	0	0	0	-15.885698	0	0	-574.2479	0
Streptococcus_pyogenes_ATCC_10782	0	0	0	0	-16.322571	0	0	-667.68293	0
Streptococcus_pyogenes_M1_GAS	0	0	0	0	-15.574092	0	0	-578.14618	0
Streptococcus_pyogenes_MGAS10394	0	0	0	0	-15.54487	0	0	-622.98851	0
Streptococcus_pyogenes_MGAS315	0	0	0	0	-15.885698	0	0	-574.2479	0
Streptococcus_pyogenes_MGAS8232	0	0	0	0	-15.880922	0	0	-578.86718	0
Streptococcus_pyogenes_MGAS9429	0	0	0	0	-24.009198	0	0	-1000	0
Streptococcus_pyogenes_SSI_1	0	0	0	0	-15.885698	0	0	-574.24702	0
Streptococcus_pyogenes_str_Manfredo	0	0	0	0	-15.545727	0	0	-569.44444	0
Streptococcus_salivarius_57_I	0	0	0	0	-22.67542	0	0	-615.92179	0
Streptococcus_salivarius_DSM_20560	0	0	0	0	-19.535857	0	0	-562.5	0
Streptococcus_salivarius_ERR2221359	0	0	0	0	-19.691669	0	0	-554.34783	0
Streptococcus_salivarius_JIM8777	0	0	0	0	-18.386467	0	0	-958.33333	0
Streptococcus_salivarius_K12	0	0	0	0	-17.827833	0	0	-572.35846	0
Streptococcus_salivarius_M18	0	0	0	0	-18.130069	0	0	-572.91735	0
Streptococcus_salivarius_PS4	0	0	0	0	-17.743694	0	0	-979.16667	0
Streptococcus_salivarius_SK126	0	0	0	0	-17.890271	0	0	-563.43499	0
Streptococcus_sanguinis_ATCC_29667	0	0	0	0	-19.07619	0	0	-1000	0
Streptococcus_sanguinis_ATCC_49296	0	0	0	0	-15.933693	0	0	-760.06651	0
Streptococcus_sanguinis_SK1	0	0	0	0	-19.077875	0	0	-1000	0
Streptococcus_sanguinis_SK1056	0	0	0	0	-19.147379	0	0	-1000	0
Streptococcus_sanguinis_SK1057	0	0	0	0	-19.021517	0	0	-1000	0
Streptococcus_sanguinis_SK1058	0	0	0	0	-19.076199	0	0	-1000	0
Streptococcus_sanguinis_SK1059	0	0	0	0	-19.114234	0	0	-1000	0
Streptococcus_sanguinis_SK1087	0	0	0	0	-18.987108	0	0	-1000	0
Streptococcus_sanguinis_SK115	0	0	0	0	-19.055951	0	0	-1000	0
Streptococcus_sanguinis_SK150	0	0	0	0	-19.05595	0	0	-1000	0
Streptococcus_sanguinis_SK160	0	0	0	0	-19.110929	0	0	-1000	0
Streptococcus_sanguinis_SK330	0	0	0	0	-19.147371	-1000	0	-1000	0
Streptococcus_sanguinis_SK340	0	0	0	0	-19.077875	0	0	-1000	0
Streptococcus_sanguinis_SK353	0	0	0	0	-19.05595	0	0	-1000	0
Streptococcus_sanguinis_SK355	0	0	0	0	-19.055958	0	0	-1000	0
Streptococcus_sanguinis_SK36	0	0	0	0	-22.274714	0	0	-1000	0
Streptococcus_sanguinis_SK405	0	0	0	0	-19.147377	0	0	-1000	0
Streptococcus_sanguinis_SK408	0	0	0	0	-19.077304	0	0	-1000	0
Streptococcus_sanguinis_SK49	0	0	0	0	-18.860123	0	0	-1000	0
Streptococcus_sanguinis_SK678	0	0	0	0	-19.076199	0	0	-1000	0
Streptococcus_sanguinis_SK72	0	0	0	0	-19.14738	0	0	-1000	0
Streptococcus_sanguinis_VMC66	0	0	0	0	-19.113208	0	0	-1000	0
Streptococcus_sobrinus_NIDR_6715_7	0	0	0	0	-23.859504	-1000	0	-479.16667	0
Streptococcus_sobrinus_TCI_13	0	0	0	0	-22.352843	-532.96812	0	-265.10406	0
Streptococcus_sobrinus_TCI_16	0	0	0	0	-17.57154	-1000	0	0	0
Streptococcus_sobrinus_TCI_28	0	0	0	0	-17.629503	-1000	0	-510.99892	0
Streptococcus_sobrinus_TCI_50	0	0	0	0	-17.573806	-1000	0	-510.83928	0

Streptococcus_sobrinus_TCI_53	0	0	0	0	-25.843384	-1000	0	-500	0
Streptococcus_sp_2_1_36FAA	0	0	0	0	-34.485195	0	0	-607.14286	0
Streptococcus_sp_BS35b	0	0	0	0	-20.535105	0	0	0	0
Streptococcus_sp_GMD5S	0	0	0	0	-20.535092	0	0	0	0
Streptococcus_sp_HPH0090	0	0	0	0	-19.620464	0	0	0	0
Streptococcus_sp_I_G2	0	0	0	0	-200.46546	-1000	0	0	0
Streptococcus_sp_I_P16	0	0	0	0	-200.46563	-1000	0	0	0
Streptococcus_sp_SK140	0	0	0	0	-19.109679	0	0	0	0
Streptococcus_thermophilus_CNRZ1066	0	0	0	0	-17.267846	0	0	-544.47072	0
Streptococcus_thermophilus_JIM_8232	0	0	0	0	-17.979232	0	0	-567.69036	0
Streptococcus_thermophilus_LMD_9	0	0	0	0	-17.976597	0	0	-567.3167	0
Streptococcus_thermophilus_LMG_18311	0	0	0	0	-18.686216	0	0	-659.09091	0
Streptococcus_thermophilus_MN_ZLW_002	0	0	0	0	-17.726726	0	0	-563.5768	0
Streptococcus_thermophilus_ND03	0	0	0	-1000	-17.717299	0	0	-563.57911	0
Streptococcus_thoraltensis_DSM_12221	0	0	-1000	-58.00302615	-36.273305	-1000	0	-1000	0
Streptococcus_uberis_0140J	0	0	-1000	-75.12259856	-40.885043	-1000	0	-1000	0
Streptococcus_vestibularis_ATCC_49124	0	0	0	0	-17.841505	0	0	-559.58065	0
Streptococcus_vestibularis_F0396	0	0	0	0	-19.038911	0	0	-968.75	0
Streptomyces_massiliensis_AP10	0	0	0	-1000	-14.170221	0	0	0	0
Subdoligranulum_sp_4_3_54A2FAA	0	0	0	0	-30.004219	0	0	0	0
Subdoligranulum_variabile_DSM_15176	0	0	0	-1000	-24.168534	0	0	-1000	0
Succinatimonas_hippei_YIT_12066	0	0	0	-1000	-19.938121	0	0	0	0
Succiniclasticum_ruminis_DSM_11005	0	0	0	-750	-1.9137395	0	0	0	0
Succiniclasticum_ruminis_DSM_9236	0	0	0	-1000	-2.3215209	0	0	0	0
Succinivibrio_dextrinosolvens_H5	0	0	0	-19.17338385	0	0	0	0	0
Sutterella_parvirubra_YIT_11816	0	0	0	-840	-9.7439724	0	0	0	0
Sutterella_wadsworthensis_2_1_59BFAA	0	0	0	-1000	-5.1084403	0	0	0	0
Sutterella_wadsworthensis_3_1_45B	0	0	0	-1000	-1.8588462	0	0	0	0
Sutterella_wadsworthensis_ERR1203961	0	0	0	-1000	-9.0866213	0	0	0	0
Sutterella_wadsworthensis_ERR1204055	0	0	0	-1000	-9.0866184	0	0	0	0
Sutterella_wadsworthensis_HGA0223	0	0	0	-1000	-11.463692	0	0	0	0
Synergistes_jonesii_78_1	0	0	0	-1000	-12.66573	0	0	0	0
Synergistes_sp_3_1_syn1	0	0	0	-1000	-20.484725	0	0	0	0
Syntrophus_aciditrophicus_SB	0	0	0	0	-11.674831	0	0	0	0
Syntrophus_gentianae_DSM_8423	0	0	0	-1000	-15.258917	0	0	0	0
Tannerella_forsythia_ATCC_43037	0	0	0	0	-1000	0	0	0	0
Tannerella_sp_6_1_58FAA_CT1	0	0	-1000	0	-1000	0	0	0	0
Tatumella_ptyseos_ATCC_33301	0	0	0	-1000	-63.639777	0	0	-1000	0
Terrisporobacter_glycolicus_ERR2221120	0	0	0	-1000	-1000	0	0	0	0
Terrisporobacter_mayombei_ERR2221147	0	0	0	-1000	-36.512856	0	0	0	0
Terrisporobacter_nov_ERR2221319	0	0	0	-1000	-27.557415	0	0	0	0
Tessaracoccus_massiliensis_SIT6	0	0	0	0	-14.122182	0	0	0	0
Tetragenococcus_koreensis_KCTC_3924	0	0	0	0	-44.211124	-1000	0	-1000	-1000
Tetragenococcus_koreensis_NBRC_106072	0	0	0	0	-44.211117	-1000	0	-1000	-1000
Thalassobacillus_massiliensis_TM_1	0	0	0	-1000	-22.239907	-1000	0	-1000	0
Thermoanaerobacter_pseudethanolicus_ATCC_33	0	0	0	-31.50882438	-15.500231	-850.03244	0	-421.53058	0
Thermus_scotoductus_DSM_8553	0	0	0	0	-19.074521	0	-1000	0	0
Thermus_scotoductus_K1_1	0	0	0	0	-14.698956	0	-1000	0	0
Thermus_scotoductus_KI2	0	0	0	0	-16.313197	0	-1000	0	0
Thermus_scotoductus_SA_01	-7.531537171	0	0	0	-20.053886	0	-1000	0	0
Timonella_senegalensis_JC301	0	0	0	0	-16.915655	0	0	0	0
Trabulsiella_guamensis_ATCC_49490	0	0	-1000	-1000	-65.686783	-1000	0	0	0
Treponema_denticola_AL_2	0	0	0	-1000	-30.405421	0	0	0	0
Treponema_denticola_ASLM	0	0	0	-1000	-32.52522	0	0	0	0
Treponema_denticola_ATCC_33520	0	0	0	-1000	-38.869802	0	0	0	0
Treponema_denticola_ATCC_33521	0	0	0	-1000	-20.211593	0	0	0	0
Treponema_denticola_ATCC_35404	0	0	0	-1000	-31.627526	0	0	0	0
Treponema_denticola_H_22	0	0	0	-1000	-22.30967	0	0	0	0
Treponema_medium_ATCC_700293	0	0	0	-1000	-31.728023	0	0	-262.3649	0
Treponema_socranskii_subsp_paredis_ATCC_355	0	0	0	-1000	-21.051185	0	0	0	0
Treponema_socranskii_subsp_socranskii_VPI_DR	0	0	0	-1000	-26.351162	0	0	-527.77778	0
Treponema_succinifaciens_DSM_2489	0	0	0	0	-18.446019	0	0	-256.84918	0
Treponema_vincentii_ATCC_35580	0	0	0	-1000	-24.191995	0	0	0	0
Treponema_vincentii_F0403	0	0	0	-1000	-36.093823	0	0	0	0
Tropheryma_whipplei_str_Twist	0	0	0	-5.868815509	-3.8617167	0	0	0	0
Tropheryma_whipplei_TW08_27	0	0	0	0	-1.4828729	0	0	0	0
Trueperella_pyogenes_MS249	0	0	0	0	-41.173785	0	0	0	0
Turicibacter_sanguinis_ERR1022280	0	0	0	-1000	-26.611719	0	0	-500	0
Turicibacter_sanguinis_ERR1022323	0	0	-1000	-1000	-27.56657	0	0	-1000	0
Turicibacter_sanguinis_ERR1022367	0	0	0	-1000	-16.911059	0	0	-255.65822	0
Turicibacter_sanguinis_ERR1022463	0	0	0	-1000	-13.782255	0	0	-255.65816	0
Turicibacter_sanguinis_ERR2221386	0	0	0	-1000	-26.611749	0	0	-500	0
Turicibacter_sanguinis_ERR2230131	0	0	0	-1000	-26.611741	0	0	-500	0
Turicibacter_sanguinis_PC909	0	0	0	-1000	-17.364644	0	0	-500	0
Turicibacter_sp_H121	0	0	0	-20.20974659	-16.006914	0	0	-257.49161	0
Turicibacter_sp_HGF1	0	0	0	-27.9153004	-22.605697	0	0	-504.12108	0
Turicimonas_muris_YL45	0	0	0	-1000	-17.195597	0	0	0	0
Tyzzerella_nov_ERR171258	0	0	0	-45.28417705	-21.002708	0	0	-458.13282	0
uncultured_Anaerotruncus_sp_ERR1022410	0	0	-1000	-44.79717023	-26.415742	0	0	0	0
uncultured_Blautia_sp_ERR1022314	0	0	0	-1000	-57.471443	0	0	-1000	0
uncultured_Blautia_sp_ERR1022472	0	0	0	-38.12122096	-19.498856	0	0	-408.79452	0
uncultured_Clostridium_sp_ERR1022385	0	0	0	-42.56563503	-21.841146	0	0	-504.85901	0
uncultured_Clostridium_sp_ERR1022438	0	0	0	-1000	-24.464582	0	0	0	0
uncultured_Clostridium_sp_ERR1022467	0	0	0	-1000	-27.916108	0	0	-1000	0
uncultured_Ruminococcus_sp_ERR1022393	0	0	0	-1000	-17.27973	-1000	0	-500.30164	0
uncultured_Ruminococcus_sp_ERR1022403	0	0	0	-1000	-26.462206	0	0	-666.66667	0
Ureaplasma_parvum_serovar_1_str_ATCC_27813	0	0	0	-1.908394625	-2.6265492	0	0	0	0
Ureaplasma_parvum_serovar_14_str_ATCC_3369	0	0	0	-2.81970613	-2.2436384	0	0	0	0
Ureaplasma_parvum_serovar_3_str_ATCC_27815	0	0	0	-2.819074596	-2.2431417	0	0	0	0
Ureaplasma_parvum_serovar_3_str_ATCC_70097	0	0	0	-2.819074596	-2.2431417	0	0	0	0
Ureaplasma_parvum_serovar_6_str_ATCC_27818	0	0	0	-2.819155609	-2.2431914	0	0	0	0

Ureaplasma_urealyticum_2033	0	0	0	-2.831871754	-2.2495242	0	0	0	0
Ureaplasma_urealyticum_2608	0	0	0	-2.81970613	-2.2436384	0	0	0	0
Ureaplasma_urealyticum_4155	0	0	0	-2.819128604	-2.2431748	0	0	0	0
Ureaplasma_urealyticum_4318	0	0	0	-2.822411857	-2.2176145	0	0	0	0
Ureaplasma_urealyticum_serovar_10_str_ATCC_3	0	0	0	-2.819760163	-2.2436715	0	0	0	0
Ureaplasma_urealyticum_serovar_11_str_ATCC_3	0	0	0	-2.819236665	-2.243241	0	0	0	0
Ureaplasma_urealyticum_serovar_12_str_ATCC_3	0	0	0	-2.819787218	-2.2436881	0	0	0	0
Ureaplasma_urealyticum_serovar_13_str_ATCC_3	0	0	0	-2.819209659	-2.2432245	0	0	0	0
Ureaplasma_urealyticum_serovar_2_str_ATCC_27	0	0	0	-2.819787218	-2.2436881	0	0	0	0
Ureaplasma_urealyticum_serovar_4_str_ATCC_27	0	0	0	-2.819155609	-2.2431914	0	0	0	0
Ureaplasma_urealyticum_serovar_5_str_ATCC_27	0	0	0	-2.819209659	-2.2432245	0	0	0	0
Ureaplasma_urealyticum_serovar_7_str_ATCC_27	0	0	0	-2.819209659	-2.2432245	0	0	0	0
Ureaplasma_urealyticum_serovar_8_str_ATCC_27	0	0	0	-1.908380245	-2.6265262	0	0	0	0
Ureaplasma_urealyticum_serovar_9_str_ATCC_33	0	0	0	-2.819868272	-2.2437377	0	0	0	0
Ureibacillus_thermosphaericus_str_Thermo_BF	0	0	0	-1000	-13.627972	0	0	0	0
Vagococcus_fluvialis_bH819	0	0	0	0	-15.146033	-1000	0	0	0
Vagococcus_fluvialis_DSM_5731	0	0	0	0	-15.875018	-1000	0	0	0
Vallitalea_guaymasensis_L81	0	0	0	-1000	-16.990644	-1000	-1000	-784.31373	0
Vallitalea_sp_S15	0	0	0	-46.15767528	-22.714378	0	0	0	0
Varibaculum_cambriense_DNF00696	0	0	0	0	-30.953655	0	0	-510.42929	0
Varibaculum_cambriense_DSM_15806	0	0	0	-555.5555556	-31.47152	0	0	-686.11169	0
Variovorax_paradoxus_110B	0	0	0	-1000	-1000	-1000	-1000	0	0
Variovorax_paradoxus_4MFCol3_1	0	0	0	-1000	-1000	-1000	-1000	0	0
Variovorax_paradoxus_B4	0	0	0	-1000	-1000	-1000	-1000	0	0
Variovorax_paradoxus_H108	0	0	0	-1000	-1000	-1000	-1000	0	0
Variovorax_paradoxus_NBRC_15149	0	0	0	-1000	-1000	-1000	-1000	0	0
Veillonella_atypica_ACS_049_V_Sch6	0	0	0	-1000	-27.389783	0	-1000	0	0
Veillonella_atypica_ACS_134_V_Col7a	0	0	0	-1000	-23.027595	0	-1000	0	0
Veillonella_atypica_KON	0	0	0	-1000	-25.508523	0	-1000	0	0
Veillonella_denticariosi_JCM_15641	0	0	0	-1000	-24.457318	0	-1000	0	0
Veillonella_dispar_ATCC_17748	0	0	0	-1000	-47.351579	0	-1000	0	0
Veillonella_magna_DSM_19857	0	0	0	-1000	-23.613184	0	-1000	0	0
Veillonella_parvula_ACS_068_V_Sch12	0	0	0	-1000	-27.31407	0	-1000	0	0
Veillonella_parvula_ATCC_17745	0	0	0	-1000	-40.103295	0	-1000	0	0
Veillonella_parvula_Te3_DSM_2008	0	0	0	-1000	-168.71534	0	-1000	0	0
Veillonella_ratti_ACS_216_V_Col6b	0	0	0	-1000	-23.432105	0	-1000	0	0
Veillonella_sp_3_1_44	0	0	0	-1000	-34.789615	0	-1000	0	0
Veillonella_sp_6_1_27	0	0	0	-1000	-34.789615	0	-1000	0	0
Veillonella_sp_ACP1	0	0	0	-1000	-36.195687	0	-1000	0	0
Veillonella_sp_HPA0037	0	0	0	-1000	-35.19241	0	-1000	0	0
Veillonella_sp_oral_taxon_158_str_F0412	0	0	0	-1000	-35.608414	0	-1000	0	0
Veillonella_sp_oral_taxon_780_str_F0422	0	0	0	-1000	-38.235122	0	-1000	0	0
Veillonellaceae_nov_ERR2221308	0	0	0	-1000	-24.878886	0	0	0	0
Vibrio_campbellii_ATCC_BAA_1116	0	0	0	-1000	-35.317408	-1000	-1000	0	0
Vibrio_campbellii_HY01	0	0	0	-1000	-37.623333	-1000	-1000	0	0
Vibrio_cholerae_A1552	0	0	0	-1000	-40.518239	-1000	-1000	-1000	0
Vibrio_cholerae_O1_biovar_El_Tor_str_N16961	0	0	0	-1000	-41.121336	-1000	-1000	-1000	0
Vibrio_fluvialis_560	0	0	-1000	-1000	-36.468913	-1000	-1000	-1000	0
Vibrio_fluvialis_I21563	0	0	-1000	-1000	-46.985835	-1000	-1000	-1000	0
Vibrio_fluvialis_PG41	0	0	-1000	-1000	-47.904057	-1000	-1000	-1000	0
Vibrio_furnissii_CIP_102972	0	0	0	-1000	-54.480747	-1000	-1000	-1000	0
Vibrio_furnissii_NCTC_11218	0	0	0	-1000	-49.163491	-1000	-1000	-1000	0
Vibrio_harveyi_1DA3	0	0	-1000	-1000	-39.003601	-1000	-1000	-1000	0
Vibrio_mimicus_CAIM_602	0	0	0	-1000	-45.541744	-1000	0	0	0
Vibrio_mimicus_MB_451	0	0	0	-1000	-56.325213	-1000	0	0	0
Vibrio_mimicus_SX_4	0	0	0	-1000	-43.655827	-1000	0	0	0
Vibrio_mimicus_VM223	0	0	0	-1000	-44.771959	-1000	0	0	0
Vibrio_mimicus_VM573	0	0	0	-1000	-45.099367	-1000	0	0	0
Vibrio_mimicus_VM603	0	0	0	-1000	-43.971965	-1000	0	0	0
Vibrio_parahaemolyticus_10329	0	0	0	-1000	-1000	-1000	0	0	0
Vibrio_parahaemolyticus_AN_5034	0	0	0	-1000	-1000	-1000	0	0	0
Vibrio_parahaemolyticus_AQ3810	0	0	0	-1000	-1000	-1000	0	0	0
Vibrio_parahaemolyticus_BB22OP	0	0	0	-1000	-1000	-1000	0	0	0
Vibrio_parahaemolyticus_FORC_008	0	0	0	-1000	-1000	-1000	0	0	0
Vibrio_parahaemolyticus_PCV08_7	0	0	0	-1000	-1000	-1000	0	0	0
Vibrio_parahaemolyticus_RIMD_2210633	0	0	0	-1000	-1000	-1000	0	0	0
Vibrio_parahaemolyticus_SNUVpS_1	0	0	0	-1000	-1000	-1000	0	0	0
Vibrio_parahaemolyticus_v110	0	0	0	-1000	-1000	-1000	0	0	0
Vibrio_rotiferianus_DAT722	0	0	-1000	-92.30681096	-38.35327	0	0	-1000	0
Vibrio_shilonii_AK1	0	0	0	-1000	-55.554711	-1000	0	-1000	0
Vibrio_sinaloensis_AD032	0	0	0	-1000	-33.427489	-1000	-1000	-1000	0
Vibrio_sinaloensis_DSM_21326	0	0	0	-1000	-31.811288	-1000	-1000	-1000	0
Victivallis_vadensis_DSM_14823	0	0	0	-1000	-19.173025	-531.70807	0	-265.37321	0
Victivallis_vadensis_MGYG_HGUT_02473	0	0	0	-1000	-14.141734	-620.67462	0	-317.29695	0
Virgibacillus_massiliensis_MGYG_HGUT_01470	0	0	0	-1000	-30.723067	-1000	0	-882.80388	0
Virgibacillus_massiliensis_Vm_5	0	0	0	-1000	-19.374786	-1000	0	-886.87108	0
Virgibacillus_senegalensis_SK_1	0	0	0	-39.08463913	-15.205616	-1000	0	-875	0
Weissella_ceti_NC36	0	0	0	0	-35.486759	0	0	0	0
Weissella_cibaria_KACC_11862	0	0	0	-1000	-18.220393	0	0	-846.15385	0
Weissella_confusa_LBAE_C39_2	0	0	0	-74.07407407	-19.254978	0	0	-763.15789	0
Weissella_koreensis_KACC_15510	0	0	0	0	-25.534505	0	0	0	0
Weissella_koreensis_KCTC_3621	0	0	0	0	-25.553409	0	0	0	0
Weissella_paramesenteroides_ATCC_33313	0	0	0	-1000	-28.401423	0	0	-1000	0
Weissella_viridescens_DSM_20410	0	0	0	0	-15.386946	0	0	0	0
Yersinia_bercovieri_ATCC_43970	0	0	0	-1000	-55.587124	-1000	0	-1000	0
Yersinia_enterocolitica_FORC_002	0	0	-1000	-1000	-38.992727	-1000	0	-1000	0
Yersinia_enterocolitica_IP_10393	0	0	-1000	-1000	-53.685544	-1000	0	-1000	0
Yersinia_enterocolitica_IP2222	0	0	-1000	-1000	-58.119308	-1000	0	-1000	0
Yersinia_enterocolitica_NFO	0	0	-1000	-1000	-56.779458	-1000	0	-1000	0
Yersinia_enterocolitica_subsp_enterocolitica_808	-1000	0	0	-1000	-54.961907	-1000	0	-1000	0
Yersinia_enterocolitica_subsp_enterocolitica_WA	0	0	-1000	-1000	-47.641938	-1000	0	-1000	0

Yersinia_enterocolitica_subsp_palearctica_105_5	-26.48199017	0	-1000	-1000	-53.838431	-1000	0	-1000	0
Yersinia_enterocolitica_subsp_palearctica_556_8	0	0	-1000	-1000	-53.685551	-1000	0	-1000	0
Yersinia_enterocolitica_subsp_palearctica_647_5	0	0	-1000	-1000	-53.685544	-1000	0	-1000	0
Yersinia_enterocolitica_subsp_palearctica_PhRBD	0	0	-1000	-1000	-53.685544	-1000	0	-1000	0
Yersinia_enterocolitica_subsp_palearctica_Y11	-1000	0	-1000	-1000	-53.883807	-1000	0	-1000	0
Yersinia_enterocolitica_subsp_palearctica_YE_14	0	0	-1000	-1000	-53.685544	-1000	0	-1000	0
Yersinia_enterocolitica_subsp_palearctica_YE_15	0	0	-1000	-1000	-53.685544	-1000	0	-1000	0
Yersinia_enterocolitica_subsp_palearctica_YE_P1	0	0	-1000	-1000	-53.685544	-1000	0	-1000	0
Yersinia_enterocolitica_subsp_palearctica_YE_P4	0	0	-1000	-1000	-53.685544	-1000	0	-1000	0
Yersinia_enterocolitica_subsp_palearctica_YO527	0	0	-1000	-1000	-44.585177	-1000	0	-1000	0
Yersinia_frederiksenii_ATCC_33641	0	0	-1000	-1000	-56.564165	-1000	0	-1000	0
Yersinia_kristensenii_ATCC_33638	0	0	-1000	-1000	-62.967748	-1000	0	0	0
Yersinia_kristensenii_Y231	0	0	-1000	-1000	-39.34026	-1000	0	0	0
Yersinia_pseudotuberculosis_B_6863	0	0	-1000	-1000	-37.016716	-1000	0	0	0
Yersinia_pseudotuberculosis_B_7194	0	0	-1000	-1000	-37.016716	-1000	0	0	0
Yersinia_pseudotuberculosis_B_7195	0	0	-1000	-1000	-37.016716	-1000	0	0	0
Yersinia_pseudotuberculosis_IP_31758	-1000	0	-1000	-1000	-46.341797	-1000	0	0	0
Yersinia_pseudotuberculosis_IP_32953	-1000	0	-1000	-1000	-37.068818	-1000	0	0	0
Yersinia_pseudotuberculosis_PB1	-1000	0	-1000	-1000	-37.016716	-1000	0	0	0
Yersinia_pseudotuberculosis_YPIII	-1000	0	0	-1000	-53.428855	-1000	0	0	0
Yersinia_rohdei_ATCC_43380	0	0	-1000	-1000	-56.002027	-1000	0	-1000	0
Yokenella_regensburgei_ATCC_43003	0	0	-1000	-1000	-81.002383	-1000	0	-1000	0

Supplementary Table 14: Depression-associated metabolites included in AGORA2 that could be produced by at least one AGORA2 strain.
Shown is the production flux potential in mmol * g dry weight^{-1} * hr^{-1}.

VMH ID	5mta	abt	bz	glcur	glu_L	ind3ppa	leu_L	mnl	pyr	srtn
**Description**	**5-Methylthioadenosine**	**Arabitol**	**Benzoate**	**D-glucuronate**	**L-glutamate**	**Indole-3-propionate**	**L-leucine**	**D-Mannitol**	**Pyruvate**	**Serotonin**
Abiotrophia_defectiva_ATCC_49176	0	0	0	0	0	0	1000	0	0	0
Acaricomes_phytoseiuli_DSM_14247	0	0	1000	0	1000	0	1000	0	0	0
Acaryochloris_marina_MBIC11017	0	0	1000	0	1000	0	0	0	0	0
Acetanaerobacterium_elongatum_CGMCC_1_501	0	0	0	0	0	0	0	0	0	0
Acetatifactor_muris_GP69	0	0	0	0	0	0	1000	0	0	0
Acetivibrio_cellulolyticus_CD2	0	0	0	0	0	0	0	0	0	0
Acetivibrio_ethanolgignens_ACET_33324	0	0	0	0	1000	0	1000	0	1000	0
Acetobacterium_wieringae_DSM_1911	0	0	1000	0	1000	0	1000	0	0	0
Acetonema_longum_DSM_6540	0	0	0	0	1000	0	0	0	1000	0
Achromobacter_insuavis_AXX_A	0	0	1000	0	1000	0	0	0	0	0
Achromobacter_xylosoxidans_A8	384.6153846	0	1000	0	1000	0	0	0	0	1000
Achromobacter_xylosoxidans_ERR2221244	357.1428571	0	1000	0	1000	0	0	0	0	0
Achromobacter_xylosoxidans_ERR2221245	0	0	1000	0	1000	0	0	0	0	0
Achromobacter_xylosoxidans_ERR2221246	0	0	1000	0	1000	0	0	0	0	0
Achromobacter_xylosoxidans_ERR2221247	0	0	1000	0	1000	0	0	0	0	0
Achromobacter_xylosoxidans_NBRC_15126	266.6666667	0	1000	0	1000	0	0	0	0	1000
Achromobacter_xylosoxidans_NCTC10807	0	0	1000	0	1000	0	0	0	0	0
Acidaminobacter_hydrogenoformans_DSM_2784	0	0	0	0	1000	0	1000	0	1000	0
Acidaminococcus_fermentans_DSM_20731	0	0	0	0	1000	0	1000	0	0	0
Acidaminococcus_intestini_RyC_MR95	0	0	0	0	270.9677419	0	1000	0	0	0
Acidaminococcus_sp_BV3L6	0	0	0	0	1000	0	571.4285714	0	0	0
Acidaminococcus_sp_D21	0	0	0	0	1000	0	1000	0	0	0
Acidaminococcus_sp_HPA0509	0	0	0	0	1000	0	1000	0	0	0
Acidobacterium_ailaaui_PMMR2	0	0	0	1000	1000	0	0	0	0	0
Acidobacterium_capsulatum_ATCC_51196	0	0	0	0	0	0	0	0	0	0
Acidovorax_caeni_R_24608	0	0	1000	0	1000	0	1000	0	1000	0
Acinetobacter_baumannii_1656_2	0	0	0	0	1000	0	1000	0	0	0
Acinetobacter_baumannii_3990	0	0	0	0	1000	0	1000	0	0	0
Acinetobacter_baumannii_48055	0	0	0	0	1000	0	1000	0	0	0
Acinetobacter_baumannii_53264	0	0	0	0	1000	0	1000	0	0	0
Acinetobacter_baumannii_6013113	0	0	0	0	1000	0	1000	0	0	0
Acinetobacter_baumannii_6013150	0	0	0	0	1000	0	1000	0	0	0
Acinetobacter_baumannii_6014059	0	0	0	0	1000	0	1000	0	0	0
Acinetobacter_baumannii_AA_014	0	0	0	0	1000	0	1000	0	0	0
Acinetobacter_baumannii_AB_1536_8	0	0	0	0	1000	0	1000	0	0	0
Acinetobacter_baumannii_AB_1582_8	0	0	0	0	1000	0	1000	0	0	0
Acinetobacter_baumannii_AB_1594_8	0	0	0	0	1000	0	1000	0	0	0
Acinetobacter_baumannii_AB_1595_8	0	0	0	0	1000	0	1000	0	0	0
Acinetobacter_baumannii_AB_1649_8	0	0	0	0	1000	0	1000	0	0	0
Acinetobacter_baumannii_AB_1650_8	0	0	0	0	1000	0	1000	0	0	0
Acinetobacter_baumannii_AB_1766_8	0	0	0	0	1000	0	1000	0	0	0
Acinetobacter_baumannii_AB_2007_09_110_01_	0	0	0	0	1000	0	1000	0	0	0
Acinetobacter_baumannii_AB_2007_16_25_01_7	0	0	0	0	1000	0	1000	0	0	0
Acinetobacter_baumannii_AB_2007_16_27_01	0	0	0	0	1000	0	1000	0	0	0
Acinetobacter_baumannii_AB_2008_15_34_7	0	0	0	0	1000	0	1000	0	0	0
Acinetobacter_baumannii_AB_2008_15_45	0	0	0	0	1000	0	1000	0	0	0
Acinetobacter_baumannii_AB_2008_15_52	0	0	0	0	1000	0	1000	0	0	0
Acinetobacter_baumannii_AB_2008_15_69	0	0	0	0	1000	0	1000	0	0	0
Acinetobacter_baumannii_AB_2008_15_70	0	0	0	0	1000	0	1000	0	0	0
Acinetobacter_baumannii_AB_2008_15_71	0	0	0	0	1000	0	1000	0	0	0
Acinetobacter_baumannii_AB_2008_23_01_01_7	0	0	0	0	1000	0	1000	0	0	0
Acinetobacter_baumannii_AB_2008_23_07_01_7	0	0	0	0	1000	0	1000	0	0	0
Acinetobacter_baumannii_AB_2009_04_01_7	0	0	0	0	1000	0	1000	0	0	0
Acinetobacter_baumannii_AB_2009_04_02_7	0	0	0	0	1000	0	1000	0	0	0
Acinetobacter_baumannii_AB_515_8	0	0	0	0	1000	0	1000	0	0	0
Acinetobacter_baumannii_AB_908_12	0	0	0	0	1000	0	1000	0	0	0
Acinetobacter_baumannii_AB_908_13	0	0	0	0	1000	0	1000	0	0	0
Acinetobacter_baumannii_AB_909_01_7	0	0	0	0	1000	0	1000	0	0	0
Acinetobacter_baumannii_AB_909_02_7	0	0	0	0	1000	0	1000	0	0	0
Acinetobacter_baumannii_AB_909_05	0	0	0	0	1000	0	1000	0	0	0
Acinetobacter_baumannii_AB_TG19617	0	0	0	0	1000	0	1000	0	0	0
Acinetobacter_baumannii_AB_TG2018	0	0	0	0	1000	0	1000	0	0	0
Acinetobacter_baumannii_AB_TG2022	0	0	0	0	1000	0	1000	0	0	0
Acinetobacter_baumannii_AB_TG2023	0	0	0	0	1000	0	1000	0	0	0
Acinetobacter_baumannii_AB_TG2026	0	0	0	0	1000	0	1000	0	0	0
Acinetobacter_baumannii_AB_TG2028	0	0	0	0	1000	0	1000	0	0	0
Acinetobacter_baumannii_AB_TG2030	0	0	0	0	1000	0	1000	0	0	0
Acinetobacter_baumannii_AB_TG2031	0	0	0	0	1000	0	1000	0	0	0
Acinetobacter_baumannii_AB_TG27335	0	0	0	0	1000	0	1000	0	0	0
Acinetobacter_baumannii_AB_TG27339	0	0	0	0	1000	0	1000	0	0	0
Acinetobacter_baumannii_AB_TG27343	0	0	0	0	1000	0	1000	0	0	0
Acinetobacter_baumannii_AB0057	0	0	0	0	1000	0	1000	0	0	1000
Acinetobacter_baumannii_Ab11111	0	0	0	0	1000	0	1000	0	0	0
Acinetobacter_baumannii_AB1H8	0	0	0	0	1000	0	1000	0	0	0
Acinetobacter_baumannii_AB210	0	0	0	0	1000	0	1000	0	0	0
Acinetobacter_baumannii_AB307_0294	0	0	0	0	1000	0	1000	0	0	0
Acinetobacter_baumannii_AB31	0	0	0	0	1000	0	1000	0	0	0
Acinetobacter_baumannii_Ab33333	0	0	0	0	1000	0	1000	0	0	0
Acinetobacter_baumannii_AB405E4	0	0	0	0	1000	0	1000	0	0	0
Acinetobacter_baumannii_Ab44444	0	0	0	0	1000	0	1000	0	0	0
Acinetobacter_baumannii_AB4A3	0	0	0	0	1000	0	1000	0	0	0
Acinetobacter_baumannii_AB5256	0	0	0	0	1000	0	1000	0	0	0
Acinetobacter_baumannii_AB5711	0	0	0	0	1000	0	1000	0	0	0
Acinetobacter_baumannii_AB900	0	0	0	0	1000	0	1000	0	0	0
Acinetobacter_baumannii_ABIsac_ColiR	0	0	0	0	1000	0	1000	0	0	0
Acinetobacter_baumannii_ABNIH1	0	0	0	0	1000	0	1000	0	0	0
Acinetobacter_baumannii_ABNIH11	0	0	0	0	1000	0	1000	0	0	0
Acinetobacter_baumannii_ABNIH14	0	0	0	0	1000	0	1000	0	0	0

Acinetobacter_baumannii_ABNIH15	0	0	0	0	1000	0	1000	0	0	0
Acinetobacter_baumannii_ABNIH16	0	0	0	0	1000	0	1000	0	0	0
Acinetobacter_baumannii_ABNIH18	0	0	0	0	1000	0	1000	0	0	0
Acinetobacter_baumannii_ABNIH19	0	0	0	0	1000	0	1000	0	0	0
Acinetobacter_baumannii_ABNIH22	0	0	0	0	1000	0	1000	0	0	0
Acinetobacter_baumannii_ABNIH23	0	0	0	0	1000	0	1000	0	0	0
Acinetobacter_baumannii_ABNIH25	0	0	0	0	1000	0	1000	0	0	0
Acinetobacter_baumannii_ABNIH26	0	0	0	0	1000	0	1000	0	0	0
Acinetobacter_baumannii_ABNIH3	0	0	0	0	1000	0	1000	0	0	0
Acinetobacter_baumannii_ABNIH4	0	0	0	0	1000	0	1000	0	0	0
Acinetobacter_baumannii_ABNIH5	0	0	0	0	1000	0	1000	0	0	0
Acinetobacter_baumannii_ABNIH6	0	0	0	0	1000	0	1000	0	0	0
Acinetobacter_baumannii_AC12	0	0	0	0	1000	0	1000	0	0	0
Acinetobacter_baumannii_AC30	0	0	0	0	1000	0	1000	0	0	0
Acinetobacter_baumannii_ACICU	0	0	0	0	1000	0	1000	0	0	0
Acinetobacter_baumannii_ANC_4097	0	0	0	0	1000	0	1000	0	0	0
Acinetobacter_baumannii_ATCC_17978	0	0	0	0	1000	0	1000	0	0	0
Acinetobacter_baumannii_ATCC_19606	0	0	0	0	1000	0	1000	0	0	0
Acinetobacter_baumannii_AYE	0	0	0	0	1000	0	1000	0	0	0
Acinetobacter_baumannii_BZICU_2	0	0	1000	0	1000	0	1000	0	0	0
Acinetobacter_baumannii_Canada_BC_5	0	0	0	0	1000	0	1000	0	0	0
Acinetobacter_baumannii_Canada_BC1	0	0	0	0	1000	0	1000	0	0	0
Acinetobacter_baumannii_IS_116	0	0	0	0	1000	0	1000	0	0	0
Acinetobacter_baumannii_IS_123	0	0	0	0	1000	0	1000	0	0	0
Acinetobacter_baumannii_IS_143	0	0	0	0	1000	0	1000	0	0	0
Acinetobacter_baumannii_IS_235	0	0	0	0	1000	0	1000	0	0	0
Acinetobacter_baumannii_IS_251	0	0	0	0	1000	0	1000	0	0	0
Acinetobacter_baumannii_IS_58	0	0	0	0	1000	0	1000	0	0	0
Acinetobacter_baumannii_LAC_4	0	0	0	0	1000	0	1000	0	0	0
Acinetobacter_baumannii_MDR_TJ	0	0	0	0	1000	0	1000	0	0	0
Acinetobacter_baumannii_MDR_ZJ06	0	0	0	0	1000	0	1000	0	0	0
Acinetobacter_baumannii_MSP4_16	0	0	0	0	1000	0	1000	0	0	0
Acinetobacter_baumannii_Naval_113	0	0	0	0	1000	0	1000	0	0	0
Acinetobacter_baumannii_Naval_13	0	0	0	0	1000	0	1000	0	0	0
Acinetobacter_baumannii_Naval_17	0	0	0	0	1000	0	1000	0	0	0
Acinetobacter_baumannii_Naval_18	0	0	0	0	1000	0	1000	0	0	0
Acinetobacter_baumannii_Naval_2	0	0	0	0	1000	0	1000	0	0	0
Acinetobacter_baumannii_Naval_21	0	0	0	0	1000	0	1000	0	0	0
Acinetobacter_baumannii_Naval_57	0	0	0	0	1000	0	1000	0	0	0
Acinetobacter_baumannii_Naval_72	0	0	0	0	1000	0	1000	0	0	0
Acinetobacter_baumannii_Naval_78	0	0	0	0	1000	0	1000	0	0	0
Acinetobacter_baumannii_Naval_81	0	0	0	0	1000	0	1000	0	0	0
Acinetobacter_baumannii_Naval_82	0	0	0	0	1000	0	1000	0	0	0
Acinetobacter_baumannii_Naval_83	0	0	0	0	1000	0	1000	0	0	0
Acinetobacter_baumannii_NIPH_1362	0	0	0	0	1000	0	1000	0	0	0
Acinetobacter_baumannii_NIPH_146	0	0	0	0	1000	0	1000	0	0	0
Acinetobacter_baumannii_NIPH_1669	0	0	0	0	1000	0	1000	0	0	0
Acinetobacter_baumannii_NIPH_1734	0	0	0	0	1000	0	1000	0	0	0
Acinetobacter_baumannii_NIPH_190	0	0	0	0	1000	0	1000	0	0	0
Acinetobacter_baumannii_NIPH_201	0	0	0	0	1000	0	1000	0	0	0
Acinetobacter_baumannii_NIPH_2061	0	0	0	0	1000	0	1000	0	0	0
Acinetobacter_baumannii_NIPH_24	0	0	0	0	1000	0	1000	0	0	0
Acinetobacter_baumannii_NIPH_290	0	0	0	0	1000	0	1000	0	0	0
Acinetobacter_baumannii_NIPH_329	0	0	0	0	1000	0	1000	0	0	0
Acinetobacter_baumannii_NIPH_335	0	0	0	0	1000	0	1000	0	0	0
Acinetobacter_baumannii_NIPH_410	0	0	0	0	1000	0	1000	0	0	0
Acinetobacter_baumannii_NIPH_527	0	0	0	0	1000	0	1000	0	0	0
Acinetobacter_baumannii_NIPH_528	0	0	0	0	1000	0	1000	0	0	0
Acinetobacter_baumannii_NIPH_60	0	0	0	0	1000	0	1000	0	0	0
Acinetobacter_baumannii_NIPH_601	0	0	0	0	1000	0	1000	0	0	0
Acinetobacter_baumannii_NIPH_615	0	0	0	0	1000	0	1000	0	0	0
Acinetobacter_baumannii_NIPH_67	0	0	0	0	1000	0	1000	0	0	0
Acinetobacter_baumannii_NIPH_70	0	0	0	0	1000	0	1000	0	0	0
Acinetobacter_baumannii_NIPH_80	0	0	0	0	1000	0	1000	0	0	0
Acinetobacter_baumannii_OIFC0162	0	0	0	0	1000	0	1000	0	0	0
Acinetobacter_baumannii_OIFC032	0	0	0	0	1000	0	1000	0	0	0
Acinetobacter_baumannii_OIFC035	0	0	0	0	1000	0	1000	0	0	0
Acinetobacter_baumannii_OIFC047	0	0	0	0	1000	0	1000	0	0	0
Acinetobacter_baumannii_OIFC065	0	0	0	0	1000	0	1000	0	0	0
Acinetobacter_baumannii_OIFC074	0	0	0	0	1000	0	1000	0	0	0
Acinetobacter_baumannii_OIFC087	0	0	0	0	1000	0	1000	0	0	0
Acinetobacter_baumannii_OIFC098	0	0	0	0	1000	0	1000	0	0	0
Acinetobacter_baumannii_OIFC099	0	0	0	0	1000	0	1000	0	0	0
Acinetobacter_baumannii_OIFC109	0	0	0	0	1000	0	1000	0	0	0
Acinetobacter_baumannii_OIFC110	0	0	0	0	1000	0	1000	0	0	0
Acinetobacter_baumannii_OIFC111	0	0	0	0	1000	0	1000	0	0	0
Acinetobacter_baumannii_OIFC137	0	0	0	0	1000	0	1000	0	0	0
Acinetobacter_baumannii_OIFC143	0	0	0	0	1000	0	1000	0	0	0
Acinetobacter_baumannii_OIFC180	0	0	0	0	1000	0	1000	0	0	0
Acinetobacter_baumannii_OIFC189	0	0	0	0	1000	0	1000	0	0	0
Acinetobacter_baumannii_OIFC338	0	0	0	0	1000	0	1000	0	0	0
Acinetobacter_baumannii_SDF	0	0	0	0	1000	0	1000	0	0	0
Acinetobacter_baumannii_TCDC_AB0715	0	0	0	0	1000	0	1000	0	0	0
Acinetobacter_baumannii_TYTH_1	0	0	0	0	1000	0	1000	0	0	0
Acinetobacter_baumannii_UMB001	0	0	0	0	1000	0	1000	0	0	0
Acinetobacter_baumannii_UMB003	0	0	0	0	1000	0	1000	0	0	0
Acinetobacter_baumannii_W7282	0	0	0	0	1000	0	1000	0	0	0
Acinetobacter_baumannii_WC_348	0	0	0	0	1000	0	1000	0	0	0
Acinetobacter_baumannii_WC_692	0	0	0	0	1000	0	1000	0	0	0
Acinetobacter_baumannii_WC_A_694	0	0	0	0	1000	0	1000	0	0	0
Acinetobacter_baumannii_WC_A_92	0	0	0	0	1000	0	1000	0	0	0
Acinetobacter_baumannii_WM99c	0	0	0	0	1000	0	1000	0	0	0
Acinetobacter_baumannii_ZWS1122	0	0	0	0	1000	0	1000	0	0	0
Acinetobacter_baumannii_ZWS1219	0	0	0	0	1000	0	1000	0	0	0

Acinetobacter_calcoaceticus_ANC_3680	0	0	0	0	1000	0	1000	0	0	0
Acinetobacter_calcoaceticus_ANC_3811	0	0	0	0	1000	0	1000	0	0	0
Acinetobacter_calcoaceticus_DSM_30006_CIP_81	0	0	0	0	1000	0	1000	0	0	0
Acinetobacter_calcoaceticus_NIPH_13	0	0	0	0	1000	0	1000	0	0	0
Acinetobacter_calcoaceticus_PHEA_2	0	0	0	0	1000	0	1000	0	0	1000
Acinetobacter_calcoaceticus_RUH2202	0	0	0	0	1000	0	1000	0	0	0
Acinetobacter_calcoaceticus_subsp_anitratus_XM	0	0	0	0	1000	0	1000	0	0	0
Acinetobacter_calcoaceticus_TG19585	0	0	0	0	1000	0	1000	0	0	0
Acinetobacter_calcoaceticus_TG19588	0	0	0	0	1000	0	1000	0	0	0
Acinetobacter_calcoaceticus_TG19593	0	0	0	0	1000	0	1000	0	0	0
Acinetobacter_guillouiae_CIP_63_46	0	0	0	0	1000	0	1000	0	0	0
Acinetobacter_guillouiae_KCTC_23200	0	0	0	0	1000	0	1000	0	0	0
Acinetobacter_guillouiae_MSP4_18	0	0	0	0	1000	0	1000	0	0	0
Acinetobacter_guillouiae_NIPH_991	0	0	0	0	1000	0	1000	0	0	0
Acinetobacter_haemolyticus_ATCC_19194	0	0	0	0	1000	0	1000	0	0	0
Acinetobacter_haemolyticus_CIP_64_3	0	0	0	0	1000	0	1000	0	0	0
Acinetobacter_haemolyticus_NIPH_261	0	0	0	0	1000	0	1000	0	0	1000
Acinetobacter_haemolyticus_TG19599	0	0	0	0	1000	0	1000	0	0	0
Acinetobacter_haemolyticus_TG21157	0	0	0	0	1000	0	1000	0	0	0
Acinetobacter_johnsonii_ANC_3681	0	0	0	0	1000	0	1000	0	0	0
Acinetobacter_johnsonii_CIP_64_6	0	0	0	0	1000	0	1000	0	0	0
Acinetobacter_johnsonii_SH046	0	0	0	0	1000	0	1000	0	0	1000
Acinetobacter_junii_CIP_107470	0	0	0	0	1000	0	1000	0	0	0
Acinetobacter_junii_CIP_64_5	0	0	0	0	1000	0	1000	0	0	0
Acinetobacter_junii_NIPH_182	0	0	0	0	1000	0	1000	0	0	0
Acinetobacter_junii_SH205	0	0	0	0	1000	0	1000	0	0	0
Acinetobacter_lwoffii_CIP_70_31	0	0	0	0	1000	0	1000	0	0	0
Acinetobacter_lwoffii_ERR2221242	0	0	0	0	1000	0	1000	0	0	0
Acinetobacter_lwoffii_NCTC_5866	0	0	0	0	1000	0	1000	0	0	0
Acinetobacter_lwoffii_NIPH_478	0	0	0	0	1000	0	1000	0	0	0
Acinetobacter_lwoffii_NIPH_715	0	0	0	0	1000	0	1000	0	0	0
Acinetobacter_lwoffii_SH145	0	0	0	0	1000	0	1000	0	0	0
Acinetobacter_lwoffii_TG19636	0	0	0	0	1000	0	1000	0	0	0
Acinetobacter_lwoffii_WJ10621	0	0	0	0	1000	0	1000	0	0	0
Acinetobacter_nosocomialis_NIPH_2119	0	0	0	0	1000	0	1000	0	0	0
Acinetobacter_nosocomialis_WC_487	0	0	0	0	1000	0	1000	0	0	0
Acinetobacter_pittii_ANC_3678	0	0	0	0	1000	0	1000	0	0	0
Acinetobacter_pittii_ANC_4050	0	0	0	0	1000	0	1000	0	0	0
Acinetobacter_pittii_ANC_4052	0	0	0	0	1000	0	1000	0	0	1000
Acinetobacter_pittii_AP_882	0	0	0	0	1000	0	1000	0	0	0
Acinetobacter_pittii_CIP_70_29	0	0	0	0	1000	0	1000	0	0	0
Acinetobacter_pittii_D499	0	0	0	0	1000	0	1000	0	0	0
Acinetobacter_pittii_IEC338SC	0	0	0	0	1000	0	1000	0	0	0
Acinetobacter_pittii_TG6411	0	0	0	0	1000	0	1000	0	0	0
Acinetobacter_pittii_WC_136	0	0	0	0	1000	0	1000	0	0	0
Acinetobacter_radioresistens_DSM_6976	0	0	0	0	1000	0	1000	0	0	0
Acinetobacter_radioresistens_NIPH_2130	0	0	0	0	1000	0	1000	0	0	1000
Acinetobacter_radioresistens_SH164	0	0	0	0	1000	0	1000	0	0	0
Acinetobacter_radioresistens_SK82	0	0	0	0	1000	0	1000	0	0	0
Acinetobacter_radioresistens_TG02010	0	0	0	0	1000	0	1000	0	0	0
Acinetobacter_radioresistens_WC_A_157	0	0	0	0	1000	0	1000	0	0	0
Acinetobacter_sp_ATCC_27244	0	0	0	0	1000	0	1000	0	0	0
Acinetobacter_sp_OIFC021	0	0	0	0	1000	0	1000	0	0	0
Acinetobacter_sp_RUH2624	0	0	0	0	1000	0	1000	0	0	0
Acinetobacter_sp_SH024	0	0	0	0	1000	0	1000	0	0	0
Acinetobacter_sp_WC_141	0	0	0	0	1000	0	1000	0	0	0
Acinetobacter_sp_WC_323	0	0	0	0	1000	0	1000	0	0	0
Actinobacillus_pleuropneumoniae_L20	0	0	0	0	1000	0	1000	0	0	1000
Actinobacillus_pleuropneumoniae_S8	0	0	0	0	1000	0	1000	0	0	0
Actinobacillus_pleuropneumoniae_serovar_1_str_	0	0	0	0	1000	0	1000	0	0	0
Actinobacillus_pleuropneumoniae_serovar_10_str	0	0	0	0	1000	0	1000	0	0	0
Actinobacillus_pleuropneumoniae_serovar_11_str	0	0	0	0	1000	0	1000	0	0	0
Actinobacillus_pleuropneumoniae_serovar_12_str	0	0	0	0	1000	0	1000	0	0	0
Actinobacillus_pleuropneumoniae_serovar_13_str	0	0	0	0	1000	0	1000	0	0	0
Actinobacillus_pleuropneumoniae_serovar_2_str_	0	0	0	0	1000	0	1000	0	0	0
Actinobacillus_pleuropneumoniae_serovar_2_str_	0	0	0	0	1000	0	1000	0	0	0
Actinobacillus_pleuropneumoniae_serovar_3_str_	0	0	0	0	1000	0	1000	0	0	0
Actinobacillus_pleuropneumoniae_serovar_4_str_	0	0	0	0	1000	0	1000	0	0	0
Actinobacillus_pleuropneumoniae_serovar_6_str_	0	0	0	0	1000	0	1000	0	0	0
Actinobacillus_pleuropneumoniae_serovar_7_str_	0	0	0	0	1000	0	1000	0	0	0
Actinobacillus_pleuropneumoniae_serovar_9_str_	0	0	0	0	1000	0	1000	0	0	0
Actinomyces_bowdenii_OH5050	0	0	0	0	1000	0	1000	0	0	0
Actinomyces_cardiffensis_F0333	0	0	0	0	1000	0	1000	0	0	0
Actinomyces_europaeus_ACS_120_V_Col10b	0	0	0	0	0	0	1000	0	0	0
Actinomyces_europaeus_UMB0652_ERR1203664_	0	0	0	0	0	0	1000	0	0	0
Actinomyces_georgiae_DSM_6843	0	0	0	0	0	0	1000	0	0	0
Actinomyces_gerencseriae_DSM_6844	0	0	0	0	1000	0	1000	0	0	0
Actinomyces_graevenitzii_C83	0	0	0	0	1000	0	1000	0	0	0
Actinomyces_hongkongensis_HKU8	0	0	0	0	0	0	1000	0	0	0
Actinomyces_israelii_DSM_43320	0	0	0	0	1000	0	1000	0	0	0
Actinomyces_johnsonii_F0510	0	0	0	0	1000	0	1000	0	0	0
Actinomyces_johnsonii_F0542	0	0	0	0	1000	0	1000	0	0	0
Actinomyces_massiliensis_4401292	0	0	0	0	1000	0	1000	0	0	0
Actinomyces_massiliensis_F0489	0	0	0	0	1000	0	1000	0	0	0
Actinomyces_meyeri_DSM_20733	0	0	0	0	0	0	1000	0	0	0
Actinomyces_naeslundii_ERR2221356	0	0	0	0	1000	0	1000	0	0	0
Actinomyces_naeslundii_str_Howell_279	0	0	0	0	1000	0	1000	0	0	0
Actinomyces_odontolyticus_ATCC_17982	0	0	0	0	1000	0	1000	0	0	0
Actinomyces_odontolyticus_F0309	0	0	0	0	1000	0	1000	0	0	0
Actinomyces_oris_K20	0	0	0	0	0	0	1000	0	0	0
Actinomyces_oris_T14V	0	0	0	0	1000	0	1000	0	0	0
Actinomyces_sp_HPA0247	0	0	0	0	1000	0	1000	0	0	0
Actinomyces_sp_ICM39	0	0	0	0	1000	0	1000	0	0	0
Actinomyces_sp_ICM47	0	0	0	0	1000	0	1000	0	0	0

Actinomyces_timonensis_DSM_23838	0	0	0	0	1000	0	1000	0	0	0
Actinomyces_turicensis_ACS_279_V_Col4	0	0	0	0	1000	0	0	0	0	0
Actinomyces_urogenitalis_DSM_15434	0	0	0	0	0	0	1000	0	0	0
Actinomyces_urogenitalis_S6_C4	0	0	0	0	1000	0	1000	0	0	0
Actinomyces_viscosus_C505	0	0	0	0	1000	0	1000	0	0	0
Acutalibacter_muris_KB18	0	0	0	0	0	0	0	0	0	0
Adlercreutzia_equolifaciens_DSM_19450	0	0	0	0	1000	0	1000	0	0	0
Adlercreutzia_equolifaciens_ERR2221192	0	0	0	0	1000	0	1000	0	0	0
Adlercreutzia_muris_DSM_29508	0	0	1000	0	1000	0	1000	0	0	0
Advenella_mimigardefordensis_DPN7	0	0	0	0	0	0	0	0	0	0
Aerococcus_christensenii_CCUG28831	0	0	0	0	1000	0	1000	0	0	0
Aerococcus_viridans_ATCC_11563	0	0	0	0	1000	0	1000	0	0	0
Aerococcus_viridans_LL1	0	0	1000	0	1000	0	1000	0	0	0
Aeromicrobium_massiliense_JC14	0	0	1000	1000	1000	0	0	0	0	0
Aeromonas_caviae_Ae398	0	0	0	0	1000	0	1000	0	0	1000
Aeromonas_caviae_FDAARGOS_72	0	0	0	0	0	0	1000	0	0	0
Aeromonas_caviae_FDAARGOS_75	0	0	0	0	0	0	1000	0	0	0
Aeromonas_dhakensis_173	0	0	0	0	1000	0	1000	0	1000	0
Aeromonas_dhakensis_277	0	0	0	0	1000	0	1000	0	1000	0
Aeromonas_dhakensis_SSU	0	0	0	0	1000	0	1000	0	1000	0
Aeromonas_hydrophila_116	0	0	0	0	0	0	1000	0	0	0
Aeromonas_hydrophila_14	0	0	0	0	1000	0	1000	0	0	0
Aeromonas_hydrophila_187	0	0	0	0	1000	0	1000	0	0	0
Aeromonas_hydrophila_259	0	0	0	0	1000	0	1000	0	0	0
Aeromonas_hydrophila_AH10	0	0	0	0	1000	0	1000	0	0	0
Aeromonas_hydrophila_ML09_119	0	0	0	0	1000	0	1000	0	0	0
Aeromonas_hydrophila_RB_AH	0	0	0	0	1000	0	1000	0	0	0
Aeromonas_hydrophila_SNUFPC_A8	0	0	0	0	1000	0	1000	0	0	0
Aeromonas_hydrophila_subsp_hydrophila_ATCC_	0	0	0	0	1000	0	1000	0	0	1000
Aeromonas_jandaei_Riv2	0	0	1000	0	1000	0	1000	0	0	1000
Aeromonas_media_ARB13	0	0	0	0	0	0	1000	0	0	0
Aeromonas_media_WS	0	0	1000	0	1000	0	1000	0	0	1000
Aeromonas_veronii_AER39	0	0	0	0	1000	0	1000	0	0	0
Aeromonas_veronii_AER397	0	0	0	0	1000	0	1000	0	0	0
Aeromonas_veronii_AMC34	0	0	0	0	1000	0	1000	0	0	0
Aeromonas_veronii_AMC35	0	0	0	0	1000	0	1000	0	0	0
Aeromonas_veronii_AVNIH1	0	0	0	0	0	0	1000	0	0	0
Aeromonas_veronii_B565	0	0	1000	0	1000	0	1000	0	0	1000
Aeromonas_veronii_TH0426	0	0	0	0	0	0	1000	0	0	0
Afipia_birgiae_34632	0	0	0	0	0	0	0	1000	0	1000
Aggregatibacter_aphrophilus_ATCC_33389	0	0	0	0	1000	0	1000	0	0	0
Aggregatibacter_aphrophilus_F0387	0	0	0	0	1000	0	1000	0	0	0
Aggregatibacter_aphrophilus_NJ8700	0	0	0	0	0	0	1000	0	0	1000
Aggregatibacter_aphrophilus_W10433	0	0	0	0	0	0	1000	0	0	0
Aggregatibacter_segnis_933_AAPH_107_59761_2	0	0	0	0	0	0	1000	0	0	0
Aggregatibacter_segnis_ATCC_33393	0	0	0	0	0	0	1000	0	0	0
Agrobacterium_fabrum_str_C58	0	0	1000	0	0	0	0	0	0	0
Agrobacterium_tumefaciens_CCNWGS0286	0	0	1000	0	1000	0	0	0	1000	0
Agrobacterium_tumefaciens_F2	0	0	0	0	1000	0	0	0	1000	0
Agrobacterium_tumefaciens_str_Cherry_2E_2_2	0	0	1000	0	1000	0	0	0	1000	0
Akkermansia_muciniphila_ATCC_BAA_835	0	0	1000	0	0	0	0	0	0	0
Akkermansia_muciniphila_YL44	0	0	1000	0	0	0	1000	0	0	0
Akkermansia_sp_KLE1797	0	0	1000	0	0	0	1000	0	0	0
Akkermansia_sp_KLE1798	0	0	1000	0	0	0	1000	0	0	0
Alcaligenes_faecalis_NBIB_017	0	0	1000	0	1000	0	1000	0	0	0
Alcaligenes_faecalis_subsp_faecalis_NBRC_13111	0	0	1000	0	1000	0	1000	0	0	1000
Alcaligenes_faecalis_subsp_faecalis_NCIB_8687	0	0	0	0	1000	0	1000	0	0	1000
Alcaligenes_faecalis_ZD02	0	0	1000	0	1000	0	1000	0	0	0
Aliivibrio_fischeri_ES114	0	0	0	0	0	0	1000	0	0	0
Aliivibrio_fischeri_MJ11	0	0	0	0	0	0	1000	0	0	0
Aliivibrio_salmonicida_LFI1238	0	0	0	0	1000	0	1000	0	0	0
Alistipes_finegoldii_DSM_17242	0	0	0	0	1000	0	0	0	0	0
Alistipes_ihumii_AP11	0	0	0	0	0	0	0	0	0	0
Alistipes_indistinctus_ERR2221377	0	0	1000	0	0	0	1000	1000	0	0
Alistipes_indistinctus_YIT_12060	0	0	0	0	1000	0	0	1000	0	0
Alistipes_nov_ERR2221210	0	0	0	0	833.3333333	0	0	0	0	0
Alistipes_nov_ERR2221392	0	0	1000	0	1000	0	0	0	0	0
Alistipes_obesi_isolate_ph8	0	0	0	0	0	0	0	0	0	0
Alistipes_onderdonkii_DSM_19147	0	0	0	0	0	0	0	0	0	0
Alistipes_onderdonkii_ERR1022348	0	0	0	0	1000	0	0	0	0	0
Alistipes_onderdonkii_ERR1022461	0	0	0	0	1000	0	0	0	0	0
Alistipes_onderdonkii_ERR1203947	0	0	0	0	1000	0	1000	0	0	0
Alistipes_onderdonkii_ERR1204041	0	0	0	0	1000	0	1000	0	0	0
Alistipes_onderdonkii_ERR2221102	0	0	0	0	1000	0	0	0	0	0
Alistipes_onderdonkii_ERR2230089	0	0	0	0	1000	0	0	0	0	0
Alistipes_onderdonkii_ERR2230093	0	0	0	0	1000	0	0	0	0	0
Alistipes_putredinis_DSM_17216	0	0	0	0	1000	0	0	0	0	0
Alistipes_senegalensis_JC50	0	0	0	1000	0	0	0	0	0	0
Alistipes_shahii_ERR2221103	0	0	0	0	1000	0	0	0	0	0
Alistipes_shahii_ERR2221307	0	0	0	0	1000	0	0	0	0	0
Alistipes_shahii_ERR2221376	0	0	0	0	1000	0	1000	0	0	0
Alistipes_shahii_WAL_8301	0	0	0	1000	0	0	0	0	0	0
Alistipes_sp_AL_1	0	0	0	0	1000	0	0	0	0	0
Alistipes_sp_cv1_ERR1022444	0	0	0	0	1000	0	1000	0	0	0
Alistipes_sp_HGB5	0	0	0	0	1000	0	1000	0	0	0
Alistipes_timonensis_ERR2221390	0	0	0	0	1000	0	0	0	0	0
Alistipes_timonensis_JC136	0	0	0	0	1000	0	0	0	0	0
Alkalibaculum_bacchi_DSM_22112_Ga0244545_1	0	0	0	0	0	0	1000	0	1000	0
Alkaliphilus_transvaalensis_ATCC_700919	0	0	1000	0	1000	0	0	0	0	0
Allobaculum_stercoricanis_DSM_13633	0	0	0	0	0	0	1000	0	0	0
Alloiococcus_otitis_ATCC_51267	0	0	0	0	1000	0	1000	0	0	0
Alloprevotella_rava_F0323	0	0	0	0	0	0	0	0	0	0
Alloprevotella_tannerae_ATCC_51259	0	0	0	0	0	0	0	0	0	0
Alloscardovia_omnicolens_1036_GVAG_103_587_	0	0	0	0	0	0	1000	0	0	0

Alloscardovia_omnicolens_DSM_21503	0	0	0	0	0	0	1000	0	0	0
Alloscardovia_omnicolens_F0580	0	0	0	0	0	0	1000	0	0	0
Amazonia_massiliensis_MS4	0	0	0	0	0	0	0	0	0	0
Aminivibrio_pyruvatiphilus_DSM_25964	0	0	1000	0	1000	0	1000	0	1000	0
Anaerobacillus_macyae_DSM_16346	0	0	1000	0	1000	0	1000	0	0	0
Anaerobaculum_hydrogeniformans_OS1_ATCC_B/	0	0	1000	0	1000	0	1000	0	0	0
Anaerobiospirillum_succiniciproducens_DSM_640	0	0	0	0	1000	0	1000	0	0	0
Anaerococcus_hydrogenalis_ACS_025_V_Sch4	0	0	0	0	1000	0	1000	0	0	0
Anaerococcus_hydrogenalis_DSM_7454	0	0	0	0	1000	0	1000	0	0	0
Anaerococcus_lactolyticus_ATCC_51172	0	0	0	0	1000	0	1000	0	0	0
Anaerococcus_lactolyticus_S7_1_13	0	0	0	0	1000	0	1000	0	0	0
Anaerococcus_obesiensis_ph10	0	0	0	0	1000	0	1000	0	0	0
Anaerococcus_prevotii_ACS_065_V_Col13	0	0	0	0	1000	0	1000	0	0	0
Anaerococcus_prevotii_DSM_20548	0	0	0	0	1000	0	1000	0	0	0
Anaerococcus_senegalensis_JC48	0	0	0	0	1000	0	1000	0	0	0
Anaerococcus_tetradius_ATCC_35098	0	0	0	1000	0	0	1000	0	0	0
Anaerococcus_tetradius_MJR8151	0	0	0	0	0	0	1000	0	0	0
Anaerococcus_vaginalis_ATCC_51170	0	0	0	0	1000	0	1000	0	0	0
Anaerofustis_stercorihominis_DSM_17244	0	0	0	0	0	0	1000	0	0	0
Anaeroglobus_geminatus_F0357	0	0	1000	0	1000	0	0	0	0	0
Anaeroplasma_bactoclasticum_ATCC_27112	0	0	0	0	0	0	1000	0	0	0
Anaerosalibacter_bizertensis_Med78_601_WT_4V	0	0	0	0	1000	0	1000	0	1000	0
Anaerosalibacter_massiliensis_ND1	0	0	0	0	1000	0	1000	0	0	0
Anaerosporobacter_mobilis_DSM_15930	0	0	0	0	0	0	1000	0	0	0
Anaerostipes_caccae_DSM_14662	0	0	0	0	1000	0	0	0	0	0
Anaerostipes_caccae_ERR1203925	0	0	0	0	1000	0	1000	0	0	0
Anaerostipes_caccae_ERR171259	0	0	0	0	1000	0	1000	0	0	0
Anaerostipes_caccae_ERR2221104	0	0	0	0	1000	0	1000	0	0	0
Anaerostipes_caccae_ERR2221232	0	0	0	0	1000	0	1000	0	0	0
Anaerostipes_hadrus_DSM_3319	0	0	0	0	1000	0	1000	0	0	0
Anaerostipes_hadrus_ERR1022288	0	0	0	0	1000	0	1000	0	0	0
Anaerostipes_hadrus_ERR1022326	0	0	0	0	1000	0	1000	0	0	0
Anaerostipes_hadrus_ERR1022376	0	0	0	0	1000	0	1000	0	0	0
Anaerostipes_hadrus_ERR1022422	0	0	0	0	1000	0	1000	0	0	0
Anaerostipes_hadrus_ERR1022423	0	0	0	0	1000	0	1000	0	0	0
Anaerostipes_hadrus_ERR1022473	0	0	0	0	1000	0	1000	0	0	0
Anaerostipes_hadrus_ERR2221197	0	0	0	0	1000	0	1000	0	0	0
Anaerostipes_hadrus_ERR2221199	0	0	0	0	1000	0	1000	0	0	0
Anaerostipes_nov_ERR2221209	0	0	0	0	1000	0	1000	0	0	0
Anaerostipes_sp_3_2_56FAA	0	0	0	0	1000	0	1000	0	0	0
Anaerotignum_lactatifermentans_DSM_14214	0	0	1000	0	1000	0	0	0	1000	0
Anaerotignum_propionicum_DSM_1682	0	0	1000	0	0	0	0	0	0	0
Anaerotruncus_colihominis_DSM_17241	0	0	0	0	0	0	1000	0	1000	0
Anaerotruncus_colihominis_ERR1022453	0	0	0	0	0	0	1000	0	1000	0
Anaerotruncus_colihominis_ERR171260	0	0	0	0	0	0	1000	0	1000	0
Anaerotruncus_colihominis_ERR2221105	0	0	0	0	0	0	1000	0	1000	0
Anaerotruncus_nov_ERR2221396	0	0	0	0	1000	0	1000	0	0	0
Anaerotruncus_sp_G3_2012	0	0	0	1000	0	0	0	0	0	0
Anaerovorax_odorimutans_DSM_5092	0	0	0	0	0	0	0	0	0	0
Aneurinibacillus_aneurinilyticus_ATCC_12856	0	0	1000	0	1000	0	1000	0	0	0
Arcanobacterium_haemolyticum_DSM_20595	0	0	0	0	0	0	1000	0	0	0
Arcobacter_butzleri_7h1h	0	0	1000	0	0	0	1000	0	0	0
Arcobacter_butzleri_ED_1	0	0	1000	0	1000	0	1000	0	0	0
Arcobacter_butzleri_JV22	0	0	1000	0	1000	0	1000	0	0	0
Arcobacter_butzleri_RM4018	0	0	0	0	0	0	0	0	0	0
Ardenticatena_maritima_110S	0	0	1000	0	0	0	0	0	1000	0
Arthrobacter_castelli_DSM_16402	0	0	1000	0	1000	0	0	0	0	0
Atopobium_minutum_10063974	0	0	0	0	0	0	1000	0	0	1000
Atopobium_parvulum_DSM_20469	0	0	0	0	1000	0	0	0	0	1000
Atopobium_rimae_ATCC_49626	0	0	0	0	1000	0	0	0	0	1000
Atopobium_vaginae_CMW7778A	0	0	0	0	0	0	0	0	0	0
Atopobium_vaginae_DSM_15829	0	0	0	0	0	0	0	0	0	0
Atopobium_vaginae_PB189_T1_4	0	0	0	0	0	0	0	0	0	0
Auritidibacter_ignavus_IMMIB_L_1656	0	0	0	0	1000	0	1000	0	0	0
Bacillus_altitudinis_41KF2b	0	0	1000	0	1000	0	1000	0	0	0
Bacillus_altitudinis_S_1	0	0	1000	0	1000	0	1000	0	0	0
Bacillus_amyloliquefaciens_DSM7	0	0	0	0	1000	0	1000	0	0	0
Bacillus_amyloliquefaciens_FZB42	0	0	1000	0	1000	0	1000	0	0	0
Bacillus_amyloliquefaciens_LL3	0	0	1000	0	1000	0	1000	0	0	0
Bacillus_amyloliquefaciens_subsp_amyloliquefacie	0	0	1000	0	1000	0	1000	0	0	0
Bacillus_amyloliquefaciens_TA208	0	0	1000	0	1000	0	1000	0	0	0
Bacillus_amyloliquefaciens_XH7	0	0	1000	0	1000	0	1000	0	0	0
Bacillus_amyloliquefaciens_Y2	0	0	1000	0	1000	0	1000	0	0	0
Bacillus_andreraoultii_KW_12	0	0	1000	0	1000	0	1000	0	0	0
Bacillus_atrophaeus_1013_1	0	0	1000	0	1000	0	1000	0	0	0
Bacillus_atrophaeus_1013_2	0	0	1000	0	1000	0	1000	0	0	0
Bacillus_atrophaeus_1942	0	0	1000	0	1000	0	1000	0	0	0
Bacillus_atrophaeus_ATCC_49822_1	0	0	0	0	1000	0	1000	0	0	0
Bacillus_atrophaeus_ATCC_49822_2	0	0	1000	0	1000	0	1000	0	0	0
Bacillus_atrophaeus_ATCC_9372_1	0	0	1000	0	1000	0	1000	0	0	0
Bacillus_atrophaeus_ATCC_9372_2	0	0	1000	0	1000	0	1000	0	0	0
Bacillus_atrophaeus_BACI051_E	0	0	1000	0	1000	0	1000	0	0	0
Bacillus_atrophaeus_BACI051_N	0	0	1000	0	1000	0	1000	0	0	0
Bacillus_atrophaeus_C89	0	0	1000	0	1000	0	1000	0	0	0
Bacillus_atrophaeus_Detrick_1	0	0	1000	0	1000	0	1000	0	0	0
Bacillus_atrophaeus_Detrick_2	0	0	1000	0	1000	0	1000	0	0	0
Bacillus_atrophaeus_Detrick_3	0	0	1000	0	1000	0	1000	0	0	0
Bacillus_atrophaeus_DJHJ8	0	0	1000	0	1000	0	1000	0	0	0
Bacillus_atrophaeus_str_Dugway	0	0	1000	0	1000	0	1000	0	0	0
Bacillus_atrophaeus_UCMB_5137	0	0	1000	0	1000	0	1000	0	0	0
Bacillus_cereus_03BB102	1000	0	1000	0	1000	0	1000	0	0	0
Bacillus_cereus_03BB108	0	0	1000	0	1000	0	1000	0	0	0
Bacillus_cereus_172560W	0	0	1000	0	1000	0	1000	0	0	0
Bacillus_cereus_95	0	0	1000	0	1000	0	1000	0	0	0

Bacillus_cereus_AH1134	0	0	1000	0	1000	0	1000	0	0	0
Bacillus_cereus_AH1271	0	0	1000	0	1000	0	1000	0	0	0
Bacillus_cereus_AH1272	0	0	1000	0	1000	0	1000	0	0	0
Bacillus_cereus_AH1273	0	0	1000	0	1000	0	1000	0	0	0
Bacillus_cereus_AH187_F4810_72	1000	0	1000	0	1000	0	1000	0	0	0
Bacillus_cereus_AH676	0	0	1000	0	1000	0	1000	0	0	0
Bacillus_cereus_AH820	1000	0	1000	0	1000	0	1000	0	0	0
Bacillus_cereus_AND1407	0	0	1000	0	1000	0	1000	0	0	0
Bacillus_cereus_ATCC_10876	0	0	1000	0	1000	0	1000	0	0	0
Bacillus_cereus_ATCC_10987	0	0	1000	0	1000	0	1000	0	0	0
Bacillus_cereus_ATCC_14579	1000	0	1000	0	1000	0	1000	0	0	0
Bacillus_cereus_ATCC_4342	0	0	1000	0	1000	0	1000	0	0	0
Bacillus_cereus_B4264	1000	0	1000	0	1000	0	1000	0	0	0
Bacillus_cereus_B5_2	0	0	1000	0	1000	0	1000	0	0	0
Bacillus_cereus_BAG1O_1	0	0	1000	0	1000	0	1000	0	0	0
Bacillus_cereus_BAG1O_3	0	0	1000	0	1000	0	1000	0	0	0
Bacillus_cereus_BAG1X1_1	0	0	1000	0	1000	0	1000	0	0	0
Bacillus_cereus_BAG1X1_2	0	0	1000	0	1000	0	1000	0	0	0
Bacillus_cereus_BAG1X1_3	0	0	1000	0	1000	0	1000	0	0	0
Bacillus_cereus_BAG1X2_1	0	0	1000	0	1000	0	1000	0	0	0
Bacillus_cereus_BAG1X2_2	0	0	1000	0	1000	0	1000	0	0	0
Bacillus_cereus_BAG1X2_3	0	0	1000	0	1000	0	1000	0	0	0
Bacillus_cereus_BAG2O_1	0	0	1000	0	1000	0	1000	0	0	0
Bacillus_cereus_BAG2O_2	0	0	1000	0	1000	0	1000	0	0	0
Bacillus_cereus_BAG2O_3	0	0	1000	0	1000	0	1000	0	0	0
Bacillus_cereus_BAG2X1_1	0	0	1000	0	1000	0	1000	0	0	0
Bacillus_cereus_BAG2X1_3	0	0	1000	0	1000	0	1000	0	0	0
Bacillus_cereus_BAG3O_1	0	0	1000	0	1000	0	1000	0	0	0
Bacillus_cereus_BAG3O_2	0	0	1000	0	1000	0	1000	0	0	0
Bacillus_cereus_BAG3X2_1	0	0	1000	0	1000	0	1000	0	0	0
Bacillus_cereus_BAG3X2_2	0	0	1000	0	1000	0	1000	0	0	0
Bacillus_cereus_BAG4O_1	0	0	1000	0	1000	0	1000	0	0	0
Bacillus_cereus_BAG4X12_1	0	0	1000	0	1000	0	1000	0	0	0
Bacillus_cereus_BAG4X2_1	0	0	1000	0	1000	0	1000	0	0	0
Bacillus_cereus_BAG5O_1	0	0	1000	0	1000	0	1000	0	0	0
Bacillus_cereus_BAG5X1_1	0	0	1000	0	1000	0	1000	0	0	0
Bacillus_cereus_BAG5X12_1	0	0	1000	0	1000	0	1000	0	0	0
Bacillus_cereus_BAG6O_1	0	0	1000	0	1000	0	1000	0	0	0
Bacillus_cereus_BAG6O_2	0	0	1000	0	1000	0	1000	0	0	0
Bacillus_cereus_BAG6X1_2	0	0	1000	0	1000	0	1000	0	0	0
Bacillus_cereus_BDRD_Cer4	0	0	1000	0	1000	0	1000	0	0	0
Bacillus_cereus_BDRD_ST196	0	0	1000	0	1000	0	1000	0	0	0
Bacillus_cereus_BDRD_ST24	0	0	1000	0	1000	0	1000	0	0	0
Bacillus_cereus_BDRD_ST26	0	0	1000	0	1000	0	1000	0	0	0
Bacillus_cereus_BGSC_6E1	0	0	1000	0	1000	0	1000	0	0	0
Bacillus_cereus_biovar_anthracis_str_CI	0	0	1000	0	1000	0	1000	0	0	0
Bacillus_cereus_BMG1_7	0	0	1000	0	1000	0	1000	0	0	0
Bacillus_cereus_E33L	1000	0	1000	0	1000	0	1000	0	0	0
Bacillus_cereus_F	0	0	1000	0	1000	0	1000	0	0	0
Bacillus_cereus_F65185	0	0	1000	0	1000	0	1000	0	0	0
Bacillus_cereus_F837	0	0	1000	0	1000	0	1000	0	0	0
Bacillus_cereus_FORC_005	0	0	1000	0	1000	0	1000	0	0	0
Bacillus_cereus_FRI_35	0	0	1000	0	1000	0	1000	0	0	0
Bacillus_cereus_G9241	0	0	1000	0	1000	0	1000	0	0	0
Bacillus_cereus_G9842	1000	0	1000	0	1000	0	1000	0	0	0
Bacillus_cereus_H3081_97	0	0	1000	0	1000	0	1000	0	0	0
Bacillus_cereus_HD73	0	0	1000	0	1000	0	1000	0	0	0
Bacillus_cereus_HuA2_1	0	0	1000	0	1000	0	1000	0	0	0
Bacillus_cereus_HuA2_3	0	0	1000	0	1000	0	1000	0	0	0
Bacillus_cereus_HuA2_4	0	0	1000	0	1000	0	1000	0	0	0
Bacillus_cereus_HuA2_9	0	0	1000	0	1000	0	1000	0	0	0
Bacillus_cereus_HuA3_9	0	0	1000	0	1000	0	1000	0	0	0
Bacillus_cereus_HuA4_10	0	0	1000	0	1000	0	1000	0	0	0
Bacillus_cereus_HuB1_1	0	0	1000	0	1000	0	1000	0	0	0
Bacillus_cereus_HuB13_1	0	0	1000	0	1000	0	1000	0	0	0
Bacillus_cereus_HuB2_9	0	0	1000	0	1000	0	1000	0	0	0
Bacillus_cereus_HuB4_4	0	0	1000	0	1000	0	1000	0	0	0
Bacillus_cereus_HuB5_5	0	0	1000	0	1000	0	1000	0	0	0
Bacillus_cereus_IS075	0	0	1000	0	1000	0	1000	0	0	0
Bacillus_cereus_IS195	0	0	1000	0	1000	0	1000	0	0	0
Bacillus_cereus_IS845_00	0	0	1000	0	1000	0	1000	0	0	0
Bacillus_cereus_ISP2954	0	0	1000	0	1000	0	1000	0	0	0
Bacillus_cereus_ISP3191	0	0	1000	0	1000	0	1000	0	0	0
Bacillus_cereus_K_5975c	0	0	1000	0	1000	0	1000	0	0	0
Bacillus_cereus_LCT_BC244	0	0	1000	0	1000	0	1000	0	0	0
Bacillus_cereus_m1293	0	0	1000	0	1000	0	1000	0	0	0
Bacillus_cereus_m1550	0	0	1000	0	1000	0	1000	0	0	0
Bacillus_cereus_MC118	0	0	1000	0	1000	0	1000	0	0	0
Bacillus_cereus_MC67	0	0	1000	0	1000	0	1000	0	0	0
Bacillus_cereus_MSX_A1	0	0	1000	0	1000	0	1000	0	0	0
Bacillus_cereus_MSX_A12	0	0	1000	0	1000	0	1000	0	0	0
Bacillus_cereus_MSX_D12	0	0	1000	0	1000	0	1000	0	0	0
Bacillus_cereus_NC7401	1000	0	1000	0	1000	0	1000	0	0	0
Bacillus_cereus_NVH0597_99	0	0	1000	0	1000	0	1000	0	0	0
Bacillus_cereus_Q1	1000	0	1000	0	1000	0	1000	0	0	0
Bacillus_cereus_R309803	0	0	1000	0	1000	0	1000	0	0	0
Bacillus_cereus_Rock1_15	0	0	1000	0	1000	0	1000	0	0	0
Bacillus_cereus_Rock1_3	0	0	1000	0	1000	0	1000	0	0	0
Bacillus_cereus_Rock3_28	0	0	1000	0	1000	0	1000	0	0	0
Bacillus_cereus_Rock3_29	0	0	1000	0	1000	0	1000	0	0	0
Bacillus_cereus_Rock3_42	0	0	1000	0	1000	0	1000	0	0	0
Bacillus_cereus_Rock3_44	0	0	1000	0	1000	0	1000	0	0	0
Bacillus_cereus_Rock4_18	0	0	1000	0	1000	0	1000	0	0	0
Bacillus_cereus_Rock4_2	0	0	1000	0	1000	0	1000	0	0	0

Bacillus_cereus_SJ1	0	0	1000	0	1000	0	1000	0	0	0
Bacillus_cereus_str_Schrouff	0	0	1000	0	1000	0	1000	0	0	0
Bacillus_cereus_subsp_cytotoxis_NVH_391_98	0	0	1000	0	1000	0	1000	0	0	0
Bacillus_cereus_TIAC219	0	0	1000	0	1000	0	1000	0	0	0
Bacillus_cereus_VD014	0	0	1000	0	1000	0	1000	0	0	0
Bacillus_cereus_VD021	0	0	1000	0	1000	0	1000	0	0	0
Bacillus_cereus_VD022	0	0	1000	0	1000	0	1000	0	0	0
Bacillus_cereus_VD045	0	0	1000	0	1000	0	1000	0	0	0
Bacillus_cereus_VD048	0	0	1000	0	1000	0	1000	0	0	0
Bacillus_cereus_VD102	0	0	1000	0	1000	0	1000	0	0	0
Bacillus_cereus_VD107	0	0	1000	0	1000	0	1000	0	0	0
Bacillus_cereus_VD115	0	0	1000	0	1000	0	1000	0	0	0
Bacillus_cereus_VD118	0	0	1000	0	1000	0	1000	0	0	0
Bacillus_cereus_VD131	0	0	1000	0	1000	0	1000	0	0	0
Bacillus_cereus_VD133	0	0	1000	0	1000	0	1000	0	0	0
Bacillus_cereus_VD136	0	0	1000	0	1000	0	1000	0	0	0
Bacillus_cereus_VD140	0	0	1000	0	1000	0	1000	0	0	0
Bacillus_cereus_VD142	0	0	1000	0	1000	0	1000	0	0	0
Bacillus_cereus_VD146	0	0	1000	0	1000	0	1000	0	0	0
Bacillus_cereus_VD148	0	0	1000	0	1000	0	1000	0	0	0
Bacillus_cereus_VD154	0	0	1000	0	1000	0	1000	0	0	0
Bacillus_cereus_VD156	0	0	1000	0	1000	0	1000	0	0	0
Bacillus_cereus_VD166	0	0	1000	0	1000	0	1000	0	0	0
Bacillus_cereus_VD169	0	0	1000	0	1000	0	1000	0	0	0
Bacillus_cereus_VD184	0	0	1000	0	1000	0	1000	0	0	0
Bacillus_cereus_VD196	0	0	1000	0	1000	0	1000	0	0	0
Bacillus_cereus_VD200	0	0	1000	0	1000	0	1000	0	0	0
Bacillus_cereus_VD214	0	0	1000	0	1000	0	1000	0	0	0
Bacillus_cereus_VDM006	0	0	1000	0	1000	0	1000	0	0	0
Bacillus_cereus_VDM021	0	0	1000	0	1000	0	1000	0	0	0
Bacillus_cereus_VDM034	0	0	1000	0	1000	0	1000	0	0	0
Bacillus_cereus_VDM053	0	0	1000	0	1000	0	1000	0	0	0
Bacillus_cereus_VDM062	0	0	1000	0	1000	0	1000	0	0	0
Bacillus_cereus_W	0	0	1000	0	1000	0	1000	0	0	0
Bacillus_clausii_KSM_K16	0	0	0	0	1000	0	1000	0	0	0
Bacillus_endophyticus_2102	0	0	1000	0	1000	0	1000	0	0	0
Bacillus_firmus_DS1	0	0	1000	0	1000	0	1000	0	0	0
Bacillus_fordii_DSM_16014	0	0	0	0	1000	0	1000	0	0	0
Bacillus_halodurans_C_125	0	0	0	0	1000	0	0	0	0	0
Bacillus_halotolerans_RRC_101	0	0	1000	0	1000	0	1000	0	0	0
Bacillus_infantis_NRRL_B_14911	0	0	1000	0	1000	0	1000	0	0	0
Bacillus_kwashiorkori_SIT6	0	0	0	0	1000	0	1000	1000	0	0
Bacillus_licheniformis_5NAP23	0	0	1000	0	1000	0	1000	0	0	0
Bacillus_licheniformis_ATCC_14580	0	0	0	0	1000	0	1000	0	0	0
Bacillus_licheniformis_ERR2221106	0	0	1000	0	1000	0	1000	0	0	0
Bacillus_licheniformis_ERR2230107	0	0	1000	0	1000	0	1000	0	0	0
Bacillus_licheniformis_ERR2230161	0	0	1000	0	1000	0	1000	0	0	0
Bacillus_licheniformis_HRBL_15TDI7	0	0	1000	0	1000	0	1000	0	0	0
Bacillus_massilioanorexius_AP8	0	0	1000	0	1000	0	1000	0	0	0
Bacillus_massiliosenegalensis_JC6	0	0	1000	0	1000	0	1000	0	0	0
Bacillus_megaterium_27Col1_1E	0	0	1000	0	1000	0	1000	0	0	1000
Bacillus_megaterium_DSM319	1000	0	1000	0	1000	0	1000	0	0	1000
Bacillus_megaterium_NBRC_15308_ATCC_14581	0	0	1000	0	1000	0	1000	0	0	0
Bacillus_megaterium_NCT_2	0	0	1000	0	1000	0	1000	0	0	0
Bacillus_megaterium_QM_B1551	636.3636364	0	1000	0	1000	0	1000	0	0	0
Bacillus_megaterium_SF185	0	0	1000	0	1000	0	1000	0	0	0
Bacillus_megaterium_WSH_002	750	0	1000	0	1000	0	1000	0	0	0
Bacillus_mojavensis_RO_H_1	0	0	1000	0	1000	0	1000	0	0	0
Bacillus_mycoides_AH603	0	0	1000	0	1000	0	1000	0	0	0
Bacillus_mycoides_AH621	0	0	1000	0	1000	0	1000	0	0	0
Bacillus_mycoides_BHP	0	0	1000	0	1000	0	1000	0	0	0
Bacillus_mycoides_BtB2_4	0	0	1000	0	1000	0	1000	0	0	0
Bacillus_mycoides_CER057	0	0	1000	0	1000	0	1000	0	0	0
Bacillus_mycoides_CER074	0	0	1000	0	1000	0	1000	0	0	0
Bacillus_mycoides_DSM_2048	0	0	0	0	1000	0	1000	0	0	0
Bacillus_mycoides_VD078	0	0	1000	0	1000	0	1000	0	0	0
Bacillus_mycoides_VDM019	0	0	1000	0	1000	0	1000	0	0	0
Bacillus_mycoides_VDM022	0	0	1000	0	1000	0	1000	0	0	0
Bacillus_nealsonii_AAU1	0	1000	1000	0	0	0	1000	0	0	1000
Bacillus_nov_ERR2221302	0	0	1000	0	1000	0	1000	0	0	0
Bacillus_paralicheniformis_ATCC_9945a	0	0	1000	0	1000	0	1000	0	0	0
Bacillus_pseudofirmus_OF4	0	0	0	0	1000	0	1000	0	0	1000
Bacillus_pseudomycoides_219298	0	0	1000	0	1000	0	1000	0	0	0
Bacillus_pseudomycoides_Rock1_4	0	0	1000	0	1000	0	1000	0	0	0
Bacillus_pseudomycoides_Rock3_17	0	0	1000	0	1000	0	1000	0	0	0
Bacillus_pumilus_ATCC_7061	0	0	0	0	1000	0	1000	0	0	0
Bacillus_pumilus_SAFR_032	0	0	1000	0	1000	0	1000	0	0	0
Bacillus_pumilus_TUAT1	0	0	1000	0	1000	0	1000	0	0	0
Bacillus_rubiinfantis_mt2	0	0	0	0	1000	0	1000	0	0	0
Bacillus_simplex_ERR2221243	0	0	1000	0	1000	0	1000	0	0	0
Bacillus_smithii_7_3_47FAA	0	0	1000	0	1000	0	1000	0	0	0
Bacillus_sonorensis_L12	0	0	0	0	1000	0	1000	0	0	0
Bacillus_sp_7_6_55CFAA_CT2	0	0	1000	0	1000	0	1000	0	0	0
Bacillus_subtilis_B_1	0	0	1000	0	1000	0	1000	0	0	0
Bacillus_subtilis_BSn5	0	0	1000	0	1000	0	1000	0	0	0
Bacillus_subtilis_ERR2221132	0	0	1000	0	1000	0	1000	0	0	0
Bacillus_subtilis_QB928	0	0	1000	0	1000	0	1000	0	0	0
Bacillus_subtilis_str_168	0	0	0	0	1000	0	1000	0	0	0
Bacillus_subtilis_subsp_natto_BEST195	0	0	1000	0	1000	0	1000	0	0	0
Bacillus_subtilis_subsp_spizizenii_ATCC_6633	0	0	1000	0	1000	0	1000	0	0	0
Bacillus_subtilis_subsp_spizizenii_DV1_B_1	0	0	1000	0	1000	0	1000	0	0	0
Bacillus_subtilis_subsp_spizizenii_str_W23	0	0	1000	0	1000	0	1000	0	0	0
Bacillus_subtilis_subsp_spizizenii_TU_B_10	0	0	0	0	1000	0	1000	0	0	0
Bacillus_subtilis_subsp_subtilis_516_BAMY_102_6	0	0	1000	0	1000	0	1000	0	0	0

Bacillus_subtilis_subsp_subtilis_6051_HGW	0	0	1000	0	1000	0	1000	0	0	0
Bacillus_subtilis_subsp_subtilis_str_BAB_1	0	0	1000	0	1000	0	1000	0	0	0
Bacillus_subtilis_subsp_subtilis_str_BSP1	0	0	1000	0	1000	0	1000	0	0	0
Bacillus_subtilis_subsp_subtilis_str_JH642	0	0	1000	0	1000	0	1000	0	0	0
Bacillus_subtilis_subsp_subtilis_str_NCIB_3610	0	0	1000	0	1000	0	1000	0	0	0
Bacillus_subtilis_subsp_subtilis_str_RO_NN_1	0	0	1000	0	1000	0	1000	0	0	0
Bacillus_subtilis_subsp_subtilis_str_SMY	0	0	1000	0	1000	0	1000	0	0	0
Bacillus_thermoamylovorans_1A1	0	0	1000	0	1000	0	1000	0	0	0
Bacillus_thuringiensis_BMB171	0	0	1000	0	1000	0	1000	0	0	0
Bacillus_thuringiensis_Bt407	538.4615385	0	1000	0	1000	0	1000	0	0	0
Bacillus_thuringiensis_ERR2230164	0	0	1000	0	1000	0	1000	0	0	0
Bacillus_thuringiensis_HD_771	625	0	1000	0	1000	0	1000	0	0	0
Bacillus_thuringiensis_HD_789	833.3333333	0	1000	0	1000	0	1000	0	0	0
Bacillus_thuringiensis_HD571	0	0	1000	0	1000	0	1000	0	0	0
Bacillus_thuringiensis_IBL_200	0	0	1000	0	1000	0	1000	0	0	0
Bacillus_thuringiensis_IBL_4222	0	0	1000	0	1000	0	1000	0	0	0
Bacillus_thuringiensis_MC28	625	0	1000	0	1000	0	1000	0	0	0
Bacillus_thuringiensis_serovar_andalousiensis_BG	0	0	1000	0	1000	0	1000	0	0	0
Bacillus_thuringiensis_serovar_berliner_ATCC_10	0	0	1000	0	1000	0	1000	0	0	0
Bacillus_thuringiensis_serovar_chinensis_CT_43	625	0	1000	0	1000	0	1000	0	0	0
Bacillus_thuringiensis_serovar_finitimus_YBT_020	625	0	1000	0	1000	0	1000	0	0	0
Bacillus_thuringiensis_serovar_huazhongensis_BG	0	0	1000	0	1000	0	1000	0	0	0
Bacillus_thuringiensis_serovar_konkukian_str_97_	0	0	1000	0	1000	0	1000	0	0	0
Bacillus_thuringiensis_serovar_kurstaki_str_HD73	0	0	1000	0	1000	0	1000	0	0	0
Bacillus_thuringiensis_serovar_kurstaki_str_T03a	0	0	1000	0	1000	0	1000	0	0	0
Bacillus_thuringiensis_serovar_monterrey_BGSC_	0	0	1000	0	1000	0	1000	0	0	0
Bacillus_thuringiensis_serovar_pakistani_str_T13	0	0	1000	0	1000	0	1000	0	0	0
Bacillus_thuringiensis_serovar_pondicheriensis_B	0	0	1000	0	1000	0	1000	0	0	0
Bacillus_thuringiensis_serovar_pulsiensis_BGSC_4	0	0	1000	0	1000	0	1000	0	0	0
Bacillus_thuringiensis_serovar_thuringiensis_str_	625	0	1000	0	1000	0	1000	0	0	0
Bacillus_thuringiensis_serovar_thuringiensis_str_	0	0	0	0	1000	0	1000	0	0	0
Bacillus_thuringiensis_serovar_tochigiensis_BGSC	0	0	1000	0	1000	0	1000	0	0	0
Bacillus_thuringiensis_str_Al_Hakam	833.3333333	0	1000	0	1000	0	1000	0	0	0
Bacillus_timonensis_10403023	0	0	1000	0	1000	0	1000	0	0	0
Bacillus_toyonensis_BAG1O_2	0	0	1000	0	1000	0	1000	0	0	0
Bacillus_toyonensis_HuB4_10	0	0	1000	0	1000	0	1000	0	0	0
Bacillus_vallismortis_DV1_F_3	0	0	1000	0	1000	0	1000	0	0	0
Bacillus_velezensis_AS433	0	0	1000	0	1000	0	1000	0	0	0
Bacillus_velezensis_L_H15	0	0	1000	0	1000	0	1000	0	0	0
Bacillus_velezensis_UCMB5036	0	0	1000	0	1000	0	1000	0	0	0
Bacillus_wiedmannii_BAG2X1_2	0	0	1000	0	1000	0	1000	0	0	0
Bacillus_wiedmannii_BAG5X2_1	0	0	1000	0	1000	0	1000	0	0	0
Bacillus_wiedmannii_BAG6X1_1	0	0	1000	0	1000	0	1000	0	0	0
Bacillus_wiedmannii_MM3	0	0	0	0	1000	0	1000	0	0	0
Bacteroidales_nov_ERR2221200	0	0	1000	0	0	0	0	0	0	0
Bacteroides_acidifaciens_1e8A	0	0	0	0	0	0	0	0	0	0
Bacteroides_acidifaciens_ERR2221207	0	0	0	0	0	0	1000	0	0	0
Bacteroides_acidifaciens_ERR2221208	0	0	1000	0	0	0	0	0	0	0
Bacteroides_acidifaciens_ERR2221211	0	0	0	0	0	0	0	0	0	0
Bacteroides_acidifaciens_JCM_10556	0	0	1000	0	0	0	1000	0	0	0
Bacteroides_barnesiae_DSM_18169_JCM_13652	0	0	1000	0	0	0	0	0	0	0
Bacteroides_caccae_ATCC_43185	0	0	0	1000	0	0	1000	0	0	0
Bacteroides_caccae_CL03T12C61	0	0	0	1000	0	0	1000	0	0	0
Bacteroides_caccae_ERR1022396	0	0	0	1000	0	0	1000	0	0	0
Bacteroides_caccae_ERR1022460	0	0	0	1000	0	0	0	0	0	0
Bacteroides_caccae_ERR2221107	0	0	0	1000	0	0	0	0	0	0
Bacteroides_caccae_ERR2221357	0	0	0	1000	0	0	0	0	0	0
Bacteroides_caecimuris_I48	0	0	1000	0	0	0	0	0	0	0
Bacteroides_cellulosilyticus_CL02T12C19	0	0	0	1000	0	0	1000	0	0	0
Bacteroides_cellulosilyticus_DSM_14838	0	0	0	1000	0	0	1000	0	0	0
Bacteroides_cellulosilyticus_ERR1022414	0	0	0	1000	0	0	0	0	0	0
Bacteroides_cellulosilyticus_ERR2221108	0	0	0	1000	0	0	0	0	0	0
Bacteroides_cellulosilyticus_ERR2221262	0	0	0	1000	0	0	0	0	0	0
Bacteroides_cellulosilyticus_ERR2221326	0	0	0	1000	0	0	0	0	0	0
Bacteroides_cellulosilyticus_ERR2230100	0	0	0	1000	0	0	0	0	0	0
Bacteroides_cellulosilyticus_WH2	0	0	0	1000	0	0	0	0	0	0
Bacteroides_clarus_ERR2221196	0	0	0	0	0	0	0	0	0	0
Bacteroides_clarus_ERR2221286	0	0	0	0	0	0	0	0	0	0
Bacteroides_clarus_ERR2230074	0	0	0	0	0	0	0	0	0	0
Bacteroides_clarus_YIT_12056	0	0	0	1000	0	0	1000	0	0	0
Bacteroides_coprocola_M16_DSM_17136	0	0	0	1000	0	0	1000	0	0	0
Bacteroides_coprophilus_DSM_18228	0	0	0	1000	0	0	1000	0	0	0
Bacteroides_coprosuis_DSM_18011	0	0	0	0	0	0	0	0	0	0
Bacteroides_dorei_CL02T00C15	0	0	1000	1000	1000	0	1000	0	0	0
Bacteroides_dorei_CL02T12C06	0	0	1000	1000	1000	0	1000	0	0	0
Bacteroides_dorei_CL03T12C01	0	0	1000	1000	1000	0	1000	0	0	0
Bacteroides_dorei_DSM_17855	0	0	0	1000	1000	0	1000	0	0	0
Bacteroides_dorei_ERR2221252	0	0	1000	0	1000	0	0	0	0	0
Bacteroides_dorei_ERR2230082	0	0	1000	0	1000	0	0	0	0	0
Bacteroides_eggerthii_1_2_48FAA	0	0	0	1000	0	0	1000	0	0	0
Bacteroides_eggerthii_DSM_20697	0	0	0	1000	0	0	1000	0	0	0
Bacteroides_faecis_ERR1022362	0	0	1000	0	0	0	0	0	0	0
Bacteroides_faecis_ERR1203949	0	0	1000	0	0	0	0	0	0	0
Bacteroides_faecis_ERR1204043	0	0	1000	0	0	0	0	0	0	0
Bacteroides_faecis_ERR2221133	0	0	1000	0	0	0	0	0	0	0
Bacteroides_faecis_MAJ27	0	0	0	1000	0	0	1000	0	0	0
Bacteroides_finegoldii_DSM_17565	0	0	0	1000	0	0	1000	0	0	0
Bacteroides_finegoldii_ERR1022298	0	0	0	0	0	0	0	0	0	0
Bacteroides_finegoldii_ERR1022319	0	0	0	0	0	0	0	0	0	0
Bacteroides_fluxus_YIT_12057	0	0	0	0	0	0	1000	1000	0	0
Bacteroides_fragilis_3_1_12	0	0	0	1000	0	0	1000	0	0	0
Bacteroides_fragilis_638R	0	0	0	0	0	0	1000	0	0	0
Bacteroides_fragilis_BOB25	0	0	1000	0	0	0	1000	0	0	0
Bacteroides_fragilis_CL03T00C08	0	0	1000	1000	0	0	1000	0	0	0

Bacteroides_fragilis_CL03T12C07	0	0	1000	1000	0	0	1000	0	0	0
Bacteroides_fragilis_CL05T00C42	0	0	1000	1000	0	0	1000	0	0	0
Bacteroides_fragilis_CL05T12C13	0	0	1000	1000	0	0	1000	0	0	0
Bacteroides_fragilis_CL07T00C01	0	0	1000	1000	0	0	1000	0	0	0
Bacteroides_fragilis_CL07T12C05	0	0	1000	1000	0	0	1000	0	0	0
Bacteroides_fragilis_ERR1022457	0	0	1000	0	0	0	1000	0	0	0
Bacteroides_fragilis_ERR1203962	0	0	1000	0	0	0	1000	0	0	0
Bacteroides_fragilis_ERR1204056	0	0	1000	0	0	0	1000	0	0	0
Bacteroides_fragilis_ERR2221134	0	0	1000	0	0	0	1000	0	0	0
Bacteroides_fragilis_ERR2221268	0	0	1000	0	0	0	1000	0	0	0
Bacteroides_fragilis_ERR2221298	0	0	1000	0	0	0	1000	0	0	0
Bacteroides_fragilis_ERR2230126	0	0	1000	0	0	0	1000	0	0	0
Bacteroides_fragilis_ERR2230137	0	0	1000	0	0	0	1000	0	0	0
Bacteroides_fragilis_HMW_610	0	0	1000	1000	0	0	1000	0	0	0
Bacteroides_fragilis_HMW_615	0	0	1000	1000	0	0	1000	0	0	0
Bacteroides_fragilis_HMW_616	0	0	1000	1000	0	0	1000	0	0	0
Bacteroides_fragilis_NCTC_9343	0	0	0	1000	0	0	1000	0	0	0
Bacteroides_fragilis_str_3397_T10	0	0	1000	0	0	0	1000	0	0	0
Bacteroides_fragilis_str_3986_T_B_9	0	0	1000	0	0	0	1000	0	0	0
Bacteroides_fragilis_str_DS_208	0	0	1000	0	0	0	1000	0	0	0
Bacteroides_fragilis_YCH46	0	0	0	1000	0	0	1000	0	0	0
Bacteroides_gallinarum_DSM_18171_JCM_13658	0	0	0	1000	0	0	0	0	0	0
Bacteroides_graminisolvens_DSM_19988	0	0	0	0	1000	0	1000	0	0	0
Bacteroides_helcogenes_P_36_108	0	0	0	0	0	0	0	0	0	0
Bacteroides_intestinalis_341_DSM_17393	0	0	0	1000	0	0	1000	0	0	0
Bacteroides_intestinalis_KLE1704	0	0	0	0	0	0	0	0	0	0
Bacteroides_luti_DSM_26991	0	0	0	0	0	0	0	0	0	0
Bacteroides_massiliensis_B84634	0	0	0	1000	0	0	1000	0	0	0
Bacteroides_neonati_MS4	0	0	1000	0	0	0	0	0	0	0
Bacteroides_nordii_CL02T12C05	0	0	0	0	0	0	1000	0	0	0
Bacteroides_nordii_ERR2221135	0	0	0	0	0	0	0	0	0	0
Bacteroides_nov_ERR1022359	0	0	1000	0	0	0	0	0	0	0
Bacteroides_nov_ERR1022361	0	0	0	0	0	0	0	0	0	0
Bacteroides_nov_ERR1022456	0	0	0	0	0	0	0	0	0	0
Bacteroides_nov_ERR2221137	0	0	1000	0	0	0	0	0	0	0
Bacteroides_nov_ERR2221300	0	0	1000	0	0	0	0	0	0	0
Bacteroides_nov_ERR2221375	0	0	1000	0	0	0	0	0	0	0
Bacteroides_nov_ERR2221405	0	0	1000	0	0	0	0	0	0	0
Bacteroides_oleiciplenus_YIT_12058	0	0	0	1000	1000	0	1000	1000	0	0
Bacteroides_ovatus_3_8_47FAA	0	0	1000	1000	0	0	1000	0	0	0
Bacteroides_ovatus_ATCC_8483	0	0	0	1000	0	0	1000	0	0	0
Bacteroides_ovatus_CL02T12C04	0	0	1000	1000	0	0	1000	0	0	0
Bacteroides_ovatus_CL03T12C18	0	0	1000	1000	0	0	1000	0	0	0
Bacteroides_ovatus_ERR1203959	0	0	1000	1000	0	0	1000	0	0	0
Bacteroides_ovatus_ERR1204053	0	0	1000	1000	0	0	1000	0	0	0
Bacteroides_ovatus_ERR2221136	0	0	1000	1000	0	0	1000	0	0	0
Bacteroides_ovatus_ERR2230063	0	0	1000	1000	0	0	1000	0	0	0
Bacteroides_ovatus_SD_CC_2a	0	0	0	1000	0	0	1000	1000	0	0
Bacteroides_ovatus_SD_CMC_3f	0	0	0	1000	0	0	1000	0	0	0
Bacteroides_pectinophilus_ATCC_43243	0	0	0	0	1000	0	1000	0	0	0
Bacteroides_plebeius_M12_DSM_17135	0	0	0	1000	0	0	1000	0	0	0
Bacteroides_propionicifaciens_DSM_19291_JCM_	0	0	0	0	0	0	0	0	1000	0
Bacteroides_pyogenes_DSM20611	0	0	0	0	0	0	1000	0	0	0
Bacteroides_pyogenes_F0041	0	0	0	0	0	0	0	0	0	0
Bacteroides_pyogenes_JCM_10003	0	0	0	0	0	0	0	0	0	0
Bacteroides_pyogenes_JCM_6292	0	0	0	0	0	0	0	0	0	0
Bacteroides_rodentium_JCM_16496	0	0	0	0	0	0	0	0	0	0
Bacteroides_salanitronis_DSM_18170	0	0	0	1000	0	0	0	0	0	0
Bacteroides_salyersiae_CL02T12C01	0	0	0	0	1000	0	1000	0	0	0
Bacteroides_salyersiae_ERR1022329	0	0	0	0	1000	0	0	0	0	0
Bacteroides_salyersiae_WAL_10018	0	0	0	0	0	0	1000	0	0	0
Bacteroides_sartorii_dnLKV3	0	0	1000	0	1000	0	0	0	0	0
Bacteroides_sartorii_JCM_17136	0	0	1000	0	1000	0	0	0	0	0
Bacteroides_sp_1_1_14	0	0	0	0	1000	0	1000	0	0	0
Bacteroides_sp_1_1_30	0	0	0	1000	0	0	1000	0	0	0
Bacteroides_sp_1_1_6	0	0	0	1000	0	0	1000	0	0	0
Bacteroides_sp_14_A	0	0	0	0	0	0	1000	0	0	0
Bacteroides_sp_2_1_22	0	0	0	1000	0	0	1000	0	0	0
Bacteroides_sp_2_1_33B	0	0	0	1000	0	0	1000	0	0	0
Bacteroides_sp_2_1_7	0	0	0	0	0	0	1000	0	0	0
Bacteroides_sp_2_2_4	0	0	0	1000	0	0	1000	0	0	0
Bacteroides_sp_20_3	0	0	0	1000	0	0	1000	0	0	0
Bacteroides_sp_3_1_19	0	0	0	1000	0	0	1000	0	0	0
Bacteroides_sp_3_1_23	0	0	0	1000	0	0	1000	0	0	0
Bacteroides_sp_3_1_33FAA	0	0	0	1000	1000	0	1000	0	0	0
Bacteroides_sp_3_1_40A	0	0	0	1000	1000	0	1000	0	0	0
Bacteroides_sp_3_2_5	0	0	0	1000	0	0	1000	0	0	0
Bacteroides_sp_4_1_36	0	0	0	1000	0	0	1000	0	0	0
Bacteroides_sp_4_3_47FAA	0	0	0	1000	1000	0	1000	0	0	0
Bacteroides_sp_9_1_42FAA	0	0	0	1000	1000	0	1000	0	0	0
Bacteroides_sp_D1	0	0	0	1000	0	0	1000	0	0	0
Bacteroides_sp_D2	0	0	0	1000	0	0	1000	0	0	0
Bacteroides_sp_D20	0	0	0	1000	0	0	1000	0	0	0
Bacteroides_sp_D22	0	0	0	1000	0	0	1000	0	0	0
Bacteroides_sp_HPS0048	0	0	0	0	0	0	0	0	0	0
Bacteroides_stercoris_ATCC_43183	0	0	0	0	0	0	1000	0	0	0
Bacteroides_stercoris_CC31F	0	0	0	0	0	0	1000	0	0	0
Bacteroides_stercoris_ERR1203945	0	0	0	0	0	0	0	0	0	0
Bacteroides_stercoris_ERR1204039	0	0	0	0	0	0	0	0	0	0
Bacteroides_stercoris_ERR2230062	0	0	0	0	0	0	0	0	0	0
Bacteroides_stercoris_ERR2230094	0	0	0	0	0	0	0	0	0	0
Bacteroides_stercoris_ERR2230103	0	0	0	0	0	0	0	0	0	0
Bacteroides_stercoris_ERR2230124	0	0	0	0	0	0	0	0	0	0
Bacteroides_stercoris_ERR2230142	0	0	0	0	0	0	0	0	0	0

Bacteroides_stercoris_ERR2230162	0	0	0	0	0	0	0	0	0	0
Bacteroides_thetaiotaomicron_3731	0	0	1000	1000	0	0	1000	0	0	0
Bacteroides_thetaiotaomicron_7330	0	0	1000	1000	0	0	1000	0	0	0
Bacteroides_thetaiotaomicron_CL09T03C10	0	0	0	1000	0	0	1000	0	0	0
Bacteroides_thetaiotaomicron_dnLKV9	0	0	1000	1000	0	0	1000	0	0	0
Bacteroides_thetaiotaomicron_ERR1022331	0	0	1000	1000	0	0	1000	0	0	0
Bacteroides_thetaiotaomicron_ERR1022413	0	0	1000	1000	0	0	1000	0	0	0
Bacteroides_thetaiotaomicron_ERR1022459	0	0	1000	1000	0	0	1000	0	0	0
Bacteroides_thetaiotaomicron_ERR2221264	0	0	1000	1000	0	0	1000	0	0	0
Bacteroides_thetaiotaomicron_ERR2221274	0	0	1000	1000	0	0	1000	0	0	0
Bacteroides_thetaiotaomicron_ERR2221312	0	0	1000	1000	0	0	1000	0	0	0
Bacteroides_thetaiotaomicron_ERR2230081	0	0	1000	1000	0	0	1000	0	0	0
Bacteroides_thetaiotaomicron_VPI_5482	0	0	1000	1000	0	0	1000	0	0	0
Bacteroides_timonensis_AP1	0	0	0	0	1000	0	1000	0	0	0
Bacteroides_uniformis_ATCC_8492	0	0	0	1000	0	0	1000	0	0	0
Bacteroides_uniformis_CL03T00C23	0	0	0	1000	0	0	1000	0	0	0
Bacteroides_uniformis_CL03T12C37	0	0	0	1000	0	0	1000	0	0	0
Bacteroides_uniformis_dnLKV2	0	0	0	1000	0	0	1000	0	0	0
Bacteroides_uniformis_ERR1022271	0	0	0	45.52352049	0	0	0	0	0	0
Bacteroides_uniformis_ERR1022322	0	0	0	45.52352049	0	0	0	0	0	0
Bacteroides_uniformis_ERR1022360	0	0	0	45.52352049	0	0	0	0	0	0
Bacteroides_uniformis_ERR1022363	0	0	0	45.52352049	0	0	1000	0	0	0
Bacteroides_uniformis_ERR1022412	0	0	0	45.52352049	0	0	1000	0	0	0
Bacteroides_uniformis_ERR1203944	0	0	0	45.52352049	0	0	0	0	0	0
Bacteroides_uniformis_ERR1204038	0	0	0	45.52352049	0	0	0	0	0	0
Bacteroides_uniformis_ERR2221138	0	0	0	45.52352049	0	0	1000	0	0	0
Bacteroides_uniformis_ERR2221253	0	0	0	45.52352049	0	0	1000	0	0	0
Bacteroides_uniformis_ERR2221261	0	0	0	45.52352049	0	0	1000	0	0	0
Bacteroides_uniformis_ERR2221281	0	0	0	45.52352049	0	0	0	0	0	0
Bacteroides_uniformis_ERR2230106	0	0	0	45.52352049	0	0	1000	0	0	0
Bacteroides_uniformis_ERR2230119	0	0	0	45.52352049	0	0	1000	0	0	0
Bacteroides_uniformis_ERR2230138	0	0	0	45.52352049	0	0	1000	0	0	0
Bacteroides_uniformis_ERR2230139	0	0	0	45.52352049	0	0	1000	0	0	0
Bacteroides_uniformis_ERR2230141	0	0	0	45.52352049	0	0	1000	0	0	0
Bacteroides_uniformis_ERR2230143	0	0	0	45.52352049	0	0	1000	0	0	0
Bacteroides_uniformis_ERR2230159	0	0	0	45.52352049	0	0	1000	0	0	0
Bacteroides_uniformis_ERR2230160	0	0	0	45.52352049	0	0	1000	0	0	0
Bacteroides_uniformis_str_3978_T3_i	0	0	0	45.52352049	0	0	0	0	0	0
Bacteroides_ureolyticus_DSM_20703	0	0	0	0	1000	0	1000	0	0	0
Bacteroides_vulgatus_ATCC_8482	0	0	1000	1000	1000	0	1000	0	0	0
Bacteroides_vulgatus_CL09T03C04	0	0	1000	1000	1000	0	1000	0	0	0
Bacteroides_vulgatus_dnLKV7	0	0	1000	1000	1000	0	1000	0	0	0
Bacteroides_vulgatus_ERR1022358	0	0	1000	0	1000	0	1000	0	0	0
Bacteroides_vulgatus_ERR1022411	0	0	1000	0	0	0	1000	0	0	0
Bacteroides_vulgatus_ERR1022458	0	0	1000	0	1000	0	1000	0	0	0
Bacteroides_vulgatus_ERR1203951	0	0	1000	0	1000	0	1000	0	0	0
Bacteroides_vulgatus_ERR1204045	0	0	1000	0	1000	0	1000	0	0	0
Bacteroides_vulgatus_ERR2221206	0	0	1000	0	1000	0	1000	0	0	0
Bacteroides_vulgatus_ERR2221254	0	0	1000	0	1000	0	1000	0	0	0
Bacteroides_vulgatus_ERR2221256	0	0	1000	0	1000	0	1000	0	0	0
Bacteroides_vulgatus_ERR2221259	0	0	1000	0	1000	0	1000	0	0	0
Bacteroides_vulgatus_ERR2221267	0	0	1000	0	1000	0	1000	0	0	0
Bacteroides_vulgatus_ERR2221272	0	0	1000	0	1000	0	1000	0	0	0
Bacteroides_vulgatus_ERR2221273	0	0	1000	0	0	0	1000	0	0	0
Bacteroides_vulgatus_ERR2221276	0	0	1000	0	0	0	1000	0	0	0
Bacteroides_vulgatus_ERR2230079	0	0	1000	0	1000	0	1000	0	0	0
Bacteroides_vulgatus_ERR2230086	0	0	1000	0	1000	0	1000	0	0	0
Bacteroides_vulgatus_ERR2230091	0	0	1000	0	0	0	1000	0	0	0
Bacteroides_vulgatus_ERR2230095	0	0	1000	0	0	0	1000	0	0	0
Bacteroides_vulgatus_ERR2230128	0	0	1000	0	1000	0	1000	0	0	0
Bacteroides_vulgatus_ERR2230134	0	0	1000	0	1000	0	1000	0	0	0
Bacteroides_vulgatus_ERR2230150	0	0	1000	0	0	0	1000	0	0	0
Bacteroides_vulgatus_mpk	0	0	1000	0	1000	0	1000	0	0	0
Bacteroides_vulgatus_PC510	0	0	1000	1000	1000	0	1000	0	0	0
Bacteroides_xylanisolvens_CL03T12C04	0	0	1000	1000	0	0	1000	1000	0	0
Bacteroides_xylanisolvens_ERR1022297	0	0	1000	0	1000	0	1000	0	0	0
Bacteroides_xylanisolvens_ERR2221139	0	0	1000	0	0	0	1000	1000	0	0
Bacteroides_xylanisolvens_ERR2221366	0	0	1000	0	0	0	1000	1000	0	0
Bacteroides_xylanisolvens_ERR2230096	0	0	1000	0	0	0	1000	1000	0	0
Bacteroides_xylanisolvens_ERR2230097	0	0	1000	0	0	0	1000	1000	0	0
Bacteroides_xylanisolvens_SD_CC_1b	0	0	0	1000	0	0	1000	1000	0	0
Bacteroides_xylanisolvens_XB1A	0	0	0	1000	0	0	1000	1000	0	0
Bacteroides_xylanolyticus_DSM_3808	0	0	0	0	1000	0	1000	0	0	0
Bacteroidetes_bacterium_oral_taxon_272_str_F0	0	0	0	0	0	0	0	0	0	0
Bacteroidetes_oral_taxon_274_str_F0058	0	0	0	0	0	0	1000	0	0	0
Barnesiella_intestinihominis_ERR1203957	0	0	0	0	0	0	0	0	0	0
Barnesiella_intestinihominis_ERR1204051	0	0	0	0	0	0	0	0	0	0
Barnesiella_intestinihominis_ERR2221140	0	0	0	0	0	0	0	0	0	0
Barnesiella_intestinihominis_ERR2230085	0	0	0	0	0	0	1000	0	0	0
Barnesiella_intestinihominis_YIT_11860	0	0	0	0	0	0	0	0	0	0
Barnesiella_viscericola_DSM_18177	0	0	0	0	0	0	0	0	0	0
Bartonella_quintana_RM_11	0	0	0	0	0	0	0	0	0	0
Bartonella_quintana_Toulouse	0	0	0	0	0	0	0	0	0	0
Bdellovibrio_bacteriovorus_HD100	117.6470588	0	1000	0	53.20723727	0	0	0	1000	0
Bdellovibrio_bacteriovorus_SSB218315	0	0	1000	0	52.05447909	0	0	0	1000	0
Bdellovibrio_bacteriovorus_str_Tiberius	0	0	1000	0	52.8533628	0	0	0	1000	0
Bdellovibrio_bacteriovorus_W	0	0	1000	0	47.50910216	0	0	0	1000	0
Bifidobacterium_adolescentis_ATCC_15703	0	0	0	45.52352049	0	0	1000	0	0	0
Bifidobacterium_adolescentis_BBMN23	0	0	0	45.52352049	0	0	1000	0	0	0
Bifidobacterium_adolescentis_DSM_20087	0	0	0	45.52352049	0	0	1000	0	0	0
Bifidobacterium_adolescentis_ERR1022283	0	0	0	45.52352049	0	0	1000	0	0	0
Bifidobacterium_adolescentis_ERR1022320	0	0	0	45.52352049	0	0	1000	0	0	0
Bifidobacterium_adolescentis_ERR1022366	0	0	0	45.52352049	0	0	1000	0	0	0
Bifidobacterium_adolescentis_ERR1203960	0	0	0	45.52352049	0	0	1000	0	0	0

Bifidobacterium_adolescentis_ERR2221193	0	0	0	45.52352049	0	0	1000	0	0	0
Bifidobacterium_adolescentis_ERR2221322	0	0	0	45.52352049	0	0	1000	0	0	0
Bifidobacterium_adolescentis_ERR2230053	0	0	0	45.52352049	0	0	1000	0	0	0
Bifidobacterium_adolescentis_ERR2230077	0	0	0	45.52352049	0	0	1000	0	0	0
Bifidobacterium_adolescentis_ERR2230153	0	0	0	45.52352049	0	0	1000	0	0	0
Bifidobacterium_adolescentis_L2_32	0	0	0	45.52352049	0	0	1000	0	0	0
Bifidobacterium_angulatum_DSM_20098	0	0	0	0	0	0	0	0	0	0
Bifidobacterium_angulatum_GT102	0	0	0	0	0	0	0	0	0	0
Bifidobacterium_animalis_ERR2221337	0	0	0	0	0	0	1000	0	0	0
Bifidobacterium_animalis_ERR2221385	0	0	0	0	0	0	1000	0	0	0
Bifidobacterium_animalis_lactis_AD011	0	0	0	0	0	0	1000	0	0	0
Bifidobacterium_animalis_lactis_BB_12	0	0	0	0	0	0	1000	0	0	0
Bifidobacterium_animalis_lactis_Bi_07	0	0	0	0	0	0	1000	0	0	0
Bifidobacterium_animalis_lactis_Bl_04_ATCC_SD5	0	0	0	0	0	0	1000	0	0	0
Bifidobacterium_animalis_lactis_CNCM_I_2494	0	0	0	0	0	0	1000	0	0	0
Bifidobacterium_animalis_lactis_DSM_10140	0	0	0	0	0	0	1000	0	0	0
Bifidobacterium_animalis_lactis_V9	0	0	0	0	0	0	1000	0	0	0
Bifidobacterium_animalis_RH	0	0	0	0	0	0	1000	0	0	0
Bifidobacterium_animalis_subsp_animalis_ATCC_	0	0	0	0	0	0	1000	0	0	0
Bifidobacterium_animalis_subsp_animalis_ATCC_	0	0	0	0	0	0	1000	0	0	0
Bifidobacterium_animalis_subsp_animalis_YL2	0	0	0	0	0	0	1000	0	0	0
Bifidobacterium_animalis_subsp_lactis_B420	0	0	0	0	0	0	1000	0	0	0
Bifidobacterium_animalis_subsp_lactis_BLC1	0	0	0	0	0	0	1000	0	0	0
Bifidobacterium_animalis_subsp_lactis_BS_01	0	0	0	0	0	0	1000	0	0	0
Bifidobacterium_animalis_subsp_lactis_HN019	0	0	0	0	0	0	1000	0	0	0
Bifidobacterium_asteroides_DSM_20089	0	0	0	0	1000	0	1000	0	0	0
Bifidobacterium_asteroides_Hma3	0	0	0	0	1000	0	0	0	0	0
Bifidobacterium_asteroides_PRL2011	0	0	0	0	1000	0	1000	0	0	0
Bifidobacterium_bifidum_156B	0	0	0	45.52352049	1000	0	1000	0	0	0
Bifidobacterium_bifidum_ATCC_29521	0	0	1000	45.52352049	1000	0	1000	0	0	0
Bifidobacterium_bifidum_BGN4	0	0	0	45.52352049	1000	0	1000	0	0	0
Bifidobacterium_bifidum_ERR1022335	0	0	1000	45.52352049	1000	0	1000	0	0	0
Bifidobacterium_bifidum_LMG_13195	0	0	1000	45.52352049	1000	0	1000	0	0	0
Bifidobacterium_bifidum_NCIMB_41171	0	0	0	45.52352049	0	0	1000	0	0	0
Bifidobacterium_bifidum_PRL2010	0	0	0	45.52352049	1000	0	1000	0	0	0
Bifidobacterium_bifidum_S17	0	0	0	45.52352049	1000	0	1000	0	0	0
Bifidobacterium_boum_DSM_20432	0	0	0	0	0	0	0	0	0	1000
Bifidobacterium_breve_12L	0	0	0	0	0	0	1000	0	0	0
Bifidobacterium_breve_2L	0	0	0	0	0	0	1000	0	0	0
Bifidobacterium_breve_689b	0	0	0	0	0	0	1000	0	0	0
Bifidobacterium_breve_ACS_071_V_Sch8b	0	0	0	0	0	0	1000	0	0	0
Bifidobacterium_breve_CECT_7263	0	0	0	0	0	0	1000	0	0	0
Bifidobacterium_breve_DPC_6330	0	0	0	0	0	0	1000	0	0	0
Bifidobacterium_breve_DSM_20213	0	0	0	0	0	0	1000	0	0	0
Bifidobacterium_breve_ERR2230051	0	0	1000	0	0	0	1000	0	0	0
Bifidobacterium_breve_HPH0326	0	0	0	0	0	0	1000	0	0	0
Bifidobacterium_breve_JCM_7017	0	0	0	0	0	0	1000	0	0	0
Bifidobacterium_breve_JCM_7019	0	0	0	0	0	0	1000	0	0	0
Bifidobacterium_breve_MCC_0121	0	0	0	0	0	0	1000	0	0	0
Bifidobacterium_breve_MCC_0305	0	0	0	0	0	0	1000	0	0	0
Bifidobacterium_breve_MCC_0476	0	0	0	0	0	0	1000	0	0	0
Bifidobacterium_breve_MCC_1094	0	0	0	0	0	0	1000	0	0	0
Bifidobacterium_breve_MCC_1114	0	0	0	0	0	0	1000	0	0	0
Bifidobacterium_breve_MCC_1128	0	0	0	0	0	0	1000	0	0	0
Bifidobacterium_breve_MCC_1340	0	0	0	0	0	0	1000	0	0	0
Bifidobacterium_breve_MCC_1454	0	0	0	0	0	0	1000	0	0	0
Bifidobacterium_breve_MCC_1604	0	0	0	0	0	0	1000	0	0	0
Bifidobacterium_breve_MCC_1605	0	0	0	0	0	0	1000	0	0	0
Bifidobacterium_breve_NCFB_2258	0	0	0	0	0	0	1000	0	0	0
Bifidobacterium_breve_UCC2003_NCIMB8807	0	0	0	0	0	0	1000	0	0	0
Bifidobacterium_catenulatum_DSM_16992	0	0	0	0	0	0	1000	0	0	0
Bifidobacterium_choerinum_DSM_20434	0	0	0	0	0	0	1000	0	0	0
Bifidobacterium_coryneforme_Bma6	0	0	0	0	1000	0	1000	0	0	0
Bifidobacterium_coryneforme_DSM_20216	0	0	0	0	1000	0	0	0	0	1000
Bifidobacterium_dentium_ATCC_27678	0	0	0	1000	0	0	1000	0	0	0
Bifidobacterium_dentium_ATCC_27679	0	0	0	1000	0	0	0	0	0	0
Bifidobacterium_dentium_Bd1	0	0	0	1000	0	0	0	0	0	0
Bifidobacterium_dentium_JCM_1195_DSM_20436	0	0	0	0	0	0	0	0	0	0
Bifidobacterium_dentium_JCVIHMP022	0	0	0	1000	0	0	0	0	0	0
Bifidobacterium_gallicum_DSM_20093	0	0	0	0	0	0	1000	0	0	0
Bifidobacterium_indicum_LMG_11587_DSM_202	0	0	0	0	1000	0	1000	0	0	0
Bifidobacterium_kashiwanohense_DSM_21854	0	0	0	0	0	0	1000	0	0	1000
Bifidobacterium_longum_BG7	0	0	0	45.52352049	0	0	1000	0	0	0
Bifidobacterium_longum_BXY01	0	0	0	45.52352049	0	0	1000	0	0	0
Bifidobacterium_longum_DJO10A	0	0	0	1000	0	0	1000	0	0	0
Bifidobacterium_longum_E18	0	0	1000	45.52352049	0	0	1000	0	0	0
Bifidobacterium_longum_ERR2221141	0	0	0	45.52352049	0	0	1000	0	0	0
Bifidobacterium_longum_ERR2221351	0	0	0	45.52352049	0	0	1000	0	0	0
Bifidobacterium_longum_ERR2221409	0	0	0	45.52352049	0	0	1000	0	0	0
Bifidobacterium_longum_ERR2230052	0	0	0	45.52352049	0	0	1000	0	0	0
Bifidobacterium_longum_ERR2230120	0	0	0	45.52352049	0	0	1000	0	0	0
Bifidobacterium_longum_ERR2230133	0	0	0	45.52352049	0	0	1000	0	0	0
Bifidobacterium_longum_ERR2230158	0	0	0	45.52352049	0	0	1000	0	0	0
Bifidobacterium_longum_infantis_157F_NC	0	0	0	0	0	0	1000	0	0	0
Bifidobacterium_longum_infantis_ATCC_15697	0	0	0	45.52352049	0	0	1000	1000	0	0
Bifidobacterium_longum_longum_ATCC_55813	0	0	0	45.52352049	0	0	1000	0	0	0
Bifidobacterium_longum_longum_BBMN68	0	0	0	45.52352049	0	0	1000	0	0	0
Bifidobacterium_longum_longum_CCUG_52486	0	0	0	1000	0	0	1000	0	0	0
Bifidobacterium_longum_longum_JCM_1217	0	0	0	1000	0	0	1000	0	0	0
Bifidobacterium_longum_longum_JDM301	0	0	0	1000	0	0	1000	0	0	0
Bifidobacterium_longum_NCC2705	0	0	0	45.52352049	0	0	1000	0	0	0
Bifidobacterium_longum_subsp_infantis_BT1	0	0	0	45.52352049	1000	0	1000	0	0	0
Bifidobacterium_longum_subsp_longum_1_6B	0	0	0	1000	0	0	1000	0	0	0
Bifidobacterium_longum_subsp_longum_2_2B	0	0	1000	45.52352049	0	0	1000	0	0	0

Bifidobacterium_longum_subsp_longum_35B	0	0	1000	45.52352049	0	0	1000	0	0	0
Bifidobacterium_longum_subsp_longum_44B	0	0	0	1000	0	0	1000	0	0	0
Bifidobacterium_longum_subsp_longum_CMCC_F	0	0	0	1000	0	0	1000	0	0	0
Bifidobacterium_longum_subsp_longum_F8	0	0	0	1000	0	0	1000	0	0	0
Bifidobacterium_longum_subsp_longum_KACC_9	0	0	0	1000	0	0	1000	0	0	0
Bifidobacterium_longum_subsp_longum_NCIMB8	0	0	0	45.52352049	0	0	1000	0	0	0
Bifidobacterium_mongoliense_DSM_21395	0	0	1000	0	0	0	1000	0	0	1000
Bifidobacterium_pseudocatenulatum_DSM_2043	0	0	0	0	0	0	0	0	0	0
Bifidobacterium_pseudocatenulatum_ERR102235	0	0	0	0	1000	0	1000	0	0	0
Bifidobacterium_pseudocatenulatum_ERR120397	0	0	0	0	1000	0	1000	0	0	0
Bifidobacterium_pseudocatenulatum_ERR120407	0	0	0	0	1000	0	1000	0	0	0
Bifidobacterium_pseudocatenulatum_ERR222114	0	0	0	0	1000	0	1000	0	0	0
Bifidobacterium_pseudocatenulatum_IPLA36007	0	0	0	0	0	0	1000	0	0	0
Bifidobacterium_pseudolongum_subsp_Pseudolon	0	0	1000	0	0	0	1000	0	0	1000
Bifidobacterium_pullorum_DSM_20433	0	0	0	0	1000	0	1000	0	0	0
Bifidobacterium_pullorum_LMG_21816	0	0	0	0	1000	0	1000	0	0	0
Bifidobacterium_ruminantium_DSM_6489	0	0	0	0	0	0	1000	0	0	1000
Bifidobacterium_scardovii_JCM_12489	0	0	1000	0	0	0	1000	0	0	1000
Bifidobacterium_sp_MSTE12	0	0	0	0	0	0	0	0	0	0
Bifidobacterium_stercoris_DSM_24849	0	0	0	0	0	0	1000	0	0	1000
Bifidobacterium_stercoris_ERR1204054	0	0	0	0	0	0	1000	0	0	0
Bifidobacterium_thermacidophilum_subsp_therm	0	0	0	0	0	0	0	0	0	1000
Bifidobacterium_thermophilum_RBL67	0	0	0	0	0	0	0	0	0	1000
Bilophila_sp_4_1_30	0	0	0	0	1000	0	1000	0	0	0
Bilophila_wadsworthia_3_1_6	0	0	0	0	1000	0	1000	0	1000	0
Bilophila_wadsworthia_ATCC_49260	0	0	0	0	1000	0	1000	0	1000	0
Bittarella_massiliensis_GD6	0	0	0	0	0	0	1000	0	0	0
Blastococcus_massiliensis_AP3	0	0	0	0	0	0	0	0	0	0
Blautia_coccoides_YL58	0	0	0	0	1000	0	1000	0	0	0
Blautia_faecis_ERR2221099	0	0	0	0	1000	0	0	0	0	0
Blautia_gnavus_ATCC_29149	0	0	0	0	1000	0	0	0	0	0
Blautia_gnavus_ERR1022310	0	0	0	0	1000	0	1000	0	0	0
Blautia_gnavus_ERR1203926	0	0	0	0	1000	0	1000	0	0	0
Blautia_gnavus_ERR171257	0	0	0	0	1000	0	1000	0	0	0
Blautia_gnavus_ERR2221233	0	0	0	0	1000	0	1000	0	0	0
Blautia_hansenii_VPI_C7_24_DSM_20583	0	0	0	0	1000	0	1000	0	0	0
Blautia_hydrogenotrophica_DSM_10507	0	0	0	0	1000	0	1000	0	0	0
Blautia_hydrogenotrophica_ERR1022315	0	0	0	0	1000	0	1000	0	0	0
Blautia_luti_DSM_14534	0	0	0	0	1000	0	0	0	0	0
Blautia_luti_ERR1022337	0	0	0	0	1000	0	0	0	0	0
Blautia_luti_ERR1022379	0	0	0	0	1000	0	0	0	0	0
Blautia_luti_ERR1022471	0	0	0	0	1000	0	0	0	0	0
Blautia_luti_ERR1203942	0	0	0	0	1000	0	0	0	0	0
Blautia_luti_ERR2221098	0	0	0	0	1000	0	0	0	0	0
Blautia_massiliensis_GD9	0	0	0	0	1000	0	1000	0	0	0
Blautia_nov_ERR1022275	0	0	0	0	1000	0	1000	0	0	0
Blautia_nov_ERR1022294	0	0	0	0	1000	0	1000	0	0	0
Blautia_nov_ERR1022295	0	0	0	0	1000	0	0	0	0	0
Blautia_nov_ERR1022302	0	0	0	0	1000	0	0	0	0	0
Blautia_nov_ERR1022306	0	0	0	0	1000	0	1000	0	0	0
Blautia_nov_ERR1022338	0	0	0	0	1000	0	1000	0	0	0
Blautia_nov_ERR1022339	0	0	0	0	1000	0	0	0	0	0
Blautia_nov_ERR1022340	0	0	0	0	1000	0	0	0	0	0
Blautia_nov_ERR1022378	0	0	0	0	0	0	1000	0	0	0
Blautia_nov_ERR1022424	0	0	0	0	1000	0	0	0	0	0
Blautia_nov_ERR1022426	0	0	0	0	1000	0	1000	0	0	0
Blautia_nov_ERR1022435	0	0	0	0	1000	0	0	0	0	0
Blautia_nov_ERR1203955	0	0	0	0	1000	0	0	0	0	0
Blautia_nov_ERR1204049	0	0	0	0	1000	0	0	0	0	0
Blautia_nov_ERR1204065	0	0	0	0	1000	0	1000	0	0	0
Blautia_nov_ERR2221143	0	0	0	0	1000	0	1000	0	0	0
Blautia_nov_ERR2221170	0	0	0	0	1000	0	1000	0	0	0
Blautia_nov_ERR2221260	0	0	0	0	1000	0	0	0	0	0
Blautia_nov_ERR2221265	0	0	0	0	1000	0	0	0	0	0
Blautia_nov_ERR2221269	0	0	0	0	1000	0	0	0	0	0
Blautia_nov_ERR2221270	0	0	0	0	1000	0	0	0	0	0
Blautia_nov_ERR2221279	0	0	0	0	1000	0	1000	0	0	0
Blautia_nov_ERR2221370	0	0	0	0	0	0	1000	0	0	0
Blautia_nov_ERR2221404	0	0	0	0	1000	0	1000	0	0	0
Blautia_nov_ERR2221410	0	0	0	0	1000	0	1000	0	0	0
Blautia_nov_ERR2230061	0	0	0	0	1000	0	1000	0	0	0
Blautia_nov_ERR2230064	0	0	0	0	1000	0	0	0	0	0
Blautia_nov_ERR2230066	0	0	0	0	1000	0	0	0	0	0
Blautia_nov_ERR2230067	0	0	0	0	1000	0	0	0	0	0
Blautia_nov_ERR2230071	0	0	0	0	1000	0	1000	0	0	0
Blautia_nov_ERR2230072	0	0	0	0	1000	0	0	0	0	0
Blautia_nov_ERR2230073	0	0	0	0	1000	0	1000	0	0	0
Blautia_nov_ERR2230075	0	0	0	0	1000	0	1000	0	0	0
Blautia_nov_ERR2230099	0	0	0	0	1000	0	0	0	0	0
Blautia_nov_ERR2230110	0	0	0	0	1000	0	0	0	0	0
Blautia_nov_ERR2230116	0	0	0	0	1000	0	1000	0	0	0
Blautia_nov_ERR2230130	0	0	0	0	1000	0	1000	0	0	0
Blautia_nov_ERR2230136	0	0	0	0	1000	0	0	0	0	0
Blautia_nov_ERR2230152	0	0	0	0	1000	0	0	0	0	0
Blautia_obeum_A2_162	0	0	0	0	1000	0	1000	0	0	0
Blautia_obeum_ATCC_29174	0	0	0	0	1000	0	0	0	0	0
Blautia_producta_DSM_2950	0	0	0	0	1000	0	1000	0	0	0
Blautia_producta_ERR171261	0	0	0	0	1000	0	1000	0	0	0
Blautia_producta_ERR2221380	0	0	0	0	1000	0	1000	0	0	0
Blautia_sp_YL58	0	0	0	0	1000	0	1000	0	0	0
Blautia_torques_ATCC_27756	0	0	0	0	0	0	1000	0	0	0
Blautia_torques_ERR1022291	0	0	0	0	1000	0	0	0	0	0
Blautia_torques_ERR1022304	0	0	0	0	0	0	1000	0	0	0
Blautia_torques_ERR1022325	0	0	0	0	1000	0	0	0	0	0

Blautia_torques_ERR1022357	0	0	0	0	0	0	1000	0	0	0
Blautia_torques_ERR1022404	0	0	0	0	1000	0	1000	0	0	0
Blautia_torques_ERR1022479	0	0	0	0	1000	0	0	0	0	0
Blautia_torques_ERR1203943	0	0	0	0	1000	0	0	0	0	0
Blautia_torques_ERR1203966	0	0	0	0	0	0	1000	0	0	0
Blautia_torques_ERR1204037	0	0	0	0	1000	0	0	0	0	0
Blautia_torques_ERR1204060	0	0	0	0	0	0	1000	0	0	0
Blautia_torques_ERR2221100	0	0	0	0	1000	0	0	0	0	0
Blautia_torques_ERR2221189	0	0	0	0	0	0	1000	0	0	0
Blautia_torques_ERR2230088	0	0	0	0	0	0	1000	0	0	0
Blautia_torques_ERR2230104	0	0	0	0	0	0	1000	0	0	0
Blautia_torques_L2_14	0	0	0	0	1000	0	1000	0	0	0
Blautia_wexlerae_AGR2146	0	0	0	0	1000	0	0	0	0	0
Blautia_wexlerae_DSM_19850	0	0	0	0	1000	0	0	0	0	0
Blautia_wexlerae_ERR1022296	0	0	0	0	1000	0	0	0	0	0
Blautia_wexlerae_ERR1022377	0	0	0	0	1000	0	0	0	0	0
Blautia_wexlerae_ERR1022425	0	0	0	0	1000	0	0	0	0	0
Blautia_wexlerae_ERR1204036	0	0	0	0	1000	0	0	0	0	0
Blautia_wexlerae_ERR2230049	0	0	0	0	1000	0	0	0	0	0
Bordetella_hinzii_F582	0	0	1000	0	1000	0	0	0	0	0
Bordetella_hinzii_OH87_BAL007II	0	0	0	0	1000	0	0	0	0	1000
Borkfalkia_ceftriaxoniphila_HDS1380	0	0	0	0	0	0	1000	0	0	0
Bosea_thiooxidans_DSM_9653	0	0	1000	0	1000	0	0	0	0	0
Brachybacterium_paraconglomeratum_LC44	0	0	0	0	1000	0	0	0	0	1000
Brachyspira_aalborgi_513A	0	0	0	0	1000	0	1000	0	0	0
Brachyspira_aalborgi_PC2022III	0	0	0	0	1000	0	1000	0	0	0
Brachyspira_aalborgi_PC2777IV	0	0	0	0	1000	0	1000	0	0	0
Brachyspira_aalborgi_PC3053II	0	0	0	0	1000	0	1000	0	0	0
Brachyspira_aalborgi_PC3517II	0	0	0	0	1000	0	1000	0	0	0
Brachyspira_aalborgi_PC3714II	0	0	0	0	1000	0	1000	0	0	0
Brachyspira_aalborgi_PC390II	0	0	0	0	1000	0	1000	0	0	0
Brachyspira_aalborgi_PC3939II	0	0	0	0	1000	0	1000	0	0	0
Brachyspira_aalborgi_PC3997IV	0	0	0	0	1000	0	1000	0	0	0
Brachyspira_aalborgi_PC4226IV	0	0	0	0	1000	0	1000	0	0	0
Brachyspira_aalborgi_PC4580III	0	0	0	0	1000	0	1000	0	0	0
Brachyspira_aalborgi_PC4597II	0	0	0	0	1000	0	1000	0	0	0
Brachyspira_aalborgi_PC5099IV	0	0	0	0	1000	0	1000	0	0	0
Brachyspira_aalborgi_PC5538III_lc	0	0	0	0	1000	0	1000	0	0	0
Brachyspira_aalborgi_PC5587_p	0	0	0	0	1000	0	1000	0	0	0
Brachyspira_aalborgi_PC5587_u	0	0	0	0	1000	0	1000	0	0	0
Brachyspira_aalborgi_W1	0	0	0	0	1000	0	1000	0	0	0
Brachyspira_pilosicoli_95_1000	0	0	0	0	1000	0	1000	0	1000	0
Brachyspira_pilosicoli_B2904	0	0	1000	1000	1000	0	1000	0	1000	0
Brachyspira_pilosicoli_P43_6_78	0	0	0	0	1000	0	1000	0	1000	0
Brachyspira_pilosicoli_WesB	0	0	0	1000	1000	0	1000	0	1000	0
Bradyrhizobium_elkanii_USDA_76	0	0	1000	0	0	0	0	0	0	1000
Bradyrhizobium_japonicum_E109	0	0	1000	0	0	0	0	0	0	0
Bradyrhizobium_japonicum_USDA_110	0	0	1000	0	1000	0	0	0	0	0
Bradyrhizobium_japonicum_USDA_124	0	0	1000	0	1000	0	0	0	0	0
Bradyrhizobium_japonicum_USDA_6	0	0	1000	0	0	0	0	0	0	1000
Bradyrhizobium_japonicum_WSM2793	0	0	1000	0	0	0	0	0	0	0
Bradyrhizobium_yuanmingense_BR3267	0	0	1000	0	0	0	0	1000	1000	0
Bradyrhizobium_yuanmingense_CCBAU_05623	0	0	1000	0	0	0	0	0	1000	0
Bradyrhizobium_yuanmingense_CCBAU_25021	0	0	1000	0	0	0	0	1000	1000	0
Bradyrhizobium_yuanmingense_CCBAU_35157	0	0	1000	0	0	0	0	1000	1000	0
Brevibacillus_agri_5_2	0	0	1000	0	1000	0	1000	0	0	0
Brevibacillus_agri_BAB_2500	0	0	0	0	1000	0	1000	0	0	0
Brevibacillus_borstelensis_AK1	0	0	1000	0	1000	0	1000	0	0	0
Brevibacillus_brevis_FJAT_0809_GLX	0	0	0	0	1000	0	1000	0	0	0
Brevibacillus_brevis_NBRC_100599	0	0	0	0	1000	0	1000	0	0	0
Brevibacterium_casei_S18	0	0	0	0	1000	0	0	0	0	0
Brevibacterium_linens_BL2	0	0	0	0	1000	0	0	0	0	0
Brevibacterium_massiliense_5401308	0	0	0	0	1000	0	1000	0	1000	0
Brevibacterium_senegalense_JC43	0	0	0	0	1000	0	0	0	0	0
Brevundimonas_bacteroides_DSM_4726	0	0	0	0	1000	0	0	0	1000	1000
Brevundimonas_diminuta_470_4	0	0	0	0	1000	0	1000	0	1000	1000
Brevundimonas_subvibrioides_ATCC_15264	0	0	0	0	1000	0	0	0	1000	0
Brochothrix_thermosphacta_DSM_20171	0	0	1000	0	1000	0	1000	0	0	0
Bulleidia_extructa_W1219	0	0	0	0	0	0	1000	0	0	0
Burkholderia_cenocepacia_AU_1054	0	0	1000	0	1000	0	0	0	1000	0
Burkholderia_cenocepacia_H111	0	0	1000	0	1000	0	0	0	1000	0
Burkholderia_cenocepacia_HI2424	0	0	1000	0	1000	0	0	0	1000	0
Burkholderia_cenocepacia_J2315	0	0	1000	0	1000	0	0	0	1000	0
Burkholderia_cenocepacia_MC0_3	0	0	1000	0	1000	0	0	0	1000	0
Burkholderia_cenocepacia_PC184	0	0	1000	0	1000	0	0	0	1000	0
Burkholderia_cepacia_GG4	0	0	1000	0	1000	0	1000	0	1000	1000
Burkholderia_cepacia_LO6	0	0	1000	0	1000	0	1000	0	1000	0
Burkholderiales_bacterium_1_1_47	0	0	0	0	1000	0	0	0	0	0
Burkholderiales_bacterium_YL45	0	0	0	0	1000	0	1000	0	0	0
Butyricicoccus_nov_ERR2221182	0	0	0	0	1000	0	1000	0	0	0
Butyricicoccus_pullicaecorum_1_2	0	0	0	0	1000	0	1000	0	0	0
Butyricimonas_synergistica_DSM_23225	0	0	0	0	1000	0	0	0	0	0
Butyricimonas_virosa_DSM_23226	0	0	1000	0	1000	0	1000	0	0	0
Butyricimonas_virosa_ERR2221144	0	0	0	0	1000	0	1000	0	0	0
Butyrivibrio_crossotus_DSM_2876	0	0	0	0	0	0	0	0	0	0
Butyrivibrio_fibrisolvens_16_4	0	0	0	0	0	0	0	0	0	0
Butyrivibrio_proteoclasticus_B316	0	0	0	0	0	0	0	0	0	0
Campylobacter_coli_1098	0	0	1000	0	1000	0	0	0	1000	0
Campylobacter_coli_111_3	0	0	1000	0	1000	0	0	0	1000	0
Campylobacter_coli_1148	0	0	1000	0	1000	0	0	0	1000	0
Campylobacter_coli_132_6	0	0	1000	0	1000	0	0	0	1000	0
Campylobacter_coli_1417	0	0	1000	0	1000	0	0	0	1000	0
Campylobacter_coli_14983A	0	0	1000	0	1000	0	0	0	1000	0
Campylobacter_coli_151_9	0	0	1000	0	1000	0	0	0	1000	0

Campylobacter_coli_1891	0	0	1000	0	1000	0	0	0	1000	0
Campylobacter_coli_1909	0	0	1000	0	1000	0	0	0	1000	0
Campylobacter_coli_1948	0	0	1000	0	1000	0	0	0	1000	0
Campylobacter_coli_1957	0	0	1000	0	1000	0	0	0	1000	0
Campylobacter_coli_1961	0	0	1000	0	1000	0	0	0	1000	0
Campylobacter_coli_202_04	0	0	1000	0	1000	0	0	0	1000	0
Campylobacter_coli_2548	0	0	1000	0	1000	0	0	0	1000	0
Campylobacter_coli_2553	0	0	1000	0	1000	0	0	0	1000	0
Campylobacter_coli_2680	0	0	1000	0	1000	0	0	0	1000	0
Campylobacter_coli_2685	0	0	1000	0	1000	0	0	0	1000	0
Campylobacter_coli_2698	0	0	1000	0	1000	0	0	0	1000	0
Campylobacter_coli_317_04	0	0	1000	0	1000	0	0	0	1000	0
Campylobacter_coli_37_05	0	0	1000	0	1000	0	0	0	1000	0
Campylobacter_coli_59_2	0	0	1000	0	1000	0	0	0	1000	0
Campylobacter_coli_67_8	0	0	1000	0	1000	0	0	0	1000	0
Campylobacter_coli_7_1	0	0	1000	0	1000	0	0	0	1000	0
Campylobacter_coli_80352	0	0	1000	0	1000	0	0	0	1000	0
Campylobacter_coli_84_2	0	0	1000	0	1000	0	0	0	1000	0
Campylobacter_coli_86119	0	0	1000	0	1000	0	0	0	1000	0
Campylobacter_coli_90_3	0	0	1000	0	1000	0	0	0	1000	0
Campylobacter_coli_BFR_CA_9557	0	0	1000	0	1000	0	0	0	1000	0
Campylobacter_coli_BIGS0003	0	0	1000	0	1000	0	0	0	1000	0
Campylobacter_coli_H56	0	0	1000	0	1000	0	0	0	1000	0
Campylobacter_coli_H6	0	0	1000	0	1000	0	0	0	1000	0
Campylobacter_coli_H8	0	0	1000	0	1000	0	0	0	1000	0
Campylobacter_coli_H9	0	0	1000	0	1000	0	0	0	1000	0
Campylobacter_coli_JV20	0	0	0	0	1000	0	0	0	1000	0
Campylobacter_coli_LMG_23336	0	0	1000	0	1000	0	0	0	1000	0
Campylobacter_coli_LMG_23341	0	0	1000	0	1000	0	0	0	1000	0
Campylobacter_coli_LMG_23342	0	0	1000	0	1000	0	0	0	1000	0
Campylobacter_coli_LMG_9853	0	0	1000	0	1000	0	0	0	1000	0
Campylobacter_coli_LMG_9854	0	0	1000	0	1000	0	0	0	1000	0
Campylobacter_coli_LMG_9860	0	0	1000	0	1000	0	0	0	1000	0
Campylobacter_coli_RM2228	0	0	1000	0	1000	0	0	0	1000	0
Campylobacter_coli_YH501	0	0	1000	0	1000	0	0	0	1000	0
Campylobacter_coli_Z156	0	0	1000	0	1000	0	0	0	1000	0
Campylobacter_coli_Z163	0	0	1000	0	1000	0	0	0	1000	0
Campylobacter_concisus_13826	0	0	1000	0	0	0	0	0	1000	0
Campylobacter_concisus_UNSWCD	0	0	1000	0	0	0	0	0	1000	0
Campylobacter_curvus_525_92	0	0	1000	0	1000	0	0	0	1000	0
Campylobacter_curvus_DSM_6644	0	0	1000	0	1000	0	0	0	1000	0
Campylobacter_fetus_subsp_fetus_006A_0073	0	0	1000	0	1000	0	0	0	0	0
Campylobacter_fetus_subsp_fetus_82_40	0	0	1000	0	0	0	1000	0	0	0
Campylobacter_fetus_subsp_venerealis_NCTC_10	0	0	1000	0	0	0	1000	0	0	0
Campylobacter_gracilis_RM3268	0	0	0	0	0	0	1000	0	0	0
Campylobacter_hominis_ATCC_BAA_381	0	0	1000	0	1000	0	1000	0	1000	0
Campylobacter_hyointestinalis_subsp_hyointestir	0	0	1000	0	1000	0	1000	0	0	0
Campylobacter_jejuni_CJM1cam	0	0	1000	0	0	0	0	0	1000	0
Campylobacter_jejuni_jejuni_81_176	0	0	1000	0	0	0	0	0	1000	0
Campylobacter_jejuni_jejuni_ICDCCJ07004	0	0	1000	0	0	0	0	0	1000	0
Campylobacter_jejuni_jejuni_M1	0	0	1000	0	0	0	0	0	1000	0
Campylobacter_jejuni_jejuni_NCTC_11168	0	0	1000	0	0	0	0	0	1000	0
Campylobacter_jejuni_NCTC11351	0	0	1000	0	0	0	0	0	1000	0
Campylobacter_jejuni_RM1221	0	0	1000	0	1000	0	0	0	1000	0
Campylobacter_jejuni_str_NCCP_No_15742	0	0	1000	0	1000	0	0	0	1000	0
Campylobacter_jejuni_subsp_doylei_269_97	0	0	1000	0	0	0	0	0	1000	0
Campylobacter_jejuni_subsp_jejuni_04197	0	0	1000	0	1000	0	0	0	1000	0
Campylobacter_jejuni_subsp_jejuni_04199	0	0	1000	0	1000	0	0	0	1000	0
Campylobacter_jejuni_subsp_jejuni_110_21	0	0	1000	0	1000	0	0	0	1000	0
Campylobacter_jejuni_subsp_jejuni_1213	0	0	1000	0	1000	0	0	0	1000	0
Campylobacter_jejuni_subsp_jejuni_129_258	0	0	1000	0	1000	0	0	0	1000	0
Campylobacter_jejuni_subsp_jejuni_1336	0	0	1000	0	1000	0	0	0	1000	0
Campylobacter_jejuni_subsp_jejuni_140_16	0	0	1000	0	1000	0	0	0	1000	0
Campylobacter_jejuni_subsp_jejuni_1798	0	0	1000	0	1000	0	0	0	1000	0
Campylobacter_jejuni_subsp_jejuni_1854	0	0	1000	0	1000	0	0	0	1000	0
Campylobacter_jejuni_subsp_jejuni_1893	0	0	1000	0	1000	0	0	0	1000	0
Campylobacter_jejuni_subsp_jejuni_1997_1	0	0	1000	0	1000	0	0	0	1000	0
Campylobacter_jejuni_subsp_jejuni_1997_11	0	0	1000	0	1000	0	0	0	1000	0
Campylobacter_jejuni_subsp_jejuni_1997_7	0	0	1000	0	1000	0	0	0	1000	0
Campylobacter_jejuni_subsp_jejuni_2008_1025	0	0	1000	0	1000	0	0	0	1000	0
Campylobacter_jejuni_subsp_jejuni_2008_831	0	0	1000	0	1000	0	0	0	1000	0
Campylobacter_jejuni_subsp_jejuni_2008_894	0	0	1000	0	0	0	0	0	1000	0
Campylobacter_jejuni_subsp_jejuni_2008_988	0	0	1000	0	1000	0	0	0	1000	0
Campylobacter_jejuni_subsp_jejuni_260_94	0	0	1000	0	1000	0	0	0	1000	0
Campylobacter_jejuni_subsp_jejuni_414	0	0	1000	0	0	0	0	0	1000	0
Campylobacter_jejuni_subsp_jejuni_51037	0	0	1000	0	0	0	0	0	1000	0
Campylobacter_jejuni_subsp_jejuni_53161	0	0	1000	0	1000	0	0	0	1000	0
Campylobacter_jejuni_subsp_jejuni_55037	0	0	1000	0	0	0	0	0	1000	0
Campylobacter_jejuni_subsp_jejuni_60004	0	0	1000	0	1000	0	0	0	1000	0
Campylobacter_jejuni_subsp_jejuni_6399	0	0	1000	0	1000	0	0	0	1000	0
Campylobacter_jejuni_subsp_jejuni_81116	0	0	1000	0	0	0	0	0	1000	0
Campylobacter_jejuni_subsp_jejuni_84_25	0	0	1000	0	1000	0	0	0	1000	0
Campylobacter_jejuni_subsp_jejuni_86605	0	0	1000	0	1000	0	0	0	1000	0
Campylobacter_jejuni_subsp_jejuni_87330	0	0	1000	0	1000	0	0	0	1000	0
Campylobacter_jejuni_subsp_jejuni_CF93_6	0	0	1000	0	1000	0	0	0	1000	0
Campylobacter_jejuni_subsp_jejuni_CG8421	0	0	1000	0	0	0	0	0	1000	0
Campylobacter_jejuni_subsp_jejuni_CG8486	0	0	1000	0	0	0	0	0	1000	0
Campylobacter_jejuni_subsp_jejuni_D2600	0	0	1000	0	0	0	0	0	1000	0
Campylobacter_jejuni_subsp_jejuni_DFVF1099	0	0	1000	0	1000	0	0	0	1000	0
Campylobacter_jejuni_subsp_jejuni_H22082	0	0	1000	0	1000	0	0	0	1000	0
Campylobacter_jejuni_subsp_jejuni_HB93_13	0	0	1000	0	1000	0	0	0	1000	0
Campylobacter_jejuni_subsp_jejuni_IA3902	0	0	1000	0	1000	0	0	0	1000	0
Campylobacter_jejuni_subsp_jejuni_ICDCCJ07001	0	0	1000	0	1000	0	0	0	1000	0
Campylobacter_jejuni_subsp_jejuni_ICDCCJ07002	0	0	1000	0	1000	0	0	0	1000	0

Campylobacter_jejuni_subsp_jejuni_LMG_23210	0	0	1000	0	0	0	0	0	1000	0
Campylobacter_jejuni_subsp_jejuni_LMG_23211	0	0	1000	0	0	0	0	0	1000	0
Campylobacter_jejuni_subsp_jejuni_LMG_23216	0	0	1000	0	1000	0	0	0	1000	0
Campylobacter_jejuni_subsp_jejuni_LMG_23218	0	0	1000	0	1000	0	0	0	1000	0
Campylobacter_jejuni_subsp_jejuni_LMG_23223	0	0	1000	0	1000	0	0	0	1000	0
Campylobacter_jejuni_subsp_jejuni_LMG_23264	0	0	1000	0	1000	0	0	0	1000	0
Campylobacter_jejuni_subsp_jejuni_LMG_23269	0	0	1000	0	0	0	0	0	1000	0
Campylobacter_jejuni_subsp_jejuni_LMG_23357	0	0	1000	0	1000	0	0	0	1000	0
Campylobacter_jejuni_subsp_jejuni_LMG_9081	0	0	1000	0	0	0	0	0	1000	0
Campylobacter_jejuni_subsp_jejuni_LMG_9217	0	0	1000	0	1000	0	0	0	1000	0
Campylobacter_jejuni_subsp_jejuni_LMG_9872	0	0	1000	0	1000	0	0	0	1000	0
Campylobacter_jejuni_subsp_jejuni_LMG_9879	0	0	1000	0	1000	0	0	0	1000	0
Campylobacter_jejuni_subsp_jejuni_NCTC_11168	0	0	1000	0	1000	0	0	0	1000	0
Campylobacter_jejuni_subsp_jejuni_NW	0	0	1000	0	1000	0	0	0	1000	0
Campylobacter_jejuni_subsp_jejuni_P110B	0	0	1000	0	1000	0	0	0	1000	0
Campylobacter_jejuni_subsp_jejuni_P854	0	0	1000	0	1000	0	0	0	1000	0
Campylobacter_jejuni_subsp_jejuni_PT14	0	0	1000	0	1000	0	0	0	1000	0
Campylobacter_jejuni_subsp_jejuni_RB922	0	0	1000	0	1000	0	0	0	1000	0
Campylobacter_jejuni_subsp_jejuni_S3	0	0	1000	0	1000	0	0	0	1000	0
Campylobacter_jejuni_subsp_jejuni_xy259	0	0	1000	0	1000	0	0	0	1000	0
Campylobacter_lari_RM2100	0	0	0	0	0	0	0	0	0	0
Campylobacter_rectus_RM3267	0	0	1000	0	0	0	1000	0	0	0
Campylobacter_showae_CSUNSWCD	0	0	1000	0	1000	0	1000	0	0	0
Campylobacter_showae_RM3277	0	0	1000	0	1000	0	1000	0	0	0
Campylobacter_sp_10_1_50	0	0	1000	0	0	0	1000	0	0	0
Campylobacter_sputorum_bv_faecalis_CCUG_207	0	0	1000	0	1000	0	1000	0	0	0
Campylobacter_troglodytis_MIT_05_9149A	0	0	1000	0	0	0	0	0	0	0
Campylobacter_upsaliensis_JV21	0	0	0	0	0	0	0	0	1000	0
Campylobacter_upsaliensis_RM3195	0	0	1000	0	1000	0	0	0	1000	0
Campylobacter_ureolyticus_ACS_301_V_Sch3b	0	0	1000	0	1000	0	1000	0	0	0
Campylobacter_ureolyticus_CIT007	0	0	1000	0	0	0	1000	0	0	0
Campylobacter_ureolyticus_RIGS_9880	0	0	1000	0	1000	0	1000	0	0	0
Campylobacter_ureolyticus_UMB0112	0	0	1000	0	1000	0	1000	0	0	0
Candidatus_Dorea_massiliensis_AP6	0	0	0	0	1000	0	1000	0	0	0
Candidatus_Nitrososphaera_gargensis_Ga9_2	0	0	1000	0	0	0	0	0	1000	0
Candidatus_Pelagibacter_sp_IMCC9063	0	0	0	0	0	0	0	0	0	0
Candidatus_Saccharimonas_aalborgensis	0	0	0	0	500	0	500	0	0	0
Candidatus_Soleaferrea_massiliensis_AP7	0	0	0	0	0	0	428.5714286	0	0	0
Capnocytophaga_granulosa_ATCC_51502	0	0	0	0	0	0	0	0	0	0
Capnocytophaga_leadbetteri_DSM_22902	0	0	0	0	0	0	1000	0	0	0
Capnocytophaga_ochracea_DSM_7271	0	0	0	0	1000	0	0	0	0	0
Capnocytophaga_ochracea_F0287	0	0	0	0	1000	0	1000	0	0	0
Capnocytophaga_ochracea_str_Holt_25	0	0	0	0	1000	0	1000	0	0	0
Capnocytophaga_sp_CM59	0	0	0	0	1000	0	1000	0	0	0
Capnocytophaga_sp_oral_taxon_326_str_F0382	0	0	0	0	1000	0	1000	0	0	0
Capnocytophaga_sp_oral_taxon_329_str_F0087	0	0	0	1000	1000	0	1000	0	0	0
Capnocytophaga_sp_oral_taxon_332_str_F0381	0	0	0	0	1000	0	1000	0	0	0
Capnocytophaga_sp_oral_taxon_335_str_F0486	0	0	0	0	1000	0	1000	0	0	0
Capnocytophaga_sp_oral_taxon_336_str_F0502	0	0	0	0	1000	0	1000	0	0	0
Capnocytophaga_sp_oral_taxon_338_str_F0234	0	0	0	0	1000	0	0	0	0	0
Capnocytophaga_sp_oral_taxon_412_str_F0487	0	0	0	0	1000	0	1000	0	0	0
Capnocytophaga_sputigena_ATCC_33612	0	0	0	0	0	0	0	0	0	0
Cardiobacterium_hominis_612_ETAR_1001_5485	0	0	0	0	0	0	1000	0	0	0
Cardiobacterium_valvarum_F0432	0	0	0	0	1000	0	1000	0	0	0
Carnobacterium_maltaromaticum_ATCC_35586	0	0	0	0	1000	0	1000	0	0	0
Carnobacterium_maltaromaticum_DSM_20342	0	0	0	0	1000	0	1000	0	0	0
Carnobacterium_maltaromaticum_DSM_20722	0	0	0	0	1000	0	1000	0	0	0
Carnobacterium_maltaromaticum_LMA28	0	0	0	0	1000	0	0	0	0	0
Catabacter_hongkongensis_HKU16	0	0	0	0	0	0	0	0	0	0
Catenibacterium_mitsuokai_DSM_15897	0	0	0	0	0	0	0	0	0	0
Catenibacterium_mitsuokai_ERR1022284	0	0	0	0	0	0	0	0	0	0
Catenibacterium_mitsuokai_ERR2221198	0	0	0	0	0	0	0	0	0	0
Catonella_morbi_ATCC_51271	0	0	0	0	0	0	1000	0	0	0
Cedecea_davisae_DSM_4568	0	0	0	0	1000	0	1000	0	0	1000
Cellulomonas_massiliensis_JC225	0	0	0	0	0	0	1000	0	0	0
Cellulomonas_massiliensis_MGYG_HGUT_01416	0	0	0	0	0	0	1000	0	0	0
Cellulosilyticum_lentocellum_DSM_5427	0	0	0	0	1000	0	1000	0	1000	0
Cellulosimicrobium_cellulans_J36	0	0	0	0	1000	0	0	0	1000	1000
Centipeda_periodontii_DSM_2778	0	0	0	0	0	0	1000	0	0	0
Cetobacterium_somerae_ATCC_BAA_47401	0	0	0	0	1000	0	1000	0	1000	0
Chlorobium_phaeobacteroides_BS1	0	0	0	0	1000	0	0	0	0	0
Chlorobium_phaeobacteroides_DSM_266	0	0	1000	0	0	0	0	0	0	0
Christensenella_massiliensis_Marseille_P2438	0	0	0	0	0	0	1000	0	0	0
Christensenella_minuta_DSM_22607	0	0	0	0	1000	0	1000	0	0	0
Christensenellaceae_nov_ERR1022455	0	0	0	0	0	0	1000	0	0	0
Citrobacter_amalonaticus_FDAARGOS_122	0	0	1000	0	1000	0	1000	0	0	0
Citrobacter_amalonaticus_FDAARGOS_166	0	0	1000	0	1000	0	1000	0	0	0
Citrobacter_amalonaticus_Y19	0	0	1000	0	1000	0	1000	0	0	1000
Citrobacter_amalonaticus_YG6	0	0	1000	0	1000	0	1000	0	0	0
Citrobacter_amalonaticus_YG8	0	0	1000	0	1000	0	1000	0	0	0
Citrobacter_freundii_4_7_47CFAA	0	0	1000	0	1000	0	1000	0	0	0
Citrobacter_freundii_ATCC_8090	0	0	0	0	1000	0	1000	0	0	1000
Citrobacter_freundii_ERR2221339	0	0	1000	0	1000	0	1000	0	0	0
Citrobacter_freundii_FDAARGOS_61	0	0	1000	0	1000	0	1000	0	0	0
Citrobacter_freundii_FDAARGOS_73	0	0	1000	0	1000	0	1000	0	0	0
Citrobacter_freundii_GTC_09479	0	0	1000	0	1000	0	1000	0	0	0
Citrobacter_freundii_GTC_09629	0	0	1000	0	1000	0	1000	0	0	0
Citrobacter_freundii_str_ballerup_7851_39	0	0	1000	0	1000	0	1000	0	0	0
Citrobacter_freundii_UCI_31	0	0	0	0	1000	0	1000	0	0	1000
Citrobacter_koseri_ATCC_BAA_895	1000	0	0	0	1000	0	1000	0	0	0
Citrobacter_koseri_FDAARGOS_164	0	0	1000	0	1000	0	1000	0	0	0
Citrobacter_nov_ERR2221352	0	0	1000	0	1000	0	1000	0	0	0
Citrobacter_portucalensis_P10159	0	0	1000	0	1000	0	1000	0	0	0
Citrobacter_rodentium_ICC168	1000	0	1000	0	1000	0	1000	0	0	0

Citrobacter_sp_30_2	0	0	0	0	1000	0	1000	0	0	0
Citrobacter_sp_A1	0	0	1000	0	1000	0	1000	0	0	0
Citrobacter_sp_KTE151	0	0	1000	0	1000	0	1000	0	0	0
Citrobacter_sp_KTE30	0	0	1000	0	1000	0	1000	0	0	0
Citrobacter_sp_KTE32	0	0	1000	0	1000	0	1000	0	0	0
Citrobacter_sp_L17	0	0	1000	0	1000	0	1000	0	0	0
Citrobacter_youngae_ATCC_29220	0	0	0	1000	1000	0	1000	0	0	0
Cloacibacillus_evryensis_DSM_19522	0	0	1000	0	1000	0	0	0	0	0
Cloacibacterium_normanense_DSM_15886	0	0	0	0	1000	0	0	0	0	0
Clostridiaceae_nov_ERR1022373	0	0	0	0	1000	0	1000	0	0	0
Clostridiaceae_nov_ERR2221146	0	0	1000	0	1000	0	1000	0	0	0
Clostridiaceae_nov_ERR2221328	0	0	0	0	1000	0	1000	0	0	0
Clostridiaceae_nov_ERR2221330	0	0	0	0	1000	0	1000	0	0	0
Clostridiaceae_nov_ERR2221331	0	0	0	0	1000	0	1000	0	0	0
Clostridiaceae_nov_ERR2221345	0	0	1000	0	0	0	1000	0	0	0
Clostridiaceae_nov_ERR2221361	0	0	1000	0	0	0	1000	0	0	0
Clostridiaceae_nov_ERR2221383	0	0	0	0	1000	0	1000	0	0	0
Clostridiaceae_nov_ERR2230154	0	0	0	0	1000	0	1000	0	0	0
Clostridiales_incertae_sedis_nov_ERR1022334	0	0	0	0	0	0	1000	0	0	0
Clostridiales_incertae_sedis_nov_ERR1022419	0	0	0	0	1000	0	1000	0	0	0
Clostridiales_incertae_sedis_nov_ERR2221176	0	0	0	0	0	0	1000	0	0	0
Clostridiales_incertae_sedis_nov_ERR2221277	0	0	0	0	1000	0	1000	0	0	0
Clostridiales_incertae_sedis_nov_ERR2221283	0	0	0	0	0	0	1000	0	0	0
Clostridiales_incertae_sedis_nov_ERR2221367	0	0	0	0	1000	0	1000	0	0	0
Clostridiales_incertae_sedis_nov_ERR2221388	0	0	0	0	0	0	1000	0	0	0
Clostridiales_sp_1_7_47FAA	0	0	0	0	1000	0	1000	0	0	0
Clostridioides_difficile_002_P50_2011	0	0	0	0	1000	1000	1000	0	1000	0
Clostridioides_difficile_050_P50_2011	0	0	0	0	1000	1000	1000	0	1000	0
Clostridioides_difficile_2007855	0	0	0	0	1000	1000	1000	0	1000	0
Clostridioides_difficile_630	1000	0	0	0	1000	1000	1000	0	1000	0
Clostridioides_difficile_6503	0	0	0	0	1000	1000	1000	0	1000	0
Clostridioides_difficile_70_100_2010	0	0	0	0	1000	1000	1000	0	1000	0
Clostridioides_difficile_ATCC_43255	0	0	0	0	1000	1000	1000	0	1000	0
Clostridioides_difficile_ATCC_9689	0	0	0	0	1000	1000	1000	0	1000	0
Clostridioides_difficile_BI1	1000	0	0	0	1000	1000	1000	0	1000	0
Clostridioides_difficile_CD196	1000	0	0	0	1000	1000	1000	0	1000	0
Clostridioides_difficile_CD37	0	0	0	0	1000	1000	1000	0	1000	0
Clostridioides_difficile_CIP_107932	0	0	0	0	1000	1000	1000	0	1000	0
Clostridioides_difficile_ERR1204032	0	0	0	0	1000	1000	1000	0	1000	0
Clostridioides_difficile_ERR2221119	0	0	0	0	1000	1000	1000	0	1000	0
Clostridioides_difficile_ERR2221219	0	0	0	0	1000	1000	1000	0	1000	0
Clostridioides_difficile_ERR2221225	0	0	0	0	1000	1000	1000	0	1000	0
Clostridioides_difficile_M120	0	0	0	0	1000	1000	1000	0	1000	0
Clostridioides_difficile_M68	0	0	0	0	1000	1000	1000	0	1000	0
Clostridioides_difficile_NAP07	0	0	0	0	1000	1000	1000	0	1000	0
Clostridioides_difficile_NAP08	0	0	0	0	1000	1000	1000	0	1000	0
Clostridioides_difficile_QCD_23m63	0	0	0	0	1000	1000	1000	0	1000	0
Clostridioides_difficile_QCD_37x79	0	0	0	0	1000	1000	1000	0	1000	0
Clostridioides_difficile_QCD_63q42	0	0	0	0	1000	1000	1000	0	1000	0
Clostridioides_difficile_QCD_66c26	0	0	0	0	1000	1000	1000	0	1000	0
Clostridioides_difficile_QCD_76w55	0	0	0	0	1000	1000	1000	0	1000	0
Clostridioides_difficile_QCD_97b34	0	0	0	0	1000	1000	1000	0	1000	0
Clostridioides_difficile_R20291	1000	0	0	0	1000	1000	1000	0	1000	0
Clostridioides_mangenotii_LM2	0	0	0	0	1000	0	1000	0	1000	0
Clostridioides_mangenotii_TR	0	0	0	0	1000	0	1000	0	1000	0
Clostridium_acetobutylicum_ATCC_824	0	0	0	0	1000	0	1000	0	0	0
Clostridium_acetobutylicum_DSM_1731	0	0	1000	0	1000	0	1000	0	0	0
Clostridium_acetobutylicum_EA_2018	0	0	1000	0	1000	0	1000	0	0	0
Clostridium_aerotolerans_DSM_5434	0	0	0	0	0	0	1000	0	0	0
Clostridium_amazonitimonense_MGYG_HGUT_01	0	0	0	0	1000	0	1000	0	0	0
Clostridium_asparagiforme_DSM_15981	0	0	0	1000	1000	0	1000	0	0	0
Clostridium_baratii_796_15	0	0	0	0	1000	0	1000	0	0	0
Clostridium_baratii_ERR1022470	0	0	0	0	1000	0	1000	0	0	0
Clostridium_baratii_str_Sullivan	0	0	0	0	1000	0	1000	0	0	0
Clostridium_bartlettii_DSM_16795	0	0	0	0	1000	0	1000	0	1000	0
Clostridium_beijerinckii_G117	0	0	1000	0	1000	0	1000	0	0	0
Clostridium_beijerinckii_NCIMB_8052	0	0	0	0	1000	0	1000	0	0	0
Clostridium_boliviensis_ERR171272	0	0	0	0	1000	0	1000	0	0	0
Clostridium_bolteae_90A5	0	0	0	0	1000	0	1000	0	0	0
Clostridium_bolteae_90A9	0	0	0	0	1000	0	1000	0	0	0
Clostridium_bolteae_90B3	0	0	0	0	1000	0	1000	0	0	0
Clostridium_bolteae_90B7	0	0	0	0	1000	0	1000	0	0	0
Clostridium_bolteae_90B8	0	0	0	0	1000	0	1000	0	0	0
Clostridium_bolteae_ATCC_BAA_613	0	0	1000	0	1000	0	1000	0	0	0
Clostridium_botulinum_A_str_ATCC_19397	0	0	0	0	1000	1000	1000	0	0	0
Clostridium_botulinum_A_str_ATCC_3502	0	0	0	0	1000	1000	1000	0	0	0
Clostridium_botulinum_A_str_Hall	0	0	0	0	1000	1000	1000	0	0	0
Clostridium_botulinum_A2_str_Kyoto	0	0	1000	0	1000	0	1000	0	0	0
Clostridium_botulinum_A3_str_Loch_Maree	0	0	1000	0	1000	0	1000	0	0	0
Clostridium_botulinum_B_str_Eklund_17B	1000	0	1000	0	1000	0	1000	0	0	0
Clostridium_botulinum_B1_str_Okra	0	0	1000	0	1000	0	1000	0	0	0
Clostridium_botulinum_Ba4_str_657	0	0	1000	0	1000	0	1000	0	0	0
Clostridium_botulinum_Bf	0	0	0	0	1000	1000	1000	0	0	0
Clostridium_botulinum_BKT015925	0	0	0	0	1000	0	1000	0	0	0
Clostridium_botulinum_C_str_Eklund	0	0	0	0	1000	0	1000	0	0	0
Clostridium_botulinum_CB11_1_1	0	0	0	0	1000	0	1000	0	0	0
Clostridium_botulinum_CDC66177	0	0	1000	0	1000	0	1000	0	0	0
Clostridium_botulinum_D_str_1873	0	0	0	0	1000	0	1000	0	0	0
Clostridium_botulinum_E1_str_BoNT_E_Beluga	0	0	0	0	1000	0	1000	0	0	0
Clostridium_botulinum_E3_str_Alaska_E43	1000	0	0	0	1000	0	1000	0	0	0
Clostridium_botulinum_F_str_230613	0	0	0	0	1000	1000	1000	0	0	0
Clostridium_botulinum_F_str_Langeland	0	0	1000	0	1000	0	1000	0	0	0
Clostridium_botulinum_H04402_065	0	0	0	0	1000	0	1000	0	0	0
Clostridium_botulinum_NCTC_2916	0	0	1000	0	1000	0	1000	0	0	0

Clostridium_butyricum_5521	0	0	1000	1000	1000	0	1000	0	0	0
Clostridium_butyricum_60E_3	0	0	1000	1000	1000	0	1000	0	0	0
Clostridium_butyricum_DKU_01	0	0	1000	1000	1000	0	1000	0	0	0
Clostridium_butyricum_DSM_10702	0	0	0	0	1000	0	1000	0	0	0
Clostridium_butyricum_E4_str_BoNT_E_BL5262	0	0	0	1000	1000	0	1000	0	0	0
Clostridium_cadaveris_AGR2141	0	0	0	0	1000	0	1000	0	0	0
Clostridium_celatum_DSM_1785	0	0	0	1000	1000	0	1000	0	0	0
Clostridium_cellobioparum_DSM_1351	0	0	0	0	0	0	1000	0	0	0
Clostridium_chauvoei_JF4335	0	0	0	0	1000	0	1000	0	0	0
Clostridium_citroniae_WAL_17108	0	0	0	1000	1000	0	1000	0	0	0
Clostridium_clariflavum_DSM_19732	0	0	0	0	0	0	1000	0	0	0
Clostridium_clostridioforme_2_1_49FAA	0	0	0	1000	1000	0	1000	0	0	0
Clostridium_clostridioforme_90A1	0	0	0	1000	1000	0	1000	0	0	0
Clostridium_clostridioforme_90A3	0	0	0	1000	1000	0	1000	0	0	0
Clostridium_clostridioforme_90A4	0	0	0	1000	1000	0	1000	0	0	0
Clostridium_clostridioforme_90A6	0	0	0	1000	1000	0	1000	0	0	0
Clostridium_clostridioforme_90A7	0	0	0	0	1000	0	1000	0	0	0
Clostridium_clostridioforme_90A8	0	0	0	1000	1000	0	1000	0	0	0
Clostridium_clostridioforme_90B1	0	0	0	1000	1000	0	1000	0	0	0
Clostridium_clostridioforme_CM201	0	0	0	1000	1000	0	1000	0	0	0
Clostridium_clostridioforme_YL32	0	0	0	0	1000	0	1000	0	0	0
Clostridium_cochlearium_NCTC13027	0	0	0	0	1000	0	0	0	1000	0
Clostridium_cocleatum_ATCC_29902	0	0	0	0	1000	0	1000	0	0	0
Clostridium_colicanis_209318	0	0	0	1000	0	0	1000	0	0	0
Clostridium_colicanis_DSM_13634	0	0	0	115.3846154	1000	0	1000	0	0	0
Clostridium_culturomicsense_CL_6	0	0	0	0	1000	0	1000	0	0	0
Clostridium_dakarense_FF1	0	0	0	0	1000	0	1000	0	0	0
Clostridium_disporicum_2789STDY5608827	0	0	1000	0	1000	0	1000	0	0	0
Clostridium_fimetarium_DSM_9179	0	0	0	0	0	0	1000	0	0	0
Clostridium_glycolicum_ATCC_14880	0	0	1000	0	1000	0	1000	0	0	0
Clostridium_hathewayi_12489931	0	0	0	1000	1000	0	1000	0	0	0
Clostridium_hathewayi_DSM_13479	0	0	0	0	1000	0	1000	0	0	0
Clostridium_hathewayi_WAL_18680	0	0	0	0	1000	0	1000	0	0	0
Clostridium_hiranonis_TO_931_DSM_13275	0	0	0	0	0	0	0	0	0	0
Clostridium_hylemonae_DSM_15053	0	0	0	0	1000	0	1000	0	0	0
Clostridium_ihumii_AP5	0	0	0	0	1000	0	1000	0	0	0
Clostridium_indolis_DSM_755	0	0	1000	0	1000	0	1000	0	0	0
Clostridium_innocuum_2959	0	0	0	0	0	0	1000	0	0	0
Clostridium_innocuum_I46	0	0	0	0	0	0	1000	0	0	0
Clostridium_isatidis_DSM_15098	0	0	0	0	1000	0	1000	0	0	0
Clostridium_jeddahense_JCD	0	0	0	0	0	0	1000	0	0	0
Clostridium_jeddahtimonense_CL_2	0	0	0	0	1000	0	1000	0	0	0
Clostridium_lavalense_NLAE_zl_G277	0	0	0	0	1000	0	1000	0	0	0
Clostridium_leptum_DSM_753	0	0	0	1000	1000	0	1000	1000	0	0
Clostridium_massilioamazoniensis_ND2	0	0	1000	0	0	0	1000	0	0	0
Clostridium_methoxybenzovorans_SR3	0	0	1000	0	1000	0	1000	0	0	0
Clostridium_methylpentosum_R2_DSM_5476	0	0	0	0	0	0	0	0	0	0
Clostridium_nexile_DSM_1787	0	0	0	0	1000	0	1000	0	1000	0
Clostridium_papyrosolvens_C7	0	0	0	0	0	0	1000	0	0	0
Clostridium_papyrosolvens_DSM_2782	0	0	0	0	0	0	1000	0	0	0
Clostridium_paraputrificum_AGR2156	0	0	0	0	1000	0	1000	0	0	0
Clostridium_paraputrificum_ERR1022469	0	0	1000	0	1000	0	1000	0	0	0
Clostridium_pasteurianum_BC1	0	0	0	0	1000	0	0	0	0	0
Clostridium_pasteurianum_DSM_525_ATCC_6013	0	0	0	0	1000	0	1000	0	0	0
Clostridium_perfringens_ATCC_13124	1000	0	0	45.52352049	1000	0	1000	0	0	0
Clostridium_perfringens_B_str_ATCC_3626	0	0	0	45.52352049	1000	0	1000	0	0	0
Clostridium_perfringens_C_str_JGS1495	0	0	0	1000	1000	0	1000	0	0	0
Clostridium_perfringens_CPE_str_F4969	0	0	0	1000	1000	0	1000	0	0	0
Clostridium_perfringens_D_str_JGS1721	0	0	0	1000	0	0	1000	0	0	0
Clostridium_perfringens_E_str_JGS1987	0	0	0	1000	1000	0	1000	0	0	0
Clostridium_perfringens_F262	0	0	0	1000	1000	0	1000	0	0	0
Clostridium_perfringens_NCTC_8239	0	0	0	1000	1000	0	1000	0	0	0
Clostridium_perfringens_SM101	0	0	0	45.52352049	1000	0	1000	0	0	0
Clostridium_perfringens_str_13	1000	0	0	1000	1000	0	1000	0	0	0
Clostridium_perfringens_WAL_14572	0	0	0	1000	1000	0	1000	0	0	0
Clostridium_polynesiense_MS1	0	1000	1000	0	1000	0	0	0	0	0
Clostridium_polysaccharolyticum_DSM_1801	0	0	1000	0	0	0	1000	0	0	0
Clostridium_ramosum_VPI_0427_DSM_1402	0	0	0	0	1000	0	1000	0	1000	0
Clostridium_saccharogumia_DSM_17460	0	0	1000	0	1000	0	1000	0	0	0
Clostridium_saccharolyticum_WM1	0	0	0	0	1000	0	1000	0	1000	0
Clostridium_saccharoperbutylacetonicum_N1_4_H	1000	0	0	1000	1000	0	1000	0	0	0
Clostridium_sartagoforme_AAU1	0	0	0	1000	1000	0	1000	0	0	0
Clostridium_saudiense_JCC	0	0	1000	0	1000	0	1000	0	0	0
Clostridium_scindens_ATCC_35704	0	0	0	0	1000	0	1000	0	1000	0
Clostridium_senegalense_JC122	0	0	1000	0	1000	0	1000	0	0	0
Clostridium_sp_7_2_43FAA	0	0	0	1000	1000	0	1000	0	0	0
Clostridium_sp_7_3_54FAA	0	0	1000	0	1000	0	1000	0	0	0
Clostridium_sp_ASF356	0	0	1000	0	1000	0	1000	0	0	0
Clostridium_sp_ATCC_BAA_442	0	0	0	0	1000	0	1000	0	0	0
Clostridium_sp_BNL1100	0	0	0	0	0	0	1000	0	0	0
Clostridium_sp_D5	0	0	0	0	1000	0	1000	0	0	0
Clostridium_sp_DL_VIII	0	0	1000	0	1000	0	0	0	0	0
Clostridium_sp_HGF2	0	0	0	0	0	0	1000	0	0	0
Clostridium_sp_KLE_1755	0	0	0	0	1000	0	1000	0	0	0
Clostridium_sp_L2_50	0	0	0	0	4.55E-13	0	0	0	0	0
Clostridium_sp_M62_1	0	0	1000	0	1000	0	1000	0	0	0
Clostridium_sp_MSTE9	0	0	0	0	1000	0	1000	0	0	0
Clostridium_sp_SS2_1	0	0	0	0	1000	0	1000	0	0	0
Clostridium_sp_SY8519	0	0	0	0	1000	0	0	0	0	0
Clostridium_sphenoides_JCM_1415	0	0	0	0	1000	0	1000	0	1000	0
Clostridium_spiroforme_DSM_1552	0	0	0	0	0	0	1000	0	0	0
Clostridium_sporogenes_ATCC_15579	0	0	0	0	1000	1000	1000	0	1000	0
Clostridium_sporogenes_PA_3679	0	0	0	0	1000	1000	1000	0	1000	0
Clostridium_sporosphaeroides_DSM_1294	0	0	0	0	1000	0	1000	0	0	0

Clostridium_stercorarium_subsp_leptospartum_D	0	0	0	0	0	0	1000	0	0	0
Clostridium_stercorarium_subsp_stercorarium_DS	0	0	0	1000	0	0	1000	0	0	0
Clostridium_stercorarium_subsp_thermolacticum_	0	0	0	0	0	0	1000	0	0	0
Clostridium_sticklandii_DSM_519	0	0	0	0	1000	1000	1000	0	0	0
Clostridium_sulfidigenes_113A_c1	0	0	1000	0	1000	0	1000	0	1000	0
Clostridium_symbiosum_ATCC_14940	0	0	1000	0	1000	0	1000	0	1000	0
Clostridium_symbiosum_WAL_14163	0	0	1000	0	1000	0	1000	0	1000	0
Clostridium_symbiosum_WAL_14673	0	0	1000	0	1000	0	1000	0	1000	0
Clostridium_tertium_Gcol_A43_Gcol_A43_1	0	0	0	0	1000	0	1000	0	0	0
Clostridium_tyrobutyricum_DSM_2637	0	0	0	0	1000	0	0	0	0	0
Clostridium_tyrobutyricum_UC7086	0	0	0	0	1000	0	0	0	0	0
Clostridium_viride_DSM_6836	0	0	1000	0	0	0	0	0	0	0
Cohnella_laeviribosi_DSM_21336	0	1000	1000	0	0	0	0	0	0	0
Collinsella_aerofaciens_ATCC_25986	0	0	0	0	1000	0	1000	0	0	0
Collinsella_aerofaciens_ERR1022282	0	0	0	0	0	0	1000	0	0	0
Collinsella_aerofaciens_ERR1022300	0	0	0	0	0	0	1000	0	0	0
Collinsella_aerofaciens_ERR1022416	0	0	0	0	0	0	1000	0	0	0
Collinsella_aerofaciens_ERR1203940	0	0	0	0	0	0	1000	0	0	0
Collinsella_aerofaciens_ERR1204034	0	0	0	0	0	0	1000	0	0	0
Collinsella_aerofaciens_ERR2221153	0	0	0	0	0	0	1000	0	0	0
Collinsella_aerofaciens_ERR2230078	0	0	0	0	0	0	1000	0	0	0
Collinsella_aerofaciens_ERR2230087	0	0	0	0	0	0	1000	0	0	0
Collinsella_aerofaciens_ERR2230090	0	0	0	0	0	0	1000	0	0	0
Collinsella_aerofaciens_ERR2230102	0	0	0	0	0	0	1000	0	0	0
Collinsella_aerofaciens_ERR2230112	0	0	0	0	0	0	1000	0	0	0
Collinsella_aerofaciens_ERR2230122	0	0	0	0	0	0	1000	0	0	0
Collinsella_aerofaciens_ERR2230127	0	0	0	0	0	0	1000	0	0	0
Collinsella_aerofaciens_ERR2230140	0	0	0	0	0	0	1000	0	0	0
Collinsella_aerofaciens_ERR2230145	0	0	0	0	0	0	1000	0	0	0
Collinsella_aerofaciens_ERR2230147	0	0	0	0	0	0	1000	0	0	0
Collinsella_aerofaciens_ERR2230156	0	0	0	0	0	0	1000	0	0	0
Collinsella_intestinalis_DSM_13280	0	0	0	0	0	0	1000	0	0	0
Collinsella_massiliensis_An5	0	0	0	0	0	0	1000	0	0	0
Collinsella_sp_4_8_47FAA	0	0	0	0	0	0	1000	0	0	0
Collinsella_sp_MS5	0	0	0	0	0	0	1000	0	0	0
Collinsella_stercoris_DSM_13279	0	0	0	0	0	0	0	0	0	0
Collinsella_tanakaei_YIT_12063	0	0	0	1000	0	0	1000	0	0	1000
Comamonas_aquatica_CJG	0	0	0	0	1000	0	1000	0	0	0
Comamonas_aquatica_DA1877	0	0	0	0	1000	0	1000	0	0	0
Comamonas_aquatica_NBRC_14918	0	0	0	0	1000	0	1000	0	0	0
Comamonas_terrigena_FDAARGOS_394_pRIID_98	0	0	0	0	0	0	1000	0	0	0
Comamonas_terrigena_NBRC_13299	0	0	0	0	0	0	1000	0	0	0
Comamonas_testosteroni_ATCC_11996	0	0	0	0	0	0	1000	0	0	0
Comamonas_testosteroni_CNB_2	111.1111111	0	0	0	0	0	1000	0	0	1000
Comamonas_testosteroni_KF_1	0	0	0	0	0	0	1000	0	0	0
Comamonas_testosteroni_NBRC_100989	0	0	0	0	0	0	1000	0	0	0
Comamonas_testosteroni_S44	0	0	0	0	0	0	1000	0	0	0
Coprobacillus_cateniformis_29_1	0	0	0	0	0	0	1000	0	0	0
Coprobacillus_sp_3_3_56FAA	0	0	0	0	1000	0	1000	0	0	0
Coprobacillus_sp_8_2_54BFAA	0	0	0	0	1000	0	1000	0	0	0
Coprobacillus_sp_D7	0	0	0	0	1000	0	1000	0	0	0
Coprobacter_fastidiosus_NSB1	0	0	0	0	0	0	0	0	0	0
Coprococcus_catus_ERR2221258	0	0	0	0	1000	0	1000	0	0	0
Coprococcus_catus_GD_7	0	0	0	0	1000	0	1000	0	0	0
Coprococcus_comes_ATCC_27758	0	0	0	0	0	0	0	0	0	0
Coprococcus_comes_ERR1022290	0	0	0	0	0	0	1000	0	0	0
Coprococcus_comes_ERR1022354	0	0	0	0	0	0	1000	0	0	0
Coprococcus_comes_ERR1022382	0	0	0	0	0	0	1000	0	0	0
Coprococcus_comes_ERR1022427	0	0	0	0	0	0	1000	0	0	0
Coprococcus_comes_ERR1022476	0	0	0	0	0	0	1000	0	0	0
Coprococcus_comes_ERR1204042	0	0	0	0	0	0	1000	0	0	0
Coprococcus_eutactus_ATCC_27759	0	0	0	0	0	0	1000	0	0	0
Coprococcus_eutactus_ERR1022287	0	0	0	0	0	0	1000	0	0	0
Coprococcus_eutactus_ERR1022301	0	0	0	0	0	0	1000	0	0	0
Coprococcus_eutactus_ERR1022346	0	0	0	0	0	0	1000	0	0	0
Coprococcus_eutactus_ERR1022477	0	0	0	0	0	0	1000	0	0	0
Coprococcus_eutactus_ERR1203946	0	0	0	0	0	0	1000	0	0	0
Coprococcus_eutactus_ERR1204040	0	0	0	0	0	0	1000	0	0	0
Coprococcus_eutactus_ERR2221154	0	0	0	0	0	0	1000	0	0	0
Coprococcus_nov_ERR1022278	0	0	0	0	0	0	1000	0	0	0
Coprococcus_nov_ERR1203948	0	0	0	0	0	0	1000	0	0	0
Coprococcus_nov_ERR2221394	0	0	0	0	0	0	0	0	0	0
Coprococcus_nov_ERR2230098	0	0	0	0	1000	0	1000	0	0	0
Coprococcus_sp_HPP0048	0	0	0	0	0	0	1000	0	0	0
Coprococcus_sp_HPP0074	0	0	0	0	0	0	1000	0	0	0
Corynebacterium_accolens_AH4003	0	0	1000	0	1000	0	1000	0	0	0
Corynebacterium_accolens_ATCC_49725	0	0	0	0	1000	0	1000	0	0	0
Corynebacterium_ammoniagenes_DSM_20306	0	0	0	0	1000	0	1000	0	0	1000
Corynebacterium_amycolatum_SK46	0	0	0	0	1000	0	1000	0	0	1000
Corynebacterium_argentoratense_CNM_46305_c	0	0	1000	0	0	0	1000	0	0	0
Corynebacterium_argentoratense_DSM_44202	0	0	1000	0	0	0	1000	0	0	0
Corynebacterium_aurimucosum_ATCC_700975	0	0	0	0	1000	0	1000	0	0	1000
Corynebacterium_casei_LMG_S_19264	0	0	1000	0	1000	0	1000	0	0	0
Corynebacterium_casei_UCMA_3821	0	0	1000	0	1000	0	1000	0	0	0
Corynebacterium_coyleae_UMB0147_16933_8_1	0	0	1000	0	1000	0	1000	0	0	0
Corynebacterium_durum_F0235	0	0	0	0	1000	0	1000	0	0	1000
Corynebacterium_efficiens_YS_314	0	0	1000	1000	1000	0	1000	0	0	0
Corynebacterium_glucuronolyticum_ATCC_51866	0	0	1000	1000	1000	0	1000	0	0	0
Corynebacterium_glucuronolyticum_ATCC_51867	0	0	0	1000	1000	0	0	0	0	0
Corynebacterium_ihumii_GD7	0	0	1000	0	1000	0	1000	0	0	0
Corynebacterium_jeddahense_JCB	0	0	1000	0	1000	0	1000	0	0	0
Corynebacterium_jeikeium_Cj30184_10	0	0	1000	0	1000	0	1000	0	0	0
Corynebacterium_kroppenstedtii_DNF00591	0	0	1000	0	1000	0	1000	0	0	0
Corynebacterium_kroppenstedtii_DSM_44385	0	0	0	0	1000	0	0	0	0	0

Corynebacterium_mastitidis_DSM_44356	0	0	1000	0	1000	0	1000	0	0	0
Corynebacterium_matruchotii_ATCC_14266	0	0	1000	0	1000	0	1000	0	0	0
Corynebacterium_matruchotii_ATCC_33806	0	0	1000	0	1000	0	1000	0	0	0
Corynebacterium_matruchotii_NCTC10254	0	0	0	0	1000	0	1000	0	0	0
Corynebacterium_propinquum_DSM_44285	0	0	0	0	1000	0	1000	0	0	0
Corynebacterium_pseudodiphtheriticum_DSM_44	0	0	1000	0	1000	0	1000	0	0	0
Corynebacterium_pseudodiphtheriticum_ERR222	0	0	1000	0	1000	0	1000	0	0	0
Corynebacterium_pseudogenitalium_ATCC_33035	0	0	1000	0	1000	0	1000	0	0	0
Corynebacterium_sp_HFH0082	0	0	1000	0	1000	0	1000	0	0	0
Corynebacterium_striatum_ATCC_6940	0	0	0	0	1000	0	1000	0	0	1000
Corynebacterium_tuberculostearicum_SK141	0	0	0	0	1000	0	1000	0	0	1000
Corynebacterium_ulcerans_809	0	0	0	0	1000	0	0	0	0	1000
Corynebacterium_ulcerans_BR_AD22	0	0	1000	0	1000	0	1000	0	0	0
Corynebacterium_ureicelerivorans_DSM_45051	0	0	1000	0	1000	0	1000	0	0	1000
Corynebacterium_variabile_DSM_44702	0	0	1000	0	1000	0	0	0	0	0
Corynebacterium_variabile_Mu292	0	0	1000	0	1000	0	0	0	0	0
Cronobacter_sakazakii_2151	0	0	0	0	1000	0	1000	0	1000	0
Cronobacter_sakazakii_680	0	0	0	0	1000	0	1000	0	1000	0
Cronobacter_sakazakii_696	0	0	0	0	1000	0	1000	0	1000	0
Cronobacter_sakazakii_701	0	0	0	0	1000	0	1000	0	1000	0
Cronobacter_sakazakii_ATCC_BAA_894	1000	0	0	0	1000	0	1000	0	1000	1000
Cronobacter_sakazakii_E764	0	0	0	0	1000	0	1000	0	1000	0
Cronobacter_sakazakii_ES15	916.6666667	0	0	0	1000	0	1000	0	1000	0
Cronobacter_sakazakii_ES35	0	0	0	0	1000	0	1000	0	1000	0
Cronobacter_sakazakii_ES713	0	0	0	0	1000	0	1000	0	1000	0
Cronobacter_sakazakii_SP291	916.6666667	0	0	0	1000	0	1000	0	1000	0
Cryocola_sp_340MFSha3_1	0	0	0	0	1000	0	0	0	0	0
Cryptobacterium_curtum_DSM_15641	0	0	0	0	1000	0	0	0	0	0
Cuneatibacter_caecimuris_DSM_29486	0	0	0	0	0	0	0	0	0	0
Cupriavidus_metallidurans_CH34	250	0	1000	0	0	0	1000	0	0	0
Curtobacterium_flaccumfaciens_MEB126	0	0	0	0	0	0	0	0	1000	0
Curtobacterium_flaccumfaciens_UCD_AKU	0	0	0	0	0	0	0	0	1000	1000
Curvibacter_gracilis_ATCC_BAA_807	0	0	1000	0	0	0	0	0	0	0
Cutibacterium_acnes_266	0	0	0	1000	0	0	1000	0	0	0
Cutibacterium_acnes_6609	0	0	0	1000	0	0	1000	0	0	0
Cutibacterium_acnes_ATCC_11828	0	0	0	1000	0	0	1000	0	0	0
Cutibacterium_acnes_C1	0	0	0	1000	0	0	1000	0	0	0
Cutibacterium_acnes_ERR2221336	0	0	0	0	0	0	1000	0	0	0
Cutibacterium_acnes_ERR2221340	0	0	0	0	0	0	1000	0	0	0
Cutibacterium_acnes_ERR2221379	0	0	0	0	0	0	1000	0	0	0
Cutibacterium_acnes_FZ1_2_0	0	0	0	1000	0	0	1000	0	0	0
Cutibacterium_acnes_HL001PA1	0	0	0	1000	0	0	1000	0	0	0
Cutibacterium_acnes_HL002PA1	0	0	0	1000	0	0	1000	0	0	0
Cutibacterium_acnes_HL002PA2	0	0	0	1000	0	0	1000	0	0	0
Cutibacterium_acnes_HL002PA3	0	0	0	1000	0	0	1000	0	0	0
Cutibacterium_acnes_HL005PA1	0	0	0	1000	0	0	1000	0	0	0
Cutibacterium_acnes_HL005PA2	0	0	0	1000	0	0	1000	0	0	0
Cutibacterium_acnes_HL005PA4	0	0	0	1000	0	0	1000	0	0	0
Cutibacterium_acnes_HL007PA1	0	0	0	1000	0	0	1000	0	0	0
Cutibacterium_acnes_HL013PA1	0	0	0	1000	0	0	1000	0	0	0
Cutibacterium_acnes_HL013PA2	0	0	0	1000	0	0	1000	0	0	0
Cutibacterium_acnes_HL020PA1	0	0	0	1000	0	0	1000	0	0	0
Cutibacterium_acnes_HL025PA1	0	0	0	1000	0	0	1000	0	0	0
Cutibacterium_acnes_HL027PA1	0	0	0	1000	0	0	1000	0	0	0
Cutibacterium_acnes_HL027PA2	0	0	0	1000	0	0	1000	0	0	0
Cutibacterium_acnes_HL030PA1	0	0	0	1000	0	0	1000	0	0	0
Cutibacterium_acnes_HL036PA1	0	0	0	1000	0	0	1000	0	0	0
Cutibacterium_acnes_HL036PA2	0	0	0	1000	0	0	1000	0	0	0
Cutibacterium_acnes_HL037PA1	0	0	0	1000	0	0	1000	0	0	0
Cutibacterium_acnes_HL037PA2	0	0	0	0	0	0	1000	0	0	0
Cutibacterium_acnes_HL038PA1	0	0	0	1000	0	0	1000	0	0	0
Cutibacterium_acnes_HL043PA1	0	0	0	1000	0	0	1000	0	0	0
Cutibacterium_acnes_HL043PA2	0	0	0	1000	0	0	1000	0	0	0
Cutibacterium_acnes_HL044PA1	0	0	0	0	0	0	1000	0	0	0
Cutibacterium_acnes_HL045PA1	0	0	0	1000	0	0	1000	0	0	0
Cutibacterium_acnes_HL046PA1	0	0	0	1000	0	0	1000	0	0	0
Cutibacterium_acnes_HL046PA2	0	0	0	1000	0	0	1000	0	0	0
Cutibacterium_acnes_HL050PA1	0	0	0	1000	0	0	1000	0	0	0
Cutibacterium_acnes_HL050PA2	0	0	0	1000	0	0	1000	0	0	0
Cutibacterium_acnes_HL050PA3	0	0	0	1000	0	0	1000	0	0	0
Cutibacterium_acnes_HL053PA1	0	0	0	1000	0	0	1000	0	0	0
Cutibacterium_acnes_HL053PA2	0	0	0	1000	0	0	1000	0	0	0
Cutibacterium_acnes_HL056PA1	0	0	0	1000	0	0	1000	0	0	0
Cutibacterium_acnes_HL059PA1	0	0	0	1000	0	0	1000	0	0	0
Cutibacterium_acnes_HL059PA2	0	0	0	1000	0	0	1000	0	0	0
Cutibacterium_acnes_HL060PA1	0	0	0	1000	0	0	1000	0	0	0
Cutibacterium_acnes_HL063PA1	0	0	0	1000	0	0	1000	0	0	0
Cutibacterium_acnes_HL063PA2	0	0	0	1000	0	0	1000	0	0	0
Cutibacterium_acnes_HL067PA1	0	0	0	1000	0	0	1000	0	0	0
Cutibacterium_acnes_HL072PA1	0	0	0	1000	0	0	1000	0	0	0
Cutibacterium_acnes_HL072PA2	0	0	0	1000	0	0	1000	0	0	0
Cutibacterium_acnes_HL074PA1	0	0	0	1000	0	0	1000	0	0	0
Cutibacterium_acnes_HL078PA1	0	0	0	1000	0	0	1000	0	0	0
Cutibacterium_acnes_HL082PA1	0	0	0	1000	0	0	1000	0	0	0
Cutibacterium_acnes_HL082PA2	0	0	0	1000	0	0	1000	0	0	0
Cutibacterium_acnes_HL083PA1	0	0	0	1000	0	0	1000	0	0	0
Cutibacterium_acnes_HL083PA2	0	0	0	1000	0	0	1000	0	0	0
Cutibacterium_acnes_HL086PA1	0	0	0	1000	0	0	1000	0	0	0
Cutibacterium_acnes_HL087PA2	0	0	0	1000	0	0	1000	0	0	0
Cutibacterium_acnes_HL087PA3	0	0	0	1000	0	0	1000	0	0	0
Cutibacterium_acnes_HL092PA1	0	0	0	1000	0	0	1000	0	0	0
Cutibacterium_acnes_HL096PA1	0	0	0	1000	0	0	1000	0	0	0
Cutibacterium_acnes_HL096PA2	0	0	0	1000	0	0	1000	0	0	0
Cutibacterium_acnes_HL096PA3	0	0	0	1000	0	0	1000	0	0	0

Cutibacterium_acnes_HL097PA1	0	0	0	1000	0	0	1000	0	0	0
Cutibacterium_acnes_HL099PA1	0	0	0	1000	0	0	1000	0	0	0
Cutibacterium_acnes_HL103PA1	0	0	0	1000	0	0	1000	0	0	0
Cutibacterium_acnes_HL110PA1	0	0	0	1000	0	0	1000	0	0	0
Cutibacterium_acnes_HL110PA2	0	0	0	1000	0	0	1000	0	0	0
Cutibacterium_acnes_HL110PA3	0	0	0	1000	0	0	1000	0	0	0
Cutibacterium_acnes_HL110PA4	0	0	0	1000	0	0	1000	0	0	0
Cutibacterium_acnes_J139	0	0	0	1000	0	0	1000	0	0	0
Cutibacterium_acnes_J165	0	0	0	1000	0	0	1000	0	0	0
Cutibacterium_acnes_KPA171202	0	0	0	1000	0	0	1000	0	0	0
Cutibacterium_acnes_PA_12_1_L1	0	0	0	0	0	0	1000	0	0	0
Cutibacterium_acnes_PA_12_1_R1	0	0	0	0	0	0	1000	0	0	0
Cutibacterium_acnes_PA_15_2_L1	0	0	0	0	0	0	1000	0	0	0
Cutibacterium_acnes_PA_21_1_L1	0	0	0	0	0	0	1000	0	0	0
Cutibacterium_acnes_PA_30_2_L1	0	0	0	0	0	0	1000	0	0	0
Cutibacterium_acnes_PRP_38	0	0	0	1000	0	0	1000	0	0	0
Cutibacterium_acnes_SK137	0	0	0	1000	0	0	1000	0	0	0
Cutibacterium_acnes_SK182	0	0	0	1000	0	0	1000	0	0	0
Cutibacterium_acnes_SK187	0	0	0	1000	0	0	1000	0	0	0
Cutibacterium_acnes_TypeIA2_P_acn17	0	0	0	1000	0	0	1000	0	0	0
Cutibacterium_acnes_TypeIA2_P_acn31	0	0	0	1000	0	0	1000	0	0	0
Cutibacterium_acnes_TypeIA2_P_acn33	0	0	0	1000	0	0	1000	0	0	0
Cutibacterium_granulosum_TM11	0	0	0	0	0	0	1000	0	0	0
Dakarella_massiliensis_ND3	0	0	0	0	1000	0	1000	0	0	0
Dechlorosoma_suillum_PS	0	0	0	0	0	0	0	0	0	0
Dehalobacterium_formicoaceticum_DMC	0	0	1000	0	0	0	1000	0	0	0
Delftia_acidovorans_CCUG_15835	0	0	1000	0	1000	0	1000	0	1000	0
Delftia_acidovorans_CCUG_274B	0	0	1000	0	1000	0	1000	0	1000	0
Delftia_acidovorans_SPH_1	1000	0	1000	0	1000	0	1000	0	1000	1000
Dermabacter_sp_HFH0086	0	0	0	0	1000	0	0	0	0	0
Dermacoccus_nishinomiyaensis_DSM_20448	0	0	1000	0	0	0	1000	0	0	1000
Dermacoccus_sp_Ellin185	0	0	0	0	0	0	0	0	0	0
Desmospora_sp_8437	0	0	1000	0	1000	0	0	0	0	0
Desulfitobacterium_hafniense_DCB_2	0	0	1000	0	0	0	1000	0	1000	0
Desulfitobacterium_hafniense_DP7	0	0	1000	0	0	0	1000	0	1000	0
Desulfitobacterium_hafniense_PCP_1	0	0	1000	0	0	0	1000	0	1000	0
Desulfitobacterium_hafniense_Y51	0	0	1000	0	0	0	1000	0	1000	0
Desulfomicrobium_orale_DSM_12838	0	0	0	0	1000	0	0	0	1000	0
Desulfovibrio_desulfuricans_subsp_aestuarii_DSM	0	0	0	0	1000	0	0	0	1000	0
Desulfovibrio_desulfuricans_subsp_desulfuricans_	0	0	0	0	1000	0	1000	0	1000	0
Desulfovibrio_desulfuricans_subsp_desulfuricans_	0	0	0	0	0	0	1000	0	1000	0
Desulfovibrio_desulfuricans_subsp_desulfuricans_	0	0	0	0	0	0	0	0	1000	0
Desulfovibrio_legallii_KHC7	0	0	0	0	0	0	0	0	0	0
Desulfovibrio_piger_ATCC_29098	0	0	0	0	1000	0	0	0	1000	0
Desulfovibrio_sp_3_1_syn3	0	0	0	0	1000	0	0	0	1000	0
Desulfovibrio_sp_6_1_46AFAA	0	0	0	0	1000	0	0	0	0	0
Dialister_invisus_DSM_15470	0	0	1000	0	1000	0	1000	0	0	0
Dialister_micraerophilus_DSM_19965	0	0	1000	0	200	0	0	0	0	0
Dialister_microaerophilus_UPII_345_E	0	0	1000	0	750	0	833.3333333	0	0	0
Dialister_pneumosintes_F0677	0	0	0	0	875	0	0	0	0	0
Dialister_succinatiphilus_YIT_11850	0	0	0	0	1000	0	1000	0	0	0
Dielma_fastidiosa_JC13	0	0	0	0	0	0	1000	0	0	0
Dietzia_cinnamea_NBRC_102147	0	0	0	0	0	0	0	0	0	0
Dietzia_cinnamea_P4	0	0	0	0	0	0	0	0	0	1000
Dolosigranulum_pigrum_ATCC_51524	0	0	0	0	1000	0	1000	0	0	0
Dolosigranulum_pigrum_KPL1931_CDC4294_98	0	0	0	0	1000	0	1000	0	0	0
Dorea_formicigenerans_4_6_53AFAA	0	0	0	0	1000	0	1000	0	0	0
Dorea_formicigenerans_ATCC_27755	0	0	0	0	1000	0	1000	0	0	0
Dorea_formicigenerans_ERR1022384	0	0	0	0	1000	0	1000	0	0	0
Dorea_formicigenerans_ERR1203975	0	0	0	0	1000	0	1000	0	0	0
Dorea_formicigenerans_ERR1204069	0	0	0	0	1000	0	1000	0	0	0
Dorea_formicigenerans_ERR2221255	0	0	0	0	1000	0	1000	0	0	0
Dorea_formicigenerans_ERR2221257	0	0	0	0	1000	0	1000	0	0	0
Dorea_formicigenerans_ERR2221275	0	0	0	0	1000	0	1000	0	0	0
Dorea_formicigenerans_ERR2230157	0	0	0	0	1000	0	1000	0	0	0
Dorea_longicatena_DSM_13814	0	0	0	0	0	0	0	0	0	0
Dorea_longicatena_ERR1022309	0	0	0	0	1000	0	1000	0	0	0
Dorea_longicatena_ERR1022324	0	0	0	0	1000	0	0	0	0	0
Dorea_longicatena_ERR1022383	0	0	1000	0	1000	0	1000	0	0	0
Dorea_longicatena_ERR1022428	0	0	0	0	1000	0	1000	0	0	0
Dorea_longicatena_ERR1022475	0	0	0	0	1000	0	1000	0	0	0
Dorea_longicatena_ERR1203969	0	0	0	0	1000	0	1000	0	0	0
Dorea_longicatena_ERR1204063	0	0	0	0	1000	0	1000	0	0	0
Dorea_longicatena_ERR2221186	0	0	0	0	1000	0	0	0	0	0
Dorea_longicatena_ERR2221271	0	0	0	0	1000	0	1000	0	0	0
Dorea_longicatena_ERR2230069	0	0	0	0	1000	0	1000	0	0	0
Dorea_nov_ERR2230118	0	0	0	0	1000	0	1000	0	0	0
Dyadobacter_beijingensis_DSM_21582	0	0	0	0	1000	0	0	0	0	1000
Dyadobacter_fermentans_DSM_18053	0	0	1000	0	1000	0	0	1000	0	1000
Dysgonomonas_gadei_ATCC_BAA_286	0	0	0	0	1000	0	0	0	0	1000
Dysgonomonas_mossii_DSM_22836	0	0	0	1000	1000	0	0	1000	0	0
Edwardsiella_tarda_080813	0	0	0	1000	1000	0	1000	0	0	0
Edwardsiella_tarda_ATCC_23685	0	0	0	1000	1000	0	1000	0	0	0
Edwardsiella_tarda_C07_087	0	0	0	1000	1000	0	1000	0	0	0
Edwardsiella_tarda_DT	0	0	0	0	1000	0	1000	0	0	0
Edwardsiella_tarda_EIB202	0	0	0	1000	1000	0	1000	0	0	0
Edwardsiella_tarda_FL6_60	0	0	0	1000	1000	0	1000	0	0	0
Edwardsiella_tarda_FL95_01	0	0	0	0	1000	0	1000	0	0	0
Edwardsiella_tarda_NBRC_105688	0	0	0	1000	1000	0	1000	0	0	0
Eggerthella_lenta_1160AFAA	0	0	1000	0	0	0	1000	0	0	0
Eggerthella_lenta_11C	0	0	1000	0	0	0	1000	0	0	0
Eggerthella_lenta_1356FAA	0	0	1000	0	0	0	0	0	0	0
Eggerthella_lenta_14A	0	0	1000	0	0	0	1000	0	0	0
Eggerthella_lenta_16A	0	0	1000	0	0	0	1000	0	0	0

Eggerthella_lenta_19C	0	0	1000	0	0	0	1000	0	0	0
Eggerthella_lenta_22C	0	0	1000	0	0	0	1000	0	0	0
Eggerthella_lenta_28B	0	0	1000	0	0	0	1000	0	0	0
Eggerthella_lenta_326I6NA	0	0	1000	0	0	0	1000	0	0	0
Eggerthella_lenta_A2	0	0	1000	0	0	0	1000	0	0	0
Eggerthella_lenta_AB12n2	0	0	1000	0	0	0	1000	0	0	0
Eggerthella_lenta_AB8n2	0	0	1000	0	0	0	1000	0	0	0
Eggerthella_lenta_AN51LG	0	0	1000	0	0	0	1000	0	0	0
Eggerthella_lenta_ATCC_25559	0	0	1000	0	0	0	1000	0	0	0
Eggerthella_lenta_C592	0	0	1000	0	0	0	1000	0	0	0
Eggerthella_lenta_CC75D52	0	0	1000	0	0	0	1000	0	0	0
Eggerthella_lenta_CC82BHI2	0	0	1000	0	0	0	1000	0	0	0
Eggerthella_lenta_CC86D54	0	0	1000	0	0	0	1000	0	0	0
Eggerthella_lenta_DSM_11767	0	0	1000	0	0	0	1000	0	0	0
Eggerthella_lenta_DSM_11863	0	0	1000	0	0	0	1000	0	0	0
Eggerthella_lenta_DSM_15644	0	0	1000	0	0	0	1000	0	0	0
Eggerthella_lenta_DSM_2243	0	0	0	0	0	0	0	0	0	0
Eggerthella_lenta_HGA1	0	0	1000	0	0	0	1000	0	0	0
Eggerthella_lenta_MR1n12	0	0	1000	0	0	0	1000	0	0	0
Eggerthella_lenta_RC46F	0	0	1000	0	0	0	1000	0	0	0
Eggerthella_lenta_Valencia	0	0	1000	0	0	0	1000	0	0	0
Eggerthella_lenta_W1BHI6	0	0	1000	0	0	0	1000	0	0	0
Eggerthella_sinensis_DSM_16107	0	0	1000	0	0	0	1000	0	0	0
Eggerthella_sp_1_3_56FAA	0	0	0	0	0	0	1000	0	0	0
Eggerthella_sp_YY7918	0	0	0	0	0	0	0	0	0	0
Eggerthia_catenaformis_OT_569	0	0	0	0	0	0	1000	0	0	0
Eikenella_corrodens_ATCC_23834	0	0	0	0	1000	0	1000	0	0	1000
Eisenbergiella_nov_ERR2221109	0	0	0	0	1000	0	1000	0	0	0
Eisenbergiella_nov_ERR2221178	0	0	0	0	1000	0	1000	0	0	0
Eisenbergiella_tayi_ERR1022439	0	0	0	0	1000	0	1000	0	0	0
Eisenbergiella_tayi_NML110678	0	0	0	0	0	0	1000	0	0	0
Elusimicrobium_minutum_Pei191	0	0	0	0	0	0	1000	0	0	0
Enhydrobacter_aerosaccus_ATCC_27094	0	0	0	0	0	0	0	0	1000	0
Enorma_massiliensis_ERR1022365	0	0	0	0	0	0	1000	0	0	0
Enorma_timonensis_GD5	0	0	0	0	0	0	1000	0	0	0
Enterobacter_aerogenes_EA1509E	1000	0	0	0	1000	0	1000	0	0	0
Enterobacter_aerogenes_ERR2221162	0	0	1000	0	1000	0	1000	0	0	0
Enterobacter_aerogenes_FGI35	0	0	0	0	1000	0	1000	0	0	0
Enterobacter_aerogenes_KCTC_2190	1000	0	0	0	0	0	1000	0	0	1000
Enterobacter_asburiae_L1	0	0	1000	0	1000	0	1000	0	0	0
Enterobacter_asburiae_LF7a	1000	0	0	0	1000	0	1000	0	0	1000
Enterobacter_cancerogenus_ATCC_35316	0	0	0	0	1000	0	1000	0	0	0
Enterobacter_cloacae_EcWSU1	1000	0	0	0	1000	0	1000	0	0	1000
Enterobacter_cloacae_ERR2221114	0	0	1000	0	1000	0	1000	0	0	0
Enterobacter_cloacae_ERR2221117	0	0	1000	0	1000	0	1000	0	0	0
Enterobacter_cloacae_ERR2221156	0	0	1000	0	1000	0	1000	0	0	0
Enterobacter_cloacae_ERR2221157	0	0	1000	0	1000	0	1000	0	0	0
Enterobacter_cloacae_ERR2221194	0	0	1000	0	1000	0	1000	0	0	0
Enterobacter_cloacae_GGT036	0	0	1000	0	1000	0	1000	0	0	0
Enterobacter_cloacae_subsp_cloacae_08XA1	0	0	1000	0	1000	0	1000	0	0	0
Enterobacter_cloacae_subsp_cloacae_ATCC_1304	1000	0	1000	0	1000	0	1000	0	0	0
Enterobacter_cloacae_subsp_cloacae_ENHKU01	0	0	1000	0	1000	0	1000	0	0	0
Enterobacter_cloacae_subsp_cloacae_GS1	0	0	1000	0	1000	0	1000	0	0	0
Enterobacter_cloacae_subsp_dissolvens_SDM	1000	0	1000	0	1000	0	1000	0	0	0
Enterobacter_hormaechei_ATCC_49162	0	0	1000	0	1000	0	1000	0	0	0
Enterobacter_hormaechei_FDAARGOS_68	0	0	1000	0	1000	0	1000	0	0	0
Enterobacter_hormaechei_YT2	0	0	0	0	1000	0	1000	0	0	1000
Enterobacter_hormaechei_YT3	0	0	0	0	1000	0	1000	0	0	1000
Enterobacter_lignolyticus_G5	0	0	1000	0	1000	0	1000	0	0	0
Enterobacter_lignolyticus_SCF1	0	0	1000	0	1000	0	1000	0	0	0
Enterobacter_ludwigii_UW5	0	0	1000	0	1000	0	1000	0	0	0
Enterobacter_mori_800721_17	0	0	1000	0	1000	0	1000	0	0	0
Enterobacter_mori_LMG_25706	0	0	1000	0	1000	0	1000	0	0	0
Enterobacter_nov_ERR2221249	0	0	0	0	1000	0	1000	0	0	0
Enterobacter_nov_ERR2221289	0	0	0	0	1000	0	1000	0	0	0
Enterobacter_nov_ERR2221350	0	0	0	0	1000	0	1000	0	0	0
Enterobacter_nov_ERR2221354	0	0	1000	0	1000	0	1000	0	0	0
Enterobacter_roggenkampii_35734	0	0	1000	0	1000	0	1000	0	0	0
Enterobacter_sp_MGH_8	0	0	1000	0	1000	0	1000	0	0	0
Enterobacteriaceae_bacterium_9_2_54FAA	0	0	0	0	1000	0	1000	0	0	0
Enterococcus_asini_ATCC_700915	0	0	0	0	0	0	1000	0	0	0
Enterococcus_avium_ATCC_14025	0	1000	0	0	0	0	1000	0	1000	0
Enterococcus_caccae_ATCC_BAA_1240	0	0	0	0	0	0	1000	0	1000	0
Enterococcus_casseliflavus_ATCC_12755	0	0	0	0	1000	0	1000	0	0	0
Enterococcus_casseliflavus_EC10	0	0	0	0	1000	0	1000	0	0	0
Enterococcus_casseliflavus_EC20	0	0	0	0	1000	0	1000	0	0	0
Enterococcus_casseliflavus_EC30	0	0	0	0	1000	0	1000	0	0	0
Enterococcus_cecorum_DSM_20682	0	0	0	1000	0	0	1000	0	1000	1000
Enterococcus_dispar_ATCC_51266	0	0	0	0	0	0	1000	0	1000	0
Enterococcus_durans_ATCC_6056	0	0	0	0	0	0	1000	0	0	0
Enterococcus_durans_ERR2230121	0	0	0	0	0	0	1000	0	0	0
Enterococcus_durans_FB129_CNAB_4	0	0	0	0	0	0	1000	0	0	0
Enterococcus_durans_IPLA_655	0	0	0	0	1000	0	1000	0	0	0
Enterococcus_faecalis_12030	0	0	0	0	1000	0	1000	0	1000	0
Enterococcus_faecalis_12107	0	0	0	0	1000	0	1000	0	1000	0
Enterococcus_faecalis_1448E03	0	0	0	0	1000	0	1000	0	1000	0
Enterococcus_faecalis_182970	0	0	0	0	1000	0	1000	0	1000	0
Enterococcus_faecalis_19116	0	0	0	0	1000	0	1000	0	1000	0
Enterococcus_faecalis_2630V05	0	0	0	0	1000	0	1000	0	1000	0
Enterococcus_faecalis_2924	0	0	0	0	1000	0	1000	0	1000	0
Enterococcus_faecalis_5952	0	0	0	0	1000	0	1000	0	1000	0
Enterococcus_faecalis_599	0	0	0	0	1000	0	1000	0	1000	0
Enterococcus_faecalis_599951	0	0	0	0	1000	0	1000	0	1000	0
Enterococcus_faecalis_62	0	0	0	0	1000	0	1000	0	1000	0

Enterococcus_faecalis_7330082_2	0	0	0	0	1000	0	1000	0	1000	0
Enterococcus_faecalis_7330112_3	0	0	0	0	1000	0	1000	0	1000	0
Enterococcus_faecalis_7330245_2	0	0	0	0	1000	0	1000	0	1000	0
Enterococcus_faecalis_7330257_1	0	0	0	0	1000	0	1000	0	1000	0
Enterococcus_faecalis_7330259_5	0	0	0	0	1000	0	1000	0	1000	0
Enterococcus_faecalis_7330948_5	0	0	0	0	1000	0	1000	0	1000	0
Enterococcus_faecalis_7430275_3	0	0	0	0	1000	0	1000	0	1000	0
Enterococcus_faecalis_7430315_3	0	0	0	0	1000	0	1000	0	1000	0
Enterococcus_faecalis_7430416_3	0	0	0	0	1000	0	1000	0	1000	0
Enterococcus_faecalis_7430821_4	0	0	0	0	1000	0	1000	0	1000	0
Enterococcus_faecalis_79_3	0	0	0	0	1000	0	1000	0	1000	0
Enterococcus_faecalis_A_2_1	0	0	0	0	1000	0	1000	0	1000	0
Enterococcus_faecalis_A_3_1	0	0	0	0	1000	0	1000	0	1000	0
Enterococcus_faecalis_AR01_DG	0	0	0	0	1000	0	1000	0	1000	0
Enterococcus_faecalis_ATCC_10100	0	0	0	0	1000	0	1000	0	1000	0
Enterococcus_faecalis_ATCC_19433	0	0	0	0	1000	0	1000	0	1000	0
Enterococcus_faecalis_ATCC_27275	0	0	0	0	1000	0	1000	0	1000	0
Enterococcus_faecalis_ATCC_27959	0	0	0	0	1000	0	1000	0	1000	0
Enterococcus_faecalis_ATCC_29200	0	0	0	0	1000	0	1000	0	1000	0
Enterococcus_faecalis_ATCC_29212	0	0	0	0	1000	0	1000	0	1000	0
Enterococcus_faecalis_ATCC_35038	0	0	0	0	1000	0	1000	0	1000	0
Enterococcus_faecalis_ATCC_4200	0	0	0	0	1000	0	1000	0	1000	0
Enterococcus_faecalis_ATCC_6055	0	0	0	0	1000	0	1000	0	1000	0
Enterococcus_faecalis_B_4_111	0	0	0	0	1000	0	1000	0	1000	0
Enterococcus_faecalis_B1005	0	0	0	0	1000	0	1000	0	1000	0
Enterococcus_faecalis_B1138	0	0	0	0	1000	0	1000	0	1000	0
Enterococcus_faecalis_B1249	0	0	0	0	1000	0	1000	0	1000	0
Enterococcus_faecalis_B1290	0	0	0	0	1000	0	1000	0	1000	0
Enterococcus_faecalis_B1327	0	0	0	0	1000	0	1000	0	1000	0
Enterococcus_faecalis_B1376	0	0	0	0	1000	0	1000	0	1000	0
Enterococcus_faecalis_B1385	0	0	0	0	1000	0	1000	0	1000	0
Enterococcus_faecalis_B1441	0	0	0	0	1000	0	1000	0	1000	0
Enterococcus_faecalis_B1505	0	0	0	0	1000	0	1000	0	1000	0
Enterococcus_faecalis_B1532	0	0	0	0	1000	0	1000	0	1000	0
Enterococcus_faecalis_B15725	0	0	0	0	1000	0	1000	0	1000	0
Enterococcus_faecalis_B1586	0	0	0	0	1000	0	1000	0	1000	0
Enterococcus_faecalis_B1618	0	0	0	0	1000	0	1000	0	1000	0
Enterococcus_faecalis_B1623	0	0	0	0	1000	0	1000	0	1000	0
Enterococcus_faecalis_B16457	0	0	0	0	1000	0	1000	0	1000	0
Enterococcus_faecalis_B1678	0	0	0	0	1000	0	1000	0	1000	0
Enterococcus_faecalis_B1696	0	0	0	0	1000	0	1000	0	1000	0
Enterococcus_faecalis_B1719	0	0	0	0	1000	0	1000	0	1000	0
Enterococcus_faecalis_B1734	0	0	0	0	1000	0	1000	0	1000	0
Enterococcus_faecalis_B1843	0	0	0	0	1000	0	1000	0	1000	0
Enterococcus_faecalis_B1851	0	0	0	0	1000	0	1000	0	1000	0
Enterococcus_faecalis_B1874	0	0	0	0	1000	0	1000	0	1000	0
Enterococcus_faecalis_B1921	0	0	0	0	1000	0	1000	0	1000	0
Enterococcus_faecalis_B1933	0	0	0	0	1000	0	1000	0	1000	0
Enterococcus_faecalis_B2202	0	0	0	0	1000	0	1000	0	1000	0
Enterococcus_faecalis_B2207	0	0	0	0	1000	0	1000	0	1000	0
Enterococcus_faecalis_B2211	0	0	0	0	1000	0	1000	0	1000	0
Enterococcus_faecalis_B2255	0	0	0	0	1000	0	1000	0	1000	0
Enterococcus_faecalis_B2277	0	0	0	0	1000	0	1000	0	1000	0
Enterococcus_faecalis_B2391	0	0	0	0	1000	0	1000	0	1000	0
Enterococcus_faecalis_B2488	0	0	0	0	1000	0	1000	0	1000	0
Enterococcus_faecalis_B2535	0	0	0	0	1000	0	1000	0	1000	0
Enterococcus_faecalis_B2557	0	0	0	0	1000	0	1000	0	1000	0
Enterococcus_faecalis_B2593	0	0	0	0	1000	0	1000	0	1000	0
Enterococcus_faecalis_B2670	0	0	0	0	1000	0	1000	0	1000	0
Enterococcus_faecalis_B2685	0	0	0	0	1000	0	1000	0	1000	0
Enterococcus_faecalis_B2687	0	0	0	0	1000	0	1000	0	1000	0
Enterococcus_faecalis_B2802	0	0	0	0	1000	0	1000	0	1000	0
Enterococcus_faecalis_B2864	0	0	0	0	1000	0	1000	0	1000	0
Enterococcus_faecalis_B2867	0	0	0	0	1000	0	1000	0	1000	0
Enterococcus_faecalis_B2949	0	0	0	0	1000	0	1000	0	1000	0
Enterococcus_faecalis_B3031	0	0	0	0	1000	0	1000	0	1000	0
Enterococcus_faecalis_B3042	0	0	0	0	1000	0	1000	0	1000	0
Enterococcus_faecalis_B3053	0	0	0	0	1000	0	1000	0	1000	0
Enterococcus_faecalis_B3119	0	0	0	0	1000	0	1000	0	1000	0
Enterococcus_faecalis_B3126	0	0	0	0	1000	0	1000	0	1000	0
Enterococcus_faecalis_B3196	0	0	0	0	1000	0	1000	0	1000	0
Enterococcus_faecalis_B3286	0	0	0	0	1000	0	1000	0	1000	0
Enterococcus_faecalis_B3336	0	0	0	0	1000	0	1000	0	1000	0
Enterococcus_faecalis_B4008	0	0	0	0	1000	0	1000	0	1000	0
Enterococcus_faecalis_B4018	0	0	0	0	1000	0	1000	0	1000	0
Enterococcus_faecalis_B4148	0	0	0	0	1000	0	1000	0	1000	0
Enterococcus_faecalis_B4163	0	0	0	0	1000	0	1000	0	1000	0
Enterococcus_faecalis_B4259	0	0	0	0	1000	0	1000	0	1000	0
Enterococcus_faecalis_B4267	0	0	0	0	1000	0	1000	0	1000	0
Enterococcus_faecalis_B4270	0	0	0	0	1000	0	1000	0	1000	0
Enterococcus_faecalis_B4411	0	0	0	0	1000	0	1000	0	1000	0
Enterococcus_faecalis_B4568	0	0	0	0	1000	0	1000	0	1000	0
Enterococcus_faecalis_B4638	0	0	0	0	1000	0	1000	0	1000	0
Enterococcus_faecalis_B4672	0	0	0	0	1000	0	1000	0	1000	0
Enterococcus_faecalis_B4674	0	0	0	0	1000	0	1000	0	1000	0
Enterococcus_faecalis_B4969	0	0	0	0	1000	0	1000	0	1000	0
Enterococcus_faecalis_B5035	0	0	0	0	1000	0	1000	0	1000	0
Enterococcus_faecalis_B5076	0	0	0	0	1000	0	1000	0	1000	0
Enterococcus_faecalis_B56765	0	0	0	0	1000	0	1000	0	1000	0
Enterococcus_faecalis_B594	0	0	0	0	1000	0	1000	0	1000	0
Enterococcus_faecalis_B653	0	0	0	0	1000	0	1000	0	1000	0
Enterococcus_faecalis_B69486	0	0	0	0	1000	0	1000	0	1000	0
Enterococcus_faecalis_B84847	0	0	0	0	1000	0	1000	0	1000	0
Enterococcus_faecalis_B878	0	0	0	0	1000	0	1000	0	1000	0

Enterococcus_faecalis_B939	0	0	0	0	1000	0	1000	0	1000	0
Enterococcus_faecalis_C19315WT	0	0	0	1000	1000	0	1000	0	1000	0
Enterococcus_faecalis_CH116	0	0	0	0	1000	0	1000	0	1000	0
Enterococcus_faecalis_CH136	0	0	0	0	1000	0	1000	0	1000	0
Enterococcus_faecalis_CH188	0	0	0	0	1000	0	1000	0	1000	0
Enterococcus_faecalis_CH19	0	0	0	0	1000	0	1000	0	1000	0
Enterococcus_faecalis_CH570	0	0	0	0	1000	0	1000	0	1000	0
Enterococcus_faecalis_Com_2	0	0	0	0	1000	0	1000	0	1000	0
Enterococcus_faecalis_Com_6	0	0	0	0	1000	0	1000	0	1000	0
Enterococcus_faecalis_Com1	0	0	0	0	1000	0	1000	0	1000	0
Enterococcus_faecalis_Com7	0	0	0	0	1000	0	1000	0	1000	0
Enterococcus_faecalis_D1	0	0	0	0	1000	0	1000	0	1000	0
Enterococcus_faecalis_D173	0	0	0	0	1000	0	1000	0	1000	0
Enterococcus_faecalis_D3	0	0	0	0	1000	0	1000	0	1000	0
Enterococcus_faecalis_D32	0	0	0	0	1000	0	1000	0	1000	0
Enterococcus_faecalis_D6	0	0	0	0	1000	0	1000	0	1000	0
Enterococcus_faecalis_DAPTO_512	0	0	0	0	1000	0	1000	0	1000	0
Enterococcus_faecalis_DAPTO_516	0	0	0	0	1000	0	1000	0	1000	0
Enterococcus_faecalis_DS16	0	0	0	0	1000	0	1000	0	1000	0
Enterococcus_faecalis_DS5	0	0	0	0	1000	0	1000	0	1000	0
Enterococcus_faecalis_E1	0	0	0	0	1000	0	1000	0	1000	0
Enterococcus_faecalis_E1Sol	0	0	0	0	1000	0	1000	0	1000	0
Enterococcus_faecalis_E99	0	0	0	0	1000	0	1000	0	1000	0
Enterococcus_faecalis_EnGen0253	0	0	0	0	1000	0	1000	0	1000	0
Enterococcus_faecalis_ERR1203921	0	0	0	0	1000	0	1000	0	1000	0
Enterococcus_faecalis_ERR1203927	0	0	0	0	1000	0	1000	0	1000	0
Enterococcus_faecalis_ERR1203928	0	0	0	0	1000	0	1000	0	1000	0
Enterococcus_faecalis_ERR1203931	0	0	0	0	1000	0	1000	0	1000	0
Enterococcus_faecalis_ERR1204022	0	0	0	0	1000	0	1000	0	1000	0
Enterococcus_faecalis_ERR1204025	0	0	0	0	1000	0	1000	0	1000	0
Enterococcus_faecalis_ERR2221203	0	0	0	0	1000	0	1000	0	1000	0
Enterococcus_faecalis_ERR2221223	0	0	0	0	1000	0	1000	0	1000	0
Enterococcus_faecalis_ERR2221228	0	0	0	0	1000	0	1000	0	1000	0
Enterococcus_faecalis_ERR2221234	0	0	0	0	1000	0	1000	0	1000	0
Enterococcus_faecalis_ERR2221236	0	0	0	0	1000	0	1000	0	1000	0
Enterococcus_faecalis_ERR2221251	0	0	0	0	1000	0	1000	0	1000	0
Enterococcus_faecalis_ERR2221313	0	0	0	0	1000	0	1000	0	1000	0
Enterococcus_faecalis_ERR2221314	0	0	0	0	1000	0	1000	0	1000	0
Enterococcus_faecalis_ERR2221315	0	0	0	0	1000	0	1000	0	1000	0
Enterococcus_faecalis_ERR2221341	0	0	0	0	1000	0	1000	0	1000	0
Enterococcus_faecalis_ERR2221346	0	0	0	0	1000	0	1000	0	1000	0
Enterococcus_faecalis_ERR2230084	0	0	0	0	1000	0	1000	0	1000	0
Enterococcus_faecalis_ERR2230105	0	0	0	0	1000	0	1000	0	1000	0
Enterococcus_faecalis_ERR2230117	0	0	0	0	1000	0	1000	0	1000	0
Enterococcus_faecalis_ERR2230123	0	0	0	0	1000	0	1000	0	1000	0
Enterococcus_faecalis_ERR2230129	0	0	0	0	1000	0	1000	0	1000	0
Enterococcus_faecalis_ERV103	0	0	0	0	1000	0	1000	0	1000	0
Enterococcus_faecalis_ERV116	0	0	0	0	1000	0	1000	0	1000	0
Enterococcus_faecalis_ERV129	0	0	0	0	1000	0	1000	0	1000	0
Enterococcus_faecalis_ERV25	0	0	0	0	1000	0	1000	0	1000	0
Enterococcus_faecalis_ERV31	0	0	0	0	1000	0	1000	0	1000	0
Enterococcus_faecalis_ERV37	0	0	0	0	1000	0	1000	0	1000	0
Enterococcus_faecalis_ERV41	0	0	0	0	1000	0	1000	0	1000	0
Enterococcus_faecalis_ERV62	0	0	0	0	1000	0	1000	0	1000	0
Enterococcus_faecalis_ERV63	0	0	0	0	1000	0	1000	0	1000	0
Enterococcus_faecalis_ERV65	0	0	0	0	1000	0	1000	0	1000	0
Enterococcus_faecalis_ERV68	0	0	0	0	1000	0	1000	0	1000	0
Enterococcus_faecalis_ERV72	0	0	0	0	1000	0	1000	0	1000	0
Enterococcus_faecalis_ERV81	0	0	0	0	1000	0	1000	0	1000	0
Enterococcus_faecalis_ERV85	0	0	0	0	1000	0	1000	0	1000	0
Enterococcus_faecalis_ERV93	0	0	0	0	1000	0	1000	0	1000	0
Enterococcus_faecalis_F1	0	0	0	0	1000	0	1000	0	1000	0
Enterococcus_faecalis_FA2_2	0	0	0	0	1000	0	1000	0	1000	0
Enterococcus_faecalis_Fly_2	0	0	0	0	1000	0	1000	0	1000	0
Enterococcus_faecalis_Fly1	0	0	0	0	1000	0	1000	0	1000	0
Enterococcus_faecalis_HEF39	0	0	0	0	1000	0	1000	0	1000	0
Enterococcus_faecalis_HH22	0	0	0	0	1000	0	1000	0	1000	0
Enterococcus_faecalis_HIP11704	0	0	0	0	1000	0	1000	0	1000	0
Enterococcus_faecalis_JH1	0	0	0	0	1000	0	1000	0	1000	0
Enterococcus_faecalis_KB1	0	0	0	0	1000	0	1000	0	1000	0
Enterococcus_faecalis_M7	0	0	0	0	1000	0	1000	0	1000	0
Enterococcus_faecalis_Merz151	0	0	0	0	1000	0	1000	0	1000	0
Enterococcus_faecalis_Merz192	0	0	0	0	1000	0	1000	0	1000	0
Enterococcus_faecalis_Merz204	0	0	0	0	1000	0	1000	0	1000	0
Enterococcus_faecalis_Merz89	0	0	0	0	1000	0	1000	0	1000	0
Enterococcus_faecalis_Merz96	0	0	0	0	1000	0	1000	0	1000	0
Enterococcus_faecalis_MMH594	0	0	0	0	1000	0	1000	0	1000	0
Enterococcus_faecalis_Ned10	0	0	0	0	1000	0	1000	0	1000	0
Enterococcus_faecalis_OG1RF_ATCC_47077	0	0	0	0	1000	0	1000	0	1000	0
Enterococcus_faecalis_OG1X	0	0	0	0	1000	0	1000	0	1000	0
Enterococcus_faecalis_Pan7	0	0	0	0	1000	0	1000	0	1000	0
Enterococcus_faecalis_PC1_1	0	0	0	0	1000	0	1000	0	1000	0
Enterococcus_faecalis_R508	0	0	0	0	1000	0	1000	0	1000	0
Enterococcus_faecalis_R712	0	0	0	0	1000	0	1000	0	1000	0
Enterococcus_faecalis_RC73	0	0	0	0	1000	0	1000	0	1000	0
Enterococcus_faecalis_RM3817	0	0	0	0	1000	0	1000	0	1000	0
Enterococcus_faecalis_RM4679	0	0	0	0	1000	0	1000	0	1000	0
Enterococcus_faecalis_RMC1	0	0	0	0	1000	0	1000	0	1000	0
Enterococcus_faecalis_RMC5	0	0	0	0	1000	0	1000	0	1000	0
Enterococcus_faecalis_RMC65	0	0	0	0	1000	0	1000	0	1000	0
Enterococcus_faecalis_S613	0	0	0	0	1000	0	1000	0	1000	0
Enterococcus_faecalis_SF100	0	0	0	0	1000	0	1000	0	1000	0
Enterococcus_faecalis_SF105	0	0	0	0	1000	0	1000	0	1000	0
Enterococcus_faecalis_SF1592	0	0	0	0	1000	0	1000	0	1000	0

Enterococcus_faecalis_SF19	0	0	0	0	1000	0	1000	0	1000	0
Enterococcus_faecalis_SF21520	0	0	0	0	1000	0	1000	0	1000	0
Enterococcus_faecalis_SF21521	0	0	0	0	1000	0	1000	0	1000	0
Enterococcus_faecalis_SF24396	0	0	0	0	1000	0	1000	0	1000	0
Enterococcus_faecalis_SF24397	0	0	0	0	1000	0	1000	0	1000	0
Enterococcus_faecalis_SF24413	0	0	0	0	1000	0	1000	0	1000	0
Enterococcus_faecalis_SF26630	0	0	0	0	1000	0	1000	0	1000	0
Enterococcus_faecalis_SF28073	0	0	0	0	1000	0	1000	0	1000	0
Enterococcus_faecalis_SF339	0	0	0	0	1000	0	1000	0	1000	0
Enterococcus_faecalis_SF350	0	0	0	0	1000	0	1000	0	1000	0
Enterococcus_faecalis_SF370	0	0	0	0	1000	0	1000	0	1000	0
Enterococcus_faecalis_SF5039	0	0	0	0	1000	0	1000	0	1000	0
Enterococcus_faecalis_SF6375	0	0	0	0	1000	0	1000	0	1000	0
Enterococcus_faecalis_SS_6	0	0	0	0	1000	0	1000	0	1000	0
Enterococcus_faecalis_SS_7	0	0	0	0	1000	0	1000	0	1000	0
Enterococcus_faecalis_str_C_19315_led_1b_pp_S	0	0	0	1000	1000	0	1000	0	1000	0
Enterococcus_faecalis_str_Symbioflor_1	0	0	0	0	1000	0	1000	0	1000	0
Enterococcus_faecalis_T1	0	0	0	0	1000	0	1000	0	1000	0
Enterococcus_faecalis_T10	0	0	0	0	1000	0	1000	0	1000	0
Enterococcus_faecalis_T11	0	0	0	0	1000	0	1000	0	1000	0
Enterococcus_faecalis_T12	0	0	0	0	1000	0	1000	0	1000	0
Enterococcus_faecalis_T13	0	0	0	0	1000	0	1000	0	1000	0
Enterococcus_faecalis_T14	0	0	0	0	1000	0	1000	0	1000	0
Enterococcus_faecalis_T16	0	0	0	0	1000	0	1000	0	1000	0
Enterococcus_faecalis_T17	0	0	0	0	1000	0	1000	0	1000	0
Enterococcus_faecalis_T18	0	0	0	0	1000	0	1000	0	1000	0
Enterococcus_faecalis_T19	0	0	0	0	1000	0	1000	0	1000	0
Enterococcus_faecalis_T2	0	0	0	0	1000	0	1000	0	1000	0
Enterococcus_faecalis_T20	0	0	0	0	1000	0	1000	0	1000	0
Enterococcus_faecalis_T21	0	0	0	0	1000	0	1000	0	1000	0
Enterococcus_faecalis_T3	0	0	0	0	1000	0	1000	0	1000	0
Enterococcus_faecalis_T4	0	0	0	0	1000	0	1000	0	1000	0
Enterococcus_faecalis_T5	0	0	0	0	1000	0	1000	0	1000	0
Enterococcus_faecalis_T6	0	0	0	0	1000	0	1000	0	1000	0
Enterococcus_faecalis_T7	0	0	0	0	1000	0	1000	0	1000	0
Enterococcus_faecalis_T8	0	0	0	0	1000	0	1000	0	1000	0
Enterococcus_faecalis_T9	0	0	0	0	1000	0	1000	0	1000	0
Enterococcus_faecalis_TR161	0	0	0	0	1000	0	1000	0	1000	0
Enterococcus_faecalis_TR197	0	0	0	0	1000	0	1000	0	1000	0
Enterococcus_faecalis_TUSoD_Ef11	0	0	0	0	1000	0	1000	0	1000	0
Enterococcus_faecalis_TX0012	0	0	0	0	1000	0	1000	0	1000	0
Enterococcus_faecalis_TX0017	0	0	0	0	1000	0	1000	0	1000	0
Enterococcus_faecalis_TX0027	0	0	0	0	1000	0	1000	0	1000	0
Enterococcus_faecalis_TX0031	0	0	0	0	1000	0	1000	0	1000	0
Enterococcus_faecalis_TX0043	0	0	0	0	1000	0	1000	0	1000	0
Enterococcus_faecalis_TX0102	0	0	0	0	1000	0	1000	0	1000	0
Enterococcus_faecalis_TX0104	0	0	0	0	1000	0	1000	0	1000	0
Enterococcus_faecalis_TX0109	0	0	0	0	1000	0	1000	0	1000	0
Enterococcus_faecalis_TX0309A	0	0	0	0	1000	0	1000	0	1000	0
Enterococcus_faecalis_TX0309B	0	0	0	0	1000	0	1000	0	1000	0
Enterococcus_faecalis_TX0312	0	0	0	0	1000	0	1000	0	1000	0
Enterococcus_faecalis_TX0411	0	0	0	0	1000	0	1000	0	1000	0
Enterococcus_faecalis_TX0470	0	0	0	0	1000	0	1000	0	1000	0
Enterococcus_faecalis_TX0630	0	0	0	0	1000	0	1000	0	1000	0
Enterococcus_faecalis_TX0635_WH245	0	0	0	0	1000	0	1000	0	1000	0
Enterococcus_faecalis_TX0645	0	0	0	0	1000	0	1000	0	1000	0
Enterococcus_faecalis_TX0855	0	0	0	0	1000	0	1000	0	1000	0
Enterococcus_faecalis_TX0860	0	0	0	0	1000	0	1000	0	1000	0
Enterococcus_faecalis_TX1302	0	0	0	0	1000	0	1000	0	1000	0
Enterococcus_faecalis_TX1322	0	0	0	0	1000	0	1000	0	1000	0
Enterococcus_faecalis_TX1337RF	0	0	0	0	0	0	1000	0	1000	0
Enterococcus_faecalis_TX1341	0	0	0	0	1000	0	1000	0	1000	0
Enterococcus_faecalis_TX1342	0	0	0	0	1000	0	1000	0	1000	0
Enterococcus_faecalis_TX1346	0	0	0	0	1000	0	1000	0	1000	0
Enterococcus_faecalis_TX2134	0	0	0	0	1000	0	1000	0	1000	0
Enterococcus_faecalis_TX2137	0	0	0	0	1000	0	1000	0	1000	0
Enterococcus_faecalis_TX2141	0	0	0	0	1000	0	1000	0	1000	0
Enterococcus_faecalis_TX4000	0	0	0	0	1000	0	1000	0	1000	0
Enterococcus_faecalis_TX4244	0	0	0	0	1000	0	1000	0	1000	0
Enterococcus_faecalis_TX4248	0	0	0	0	1000	0	1000	0	1000	0
Enterococcus_faecalis_UAA1014	0	0	0	0	1000	0	1000	0	1000	0
Enterococcus_faecalis_UAA1180	0	0	0	0	1000	0	1000	0	1000	0
Enterococcus_faecalis_UAA1489	0	0	0	0	1000	0	1000	0	1000	0
Enterococcus_faecalis_UAA409	0	0	0	0	1000	0	1000	0	1000	0
Enterococcus_faecalis_UAA702	0	0	0	1000	1000	0	1000	0	1000	0
Enterococcus_faecalis_UAA769	0	0	0	0	1000	0	1000	0	1000	0
Enterococcus_faecalis_UAA823	0	0	0	0	1000	0	1000	0	1000	0
Enterococcus_faecalis_UAA902	0	0	0	0	1000	0	1000	0	1000	0
Enterococcus_faecalis_UAA903	0	0	0	0	1000	0	1000	0	1000	0
Enterococcus_faecalis_UAA904	0	0	0	0	1000	0	1000	0	1000	0
Enterococcus_faecalis_UAA905	0	0	0	0	1000	0	1000	0	1000	0
Enterococcus_faecalis_UAA906	0	0	0	0	1000	0	1000	0	1000	0
Enterococcus_faecalis_UAA907	0	0	0	0	1000	0	1000	0	1000	0
Enterococcus_faecalis_UAA943	0	0	0	0	1000	0	1000	0	1000	0
Enterococcus_faecalis_UAA948	0	0	0	0	1000	0	1000	0	1000	0
Enterococcus_faecalis_V583	0	0	0	0	1000	0	1000	0	1000	0
Enterococcus_faecalis_V587	0	0	0	0	1000	0	1000	0	1000	0
Enterococcus_faecalis_WH257	0	0	0	0	1000	0	1000	0	1000	0
Enterococcus_faecalis_WH571	0	0	0	0	1000	0	1000	0	1000	0
Enterococcus_faecalis_X98	0	0	0	0	1000	0	1000	0	1000	0
Enterococcus_faecalis_YI6_1	0	0	0	0	1000	0	1000	0	1000	0
Enterococcus_faecium_109_A1	0	0	0	0	0	0	1000	0	0	0
Enterococcus_faecium_1141733	0	0	0	0	0	0	1000	0	0	0
Enterococcus_faecium_1230933	0	0	0	0	0	0	1000	0	0	0

Enterococcus_faecium_1231408	0	0	0	0	0	0	1000	0	0	0
Enterococcus_faecium_1231410	0	0	0	0	0	0	1000	0	0	0
Enterococcus_faecium_1231501	0	0	0	0	0	0	1000	0	0	0
Enterococcus_faecium_1231502	0	0	0	0	0	0	1000	0	0	0
Enterococcus_faecium_2006_70_121	0	0	0	0	0	0	1000	0	0	0
Enterococcus_faecium_504	0	0	0	0	0	0	1000	0	0	0
Enterococcus_faecium_505	0	0	0	0	0	0	1000	0	0	0
Enterococcus_faecium_7330381_1	0	0	0	0	0	0	1000	0	0	0
Enterococcus_faecium_7330446_2	0	0	0	0	0	0	1000	0	0	0
Enterococcus_faecium_7330519_3	0	0	0	0	0	0	1000	0	0	0
Enterococcus_faecium_7330614_1	0	0	0	0	0	0	1000	0	0	0
Enterococcus_faecium_7330884_2	0	0	0	0	0	0	1000	0	0	0
Enterococcus_faecium_7430166_3	0	0	0	0	0	0	1000	0	0	0
Enterococcus_faecium_841V03	0	0	0	0	0	0	1000	0	0	0
Enterococcus_faecium_9730219_1	0	0	0	0	0	0	1000	0	0	0
Enterococcus_faecium_9730357_1	0	0	0	0	0	0	1000	0	0	0
Enterococcus_faecium_9731349_1	0	0	0	0	0	0	1000	0	0	0
Enterococcus_faecium_9731352_4	0	0	0	0	0	0	1000	0	0	0
Enterococcus_faecium_9830091_5	0	0	0	0	0	0	1000	0	0	0
Enterococcus_faecium_9830512_2	0	0	0	0	0	0	1000	0	0	0
Enterococcus_faecium_9830565_4	0	0	0	0	0	0	1000	0	0	0
Enterococcus_faecium_9930238_2	0	0	0	0	0	0	1000	0	0	0
Enterococcus_faecium_9931110_4	0	0	0	0	0	0	1000	0	0	0
Enterococcus_faecium_A17_Sv1	0	0	0	0	0	0	1000	0	0	0
Enterococcus_faecium_ATCC_8459	0	0	0	0	0	0	1000	0	0	0
Enterococcus_faecium_Aus0004	0	0	0	0	0	0	1000	0	0	0
Enterococcus_faecium_C309	0	0	0	0	0	0	1000	0	0	0
Enterococcus_faecium_C68	0	0	0	0	0	0	1000	0	0	0
Enterococcus_faecium_Com12	0	0	0	0	0	0	1000	0	0	0
Enterococcus_faecium_Com15	0	0	0	0	0	0	1000	0	0	0
Enterococcus_faecium_CRL1879	0	0	0	0	0	0	1000	0	0	0
Enterococcus_faecium_D344SRF	0	0	0	0	0	0	1000	0	0	0
Enterococcus_faecium_DO	0	0	0	0	0	0	1000	0	0	0
Enterococcus_faecium_E0045	0	0	0	0	0	0	1000	0	0	0
Enterococcus_faecium_E0120	0	0	0	0	0	0	1000	0	0	0
Enterococcus_faecium_E0164	0	0	0	0	0	0	1000	0	0	0
Enterococcus_faecium_E0269	0	0	0	0	0	0	1000	0	0	0
Enterococcus_faecium_E0333	0	0	0	0	0	0	1000	0	0	0
Enterococcus_faecium_E0679	0	0	0	0	0	0	1000	0	0	0
Enterococcus_faecium_E0680	0	0	0	0	0	0	1000	0	0	0
Enterococcus_faecium_E0688	0	0	0	0	0	0	1000	0	0	0
Enterococcus_faecium_E1007	0	0	0	0	0	0	1000	0	0	0
Enterococcus_faecium_E1039	0	0	0	0	0	0	1000	0	0	0
Enterococcus_faecium_E1050	0	0	0	0	0	0	1000	0	0	0
Enterococcus_faecium_E1071	0	0	0	0	0	0	1000	0	0	0
Enterococcus_faecium_E1133	0	0	0	0	0	0	1000	0	0	0
Enterococcus_faecium_E1162	0	0	0	0	0	0	1000	0	0	0
Enterococcus_faecium_E1185	0	0	0	0	0	0	1000	0	0	0
Enterococcus_faecium_E1258	0	0	0	0	0	0	1000	0	0	0
Enterococcus_faecium_E1293	0	0	0	0	0	0	1000	0	0	0
Enterococcus_faecium_E1321	0	0	0	0	0	0	1000	0	0	0
Enterococcus_faecium_E1392	0	0	0	0	0	0	1000	0	0	0
Enterococcus_faecium_E1552	0	0	0	0	0	0	1000	0	0	0
Enterococcus_faecium_E1573	0	0	0	0	0	0	1000	0	0	0
Enterococcus_faecium_E1574	0	0	0	0	0	0	1000	0	0	0
Enterococcus_faecium_E1575	0	0	0	0	0	0	1000	0	0	0
Enterococcus_faecium_E1576	0	0	0	0	0	0	1000	0	0	0
Enterococcus_faecium_E1578	0	0	0	0	0	0	1000	0	0	0
Enterococcus_faecium_E1590	0	0	0	0	0	0	1000	0	0	0
Enterococcus_faecium_E1604	0	0	0	0	0	0	1000	0	0	0
Enterococcus_faecium_E1613	0	0	0	0	0	0	1000	0	0	0
Enterococcus_faecium_E1620	0	0	0	0	0	0	1000	0	0	0
Enterococcus_faecium_E1622	0	0	0	0	0	0	1000	0	0	0
Enterococcus_faecium_E1623	0	0	0	0	0	0	1000	0	0	0
Enterococcus_faecium_E1626	0	0	0	0	0	0	1000	0	0	0
Enterococcus_faecium_E1627	0	0	0	0	0	0	1000	0	0	0
Enterococcus_faecium_E1630	0	0	0	0	0	0	1000	0	0	0
Enterococcus_faecium_E1634	0	0	0	0	0	0	1000	0	0	0
Enterococcus_faecium_E1636	0	0	0	0	0	0	1000	0	0	0
Enterococcus_faecium_E1644	0	0	0	0	0	0	1000	0	0	0
Enterococcus_faecium_E1731	0	0	0	0	0	0	1000	0	0	0
Enterococcus_faecium_E1861	0	0	0	0	0	0	1000	0	0	0
Enterococcus_faecium_E1904	0	0	0	0	0	0	1000	0	0	0
Enterococcus_faecium_E1972	0	0	0	0	0	0	1000	0	0	0
Enterococcus_faecium_E2039	0	0	0	0	0	0	1000	0	0	0
Enterococcus_faecium_E2071	0	0	0	0	0	0	1000	0	0	0
Enterococcus_faecium_E2134	0	0	0	0	0	0	1000	0	0	0
Enterococcus_faecium_E2297	0	0	0	0	0	0	1000	0	0	0
Enterococcus_faecium_E2369	0	0	0	0	0	0	1000	0	0	0
Enterococcus_faecium_E2560	0	0	0	0	0	0	1000	0	0	0
Enterococcus_faecium_E2620	0	0	0	0	0	0	1000	0	0	0
Enterococcus_faecium_E2883	0	0	0	0	0	0	1000	0	0	0
Enterococcus_faecium_E2966	0	0	0	0	0	0	1000	0	0	0
Enterococcus_faecium_E3083	0	0	0	0	0	0	1000	0	0	0
Enterococcus_faecium_E3346	0	0	0	0	0	0	1000	0	0	0
Enterococcus_faecium_E3548	0	0	0	0	0	0	1000	0	0	0
Enterococcus_faecium_E4215	0	0	0	0	0	0	1000	0	0	0
Enterococcus_faecium_E4389	0	0	0	0	0	0	1000	0	0	0
Enterococcus_faecium_E6012	0	0	0	1000	0	0	1000	0	0	0
Enterococcus_faecium_E6045	0	0	0	1000	0	0	1000	0	0	0
Enterococcus_faecium_E8sv3	0	0	0	0	0	0	1000	0	0	0
Enterococcus_faecium_E980	0	0	0	0	0	0	1000	0	0	0
Enterococcus_faecium_EnGen0305	0	0	0	0	0	0	1000	0	0	0
Enterococcus_faecium_EnGen0308	0	0	0	0	0	0	1000	0	0	0

Enterococcus_faecium_EnGen0312	0	0	0	0	0	0	1000	0	0	0
Enterococcus_faecium_EnGen0314	0	0	0	0	0	0	1000	0	0	0
Enterococcus_faecium_EnGen0316	0	0	0	0	0	0	1000	0	0	0
Enterococcus_faecium_EnGen0318	0	0	0	0	0	0	1000	0	0	0
Enterococcus_faecium_EnGen0319	0	0	0	0	0	0	1000	0	0	0
Enterococcus_faecium_EnGen0321	0	0	0	0	0	0	1000	0	0	0
Enterococcus_faecium_EnGen0323	0	0	0	0	0	0	1000	0	0	0
Enterococcus_faecium_EnGen0375	0	0	0	0	0	0	1000	0	0	0
Enterococcus_faecium_EnGen0376	0	0	0	0	0	0	1000	0	0	0
Enterococcus_faecium_EnGen0377	0	0	0	0	0	0	1000	0	0	0
Enterococcus_faecium_ERR2221216	0	0	0	0	0	0	1000	0	0	0
Enterococcus_faecium_ERR2221309	0	0	0	0	0	0	1000	0	0	0
Enterococcus_faecium_ERR2221310	0	0	0	0	0	0	1000	0	0	0
Enterococcus_faecium_ERR2221317	0	0	0	0	0	0	1000	0	0	0
Enterococcus_faecium_ERR2221332	0	0	0	0	0	0	1000	0	0	0
Enterococcus_faecium_ERV1	0	0	0	0	0	0	1000	0	0	0
Enterococcus_faecium_F9730129_1	0	0	0	0	0	0	1000	0	0	0
Enterococcus_faecium_H17243	0	0	0	0	0	0	1000	0	0	0
Enterococcus_faecium_H17575	0	0	0	0	0	0	1000	0	0	0
Enterococcus_faecium_HF50104	0	0	0	0	0	0	1000	0	0	0
Enterococcus_faecium_HF50105	0	0	0	0	0	0	1000	0	0	0
Enterococcus_faecium_HF50106	0	0	0	0	0	0	1000	0	0	0
Enterococcus_faecium_HF50203	0	0	0	0	0	0	1000	0	0	0
Enterococcus_faecium_HF50204	0	0	0	0	0	0	1000	0	0	0
Enterococcus_faecium_HF50215	0	0	0	0	0	0	1000	0	0	0
Enterococcus_faecium_HM1071	0	0	0	0	0	0	1000	0	0	0
Enterococcus_faecium_HM1072	0	0	0	0	0	0	1000	0	0	0
Enterococcus_faecium_HM1073	0	0	0	0	0	0	1000	0	0	0
Enterococcus_faecium_HM1074	0	0	0	0	0	0	1000	0	0	0
Enterococcus_faecium_LCT_EF128	0	0	0	0	0	0	1000	0	0	0
Enterococcus_faecium_LCT_EF20	0	0	0	0	0	0	1000	0	0	0
Enterococcus_faecium_LCT_EF258	0	0	0	0	0	0	1000	0	0	0
Enterococcus_faecium_LCT_EF90	0	0	0	0	0	0	1000	0	0	0
Enterococcus_faecium_PC4_1	0	0	0	0	0	0	1000	0	0	0
Enterococcus_faecium_S658_3	0	0	0	0	0	0	1000	0	0	0
Enterococcus_faecium_TC_6	0	0	0	0	0	0	1000	0	0	0
Enterococcus_faecium_TX0082	0	0	0	0	0	0	1000	0	0	0
Enterococcus_faecium_TX0133A	0	0	0	0	0	0	1000	0	0	0
Enterococcus_faecium_TX0133a04	0	0	0	0	0	0	1000	0	0	0
Enterococcus_faecium_TX0133B	0	0	0	0	0	0	1000	0	0	0
Enterococcus_faecium_TX0133C	0	0	0	0	0	0	1000	0	0	0
Enterococcus_faecium_TX1330	0	0	0	0	0	0	1000	0	0	0
Enterococcus_faecium_U0317	0	0	0	0	0	0	1000	0	0	0
Enterococcus_faecium_UAA1007	0	0	0	0	0	0	1000	0	0	0
Enterococcus_faecium_UAA1019	0	0	0	0	0	0	1000	0	0	0
Enterococcus_faecium_UAA1022	0	0	0	0	0	0	1000	0	0	0
Enterococcus_faecium_UAA1023	0	0	0	0	0	0	1000	0	0	0
Enterococcus_faecium_UAA1024	0	0	0	0	0	0	1000	0	0	0
Enterococcus_faecium_UAA1025	0	0	0	0	0	0	1000	0	0	0
Enterococcus_faecium_UAA1280	0	0	0	0	0	0	1000	0	0	0
Enterococcus_faecium_UAA1433	0	0	0	0	0	0	1000	0	0	0
Enterococcus_faecium_UAA1484	0	0	0	0	0	0	1000	0	0	0
Enterococcus_faecium_UAA210	0	0	0	0	0	0	1000	0	0	0
Enterococcus_faecium_UAA407	0	0	0	0	0	0	1000	0	0	0
Enterococcus_faecium_UAA430	0	0	0	0	0	0	1000	0	0	0
Enterococcus_faecium_UAA431	0	0	0	0	0	0	1000	0	0	0
Enterococcus_faecium_UAA714	0	0	0	0	0	0	1000	0	0	0
Enterococcus_faecium_UAA715	0	0	0	0	0	0	1000	0	0	0
Enterococcus_faecium_UAA716	0	0	0	0	0	0	1000	0	0	0
Enterococcus_faecium_UAA718	0	0	0	0	0	0	1000	0	0	0
Enterococcus_faecium_UAA719	0	0	0	0	0	0	1000	0	0	0
Enterococcus_faecium_UAA720	0	0	0	0	0	0	1000	0	0	0
Enterococcus_faecium_UAA721	0	0	0	0	0	0	1000	0	0	0
Enterococcus_faecium_UAA722	0	0	0	0	0	0	1000	0	0	0
Enterococcus_faecium_UAA723	0	0	0	0	0	0	1000	0	0	0
Enterococcus_faecium_UAA724	0	0	0	0	0	0	1000	0	0	0
Enterococcus_faecium_UAA725	0	0	0	0	0	0	1000	0	0	0
Enterococcus_faecium_UAA825	0	0	0	0	0	0	1000	0	0	0
Enterococcus_faecium_UAA909	0	0	0	0	0	0	1000	0	0	0
Enterococcus_faecium_UAA910	0	0	0	0	0	0	1000	0	0	0
Enterococcus_faecium_UAA911	0	0	0	0	0	0	1000	0	0	0
Enterococcus_faecium_UAA944	0	0	0	0	0	0	1000	0	0	0
Enterococcus_faecium_UAA945	0	0	0	0	0	0	1000	0	0	0
Enterococcus_faecium_UAA947	0	0	0	0	0	0	1000	0	0	0
Enterococcus_faecium_UAA949	0	0	0	0	0	0	1000	0	0	0
Enterococcus_faecium_UAA950	0	0	0	0	0	0	1000	0	0	0
Enterococcus_faecium_UAA951	0	0	0	0	0	0	1000	0	0	0
Enterococcus_faecium_UAA952	0	0	0	0	0	0	1000	0	0	0
Enterococcus_faecium_UC7251	0	0	0	0	0	0	1000	0	0	0
Enterococcus_faecium_VAN_219	0	0	0	0	0	0	1000	0	0	0
Enterococcus_faecium_VAN_222	0	0	0	0	0	0	1000	0	0	0
Enterococcus_faecium_VAN_327	0	0	0	0	0	0	1000	0	0	0
Enterococcus_faecium_VAN_332	0	0	0	0	0	0	1000	0	0	0
Enterococcus_faecium_VAN_335	0	0	0	0	0	0	1000	0	0	0
Enterococcus_faecium_VAN_342	0	0	0	0	0	0	1000	0	0	0
Enterococcus_faecium_VAN_345	0	0	0	0	0	0	1000	0	0	0
Enterococcus_faecium_VAN_476	0	0	0	0	0	0	1000	0	0	0
Enterococcus_faecium_VRE_108	0	0	0	1000	0	0	1000	0	0	0
Enterococcus_faecium_VRE_110	0	0	0	0	0	0	1000	0	0	0
Enterococcus_faecium_VRE_13	0	0	0	0	0	0	1000	0	0	0
Enterococcus_faecium_VRE_84	0	0	0	1000	0	0	1000	0	0	0
Enterococcus_flavescens_ERR2221217	0	0	0	0	1000	0	1000	0	0	0
Enterococcus_flavescens_ERR2221403	0	0	0	0	1000	0	1000	0	0	0
Enterococcus_gallinarum_EG2	0	0	0	0	1000	0	1000	0	0	0

Enterococcus_gallinarum_ERR2221218	0	0	0	0	1000	0	1000	0	0	0
Enterococcus_gallinarum_ERR2221324	0	0	0	0	1000	0	1000	0	0	0
Enterococcus_gallinarum_ERR2221329	0	0	0	0	1000	0	1000	0	0	0
Enterococcus_gallinarum_ERR2221342	0	0	0	0	1000	0	1000	0	0	0
Enterococcus_gallinarum_ERR2221347	0	0	0	0	1000	0	1000	0	0	0
Enterococcus_gallinarum_ERR2221362	0	0	0	0	1000	0	1000	0	0	0
Enterococcus_gilvus_ATCC_BAA_350	0	0	0	0	1000	0	1000	0	0	0
Enterococcus_gilvus_CR1	0	0	0	0	1000	0	1000	0	0	0
Enterococcus_hirae_ATCC_9790	0	0	0	0	0	0	1000	0	0	0
Enterococcus_mundtii_ATCC_882	0	0	0	0	1000	0	1000	0	0	0
Enterococcus_mundtii_crl1656	0	0	0	0	0	0	1000	0	0	0
Enterococcus_mundtii_CRL35	0	0	0	0	0	0	1000	0	0	0
Enterococcus_mundtii_QU_25	0	0	0	0	1000	0	1000	0	0	0
Enterococcus_pallens_ATCC_BAA_351	0	0	1000	0	0	0	1000	0	0	0
Enterococcus_pallens_DSM_15690	0	0	1000	0	0	0	1000	0	0	0
Enterococcus_phoeniculicola_ATCC_BAA_412	0	0	0	0	1000	0	1000	0	0	0
Enterococcus_raffinosus_ATCC_49464	0	0	0	0	0	0	0	0	0	0
Enterococcus_raffinosus_DSM_5633	0	0	0	0	0	0	0	0	0	0
Enterococcus_raffinosus_NBRC_100492	0	0	0	0	0	0	0	0	0	0
Enterococcus_saccharolyticus_30_1	0	0	0	0	1000	0	1000	0	0	0
Enterococcus_saccharolyticus_ATCC_43076	0	0	1000	0	1000	0	1000	0	0	0
Enterococcus_saccharolyticus_DSM8903	0	0	1000	0	0	0	0	0	0	0
Enterococcus_sp_7L76	0	0	0	0	1000	0	1000	0	0	0
Enterococcus_sp_C1	0	0	0	0	1000	0	1000	0	0	0
Enterococcus_sp_GMD2E	0	0	0	0	0	0	1000	0	0	0
Enterococcus_sp_GMD3E	0	0	0	0	0	0	1000	0	0	0
Enterococcus_sp_GMD4E	0	0	0	0	0	0	1000	0	0	0
Enterococcus_sp_GMD5E	0	0	0	0	0	0	1000	0	0	0
Enterorhabdus_caecimuris_B7	0	0	1000	0	1000	0	1000	0	0	0
Enterorhabdus_mucosicola_DSM_19490	0	0	1000	0	1000	0	1000	0	0	0
Eremococcus_coleocola_ACS_139_V_Col8	0	0	0	0	0	0	1000	0	0	0
Erwinia_psidii_IBSBF_435	0	0	0	0	1000	0	1000	0	0	0
Erwinia_rhapontici_BIGb0435	0	0	0	0	1000	0	1000	0	0	0
Erysipelatoclostridium_nov_ERR2221185	0	0	0	0	1000	0	1000	0	0	0
Erysipelatoclostridium_nov_ERR2221240	0	0	0	0	0	0	500	0	0	0
Erysipelatoclostridium_ramosum_ERR1203924	0	0	0	0	1000	0	1000	0	1000	0
Erysipelatoclostridium_ramosum_ERR171271	0	0	0	0	1000	0	1000	0	1000	0
Erysipelatoclostridium_ramosum_ERR2221150	0	0	0	0	1000	0	1000	0	1000	0
Erysipelatoclostridium_ramosum_ERR2221231	0	0	0	0	1000	0	1000	0	1000	0
Erysipelatoclostridium_ramosum_ERR2221239	0	0	0	0	1000	0	1000	0	1000	0
Erysipelothrix_rhusiopathiae_ATCC_19414	0	0	0	0	0	0	1000	0	0	0
Erysipelothrix_rhusiopathiae_str_Fujisawa	0	0	0	0	0	0	1000	0	0	0
Erysipelothrix_rhusiopathiae_SY1027	0	0	0	0	0	0	1000	0	0	0
Erysipelotrichaceae_bacterium_2_2_44A	0	0	0	0	0	0	1000	0	0	0
Erysipelotrichaceae_bacterium_21_3	0	0	0	0	0	0	1000	0	0	0
Erysipelotrichaceae_bacterium_5_2_54FAA	0	0	0	0	0	0	1000	0	0	0
Erysipelotrichaceae_bacterium_6_1_45	0	0	0	0	0	0	1000	0	0	0
Erysipelotrichaceae_bacterium_I46	0	0	0	0	0	0	1000	0	0	0
Erysipelotrichaceae_bacterium_sp_3_1_53	0	0	0	0	0	0	1000	0	0	0
Erysipelotrichaceae_nov_ERR1022332	0	0	0	0	1000	0	1000	0	0	0
Erysipelotrichaceae_nov_ERR1022333	0	0	0	0	1000	0	1000	0	0	0
Erysipelotrichaceae_nov_ERR1022368	0	0	0	0	1000	0	1000	0	0	0
Erysipelotrichaceae_nov_ERR1022369	0	0	0	0	0	0	1000	0	0	0
Erysipelotrichaceae_nov_ERR1022417	0	0	0	0	0	0	1000	0	0	0
Erysipelotrichaceae_nov_ERR1022464	0	0	0	0	1000	0	1000	0	0	0
Erysipelotrichaceae_nov_ERR1203923	0	0	0	0	0	0	1000	0	0	0
Erysipelotrichaceae_nov_ERR1203963	0	0	0	0	1000	0	1000	0	0	0
Erysipelotrichaceae_nov_ERR1204057	0	0	0	0	1000	0	1000	0	0	0
Erysipelotrichaceae_nov_ERR171269	0	0	0	0	0	0	1000	0	0	0
Erysipelotrichaceae_nov_ERR2221179	0	0	0	0	0	0	1000	0	0	0
Erysipelotrichaceae_nov_ERR2221230	0	0	0	0	0	0	1000	0	0	0
Erysipelotrichaceae_nov_ERR2221343	0	0	0	0	0	0	1000	0	0	0
Erysipelotrichaceae_nov_ERR2230080	0	0	0	0	0	0	1000	0	0	0
Erysipelotrichaceae_nov_ERR2230108	0	0	0	0	1000	0	1000	0	0	0
Escherichia_albertii_EC06_170	0	0	1000	0	1000	0	1000	0	0	0
Escherichia_albertii_KF1	0	0	0	0	1000	0	1000	0	0	1000
Escherichia_albertii_TW07627	0	0	0	0	1000	0	1000	0	0	1000
Escherichia_albertii_TW11588	0	0	1000	1000	1000	0	1000	0	0	0
Escherichia_coli_0_1288	0	0	1000	1000	1000	0	1000	0	0	0
Escherichia_coli_0_1304	0	0	1000	1000	1000	0	1000	0	0	0
Escherichia_coli_042	1000	0	1000	1000	1000	0	1000	0	0	0
Escherichia_coli_07798	0	0	1000	1000	1000	0	1000	0	0	0
Escherichia_coli_09BKT078844	0	0	1000	1000	1000	0	1000	0	0	0
Escherichia_coli_1_2264	0	0	1000	1000	1000	0	1000	0	0	0
Escherichia_coli_1_2741	0	0	1000	1000	1000	0	1000	0	0	0
Escherichia_coli_10_0821	0	0	1000	1000	1000	0	1000	0	0	0
Escherichia_coli_10_0833	0	0	1000	1000	1000	0	1000	0	0	0
Escherichia_coli_10_0869	0	0	1000	1000	1000	0	1000	0	0	0
Escherichia_coli_101_1	0	0	1000	1000	1000	0	1000	0	0	0
Escherichia_coli_1357	0	0	1000	1000	1000	0	1000	0	0	0
Escherichia_coli_174750	0	0	1000	1000	1000	0	1000	0	0	0
Escherichia_coli_174900	0	0	1000	1000	1000	0	1000	0	0	0
Escherichia_coli_178900	0	0	0	1000	1000	0	1000	0	0	0
Escherichia_coli_179100	0	0	1000	1000	1000	0	1000	0	0	0
Escherichia_coli_179550	0	0	1000	1000	1000	0	1000	0	0	0
Escherichia_coli_180050	0	0	1000	1000	1000	0	1000	0	0	0
Escherichia_coli_180200	0	0	1000	1000	1000	0	1000	0	0	0
Escherichia_coli_180600	0	0	1000	1000	1000	0	1000	0	0	0
Escherichia_coli_1827_70	0	0	1000	1000	1000	0	1000	0	0	0
Escherichia_coli_199900_1	0	0	1000	1000	1000	0	1000	0	0	0
Escherichia_coli_2_3916	0	0	1000	1000	1000	0	1000	0	0	0
Escherichia_coli_2_4168	0	0	1000	1000	1000	0	1000	0	0	0
Escherichia_coli_201600_1	0	0	1000	1000	1000	0	1000	0	0	0
Escherichia_coli_2362_75	0	0	1000	1000	1000	0	1000	0	0	0

Escherichia_coli_2534_86	0	0	1000	1000	1000	0	1000	0	0	0
Escherichia_coli_2719100	0	0	1000	1000	1000	0	1000	0	0	0
Escherichia_coli_2720900	0	0	0	1000	1000	0	1000	0	0	0
Escherichia_coli_2722950	0	0	1000	1000	1000	0	1000	0	0	0
Escherichia_coli_2726800	0	0	1000	1000	1000	0	1000	0	0	0
Escherichia_coli_2726950	0	0	1000	1000	1000	0	1000	0	0	0
Escherichia_coli_2729250	0	0	1000	1000	1000	0	1000	0	0	0
Escherichia_coli_2730350	0	0	1000	1000	1000	0	1000	0	0	0
Escherichia_coli_2730450	0	0	1000	1000	1000	0	1000	0	0	0
Escherichia_coli_2731150	0	0	1000	1000	1000	0	1000	0	0	0
Escherichia_coli_2733950	0	0	1000	1000	1000	0	1000	0	0	0
Escherichia_coli_2735000	0	0	1000	1000	1000	0	1000	0	0	0
Escherichia_coli_2741950	0	0	1000	1000	1000	0	1000	0	0	0
Escherichia_coli_2747800	0	0	1000	1000	1000	0	1000	0	0	0
Escherichia_coli_2749250	0	0	1000	1000	1000	0	1000	0	0	0
Escherichia_coli_2756500	0	0	1000	1000	1000	0	1000	0	0	0
Escherichia_coli_2762100	0	0	1000	1000	1000	0	1000	0	0	0
Escherichia_coli_2770900	0	0	1000	1000	1000	0	1000	0	0	0
Escherichia_coli_2780750	0	0	1000	1000	1000	0	1000	0	0	0
Escherichia_coli_2785200	0	0	1000	1000	1000	0	1000	0	0	0
Escherichia_coli_2788150	0	0	1000	1000	1000	0	1000	0	0	0
Escherichia_coli_2845350	0	0	1000	1000	1000	0	1000	0	0	0
Escherichia_coli_2845650	0	0	1000	1000	1000	0	1000	0	0	0
Escherichia_coli_2846750	0	0	1000	1000	1000	0	1000	0	0	0
Escherichia_coli_2848050	0	0	1000	1000	1000	0	1000	0	0	0
Escherichia_coli_2850400	0	0	1000	1000	1000	0	1000	0	0	0
Escherichia_coli_2850750	0	0	1000	1000	1000	0	1000	0	0	0
Escherichia_coli_2851500	0	0	1000	1000	1000	0	1000	0	0	0
Escherichia_coli_2853500	0	0	1000	1000	1000	0	1000	0	0	0
Escherichia_coli_2854350	0	0	1000	1000	1000	0	1000	0	0	0
Escherichia_coli_2860050	0	0	1000	1000	1000	0	1000	0	0	0
Escherichia_coli_2860650	0	0	1000	1000	1000	0	1000	0	0	0
Escherichia_coli_2861200	0	0	1000	1000	1000	0	1000	0	0	0
Escherichia_coli_2862600	0	0	1000	1000	1000	0	1000	0	0	0
Escherichia_coli_2864350	0	0	1000	1000	1000	0	1000	0	0	0
Escherichia_coli_2865200	0	0	1000	1000	1000	0	1000	0	0	0
Escherichia_coli_2866350	0	0	1000	1000	1000	0	1000	0	0	0
Escherichia_coli_2866450	0	0	1000	1000	1000	0	1000	0	0	0
Escherichia_coli_2866550	0	0	1000	1000	1000	0	1000	0	0	0
Escherichia_coli_2866750	0	0	1000	1000	1000	0	1000	0	0	0
Escherichia_coli_2867750	0	0	1000	1000	1000	0	1000	0	0	0
Escherichia_coli_2871950	0	0	1000	1000	1000	0	1000	0	0	0
Escherichia_coli_2872000	0	0	1000	1000	1000	0	1000	0	0	0
Escherichia_coli_2872800	0	0	1000	1000	1000	0	1000	0	0	0
Escherichia_coli_2875000	0	0	1000	1000	1000	0	1000	0	0	0
Escherichia_coli_2875150	0	0	1000	1000	1000	0	1000	0	0	0
Escherichia_coli_3_2303	0	0	1000	1000	1000	0	1000	0	0	0
Escherichia_coli_3_2608	0	0	1000	1000	1000	0	1000	0	0	0
Escherichia_coli_3_3884	0	0	1000	1000	1000	0	1000	0	0	0
Escherichia_coli_3_4870	0	0	1000	1000	1000	0	1000	0	0	0
Escherichia_coli_3003	0	0	1000	1000	1000	0	1000	0	0	0
Escherichia_coli_3006	0	0	1000	1000	1000	0	1000	0	0	0
Escherichia_coli_3431	0	0	1000	1000	1000	0	1000	0	0	0
Escherichia_coli_4_0522	0	0	1000	1000	1000	0	1000	0	0	0
Escherichia_coli_4_0967	0	0	1000	1000	1000	0	1000	0	0	0
Escherichia_coli_4_1_47FAA	0	0	1000	1000	1000	0	1000	0	0	0
Escherichia_coli_5_0588	0	0	1000	1000	1000	0	1000	0	0	0
Escherichia_coli_5_0959	0	0	1000	1000	1000	0	1000	0	0	0
Escherichia_coli_5_2239	0	0	1000	1000	1000	0	1000	0	0	0
Escherichia_coli_536	1000	0	1000	1000	1000	0	1000	0	0	0
Escherichia_coli_53638	0	0	1000	1000	1000	0	1000	0	0	0
Escherichia_coli_536A	0	0	1000	1000	1000	0	1000	0	0	0
Escherichia_coli_541_1	0	0	1000	1000	1000	0	1000	0	0	0
Escherichia_coli_541_15	0	0	1000	1000	1000	0	1000	0	0	0
Escherichia_coli_5412	0	0	1000	1000	1000	0	1000	0	0	0
Escherichia_coli_55989	1000	0	1000	1000	1000	0	1000	0	0	0
Escherichia_coli_5905	0	0	1000	1000	1000	0	1000	0	0	0
Escherichia_coli_6_0172	0	0	1000	1000	1000	0	1000	0	0	0
Escherichia_coli_7_1982	0	0	1000	1000	1000	0	1000	0	0	0
Escherichia_coli_75	0	0	1000	1000	1000	0	1000	0	0	0
Escherichia_coli_8_0416	0	0	1000	1000	1000	0	1000	0	0	0
Escherichia_coli_8_0566	0	0	1000	1000	1000	0	1000	0	0	0
Escherichia_coli_8_0569	0	0	1000	1000	1000	0	1000	0	0	0
Escherichia_coli_8_0586	0	0	1000	1000	1000	0	1000	0	0	0
Escherichia_coli_8_2524	0	0	1000	1000	1000	0	1000	0	0	0
Escherichia_coli_83972	0	0	1000	1000	1000	0	1000	0	0	0
Escherichia_coli_88_0221	0	0	1000	1000	1000	0	1000	0	0	0
Escherichia_coli_88_1042	0	0	1000	1000	1000	0	1000	0	0	0
Escherichia_coli_88_1467	0	0	1000	1000	1000	0	1000	0	0	0
Escherichia_coli_89_0511	0	0	1000	1000	1000	0	1000	0	0	0
Escherichia_coli_9_0111	0	0	1000	1000	1000	0	1000	0	0	0
Escherichia_coli_9_1649	0	0	1000	1000	1000	0	1000	0	0	0
Escherichia_coli_90_0039	0	0	1000	1000	1000	0	1000	0	0	0
Escherichia_coli_90_0091	0	0	1000	1000	1000	0	1000	0	0	0
Escherichia_coli_90_2281	0	0	1000	1000	1000	0	1000	0	0	0
Escherichia_coli_900105_10e	0	0	1000	1000	1000	0	1000	0	0	0
Escherichia_coli_93_001	0	0	1000	1000	1000	0	1000	0	0	0
Escherichia_coli_93_0055	0	0	1000	1000	1000	0	1000	0	0	0
Escherichia_coli_93_0056	0	0	1000	1000	1000	0	1000	0	0	0
Escherichia_coli_93_0624	0	0	1000	1000	1000	0	1000	0	0	0
Escherichia_coli_94_0618	0	0	1000	1000	1000	0	1000	0	0	0
Escherichia_coli_95_0083	0	0	1000	1000	1000	0	1000	0	0	0
Escherichia_coli_95_0183	0	0	1000	1000	1000	0	1000	0	0	0
Escherichia_coli_95_0941	0	0	1000	1000	1000	0	1000	0	0	0

Escherichia_coli_95_0943	0	0	1000	1000	1000	0	1000	0	0	0
Escherichia_coli_95_1288	0	0	1000	1000	1000	0	1000	0	0	0
Escherichia_coli_96_0107	0	0	1000	1000	1000	0	1000	0	0	0
Escherichia_coli_96_0109	0	0	1000	1000	1000	0	1000	0	0	0
Escherichia_coli_96_0427	0	0	1000	1000	1000	0	1000	0	0	0
Escherichia_coli_96_0428	0	0	1000	1000	1000	0	1000	0	0	0
Escherichia_coli_96_0497	0	0	1000	1000	1000	0	1000	0	0	0
Escherichia_coli_96_0932	0	0	1000	1000	1000	0	1000	0	0	0
Escherichia_coli_96_0939	0	0	1000	1000	1000	0	1000	0	0	0
Escherichia_coli_96_154	0	0	1000	1000	1000	0	1000	0	0	0
Escherichia_coli_97_0003	0	0	1000	1000	1000	0	1000	0	0	0
Escherichia_coli_97_0007	0	0	1000	1000	1000	0	1000	0	0	0
Escherichia_coli_97_0010	0	0	1000	1000	1000	0	1000	0	0	0
Escherichia_coli_97_0246	0	0	1000	1000	1000	0	1000	0	0	0
Escherichia_coli_97_0259	0	0	1000	1000	1000	0	1000	0	0	0
Escherichia_coli_97_0264	0	0	1000	1000	1000	0	1000	0	0	0
Escherichia_coli_99_0670	0	0	1000	1000	1000	0	1000	0	0	0
Escherichia_coli_99_0672	0	0	1000	1000	1000	0	1000	0	0	0
Escherichia_coli_99_0678	0	0	1000	1000	1000	0	1000	0	0	0
Escherichia_coli_99_0713	0	0	1000	1000	1000	0	1000	0	0	0
Escherichia_coli_99_0741	0	0	1000	1000	1000	0	1000	0	0	0
Escherichia_coli_99_0814	0	0	1000	1000	1000	0	1000	0	0	0
Escherichia_coli_99_0815	0	0	1000	1000	1000	0	1000	0	0	0
Escherichia_coli_99_0816	0	0	1000	1000	1000	0	1000	0	0	0
Escherichia_coli_99_0839	0	0	1000	1000	1000	0	1000	0	0	0
Escherichia_coli_99_0848	0	0	1000	1000	1000	0	1000	0	0	0
Escherichia_coli_99_1753	0	0	1000	1000	1000	0	1000	0	0	0
Escherichia_coli_99_1762	0	0	1000	1000	1000	0	1000	0	0	0
Escherichia_coli_99_1775	0	0	1000	1000	1000	0	1000	0	0	0
Escherichia_coli_99_1781	0	0	1000	1000	1000	0	1000	0	0	0
Escherichia_coli_99_1805	0	0	1000	1000	1000	0	1000	0	0	0
Escherichia_coli_AA86	0	0	1000	1000	1000	0	1000	0	0	0
Escherichia_coli_ABU_83972	1000	0	1000	1000	1000	0	1000	0	0	0
Escherichia_coli_AD30	0	0	1000	1000	1000	0	1000	0	0	0
Escherichia_coli_AI27	0	0	1000	1000	1000	0	1000	0	0	0
Escherichia_coli_APEC_O1	1000	0	1000	1000	1000	0	1000	0	0	0
Escherichia_coli_APEC_O78	1000	0	1000	1000	1000	0	1000	0	0	0
Escherichia_coli_ARS4_2123	0	0	1000	1000	1000	0	1000	0	0	0
Escherichia_coli_ATCC_25922	0	0	1000	1000	1000	0	1000	0	0	0
Escherichia_coli_ATCC_700728	0	0	1000	1000	1000	0	1000	0	0	0
Escherichia_coli_ATCC_8739	1000	0	1000	1000	1000	0	1000	0	0	0
Escherichia_coli_B_str_REL606	0	0	1000	1000	1000	0	1000	0	0	0
Escherichia_coli_B088	0	0	1000	1000	1000	0	1000	0	0	0
Escherichia_coli_B093	0	0	1000	1000	1000	0	1000	0	0	0
Escherichia_coli_B171	0	0	1000	1000	1000	0	1000	0	0	0
Escherichia_coli_B185	0	0	1000	1000	1000	0	1000	0	0	0
Escherichia_coli_B354	0	0	1000	1000	1000	0	1000	0	0	0
Escherichia_coli_B41	0	0	1000	1000	1000	0	1000	0	0	0
Escherichia_coli_B799	0	0	1000	1000	1000	0	1000	0	0	0
Escherichia_coli_B7A	0	0	1000	1000	1000	0	1000	0	0	0
Escherichia_coli_BCE001_MS16	0	0	1000	1000	1000	0	1000	0	0	0
Escherichia_coli_BCE002_MS12	0	0	1000	1000	1000	0	1000	0	0	0
Escherichia_coli_BCE006_MS_23	0	0	1000	1000	1000	0	1000	0	0	0
Escherichia_coli_BCE007_MS_11	0	0	1000	1000	1000	0	1000	0	0	0
Escherichia_coli_BCE008_MS_13	0	0	1000	1000	1000	0	1000	0	0	0
Escherichia_coli_BCE011_MS_01	0	0	1000	1000	1000	0	1000	0	0	0
Escherichia_coli_BCE019_MS_13	0	0	1000	1000	1000	0	1000	0	0	0
Escherichia_coli_BCE030_MS_09	0	0	1000	1000	1000	0	1000	0	0	0
Escherichia_coli_BCE032_MS_12	0	0	1000	1000	1000	0	1000	0	0	0
Escherichia_coli_BCE034_MS_14	0	0	1000	1000	1000	0	1000	0	0	0
Escherichia_coli_BL21	0	0	1000	1000	1000	0	1000	0	0	0
Escherichia_coli_BL21DE3	0	0	1000	1000	1000	0	1000	0	0	0
Escherichia_coli_BW25113	0	0	1000	1000	1000	0	1000	0	0	0
Escherichia_coli_BW2952	1000	0	1000	1000	1000	0	1000	0	0	0
Escherichia_coli_C_34666	0	0	1000	1000	1000	0	1000	0	0	0
Escherichia_coli_C639_08	0	0	1000	1000	1000	0	1000	0	0	0
Escherichia_coli_C844_97	0	0	1000	1000	1000	0	1000	0	0	0
Escherichia_coli_CB7326	0	0	1000	1000	1000	0	1000	0	0	0
Escherichia_coli_CFT073	1000	0	1000	1000	1000	0	1000	0	0	0
Escherichia_coli_chi7122	0	0	1000	1000	1000	0	1000	0	0	0
Escherichia_coli_cloneA_i1	0	0	1000	1000	1000	0	1000	0	0	0
Escherichia_coli_CUMT8	0	0	1000	1000	1000	0	1000	0	0	0
Escherichia_coli_D9	0	0	1000	1000	1000	0	1000	0	0	0
Escherichia_coli_DEC10A	0	0	1000	1000	1000	0	1000	0	0	0
Escherichia_coli_DEC10B	0	0	1000	1000	1000	0	1000	0	0	0
Escherichia_coli_DEC10C	0	0	1000	1000	1000	0	1000	0	0	0
Escherichia_coli_DEC10D	0	0	1000	1000	1000	0	1000	0	0	0
Escherichia_coli_DEC10E	0	0	1000	1000	1000	0	1000	0	0	0
Escherichia_coli_DEC10F	0	0	1000	1000	1000	0	1000	0	0	0
Escherichia_coli_DEC11A	0	0	1000	1000	1000	0	1000	0	0	0
Escherichia_coli_DEC11B	0	0	1000	1000	1000	0	1000	0	0	0
Escherichia_coli_DEC11C	0	0	1000	1000	1000	0	1000	0	0	0
Escherichia_coli_DEC11D	0	0	1000	1000	1000	0	1000	0	0	0
Escherichia_coli_DEC11E	0	0	1000	1000	1000	0	1000	0	0	0
Escherichia_coli_DEC12A	0	0	1000	1000	1000	0	1000	0	0	0
Escherichia_coli_DEC12B	0	0	1000	1000	1000	0	1000	0	0	0
Escherichia_coli_DEC12C	0	0	1000	1000	1000	0	1000	0	0	0
Escherichia_coli_DEC12D	0	0	1000	1000	1000	0	1000	0	0	0
Escherichia_coli_DEC12E	0	0	1000	1000	1000	0	1000	0	0	0
Escherichia_coli_DEC13A	0	0	1000	1000	1000	0	1000	0	0	0
Escherichia_coli_DEC13B	0	0	1000	1000	1000	0	1000	0	0	0
Escherichia_coli_DEC13C	0	0	1000	1000	1000	0	1000	0	0	0
Escherichia_coli_DEC13D	0	0	0	1000	1000	0	1000	0	0	0
Escherichia_coli_DEC13E	0	0	0	1000	1000	0	1000	0	0	0

Escherichia_coli_DEC14A	0	0	1000	1000	1000	0	1000	0	0	0
Escherichia_coli_DEC14B	0	0	1000	1000	1000	0	1000	0	0	0
Escherichia_coli_DEC14C	0	0	1000	1000	1000	0	1000	0	0	0
Escherichia_coli_DEC14D	0	0	1000	1000	1000	0	1000	0	0	0
Escherichia_coli_DEC15A	0	0	1000	1000	1000	0	1000	0	0	0
Escherichia_coli_DEC15B	0	0	1000	1000	1000	0	1000	0	0	0
Escherichia_coli_DEC15C	0	0	1000	1000	1000	0	1000	0	0	0
Escherichia_coli_DEC15D	0	0	1000	1000	1000	0	1000	0	0	0
Escherichia_coli_DEC15E	0	0	1000	1000	1000	0	1000	0	0	0
Escherichia_coli_DEC1A	0	0	1000	1000	1000	0	1000	0	0	0
Escherichia_coli_DEC1B	0	0	1000	1000	1000	0	1000	0	0	0
Escherichia_coli_DEC1C	0	0	1000	1000	1000	0	1000	0	0	0
Escherichia_coli_DEC1D	0	0	1000	1000	1000	0	1000	0	0	0
Escherichia_coli_DEC1E	0	0	1000	1000	1000	0	1000	0	0	0
Escherichia_coli_DEC2A	0	0	1000	1000	1000	0	1000	0	0	0
Escherichia_coli_DEC2B	0	0	1000	1000	1000	0	1000	0	0	0
Escherichia_coli_DEC2C	0	0	1000	1000	1000	0	1000	0	0	0
Escherichia_coli_DEC2D	0	0	1000	1000	1000	0	1000	0	0	0
Escherichia_coli_DEC2E	0	0	1000	1000	1000	0	1000	0	0	0
Escherichia_coli_DEC3A	0	0	1000	1000	1000	0	1000	0	0	0
Escherichia_coli_DEC3B	0	0	1000	1000	1000	0	1000	0	0	0
Escherichia_coli_DEC3C	0	0	1000	1000	1000	0	1000	0	0	0
Escherichia_coli_DEC3D	0	0	1000	1000	1000	0	1000	0	0	0
Escherichia_coli_DEC3E	0	0	1000	1000	1000	0	1000	0	0	0
Escherichia_coli_DEC3F	0	0	1000	1000	1000	0	1000	0	0	0
Escherichia_coli_DEC4A	0	0	1000	1000	1000	0	1000	0	0	0
Escherichia_coli_DEC4B	0	0	1000	1000	1000	0	1000	0	0	0
Escherichia_coli_DEC4C	0	0	1000	1000	1000	0	1000	0	0	0
Escherichia_coli_DEC4D	0	0	1000	1000	1000	0	1000	0	0	0
Escherichia_coli_DEC4E	0	0	1000	1000	1000	0	1000	0	0	0
Escherichia_coli_DEC4F	0	0	1000	1000	1000	0	1000	0	0	0
Escherichia_coli_DEC5A	0	0	1000	1000	1000	0	1000	0	0	0
Escherichia_coli_DEC5B	0	0	1000	1000	1000	0	1000	0	0	0
Escherichia_coli_DEC5C	0	0	1000	1000	1000	0	1000	0	0	0
Escherichia_coli_DEC5D	0	0	1000	1000	1000	0	1000	0	0	0
Escherichia_coli_DEC5E	0	0	1000	1000	1000	0	1000	0	0	0
Escherichia_coli_DEC6A	0	0	1000	1000	1000	0	1000	0	0	0
Escherichia_coli_DEC6B	0	0	1000	1000	1000	0	1000	0	0	0
Escherichia_coli_DEC6C	0	0	1000	1000	1000	0	1000	0	0	0
Escherichia_coli_DEC6D	0	0	1000	1000	1000	0	1000	0	0	0
Escherichia_coli_DEC6E	0	0	1000	1000	1000	0	1000	0	0	0
Escherichia_coli_DEC7A	0	0	1000	1000	1000	0	1000	0	0	0
Escherichia_coli_DEC7B	0	0	1000	1000	1000	0	1000	0	0	0
Escherichia_coli_DEC7C	0	0	1000	1000	1000	0	1000	0	0	0
Escherichia_coli_DEC7D	0	0	1000	1000	1000	0	1000	0	0	0
Escherichia_coli_DEC7E	0	0	1000	1000	1000	0	1000	0	0	0
Escherichia_coli_DEC8A	0	0	1000	1000	1000	0	1000	0	0	0
Escherichia_coli_DEC8B	0	0	1000	1000	1000	0	1000	0	0	0
Escherichia_coli_DEC8C	0	0	1000	1000	1000	0	1000	0	0	0
Escherichia_coli_DEC8D	0	0	1000	1000	1000	0	1000	0	0	0
Escherichia_coli_DEC8E	0	0	1000	1000	1000	0	1000	0	0	0
Escherichia_coli_DEC9A	0	0	1000	1000	1000	0	1000	0	0	0
Escherichia_coli_DEC9B	0	0	1000	1000	1000	0	1000	0	0	0
Escherichia_coli_DEC9C	0	0	1000	1000	1000	0	1000	0	0	0
Escherichia_coli_DEC9D	0	0	1000	1000	1000	0	1000	0	0	0
Escherichia_coli_DEC9E	0	0	1000	1000	1000	0	1000	0	0	0
Escherichia_coli_DH1	1000	0	1000	1000	1000	0	1000	0	0	0
Escherichia_coli_E101	0	0	1000	1000	1000	0	1000	0	0	0
Escherichia_coli_E110019	0	0	1000	1000	1000	0	1000	0	0	0
Escherichia_coli_E1167	0	0	1000	1000	1000	0	1000	0	0	0
Escherichia_coli_E128010	0	0	1000	1000	1000	0	1000	0	0	0
Escherichia_coli_E1520	0	0	1000	1000	1000	0	1000	0	0	0
Escherichia_coli_E22	0	0	1000	1000	1000	0	1000	0	0	0
Escherichia_coli_E2348_69	0	0	1000	1000	1000	0	1000	0	0	0
Escherichia_coli_E24377A	1000	0	1000	1000	1000	0	1000	0	0	0
Escherichia_coli_E482	0	0	1000	1000	1000	0	1000	0	0	0
Escherichia_coli_EC1734	0	0	1000	1000	1000	0	1000	0	0	0
Escherichia_coli_EC1735	0	0	1000	1000	1000	0	1000	0	0	0
Escherichia_coli_EC1736	0	0	1000	1000	1000	0	1000	0	0	0
Escherichia_coli_EC1737	0	0	1000	1000	1000	0	1000	0	0	0
Escherichia_coli_EC1738	0	0	1000	1000	1000	0	1000	0	0	0
Escherichia_coli_EC1845	0	0	1000	1000	1000	0	1000	0	0	0
Escherichia_coli_EC1846	0	0	1000	1000	1000	0	1000	0	0	0
Escherichia_coli_EC1847	0	0	1000	1000	1000	0	1000	0	0	0
Escherichia_coli_EC1848	0	0	1000	1000	1000	0	1000	0	0	0
Escherichia_coli_EC1849	0	0	1000	1000	1000	0	1000	0	0	0
Escherichia_coli_EC1850	0	0	1000	1000	1000	0	1000	0	0	0
Escherichia_coli_EC1856	0	0	1000	1000	1000	0	1000	0	0	0
Escherichia_coli_EC1862	0	0	1000	1000	1000	0	1000	0	0	0
Escherichia_coli_EC1863	0	0	1000	1000	1000	0	1000	0	0	0
Escherichia_coli_EC1864	0	0	1000	1000	1000	0	1000	0	0	0
Escherichia_coli_EC1865	0	0	1000	1000	1000	0	1000	0	0	0
Escherichia_coli_EC1866	0	0	1000	1000	1000	0	1000	0	0	0
Escherichia_coli_EC1869	0	0	1000	1000	1000	0	1000	0	0	0
Escherichia_coli_EC1870	0	0	1000	1000	1000	0	1000	0	0	0
Escherichia_coli_EC302_04	0	0	1000	1000	1000	0	1000	0	0	0
Escherichia_coli_EC4013	0	0	1000	1000	1000	0	1000	0	0	0
Escherichia_coli_EC4100B	0	0	1000	1000	1000	0	1000	0	0	0
Escherichia_coli_EC4196	0	0	1000	1000	1000	0	1000	0	0	0
Escherichia_coli_EC4203	0	0	1000	1000	1000	0	1000	0	0	0
Escherichia_coli_EC4402	0	0	1000	1000	1000	0	1000	0	0	0
Escherichia_coli_EC4421	0	0	1000	1000	1000	0	1000	0	0	0
Escherichia_coli_EC4422	0	0	1000	1000	1000	0	1000	0	0	0
Escherichia_coli_EC4436	0	0	1000	1000	1000	0	1000	0	0	0

Escherichia_coli_EC4437	0	0	1000	1000	1000	0	1000	0	0	0
Escherichia_coli_EC4439	0	0	1000	1000	1000	0	1000	0	0	0
Escherichia_coli_EC4448	0	0	1000	1000	1000	0	1000	0	0	0
Escherichia_coli_ED1a	1000	0	1000	1000	1000	0	1000	0	0	0
Escherichia_coli_Envira_10_1	0	0	1000	1000	1000	0	1000	0	0	0
Escherichia_coli_Envira_8_11	0	0	1000	1000	1000	0	1000	0	0	0
Escherichia_coli_EPEC_C342_62	0	0	1000	1000	1000	0	1000	0	0	0
Escherichia_coli_EPECa12	0	0	1000	1000	1000	0	1000	0	0	0
Escherichia_coli_EPECa14	0	0	1000	1000	1000	0	1000	0	0	0
Escherichia_coli_ERR1203920	0	0	1000	1000	1000	0	1000	0	0	0
Escherichia_coli_ERR2221112	0	0	1000	1000	1000	0	1000	0	0	0
Escherichia_coli_ERR2221113	0	0	1000	1000	1000	0	1000	0	0	0
Escherichia_coli_ERR2221128	0	0	1000	1000	1000	0	1000	0	0	0
Escherichia_coli_ERR2221214	0	0	1000	1000	1000	0	1000	0	0	0
Escherichia_coli_ERR2221221	0	0	1000	1000	1000	0	1000	0	0	0
Escherichia_coli_ERR2221222	0	0	1000	1000	1000	0	1000	0	0	0
Escherichia_coli_ERR2221227	0	0	1000	1000	1000	0	1000	0	0	0
Escherichia_coli_ERR2221250	0	0	1000	1000	1000	0	1000	0	0	0
Escherichia_coli_ERR2221318	0	0	1000	1000	1000	0	1000	0	0	0
Escherichia_coli_ERR2221398	0	0	1000	1000	1000	0	1000	0	0	0
Escherichia_coli_ERR2221401	0	0	1000	1000	1000	0	1000	0	0	0
Escherichia_coli_ETEC_H10407	1000	0	1000	1000	1000	0	1000	0	0	0
Escherichia_coli_F11	0	0	1000	1000	1000	0	1000	0	0	0
Escherichia_coli_FDA504	0	0	1000	1000	1000	0	1000	0	0	0
Escherichia_coli_FDA505	0	0	1000	1000	1000	0	1000	0	0	0
Escherichia_coli_FDA506	0	0	1000	1000	1000	0	1000	0	0	0
Escherichia_coli_FDA507	0	0	1000	1000	1000	0	1000	0	0	0
Escherichia_coli_FDA517	0	0	1000	1000	1000	0	1000	0	0	0
Escherichia_coli_FDAARGOS_170	0	0	1000	1000	1000	0	1000	0	0	0
Escherichia_coli_FRIK1985	0	0	1000	1000	1000	0	1000	0	0	0
Escherichia_coli_FRIK1990	0	0	1000	1000	1000	0	1000	0	0	0
Escherichia_coli_FRIK1996	0	0	1000	1000	1000	0	1000	0	0	0
Escherichia_coli_FRIK1997	0	0	1000	1000	1000	0	1000	0	0	0
Escherichia_coli_FRIK1999	0	0	1000	1000	1000	0	1000	0	0	0
Escherichia_coli_FRIK2001	0	0	1000	1000	1000	0	1000	0	0	0
Escherichia_coli_FRIK523	0	0	1000	1000	1000	0	1000	0	0	0
Escherichia_coli_FRIK920	0	0	1000	1000	1000	0	1000	0	0	0
Escherichia_coli_FVEC1302	0	0	1000	1000	1000	0	1000	0	0	0
Escherichia_coli_FVEC1412	0	0	1000	1000	1000	0	1000	0	0	0
Escherichia_coli_G58_1	0	0	1000	1000	1000	0	1000	0	0	0
Escherichia_coli_H120	0	0	1000	1000	1000	0	1000	0	0	0
Escherichia_coli_H252	0	0	1000	1000	1000	0	1000	0	0	0
Escherichia_coli_H263	0	0	1000	1000	1000	0	1000	0	0	0
Escherichia_coli_H299	0	0	1000	1000	1000	0	1000	0	0	0
Escherichia_coli_H30	0	0	1000	1000	1000	0	1000	0	0	0
Escherichia_coli_H397	0	0	1000	1000	1000	0	1000	0	0	0
Escherichia_coli_H489	0	0	1000	1000	1000	0	1000	0	0	0
Escherichia_coli_H494	0	0	1000	1000	1000	0	1000	0	0	0
Escherichia_coli_H591	0	0	1000	1000	1000	0	1000	0	0	0
Escherichia_coli_H730	0	0	1000	1000	1000	0	1000	0	0	0
Escherichia_coli_H736	0	0	0	1000	1000	0	1000	0	0	0
Escherichia_coli_HM26	0	0	1000	1000	1000	0	1000	0	0	0
Escherichia_coli_HM27	0	0	1000	1000	1000	0	1000	0	0	0
Escherichia_coli_HM46	0	0	1000	1000	1000	0	1000	0	0	0
Escherichia_coli_HM605	0	0	1000	1000	1000	0	1000	0	0	0
Escherichia_coli_HM65	0	0	1000	1000	1000	0	1000	0	0	0
Escherichia_coli_HS	1000	0	1000	1000	1000	0	1000	0	0	0
Escherichia_coli_IAI1	1000	0	1000	1000	1000	0	1000	0	0	0
Escherichia_coli_IAI39	1000	0	1000	1000	1000	0	1000	0	0	0
Escherichia_coli_IHE3034	1000	0	1000	1000	1000	0	1000	0	0	0
Escherichia_coli_IMT2125	0	0	1000	1000	1000	0	1000	0	0	0
Escherichia_coli_J53	0	0	1000	1000	1000	0	1000	0	0	0
Escherichia_coli_JB1_95	0	0	1000	1000	1000	0	1000	0	0	0
Escherichia_coli_Jurua_18_11	0	0	1000	1000	1000	0	1000	0	0	0
Escherichia_coli_Jurua_20_10	0	0	1000	1000	1000	0	1000	0	0	0
Escherichia_coli_KD2	0	0	1000	1000	1000	0	1000	0	0	0
Escherichia_coli_KO11	1000	0	1000	1000	1000	0	1000	0	0	0
Escherichia_coli_KTE1	0	0	1000	1000	1000	0	1000	0	0	0
Escherichia_coli_KTE10	0	0	1000	1000	1000	0	1000	0	0	0
Escherichia_coli_KTE100	0	0	1000	1000	1000	0	1000	0	0	0
Escherichia_coli_KTE101	0	0	1000	1000	1000	0	1000	0	0	0
Escherichia_coli_KTE102	0	0	0	1000	1000	0	1000	0	0	0
Escherichia_coli_KTE103	0	0	1000	1000	1000	0	1000	0	0	0
Escherichia_coli_KTE104	0	0	1000	1000	1000	0	1000	0	0	0
Escherichia_coli_KTE105	0	0	1000	1000	1000	0	1000	0	0	0
Escherichia_coli_KTE106	0	0	1000	1000	1000	0	1000	0	0	0
Escherichia_coli_KTE107	0	0	1000	1000	1000	0	1000	0	0	0
Escherichia_coli_KTE108	0	0	1000	1000	1000	0	1000	0	0	0
Escherichia_coli_KTE109	0	0	1000	1000	1000	0	1000	0	0	0
Escherichia_coli_KTE11	0	0	1000	1000	1000	0	1000	0	0	0
Escherichia_coli_KTE111	0	0	1000	1000	1000	0	1000	0	0	0
Escherichia_coli_KTE112	0	0	1000	1000	1000	0	1000	0	0	0
Escherichia_coli_KTE113	0	0	1000	1000	1000	0	1000	0	0	0
Escherichia_coli_KTE114	0	0	1000	1000	1000	0	1000	0	0	0
Escherichia_coli_KTE115	0	0	1000	1000	1000	0	1000	0	0	0
Escherichia_coli_KTE116	0	0	1000	1000	1000	0	1000	0	0	0
Escherichia_coli_KTE117	0	0	1000	1000	1000	0	1000	0	0	0
Escherichia_coli_KTE118	0	0	1000	1000	1000	0	1000	0	0	0
Escherichia_coli_KTE119	0	0	1000	1000	1000	0	1000	0	0	0
Escherichia_coli_KTE12	0	0	1000	1000	1000	0	1000	0	0	0
Escherichia_coli_KTE120	0	0	1000	1000	1000	0	1000	0	0	0
Escherichia_coli_KTE121	0	0	1000	1000	1000	0	1000	0	0	0
Escherichia_coli_KTE122	0	0	1000	1000	1000	0	1000	0	0	0
Escherichia_coli_KTE123	0	0	1000	1000	1000	0	1000	0	0	0

Escherichia_coli_KTE124	0	0	1000	1000	1000	0	1000	0	0	0
Escherichia_coli_KTE125	0	0	1000	1000	1000	0	1000	0	0	0
Escherichia_coli_KTE126	0	0	1000	1000	1000	0	1000	0	0	0
Escherichia_coli_KTE127	0	0	1000	1000	1000	0	1000	0	0	0
Escherichia_coli_KTE128	0	0	1000	1000	1000	0	1000	0	0	0
Escherichia_coli_KTE129	0	0	1000	1000	1000	0	1000	0	0	0
Escherichia_coli_KTE13	0	0	1000	1000	1000	0	1000	0	0	0
Escherichia_coli_KTE130	0	0	1000	1000	1000	0	1000	0	0	0
Escherichia_coli_KTE131	0	0	1000	1000	1000	0	1000	0	0	0
Escherichia_coli_KTE132	0	0	1000	1000	1000	0	1000	0	0	0
Escherichia_coli_KTE133	0	0	1000	1000	1000	0	1000	0	0	0
Escherichia_coli_KTE134	0	0	1000	1000	1000	0	1000	0	0	0
Escherichia_coli_KTE135	0	0	1000	1000	1000	0	1000	0	0	0
Escherichia_coli_KTE136	0	0	1000	1000	1000	0	1000	0	0	0
Escherichia_coli_KTE137	0	0	1000	1000	1000	0	1000	0	0	0
Escherichia_coli_KTE138	0	0	1000	1000	1000	0	1000	0	0	0
Escherichia_coli_KTE139	0	0	1000	1000	1000	0	1000	0	0	0
Escherichia_coli_KTE14	0	0	1000	1000	1000	0	1000	0	0	0
Escherichia_coli_KTE140	0	0	1000	1000	1000	0	1000	0	0	0
Escherichia_coli_KTE141	0	0	1000	1000	1000	0	1000	0	0	0
Escherichia_coli_KTE142	0	0	1000	1000	1000	0	1000	0	0	0
Escherichia_coli_KTE143	0	0	1000	1000	1000	0	1000	0	0	0
Escherichia_coli_KTE144	0	0	1000	1000	1000	0	1000	0	0	0
Escherichia_coli_KTE145	0	0	1000	1000	1000	0	1000	0	0	0
Escherichia_coli_KTE146	0	0	1000	1000	1000	0	1000	0	0	0
Escherichia_coli_KTE147	0	0	1000	1000	1000	0	1000	0	0	0
Escherichia_coli_KTE148	0	0	1000	1000	1000	0	1000	0	0	0
Escherichia_coli_KTE15	0	0	1000	1000	1000	0	1000	0	0	0
Escherichia_coli_KTE150	0	0	1000	1000	1000	0	1000	0	0	0
Escherichia_coli_KTE153	0	0	1000	1000	1000	0	1000	0	0	0
Escherichia_coli_KTE154	0	0	1000	1000	1000	0	1000	0	0	0
Escherichia_coli_KTE155	0	0	1000	1000	1000	0	1000	0	0	0
Escherichia_coli_KTE156	0	0	1000	1000	1000	0	1000	0	0	0
Escherichia_coli_KTE157	0	0	1000	1000	1000	0	1000	0	0	0
Escherichia_coli_KTE158	0	0	1000	1000	1000	0	1000	0	0	0
Escherichia_coli_KTE159	0	0	1000	1000	1000	0	1000	0	0	0
Escherichia_coli_KTE16	0	0	1000	1000	1000	0	1000	0	0	0
Escherichia_coli_KTE160	0	0	1000	1000	1000	0	1000	0	0	0
Escherichia_coli_KTE161	0	0	1000	1000	1000	0	1000	0	0	0
Escherichia_coli_KTE162	0	0	1000	1000	1000	0	1000	0	0	0
Escherichia_coli_KTE163	0	0	1000	1000	1000	0	1000	0	0	0
Escherichia_coli_KTE165	0	0	1000	1000	1000	0	1000	0	0	0
Escherichia_coli_KTE166	0	0	1000	1000	1000	0	1000	0	0	0
Escherichia_coli_KTE167	0	0	1000	1000	1000	0	1000	0	0	0
Escherichia_coli_KTE168	0	0	1000	1000	1000	0	1000	0	0	0
Escherichia_coli_KTE169	0	0	1000	1000	1000	0	1000	0	0	0
Escherichia_coli_KTE17	0	0	1000	1000	1000	0	1000	0	0	0
Escherichia_coli_KTE170	0	0	1000	1000	1000	0	1000	0	0	0
Escherichia_coli_KTE171	0	0	1000	1000	1000	0	1000	0	0	0
Escherichia_coli_KTE172	0	0	1000	1000	1000	0	1000	0	0	0
Escherichia_coli_KTE173	0	0	1000	1000	1000	0	1000	0	0	0
Escherichia_coli_KTE174	0	0	1000	1000	1000	0	1000	0	0	0
Escherichia_coli_KTE175	0	0	1000	1000	1000	0	1000	0	0	0
Escherichia_coli_KTE176	0	0	1000	1000	1000	0	1000	0	0	0
Escherichia_coli_KTE177	0	0	1000	1000	1000	0	1000	0	0	0
Escherichia_coli_KTE178	0	0	1000	1000	1000	0	1000	0	0	0
Escherichia_coli_KTE179	0	0	1000	1000	1000	0	1000	0	0	0
Escherichia_coli_KTE18	0	0	1000	1000	1000	0	1000	0	0	0
Escherichia_coli_KTE180	0	0	1000	1000	1000	0	1000	0	0	0
Escherichia_coli_KTE181	0	0	1000	1000	1000	0	1000	0	0	0
Escherichia_coli_KTE182	0	0	1000	1000	1000	0	1000	0	0	0
Escherichia_coli_KTE183	0	0	1000	1000	1000	0	1000	0	0	0
Escherichia_coli_KTE184	0	0	1000	1000	1000	0	1000	0	0	0
Escherichia_coli_KTE185	0	0	1000	1000	1000	0	1000	0	0	0
Escherichia_coli_KTE186	0	0	1000	1000	1000	0	1000	0	0	0
Escherichia_coli_KTE187	0	0	1000	1000	1000	0	1000	0	0	0
Escherichia_coli_KTE188	0	0	1000	1000	1000	0	1000	0	0	0
Escherichia_coli_KTE189	0	0	1000	1000	1000	0	1000	0	0	0
Escherichia_coli_KTE19	0	0	1000	1000	1000	0	1000	0	0	0
Escherichia_coli_KTE190	0	0	1000	1000	1000	0	1000	0	0	0
Escherichia_coli_KTE191	0	0	1000	1000	1000	0	1000	0	0	0
Escherichia_coli_KTE192	0	0	1000	1000	1000	0	1000	0	0	0
Escherichia_coli_KTE193	0	0	1000	1000	1000	0	1000	0	0	0
Escherichia_coli_KTE194	0	0	1000	1000	1000	0	1000	0	0	0
Escherichia_coli_KTE195	0	0	1000	1000	1000	0	1000	0	0	0
Escherichia_coli_KTE196	0	0	1000	1000	1000	0	1000	0	0	0
Escherichia_coli_KTE197	0	0	1000	1000	1000	0	1000	0	0	0
Escherichia_coli_KTE198	0	0	1000	1000	1000	0	1000	0	0	0
Escherichia_coli_KTE199	0	0	1000	1000	1000	0	1000	0	0	0
Escherichia_coli_KTE2	0	0	1000	1000	1000	0	1000	0	0	0
Escherichia_coli_KTE20	0	0	1000	1000	1000	0	1000	0	0	0
Escherichia_coli_KTE200	0	0	1000	1000	1000	0	1000	0	0	0
Escherichia_coli_KTE201	0	0	1000	1000	1000	0	1000	0	0	0
Escherichia_coli_KTE202	0	0	1000	1000	1000	0	1000	0	0	0
Escherichia_coli_KTE203	0	0	1000	1000	1000	0	1000	0	0	0
Escherichia_coli_KTE204	0	0	1000	1000	1000	0	1000	0	0	0
Escherichia_coli_KTE205	0	0	1000	1000	1000	0	1000	0	0	0
Escherichia_coli_KTE206	0	0	1000	1000	1000	0	1000	0	0	0
Escherichia_coli_KTE207	0	0	1000	1000	1000	0	1000	0	0	0
Escherichia_coli_KTE208	0	0	1000	1000	1000	0	1000	0	0	0
Escherichia_coli_KTE209	0	0	1000	1000	1000	0	1000	0	0	0
Escherichia_coli_KTE21	0	0	1000	1000	1000	0	1000	0	0	0
Escherichia_coli_KTE210	0	0	1000	1000	1000	0	1000	0	0	0
Escherichia_coli_KTE211	0	0	1000	1000	1000	0	1000	0	0	0

Escherichia_coli_KTE212	0	0	1000	1000	1000	0	1000	0	0	0
Escherichia_coli_KTE213	0	0	1000	1000	1000	0	1000	0	0	0
Escherichia_coli_KTE214	0	0	1000	1000	1000	0	1000	0	0	0
Escherichia_coli_KTE215	0	0	1000	1000	1000	0	1000	0	0	0
Escherichia_coli_KTE216	0	0	1000	1000	1000	0	1000	0	0	0
Escherichia_coli_KTE217	0	0	1000	1000	1000	0	1000	0	0	0
Escherichia_coli_KTE218	0	0	1000	1000	1000	0	1000	0	0	0
Escherichia_coli_KTE219	0	0	1000	1000	1000	0	1000	0	0	0
Escherichia_coli_KTE22	0	0	1000	1000	1000	0	1000	0	0	0
Escherichia_coli_KTE220	0	0	1000	1000	1000	0	1000	0	0	0
Escherichia_coli_KTE221	0	0	1000	1000	1000	0	1000	0	0	0
Escherichia_coli_KTE222	0	0	1000	1000	1000	0	1000	0	0	0
Escherichia_coli_KTE223	0	0	1000	1000	1000	0	1000	0	0	0
Escherichia_coli_KTE224	0	0	1000	1000	1000	0	1000	0	0	0
Escherichia_coli_KTE225	0	0	1000	1000	1000	0	1000	0	0	0
Escherichia_coli_KTE226	0	0	1000	1000	1000	0	1000	0	0	0
Escherichia_coli_KTE227	0	0	1000	1000	1000	0	1000	0	0	0
Escherichia_coli_KTE228	0	0	1000	1000	1000	0	1000	0	0	0
Escherichia_coli_KTE229	0	0	1000	1000	1000	0	1000	0	0	0
Escherichia_coli_KTE23	0	0	1000	1000	1000	0	1000	0	0	0
Escherichia_coli_KTE230	0	0	1000	1000	1000	0	1000	0	0	0
Escherichia_coli_KTE231	0	0	1000	1000	1000	0	1000	0	0	0
Escherichia_coli_KTE232	0	0	1000	1000	1000	0	1000	0	0	0
Escherichia_coli_KTE233	0	0	1000	1000	1000	0	1000	0	0	0
Escherichia_coli_KTE234	0	0	1000	1000	1000	0	1000	0	0	0
Escherichia_coli_KTE235	0	0	1000	1000	1000	0	1000	0	0	0
Escherichia_coli_KTE236	0	0	1000	1000	1000	0	1000	0	0	0
Escherichia_coli_KTE237	0	0	1000	1000	1000	0	1000	0	0	0
Escherichia_coli_KTE24	0	0	1000	1000	1000	0	1000	0	0	0
Escherichia_coli_KTE240	0	0	1000	1000	1000	0	1000	0	0	0
Escherichia_coli_KTE25	0	0	1000	1000	1000	0	1000	0	0	0
Escherichia_coli_KTE26	0	0	1000	1000	1000	0	1000	0	0	0
Escherichia_coli_KTE27	0	0	1000	1000	1000	0	1000	0	0	0
Escherichia_coli_KTE28	0	0	1000	1000	1000	0	1000	0	0	0
Escherichia_coli_KTE29	0	0	1000	1000	1000	0	1000	0	0	0
Escherichia_coli_KTE3	0	0	1000	1000	1000	0	1000	0	0	0
Escherichia_coli_KTE31	0	0	1000	1000	1000	0	1000	0	0	0
Escherichia_coli_KTE33	0	0	1000	1000	1000	0	1000	0	0	0
Escherichia_coli_KTE34	0	0	1000	1000	1000	0	1000	0	0	0
Escherichia_coli_KTE35	0	0	1000	1000	1000	0	1000	0	0	0
Escherichia_coli_KTE36	0	0	1000	1000	1000	0	1000	0	0	0
Escherichia_coli_KTE37	0	0	1000	1000	1000	0	1000	0	0	0
Escherichia_coli_KTE38	0	0	1000	1000	1000	0	1000	0	0	0
Escherichia_coli_KTE39	0	0	1000	1000	1000	0	1000	0	0	0
Escherichia_coli_KTE4	0	0	1000	1000	1000	0	1000	0	0	0
Escherichia_coli_KTE40	0	0	1000	1000	1000	0	1000	0	0	0
Escherichia_coli_KTE41	0	0	1000	1000	1000	0	1000	0	0	0
Escherichia_coli_KTE42	0	0	1000	1000	1000	0	1000	0	0	0
Escherichia_coli_KTE43	0	0	1000	1000	1000	0	1000	0	0	0
Escherichia_coli_KTE44	0	0	1000	1000	1000	0	1000	0	0	0
Escherichia_coli_KTE45	0	0	1000	1000	1000	0	1000	0	0	0
Escherichia_coli_KTE46	0	0	1000	1000	1000	0	1000	0	0	0
Escherichia_coli_KTE47	0	0	1000	1000	1000	0	1000	0	0	0
Escherichia_coli_KTE48	0	0	1000	1000	1000	0	1000	0	0	0
Escherichia_coli_KTE49	0	0	1000	1000	1000	0	1000	0	0	0
Escherichia_coli_KTE5	0	0	1000	1000	1000	0	1000	0	0	0
Escherichia_coli_KTE50	0	0	1000	1000	1000	0	1000	0	0	0
Escherichia_coli_KTE51	0	0	1000	1000	1000	0	1000	0	0	0
Escherichia_coli_KTE53	0	0	1000	1000	1000	0	1000	0	0	0
Escherichia_coli_KTE54	0	0	1000	1000	1000	0	1000	0	0	0
Escherichia_coli_KTE55	0	0	1000	1000	1000	0	1000	0	0	0
Escherichia_coli_KTE56	0	0	1000	1000	1000	0	1000	0	0	0
Escherichia_coli_KTE57	0	0	1000	1000	1000	0	1000	0	0	0
Escherichia_coli_KTE58	0	0	1000	1000	1000	0	1000	0	0	0
Escherichia_coli_KTE59	0	0	1000	1000	1000	0	1000	0	0	0
Escherichia_coli_KTE6	0	0	1000	1000	1000	0	1000	0	0	0
Escherichia_coli_KTE60	0	0	1000	1000	1000	0	1000	0	0	0
Escherichia_coli_KTE61	0	0	1000	1000	1000	0	1000	0	0	0
Escherichia_coli_KTE62	0	0	1000	1000	1000	0	1000	0	0	0
Escherichia_coli_KTE63	0	0	1000	1000	1000	0	1000	0	0	0
Escherichia_coli_KTE64	0	0	1000	1000	1000	0	1000	0	0	0
Escherichia_coli_KTE65	0	0	1000	1000	1000	0	1000	0	0	0
Escherichia_coli_KTE66	0	0	1000	1000	1000	0	1000	0	0	0
Escherichia_coli_KTE67	0	0	1000	1000	1000	0	1000	0	0	0
Escherichia_coli_KTE68	0	0	1000	1000	1000	0	1000	0	0	0
Escherichia_coli_KTE69	0	0	1000	1000	1000	0	1000	0	0	0
Escherichia_coli_KTE7	0	0	1000	1000	1000	0	1000	0	0	0
Escherichia_coli_KTE70	0	0	1000	1000	1000	0	1000	0	0	0
Escherichia_coli_KTE71	0	0	1000	1000	1000	0	1000	0	0	0
Escherichia_coli_KTE72	0	0	1000	1000	1000	0	1000	0	0	0
Escherichia_coli_KTE73	0	0	1000	1000	1000	0	1000	0	0	0
Escherichia_coli_KTE74	0	0	1000	1000	1000	0	1000	0	0	0
Escherichia_coli_KTE75	0	0	1000	1000	1000	0	1000	0	0	0
Escherichia_coli_KTE76	0	0	1000	1000	1000	0	1000	0	0	0
Escherichia_coli_KTE77	0	0	1000	1000	1000	0	1000	0	0	0
Escherichia_coli_KTE78	0	0	1000	1000	1000	0	1000	0	0	0
Escherichia_coli_KTE79	0	0	1000	1000	1000	0	1000	0	0	0
Escherichia_coli_KTE8	0	0	1000	1000	1000	0	1000	0	0	0
Escherichia_coli_KTE80	0	0	1000	1000	1000	0	1000	0	0	0
Escherichia_coli_KTE81	0	0	1000	1000	1000	0	1000	0	0	0
Escherichia_coli_KTE82	0	0	1000	1000	1000	0	1000	0	0	0
Escherichia_coli_KTE83	0	0	1000	1000	1000	0	1000	0	0	0
Escherichia_coli_KTE84	0	0	1000	1000	1000	0	1000	0	0	0
Escherichia_coli_KTE85	0	0	1000	1000	1000	0	1000	0	0	0

Escherichia_coli_KTE86	0	0	1000	1000	1000	0	1000	0	0	0
Escherichia_coli_KTE87	0	0	1000	1000	1000	0	1000	0	0	0
Escherichia_coli_KTE88	0	0	1000	1000	1000	0	1000	0	0	0
Escherichia_coli_KTE89	0	0	1000	1000	1000	0	1000	0	0	0
Escherichia_coli_KTE9	0	0	1000	1000	1000	0	1000	0	0	0
Escherichia_coli_KTE90	0	0	1000	1000	1000	0	1000	0	0	0
Escherichia_coli_KTE91	0	0	1000	1000	1000	0	1000	0	0	0
Escherichia_coli_KTE93	0	0	1000	1000	1000	0	1000	0	0	0
Escherichia_coli_KTE94	0	0	1000	1000	1000	0	1000	0	0	0
Escherichia_coli_KTE95	0	0	1000	1000	1000	0	1000	0	0	0
Escherichia_coli_KTE96	0	0	1000	1000	1000	0	1000	0	0	0
Escherichia_coli_KTE97	0	0	1000	1000	1000	0	1000	0	0	0
Escherichia_coli_KTE98	0	0	1000	1000	1000	0	1000	0	0	0
Escherichia_coli_KTE99	0	0	1000	1000	1000	0	1000	0	0	0
Escherichia_coli_LCT_EC106	0	0	1000	1000	1000	0	1000	0	0	0
Escherichia_coli_LCT_EC52	0	0	1000	1000	1000	0	1000	0	0	0
Escherichia_coli_LCT_EC59	0	0	1000	1000	1000	0	1000	0	0	0
Escherichia_coli_LF82	1000	0	1000	1000	1000	0	1000	0	0	0
Escherichia_coli_LT_68	0	0	1000	1000	1000	0	1000	0	0	0
Escherichia_coli_M605	0	0	1000	1000	1000	0	1000	0	0	0
Escherichia_coli_M718	0	0	1000	1000	1000	0	1000	0	0	0
Escherichia_coli_M863	0	0	1000	1000	1000	0	1000	0	0	0
Escherichia_coli_MA6	0	0	1000	1000	1000	0	1000	0	0	0
Escherichia_coli_MP020940_1	0	0	1000	1000	1000	0	1000	0	0	0
Escherichia_coli_MP020980_1	0	0	1000	1000	1000	0	1000	0	0	0
Escherichia_coli_MP020980_2	0	0	1000	1000	1000	0	1000	0	0	0
Escherichia_coli_MP021017_1	0	0	1000	1000	1000	0	1000	0	0	0
Escherichia_coli_MP021017_10	0	0	1000	1000	1000	0	1000	0	0	0
Escherichia_coli_MP021017_11	0	0	1000	1000	1000	0	1000	0	0	0
Escherichia_coli_MP021017_12	0	0	1000	1000	1000	0	1000	0	0	0
Escherichia_coli_MP021017_2	0	0	1000	1000	1000	0	1000	0	0	0
Escherichia_coli_MP021017_3	0	0	1000	1000	1000	0	1000	0	0	0
Escherichia_coli_MP021017_4	0	0	1000	1000	1000	0	1000	0	0	0
Escherichia_coli_MP021017_5	0	0	1000	1000	1000	0	1000	0	0	0
Escherichia_coli_MP021017_6	0	0	1000	1000	1000	0	1000	0	0	0
Escherichia_coli_MP021017_9	0	0	1000	1000	1000	0	1000	0	0	0
Escherichia_coli_MP021552_11	0	0	1000	1000	1000	0	1000	0	0	0
Escherichia_coli_MP021552_12	0	0	1000	1000	1000	0	1000	0	0	0
Escherichia_coli_MP021552_7	0	0	1000	1000	1000	0	1000	0	0	0
Escherichia_coli_MP021552_8	0	0	1000	1000	1000	0	1000	0	0	0
Escherichia_coli_MP021561_2	0	0	1000	1000	1000	0	1000	0	0	0
Escherichia_coli_MP021561_3	0	0	1000	1000	1000	0	1000	0	0	0
Escherichia_coli_MP021566_1	0	0	1000	1000	1000	0	1000	0	0	0
Escherichia_coli_MS_107_1	0	0	1000	1000	1000	0	1000	0	0	0
Escherichia_coli_MS_115_1	0	0	0	1000	1000	0	1000	0	0	0
Escherichia_coli_MS_116_1	0	0	1000	1000	1000	0	1000	0	0	0
Escherichia_coli_MS_119_7	0	0	1000	1000	1000	0	1000	0	0	0
Escherichia_coli_MS_124_1	0	0	1000	1000	1000	0	1000	0	0	0
Escherichia_coli_MS_145_7	0	0	1000	1000	1000	0	1000	0	0	0
Escherichia_coli_MS_146_1	0	0	1000	1000	1000	0	1000	0	0	0
Escherichia_coli_MS_182_1	0	0	1000	1000	1000	0	1000	0	0	0
Escherichia_coli_MS_185_1	0	0	1000	1000	1000	0	1000	0	0	0
Escherichia_coli_MS_187_1	0	0	1000	1000	1000	0	1000	0	0	0
Escherichia_coli_MS_196_1	0	0	1000	1000	1000	0	1000	0	0	0
Escherichia_coli_MS_198_1	0	0	1000	1000	1000	0	1000	0	0	0
Escherichia_coli_MS_200_1	0	0	1000	1000	1000	0	1000	0	0	0
Escherichia_coli_MS_21_1	0	0	1000	1000	1000	0	1000	0	0	0
Escherichia_coli_MS_45_1	0	0	1000	1000	1000	0	1000	0	0	0
Escherichia_coli_MS_69_1	0	0	1000	1000	1000	0	1000	0	0	0
Escherichia_coli_MS_78_1	0	0	1000	1000	1000	0	1000	0	0	0
Escherichia_coli_MS_79_10	0	0	1000	1000	1000	0	1000	0	0	0
Escherichia_coli_MS_84_1	0	0	1000	1000	1000	0	1000	0	0	0
Escherichia_coli_MS_85_1	0	0	1000	1000	1000	0	1000	0	0	0
Escherichia_coli_Mt1B1	0	0	1000	1000	1000	0	1000	0	0	0
Escherichia_coli_N1	0	0	1000	1000	1000	0	1000	0	0	0
Escherichia_coli_NA114	1000	0	1000	1000	1000	0	1000	0	0	0
Escherichia_coli_NC101	0	0	1000	1000	1000	0	1000	0	0	0
Escherichia_coli_NCCP15647	0	0	1000	1000	1000	0	1000	0	0	0
Escherichia_coli_NCCP15657	0	0	1000	1000	1000	0	1000	0	0	0
Escherichia_coli_NCCP15658	0	0	1000	1000	1000	0	1000	0	0	0
Escherichia_coli_NE037	0	0	1000	1000	1000	0	1000	0	0	0
Escherichia_coli_NE098	0	0	1000	1000	1000	0	1000	0	0	0
Escherichia_coli_NE1487	0	0	1000	1000	1000	0	1000	0	0	0
Escherichia_coli_Nissle_1917	0	0	1000	1000	1000	0	1000	0	0	0
Escherichia_coli_O08	0	0	1000	1000	1000	0	1000	0	0	0
Escherichia_coli_O103H2_str_12009	0	0	1000	1000	1000	0	1000	0	0	0
Escherichia_coli_O103H2_str_CVM9450	0	0	1000	1000	1000	0	1000	0	0	0
Escherichia_coli_O103H25_str_CVM9340	0	0	1000	1000	1000	0	1000	0	0	0
Escherichia_coli_O103H25_str_NIPH_11060424	0	0	1000	1000	1000	0	1000	0	0	0
Escherichia_coli_O104_H4_str_01_09591	0	0	1000	1000	1000	0	1000	0	0	0
Escherichia_coli_O104_H4_str_04_8351	0	0	1000	1000	1000	0	1000	0	0	0
Escherichia_coli_O104_H4_str_09_7901	0	0	1000	1000	1000	0	1000	0	0	0
Escherichia_coli_O104_H4_str_11_02030	0	0	1000	1000	1000	0	1000	0	0	0
Escherichia_coli_O104_H4_str_11_02033_1	0	0	1000	1000	1000	0	1000	0	0	0
Escherichia_coli_O104_H4_str_11_02092	0	0	1000	1000	1000	0	1000	0	0	0
Escherichia_coli_O104_H4_str_11_02093	0	0	1000	1000	1000	0	1000	0	0	0
Escherichia_coli_O104_H4_str_11_02281	0	0	1000	1000	1000	0	1000	0	0	0
Escherichia_coli_O104_H4_str_11_02318	0	0	1000	1000	1000	0	1000	0	0	0
Escherichia_coli_O104_H4_str_11_02913	0	0	1000	1000	1000	0	1000	0	0	0
Escherichia_coli_O104_H4_str_11_03439	0	0	1000	1000	1000	0	1000	0	0	0
Escherichia_coli_O104_H4_str_11_03943	0	0	1000	1000	1000	0	1000	0	0	0
Escherichia_coli_O104_H4_str_11_04080	0	0	1000	1000	1000	0	1000	0	0	0
Escherichia_coli_O104_H4_str_11_3677	0	0	1000	1000	1000	0	1000	0	0	0
Escherichia_coli_O104_H4_str_11_4404	0	0	1000	1000	1000	0	1000	0	0	0

Escherichia_coli_O104_H4_str_11_4522	0	0	1000	1000	1000	0	1000	0	0	0
Escherichia_coli_O104_H4_str_11_4623	0	0	1000	1000	1000	0	1000	0	0	0
Escherichia_coli_O104_H4_str_11_4632_C1	0	0	1000	1000	1000	0	1000	0	0	0
Escherichia_coli_O104_H4_str_11_4632_C2	0	0	1000	1000	1000	0	1000	0	0	0
Escherichia_coli_O104_H4_str_11_4632_C3	0	0	1000	1000	1000	0	1000	0	0	0
Escherichia_coli_O104_H4_str_11_4632_C4	0	0	1000	1000	1000	0	1000	0	0	0
Escherichia_coli_O104_H4_str_11_4632_C5	0	0	1000	1000	1000	0	1000	0	0	0
Escherichia_coli_O104_H4_str_2009EL_2050	1000	0	1000	1000	1000	0	1000	0	0	0
Escherichia_coli_O104_H4_str_2009EL_2071	1000	0	1000	1000	1000	0	1000	0	0	0
Escherichia_coli_O104_H4_str_2011C_3493	1000	0	1000	1000	1000	0	1000	0	0	0
Escherichia_coli_O104_H4_str_C227_11	0	0	1000	1000	1000	0	1000	0	0	0
Escherichia_coli_O104_H4_str_C236_11	0	0	1000	1000	1000	0	1000	0	0	0
Escherichia_coli_O104_H4_str_Ec11_4984	0	0	1000	1000	1000	0	1000	0	0	0
Escherichia_coli_O104_H4_str_Ec11_4986	0	0	1000	1000	1000	0	1000	0	0	0
Escherichia_coli_O104_H4_str_Ec11_4987	0	0	1000	1000	1000	0	1000	0	0	0
Escherichia_coli_O104_H4_str_Ec11_4988	0	0	1000	1000	1000	0	1000	0	0	0
Escherichia_coli_O104_H4_str_Ec11_5603	0	0	1000	1000	1000	0	1000	0	0	0
Escherichia_coli_O104_H4_str_Ec11_5604	0	0	1000	1000	1000	0	1000	0	0	0
Escherichia_coli_O104_H4_str_Ec11_6006	0	0	1000	1000	1000	0	1000	0	0	0
Escherichia_coli_O104_H4_str_Ec11_9450	0	0	1000	1000	1000	0	1000	0	0	0
Escherichia_coli_O104_H4_str_Ec11_9941	0	0	1000	1000	1000	0	1000	0	0	0
Escherichia_coli_O104_H4_str_Ec11_9990	0	0	1000	1000	1000	0	1000	0	0	0
Escherichia_coli_O104_H4_str_Ec12_0465	0	0	1000	1000	1000	0	1000	0	0	0
Escherichia_coli_O104_H4_str_Ec12_0466	0	0	1000	1000	1000	0	1000	0	0	0
Escherichia_coli_O104_H4_str_GOS1	0	0	1000	1000	1000	0	1000	0	0	0
Escherichia_coli_O104_H4_str_GOS2	0	0	1000	1000	1000	0	1000	0	0	0
Escherichia_coli_O104_H4_str_H112180280	0	0	1000	1000	1000	0	1000	0	0	0
Escherichia_coli_O104_H4_str_H112180282	0	0	1000	1000	1000	0	1000	0	0	0
Escherichia_coli_O104_H4_str_H112180283	0	0	1000	1000	1000	0	1000	0	0	0
Escherichia_coli_O104_H4_str_LB226692	0	0	1000	1000	1000	0	1000	0	0	0
Escherichia_coli_O104_H4_str_ON2010	0	0	1000	1000	1000	0	1000	0	0	0
Escherichia_coli_O104_H4_str_ON2011	0	0	1000	1000	1000	0	1000	0	0	0
Escherichia_coli_O104_H4_str_TY_2482	0	0	1000	1000	1000	0	1000	0	0	0
Escherichia_coli_O10K5LH4_str_ATCC_23506	0	0	1000	1000	1000	0	1000	0	0	0
Escherichia_coli_O111_H_str_11128	0	0	1000	1000	1000	0	1000	0	0	0
Escherichia_coli_O111_H11_str_CFSAN001630	0	0	1000	1000	1000	0	1000	0	0	0
Escherichia_coli_O111_H11_str_CVM9534	0	0	1000	1000	1000	0	1000	0	0	0
Escherichia_coli_O111_H11_str_CVM9553	0	0	1000	1000	1000	0	1000	0	0	0
Escherichia_coli_O111_H8_str_CFSAN001632	0	0	1000	1000	1000	0	1000	0	0	0
Escherichia_coli_O111_H8_str_CVM9570	0	0	1000	1000	1000	0	1000	0	0	0
Escherichia_coli_O111_H8_str_CVM9574	0	0	1000	1000	1000	0	1000	0	0	0
Escherichia_coli_O111_H8_str_CVM9602	0	0	1000	1000	1000	0	1000	0	0	0
Escherichia_coli_O111_H8_str_CVM9634	0	0	1000	1000	1000	0	1000	0	0	0
Escherichia_coli_O113_H21_str_CL_3	0	0	1000	1000	1000	0	1000	0	0	0
Escherichia_coli_O121_H19_str_MT_2	0	0	1000	1000	1000	0	1000	0	0	0
Escherichia_coli_O127_H27_str_C43_90	0	0	1000	1000	1000	0	1000	0	0	0
Escherichia_coli_O127_H6_str_E2348_69	1000	0	1000	1000	1000	0	1000	0	0	0
Escherichia_coli_O145_H28_str_4865_96	0	0	1000	1000	1000	0	1000	0	0	0
Escherichia_coli_O157_H_str_493_89	0	0	1000	1000	1000	0	1000	0	0	0
Escherichia_coli_O157_H_str_H_2687	0	0	1000	1000	1000	0	1000	0	0	0
Escherichia_coli_O157_H7_EDL933	0	0	1000	1000	1000	0	1000	0	0	0
Escherichia_coli_O157_H7_str_1044	0	0	1000	1000	1000	0	1000	0	0	0
Escherichia_coli_O157_H7_str_1125	0	0	1000	1000	1000	0	1000	0	0	0
Escherichia_coli_O157_H7_str_2149	0	0	1000	1000	1000	0	1000	0	0	0
Escherichia_coli_O157_H7_str_EC1212	0	0	1000	1000	1000	0	1000	0	0	0
Escherichia_coli_O157_H7_str_EC4009	0	0	1000	1000	1000	0	1000	0	0	0
Escherichia_coli_O157_H7_str_EC4042	0	0	1000	1000	1000	0	1000	0	0	0
Escherichia_coli_O157_H7_str_EC4045	0	0	1000	1000	1000	0	1000	0	0	0
Escherichia_coli_O157_H7_str_EC4084	0	0	1000	1000	1000	0	1000	0	0	0
Escherichia_coli_O157_H7_str_EC4115	1000	0	1000	1000	1000	0	1000	0	0	0
Escherichia_coli_O157_H7_str_EC4191	0	0	1000	1000	1000	0	1000	0	0	0
Escherichia_coli_O157_H7_str_EC536	0	0	1000	1000	1000	0	1000	0	0	0
Escherichia_coli_O157_H7_str_FRIK2000	0	0	1000	1000	1000	0	1000	0	0	0
Escherichia_coli_O157_H7_str_FRIK966	0	0	1000	1000	1000	0	1000	0	0	0
Escherichia_coli_O157_H7_str_G5101	0	0	1000	1000	1000	0	1000	0	0	0
Escherichia_coli_O157_H7_str_H093800014	0	0	1000	1000	1000	0	1000	0	0	0
Escherichia_coli_O157_H7_str_LSU_61	0	0	1000	1000	1000	0	1000	0	0	0
Escherichia_coli_O157_H7_str_Sakai	1000	0	0	1000	1000	0	1000	0	0	0
Escherichia_coli_O157_H7_str_TW14359	1000	0	1000	1000	1000	0	1000	0	0	0
Escherichia_coli_O157_H7_str_TW14588	0	0	1000	1000	1000	0	1000	0	0	0
Escherichia_coli_O157_str_NCCP15738	0	0	1000	1000	1000	0	1000	0	0	0
Escherichia_coli_O157_str_NCCP15739	0	0	1000	1000	1000	0	1000	0	0	0
Escherichia_coli_O25b_H4_ST131_str_EC958	0	0	1000	1000	1000	0	1000	0	0	0
Escherichia_coli_O26_H11_str_11368	1000	0	1000	1000	1000	0	1000	0	0	0
Escherichia_coli_O26_H11_str_CFSAN001629	0	0	1000	1000	1000	0	1000	0	0	0
Escherichia_coli_O26_H11_str_CVM10021	0	0	1000	1000	1000	0	1000	0	0	0
Escherichia_coli_O26_H11_str_CVM10026	0	0	1000	1000	1000	0	1000	0	0	0
Escherichia_coli_O26_H11_str_CVM10030	0	0	1000	1000	1000	0	1000	0	0	0
Escherichia_coli_O26_H11_str_CVM10224	0	0	1000	1000	1000	0	1000	0	0	0
Escherichia_coli_O26_H11_str_CVM9942	0	0	1000	1000	1000	0	1000	0	0	0
Escherichia_coli_O26_H11_str_CVM9952	0	0	1000	1000	1000	0	1000	0	0	0
Escherichia_coli_O32_H37_str_P4	0	0	1000	1000	1000	0	1000	0	0	0
Escherichia_coli_O45_H2_str_03_EN_705	0	0	1000	1000	1000	0	1000	0	0	0
Escherichia_coli_O5_K4_L_H4_str_ATCC_23502	0	0	1000	1000	1000	0	1000	0	0	0
Escherichia_coli_O55_H7_str_3256_97	0	0	1000	1000	1000	0	1000	0	0	0
Escherichia_coli_O55_H7_str_CB9615	1000	0	1000	1000	1000	0	1000	0	0	0
Escherichia_coli_O55_H7_str_USDA_5905	0	0	1000	1000	1000	0	1000	0	0	0
Escherichia_coli_O7_K1_str_CE10	0	0	1000	1000	1000	0	1000	0	0	0
Escherichia_coli_O83_H1_str_NRG_857C	0	0	1000	1000	1000	0	1000	0	0	0
Escherichia_coli_O91	0	0	1000	1000	1000	0	1000	0	0	0
Escherichia_coli_O91_H21_str_B2F1	0	0	1000	1000	1000	0	1000	0	0	0
Escherichia_coli_OK1180	0	0	1000	1000	1000	0	1000	0	0	0
Escherichia_coli_ONT_H33_str_C48_93	0	0	1000	1000	1000	0	1000	0	0	0
Escherichia_coli_P0298942_1	0	0	1000	1000	1000	0	1000	0	0	0

Escherichia_coli_P0298942_10	0	0	1000	1000	1000	0	1000	0	0	0
Escherichia_coli_P0298942_11	0	0	1000	1000	1000	0	1000	0	0	0
Escherichia_coli_P0298942_12	0	0	1000	1000	1000	0	1000	0	0	0
Escherichia_coli_P0298942_14	0	0	1000	1000	1000	0	1000	0	0	0
Escherichia_coli_P0298942_15	0	0	1000	1000	1000	0	1000	0	0	0
Escherichia_coli_P0298942_3	0	0	1000	1000	1000	0	1000	0	0	0
Escherichia_coli_P0298942_4	0	0	1000	1000	1000	0	1000	0	0	0
Escherichia_coli_P0298942_6	0	0	1000	1000	1000	0	1000	0	0	0
Escherichia_coli_P0298942_7	0	0	1000	1000	1000	0	1000	0	0	0
Escherichia_coli_P0299438_10	0	0	1000	1000	1000	0	1000	0	0	0
Escherichia_coli_P0299438_11	0	0	1000	1000	1000	0	1000	0	0	0
Escherichia_coli_P0299438_2	0	0	1000	1000	1000	0	1000	0	0	0
Escherichia_coli_P0299438_3	0	0	1000	1000	1000	0	1000	0	0	0
Escherichia_coli_P0299438_4	0	0	1000	1000	1000	0	1000	0	0	0
Escherichia_coli_P0299438_5	0	0	1000	1000	1000	0	1000	0	0	0
Escherichia_coli_P0299438_6	0	0	1000	1000	1000	0	1000	0	0	0
Escherichia_coli_P0299438_7	0	0	1000	1000	1000	0	1000	0	0	0
Escherichia_coli_P0299438_8	0	0	1000	1000	1000	0	1000	0	0	0
Escherichia_coli_P0299438_9	0	0	1000	1000	1000	0	1000	0	0	0
Escherichia_coli_P0299483_1	0	0	1000	1000	1000	0	1000	0	0	0
Escherichia_coli_P0299483_2	0	0	1000	1000	1000	0	1000	0	0	0
Escherichia_coli_P0299483_3	0	0	1000	1000	1000	0	1000	0	0	0
Escherichia_coli_P02997067_6	0	0	1000	1000	1000	0	1000	0	0	0
Escherichia_coli_P0299917_1	0	0	1000	1000	1000	0	1000	0	0	0
Escherichia_coli_P0299917_10	0	0	1000	1000	1000	0	1000	0	0	0
Escherichia_coli_P0299917_2	0	0	1000	1000	1000	0	1000	0	0	0
Escherichia_coli_P0299917_4	0	0	1000	1000	1000	0	1000	0	0	0
Escherichia_coli_P0299917_5	0	0	1000	1000	1000	0	1000	0	0	0
Escherichia_coli_P0299917_6	0	0	1000	1000	1000	0	1000	0	0	0
Escherichia_coli_P0299917_7	0	0	1000	1000	1000	0	1000	0	0	0
Escherichia_coli_P0299917_8	0	0	1000	1000	1000	0	1000	0	0	0
Escherichia_coli_P0299917_9	0	0	1000	1000	1000	0	1000	0	0	0
Escherichia_coli_P0301867_1	0	0	0	1000	1000	0	1000	0	0	0
Escherichia_coli_P0301867_11	0	0	0	1000	1000	0	1000	0	0	0
Escherichia_coli_P0301867_13	0	0	0	1000	1000	0	1000	0	0	0
Escherichia_coli_P0301867_2	0	0	0	1000	1000	0	1000	0	0	0
Escherichia_coli_P0301867_3	0	0	0	1000	1000	0	1000	0	0	0
Escherichia_coli_P0301867_4	0	0	0	1000	1000	0	1000	0	0	0
Escherichia_coli_P0301867_5	0	0	0	1000	1000	0	1000	0	0	0
Escherichia_coli_P0301867_7	0	0	0	1000	1000	0	1000	0	0	0
Escherichia_coli_P0301904_3	0	0	1000	1000	1000	0	1000	0	0	0
Escherichia_coli_P0302293_10	0	0	1000	1000	1000	0	1000	0	0	0
Escherichia_coli_P0302293_2	0	0	1000	1000	1000	0	1000	0	0	0
Escherichia_coli_P0302293_3	0	0	1000	1000	1000	0	1000	0	0	0
Escherichia_coli_P0302293_4	0	0	1000	1000	1000	0	1000	0	0	0
Escherichia_coli_P0302293_6	0	0	1000	1000	1000	0	1000	0	0	0
Escherichia_coli_P0302293_7	0	0	1000	1000	1000	0	1000	0	0	0
Escherichia_coli_P0302293_8	0	0	1000	1000	1000	0	1000	0	0	0
Escherichia_coli_P0302293_9	0	0	1000	1000	1000	0	1000	0	0	0
Escherichia_coli_P0302308_1	0	0	1000	1000	1000	0	1000	0	0	0
Escherichia_coli_P0302308_10	0	0	1000	1000	1000	0	1000	0	0	0
Escherichia_coli_P0302308_11	0	0	1000	1000	1000	0	1000	0	0	0
Escherichia_coli_P0302308_12	0	0	1000	1000	1000	0	1000	0	0	0
Escherichia_coli_P0302308_13	0	0	1000	1000	1000	0	1000	0	0	0
Escherichia_coli_P0302308_14	0	0	1000	1000	1000	0	1000	0	0	0
Escherichia_coli_P0302308_2	0	0	1000	1000	1000	0	1000	0	0	0
Escherichia_coli_P0302308_3	0	0	1000	1000	1000	0	1000	0	0	0
Escherichia_coli_P0302308_4	0	0	1000	1000	1000	0	1000	0	0	0
Escherichia_coli_P0302308_5	0	0	1000	1000	1000	0	1000	0	0	0
Escherichia_coli_P0304777_1	0	0	1000	1000	1000	0	1000	0	0	0
Escherichia_coli_P0304777_10	0	0	1000	1000	1000	0	1000	0	0	0
Escherichia_coli_P0304777_11	0	0	1000	1000	1000	0	1000	0	0	0
Escherichia_coli_P0304777_12	0	0	1000	1000	1000	0	1000	0	0	0
Escherichia_coli_P0304777_13	0	0	1000	1000	1000	0	1000	0	0	0
Escherichia_coli_P0304777_14	0	0	1000	1000	1000	0	1000	0	0	0
Escherichia_coli_P0304777_15	0	0	1000	1000	1000	0	1000	0	0	0
Escherichia_coli_P0304777_2	0	0	1000	1000	1000	0	1000	0	0	0
Escherichia_coli_P0304777_3	0	0	1000	1000	1000	0	1000	0	0	0
Escherichia_coli_P0304777_4	0	0	1000	1000	1000	0	1000	0	0	0
Escherichia_coli_P0304777_7	0	0	1000	1000	1000	0	1000	0	0	0
Escherichia_coli_P0304777_8	0	0	1000	1000	1000	0	1000	0	0	0
Escherichia_coli_P0304777_9	0	0	1000	1000	1000	0	1000	0	0	0
Escherichia_coli_P0304799_3	0	0	1000	1000	1000	0	1000	0	0	0
Escherichia_coli_P0304816_1	0	0	1000	1000	1000	0	1000	0	0	0
Escherichia_coli_P0304816_11	0	0	1000	1000	1000	0	1000	0	0	0
Escherichia_coli_P0304816_12	0	0	1000	1000	1000	0	1000	0	0	0
Escherichia_coli_P0304816_13	0	0	1000	1000	1000	0	1000	0	0	0
Escherichia_coli_P0304816_14	0	0	1000	1000	1000	0	1000	0	0	0
Escherichia_coli_P0304816_15	0	0	1000	1000	1000	0	1000	0	0	0
Escherichia_coli_P0304816_2	0	0	1000	1000	1000	0	1000	0	0	0
Escherichia_coli_P0304816_3	0	0	1000	1000	1000	0	1000	0	0	0
Escherichia_coli_P0304816_4	0	0	1000	1000	1000	0	1000	0	0	0
Escherichia_coli_P0304816_5	0	0	1000	1000	1000	0	1000	0	0	0
Escherichia_coli_P0304816_6	0	0	1000	1000	1000	0	1000	0	0	0
Escherichia_coli_P0304816_7	0	0	1000	1000	1000	0	1000	0	0	0
Escherichia_coli_P0304816_8	0	0	1000	1000	1000	0	1000	0	0	0
Escherichia_coli_P0304816_9	0	0	1000	1000	1000	0	1000	0	0	0
Escherichia_coli_P0305260_1	0	0	1000	1000	1000	0	1000	0	0	0
Escherichia_coli_P0305260_10	0	0	1000	1000	1000	0	1000	0	0	0
Escherichia_coli_P0305260_11	0	0	1000	1000	1000	0	1000	0	0	0
Escherichia_coli_P0305260_12	0	0	1000	1000	1000	0	1000	0	0	0
Escherichia_coli_P0305260_13	0	0	1000	1000	1000	0	1000	0	0	0
Escherichia_coli_P0305260_15	0	0	1000	1000	1000	0	1000	0	0	0
Escherichia_coli_P0305260_2	0	0	1000	1000	1000	0	1000	0	0	0

Escherichia_coli_P0305260_3	0	0	1000	1000	1000	0	1000	0	0	0
Escherichia_coli_P0305260_4	0	0	1000	1000	1000	0	1000	0	0	0
Escherichia_coli_P0305260_5	0	0	1000	1000	1000	0	1000	0	0	0
Escherichia_coli_P0305260_6	0	0	1000	1000	1000	0	1000	0	0	0
Escherichia_coli_P0305260_7	0	0	1000	1000	1000	0	1000	0	0	0
Escherichia_coli_P0305260_8	0	0	1000	1000	1000	0	1000	0	0	0
Escherichia_coli_P0305260_9	0	0	1000	1000	1000	0	1000	0	0	0
Escherichia_coli_p0305293_1	0	0	1000	1000	1000	0	1000	0	0	0
Escherichia_coli_p0305293_10	0	0	1000	1000	1000	0	1000	0	0	0
Escherichia_coli_p0305293_11	0	0	1000	1000	1000	0	1000	0	0	0
Escherichia_coli_p0305293_12	0	0	1000	1000	1000	0	1000	0	0	0
Escherichia_coli_p0305293_14	0	0	1000	1000	1000	0	1000	0	0	0
Escherichia_coli_p0305293_15	0	0	1000	1000	1000	0	1000	0	0	0
Escherichia_coli_p0305293_2	0	0	1000	1000	1000	0	1000	0	0	0
Escherichia_coli_p0305293_3	0	0	1000	1000	1000	0	1000	0	0	0
Escherichia_coli_p0305293_4	0	0	1000	1000	1000	0	1000	0	0	0
Escherichia_coli_p0305293_5	0	0	1000	1000	1000	0	1000	0	0	0
Escherichia_coli_p0305293_6	0	0	1000	1000	1000	0	1000	0	0	0
Escherichia_coli_p0305293_7	0	0	1000	1000	1000	0	1000	0	0	0
Escherichia_coli_p0305293_8	0	0	1000	1000	1000	0	1000	0	0	0
Escherichia_coli_p0305293_9	0	0	1000	1000	1000	0	1000	0	0	0
Escherichia_coli_P12b	1000	0	1000	1000	1000	0	1000	0	0	0
Escherichia_coli_PA10	0	0	1000	1000	1000	0	1000	0	0	0
Escherichia_coli_PA11	0	0	1000	1000	1000	0	1000	0	0	0
Escherichia_coli_PA13	0	0	1000	1000	1000	0	1000	0	0	0
Escherichia_coli_PA14	0	0	1000	1000	1000	0	1000	0	0	0
Escherichia_coli_PA15	0	0	1000	1000	1000	0	1000	0	0	0
Escherichia_coli_PA19	0	0	1000	1000	1000	0	1000	0	0	0
Escherichia_coli_PA2	0	0	1000	1000	1000	0	1000	0	0	0
Escherichia_coli_PA22	0	0	1000	1000	1000	0	1000	0	0	0
Escherichia_coli_PA23	0	0	1000	1000	1000	0	1000	0	0	0
Escherichia_coli_PA24	0	0	1000	1000	1000	0	1000	0	0	0
Escherichia_coli_PA25	0	0	1000	1000	1000	0	1000	0	0	0
Escherichia_coli_PA28	0	0	1000	1000	1000	0	1000	0	0	0
Escherichia_coli_PA3	0	0	1000	1000	1000	0	1000	0	0	0
Escherichia_coli_PA31	0	0	1000	1000	1000	0	1000	0	0	0
Escherichia_coli_PA32	0	0	1000	1000	1000	0	1000	0	0	0
Escherichia_coli_PA33	0	0	1000	1000	1000	0	1000	0	0	0
Escherichia_coli_PA34	0	0	1000	1000	1000	0	1000	0	0	0
Escherichia_coli_PA35	0	0	1000	1000	1000	0	1000	0	0	0
Escherichia_coli_PA38	0	0	1000	1000	1000	0	1000	0	0	0
Escherichia_coli_PA4	0	0	1000	1000	1000	0	1000	0	0	0
Escherichia_coli_PA40	0	0	1000	1000	1000	0	1000	0	0	0
Escherichia_coli_PA41	0	0	1000	1000	1000	0	1000	0	0	0
Escherichia_coli_PA42	0	0	1000	1000	1000	0	1000	0	0	0
Escherichia_coli_PA45	0	0	1000	1000	1000	0	1000	0	0	0
Escherichia_coli_PA47	0	0	1000	1000	1000	0	1000	0	0	0
Escherichia_coli_PA48	0	0	1000	1000	1000	0	1000	0	0	0
Escherichia_coli_PA5	0	0	1000	1000	1000	0	1000	0	0	0
Escherichia_coli_PA7	0	0	1000	1000	1000	0	1000	0	0	0
Escherichia_coli_PA8	0	0	1000	1000	1000	0	1000	0	0	0
Escherichia_coli_PA9	0	0	1000	1000	1000	0	1000	0	0	0
Escherichia_coli_PCN033	0	0	1000	1000	1000	0	1000	0	0	0
Escherichia_coli_RN587_1	0	0	1000	1000	1000	0	1000	0	0	0
Escherichia_coli_S17	0	0	1000	1000	1000	0	1000	0	0	0
Escherichia_coli_S88	1000	0	1000	1000	1000	0	1000	0	0	0
Escherichia_coli_SCI_07	0	0	1000	1000	1000	0	1000	0	0	0
Escherichia_coli_SE11	1000	0	0	1000	1000	0	1000	0	0	0
Escherichia_coli_SE15	1000	0	1000	1000	1000	0	1000	0	0	0
Escherichia_coli_SEPT362	0	0	1000	1000	1000	0	1000	0	0	0
Escherichia_coli_SMS_3_5	1000	0	1000	1000	1000	0	1000	0	0	0
Escherichia_coli_STEC_7v	0	0	1000	1000	1000	0	1000	0	0	0
Escherichia_coli_STEC_94C	0	0	1000	1000	1000	0	1000	0	0	0
Escherichia_coli_STEC_B2F1	0	0	1000	1000	1000	0	1000	0	0	0
Escherichia_coli_STEC_C165_02	0	0	1000	1000	1000	0	1000	0	0	0
Escherichia_coli_STEC_DG131_3	0	0	1000	1000	1000	0	1000	0	0	0
Escherichia_coli_STEC_EH250	0	0	1000	1000	1000	0	1000	0	0	0
Escherichia_coli_STEC_H_1_8	0	0	0	1000	1000	0	1000	0	0	0
Escherichia_coli_STEC_MHI813	0	0	1000	1000	1000	0	1000	0	0	0
Escherichia_coli_STEC_O31	0	0	1000	1000	1000	0	1000	0	0	0
Escherichia_coli_STEC_S1191	0	0	1000	1000	1000	0	1000	0	0	0
Escherichia_coli_str_clone_D_i14	0	0	1000	1000	1000	0	1000	0	0	0
Escherichia_coli_str_clone_D_i2	0	0	1000	1000	1000	0	1000	0	0	0
Escherichia_coli_str_K_12_substr_DH10B	0	0	1000	1000	1000	0	1000	0	0	0
Escherichia_coli_str_K_12_substr_MDS42	0	0	1000	1000	1000	0	1000	0	0	0
Escherichia_coli_str_K_12_substr_MG1655	0	0	0	1000	1000	0	1000	0	0	0
Escherichia_coli_str_K_12_substr_MG1655star	0	0	1000	1000	1000	0	1000	0	0	0
Escherichia_coli_str_K_12_substr_W3110	0	0	1000	1000	1000	0	1000	0	0	0
Escherichia_coli_SWW33	0	0	1000	1000	1000	0	1000	0	0	0
Escherichia_coli_TA007	0	0	1000	1000	1000	0	1000	0	0	0
Escherichia_coli_TA124	0	0	1000	1000	1000	0	1000	0	0	0
Escherichia_coli_TA143	0	0	1000	1000	1000	0	1000	0	0	0
Escherichia_coli_TA206	0	0	1000	1000	1000	0	1000	0	0	0
Escherichia_coli_TA271	0	0	1000	1000	1000	0	1000	0	0	0
Escherichia_coli_TA280	0	0	1000	1000	1000	0	1000	0	0	0
Escherichia_coli_ThroopD	0	0	1000	1000	1000	0	1000	0	0	0
Escherichia_coli_TT12B	0	0	1000	1000	1000	0	1000	0	0	0
Escherichia_coli_TW00353	0	0	1000	1000	1000	0	1000	0	0	0
Escherichia_coli_TW06591	0	0	1000	1000	1000	0	1000	0	0	0
Escherichia_coli_TW07793	0	0	1000	1000	1000	0	1000	0	0	0
Escherichia_coli_TW07945	0	0	1000	1000	1000	0	1000	0	0	0
Escherichia_coli_TW09098	0	0	1000	1000	1000	0	1000	0	0	0
Escherichia_coli_TW09109	0	0	1000	1000	1000	0	1000	0	0	0
Escherichia_coli_TW10119	0	0	1000	1000	1000	0	1000	0	0	0

Escherichia_coli_TW10246	0	0	1000	1000	1000	0	1000	0	0	0
Escherichia_coli_TW10509	0	0	1000	1000	1000	0	1000	0	0	0
Escherichia_coli_TW10598	0	0	1000	1000	1000	0	1000	0	0	0
Escherichia_coli_TW10722	0	0	1000	1000	1000	0	1000	0	0	0
Escherichia_coli_TW10828	0	0	1000	1000	1000	0	1000	0	0	0
Escherichia_coli_TW11039	0	0	1000	1000	1000	0	1000	0	0	0
Escherichia_coli_TW11681	0	0	1000	1000	1000	0	1000	0	0	0
Escherichia_coli_TW14301	0	0	1000	1000	1000	0	1000	0	0	0
Escherichia_coli_TW14425	0	0	1000	1000	1000	0	1000	0	0	0
Escherichia_coli_TW15901	0	0	1000	1000	1000	0	1000	0	0	0
Escherichia_coli_TX1999	0	0	1000	1000	1000	0	1000	0	0	0
Escherichia_coli_UM146	1000	0	1000	1000	1000	0	1000	0	0	0
Escherichia_coli_UMN026	1000	0	1000	1000	1000	0	1000	0	0	0
Escherichia_coli_UMNF18	0	0	1000	1000	1000	0	1000	0	0	0
Escherichia_coli_UMNK88	1000	0	1000	1000	1000	0	1000	0	0	0
Escherichia_coli_UTI89_UPEC	1000	0	0	1000	1000	0	1000	0	0	0
Escherichia_coli_W	1000	0	1000	1000	1000	0	1000	0	0	0
Escherichia_coli_W26	0	0	1000	1000	1000	0	1000	0	0	0
Escherichia_coli_XH001	0	0	1000	1000	1000	0	1000	0	0	0
Escherichia_coli_XH140A	0	0	1000	1000	1000	0	1000	0	0	0
Escherichia_coli_Xuzhou21	1000	0	1000	1000	1000	0	1000	0	0	0
Escherichia_fergusonii_ATCC_35469	1000	0	0	0	1000	0	1000	0	0	1000
Escherichia_fergusonii_B253	0	0	1000	0	1000	0	1000	0	0	0
Escherichia_fergusonii_ECD227	0	0	1000	0	1000	0	1000	0	0	0
Escherichia_hermannii_NBRC_105704	0	0	0	0	1000	0	1000	0	0	1000
Escherichia_sp_1_1_43	0	0	0	0	1000	0	0	0	0	0
Escherichia_sp_3_2_53FAA	0	0	0	1000	1000	0	1000	0	0	0
Escherichia_sp_4_1_40B	0	0	0	1000	1000	0	1000	0	0	0
Escherichia_sp_TW09308	0	0	1000	1000	1000	0	1000	0	0	0
Ethanoligenens_harbinense_YUAN_3	121.2121212	0	0	0	0	0	0	0	0	0
Eubacterium_biforme_DSM_3989	0	0	0	0	0	0	1000	0	1000	0
Eubacterium_brachy_ATCC_33089	0	0	0	0	0	0	1000	0	0	0
Eubacterium_callanderi_KIST612	0	0	0	0	1000	0	1000	0	0	0
Eubacterium_cellulosolvens_6	0	0	0	0	0	0	1000	0	0	0
Eubacterium_cellulosolvens_LD2006	0	0	0	0	0	0	1000	0	0	0
Eubacterium_cylindroides_T2_87	0	0	0	0	0	0	0	0	1000	0
Eubacterium_desmolans_ATCC_43058	0	0	1000	0	0	0	1000	0	0	0
Eubacterium_dolichum_DSM_3991	0	0	0	0	0	0	1000	0	0	0
Eubacterium_eligens_ATCC_27750	0	0	0	1000	1000	0	0	0	1000	0
Eubacterium_hallii_DSM_3353	0	0	0	0	1000	0	0	0	1000	0
Eubacterium_hallii_L2_7	0	0	0	0	1000	0	0	0	0	0
Eubacterium_infirmum_F0142	0	0	0	0	1000	0	1000	0	0	0
Eubacterium_limosum_DSM_20543	0	0	0	0	1000	0	1000	0	0	0
Eubacterium_limosum_ERR2221373	0	0	0	0	1000	0	1000	0	0	0
Eubacterium_oxidoreducens_DSM_3217	0	0	0	0	0	0	0	0	0	0
Eubacterium_plexicaudatum_ASF492	0	0	0	0	1000	0	0	0	0	0
Eubacterium_pyruvativorans_KHPC4	0	0	0	0	0	0	0	0	1000	0
Eubacterium_ramulus_ATCC_29099	0	0	1000	0	1000	0	0	0	0	0
Eubacterium_ramulus_ERR1022349	0	0	0	0	1000	0	0	0	0	0
Eubacterium_rectale_ATCC_33656	0	0	1000	0	1000	0	0	0	0	0
Eubacterium_rectale_DSM_17629	0	0	0	0	1000	0	0	0	0	0
Eubacterium_rectale_ERR1022318	0	0	0	0	1000	0	1000	0	0	0
Eubacterium_rectale_ERR1022399	0	0	0	0	1000	0	1000	0	0	0
Eubacterium_rectale_ERR1022482	0	0	0	0	1000	0	1000	0	0	0
Eubacterium_rectale_ERR1203958	0	0	0	0	1000	0	1000	0	0	0
Eubacterium_rectale_ERR1204052	0	0	0	0	1000	0	1000	0	0	0
Eubacterium_rectale_ERR2221160	0	0	0	0	1000	0	1000	0	0	0
Eubacterium_rectale_ERR2230060	0	0	0	0	1000	0	1000	0	0	0
Eubacterium_rectale_M104_1	0	0	0	0	1000	0	0	0	0	0
Eubacterium_saphenum_ATCC_49989	0	0	0	0	0	0	1000	0	0	0
Eubacterium_siraeum_70_3	0	0	0	1000	0	0	0	0	0	0
Eubacterium_siraeum_DSM_15702	0	0	0	1000	0	0	0	0	0	0
Eubacterium_siraeum_V10Sc8a	0	0	0	1000	0	0	0	0	0	0
Eubacterium_sp_14_2	0	0	0	0	0	0	0	0	0	0
Eubacterium_sp_3_1_31	0	0	0	0	0	0	1000	0	0	0
Eubacterium_sulci_ATCC_35585	0	0	0	0	0	0	1000	0	1000	0
Eubacterium_ventriosum_ATCC_27560	0	0	0	0	0	0	0	0	0	0
Eubacterium_yurii_ATCC_43714	0	0	0	0	1000	0	1000	0	0	0
Eubacterium_yurii_subsp_margaretiae_ATCC_437	0	0	0	0	1000	0	1000	0	0	0
Ewingella_americana_ATCC_33852	0	0	0	0	1000	0	1000	0	0	0
Ewingella_americana_BRK18a	0	0	0	0	1000	0	1000	0	0	0
Exiguobacterium_aurantiacum_DSM_6208	0	0	0	0	1000	0	1000	0	0	0
Exiguobacterium_marinum_ERR2221224	0	0	0	0	1000	0	1000	0	0	0
Exiguobacterium_nov_ERR2221364	0	0	0	0	1000	0	1000	0	0	0
Exiguobacterium_undae_ERR2221363	0	0	0	0	1000	0	1000	0	0	0
Extibacter_muris_DSM_28560	0	0	0	0	1000	0	1000	0	0	0
Facklamia_languida_CCUG_37842	0	0	0	0	1000	0	1000	0	0	0
Faecalibacterium_cf_ERR1022328	0	0	0	0	1000	0	1000	0	0	0
Faecalibacterium_cf_prausnitzii_KLE1255	0	0	0	1000	1000	0	1000	0	0	0
Faecalibacterium_prausnitzii_A2_165	0	0	0	1000	0	0	1000	0	0	0
Faecalibacterium_prausnitzii_ERR1022279	0	0	0	0	0	0	1000	0	0	0
Faecalibacterium_prausnitzii_ERR1022327	0	0	0	0	1000	0	1000	0	0	0
Faecalibacterium_prausnitzii_ERR1022484	0	0	0	0	1000	0	1000	0	0	0
Faecalibacterium_prausnitzii_ERR2221188	0	0	0	0	0	0	1000	0	0	0
Faecalibacterium_prausnitzii_L2_6	0	0	0	1000	1000	0	1000	0	0	0
Faecalibacterium_prausnitzii_M21_2	0	0	0	1000	1000	0	1000	0	0	0
Faecalibacterium_prausnitzii_SL3_3	0	0	0	1000	1000	0	1000	0	0	0
Filifactor_alocis_ATCC_35896	0	0	0	0	0	0	666.6666667	0	0	0
Finegoldia_magna_ACS_171_V_Col3	0	0	0	0	1000	0	1000	0	0	0
Finegoldia_magna_ATCC_29328	0	0	0	0	1000	0	1000	0	0	0
Finegoldia_magna_ATCC_53516	0	0	0	0	1000	0	1000	0	0	0
Finegoldia_magna_BVS033A4	0	0	0	0	1000	0	1000	0	0	0
Finegoldia_magna_SY403409CC001050417	0	0	0	0	1000	0	1000	0	0	0
Flaviflexus_massiliensis_SIT4	0	0	0	0	0	0	1000	0	0	0

Flavobacterium_cheniae_CGMCC_16844	0	0	1000	0	1000	0	0	0	0	0
Flavobacterium_cheniae_DSM_22462	0	0	1000	0	1000	0	0	0	0	0
Flavobacterium_cucumis_DSM_18830	0	0	1000	0	0	0	0	0	0	0
Flavonifractor_plautii_ATCC_29863	0	0	0	0	1000	0	1000	0	0	0
Flavonifractor_plautii_ERR1022312	0	0	0	0	1000	0	1000	0	0	0
Flavonifractor_plautii_ERR1022406	0	0	0	0	1000	0	1000	0	0	0
Flavonifractor_plautii_ERR1022420	0	0	0	0	1000	0	1000	0	0	0
Flavonifractor_plautii_ERR1022446	0	0	0	0	1000	0	1000	0	0	0
Flavonifractor_plautii_ERR2221213	0	0	0	0	1000	0	1000	0	0	0
Flavonifractor_plautii_ERR2221305	0	0	0	0	1000	0	1000	0	0	0
Flavonifractor_plautii_YL31	0	0	0	0	1000	0	1000	0	0	0
Frisingicoccus_caecimuris_DSM_28559	0	0	0	0	1000	0	1000	0	0	0
Fructobacillus_durionis_DSM_19113	0	0	0	0	0	0	1000	1000	0	0
Fructobacillus_pseudoficulneus_DSM_15468	0	0	0	0	0	0	1000	1000	0	0
Fructobacillus_tropaeoli_F214_1	0	0	0	0	0	0	1000	1000	0	0
Fulvimarina_pelagi_DSM_15513	0	0	1000	0	1000	0	0	1000	1000	0
Fulvimarina_pelagi_HTCC2506	0	0	1000	0	1000	0	0	1000	1000	0
Fusicatenibacter_saccharivorans_ERR1022307	0	0	0	0	1000	0	1000	0	0	0
Fusicatenibacter_saccharivorans_ERR1022400	0	0	0	0	1000	0	1000	0	0	0
Fusicatenibacter_saccharivorans_ERR1022437	0	0	0	0	1000	0	1000	0	0	0
Fusobacterium_gonidiaformans_3_1_5R	0	0	0	0	1000	0	1000	0	1000	0
Fusobacterium_gonidiaformans_ATCC_25563	0	0	0	0	1000	0	1000	0	1000	0
Fusobacterium_mortiferum_ATCC_9817	0	0	0	0	1000	0	1000	0	1000	0
Fusobacterium_necrogenes_NCTC10723	0	0	0	0	750	0	1000	0	0	0
Fusobacterium_necrophorum_D12	0	0	0	0	1000	0	1000	0	1000	0
Fusobacterium_necrophorum_subsp_funduliform	0	0	0	0	1000	0	1000	0	1000	0
Fusobacterium_necrophorum_subsp_funduliform	0	0	0	0	1000	0	1000	0	1000	0
Fusobacterium_necrophorum_subsp_funduliform	0	0	0	0	1000	0	1000	0	1000	0
Fusobacterium_nucleatum_subsp_animalis_11_3_	0	0	0	0	1000	0	1000	0	1000	0
Fusobacterium_nucleatum_subsp_animalis_21_1A	0	0	0	0	1000	0	1000	0	1000	0
Fusobacterium_nucleatum_subsp_animalis_3_1_3	0	0	0	0	1000	0	1000	0	1000	0
Fusobacterium_nucleatum_subsp_animalis_4_8	0	0	0	0	1000	0	1000	0	1000	0
Fusobacterium_nucleatum_subsp_animalis_7_1	0	0	0	0	1000	0	1000	0	1000	0
Fusobacterium_nucleatum_subsp_animalis_ATCC	0	0	0	0	1000	0	1000	0	1000	0
Fusobacterium_nucleatum_subsp_animalis_D11	0	0	0	0	1000	0	1000	0	1000	0
Fusobacterium_nucleatum_subsp_animalis_OT_4	0	0	0	0	1000	0	1000	0	1000	0
Fusobacterium_nucleatum_subsp_nucleatum_AT	0	0	0	0	1000	0	1000	0	1000	0
Fusobacterium_nucleatum_subsp_nucleatum_AT	0	0	0	0	1000	0	1000	0	1000	0
Fusobacterium_nucleatum_subsp_polymorphum_	0	0	0	0	1000	0	1000	0	1000	0
Fusobacterium_nucleatum_subsp_polymorphum_	0	0	0	0	1000	0	1000	0	1000	0
Fusobacterium_nucleatum_subsp_vincentii_3_1_	0	0	0	0	1000	0	1000	0	1000	0
Fusobacterium_nucleatum_subsp_vincentii_3_1_	0	0	0	0	1000	0	1000	0	1000	0
Fusobacterium_nucleatum_subsp_vincentii_4_1_	0	0	0	0	1000	0	1000	0	1000	0
Fusobacterium_nucleatum_subsp_vincentii_ATCC	0	0	0	0	1000	0	1000	0	1000	0
Fusobacterium_perfoetens_ATCC_29250	0	0	1000	0	1000	0	1000	0	0	0
Fusobacterium_periodonticum_1_1_41FAA	0	0	0	0	1000	0	1000	0	1000	0
Fusobacterium_periodonticum_2_1_31	0	0	0	0	1000	0	1000	0	1000	0
Fusobacterium_periodonticum_ATCC_33693	0	0	0	0	1000	0	1000	0	1000	0
Fusobacterium_russii_ATCC_25533	0	0	0	0	1000	0	1000	0	0	0
Fusobacterium_ulcerans_12_1B	0	0	0	0	1000	0	1000	0	1000	0
Fusobacterium_ulcerans_ATCC_49185	0	0	0	0	1000	0	1000	0	1000	0
Fusobacterium_varium_ATCC_27725	0	0	1000	0	1000	0	1000	0	1000	0
Gardnerella_vaginalis_00703Bmash	0	0	0	0	1000	0	1000	0	0	0
Gardnerella_vaginalis_14019_MetR	0	0	0	0	1000	0	1000	0	0	0
Gardnerella_vaginalis_JCP7672	0	0	0	0	1000	0	1000	0	0	0
Gemella_haemolysans_ATCC_10379	0	0	0	0	1000	0	1000	0	0	1000
Gemella_haemolysans_M341	0	0	0	0	1000	0	1000	0	0	0
Gemella_morbillorum_M424	0	0	0	0	1000	0	1000	0	0	1000
Gemella_sanguinis_ATCC700632	0	0	0	0	1000	0	1000	0	0	1000
Gemella_sanguinis_M325	0	0	0	0	1000	0	1000	0	0	1000
Gemmata_obscuriglobus_DSM_5831	0	0	0	0	1000	0	0	0	0	0
Gemmata_obscuriglobus_UQM_2246	0	0	0	0	1000	0	0	0	0	0
Gemmatimonas_aurantiaca_T_27	198.3471074	0	0	0	1000	0	0	0	0	0
Gemmiger_formicilis_ATCC_27749	0	0	0	0	0	0	1000	0	0	0
Gemmiger_formicilis_ERR1203953	0	0	0	0	0	0	1000	0	0	0
Gemmiger_formicilis_ERR1204047	0	0	0	0	0	0	1000	0	0	0
Gemmiger_formicilis_ERR2221191	0	0	0	0	0	0	1000	0	0	0
Geobacillus_stearothermophilus_ATCC_7953	0	0	1000	0	1000	0	1000	0	0	0
Geobacillus_vulcani_PSS1	0	0	1000	0	1000	0	1000	0	0	0
Gordonia_rubripertincta_NBRC_101908	0	0	1000	0	0	0	0	0	0	1000
Gordonia_terrae_C_6	0	0	1000	0	0	0	0	0	1000	1000
Gordonia_terrae_NBRC_100016	0	0	1000	0	0	0	0	0	1000	1000
Gordonibacter_pamelaeae_7_10_1_bT_DSM_193	0	0	0	0	0	0	0	0	1000	0
Gordonibacter_pamelaeae_ERR2221155	0	0	0	0	0	0	1000	0	1000	0
Gordonibacter_pamelaeae_ERR2221387	0	0	0	0	0	0	1000	0	1000	0
Gottschalkia_acidurici_9a	0	0	0	0	1000	0	1000	0	0	0
Gottschalkia_purinilyticum_DSM_1384	0	0	0	0	1000	0	1000	0	0	0
Gracilibacter_sp_BRH_c7a	0	0	1000	0	1000	0	0	0	0	0
Granulicatella_adiacens_ATCC_49175	0	0	0	0	0	0	1000	0	0	0
Granulicatella_elegans_ATCC_700633	0	0	0	0	0	0	1000	0	0	0
Grimontia_hollisae_CIP_101886	0	0	0	0	0	0	1000	0	0	1000
Guyana_massiliensis_LF_3	0	0	0	0	1000	0	1000	0	0	0
Haemophilus_aegyptius_ATCC_11116	0	0	0	0	0	0	1000	0	0	0
Haemophilus_haemolyticus_HK386	0	0	0	0	1000	0	1000	0	0	0
Haemophilus_haemolyticus_M19501	0	0	0	0	0	0	1000	0	0	1000
Haemophilus_haemolyticus_M21127	0	0	0	0	1000	0	1000	0	0	0
Haemophilus_haemolyticus_M21621	0	0	0	0	0	0	1000	0	0	0
Haemophilus_haemolyticus_M21639	0	0	0	0	1000	0	1000	0	0	0
Haemophilus_influenzae_10810	0	0	0	0	0	0	1000	0	0	0
Haemophilus_influenzae_477	0	0	0	0	0	0	1000	0	0	0
Haemophilus_influenzae_6P18H1	0	0	0	0	0	0	1000	0	0	0
Haemophilus_influenzae_7P49H1	0	0	0	0	0	0	1000	0	0	0
Haemophilus_influenzae_86_028NP	0	0	0	0	0	0	1000	0	0	0
Haemophilus_influenzae_F3031	0	0	0	0	0	0	1000	0	0	0

Haemophilus_influenzae_F3047	0	0	0	0	0	0	1000	0	0	0
Haemophilus_influenzae_Hi375	0	0	0	0	0	0	1000	0	0	0
Haemophilus_influenzae_NT127	0	0	0	0	0	0	1000	0	0	0
Haemophilus_influenzae_PittAA	0	0	0	0	0	0	1000	0	0	0
Haemophilus_influenzae_PittEE	0	0	0	0	0	0	1000	0	0	0
Haemophilus_influenzae_PittGG	0	0	0	0	0	0	1000	0	0	0
Haemophilus_influenzae_PittHH	0	0	0	0	0	0	1000	0	0	0
Haemophilus_influenzae_PittII	0	0	0	0	0	0	1000	0	0	0
Haemophilus_influenzae_R2846	0	0	0	0	0	0	1000	0	0	1000
Haemophilus_influenzae_R2866	0	0	0	0	0	0	1000	0	0	0
Haemophilus_influenzae_Rd_KW20	0	0	0	0	0	0	1000	0	0	0
Haemophilus_influenzae_RdAW	0	0	0	0	0	0	1000	0	0	0
Haemophilus_parahaemolyticus_CCUG_3716_371	0	0	0	0	0	0	1000	0	0	0
Haemophilus_parahaemolyticus_G321	0	0	0	0	0	0	1000	0	0	0
Haemophilus_parahaemolyticus_HK385	0	0	0	0	0	0	1000	0	0	0
Haemophilus_parainfluenzae_215035_2_ISO5	0	0	0	0	0	0	1000	0	0	0
Haemophilus_parainfluenzae_ATCC_33392	0	0	0	0	1000	0	1000	0	0	0
Haemophilus_parainfluenzae_HK2019	0	0	0	0	1000	0	0	0	0	0
Haemophilus_parainfluenzae_HK262	0	0	0	0	1000	0	1000	0	0	0
Haemophilus_parainfluenzae_T3T1	0	0	0	0	1000	0	1000	0	0	1000
Haemophilus_paraphrohaemolyticus_CCUG_3718	0	0	0	0	0	0	1000	0	0	0
Haemophilus_paraphrohaemolyticus_HK411	0	0	0	0	0	0	1000	0	0	0
Haemophilus_pittmaniae_HK_85	0	0	0	0	1000	0	1000	0	0	0
Haemophilus_pittmaniae_NCTC13334	0	0	0	0	0	0	1000	0	0	0
Haemophilus_sputorum_CCUG_13788	0	0	0	0	1000	0	1000	0	0	0
Haemophilus_sputorum_HK_2154	0	0	0	0	1000	0	1000	0	0	0
Hafnia_alvei_ATCC_51873	0	0	0	0	1000	0	1000	0	0	1000
Hafnia_alvei_BIDMC_31	0	1000	0	0	1000	0	1000	0	0	1000
Hafnia_alvei_ERR2221402	0	0	0	0	1000	0	1000	0	0	0
Hafnia_alvei_FB1	0	0	0	0	1000	0	1000	0	0	0
Hafnia_alvei_HUMV_5920	0	0	0	0	1000	0	1000	0	0	0
Hafnia_paralvei_FDAARGOS_158	0	0	0	0	1000	0	1000	0	0	0
Halococcus_morrhuae_DSM1307	0	0	1000	0	1000	0	0	0	0	1000
Halomonas_campaniensis_LS21	0	0	1000	0	1000	0	0	0	0	0
Helicobacter_apodemus_SCJK1	0	0	1000	0	0	0	0	0	0	0
Helicobacter_bilis_ATCC_43879	0	0	1000	0	1000	0	0	0	0	0
Helicobacter_bilis_WiWa	0	0	1000	0	1000	0	0	0	0	0
Helicobacter_canadensis_MIT_98_5491	0	0	0	0	1000	0	1000	0	0	0
Helicobacter_cinaedi_CCUG_18818	0	0	0	0	0	0	1000	0	0	0
Helicobacter_fennelliae_MRY12_0050	0	0	1000	0	0	0	1000	0	0	0
Helicobacter_fennelliae_NCTC13102	0	0	1000	0	0	0	1000	0	0	0
Helicobacter_hepaticus_ATCC_51449	0	0	1000	0	0	0	1000	0	0	0
Helicobacter_pullorum_MIT_98_5489	0	0	0	0	1000	0	0	0	0	0
Helicobacter_pylori_2017	0	0	1000	0	0	0	1000	0	0	0
Helicobacter_pylori_2018	0	0	1000	0	0	0	1000	0	0	0
Helicobacter_pylori_26695	0	0	1000	0	1000	0	1000	0	1000	0
Helicobacter_pylori_26695_1MET	0	0	1000	0	0	0	1000	0	0	0
Helicobacter_pylori_35A	0	0	1000	0	0	0	1000	0	0	0
Helicobacter_pylori_51	0	0	1000	0	0	0	1000	0	0	0
Helicobacter_pylori_52	0	0	1000	0	0	0	1000	0	0	0
Helicobacter_pylori_83	0	0	1000	0	0	0	1000	0	0	0
Helicobacter_pylori_8A3	0	0	1000	0	1000	0	1000	0	0	0
Helicobacter_pylori_908	0	0	1000	0	0	0	1000	0	0	0
Helicobacter_pylori_98_10	0	0	1000	0	0	0	1000	0	0	0
Helicobacter_pylori_A45	0	0	1000	0	0	0	1000	0	0	0
Helicobacter_pylori_Aklavik117	0	0	1000	0	0	0	1000	0	0	0
Helicobacter_pylori_Aklavik86	0	0	0	0	0	0	1000	0	0	0
Helicobacter_pylori_B128	0	0	1000	0	0	0	1000	0	0	0
Helicobacter_pylori_B38	0	0	1000	0	0	0	1000	0	0	0
Helicobacter_pylori_B45	0	0	1000	0	0	0	1000	0	0	0
Helicobacter_pylori_B8	0	0	1000	0	0	0	1000	0	0	0
Helicobacter_pylori_BCS100H1	0	0	1000	0	0	0	1000	0	0	0
Helicobacter_pylori_BM013A	0	0	1000	0	0	0	1000	0	0	0
Helicobacter_pylori_CCHI_33	0	0	1000	0	0	0	1000	0	0	0
Helicobacter_pylori_CPY1124	0	0	1000	0	1000	0	1000	0	0	0
Helicobacter_pylori_CPY1313	0	0	1000	0	0	0	1000	0	0	0
Helicobacter_pylori_CPY1662	0	0	1000	0	0	0	1000	0	0	0
Helicobacter_pylori_CPY1962	0	0	1000	0	0	0	1000	0	0	0
Helicobacter_pylori_CPY3281	0	0	1000	0	1000	0	1000	0	0	0
Helicobacter_pylori_CPY6081	0	0	1000	0	0	0	1000	0	0	0
Helicobacter_pylori_CPY6261	0	0	1000	0	0	0	1000	0	0	0
Helicobacter_pylori_CPY6271	0	0	1000	0	0	0	1000	0	0	0
Helicobacter_pylori_CPY6311	0	0	1000	0	0	0	1000	0	0	0
Helicobacter_pylori_Cuz20	0	0	1000	0	0	0	1000	0	0	0
Helicobacter_pylori_ELS37	0	0	1000	0	0	0	1000	0	0	0
Helicobacter_pylori_F16	0	0	1000	0	0	0	1000	0	0	0
Helicobacter_pylori_F30	0	0	1000	0	0	0	1000	0	0	0
Helicobacter_pylori_F32	0	0	1000	0	0	0	1000	0	0	0
Helicobacter_pylori_F57	0	0	1000	0	0	0	1000	0	0	0
Helicobacter_pylori_G27	0	0	1000	0	0	0	800	0	0	0
Helicobacter_pylori_GAM100Ai	0	0	1000	0	0	0	1000	0	0	0
Helicobacter_pylori_GAM101Biv	0	0	1000	0	0	0	1000	0	0	0
Helicobacter_pylori_GAM103Bi	0	0	1000	0	0	0	1000	0	0	0
Helicobacter_pylori_GAM105Ai	0	0	1000	0	0	0	1000	0	0	0
Helicobacter_pylori_GAM114Ai	0	0	1000	0	0	0	1000	0	0	0
Helicobacter_pylori_GAM115Ai	0	0	1000	0	0	0	1000	0	0	0
Helicobacter_pylori_GAM118Bi	0	0	1000	0	0	0	1000	0	0	0
Helicobacter_pylori_GAM119Bi	0	0	1000	0	0	0	1000	0	0	0
Helicobacter_pylori_GAM120Ai	0	0	1000	0	0	0	1000	0	0	0
Helicobacter_pylori_GAM121Aii	0	0	1000	0	0	0	1000	0	0	0
Helicobacter_pylori_GAM201Ai	0	0	1000	0	0	0	1000	0	0	0
Helicobacter_pylori_GAM210Bi	0	0	1000	0	0	0	1000	0	0	0
Helicobacter_pylori_GAM231Ai	0	0	1000	0	0	0	1000	0	0	0
Helicobacter_pylori_GAM239Bi	0	0	1000	0	0	0	1000	0	0	0

Helicobacter_pylori_GAM244Ai	0	0	1000	0	0	0	1000	0	0	0
Helicobacter_pylori_GAM245Ai	0	0	1000	0	0	0	1000	0	0	0
Helicobacter_pylori_GAM246Ai	0	0	1000	0	0	0	1000	0	0	0
Helicobacter_pylori_GAM249T	0	0	1000	0	0	0	1000	0	0	0
Helicobacter_pylori_GAM250AFi	0	0	1000	0	0	0	1000	0	0	0
Helicobacter_pylori_GAM250T	0	0	1000	0	0	0	1000	0	0	0
Helicobacter_pylori_GAM252Bi	0	0	1000	0	0	0	1000	0	0	0
Helicobacter_pylori_GAM252T	0	0	1000	0	0	0	1000	0	0	0
Helicobacter_pylori_GAM254Ai	0	0	1000	0	0	0	1000	0	0	0
Helicobacter_pylori_GAM260ASi	0	0	1000	0	0	0	1000	0	0	0
Helicobacter_pylori_GAM260Bi	0	0	1000	0	0	0	1000	0	0	0
Helicobacter_pylori_GAM260BSi	0	0	1000	0	0	0	1000	0	0	0
Helicobacter_pylori_GAM263BFi	0	0	1000	0	0	0	1000	0	0	0
Helicobacter_pylori_GAM264Ai	0	0	1000	0	0	0	1000	0	0	0
Helicobacter_pylori_GAM265BSii	0	0	1000	0	0	0	1000	0	0	0
Helicobacter_pylori_GAM270ASi	0	0	1000	0	0	0	1000	0	0	0
Helicobacter_pylori_GAM42Ai	0	0	1000	0	0	0	1000	0	0	0
Helicobacter_pylori_GAM80Ai	0	0	1000	0	0	0	1000	0	0	0
Helicobacter_pylori_GAM83Bi	0	0	1000	0	0	0	1000	0	0	0
Helicobacter_pylori_GAM83T	0	0	1000	0	0	0	1000	0	0	0
Helicobacter_pylori_GAM96Ai	0	0	1000	0	0	0	1000	0	0	0
Helicobacter_pylori_Gambia94_24	0	0	1000	0	0	0	1000	0	0	0
Helicobacter_pylori_GAMchJs106B	0	0	1000	0	0	0	1000	0	0	0
Helicobacter_pylori_GAMchJs114i	0	0	1000	0	0	0	1000	0	0	0
Helicobacter_pylori_GAMchJs117Ai	0	0	1000	0	0	0	1000	0	0	0
Helicobacter_pylori_GAMchJs124i	0	0	1000	0	0	0	1000	0	0	0
Helicobacter_pylori_GAMchJs136i	0	0	1000	0	0	0	1000	0	0	0
Helicobacter_pylori_HLJHP193	0	0	1000	0	1000	0	1000	0	0	0
Helicobacter_pylori_HLJHP253	0	0	1000	0	0	0	1000	0	0	0
Helicobacter_pylori_HLJHP256	0	0	1000	0	0	0	1000	0	0	0
Helicobacter_pylori_HLJHP271	0	0	1000	0	0	0	1000	0	0	0
Helicobacter_pylori_Hp_A_11	0	0	1000	0	0	0	1000	0	0	0
Helicobacter_pylori_Hp_A_14	0	0	1000	0	0	0	1000	0	0	0
Helicobacter_pylori_Hp_A_16	0	0	1000	0	0	0	1000	0	0	0
Helicobacter_pylori_Hp_A_17	0	0	1000	0	0	0	1000	0	0	0
Helicobacter_pylori_Hp_A_20	0	0	1000	0	0	0	1000	0	0	0
Helicobacter_pylori_Hp_A_26	0	0	1000	0	0	0	1000	0	0	0
Helicobacter_pylori_Hp_A_27	0	0	1000	0	0	0	1000	0	0	0
Helicobacter_pylori_Hp_A_4	0	0	1000	0	0	0	1000	0	0	0
Helicobacter_pylori_Hp_A_5	0	0	1000	0	0	0	1000	0	0	0
Helicobacter_pylori_Hp_A_6	0	0	1000	0	0	0	1000	0	0	0
Helicobacter_pylori_Hp_A_8	0	0	1000	0	0	0	1000	0	0	0
Helicobacter_pylori_Hp_A_9	0	0	1000	0	0	0	1000	0	0	0
Helicobacter_pylori_Hp_H_1	0	0	1000	0	0	0	1000	0	0	0
Helicobacter_pylori_Hp_H_10	0	0	1000	0	0	0	1000	0	0	0
Helicobacter_pylori_Hp_H_11	0	0	1000	0	0	0	1000	0	0	0
Helicobacter_pylori_Hp_H_16	0	0	1000	0	0	0	1000	0	0	0
Helicobacter_pylori_Hp_H_18	0	0	1000	0	0	0	1000	0	0	0
Helicobacter_pylori_Hp_H_19	0	0	1000	0	0	0	1000	0	0	0
Helicobacter_pylori_Hp_H_21	0	0	1000	0	0	0	1000	0	0	0
Helicobacter_pylori_Hp_H_23	0	0	0	0	0	0	1000	0	0	0
Helicobacter_pylori_Hp_H_24	0	0	1000	0	0	0	1000	0	0	0
Helicobacter_pylori_Hp_H_24b	0	0	1000	0	0	0	1000	0	0	0
Helicobacter_pylori_Hp_H_24c	0	0	1000	0	0	0	1000	0	0	0
Helicobacter_pylori_Hp_H_27	0	0	1000	0	1000	0	1000	0	0	0
Helicobacter_pylori_Hp_H_28	0	0	1000	0	0	0	1000	0	0	0
Helicobacter_pylori_Hp_H_29	0	0	1000	0	0	0	1000	0	0	0
Helicobacter_pylori_Hp_H_3	0	0	1000	0	0	0	1000	0	0	0
Helicobacter_pylori_Hp_H_30	0	0	1000	0	0	0	1000	0	0	0
Helicobacter_pylori_Hp_H_34	0	0	1000	0	0	0	1000	0	0	0
Helicobacter_pylori_Hp_H_36	0	0	1000	0	0	0	1000	0	0	0
Helicobacter_pylori_Hp_H_4	0	0	1000	0	0	0	1000	0	0	0
Helicobacter_pylori_Hp_H_41	0	0	1000	0	0	0	1000	0	0	0
Helicobacter_pylori_Hp_H_42	0	0	1000	0	0	0	1000	0	0	0
Helicobacter_pylori_Hp_H_43	0	0	1000	0	0	0	1000	0	0	0
Helicobacter_pylori_Hp_H_44	0	0	1000	0	0	0	1000	0	0	0
Helicobacter_pylori_Hp_H_45	0	0	1000	0	0	0	1000	0	0	0
Helicobacter_pylori_Hp_H_5b	0	0	1000	0	0	0	1000	0	0	0
Helicobacter_pylori_Hp_H_6	0	0	1000	0	0	0	1000	0	0	0
Helicobacter_pylori_Hp_H_9	0	0	1000	0	0	0	1000	0	0	0
Helicobacter_pylori_Hp_M1	0	0	1000	0	0	0	1000	0	0	0
Helicobacter_pylori_Hp_M2	0	0	1000	0	0	0	1000	0	0	0
Helicobacter_pylori_Hp_M3	0	0	1000	0	0	0	1000	0	0	0
Helicobacter_pylori_Hp_M4	0	0	1000	0	0	0	1000	0	0	0
Helicobacter_pylori_Hp_M5	0	0	1000	0	0	0	1000	0	0	0
Helicobacter_pylori_Hp_M6	0	0	1000	0	0	0	1000	0	0	0
Helicobacter_pylori_Hp_M9	0	0	1000	0	0	0	1000	0	0	0
Helicobacter_pylori_Hp_P_1	0	0	1000	0	0	0	1000	0	0	0
Helicobacter_pylori_Hp_P_11	0	0	1000	0	0	0	1000	0	0	0
Helicobacter_pylori_Hp_P_11b	0	0	1000	0	0	0	1000	0	0	0
Helicobacter_pylori_Hp_P_13	0	0	1000	0	1000	0	1000	0	0	0
Helicobacter_pylori_Hp_P_13b	0	0	1000	0	0	0	1000	0	0	0
Helicobacter_pylori_Hp_P_15	0	0	1000	0	0	0	1000	0	0	0
Helicobacter_pylori_Hp_P_15b	0	0	1000	0	0	0	800	0	0	0
Helicobacter_pylori_Hp_P_16	0	0	1000	0	0	0	1000	0	0	0
Helicobacter_pylori_Hp_P_1b	0	0	1000	0	0	0	1000	0	0	0
Helicobacter_pylori_Hp_P_2	0	0	1000	0	0	0	1000	0	0	0
Helicobacter_pylori_Hp_P_23	0	0	1000	0	0	0	1000	0	0	0
Helicobacter_pylori_Hp_P_25	0	0	1000	0	0	0	1000	0	0	0
Helicobacter_pylori_Hp_P_25c	0	0	1000	0	0	0	1000	0	0	0
Helicobacter_pylori_Hp_P_25d	0	0	1000	0	0	0	1000	0	0	0
Helicobacter_pylori_Hp_P_26	0	0	1000	0	0	0	1000	0	0	0
Helicobacter_pylori_Hp_P_28b	0	0	1000	0	0	0	1000	0	0	0
Helicobacter_pylori_Hp_P_2b	0	0	1000	0	0	0	1000	0	0	0

Helicobacter_pylori_Hp_P_3	0	0	1000	0	0	0	1000	0	0	0
Helicobacter_pylori_Hp_P_30	0	0	1000	0	0	0	1000	0	0	0
Helicobacter_pylori_Hp_P_3b	0	0	1000	0	0	0	1000	0	0	0
Helicobacter_pylori_Hp_P_4	0	0	1000	0	0	0	1000	0	0	0
Helicobacter_pylori_Hp_P_41	0	0	1000	0	0	0	1000	0	0	0
Helicobacter_pylori_Hp_P_4c	0	0	1000	0	0	0	1000	0	0	0
Helicobacter_pylori_Hp_P_4d	0	0	1000	0	0	0	1000	0	0	0
Helicobacter_pylori_Hp_P_62	0	0	1000	0	0	0	1000	0	0	0
Helicobacter_pylori_Hp_P_74	0	0	1000	0	0	0	1000	0	0	0
Helicobacter_pylori_Hp_P_8	0	0	1000	0	0	0	1000	0	0	0
Helicobacter_pylori_Hp_P_8b	0	0	1000	0	0	0	1000	0	0	0
Helicobacter_pylori_HP116Bi	0	0	1000	0	0	0	1000	0	0	0
Helicobacter_pylori_Hp238	0	0	1000	0	0	0	1000	0	0	0
Helicobacter_pylori_HP250AFii	0	0	1000	0	0	0	1000	0	0	0
Helicobacter_pylori_HP250AFiii	0	0	1000	0	0	0	1000	0	0	0
Helicobacter_pylori_HP250AFiV	0	0	1000	0	0	0	1000	0	0	0
Helicobacter_pylori_HP250ASi	0	0	1000	0	0	0	1000	0	0	0
Helicobacter_pylori_HP250ASii	0	0	1000	0	0	0	1000	0	0	0
Helicobacter_pylori_HP250BFii	0	0	1000	0	0	0	1000	0	0	0
Helicobacter_pylori_HP250BFiii	0	0	1000	0	0	0	1000	0	0	0
Helicobacter_pylori_HP250BFiV	0	0	1000	0	0	0	1000	0	0	0
Helicobacter_pylori_HP250BSi	0	0	1000	0	0	0	1000	0	0	0
Helicobacter_pylori_HP260AFi	0	0	1000	0	0	0	1000	0	0	0
Helicobacter_pylori_HP260AFii	0	0	1000	0	0	0	1000	0	0	0
Helicobacter_pylori_HP260ASii	0	0	1000	0	0	0	1000	0	0	0
Helicobacter_pylori_HP260BFii	0	0	1000	0	0	0	1000	0	0	0
Helicobacter_pylori_HP260Bi	0	0	1000	0	0	0	1000	0	0	0
Helicobacter_pylori_HPAG1	0	0	1000	0	0	0	800	0	0	0
Helicobacter_pylori_HUP_B14	0	0	1000	0	0	0	1000	0	0	0
Helicobacter_pylori_India7	0	0	1000	0	0	0	1000	0	0	0
Helicobacter_pylori_J99	0	0	1000	0	0	0	1000	0	0	0
Helicobacter_pylori_Lithuania75	0	0	1000	0	0	0	1000	0	0	0
Helicobacter_pylori_MALT	0	0	1000	0	0	0	1000	0	0	0
Helicobacter_pylori_N6	0	0	1000	0	0	0	1000	0	0	0
Helicobacter_pylori_NAK7	0	0	1000	0	0	0	1000	0	0	0
Helicobacter_pylori_NQ1671	0	0	1000	0	0	0	800	0	0	0
Helicobacter_pylori_NQ1707	0	0	1000	0	0	0	1000	0	0	0
Helicobacter_pylori_NQ1712	0	0	1000	0	0	0	1000	0	0	0
Helicobacter_pylori_NQ315	0	0	1000	0	0	0	1000	0	0	0
Helicobacter_pylori_NQ352	0	0	1000	0	1000	0	1000	0	0	0
Helicobacter_pylori_NQ367	0	0	1000	0	0	0	1000	0	0	0
Helicobacter_pylori_NQ392	0	0	1000	0	0	0	1000	0	0	0
Helicobacter_pylori_NQ4044	0	0	1000	0	0	0	1000	0	0	0
Helicobacter_pylori_NQ4053	0	0	1000	0	0	0	1000	0	0	0
Helicobacter_pylori_NQ4060	0	0	1000	0	0	0	800	0	0	0
Helicobacter_pylori_NQ4076	0	0	1000	0	0	0	1000	0	0	0
Helicobacter_pylori_NQ4099	0	0	1000	0	0	0	1000	0	0	0
Helicobacter_pylori_NQ4110	0	0	1000	0	0	0	1000	0	0	0
Helicobacter_pylori_NQ4161	0	0	1000	0	0	0	1000	0	0	0
Helicobacter_pylori_NQ4191	0	0	1000	0	0	0	1000	0	0	0
Helicobacter_pylori_NQ4200	0	0	1000	0	0	0	1000	0	0	0
Helicobacter_pylori_NQ4216	0	0	1000	0	0	0	1000	0	0	0
Helicobacter_pylori_NQ4228	0	0	1000	0	0	0	1000	0	0	0
Helicobacter_pylori_OK113	0	0	1000	0	0	0	1000	0	0	0
Helicobacter_pylori_OK310	0	0	1000	0	0	0	1000	0	0	0
Helicobacter_pylori_P12	0	0	1000	0	0	0	800	0	0	0
Helicobacter_pylori_PeCan18	0	0	1000	0	0	0	1000	0	0	0
Helicobacter_pylori_PeCan4	0	0	1000	0	0	0	1000	0	0	0
Helicobacter_pylori_Puno120	0	0	1000	0	0	0	1000	0	0	0
Helicobacter_pylori_Puno135	0	0	1000	0	0	0	1000	0	0	0
Helicobacter_pylori_R036d	0	0	1000	0	0	0	1000	0	0	0
Helicobacter_pylori_R037c	0	0	1000	0	0	0	1000	0	0	0
Helicobacter_pylori_R038b	0	0	1000	0	0	0	1000	0	0	0
Helicobacter_pylori_R046Wa	0	0	1000	0	0	0	1000	0	0	0
Helicobacter_pylori_R055a	0	0	1000	0	0	0	1000	0	0	0
Helicobacter_pylori_R056a	0	0	1000	0	0	0	1000	0	0	0
Helicobacter_pylori_R32b	0	0	1000	0	0	0	1000	0	0	0
Helicobacter_pylori_Rif1	0	0	1000	0	0	0	1000	0	0	0
Helicobacter_pylori_Rif2	0	0	1000	0	0	0	1000	0	0	0
Helicobacter_pylori_Sat464	0	0	1000	0	0	0	1000	0	0	0
Helicobacter_pylori_Shi112	0	0	1000	0	0	0	1000	0	0	0
Helicobacter_pylori_Shi169	0	0	1000	0	0	0	1000	0	0	0
Helicobacter_pylori_Shi417	0	0	1000	0	0	0	1000	0	0	0
Helicobacter_pylori_Shi470	0	0	1000	0	0	0	1000	0	0	0
Helicobacter_pylori_SJM180	0	0	1000	0	0	0	1000	0	0	0
Helicobacter_pylori_SNT49	0	0	1000	0	0	0	1000	0	0	0
Helicobacter_pylori_SouthAfrica7	0	0	1000	0	0	0	1000	0	0	0
Helicobacter_pylori_UM007	0	0	1000	0	0	0	1000	0	0	0
Helicobacter_pylori_UM018	0	0	1000	0	0	0	1000	0	0	0
Helicobacter_pylori_UM034	0	0	1000	0	0	0	1000	0	0	0
Helicobacter_pylori_UM037	0	0	1000	0	0	0	1000	0	0	0
Helicobacter_pylori_UM045	0	0	1000	0	0	0	1000	0	0	0
Helicobacter_pylori_UM054	0	0	1000	0	0	0	1000	0	0	0
Helicobacter_pylori_UM066	0	0	1000	0	0	0	1000	0	0	0
Helicobacter_pylori_UM299	0	0	1000	0	0	0	1000	0	0	0
Helicobacter_pylori_v225d	0	0	1000	0	0	0	1000	0	0	0
Helicobacter_pylori_XZ274	0	0	1000	0	0	0	1000	0	0	0
Helicobacter_rodentium_ATCC_700285	0	0	1000	0	0	0	0	0	0	0
Helicobacter_trogontum_50960_7	0	0	1000	0	0	0	500	0	0	0
Helicobacter_typhlonius_ATCC_BAA_367	0	0	1000	0	0	0	1000	0	0	0
Helicobacter_winghamensis_ATCC_BAA_430	0	0	0	0	1000	0	0	0	0	0
Herbaspirillum_huttiense_1147	0	1000	1000	0	1000	0	0	0	0	0
Herbaspirillum_huttiense_AU6965_10_3	0	1000	1000	0	1000	0	0	0	0	0
Herbaspirillum_huttiense_NFYY_53159	0	1000	1000	0	1000	0	0	0	0	0

Herbaspirillum_huttiense_subsp_putei_IAM_1503	0	1000	1000	0	1000	0	0	0	0	0
Holdemania_filiformis_VPI_J1_31B_1_DSM_12042	0	0	0	0	0	0	1000	0	0	0
Holdemania_massiliensis_AP2	0	0	0	0	0	0	1000	0	0	0
Hydrotalea_flava_isolate_2	0	0	0	0	0	0	812.3002422	0	0	0
Intestinibacter_bartlettii_ERR1022395	0	0	0	0	1000	0	1000	0	1000	0
Intestinibacter_bartlettii_ERR1022418	0	0	0	0	1000	0	1000	0	1000	0
Intestinibacter_nov_ERR2221096	0	0	0	0	1000	0	1000	0	0	0
Intestinimonas_butyriciproducens_AF211	0	0	0	0	0	0	1000	0	0	0
Intestinimonas_butyriciproducens_ERR1022450	0	0	0	0	0	0	1000	0	0	0
Intestinimonas_butyriciproducens_SRB_521_5_I	0	0	0	0	0	0	1000	0	0	0
Intestinimonas_nov_ERR1022454	0	0	0	0	1000	0	1000	0	0	0
Intestinimonas_nov_ERR171285	0	0	0	0	1000	0	1000	0	0	0
Jeddahella_massiliensis_OL_1	0	0	1000	0	1000	0	0	0	0	0
Johnsonella_ignava_ATCC_51276	0	0	0	0	0	0	1000	0	0	0
Jonquetella_anthropi_DSM_22815	0	0	1000	0	1000	0	1000	0	0	0
Jonquetella_anthropi_E3_33_E1	0	0	1000	0	1000	0	1000	0	0	0
Kallipyga_massiliensis_ph2	0	0	1000	0	1000	0	1000	0	0	0
Kandleria_vitulina_DSM_20405	0	0	0	0	1000	0	0	0	0	0
Kingella_denitrificans_ATCC_33394	0	0	0	0	1000	0	0	0	0	0
Kingella_denitrificans_isolate_GC77	0	0	0	0	1000	0	0	0	0	0
Kingella_oralis_ATCC_51147	0	0	0	0	1000	0	1000	0	0	1000
Klebsiella_aerogenes_CAV1320	0	0	0	0	1000	0	1000	0	0	0
Klebsiella_aerogenes_G7	0	0	0	0	1000	0	1000	0	0	0
Klebsiella_michiganensis_10_5242	0	0	0	0	1000	0	1000	0	0	0
Klebsiella_michiganensis_10_5250	0	0	0	0	1000	0	1000	0	0	0
Klebsiella_michiganensis_FDAARGOS_66	0	0	0	0	1000	0	1000	0	0	0
Klebsiella_michiganensis_M1	0	0	0	0	1000	0	1000	0	0	0
Klebsiella_michiganensis_MGH87	0	0	0	0	1000	0	1000	0	0	0
Klebsiella_oxytoca_10_5243	0	0	0	0	1000	0	1000	0	0	0
Klebsiella_oxytoca_10_5245	0	0	0	0	1000	0	1000	0	0	0
Klebsiella_oxytoca_10_5248	0	0	0	0	1000	0	1000	0	0	0
Klebsiella_oxytoca_11492_1	0	0	0	0	1000	0	1000	0	0	0
Klebsiella_oxytoca_CAV1099	0	0	0	0	1000	0	1000	0	0	0
Klebsiella_oxytoca_CAV1335	0	0	0	0	1000	0	1000	0	0	0
Klebsiella_oxytoca_CAV1374	0	0	0	0	1000	0	1000	0	0	0
Klebsiella_oxytoca_CHS143	0	0	0	0	1000	0	1000	0	0	0
Klebsiella_oxytoca_E718	833.3333333	0	0	0	1000	0	1000	0	0	0
Klebsiella_oxytoca_ERR2221287	0	0	0	0	1000	0	1000	0	0	0
Klebsiella_oxytoca_ERR2221335	0	0	0	0	1000	0	1000	0	0	0
Klebsiella_oxytoca_ERR2221348	0	0	0	0	1000	0	1000	0	0	0
Klebsiella_oxytoca_ERR2221399	0	0	0	0	1000	0	1000	0	0	0
Klebsiella_oxytoca_ERR2221400	0	0	0	0	1000	0	1000	0	0	0
Klebsiella_oxytoca_ICU1_2b	0	0	0	0	1000	0	1000	0	0	0
Klebsiella_oxytoca_JKo3	0	0	0	0	1000	0	1000	0	0	0
Klebsiella_oxytoca_KCTC_1686	1000	0	0	0	0	0	1000	0	0	1000
Klebsiella_oxytoca_MGH88	0	0	0	0	1000	0	1000	0	0	0
Klebsiella_pneumoniae_1162281	0	1000	1000	0	1000	0	1000	0	0	0
Klebsiella_pneumoniae_1191100241	0	1000	1000	0	1000	0	1000	0	0	0
Klebsiella_pneumoniae_342	0	0	1000	0	1000	0	1000	0	0	0
Klebsiella_pneumoniae_361_1301	0	1000	1000	0	1000	0	1000	0	0	0
Klebsiella_pneumoniae_500_1420	0	1000	1000	0	1000	0	1000	0	0	0
Klebsiella_pneumoniae_540_1460	0	1000	1000	0	1000	0	1000	0	0	0
Klebsiella_pneumoniae_646_1568	0	1000	1000	0	1000	0	1000	0	0	0
Klebsiella_pneumoniae_ATCC_25955	0	1000	1000	0	1000	0	1000	0	0	0
Klebsiella_pneumoniae_ATCC_BAA_1705	0	1000	1000	0	1000	0	1000	0	0	0
Klebsiella_pneumoniae_ATCC_BAA_2146	0	1000	1000	0	1000	0	1000	0	0	0
Klebsiella_pneumoniae_CHS153	0	1000	1000	0	1000	0	1000	0	0	0
Klebsiella_pneumoniae_CHS222	0	1000	1000	0	1000	0	1000	0	0	0
Klebsiella_pneumoniae_DMC0526	0	1000	1000	0	1000	0	1000	0	0	0
Klebsiella_pneumoniae_ERR2221115	0	1000	1000	0	1000	0	1000	0	0	0
Klebsiella_pneumoniae_ERR2221116	0	1000	1000	0	1000	0	1000	0	0	0
Klebsiella_pneumoniae_ERR2221118	0	1000	1000	0	1000	0	1000	0	0	0
Klebsiella_pneumoniae_ERR2221121	0	1000	1000	0	1000	0	1000	0	0	0
Klebsiella_pneumoniae_ERR2221297	0	1000	1000	0	1000	0	1000	0	0	0
Klebsiella_pneumoniae_ERR2221368	0	1000	1000	0	1000	0	1000	0	0	0
Klebsiella_pneumoniae_ERR2221372	0	1000	1000	0	1000	0	1000	0	0	0
Klebsiella_pneumoniae_G5_2	0	1000	1000	0	1000	0	1000	0	0	0
Klebsiella_pneumoniae_hvKP1	0	1000	1000	0	1000	0	1000	0	0	0
Klebsiella_pneumoniae_JH1	0	1000	1000	0	1000	0	1000	0	0	0
Klebsiella_pneumoniae_KCTC_2242	1000	1000	1000	0	1000	0	1000	0	0	0
Klebsiella_pneumoniae_KP_11	0	1000	1000	0	1000	0	1000	0	0	0
Klebsiella_pneumoniae_NTUH_K2044	1000	1000	1000	0	1000	0	1000	0	0	0
Klebsiella_pneumoniae_pneumoniae_MGH78578	1000	1000	0	0	1000	0	1000	0	0	0
Klebsiella_pneumoniae_PR04	0	1000	1000	0	1000	0	1000	0	0	0
Klebsiella_pneumoniae_subsp_pneumoniae_1084	0	1000	1000	0	1000	0	1000	0	0	0
Klebsiella_pneumoniae_subsp_pneumoniae_12_3	0	1000	1000	0	1000	0	1000	0	0	0
Klebsiella_pneumoniae_subsp_pneumoniae_DSM	0	1000	1000	0	1000	0	1000	0	0	0
Klebsiella_pneumoniae_subsp_pneumoniae_Ecl8	0	1000	1000	0	1000	0	1000	0	0	0
Klebsiella_pneumoniae_subsp_pneumoniae_HS11	0	1000	1000	0	1000	0	1000	0	0	0
Klebsiella_pneumoniae_subsp_pneumoniae_KPNI	0	1000	1000	0	1000	0	1000	0	0	0
Klebsiella_pneumoniae_subsp_pneumoniae_KPNI	0	1000	1000	0	1000	0	1000	0	0	0
Klebsiella_pneumoniae_subsp_pneumoniae_KPNI	0	1000	1000	0	1000	0	1000	0	0	0
Klebsiella_pneumoniae_subsp_pneumoniae_KPNI	0	1000	1000	0	1000	0	1000	0	0	0
Klebsiella_pneumoniae_subsp_pneumoniae_KPNI	0	1000	1000	0	1000	0	1000	0	0	0
Klebsiella_pneumoniae_subsp_pneumoniae_KPNI	0	1000	1000	0	1000	0	1000	0	0	0
Klebsiella_pneumoniae_subsp_pneumoniae_KPNI	0	1000	1000	0	1000	0	1000	0	0	0
Klebsiella_pneumoniae_subsp_pneumoniae_KPNI	0	1000	1000	0	1000	0	1000	0	0	0
Klebsiella_pneumoniae_subsp_pneumoniae_KPNI	0	1000	1000	0	1000	0	1000	0	0	0
Klebsiella_pneumoniae_subsp_pneumoniae_KPNI	0	1000	1000	0	1000	0	1000	0	0	0
Klebsiella_pneumoniae_subsp_pneumoniae_KPNI	0	1000	1000	0	1000	0	1000	0	0	0
Klebsiella_pneumoniae_subsp_pneumoniae_KPNI	0	1000	1000	0	1000	0	1000	0	0	0
Klebsiella_pneumoniae_subsp_pneumoniae_KPNI	0	1000	1000	0	1000	0	1000	0	0	0
Klebsiella_pneumoniae_subsp_pneumoniae_KPNI	0	1000	1000	0	1000	0	1000	0	0	0
Klebsiella_pneumoniae_subsp_pneumoniae_KPNI	0	1000	1000	0	1000	0	1000	0	0	0

Klebsiella_pneumoniae_subsp_pneumoniae_KPNI	0	1000	1000	0	1000	0	1000	0	0	0
Klebsiella_pneumoniae_subsp_pneumoniae_KPNI	0	1000	1000	0	1000	0	1000	0	0	0
Klebsiella_pneumoniae_subsp_pneumoniae_KPNI	0	1000	1000	0	1000	0	1000	0	0	0
Klebsiella_pneumoniae_subsp_pneumoniae_KPNI	0	1000	1000	0	1000	0	1000	0	0	0
Klebsiella_pneumoniae_subsp_pneumoniae_KPNI	0	1000	1000	0	1000	0	1000	0	0	0
Klebsiella_pneumoniae_subsp_pneumoniae_KpQ3	0	1000	1000	0	1000	0	1000	0	0	0
Klebsiella_pneumoniae_subsp_pneumoniae_LZ	0	1000	1000	0	1000	0	1000	0	0	0
Klebsiella_pneumoniae_subsp_pneumoniae_ST25	0	1000	1000	0	1000	0	1000	0	0	0
Klebsiella_pneumoniae_subsp_pneumoniae_ST25	0	1000	1000	0	1000	0	1000	0	0	0
Klebsiella_pneumoniae_subsp_pneumoniae_ST25	0	1000	1000	0	1000	0	1000	0	0	0
Klebsiella_pneumoniae_subsp_pneumoniae_ST51	0	1000	1000	0	1000	0	1000	0	0	0
Klebsiella_pneumoniae_subsp_pneumoniae_WGL	0	1000	1000	0	1000	0	1000	0	0	0
Klebsiella_pneumoniae_subsp_pneumoniae_WGL	0	1000	1000	0	1000	0	1000	0	0	0
Klebsiella_pneumoniae_subsp_pneumoniae_WGL	0	1000	1000	0	1000	0	1000	0	0	0
Klebsiella_pneumoniae_subsp_rhinoscleromatis_A	0	1000	1000	0	1000	0	1000	0	0	0
Klebsiella_pneumoniae_UHKPC_52	0	1000	1000	0	1000	0	1000	0	0	0
Klebsiella_pneumoniae_UHKPC01	0	1000	1000	0	1000	0	1000	0	0	0
Klebsiella_pneumoniae_UHKPC04	0	1000	1000	0	1000	0	1000	0	0	0
Klebsiella_pneumoniae_UHKPC05	0	1000	1000	0	1000	0	1000	0	0	0
Klebsiella_pneumoniae_UHKPC09	0	1000	1000	0	1000	0	1000	0	0	0
Klebsiella_pneumoniae_UHKPC22	0	1000	1000	0	1000	0	1000	0	0	0
Klebsiella_pneumoniae_UHKPC23	0	1000	1000	0	1000	0	1000	0	0	0
Klebsiella_pneumoniae_UHKPC24	0	1000	1000	0	1000	0	1000	0	0	0
Klebsiella_pneumoniae_UHKPC26	0	1000	1000	0	1000	0	1000	0	0	0
Klebsiella_pneumoniae_UHKPC27	0	1000	1000	0	1000	0	1000	0	0	0
Klebsiella_pneumoniae_UHKPC29	0	1000	1000	0	1000	0	1000	0	0	0
Klebsiella_pneumoniae_UHKPC32	0	1000	1000	0	1000	0	1000	0	0	0
Klebsiella_pneumoniae_UHKPC40	0	1000	1000	0	1000	0	1000	0	0	0
Klebsiella_pneumoniae_UHKPC45	0	1000	1000	0	1000	0	1000	0	0	0
Klebsiella_pneumoniae_UHKPC48	0	1000	1000	0	1000	0	1000	0	0	0
Klebsiella_pneumoniae_UHKPC57	0	1000	1000	0	1000	0	1000	0	0	0
Klebsiella_pneumoniae_UHKPC81	0	1000	1000	0	1000	0	1000	0	0	0
Klebsiella_pneumoniae_VAKPC252	0	1000	1000	0	1000	0	1000	0	0	0
Klebsiella_pneumoniae_VAKPC254	0	1000	1000	0	1000	0	1000	0	0	0
Klebsiella_pneumoniae_VAKPC269	0	1000	1000	0	1000	0	1000	0	0	0
Klebsiella_pneumoniae_VAKPC270	0	1000	1000	0	1000	0	1000	0	0	0
Klebsiella_pneumoniae_VAKPC276	0	1000	1000	0	1000	0	1000	0	0	0
Klebsiella_pneumoniae_VAKPC278	0	1000	1000	0	1000	0	1000	0	0	0
Klebsiella_pneumoniae_VAKPC280	0	1000	1000	0	1000	0	1000	0	0	0
Klebsiella_pneumoniae_VAKPC297	0	1000	1000	0	1000	0	1000	0	0	0
Klebsiella_pneumoniae_VAKPC309	0	1000	1000	0	1000	0	1000	0	0	0
Klebsiella_quasipneumoniae_700603	0	1000	1000	0	1000	0	1000	0	0	0
Klebsiella_sp_4_1_44FAA	0	0	1000	0	1000	0	1000	0	0	0
Klebsiella_sp_M5al	0	0	0	0	1000	0	1000	0	0	0
Klebsiella_sp_MS_92_3	0	0	1000	0	1000	0	1000	0	0	0
Klebsiella_sp_OBRC7	0	0	0	0	1000	0	1000	0	0	0
Klebsiella_variicola_1_1_55	0	0	0	0	1000	0	1000	0	0	0
Klebsiella_variicola_KV321_ZB100000	0	0	1000	0	1000	0	1000	0	0	0
Kluyvera_ascorbata_ATCC_33433	0	0	1000	0	1000	0	1000	0	0	1000
Kluyvera_cryocrescens_L2	0	0	1000	0	1000	0	1000	0	0	0
Kluyvera_cryocrescens_NBRC_102467	0	0	1000	0	1000	0	1000	0	0	0
Kluyvera_georgiana_ATCC_51603	0	0	1000	0	1000	0	1000	0	0	0
Kluyvera_intermedia_FOSA7093	0	0	1000	0	1000	0	1000	0	0	0
Kluyvera_intermedia_NBRC_102594_ATCC_33110	0	0	1000	0	1000	0	1000	0	0	0
Kocuria_palustris_CD07_3	0	0	0	0	1000	0	1000	0	0	0
Kocuria_palustris_PEL	0	0	0	0	1000	0	1000	0	0	0
Kocuria_rhizophila_DC2201	0	0	0	0	1000	0	0	0	0	0
Kocuria_rhizophila_P7_4	0	0	1000	0	0	0	0	0	0	0
Kosakonia_sacchari_SP1	0	0	1000	0	1000	0	1000	0	0	0
Kurthia_massiliensis_JC30	0	0	1000	0	1000	0	1000	0	0	0
Kurthia_senegalensis_JC8E	0	0	1000	0	1000	0	1000	0	0	0
Kytococcus_sedentarius_1083_KSED	0	0	0	0	1000	0	1000	0	0	0
Kytococcus_sedentarius_262_KSED	0	0	0	0	1000	0	1000	0	0	0
Kytococcus_sedentarius_DSM_20547	0	0	0	0	1000	0	1000	0	0	0
Lachnoanaerobaculum_orale_DSM_24553	0	0	0	0	1000	0	1000	0	0	0
Lachnoanaerobaculum_saburreum_DNF00896	0	0	0	0	1000	0	1000	0	0	0
Lachnoanaerobaculum_saburreum_DSM_3986	0	0	0	0	1000	0	1000	0	0	0
Lachnoanaerobaculum_saburreum_F0468	0	0	0	0	1000	0	1000	0	0	0
Lachnobacterium_bovis_AE2004	0	0	0	0	1000	0	1000	0	0	0
Lachnobacterium_bovis_C6A12	0	0	0	0	1000	0	1000	0	0	0
Lachnobacterium_bovis_DSM_14045	0	0	0	0	1000	0	1000	0	0	0
Lachnobacterium_bovis_NK4B19	0	0	0	0	1000	0	1000	0	0	0
Lachnobacterium_bovis_S1b	0	0	0	0	1000	0	1000	0	0	0
Lachnoclostridium_nov_ERR1022273	0	0	0	0	0	0	1000	0	0	0
Lachnoclostridium_nov_ERR1022276	0	0	0	0	1000	0	1000	0	0	0
Lachnoclostridium_nov_ERR1022305	0	0	0	0	0	0	1000	0	0	0
Lachnoclostridium_nov_ERR1022387	0	0	0	0	1000	0	1000	0	0	0
Lachnoclostridium_nov_ERR1022388	0	0	0	0	1000	0	1000	0	0	0
Lachnoclostridium_nov_ERR1022392	0	0	0	0	1000	0	1000	0	0	0
Lachnoclostridium_nov_ERR1022405	0	0	0	0	0	0	1000	0	0	0
Lachnoclostridium_nov_ERR1022430	0	0	0	0	1000	0	1000	0	0	0
Lachnoclostridium_nov_ERR1022434	0	0	0	0	0	0	1000	0	0	0
Lachnoclostridium_nov_ERR1203939	0	0	0	0	1000	0	1000	0	0	0
Lachnoclostridium_nov_ERR1203968	0	0	0	0	0	0	1000	0	0	0
Lachnoclostridium_nov_ERR1204033	0	0	0	0	1000	0	1000	0	0	0
Lachnoclostridium_nov_ERR1204062	0	0	0	0	0	0	1000	0	0	0
Lachnoclostridium_nov_ERR171262	0	0	0	0	1000	0	1000	0	0	0
Lachnoclostridium_nov_ERR171273	0	0	0	0	1000	0	1000	0	0	0
Lachnoclostridium_nov_ERR171281	0	0	1000	0	1000	0	1000	0	0	0
Lachnoclostridium_nov_ERR2221145	0	0	0	0	1000	0	1000	0	0	0
Lachnoclostridium_nov_ERR2221158	0	0	0	0	1000	0	1000	0	0	0
Lachnoclostridium_nov_ERR2221159	0	0	0	0	1000	0	1000	0	0	0
Lachnoclostridium_nov_ERR2230065	0	0	0	0	1000	0	1000	0	0	0
Lachnoclostridium_nov_ERR2230076	0	0	0	0	1000	0	0	0	0	0

Lachnoclostridium_nov_ERR2230092	0	0	0	0	1000	0	1000	0	0	0
Lachnoclostridium_nov_ERR2230109	0	0	0	0	1000	0	1000	0	0	0
Lachnoclostridium_nov_ERR2230115	0	0	0	0	0	0	1000	0	0	0
Lachnoclostridium_nov_ERR2230132	0	0	0	0	0	0	1000	0	0	0
Lachnoclostridium_nov_ERR2230151	0	0	0	0	0	0	1000	0	0	0
Lachnoclostridium_sp_YL32	0	0	0	0	1000	0	1000	0	0	0
Lachnospira_multipara_ATCC_19207	0	0	1000	0	1000	0	0	0	0	0
Lachnospira_multipara_D15d	0	0	0	0	1000	0	1000	0	0	0
Lachnospira_multipara_LB2003	0	0	0	0	1000	0	1000	0	0	0
Lachnospira_multipara_MC2003	0	0	0	0	1000	0	1000	0	0	0
Lachnospira_nov_ERR1022391	0	0	0	0	1000	0	1000	0	0	0
Lachnospira_nov_ERR1022394	0	0	0	0	0	0	1000	0	0	0
Lachnospira_nov_ERR1203977	0	0	0	0	1000	0	1000	0	0	0
Lachnospira_nov_ERR1204071	0	0	0	0	1000	0	1000	0	0	0
Lachnospira_pectinoschiza_ERR1022352	0	0	0	0	1000	0	1000	0	0	0
Lachnospira_pectinoschiza_ERR1022401	0	0	0	0	1000	0	1000	0	0	0
Lachnospira_pectinoschiza_M83	0	0	0	0	1000	0	1000	0	0	0
Lachnospiraceae_bacterium_1_1_57FAA	0	0	0	0	0	0	1000	0	0	0
Lachnospiraceae_bacterium_1_4_56FAA	0	0	0	0	1000	0	1000	0	0	0
Lachnospiraceae_bacterium_2_1_46FAA	0	0	0	0	0	0	1000	0	0	0
Lachnospiraceae_bacterium_2_1_58FAA	0	0	0	1000	1000	0	1000	0	0	0
Lachnospiraceae_bacterium_3_1_46FAA	0	0	0	0	0	0	1000	0	0	0
Lachnospiraceae_bacterium_3_1_57FAA_CT1	0	0	0	1000	0	0	1000	0	0	0
Lachnospiraceae_bacterium_5_1_57FAA	0	0	0	0	1000	0	1000	0	0	0
Lachnospiraceae_bacterium_6_1_63FAA	0	0	0	0	1000	0	1000	0	0	0
Lachnospiraceae_bacterium_7_1_58FAA	0	0	0	0	1000	0	1000	0	0	0
Lachnospiraceae_bacterium_9_1_43BFAA	0	0	0	0	0	0	1000	0	0	0
Lachnospiraceae_bacterium_oral_taxon_082_str_	0	0	0	1000	1000	0	1000	0	0	0
Lachnospiraceae_bacterium_sp_5_1_63FAA	0	0	0	0	0	0	1000	0	0	0
Lachnospiraceae_bacterium_sp_8_1_57FAA	0	0	0	0	0	0	1000	0	0	0
Lachnospiraceae_nov_ERR1022272	0	0	0	0	0	0	1000	0	0	0
Lachnospiraceae_nov_ERR1022274	0	0	0	0	0	0	0	0	0	0
Lachnospiraceae_nov_ERR1022277	0	0	0	0	0	0	0	0	0	0
Lachnospiraceae_nov_ERR1022289	0	0	0	0	0	0	0	0	0	0
Lachnospiraceae_nov_ERR1022303	0	0	0	0	1000	0	1000	0	0	0
Lachnospiraceae_nov_ERR1022308	0	0	0	0	1000	0	1000	0	0	0
Lachnospiraceae_nov_ERR1022341	0	0	0	0	1000	0	1000	0	0	0
Lachnospiraceae_nov_ERR1022342	0	0	0	0	0	0	1000	0	0	0
Lachnospiraceae_nov_ERR1022343	0	0	0	0	0	0	0	0	0	0
Lachnospiraceae_nov_ERR1022351	0	0	0	0	1000	0	1000	0	0	0
Lachnospiraceae_nov_ERR1022353	0	0	0	0	1000	0	1000	0	0	0
Lachnospiraceae_nov_ERR1022380	0	0	1000	0	1000	0	1000	0	0	0
Lachnospiraceae_nov_ERR1022381	0	0	0	0	1000	0	1000	0	0	0
Lachnospiraceae_nov_ERR1022386	0	0	0	0	0	0	1000	0	0	0
Lachnospiraceae_nov_ERR1022389	0	0	0	0	1000	0	1000	0	0	0
Lachnospiraceae_nov_ERR1022390	0	0	0	0	0	0	0	0	0	0
Lachnospiraceae_nov_ERR1022398	0	0	0	0	1000	0	1000	0	0	0
Lachnospiraceae_nov_ERR1022402	0	0	1000	0	0	0	1000	0	0	0
Lachnospiraceae_nov_ERR1022429	0	0	1000	0	1000	0	1000	0	0	0
Lachnospiraceae_nov_ERR1022431	0	0	0	0	1000	0	1000	0	0	0
Lachnospiraceae_nov_ERR1022433	0	0	0	0	0	0	1000	0	0	0
Lachnospiraceae_nov_ERR1022436	0	0	0	0	0	0	1000	0	0	0
Lachnospiraceae_nov_ERR1022478	0	0	0	0	1000	0	0	0	0	0
Lachnospiraceae_nov_ERR1022480	0	0	0	0	1000	0	1000	0	0	0
Lachnospiraceae_nov_ERR1022481	0	0	0	0	0	0	1000	0	0	0
Lachnospiraceae_nov_ERR1203950	0	0	0	0	1000	0	1000	0	0	0
Lachnospiraceae_nov_ERR1203952	0	0	0	0	1000	0	1000	0	0	0
Lachnospiraceae_nov_ERR1203964	0	0	0	0	1000	0	1000	0	0	0
Lachnospiraceae_nov_ERR1203967	0	0	1000	0	1000	0	1000	0	0	0
Lachnospiraceae_nov_ERR1203970	0	0	0	0	1000	0	1000	0	0	0
Lachnospiraceae_nov_ERR1203972	0	0	0	0	0	0	1000	0	0	0
Lachnospiraceae_nov_ERR1204044	0	0	0	0	1000	0	1000	0	0	0
Lachnospiraceae_nov_ERR1204046	0	0	0	0	1000	0	1000	0	0	0
Lachnospiraceae_nov_ERR1204058	0	0	0	0	1000	0	1000	0	0	0
Lachnospiraceae_nov_ERR1204061	0	0	1000	0	1000	0	1000	0	0	0
Lachnospiraceae_nov_ERR1204064	0	0	0	0	1000	0	1000	0	0	0
Lachnospiraceae_nov_ERR1204066	0	0	0	0	0	0	1000	0	0	0
Lachnospiraceae_nov_ERR171267	0	0	0	0	1000	0	1000	0	0	0
Lachnospiraceae_nov_ERR171274	0	0	1000	0	1000	0	1000	0	0	0
Lachnospiraceae_nov_ERR171283	0	0	0	0	0	0	1000	0	0	0
Lachnospiraceae_nov_ERR2221097	0	0	0	0	1000	0	0	0	0	0
Lachnospiraceae_nov_ERR2221111	0	0	0	0	1000	0	1000	0	0	0
Lachnospiraceae_nov_ERR2221148	0	0	1000	0	1000	0	0	0	0	0
Lachnospiraceae_nov_ERR2221151	0	0	1000	0	1000	0	1000	0	0	0
Lachnospiraceae_nov_ERR2221161	0	0	0	0	0	0	1000	0	0	0
Lachnospiraceae_nov_ERR2221181	0	0	0	0	1000	0	0	0	0	0
Lachnospiraceae_nov_ERR2221183	0	0	0	0	1000	0	1000	0	0	0
Lachnospiraceae_nov_ERR2221187	0	0	0	0	1000	0	1000	0	0	0
Lachnospiraceae_nov_ERR2221212	0	0	0	0	1000	0	1000	0	0	0
Lachnospiraceae_nov_ERR2221263	0	0	0	0	0	0	1000	0	0	0
Lachnospiraceae_nov_ERR2221266	0	0	0	0	0	0	1000	0	0	0
Lachnospiraceae_nov_ERR2221278	0	0	0	0	0	0	1000	0	0	0
Lachnospiraceae_nov_ERR2221285	0	0	0	0	1000	0	1000	0	0	0
Lachnospiraceae_nov_ERR2221344	0	0	0	0	0	0	0	0	0	0
Lachnospiraceae_nov_ERR2221369	0	0	0	0	1000	0	0	0	0	0
Lachnospiraceae_nov_ERR2221389	0	0	0	0	1000	0	1000	0	0	0
Lachnospiraceae_nov_ERR2230050	0	0	0	0	1000	0	1000	0	0	0
Lachnospiraceae_nov_ERR2230056	0	0	0	0	1000	0	1000	0	0	0
Lachnospiraceae_nov_ERR2230057	0	0	0	0	0	0	1000	0	0	0
Lachnospiraceae_nov_ERR2230058	0	0	0	0	0	0	1000	0	0	0
Lachnospiraceae_nov_ERR2230083	0	0	0	0	1000	0	1000	0	0	0
Lachnospiraceae_nov_ERR2230113	0	0	0	0	1000	0	1000	0	0	0
Lachnospiraceae_nov_ERR2230114	0	0	0	0	0	0	1000	0	0	0
Lachnospiraceae_nov_ERR2230144	0	0	0	0	0	0	1000	0	0	0

Lachnospiraceae_nov_ERR2230148	0	0	1000	0	1000	0	1000	0	0	0
Lachnospiraceae_oral_taxon_107_str_F0167	0	0	0	0	1000	0	1000	0	0	0
Lactobacillus_acidipiscis_ACA_DC_1533	0	0	0	0	1000	0	1000	0	0	0
Lactobacillus_acidipiscis_DSM_15836	0	0	0	0	1000	0	1000	0	0	0
Lactobacillus_acidipiscis_KCTC_13900	0	0	0	0	1000	0	1000	0	0	0
Lactobacillus_acidophilus_ATCC_4796	0	0	0	0	0	0	1000	0	0	0
Lactobacillus_acidophilus_La_14	0	0	0	0	1000	0	1000	0	0	0
Lactobacillus_acidophilus_NCFM	0	0	0	0	1000	0	1000	0	0	0
Lactobacillus_agilis_DSM_20509	0	0	0	0	1000	0	1000	0	0	0
Lactobacillus_agilis_UMNLA4	0	0	0	0	1000	0	1000	0	0	0
Lactobacillus_algidus_CMTALT10	0	0	0	0	1000	0	1000	0	0	0
Lactobacillus_algidus_DSM_15638	0	0	0	0	1000	0	1000	0	0	0
Lactobacillus_amylolyticus_DSM_11664	0	0	0	0	0	0	1000	0	0	0
Lactobacillus_amylovorus_30SC	0	0	0	0	0	0	1000	0	0	0
Lactobacillus_amylovorus_GRL_1112	0	0	0	0	1000	0	1000	0	0	1000
Lactobacillus_amylovorus_GRL1118	0	0	0	0	0	0	1000	0	0	0
Lactobacillus_animalis_ERR2221125	0	0	0	0	1000	0	1000	0	0	0
Lactobacillus_animalis_ERR2221280	0	0	0	0	1000	0	1000	0	0	0
Lactobacillus_animalis_ERR2221327	0	0	0	0	1000	0	1000	0	0	0
Lactobacillus_animalis_KCTC_3501	0	0	0	0	1000	0	1000	0	0	0
Lactobacillus_animalis_NP51	0	0	0	0	1000	0	1000	0	0	0
Lactobacillus_antri_DSM_16041	0	0	1000	0	0	0	0	0	0	0
Lactobacillus_apis_LMG_26964	0	0	0	0	0	0	1000	0	0	0
Lactobacillus_apodemi_DSM_16634_JCM_16172	0	0	0	0	1000	0	1000	0	0	0
Lactobacillus_brevis_ATCC_367	0	0	0	1000	1000	0	1000	0	0	0
Lactobacillus_brevis_KB290	0	0	0	1000	0	0	1000	0	0	0
Lactobacillus_brevis_subsp_gravesensis_ATCC_27	0	0	1000	1000	1000	0	1000	0	0	0
Lactobacillus_buchneri_ATCC_11577	0	0	1000	0	1000	0	1000	0	0	0
Lactobacillus_buchneri_CD034	0	0	1000	1000	1000	0	1000	0	0	0
Lactobacillus_buchneri_NRRL_B_30929	0	0	1000	1000	1000	0	1000	0	0	0
Lactobacillus_camelliae_DSM_22697_JCM_13995	0	0	0	0	0	0	0	0	0	0
Lactobacillus_casei_12A	0	0	0	0	1000	0	1000	0	0	0
Lactobacillus_casei_21_1	0	0	0	0	1000	0	1000	0	0	0
Lactobacillus_casei_A2_362	0	0	0	0	0	0	1000	0	0	0
Lactobacillus_casei_ATCC_334	0	0	0	0	1000	0	1000	0	0	0
Lactobacillus_casei_casei_BL23	0	0	0	0	1000	0	1000	0	0	0
Lactobacillus_casei_ERR2221237	0	0	0	0	0	0	1000	0	0	0
Lactobacillus_casei_ERR2221291	0	0	0	0	0	0	1000	0	0	0
Lactobacillus_casei_ERR2221303	0	0	0	0	0	0	1000	0	0	0
Lactobacillus_casei_ERR2221304	0	0	0	0	0	0	1000	0	0	0
Lactobacillus_casei_ERR2221325	0	0	0	0	0	0	1000	0	0	0
Lactobacillus_casei_UW4	0	0	0	0	0	0	1000	0	0	0
Lactobacillus_casei_W56	0	0	0	0	1000	0	1000	0	0	0
Lactobacillus_coleohominis_101_4_CHN	0	0	0	1000	1000	0	1000	0	0	0
Lactobacillus_coryniformis_subsp_coryniformis_C	0	0	0	0	1000	0	1000	0	0	0
Lactobacillus_coryniformis_subsp_coryniformis_K	0	0	0	0	1000	0	1000	0	0	0
Lactobacillus_crispatus_125_2_CHN	0	0	0	0	0	0	1000	0	0	0
Lactobacillus_crispatus_214_1	0	0	0	0	0	0	1000	0	0	0
Lactobacillus_crispatus_CTV_05	0	0	0	0	0	0	1000	0	0	0
Lactobacillus_crispatus_FB049_03	0	0	0	0	0	0	1000	0	0	0
Lactobacillus_crispatus_FB077_07	0	0	0	0	0	0	1000	0	0	0
Lactobacillus_crispatus_JV_V01	0	0	0	0	0	0	1000	0	0	0
Lactobacillus_crispatus_MV_1A_US	0	0	0	0	0	0	1000	0	0	0
Lactobacillus_crispatus_MV_3A_US	0	0	0	0	0	0	1000	0	0	0
Lactobacillus_crispatus_ST1	0	0	0	0	0	0	1000	0	0	0
Lactobacillus_curvatus_CRL_705	0	0	0	0	1000	0	1000	0	0	0
Lactobacillus_curvatus_ERR2221163	0	0	0	0	1000	0	1000	0	0	0
Lactobacillus_curvatus_JCM_1096_DSM_20019	0	0	0	0	1000	0	1000	0	0	0
Lactobacillus_delbrueckii_subsp_bulgaricus_2038	0	0	0	0	1000	0	1000	0	0	0
Lactobacillus_delbrueckii_subsp_bulgaricus_ATCC	0	0	0	0	1000	0	1000	0	0	0
Lactobacillus_delbrueckii_subsp_bulgaricus_ATCC	0	0	0	0	1000	0	1000	0	0	0
Lactobacillus_delbrueckii_subsp_bulgaricus_CNCN	0	0	0	0	1000	0	1000	0	0	0
Lactobacillus_delbrueckii_subsp_bulgaricus_CNCN	0	0	0	0	1000	0	1000	0	0	0
Lactobacillus_delbrueckii_subsp_bulgaricus_ND02	0	0	0	0	1000	0	1000	0	0	0
Lactobacillus_delbrueckii_subsp_delbrueckii_DSM	0	0	0	0	1000	0	1000	0	0	0
Lactobacillus_delbrueckii_ZN7a_9	0	0	0	0	1000	0	1000	0	0	0
Lactobacillus_dextrinicus_DSM_20335	0	0	0	0	0	0	1000	0	0	0
Lactobacillus_equicursoris_66c	0	0	0	0	0	0	1000	0	0	0
Lactobacillus_equicursoris_DSM_19284	0	0	0	0	0	0	1000	0	0	0
Lactobacillus_farciminis_KCTC_3681_DSM_20184	0	0	0	0	1000	0	1000	0	0	0
Lactobacillus_fermentum_28_3_CHN	0	0	1000	0	1000	0	0	0	0	0
Lactobacillus_fermentum_ATCC_14931	0	0	1000	0	0	0	0	0	0	0
Lactobacillus_fermentum_CECT_5716	0	0	1000	0	1000	0	0	0	0	0
Lactobacillus_fermentum_F_6	0	0	1000	0	500	0	1000	0	0	0
Lactobacillus_fermentum_IFO_3956	0	0	1000	0	1000	0	0	0	0	0
Lactobacillus_florum_2F	0	0	0	0	0	0	1000	0	0	0
Lactobacillus_fructivorans_KCTC_3543_DSM_202	0	0	0	0	1000	0	1000	0	0	0
Lactobacillus_frumenti_DSM_13145	0	0	0	0	1000	0	0	0	0	0
Lactobacillus_gasseri_202_4	0	0	0	0	1000	0	1000	0	0	0
Lactobacillus_gasseri_224_1	0	0	0	0	1000	0	1000	0	0	0
Lactobacillus_gasseri_ATCC_33323	0	0	0	0	0	0	1000	0	0	0
Lactobacillus_gasseri_CECT_5714	0	0	0	0	1000	0	1000	0	0	0
Lactobacillus_gasseri_ERR1203930	0	0	0	0	1000	0	1000	0	0	0
Lactobacillus_gasseri_ERR1204024	0	0	0	0	1000	0	1000	0	0	0
Lactobacillus_gasseri_ERR2221164	0	0	0	0	1000	0	1000	0	0	0
Lactobacillus_gasseri_ERR2221406	0	0	0	0	1000	0	1000	0	0	0
Lactobacillus_gasseri_JV_V03	0	0	0	1000	1000	0	1000	0	0	0
Lactobacillus_gasseri_K7	0	0	0	1000	1000	0	1000	0	0	0
Lactobacillus_gasseri_MV_22	0	0	0	0	1000	0	1000	0	0	0
Lactobacillus_gasseri_SJ_9E_US	0	0	0	0	1000	0	1000	0	0	0
Lactobacillus_gasseri_SV_16A_US	0	0	0	0	1000	0	1000	0	0	0
Lactobacillus_gastricus_PS3	0	0	1000	0	0	0	0	0	0	0
Lactobacillus_helveticus_DPC_4571	0	0	1000	0	1000	0	1000	0	0	0
Lactobacillus_helveticus_DSM_20075	0	0	1000	0	0	0	1000	0	0	0

Lactobacillus_helveticus_H10	0	0	0	0	0	0	1000	0	0	0
Lactobacillus_helveticus_R0052	0	0	0	0	0	0	1000	0	0	0
Lactobacillus_hilgardii_ATCC_8290	0	0	1000	0	1000	0	1000	0	0	0
Lactobacillus_iners_AB_1	0	0	0	0	0	0	1000	0	0	0
Lactobacillus_iners_ATCC_55195	0	0	0	0	0	0	1000	0	0	0
Lactobacillus_iners_DSM_13335	0	0	0	0	0	0	1000	0	0	0
Lactobacillus_iners_LactinV_03V1_b	0	0	0	0	0	0	1000	0	0	0
Lactobacillus_iners_LactinV_09V1_c	0	0	0	0	0	0	1000	0	0	0
Lactobacillus_iners_LactinV_11V1_d	0	0	0	0	0	0	1000	0	0	0
Lactobacillus_iners_LEAF_2052A_d	0	0	0	0	0	0	1000	0	0	0
Lactobacillus_iners_LEAF_2053A_b	0	0	0	0	0	0	1000	0	0	0
Lactobacillus_iners_LEAF_2062A_h1	0	0	0	0	0	0	1000	0	0	0
Lactobacillus_iners_LEAF_3008A_a	0	0	0	0	0	0	1000	0	0	0
Lactobacillus_iners_SPIN_1401G	0	0	0	0	0	0	1000	0	0	0
Lactobacillus_iners_SPIN_2503V10_D	0	0	0	0	0	0	1000	0	0	0
Lactobacillus_iners_UPII_143_D	0	0	0	0	0	0	1000	0	0	0
Lactobacillus_iners_UPII_60_B	0	0	0	0	0	0	1000	0	0	0
Lactobacillus_ingluviei_str_Autruche_4	0	0	1000	0	1000	0	1000	0	0	0
Lactobacillus_intestinalis_ERR2221123	0	0	0	0	0	0	1000	0	0	0
Lactobacillus_jensenii_115_3_CHN	0	0	0	0	1000	0	1000	0	0	0
Lactobacillus_jensenii_1153	0	0	0	0	1000	0	1000	0	0	0
Lactobacillus_jensenii_269_3	0	0	0	1000	1000	0	1000	0	0	0
Lactobacillus_jensenii_27_2_CHN	0	0	0	0	1000	0	1000	0	0	0
Lactobacillus_jensenii_JV_V16	0	0	0	0	1000	0	1000	0	0	0
Lactobacillus_jensenii_SJ_7A_US	0	0	0	0	1000	0	1000	0	0	0
Lactobacillus_johnsonii_ATCC_33200	0	0	0	0	1000	0	1000	0	0	0
Lactobacillus_johnsonii_DPC_6026	0	0	0	0	0	0	1000	0	0	0
Lactobacillus_johnsonii_ERR2221124	0	0	0	0	1000	0	1000	0	0	0
Lactobacillus_johnsonii_ERR2221205	0	0	0	0	1000	0	1000	0	0	0
Lactobacillus_johnsonii_FI9785	0	0	0	0	1000	0	1000	0	0	0
Lactobacillus_johnsonii_NCC_533	0	0	0	0	0	0	1000	0	0	0
Lactobacillus_johnsonii_pf01	0	0	0	0	1000	0	1000	0	0	0
Lactobacillus_kalixensis_DSM_16043	0	0	0	0	0	0	1000	0	0	0
Lactobacillus_kefiranofaciens_ZW3	0	0	0	0	0	0	1000	0	0	0
Lactobacillus_kunkeei_DSM_12361_ATCC_700308	0	0	0	0	1000	0	1000	1000	0	0
Lactobacillus_kunkeei_EFB6	0	0	0	0	1000	0	1000	1000	0	0
Lactobacillus_kunkeei_MP2	0	0	0	0	1000	0	1000	1000	0	0
Lactobacillus_lindneri_DSM_20690_JCM_11027	0	0	0	0	1000	0	1000	0	0	0
Lactobacillus_lindneri_TMW_1_2007	0	0	0	0	1000	0	1000	0	0	0
Lactobacillus_mali_KCTC_3596_DSM_20444	0	0	1000	0	0	0	0	0	0	0
Lactobacillus_manihotivorans_DSM_13343_JCM_	0	0	0	0	0	0	1000	0	0	0
Lactobacillus_mindensis_DSM_14500	0	0	0	0	1000	0	1000	0	0	0
Lactobacillus_mucosae_LM1	0	0	0	0	0	0	0	0	0	0
Lactobacillus_murinus_ASF361	0	0	0	0	1000	0	1000	0	0	0
Lactobacillus_murinus_ERR2221126	0	0	0	0	1000	0	1000	0	0	0
Lactobacillus_nov_ERR2221311	0	0	0	0	1000	0	1000	0	0	0
Lactobacillus_nov_ERR2221384	0	0	1000	0	0	0	1000	0	0	0
Lactobacillus_oligofermentans_DSM_15707_LMG	0	0	0	0	1000	0	1000	0	0	0
Lactobacillus_oris_F0423	0	0	1000	0	0	0	0	0	0	0
Lactobacillus_oris_PB013_T2_3	0	0	1000	0	1000	0	0	0	0	0
Lactobacillus_panis_DSM_6035	0	0	1000	0	0	0	0	0	0	0
Lactobacillus_parabrevis_ATCC_53295	0	0	0	1000	1000	0	1000	0	0	0
Lactobacillus_parabuchneri_DSM_5707_NBRC_10	0	0	1000	0	1000	0	1000	0	0	0
Lactobacillus_parabuchneri_FAM21829	0	0	1000	0	1000	0	1000	0	0	0
Lactobacillus_paracasei_32G	0	0	0	1000	0	0	1000	0	0	0
Lactobacillus_paracasei_BD_II	0	0	0	0	0	0	1000	0	0	0
Lactobacillus_paracasei_CRF28	0	0	0	0	0	0	1000	0	0	0
Lactobacillus_paracasei_Lc_10	0	0	0	0	0	0	1000	0	0	0
Lactobacillus_paracasei_LC2W	0	0	0	0	0	0	1000	0	0	0
Lactobacillus_paracasei_LPC_37	0	0	0	0	0	0	1000	0	0	0
Lactobacillus_paracasei_M36	0	0	0	0	0	0	1000	0	0	0
Lactobacillus_paracasei_subsp_paracasei_8700_2	0	0	0	0	1000	0	1000	0	0	0
Lactobacillus_paracasei_subsp_paracasei_ATCC_2	0	0	0	0	1000	0	1000	0	0	0
Lactobacillus_paracasei_subsp_paracasei_Lpp120	0	0	0	0	0	0	1000	0	0	0
Lactobacillus_paracasei_subsp_paracasei_Lpp122	0	0	0	0	1000	0	1000	0	0	0
Lactobacillus_paracasei_subsp_paracasei_Lpp17	0	0	0	0	1000	0	1000	0	0	0
Lactobacillus_paracasei_subsp_paracasei_Lpp225	0	0	0	0	1000	0	1000	0	0	0
Lactobacillus_paracasei_subsp_paracasei_Lpp226	0	0	0	0	1000	0	1000	0	0	0
Lactobacillus_paracasei_subsp_paracasei_Lpp230	0	0	0	0	1000	0	1000	0	0	0
Lactobacillus_paracasei_subsp_paracasei_Lpp46	0	0	0	0	1000	0	1000	0	0	0
Lactobacillus_paracasei_subsp_paracasei_Lpp49	0	0	0	0	1000	0	1000	0	0	0
Lactobacillus_paracasei_subsp_paracasei_Lpp74	0	0	0	0	0	0	1000	0	0	0
Lactobacillus_paracasei_subsp_tolerans_Lpl14	0	0	0	0	1000	0	1000	0	0	0
Lactobacillus_paracasei_subsp_tolerans_Lpl7	0	0	0	0	1000	0	1000	0	0	0
Lactobacillus_paracasei_T71499	0	0	0	0	0	0	1000	0	0	0
Lactobacillus_paracasei_UCD174	0	0	0	0	0	0	1000	0	0	0
Lactobacillus_paracasei_UW1	0	0	0	0	0	0	1000	0	0	0
Lactobacillus_paracasei_Zhang	0	0	0	0	0	0	1000	0	0	0
Lactobacillus_paraplantarum_D2_1	0	0	1000	0	1000	0	1000	0	0	0
Lactobacillus_paraplantarum_DSM_10667	0	0	1000	0	1000	0	1000	0	0	0
Lactobacillus_pasteurii_DSM_23907_CRBIP_24_7	0	0	0	0	0	0	1000	0	0	0
Lactobacillus_pentosus_KCA1	0	0	1000	0	1000	0	1000	0	0	0
Lactobacillus_plantarum_16	0	0	1000	0	1000	0	1000	0	0	0
Lactobacillus_plantarum_ERR2221349	0	0	1000	0	1000	0	1000	0	0	0
Lactobacillus_plantarum_IPLA88	0	0	1000	0	1000	0	1000	0	0	0
Lactobacillus_plantarum_JDM1	0	0	1000	0	1000	0	1000	0	0	0
Lactobacillus_plantarum_subsp_plantarum_ATCC	0	0	1000	0	1000	0	1000	0	0	0
Lactobacillus_plantarum_subsp_plantarum_NC8	0	0	1000	0	1000	0	1000	0	0	0
Lactobacillus_plantarum_subsp_plantarum_P_8	0	0	1000	0	1000	0	1000	0	0	0
Lactobacillus_plantarum_subsp_plantarum_ST_III	0	0	1000	0	1000	0	1000	0	0	0
Lactobacillus_plantarum_UCMA_3037	0	0	1000	0	1000	0	1000	0	0	0
Lactobacillus_plantarum_WCFS1	0	0	1000	0	1000	0	1000	0	0	0
Lactobacillus_plantarum_ZJ316	0	0	1000	0	1000	0	1000	0	0	0
Lactobacillus_pobuzihii_E100301_KCTC_13174	0	0	0	0	0	0	1000	0	0	0

Lactobacillus_pontis_DSM_8475	0	0	0	0	1000	0	0	0	0	0
Lactobacillus_pontis_UMB0683_21837_8_53_1	0	0	0	0	1000	0	0	0	0	0
Lactobacillus_rapi_DSM_19907_JCM_15042	0	0	1000	0	1000	0	1000	0	0	0
Lactobacillus_reuteri_100_23	0	0	1000	0	1000	0	1000	0	0	0
Lactobacillus_reuteri_ATCC_53608	0	0	1000	0	500	0	0	0	0	0
Lactobacillus_reuteri_CF48_3A	0	0	1000	0	0	0	0	0	0	0
Lactobacillus_reuteri_DSM_20016	0	0	1000	0	1000	0	0	0	0	0
Lactobacillus_reuteri_ERR2221127	0	0	1000	0	1000	0	0	0	0	0
Lactobacillus_reuteri_ERR2221204	0	0	1000	0	1000	0	0	0	0	0
Lactobacillus_reuteri_F275_JCM_1112	0	0	0	0	0	0	0	0	0	0
Lactobacillus_reuteri_I49	0	0	1000	0	1000	0	0	0	0	0
Lactobacillus_reuteri_I5007	0	0	1000	0	500	0	0	0	0	0
Lactobacillus_reuteri_lpuph	0	0	1000	0	500	0	0	0	0	0
Lactobacillus_reuteri_mlc3	0	0	1000	0	1000	0	0	0	0	0
Lactobacillus_reuteri_MM2_3	0	0	1000	0	1000	0	0	0	0	0
Lactobacillus_reuteri_MM4_1A	0	0	1000	0	0	0	0	0	0	0
Lactobacillus_reuteri_SD2112_ATCC_55730	0	0	1000	0	0	0	0	0	0	0
Lactobacillus_rhamnosus_ATCC_21052	0	0	1000	1000	1000	0	1000	0	0	0
Lactobacillus_rhamnosus_ATCC_8530	0	0	1000	1000	1000	0	1000	0	0	0
Lactobacillus_rhamnosus_ERR1203919	0	0	1000	0	0	0	1000	0	0	0
Lactobacillus_rhamnosus_ERR2221226	0	0	1000	0	0	0	1000	0	0	0
Lactobacillus_rhamnosus_GG_ATCC_53103	0	0	1000	1000	1000	0	1000	0	0	0
Lactobacillus_rhamnosus_HN001	0	0	1000	1000	1000	0	1000	0	0	0
Lactobacillus_rhamnosus_Lc_705	0	0	1000	1000	1000	0	1000	0	0	0
Lactobacillus_rhamnosus_LMS2_1	0	0	1000	1000	1000	0	1000	0	0	0
Lactobacillus_rhamnosus_LRHMDP2	0	0	1000	1000	1000	0	1000	0	0	0
Lactobacillus_rhamnosus_LRHMDP3	0	0	1000	1000	1000	0	1000	0	0	0
Lactobacillus_rhamnosus_R0011	0	0	1000	1000	1000	0	1000	0	0	0
Lactobacillus_rodentium_DSM_24759	0	0	0	0	0	0	1000	0	0	0
Lactobacillus_ruminis_ATCC_25644	0	0	1000	0	0	0	1000	0	0	0
Lactobacillus_ruminis_ATCC_27782	0	0	1000	0	0	0	1000	0	0	0
Lactobacillus_ruminis_DSM_20403_NBRC_102161	0	0	1000	0	0	0	1000	0	0	0
Lactobacillus_ruminis_ERR2221165	0	0	1000	0	0	0	1000	0	0	0
Lactobacillus_ruminis_ERR2221355	0	0	1000	0	0	0	1000	0	0	0
Lactobacillus_ruminis_SPM0211	0	0	1000	0	0	0	1000	0	0	0
Lactobacillus_saerimneri_30a	0	0	0	0	0	0	1000	0	0	0
Lactobacillus_sakei_subsp_sakei_23K	0	0	0	0	1000	0	1000	0	0	0
Lactobacillus_sakei_subsp_sakei_DSM_20017_JCM	0	0	0	0	1000	0	1000	0	0	0
Lactobacillus_salivarius_CECT_5713	0	0	1000	0	1000	0	1000	0	0	0
Lactobacillus_salivarius_ERR2221215	0	0	1000	0	1000	0	1000	0	0	0
Lactobacillus_salivarius_ERR2221301	0	0	1000	0	1000	0	1000	0	0	0
Lactobacillus_salivarius_ERR2221323	0	0	1000	0	1000	0	1000	0	0	0
Lactobacillus_salivarius_GJ_24	0	0	1000	0	1000	0	1000	0	0	0
Lactobacillus_salivarius_HO66_ATCC_11741	0	0	1000	0	1000	0	1000	0	0	0
Lactobacillus_salivarius_NIAS840	0	0	1000	0	1000	0	1000	0	0	0
Lactobacillus_salivarius_salivarius_UCC118	0	0	1000	0	1000	0	1000	0	0	0
Lactobacillus_sanfranciscensis_DSM_20451	0	0	0	0	1000	0	1000	0	0	0
Lactobacillus_sanfranciscensis_TMW_1_1304	0	0	0	0	1000	0	1000	0	0	0
Lactobacillus_sanfranciscensis_TMW_1_2137	0	0	0	0	1000	0	1000	0	0	0
Lactobacillus_siliginis_DSM_22696	0	0	0	0	0	0	1000	0	0	0
Lactobacillus_siliginis_NBRC_101315	0	0	0	0	0	0	1000	0	0	0
Lactobacillus_suebicus_DSM_5007_KCTC_3549	0	0	1000	1000	1000	0	0	0	0	0
Lactobacillus_taiwanensis_DSM_21401	0	0	0	0	0	0	1000	0	0	0
Lactobacillus_ultunensis_DSM_16047	0	0	0	0	0	0	1000	1000	0	0
Lactobacillus_vaccinostercus_DSM_20634	0	0	1000	0	1000	0	1000	0	0	0
Lactobacillus_vaginalis_ATCC_49540	0	0	0	0	0	0	0	0	0	0
Lactobacillus_vaginalis_ERR2221166	0	0	0	0	1000	0	1000	0	0	0
Lactobacillus_versmoldensis_DSM_14857_KCTC_3	0	0	0	0	1000	0	1000	0	0	0
Lactobacillus_zeae_DSM_20178_KCTC_3804	0	0	1000	1000	0	0	1000	0	0	0
Lactococcus_garvieae_21881	0	0	0	0	0	0	1000	0	0	0
Lactococcus_garvieae_8831	0	0	0	0	0	0	1000	0	0	0
Lactococcus_garvieae_ATCC_49156	0	0	0	0	0	0	1000	0	0	0
Lactococcus_garvieae_DCC43	0	0	0	0	0	0	1000	0	0	0
Lactococcus_garvieae_I113	0	0	0	0	0	0	1000	0	0	0
Lactococcus_garvieae_IPLA_31405	0	0	0	0	0	0	1000	0	0	0
Lactococcus_garvieae_Lg2	0	0	0	0	0	0	1000	0	0	0
Lactococcus_garvieae_LG9	0	0	0	0	0	0	1000	0	0	0
Lactococcus_garvieae_Tac2	0	0	0	0	0	0	1000	0	0	0
Lactococcus_garvieae_TB25	0	0	0	0	0	0	1000	0	0	0
Lactococcus_garvieae_UNIUD074	0	0	0	0	0	0	1000	0	0	0
Lactococcus_lactis_subsp_cremoris_A76	0	0	0	0	0	0	1000	0	0	0
Lactococcus_lactis_subsp_cremoris_CNCM_I_163	0	0	0	0	0	0	1000	0	0	0
Lactococcus_lactis_subsp_cremoris_MG1363	0	0	0	0	0	0	1000	0	0	0
Lactococcus_lactis_subsp_cremoris_NZ9000	0	0	0	0	0	0	1000	0	0	0
Lactococcus_lactis_subsp_cremoris_SK11	0	0	0	0	0	0	1000	0	0	0
Lactococcus_lactis_subsp_cremoris_UC509_9	0	0	0	0	0	0	1000	0	0	0
Lactococcus_lactis_subsp_lactis_CV56	0	0	0	0	0	0	1000	0	0	0
Lactococcus_lactis_subsp_lactis_Il1403	0	0	0	0	0	0	1000	0	0	0
Lactococcus_lactis_subsp_lactis_IO_1	0	0	0	0	0	0	1000	0	0	0
Lactococcus_lactis_subsp_lactis_KF147	0	0	0	0	0	0	1000	0	0	0
Lactococcus_lactis_subsp_lactis_YF11	0	0	0	0	0	0	1000	0	0	0
Lactococcus_raffinolactis_4877	0	0	0	0	0	0	1000	0	0	0
Lactonifactor_longoviformis_DSM_17459	0	0	0	0	1000	0	1000	0	0	0
Laribacter_hongkongensis_HLHK9	0	0	0	0	1000	0	0	0	0	0
Lautropia_mirabilis_ATCC_51599	0	0	0	0	0	0	1000	0	0	0
Leclercia_adecarboxylata_ATCC_23216_NBRC_10	0	0	1000	0	1000	0	1000	0	0	0
Leclercia_adecarboxylata_KY2	0	0	1000	0	1000	0	1000	0	0	0
Leminorella_grimontii_ATCC_33999	0	0	0	0	1000	0	1000	0	0	1000
Leptotrichia_buccalis_C_1013_b	466.6666667	0	0	0	1000	0	0	0	0	1000
Leptotrichia_goodfellowii_DSM_19756	0	0	0	0	1000	0	1000	0	0	0
Leptotrichia_goodfellowii_F0264	0	0	0	0	1000	0	1000	0	0	0
Leptotrichia_hofstadii_DSM_21651	0	0	0	0	1000	0	0	0	0	0
Leptotrichia_hofstadii_F0254	0	0	0	0	1000	0	0	0	0	0
Leptotrichia_hongkongensis_JMUB5056	0	0	0	0	1000	0	0	0	0	0

Leptotrichia_shahii_DSM_19757	0	0	0	0	1000	0	0	0	0	0
Leptotrichia_wadei_DSM_19758	0	0	0	0	1000	0	0	0	0	0
Leptotrichia_wadei_F0279	0	0	0	0	1000	0	0	0	0	0
Leptotrichia_wadei_KA00185	0	0	0	0	1000	0	0	0	0	0
Leuconostoc_argentinum_KCTC_3773	0	0	1000	0	0	0	1000	0	0	0
Leuconostoc_carnosum_JB16	0	0	1000	0	0	0	1000	0	0	0
Leuconostoc_citreum_1300_LCIT_132_3537_3087	0	0	1000	0	0	0	0	0	0	0
Leuconostoc_citreum_KM20	0	0	1000	0	0	0	1000	0	0	0
Leuconostoc_citreum_LBAE_C10	0	0	1000	0	0	0	1000	0	0	0
Leuconostoc_citreum_LBAE_C11	0	0	1000	0	0	0	1000	0	0	0
Leuconostoc_citreum_LBAE_E16	0	0	1000	0	0	0	1000	0	0	0
Leuconostoc_gelidum_JB7	0	0	1000	0	0	0	0	0	0	0
Leuconostoc_gelidum_KCTC_3527	0	0	1000	0	0	0	0	0	0	0
Leuconostoc_gelidum_subsp_gasicomitatum_C12	0	0	1000	0	0	0	1000	0	0	0
Leuconostoc_lactis_KACC_91922	0	0	1000	0	0	0	1000	0	0	0
Leuconostoc_mesenteroides_ERR2221167	0	0	1000	0	0	0	1000	0	0	0
Leuconostoc_mesenteroides_subsp_cremoris_AT	0	0	1000	0	0	0	1000	0	0	0
Leuconostoc_mesenteroides_subsp_mesenteroid	0	0	1000	0	0	0	1000	0	0	0
Leuconostoc_mesenteroides_subsp_mesenteroid	0	0	1000	0	0	0	1000	0	0	0
Leuconostoc_pseudomesenteroides_4882	0	0	1000	0	0	0	1000	0	0	0
Leuconostoc_pseudomesenteroides_ERR2221296	0	0	1000	0	0	0	1000	0	0	0
Listeria_grayi_DSM_20601	0	0	1000	0	0	0	1000	0	0	0
Listeria_innocua_ATCC_33091	0	0	1000	0	0	0	1000	0	0	0
Listeria_innocua_Clip11262	0	0	1000	0	0	0	1000	0	0	0
Listeria_monocytogenes_07PF0776	0	0	1000	0	0	0	1000	0	0	0
Listeria_monocytogenes_08_5578	0	0	1000	0	0	0	1000	0	0	0
Listeria_monocytogenes_08_5923	0	0	1000	0	0	0	1000	0	0	0
Listeria_monocytogenes_10403S	0	0	1000	0	0	0	1000	0	0	0
Listeria_monocytogenes_4a_L99	0	0	1000	0	0	0	1000	0	0	0
Listeria_monocytogenes_4b_F2365	0	0	1000	0	0	0	1000	0	0	0
Listeria_monocytogenes_ATCC_19117	0	0	1000	0	0	0	1000	0	0	0
Listeria_monocytogenes_Clip80459	0	0	1000	0	0	0	1000	0	0	0
Listeria_monocytogenes_EGD_e	0	0	1000	0	0	0	1000	0	0	0
Listeria_monocytogenes_F6900	0	0	1000	0	0	0	1000	0	0	0
Listeria_monocytogenes_Finland_1988	0	0	1000	0	0	0	1000	0	0	0
Listeria_monocytogenes_FSL_J1_194	0	0	1000	0	0	0	1000	0	0	0
Listeria_monocytogenes_FSL_J2_071	0	0	1000	0	0	0	1000	0	0	0
Listeria_monocytogenes_FSL_N1_017	0	0	1000	0	0	0	1000	0	0	0
Listeria_monocytogenes_FSL_N3_165	0	0	1000	0	0	0	1000	0	0	0
Listeria_monocytogenes_FSL_R2_503	0	0	1000	0	0	0	1000	0	0	0
Listeria_monocytogenes_FSL_R2_561	0	0	1000	0	0	0	1000	0	0	0
Listeria_monocytogenes_HCC23	0	0	1000	0	0	0	1000	0	0	0
Listeria_monocytogenes_HPB2262	0	0	1000	0	0	0	1000	0	0	0
Listeria_monocytogenes_J0161_FSL_R2_499	0	0	1000	0	0	0	1000	0	0	0
Listeria_monocytogenes_J2818	0	0	1000	0	0	0	1000	0	0	0
Listeria_monocytogenes_JF5171	0	0	1000	0	0	0	1000	0	0	0
Listeria_monocytogenes_L312	0	0	1000	0	0	0	1000	0	0	0
Listeria_monocytogenes_L99	0	0	1000	0	0	0	1000	0	0	0
Listeria_monocytogenes_M7	0	0	1000	0	0	0	1000	0	0	0
Listeria_monocytogenes_N53_1	0	0	1000	0	0	0	1000	0	0	0
Listeria_monocytogenes_serotype_1_2a_str_NCC	0	0	1000	0	0	0	1000	0	0	0
Listeria_monocytogenes_serotype_1_2b_str_SLC	0	0	1000	0	0	0	1000	0	0	0
Listeria_monocytogenes_serotype_1_2c_str_SLC	0	0	1000	0	0	0	1000	0	0	0
Listeria_monocytogenes_serotype_4b_str_LL195	0	0	1000	0	0	0	1000	0	0	0
Listeria_monocytogenes_serotype_7_SLCC_2482	0	0	1000	0	0	0	1000	0	0	0
Listeria_monocytogenes_SLCC_2378	0	0	1000	0	0	0	1000	0	0	0
Listeria_monocytogenes_SLCC_2540	0	0	1000	0	0	0	1000	0	0	0
Listeria_monocytogenes_SLCC_7179	0	0	1000	0	0	0	1000	0	0	0
Listeria_monocytogenes_SLCC2376	0	0	1000	0	0	0	1000	0	0	0
Listeria_monocytogenes_SLCC2479	0	0	1000	0	0	0	1000	0	0	0
Listeria_monocytogenes_SLCC5850	0	0	1000	0	0	0	1000	0	0	0
Listeria_monocytogenes_str_1_2a_F6854	0	0	1000	0	0	0	1000	0	0	0
Listeria_monocytogenes_str_4b_H7858	0	0	1000	0	0	0	1000	0	0	0
Listeria_monocytogenes_str_Scott_A	0	0	1000	0	0	0	1000	0	0	0
Loktanella_vestfoldensis_SMR4r	0	0	1000	0	0	0	0	0	0	0
Longibaculum_muris_DSM_29487	0	0	0	0	0	0	1000	0	0	0
Longicatena_caecimuris_DSM_29481	0	0	0	0	0	0	1000	0	1000	0
Lysinibacillus_fusiformis_ZB2	0	0	1000	0	0	0	1000	0	1000	0
Lysinibacillus_fusiformis_ZC1	0	0	1000	0	1000	0	1000	0	1000	0
Lysinibacillus_sphaericus_2362	0	0	1000	0	1000	0	1000	0	1000	0
Lysinibacillus_sphaericus_C3_41	0	0	1000	0	1000	0	1000	0	1000	0
Lysinibacillus_sphaericus_OT4b_31	0	0	1000	0	1000	0	1000	0	1000	0
Lysinibacillus_sphaericus_OT4b25	0	0	1000	0	1000	0	1000	0	1000	0
Lysinibacillus_sphaericus_OT4b49	0	0	1000	0	1000	0	1000	0	1000	0
Marinospirillum_alkaliphilum_DSM_21637	0	0	0	0	0	0	0	0	0	0
Marvinbryantia_formatexigens_I_52_DSM_14469	0	0	0	1000	0	0	1000	0	0	0
Megamonas_funiformis_YIT_11815	0	0	0	0	1000	0	1000	0	0	0
Megamonas_hypermegale_ART12_1	0	0	0	0	1000	0	1000	0	0	0
Megamonas_rupellensis_DSM_19944	0	0	1000	0	1000	0	1000	0	0	0
Megasphaera_elsdenii_DSM_20460	0	0	0	0	1000	0	1000	0	1000	0
Megasphaera_massiliensis_NP3	0	0	0	0	1000	0	1000	0	0	0
Megasphaera_micronuciformis_F0359	0	0	1000	0	1000	0	1000	0	0	0
Megasphaera_nov_ERR1022370	0	0	1000	0	1000	0	1000	0	0	0
Megasphaera_paucivorans_DSM_16981	0	0	1000	0	0	0	1000	0	0	0
Meiothermus_silvanus_DSM_9946	153.8461538	0	1000	0	1000	0	1000	1000	1000	0
Melainabacterium_MEL_A1	0	0	1000	0	1000	0	1000	0	0	0
Merdibacter_massiliensis_Marseille_P3254	0	0	0	0	0	0	1000	0	0	0
Mesorhizobium_loti_MAFF303099	0	0	0	0	0	0	0	0	0	0
Mesorhizobium_loti_NZP2037	0	0	1000	0	0	0	0	0	0	0
Metakosakonia_massiliensis_JC163	0	0	1000	0	1000	0	1000	0	0	0
Methanobrevibacter_ruminantium_M1	0	0	0	0	1000	0	0	0	0	0
Methanobrevibacter_smithii_ATCC_35061	0	0	0	0	0	0	0	0	500	0
Methanomassiliicoccus_luminyensis_B10	0	0	1000	0	0	0	0	0	0	0
Methanosphaera_stadtmanae_DSM_3091	0	0	0	0	0	0	1000	0	0	0

Methylobacterium_mesophilicum_SR1_6_6	0	0	1000	0	1000	0	0	0	1000	1000
Methylobacterium_populi_BJ001	0	0	1000	0	0	0	0	0	1000	1000
Methylobacterium_radiotolerans_JCM_2831	0	0	1000	0	1000	0	0	0	1000	1000
Methyloversatilis_universalis_EHg5	0	0	1000	0	1000	0	0	0	0	0
Methyloversatilis_universalis_FAM5	0	0	1000	0	1000	0	0	0	0	1000
Methyloversatilis_universalis_Fam50001	0	0	1000	0	1000	0	0	0	0	1000
Microbacterium_gubbeenense_DSM_15944	0	0	0	0	0	0	0	0	0	0
Microbacterium_oleivorans_NBRC_103075	0	0	0	0	0	0	0	0	1000	1000
Microbacterium_paraoxydans_77MFTsu3_2	0	0	0	0	0	0	0	0	0	1000
Micrococcus_luteus_1058_MLUT	0	0	0	0	1000	0	1000	0	1000	0
Micrococcus_luteus_ERR2221282	0	0	0	0	1000	0	1000	0	1000	0
Micrococcus_luteus_NCTC_2665	0	0	0	0	1000	0	1000	0	1000	0
Micrococcus_luteus_NDB3Y10	0	0	0	0	1000	0	0	0	1000	0
Micrococcus_luteus_RIT304	0	0	0	0	1000	0	1000	0	1000	0
Micrococcus_luteus_RIT305	0	0	0	0	1000	0	1000	0	1000	0
Micrococcus_luteus_RIT324w	0	0	0	0	1000	0	1000	0	1000	0
Micrococcus_luteus_SK58	0	0	0	0	1000	0	0	0	1000	0
Micrococcus_luteus_trpE16	0	0	0	0	1000	0	1000	0	1000	0
Micromonospora_aurantiaca_ATCC_27029	0	0	0	0	0	0	0	0	0	1000
Microvirga_massiliensis_JC119	0	0	1000	0	0	0	0	0	0	0
Mitsuokella_jalaludinii_DSM_13811	0	0	1000	0	1000	0	1000	0	0	0
Mitsuokella_jalaludinii_ERR1022286	0	0	1000	0	1000	0	1000	0	0	0
Mitsuokella_multacida_DSM_20544	0	0	1000	0	1000	0	1000	0	0	0
Mobiluncus_curtisii_ATCC_43063	0	0	0	0	1000	0	1000	0	0	0
Mobiluncus_curtisii_ATCC_51333	0	0	0	0	1000	0	1000	0	0	0
Mobiluncus_curtisii_subsp_holmesii_ATCC_35242	0	0	0	0	1000	0	1000	0	0	0
Mobiluncus_mulieris_28_1	0	0	0	0	1000	0	1000	0	0	0
Mobiluncus_mulieris_ATCC_35239	0	0	0	0	1000	0	1000	0	0	0
Mobiluncus_mulieris_ATCC_35243	0	0	0	0	1000	0	1000	0	0	0
Mobiluncus_mulieris_FB024_16	0	0	0	0	1000	0	1000	0	0	0
Mogibacterium_sp_CM50	0	0	0	0	0	0	1000	0	0	0
Mogibacterium_timidum_ATCC_33093	0	0	0	0	0	0	1000	0	0	0
Moraxella_bovoculi_22581	0	0	0	0	1000	0	1000	0	0	0
Moraxella_bovoculi_237	0	0	0	0	1000	0	1000	0	0	0
Moraxella_canis_CCUG_8415A_8415T1	0	0	0	0	1000	0	1000	0	0	0
Moraxella_catarrhalis_101P30B1	0	0	0	0	1000	0	0	0	0	0
Moraxella_catarrhalis_103P14B1	0	0	0	0	1000	0	0	0	0	0
Moraxella_catarrhalis_12P80B1	0	0	0	0	1000	0	0	0	0	0
Moraxella_catarrhalis_25240	0	0	0	0	1000	0	1000	0	0	0
Moraxella_catarrhalis_46P47B1	0	0	0	0	1000	0	0	0	0	0
Moraxella_catarrhalis_7169	0	0	0	0	1000	0	0	0	0	0
Moraxella_catarrhalis_BC1	0	0	0	0	1000	0	0	0	0	0
Moraxella_catarrhalis_BC7	0	0	0	0	1000	0	0	0	0	0
Moraxella_catarrhalis_BC8	0	0	0	0	1000	0	0	0	0	0
Moraxella_catarrhalis_CO72	0	0	0	0	1000	0	0	0	0	0
Moraxella_catarrhalis_O35E	0	0	0	0	1000	0	0	0	0	0
Moraxella_catarrhalis_RH4	0	0	0	0	1000	0	0	0	0	0
Morganella_morganii_FDAARGOS_172	0	0	0	0	1000	0	1000	0	0	0
Morganella_morganii_FDAARGOS_63	0	0	0	0	1000	0	1000	0	0	0
Morganella_morganii_SC01	0	0	0	0	1000	0	1000	0	0	0
Morganella_morganii_subsp_morganii_KT	0	0	0	0	0	0	1000	0	0	1000
Mucispirillum_schaedleri_ASF457	0	0	0	0	1000	0	0	0	0	0
Muribaculum_intestinale_YL27	0	0	1000	0	0	0	0	0	0	0
Muricomes_intestini_DSM_29489	0	0	0	0	0	0	1000	0	0	0
Murimonas_intestini_DSM_26524	0	0	0	0	0	0	0	0	0	0
Mycobacterium_abscessus_3A_0119_R	0	0	1000	0	0	0	0	0	0	0
Mycobacterium_abscessus_3A_0122_R	0	0	1000	0	0	0	0	0	0	0
Mycobacterium_abscessus_3A_0122_S	0	0	1000	0	0	0	0	0	0	0
Mycobacterium_abscessus_3A_0731	0	0	1000	0	0	0	0	0	0	0
Mycobacterium_abscessus_3A_0810_R	0	0	1000	0	0	0	0	0	0	0
Mycobacterium_abscessus_3A_0930_R	0	0	1000	0	0	0	0	0	0	0
Mycobacterium_abscessus_3A_0930_S	0	0	1000	0	0	0	0	0	0	0
Mycobacterium_abscessus_47J26	0	0	1000	0	0	0	0	0	0	0
Mycobacterium_abscessus_4S_0116_R	0	0	1000	0	0	0	0	0	0	0
Mycobacterium_abscessus_4S_0116_S	0	0	1000	0	0	0	0	0	0	0
Mycobacterium_abscessus_4S_0206	0	0	1000	0	0	0	0	0	0	0
Mycobacterium_abscessus_4S_0303	0	0	1000	0	0	0	0	0	0	0
Mycobacterium_abscessus_4S_0726_RA	0	0	1000	0	0	0	0	0	0	0
Mycobacterium_abscessus_4S_0726_RB	0	0	1000	0	0	0	0	0	0	0
Mycobacterium_abscessus_5S_0304	0	0	1000	0	0	0	0	0	0	0
Mycobacterium_abscessus_5S_0421	0	0	1000	0	0	0	0	0	0	0
Mycobacterium_abscessus_5S_0422	0	0	1000	0	0	0	0	0	0	0
Mycobacterium_abscessus_5S_0708	0	0	1000	0	0	0	0	0	0	0
Mycobacterium_abscessus_5S_0817	0	0	1000	0	0	0	0	0	0	0
Mycobacterium_abscessus_5S_0921	0	0	1000	0	0	0	0	0	0	0
Mycobacterium_abscessus_5S_1212	0	0	1000	0	0	0	0	0	0	0
Mycobacterium_abscessus_5S_1215	0	0	1000	0	0	0	0	0	0	0
Mycobacterium_abscessus_6G_0125_R	0	0	1000	0	0	0	0	0	0	0
Mycobacterium_abscessus_6G_0212	0	0	1000	0	0	0	0	0	0	0
Mycobacterium_abscessus_9808	0	0	1000	0	0	0	0	0	0	0
Mycobacterium_abscessus_M115	0	0	1000	0	0	0	0	0	0	0
Mycobacterium_abscessus_M139	0	0	1000	0	0	0	0	0	0	0
Mycobacterium_abscessus_M152	0	0	1000	0	0	0	0	0	0	0
Mycobacterium_abscessus_M154	0	0	1000	0	0	0	0	0	0	0
Mycobacterium_abscessus_M156	0	0	1000	0	0	0	0	0	0	0
Mycobacterium_abscessus_M159	0	0	1000	0	0	0	0	0	0	0
Mycobacterium_abscessus_M172	0	0	1000	0	0	0	0	0	0	0
Mycobacterium_abscessus_M24	0	0	1000	0	0	0	0	0	0	0
Mycobacterium_abscessus_M93	0	0	1000	0	0	0	0	0	0	0
Mycobacterium_abscessus_M94	0	0	1000	0	0	0	0	0	0	0
Mycobacterium_abscessus_subsp_bolletii_50594	0	0	1000	0	0	0	0	0	0	0
Mycobacterium_abscessus_subsp_bolletii_BD	0	0	1000	0	0	0	0	0	0	0
Mycobacterium_avium_104	0	0	1000	0	0	0	0	0	0	0
Mycobacterium_avium_subsp_avium_ATCC_2529	0	0	0	0	0	0	0	0	0	1000

Mycobacterium_avium_subsp_paratuberculosis_J	0	0	1000	0	0	0	0	0	0	0
Mycobacterium_avium_subsp_paratuberculosis_J	0	0	0	0	0	0	0	0	0	0
Mycobacterium_avium_subsp_paratuberculosis_K	0	0	1000	0	0	0	0	0	0	0
Mycobacterium_avium_subsp_paratuberculosis_N	0	0	1000	0	0	0	0	0	0	0
Mycobacterium_avium_subsp_paratuberculosis_S	0	0	1000	0	0	0	0	0	0	0
Mycobacterium_avium_subsp_paratuberculosis_S	0	0	1000	0	0	0	0	0	0	0
Mycobacterium_fortuitum_subsp_fortuitum_DSM	0	0	1000	0	0	0	1000	0	0	1000
Mycobacterium_tuberculosis_H37Rv	0	0	1000	0	0	0	0	0	0	0
Mycobacterium_tuberculosis_variant_pinnipedii_	0	0	1000	0	0	0	0	0	0	0
Mycoplasma_hominis_ATCC_23114	0	0	0	0	970.5882353	0	1000	0	0	0
Mycoplasma_hominis_ATCC_27545	0	0	0	0	767.8509886	0	767.0619009	0	0	0
Mycoplasma_hyopneumoniae_168	0	0	0	0	0	0	1000	0	0	0
Mycoplasma_hyopneumoniae_168_L	0	0	0	0	0	0	1000	0	0	0
Mycoplasma_hyopneumoniae_232	0	0	0	0	0	0	1000	0	0	0
Mycoplasma_hyopneumoniae_7448	0	0	0	0	0	0	1000	0	0	0
Mycoplasma_hyopneumoniae_J	0	0	0	0	0	0	1000	0	0	0
Mycoplasma_pneumoniae_19294	0	0	0	0	1000	0	1000	0	0	0
Mycoplasma_pneumoniae_309	0	0	0	0	0	0	1000	0	0	0
Mycoplasma_pneumoniae_FH	0	0	0	0	1000	0	1000	0	0	0
Mycoplasma_pneumoniae_M129	0	0	0	0	1000	0	1000	0	0	0
Mycoplasma_pneumoniae_M129_B7	0	0	0	0	1000	0	1000	0	0	0
Mycoplasma_pneumoniae_PI_1428	0	0	0	0	1000	0	1000	0	0	0
Mycoplasma_pneumoniae_PO1	0	0	0	0	1000	0	1000	0	0	0
Myroides_odoratimimus_CCUG_10230	0	0	1000	0	1000	0	1000	0	0	0
Myroides_odoratimimus_CCUG_12700	0	0	1000	0	1000	0	1000	0	0	0
Myroides_odoratimimus_CCUG_12901	0	0	1000	0	1000	0	1000	0	0	0
Myroides_odoratimimus_CCUG_3837	0	0	1000	0	1000	0	1000	0	0	0
Myroides_odoratimimus_CIP_101113	0	0	1000	0	1000	0	1000	0	0	0
Myroides_odoratimimus_PR63039	0	0	1000	0	1000	0	1000	0	0	0
Natranaerovirga_hydrolytica_DSM_24176	0	0	0	0	0	0	1000	0	0	0
Natranaerovirga_pectinivora_DSM_24629	0	0	0	0	0	0	0	0	0	0
Neisseria_bacilliformis_914_NLAC_0_29744_6743	0	0	0	0	1000	0	1000	0	0	0
Neisseria_bacilliformis_ATCC_BAA_1200	0	0	0	0	1000	0	1000	0	0	0
Neisseria_cinerea_ATCC_14685	0	0	0	0	0	0	1000	0	0	1000
Neisseria_elongata_subsp_glycolytica_ATCC_2931	0	0	0	0	0	0	0	0	0	0
Neisseria_flavescens_NRL30031_H210	0	0	0	0	0	0	1000	0	0	0
Neisseria_flavescens_SK114	0	0	0	0	0	0	1000	0	0	1000
Neisseria_lactamica_ATCC_23970	0	0	0	0	1000	0	0	0	0	0
Neisseria_macacae_ATCC_33926	0	0	0	0	0	0	1000	0	0	1000
Neisseria_meningitidis_2842STDY5881431	0	0	0	0	1000	0	0	0	0	0
Neisseria_meningitidis_ATCC_13091	0	0	0	0	1000	0	0	0	0	0
Neisseria_mucosa_ATCC_25996	0	0	0	0	0	0	1000	0	0	1000
Neisseria_mucosa_C102	0	0	0	0	0	0	1000	0	0	0
Neisseria_shayeganii_871	0	0	0	0	0	0	1000	0	0	0
Neisseria_sicca_4320	0	0	0	0	1000	0	1000	0	0	0
Neisseria_sicca_ATCC_29256	0	0	0	0	1000	0	1000	0	0	0
Neisseria_sicca_C2010005502	0	0	0	0	1000	0	1000	0	0	0
Neisseria_sicca_VK64	0	0	0	0	1000	0	1000	0	0	0
Neisseria_subflava_NJ9703	0	0	0	0	0	0	1000	0	0	1000
Nesterenkonia_massiliensis_NP1	0	0	1000	0	1000	0	1000	0	0	0
Nevskia_ramosa_DSM_11499	0	0	0	0	1000	0	0	0	1000	1000
Nigerium_massiliense_SIT5	0	0	1000	0	1000	0	0	0	0	0
Nocardioides_massiliensis_GD13	0	0	1000	0	1000	0	0	0	0	0
Nosocomiicoccus_massiliensis_NP2	0	0	0	0	1000	0	1000	0	0	0
Noviherbaspirillum_massiliense_JC206	0	0	0	0	0	0	0	0	0	0
Novosphingobium_aromaticivorans_DSM_12444	0	0	1000	0	0	0	0	0	0	0
Numidum_massiliense_mt3	0	0	0	0	1000	0	1000	0	0	0
Obesumbacterium_proteus_ATCC_12841	0	0	0	0	1000	0	1000	0	0	0
Obesumbacterium_proteus_DSM_2777	0	0	0	0	1000	0	1000	0	0	0
Oceanobacillus_caeni_HM6	0	0	1000	0	1000	0	1000	0	0	0
Oceanobacillus_jeddahense_S5	0	0	1000	0	1000	0	1000	0	0	0
Oceanobacillus_massiliensis_str_N_diop	0	0	1000	0	1000	0	1000	0	0	0
Oceanobacillus_picturae_strain_S1	0	0	1000	0	1000	0	1000	0	0	0
Ochrobactrum_anthropi_ATCC_49188	0	0	0	0	1000	0	1000	0	0	0
Ochrobactrum_anthropi_CTS_325	0	0	1000	0	1000	0	1000	0	0	0
Ochrobactrum_anthropi_FRAF13	0	0	1000	0	1000	0	1000	0	0	0
Ochrobactrum_intermedium_LMG_3301	0	0	0	0	1000	0	1000	0	0	0
Odoribacter_laneus_YIT_12061	0	0	0	0	1000	0	1000	0	0	0
Odoribacter_splanchnicus_1651_6_DSM_20712	0	0	1000	0	1000	0	1000	0	0	0
Odoribacter_splanchnicus_ERR1203973	0	0	0	0	1000	0	1000	0	0	0
Odoribacter_splanchnicus_ERR1204067	0	0	0	0	1000	0	1000	0	0	0
Odoribacter_splanchnicus_ERR2230125	0	0	0	0	1000	0	1000	0	0	0
Oligella_urethralis_DNF00040	0	0	0	0	1000	0	1000	0	1000	0
Oligella_urethralis_DSM_7531	0	0	0	0	1000	0	1000	0	1000	0
Oligella_urethralis_UMB0345_21837_8_92_1	0	0	0	0	1000	0	1000	0	1000	0
Olsenella_profusa_F019501	0	0	0	0	0	0	0	0	0	1000
Olsenella_uli_DSM_7084	0	0	0	0	0	0	1000	0	0	1000
Oribacterium_sinus_F0268	0	0	0	0	1000	0	1000	0	0	0
Oribacterium_sp_oral_taxon_078_str_F0262	0	0	0	0	0	0	0	0	0	0
Oscillibacter_nov_ERR1022355	0	0	0	0	1000	0	1000	0	0	0
Oscillibacter_nov_ERR1022407	0	0	0	0	1000	0	1000	0	0	0
Oscillibacter_ruminantium_GH1	0	0	1000	0	1000	0	1000	0	0	0
Oscillibacter_sp_KLE_1728	0	0	0	0	1000	0	1000	0	0	0
Oscillibacter_sp_KLE_1745	0	0	0	0	1000	0	1000	0	0	0
Oxalobacter_formigenes_HOxBLS	0	0	0	0	1000	0	1000	0	0	0
Oxalobacter_formigenes_OXCC13	0	0	0	0	0	0	0	0	0	0
Paenibacillus_alvei_DSM_29	0	0	1000	0	1000	0	1000	0	0	1000
Paenibacillus_antibioticophila_GD11	0	0	1000	0	0	0	1000	0	0	0
Paenibacillus_barengoltzii_G22	0	0	1000	0	0	0	0	0	0	1000
Paenibacillus_daejeonensis_DSM_15491	0	0	1000	1000	1000	0	0	0	0	1000
Paenibacillus_graminis_RSA19	0	0	1000	0	0	0	0	0	0	0
Paenibacillus_ihuae_GD6	0	0	1000	0	0	0	0	0	0	0
Paenibacillus_lactis_154	0	0	1000	0	0	0	1000	0	0	1000
Paenibacillus_senegalensis_JC66	0	0	1000	0	0	0	0	0	0	0

Paenibacillus_sp_HGF5	0	0	1000	0	0	0	1000	0	0	0
Paenibacillus_sp_HGF7	0	0	1000	0	0	0	0	0	0	0
Paenibacillus_sp_HGH0039	0	0	1000	0	0	0	0	0	0	0
Paenibacillus_sp_HW567	0	0	1000	1000	0	0	0	0	0	0
Paenibacillus_sp_ICGEB2008	0	0	1000	0	1000	0	1000	0	0	0
Paenibacillus_sp_JDR_2	0	0	1000	1000	0	0	0	0	0	0
Paeniclostridium_sordellii_8483	0	0	0	0	1000	0	1000	0	0	0
Paeniclostridium_sordellii_AF05_25	0	0	0	0	1000	0	1000	0	0	0
Paeniclostridium_sordellii_AM370	0	0	0	0	1000	0	1000	0	0	0
Paeniclostridium_sordellii_ATCC_9714	0	0	1000	0	1000	0	1000	0	0	0
Paeniclostridium_sordellii_CBA7122	0	0	0	0	1000	0	1000	0	0	0
Paeniclostridium_sordellii_DA108	0	0	0	0	1000	0	1000	0	0	0
Paeniclostridium_sordellii_E204	0	0	0	0	1000	0	1000	0	0	0
Paeniclostridium_sordellii_JGS444	0	0	0	0	1000	0	1000	0	0	0
Paeniclostridium_sordellii_JGS445	0	0	0	0	1000	0	1000	0	0	0
Paeniclostridium_sordellii_JGS6956	0	0	0	0	1000	0	1000	0	0	0
Paeniclostridium_sordellii_JGS6961	0	0	0	0	1000	0	1000	0	0	0
Paeniclostridium_sordellii_MGYG_HGUT_00157	0	0	0	0	1000	0	1000	0	0	0
Paeniclostridium_sordellii_R15892	0	0	0	0	1000	0	1000	0	0	0
Paeniclostridium_sordellii_R26833	0	0	0	0	1000	0	1000	0	0	0
Paeniclostridium_sordellii_R27882	0	0	0	0	1000	0	1000	0	0	0
Paeniclostridium_sordellii_R28058	0	0	0	0	1000	0	1000	0	0	0
Paeniclostridium_sordellii_R29426	0	0	0	0	1000	0	1000	0	0	0
Paeniclostridium_sordellii_R30684	0	0	0	0	1000	0	1000	0	0	0
Paeniclostridium_sordellii_R31809	0	0	0	0	1000	0	1000	0	0	0
Paeniclostridium_sordellii_R32462	0	0	0	0	1000	0	1000	0	0	0
Paeniclostridium_sordellii_R32668	0	0	0	0	1000	0	1000	0	0	0
Paeniclostridium_sordellii_R32921	0	0	0	0	1000	0	1000	0	0	0
Paeniclostridium_sordellii_R32977	0	0	0	0	1000	0	1000	0	0	0
Paeniclostridium_sordellii_SSCC18392	0	0	0	0	1000	0	1000	0	0	0
Paeniclostridium_sordellii_SSCC18838	0	0	0	0	1000	0	1000	0	0	0
Paeniclostridium_sordellii_SSCC26591	0	0	0	0	1000	0	1000	0	0	0
Paeniclostridium_sordellii_SSCC32135	0	0	0	0	1000	0	1000	0	0	0
Paeniclostridium_sordellii_SSCC33587	0	0	0	0	1000	0	1000	0	0	0
Paeniclostridium_sordellii_SSCC33589	0	0	0	0	1000	0	1000	0	0	0
Paeniclostridium_sordellii_SSCC35109	0	0	0	0	1000	0	1000	0	0	0
Paeniclostridium_sordellii_SSCC37615	0	0	0	0	1000	0	1000	0	0	0
Paeniclostridium_sordellii_SSCC42239	0	0	0	0	1000	0	1000	0	0	0
Paeniclostridium_sordellii_UMC1	0	0	0	0	1000	0	1000	0	0	0
Paeniclostridium_sordellii_UMC164	0	0	0	0	1000	0	1000	0	0	0
Paeniclostridium_sordellii_UMC178	0	0	0	0	1000	0	1000	0	0	0
Paeniclostridium_sordellii_UMC4401	0	0	0	0	1000	0	1000	0	0	0
Paeniclostridium_sordellii_UMC4404	0	0	0	0	1000	0	1000	0	0	0
Paeniclostridium_sordellii_VPI_9048	0	0	0	0	1000	0	1000	0	0	0
Paeniclostridium_sordellii_W10	0	0	0	0	1000	0	1000	0	0	0
Paeniclostridium_sordellii_W2922	0	0	0	0	1000	0	1000	0	0	0
Paeniclostridium_sordellii_W2945	0	0	0	0	1000	0	1000	0	0	0
Paeniclostridium_sordellii_W2946	0	0	0	0	1000	0	1000	0	0	0
Paeniclostridium_sordellii_W2948	0	0	0	0	1000	0	1000	0	0	0
Paeniclostridium_sordellii_W2967	0	0	0	0	1000	0	1000	0	0	0
Paeniclostridium_sordellii_W2975	0	0	0	0	1000	0	1000	0	0	0
Paeniclostridium_sordellii_W3025	0	0	0	0	1000	0	1000	0	0	0
Paeniclostridium_sordellii_W3026	0	0	0	0	1000	0	1000	0	0	0
Paenisporosarcina_sp_HGH0030	0	0	1000	0	0	0	1000	0	0	0
Paenisporosarcina_sp_TG_14	0	0	1000	0	1000	0	1000	0	0	0
Paludibacter_jiangxiensis_NM7	0	0	0	0	0	0	0	0	0	0
Paludibacter_propionicigenes_WB4	0	0	0	1000	0	0	0	0	0	0
Pantoea_agglomerans_190	0	0	1000	0	1000	0	1000	0	0	0
Pantoea_agglomerans_299R	0	0	0	0	1000	0	1000	0	0	0
Pantoea_agglomerans_3	0	0	1000	0	1000	0	1000	0	0	0
Pantoea_agglomerans_4	0	0	1000	0	1000	0	1000	0	0	0
Pantoea_agglomerans_IG1	0	0	0	0	1000	0	1000	0	0	1000
Pantoea_agglomerans_MP2	0	0	1000	0	1000	0	1000	0	0	0
Pantoea_agglomerans_P10c	0	0	1000	0	1000	0	1000	0	0	0
Pantoea_sp_YR343	0	0	1000	0	1000	0	1000	0	0	0
Papillibacter_cinnamivorans_DSM_12816	0	0	0	0	0	0	0	0	0	0
Parabacteroides_chinchillae_DSM_29073	0	0	0	0	0	0	0	0	0	0
Parabacteroides_distasonis_ATCC_8503	0	0	1000	1000	0	0	1000	0	0	0
Parabacteroides_distasonis_CL03T12C09	0	0	0	1000	1000	0	1000	0	0	0
Parabacteroides_distasonis_CL09T03C24	0	0	0	1000	1000	0	1000	0	0	0
Parabacteroides_distasonis_ERR1022281	0	0	0	0	1000	0	1000	0	0	0
Parabacteroides_distasonis_ERR1022330	0	0	0	0	1000	0	1000	0	0	0
Parabacteroides_distasonis_ERR1022415	0	0	0	0	1000	0	1000	0	0	0
Parabacteroides_distasonis_ERR1022462	0	0	0	0	1000	0	1000	0	0	0
Parabacteroides_distasonis_ERR1204048	0	0	0	0	1000	0	1000	0	0	0
Parabacteroides_distasonis_ERR2221129	0	0	0	0	1000	0	1000	0	0	0
Parabacteroides_distasonis_ERR2221168	0	0	0	0	1000	0	1000	0	0	0
Parabacteroides_distasonis_ERR2230146	0	0	0	0	1000	0	1000	0	0	0
Parabacteroides_distasonis_ERR2230165	0	0	0	0	1000	0	1000	0	0	0
Parabacteroides_goldsteinii_CL02T12C30	0	0	0	1000	0	0	1000	0	0	0
Parabacteroides_goldsteinii_dnLKV18	0	0	0	1000	0	0	1000	0	0	0
Parabacteroides_goldsteinii_DSM_19448_WAL_12	0	0	0	0	0	0	1000	0	0	0
Parabacteroides_goldsteinii_ERR2230163	0	0	0	0	0	0	1000	0	0	0
Parabacteroides_gordonii_DSM_23371	0	0	0	0	0	0	0	0	0	0
Parabacteroides_johnsonii_CL02T12C29	0	0	0	1000	0	0	0	0	0	0
Parabacteroides_johnsonii_DSM_18315	0	0	0	1000	0	0	0	0	0	0
Parabacteroides_merdae_ATCC_43184	0	0	0	1000	0	0	1000	0	0	0
Parabacteroides_merdae_CL03T12C32	0	0	0	1000	0	0	1000	0	0	0
Parabacteroides_merdae_CL09T00C40	0	0	0	45.52352049	0	0	1000	0	0	0
Parabacteroides_merdae_ERR1022364	0	0	0	45.52352049	0	0	1000	0	0	0
Parabacteroides_merdae_ERR2230111	0	0	0	45.52352049	0	0	0	0	0	0
Parabacteroides_nov_ERR1203954	0	0	0	0	1000	0	1000	0	0	0
Parabacteroides_sp_ASF519	0	0	0	1000	0	0	1000	0	0	0
Parabacteroides_sp_D13	0	0	0	1000	0	0	0	0	0	0

Parabacteroides_sp_D25	0	0	0	1000	1000	0	0	0	0	0
Parabacteroides_sp_D26	0	0	1000	0	1000	0	0	0	0	0
Paraburkholderia_sediminicola_LMG_24238	0	0	1000	0	0	0	0	0	0	0
Paraclostridium_bifermentans_ATCC_19299	0	0	0	0	1000	0	1000	0	0	0
Paraclostridium_bifermentans_ATCC_638	0	0	0	0	1000	0	1000	0	0	0
Paraclostridium_bifermentans_Cbm	0	0	0	0	1000	0	1000	0	0	0
Paraclostridium_bifermentans_MGYG_HGUT_000	0	0	0	0	1000	0	1000	0	0	0
Paraclostridium_bifermentans_MHMC_14	0	0	0	0	1000	0	1000	0	0	0
Paraclostridium_bifermentans_SU1074NT	0	0	0	0	1000	0	1000	0	0	0
Paraclostridium_bifermentans_WYM	0	0	0	0	1000	0	1000	0	0	0
Paracoccus_yeei_ATCC_BAA_599	0	0	1000	0	0	0	0	0	0	0
Paraeggerthella_hongkongensis_RC2_2	0	0	1000	0	0	0	1000	0	0	0
Parapedobacter_koreensis_Jip14	0	0	1000	0	1000	0	0	1000	0	0
Paraprevotella_clara_YIT_11840	0	0	0	1000	0	0	0	0	0	0
Paraprevotella_xylaniphila_YIT_11841	0	0	0	1000	0	0	0	0	0	0
Parascardovia_denticolens_DSM_10105_JCM_125	0	0	0	0	0	0	1000	0	0	0
Parascardovia_denticolens_F0305	0	0	0	0	0	0	1000	0	0	0
Parascardovia_denticolens_IPLA_20019	0	0	0	0	0	0	1000	0	0	0
Parasporobacterium_paucivorans_DSM_15970	0	0	0	0	0	0	1000	0	0	0
Parasutterella_excrementihominis_YIT_11859	0	0	0	0	1000	0	0	0	0	0
Parvibacter_caecicola_DSM_22242	0	0	0	0	1000	0	1000	0	0	0
Parvibacter_caecicola_NM48_B13	0	0	0	0	1000	0	1000	0	0	0
Parvimonas_micra_ATCC_33270	0	0	0	0	1000	0	1000	0	0	0
Parvimonas_sp_oral_taxon_110_str_F0139	0	0	0	0	829.2682927	0	1000	0	0	0
Pasteurella_caecimuris_NM44_TS2_9	0	0	0	0	0	0	1000	0	1000	0
Pediococcus_acidilactici_7_4	0	0	0	0	1000	0	0	0	0	0
Pediococcus_acidilactici_D3	0	0	0	0	1000	0	1000	0	0	0
Pediococcus_acidilactici_DSM_20284	0	0	0	0	0	0	0	0	0	0
Pediococcus_acidilactici_ERR2221169	0	0	0	0	1000	0	0	0	0	0
Pediococcus_acidilactici_ERR2221292	0	0	0	0	1000	0	0	0	0	0
Pediococcus_acidilactici_ERR2221293	0	0	0	0	1000	0	0	0	0	0
Pediococcus_acidilactici_ERR2221294	0	0	0	0	1000	0	0	0	0	0
Pediococcus_acidilactici_ERR2221295	0	0	0	0	1000	0	0	0	0	0
Pediococcus_acidilactici_ERR2221299	0	0	0	0	1000	0	0	0	0	0
Pediococcus_acidilactici_ERR2221316	0	0	0	0	1000	0	0	0	0	0
Pediococcus_acidilactici_ERR2221333	0	0	0	0	1000	0	0	0	0	0
Pediococcus_acidilactici_ERR2221334	0	0	0	0	1000	0	0	0	0	0
Pediococcus_acidilactici_ERR2221382	0	0	0	0	1000	0	0	0	0	0
Pediococcus_acidilactici_MA18_5M	0	0	0	0	0	0	0	0	0	0
Pediococcus_pentosaceus_ATCC_25745	0	0	0	0	1000	0	1000	0	0	0
Pediococcus_pentosaceus_IE_3	0	0	0	0	1000	0	1000	0	0	0
Pelomonas_saccharophila_DSM_654	0	0	0	0	0	0	0	0	1000	0
Peptoniphilus_coxii_DNF00729	0	0	1000	0	1000	0	1000	0	0	0
Peptoniphilus_duerdenii_ATCC_BAA_1640	0	0	0	0	1000	0	1000	0	0	0
Peptoniphilus_grossensis_35_6_1	0	0	1000	0	1000	0	1000	0	0	0
Peptoniphilus_harei_ACS_146_V_Sch2b	0	0	0	0	1000	0	1000	0	0	0
Peptoniphilus_harei_ERR2221371	0	0	0	0	1000	0	1000	0	0	0
Peptoniphilus_indolicus_ATCC_29427	0	0	1000	0	1000	0	1000	0	0	0
Peptoniphilus_lacrimalis_315_B	0	0	0	0	1000	0	1000	0	0	0
Peptoniphilus_lacrimalis_DSM_7455	0	0	0	0	1000	0	1000	0	0	0
Peptoniphilus_nov_ERR2221395	0	0	1000	0	1000	0	1000	0	0	0
Peptoniphilus_obesi_ph1	0	0	0	0	1000	0	1000	0	0	0
Peptoniphilus_senegalensis_JC140	0	0	0	0	1000	0	1000	0	0	0
Peptoniphilus_timonensis_JC401	0	0	0	0	1000	0	1000	0	0	0
Peptostreptococcus_anaerobius_653_L	0	0	0	0	1000	0	1000	0	0	0
Peptostreptococcus_anaerobius_DSM_2949	0	0	0	0	1000	0	1000	0	0	0
Peptostreptococcus_anaerobius_VPI_4330	0	0	0	0	1000	0	1000	0	0	0
Peptostreptococcus_russellii_Calf135	0	0	0	0	1000	0	1000	0	0	0
Peptostreptococcus_stomatis_DSM_17678	0	0	0	0	1000	0	1000	0	0	0
Phascolarctobacterium_faecium_DSM_14760	0	0	1000	0	1000	0	1000	0	0	0
Phascolarctobacterium_succinatutens_YIT_12067	0	0	0	0	1000	0	1000	0	0	0
Phocaeicola_abscessus_CCUG_55929	0	0	0	0	1000	0	0	0	1000	0
Photobacterium_angustum_S14	0	0	0	0	1000	0	1000	0	0	0
Photobacterium_damselae_Phdp_Wu_1	0	0	0	0	1000	0	1000	0	0	0
Photobacterium_damselae_subsp_damselae_CIP_	0	0	0	0	1000	0	1000	0	0	0
Photobacterium_damselae_subsp_damselae_KC_	0	0	0	0	1000	0	1000	0	0	0
Photobacterium_leiognathi_subsp_mandapamens	0	0	0	0	1000	0	1000	0	0	0
Photobacterium_sp_SKA34	0	0	0	0	1000	0	1000	0	0	0
Photorhabdus_luminescens_BA1	0	0	0	0	0	0	1000	0	0	0
Photorhabdus_luminescens_H1_H1_C1	0	0	0	0	0	0	1000	0	0	0
Photorhabdus_luminescens_NBAII_H75HRPL105	0	0	0	0	0	0	1000	0	0	0
Pirellula_staleyi_DSM_6068	0	0	0	0	1000	0	0	0	1000	0
Plesiomonas_shigelloides_302_73	0	0	0	0	1000	0	1000	0	0	1000
Plesiomonas_shigelloides_GN7	0	0	0	0	1000	0	1000	0	0	0
Polynesia_massiliensis_MS3	0	0	0	0	1000	0	1000	0	0	0
Porphyromonadaceae_nov_ERR2221201	0	0	1000	0	0	0	0	0	0	0
Porphyromonadaceae_nov_ERR2221202	0	0	1000	0	0	0	0	0	0	0
Porphyromonadaceae_nov_ERR2221365	0	0	0	0	0	0	0	0	0	0
Porphyromonadaceae_nov_ERR2221374	0	0	0	0	1000	0	0	0	0	0
Porphyromonadaceae_nov_ERR2221378	0	0	0	0	0	0	0	0	0	0
Porphyromonas_asaccharolytica_DSM_20707	0	0	0	0	0	0	0	0	0	0
Porphyromonas_asaccharolytica_PR426713P_I	0	0	0	0	0	0	0	0	0	0
Porphyromonas_bennonis_DSM_23058_JCM_163	0	0	1000	0	0	0	1000	0	0	0
Porphyromonas_endodontalis_ATCC_35406	0	0	0	0	0	0	1000	0	0	0
Porphyromonas_gingivalis_A7436	0	0	0	0	0	0	0	0	0	0
Porphyromonas_gingivalis_ATCC_33277	0	0	0	0	0	0	0	0	0	0
Porphyromonas_gingivalis_TDC60	0	0	0	0	0	0	0	0	0	0
Porphyromonas_gingivalis_W83	0	0	1000	0	0	0	0	0	0	0
Porphyromonas_gingivicanis_JCM_15907	0	0	0	0	0	0	0	0	0	0
Porphyromonas_somerae_DSM_23386	0	0	0	0	0	0	1000	0	0	0
Porphyromonas_uenonis_60_3	0	0	0	0	0	0	0	0	0	0
Porphyromonas_uenonis_DSM_23387	0	0	0	0	0	0	0	0	0	0
Prevotella_albensis_DSM_11370	0	0	0	0	0	0	1000	0	0	0
Prevotella_amnii_CRIS_21A_A	0	0	0	0	0	0	0	0	0	0

Prevotella_amnii_DNF00058	0	0	0	0	0	0	0	0	0	0
Prevotella_amnii_DNF00307	0	0	0	0	0	0	0	0	0	0
Prevotella_amnii_DSM_23384_JCM_14753	0	0	0	0	0	0	0	0	0	0
Prevotella_aurantiaca_JCM_15754	0	0	0	0	0	0	0	0	0	0
Prevotella_baroniae_DSM_16972_JCM_13447	0	0	0	0	0	0	0	0	0	0
Prevotella_baroniae_F0067	0	0	0	0	0	0	0	0	0	0
Prevotella_bergensis_DSM_17361	0	0	1000	0	0	0	0	0	0	0
Prevotella_bivia_DSM_20514	0	0	0	0	0	0	1000	0	0	0
Prevotella_bivia_JCVIHMP010	0	0	0	0	0	0	0	0	0	0
Prevotella_brevis_ATCC_19188	0	0	1000	0	1000	0	1000	0	0	0
Prevotella_bryantii_B14	0	0	0	1000	1000	0	1000	0	0	0
Prevotella_bryantii_C21a	0	0	1000	0	1000	0	1000	0	0	0
Prevotella_buccae_ATCC_33574	0	0	0	0	0	0	1000	0	0	0
Prevotella_buccae_D17	0	0	0	0	0	0	1000	0	0	0
Prevotella_buccalis_ATCC_35310	0	0	0	0	0	0	0	0	0	0
Prevotella_buccalis_DNF00853	0	0	0	0	0	0	0	0	0	0
Prevotella_buccalis_DNF00985	0	0	0	0	0	0	0	0	0	0
Prevotella_buccalis_UMB0536_16933_8_22_1	0	0	0	0	0	0	0	0	0	0
Prevotella_conceptionensis_9403948	0	0	0	0	0	0	1000	0	0	0
Prevotella_copri_CB7_DSM_18205	0	0	0	1000	1000	0	0	0	0	0
Prevotella_copri_ERR1022397	0	0	0	0	1000	0	0	0	0	0
Prevotella_corporis_DSM_18810	0	0	0	0	0	0	0	0	0	0
Prevotella_corporis_MJR7716	0	0	1000	0	0	0	0	0	0	0
Prevotella_dentalis_DSM_3688	0	0	0	1000	0	0	0	0	0	0
Prevotella_dentasini_JCM_15908	0	0	0	0	0	0	0	0	0	0
Prevotella_denticola_CRIS_18C_A	0	0	0	0	0	0	0	0	0	0
Prevotella_denticola_DSM20614	0	0	0	0	0	0	0	0	0	0
Prevotella_denticola_F0289	0	0	0	0	0	0	0	0	0	0
Prevotella_disiens_FB035_09AN	0	0	0	0	0	0	0	0	0	0
Prevotella_disiens_JCM_6334	0	0	0	0	0	0	0	0	0	0
Prevotella_enoeca_JCM_12259	0	0	0	0	0	0	0	0	0	0
Prevotella_histicola_F0411	0	0	0	0	0	0	0	0	0	0
Prevotella_histicola_JCM_15637_DNF00424	0	0	0	0	0	0	0	0	0	0
Prevotella_intermedia_17	0	0	0	0	0	0	0	0	0	0
Prevotella_intermedia_ATCC_25611	0	0	0	0	0	0	0	0	0	0
Prevotella_loescheii_DSM_19665	0	0	0	0	0	0	0	0	0	0
Prevotella_maculosa_DSM_19339_JCM_15638	0	0	0	0	0	0	0	0	0	0
Prevotella_maculosa_OT_289	0	0	0	0	0	0	0	0	0	0
Prevotella_melaninogenica_ATCC_25845	0	0	0	0	0	0	0	0	0	0
Prevotella_melaninogenica_D18	0	0	0	0	0	0	0	0	0	0
Prevotella_micans_DSM_21469_JCM_16134	0	0	0	0	0	0	0	0	0	0
Prevotella_micans_F0438	0	0	0	0	0	0	0	0	0	0
Prevotella_multiformis_DSM_16608	0	0	0	0	0	0	0	0	0	0
Prevotella_multisaccharivorax_DSM_17128	0	0	0	0	0	0	1000	0	0	0
Prevotella_nanceiensis_DSM_19126	0	0	0	0	0	0	0	0	0	0
Prevotella_nigrescens_ATCC_33563	0	0	0	0	0	0	0	0	0	0
Prevotella_nigrescens_F0103	0	0	0	0	0	0	0	0	0	0
Prevotella_oralis_ATCC_33269	0	0	0	0	0	0	1000	0	0	0
Prevotella_oralis_HGA0225	0	0	0	0	0	0	1000	0	0	0
Prevotella_oris_C735	0	0	0	0	0	0	0	0	0	0
Prevotella_oris_DSM_18711_JCM_12252	0	0	0	0	0	0	0	0	0	0
Prevotella_oris_F0302	0	0	0	0	0	0	0	0	0	0
Prevotella_oulorum_ATCC_43324	0	0	0	0	0	0	0	0	0	0
Prevotella_oulorum_F0390	0	0	0	0	0	0	0	0	0	0
Prevotella_oulorum_JCM_14966	0	0	0	0	1000	0	0	0	0	0
Prevotella_pallens_ATCC_700821	0	0	0	0	0	0	0	0	0	0
Prevotella_pleuritidis_F0068	0	0	0	0	0	0	0	0	0	0
Prevotella_pleuritidis_JCM_14110	0	0	0	0	0	0	0	0	0	0
Prevotella_ruminicola_23	0	0	0	1000	0	0	1000	0	0	0
Prevotella_salivae_DSM_15606	0	0	0	0	0	0	0	0	0	0
Prevotella_shahii_JCM_12083	0	0	0	0	0	0	0	0	0	0
Prevotella_sp_C561	0	0	0	0	0	0	0	0	0	0
Prevotella_sp_oral_taxon_473_str_F0040	0	0	0	0	0	0	0	0	0	0
Prevotella_stercorea_DSM_18206	0	0	0	0	1000	0	0	0	0	0
Prevotella_timonensis_4401737	0	0	0	0	0	0	0	0	0	0
Prevotella_timonensis_CRIS_5C_B1	0	0	0	0	0	0	0	0	0	0
Prevotella_veroralis_DSM_19559	0	0	0	0	0	0	0	0	0	0
Prevotella_veroralis_F0319	0	0	0	0	0	0	0	0	0	0
Propionibacterium_acidifaciens_DSM_21887	0	0	1000	0	1000	0	1000	0	0	0
Propionibacterium_acidifaciens_F0233	0	0	1000	0	1000	0	1000	0	0	0
Propionibacterium_acidipropionici_ATCC_4875	0	0	0	0	0	0	1000	0	0	0
Propionibacterium_avidum_44067	0	0	0	1000	0	0	1000	0	0	0
Propionibacterium_avidum_ATCC_25577	0	0	0	1000	0	0	1000	0	0	0
Propionibacterium_freudenreichii_ERR2221306	0	0	1000	0	1000	0	0	0	0	0
Propionibacterium_freudenreichii_subsp_shermar	0	0	0	0	1000	0	0	0	0	0
Propionibacterium_granulosum_DSM_20700	0	0	0	0	0	0	1000	0	0	0
Propionibacterium_jensenii_DSM_20535	0	0	1000	0	0	0	1000	0	0	0
Propionibacterium_namnetense_NTS_31307302	0	0	0	0	0	0	1000	0	1000	0
Propionibacterium_namnetense_SK182B_JCVI	0	0	0	1000	0	0	1000	0	1000	0
Propionibacterium_propionicum_F0230a	0	0	0	0	0	0	1000	0	0	0
Propionibacterium_sp_409_HC1	0	0	0	1000	0	0	1000	0	0	0
Propionibacterium_sp_5_U_42AFAA	0	0	0	1000	0	0	1000	0	0	0
Propionibacterium_sp_HGH0353	0	0	0	1000	0	0	1000	0	0	0
Proteiniphilum_acetatigenes_DSM_18083	0	0	1000	0	1000	0	0	0	1000	0
Proteus_mirabilis_AOUC_001	0	0	0	0	0	0	1000	0	0	0
Proteus_mirabilis_ATCC_29906	0	0	0	0	0	0	1000	0	0	1000
Proteus_mirabilis_C05028	0	0	0	0	1000	0	1000	0	0	0
Proteus_mirabilis_CYPM1	0	0	0	0	0	0	1000	0	0	0
Proteus_mirabilis_HI4320	0	0	0	0	1000	0	1000	0	0	0
Proteus_mirabilis_PR03	0	0	0	0	1000	0	1000	0	0	0
Proteus_mirabilis_WGLW4	0	0	0	0	1000	0	1000	0	0	0
Proteus_mirabilis_WGLW6	0	0	0	0	1000	0	1000	0	0	0
Proteus_penneri_ATCC_35198	0	0	0	0	0	0	1000	0	0	1000
Proteus_vulgaris_08MAS1600	0	0	0	0	0	0	1000	0	0	0

Proteus_vulgaris_ATCC_49132	0	0	0	0	0	0	1000	0	0	0
Proteus_vulgaris_CICC	0	0	0	0	0	0	1000	0	0	0
Proteus_vulgaris_CSUR_P1867	0	0	0	0	0	0	1000	0	0	0
Proteus_vulgaris_CSUR_P1868	0	0	0	0	0	0	1000	0	0	0
Proteus_vulgaris_FDAARGOS_366	0	0	0	0	0	0	1000	0	0	0
Proteus_vulgaris_FDAARGOS_556	0	0	0	0	0	0	1000	0	0	0
Proteus_vulgaris_KCTC_2579	0	0	0	0	0	0	1000	0	0	0
Proteus_vulgaris_MGYG_HGUT_02516	0	0	0	0	0	0	1000	0	0	0
Proteus_vulgaris_NCTC10376	0	0	0	0	0	0	1000	0	0	0
Proteus_vulgaris_NCTC13145	0	0	0	0	0	0	1000	0	0	0
Proteus_vulgaris_NCTC401	0	0	0	0	0	0	1000	0	0	0
Providencia_alcalifaciens_Dmel2	0	0	0	0	1000	0	1000	0	0	0
Providencia_alcalifaciens_DSM_30120	0	0	0	0	1000	0	1000	0	0	0
Providencia_burhodogranariea_DSM_19968	0	0	1000	0	1000	0	1000	0	0	0
Providencia_rettgeri_Dmel1	0	0	0	0	1000	0	1000	0	0	0
Providencia_rettgeri_DSM_1131	0	0	0	0	1000	0	1000	0	0	0
Providencia_rustigianii_DSM_4541	0	0	0	0	1000	0	1000	0	0	0
Providencia_sneebia_DSM_19967	0	0	0	0	1000	0	1000	0	0	0
Providencia_stuartii_ATCC_25827	0	0	1000	0	1000	0	1000	0	0	0
Providencia_stuartii_FDAARGOS_87	0	0	1000	0	1000	0	1000	0	0	0
Providencia_stuartii_MRSN_2154	0	0	1000	0	1000	0	1000	0	0	0
Pseudoalteromonas_arctica_A_37_1_2	0	0	1000	0	1000	0	0	1000	0	0
Pseudoalteromonas_arctica_MelAa3	0	0	1000	0	1000	0	0	1000	0	0
Pseudobutyrivibrio_ruminis_AD2017	0	0	0	0	1000	0	0	0	0	0
Pseudoflavonifractor_capillosus_ERR1022447	0	0	0	0	0	0	1000	0	0	0
Pseudoflavonifractor_capillosus_strain_ATCC_297	0	0	0	0	0	0	1000	0	0	0
Pseudomonas_aeruginosa_152504	0	0	1000	0	1000	0	1000	0	1000	0
Pseudomonas_aeruginosa_18A	0	0	1000	0	1000	0	1000	0	1000	0
Pseudomonas_aeruginosa_19BR	0	0	1000	0	1000	0	1000	0	1000	0
Pseudomonas_aeruginosa_213BR	0	0	1000	0	1000	0	1000	0	1000	0
Pseudomonas_aeruginosa_2192	0	0	1000	0	1000	0	1000	0	1000	0
Pseudomonas_aeruginosa_39016	0	0	1000	0	1000	0	1000	0	1000	0
Pseudomonas_aeruginosa_9BR	0	0	1000	0	1000	0	1000	0	1000	0
Pseudomonas_aeruginosa_AH16	0	0	1000	0	1000	0	1000	0	1000	0
Pseudomonas_aeruginosa_ATCC_14886	0	0	1000	0	1000	0	1000	0	1000	0
Pseudomonas_aeruginosa_B136_33	875	0	1000	0	1000	0	1000	0	1000	0
Pseudomonas_aeruginosa_C3719	0	0	1000	0	1000	0	1000	0	1000	0
Pseudomonas_aeruginosa_CI27	0	0	1000	0	1000	0	1000	0	1000	0
Pseudomonas_aeruginosa_DK2	875	0	1000	0	1000	0	1000	0	1000	0
Pseudomonas_aeruginosa_DQ8	0	0	1000	0	1000	0	1000	0	1000	0
Pseudomonas_aeruginosa_E2	0	0	1000	0	1000	0	1000	0	1000	0
Pseudomonas_aeruginosa_F22031	0	0	1000	0	1000	0	1000	0	1000	0
Pseudomonas_aeruginosa_LCT_PA102	0	0	1000	0	1000	0	1000	0	1000	0
Pseudomonas_aeruginosa_LESB58	875	0	1000	0	1000	0	1000	0	1000	0
Pseudomonas_aeruginosa_M18	833.3333333	0	1000	0	1000	0	1000	0	1000	0
Pseudomonas_aeruginosa_MPAO1_P1	0	0	1000	0	1000	0	1000	0	1000	0
Pseudomonas_aeruginosa_MPAO1_P2	0	0	1000	0	1000	0	1000	0	1000	0
Pseudomonas_aeruginosa_MRW44_1	0	0	1000	0	1000	0	1000	0	1000	0
Pseudomonas_aeruginosa_MSH_10	0	0	1000	0	1000	0	1000	0	1000	0
Pseudomonas_aeruginosa_NCGM2_S1	922.0779221	0	1000	0	1000	0	1000	0	1000	0
Pseudomonas_aeruginosa_NCMG1179	0	0	1000	0	1000	0	1000	0	1000	0
Pseudomonas_aeruginosa_PA14	0	0	1000	0	1000	0	1000	0	1000	0
Pseudomonas_aeruginosa_PA21_ST175	0	0	1000	0	1000	0	1000	0	1000	0
Pseudomonas_aeruginosa_PA45	0	0	1000	0	1000	0	1000	0	1000	0
Pseudomonas_aeruginosa_PA7	875	0	1000	0	1000	0	1000	0	1000	0
Pseudomonas_aeruginosa_PABL056	0	0	1000	0	1000	0	1000	0	1000	0
Pseudomonas_aeruginosa_PACS2	0	0	1000	0	1000	0	1000	0	1000	0
Pseudomonas_aeruginosa_PADK2_CF510	0	0	1000	0	1000	0	1000	0	1000	0
Pseudomonas_aeruginosa_PAK	0	0	1000	0	1000	0	1000	1000	1000	0
Pseudomonas_aeruginosa_PAO1	814.8148148	0	1000	0	1000	0	1000	0	1000	0
Pseudomonas_aeruginosa_PAO579	0	0	1000	0	1000	0	1000	0	1000	0
Pseudomonas_aeruginosa_PGPR2	0	0	1000	0	1000	0	1000	0	1000	0
Pseudomonas_aeruginosa_SJTD_1	0	0	1000	0	1000	0	1000	0	1000	0
Pseudomonas_aeruginosa_UCBPP_PA14	875	0	1000	0	1000	0	1000	0	1000	0
Pseudomonas_aeruginosa_XMG	0	0	1000	0	1000	0	1000	0	1000	0
Pseudomonas_alcaliphila_34	0	0	1000	0	1000	0	1000	0	0	1000
Pseudomonas_fluorescens_A506	0	0	1000	0	1000	0	1000	0	1000	0
Pseudomonas_fluorescens_F113	0	0	1000	0	1000	0	1000	0	1000	0
Pseudomonas_fluorescens_LMG_5329	0	0	1000	0	1000	0	1000	0	1000	0
Pseudomonas_fluorescens_NCIMB_11764	0	0	1000	0	1000	0	1000	0	1000	0
Pseudomonas_fluorescens_Pf_5	0	0	1000	0	1000	0	1000	0	0	0
Pseudomonas_fluorescens_PfO_1	0	0	1000	0	1000	0	1000	0	1000	1000
Pseudomonas_fluorescens_Q2_87	0	0	1000	0	1000	0	1000	0	1000	0
Pseudomonas_fluorescens_Q8r1_96	0	0	1000	0	1000	0	1000	0	1000	0
Pseudomonas_fluorescens_R124	0	0	1000	0	1000	0	1000	0	1000	0
Pseudomonas_fluorescens_SBW25	0	0	1000	0	1000	0	1000	0	1000	0
Pseudomonas_fluorescens_SS101	0	0	1000	0	1000	0	1000	0	1000	0
Pseudomonas_fluorescens_UK4	0	0	1000	0	1000	0	1000	0	1000	0
Pseudomonas_fluorescens_WH6	0	0	1000	0	1000	0	1000	0	1000	0
Pseudomonas_fragi_A22	0	0	1000	0	1000	0	1000	0	0	0
Pseudomonas_fragi_B25	0	0	1000	0	1000	0	1000	0	0	0
Pseudomonas_fragi_F1794	0	0	1000	0	1000	0	1000	0	0	0
Pseudomonas_fragi_NBRC_3458	0	0	1000	0	1000	0	1000	0	0	0
Pseudomonas_mendocina_DLHK	0	0	1000	0	0	0	1000	0	0	0
Pseudomonas_mendocina_EGD_AQ5	0	0	1000	0	0	0	1000	0	0	0
Pseudomonas_mendocina_NBRC_14162	0	0	1000	0	1000	0	1000	0	0	0
Pseudomonas_mendocina_NEB698	0	0	1000	0	0	0	1000	0	0	0
Pseudomonas_monteilii_NBRC_103158	0	0	1000	0	1000	0	1000	0	1000	1000
Pseudomonas_monteilii_QM	0	0	1000	0	1000	0	1000	0	1000	1000
Pseudomonas_monteilii_USDA_ARS_USMARC_56	0	0	1000	0	1000	0	1000	0	1000	0
Pseudomonas_nitroreducens_HBP1	0	0	1000	0	1000	0	1000	0	1000	1000
Pseudomonas_oleovorans_MOIL14HWK12	0	0	1000	0	1000	0	1000	0	1000	1000
Pseudomonas_otitidis_LNU_E_001	0	0	1000	0	1000	0	1000	0	0	0
Pseudomonas_psychrophila_HA_4	0	0	1000	0	1000	0	1000	0	0	0

Pseudomonas_psychrophila_RGCB_166	0	0	1000	0	1000	0	1000	0	0	0
Pseudomonas_putida_B001	0	0	1000	0	1000	0	1000	0	1000	0
Pseudomonas_putida_B6_2	0	0	1000	0	1000	0	1000	0	1000	0
Pseudomonas_putida_BIRD_1	0	0	1000	0	1000	0	1000	0	1000	0
Pseudomonas_putida_DLL_E4	0	0	1000	0	1000	0	1000	0	1000	0
Pseudomonas_putida_DOT_T1E	0	0	1000	0	1000	0	1000	0	1000	0
Pseudomonas_putida_F1	0	0	1000	0	1000	0	1000	0	1000	1000
Pseudomonas_putida_GB_1	0	0	1000	0	1000	0	1000	0	1000	0
Pseudomonas_putida_H8234	0	0	1000	0	1000	0	1000	0	1000	0
Pseudomonas_putida_HB3267	0	0	1000	0	1000	0	1000	0	1000	0
Pseudomonas_putida_KT2440	0	0	1000	0	1000	0	1000	0	1000	0
Pseudomonas_putida_LS46	0	0	1000	0	1000	0	1000	0	1000	0
Pseudomonas_putida_NB2011	0	0	1000	0	1000	0	1000	0	0	0
Pseudomonas_putida_NBRC_14164	0	0	1000	0	1000	0	1000	0	1000	0
Pseudomonas_putida_ND6	0	0	1000	0	1000	0	1000	0	1000	0
Pseudomonas_putida_PC2	0	0	1000	0	1000	0	1000	0	1000	0
Pseudomonas_putida_S12	0	0	1000	0	1000	0	1000	0	1000	0
Pseudomonas_putida_S16	0	0	1000	0	1000	0	1000	0	1000	0
Pseudomonas_putida_SJTE_1	0	0	1000	0	1000	0	1000	0	1000	0
Pseudomonas_putida_TRO1	0	0	1000	0	1000	0	1000	0	1000	0
Pseudomonas_putida_W619	0	0	1000	0	1000	0	1000	0	1000	0
Pseudomonas_stutzeri_28a24	0	0	1000	0	1000	0	1000	0	1000	0
Pseudomonas_stutzeri_A1501	0	0	1000	0	1000	0	1000	0	1000	0
Pseudomonas_stutzeri_ATCC_14405_CCUG_1615	0	0	1000	0	1000	0	1000	0	1000	0
Pseudomonas_stutzeri_ATCC_17588_LMG_11199	0	0	1000	0	1000	0	1000	0	1000	0
Pseudomonas_stutzeri_CCUG_29243	0	0	1000	0	1000	0	1000	0	1000	0
Pseudomonas_stutzeri_DSM_10701	0	0	1000	0	1000	0	1000	0	1000	0
Pseudomonas_stutzeri_DSM_4166	0	0	1000	0	1000	0	1000	0	1000	1000
Pseudomonas_stutzeri_KOS6	0	0	1000	0	1000	0	1000	0	1000	0
Pseudomonas_stutzeri_NF13	0	0	1000	0	1000	0	1000	0	1000	0
Pseudomonas_stutzeri_RCH2	0	0	1000	0	1000	0	1000	0	1000	0
Pseudomonas_stutzeri_SDM_LAC	0	0	1000	0	1000	0	1000	0	1000	0
Pseudomonas_stutzeri_SLG510A3_8	0	0	1000	0	1000	0	1000	0	1000	0
Pseudomonas_stutzeri_T13	0	0	1000	0	1000	0	1000	0	1000	0
Pseudomonas_stutzeri_TS44	0	0	1000	0	1000	0	1000	0	1000	0
Pseudomonas_stutzeri_XLDN_R	0	0	1000	0	1000	0	1000	0	1000	0
Pseudoramibacter_alactolyticus_ATCC_23263	0	0	0	0	0	0	1000	0	0	0
Pseudoxanthomonas_mexicana_CCH9_G4	0	0	1000	0	1000	0	0	0	0	0
Pyramidobacter_piscolens_W5455	0	0	0	0	1000	0	1000	0	0	0
Rahnella_aquatilis_CIP_78_65_ATCC_33071	0	0	0	0	1000	0	1000	0	0	0
Rahnella_aquatilis_DLL7529	0	0	0	0	1000	0	1000	0	0	0
Rahnella_aquatilis_HX2	1000	0	0	0	1000	0	1000	0	0	0
Ralstonia_insidiosa_FC1138	0	0	0	0	1000	0	0	0	0	0
Ralstonia_pickettii_12D	0	0	0	0	1000	0	0	0	1000	0
Ralstonia_pickettii_12J	0	0	0	0	1000	0	0	0	1000	0
Ralstonia_pickettii_5_7_47FAA	0	0	0	0	1000	0	0	0	1000	0
Ralstonia_pickettii_52	0	0	1000	0	1000	0	0	0	1000	0
Ralstonia_pickettii_NBRC_102503	0	0	0	0	1000	0	0	0	1000	0
Ralstonia_pickettii_OR214	0	0	0	0	1000	0	0	0	1000	0
Ralstonia_sp_5_2_56FAA	0	0	0	0	1000	0	0	0	0	0
Raoultella_ornithinolytica_10_5246	0	0	1000	0	1000	0	1000	0	0	0
Raoultella_ornithinolytica_2_156_04_S1_C1	0	0	1000	0	1000	0	1000	0	0	0
Raoultella_ornithinolytica_B6	720	0	1000	0	1000	0	1000	0	0	0
Raoultella_ornithinolytica_ornithinolytica_strain_S	0	0	0	0	1000	0	1000	0	0	0
Raoultella_planticola_ATCC_33531	0	0	1000	0	1000	0	1000	0	0	1000
Reyranella_massiliensis_521	0	0	0	0	0	0	0	0	0	0
Reyranella_soli_NBRC_108950	0	0	0	0	0	0	0	0	0	0
Rhizobium_giardinii_bv_giardinii_H152	0	0	1000	0	0	0	0	1000	0	0
Rhizobium_leguminosarum_bv_phaseoli_4292	0	0	1000	0	1000	0	1000	0	0	0
Rhizobium_leguminosarum_bv_phaseoli_CCGM1	0	0	1000	0	1000	0	1000	1000	0	0
Rhizobium_leguminosarum_bv_viciae_WSM1481	0	0	1000	0	1000	0	1000	0	0	0
Rhizobium_leguminosarum_Vaf_108	0	0	1000	0	1000	0	1000	0	0	0
Rhodococcus_equi_103S	0	0	1000	0	1000	0	0	0	1000	0
Rhodococcus_equi_ATCC_33707	0	0	1000	0	1000	0	0	0	1000	1000
Rhodococcus_equi_NBRC_101255	0	0	1000	0	1000	0	0	0	1000	1000
Rhodococcus_erythropolis_BG43	0	0	1000	0	1000	0	1000	0	1000	0
Rhodococcus_erythropolis_PR4	0	0	1000	0	1000	0	0	0	1000	1000
Rhodococcus_erythropolis_SK121	0	0	1000	0	1000	0	1000	0	1000	0
Rhodococcus_erythropolis_XP	0	0	1000	0	1000	0	1000	0	1000	0
Rhodococcus_rhodochrous_ATCC_17895	0	0	1000	0	1000	0	1000	0	1000	0
Rhodococcus_rhodochrous_ATCC_21198	0	0	1000	0	0	0	0	0	1000	1000
Rickettsiella_grylli	0	0	0	0	1000	0	0	0	0	0
Rikenella_microfusus_DSM_15922	0	0	1000	0	0	0	1000	0	0	0
Rikenellaceae_nov_ERR2221101	0	0	0	0	-1.14E-13	0	0	0	0	0
Rikenellaceae_nov_ERR2221110	0	0	0	0	0	0	0	0	0	0
Risungbinella_massiliensis_GD1	0	0	1000	0	1000	0	1000	0	0	0
Robinsoniella_nov_ERR2221391	0	0	0	0	0	0	1000	0	0	0
Robinsoniella_peoriensis_WT	0	0	0	0	0	0	1000	0	0	0
Rodentibacter_pneumotropicus_DSM_21403	0	0	0	0	0	0	1000	0	0	0
Romboutsia_nov_ERR1022375	0	0	0	0	1000	0	1000	0	0	0
Romboutsia_nov_ERR1022465	0	0	0	0	1000	0	1000	0	0	0
Romboutsia_nov_ERR1022466	0	0	0	0	1000	0	1000	0	0	0
Roseburia_faecis_ERR1022321	0	0	0	0	0	0	1000	0	0	0
Roseburia_faecis_ERR2221408	0	0	0	0	0	0	0	0	0	0
Roseburia_faecis_ERR2230155	0	0	0	0	0	0	1000	0	0	0
Roseburia_faecis_M72	0	0	0	0	0	0	1000	0	0	0
Roseburia_hominis_A2_183	0	0	0	1000	1000	0	1000	0	0	0
Roseburia_hominis_ERR1022292	0	0	0	0	0	0	1000	0	0	0
Roseburia_intestinalis_ERR1022474	0	0	0	0	0	0	1000	0	0	0
Roseburia_intestinalis_ERR2221171	0	0	0	0	0	0	1000	0	0	0
Roseburia_intestinalis_ERR2230070	0	0	0	0	0	0	1000	0	0	0
Roseburia_intestinalis_L1_82	0	0	0	1000	0	0	1000	0	0	0
Roseburia_intestinalis_XB6B4	0	0	0	1000	0	0	1000	0	0	0
Roseburia_inulinivorans_DSM_16841	0	0	0	0	1000	0	0	0	0	0

Roseburia_inulinivorans_ERR1022293	0	0	0	0	1000	0	0	0	0	0
Roseburia_inulinivorans_ERR1022345	0	0	0	0	1000	0	0	0	0	0
Roseburia_inulinivorans_ERR1203941	0	0	0	0	1000	0	0	0	0	0
Roseburia_nov_ERR1022344	0	0	0	0	0	0	0	0	0	0
Roseburia_nov_ERR1204035	0	0	0	0	1000	0	0	0	0	0
Roseburia_nov_ERR171282	0	0	0	0	0	0	1000	0	0	0
Roseburia_nov_ERR2221407	0	0	0	0	0	0	0	0	0	0
Roseomonas_cervicalis_ATCC_49957	0	0	0	0	0	0	0	0	0	0
Roseomonas_mucosa_ATCC_BAA_692	0	0	0	0	1000	0	0	0	0	1000
Rothia_aeria_F0474	0	0	0	0	1000	0	0	0	0	0
Rothia_dentocariosa_ATCC_17931	0	0	0	0	1000	0	0	0	0	0
Rothia_dentocariosa_M567	0	0	0	0	1000	0	0	0	0	0
Rothia_mucilaginosa_ATCC_25296	0	0	0	0	1000	0	0	0	0	0
Rothia_mucilaginosa_DY_18	0	0	0	0	1000	0	0	0	0	0
Rothia_mucilaginosa_M508	0	0	0	0	1000	0	0	0	0	0
Rothia_mucilaginosa_NUM_Rm6536	0	0	0	0	1000	0	1000	0	0	0
Rubrobacter_aplysinae_RV113	0	0	1000	0	0	0	0	0	1000	0
Rubrobacter_radiotolerans_DSM_5868	0	0	1000	0	0	0	0	0	0	0
Rubrobacter_xylanophilus_DSM_9941	0	0	1000	0	0	0	0	0	0	0
Rudanella_lutea_DSM_19387	0	0	1000	0	0	0	0	0	0	0
Ruminiclostridium_nov_ERR1022440	0	0	0	0	1000	0	1000	0	0	0
Ruminiclostridium_nov_ERR1022441	0	0	0	0	-1.42E-13	0	0	0	0	0
Ruminiclostridium_nov_ERR2221177	0	0	1000	0	1000	0	1000	0	0	0
Ruminiclostridium_sp_KB18	0	0	0	0	0	0	0	0	0	0
Ruminiclostridium_thermocellum_AD2	0	0	1000	0	0	0	1000	0	0	0
Ruminiclostridium_thermocellum_ATCC_27405	0	0	1000	0	0	0	1000	0	0	0
Ruminiclostridium_thermocellum_DSM_1313	0	0	1000	0	0	0	1000	0	0	0
Ruminiclostridium_thermocellum_DSM_2360	0	0	1000	0	0	0	1000	0	0	0
Ruminococcaceae_bacterium_D16	0	0	0	0	480.8013356	0	1000	0	0	0
Ruminococcaceae_nov_ERR1022299	0	0	0	0	0	0	1000	0	0	0
Ruminococcaceae_nov_ERR1022311	0	0	0	0	110.1395849	0	1000	0	0	0
Ruminococcaceae_nov_ERR1022313	0	0	0	0	0	0	1000	0	0	0
Ruminococcaceae_nov_ERR1022317	0	0	0	0	0	0	1000	0	0	0
Ruminococcaceae_nov_ERR1022336	0	0	1000	0	1000	0	1000	0	0	0
Ruminococcaceae_nov_ERR1022409	0	0	1000	0	1000	0	1000	0	0	0
Ruminococcaceae_nov_ERR1022442	0	0	0	0	0	0	0	0	0	0
Ruminococcaceae_nov_ERR1022443	0	0	0	0	0	0	1000	0	0	0
Ruminococcaceae_nov_ERR1022445	0	0	1000	0	1000	0	1000	0	0	0
Ruminococcaceae_nov_ERR1022448	0	0	0	0	0	0	0	0	0	0
Ruminococcaceae_nov_ERR1022449	0	0	0	0	109.4451988	0	1000	0	0	0
Ruminococcaceae_nov_ERR1022451	0	0	1000	0	1000	0	1000	0	0	0
Ruminococcaceae_nov_ERR1022452	0	0	0	0	67.37073685	0	0	0	0	0
Ruminococcaceae_nov_ERR1022483	0	0	0	0	0	0	1000	0	0	0
Ruminococcaceae_nov_ERR1203965	0	0	1000	0	1000	0	1000	0	0	0
Ruminococcaceae_nov_ERR1204059	0	0	1000	0	1000	0	1000	0	0	0
Ruminococcaceae_nov_ERR2221149	0	0	0	0	1000	0	1000	0	0	0
Ruminococcaceae_nov_ERR2221180	0	0	0	0	100.4070744	0	0	0	0	0
Ruminococcaceae_nov_ERR2221184	0	0	0	0	0	0	1000	0	0	0
Ruminococcaceae_nov_ERR2221393	0	0	0	0	1000	0	1000	0	0	0
Ruminococcaceae_nov_ERR2221397	0	0	0	0	100.406655	0	0	0	0	0
Ruminococcaceae_nov_ERR2230059	0	0	0	0	67.53928467	0	0	0	0	0
Ruminococcaceae_nov_ERR2230068	0	0	0	0	1000	0	1000	0	0	0
Ruminococcus_albus_7	0	0	0	1000	0	0	1000	0	0	0
Ruminococcus_albus_8	0	0	0	1000	0	0	0	0	0	0
Ruminococcus_bicirculans_80_3	0	0	0	0	782.6086957	0	0	0	0	0
Ruminococcus_bicirculans_ERR1022350	0	0	0	0	119.4851214	0	0	0	0	0
Ruminococcus_bicirculans_ERR1022408	0	0	0	0	127.23157	0	0	0	0	0
Ruminococcus_bicirculans_ERR1022485	0	0	0	0	119.4569672	0	0	0	0	0
Ruminococcus_bicirculans_ERR2221172	0	0	0	0	119.4570565	0	0	0	0	0
Ruminococcus_bicirculans_ERR2230101	0	0	0	0	119.4840102	0	0	0	0	0
Ruminococcus_bromii_ATCC_27255	0	0	0	0	0	0	0	0	0	0
Ruminococcus_bromii_ERR1022486	0	0	0	0	0	0	0	0	0	0
Ruminococcus_bromii_ERR2221220	0	0	0	0	0	0	0	0	0	0
Ruminococcus_bromii_L2_63	0	0	0	0	0	0	0	0	0	0
Ruminococcus_callidus_ATCC_2776001	0	0	0	0	0	0	0	0	0	0
Ruminococcus_champanellensis_18P13	0	0	0	1000	0	0	1000	0	0	0
Ruminococcus_faecis_ERR1022432	0	0	0	0	1000	0	0	0	0	0
Ruminococcus_flavefaciens_ATCC_19208	0	0	0	0	0	0	0	0	0	0
Ruminococcus_flavefaciens_FD_1	0	0	0	1000	0	0	0	0	0	0
Ruminococcus_gauvreauii_DSM_19829	0	0	1000	0	1000	0	1000	0	0	0
Ruminococcus_lactaris_ATCC_29176	0	0	0	0	0	0	0	1000	0	0
Ruminococcus_nov_ERR2230055	0	0	0	0	67.5395783	0	0	0	0	0
Ruminococcus_sp_5_1_39BFAA	0	0	0	0	1000	0	0	0	0	0
Ruminococcus_sp_SR1_5	0	0	0	0	1000	0	0	0	0	0
Ruthenibacterium_lactatiformans_585_1	0	0	0	0	0	0	1000	0	0	0
Ruthenibacterium_lactatiformans_668	0	0	0	0	0	0	1000	0	0	0
Saccharolobus_solfataricus_P2	0	0	0	1000	1000	0	0	0	0	0
Salinibacterium_amurskyense_DSM_16400	0	0	1000	0	0	0	0	0	0	0
Salmonella_bongori_BCW_1556	0	0	1000	0	1000	0	1000	0	0	0
Salmonella_bongori_N268_08	0	0	1000	0	1000	0	1000	0	0	0
Salmonella_bongori_NCTC_12419	1000	0	1000	0	1000	0	1000	0	0	0
Salmonella_bongori_serovar_44_z39_	0	0	1000	0	1000	0	1000	0	0	0
Salmonella_bongori_serovar_66_z41_str_SA1998	0	0	1000	0	1000	0	1000	0	0	0
Salmonella_enterica_enterica_sv_Typhimurium_L	1000	0	0	0	1000	0	1000	0	1000	0
Salmonella_enterica_subsp_arizonae_serovar_62	1000	0	1000	0	1000	0	0	0	1000	0
Salmonella_enterica_subsp_enterica_serovar_451	0	0	1000	0	1000	0	1000	0	1000	0
Salmonella_enterica_subsp_enterica_serovar_Ag	0	0	1000	0	1000	0	1000	0	1000	0
Salmonella_enterica_subsp_enterica_serovar_Ag	0	0	1000	0	1000	0	1000	0	1000	0
Salmonella_enterica_subsp_enterica_serovar_Ag	0	0	1000	0	1000	0	1000	0	1000	0
Salmonella_enterica_subsp_enterica_serovar_Ag	0	0	1000	0	1000	0	1000	0	1000	0
Salmonella_enterica_subsp_enterica_serovar_Ag	0	0	1000	0	1000	0	1000	0	1000	0
Salmonella_enterica_subsp_enterica_serovar_Ag	0	0	1000	0	1000	0	1000	0	1000	0
Salmonella_enterica_subsp_enterica_serovar_Ag	0	0	1000	0	1000	0	1000	0	1000	0
Salmonella_enterica_subsp_enterica_serovar_Ag	0	0	1000	0	1000	0	1000	0	1000	0

Salmonella_enterica_subsp_enterica_serovar_Ag	0	0	1000	0	1000	0	1000	0	1000	0
Salmonella_enterica_subsp_enterica_serovar_Ag	0	0	1000	0	1000	0	1000	0	1000	0
Salmonella_enterica_subsp_enterica_serovar_Ag	0	0	1000	0	1000	0	1000	0	1000	0
Salmonella_enterica_subsp_enterica_serovar_Ag	0	0	1000	0	1000	0	1000	0	1000	0
Salmonella_enterica_subsp_enterica_serovar_Ag	0	0	1000	0	1000	0	1000	0	1000	0
Salmonella_enterica_subsp_enterica_serovar_Ag	0	0	1000	0	1000	0	1000	0	1000	0
Salmonella_enterica_subsp_enterica_serovar_Ag	0	0	1000	0	1000	0	1000	0	1000	0
Salmonella_enterica_subsp_enterica_serovar_Ag	0	0	1000	0	1000	0	1000	0	1000	0
Salmonella_enterica_subsp_enterica_serovar_Ag	0	0	1000	0	1000	0	1000	0	1000	0
Salmonella_enterica_subsp_enterica_serovar_Ag	0	0	1000	0	1000	0	1000	0	1000	0
Salmonella_enterica_subsp_enterica_serovar_Ag	0	0	1000	0	1000	0	1000	0	1000	0
Salmonella_enterica_subsp_enterica_serovar_Ag	0	0	1000	0	1000	0	1000	0	1000	0
Salmonella_enterica_subsp_enterica_serovar_Ag	0	0	1000	0	1000	0	1000	0	1000	0
Salmonella_enterica_subsp_enterica_serovar_Ag	0	0	1000	0	1000	0	1000	0	1000	0
Salmonella_enterica_subsp_enterica_serovar_Ag	0	0	1000	0	1000	0	1000	0	1000	0
Salmonella_enterica_subsp_enterica_serovar_Ag	0	0	1000	0	1000	0	1000	0	1000	0
Salmonella_enterica_subsp_enterica_serovar_Ag	0	0	1000	0	1000	0	1000	0	1000	0
Salmonella_enterica_subsp_enterica_serovar_Ag	0	0	1000	0	1000	0	1000	0	1000	0
Salmonella_enterica_subsp_enterica_serovar_Ag	0	0	1000	0	1000	0	1000	0	1000	0
Salmonella_enterica_subsp_enterica_serovar_Ag	0	0	1000	0	1000	0	1000	0	1000	0
Salmonella_enterica_subsp_enterica_serovar_Ag	0	0	1000	0	1000	0	1000	0	1000	0
Salmonella_enterica_subsp_enterica_serovar_Ag	0	0	1000	0	1000	0	1000	0	1000	0
Salmonella_enterica_subsp_enterica_serovar_Ag	0	0	1000	0	1000	0	1000	0	1000	0
Salmonella_enterica_subsp_enterica_serovar_Ag	0	0	1000	0	1000	0	1000	0	1000	0
Salmonella_enterica_subsp_enterica_serovar_Ag	0	0	1000	0	1000	0	1000	0	1000	0
Salmonella_enterica_subsp_enterica_serovar_Ag	0	0	1000	0	1000	0	1000	0	1000	0
Salmonella_enterica_subsp_enterica_serovar_Ag	0	0	1000	0	1000	0	1000	0	1000	0
Salmonella_enterica_subsp_enterica_serovar_Ag	0	0	1000	0	1000	0	1000	0	1000	0
Salmonella_enterica_subsp_enterica_serovar_Ag	0	0	1000	0	1000	0	1000	0	1000	0
Salmonella_enterica_subsp_enterica_serovar_Ag	0	0	1000	0	1000	0	1000	0	1000	0
Salmonella_enterica_subsp_enterica_serovar_Ag	0	0	1000	0	1000	0	1000	0	1000	0
Salmonella_enterica_subsp_enterica_serovar_Ag	0	0	1000	0	1000	0	1000	0	1000	0
Salmonella_enterica_subsp_enterica_serovar_Ag	0	0	1000	0	1000	0	1000	0	1000	0
Salmonella_enterica_subsp_enterica_serovar_Ag	0	0	1000	0	1000	0	1000	0	1000	0
Salmonella_enterica_subsp_enterica_serovar_Ag	0	0	1000	0	1000	0	1000	0	1000	0
Salmonella_enterica_subsp_enterica_serovar_Ag	0	0	1000	0	1000	0	1000	0	1000	0
Salmonella_enterica_subsp_enterica_serovar_Ag	0	0	1000	0	1000	0	1000	0	1000	0
Salmonella_enterica_subsp_enterica_serovar_Ag	0	0	1000	0	1000	0	1000	0	1000	0
Salmonella_enterica_subsp_enterica_serovar_Ag	0	0	1000	0	1000	0	1000	0	1000	0
Salmonella_enterica_subsp_enterica_serovar_Ag	0	0	1000	0	1000	0	1000	0	1000	0
Salmonella_enterica_subsp_enterica_serovar_Ag	0	0	1000	0	1000	0	1000	0	1000	0
Salmonella_enterica_subsp_enterica_serovar_Ag	0	0	1000	0	1000	0	1000	0	1000	0
Salmonella_enterica_subsp_enterica_serovar_Ag	0	0	1000	0	1000	0	1000	0	1000	0
Salmonella_enterica_subsp_enterica_serovar_Ag	0	0	1000	0	1000	0	1000	0	1000	0
Salmonella_enterica_subsp_enterica_serovar_Ag	0	0	1000	0	1000	0	1000	0	1000	0
Salmonella_enterica_subsp_enterica_serovar_Ag	0	0	1000	0	1000	0	1000	0	1000	0
Salmonella_enterica_subsp_enterica_serovar_Ag	0	0	1000	0	1000	0	1000	0	1000	0
Salmonella_enterica_subsp_enterica_serovar_Ag	0	0	1000	0	1000	0	1000	0	1000	0
Salmonella_enterica_subsp_enterica_serovar_Ag	0	0	1000	0	1000	0	1000	0	1000	0
Salmonella_enterica_subsp_enterica_serovar_Ag	0	0	1000	0	1000	0	1000	0	1000	0
Salmonella_enterica_subsp_enterica_serovar_Ag	0	0	1000	0	1000	0	1000	0	1000	0
Salmonella_enterica_subsp_enterica_serovar_Ag	0	0	1000	0	1000	0	1000	0	1000	0
Salmonella_enterica_subsp_enterica_serovar_Ag	0	0	1000	0	1000	0	1000	0	1000	0
Salmonella_enterica_subsp_enterica_serovar_Ag	0	0	1000	0	1000	0	1000	0	1000	0
Salmonella_enterica_subsp_enterica_serovar_Ag	0	0	1000	0	1000	0	1000	0	1000	0
Salmonella_enterica_subsp_enterica_serovar_Ag	0	0	1000	0	1000	0	1000	0	1000	0
Salmonella_enterica_subsp_enterica_serovar_Ag	0	0	1000	0	1000	0	1000	0	1000	0
Salmonella_enterica_subsp_enterica_serovar_Ag	0	0	1000	0	1000	0	1000	0	1000	0
Salmonella_enterica_subsp_enterica_serovar_Ag	0	0	1000	0	1000	0	1000	0	1000	0
Salmonella_enterica_subsp_enterica_serovar_Ag	0	0	1000	0	1000	0	1000	0	1000	0
Salmonella_enterica_subsp_enterica_serovar_Ag	0	0	1000	0	1000	0	1000	0	1000	0
Salmonella_enterica_subsp_enterica_serovar_Ag	0	0	1000	0	1000	0	1000	0	1000	0
Salmonella_enterica_subsp_enterica_serovar_Ag	0	0	0	0	1000	0	1000	0	1000	0
Salmonella_enterica_subsp_enterica_serovar_Ag	1000	0	1000	0	1000	0	1000	0	1000	0
Salmonella_enterica_subsp_enterica_serovar_Ana	0	0	1000	0	1000	0	1000	0	1000	0
Salmonella_enterica_subsp_enterica_serovar_Bar	0	0	1000	0	1000	0	1000	0	1000	0
Salmonella_enterica_subsp_enterica_serovar_Bar	0	0	1000	0	1000	0	1000	0	1000	0
Salmonella_enterica_subsp_enterica_serovar_Cho	0	0	1000	0	1000	0	1000	0	1000	0
Salmonella_enterica_subsp_enterica_serovar_Cho	0	0	1000	0	1000	0	1000	0	1000	0
Salmonella_enterica_subsp_enterica_serovar_Dul	0	0	1000	0	1000	0	1000	0	1000	0
Salmonella_enterica_subsp_enterica_serovar_Dul	1000	0	1000	0	1000	0	1000	0	1000	0
Salmonella_enterica_subsp_enterica_serovar_Dul	0	0	1000	0	1000	0	1000	0	1000	0
Salmonella_enterica_subsp_enterica_serovar_Dul	0	0	1000	0	1000	0	1000	0	1000	0
Salmonella_enterica_subsp_enterica_serovar_Dul	0	0	1000	0	1000	0	1000	0	1000	0
Salmonella_enterica_subsp_enterica_serovar_Ent	0	0	1000	0	1000	0	1000	0	1000	0
Salmonella_enterica_subsp_enterica_serovar_Ent	0	0	1000	0	1000	0	1000	0	1000	0
Salmonella_enterica_subsp_enterica_serovar_Ent	0	0	1000	0	1000	0	1000	0	1000	0
Salmonella_enterica_subsp_enterica_serovar_Ent	0	0	1000	0	1000	0	1000	0	1000	0
Salmonella_enterica_subsp_enterica_serovar_Ent	0	0	1000	0	1000	0	1000	0	1000	0
Salmonella_enterica_subsp_enterica_serovar_Ent	0	0	1000	0	1000	0	1000	0	1000	0
Salmonella_enterica_subsp_enterica_serovar_Ent	0	0	1000	0	1000	0	1000	0	1000	0
Salmonella_enterica_subsp_enterica_serovar_Ent	0	0	1000	0	1000	0	1000	0	1000	0
Salmonella_enterica_subsp_enterica_serovar_Ent	0	0	1000	0	1000	0	1000	0	1000	0
Salmonella_enterica_subsp_enterica_serovar_Ent	0	0	1000	0	1000	0	1000	0	1000	0
Salmonella_enterica_subsp_enterica_serovar_Ent	0	0	1000	0	1000	0	1000	0	1000	0
Salmonella_enterica_subsp_enterica_serovar_Ent	0	0	1000	0	1000	0	1000	0	1000	0
Salmonella_enterica_subsp_enterica_serovar_Ent	0	0	1000	0	1000	0	1000	0	1000	0
Salmonella_enterica_subsp_enterica_serovar_Ent	0	0	1000	0	1000	0	1000	0	1000	0
Salmonella_enterica_subsp_enterica_serovar_Ent	0	0	1000	0	1000	0	1000	0	1000	0
Salmonella_enterica_subsp_enterica_serovar_Ent	0	0	1000	0	1000	0	0	0	1000	0
Salmonella_enterica_subsp_enterica_serovar_Ent	0	0	1000	0	1000	0	0	0	1000	0
Salmonella_enterica_subsp_enterica_serovar_Ent	0	0	1000	0	1000	0	1000	0	1000	0
Salmonella_enterica_subsp_enterica_serovar_Ent	0	0	1000	0	1000	0	1000	0	1000	0
Salmonella_enterica_subsp_enterica_serovar_Ent	0	0	1000	0	1000	0	1000	0	1000	0

Salmonella_enterica_subsp_enterica_serovar_Ent	0	0	1000	0	1000	0	1000	0	1000	0
Salmonella_enterica_subsp_enterica_serovar_Ent	0	0	1000	0	1000	0	1000	0	1000	0
Salmonella_enterica_subsp_enterica_serovar_Ent	0	0	1000	0	1000	0	1000	0	1000	0
Salmonella_enterica_subsp_enterica_serovar_Ent	0	0	1000	0	1000	0	1000	0	1000	0
Salmonella_enterica_subsp_enterica_serovar_Ent	0	0	1000	0	1000	0	1000	0	1000	0
Salmonella_enterica_subsp_enterica_serovar_Ent	0	0	1000	0	1000	0	1000	0	1000	0
Salmonella_enterica_subsp_enterica_serovar_Ent	0	0	1000	0	1000	0	1000	0	1000	0
Salmonella_enterica_subsp_enterica_serovar_Ent	0	0	1000	0	1000	0	1000	0	1000	0
Salmonella_enterica_subsp_enterica_serovar_Ent	0	0	1000	0	1000	0	1000	0	1000	0
Salmonella_enterica_subsp_enterica_serovar_Ent	0	0	1000	0	1000	0	1000	0	1000	0
Salmonella_enterica_subsp_enterica_serovar_Ent	0	0	1000	0	1000	0	1000	0	1000	0
Salmonella_enterica_subsp_enterica_serovar_Ent	0	0	1000	0	1000	0	1000	0	1000	0
Salmonella_enterica_subsp_enterica_serovar_Ent	0	0	1000	0	1000	0	1000	0	1000	0
Salmonella_enterica_subsp_enterica_serovar_Ent	0	0	1000	0	1000	0	1000	0	1000	0
Salmonella_enterica_subsp_enterica_serovar_Ent	0	0	1000	0	1000	0	1000	0	1000	0
Salmonella_enterica_subsp_enterica_serovar_Ent	0	0	1000	0	1000	0	1000	0	1000	0
Salmonella_enterica_subsp_enterica_serovar_Ent	0	0	1000	0	1000	0	1000	0	1000	0
Salmonella_enterica_subsp_enterica_serovar_Ent	0	0	1000	0	1000	0	1000	0	1000	0
Salmonella_enterica_subsp_enterica_serovar_Ent	0	0	1000	0	1000	0	1000	0	1000	0
Salmonella_enterica_subsp_enterica_serovar_Ent	0	0	1000	0	1000	0	1000	0	1000	0
Salmonella_enterica_subsp_enterica_serovar_Ent	0	0	1000	0	1000	0	1000	0	1000	0
Salmonella_enterica_subsp_enterica_serovar_Ent	0	0	1000	0	1000	0	1000	0	1000	0
Salmonella_enterica_subsp_enterica_serovar_Ent	0	0	1000	0	1000	0	1000	0	1000	0
Salmonella_enterica_subsp_enterica_serovar_Ent	0	0	1000	0	1000	0	1000	0	1000	0
Salmonella_enterica_subsp_enterica_serovar_Ent	0	0	1000	0	1000	0	1000	0	1000	0
Salmonella_enterica_subsp_enterica_serovar_Ent	0	0	1000	0	1000	0	1000	0	1000	0
Salmonella_enterica_subsp_enterica_serovar_Ent	0	0	1000	0	1000	0	1000	0	1000	0
Salmonella_enterica_subsp_enterica_serovar_Ent	0	0	1000	0	1000	0	1000	0	1000	0
Salmonella_enterica_subsp_enterica_serovar_Ent	0	0	1000	0	1000	0	1000	0	1000	0
Salmonella_enterica_subsp_enterica_serovar_Ent	0	0	1000	0	1000	0	1000	0	1000	0
Salmonella_enterica_subsp_enterica_serovar_Ent	0	0	1000	0	1000	0	1000	0	1000	0
Salmonella_enterica_subsp_enterica_serovar_Ent	0	0	1000	0	1000	0	1000	0	1000	0
Salmonella_enterica_subsp_enterica_serovar_Ent	0	0	1000	0	1000	0	1000	0	1000	0
Salmonella_enterica_subsp_enterica_serovar_Ent	0	0	1000	0	1000	0	1000	0	1000	0
Salmonella_enterica_subsp_enterica_serovar_Ent	0	0	1000	0	1000	0	1000	0	1000	0
Salmonella_enterica_subsp_enterica_serovar_Ent	0	0	1000	0	1000	0	1000	0	1000	0
Salmonella_enterica_subsp_enterica_serovar_Ent	0	0	1000	0	1000	0	1000	0	1000	0
Salmonella_enterica_subsp_enterica_serovar_Ent	0	0	1000	0	1000	0	1000	0	1000	0
Salmonella_enterica_subsp_enterica_serovar_Ent	0	0	1000	0	1000	0	1000	0	1000	0
Salmonella_enterica_subsp_enterica_serovar_Ent	0	0	1000	0	1000	0	1000	0	1000	0
Salmonella_enterica_subsp_enterica_serovar_Ent	0	0	1000	0	1000	0	1000	0	1000	0
Salmonella_enterica_subsp_enterica_serovar_Ent	0	0	1000	0	1000	0	1000	0	1000	0
Salmonella_enterica_subsp_enterica_serovar_Ent	0	0	1000	0	1000	0	1000	0	1000	0
Salmonella_enterica_subsp_enterica_serovar_Ent	0	0	1000	0	1000	0	1000	0	1000	0
Salmonella_enterica_subsp_enterica_serovar_Ent	0	0	1000	0	1000	0	1000	0	1000	0
Salmonella_enterica_subsp_enterica_serovar_Ent	0	0	1000	0	1000	0	1000	0	1000	0
Salmonella_enterica_subsp_enterica_serovar_Ent	0	0	1000	0	1000	0	1000	0	1000	0
Salmonella_enterica_subsp_enterica_serovar_Ent	0	0	1000	0	1000	0	1000	0	1000	0
Salmonella_enterica_subsp_enterica_serovar_Ent	0	0	1000	0	1000	0	1000	0	1000	0
Salmonella_enterica_subsp_enterica_serovar_Ent	0	0	1000	0	1000	0	1000	0	1000	0
Salmonella_enterica_subsp_enterica_serovar_Ent	0	0	1000	0	1000	0	1000	0	1000	0
Salmonella_enterica_subsp_enterica_serovar_Ent	0	0	1000	0	1000	0	1000	0	1000	0
Salmonella_enterica_subsp_enterica_serovar_Ent	0	0	1000	0	1000	0	1000	0	1000	0
Salmonella_enterica_subsp_enterica_serovar_Ent	0	0	1000	0	1000	0	1000	0	1000	0
Salmonella_enterica_subsp_enterica_serovar_Ent	0	0	1000	0	1000	0	1000	0	1000	0
Salmonella_enterica_subsp_enterica_serovar_Ent	0	0	1000	0	1000	0	1000	0	1000	0
Salmonella_enterica_subsp_enterica_serovar_Ent	0	0	1000	0	1000	0	1000	0	1000	0
Salmonella_enterica_subsp_enterica_serovar_Ent	0	0	1000	0	1000	0	1000	0	1000	0
Salmonella_enterica_subsp_enterica_serovar_Ent	0	0	1000	0	1000	0	1000	0	1000	0
Salmonella_enterica_subsp_enterica_serovar_Ent	0	0	1000	0	1000	0	1000	0	1000	0
Salmonella_enterica_subsp_enterica_serovar_Ent	0	0	1000	0	1000	0	1000	0	1000	0
Salmonella_enterica_subsp_enterica_serovar_Ent	0	0	1000	0	1000	0	1000	0	1000	0
Salmonella_enterica_subsp_enterica_serovar_Ent	0	0	1000	0	1000	0	1000	0	1000	0
Salmonella_enterica_subsp_enterica_serovar_Ent	0	0	1000	0	1000	0	1000	0	1000	0
Salmonella_enterica_subsp_enterica_serovar_Ent	0	0	1000	0	1000	0	1000	0	1000	0
Salmonella_enterica_subsp_enterica_serovar_Ent	0	0	1000	0	1000	0	1000	0	1000	0
Salmonella_enterica_subsp_enterica_serovar_Ent	0	0	1000	0	1000	0	1000	0	1000	0
Salmonella_enterica_subsp_enterica_serovar_Ent	0	0	1000	0	1000	0	1000	0	1000	0
Salmonella_enterica_subsp_enterica_serovar_Ent	0	0	1000	0	1000	0	1000	0	1000	0
Salmonella_enterica_subsp_enterica_serovar_Ent	0	0	1000	0	1000	0	1000	0	1000	0
Salmonella_enterica_subsp_enterica_serovar_Ent	0	0	1000	0	1000	0	1000	0	1000	0
Salmonella_enterica_subsp_enterica_serovar_Ent	0	0	1000	0	1000	0	1000	0	1000	0
Salmonella_enterica_subsp_enterica_serovar_Ent	0	0	1000	0	1000	0	1000	0	1000	0
Salmonella_enterica_subsp_enterica_serovar_Ent	0	0	1000	0	1000	0	1000	0	1000	0
Salmonella_enterica_subsp_enterica_serovar_Ent	0	0	1000	0	1000	0	1000	0	1000	0
Salmonella_enterica_subsp_enterica_serovar_Ent	0	0	1000	0	1000	0	1000	0	1000	0
Salmonella_enterica_subsp_enterica_serovar_Ent	0	0	1000	0	1000	0	1000	0	1000	0
Salmonella_enterica_subsp_enterica_serovar_Ent	0	0	1000	0	1000	0	1000	0	1000	0
Salmonella_enterica_subsp_enterica_serovar_Ent	0	0	1000	0	1000	0	1000	0	1000	0
Salmonella_enterica_subsp_enterica_serovar_Ent	0	0	1000	0	1000	0	1000	0	1000	0
Salmonella_enterica_subsp_enterica_serovar_Ent	0	0	1000	0	1000	0	1000	0	1000	0
Salmonella_enterica_subsp_enterica_serovar_Ent	0	0	1000	0	1000	0	1000	0	1000	0
Salmonella_enterica_subsp_enterica_serovar_Ent	0	0	1000	0	1000	0	1000	0	1000	0
Salmonella_enterica_subsp_enterica_serovar_Ent	0	0	1000	0	1000	0	1000	0	1000	0
Salmonella_enterica_subsp_enterica_serovar_Ent	0	0	1000	0	1000	0	1000	0	1000	0
Salmonella_enterica_subsp_enterica_serovar_Ent	0	0	1000	0	1000	0	1000	0	1000	0
Salmonella_enterica_subsp_enterica_serovar_Gal	1000	0	1000	0	1000	0	1000	0	1000	0
Salmonella_enterica_subsp_enterica_serovar_Gal	1000	0	1000	0	1000	0	1000	0	1000	0
Salmonella_enterica_subsp_enterica_serovar_Gal	0	0	1000	0	1000	0	1000	0	1000	0
Salmonella_enterica_subsp_enterica_serovar_Gal	0	0	1000	0	1000	0	1000	0	1000	0
Salmonella_enterica_subsp_enterica_serovar_Had	0	0	1000	0	1000	0	1000	0	1000	0
Salmonella_enterica_subsp_enterica_serovar_Had	0	0	1000	0	1000	0	1000	0	1000	0
Salmonella_enterica_subsp_enterica_serovar_Had	0	0	1000	0	1000	0	1000	0	1000	0
Salmonella_enterica_subsp_enterica_serovar_Har	0	0	1000	0	1000	0	1000	0	1000	0

Salmonella_enterica_subsp_enterica_serovar_Har	0	0	1000	0	1000	0	1000	0	1000	0
Salmonella_enterica_subsp_enterica_serovar_Hei	0	0	1000	0	1000	0	1000	0	1000	0
Salmonella_enterica_subsp_enterica_serovar_Hei	0	0	1000	0	1000	0	1000	0	1000	0
Salmonella_enterica_subsp_enterica_serovar_Hei	0	0	1000	0	1000	0	1000	0	1000	0
Salmonella_enterica_subsp_enterica_serovar_Hei	0	0	1000	0	1000	0	1000	0	1000	0
Salmonella_enterica_subsp_enterica_serovar_Hei	0	0	1000	0	1000	0	1000	0	1000	0
Salmonella_enterica_subsp_enterica_serovar_Hei	1000	0	1000	0	1000	0	1000	0	1000	0
Salmonella_enterica_subsp_enterica_serovar_Hei	0	0	1000	0	1000	0	1000	0	1000	0
Salmonella_enterica_subsp_enterica_serovar_Hei	0	0	1000	0	1000	0	1000	0	1000	0
Salmonella_enterica_subsp_enterica_serovar_Hei	0	0	1000	0	1000	0	1000	0	1000	0
Salmonella_enterica_subsp_enterica_serovar_Hei	0	0	1000	0	1000	0	1000	0	1000	0
Salmonella_enterica_subsp_enterica_serovar_Hei	1000	0	1000	0	1000	0	1000	0	1000	0
Salmonella_enterica_subsp_enterica_serovar_Hei	0	0	1000	0	1000	0	1000	0	1000	0
Salmonella_enterica_subsp_enterica_serovar_Infa	0	0	1000	1000	1000	0	1000	0	1000	0
Salmonella_enterica_subsp_enterica_serovar_Jav	0	0	1000	1000	1000	0	1000	0	1000	0
Salmonella_enterica_subsp_enterica_serovar_Jav	0	0	1000	1000	1000	0	1000	0	1000	0
Salmonella_enterica_subsp_enterica_serovar_Ker	0	0	1000	0	1000	0	1000	0	1000	0
Salmonella_enterica_subsp_enterica_serovar_Ker	0	0	1000	0	1000	0	1000	0	1000	0
Salmonella_enterica_subsp_enterica_serovar_Mb	0	0	1000	0	1000	0	1000	0	1000	0
Salmonella_enterica_subsp_enterica_serovar_Mis	0	0	1000	0	1000	0	1000	0	1000	0
Salmonella_enterica_subsp_enterica_serovar_Mo	0	0	1000	1000	1000	0	1000	0	1000	0
Salmonella_enterica_subsp_enterica_serovar_Mo	0	0	1000	1000	1000	0	1000	0	1000	0
Salmonella_enterica_subsp_enterica_serovar_Mo	0	0	1000	1000	1000	0	1000	0	1000	0
Salmonella_enterica_subsp_enterica_serovar_Mo	0	0	1000	1000	1000	0	1000	0	1000	0
Salmonella_enterica_subsp_enterica_serovar_Mo	0	0	1000	1000	1000	0	1000	0	1000	0
Salmonella_enterica_subsp_enterica_serovar_Mo	0	0	1000	1000	1000	0	1000	0	1000	0
Salmonella_enterica_subsp_enterica_serovar_Mo	0	0	1000	1000	1000	0	1000	0	1000	0
Salmonella_enterica_subsp_enterica_serovar_Mo	0	0	1000	1000	1000	0	1000	0	1000	0
Salmonella_enterica_subsp_enterica_serovar_Mo	0	0	1000	1000	1000	0	1000	0	1000	0
Salmonella_enterica_subsp_enterica_serovar_Mo	0	0	1000	1000	1000	0	1000	0	1000	0
Salmonella_enterica_subsp_enterica_serovar_Mo	0	0	1000	1000	1000	0	1000	0	1000	0
Salmonella_enterica_subsp_enterica_serovar_Mo	0	0	1000	1000	1000	0	1000	0	1000	0
Salmonella_enterica_subsp_enterica_serovar_Mo	0	0	1000	1000	1000	0	1000	0	1000	0
Salmonella_enterica_subsp_enterica_serovar_Mo	0	0	1000	1000	1000	0	1000	0	1000	0
Salmonella_enterica_subsp_enterica_serovar_Mo	0	0	1000	1000	1000	0	1000	0	1000	0
Salmonella_enterica_subsp_enterica_serovar_Mo	0	0	1000	1000	1000	0	1000	0	1000	0
Salmonella_enterica_subsp_enterica_serovar_Mo	0	0	1000	1000	1000	0	1000	0	1000	0
Salmonella_enterica_subsp_enterica_serovar_Mo	0	0	1000	1000	1000	0	1000	0	1000	0
Salmonella_enterica_subsp_enterica_serovar_Mo	0	0	1000	1000	1000	0	1000	0	1000	0
Salmonella_enterica_subsp_enterica_serovar_Mo	0	0	1000	1000	1000	0	1000	0	1000	0
Salmonella_enterica_subsp_enterica_serovar_Mo	0	0	1000	1000	1000	0	1000	0	1000	0
Salmonella_enterica_subsp_enterica_serovar_Mu	0	0	1000	0	1000	0	1000	0	1000	0
Salmonella_enterica_subsp_enterica_serovar_Mu	0	0	1000	0	1000	0	1000	0	1000	0
Salmonella_enterica_subsp_enterica_serovar_Nev	0	0	1000	0	1000	0	1000	0	1000	0
Salmonella_enterica_subsp_enterica_serovar_Nev	0	0	1000	0	1000	0	1000	0	1000	0
Salmonella_enterica_subsp_enterica_serovar_Nev	0	0	1000	0	1000	0	1000	0	1000	0
Salmonella_enterica_subsp_enterica_serovar_Nev	0	0	1000	0	1000	0	1000	0	1000	0
Salmonella_enterica_subsp_enterica_serovar_Nev	0	0	1000	0	1000	0	1000	0	1000	0
Salmonella_enterica_subsp_enterica_serovar_Nev	0	0	1000	0	1000	0	1000	0	1000	0
Salmonella_enterica_subsp_enterica_serovar_Nev	0	0	1000	0	1000	0	1000	0	1000	0
Salmonella_enterica_subsp_enterica_serovar_Nev	0	0	1000	0	1000	0	1000	0	1000	0
Salmonella_enterica_subsp_enterica_serovar_Nev	0	0	1000	0	1000	0	1000	0	1000	0
Salmonella_enterica_subsp_enterica_serovar_Nev	0	0	1000	0	1000	0	1000	0	1000	0
Salmonella_enterica_subsp_enterica_serovar_Nev	0	0	1000	0	1000	0	1000	1000	1000	0
Salmonella_enterica_subsp_enterica_serovar_Nev	0	0	1000	0	1000	0	1000	0	1000	0
Salmonella_enterica_subsp_enterica_serovar_Nev	0	0	1000	0	1000	0	1000	0	1000	0
Salmonella_enterica_subsp_enterica_serovar_Nev	0	0	1000	0	1000	0	1000	0	1000	0
Salmonella_enterica_subsp_enterica_serovar_Nev	0	0	1000	0	1000	0	1000	0	1000	0
Salmonella_enterica_subsp_enterica_serovar_Nev	0	0	1000	0	1000	0	1000	0	1000	0
Salmonella_enterica_subsp_enterica_serovar_Nev	0	0	1000	0	1000	0	1000	0	1000	0
Salmonella_enterica_subsp_enterica_serovar_Nev	0	0	1000	0	1000	0	1000	0	1000	0
Salmonella_enterica_subsp_enterica_serovar_Nev	0	0	1000	0	1000	0	1000	0	1000	0
Salmonella_enterica_subsp_enterica_serovar_Nev	0	0	1000	0	1000	0	1000	0	1000	0
Salmonella_enterica_subsp_enterica_serovar_Nev	0	0	1000	0	1000	0	1000	0	1000	0
Salmonella_enterica_subsp_enterica_serovar_Nev	0	0	1000	0	1000	0	1000	0	1000	0
Salmonella_enterica_subsp_enterica_serovar_Nev	0	0	1000	0	1000	0	1000	0	1000	0
Salmonella_enterica_subsp_enterica_serovar_Nev	0	0	1000	0	1000	0	1000	0	1000	0
Salmonella_enterica_subsp_enterica_serovar_Nev	0	0	1000	0	1000	0	1000	0	1000	0
Salmonella_enterica_subsp_enterica_serovar_Nev	0	0	1000	0	1000	0	1000	0	1000	0
Salmonella_enterica_subsp_enterica_serovar_Nev	0	0	1000	0	1000	0	1000	0	1000	0
Salmonella_enterica_subsp_enterica_serovar_Nev	0	0	1000	0	1000	0	1000	0	1000	0
Salmonella_enterica_subsp_enterica_serovar_Nev	0	0	1000	0	1000	0	1000	0	1000	0
Salmonella_enterica_subsp_enterica_serovar_Nev	1000	0	1000	0	1000	0	1000	0	1000	0
Salmonella_enterica_subsp_enterica_serovar_Nev	0	0	1000	0	1000	0	1000	0	1000	0
Salmonella_enterica_subsp_enterica_serovar_Par	1000	0	1000	0	1000	0	1000	0	1000	0
Salmonella_enterica_subsp_enterica_serovar_Par	0	0	1000	0	1000	0	1000	0	1000	0
Salmonella_enterica_subsp_enterica_serovar_Par	0	0	1000	0	1000	0	1000	0	1000	0
Salmonella_enterica_subsp_enterica_serovar_Par	0	0	1000	0	1000	0	1000	0	1000	0
Salmonella_enterica_subsp_enterica_serovar_Par	0	0	1000	0	1000	0	1000	0	1000	0
Salmonella_enterica_subsp_enterica_serovar_Par	0	0	1000	0	1000	0	0	0	1000	0
Salmonella_enterica_subsp_enterica_serovar_Par	1000	0	1000	0	1000	0	1000	0	1000	0
Salmonella_enterica_subsp_enterica_serovar_Par	1000	0	1000	0	1000	0	1000	0	1000	0
Salmonella_enterica_subsp_enterica_serovar_Por	0	0	1000	1000	1000	0	1000	0	1000	0
Salmonella_enterica_subsp_enterica_serovar_Poo	0	0	1000	1000	1000	0	1000	0	1000	0
Salmonella_enterica_subsp_enterica_serovar_Sai	0	0	1000	0	1000	0	1000	0	1000	0
Salmonella_enterica_subsp_enterica_serovar_Sch	1000	0	1000	1000	1000	0	1000	0	1000	0
Salmonella_enterica_subsp_enterica_serovar_Sch	0	0	1000	1000	1000	0	1000	0	1000	0
Salmonella_enterica_subsp_enterica_serovar_Ser	0	0	1000	0	1000	0	1000	0	1000	0
Salmonella_enterica_subsp_enterica_serovar_Sta	0	0	1000	0	1000	0	1000	0	1000	0
Salmonella_enterica_subsp_enterica_serovar_Ter	0	0	1000	0	1000	0	1000	0	1000	0
Salmonella_enterica_subsp_enterica_serovar_Tho	0	0	1000	0	1000	0	1000	0	1000	0
Salmonella_enterica_subsp_enterica_serovar_Typ	0	0	1000	0	1000	0	1000	0	1000	0
Salmonella_enterica_subsp_enterica_serovar_Typ	0	0	1000	0	1000	0	1000	0	1000	0
Salmonella_enterica_subsp_enterica_serovar_Typ	1000	0	1000	0	1000	0	1000	0	1000	0

Salmonella_enterica_subsp_enterica_serovar_Typ	0	0	1000	0	0	0	1000	0	1000	0
Salmonella_enterica_subsp_enterica_serovar_Typ	1000	0	1000	0	1000	0	1000	0	1000	0
Salmonella_enterica_subsp_enterica_serovar_Typ	0	0	1000	0	1000	0	1000	0	1000	0
Salmonella_enterica_subsp_enterica_serovar_Typ	1000	0	1000	0	1000	0	1000	0	1000	0
Salmonella_enterica_subsp_enterica_serovar_Typ	1000	0	1000	0	1000	0	1000	0	1000	0
Salmonella_enterica_subsp_enterica_serovar_Typ	0	0	1000	0	1000	0	1000	0	1000	0
Salmonella_enterica_subsp_enterica_serovar_Typ	1000	0	1000	0	1000	0	1000	0	1000	0
Salmonella_enterica_subsp_enterica_serovar_Typ	0	0	1000	0	1000	0	1000	0	1000	0
Salmonella_enterica_subsp_enterica_serovar_Typ	1000	0	1000	0	1000	0	1000	0	1000	0
Salmonella_enterica_subsp_enterica_serovar_Typ	1000	0	1000	0	1000	0	1000	0	1000	0
Salmonella_enterica_subsp_enterica_serovar_Typ	0	0	1000	0	1000	0	1000	0	1000	0
Salmonella_enterica_subsp_enterica_serovar_Typ	0	0	1000	0	1000	0	1000	0	1000	0
Salmonella_enterica_subsp_enterica_serovar_Typ	0	0	1000	0	1000	0	1000	0	1000	0
Salmonella_enterica_subsp_enterica_serovar_Typ	0	0	1000	0	1000	0	1000	0	1000	0
Salmonella_enterica_subsp_enterica_serovar_Typ	0	0	1000	0	1000	0	1000	0	1000	0
Salmonella_enterica_subsp_enterica_serovar_Typ	0	0	1000	0	1000	0	1000	0	1000	0
Salmonella_enterica_subsp_enterica_serovar_Typ	0	0	1000	0	1000	0	1000	0	1000	0
Salmonella_enterica_subsp_enterica_serovar_Typ	0	0	1000	0	1000	0	1000	0	1000	0
Salmonella_enterica_subsp_enterica_serovar_Typ	0	0	1000	0	1000	0	1000	0	1000	0
Salmonella_enterica_subsp_enterica_serovar_Typ	0	0	1000	0	1000	0	1000	0	1000	0
Salmonella_enterica_subsp_enterica_serovar_Typ	0	0	1000	0	1000	0	1000	0	1000	0
Salmonella_enterica_subsp_enterica_serovar_Typ	1000	0	1000	0	1000	0	1000	0	1000	0
Salmonella_enterica_subsp_enterica_serovar_Typ	0	0	1000	0	1000	0	1000	0	1000	0
Salmonella_enterica_subsp_enterica_serovar_Typ	1000	0	1000	0	1000	0	1000	0	1000	0
Salmonella_enterica_subsp_enterica_serovar_Typ	1000	0	1000	0	1000	0	1000	0	1000	0
Salmonella_enterica_subsp_enterica_serovar_Vir	0	0	1000	0	1000	0	1000	0	1000	0
Salmonella_enterica_subsp_enterica_serovar_We	0	0	1000	0	1000	0	1000	0	1000	0
Salmonella_enterica_subsp_houtenae_serovar_16	0	0	1000	0	1000	0	1000	0	1000	0
Salmonella_enterica_subsp_houtenae_str_ATCC_	0	0	1000	0	1000	0	1000	0	1000	0
Salmonella_enterica_subsp_salamae_str_3588_07	0	0	1000	0	1000	0	1000	0	1000	0
Sarcina_nov_ERR1022285	0	0	1000	0	1000	0	1000	0	0	0
Sarcina_nov_ERR1022316	0	0	1000	0	1000	0	1000	0	0	0
Sarcina_nov_ERR1022347	0	0	0	0	1000	0	1000	0	0	0
Sarcina_nov_ERR1022371	0	0	1000	0	1000	0	1000	0	0	0
Sarcina_nov_ERR1022372	0	0	0	0	1000	0	1000	0	0	0
Sarcina_nov_ERR1022421	0	0	0	0	1000	0	1000	0	0	0
Sarcina_nov_ERR1022468	0	0	1000	0	1000	0	1000	0	0	0
Sarcina_nov_ERR1203922	0	0	0	0	1000	0	1000	0	0	0
Sarcina_nov_ERR1203929	0	0	0	0	1000	0	1000	0	0	0
Sarcina_nov_ERR1203932	0	0	0	0	1000	0	1000	0	0	0
Sarcina_nov_ERR1204023	0	0	0	0	1000	0	1000	0	0	0
Sarcina_nov_ERR1204026	0	0	0	0	1000	0	1000	0	0	0
Sarcina_nov_ERR171255	0	0	0	0	1000	0	1000	0	0	0
Sarcina_nov_ERR171264	0	0	1000	0	1000	0	1000	0	0	0
Sarcina_nov_ERR171266	0	0	1000	0	1000	0	1000	0	0	0
Sarcina_nov_ERR171270	0	0	0	0	1000	0	1000	0	0	0
Sarcina_nov_ERR2221152	0	0	0	0	1000	0	1000	0	0	0
Sarcina_nov_ERR2221229	0	0	0	0	1000	0	1000	0	0	0
Sarcina_nov_ERR2221238	0	0	0	0	0	0	1000	0	0	0
Sarcina_nov_ERR2221241	0	0	0	0	1000	0	1000	0	0	0
Sarcina_nov_ERR2221248	0	0	0	0	1000	0	1000	0	0	0
Sarcina_nov_ERR2221284	0	0	0	0	0	0	1000	0	0	0
Sarcina_nov_ERR2221290	0	0	0	0	1000	0	1000	0	0	0
Sarcina_nov_ERR2221381	0	0	0	0	1000	0	1000	0	0	0
Sarcina_nov_ERR2230135	0	0	0	0	1000	0	1000	0	0	0
Sarcina_nov_ERR2230149	0	0	0	0	1000	0	1000	0	0	0
Sarcina_ventriculi_ERR1022374	0	0	0	0	0	0	1000	0	0	0
Scardovia_inopinata_F0304	0	0	0	0	1000	0	1000	0	0	1000
Scardovia_wiggsiae_F0424	0	0	0	0	0	0	1000	0	0	0
Schlesneria_paludicola_DSM_18645	0	0	1000	1000	1000	0	0	0	0	1000
Schwartzia_succinivorans_DSM_10502	0	0	1000	0	1000	0	1000	0	0	0
Sediminibacterium_salmoneum_NBRC_103935	0	0	0	0	1000	0	0	0	0	0
Selenomonas_bovis_8_14_1	0	0	0	0	1000	0	0	0	0	0
Selenomonas_bovis_DSM_23594	0	0	0	0	1000	0	0	0	0	0
Selenomonas_flueggei_ATCC_43531	0	0	0	0	1000	0	1000	0	0	0
Selenomonas_infelix_ATCC_43532	0	0	0	0	0	0	1000	0	0	0
Selenomonas_noxia_ATCC_43541	0	0	0	0	0	0	1000	0	0	0
Selenomonas_noxia_F0398	0	0	0	0	0	0	1000	0	0	0
Selenomonas_ruminantium_AC2024	0	0	1000	0	1000	0	1000	0	0	0
Selenomonas_ruminantium_L14	0	0	1000	0	1000	0	1000	0	0	0
Selenomonas_ruminantium_subsp_ruminantium_	0	0	1000	0	1000	0	1000	0	0	0
Selenomonas_sp_F0473	0	0	0	0	0	0	1000	0	0	0
Selenomonas_sputigena_ATCC_35185	250	0	0	0	1000	0	1000	0	0	0
Sellimonas_intestinalis_BR72	0	0	0	0	0	0	1000	0	0	0
Senegalimassilia_anaerobia_JC110	0	0	0	0	1000	0	1000	0	0	0
Serratia_fonticola_GS2	0	1000	0	0	1000	0	1000	0	0	0
Serratia_fonticola_RB_25	0	1000	0	0	1000	0	1000	0	0	1000
Serratia_liquefaciens_ATCC_27592	1000	0	1000	0	1000	0	1000	0	0	1000
Serratia_liquefaciens_FDAARGOS_125	0	0	1000	0	1000	0	1000	0	0	0
Serratia_marcescens_LCT_SM213	0	0	1000	0	1000	0	1000	0	0	0
Serratia_marcescens_subsp_marcescens_Db11	0	1000	1000	0	1000	0	1000	0	0	1000
Serratia_marcescens_UCI88	0	1000	1000	0	1000	0	1000	0	0	0
Serratia_marcescens_WW4	1000	0	1000	0	1000	0	1000	0	0	0
Serratia_odorifera_DSM_4582	0	1000	1000	0	1000	0	1000	0	0	0
Serratia_rubidaea_NBRC_103169	0	0	1000	0	1000	0	1000	0	0	0
Serratia_rubidaea_NCTC10848	0	0	1000	0	1000	0	1000	0	0	0
Serratia_sp_FGI94	0	0	1000	0	1000	0	1000	0	0	0
Shewanella_halifaxensis_HAW_EB4	0	0	0	0	0	0	1000	0	1000	0
Shewanella_woodyi_ATCC_51908	0	0	1000	0	1000	0	1000	0	0	0
Shigella_dysenteriae_155_74	0	0	1000	0	1000	0	1000	0	0	0
Shigella_dysenteriae_1617	833.3333333	0	1000	1000	0	0	1000	0	0	0
Shigella_dysenteriae_225_75	0	0	1000	1000	1000	0	1000	0	0	0
Shigella_dysenteriae_CDC_74_1112	0	0	1000	1000	1000	0	1000	0	0	0
Shigella_dysenteriae_Sd197	1000	0	0	1000	1000	0	1000	0	0	1000

Shigella_flexneri_1485_80	0	0	1000	1000	1000	0	1000	0	0	0
Shigella_flexneri_2002017	1000	0	0	1000	1000	0	1000	0	0	1000
Shigella_flexneri_2747_71	0	0	1000	1000	1000	0	1000	0	0	0
Shigella_flexneri_2850_71	0	0	1000	1000	0	0	1000	0	0	0
Shigella_flexneri_2930_71	0	0	1000	1000	1000	0	1000	0	0	0
Shigella_flexneri_2a_str_2457T	800	0	1000	1000	1000	0	1000	0	0	0
Shigella_flexneri_2a_str_301	800	0	1000	1000	0	0	1000	0	0	0
Shigella_flexneri_2a_strain_BS1025	0	0	1000	0	1000	0	1000	0	0	0
Shigella_flexneri_4343_70	0	0	1000	1000	1000	0	1000	0	0	0
Shigella_flexneri_5_str_8401	1000	0	1000	1000	1000	0	1000	0	0	0
Shigella_flexneri_5a_str_M90T	0	0	1000	1000	1000	0	1000	0	0	0
Shigella_flexneri_6603_63	0	0	1000	1000	0	0	1000	0	0	0
Shigella_flexneri_CCH060	0	0	1000	1000	1000	0	1000	0	0	0
Shigella_flexneri_CDC_796_83	0	0	1000	1000	1000	0	1000	0	0	0
Shigella_flexneri_K_218	0	0	1000	1000	1000	0	1000	0	0	0
Shigella_flexneri_K_227	0	0	1000	1000	0	0	1000	0	0	0
Shigella_flexneri_K_272	0	0	1000	1000	0	0	1000	0	0	0
Shigella_flexneri_K_304	0	0	1000	1000	1000	0	1000	0	0	0
Shigella_flexneri_K_315	0	0	1000	1000	1000	0	1000	0	0	0
Shigella_flexneri_SFJ17B	0	0	1000	1000	0	0	1000	0	0	0
Shigella_flexneri_VA_6	0	0	1000	1000	1000	0	1000	0	0	0
Shigella_sonnei_3226_85	0	0	1000	0	1000	0	1000	0	0	0
Shigella_sonnei_3233_85	0	0	1000	1000	1000	0	1000	0	0	0
Shigella_sonnei_4822_66	0	0	1000	1000	1000	0	1000	0	0	0
Shigella_sonnei_53G	888.8888889	0	1000	0	1000	0	1000	0	0	0
Shigella_sonnei_FDAARGOS_128	0	0	1000	0	1000	0	1000	0	0	0
Shigella_sonnei_FDAARGOS_71	0	0	1000	0	1000	0	1000	0	0	0
Shigella_sonnei_Ss046	1000	0	0	1000	1000	0	1000	0	0	1000
Shigella_sonnei_str_Moseley	0	0	1000	1000	1000	0	1000	0	0	0
Shuttleworthia_satelles_DSM_14600	0	0	0	1000	1000	0	1000	0	0	0
Silanimonas_lenta_DSM_16282	0	0	0	0	1000	0	0	0	0	1000
Sinobacterium_caligoides_DSM_100316	0	0	1000	0	1000	0	1000	0	0	0
Slackia_equolifaciens_DSM_24851	0	0	1000	0	1000	0	1000	0	0	0
Slackia_exigua_ATCC_700122	0	0	0	0	1000	0	0	0	0	0
Slackia_faecicanis_DSM_17537	0	0	1000	0	1000	0	1000	0	0	0
Slackia_piriformis_YIT_12062	0	0	0	0	1000	0	0	0	0	0
Sneathia_sanguinegens_CCUG41628	0	0	0	0	1000	0	1000	0	0	0
Solobacterium_moorei_DSM_22971	0	0	0	0	0	0	1000	0	0	1000
Solobacterium_moorei_F0204	0	0	0	0	0	0	1000	0	0	1000
Sphingobium_amiense_NBRC_102518	0	0	0	0	1000	0	0	0	0	0
Sphingomonas_paucimobilis_HER1398	0	0	1000	0	1000	0	1000	0	0	1000
Sphingosinicella_microcystinivorans_B9	0	0	0	0	1000	0	1000	0	0	0
Sphingosinicella_microcystinivorans_DSM_19791	0	0	0	0	1000	0	1000	0	0	0
Spiroplasma_culicicola_AES_1	0	0	0	0	0	0	1000	0	0	0
Spirosoma_linguale_DSM_74	0	0	1000	0	0	0	0	0	0	0
Sporobacter_termitidis_DSM_10068	0	0	0	0	0	0	0	0	0	0
Staphylococcus_arlettae_CVD059	0	0	1000	1000	1000	0	1000	0	0	0
Staphylococcus_aureus_04_02981	0	0	1000	0	1000	0	1000	0	0	0
Staphylococcus_aureus_08BA02176	0	0	1000	0	1000	0	1000	0	0	0
Staphylococcus_aureus_16K	0	0	1000	0	1000	0	1000	0	0	0
Staphylococcus_aureus_3957	0	0	1000	0	1000	0	1000	0	0	0
Staphylococcus_aureus_930918_3	0	0	1000	0	1000	0	1000	0	0	0
Staphylococcus_aureus_A10102	0	0	1000	0	1000	0	1000	0	0	0
Staphylococcus_aureus_A5937	0	0	1000	0	1000	0	1000	0	0	0
Staphylococcus_aureus_A5948	0	0	1000	0	1000	0	1000	0	0	0
Staphylococcus_aureus_A6224	0	0	1000	0	1000	0	1000	0	0	0
Staphylococcus_aureus_A6300	0	0	1000	0	1000	0	1000	0	0	0
Staphylococcus_aureus_A8115	0	0	1000	0	1000	0	1000	0	0	0
Staphylococcus_aureus_A8117	0	0	1000	0	1000	0	1000	0	0	0
Staphylococcus_aureus_A8796	0	0	1000	0	1000	0	1000	0	0	0
Staphylococcus_aureus_A8819	0	0	1000	0	1000	0	1000	0	0	0
Staphylococcus_aureus_A9299	0	0	1000	0	1000	0	1000	0	0	0
Staphylococcus_aureus_A9635	0	0	1000	0	1000	0	1000	0	0	0
Staphylococcus_aureus_A9719	0	0	1000	0	1000	0	1000	0	0	0
Staphylococcus_aureus_A9754	0	0	1000	0	1000	0	1000	0	0	0
Staphylococcus_aureus_A9765	0	0	1000	0	1000	0	1000	0	0	0
Staphylococcus_aureus_A9781	0	0	1000	0	1000	0	1000	0	0	0
Staphylococcus_aureus_B147830	0	0	1000	0	1000	0	1000	0	0	0
Staphylococcus_aureus_B40723	0	0	1000	0	1000	0	1000	0	0	0
Staphylococcus_aureus_B40950	0	0	1000	0	1000	0	1000	0	0	0
Staphylococcus_aureus_B53639	0	0	1000	0	1000	0	1000	0	0	0
Staphylococcus_aureus_CA_347	0	0	1000	0	1000	0	1000	0	0	0
Staphylococcus_aureus_CN79	0	0	1000	0	1000	0	1000	0	0	0
Staphylococcus_aureus_D30	0	0	1000	0	1000	0	1000	0	0	0
Staphylococcus_aureus_HI010	0	0	1000	0	1000	0	1000	0	0	0
Staphylococcus_aureus_HI010B	0	0	1000	0	1000	0	1000	0	0	0
Staphylococcus_aureus_HI013	0	0	1000	0	1000	0	1000	0	0	0
Staphylococcus_aureus_HI022	0	0	1000	0	1000	0	1000	0	0	0
Staphylococcus_aureus_HI049	0	0	1000	0	1000	0	1000	0	0	0
Staphylococcus_aureus_HI049B	0	0	1000	0	1000	0	1000	0	0	0
Staphylococcus_aureus_HI049C	0	0	1000	0	1000	0	1000	0	0	0
Staphylococcus_aureus_HI111	0	0	1000	0	1000	0	1000	0	0	0
Staphylococcus_aureus_HI168	0	0	1000	0	1000	0	1000	0	0	0
Staphylococcus_aureus_HIF003_B2N_C	0	0	1000	0	1000	0	1000	0	0	0
Staphylococcus_aureus_KLT6	0	0	1000	0	1000	0	1000	0	0	0
Staphylococcus_aureus_KT_314250	0	0	1000	0	1000	0	1000	0	0	0
Staphylococcus_aureus_KT_Y21	0	0	1000	0	1000	0	1000	0	0	0
Staphylococcus_aureus_LVP2	0	0	1000	0	1000	0	1000	0	0	0
Staphylococcus_aureus_LVP5	0	0	1000	0	1000	0	1000	0	0	0
Staphylococcus_aureus_M0001	0	0	1000	0	1000	0	1000	0	0	0
Staphylococcus_aureus_M0006	0	0	1000	0	1000	0	1000	0	0	0
Staphylococcus_aureus_M0029	0	0	1000	0	1000	0	1000	0	0	0
Staphylococcus_aureus_M0035	0	0	1000	0	1000	0	1000	0	0	0
Staphylococcus_aureus_M0045	0	0	1000	0	1000	0	1000	0	0	0

Staphylococcus_aureus_M0055	0	0	1000	0	1000	0	1000	0	0	0
Staphylococcus_aureus_M0060	0	0	1000	0	1000	0	1000	0	0	0
Staphylococcus_aureus_M0066	0	0	1000	0	1000	0	1000	0	0	0
Staphylococcus_aureus_M0075	0	0	1000	0	1000	0	1000	0	0	0
Staphylococcus_aureus_M0077	0	0	1000	0	1000	0	1000	0	0	0
Staphylococcus_aureus_M0102	0	0	1000	0	1000	0	1000	0	0	0
Staphylococcus_aureus_M0103	0	0	1000	0	1000	0	1000	0	0	0
Staphylococcus_aureus_M0104	0	0	1000	0	1000	0	1000	0	0	0
Staphylococcus_aureus_M0108	0	0	1000	0	1000	0	1000	0	0	0
Staphylococcus_aureus_M0144	0	0	1000	0	1000	0	1000	0	0	0
Staphylococcus_aureus_M0150	0	0	1000	0	1000	0	1000	0	0	0
Staphylococcus_aureus_M0154	0	0	1000	0	1000	0	1000	0	0	0
Staphylococcus_aureus_M0171	0	0	1000	0	1000	0	1000	0	0	0
Staphylococcus_aureus_M0173	0	0	1000	0	1000	0	1000	0	0	0
Staphylococcus_aureus_M0177	0	0	1000	0	1000	0	1000	0	0	0
Staphylococcus_aureus_M0192	0	0	1000	0	1000	0	1000	0	0	0
Staphylococcus_aureus_M0197	0	0	1000	0	1000	0	1000	0	0	0
Staphylococcus_aureus_M0200	0	0	1000	0	1000	0	1000	0	0	0
Staphylococcus_aureus_M0210	0	0	1000	0	1000	0	1000	0	0	0
Staphylococcus_aureus_M0212	0	0	1000	0	1000	0	1000	0	0	0
Staphylococcus_aureus_M0213	0	0	1000	0	1000	0	1000	0	0	0
Staphylococcus_aureus_M0216	0	0	1000	0	1000	0	1000	0	0	0
Staphylococcus_aureus_M0221	0	0	1000	0	1000	0	1000	0	0	0
Staphylococcus_aureus_M0235	0	0	1000	0	1000	0	1000	0	0	0
Staphylococcus_aureus_M0237	0	0	1000	0	1000	0	1000	0	0	0
Staphylococcus_aureus_M0239	0	0	1000	0	1000	0	1000	0	0	0
Staphylococcus_aureus_M0240	0	0	1000	0	1000	0	1000	0	0	0
Staphylococcus_aureus_M0250	0	0	1000	0	1000	0	1000	0	0	0
Staphylococcus_aureus_M0252	0	0	1000	0	1000	0	1000	0	0	0
Staphylococcus_aureus_M0270	0	0	1000	0	1000	0	1000	0	0	0
Staphylococcus_aureus_M0273	0	0	1000	0	1000	0	1000	0	0	0
Staphylococcus_aureus_M0279	0	0	1000	0	1000	0	1000	0	0	0
Staphylococcus_aureus_M0280	0	0	1000	0	1000	0	1000	0	0	0
Staphylococcus_aureus_M0288	0	0	1000	0	1000	0	1000	0	0	0
Staphylococcus_aureus_M0294	0	0	1000	0	1000	0	1000	0	0	0
Staphylococcus_aureus_M0306	0	0	1000	0	1000	0	1000	0	0	0
Staphylococcus_aureus_M0312	0	0	1000	0	1000	0	1000	0	0	0
Staphylococcus_aureus_M0326	0	0	1000	0	1000	0	1000	0	0	0
Staphylococcus_aureus_M0328	0	0	1000	0	1000	0	1000	0	0	0
Staphylococcus_aureus_M0329	0	0	1000	0	1000	0	1000	0	0	0
Staphylococcus_aureus_M0330	0	0	1000	0	1000	0	1000	0	0	0
Staphylococcus_aureus_M0334	0	0	1000	0	1000	0	1000	0	0	0
Staphylococcus_aureus_M0340	0	0	1000	0	1000	0	1000	0	0	0
Staphylococcus_aureus_M0347	0	0	1000	0	1000	0	1000	0	0	0
Staphylococcus_aureus_M0350	0	0	1000	0	1000	0	1000	0	0	0
Staphylococcus_aureus_M0351	0	0	1000	0	1000	0	1000	0	0	0
Staphylococcus_aureus_M0363	0	0	1000	0	1000	0	1000	0	0	0
Staphylococcus_aureus_M0364	0	0	1000	0	1000	0	1000	0	0	0
Staphylococcus_aureus_M0367	0	0	1000	0	1000	0	1000	0	0	0
Staphylococcus_aureus_M0374	0	0	1000	0	1000	0	1000	0	0	0
Staphylococcus_aureus_M0375	0	0	1000	0	1000	0	1000	0	0	0
Staphylococcus_aureus_M0391	0	0	1000	0	1000	0	1000	0	0	0
Staphylococcus_aureus_M0396	0	0	1000	0	1000	0	1000	0	0	0
Staphylococcus_aureus_M0404	0	0	1000	0	1000	0	1000	0	0	0
Staphylococcus_aureus_M0408	0	0	1000	0	1000	0	1000	0	0	0
Staphylococcus_aureus_M0415	0	0	1000	0	1000	0	1000	0	0	0
Staphylococcus_aureus_M0424	0	0	1000	0	1000	0	1000	0	0	0
Staphylococcus_aureus_M0427	0	0	1000	0	1000	0	1000	0	0	0
Staphylococcus_aureus_M0438	0	0	1000	0	1000	0	1000	0	0	0
Staphylococcus_aureus_M0450	0	0	1000	0	1000	0	1000	0	0	0
Staphylococcus_aureus_M0455	0	0	1000	0	1000	0	1000	0	0	0
Staphylococcus_aureus_M0460	0	0	1000	0	1000	0	1000	0	0	0
Staphylococcus_aureus_M0467	0	0	1000	0	1000	0	1000	0	0	0
Staphylococcus_aureus_M0468	0	0	1000	0	1000	0	1000	0	0	0
Staphylococcus_aureus_M0478	0	0	1000	0	1000	0	1000	0	0	0
Staphylococcus_aureus_M0489	0	0	1000	0	1000	0	1000	0	0	0
Staphylococcus_aureus_M0493	0	0	1000	0	1000	0	1000	0	0	0
Staphylococcus_aureus_M0494	0	0	1000	0	1000	0	1000	0	0	0
Staphylococcus_aureus_M0510	0	0	1000	0	1000	0	1000	0	0	0
Staphylococcus_aureus_M0513	0	0	1000	0	1000	0	1000	0	0	0
Staphylococcus_aureus_M0528	0	0	1000	0	1000	0	1000	0	0	0
Staphylococcus_aureus_M0529	0	0	1000	0	1000	0	1000	0	0	0
Staphylococcus_aureus_M0531	0	0	1000	0	1000	0	1000	0	0	0
Staphylococcus_aureus_M0536	0	0	1000	0	1000	0	1000	0	0	0
Staphylococcus_aureus_M0539	0	0	1000	0	1000	0	1000	0	0	0
Staphylococcus_aureus_M0547	0	0	1000	0	1000	0	1000	0	0	0
Staphylococcus_aureus_M0562	0	0	1000	0	1000	0	1000	0	0	0
Staphylococcus_aureus_M0565	0	0	1000	0	1000	0	1000	0	0	0
Staphylococcus_aureus_M0571	0	0	1000	0	1000	0	1000	0	0	0
Staphylococcus_aureus_M0580	0	0	1000	0	1000	0	1000	0	0	0
Staphylococcus_aureus_M0584	0	0	1000	0	1000	0	1000	0	0	0
Staphylococcus_aureus_M0586	0	0	1000	0	1000	0	1000	0	0	0
Staphylococcus_aureus_M0602	0	0	1000	0	1000	0	1000	0	0	0
Staphylococcus_aureus_M0622	0	0	1000	0	1000	0	1000	0	0	0
Staphylococcus_aureus_M0628	0	0	1000	0	1000	0	1000	0	0	0
Staphylococcus_aureus_M0633	0	0	1000	0	1000	0	1000	0	0	0
Staphylococcus_aureus_M0646	0	0	1000	0	1000	0	1000	0	0	0
Staphylococcus_aureus_M0648	0	0	1000	0	1000	0	1000	0	0	0
Staphylococcus_aureus_M0660	0	0	1000	0	1000	0	1000	0	0	0
Staphylococcus_aureus_M0663	0	0	1000	0	1000	0	1000	0	0	0
Staphylococcus_aureus_M0673	0	0	1000	0	1000	0	1000	0	0	0
Staphylococcus_aureus_M0676	0	0	1000	0	1000	0	1000	0	0	0
Staphylococcus_aureus_M0687	0	0	1000	0	1000	0	1000	0	0	0
Staphylococcus_aureus_M0692	0	0	1000	0	1000	0	1000	0	0	0

Staphylococcus_aureus_M0695	0	0	1000	0	1000	0	1000	0	0	0
Staphylococcus_aureus_M0719	0	0	1000	0	1000	0	1000	0	0	0
Staphylococcus_aureus_M0769	0	0	1000	0	1000	0	1000	0	0	0
Staphylococcus_aureus_M0770	0	0	1000	0	1000	0	1000	0	0	0
Staphylococcus_aureus_M0780	0	0	1000	0	1000	0	1000	0	0	0
Staphylococcus_aureus_M0792	0	0	1000	0	1000	0	1000	0	0	0
Staphylococcus_aureus_M0799	0	0	1000	0	1000	0	1000	0	0	0
Staphylococcus_aureus_M0822	0	0	1000	0	1000	0	1000	0	0	0
Staphylococcus_aureus_M0823	0	0	1000	0	1000	0	1000	0	0	0
Staphylococcus_aureus_M0831	0	0	1000	0	1000	0	1000	0	0	0
Staphylococcus_aureus_M0844	0	0	1000	0	1000	0	1000	0	0	0
Staphylococcus_aureus_M0871	0	0	1000	0	1000	0	1000	0	0	0
Staphylococcus_aureus_M0877	0	0	1000	0	1000	0	1000	0	0	0
Staphylococcus_aureus_M0892	0	0	1000	0	1000	0	1000	0	0	0
Staphylococcus_aureus_M0900	0	0	1000	0	1000	0	1000	0	0	0
Staphylococcus_aureus_M0927	0	0	1000	0	1000	0	1000	0	0	0
Staphylococcus_aureus_M0934	0	0	1000	0	1000	0	1000	0	0	0
Staphylococcus_aureus_M0943	0	0	1000	0	1000	0	1000	0	0	0
Staphylococcus_aureus_M0944	0	0	1000	0	1000	0	1000	0	0	0
Staphylococcus_aureus_M0946	0	0	1000	0	1000	0	1000	0	0	0
Staphylococcus_aureus_M0953	0	0	1000	0	1000	0	1000	0	0	0
Staphylococcus_aureus_M0964	0	0	1000	0	1000	0	1000	0	0	0
Staphylococcus_aureus_M0978	0	0	1000	0	1000	0	1000	0	0	0
Staphylococcus_aureus_M0994	0	0	1000	0	1000	0	1000	0	0	0
Staphylococcus_aureus_M0998	0	0	1000	0	1000	0	1000	0	0	0
Staphylococcus_aureus_M0999	0	0	1000	0	1000	0	1000	0	0	0
Staphylococcus_aureus_M1	0	0	1000	0	1000	0	1000	0	0	0
Staphylococcus_aureus_M1007	0	0	1000	0	1000	0	1000	0	0	0
Staphylococcus_aureus_M1010	0	0	1000	0	1000	0	1000	0	0	0
Staphylococcus_aureus_M1015	0	0	1000	0	1000	0	1000	0	0	0
Staphylococcus_aureus_M1016	0	0	1000	0	1000	0	1000	0	0	0
Staphylococcus_aureus_M1034	0	0	1000	0	1000	0	1000	0	0	0
Staphylococcus_aureus_M1036	0	0	1000	0	1000	0	1000	0	0	0
Staphylococcus_aureus_M1037	0	0	1000	0	1000	0	1000	0	0	0
Staphylococcus_aureus_M1044	0	0	1000	0	1000	0	1000	0	0	0
Staphylococcus_aureus_M1060	0	0	1000	0	1000	0	1000	0	0	0
Staphylococcus_aureus_M1061	0	0	1000	0	1000	0	1000	0	0	0
Staphylococcus_aureus_M1062	0	0	1000	0	1000	0	1000	0	0	0
Staphylococcus_aureus_M1063	0	0	1000	0	1000	0	1000	0	0	0
Staphylococcus_aureus_M1064	0	0	1000	0	1000	0	1000	0	0	0
Staphylococcus_aureus_M1068	0	0	1000	0	1000	0	1000	0	0	0
Staphylococcus_aureus_M1076	0	0	1000	0	1000	0	1000	0	0	0
Staphylococcus_aureus_M1078	0	0	1000	0	1000	0	1000	0	0	0
Staphylococcus_aureus_M1083	0	0	1000	0	1000	0	1000	0	0	0
Staphylococcus_aureus_M1092	0	0	1000	0	1000	0	1000	0	0	0
Staphylococcus_aureus_M1093	0	0	1000	0	1000	0	1000	0	0	0
Staphylococcus_aureus_M1095	0	0	1000	0	1000	0	1000	0	0	0
Staphylococcus_aureus_M1103	0	0	1000	0	1000	0	1000	0	0	0
Staphylococcus_aureus_M1109	0	0	1000	0	1000	0	1000	0	0	0
Staphylococcus_aureus_M1119	0	0	1000	0	1000	0	1000	0	0	0
Staphylococcus_aureus_M1126	0	0	1000	0	1000	0	1000	0	0	0
Staphylococcus_aureus_M1142	0	0	1000	0	1000	0	1000	0	0	0
Staphylococcus_aureus_M1167	0	0	1000	0	1000	0	1000	0	0	0
Staphylococcus_aureus_M1170	0	0	1000	0	1000	0	1000	0	0	0
Staphylococcus_aureus_M1188	0	0	1000	0	1000	0	1000	0	0	0
Staphylococcus_aureus_M1193	0	0	1000	0	1000	0	1000	0	0	0
Staphylococcus_aureus_M1198	0	0	1000	0	1000	0	1000	0	0	0
Staphylococcus_aureus_M1199	0	0	1000	0	1000	0	1000	0	0	0
Staphylococcus_aureus_M1215	0	0	1000	0	1000	0	1000	0	0	0
Staphylococcus_aureus_M1216	0	0	1000	0	1000	0	1000	0	0	0
Staphylococcus_aureus_M1223	0	0	1000	0	1000	0	1000	0	0	0
Staphylococcus_aureus_M1224	0	0	1000	0	1000	0	1000	0	0	0
Staphylococcus_aureus_M1228	0	0	1000	0	1000	0	1000	0	0	0
Staphylococcus_aureus_M1229	0	0	1000	0	1000	0	1000	0	0	0
Staphylococcus_aureus_M1244	0	0	1000	0	1000	0	1000	0	0	0
Staphylococcus_aureus_M1248	0	0	1000	0	1000	0	1000	0	0	0
Staphylococcus_aureus_M1253	0	0	1000	0	1000	0	1000	0	0	0
Staphylococcus_aureus_M1255	0	0	1000	0	1000	0	1000	0	0	0
Staphylococcus_aureus_M1256	0	0	1000	0	1000	0	1000	0	0	0
Staphylococcus_aureus_M1257	0	0	1000	0	1000	0	1000	0	0	0
Staphylococcus_aureus_M1275	0	0	1000	0	1000	0	1000	0	0	0
Staphylococcus_aureus_M1277	0	0	1000	0	1000	0	1000	0	0	0
Staphylococcus_aureus_M1286	0	0	1000	0	1000	0	1000	0	0	0
Staphylococcus_aureus_M1291	0	0	1000	0	1000	0	1000	0	0	0
Staphylococcus_aureus_M1309	0	0	1000	0	1000	0	1000	0	0	0
Staphylococcus_aureus_M1311	0	0	1000	0	1000	0	1000	0	0	0
Staphylococcus_aureus_M1320	0	0	1000	0	1000	0	1000	0	0	0
Staphylococcus_aureus_M1321	0	0	1000	0	1000	0	1000	0	0	0
Staphylococcus_aureus_M1322	0	0	1000	0	1000	0	1000	0	0	0
Staphylococcus_aureus_M1359	0	0	1000	0	1000	0	1000	0	0	0
Staphylococcus_aureus_M1367	0	0	1000	0	1000	0	1000	0	0	0
Staphylococcus_aureus_M1373	0	0	1000	0	1000	0	1000	0	0	0
Staphylococcus_aureus_M1374	0	0	1000	0	1000	0	1000	0	0	0
Staphylococcus_aureus_M1394	0	0	1000	0	1000	0	1000	0	0	0
Staphylococcus_aureus_M1405	0	0	1000	0	1000	0	1000	0	0	0
Staphylococcus_aureus_M1407	0	0	1000	0	1000	0	1000	0	0	0
Staphylococcus_aureus_M1450	0	0	1000	0	1000	0	1000	0	0	0
Staphylococcus_aureus_M1451	0	0	1000	0	1000	0	1000	0	0	0
Staphylococcus_aureus_M1462	0	0	1000	0	1000	0	1000	0	0	0
Staphylococcus_aureus_M1463	0	0	1000	0	1000	0	1000	0	0	0
Staphylococcus_aureus_M1466	0	0	1000	0	1000	0	1000	0	0	0
Staphylococcus_aureus_M1481	0	0	1000	0	1000	0	1000	0	0	0
Staphylococcus_aureus_M1510	0	0	1000	0	1000	0	1000	0	0	0
Staphylococcus_aureus_M1520	0	0	1000	0	1000	0	1000	0	0	0

Staphylococcus_aureus_M1521	0	0	1000	0	1000	0	1000	0	0	0
Staphylococcus_aureus_M1531	0	0	1000	0	1000	0	1000	0	0	0
Staphylococcus_aureus_M1533	0	0	1000	0	1000	0	1000	0	0	0
Staphylococcus_aureus_M1544	0	0	1000	0	1000	0	1000	0	0	0
Staphylococcus_aureus_M1556	0	0	1000	0	1000	0	1000	0	0	0
Staphylococcus_aureus_M1563	0	0	1000	0	1000	0	1000	0	0	0
Staphylococcus_aureus_M1565	0	0	1000	0	1000	0	1000	0	0	0
Staphylococcus_aureus_M1578	0	0	1000	0	1000	0	1000	0	0	0
Staphylococcus_aureus_M2	0	0	1000	0	1000	0	1000	0	0	0
Staphylococcus_aureus_NN54	0	0	1000	0	1000	0	1000	0	0	0
Staphylococcus_aureus_O11	0	0	1000	0	1000	0	1000	0	0	0
Staphylococcus_aureus_O46	0	0	1000	0	1000	0	1000	0	0	0
Staphylococcus_aureus_PM1	0	0	1000	0	1000	0	1000	0	0	0
Staphylococcus_aureus_PPUKM_261_2009	0	0	1000	0	1000	0	1000	0	0	0
Staphylococcus_aureus_PPUKM_332_2009	0	0	1000	0	1000	0	1000	0	0	0
Staphylococcus_aureus_PPUKM_377_2009	0	0	1000	0	1000	0	1000	0	0	0
Staphylococcus_aureus_PPUKM_775_2009	0	0	1000	0	1000	0	1000	0	0	0
Staphylococcus_aureus_RF122	0	0	1000	0	1000	0	1000	0	0	0
Staphylococcus_aureus_ST228_10388	0	0	1000	0	1000	0	1000	0	0	0
Staphylococcus_aureus_ST228_10497	0	0	1000	0	1000	0	1000	0	0	0
Staphylococcus_aureus_ST228_15532	0	0	1000	0	1000	0	1000	0	0	0
Staphylococcus_aureus_ST228_16035	0	0	1000	0	1000	0	1000	0	0	0
Staphylococcus_aureus_ST228_18412	0	0	1000	0	1000	0	1000	0	0	0
Staphylococcus_aureus_subsp_aureus_06BA1836	0	0	1000	0	1000	0	1000	0	0	0
Staphylococcus_aureus_subsp_aureus_091751	0	0	1000	0	1000	0	1000	0	0	0
Staphylococcus_aureus_subsp_aureus_103564	0	0	1000	0	1000	0	1000	0	0	0
Staphylococcus_aureus_subsp_aureus_112808A	0	0	1000	0	1000	0	1000	0	0	0
Staphylococcus_aureus_subsp_aureus_118	0	0	1000	0	1000	0	1000	0	0	0
Staphylococcus_aureus_subsp_aureus_11819_97	0	0	1000	0	1000	0	1000	0	0	0
Staphylococcus_aureus_subsp_aureus_120	0	0	1000	0	1000	0	1000	0	0	0
Staphylococcus_aureus_subsp_aureus_122051	0	0	1000	0	1000	0	1000	0	0	0
Staphylococcus_aureus_subsp_aureus_132	0	0	1000	0	1000	0	1000	0	0	0
Staphylococcus_aureus_subsp_aureus_21172	0	0	1000	0	1000	0	1000	0	0	0
Staphylococcus_aureus_subsp_aureus_21178	0	0	1000	0	1000	0	1000	0	0	0
Staphylococcus_aureus_subsp_aureus_21189	0	0	1000	0	1000	0	1000	0	0	0
Staphylococcus_aureus_subsp_aureus_21193	0	0	1000	0	1000	0	1000	0	0	0
Staphylococcus_aureus_subsp_aureus_21195	0	0	1000	0	1000	0	1000	0	0	0
Staphylococcus_aureus_subsp_aureus_21196	0	0	1000	0	1000	0	1000	0	0	0
Staphylococcus_aureus_subsp_aureus_21200	0	0	1000	0	1000	0	1000	0	0	0
Staphylococcus_aureus_subsp_aureus_21201	0	0	1000	0	1000	0	1000	0	0	0
Staphylococcus_aureus_subsp_aureus_21202	0	0	1000	0	1000	0	1000	0	0	0
Staphylococcus_aureus_subsp_aureus_21209	0	0	1000	0	1000	0	1000	0	0	0
Staphylococcus_aureus_subsp_aureus_21232	0	0	1000	0	1000	0	1000	0	0	0
Staphylococcus_aureus_subsp_aureus_21236	0	0	1000	0	1000	0	1000	0	0	0
Staphylococcus_aureus_subsp_aureus_21252	0	0	1000	0	1000	0	1000	0	0	0
Staphylococcus_aureus_subsp_aureus_21259	0	0	1000	0	1000	0	1000	0	0	0
Staphylococcus_aureus_subsp_aureus_21262	0	0	1000	0	1000	0	1000	0	0	0
Staphylococcus_aureus_subsp_aureus_21264	0	0	1000	0	1000	0	1000	0	0	0
Staphylococcus_aureus_subsp_aureus_21266	0	0	1000	0	1000	0	1000	0	0	0
Staphylococcus_aureus_subsp_aureus_21269	0	0	1000	0	1000	0	1000	0	0	0
Staphylococcus_aureus_subsp_aureus_21272	0	0	1000	0	1000	0	1000	0	0	0
Staphylococcus_aureus_subsp_aureus_21282	0	0	1000	0	1000	0	1000	0	0	0
Staphylococcus_aureus_subsp_aureus_21283	0	0	1000	0	1000	0	1000	0	0	0
Staphylococcus_aureus_subsp_aureus_21305	0	0	0	0	1000	0	1000	0	0	0
Staphylococcus_aureus_subsp_aureus_21310	0	0	1000	0	1000	0	1000	0	0	0
Staphylococcus_aureus_subsp_aureus_21318	0	0	1000	0	1000	0	1000	0	0	0
Staphylococcus_aureus_subsp_aureus_21331	0	0	1000	0	1000	0	1000	0	0	0
Staphylococcus_aureus_subsp_aureus_21333	0	0	1000	0	1000	0	1000	0	0	0
Staphylococcus_aureus_subsp_aureus_21334	0	0	1000	0	1000	0	1000	0	0	0
Staphylococcus_aureus_subsp_aureus_21340	0	0	1000	0	1000	0	1000	0	0	0
Staphylococcus_aureus_subsp_aureus_21342	0	0	1000	0	1000	0	1000	0	0	0
Staphylococcus_aureus_subsp_aureus_21343	0	0	1000	0	1000	0	1000	0	0	0
Staphylococcus_aureus_subsp_aureus_21345	0	0	1000	0	1000	0	1000	0	0	0
Staphylococcus_aureus_subsp_aureus_333	0	0	1000	0	1000	0	1000	0	0	0
Staphylococcus_aureus_subsp_aureus_3989	0	0	1000	0	1000	0	1000	0	0	0
Staphylococcus_aureus_subsp_aureus_55_2053	0	0	1000	0	1000	0	1000	0	0	0
Staphylococcus_aureus_subsp_aureus_58_424	0	0	1000	0	1000	0	1000	0	0	0
Staphylococcus_aureus_subsp_aureus_65_1322	0	0	1000	0	1000	0	1000	0	0	0
Staphylococcus_aureus_subsp_aureus_68_397	0	0	1000	0	1000	0	1000	0	0	0
Staphylococcus_aureus_subsp_aureus_71193	0	0	1000	0	1000	0	1000	0	0	0
Staphylococcus_aureus_subsp_aureus_A017934_	0	0	1000	0	1000	0	1000	0	0	0
Staphylococcus_aureus_subsp_aureus_ATCC_518	0	0	1000	0	1000	0	1000	0	0	0
Staphylococcus_aureus_subsp_aureus_ATCC_BAA	0	0	1000	0	1000	0	1000	0	0	0
Staphylococcus_aureus_subsp_aureus_Btn1260	0	0	1000	0	1000	0	1000	0	0	0
Staphylococcus_aureus_subsp_aureus_C101	0	0	1000	0	1000	0	1000	0	0	0
Staphylococcus_aureus_subsp_aureus_C160	0	0	1000	0	1000	0	1000	0	0	0
Staphylococcus_aureus_subsp_aureus_C427	0	0	1000	0	1000	0	1000	0	0	0
Staphylococcus_aureus_subsp_aureus_CBD_635	0	0	1000	0	1000	0	1000	0	0	0
Staphylococcus_aureus_subsp_aureus_CGS00	0	0	1000	0	1000	0	1000	0	0	0
Staphylococcus_aureus_subsp_aureus_CGS01	0	0	1000	0	1000	0	1000	0	0	0
Staphylococcus_aureus_subsp_aureus_CGS03	0	0	1000	0	1000	0	1000	0	0	0
Staphylococcus_aureus_subsp_aureus_CIG1057	0	0	1000	0	1000	0	1000	0	0	0
Staphylococcus_aureus_subsp_aureus_CIG1096	0	0	1000	0	1000	0	1000	0	0	0
Staphylococcus_aureus_subsp_aureus_CIG1114	0	0	1000	0	1000	0	1000	0	0	0
Staphylococcus_aureus_subsp_aureus_CIG1150	0	0	1000	0	1000	0	1000	0	0	0
Staphylococcus_aureus_subsp_aureus_CIG1165	0	0	1000	0	1000	0	1000	0	0	0
Staphylococcus_aureus_subsp_aureus_CIG1176	0	0	1000	0	1000	0	1000	0	0	0
Staphylococcus_aureus_subsp_aureus_CIG1213	0	0	1000	0	1000	0	1000	0	0	0
Staphylococcus_aureus_subsp_aureus_CIG1214	0	0	1000	0	1000	0	1000	0	0	0
Staphylococcus_aureus_subsp_aureus_CIG1233	0	0	1000	0	1000	0	1000	0	0	0
Staphylococcus_aureus_subsp_aureus_CIG1242	0	0	1000	0	1000	0	1000	0	0	0
Staphylococcus_aureus_subsp_aureus_CIG1267	0	0	1000	0	1000	0	1000	0	0	0
Staphylococcus_aureus_subsp_aureus_CIG149	0	0	1000	0	1000	0	1000	0	0	0
Staphylococcus_aureus_subsp_aureus_CIG1500	0	0	1000	0	1000	0	1000	0	0	0

Staphylococcus_aureus_subsp_aureus_CIG1524	0	0	1000	0	1000	0	1000	0	0	0
Staphylococcus_aureus_subsp_aureus_CIG1605	0	0	1000	0	1000	0	1000	0	0	0
Staphylococcus_aureus_subsp_aureus_CIG1612	0	0	1000	0	1000	0	1000	0	0	0
Staphylococcus_aureus_subsp_aureus_CIG1750	0	0	1000	0	1000	0	1000	0	0	0
Staphylococcus_aureus_subsp_aureus_CIG1769	0	0	1000	0	1000	0	1000	0	0	0
Staphylococcus_aureus_subsp_aureus_CIG1770	0	0	1000	0	1000	0	1000	0	0	0
Staphylococcus_aureus_subsp_aureus_CIG1835	0	0	1000	0	1000	0	1000	0	0	0
Staphylococcus_aureus_subsp_aureus_CIG2018	0	0	1000	0	1000	0	1000	0	0	0
Staphylococcus_aureus_subsp_aureus_CIG290	0	0	1000	0	1000	0	1000	0	0	0
Staphylococcus_aureus_subsp_aureus_CIG547	0	0	1000	0	1000	0	1000	0	0	0
Staphylococcus_aureus_subsp_aureus_CIGC128	0	0	1000	0	1000	0	1000	0	0	0
Staphylococcus_aureus_subsp_aureus_CIGC340D	0	0	1000	0	1000	0	1000	0	0	0
Staphylococcus_aureus_subsp_aureus_CIGC341D	0	0	1000	0	1000	0	1000	0	0	0
Staphylococcus_aureus_subsp_aureus_CIGC345D	0	0	1000	0	1000	0	1000	0	0	0
Staphylococcus_aureus_subsp_aureus_CIGC348	0	0	1000	0	1000	0	1000	0	0	0
Staphylococcus_aureus_subsp_aureus_CIGC93	0	0	1000	0	1000	0	1000	0	0	0
Staphylococcus_aureus_subsp_aureus_CM05	0	0	1000	0	1000	0	1000	0	0	0
Staphylococcus_aureus_subsp_aureus_COL	0	0	1000	0	1000	0	1000	0	0	0
Staphylococcus_aureus_subsp_aureus_D139	0	0	1000	0	1000	0	1000	0	0	0
Staphylococcus_aureus_subsp_aureus_DR10	0	0	1000	0	1000	0	1000	0	0	0
Staphylococcus_aureus_subsp_aureus_DSM_202	0	0	1000	0	1000	0	1000	0	0	0
Staphylococcus_aureus_subsp_aureus_E1410	0	0	1000	0	1000	0	1000	0	0	0
Staphylococcus_aureus_subsp_aureus_ECT_R_2	0	0	1000	0	1000	0	1000	0	0	0
Staphylococcus_aureus_subsp_aureus_ED133	0	0	1000	0	1000	0	1000	0	0	0
Staphylococcus_aureus_subsp_aureus_ED98	0	0	1000	0	1000	0	1000	0	0	0
Staphylococcus_aureus_subsp_aureus_EMRSA16	0	0	1000	0	1000	0	1000	0	0	0
Staphylococcus_aureus_subsp_aureus_GR1	0	0	1000	0	1000	0	1000	0	0	0
Staphylococcus_aureus_subsp_aureus_H19	0	0	1000	0	1000	0	1000	0	0	0
Staphylococcus_aureus_subsp_aureus_H29	0	0	1000	0	1000	0	1000	0	0	0
Staphylococcus_aureus_subsp_aureus_HO_5096_	0	0	1000	0	1000	0	1000	0	0	0
Staphylococcus_aureus_subsp_aureus_IS_105	0	0	0	0	1000	0	1000	0	0	0
Staphylococcus_aureus_subsp_aureus_IS_111	0	0	1000	0	1000	0	1000	0	0	0
Staphylococcus_aureus_subsp_aureus_IS_122	0	0	1000	0	1000	0	1000	0	0	0
Staphylococcus_aureus_subsp_aureus_IS_189	0	0	1000	0	1000	0	1000	0	0	0
Staphylococcus_aureus_subsp_aureus_IS_250	0	0	1000	0	1000	0	1000	0	0	0
Staphylococcus_aureus_subsp_aureus_IS_3	0	0	1000	0	1000	0	1000	0	0	0
Staphylococcus_aureus_subsp_aureus_IS_55	0	0	1000	0	1000	0	1000	0	0	0
Staphylococcus_aureus_subsp_aureus_IS_88	0	0	1000	0	1000	0	1000	0	0	0
Staphylococcus_aureus_subsp_aureus_IS_99	0	0	1000	0	1000	0	1000	0	0	0
Staphylococcus_aureus_subsp_aureus_IS_K	0	0	1000	0	1000	0	1000	0	0	0
Staphylococcus_aureus_subsp_aureus_JH1	0	0	1000	0	1000	0	1000	0	0	0
Staphylococcus_aureus_subsp_aureus_JH9	0	0	1000	0	1000	0	1000	0	0	0
Staphylococcus_aureus_subsp_aureus_JKD6159	0	0	1000	0	1000	0	1000	0	0	0
Staphylococcus_aureus_subsp_aureus_LCT_SA11	0	0	1000	0	1000	0	1000	0	0	0
Staphylococcus_aureus_subsp_aureus_LGA251	0	0	1000	0	1000	0	1000	0	0	0
Staphylococcus_aureus_subsp_aureus_M013	0	0	1000	0	1000	0	1000	0	0	0
Staphylococcus_aureus_subsp_aureus_M1015	0	0	1000	0	1000	0	1000	0	0	0
Staphylococcus_aureus_subsp_aureus_M809	0	0	1000	0	1000	0	1000	0	0	0
Staphylococcus_aureus_subsp_aureus_M876	0	0	1000	0	1000	0	1000	0	0	0
Staphylococcus_aureus_subsp_aureus_M899	0	0	1000	0	1000	0	1000	0	0	0
Staphylococcus_aureus_subsp_aureus_MN8	0	0	1000	0	1000	0	1000	0	0	0
Staphylococcus_aureus_subsp_aureus_MRGR3	0	0	1000	0	1000	0	1000	0	0	0
Staphylococcus_aureus_subsp_aureus_MRSA131	0	0	1000	0	1000	0	1000	0	0	0
Staphylococcus_aureus_subsp_aureus_MRSA177	0	0	1000	0	1000	0	1000	0	0	0
Staphylococcus_aureus_subsp_aureus_MRSA252	0	0	1000	0	1000	0	1000	0	0	0
Staphylococcus_aureus_subsp_aureus_MSHR113	0	0	1000	0	1000	0	1000	0	0	0
Staphylococcus_aureus_subsp_aureus_MSSA476	0	0	1000	0	1000	0	1000	0	0	0
Staphylococcus_aureus_subsp_aureus_Mu3	0	0	1000	0	1000	0	1000	0	0	0
Staphylococcus_aureus_subsp_aureus_Mu50	0	0	1000	0	1000	0	1000	0	0	0
Staphylococcus_aureus_subsp_aureus_Mu50_om	0	0	1000	0	1000	0	1000	0	0	0
Staphylococcus_aureus_subsp_aureus_MW2	0	0	1000	0	1000	0	1000	0	0	0
Staphylococcus_aureus_subsp_aureus_N315	0	0	1000	0	1000	0	1000	0	0	0
Staphylococcus_aureus_subsp_aureus_NBRC_100	0	0	1000	0	1000	0	1000	0	0	0
Staphylococcus_aureus_subsp_aureus_NCTC_832	0	0	1000	0	1000	0	1000	0	0	0
Staphylococcus_aureus_subsp_aureus_NN50	0	0	1000	0	1000	0	1000	0	0	0
Staphylococcus_aureus_subsp_aureus_PB32	0	0	1000	0	1000	0	1000	0	0	0
Staphylococcus_aureus_subsp_aureus_RN4220	0	0	1000	0	1000	0	1000	0	0	0
Staphylococcus_aureus_subsp_aureus_str_JKD60	0	0	1000	0	1000	0	1000	0	0	0
Staphylococcus_aureus_subsp_aureus_str_JKD60	0	0	1000	0	1000	0	1000	0	0	0
Staphylococcus_aureus_subsp_aureus_str_Newm	0	0	1000	0	1000	0	1000	0	0	0
Staphylococcus_aureus_subsp_aureus_T0131	0	0	1000	0	1000	0	1000	0	0	0
Staphylococcus_aureus_subsp_aureus_TCH130	0	0	1000	0	1000	0	1000	0	0	0
Staphylococcus_aureus_subsp_aureus_TCH60	0	0	1000	0	1000	0	1000	0	0	0
Staphylococcus_aureus_subsp_aureus_TCH70	0	0	1000	0	1000	0	1000	0	0	0
Staphylococcus_aureus_subsp_aureus_TW20	0	0	1000	0	1000	0	1000	0	0	0
Staphylococcus_aureus_subsp_aureus_USA300	0	0	1000	0	1000	0	1000	0	0	0
Staphylococcus_aureus_subsp_aureus_USA300_F	0	0	0	0	1000	0	1000	0	0	0
Staphylococcus_aureus_subsp_aureus_USA300_T	0	0	1000	0	1000	0	1000	0	0	0
Staphylococcus_aureus_subsp_aureus_USA300_T	0	0	1000	0	1000	0	1000	0	0	0
Staphylococcus_aureus_subsp_aureus_VC40	0	0	1000	0	1000	0	1000	0	0	0
Staphylococcus_aureus_subsp_aureus_VCU006	0	0	1000	0	1000	0	1000	0	0	0
Staphylococcus_aureus_subsp_aureus_VH60	0	0	1000	0	1000	0	1000	0	0	0
Staphylococcus_aureus_subsp_aureus_VRS1	0	0	0	0	1000	0	1000	0	0	0
Staphylococcus_aureus_subsp_aureus_VRS10	0	0	1000	0	1000	0	1000	0	0	0
Staphylococcus_aureus_subsp_aureus_VRS11a	0	0	1000	0	1000	0	1000	0	0	0
Staphylococcus_aureus_subsp_aureus_VRS11b	0	0	1000	0	1000	0	1000	0	0	0
Staphylococcus_aureus_subsp_aureus_VRS2	0	0	1000	0	1000	0	1000	0	0	0
Staphylococcus_aureus_subsp_aureus_VRS3a	0	0	1000	0	1000	0	1000	0	0	0
Staphylococcus_aureus_subsp_aureus_VRS4	0	0	1000	0	1000	0	1000	0	0	0
Staphylococcus_aureus_subsp_aureus_VRS5	0	0	1000	0	1000	0	1000	0	0	0
Staphylococcus_aureus_subsp_aureus_VRS6	0	0	1000	0	1000	0	1000	0	0	0
Staphylococcus_aureus_subsp_aureus_VRS7	0	0	1000	0	1000	0	1000	0	0	0
Staphylococcus_aureus_subsp_aureus_VRS8	0	0	1000	0	1000	0	1000	0	0	0
Staphylococcus_aureus_subsp_aureus_VRS9	0	0	1000	0	1000	0	1000	0	0	0

Staphylococcus_aureus_subsp_aureus_WBG1004	0	0	1000	0	1000	0	1000	0	0	0
Staphylococcus_aureus_subsp_aureus_WW2703_	0	0	1000	0	1000	0	1000	0	0	0
Staphylococcus_aureus_VH221	0	0	1000	0	1000	0	1000	0	0	0
Staphylococcus_capitis_CR0101	0	0	1000	0	1000	0	1000	0	0	0
Staphylococcus_capitis_QN1	0	0	0	0	1000	0	1000	0	0	0
Staphylococcus_capitis_SK14	0	0	1000	0	1000	0	1000	0	0	0
Staphylococcus_capitis_VCU116	0	0	1000	0	1000	0	1000	0	0	0
Staphylococcus_caprae_C87	0	0	1000	0	1000	0	1000	0	0	0
Staphylococcus_cohnii_hu_01	0	0	1000	0	1000	0	1000	0	0	0
Staphylococcus_cohnii_subsp_cohnii_532	0	0	1000	0	1000	0	1000	0	0	0
Staphylococcus_epidermidis_12142587	0	0	1000	0	1000	0	1000	0	0	0
Staphylococcus_epidermidis_14_1_R1_SE	0	0	1000	0	1000	0	1000	0	0	0
Staphylococcus_epidermidis_41tr	0	0	1000	0	1000	0	1000	0	0	0
Staphylococcus_epidermidis_528m	0	0	0	0	0	0	1000	0	0	0
Staphylococcus_epidermidis_A487	0	0	1000	1000	1000	0	1000	0	0	0
Staphylococcus_epidermidis_ATCC_12228	0	0	0	0	1000	0	1000	0	0	0
Staphylococcus_epidermidis_AU12_03	0	0	1000	0	1000	0	1000	0	0	0
Staphylococcus_epidermidis_BCM_HMP0060	0	0	1000	0	1000	0	1000	0	0	0
Staphylococcus_epidermidis_BVS058A4	0	0	1000	0	1000	0	1000	0	0	0
Staphylococcus_epidermidis_ERR2221338	0	0	1000	0	1000	0	1000	0	0	0
Staphylococcus_epidermidis_FRI909	0	0	1000	0	1000	0	1000	0	0	0
Staphylococcus_epidermidis_M0881	0	0	1000	0	1000	0	1000	0	0	0
Staphylococcus_epidermidis_M23864W2grey	0	0	1000	0	1000	0	1000	0	0	0
Staphylococcus_epidermidis_NIH04003	0	0	1000	0	1000	0	1000	0	0	0
Staphylococcus_epidermidis_NIH04008	0	0	1000	0	1000	0	1000	0	0	0
Staphylococcus_epidermidis_NIH05001	0	0	1000	0	1000	0	1000	0	0	0
Staphylococcus_epidermidis_NIH05003	0	0	1000	0	1000	0	1000	0	0	0
Staphylococcus_epidermidis_NIH05005	0	0	1000	0	1000	0	1000	0	0	0
Staphylococcus_epidermidis_NIH051475	0	0	1000	0	1000	0	1000	0	0	0
Staphylococcus_epidermidis_NIH051668	0	0	1000	0	1000	0	1000	0	0	0
Staphylococcus_epidermidis_NIH06004	0	0	1000	0	1000	0	1000	0	0	0
Staphylococcus_epidermidis_NIH08001	0	0	1000	0	1000	0	1000	0	0	0
Staphylococcus_epidermidis_NIHLM001	0	0	1000	0	1000	0	1000	0	0	0
Staphylococcus_epidermidis_NIHLM003	0	0	1000	0	1000	0	1000	0	0	0
Staphylococcus_epidermidis_NIHLM008	0	0	1000	0	1000	0	1000	0	0	0
Staphylococcus_epidermidis_NIHLM015	0	0	1000	0	1000	0	1000	0	0	0
Staphylococcus_epidermidis_NIHLM018	0	0	1000	0	1000	0	1000	0	0	0
Staphylococcus_epidermidis_NIHLM020	0	0	1000	0	1000	0	1000	0	0	0
Staphylococcus_epidermidis_NIHLM021	0	0	1000	0	1000	0	1000	0	0	0
Staphylococcus_epidermidis_NIHLM023	0	0	1000	0	1000	0	1000	0	0	0
Staphylococcus_epidermidis_NIHLM031	0	0	1000	0	1000	0	1000	0	0	0
Staphylococcus_epidermidis_NIHLM037	0	0	1000	0	1000	0	1000	0	0	0
Staphylococcus_epidermidis_NIHLM039	0	0	1000	0	1000	0	1000	0	0	0
Staphylococcus_epidermidis_NIHLM040	0	0	1000	0	1000	0	1000	0	0	0
Staphylococcus_epidermidis_NIHLM049	0	0	1000	0	1000	0	1000	0	0	0
Staphylococcus_epidermidis_NIHLM053	0	0	1000	0	1000	0	1000	0	0	0
Staphylococcus_epidermidis_NIHLM057	0	0	1000	0	1000	0	1000	0	0	0
Staphylococcus_epidermidis_NIHLM061	0	0	1000	0	1000	0	1000	0	0	0
Staphylococcus_epidermidis_NIHLM067	0	0	1000	0	1000	0	1000	0	0	0
Staphylococcus_epidermidis_NIHLM070	0	0	1000	0	1000	0	1000	0	0	0
Staphylococcus_epidermidis_NIHLM087	0	0	1000	0	1000	0	1000	0	0	0
Staphylococcus_epidermidis_NIHLM088	0	0	1000	0	1000	0	1000	0	0	0
Staphylococcus_epidermidis_NIHLM095	0	0	1000	0	1000	0	1000	0	0	0
Staphylococcus_epidermidis_RP62A	0	0	1000	0	1000	0	1000	0	0	0
Staphylococcus_epidermidis_SK135	0	0	1000	0	1000	0	1000	0	0	0
Staphylococcus_epidermidis_UC7032	0	0	1000	0	1000	0	1000	0	0	0
Staphylococcus_epidermidis_VCU028	0	0	1000	0	1000	0	1000	0	0	0
Staphylococcus_epidermidis_VCU037	0	0	1000	0	1000	0	1000	0	0	0
Staphylococcus_epidermidis_VCU041	0	0	1000	0	1000	0	1000	0	0	0
Staphylococcus_epidermidis_VCU045	0	0	1000	0	1000	0	1000	0	0	0
Staphylococcus_epidermidis_VCU065	0	0	1000	0	1000	0	1000	0	0	0
Staphylococcus_epidermidis_VCU071	0	0	1000	0	1000	0	1000	0	0	0
Staphylococcus_epidermidis_VCU081	0	0	1000	0	1000	0	1000	0	0	0
Staphylococcus_epidermidis_VCU105	0	0	1000	0	1000	0	1000	0	0	0
Staphylococcus_epidermidis_VCU109	0	0	1000	0	1000	0	1000	0	0	0
Staphylococcus_epidermidis_VCU117	0	0	1000	0	1000	0	1000	0	0	0
Staphylococcus_epidermidis_VCU118	0	0	1000	0	1000	0	1000	0	0	0
Staphylococcus_epidermidis_VCU120	0	0	1000	0	1000	0	1000	0	0	0
Staphylococcus_epidermidis_VCU123	0	0	1000	0	1000	0	1000	0	0	0
Staphylococcus_epidermidis_VCU125	0	0	1000	0	1000	0	1000	0	0	0
Staphylococcus_epidermidis_VCU126	0	0	1000	0	1000	0	1000	0	0	0
Staphylococcus_epidermidis_VCU127	0	0	1000	0	1000	0	1000	0	0	0
Staphylococcus_epidermidis_VCU128	0	0	1000	0	1000	0	1000	0	0	0
Staphylococcus_epidermidis_VCU129	0	0	1000	0	1000	0	1000	0	0	0
Staphylococcus_epidermidis_VCU144	0	0	1000	0	1000	0	1000	0	0	0
Staphylococcus_epidermidis_W23144	0	0	1000	0	1000	0	1000	0	0	0
Staphylococcus_equorum_subsp_equorum_Mu2	0	0	1000	1000	1000	0	1000	0	0	0
Staphylococcus_haemolyticus_JCSC1435	0	0	0	1000	1000	0	1000	0	0	0
Staphylococcus_haemolyticus_R1P1	0	0	1000	1000	1000	0	1000	0	0	0
Staphylococcus_hominis_SK119	0	0	1000	0	1000	0	1000	0	0	0
Staphylococcus_hominis_subsp_hominis_C80	0	0	1000	0	1000	0	1000	0	0	0
Staphylococcus_hominis_subsp_hominis_ZBW5	0	0	1000	0	1000	0	1000	0	0	0
Staphylococcus_hominis_VCU122	0	0	1000	0	1000	0	1000	0	0	0
Staphylococcus_intermedius_NCTC_11048	0	0	1000	0	1000	0	1000	0	0	0
Staphylococcus_lentus_ERR2221130	0	0	1000	0	1000	0	1000	0	0	0
Staphylococcus_lugdunensis_ACS_027_V_Sch2	0	0	1000	0	1000	0	1000	0	0	0
Staphylococcus_lugdunensis_HKU09_01	0	0	0	0	1000	0	1000	0	0	0
Staphylococcus_lugdunensis_M23590	0	0	1000	0	1000	0	1000	0	0	0
Staphylococcus_lugdunensis_N920143	0	0	1000	0	1000	0	1000	0	0	0
Staphylococcus_lugdunensis_VCU139	0	0	1000	0	1000	0	1000	0	0	0
Staphylococcus_nepalensis_ERR2221195	0	0	1000	0	1000	0	1000	0	0	0
Staphylococcus_pasteuri_BAB3	0	0	1000	0	1000	0	1000	0	0	0
Staphylococcus_pasteuri_SP1	0	0	1000	0	1000	0	1000	0	0	0
Staphylococcus_pettenkoferi_VCU012	0	0	0	0	1000	0	1000	0	0	0

Staphylococcus_pseudintermedius_ED99	0	0	1000	0	1000	0	1000	0	0	0
Staphylococcus_pseudintermedius_HKU10_03	0	0	1000	0	1000	0	1000	0	0	0
Staphylococcus_saprophyticus_subsp_saprophytic	0	0	1000	0	1000	0	1000	0	0	0
Staphylococcus_saprophyticus_subsp_saprophytic	0	0	1000	0	1000	0	1000	0	0	0
Staphylococcus_sciuri_subsp_sciuri_DSM_20345	0	0	1000	0	1000	0	1000	0	0	0
Staphylococcus_sciuri_subsp_sciuri_Z8	0	0	1000	0	1000	0	1000	0	0	0
Staphylococcus_simulans_ACS_120_V_Sch1	0	0	1000	1000	1000	0	1000	0	0	0
Staphylococcus_succinus_SNUC_1280	0	0	1000	0	1000	0	1000	0	0	0
Staphylococcus_succinus_subsp_casei_DSM_1509	0	0	1000	0	1000	0	1000	0	0	0
Staphylococcus_succinus_subsp_succinus_DSM_1	0	0	1000	0	1000	0	1000	0	0	0
Staphylococcus_vitulinus_F1028	0	0	0	0	1000	0	1000	0	0	0
Staphylococcus_warneri_L37603	0	0	1000	1000	1000	0	1000	0	0	0
Staphylococcus_warneri_SG1	0	0	1000	1000	1000	0	1000	0	0	0
Staphylococcus_xylosus_DMB3_Bh1	0	0	1000	0	1000	0	1000	0	0	0
Staphylococcus_xylosus_ERR2221122	0	0	1000	0	1000	0	1000	0	0	0
Staphylococcus_xylosus_ERR2221131	0	0	1000	0	1000	0	1000	0	0	0
Staphylococcus_xylosus_NJ	0	0	1000	1000	1000	0	1000	0	0	0
Stenotrophomonas_maltophilia_Ab55555	0	0	1000	0	1000	0	0	0	1000	0
Stenotrophomonas_maltophilia_ATCC_19867	0	0	1000	0	1000	0	0	0	1000	0
Stenotrophomonas_maltophilia_AU12_09	0	0	1000	0	1000	0	1000	0	1000	0
Stenotrophomonas_maltophilia_D457	0	0	0	0	1000	0	0	0	1000	1000
Stenotrophomonas_maltophilia_EPM1	0	0	1000	0	1000	0	0	0	1000	0
Stenotrophomonas_maltophilia_ISMMS3	0	0	1000	0	1000	0	0	0	1000	0
Stenotrophomonas_maltophilia_K279a	750	0	1000	0	1000	0	0	0	1000	0
Stenotrophomonas_maltophilia_PML168	0	0	1000	0	1000	0	0	0	1000	0
Stenotrophomonas_maltophilia_R551_3	750	0	1000	0	1000	0	0	0	1000	0
Stenotrophomonas_maltophilia_RR_10	0	0	1000	0	1000	0	0	0	1000	0
Stenotrophomonas_rhizophila_PCA13	0	0	1000	0	1000	0	0	0	0	1000
Stomatobaculum_longum_ACC2	0	0	0	0	1000	0	1000	0	0	0
Stoquefichus_massiliensis_AP9	0	0	0	0	1000	0	1000	0	0	0
Streptococcus_agalactiae_09mas018883	0	0	0	1000	0	0	1000	0	0	0
Streptococcus_agalactiae_2603V_R	0	0	0	1000	0	0	1000	0	0	0
Streptococcus_agalactiae_A909	0	0	0	1000	0	0	1000	0	0	1000
Streptococcus_agalactiae_BSU108	0	0	0	1000	0	0	1000	0	0	0
Streptococcus_agalactiae_BSU133	0	0	0	1000	0	0	1000	0	0	0
Streptococcus_agalactiae_BSU165	0	0	0	1000	0	0	1000	0	0	0
Streptococcus_agalactiae_BSU167	0	0	0	1000	0	0	1000	0	0	0
Streptococcus_agalactiae_BSU174	0	0	0	1000	0	0	1000	0	0	0
Streptococcus_agalactiae_BSU178	0	0	0	1000	0	0	1000	0	0	0
Streptococcus_agalactiae_BSU247	0	0	0	1000	0	0	1000	0	0	0
Streptococcus_agalactiae_BSU248	0	0	0	1000	0	0	1000	0	0	0
Streptococcus_agalactiae_BSU252	0	0	0	1000	0	0	1000	0	0	0
Streptococcus_agalactiae_BSU253	0	0	0	1000	0	0	1000	0	0	0
Streptococcus_agalactiae_BSU260	0	0	0	1000	0	0	1000	0	0	0
Streptococcus_agalactiae_BSU442	0	0	0	1000	0	0	1000	0	0	0
Streptococcus_agalactiae_BSU447	0	0	0	1000	0	0	1000	0	0	0
Streptococcus_agalactiae_BSU450	0	0	0	1000	0	0	1000	0	0	0
Streptococcus_agalactiae_BSU451	0	0	0	1000	0	0	1000	0	0	0
Streptococcus_agalactiae_BSU454	0	0	0	1000	0	0	1000	0	0	0
Streptococcus_agalactiae_BSU92	0	0	0	1000	0	0	1000	0	0	0
Streptococcus_agalactiae_BSU96	0	0	0	1000	0	0	1000	0	0	0
Streptococcus_agalactiae_CCUG_17336	0	0	0	1000	0	0	1000	0	0	0
Streptococcus_agalactiae_CCUG_19094	0	0	0	1000	0	0	1000	0	0	0
Streptococcus_agalactiae_CCUG_24810	0	0	0	1000	0	0	1000	0	0	0
Streptococcus_agalactiae_CCUG_25532	0	0	0	1000	0	0	1000	0	0	0
Streptococcus_agalactiae_CCUG_28551	0	0	0	1000	0	0	1000	0	0	0
Streptococcus_agalactiae_CCUG_29376	0	0	0	1000	0	0	1000	0	0	0
Streptococcus_agalactiae_CCUG_29782	0	0	0	1000	0	0	1000	0	0	0
Streptococcus_agalactiae_CCUG_30636	0	0	0	1000	0	0	1000	0	0	0
Streptococcus_agalactiae_CCUG_37430	0	0	0	1000	0	0	1000	0	0	0
Streptococcus_agalactiae_CCUG_37736	0	0	0	1000	0	0	1000	0	0	0
Streptococcus_agalactiae_CCUG_37737	0	0	0	1000	0	0	1000	0	0	0
Streptococcus_agalactiae_CCUG_37738	0	0	0	1000	0	0	1000	0	0	0
Streptococcus_agalactiae_CCUG_37739	0	0	0	1000	0	0	1000	0	0	0
Streptococcus_agalactiae_CCUG_37740	0	0	0	1000	0	0	1000	0	0	0
Streptococcus_agalactiae_CCUG_37741	0	0	0	1000	0	0	1000	0	0	0
Streptococcus_agalactiae_CCUG_37742	0	0	0	1000	0	0	1000	0	0	0
Streptococcus_agalactiae_CCUG_38383	0	0	0	1000	0	0	1000	0	0	0
Streptococcus_agalactiae_CCUG_39096_A	0	0	0	1000	0	0	1000	0	0	0
Streptococcus_agalactiae_CCUG_44050	0	0	0	1000	0	0	1000	0	0	0
Streptococcus_agalactiae_CCUG_44074	0	0	0	1000	0	0	1000	0	0	0
Streptococcus_agalactiae_CCUG_44077	0	0	0	1000	0	0	1000	0	0	0
Streptococcus_agalactiae_CCUG_44110	0	0	0	1000	0	0	1000	0	0	0
Streptococcus_agalactiae_CCUG_44140	0	0	0	1000	0	0	1000	0	0	0
Streptococcus_agalactiae_CCUG_44186	0	0	0	1000	0	0	1000	0	0	0
Streptococcus_agalactiae_CCUG_45061	0	0	0	1000	0	0	1000	0	0	0
Streptococcus_agalactiae_CCUG_47293	0	0	0	1000	0	0	1000	0	0	0
Streptococcus_agalactiae_CCUG_49072	0	0	0	1000	0	0	1000	0	0	0
Streptococcus_agalactiae_CCUG_49086	0	0	0	1000	0	0	1000	0	0	0
Streptococcus_agalactiae_CCUG_49087	0	0	0	1000	0	0	1000	0	0	0
Streptococcus_agalactiae_CCUG_49100	0	0	0	1000	0	0	1000	0	0	0
Streptococcus_agalactiae_CCUG_91	0	0	0	1000	0	0	1000	0	0	0
Streptococcus_agalactiae_CJB111	0	0	0	1000	0	0	1000	0	0	0
Streptococcus_agalactiae_ERR2221173	0	0	0	0	0	0	1000	0	0	0
Streptococcus_agalactiae_ERR2221321	0	0	0	0	0	0	1000	0	0	0
Streptococcus_agalactiae_FSL_C1_487	0	0	0	1000	0	0	1000	0	0	0
Streptococcus_agalactiae_FSL_C1_494	0	0	0	1000	0	0	1000	0	0	0
Streptococcus_agalactiae_FSL_F2_338	0	0	0	1000	0	0	1000	0	0	0
Streptococcus_agalactiae_FSL_F2_343	0	0	0	1000	0	0	1000	0	0	0
Streptococcus_agalactiae_FSL_S3_001	0	0	0	1000	0	0	1000	0	0	0
Streptococcus_agalactiae_FSL_S3_003	0	0	0	1000	0	0	1000	0	0	0
Streptococcus_agalactiae_FSL_S3_005	0	0	0	1000	0	0	1000	0	0	0
Streptococcus_agalactiae_FSL_S3_014	0	0	0	1000	0	0	1000	0	0	0
Streptococcus_agalactiae_FSL_S3_023	0	0	0	1000	0	0	1000	0	0	0

Streptococcus_agalactiae_FSL_S3_026	0	0	0	1000	0	0	1000	0	0	0
Streptococcus_agalactiae_FSL_S3_034	0	0	0	1000	0	0	1000	0	0	0
Streptococcus_agalactiae_FSL_S3_062	0	0	0	1000	0	0	1000	0	0	0
Streptococcus_agalactiae_FSL_S3_090	0	0	0	1000	0	0	1000	0	0	0
Streptococcus_agalactiae_FSL_S3_102	0	0	0	1000	0	0	1000	0	0	0
Streptococcus_agalactiae_FSL_S3_105	0	0	0	1000	0	0	1000	0	0	0
Streptococcus_agalactiae_FSL_S3_128	0	0	0	1000	0	0	1000	0	0	0
Streptococcus_agalactiae_FSL_S3_137	0	0	0	1000	0	0	1000	0	0	0
Streptococcus_agalactiae_FSL_S3_170	0	0	0	1000	0	0	1000	0	0	0
Streptococcus_agalactiae_FSL_S3_222	0	0	0	1000	0	0	1000	0	0	0
Streptococcus_agalactiae_FSL_S3_268	0	0	0	1000	0	0	1000	0	0	0
Streptococcus_agalactiae_FSL_S3_277	0	0	0	1000	0	0	1000	0	0	0
Streptococcus_agalactiae_FSL_S3_337	0	0	0	1000	0	0	1000	0	0	0
Streptococcus_agalactiae_FSL_S3_586	0	0	0	1000	0	0	1000	0	0	0
Streptococcus_agalactiae_FSL_S3_603	0	0	0	1000	0	0	1000	0	0	0
Streptococcus_agalactiae_FSL_S3_608	0	0	0	1000	0	0	1000	0	0	0
Streptococcus_agalactiae_FSL_S3_654	0	0	0	1000	0	0	1000	0	0	0
Streptococcus_agalactiae_GB00002	0	0	0	1000	0	0	1000	0	0	0
Streptococcus_agalactiae_GB00003	0	0	0	1000	0	0	1000	0	0	0
Streptococcus_agalactiae_GB00012	0	0	0	1000	0	0	1000	0	0	0
Streptococcus_agalactiae_GB00013	0	0	0	1000	0	0	1000	0	0	0
Streptococcus_agalactiae_GB00018	0	0	0	1000	0	0	1000	0	0	0
Streptococcus_agalactiae_GB00020	0	0	0	1000	0	0	1000	0	0	0
Streptococcus_agalactiae_GB00082	0	0	0	1000	0	0	1000	0	0	0
Streptococcus_agalactiae_GB00083	0	0	0	1000	0	0	1000	0	0	0
Streptococcus_agalactiae_GB00084	0	0	0	1000	0	0	1000	0	0	0
Streptococcus_agalactiae_GB00092	0	0	0	1000	0	0	1000	0	0	0
Streptococcus_agalactiae_GB00097	0	0	0	1000	0	0	1000	0	0	0
Streptococcus_agalactiae_GB00111	0	0	0	1000	0	0	1000	0	0	0
Streptococcus_agalactiae_GB00112	0	0	0	1000	0	0	1000	0	0	0
Streptococcus_agalactiae_GB00115	0	0	0	1000	0	0	1000	0	0	0
Streptococcus_agalactiae_GB00174	0	0	0	1000	0	0	1000	0	0	0
Streptococcus_agalactiae_GB00190	0	0	0	1000	0	0	1000	0	0	0
Streptococcus_agalactiae_GB00202	0	0	0	1000	0	0	1000	0	0	0
Streptococcus_agalactiae_GB00206	0	0	0	1000	0	0	1000	0	0	0
Streptococcus_agalactiae_GB00219	0	0	0	1000	0	0	1000	0	0	0
Streptococcus_agalactiae_GB00226	0	0	0	1000	0	0	1000	0	0	0
Streptococcus_agalactiae_GB00241	0	0	0	1000	0	0	1000	0	0	0
Streptococcus_agalactiae_GB00245	0	0	0	1000	0	0	1000	0	0	0
Streptococcus_agalactiae_GB00247	0	0	0	1000	0	0	1000	0	0	0
Streptococcus_agalactiae_GB00264	0	0	0	1000	0	0	1000	0	0	0
Streptococcus_agalactiae_GB00279	0	0	0	1000	0	0	1000	0	0	0
Streptococcus_agalactiae_GB00300	0	0	0	1000	0	0	1000	0	0	0
Streptococcus_agalactiae_GB00535	0	0	0	1000	0	0	1000	0	0	0
Streptococcus_agalactiae_GB00543	0	0	0	1000	0	0	1000	0	0	0
Streptococcus_agalactiae_GB00548	0	0	0	1000	0	0	1000	0	0	0
Streptococcus_agalactiae_GB00555	0	0	0	1000	0	0	1000	0	0	0
Streptococcus_agalactiae_GB00557	0	0	0	1000	0	0	1000	0	0	0
Streptococcus_agalactiae_GB00561	0	0	0	1000	0	0	1000	0	0	0
Streptococcus_agalactiae_GB00588	0	0	0	1000	0	0	1000	0	0	0
Streptococcus_agalactiae_GB00601	0	0	0	1000	0	0	1000	0	0	0
Streptococcus_agalactiae_GB00614	0	0	0	1000	0	0	1000	0	0	0
Streptococcus_agalactiae_GB00640	0	0	0	1000	0	0	1000	0	0	0
Streptococcus_agalactiae_GB00651	0	0	0	1000	0	0	1000	0	0	0
Streptococcus_agalactiae_GB00653	0	0	0	1000	0	0	1000	0	0	0
Streptococcus_agalactiae_GB00654	0	0	0	1000	0	0	1000	0	0	0
Streptococcus_agalactiae_GB00663	0	0	0	1000	0	0	1000	0	0	0
Streptococcus_agalactiae_GB00679	0	0	0	1000	0	0	1000	0	0	0
Streptococcus_agalactiae_GB00864	0	0	0	1000	0	0	1000	0	0	0
Streptococcus_agalactiae_GB00865	0	0	0	1000	0	0	1000	0	0	0
Streptococcus_agalactiae_GB00867	0	0	0	1000	0	0	1000	0	0	0
Streptococcus_agalactiae_GB00874	0	0	0	1000	0	0	1000	0	0	0
Streptococcus_agalactiae_GB00884	0	0	0	1000	0	0	1000	0	0	0
Streptococcus_agalactiae_GB00887	0	0	0	1000	0	0	1000	0	0	0
Streptococcus_agalactiae_GB00888	0	0	0	1000	0	0	1000	0	0	0
Streptococcus_agalactiae_GB00891	0	0	0	1000	0	0	1000	0	0	0
Streptococcus_agalactiae_GB00893	0	0	0	1000	0	0	1000	0	0	0
Streptococcus_agalactiae_GB00900	0	0	0	1000	0	0	1000	0	0	0
Streptococcus_agalactiae_GB00901	0	0	0	1000	0	0	1000	0	0	0
Streptococcus_agalactiae_GB00904	0	0	0	1000	0	0	1000	0	0	0
Streptococcus_agalactiae_GB00909	0	0	0	1000	0	0	1000	0	0	0
Streptococcus_agalactiae_GB00911	0	0	0	1000	0	0	1000	0	0	0
Streptococcus_agalactiae_GB00914	0	0	0	1000	0	0	1000	0	0	0
Streptococcus_agalactiae_GB00919	0	0	0	1000	0	0	1000	0	0	0
Streptococcus_agalactiae_GB00922	0	0	0	1000	0	0	1000	0	0	0
Streptococcus_agalactiae_GB00923	0	0	0	1000	0	0	1000	0	0	0
Streptococcus_agalactiae_GB00924	0	0	0	1000	0	0	1000	0	0	0
Streptococcus_agalactiae_GB00929	0	0	0	1000	0	0	1000	0	0	0
Streptococcus_agalactiae_GB00932	0	0	0	1000	0	0	1000	0	0	0
Streptococcus_agalactiae_GB00933	0	0	0	1000	0	0	1000	0	0	0
Streptococcus_agalactiae_GB00954	0	0	0	1000	0	0	1000	0	0	0
Streptococcus_agalactiae_GB00955	0	0	0	1000	0	0	1000	0	0	0
Streptococcus_agalactiae_GB00963	0	0	0	1000	0	0	1000	0	0	0
Streptococcus_agalactiae_GB00965	0	0	0	1000	0	0	1000	0	0	0
Streptococcus_agalactiae_GB00975	0	0	0	1000	0	0	1000	0	0	0
Streptococcus_agalactiae_GB00984	0	0	0	1000	0	0	1000	0	0	0
Streptococcus_agalactiae_GB00986	0	0	0	0	0	0	1000	0	0	0
Streptococcus_agalactiae_GB00999	0	0	0	1000	0	0	1000	0	0	0
Streptococcus_agalactiae_GD201008_001	0	0	0	1000	0	0	1000	0	0	0
Streptococcus_agalactiae_ILRI005	0	0	0	1000	0	0	1000	0	0	0
Streptococcus_agalactiae_ILRI112	0	0	0	1000	0	0	1000	0	0	0
Streptococcus_agalactiae_LADL_05_108a	0	0	0	1000	0	0	1000	0	0	0
Streptococcus_agalactiae_LADL_90_503	0	0	0	1000	0	0	1000	0	0	0
Streptococcus_agalactiae_LMG_14609	0	0	0	1000	0	0	1000	0	0	0

Streptococcus_agalactiae_LMG_14838	0	0	0	1000	0	0	1000	0	0	0
Streptococcus_agalactiae_LMG_15081	0	0	0	1000	0	0	1000	0	0	0
Streptococcus_agalactiae_LMG_15083	0	0	0	1000	0	0	1000	0	0	0
Streptococcus_agalactiae_LMG_15084	0	0	0	1000	0	0	1000	0	0	0
Streptococcus_agalactiae_LMG_15085	0	0	0	1000	0	0	1000	0	0	0
Streptococcus_agalactiae_LMG_15089	0	0	0	1000	0	0	1000	0	0	0
Streptococcus_agalactiae_LMG_15090	0	0	0	1000	0	0	1000	0	0	0
Streptococcus_agalactiae_LMG_15091	0	0	0	1000	0	0	1000	0	0	0
Streptococcus_agalactiae_LMG_15092	0	0	0	1000	0	0	1000	0	0	0
Streptococcus_agalactiae_LMG_15094	0	0	0	1000	0	0	1000	0	0	0
Streptococcus_agalactiae_LMG_15095	0	0	0	1000	0	0	1000	0	0	0
Streptococcus_agalactiae_MRI_Z1_012	0	0	0	1000	0	0	1000	0	0	0
Streptococcus_agalactiae_MRI_Z1_022	0	0	0	1000	0	0	1000	0	0	0
Streptococcus_agalactiae_MRI_Z1_023	0	0	0	1000	0	0	1000	0	0	0
Streptococcus_agalactiae_MRI_Z1_025	0	0	0	1000	0	0	1000	0	0	0
Streptococcus_agalactiae_MRI_Z1_035	0	0	0	1000	0	0	1000	0	0	0
Streptococcus_agalactiae_MRI_Z1_038	0	0	0	1000	0	0	1000	0	0	0
Streptococcus_agalactiae_MRI_Z1_198	0	0	0	1000	0	0	1000	0	0	0
Streptococcus_agalactiae_MRI_Z1_199	0	0	0	1000	0	0	1000	0	0	0
Streptococcus_agalactiae_MRI_Z1_200	0	0	0	1000	0	0	1000	0	0	0
Streptococcus_agalactiae_MRI_Z1_201	0	0	0	1000	0	0	1000	0	0	0
Streptococcus_agalactiae_MRI_Z1_202	0	0	0	1000	0	0	1000	0	0	0
Streptococcus_agalactiae_MRI_Z1_203	0	0	0	1000	0	0	1000	0	0	0
Streptococcus_agalactiae_MRI_Z1_204	0	0	0	1000	0	0	1000	0	0	0
Streptococcus_agalactiae_MRI_Z1_205	0	0	0	1000	0	0	1000	0	0	0
Streptococcus_agalactiae_MRI_Z1_206	0	0	0	1000	0	0	1000	0	0	0
Streptococcus_agalactiae_MRI_Z1_211	0	0	0	1000	0	0	1000	0	0	0
Streptococcus_agalactiae_MRI_Z1_212	0	0	0	1000	0	0	1000	0	0	0
Streptococcus_agalactiae_MRI_Z1_213	0	0	0	1000	0	0	1000	0	0	0
Streptococcus_agalactiae_MRI_Z1_214	0	0	0	1000	0	0	1000	0	0	0
Streptococcus_agalactiae_MRI_Z1_215	0	0	0	1000	0	0	1000	0	0	0
Streptococcus_agalactiae_MRI_Z1_216	0	0	0	1000	0	0	1000	0	0	0
Streptococcus_agalactiae_MRI_Z1_217	0	0	0	1000	0	0	1000	0	0	0
Streptococcus_agalactiae_MRI_Z1_218	0	0	0	1000	0	0	1000	0	0	0
Streptococcus_agalactiae_MRI_Z1_219	0	0	0	1000	0	0	1000	0	0	0
Streptococcus_agalactiae_NEM316	0	0	0	1000	0	0	1000	0	0	0
Streptococcus_agalactiae_PR06	0	0	0	1000	0	0	1000	0	0	0
Streptococcus_agalactiae_SA20_06	0	0	0	1000	0	0	1000	0	0	0
Streptococcus_agalactiae_SS1014	0	0	0	1000	0	0	1000	0	0	0
Streptococcus_agalactiae_SS1218	0	0	0	1000	0	0	1000	0	0	0
Streptococcus_agalactiae_SS1219	0	0	0	1000	0	0	1000	0	0	0
Streptococcus_agalactiae_STIR_CD_01	0	0	0	1000	0	0	1000	0	0	0
Streptococcus_agalactiae_STIR_CD_09	0	0	0	1000	0	0	1000	0	0	0
Streptococcus_agalactiae_STIR_CD_14	0	0	0	1000	0	0	1000	0	0	0
Streptococcus_agalactiae_STIR_CD_17	0	0	0	1000	0	0	1000	0	0	0
Streptococcus_agalactiae_STIR_CD_21	0	0	0	1000	0	0	1000	0	0	0
Streptococcus_agalactiae_STIR_CD_22	0	0	0	1000	0	0	1000	0	0	0
Streptococcus_agalactiae_STIR_CD_23	0	0	0	1000	0	0	1000	0	0	0
Streptococcus_agalactiae_STIR_CD_24	0	0	0	1000	0	0	1000	0	0	0
Streptococcus_agalactiae_STIR_CD_26	0	0	0	1000	0	0	1000	0	0	0
Streptococcus_agalactiae_STIR_CD_27	0	0	0	1000	0	0	1000	0	0	0
Streptococcus_agalactiae_STIR_CD_28	0	0	0	1000	0	0	1000	0	0	0
Streptococcus_agalactiae_STIR_CD_29	0	0	0	1000	0	0	1000	0	0	0
Streptococcus_agalactiae_str_Gottschalk_1002A	0	0	0	1000	0	0	1000	0	0	0
Streptococcus_agalactiae_str_Gottschalk_1003A	0	0	0	1000	0	0	1000	0	0	0
Streptococcus_agalactiae_str_Gottschalk_13227	0	0	0	1000	0	0	1000	0	0	0
Streptococcus_agalactiae_str_Gottschalk_2864	0	0	0	1000	0	0	1000	0	0	0
Streptococcus_agalactiae_str_Gottschalk_31825	0	0	0	1000	0	0	1000	0	0	0
Streptococcus_agalactiae_str_Gottschalk_992B	0	0	0	1000	0	0	1000	0	0	0
Streptococcus_agalactiae_str_Gottschalk_998A	0	0	0	1000	0	0	1000	0	0	0
Streptococcus_agalactiae_str_Gottschalk_999B	0	0	0	1000	0	0	1000	0	0	0
Streptococcus_agalactiae_ZQ0910	0	0	0	1000	0	0	1000	0	0	0
Streptococcus_anginosus_1_2_62CV	0	0	0	0	0	0	1000	0	0	0
Streptococcus_anginosus_CCUG_39159	0	0	0	0	0	0	1000	0	0	0
Streptococcus_anginosus_ERR2221320	0	0	0	0	0	0	1000	0	0	0
Streptococcus_anginosus_F0211	0	0	0	0	0	0	1000	0	0	0
Streptococcus_anginosus_SK1138	0	0	0	0	0	0	1000	0	0	0
Streptococcus_anginosus_SK52	0	0	0	0	0	0	1000	0	0	0
Streptococcus_australis_ATCC_700641	0	0	0	0	0	0	1000	0	0	1000
Streptococcus_australis_ERR2221360	0	0	0	0	0	0	1000	0	0	0
Streptococcus_bovis_ATCC_700338	0	0	1000	1000	0	0	1000	0	0	0
Streptococcus_constellatus_subsp_constellatus_S	0	0	0	0	0	0	1000	0	0	1000
Streptococcus_constellatus_subsp_pharyngis_SK1	0	0	0	0	0	0	1000	0	0	1000
Streptococcus_cristatus_ATCC_51100	0	0	0	0	0	0	1000	0	0	1000
Streptococcus_cristatus_LRIIBV4	0	0	0	0	0	0	1000	0	0	0
Streptococcus_danieliae_NM51_B2_22	0	0	0	0	0	0	1000	0	0	0
Streptococcus_downei_F0415	0	0	1000	0	0	0	1000	0	0	0
Streptococcus_downei_MFe28	0	0	1000	0	0	0	1000	0	0	0
Streptococcus_dysgalactiae_subsp_dysgalactiae_	0	0	0	1000	0	0	1000	0	0	1000
Streptococcus_dysgalactiae_subsp_equisimilis_AC	0	0	0	1000	0	0	1000	0	0	0
Streptococcus_dysgalactiae_subsp_equisimilis_AT	0	0	0	1000	0	0	1000	0	0	0
Streptococcus_dysgalactiae_subsp_equisimilis_GC	0	0	0	1000	0	0	1000	0	0	0
Streptococcus_dysgalactiae_subsp_equisimilis_RE	0	0	0	1000	0	0	1000	0	0	0
Streptococcus_dysgalactiae_subsp_equisimilis_SK	0	0	0	1000	0	0	1000	0	0	0
Streptococcus_equi_subsp_equi_4047	0	0	0	1000	0	0	1000	0	0	0
Streptococcus_equi_subsp_zooepidemicus_ATCC_	0	0	0	1000	0	0	1000	0	0	0
Streptococcus_equi_subsp_zooepidemicus_BHS5	0	0	0	1000	0	0	1000	0	0	0
Streptococcus_equi_subsp_zooepidemicus_MGCS	0	0	0	1000	0	0	1000	0	0	0
Streptococcus_equinus_ATCC_9812	0	0	1000	0	0	0	1000	0	0	0
Streptococcus_gallolyticus_subsp_gallolyticus_AT	0	0	0	0	0	0	1000	0	0	1000
Streptococcus_gallolyticus_subsp_gallolyticus_AT	0	0	1000	0	0	0	1000	0	0	0
Streptococcus_gallolyticus_subsp_gallolyticus_TX	0	0	1000	0	0	0	1000	0	0	0
Streptococcus_gallolyticus_UCN34	0	0	1000	0	0	0	1000	0	0	0
Streptococcus_gordonii_str_Challis_substr_CH1	0	0	0	0	0	0	1000	0	0	1000

Streptococcus_infantarius_subsp_infantarius_ATC	0	0	1000	0	0	0	1000	0	0	0
Streptococcus_infantarius_subsp_infantarius_CJ1	0	0	1000	0	0	0	1000	0	0	0
Streptococcus_infantis_ATCC_700779	0	0	0	0	1000	0	1000	0	0	1000
Streptococcus_infantis_SK1076	0	0	0	0	0	0	1000	0	0	0
Streptococcus_infantis_SK970	0	0	0	0	0	0	1000	0	0	0
Streptococcus_infantis_X	0	0	0	0	0	0	1000	0	0	0
Streptococcus_intermedius_ATCC_27335	0	0	0	0	0	0	1000	0	0	0
Streptococcus_intermedius_BA1	0	0	0	0	0	0	1000	0	0	0
Streptococcus_intermedius_F0395	0	0	0	0	0	0	1000	0	0	0
Streptococcus_intermedius_F0413	0	0	0	0	0	0	1000	0	0	0
Streptococcus_intermedius_JTH08	0	0	0	0	0	0	1000	0	0	1000
Streptococcus_lutetiensis_033	0	0	1000	0	0	0	1000	0	0	1000
Streptococcus_lutetiensis_ERR2230054	0	0	1000	0	0	0	1000	0	0	0
Streptococcus_macedonicus_679	0	0	1000	0	0	0	1000	0	0	0
Streptococcus_macedonicus_ACA_DC_198	0	0	1000	0	0	0	1000	0	0	0
Streptococcus_massiliensis_4401825	0	0	0	0	0	0	1000	0	0	0
Streptococcus_massiliensis_DSM_18628	0	0	0	0	0	0	1000	0	0	0
Streptococcus_mitis_11_5	0	0	0	0	0	0	1000	0	0	0
Streptococcus_mitis_13_39	0	0	0	0	0	0	1000	0	0	0
Streptococcus_mitis_ATCC_6249	0	0	0	0	0	0	1000	0	0	0
Streptococcus_mitis_B6	0	0	0	0	0	0	1000	0	0	0
Streptococcus_mitis_bv_2_str_F0392	0	0	0	0	0	0	1000	0	0	0
Streptococcus_mitis_bv_2_str_SK95	0	0	0	0	0	0	1000	0	0	0
Streptococcus_mitis_NCTC_12261	0	0	0	0	0	0	1000	0	0	1000
Streptococcus_mitis_SK1073	0	0	0	0	0	0	1000	0	0	0
Streptococcus_mitis_SK1080	0	0	0	0	0	0	1000	0	0	0
Streptococcus_mitis_SK321	0	0	0	0	0	0	1000	0	0	0
Streptococcus_mitis_SK564	0	0	0	0	0	0	1000	0	0	0
Streptococcus_mitis_SK569	0	0	0	0	0	0	1000	0	0	0
Streptococcus_mitis_SK575	0	0	0	0	0	0	1000	0	0	0
Streptococcus_mitis_SK579	0	0	0	0	0	0	1000	0	0	0
Streptococcus_mitis_SK616	0	0	0	0	0	0	1000	0	0	0
Streptococcus_mitis_SPAR10	0	0	0	0	0	0	1000	0	0	0
Streptococcus_mutans_11A1	0	0	1000	0	0	0	0	0	0	0
Streptococcus_mutans_11SSST2	0	0	1000	0	0	0	0	0	0	0
Streptococcus_mutans_14D	0	0	1000	0	0	0	0	0	0	0
Streptococcus_mutans_15JP3	0	0	1000	0	0	0	0	0	0	0
Streptococcus_mutans_15VF2	0	0	1000	0	0	0	0	0	0	0
Streptococcus_mutans_1SM1	0	0	1000	0	0	0	0	0	0	0
Streptococcus_mutans_21	0	0	1000	0	0	0	0	0	0	0
Streptococcus_mutans_24	0	0	1000	0	0	0	0	0	0	0
Streptococcus_mutans_2ST1	0	0	1000	0	0	0	0	0	0	0
Streptococcus_mutans_2VS1	0	0	1000	0	0	0	0	0	0	0
Streptococcus_mutans_3SN1	0	0	1000	0	0	0	0	0	0	0
Streptococcus_mutans_4SM1	0	0	1000	0	0	0	0	0	0	0
Streptococcus_mutans_4VF1	0	0	1000	0	0	0	0	0	0	0
Streptococcus_mutans_5DC8	0	0	1000	0	0	0	0	0	0	0
Streptococcus_mutans_5SM3	0	0	1000	0	0	0	0	0	0	0
Streptococcus_mutans_66_2A	0	0	1000	0	0	0	0	0	0	0
Streptococcus_mutans_8ID3	0	0	1000	0	0	0	0	0	0	0
Streptococcus_mutans_A19	0	0	1000	0	0	0	0	0	0	0
Streptococcus_mutans_A9	0	0	1000	0	0	0	0	0	0	0
Streptococcus_mutans_AC4446	0	0	1000	0	0	0	0	0	0	0
Streptococcus_mutans_ATCC_25175	0	0	1000	0	0	0	0	0	0	1000
Streptococcus_mutans_B	0	0	1000	0	0	0	0	0	0	0
Streptococcus_mutans_DSM_20523	0	0	1000	0	0	0	0	0	0	0
Streptococcus_mutans_ERR2221174	0	0	1000	0	0	0	0	0	0	0
Streptococcus_mutans_ERR2221288	0	0	1000	0	0	0	0	0	0	0
Streptococcus_mutans_G123	0	0	1000	0	0	0	0	0	0	0
Streptococcus_mutans_GS_5	0	0	1000	0	0	0	1000	0	0	0
Streptococcus_mutans_KK21	0	0	1000	0	0	0	0	0	0	0
Streptococcus_mutans_KK23	0	0	1000	0	0	0	0	0	0	0
Streptococcus_mutans_M21	0	0	1000	0	0	0	0	0	0	0
Streptococcus_mutans_M230	0	0	1000	0	0	0	0	0	0	0
Streptococcus_mutans_M2A	0	0	1000	0	0	0	0	0	0	0
Streptococcus_mutans_N29	0	0	1000	0	0	0	0	0	0	0
Streptococcus_mutans_N3209	0	0	1000	0	0	0	0	0	0	0
Streptococcus_mutans_N34	0	0	1000	0	0	0	0	0	0	0
Streptococcus_mutans_N66	0	0	1000	0	0	0	0	0	0	0
Streptococcus_mutans_NCTC_11060	0	0	1000	0	0	0	0	0	0	0
Streptococcus_mutans_NFSM1	0	0	1000	0	0	0	0	0	0	0
Streptococcus_mutans_NFSM2	0	0	1000	0	0	0	0	0	0	0
Streptococcus_mutans_NLML1	0	0	1000	0	0	0	0	0	0	0
Streptococcus_mutans_NLML4	0	0	1000	0	0	0	0	0	0	0
Streptococcus_mutans_NLML5	0	0	1000	0	0	0	0	0	0	0
Streptococcus_mutans_NLML8	0	0	1000	0	0	0	0	0	0	0
Streptococcus_mutans_NLML9	0	0	1000	0	0	0	0	0	0	0
Streptococcus_mutans_NMT4863	0	0	1000	0	0	0	0	0	0	0
Streptococcus_mutans_NN2025	0	0	1000	0	0	0	1000	0	0	0
Streptococcus_mutans_NV1996	0	0	1000	0	0	0	0	0	0	0
Streptococcus_mutans_NVAB	0	0	1000	0	0	0	0	0	0	0
Streptococcus_mutans_OMZ175	0	0	1000	0	0	0	0	0	0	0
Streptococcus_mutans_R221	0	0	1000	0	0	0	0	0	0	0
Streptococcus_mutans_S1B	0	0	1000	0	0	0	0	0	0	0
Streptococcus_mutans_SA38	0	0	1000	0	0	0	0	0	0	0
Streptococcus_mutans_SA41	0	0	1000	0	0	0	0	0	0	0
Streptococcus_mutans_SF1	0	0	1000	0	0	0	0	0	0	0
Streptococcus_mutans_SF12	0	0	1000	0	0	0	0	0	0	0
Streptococcus_mutans_SF14	0	0	1000	0	0	0	0	0	0	0
Streptococcus_mutans_SM1	0	0	1000	0	0	0	0	0	0	0
Streptococcus_mutans_SM4	0	0	1000	0	0	0	0	0	0	0
Streptococcus_mutans_SM6	0	0	1000	0	0	0	0	0	0	0
Streptococcus_mutans_ST1	0	0	1000	0	0	0	0	0	0	0
Streptococcus_mutans_ST6	0	0	1000	0	0	0	0	0	0	0

Streptococcus_mutans_T4	0	0	1000	0	0	0	0	0	0	0
Streptococcus_mutans_TCI_116	0	0	1000	0	0	0	0	0	0	0
Streptococcus_mutans_TCI_123	0	0	1000	0	0	0	0	0	0	0
Streptococcus_mutans_TCI_125	0	0	1000	0	0	0	0	0	0	0
Streptococcus_mutans_TCI_138	0	0	1000	0	0	0	0	0	0	0
Streptococcus_mutans_TCI_145	0	0	1000	0	0	0	0	0	0	0
Streptococcus_mutans_TCI_152	0	0	1000	0	0	0	0	0	0	0
Streptococcus_mutans_TCI_153	0	0	1000	0	0	0	0	0	0	0
Streptococcus_mutans_TCI_163	0	0	1000	0	0	0	0	0	0	0
Streptococcus_mutans_TCI_169	0	0	1000	0	0	0	0	0	0	0
Streptococcus_mutans_TCI_173	0	0	1000	0	0	0	0	0	0	0
Streptococcus_mutans_TCI_177	0	0	1000	0	0	0	0	0	0	0
Streptococcus_mutans_TCI_191	0	0	1000	0	0	0	0	0	0	0
Streptococcus_mutans_TCI_196	0	0	1000	0	0	0	0	0	0	0
Streptococcus_mutans_TCI_219	0	0	1000	0	0	0	0	0	0	0
Streptococcus_mutans_TCI_222	0	0	1000	0	0	0	0	0	0	0
Streptococcus_mutans_TCI_228	0	0	1000	0	0	0	0	0	0	0
Streptococcus_mutans_TCI_234	0	0	1000	0	0	0	0	0	0	0
Streptococcus_mutans_TCI_239	0	0	1000	0	0	0	0	0	0	0
Streptococcus_mutans_TCI_242	0	0	1000	0	0	0	0	0	0	0
Streptococcus_mutans_TCI_243	0	0	1000	0	0	0	0	0	0	0
Streptococcus_mutans_TCI_249	0	0	1000	0	0	0	0	0	0	0
Streptococcus_mutans_TCI_256	0	0	1000	0	0	0	0	0	0	0
Streptococcus_mutans_TCI_268	0	0	1000	0	0	0	0	0	0	0
Streptococcus_mutans_TCI_278	0	0	1000	0	0	0	0	0	0	0
Streptococcus_mutans_TCI_294	0	0	1000	0	0	0	0	0	0	0
Streptococcus_mutans_TCI_30	0	0	1000	0	0	0	0	0	0	0
Streptococcus_mutans_TCI_399	0	0	1000	0	0	0	0	0	0	0
Streptococcus_mutans_TCI_400	0	0	1000	0	0	0	0	0	0	0
Streptococcus_mutans_TCI_51	0	0	1000	0	0	0	0	0	0	0
Streptococcus_mutans_TCI_70	0	0	1000	0	0	0	0	0	0	0
Streptococcus_mutans_TCI_75	0	0	1000	0	0	0	0	0	0	0
Streptococcus_mutans_TCI_78	0	0	1000	0	0	0	0	0	0	0
Streptococcus_mutans_TCI_82	0	0	1000	0	0	0	0	0	0	0
Streptococcus_mutans_TCI_85	0	0	1000	0	0	0	0	0	0	0
Streptococcus_mutans_TCI_86	0	0	1000	0	0	0	0	0	0	0
Streptococcus_mutans_TCI_92	0	0	1000	0	0	0	0	0	0	0
Streptococcus_mutans_TCI_96	0	0	1000	0	0	0	0	0	0	0
Streptococcus_mutans_U138	0	0	1000	0	0	0	0	0	0	0
Streptococcus_mutans_U2A	0	0	1000	0	0	0	0	0	0	0
Streptococcus_mutans_U2B	0	0	1000	0	0	0	0	0	0	0
Streptococcus_mutans_UA159	0	0	1000	0	0	0	0	0	0	0
Streptococcus_mutans_W6	0	0	1000	0	0	0	0	0	0	0
Streptococcus_nov_ERR2221358	0	0	0	0	0	0	1000	0	0	0
Streptococcus_oralis_ATCC_35037	0	0	0	0	0	0	1000	0	0	0
Streptococcus_oralis_ERR2221353	0	0	0	0	0	0	1000	0	0	0
Streptococcus_oralis_SK10	0	0	0	0	0	0	1000	0	0	0
Streptococcus_oralis_SK100	0	0	0	0	0	0	1000	0	0	0
Streptococcus_oralis_SK1074	0	0	0	0	0	0	1000	0	0	0
Streptococcus_oralis_SK255	0	0	0	0	0	0	1000	0	0	0
Streptococcus_oralis_SK304	0	0	0	0	0	0	1000	0	0	0
Streptococcus_oralis_SK313	0	0	0	0	0	0	1000	0	0	0
Streptococcus_oralis_SK610	0	0	0	0	0	0	1000	0	0	0
Streptococcus_oralis_subsp_tigurinus_1366	0	0	0	0	0	0	1000	0	0	0
Streptococcus_oralis_subsp_tigurinus_2425	0	0	0	0	0	0	1000	0	0	0
Streptococcus_oralis_subsp_tigurinus_UC5873_TZ	0	0	0	0	0	0	1000	0	0	0
Streptococcus_oralis_Uo5	0	0	0	0	0	0	1000	0	0	1000
Streptococcus_parasanguinis_ATCC_15912	0	0	0	0	0	0	1000	0	0	0
Streptococcus_parasanguinis_ATCC_903	0	0	0	0	0	0	1000	0	0	1000
Streptococcus_parasanguinis_ERR2221175	0	0	0	0	0	0	1000	0	0	0
Streptococcus_parasanguinis_ERR2221190	0	0	0	0	0	0	1000	0	0	0
Streptococcus_parasanguinis_F0405	0	0	0	0	0	0	1000	0	0	0
Streptococcus_parasanguinis_FW213	0	0	0	0	0	0	1000	0	0	0
Streptococcus_parasanguinis_SK236	0	0	0	0	0	0	0	0	0	0
Streptococcus_parauberis_KCTC_11537	0	0	0	0	0	0	1000	0	0	1000
Streptococcus_parauberis_KCTC_11980BP	0	0	0	0	0	0	1000	0	0	0
Streptococcus_parauberis_KRS_02083	0	0	0	0	0	0	1000	0	0	0
Streptococcus_parauberis_KRS_02109	0	0	0	0	0	0	1000	0	0	0
Streptococcus_parauberis_NCFD_2020	0	0	0	0	0	0	1000	0	0	0
Streptococcus_pasteurianus_651_SPAS_0_477_18	0	0	1000	0	0	0	1000	0	0	0
Streptococcus_pasteurianus_ATCC_43144	0	0	1000	1000	0	0	1000	0	0	0
Streptococcus_peroris_ATCC_700780	0	0	0	0	1000	0	1000	0	0	1000
Streptococcus_pleomorphus_DSM_20574	0	0	1000	0	0	0	1000	0	0	0
Streptococcus_pneumoniae_1974M1_LZD	0	0	0	1000	0	0	1000	0	0	0
Streptococcus_pneumoniae_2009	0	0	0	1000	0	0	1000	0	0	0
Streptococcus_pneumoniae_2061376	0	0	0	1000	0	0	1000	0	0	0
Streptococcus_pneumoniae_2061617	0	0	0	1000	0	0	1000	0	0	0
Streptococcus_pneumoniae_2070005	0	0	0	1000	0	0	1000	0	0	0
Streptococcus_pneumoniae_2070035	0	0	0	1000	0	0	1000	0	0	0
Streptococcus_pneumoniae_2070109	0	0	0	1000	0	0	1000	0	0	0
Streptococcus_pneumoniae_2070425	0	0	0	1000	0	0	1000	0	0	0
Streptococcus_pneumoniae_2070531	0	0	0	1000	0	0	1000	0	0	0
Streptococcus_pneumoniae_2071004	0	0	0	1000	0	0	1000	0	0	0
Streptococcus_pneumoniae_2071247	0	0	0	1000	0	0	1000	0	0	0
Streptococcus_pneumoniae_2072047	0	0	0	1000	0	0	1000	0	0	0
Streptococcus_pneumoniae_2080076	0	0	0	1000	0	0	1000	0	0	0
Streptococcus_pneumoniae_2080913	0	0	0	1000	0	0	1000	0	0	0
Streptococcus_pneumoniae_2081074	0	0	0	1000	0	0	1000	0	0	0
Streptococcus_pneumoniae_2081685	0	0	0	1000	0	0	1000	0	0	0
Streptococcus_pneumoniae_2082170	0	0	0	1000	0	0	1000	0	0	0
Streptococcus_pneumoniae_2090008	0	0	0	1000	0	0	1000	0	0	0
Streptococcus_pneumoniae_23F	0	0	0	1000	0	0	1000	0	0	0
Streptococcus_pneumoniae_3063_00	0	0	0	1000	0	0	1000	0	0	0
Streptococcus_pneumoniae_357	0	0	0	1000	0	0	1000	0	0	0

Streptococcus_pneumoniae_4027_06	0	0	0	1000	0	0	1000	0	0	0
Streptococcus_pneumoniae_4075_00	0	0	0	1000	0	0	1000	0	0	0
Streptococcus_pneumoniae_459_5	0	0	0	1000	0	0	1000	0	0	0
Streptococcus_pneumoniae_5185_06	0	0	0	1000	0	0	1000	0	0	0
Streptococcus_pneumoniae_5652_06	0	0	0	1000	0	0	1000	0	0	0
Streptococcus_pneumoniae_5787_06	0	0	0	1000	0	0	1000	0	0	0
Streptococcus_pneumoniae_670_6B	0	0	0	1000	0	0	1000	0	0	0
Streptococcus_pneumoniae_6901_05	0	0	0	1000	0	0	1000	0	0	0
Streptococcus_pneumoniae_6963_05	0	0	0	1000	0	0	1000	0	0	0
Streptococcus_pneumoniae_70585	0	0	0	1000	0	0	1000	0	0	0
Streptococcus_pneumoniae_7286_06	0	0	0	1000	0	0	1000	0	0	0
Streptococcus_pneumoniae_7533_05	0	0	0	1000	0	0	1000	0	0	0
Streptococcus_pneumoniae_7879_04	0	0	0	1000	0	0	1000	0	0	0
Streptococcus_pneumoniae_8190_05	0	0	0	1000	0	0	1000	0	0	0
Streptococcus_pneumoniae_AP200	0	0	0	1000	0	0	1000	0	0	0
Streptococcus_pneumoniae_ATCC_700669	0	0	0	1000	0	0	1000	0	0	0
Streptococcus_pneumoniae_BS397	0	0	0	1000	0	0	1000	0	0	0
Streptococcus_pneumoniae_BS455	0	0	0	1000	0	0	1000	0	0	0
Streptococcus_pneumoniae_BS457	0	0	0	1000	0	0	1000	0	0	0
Streptococcus_pneumoniae_BS458	0	0	0	1000	0	0	1000	0	0	0
Streptococcus_pneumoniae_CDC0288_04	0	0	0	1000	0	0	1000	0	0	0
Streptococcus_pneumoniae_CDC1873_00	0	0	0	1000	0	0	1000	0	0	0
Streptococcus_pneumoniae_CDC3059_06	0	0	0	1000	0	0	1000	0	0	0
Streptococcus_pneumoniae_CGSP14	0	0	0	1000	0	0	1000	0	0	0
Streptococcus_pneumoniae_D39	0	0	0	1000	0	0	1000	0	0	0
Streptococcus_pneumoniae_England14_9	0	0	0	1000	0	0	1000	0	0	0
Streptococcus_pneumoniae_EU_NP01	0	0	0	1000	0	0	1000	0	0	0
Streptococcus_pneumoniae_EU_NP02	0	0	0	1000	0	0	1000	0	0	0
Streptococcus_pneumoniae_EU_NP03	0	0	0	1000	0	0	1000	0	0	0
Streptococcus_pneumoniae_EU_NP04	0	0	0	1000	0	0	1000	0	0	0
Streptococcus_pneumoniae_EU_NP05	0	0	0	1000	0	0	1000	0	0	0
Streptococcus_pneumoniae_G54	0	0	0	1000	1000	0	1000	0	0	1000
Streptococcus_pneumoniae_GA02254	0	0	0	1000	0	0	1000	0	0	0
Streptococcus_pneumoniae_GA02270	0	0	0	1000	0	0	1000	0	0	0
Streptococcus_pneumoniae_GA02506	0	0	0	1000	0	0	1000	0	0	0
Streptococcus_pneumoniae_GA02714	0	0	0	1000	0	0	1000	0	0	0
Streptococcus_pneumoniae_GA04175	0	0	0	1000	0	0	1000	0	0	0
Streptococcus_pneumoniae_GA04216	0	0	0	1000	0	0	1000	0	0	0
Streptococcus_pneumoniae_GA04375	0	0	0	1000	0	0	1000	0	0	0
Streptococcus_pneumoniae_GA04672	0	0	0	1000	0	0	1000	0	0	0
Streptococcus_pneumoniae_GA05245	0	0	0	1000	0	0	1000	0	0	0
Streptococcus_pneumoniae_GA05248	0	0	0	1000	0	0	1000	0	0	0
Streptococcus_pneumoniae_GA05578	0	0	0	1000	0	0	1000	0	0	0
Streptococcus_pneumoniae_GA06083	0	0	0	1000	0	0	1000	0	0	0
Streptococcus_pneumoniae_GA07228	0	0	0	1000	0	0	1000	0	0	0
Streptococcus_pneumoniae_GA07643	0	0	0	1000	0	0	1000	0	0	0
Streptococcus_pneumoniae_GA07914	0	0	0	1000	0	0	1000	0	0	0
Streptococcus_pneumoniae_GA08780	0	0	0	1000	0	0	1000	0	0	0
Streptococcus_pneumoniae_GA08825	0	0	0	1000	0	0	1000	0	0	0
Streptococcus_pneumoniae_GA11304	0	0	0	1000	0	0	1000	0	0	0
Streptococcus_pneumoniae_GA11426	0	0	0	1000	0	0	1000	0	0	0
Streptococcus_pneumoniae_GA13224	0	0	0	1000	0	0	1000	0	0	0
Streptococcus_pneumoniae_GA13338	0	0	0	1000	0	0	1000	0	0	0
Streptococcus_pneumoniae_GA13430	0	0	0	1000	0	0	1000	0	0	0
Streptococcus_pneumoniae_GA13455	0	0	0	1000	0	0	1000	0	0	0
Streptococcus_pneumoniae_GA13494	0	0	0	1000	0	0	1000	0	0	0
Streptococcus_pneumoniae_GA13856	0	0	0	1000	0	0	1000	0	0	0
Streptococcus_pneumoniae_GA14373	0	0	0	1000	0	0	1000	0	0	0
Streptococcus_pneumoniae_GA14688	0	0	0	1000	0	0	1000	0	0	0
Streptococcus_pneumoniae_GA14798	0	0	0	1000	0	0	1000	0	0	0
Streptococcus_pneumoniae_GA16121	0	0	0	1000	0	0	1000	0	0	0
Streptococcus_pneumoniae_GA16242	0	0	0	1000	0	0	1000	0	0	0
Streptococcus_pneumoniae_GA16531	0	0	0	1000	0	0	1000	0	0	0
Streptococcus_pneumoniae_GA17301	0	0	0	1000	0	0	1000	0	0	0
Streptococcus_pneumoniae_GA17328	0	0	0	1000	0	0	1000	0	0	0
Streptococcus_pneumoniae_GA17371	0	0	0	1000	0	0	1000	0	0	0
Streptococcus_pneumoniae_GA17457	0	0	0	1000	0	0	1000	0	0	0
Streptococcus_pneumoniae_GA17484	0	0	0	1000	0	0	1000	0	0	0
Streptococcus_pneumoniae_GA17545	0	0	0	1000	0	0	1000	0	0	0
Streptococcus_pneumoniae_GA17570	0	0	0	1000	0	0	1000	0	0	0
Streptococcus_pneumoniae_GA17719	0	0	0	1000	0	0	1000	0	0	0
Streptococcus_pneumoniae_GA17971	0	0	0	1000	0	0	1000	0	0	0
Streptococcus_pneumoniae_GA18068	0	0	0	1000	0	0	1000	0	0	0
Streptococcus_pneumoniae_GA18523	0	0	0	1000	0	0	1000	0	0	0
Streptococcus_pneumoniae_GA19077	0	0	0	1000	0	0	1000	0	0	0
Streptococcus_pneumoniae_GA19101	0	0	0	1000	0	0	1000	0	0	0
Streptococcus_pneumoniae_GA19690	0	0	0	1000	0	0	1000	0	0	0
Streptococcus_pneumoniae_GA19923	0	0	0	1000	0	0	1000	0	0	0
Streptococcus_pneumoniae_GA40028	0	0	0	1000	0	0	1000	0	0	0
Streptococcus_pneumoniae_GA40183	0	0	0	1000	0	0	1000	0	0	0
Streptococcus_pneumoniae_GA40410	0	0	0	1000	0	0	1000	0	0	0
Streptococcus_pneumoniae_GA40563	0	0	0	1000	0	0	1000	0	0	0
Streptococcus_pneumoniae_GA41277	0	0	0	1000	0	0	1000	0	0	0
Streptococcus_pneumoniae_GA41301	0	0	0	1000	0	0	1000	0	0	0
Streptococcus_pneumoniae_GA41317	0	0	0	1000	0	0	1000	0	0	0
Streptococcus_pneumoniae_GA41410	0	0	0	1000	0	0	1000	0	0	0
Streptococcus_pneumoniae_GA41538	0	0	0	1000	0	0	1000	0	0	0
Streptococcus_pneumoniae_GA41565	0	0	0	1000	0	0	1000	0	0	0
Streptococcus_pneumoniae_GA41688	0	0	0	1000	0	0	1000	0	0	0
Streptococcus_pneumoniae_GA43257	0	0	0	1000	0	0	1000	0	0	0
Streptococcus_pneumoniae_GA43264	0	0	0	1000	0	0	1000	0	0	0
Streptococcus_pneumoniae_GA43265	0	0	0	1000	0	0	1000	0	0	0
Streptococcus_pneumoniae_GA43380	0	0	0	1000	0	0	1000	0	0	0
Streptococcus_pneumoniae_GA44128	0	0	0	1000	0	0	1000	0	0	0

Streptococcus_pneumoniae_GA44194	0	0	0	1000	0	0	1000	0	0	0
Streptococcus_pneumoniae_GA44288	0	0	0	1000	0	0	1000	0	0	0
Streptococcus_pneumoniae_GA44378	0	0	0	1000	0	0	1000	0	0	0
Streptococcus_pneumoniae_GA44386	0	0	0	1000	0	0	1000	0	0	0
Streptococcus_pneumoniae_GA44452	0	0	0	1000	0	0	1000	0	0	0
Streptococcus_pneumoniae_GA44500	0	0	0	1000	0	0	1000	0	0	0
Streptococcus_pneumoniae_GA44511	0	0	0	1000	0	0	1000	0	0	0
Streptococcus_pneumoniae_GA47033	0	0	0	1000	0	0	1000	0	0	0
Streptococcus_pneumoniae_GA47179	0	0	0	1000	0	0	1000	0	0	0
Streptococcus_pneumoniae_GA47210	0	0	0	1000	0	0	1000	0	0	0
Streptococcus_pneumoniae_GA47281	0	0	0	1000	0	0	1000	0	0	0
Streptococcus_pneumoniae_GA47283	0	0	0	1000	0	0	1000	0	0	0
Streptococcus_pneumoniae_GA47360	0	0	0	1000	0	0	1000	0	0	0
Streptococcus_pneumoniae_GA47388	0	0	0	1000	0	0	1000	0	0	0
Streptococcus_pneumoniae_GA47439	0	0	0	1000	0	0	1000	0	0	0
Streptococcus_pneumoniae_GA47461	0	0	0	1000	0	0	1000	0	0	0
Streptococcus_pneumoniae_GA47502	0	0	0	1000	0	0	1000	0	0	0
Streptococcus_pneumoniae_GA47522	0	0	0	1000	0	0	1000	0	0	0
Streptococcus_pneumoniae_GA47562	0	0	0	1000	0	0	1000	0	0	0
Streptococcus_pneumoniae_GA47597	0	0	0	1000	0	0	1000	0	0	0
Streptococcus_pneumoniae_GA47628	0	0	0	1000	0	0	1000	0	0	0
Streptococcus_pneumoniae_GA47688	0	0	0	1000	0	0	1000	0	0	0
Streptococcus_pneumoniae_GA47751	0	0	0	1000	0	0	1000	0	0	0
Streptococcus_pneumoniae_GA47760	0	0	0	1000	0	0	1000	0	0	0
Streptococcus_pneumoniae_GA47794	0	0	0	1000	0	0	1000	0	0	0
Streptococcus_pneumoniae_GA47901	0	0	0	1000	0	0	1000	0	0	0
Streptococcus_pneumoniae_GA47976	0	0	0	1000	0	0	1000	0	0	0
Streptococcus_pneumoniae_GA49138	0	0	0	1000	0	0	1000	0	0	0
Streptococcus_pneumoniae_GA49194	0	0	0	1000	0	0	1000	0	0	0
Streptococcus_pneumoniae_GA49447	0	0	0	1000	0	0	1000	0	0	0
Streptococcus_pneumoniae_GA49542	0	0	0	1000	0	0	1000	0	0	0
Streptococcus_pneumoniae_GA52306	0	0	0	1000	0	0	1000	0	0	0
Streptococcus_pneumoniae_GA52612	0	0	0	1000	0	0	1000	0	0	0
Streptococcus_pneumoniae_GA54354	0	0	0	1000	0	0	1000	0	0	0
Streptococcus_pneumoniae_GA54644	0	0	0	1000	0	0	1000	0	0	0
Streptococcus_pneumoniae_GA56113	0	0	0	1000	0	0	1000	0	0	0
Streptococcus_pneumoniae_GA58581	0	0	0	1000	0	0	1000	0	0	0
Streptococcus_pneumoniae_GA58771	0	0	0	1000	0	0	1000	0	0	0
Streptococcus_pneumoniae_GA58981	0	0	0	1000	0	0	1000	0	0	0
Streptococcus_pneumoniae_GA60080	0	0	0	1000	0	0	1000	0	0	0
Streptococcus_pneumoniae_GA60132	0	0	0	1000	0	0	1000	0	0	0
Streptococcus_pneumoniae_GA60190	0	0	0	1000	0	0	1000	0	0	0
Streptococcus_pneumoniae_GA62331	0	0	0	1000	0	0	1000	0	0	0
Streptococcus_pneumoniae_GA62681	0	0	0	1000	0	0	1000	0	0	0
Streptococcus_pneumoniae_gamPNI0373	0	0	0	1000	0	0	1000	0	0	0
Streptococcus_pneumoniae_Hungary19A_6	0	0	0	1000	0	0	1000	0	0	0
Streptococcus_pneumoniae_INV104	0	0	0	1000	0	0	1000	0	0	0
Streptococcus_pneumoniae_INV200	0	0	0	1000	0	0	1000	0	0	0
Streptococcus_pneumoniae_JJA	0	0	0	1000	0	0	1000	0	0	0
Streptococcus_pneumoniae_MLV_016	0	0	0	1000	0	0	1000	0	0	0
Streptococcus_pneumoniae_MNZ11b	0	0	0	1000	0	0	1000	0	0	0
Streptococcus_pneumoniae_MNZ14	0	0	0	1000	0	0	1000	0	0	0
Streptococcus_pneumoniae_MNZ37	0	0	0	1000	0	0	1000	0	0	0
Streptococcus_pneumoniae_MNZ41	0	0	0	1000	0	0	1000	0	0	0
Streptococcus_pneumoniae_NorthCarolina6A_23	0	0	0	1000	0	0	1000	0	0	0
Streptococcus_pneumoniae_NP070	0	0	0	1000	0	0	1000	0	0	0
Streptococcus_pneumoniae_NP112	0	0	0	1000	0	0	1000	0	0	0
Streptococcus_pneumoniae_NP127	0	0	0	1000	0	0	1000	0	0	0
Streptococcus_pneumoniae_NP141	0	0	0	1000	0	0	1000	0	0	0
Streptococcus_pneumoniae_OXC141	0	0	0	1000	0	0	1000	0	0	0
Streptococcus_pneumoniae_P1031	0	0	0	1000	0	0	1000	0	0	0
Streptococcus_pneumoniae_PCS125219	0	0	0	1000	0	0	1000	0	0	0
Streptococcus_pneumoniae_PCS70012	0	0	0	1000	0	0	1000	0	0	0
Streptococcus_pneumoniae_PCS8106	0	0	0	1000	0	0	1000	0	0	0
Streptococcus_pneumoniae_PCS8203	0	0	0	1000	0	0	1000	0	0	0
Streptococcus_pneumoniae_PNI0002	0	0	0	1000	0	0	1000	0	0	0
Streptococcus_pneumoniae_PNI0006	0	0	0	1000	0	0	1000	0	0	0
Streptococcus_pneumoniae_PNI0010	0	0	0	1000	0	0	1000	0	0	0
Streptococcus_pneumoniae_PNI0153	0	0	0	1000	0	0	1000	0	0	0
Streptococcus_pneumoniae_R6	0	0	0	1000	0	0	1000	0	0	0
Streptococcus_pneumoniae_R6M2_PG	0	0	0	1000	0	0	1000	0	0	0
Streptococcus_pneumoniae_SP11_BS70	0	0	0	1000	0	0	1000	0	0	0
Streptococcus_pneumoniae_SP14_BS292	0	0	0	1000	0	0	1000	0	0	0
Streptococcus_pneumoniae_SP18_BS74	0	0	0	1000	0	0	1000	0	0	0
Streptococcus_pneumoniae_SP19_BS75	0	0	0	1000	0	0	1000	0	0	0
Streptococcus_pneumoniae_SP195	0	0	0	1000	0	0	1000	0	0	0
Streptococcus_pneumoniae_SP23_BS72	0	0	0	1000	0	0	1000	0	0	0
Streptococcus_pneumoniae_SP3_BS71	0	0	0	1000	0	0	1000	0	0	0
Streptococcus_pneumoniae_SP6_BS73	0	0	0	1000	0	0	1000	0	0	0
Streptococcus_pneumoniae_SP9_BS68	0	0	0	1000	0	0	1000	0	0	0
Streptococcus_pneumoniae_SPAR27	0	0	0	1000	0	0	1000	0	0	0
Streptococcus_pneumoniae_SPAR55	0	0	0	1000	0	0	1000	0	0	0
Streptococcus_pneumoniae_SPAR95	0	0	0	1000	0	0	1000	0	0	0
Streptococcus_pneumoniae_SPN021198	0	0	0	1000	0	0	1000	0	0	0
Streptococcus_pneumoniae_SPN032672	0	0	0	1000	0	0	1000	0	0	0
Streptococcus_pneumoniae_SPN033038	0	0	0	1000	0	0	1000	0	0	0
Streptococcus_pneumoniae_SPN034156	0	0	0	1000	0	0	1000	0	0	0
Streptococcus_pneumoniae_SPN034183	0	0	0	1000	0	0	1000	0	0	0
Streptococcus_pneumoniae_SPN061370	0	0	0	1000	0	0	1000	0	0	0
Streptococcus_pneumoniae_SPN072838	0	0	0	1000	0	0	1000	0	0	0
Streptococcus_pneumoniae_SPN1041	0	0	0	1000	0	0	1000	0	0	0
Streptococcus_pneumoniae_SPN7465	0	0	0	1000	0	0	1000	0	0	0
Streptococcus_pneumoniae_SPNA45	0	0	0	1000	0	0	1000	0	0	0
Streptococcus_pneumoniae_ST556	0	0	0	1000	0	0	1000	0	0	0

Streptococcus_pneumoniae_SV35	0	0	0	1000	0	0	1000	0	0	0
Streptococcus_pneumoniae_SV36	0	0	0	1000	0	0	1000	0	0	0
Streptococcus_pneumoniae_Taiwan19F_14	0	0	0	1000	0	0	1000	0	0	0
Streptococcus_pneumoniae_TCH8431_19A	0	0	0	1000	0	0	1000	0	0	0
Streptococcus_pneumoniae_TIGR4	0	0	0	1000	0	0	1000	0	0	0
Streptococcus_pseudopneumoniae_IS7493	0	0	0	0	1000	0	1000	0	0	1000
Streptococcus_pyogenes_A20	0	0	0	0	0	0	1000	0	0	0
Streptococcus_pyogenes_Alab49	0	0	0	0	0	0	1000	0	0	0
Streptococcus_pyogenes_ATCC_10782	0	0	0	0	0	0	1000	0	0	0
Streptococcus_pyogenes_M1_GAS	0	0	0	0	0	0	1000	0	0	0
Streptococcus_pyogenes_MGAS10394	0	0	0	0	0	0	1000	0	0	0
Streptococcus_pyogenes_MGAS315	0	0	0	0	0	0	1000	0	0	0
Streptococcus_pyogenes_MGAS8232	0	0	0	0	0	0	1000	0	0	0
Streptococcus_pyogenes_MGAS9429	0	0	0	0	0	0	1000	0	0	1000
Streptococcus_pyogenes_SSI_1	0	0	0	0	0	0	1000	0	0	0
Streptococcus_pyogenes_str_Manfredo	0	0	0	0	0	0	1000	0	0	0
Streptococcus_salivarius_57_I	0	0	1000	0	0	0	1000	0	0	0
Streptococcus_salivarius_DSM_20560	0	0	1000	0	0	0	1000	0	0	0
Streptococcus_salivarius_ERR2221359	0	0	1000	0	0	0	1000	0	0	0
Streptococcus_salivarius_JIM8777	0	0	0	0	0	0	1000	0	0	1000
Streptococcus_salivarius_K12	0	0	1000	0	0	0	1000	0	0	0
Streptococcus_salivarius_M18	0	0	1000	0	0	0	1000	0	0	0
Streptococcus_salivarius_PS4	0	0	1000	0	0	0	1000	0	0	0
Streptococcus_salivarius_SK126	0	0	1000	0	0	0	1000	0	0	0
Streptococcus_sanguinis_ATCC_29667	0	0	0	0	0	0	1000	0	0	0
Streptococcus_sanguinis_ATCC_49296	0	0	0	0	0	0	1000	0	0	0
Streptococcus_sanguinis_SK1	0	0	0	0	0	0	1000	0	0	0
Streptococcus_sanguinis_SK1056	0	0	0	0	0	0	1000	0	0	0
Streptococcus_sanguinis_SK1057	0	0	0	0	0	0	1000	0	0	0
Streptococcus_sanguinis_SK1058	0	0	0	0	0	0	1000	0	0	0
Streptococcus_sanguinis_SK1059	0	0	0	0	0	0	1000	0	0	0
Streptococcus_sanguinis_SK1087	0	0	0	0	0	0	1000	0	0	0
Streptococcus_sanguinis_SK115	0	0	0	0	0	0	1000	0	0	0
Streptococcus_sanguinis_SK150	0	0	0	0	0	0	1000	0	0	0
Streptococcus_sanguinis_SK160	0	0	0	0	0	0	1000	0	0	0
Streptococcus_sanguinis_SK330	0	0	0	0	0	0	1000	0	0	0
Streptococcus_sanguinis_SK340	0	0	0	0	0	0	1000	0	0	0
Streptococcus_sanguinis_SK353	0	0	0	0	0	0	1000	0	0	0
Streptococcus_sanguinis_SK355	0	0	0	0	0	0	1000	0	0	0
Streptococcus_sanguinis_SK36	0	0	0	0	0	0	1000	0	0	1000
Streptococcus_sanguinis_SK405	0	0	0	0	0	0	1000	0	0	0
Streptococcus_sanguinis_SK408	0	0	0	0	0	0	1000	0	0	0
Streptococcus_sanguinis_SK49	0	0	0	0	0	0	1000	0	0	0
Streptococcus_sanguinis_SK678	0	0	0	0	0	0	1000	0	0	0
Streptococcus_sanguinis_SK72	0	0	0	0	0	0	1000	0	0	0
Streptococcus_sanguinis_VMC66	0	0	0	0	0	0	1000	0	0	0
Streptococcus_sobrinus_NIDR_6715_7	0	0	1000	0	0	0	1000	0	0	0
Streptococcus_sobrinus_TCI_13	0	0	1000	0	0	0	1000	0	0	0
Streptococcus_sobrinus_TCI_16	0	0	1000	0	0	0	1000	0	0	0
Streptococcus_sobrinus_TCI_28	0	0	1000	0	0	0	1000	0	0	0
Streptococcus_sobrinus_TCI_50	0	0	1000	0	0	0	1000	0	0	0
Streptococcus_sobrinus_TCI_53	0	0	1000	0	0	0	1000	0	0	0
Streptococcus_sp_2_1_36FAA	0	0	0	0	0	0	1000	0	0	0
Streptococcus_sp_BS35b	0	0	0	0	0	0	1000	0	0	0
Streptococcus_sp_GMD5S	0	0	0	0	0	0	1000	0	0	0
Streptococcus_sp_HPH0090	0	0	0	0	0	0	1000	0	0	0
Streptococcus_sp_I_G2	0	0	0	0	0	0	1000	0	0	0
Streptococcus_sp_I_P16	0	0	0	0	0	0	1000	0	0	0
Streptococcus_sp_SK140	0	0	0	0	0	0	1000	0	0	0
Streptococcus_thermophilus_CNRZ1066	0	0	1000	0	0	0	1000	0	0	0
Streptococcus_thermophilus_JIM_8232	0	0	1000	0	0	0	1000	0	0	0
Streptococcus_thermophilus_LMD_9	0	0	1000	0	0	0	1000	0	0	0
Streptococcus_thermophilus_LMG_18311	0	0	1000	0	0	0	1000	0	0	0
Streptococcus_thermophilus_MN_ZLW_002	0	0	1000	0	0	0	1000	0	0	0
Streptococcus_thermophilus_ND03	0	0	1000	0	0	0	1000	0	0	0
Streptococcus_thoraltensis_DSM_12221	0	0	1000	1000	0	0	1000	0	0	1000
Streptococcus_uberis_0140J	0	0	0	1000	1000	0	1000	0	0	1000
Streptococcus_vestibularis_ATCC_49124	0	0	1000	0	0	0	1000	0	0	0
Streptococcus_vestibularis_F0396	0	0	1000	0	0	0	1000	0	0	1000
Streptomyces_massiliensis_AP10	0	0	1000	0	1000	0	0	0	0	0
Subdoligranulum_sp_4_3_54A2FAA	0	0	0	0	0	0	1000	0	0	0
Subdoligranulum_variabile_DSM_15176	0	0	0	0	1000	0	1000	0	0	0
Succinatimonas_hippei_YIT_12066	0	0	0	0	1000	0	1000	0	0	0
Succiniclasticum_ruminis_DSM_11005	0	0	0	0	500	0	500	0	0	0
Succiniclasticum_ruminis_DSM_9236	0	0	0	0	500	0	625	0	0	0
Succinivibrio_dextrinosolvens_H5	0	0	1000	0	1000	0	0	0	0	0
Sutterella_parvirubra_YIT_11816	0	0	0	0	1000	0	1000	0	0	0
Sutterella_wadsworthensis_2_1_59BFAA	0	0	0	0	1000	0	1000	0	0	0
Sutterella_wadsworthensis_3_1_45B	0	0	0	0	1000	0	1000	0	0	0
Sutterella_wadsworthensis_ERR1203961	0	0	0	0	1000	0	1000	0	0	0
Sutterella_wadsworthensis_ERR1204055	0	0	0	0	1000	0	1000	0	0	0
Sutterella_wadsworthensis_HGA0223	0	0	0	0	1000	0	1000	0	0	0
Synergistes_jonesii_78_1	0	0	1000	0	0	0	1000	0	0	0
Synergistes_sp_3_1_syn1	0	0	0	0	1000	0	1000	0	0	0
Syntrophus_aciditrophicus_SB	133.9285714	0	1000	0	1000	0	0	0	0	0
Syntrophus_gentianae_DSM_8423	0	0	1000	0	0	0	0	0	0	0
Tannerella_forsythia_ATCC_43037	0	0	0	0	0	0	1000	0	0	0
Tannerella_sp_6_1_58FAA_CT1	0	0	0	1000	0	0	0	0	0	0
Tatumella_ptyseos_ATCC_33301	0	0	1000	0	1000	0	1000	0	0	1000
Terrisporobacter_glycolicus_ERR2221120	0	0	0	0	1000	0	1000	0	0	0
Terrisporobacter_mayombei_ERR2221147	0	0	0	0	1000	0	1000	0	0	0
Terrisporobacter_nov_ERR2221319	0	0	0	0	1000	0	1000	0	0	0
Tessaracoccus_massiliensis_SIT6	0	0	0	0	0	0	1000	0	0	0
Tetragenococcus_koreensis_KCTC_3924	0	0	0	0	0	0	1000	0	0	0

Tetragenococcus_koreensis_NBRC_106072	0	0	0	0	0	0	1000	0	0	0
Thalassobacillus_massiliensis_TM_1	0	0	1000	0	1000	0	1000	0	0	0
Thermoanaerobacter_pseudethanolicus_ATCC_33	0	0	0	0	0	0	1000	0	0	0
Thermus_scotoductus_DSM_8553	0	0	1000	0	0	0	0	0	1000	0
Thermus_scotoductus_K1_1	0	0	1000	0	0	0	0	0	1000	0
Thermus_scotoductus_KI2	0	0	1000	0	0	0	0	0	1000	0
Thermus_scotoductus_SA_01	200	0	1000	0	0	0	0	0	1000	0
Timonella_senegalensis_JC301	0	0	0	0	0	0	1000	0	0	0
Trabulsiella_guamensis_ATCC_49490	0	0	1000	0	1000	0	1000	0	0	1000
Treponema_denticola_AL_2	0	0	0	0	1000	0	1000	0	0	0
Treponema_denticola_ASLM	0	0	0	0	1000	0	1000	0	0	0
Treponema_denticola_ATCC_33520	0	0	0	0	1000	0	1000	0	0	0
Treponema_denticola_ATCC_33521	0	0	0	0	1000	0	1000	0	0	0
Treponema_denticola_ATCC_35404	0	0	0	0	1000	0	1000	0	0	0
Treponema_denticola_H_22	0	0	0	0	1000	0	1000	0	0	0
Treponema_medium_ATCC_700293	0	0	0	0	1000	0	1000	0	0	0
Treponema_socranskii_subsp_paredis_ATCC_355	0	0	0	0	1000	0	0	0	0	0
Treponema_socranskii_subsp_socranskii_VPI_DR	0	0	0	0	1000	0	0	0	0	0
Treponema_succinifaciens_DSM_2489	0	0	0	0	0	0	1000	0	0	0
Treponema_vincentii_ATCC_35580	0	0	0	0	1000	0	1000	0	0	0
Treponema_vincentii_F0403	0	0	0	0	1000	0	1000	0	0	0
Tropheryma_whipplei_str_Twist	0	0	0	0	1000	0	0	0	0	0
Tropheryma_whipplei_TW08_27	0	0	0	0	0	0	0	0	0	0
Trueperella_pyogenes_MS249	0	0	0	0	0	0	1000	0	0	0
Turicibacter_sanguinis_ERR1022280	0	0	0	0	1000	0	1000	0	0	0
Turicibacter_sanguinis_ERR1022323	0	0	0	0	1000	0	1000	0	0	0
Turicibacter_sanguinis_ERR1022367	0	0	0	0	1000	0	1000	0	0	0
Turicibacter_sanguinis_ERR1022463	0	0	0	0	0	0	1000	0	0	0
Turicibacter_sanguinis_ERR2221386	0	0	0	0	1000	0	1000	0	0	0
Turicibacter_sanguinis_ERR2230131	0	0	0	0	1000	0	1000	0	0	0
Turicibacter_sanguinis_PC909	0	0	0	0	1000	0	1000	0	0	0
Turicibacter_sp_H121	0	0	0	0	1000	0	1000	0	0	0
Turicibacter_sp_HGF1	0	0	0	0	1000	0	1000	0	0	0
Turicimonas_muris_YL45	0	0	0	0	1000	0	1000	0	0	0
Tyzzerella_nov_ERR171258	0	0	0	0	1000	0	1000	0	0	0
uncultured_Anaerotruncus_sp_ERR1022410	0	0	1000	0	0	0	1000	0	0	0
uncultured_Blautia_sp_ERR1022314	0	0	0	0	1000	0	1000	0	0	0
uncultured_Blautia_sp_ERR1022472	0	0	0	0	1000	0	0	0	0	0
uncultured_Clostridium_sp_ERR1022385	0	0	0	0	1000	0	1000	0	0	0
uncultured_Clostridium_sp_ERR1022438	0	0	0	0	0	0	1000	0	0	0
uncultured_Clostridium_sp_ERR1022467	0	0	0	0	1000	0	1000	0	0	0
uncultured_Ruminococcus_sp_ERR1022393	0	0	0	0	1000	0	1000	0	0	0
uncultured_Ruminococcus_sp_ERR1022403	0	0	1000	0	1000	0	1000	0	0	0
Ureaplasma_parvum_serovar_1_str_ATCC_27813	0	0	0	0	500	0	1000	0	0	0
Ureaplasma_parvum_serovar_14_str_ATCC_3369	0	0	0	0	500	0	500	0	0	0
Ureaplasma_parvum_serovar_3_str_ATCC_27815	0	0	0	0	500	0	500	0	0	0
Ureaplasma_parvum_serovar_3_str_ATCC_70097	0	0	0	0	500	0	500	0	0	0
Ureaplasma_parvum_serovar_6_str_ATCC_27818	0	0	0	0	500	0	500	0	0	0
Ureaplasma_urealyticum_2033	0	0	0	0	500	0	500	0	0	0
Ureaplasma_urealyticum_2608	0	0	0	0	500	0	500	0	0	0
Ureaplasma_urealyticum_4155	0	0	0	0	500	0	500	0	0	0
Ureaplasma_urealyticum_4318	0	0	0	0	500	0	500	0	0	0
Ureaplasma_urealyticum_serovar_10_str_ATCC_3	0	0	0	0	500	0	500	0	0	0
Ureaplasma_urealyticum_serovar_11_str_ATCC_3	0	0	0	0	500	0	500	0	0	0
Ureaplasma_urealyticum_serovar_12_str_ATCC_3	0	0	0	0	500	0	500	0	0	0
Ureaplasma_urealyticum_serovar_13_str_ATCC_3	0	0	0	0	500	0	500	0	0	0
Ureaplasma_urealyticum_serovar_2_str_ATCC_27	0	0	0	0	500	0	500	0	0	0
Ureaplasma_urealyticum_serovar_4_str_ATCC_27	0	0	0	0	500	0	500	0	0	0
Ureaplasma_urealyticum_serovar_5_str_ATCC_27	0	0	0	0	500	0	500	0	0	0
Ureaplasma_urealyticum_serovar_7_str_ATCC_27	0	0	0	0	500	0	500	0	0	0
Ureaplasma_urealyticum_serovar_8_str_ATCC_27	0	0	0	0	500	0	1000	0	0	0
Ureaplasma_urealyticum_serovar_9_str_ATCC_33	0	0	0	0	500	0	500	0	0	0
Ureibacillus_thermosphaericus_str_Thermo_BF	0	0	1000	0	1000	0	1000	0	0	0
Vagococcus_fluvialis_bH819	0	0	0	0	0	0	1000	0	0	0
Vagococcus_fluvialis_DSM_5731	0	0	0	0	0	0	1000	0	0	0
Vallitalea_guaymasensis_L81	0	0	0	0	0	0	1000	0	1000	0
Vallitalea_sp_S15	0	0	0	0	1000	0	1000	0	0	0
Varibaculum_cambriense_DNF00696	0	0	0	0	0	0	1000	0	0	0
Varibaculum_cambriense_DSM_15806	0	0	0	0	1000	0	1000	0	0	0
Variovorax_paradoxus_110B	0	0	1000	0	1000	0	1000	0	1000	0
Variovorax_paradoxus_4MFCol3_1	0	0	1000	0	1000	0	1000	0	1000	0
Variovorax_paradoxus_B4	0	0	1000	0	1000	0	1000	0	1000	0
Variovorax_paradoxus_H108	0	0	1000	0	1000	0	1000	0	1000	0
Variovorax_paradoxus_NBRC_15149	0	0	1000	0	1000	0	1000	0	1000	0
Veillonella_atypica_ACS_049_V_Sch6	0	0	1000	0	1000	0	1000	0	1000	0
Veillonella_atypica_ACS_134_V_Col7a	0	0	1000	0	1000	0	1000	0	1000	0
Veillonella_atypica_KON	0	0	1000	0	1000	0	1000	0	1000	0
Veillonella_denticariosi_JCM_15641	0	0	1000	0	1000	0	1000	0	1000	0
Veillonella_dispar_ATCC_17748	0	0	0	0	1000	0	1000	0	1000	0
Veillonella_magna_DSM_19857	0	0	1000	0	1000	0	1000	0	1000	0
Veillonella_parvula_ACS_068_V_Sch12	0	0	1000	0	1000	0	1000	0	1000	0
Veillonella_parvula_ATCC_17745	0	0	1000	0	1000	0	1000	0	1000	0
Veillonella_parvula_Te3_DSM_2008	0	0	1000	0	1000	0	1000	0	1000	0
Veillonella_ratti_ACS_216_V_Col6b	0	0	1000	0	1000	0	1000	0	1000	0
Veillonella_sp_3_1_44	0	0	1000	0	1000	0	1000	0	1000	0
Veillonella_sp_6_1_27	0	0	1000	0	1000	0	1000	0	1000	0
Veillonella_sp_ACP1	0	0	1000	0	1000	0	1000	0	1000	0
Veillonella_sp_HPA0037	0	0	1000	0	1000	0	1000	0	1000	0
Veillonella_sp_oral_taxon_158_str_F0412	0	0	1000	0	0	0	1000	0	1000	0
Veillonella_sp_oral_taxon_780_str_F0422	0	0	1000	0	1000	0	1000	0	1000	0
Veillonellaceae_nov_ERR2221308	0	0	1000	0	1000	0	0	0	0	0
Vibrio_campbellii_ATCC_BAA_1116	0	0	1000	0	0	0	1000	0	1000	0
Vibrio_campbellii_HY01	0	0	1000	0	1000	0	1000	0	1000	0
Vibrio_cholerae_A1552	0	0	0	0	1000	0	1000	0	1000	0

Vibrio_cholerae_O1_biovar_El_Tor_str_N16961	0	0	0	0	1000	0	1000	0	1000	0
Vibrio_fluvialis_560	0	0	1000	0	1000	0	1000	0	1000	1000
Vibrio_fluvialis_I21563	0	0	0	0	1000	0	1000	0	1000	1000
Vibrio_fluvialis_PG41	0	0	0	0	1000	0	1000	0	1000	1000
Vibrio_furnissii_CIP_102972	0	0	0	0	1000	0	1000	0	1000	0
Vibrio_furnissii_NCTC_11218	0	0	0	0	1000	0	1000	0	1000	1000
Vibrio_harveyi_1DA3	0	0	1000	0	1000	0	1000	0	1000	0
Vibrio_mimicus_CAIM_602	0	0	0	0	1000	0	1000	0	0	0
Vibrio_mimicus_MB_451	0	0	0	0	1000	0	1000	0	0	1000
Vibrio_mimicus_SX_4	0	0	0	0	1000	0	1000	0	0	0
Vibrio_mimicus_VM223	0	0	0	0	1000	0	1000	0	0	0
Vibrio_mimicus_VM573	0	0	0	0	1000	0	1000	0	0	0
Vibrio_mimicus_VM603	0	0	0	0	1000	0	1000	0	0	0
Vibrio_parahaemolyticus_10329	0	0	0	0	1000	0	1000	0	0	0
Vibrio_parahaemolyticus_AN_5034	0	0	0	0	1000	0	1000	0	0	0
Vibrio_parahaemolyticus_AQ3810	0	0	1000	0	1000	0	1000	0	0	0
Vibrio_parahaemolyticus_BB22OP	0	0	0	0	1000	0	1000	0	0	0
Vibrio_parahaemolyticus_FORC_008	0	0	0	0	1000	0	1000	0	0	0
Vibrio_parahaemolyticus_PCV08_7	0	0	0	0	1000	0	1000	0	0	0
Vibrio_parahaemolyticus_RIMD_2210633	0	0	0	0	1000	0	1000	0	0	1000
Vibrio_parahaemolyticus_SNUVpS_1	0	0	0	0	1000	0	1000	0	0	0
Vibrio_parahaemolyticus_v110	0	0	0	0	1000	0	1000	0	0	0
Vibrio_rotiferianus_DAT722	0	0	0	0	1000	0	1000	0	0	0
Vibrio_shilonii_AK1	0	0	0	0	1000	0	1000	0	0	0
Vibrio_sinaloensis_AD032	0	0	0	0	1000	0	1000	0	1000	0
Vibrio_sinaloensis_DSM_21326	0	0	0	0	1000	0	1000	0	1000	0
Victivallis_vadensis_DSM_14823	0	0	0	0	1000	0	0	0	0	0
Victivallis_vadensis_MGYG_HGUT_02473	0	0	0	0	-2.36E-13	0	0	0	0	0
Virgibacillus_massiliensis_MGYG_HGUT_01470	0	0	1000	0	1000	0	1000	0	0	0
Virgibacillus_massiliensis_Vm_5	0	0	1000	0	1000	0	1000	0	0	0
Virgibacillus_senegalensis_SK_1	0	0	1000	0	1000	0	1000	0	0	0
Weissella_ceti_NC36	0	0	0	0	0	0	1000	0	0	0
Weissella_cibaria_KACC_11862	0	0	0	0	1000	0	1000	0	0	0
Weissella_confusa_LBAE_C39_2	0	0	0	0	1000	0	1000	0	0	0
Weissella_koreensis_KACC_15510	0	0	0	0	0	0	0	0	0	0
Weissella_koreensis_KCTC_3621	0	0	0	0	0	0	0	0	0	0
Weissella_paramesenteroides_ATCC_33313	0	0	0	0	1000	0	1000	0	0	0
Weissella_viridescens_DSM_20410	0	0	0	0	0	0	1000	0	0	0
Yersinia_bercovieri_ATCC_43970	0	0	0	0	1000	0	1000	0	0	1000
Yersinia_enterocolitica_FORC_002	0	0	1000	0	1000	0	1000	0	0	0
Yersinia_enterocolitica_IP_10393	0	0	1000	0	1000	0	1000	0	0	0
Yersinia_enterocolitica_IP2222	0	0	1000	0	1000	0	1000	0	0	0
Yersinia_enterocolitica_NFO	0	0	1000	0	1000	0	1000	0	0	0
Yersinia_enterocolitica_subsp_enterocolitica_808	1000	0	1000	0	1000	0	1000	0	0	1000
Yersinia_enterocolitica_subsp_enterocolitica_WA_	0	0	1000	0	1000	0	1000	0	0	0
Yersinia_enterocolitica_subsp_palearctica_105_5I	916.6666667	0	1000	0	1000	0	1000	0	0	0
Yersinia_enterocolitica_subsp_palearctica_556_82	0	0	1000	0	1000	0	1000	0	0	0
Yersinia_enterocolitica_subsp_palearctica_647_53	0	0	1000	0	1000	0	1000	0	0	0
Yersinia_enterocolitica_subsp_palearctica_PhRBD	0	0	1000	0	1000	0	1000	0	0	0
Yersinia_enterocolitica_subsp_palearctica_Y11	916.6666667	0	1000	0	1000	0	1000	0	0	0
Yersinia_enterocolitica_subsp_palearctica_YE_149	0	0	1000	0	1000	0	1000	0	0	0
Yersinia_enterocolitica_subsp_palearctica_YE_150	0	0	1000	0	1000	0	1000	0	0	0
Yersinia_enterocolitica_subsp_palearctica_YE_P1	0	0	1000	0	1000	0	1000	0	0	0
Yersinia_enterocolitica_subsp_palearctica_YE_P4	0	0	1000	0	1000	0	1000	0	0	0
Yersinia_enterocolitica_subsp_palearctica_YO527	0	0	1000	0	1000	0	1000	0	0	0
Yersinia_frederiksenii_ATCC_33641	0	0	0	0	1000	0	1000	0	0	1000
Yersinia_kristensenii_ATCC_33638	0	0	0	0	1000	0	1000	0	0	1000
Yersinia_kristensenii_Y231	0	0	1000	0	1000	0	1000	0	0	0
Yersinia_pseudotuberculosis_B_6863	0	0	1000	0	1000	0	1000	0	0	0
Yersinia_pseudotuberculosis_B_7194	0	0	1000	0	1000	0	1000	0	0	0
Yersinia_pseudotuberculosis_B_7195	0	0	1000	0	1000	0	1000	0	0	0
Yersinia_pseudotuberculosis_IP_31758	714.2857143	0	1000	0	1000	0	1000	0	0	0
Yersinia_pseudotuberculosis_IP_32953	571.4285714	0	1000	0	1000	0	1000	0	0	0
Yersinia_pseudotuberculosis_PB1	571.4285714	0	1000	0	1000	0	1000	0	0	0
Yersinia_pseudotuberculosis_YPIII	796.875	0	1000	0	1000	0	1000	0	0	1000
Yersinia_rohdei_ATCC_43380	0	0	0	0	1000	0	1000	0	0	1000
Yokenella_regensburgei_ATCC_43003	0	0	0	1000	1000	0	1000	0	0	1000

Supplementary Figure 1

Supplementary Figure 1: Change in ciculating hippurate levels over 12 weeks of treatment

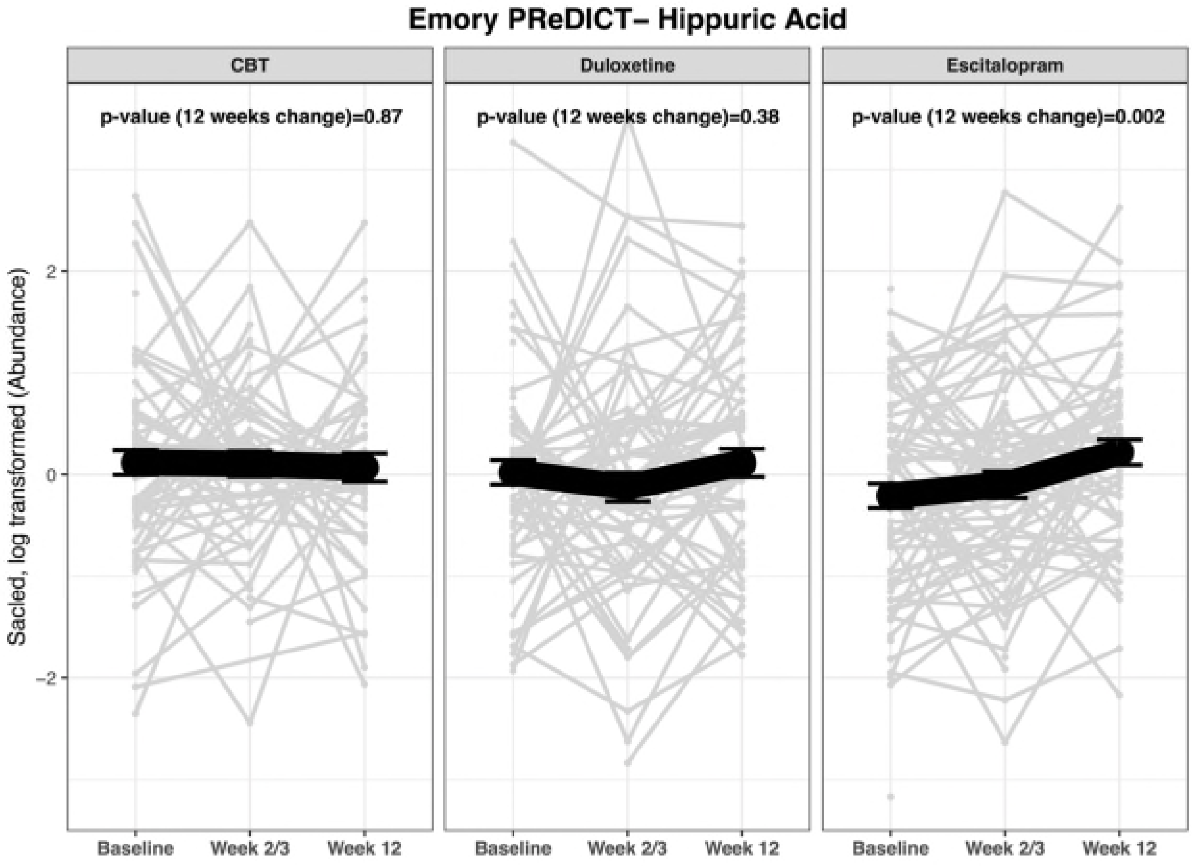

Supplementary Figure 2

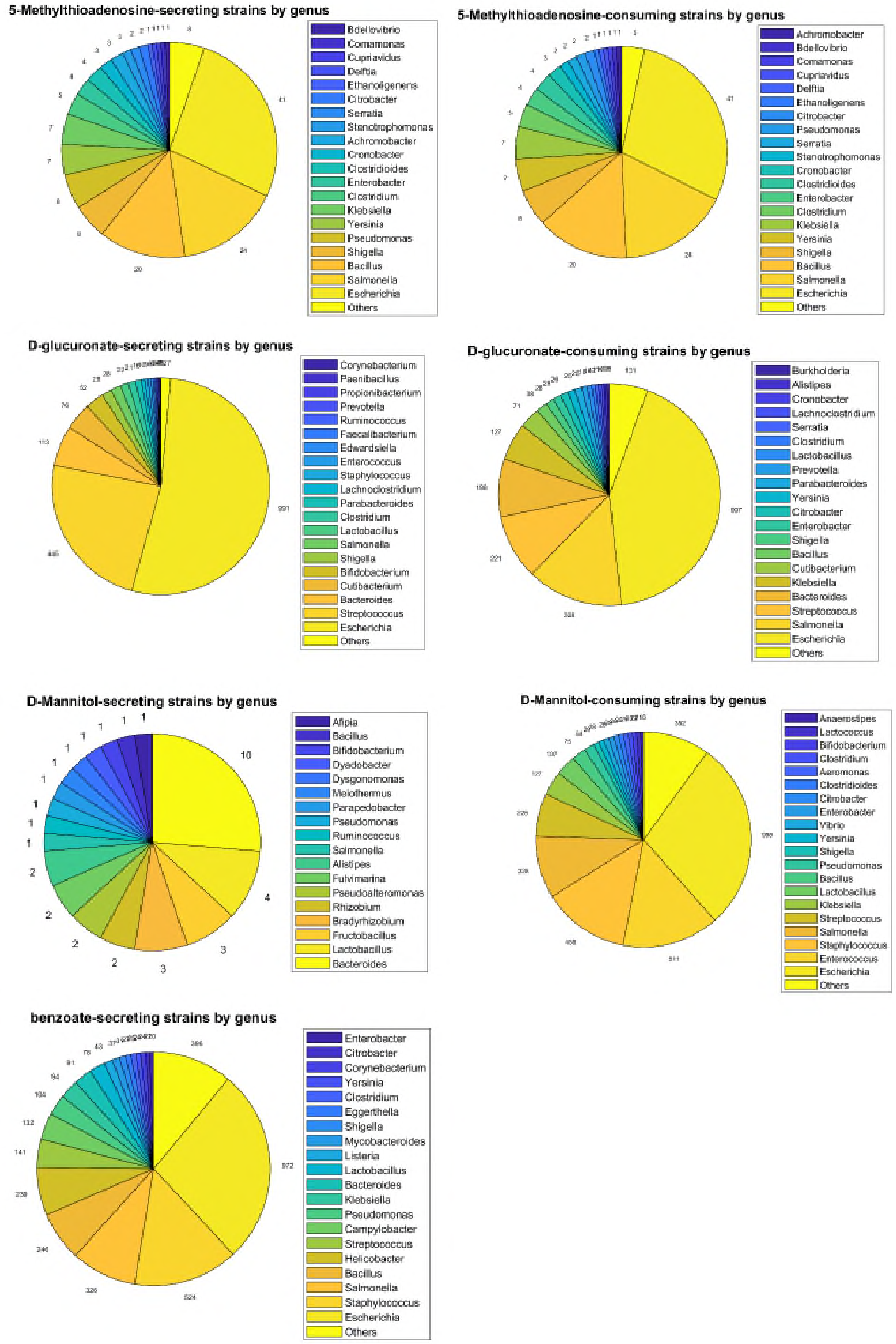

L-arabinitol-secreting strains by genus

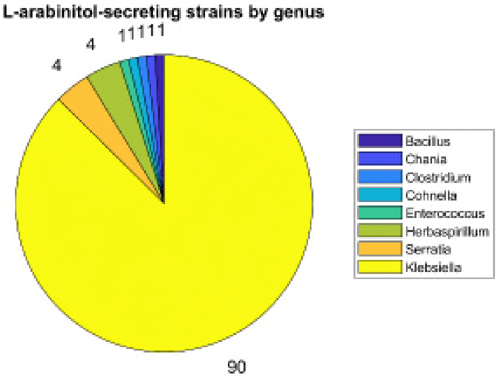

L-arabinitol-consuming strains by genus

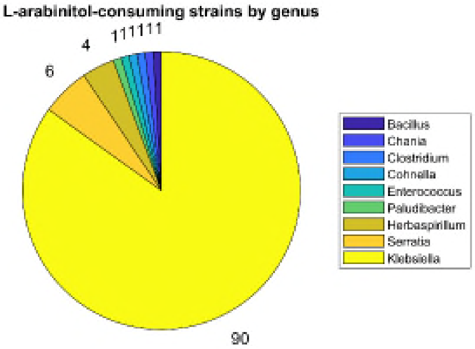

L-glutamate-secreting strains by genus

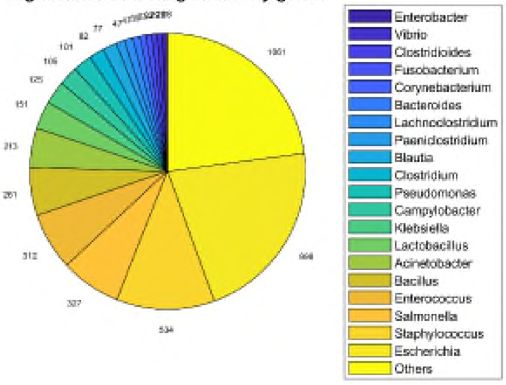

L-glutamate-consuming strains by genus

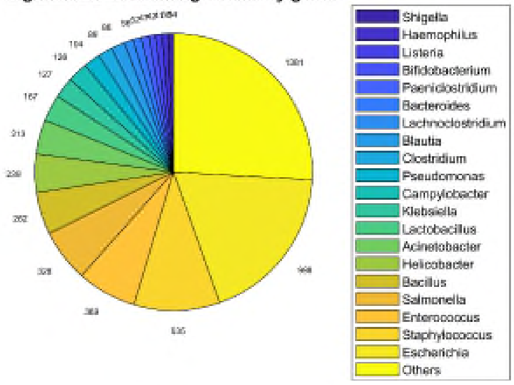

L-leucine-secreting strains by genus

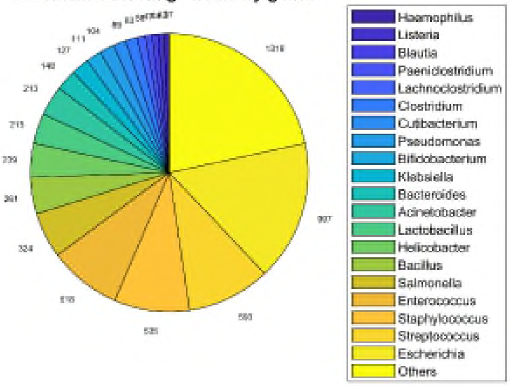

L-leucine-consuming strains by genus

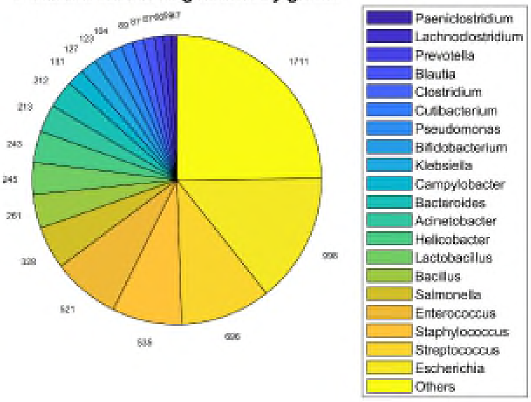

Pyruvate-secreting strains by genus

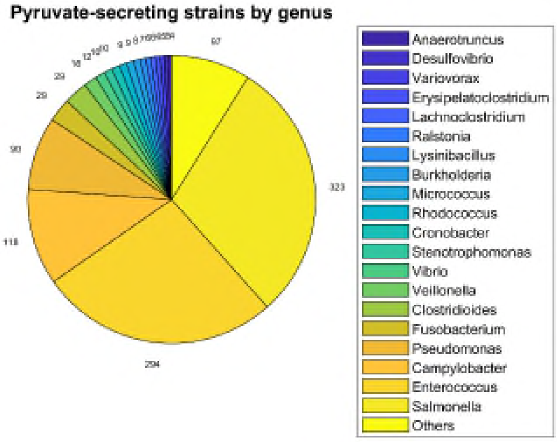

Pyruvate-consuming strains by genus

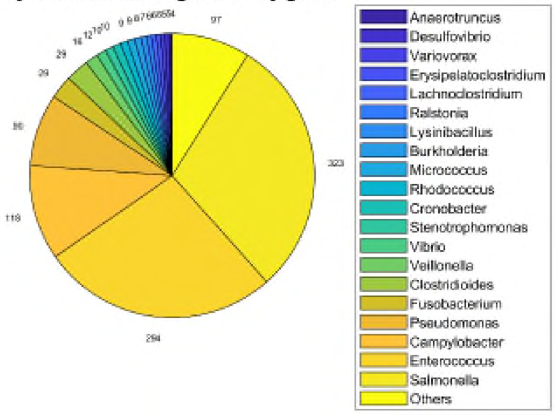

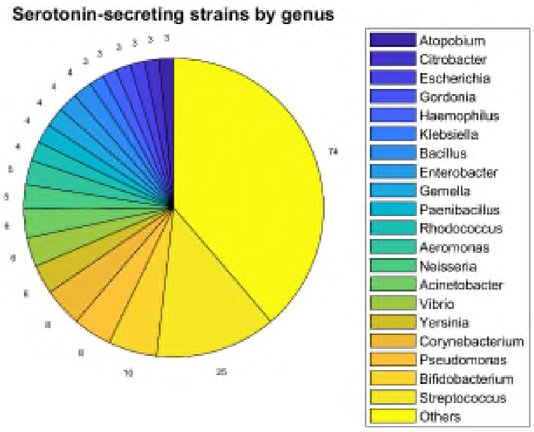
Serotonin-secreting strains by genus
Atopobium
Citrobacter
Escherichia
Gordonia
Haemophilus
Klebsiella
Bacillus
Enterobacter
Gemella
Paenibacillus
Rhodococcus
Aeromonas
Neisseria
Acinetobacter
Vibrio
Yersinia
Corynebacterium
Pseudomonas
Bifidobacterium
Streptococcus
Others

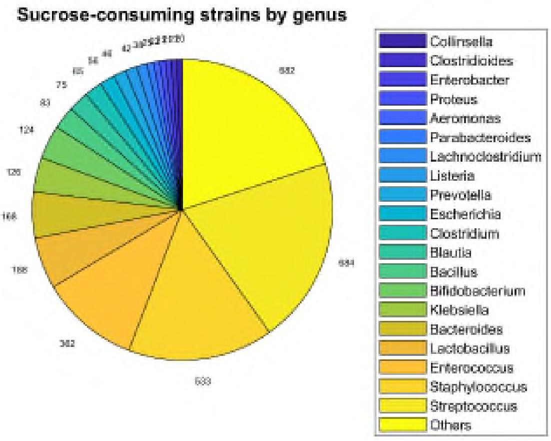
Sucrose-consuming strains by genus
Collinsella
Clostridioides
Enterobacter
Proteus
Aeromonas
Parabacteroides
Lachnoclostridium
Listeria
Prevotella
Escherichia
Clostridium
Blautia
Bacillus
Bifidobacterium
Klebsiella
Bacteroides
Lactobacillus
Enterococcus
Staphylococcus
Streptococcus
Others

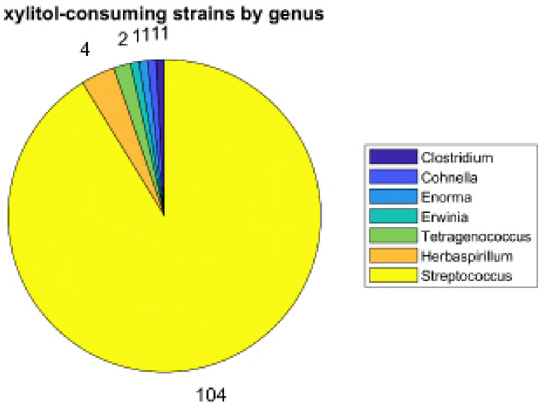
xylitol-consuming strains by genus
4
2
1
1
1
1
104
Clostridium
Cohnella
Enorma
Erwinia
Tetragenococcus
Herbaspirillum
Streptococcus

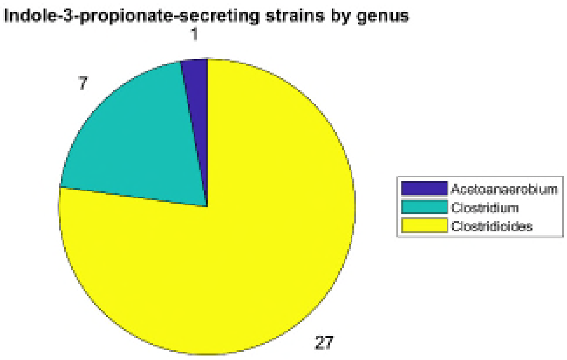
Indole-3-propionate-secreting strains by genus
1
7
27
Acetoanaerobium
Clostridium
Clostridioides
